# Gene association analysis to determine the causal relationship between immune cells and juvenile idiopathic arthritis

**DOI:** 10.1186/s12969-024-00970-8

**Published:** 2024-03-08

**Authors:** Longhao Chen, Xingchen Zhou, Chao Yang, Hong Jiao Wu, Yu Tian, Shuangwei Hong, Huijie Hu, Kaizheng Wang, Shuang Wu, Zicheng Wei, Tao Li, Yuanshen Huang, Zihan Hua, Qiong Xia, Xiao Jie Chen, Zhizhen Lv, Lijiang Lv

**Affiliations:** 1 The Third Affiliated Hospital of Zhejiang Chinese Medical University (Zhongshan Hospital of Zhejiang Province), Zhejiang, Hangzhou China; 2 The Third School of Clinical Medicine (School of Rehabilitation Medicine), Zhejiang Chinese Medical University, Zhejiang, Hangzhou China; 3 Research Institute of Tuina (Spinal disease), Zhejiang Chinese Medical University, Zhejiang, Hangzhou China; 4 Hangzhou TCM Hospital of Zhejiang Chinese Medical University (Hangzhou Hospital of Traditional Chinese Medicine), Zhejiang, Hangzhou China; 5 The 72nd Group Army Hospital of Chinese People’s Liberation Army, Zhejiang, Huzhou China

**Keywords:** Immune cell, Juvenile idiopathic arthritis (JIA), Mendelian randomization, Gene-wide Association, Causation analysis

## Abstract

**Background:**

Juvenile idiopathic arthritis (JIA) is a type of chronic childhood arthritis with complex pathogenesis. Immunological studies have shown that JIA is an acquired self-inflammatory disease, involving a variety of immune cells, and it is also affected by genetic and environmental susceptibility. However, the precise causative relationship between the phenotype of immune cells and JIA remains unclear to date. The objective of our study is to approach this inquiry from a genetic perspective, employing a method of genetic association analysis to ascertain the causal relationship between immune phenotypes and the onset of JIA.

**Methods:**

In this study, a two-sample Mendelian randomization (MR) analysis was used to select single nucleotide polymorphisms (SNPs) significantly associated with immune cells as instrumental variables to analyze the bidirectional causal relationship between 731 immune cells and JIA. There were four types of immune features (median fluorescence intensity (MFI), relative cellular (RC), absolute cellular (AC), and morphological parameters (MP)). Finally, the heterogeneity and horizontal reproducibility of the results were verified by sensitivity analysis, which ensured more robust results.

**Results:**

We found that *CD3 on CM CD8br* was causally associated with JIA at the level of 0.05 significant difference (95% CI = 0.630 ~ 0.847, *P* = 3.33 × 10^−5^, P_FDR_ = 0.024). At the significance level of 0.20, two immunophenotypes were causally associated with JIA, namely: *HLA DR on CD14+ CD16- monocyte* (95% CI = 0.633 ~ 0.884, *P* = 6.83 × 10^–4,^ P_FDR_ = 0.16) and *HLA DR on CD14+ monocyte* (95% CI = 0.627 ~ 0.882, *P* = 6.9 × 10^−4^, P_FDR_ = 0.16).

**Conclusion:**

Our study assessed the causal effect of immune cells on JIA from a genetic perspective. These findings emphasize the complex and important role of immune cells in the pathogenesis of JIA and lay a foundation for further study of the pathogenesis of JIA.

**Supplementary Information:**

The online version contains supplementary material available at 10.1186/s12969-024-00970-8.

## Background

Juvenile arthritis (JA) refers to arthritis that occurs before the age of 16, which has been reclassified as internationally recognized juvenile idiopathic arthritis (JIA) [1] JIA is the most common chronic rheumatic disease in pediatrics, with a prevalence rate of about 1 million. Characterized primarily by peripheral arthritis, JIA poses a substantial disability risk in children, potentially affecting multiple body systems [2]. Juvenile-psoriatic arthritis (JPA), enteritis-related arthritis (ERA), oligo-arthritis, rheumatoid factor (RF)-negative polyarthritis, RF-positive polyarthritis, and undifferentiated arthritis are the seven types recognized under the current classification of JIA [3]. Its pathogenesis is very complex, involving not only immune cells, but also various types of parenchymal cells. In addition, it will be affected by genetic and environmental factors. In the past 5 years of clinical research, substantial progress has been made in various fields, from disease classification to new treatments [4]. JIA is involved in synovitis and destruction of joint tissue, and no blood tests have been found to diagnose JIA. Hence, diagnosing JIA becomes challenging for children initially reporting pain and joint swelling [5]. Given that JIA patients experience immunosuppression from drug use, increasing the risk of cardiovascular disease infection, the condition exhibits elevated morbidity and mortality. Although a comprehensive understanding of JIA’s pathogenesis remains elusive, some studies have affirmed the intricate relationship between immune-related factors and JIA [5, 6].

JIA is recognized as an autoimmune inflammatory disease where the contribution of the immune system holds undeniable significance. Within adaptive immunity, several factors are acknowledged to initiate and perpetuate the persistent and chronic characteristics of JIA [7]. The intricate nature of the immune system manifests in the diverse and adaptable immune cell subsets and their functional phenotypes. This intricacy implies that the diversity among immune cells may directly influence the pathogenesis and prognosis of JIA [8]. Therefore, investigating the relationship between immune cells and JIA is essential for a deeper understanding of the underlying mechanisms of the disease. The immune pathogenesis of JIA remains largely unknown, and the disease categories are intricate and heterogeneous, involving diverse contributions from immune system participants and effector cells [2, 9].According to certain research, adaptive immunity plays a major part in the pathophysiology of JIA and is therefore classified as an autoimmune illness [9, 10]. Joint swelling, inflammation, and tissue deterioration are the hallmarks of JIA. The pathophysiological mechanism of JIA is linked to the abnormal activation of immune system components, including T cells, B cells, natural killer cells, monocytes, dendritic cells, plasma cells and so on. Additionally, the synthesis and release of pro-inflammatory mediators such as chemokines, cytokines, matrix metalloproteinases, and cathepases contribute to this process. These processes ultimately result in systemic symptoms as well as the deterioration of bone and cartilage. JIA is often accompanied by synovial hypertrophy and pathological angiogenesis, which is due to excessive synovial cell proliferation, immune cell infiltration, intra-articular hypoxia, increased blood flow and other reasons, which will eventually lead to the migration of pro-inflammatory cells to the joint. Overall, chondrocytes and osteoclasts are stimulated by the complex cellular network and the production of inflammatory mediators in the synovium of JIA, which results in the erosion of cartilage and bone [11–13].

The identification of genetic variants, particularly SNPs, that are linked to diseases has been made possible by genome-wide association studies (GWAS). Our understanding of the genetic basis of many complicated features in human diseases has been greatly improved by this [14]. Mendelian randomization (MR) leverages genetic variations linked to an exposure as a proxy for that exposure, enabling the exploration of causal relationships between exposure and outcome [15]. The selected SNPs, termed instrumental variance (IV), serve as key components in this approach. Two-sample MR mimics randomization by employing a random distribution of genetic variation during gametogenesis, akin to the conceptual framework of a randomized controlled trial [16]. Since these genetic variations are independent of postnatal lifestyle and environmental factors and precede the development of disease, MR can reduce the influence of confounding variables and reverse causality.

In our present study, we utilized a recently acquired GWAS database to conduct a two-sample MR analysis, aiming to elucidate potential causal links between immune cells and JIA.

## Methods

### Study design

**Fig. 1:**
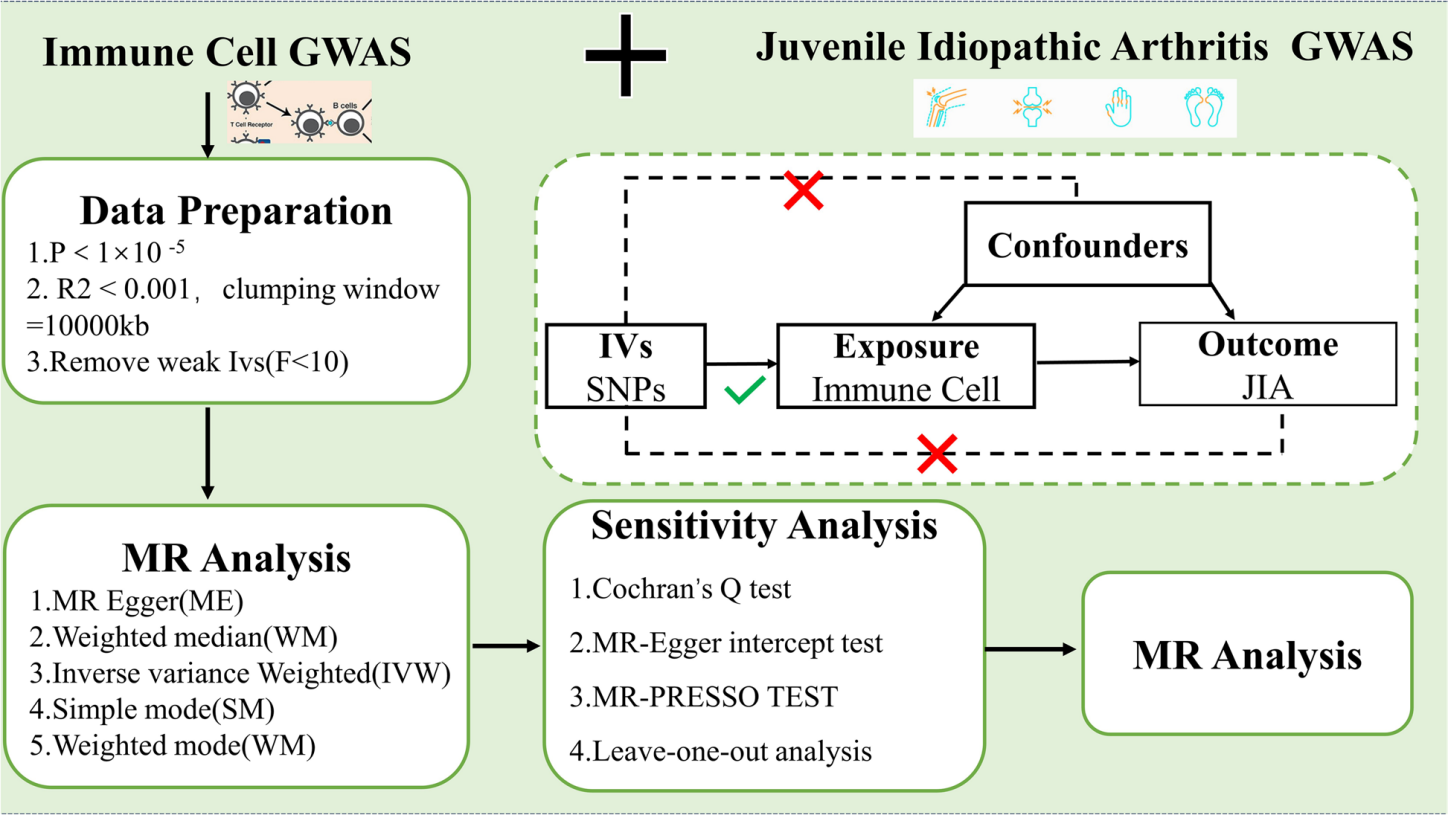
Overview of present MR analyses and study design. MR, Mendelian randomization; GWAS, Genome-wide association studies; JIA, juvenile idiopathic arthritis; IV, Instrumental Variance; SNP, Single Nucleotide Polymorphism

We carried out a thorough analysis of the causal link between 731 immune cell profiles classified into seven categories and JIA. Based on a two-sample MR study, which uses genetic variation as a stand-in for risk factors, this assessment was conducted. Within the framework of causal inference, legitimate IVs need to satisfy three fundamental presumptions: (1) genetic variation is a direct correlate of the exposure; (2) genetic variation does not impact the outcome via pathways besides the exposure (3) genetic variation is not connected to potential confounders between the exposure and the outcome. Figure 1 shows the study design procedure.

### Exposure data

In order to draw more comprehensive and reliable conclusions about the causal relationship between immune markers and JIA, we chose the largest peripheral blood immunophenotype GWAS published to date. The GWAS catalog provides summary statistics of GWAS with immunological characteristics. The entry numbers for these studies range from GCST0001391 to GCST0002121 [17]. It included 119 absolute cell counts, 192 relative cell counts, 32 morphological parameters (MP) and 389 median fluorescence intensity (MFI) representing surface antigen level. B cells, mature T cell stage, myeloid cells, monocytes, TBNK (T cells, B cells, natural killer cells) and Treg panels are specifically included in MFI, AC and RC features. Panels from TBNK and CDC make up the MP function.

Three thousand six hundred fifty-seven European people’s data, from non-overlapping cohorts, were included in the initial GWAS on immunological characteristics. A reference panel based on the Sardinian sequence was used to impute 21 million SNPs that were genotyped using high-density arrays [18]. Following covariate adjustments for age, sex, and age squared, associations were examined.

### Outcome data

JIA GWAS summary data were obtained from FinnGen biobank (finn-b-JUVEN_ARTHR). The study performed a GWAS on 173,622 European individuals (Ncase = 788, Ncontrol = 172,834), with approximately 16,380,296 variants analyzed after quality control and imputation.

### Instrumental variable

The selection criteria are as follows: considering the limited number of SNP available, we use SNP under the condition of *p* < 1e-05, which is widely used when the number of SNP available is limited [19, 20]. Based on the sample data of 1000 European Genome Project samples as reference data, we gathered the SNP in 10,000 kb under the threshold of linkage disequilibrium (LD) R^2^ < 0.001, and only the SNP with the lowest *P* value was retained. At the same time, we also calculate the F statistics of each SNP to quantify the statistical strength, and discard the SNP with F < 10. After harmonization, we aligned the effect alleles of exposure and outcome SNPs, excluding those with incompatible alleles (e.g., A/C paired with A/G) or exhibiting a palindromic pattern with intermediate allele frequency.

### Statistical analysis

Causal relationships between immune cells and JIA were analyzed by MR. When an MR analysis results in *P* < 0.05, it suggests that there is a causal relationship between immune cells and JIA. In this study, we used five causal detection methods: inverse variance weighting (IVW), weighted median estimation, MR-Egger regression, simple mode, and weighted mode [21]. These methods of causal reasoning have their own modeling construction. In the absence of horizontal pleiotropy, the IVW method is the primary method to analyze causality in MR analysis [22]. The application of the weighted median method assumes that less than 50% of the IVs show horizontal multiplicity. When heterogeneity occurs and there is no horizontal pleiotropy, the weighted median method can be considered [23]. MR-Egger regression assumes that more than 50% of IVs may be affected by horizontal pleiotropy. The simple mode represents an estimation approach based on the effects of individual genetic variations, which is suitable when data exhibit heterogeneity but lack consistent bias. Weighted mode, like the simple model, assigns weights to each effect estimate. When most research results carry similar weights, the weighted model becomes a viable option.

We used Cochran’s Q test to assess the heterogeneity of the IVs. If heterogeneity was detected (*P* < 0.05), a random effects IVW model could provide a more conservative estimate [24]. The MR-Egger and MR Pleiotropy RESidual Sum and Outlier (MR-PRESSO) tests were used to test horizontal multiplicity and outliers. MR-Egger is specifically used to initially determine the existence of horizontal multiplicity. If *P* > 0.05, it indicates that there is no significant horizontal pleiotropy. Subsequently, the leave-one-out sensitivity analysis was used to test the outliers and the stability of the results. Detailed results and original data are shown in Supplementary Tables S1/S2/S3/S4/S5/S6/S7 and File S1.

For *p*-value correction to address potential false positives, the FDR (Benjamini-Hochberg) method was applied.

All the above analyses are based on the R program (version 4.3.1) loading “VariantAnnotation” package, “mrcieu/gwasglue” package, “mrcieu/ieugwasr” package and “MRCIEU/TwoSampleMR” package. (The related R language script has been uploaded to the supplementary file).

## Result

### Effect of genetically predicted immune cell phenotypes on JIA

**Fig. 2:**
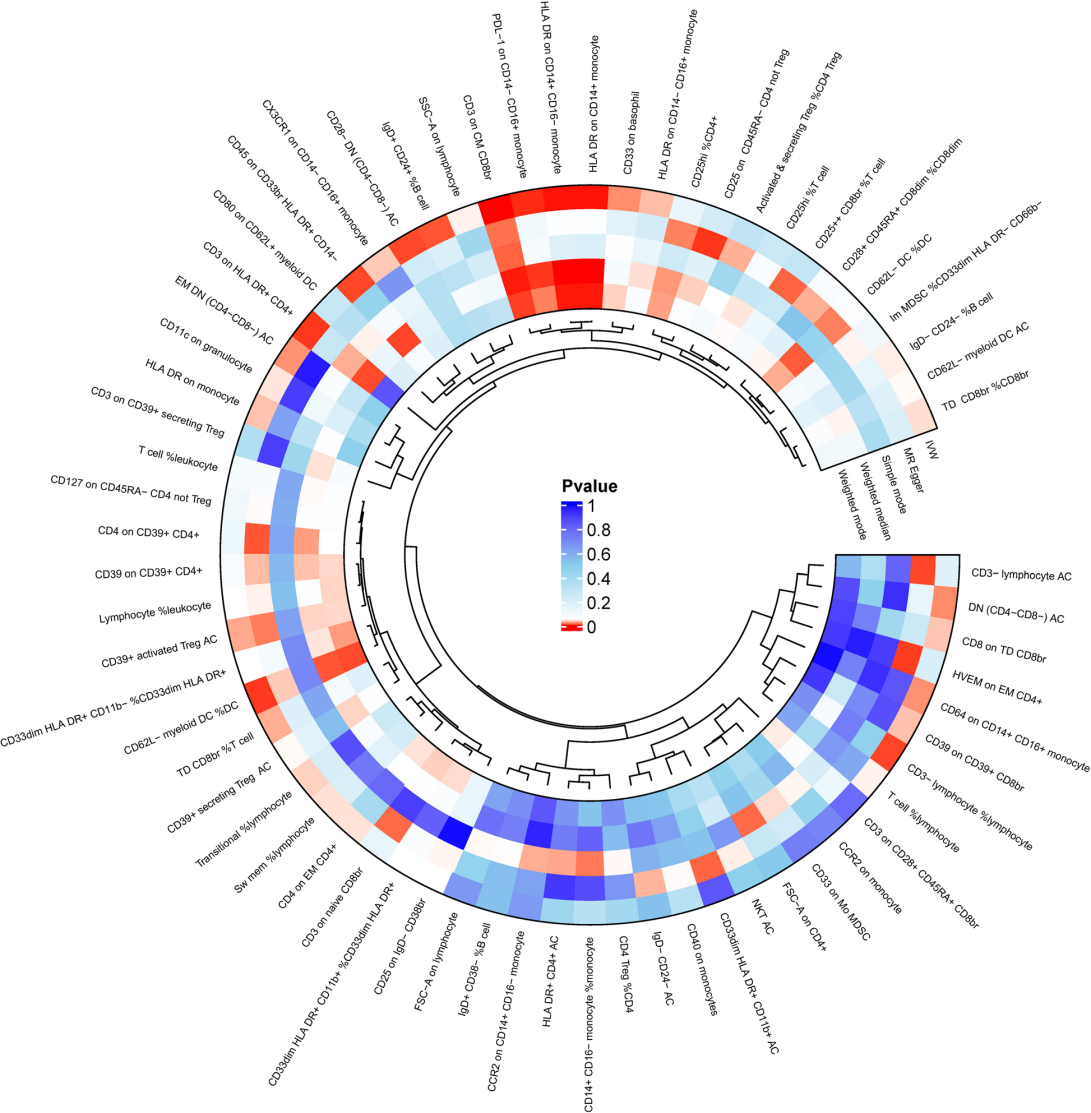
MR Analysis Circle Diagram of Immune Cells and JIA. The outermost circle indicates the name of the immune cell. The five circles in the center represent the corresponding MR assays for each immune cell, and the five circles represent five MR assays

In order to explore the causal effect of immunophenotype on JIA, two-sample MR analysis was carried out, and five detection methods were used, of which IVW method was the main analysis basis, and the results were shown in Fig. 2. In order to avoid the occurrence of false positive results, we use FDR method for multiple tests. From the statistical results of Fig. 2 and Table 1, We found that only *CD3 on CM CD8br* was causally associated with JIA at the level of 0.05 significant difference (95% CI = 0.630 ~ 0.847, *P* = 3.33 × 10^−5^, P_FDR_ = 0.024).

**Table 1:** MR results of causal relationships between immune cells and JIA

Outcome	Exposure	No. SNP	P_IVW_	P_FDR_	OR	95% CI	Horizontal pleiotropy			Heterogeneity	
							Egger intercept	SE	*P*-value	Cochran’s Q	*P*-value
**JIA**	CD3 on CM CD8br	18	3.33e-05	0.024**	0.731	0.63–0.848	0.028	0.035	0.437	13.447	0.706
	HLA DR on CD14+ CD16- monocyte	22	0.000683	0.168*	0.748	0.633–0.885	0.012	0.052	0.821	40.645	0.006
	HLA DR on CD14+ monocyte	21	0.00069	0.168*	0.743	0.627–0.883	0.011	0.053	0.826	38.655	0.007

FDR, FDR, false negative discovery rate, Benjamini-Hochberg method was used for adjustment

*SNP* Single Nucleotide Polymorphism: *OR* odds ratio: *CI* confidence interval

*Indicates significant difference in P_FDR_ at 0.2 level

**indicates significant difference in P_FDR_ at 0.05 level

**Fig. 3:**
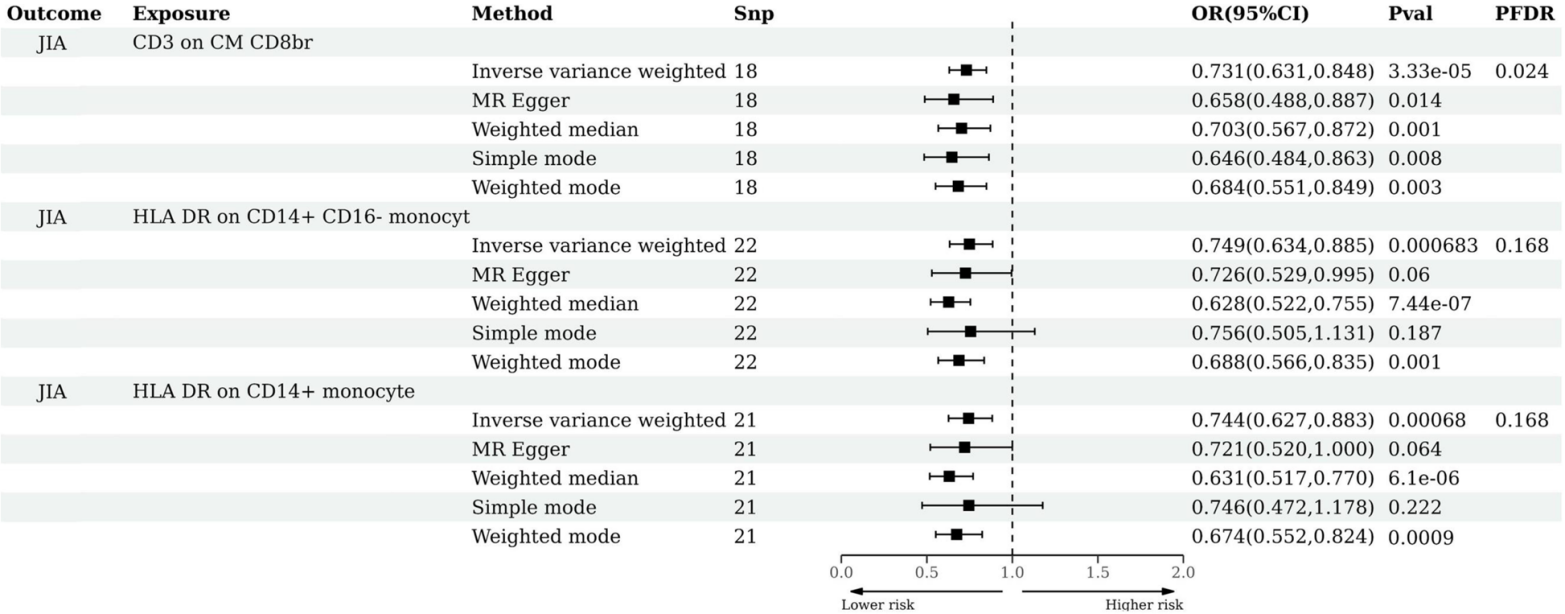
Forest plot of MR analysis results of immune cells and JIA. JIA, juvenile idiopathic arthritis; SNP, Single Nucleotide Polymorphism; OR, odds ratio; CI, confidence interval; FDR, false negative discovery rate, Benjamini-Hochberg method was used for adjustment

At the significance level of 0.20, two immunophenotypes were causally associated with JIA. They are: *HLA DR on CD14+ CD16- monocyte* (95% CI = 0.633 ~ 0.88, *P* = 6.83 × 10^−4^, P_FDR_ = 0.16) and *HLA DR on CD14+ monocyte* (95% CI = 0.627 ~ 0.882, *P* = 6.9 × 10–4, P_FDR_ = 0.16). However, we found that the heterogeneity test results of *HLADRonCD14 + CD16-monocyte* and *HLADRonCD14 + monocyte* were both less than 0.05, indicating that there may be heterogeneity in tool variables from different analysis platforms, or from different experiments, or between different populations. The weighted median method may also be considered when only heterogeneity is present, without horizontal pleiotropy. According to Fig. 3, it is evident that their MR analysis results with JIA show *P* < 0.05 for both IVW and weighted median methods. After FDR correction, the values become *P* < 0.2 for IVW and P < 0.05 for the weighted median method. Therefore, we still consider *HLA DR on CD14 + CD16-monocyte* and *HLA DR on CD14+ monocyte* as the positive results. It is worth mentioning that both *CD3 on CM CD8br*, *HLA DR on CD14 + CD16-monocyte* and *HLA DR on CD14+ monocyte* were causally associated with JIA. The test results of horizontal pleiotropy and heterogeneity are shown in Table 1.

**Fig. 4:**
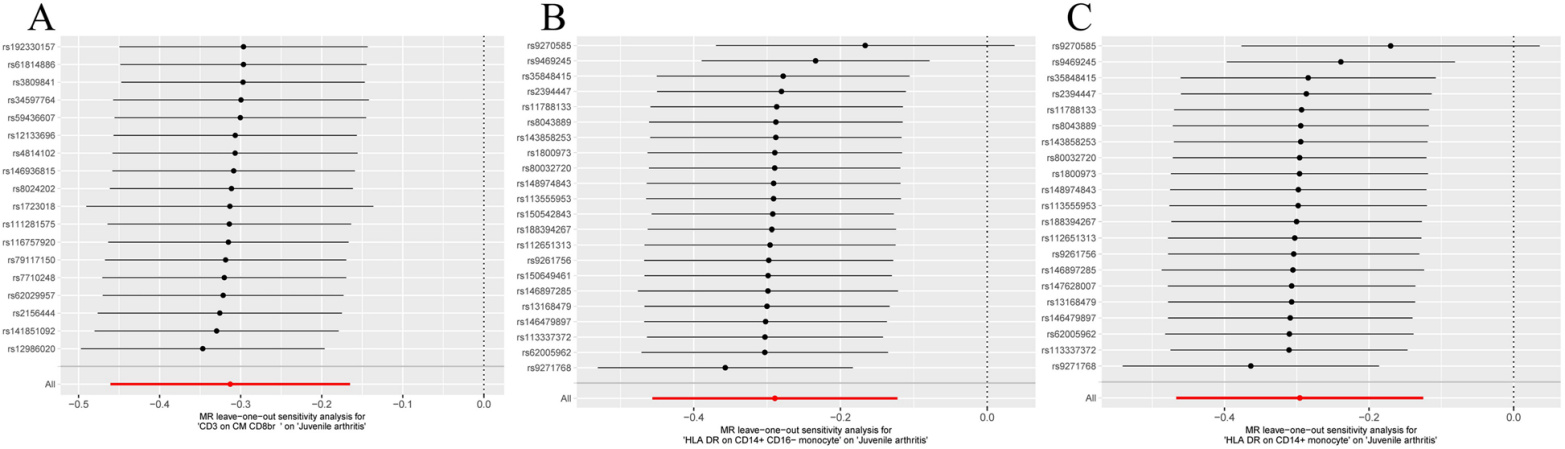
Scatter plot of causality between immune cell and risk of JIA. The X axis represents the effect value of SNP and the Y axis represents the effect value of JIA. **A** *CD3 on CM CD8br* on JIA. **B** *HLA DR on CD14+ CD16- monocyte* on JIA. **C** *HLA DR on CD14+ monocyte* on JIA

**Fig. 5:**
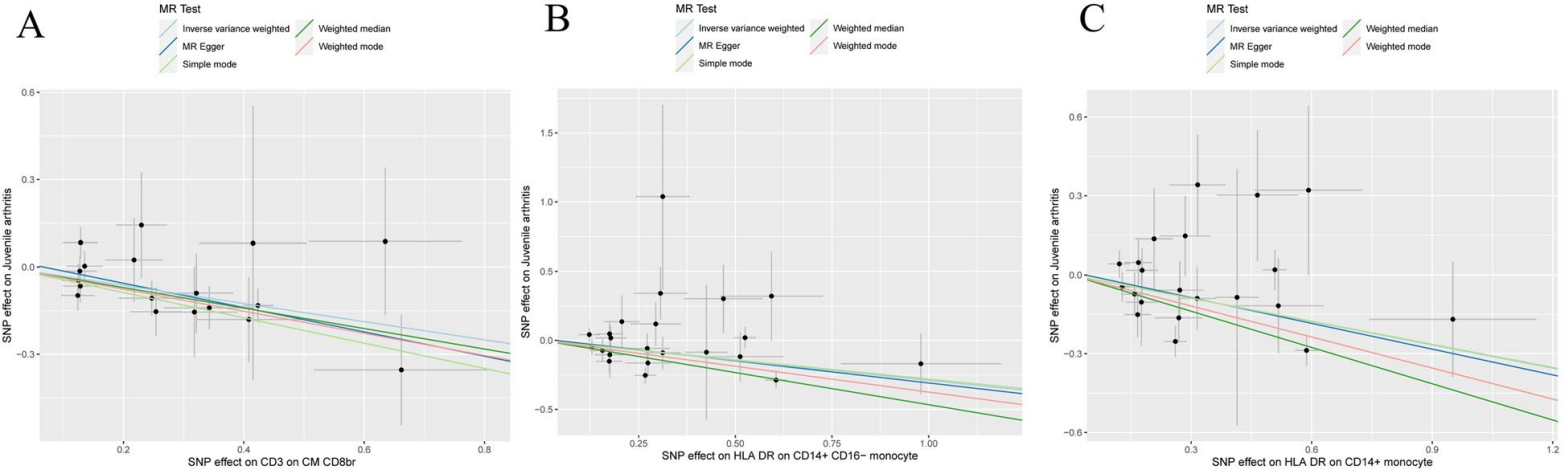
Leave-one-out plot to visualize causal effect of immune cell on JIA risk when leaving one SNP out. Leave-one-out Single nucleotide polymorphism sensitivity analysis of immunocyte phenotype and JIA; The Y-axis represents the IDs of SNPs omitted from the analysis, and the X-axis represents MR odds ratios; The red lines represent MR estimates that include all SNPs; **A** CD3 on CM CD8br on JIA; **B** HLA DR on CD14+ CD16- monocyte on JIA; **C** HLA DR on CD14+ monocyte on JIA

SNPs are strong IVs. The causal effect of each genetic variation on JIA is shown in Fig. 4. As shown in Fig. 5, Leave-one-out sensitivity analysis. We systematically repeated the MR analysis for the remaining SNPs after removing one SNP at a time, and the combined effect size was unaffected, indicating that the calculation of all SNPs made the causal relationship significant.

### Effect of genetically predicted JIA on immune cell phenotypes

Reverse two-sample MR analysis was performed with IVW as the primary detection method in order to investigate the causal relationship between JIA and immunophenotype. There was no correlation between JIA and immunophenotype (P_FDR_ > 0.2) when multiple tests were taken into account using the FDR technique. The statistical results can be found in the supplementary Table S5. Reverse MR analysis result.

## Discussion

Leveraging extensive genetic data from published sources, we investigated the causal connection between 731 immune cell traits and JIA. This may be the first time to use MR analysis to study the causal relationship between multiple immunophenotypes and JIA. Notably, we observed a significant causal impact of *CD3 on CM CD8br* on JIA (P_FDR_ < 0.05) in this study. And, the higher the number of CD3 on CM CD8br, the better the prognosis for JIA. *HLA DR on CD14+ CD16-monocyte* and *HLA DR on CD14+ monocyte* also were causally associated with JIA (P_FDR_ < 0.2). Therefore, our study suggests that *CD3 on CM CD8br, HLA DR on CD14+ CD16-monocyte,* and *HLA DR on CD14+ monocyte* may be a kind of phenotype that has a palliative effect on JIA. In order to ensure the reliability and robustness of the research results, we also carried out a sensitivity test of the MR results. As shown in Fig. 5 and Table 1, there is no horizontal multiplicity in the MR results, and the sensitivity test of the Leave-one-out method also indicates that the effect values of all SNP are stable.

The pathogenesis of JIA is intricate, involving diverse immune cells and cytokines. Research indicates a correlation between the dysregulation of T lymphocyte subsets and the occurrence of both local inflammation and systemic immune responses in arthritis [25]. Disruption of the dynamic balance and activation of the immune system elevate susceptibility to autoimmune diseases. The equilibrium between CD3 and CD8+ cells is intricately linked to the onset and progression of autoimmune diseases, a relationship influenced by disease activity [26]. Differentiation antigen 3 (CD3) is a crucial leukocyte differentiation antigen present on nearly all T cells. In arthritis, CD3 expression was observed in lymphocytes within synovial tissue, with staining detected on the cell membrane and/or cytoplasm, yielding a total lymphocyte positivity rate of 72%. Rheumatoid arthritis exhibits high CD3 expression, whereas osteoarthritis shows lower levels. This suggests a potential association between CD3 + T lymphocyte count and the pathogenesis and course of rheumatoid arthritis and osteoarthritis [27]. Functioning as a T lymphocyte coreceptor, CD8 antigen comprises CD8 α and CD8 β chain subtypes encoded by CD8a and CD8b, respectively. CD8b influences osteolysis and the immune response in osteoarthritis, impacting osteoclast formation [28]. Differentiation antigen 8 (CD8) + T cells possess cytotoxic effects and can modulate cellular and humoral immunity [29, 30]. CD8+ lymphocytes disperse and infiltrate into synovial tissue diffusely [31]. Similar to CD3+, they show low expression in osteoarthritis. It was also found that the number of CD3 + CD8+ cell subsets decreased significantly in JIA [32], and CD8 cells proliferated significantly after methotrexate treatment [33]. These studies demonstrate a close association between the equilibrium of CD3+ and CD8+ cells and the onset and progression of JIA. It is noteworthy that these studies revealed significant distinctions between osteoarthritis and rheumatoid arthritis, providing valuable insights for distinguishing JIA from other forms of arthritis. In addition to their relevance to arthritis, CD3 and CD8+ are also specifically expressed in the context of tumor immune responses. In the study of colon cancer, Galon et al [34] thought that the high density of CD3 or CD8 positive immune cells predicted better survival rate. It is also found that compared with other biomarkers, CD3 seems to have the strongest prognostic value. In another study, the low density of CD3 and CD8 immune cells indicated a poor prognosis, but they were still equally strong prognostic markers [35]. This is similar to our results. Our study shows that the higher the count density of CD3 and CD8 immune cell subsets in JIA, the better the prognosis. Generally speaking, the specific effects of CD3 and CD8 on JIA may be affected by a variety of factors, including immune system regulation, inflammatory response and so on. In-depth research and clinical observation will help to better understand their role in JIA.

At the significant level of P_FDR_ < 0.2, there was a causal relationship between *HLA DR on CD14 + CD16- monocytes, HLADR on CD14+ monocytes* and JIA. The results of MR analysis show that both of them have heterogeneity, which may be related to the analysis platform, research population and other factors. Because there is only heterogeneity and no multiplicity in this study, we can comprehensively consider the results of IVW method and weighted median method. Therefore, *HLADR on CD14 + CD16- monocytes* and *HLADR on CD14+ monocytes* were also identified as positive indicators. HLA-DR (Human Leukocyte Antigen - DR isotype) is a major histocompatibility complex class II molecule that plays a crucial role in presenting antigens to T cells, initiating immune responses [36]. In the context of monocytes, the expression of HLA-DR is commonly utilized as a marker to assess the activation or maturation status of these cells. CD14 + CD16- monocytes, also referred to as classical monocytes, express CD14 but not CD16. Classical monocytes are typically associated with pro-inflammatory responses [37]. CD14+ monocytes may encompass a broader category of monocytes. The CD14++/CD16- subset constitutes 80% of circulating monocytes, demonstrating heightened cytokine production (IL1, IL6, TNF-α) and robust phagocytic activity [38]. CD14, capable of binding to Lipopolysaccharide (LPS) and viruses on the cell surface, serves not only as a transporter but also as an amplifier of Toll-like receptor responses. CD16 monocytes, known for TNF α production upon LPS stimulation, may undergo expansion during acute inflammation and infectious diseases, yet their precise function remains elusive [39]. The proportion of CD14++/CD16+ mononuclear cells increases in resting samples from JIA. During the eruption and resting phases of JIA, CD16 expression on CD14+/CD16+ monocytes is found to increase [40]. Even during the quiescent phase of JIA, elevated CD16 expression in CD14+/CD16+ monocytes suggests an inherent characteristic of JIA monocytes, potentially contributing to the maintenance of a pro-inflammatory environment in the absence of clinical manifestations. This increased CD16 expression may also play a role in the pro-inflammatory environment maintenance. An upsurge in circulating monocyte levels and an augmentation of CD16 surface levels on CD16+ monocyte subsets are observed in inflammatory responses [41, 42]. Another noteworthy observation related to monocytes is the enhanced expression of CD14 on the surface of CD14++/CD16- monocytes in systemic JIA and polyarticular JIA. This increased CD14 expression is associated with resistance to apoptosis, suggesting that apoptosis dysregulation may contribute to monocyte accumulation during JIA attacks [43]. Based on the above studies, alterations in the expression profiles of monocyte subsets, particularly CD14++/CD16- and CD14+/CD16+ monocytes, have been observed in patients with JIA. These changes are likely associated with the regulation of immune responses and the inflammatory microenvironment. The heightened expression of CD16 appears to be present during both the active and quiescent phases of JIA, suggesting its potential role in sustaining the inflammatory milieu. Additionally, the increased expression of CD14 on specific monocyte subsets is associated with anti-apoptotic effects, potentially contributing to the abnormal accumulation of monocytes during JIA flares. The correlation of HLADR with CD14 + CD16- and CD14+ monocytes further underscores its close association with the immune response in JIA. While our study reinforces these relationships, further validation through biological experiments is essential to confirm these findings.

This work also has some limitations. First of all, although it satisfies the MR hypothesis, it still cannot guarantee that there is no weak tool bias, which is a common phenomenon in this kind of research. Second, the study was limited to people of European origin. Although this helps to reduce the potential deviation from population structure, it limits the universality of MR results to other populations. In the future research, our goal is to expand the sample size as much as possible, to explore the relationship between immune cells and JIA at the biomolecular level, and to provide more theoretical support for understanding the mechanism of immune cells-JIA.

## Conclusion

We have proved the causal relationship between immunophenotype and JIA through comprehensive two-way MR analysis, but reverse MR analysis did not find that JIA has an effect on immunophenotype. Our study can only show the one-way effect of the immune system on JIA. Furthermore, our study has notably mitigated the impact of inherent confounding variables, reverse causality, and other factors, offering researchers a novel avenue to delve into the immunological mechanisms of JIA. The findings from our study not only extend the current knowledge base in JIA immunology but also furnish valuable insights for the prevention and immune intervention strategies related to JIA.

### Supplementary Information

**Additional File 1:** **Supplementary Table 1.** IVs used in MR analysis. **Supplementary Table 2.**  Full result of MR estimates. **Supplementary Table 3.** The heterogeneity of immune cell IVs. **Supplementary Table 4.** The horizontal pleiotropy of immune cell  IVs. **Supplementary Table 5.** Reverse MR analysis result. **Supplementary Table 6.** Exposure. **Supplementary Table 7.** Exposure.F.

**Additional File 2.** Leave-one-out analysis results.

**Additional File 3.** All R language coding programs.

## Data Availability

This study is based on summary statistics. GWAS data for immune cells and JIA are publicly available. Summary statistics used in this study are provided in supplementary documents. Detailed studies of these GWAS can be found in the original research papers.

## Abbreviations

JIA: juvenile idiopathic arthritis
MR: mendelian randomization
SNPs: single nucleotide polymorphisms
GWAS: genome-wide association studies
IV: instrumental variance
LD: linkage disequilibrium
IVW: inverse variance-weighted
MFI: median fluorescence intensity
RC: relative cellular
AC: absolute cellular
MP: morphological parameters
CD3: Differentiation antigen 3
CD8: Differentiation antigen 8
