# Supplementary material for: Gene association analysis to determine the causal relationship between immune cells and juvenile idiopathic arthritis
Source: Pediatr Rheumatol Online J. 2024 Mar 8;22:35. doi: 10.1186/s12969-024-00970-8 (PMC10921670; doi:10.1186/s12969-024-00970-8)
Supplement: Supplementary file 3 — Additional File 3. All R language coding programs. [file 12969_2024_970_MOESM3_ESM.docx]

**immuneMR.exposure.R**

library(TwoSampleMR)

inputFile="exposureID.txt"

setwd("C:\\Users\\lexb\\Desktop\\immuneMR\\03.exposure")

rt=read.table(inputFile, header=T, sep="\t", check.names=F)

outTab=data.frame()

for(id in rt$ID) {

expoData=extract_instruments(id,

p1 = 1e-5, p2 = 1e-5,

clump = T,

kb = 10000, r2 = 0.001)

outTab=rbind(outTab, expoData)

}

write.csv(outTab, file="exposure_data.csv", row.names=F)

**immuneMR.F.R**

library(ieugwasr)

inputFile="exposure_data.csv"

setwd("C:\\Users\\lexb\\Desktop\\immuneMR\\05.F")

dat=read.csv(inputFile, header=T, sep=",", check.names=F)

dat$R2<-(2*dat$beta.exposure*dat$beta.exposure*dat$eaf.exposure*(1-dat$eaf.exposure)/(2*dat$beta.exposure*dat$beta.exposure*dat$eaf.exposure*(1-dat$eaf.exposure)+2*dat$se.exposure*dat$se.exposure*dat$samplesize.exposure*dat$eaf.exposure*(1-dat$eaf.exposure)))

dat$F<-dat$R2*(dat$samplesize.exposure-2)/(1-dat$R2)

outTab=dat[as.numeric(dat$F)>10,]

write.csv(outTab, file="exposure.F.csv", row.names=F)

**immuneMR.IVWfilter.R**

mrFile="table.MRresult.csv"

pleFile="table.pleiotropy.csv"

setwd("C:\\Users\\84290\\Desktop\\184.immuneMR\\07.IVWfilter")

rt=read.csv(mrFile, header=T, sep=",", check.names=F)

ivw=data.frame()

for(immuneCell in unique(rt$exposure)){

immData=rt[rt$exposure==immuneCell,]

if(immData[immData$method=="Inverse variance weighted","pval"]<0.05){

if(sum(immData$or>1)==nrow(immData) | sum(immData$or<1)==nrow(immData)){

ivw=rbind(ivw, immData)

}

}

}

pleRT=read.csv(pleFile, header=T, sep=",", check.names=F)

pleRT=pleRT[pleRT$pval>0.05,]

immuneLists=as.vector(pleRT$exposure)

outTab=ivw[ivw$exposure %in% immuneLists,]

write.csv(outTab, file="IVW.filter.csv", row.names=F)

**immuneMR.MR.R**

library(VariantAnnotation)

library(gwasglue)

library(TwoSampleMR)

exposureFile="exposure.F.csv"

outcomeID="finn-b-JUVEN_ARTHR"

outcomeName="Juvenile arthritis"

setwd("C:\\Users\\84290\\Desktop\\184.immuneMR\\06.MR")

exposure_dat=read_exposure_data(filename=exposureFile,

sep = ",",

snp_col = "SNP",

beta_col = "beta.exposure",

se_col = "se.exposure",

pval_col = "pval.exposure",

effect_allele_col="effect_allele.exposure",

other_allele_col = "other_allele.exposure",

eaf_col = "eaf.exposure",

phenotype_col = "exposure",

samplesize_col = "samplesize.exposure",

chr_col="chr.exposure", pos_col = "pos.exposure",

clump=FALSE)

outcomeData=extract_outcome_data(snps=exposure_dat$SNP, outcomes=outcomeID)

write.csv(outcomeData, file="outcome.csv", row.names=F)

outcomeData$outcome=outcomeName

dat=harmonise_data(exposure_dat, outcomeData)

outTab=dat[dat$mr_keep=="TRUE",]

write.csv(outTab, file="table.SNP.csv", row.names=F)

mrResult=mr(dat)

mrTab=generate_odds_ratios(mrResult)

write.csv(mrTab, file="table.MRresult.csv", row.names=F)

heterTab=mr_heterogeneity(dat)

write.csv(heterTab, file="table.heterogeneity.csv", row.names=F)

pleioTab=mr_pleiotropy_test(dat)

write.csv(pleioTab, file="table.pleiotropy.csv", row.names=F)

pdf(file="pic.scatter_plot.pdf", width=7.5, height=7)

mr_scatter_plot(mrResult, dat)

dev.off()

res_single=mr_singlesnp(dat)

pdf(file="pic.forest.pdf", width=7, height=5.5)

mr_forest_plot(res_single)

dev.off()

pdf(file="pic.funnel_plot.pdf", width=7, height=6.5)

mr_funnel_plot(singlesnp_results = res_single)

dev.off()

pdf(file="pic.leaveoneout.pdf", width=7, height=5.5)

mr_leaveoneout_plot(leaveoneout_results = mr_leaveoneout(dat))

dev.off()

**immuneMR.MRpic.R**

library(TwoSampleMR)

exposureFile="exposure.F.csv"

outcomeFile="outcome.csv"

sigImmuneFile="IVW.filter.csv"

outcomeName="Prolapsed or slipped disc"

setwd("C:\\Users\\84290\\Desktop\\184.immuneMR\\08.MRpic")

rt=read.csv(exposureFile, header=T, sep=",", check.names=F)

sigImmune=read.csv(sigImmuneFile, header=T, sep=",", check.names=F)

for(immuneCell in unique(sigImmune$exposure)){

i=gsub("\\%|\\/", "_", immuneCell)

singleExposureFile=paste0(i, ".exposure.csv")

exposure_set=rt[rt$exposure==immuneCell,]

write.csv(exposure_set, file=singleExposureFile, row.names=F)

exposure_dat=read_exposure_data(filename=singleExposureFile,

sep = ",",

snp_col = "SNP",

beta_col = "beta.exposure",

se_col = "se.exposure",

pval_col = "pval.exposure",

effect_allele_col="effect_allele.exposure",

other_allele_col = "other_allele.exposure",

eaf_col = "eaf.exposure",

phenotype_col = "exposure",

samplesize_col = "samplesize.exposure",

chr_col="chr.exposure", pos_col = "pos.exposure",

clump=FALSE)

outcome_data=read_outcome_data(snps=exposure_dat$SNP,

filename="outcome.csv", sep = ",",

snp_col = "SNP",

beta_col = "beta.outcome",

se_col = "se.outcome",

effect_allele_col = "effect_allele.outcome",

other_allele_col = "other_allele.outcome",

pval_col = "pval.outcome",

eaf_col = "eaf.outcome")

outcome_data$outcome=outcomeName

dat=harmonise_data(exposure_dat, outcome_data)

outTab=dat[dat$mr_keep=="TRUE",]

write.csv(outTab, file=paste0(i, ".table.SNP.csv"), row.names=F)

presso=run_mr_presso(dat)

write.csv(presso[[1]]$`MR-PRESSO results`$`Global Test`, file=paste0(i, ".table.MR-PRESSO_Global.csv"))

write.csv(presso[[1]]$`MR-PRESSO results`$`Outlier Test`, file=paste0(i, ".table.MR-PRESSO_Outlier.csv"))

mrResult=mr(dat)

mrTab=generate_odds_ratios(mrResult)

write.csv(mrTab, file=paste0(i, ".table.MRresult.csv"), row.names=F)

heterTab=mr_heterogeneity(dat)

write.csv(heterTab, file=paste0(i, ".table.heterogeneity.csv"), row.names=F)

pleioTab=mr_pleiotropy_test(dat)

write.csv(pleioTab, file=paste0(i, ".table.pleiotropy.csv"), row.names=F)

pdf(file=paste0(i, ".scatter_plot.pdf"), width=7, height=6.5)

p1=mr_scatter_plot(mrResult, dat)

print(p1)

dev.off()

res_single=mr_singlesnp(dat)

pdf(file=paste0(i, ".forest.pdf"), width=6.5, height=5)

p2=mr_forest_plot(res_single)

print(p2)

dev.off()

pdf(file=paste0(i, ".funnel_plot.pdf"), width=6.5, height=6)

p3=mr_funnel_plot(singlesnp_results = res_single)

print(p3)

dev.off()

pdf(file=paste0(i, ".leaveoneout.pdf"), width=6.5, height=5)

p4=mr_leaveoneout_plot(leaveoneout_results = mr_leaveoneout(dat))

print(p4)

dev.off()

}

**immuneMR.reverse.R**

library(TwoSampleMR)

exposureID="finn-b-JUVEN_ARTHR"

outcomeFile="exposureID.txt"

setwd("C:\\Users\\84290\\Desktop\\184.immuneMR\\10.reverse")

exposure_dat <- extract_instruments(exposureID, p1=5e-08, p2=5e-08, clump=TRUE)

rt=read.table(outcomeFile, header=T, sep="\t", check.names=F)

for(i in unique(rt$ID)){

outcome_dat <- extract_outcome_data(snps=exposure_dat$SNP, outcomes=i)

dat <- harmonise_data(exposure_dat, outcome_dat)

#presso=run_mr_presso(dat)

#write.csv(presso[[1]]$`MR-PRESSO results`$`Global Test`, file=paste0(i, ".table.MR-PRESSO_Global.csv"))

#write.csv(presso[[1]]$`MR-PRESSO results`$`Outlier Test`, file=paste0(i, ".table.MR-PRESSO_Outlier.csv"))

mrResult=mr(dat)

mrTab=generate_odds_ratios(mrResult)

if(mrResult$pval[3]<0.05){

if(sum(mrTab$or>1)==nrow(mrTab) | sum(mrTab$or<1)==nrow(mrTab)){

outTab=dat[dat$mr_keep=="TRUE",]

write.csv(outTab, file=paste0(i, ".table.SNP.csv"), row.names=F)

write.csv(mrTab, file=paste0(i, ".table.MRresult.csv"), row.names=F)

heterTab=mr_heterogeneity(dat)

write.csv(heterTab, file=paste0(i, ".table.heterogeneity.csv"), row.names=F)

pleioTab=mr_pleiotropy_test(dat)

write.csv(pleioTab, file=paste0(i, ".table.pleiotropy.csv"), row.names=F)

pdf(file=paste0(i, ".scatter_plot.pdf"), width=7, height=6.5)

p1=mr_scatter_plot(mrResult, dat)

print(p1)

dev.off()

res_single=mr_singlesnp(dat)

pdf(file=paste0(i, ".forest.pdf"), width=6.5, height=5)

p2=mr_forest_plot(res_single)

print(p2)

dev.off()

pdf(file=paste0(i, ".funnel_plot.pdf"), width=6.5, height=6)

p3=mr_funnel_plot(singlesnp_results = res_single)

print(p3)

dev.off()

pdf(file=paste0(i, ".leaveoneout.pdf"), width=6.5, height=5)

p4=mr_leaveoneout_plot(leaveoneout_results = mr_leaveoneout(dat))

print(p4)

dev.off()

}

}

}
