# Supplementary material for: Gene association analysis to determine the causal relationship between immune cells and juvenile idiopathic arthritis
Source: Pediatr Rheumatol Online J. 2024 Mar 8;22:35. doi: 10.1186/s12969-024-00970-8 (PMC10921670; doi:10.1186/s12969-024-00970-8)
Supplement: Supplementary file 2 — Additional File 2. Leave-one-out analysis results. [file 12969_2024_970_MOESM2_ESM.pdf]

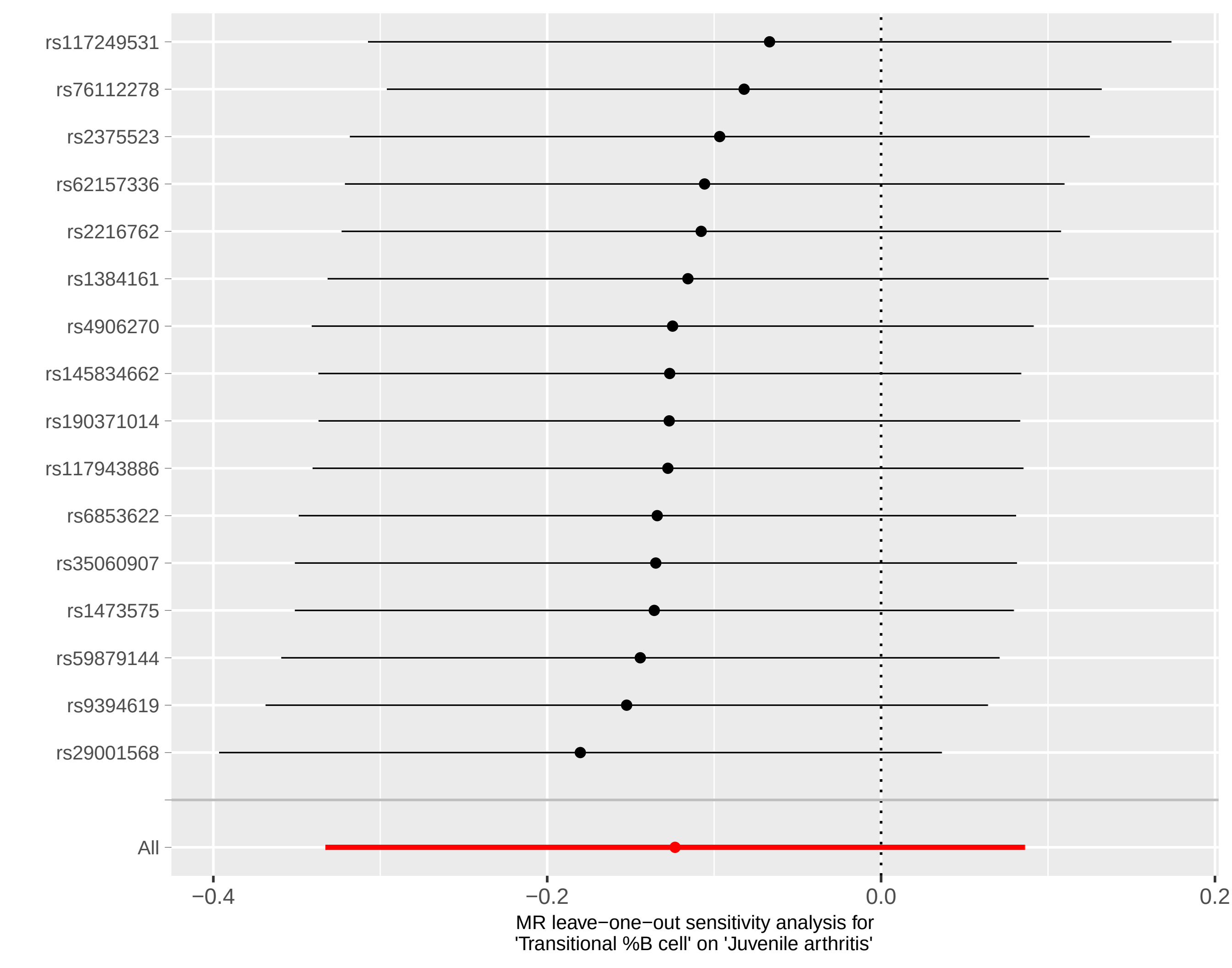
rs117249531
rs76112278
rs2375523
rs62157336
rs2216762
rs1384161
rs4906270
rs145834662
rs190371014
rs117943886
rs6853622
rs35060907
rs1473575
rs59879144
rs9394619
rs29001568
All
−0.4
−0.2
0.0
0.2
MR leave−one−out sensitivity analysis for
'Transitional %B cell' on 'Juvenile arthritis'

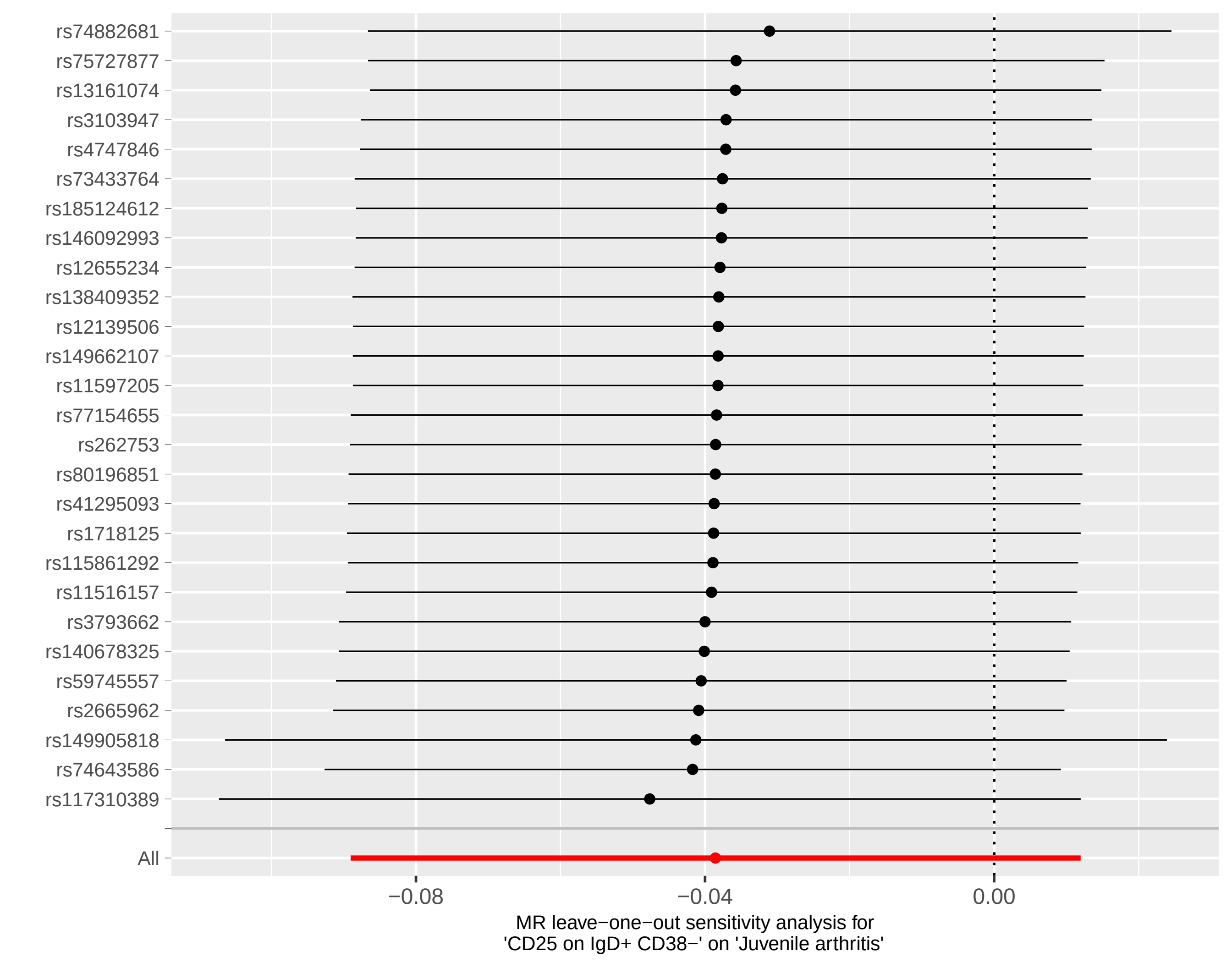
rs74882681
rs75727877
rs13161074
rs3103947
rs4747846
rs73433764
rs185124612
rs146092993
rs12655234
rs138409352
rs12139506
rs149662107
rs11597205
rs77154655
rs262753
rs80196851
rs41295093
rs1718125
rs115861292
rs11516157
rs3793662
rs140678325
rs59745557
rs2665962
rs149905818
rs74643586
rs117310389
All
−0.08
−0.04
0.00
MR leave−one−out sensitivity analysis for
'CD25 on IgD+ CD38−' on 'Juvenile arthritis'

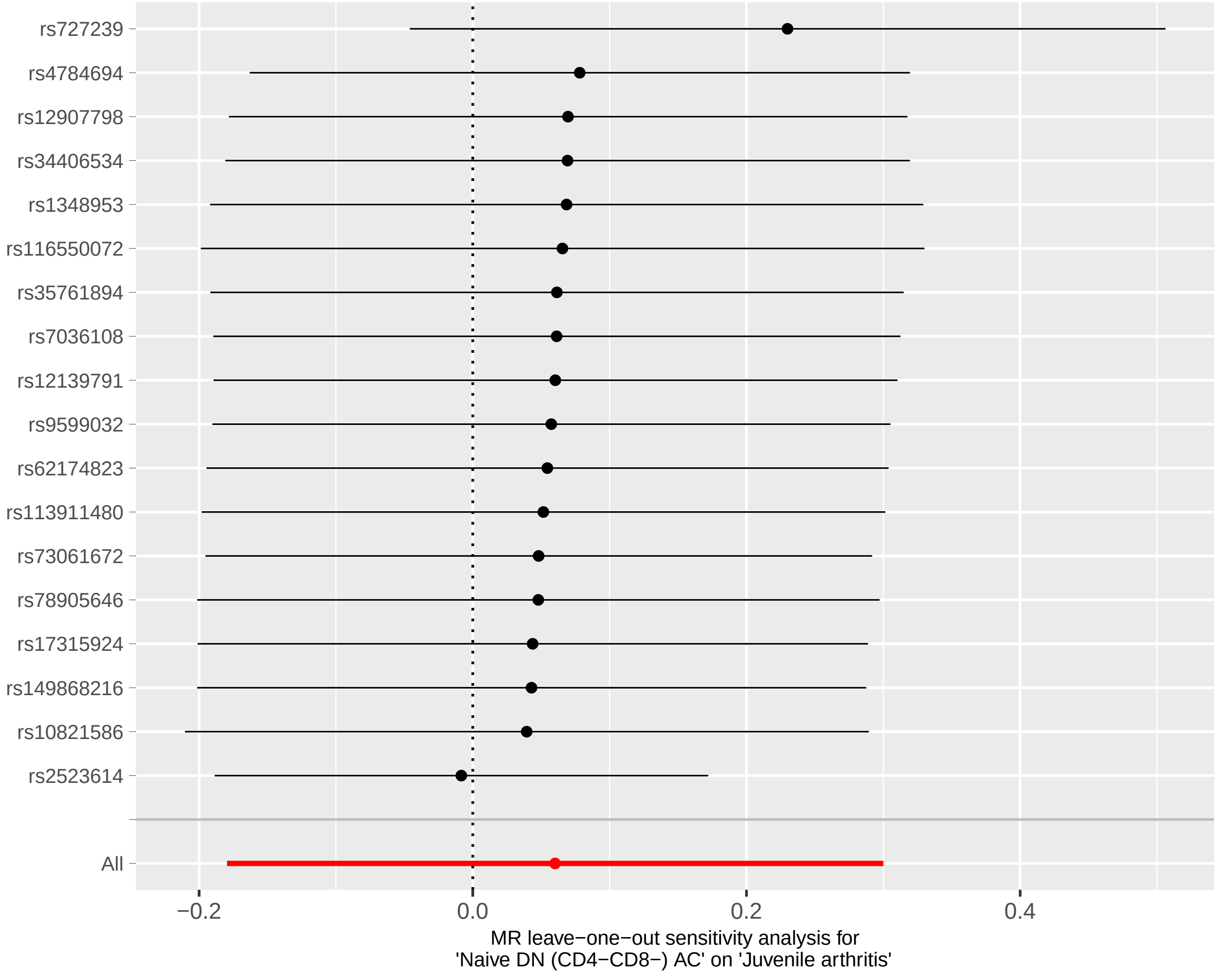
rs727239
rs4784694
rs12907798
rs34406534
rs1348953
rs116550072
rs35761894
rs7036108
rs12139791
rs9599032
rs62174823
rs113911480
rs73061672
rs78905646
rs17315924
rs149868216
rs10821586
rs2523614
All
−0.2
0.0
0.2
0.4
MR leave−one−out sensitivity analysis for
'Naive DN (CD4−CD8−) AC' on 'Juvenile arthritis'

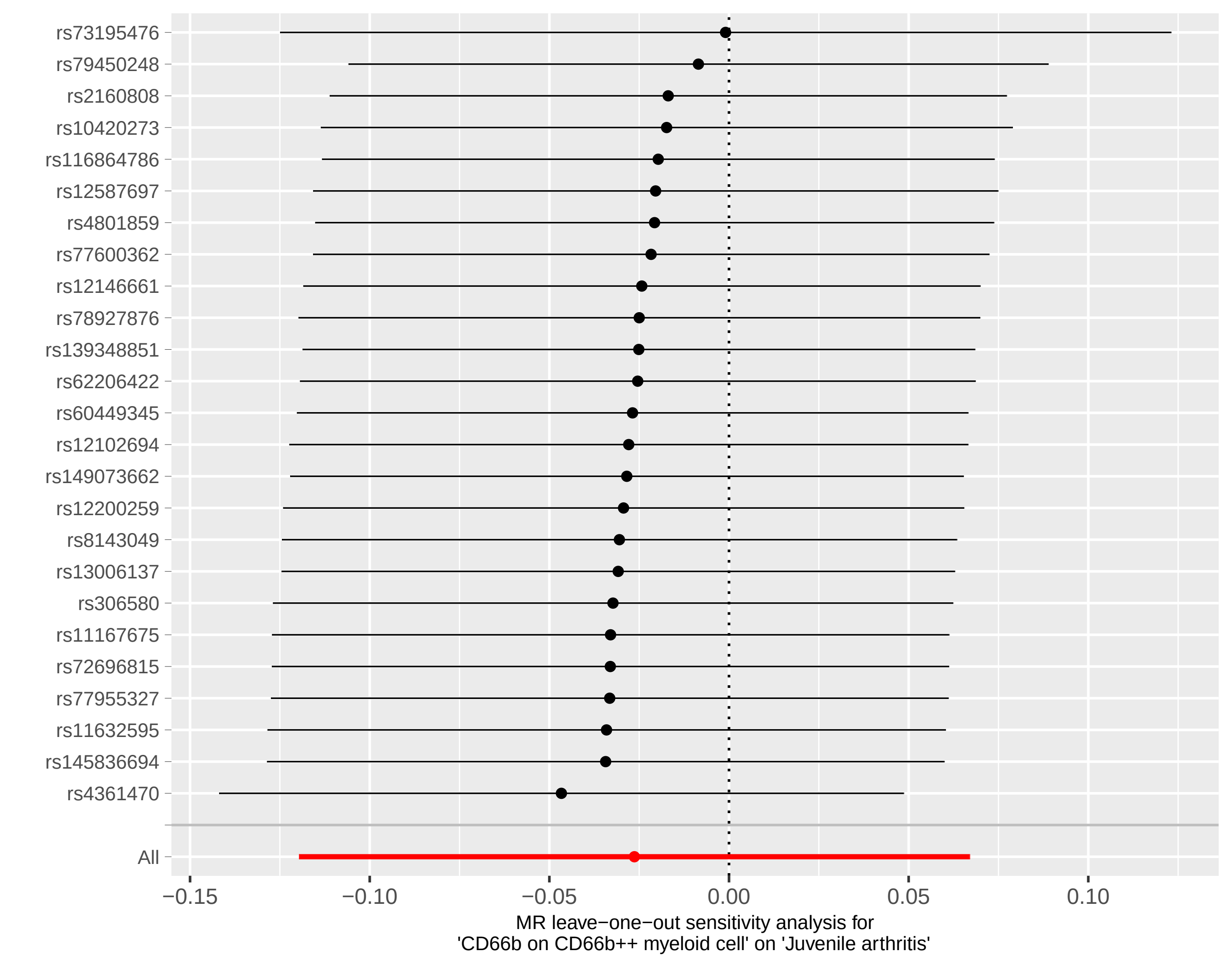

rs73195476
rs79450248
rs2160808
rs10420273
rs116864786
rs12587697
rs4801859
rs77600362
rs12146661
rs78927876
rs139348851
rs62206422
rs60449345
rs12102694
rs149073662
rs12200259
rs8143049
rs13006137
rs306580
rs11167675
rs72696815
rs77955327
rs11632595
rs145836694
rs4361470
All
−0.15
−0.10
−0.05
0.00
0.05
0.10
MR leave−one−out sensitivity analysis for
'CD66b on CD66b++ myeloid cell' on 'Juvenile arthritis'

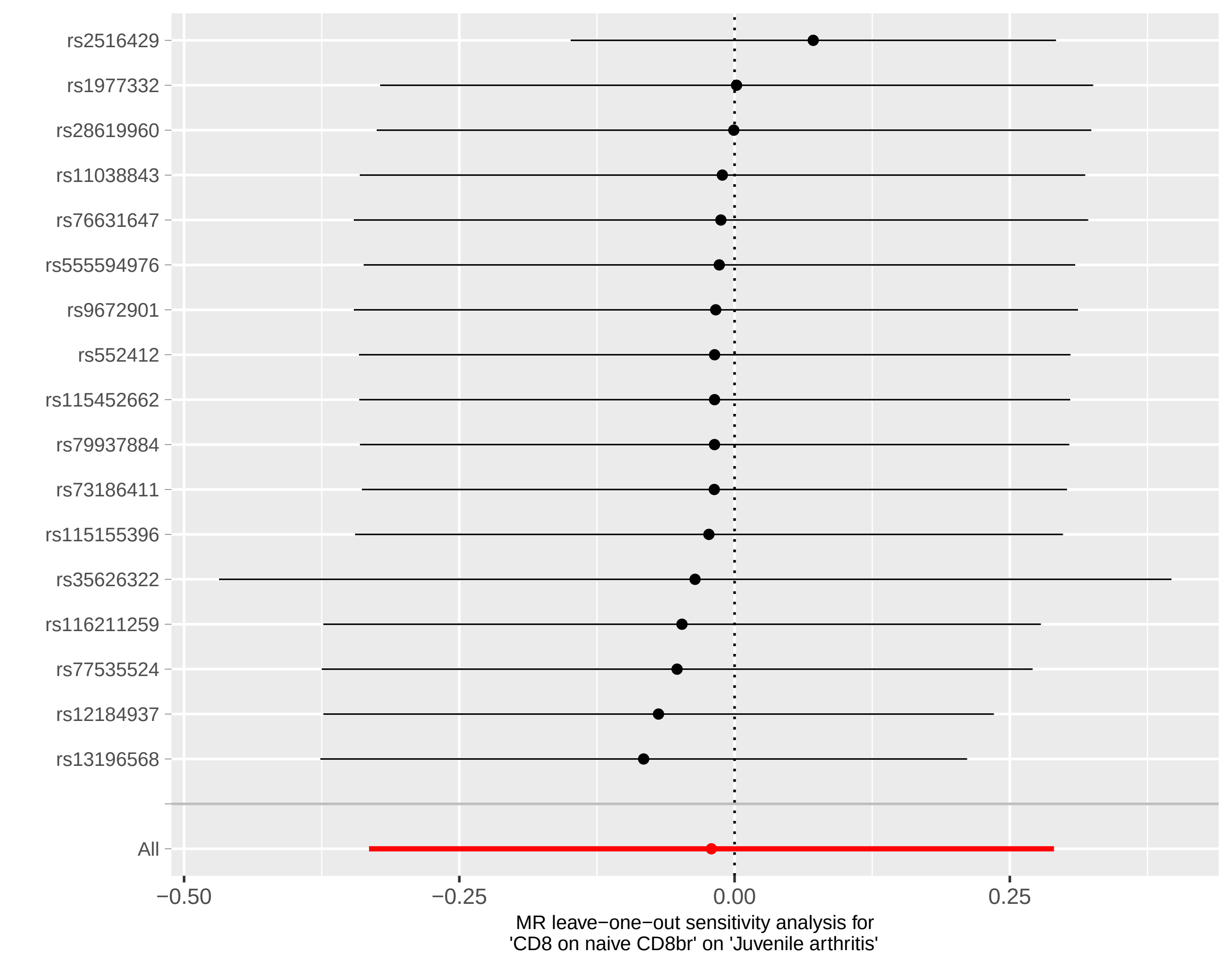
rs2516429
rs1977332
rs28619960
rs11038843
rs76631647
rs555594976
rs9672901
rs552412
rs115452662
rs79937884
rs73186411
rs115155396
rs35626322
rs116211259
rs77535524
rs12184937
rs13196568
All
−0.50
−0.25
0.00
0.25
MR leave−one−out sensitivity analysis for
'CD8 on naive CD8br' on 'Juvenile arthritis'

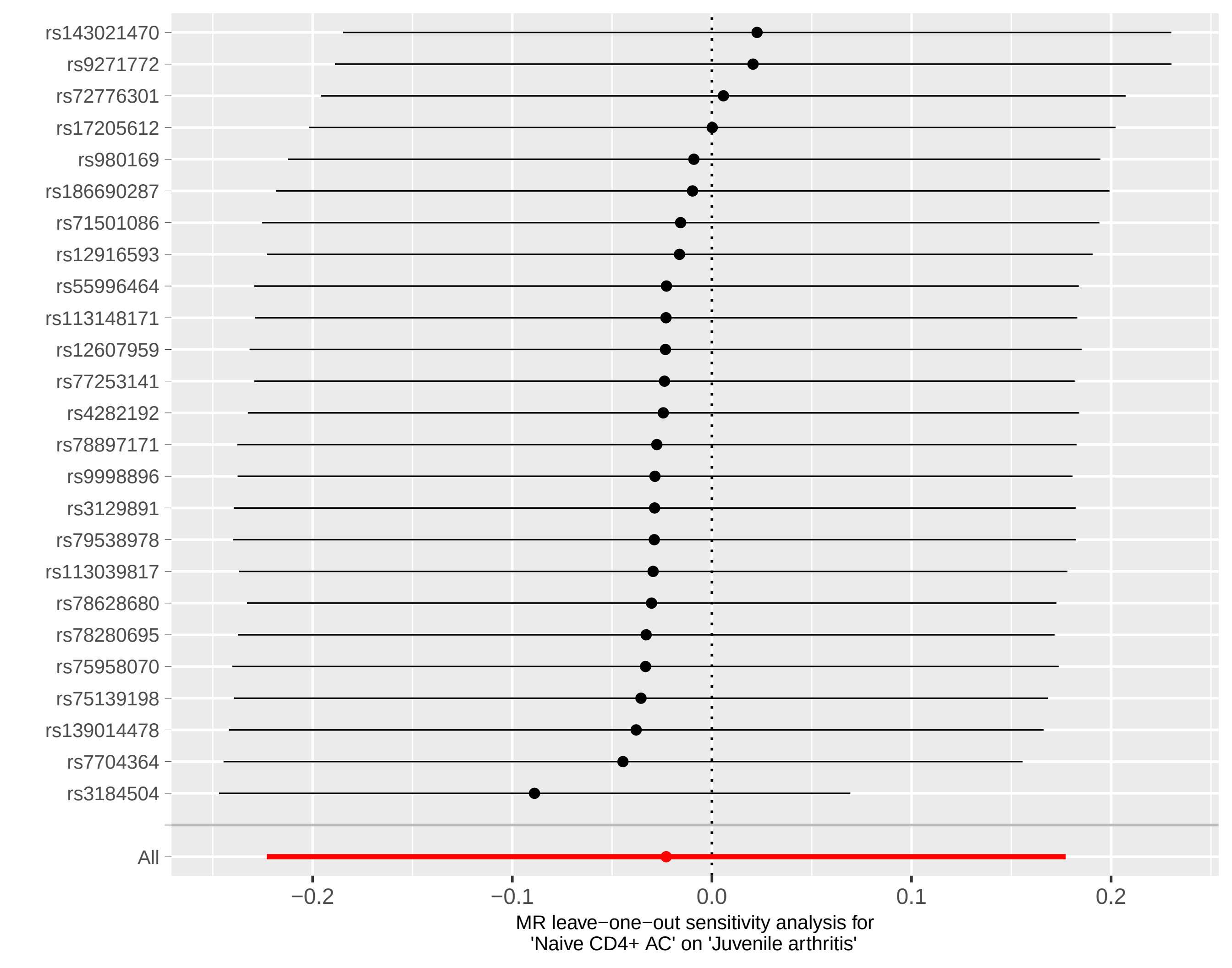
rs143021470
rs9271772
rs72776301
rs17205612
rs980169
rs186690287
rs71501086
rs12916593
rs55996464
rs113148171
rs12607959
rs77253141
rs4282192
rs78897171
rs9998896
rs3129891
rs79538978
rs113039817
rs78628680
rs78280695
rs75958070
rs75139198
rs139014478
rs7704364
rs3184504
All
−0.2
−0.1
0.0
0.1
0.2
MR leave−one−out sensitivity analysis for
'Naive CD4+ AC' on 'Juvenile arthritis'

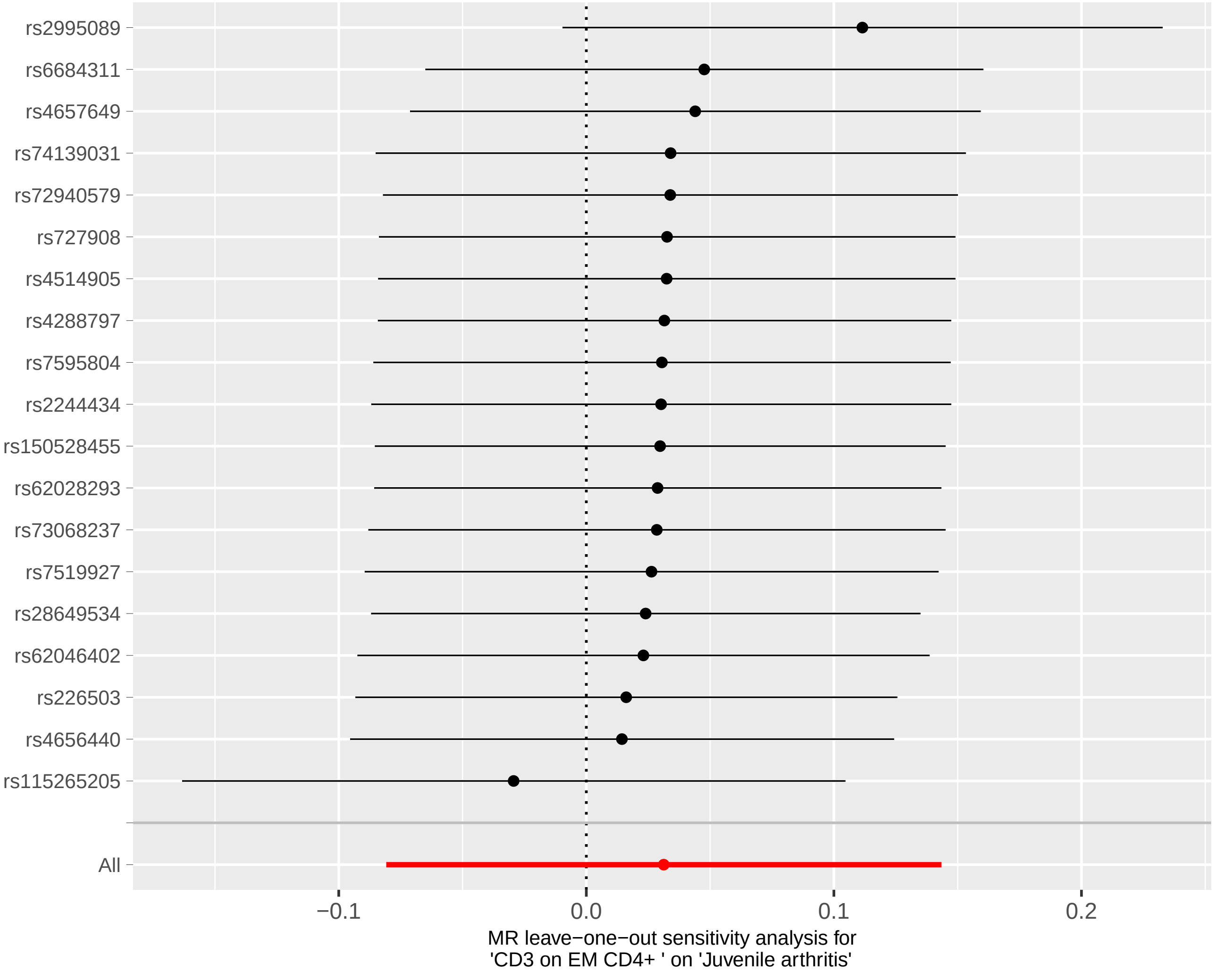
rs2995089
rs6684311
rs4657649
rs74139031
rs72940579
rs727908
rs4514905
rs4288797
rs7595804
rs2244434
rs150528455
rs62028293
rs73068237
rs7519927
rs28649534
rs62046402
rs226503
rs4656440
rs115265205
All
−0.1
0.0
0.1
0.2
MR leave−one−out sensitivity analysis for
'CD3 on EM CD4+ ' on 'Juvenile arthritis'

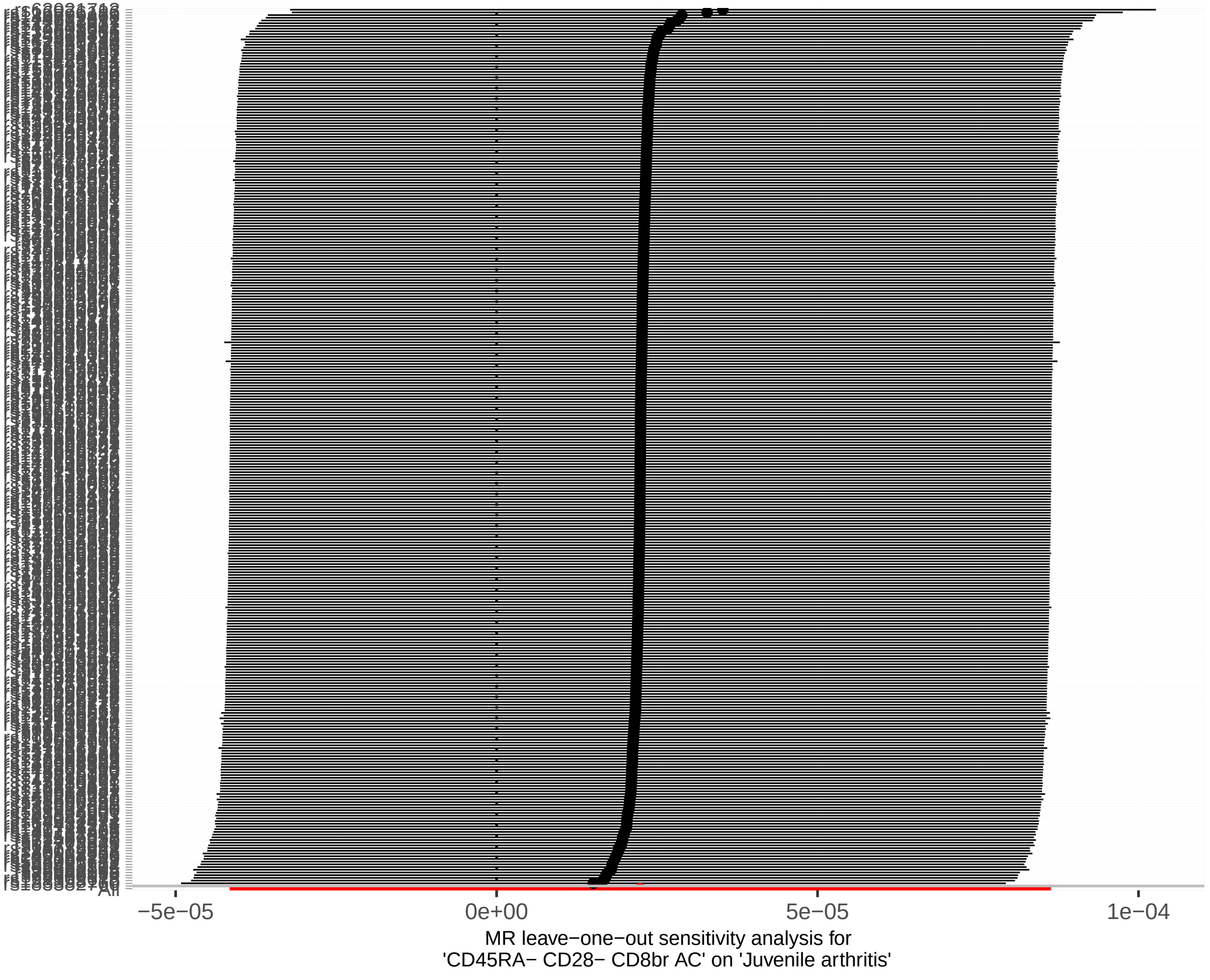
All
−5e−05
0e+00
5e−05
1e−04
MR leave−one−out sensitivity analysis for
'CD45RA− CD28− CD8br AC' on 'Juvenile arthritis'

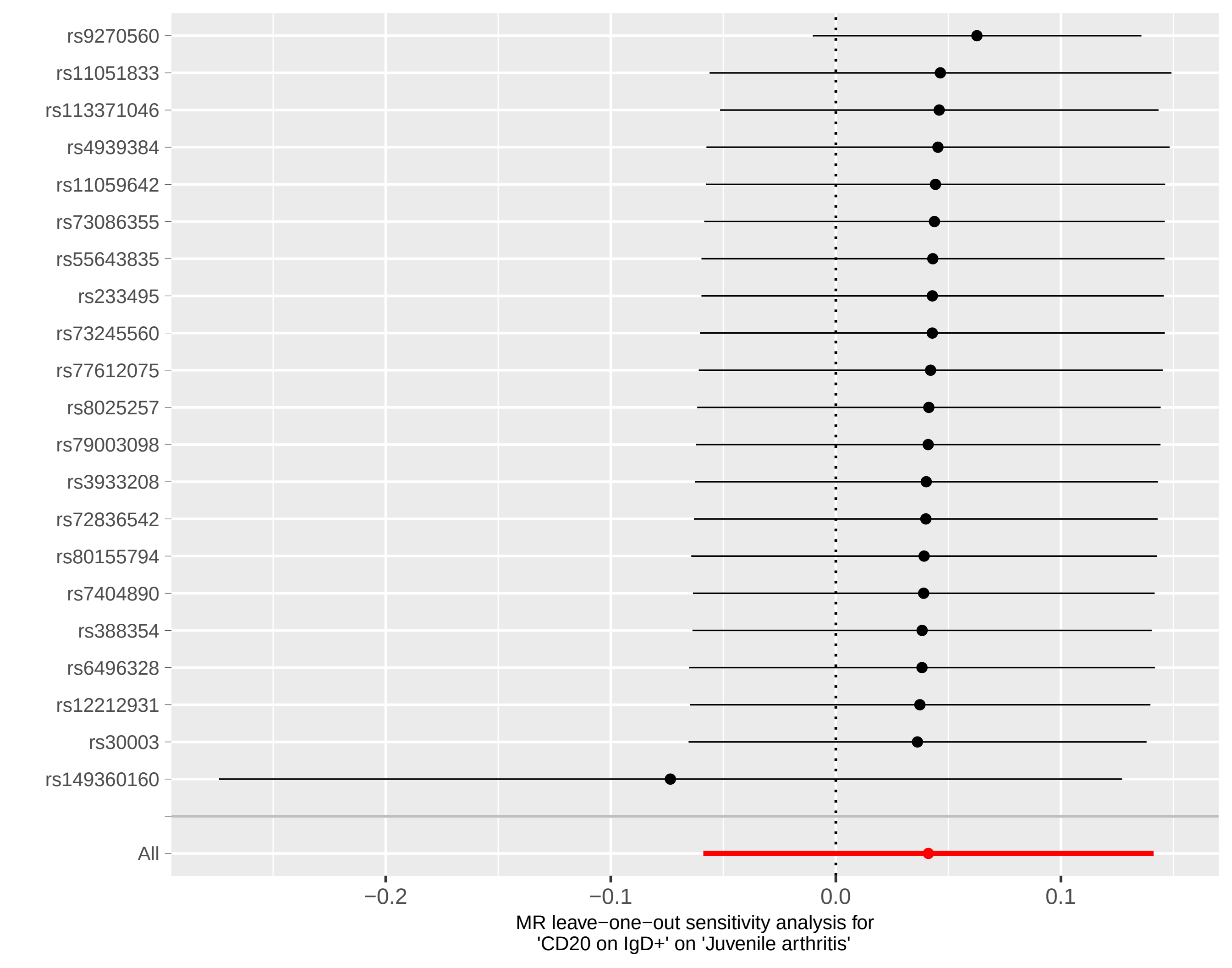
rs9270560
rs11051833
rs113371046
rs4939384
rs11059642
rs73086355
rs55643835
rs233495
rs73245560
rs77612075
rs8025257
rs79003098
rs3933208
rs72836542
rs80155794
rs7404890
rs388354
rs6496328
rs12212931
rs30003
rs149360160
All
−0.2
−0.1
0.0
0.1
MR leave−one−out sensitivity analysis for
'CD20 on IgD+' on 'Juvenile arthritis'

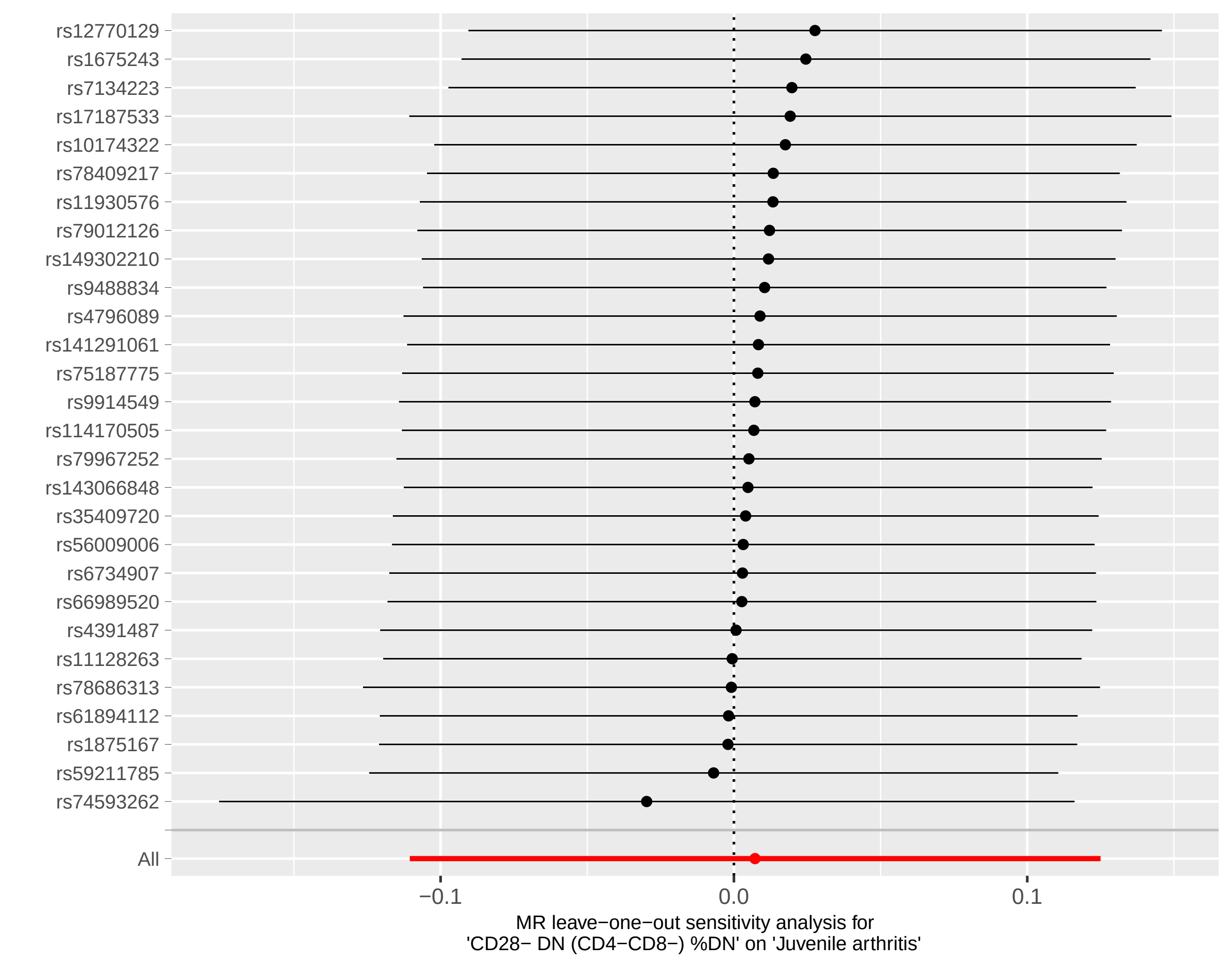

rs12770129
rs1675243
rs7134223
rs17187533
rs10174322
rs78409217
rs11930576
rs79012126
rs149302210
rs9488834
rs4796089
rs141291061
rs75187775
rs9914549
rs114170505
rs79967252
rs143066848
rs35409720
rs56009006
rs6734907
rs66989520
rs4391487
rs11128263
rs78686313
rs61894112
rs1875167
rs59211785
rs74593262
All
−0.1
0.0
0.1
MR leave−one−out sensitivity analysis for
'CD28− DN (CD4−CD8−) %DN' on 'Juvenile arthritis'

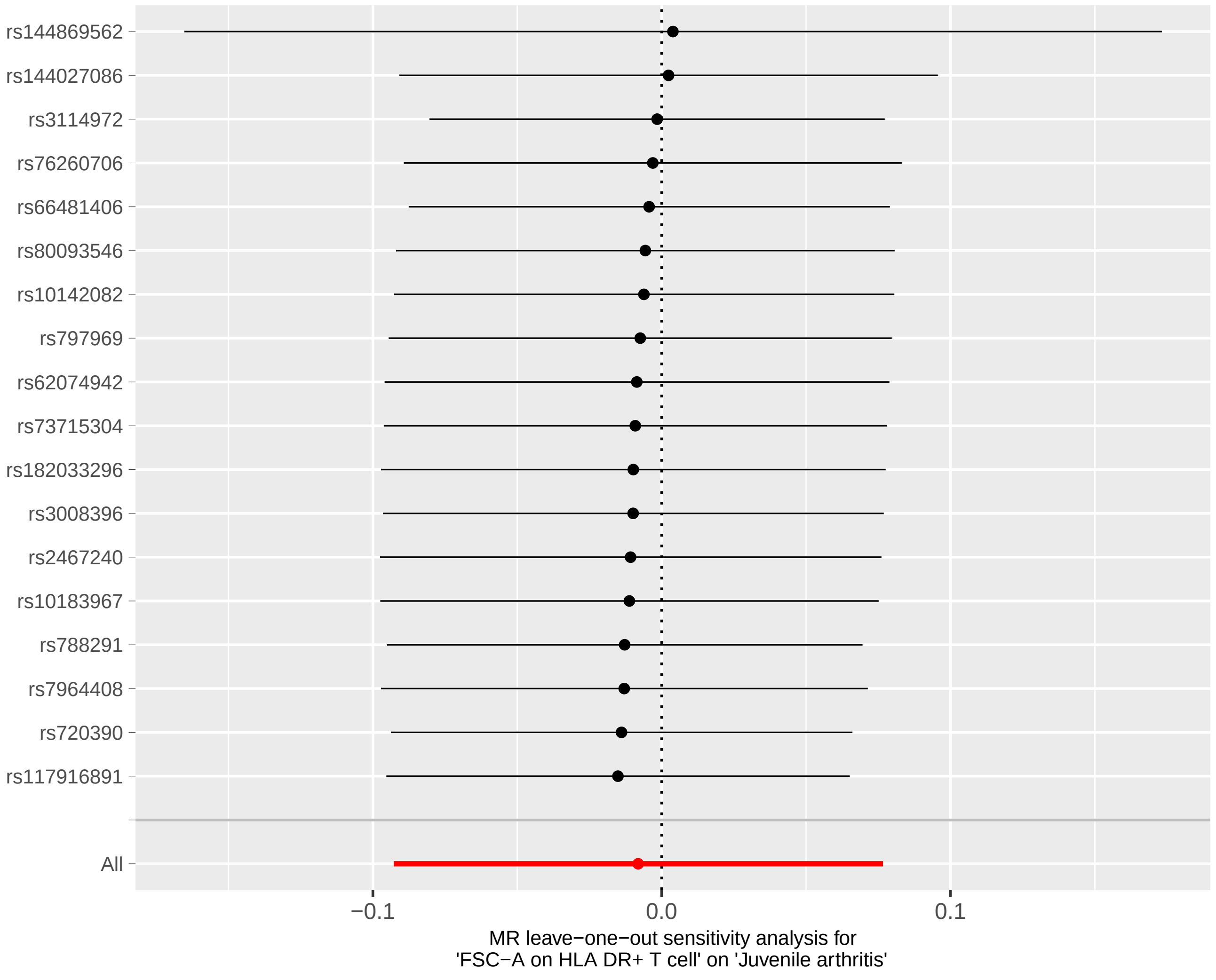
rs144869562
rs144027086
rs3114972
rs76260706
rs66481406
rs80093546
rs10142082
rs797969
rs62074942
rs73715304
rs182033296
rs3008396
rs2467240
rs10183967
rs788291
rs7964408
rs720390
rs117916891
All
−0.1
0.0
0.1
MR leave−one−out sensitivity analysis for
'FSC−A on HLA DR+ T cell' on 'Juvenile arthritis'

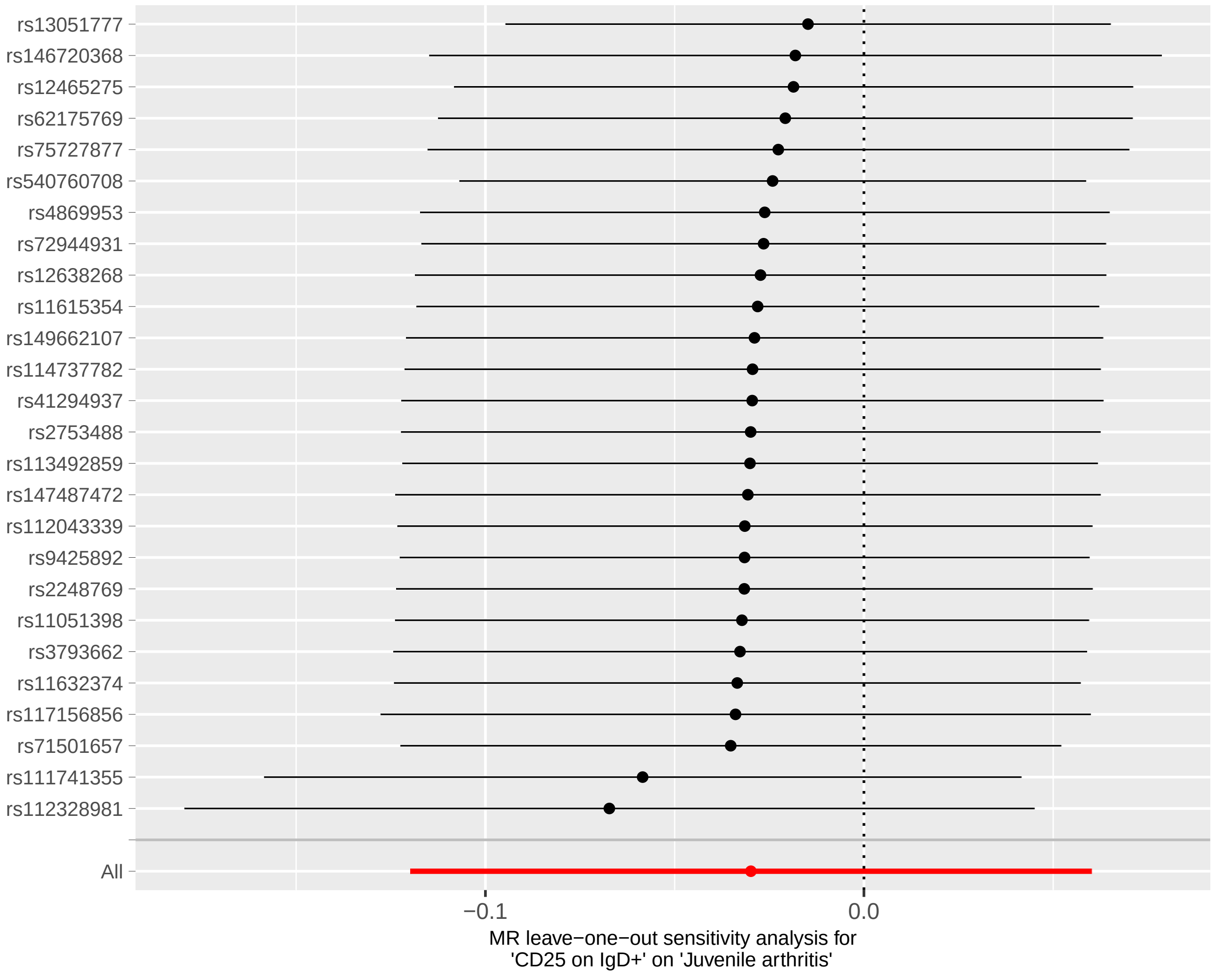
rs13051777
rs146720368
rs12465275
rs62175769
rs75727877
rs540760708
rs4869953
rs72944931
rs12638268
rs11615354
rs149662107
rs114737782
rs41294937
rs2753488
rs113492859
rs147487472
rs112043339
rs9425892
rs2248769
rs11051398
rs3793662
rs11632374
rs117156856
rs71501657
rs111741355
rs112328981
All
−0.1
0.0
MR leave−one−out sensitivity analysis for
'CD25 on IgD+' on 'Juvenile arthritis'

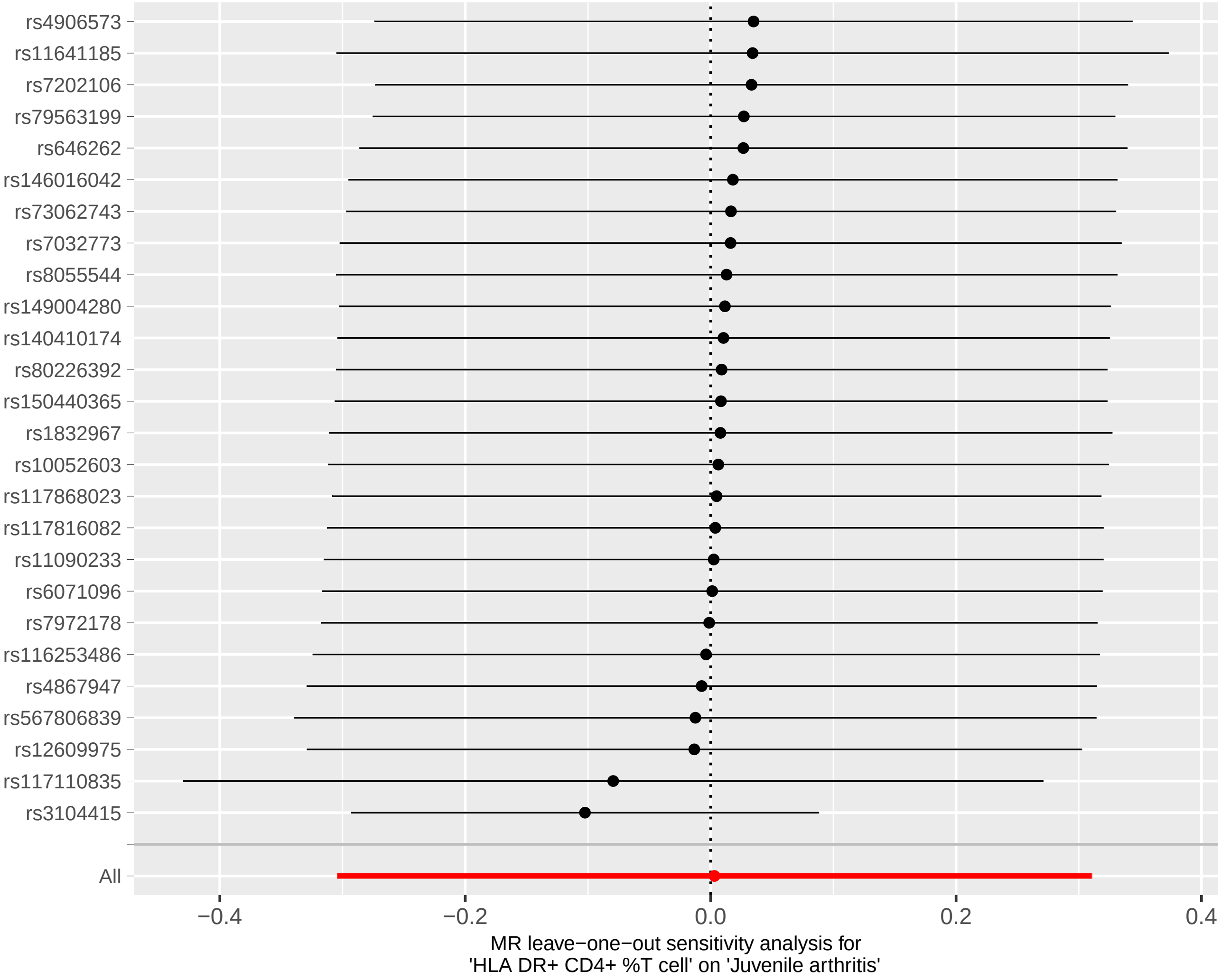
rs4906573
rs11641185
rs7202106
rs79563199
rs646262
rs146016042
rs73062743
rs7032773
rs8055544
rs149004280
rs140410174
rs80226392
rs150440365
rs1832967
rs10052603
rs117868023
rs117816082
rs11090233
rs6071096
rs7972178
rs116253486
rs4867947
rs567806839
rs12609975
rs117110835
rs3104415
All
−0.4
−0.2
0.0
0.2
0.4
MR leave−one−out sensitivity analysis for
'HLA DR+ CD4+ %T cell' on 'Juvenile arthritis'

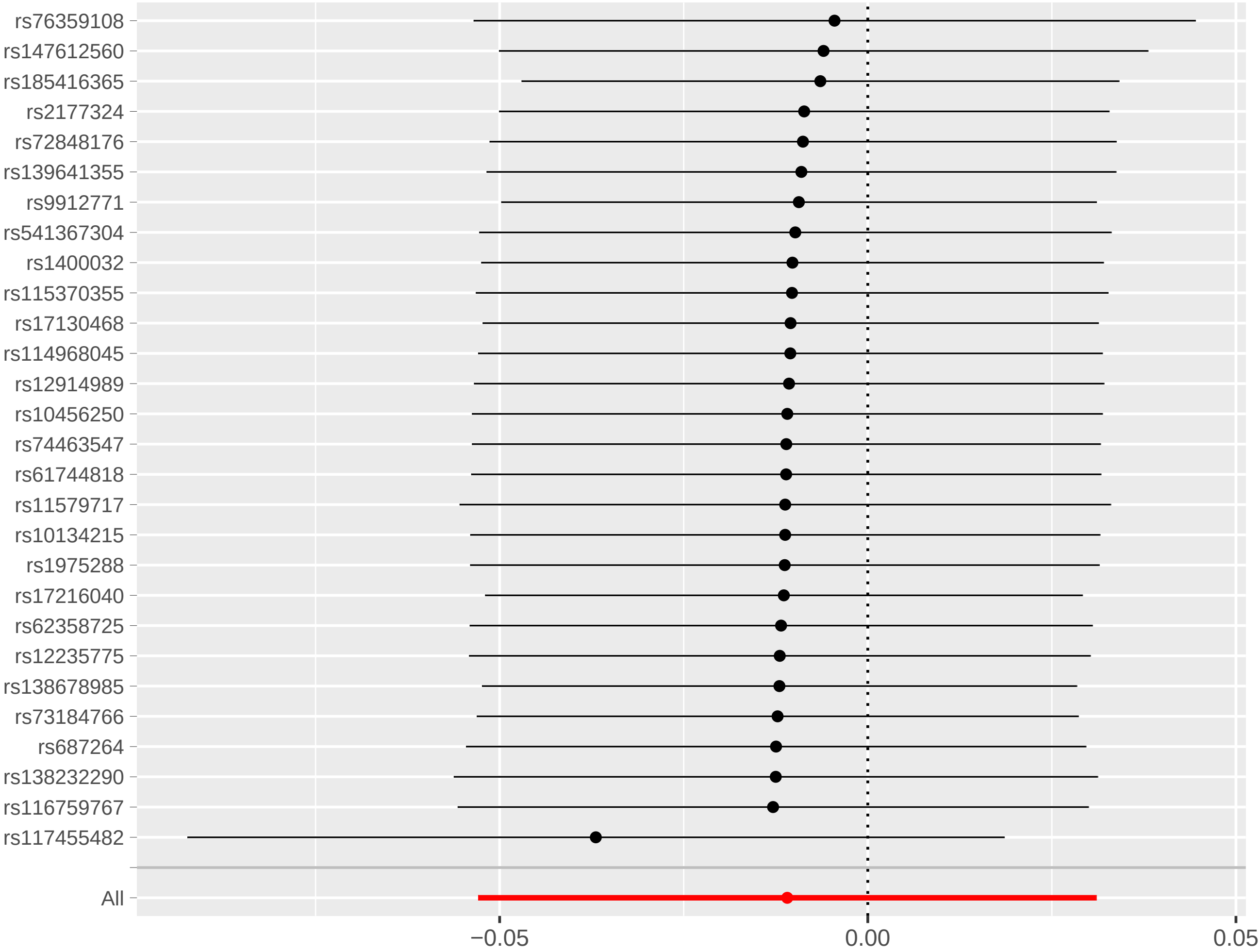

MR leave−one−out sensitivity analysis for
'CD28+ CD45RA− CD8dim %T cell' on 'Juvenile arthritis'

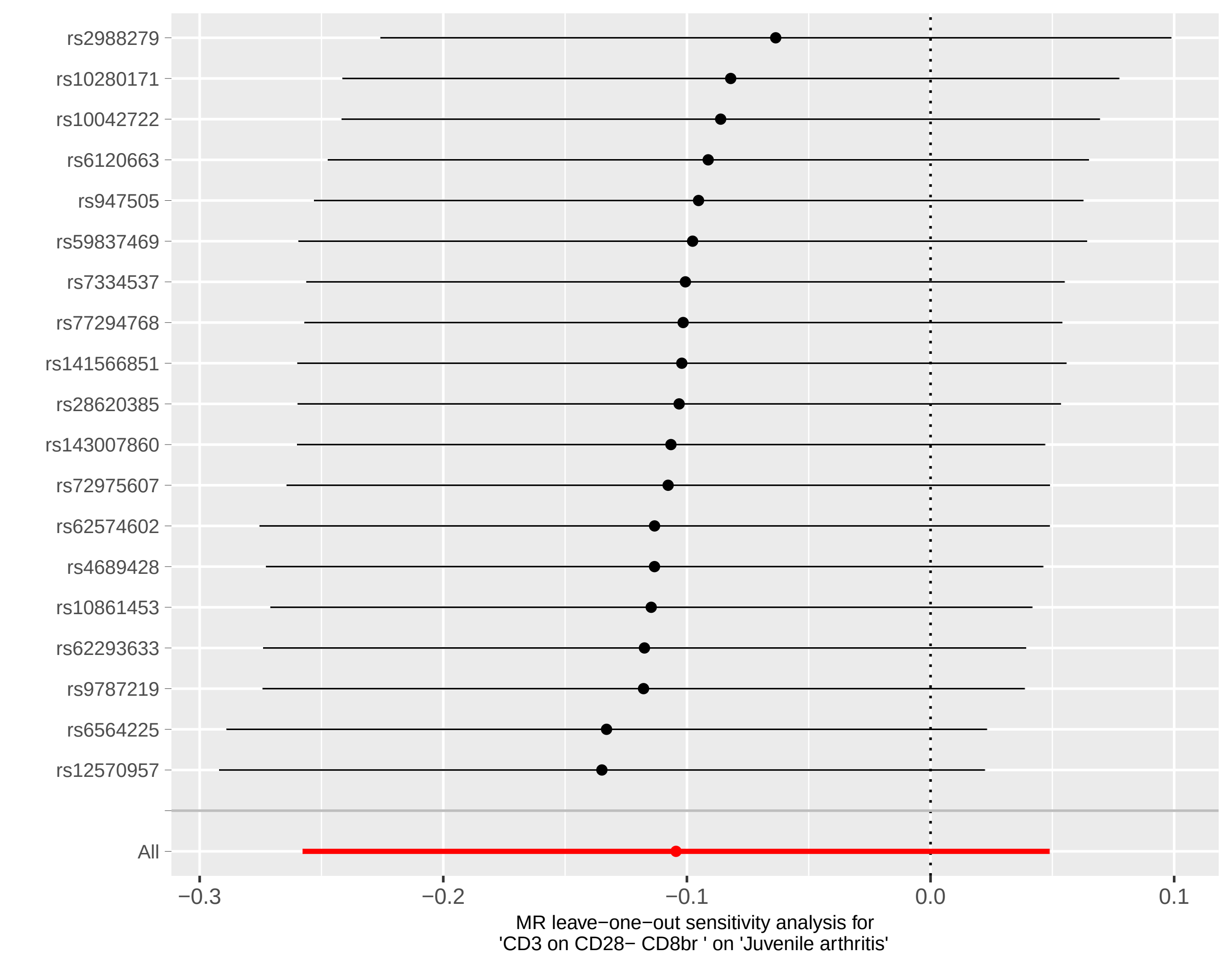

rs2988279
rs10280171
rs10042722
rs6120663
rs947505
rs59837469
rs7334537
rs77294768
rs141566851
rs28620385
rs143007860
rs72975607
rs62574602
rs4689428
rs10861453
rs62293633
rs9787219
rs6564225
rs12570957
All
−0.3
−0.2
−0.1
0.0
0.1
MR leave−one−out sensitivity analysis for
'CD3 on CD28− CD8br ' on 'Juvenile arthritis'

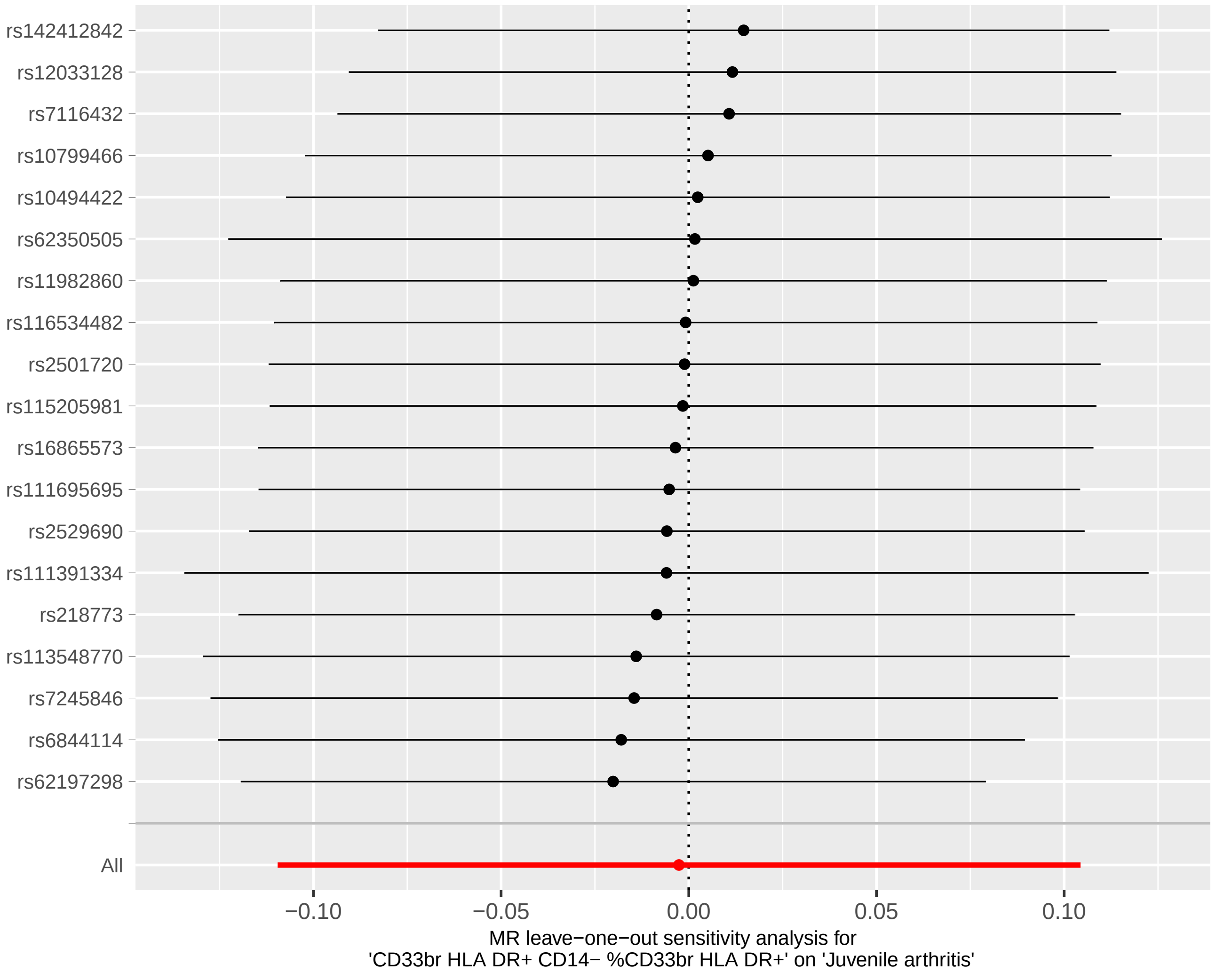
rs142412842
rs12033128
rs7116432
rs10799466
rs10494422
rs62350505
rs11982860
rs116534482
rs2501720
rs115205981
rs16865573
rs111695695
rs2529690
rs111391334
rs218773
rs113548770
rs7245846
rs6844114
rs62197298
All
−0.10
−0.05
0.00
0.05
0.10
MR leave−one−out sensitivity analysis for
'CD33br HLA DR+ CD14− %CD33br HLA DR+' on 'Juvenile arthritis'

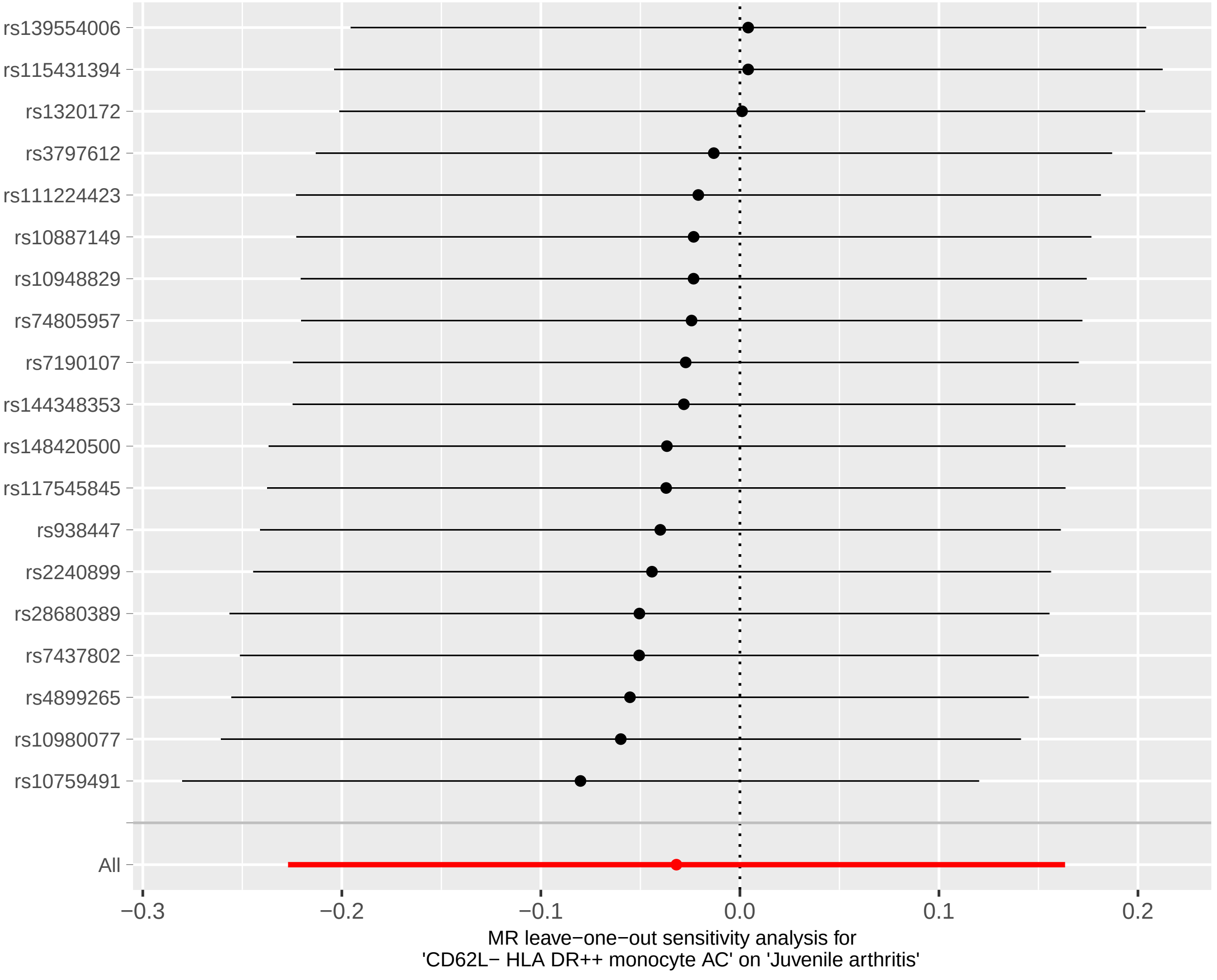
rs139554006
rs115431394
rs1320172
rs3797612
rs111224423
rs10887149
rs10948829
rs74805957
rs7190107
rs144348353
rs148420500
rs117545845
rs938447
rs2240899
rs28680389
rs7437802
rs4899265
rs10980077
rs10759491
All
−0.3
−0.2
−0.1
0.0
0.1
0.2
MR leave−one−out sensitivity analysis for
'CD62L− HLA DR++ monocyte AC' on 'Juvenile arthritis'

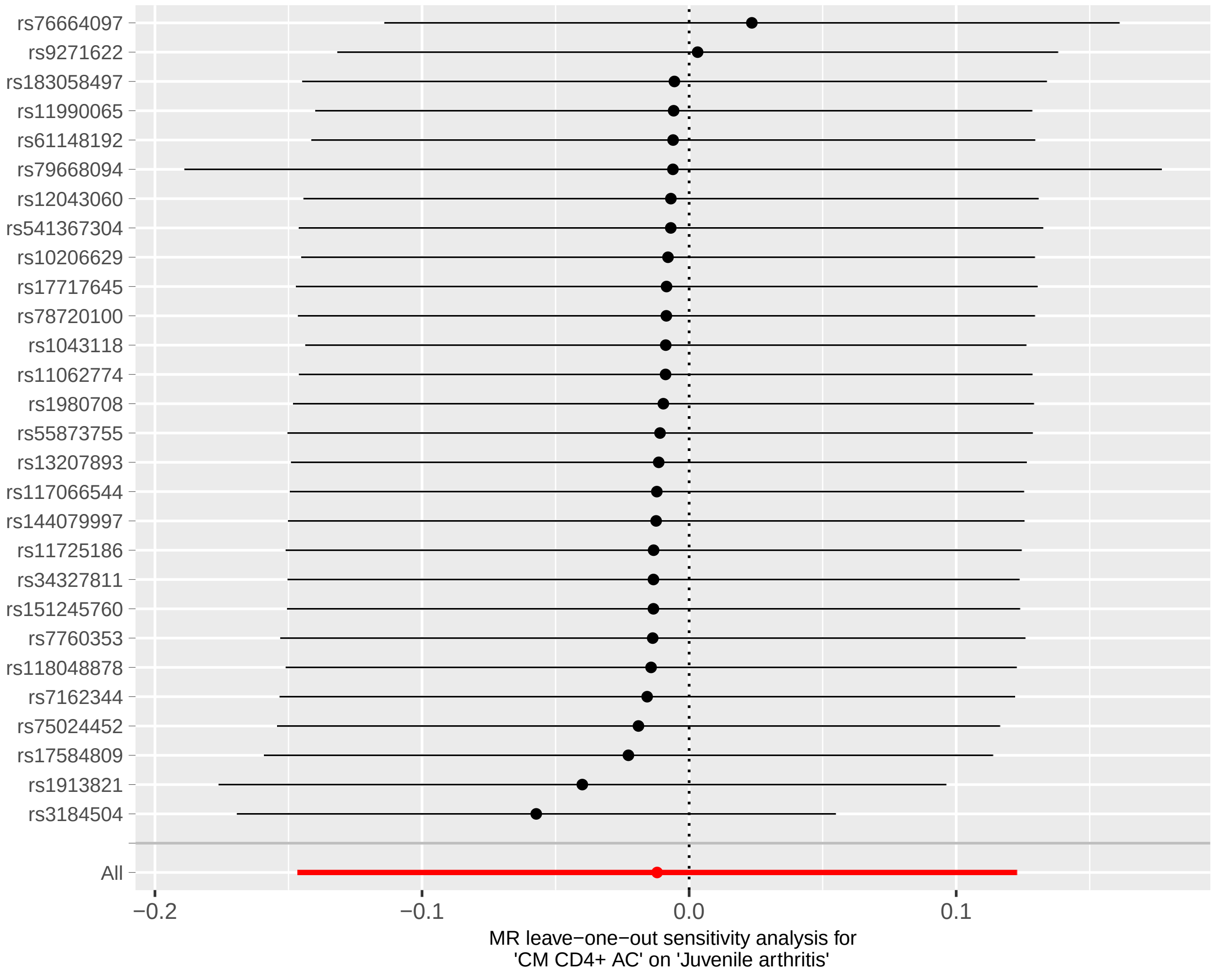

rs76664097
rs9271622
rs183058497
rs11990065
rs61148192
rs79668094
rs12043060
rs541367304
rs10206629
rs17717645
rs78720100
rs1043118
rs11062774
rs1980708
rs55873755
rs13207893
rs117066544
rs144079997
rs11725186
rs34327811
rs151245760
rs7760353
rs118048878
rs7162344
rs75024452
rs17584809
rs1913821
rs3184504
All
−0.2
−0.1
0.0
0.1
MR leave−one−out sensitivity analysis for
'CM CD4+ AC' on 'Juvenile arthritis'

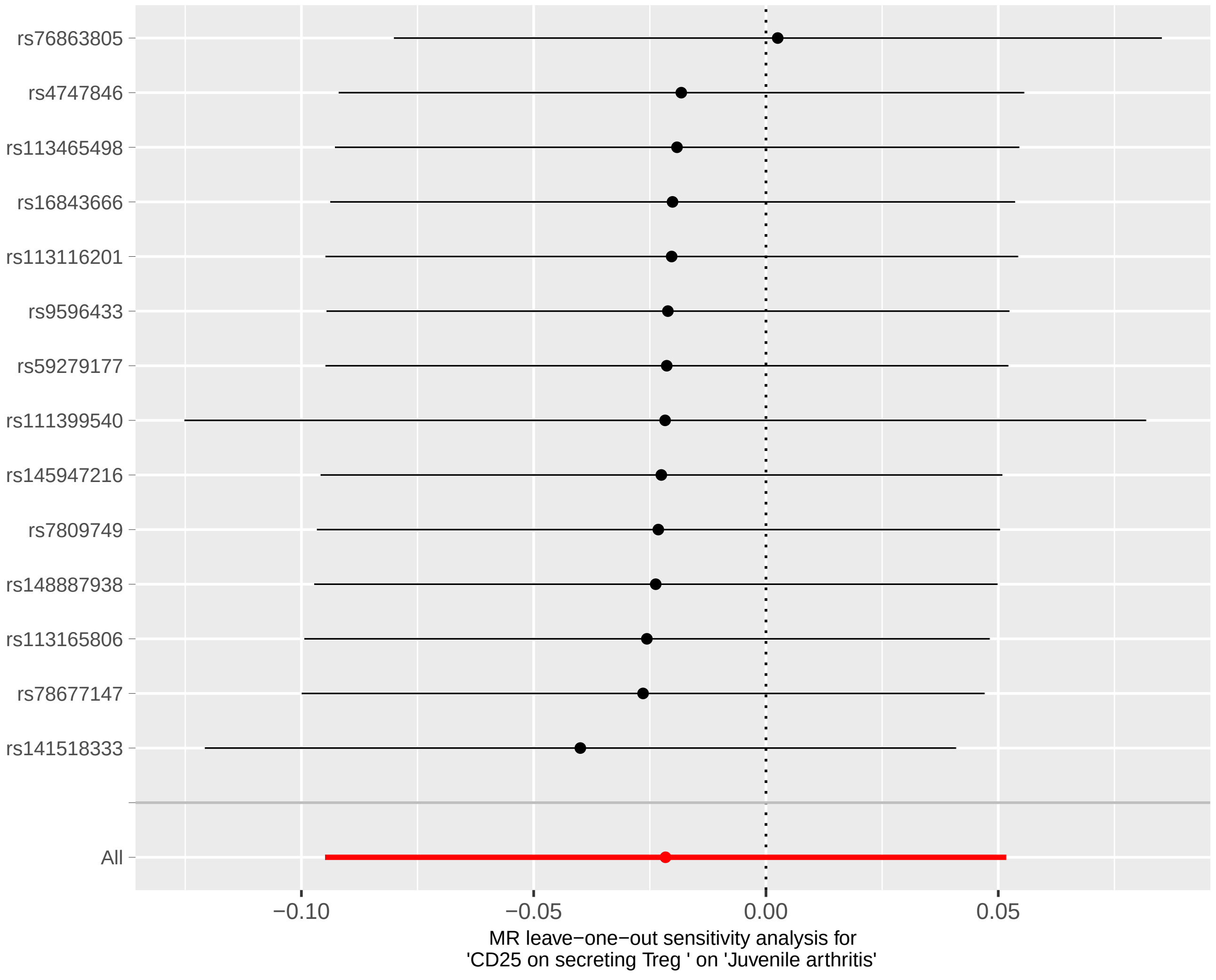
rs76863805
rs4747846
rs113465498
rs16843666
rs113116201
rs9596433
rs59279177
rs111399540
rs145947216
rs7809749
rs148887938
rs113165806
rs78677147
rs141518333
All
−0.10
−0.05
0.00
0.05
MR leave−one−out sensitivity analysis for
'CD25 on secreting Treg ' on 'Juvenile arthritis'

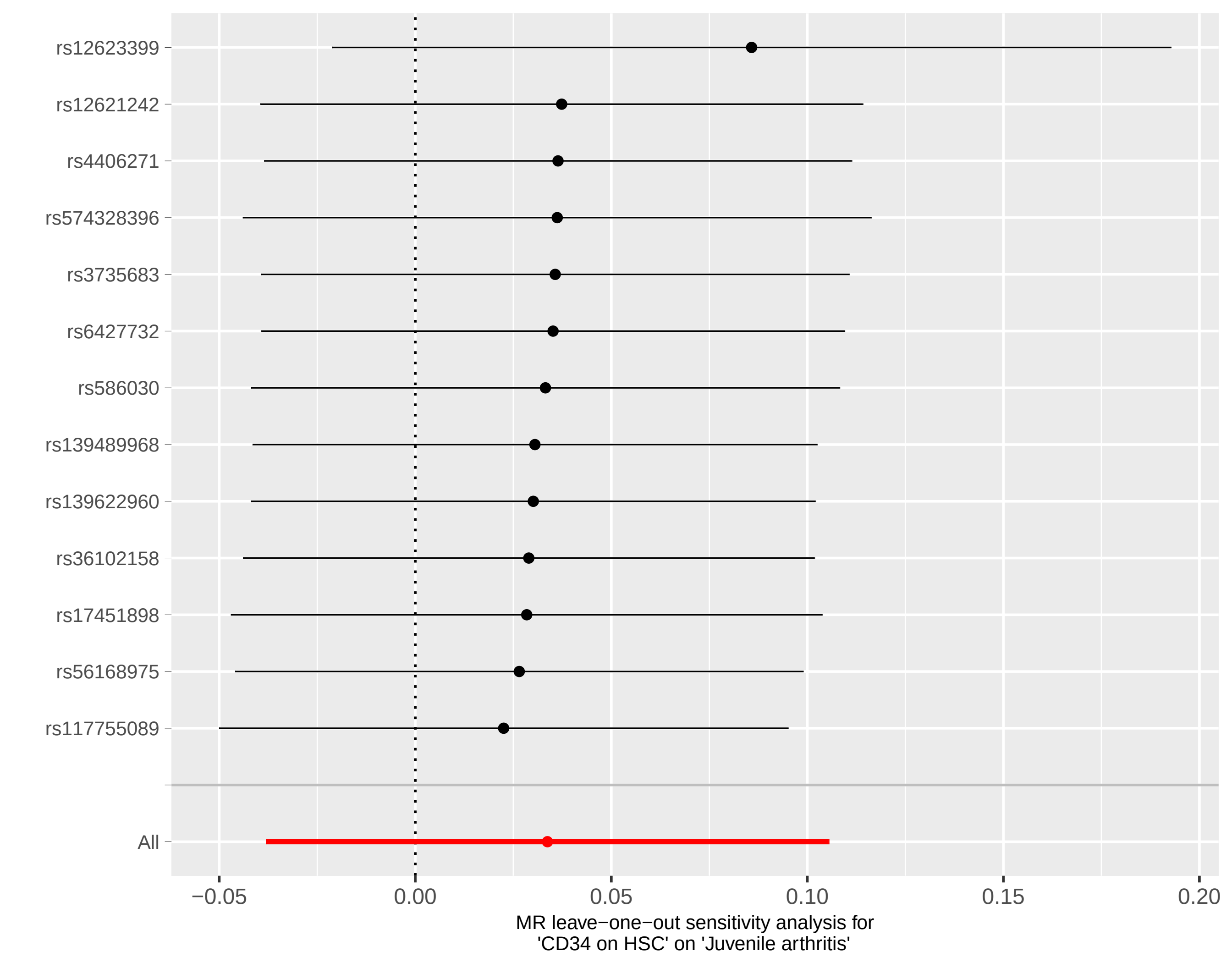

rs12623399
rs12621242
rs4406271
rs574328396
rs3735683
rs6427732
rs586030
rs139489968
rs139622960
rs36102158
rs17451898
rs56168975
rs117755089
All
−0.05
0.00
0.05
0.10
0.15
0.20
MR leave−one−out sensitivity analysis for
'CD34 on HSC' on 'Juvenile arthritis'

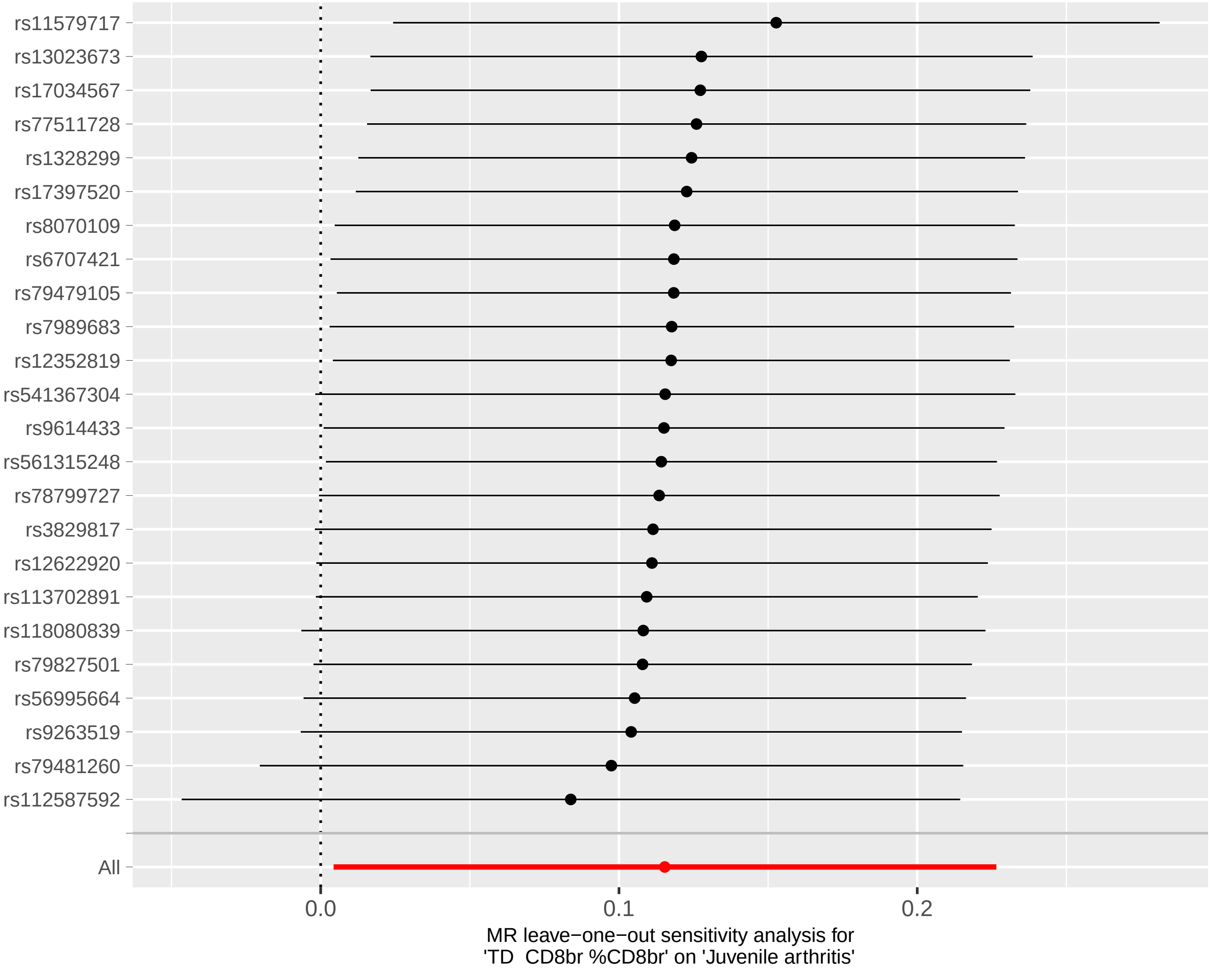
rs11579717
rs13023673
rs17034567
rs77511728
rs1328299
rs17397520
rs8070109
rs6707421
rs79479105
rs7989683
rs12352819
rs541367304
rs9614433
rs561315248
rs78799727
rs3829817
rs12622920
rs113702891
rs118080839
rs79827501
rs56995664
rs9263519
rs79481260
rs112587592
All
0.0
0.1
0.2
MR leave−one−out sensitivity analysis for
'TD CD8br %CD8br' on 'Juvenile arthritis'

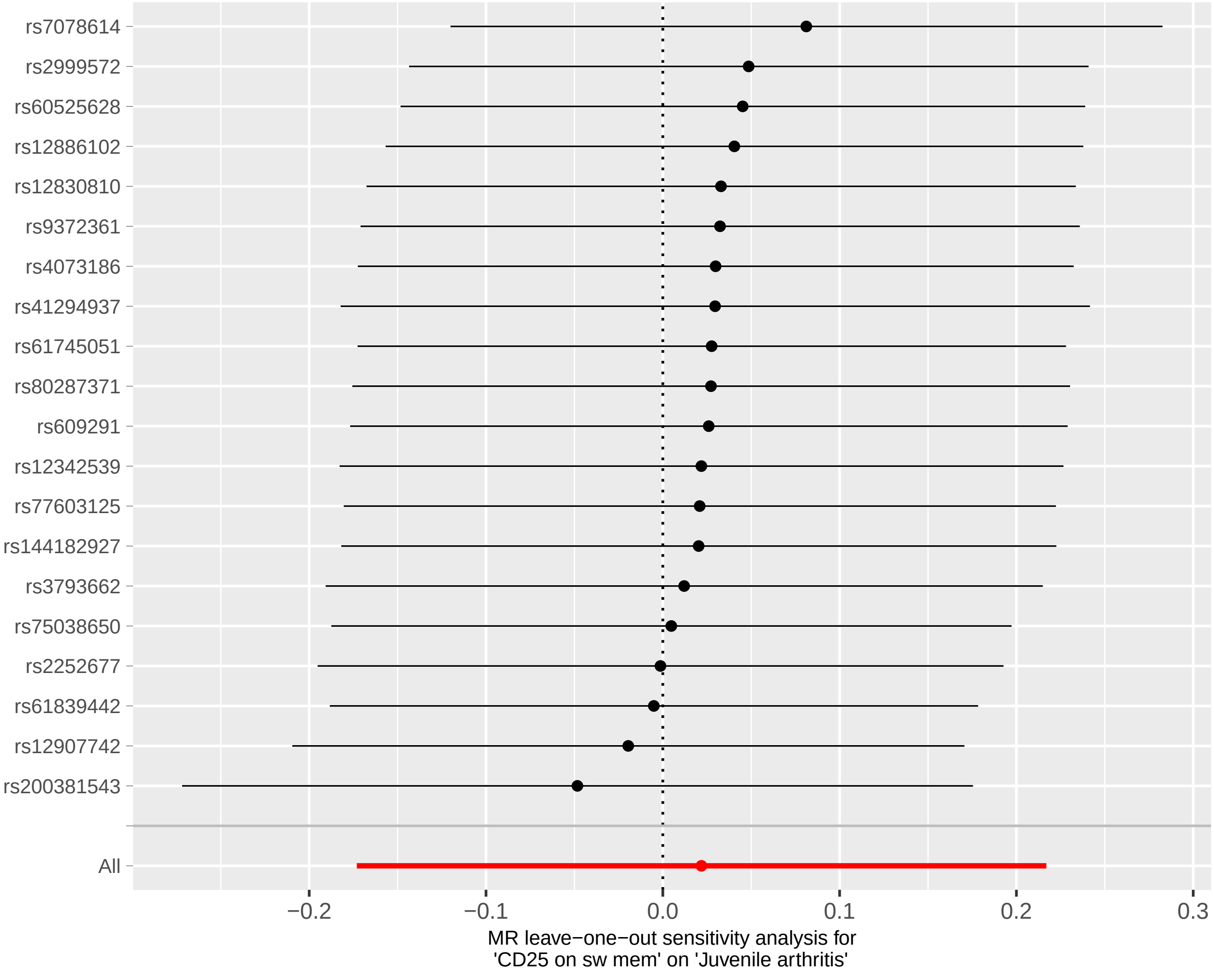
rs7078614
rs2999572
rs60525628
rs12886102
rs12830810
rs9372361
rs4073186
rs41294937
rs61745051
rs80287371
rs609291
rs12342539
rs77603125
rs144182927
rs3793662
rs75038650
rs2252677
rs61839442
rs12907742
rs200381543
All
−0.2
−0.1
0.0
0.1
0.2
0.3
MR leave−one−out sensitivity analysis for
'CD25 on sw mem' on 'Juvenile arthritis'

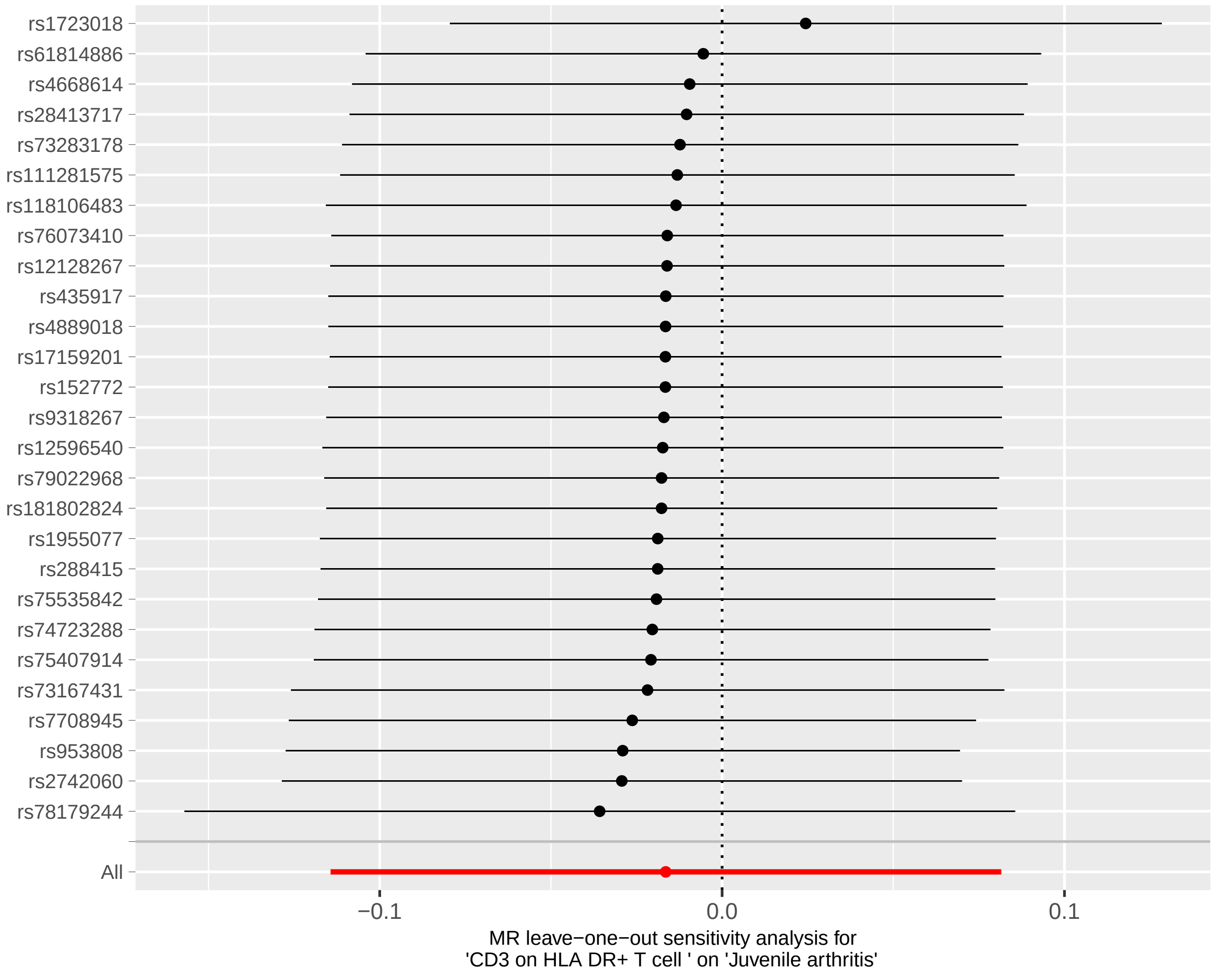
rs1723018
rs61814886
rs4668614
rs28413717
rs73283178
rs111281575
rs118106483
rs76073410
rs12128267
rs435917
rs4889018
rs17159201
rs152772
rs9318267
rs12596540
rs79022968
rs181802824
rs1955077
rs288415
rs75535842
rs74723288
rs75407914
rs73167431
rs7708945
rs953808
rs2742060
rs78179244
All
−0.1
0.0
0.1
MR leave−one−out sensitivity analysis for
'CD3 on HLA DR+ T cell ' on 'Juvenile arthritis'

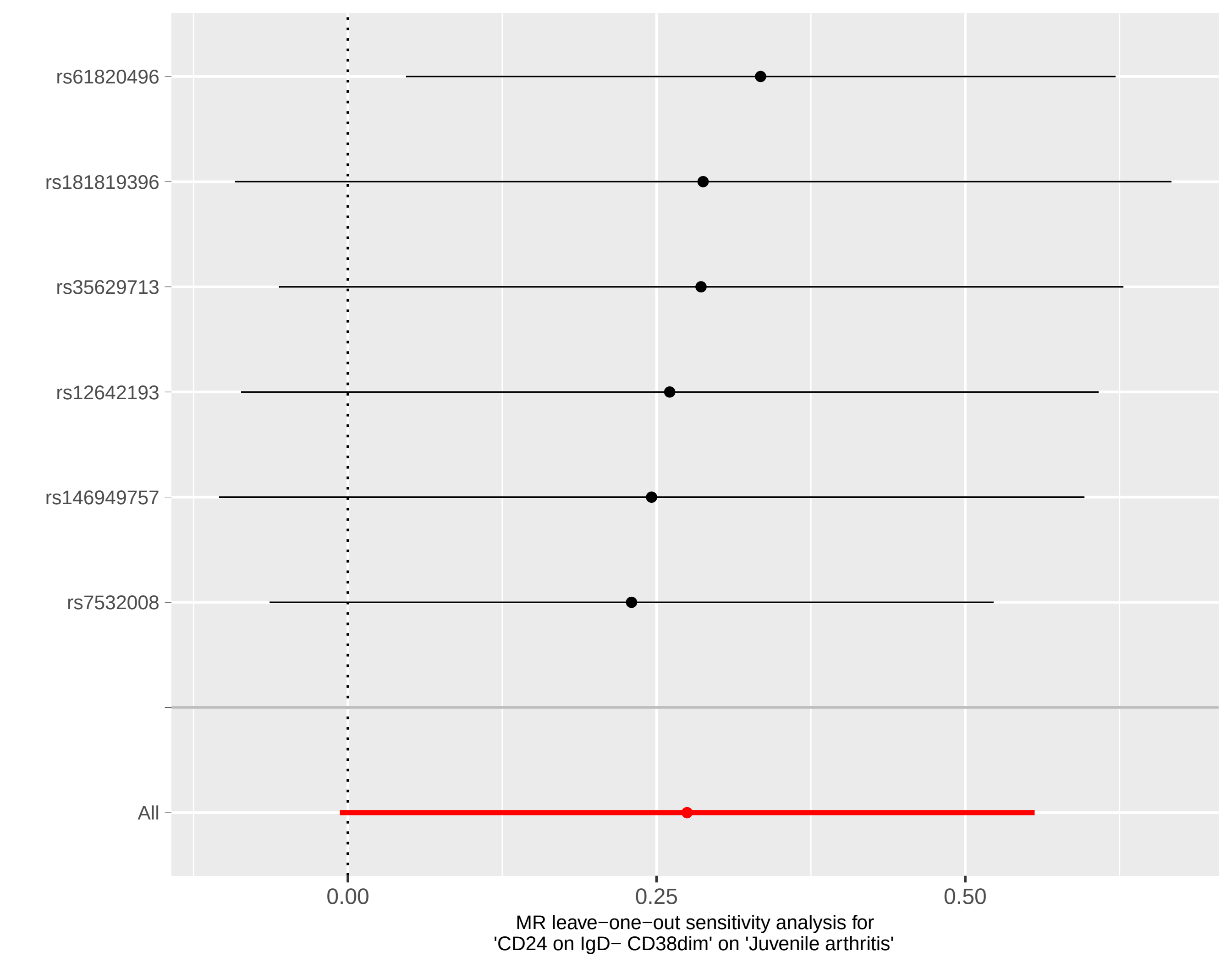
rs61820496
rs181819396
rs35629713
rs12642193
rs146949757
rs7532008
All
0.00
0.25
0.50
MR leave−one−out sensitivity analysis for
'CD24 on IgD− CD38dim' on 'Juvenile arthritis'

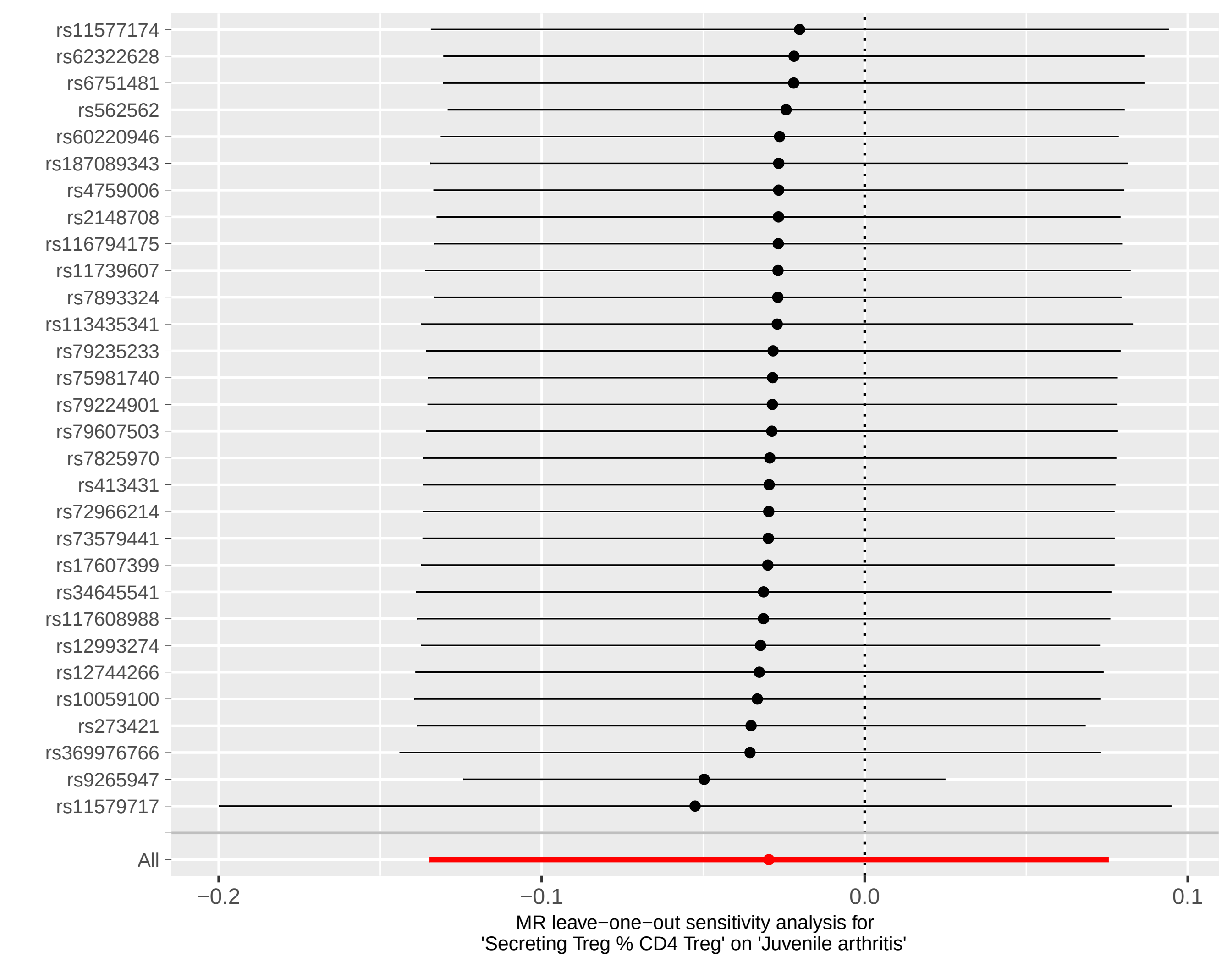
rs11577174
rs62322628
rs6751481
rs562562
rs60220946
rs187089343
rs4759006
rs2148708
rs116794175
rs11739607
rs7893324
rs113435341
rs79235233
rs75981740
rs79224901
rs79607503
rs7825970
rs413431
rs72966214
rs73579441
rs17607399
rs34645541
rs117608988
rs12993274
rs12744266
rs10059100
rs273421
rs369976766
rs9265947
rs11579717
All
−0.2
−0.1
0.0
0.1
MR leave−one−out sensitivity analysis for
'Secreting Treg % CD4 Treg' on 'Juvenile arthritis'

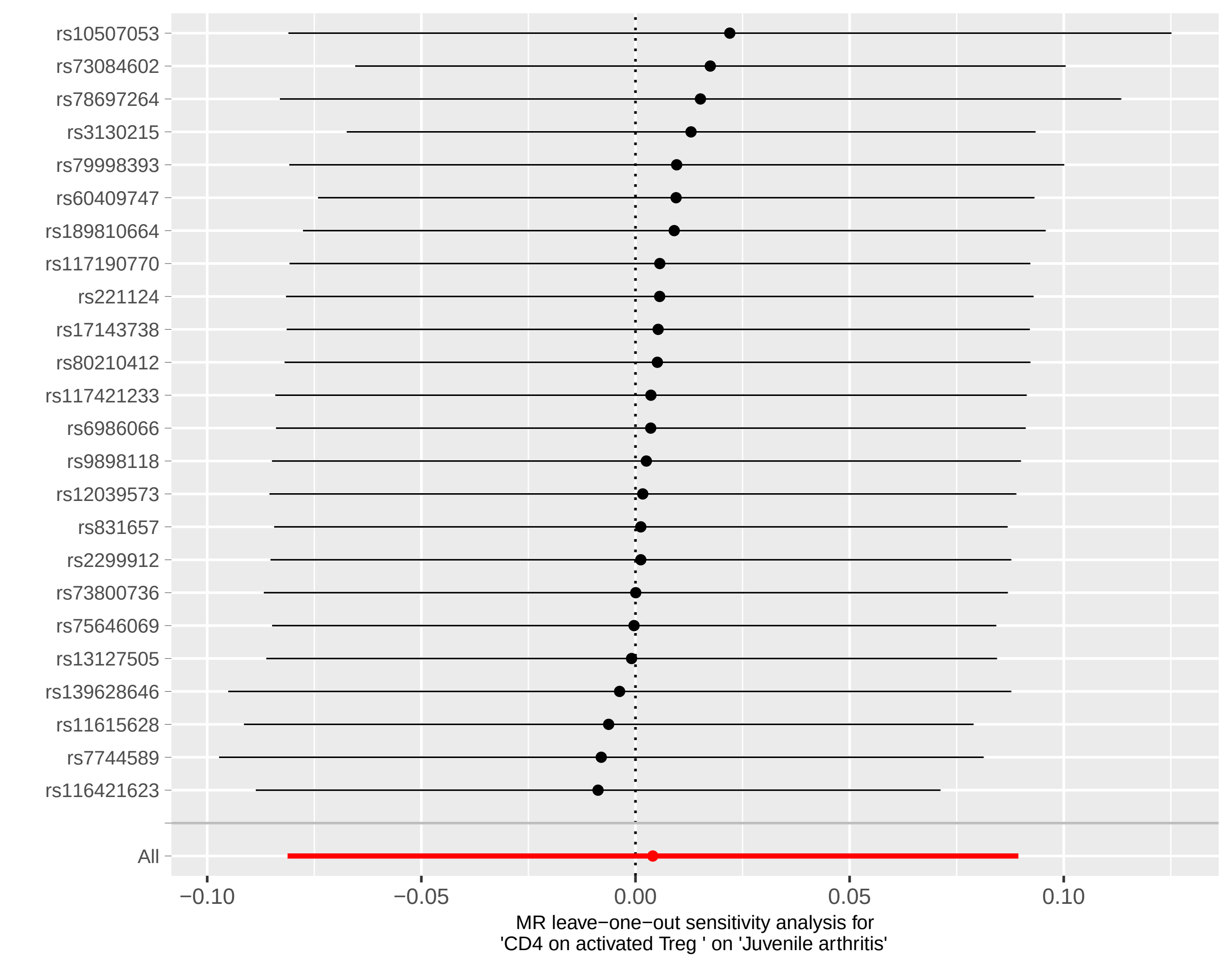
rs10507053
rs73084602
rs78697264
rs3130215
rs79998393
rs60409747
rs189810664
rs117190770
rs221124
rs17143738
rs80210412
rs117421233
rs6986066
rs9898118
rs12039573
rs831657
rs2299912
rs73800736
rs75646069
rs13127505
rs139628646
rs11615628
rs7744589
rs116421623
All
−0.10
−0.05
0.00
0.05
0.10
MR leave−one−out sensitivity analysis for
'CD4 on activated Treg ' on 'Juvenile arthritis'

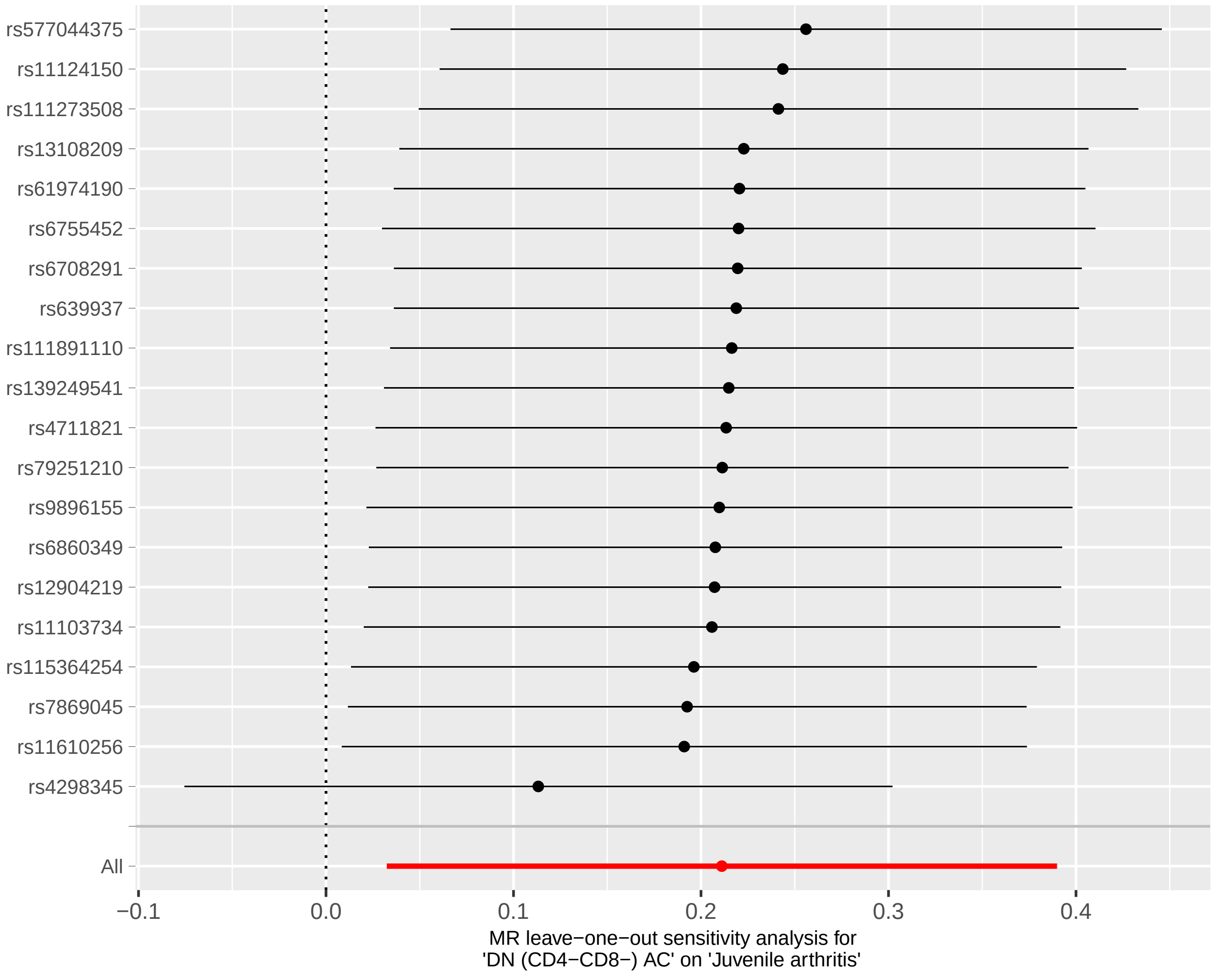

rs577044375
rs11124150
rs111273508
rs13108209
rs61974190
rs6755452
rs6708291
rs639937
rs111891110
rs139249541
rs4711821
rs79251210
rs9896155
rs6860349
rs12904219
rs11103734
rs115364254
rs7869045
rs11610256
rs4298345
All
−0.1
0.0
0.1
0.2
0.3
0.4
MR leave−one−out sensitivity analysis for
'DN (CD4−CD8−) AC' on 'Juvenile arthritis'

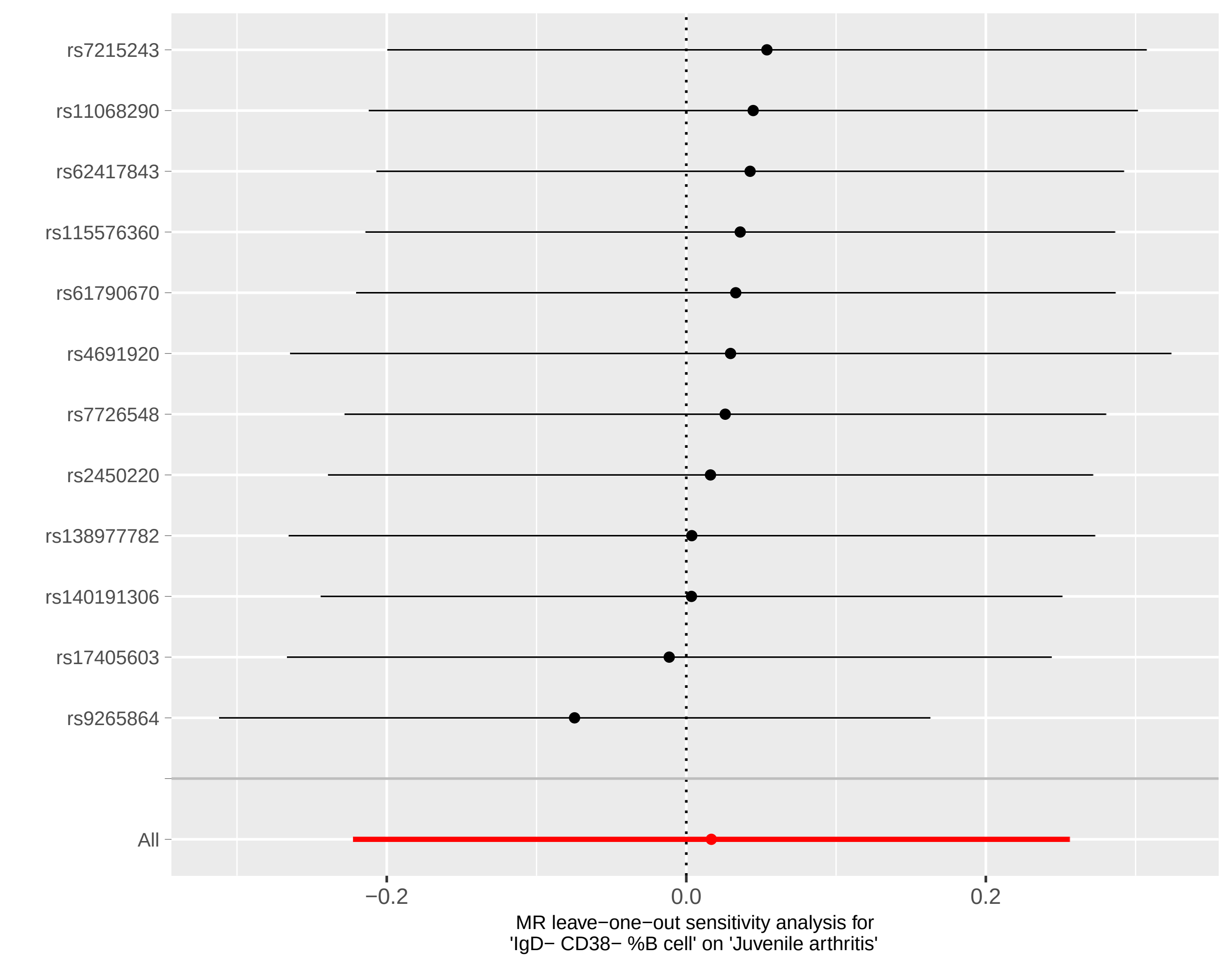
rs7215243
rs11068290
rs62417843
rs115576360
rs61790670
rs4691920
rs7726548
rs2450220
rs138977782
rs140191306
rs17405603
rs9265864
All
−0.2
0.0
0.2
MR leave−one−out sensitivity analysis for
'IgD− CD38− %B cell' on 'Juvenile arthritis'

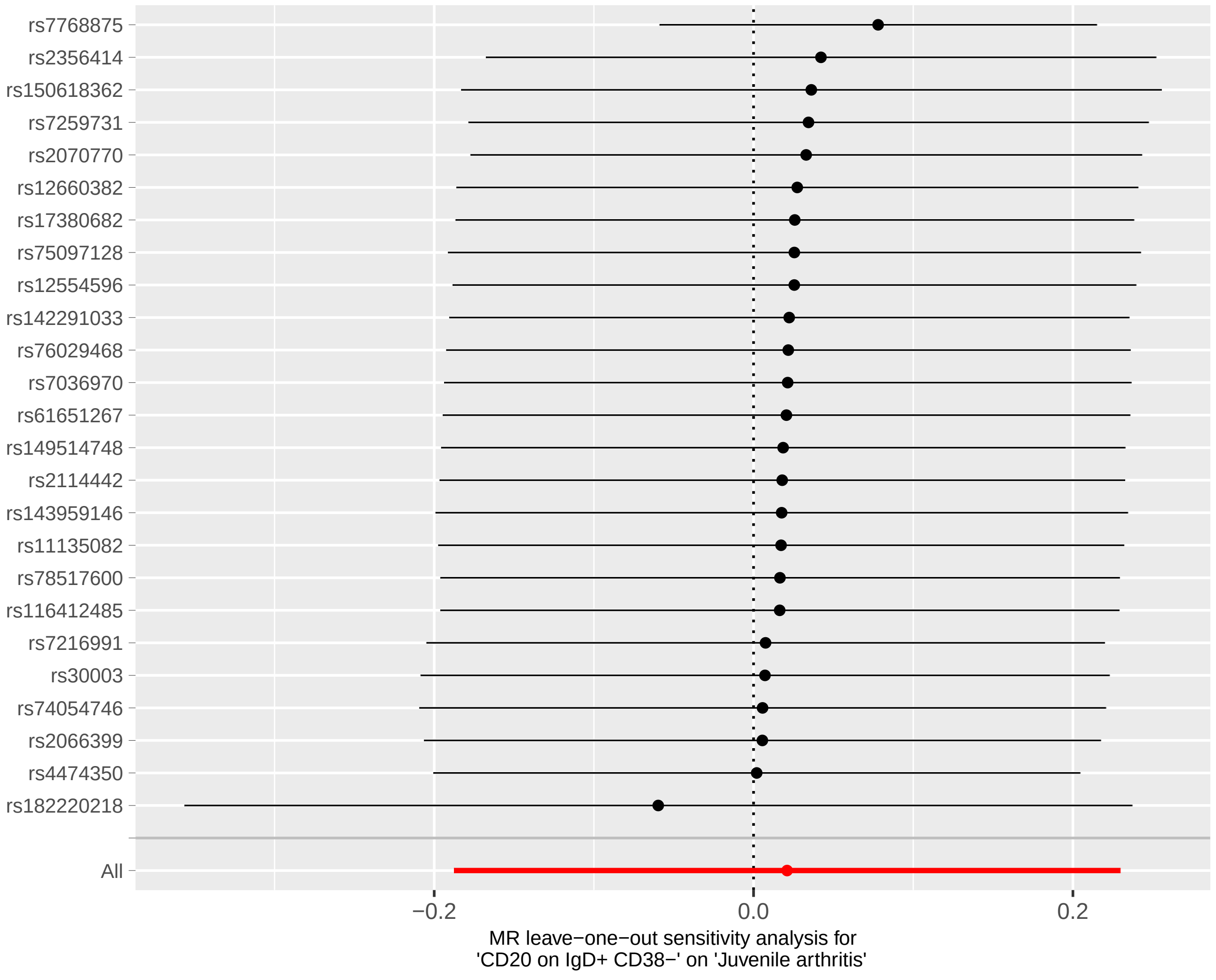
rs7768875
rs2356414
rs150618362
rs7259731
rs2070770
rs12660382
rs17380682
rs75097128
rs12554596
rs142291033
rs76029468
rs7036970
rs61651267
rs149514748
rs2114442
rs143959146
rs11135082
rs78517600
rs116412485
rs7216991
rs30003
rs74054746
rs2066399
rs4474350
rs182220218
All
−0.2
0.0
0.2
MR leave−one−out sensitivity analysis for
'CD20 on IgD+ CD38−' on 'Juvenile arthritis'

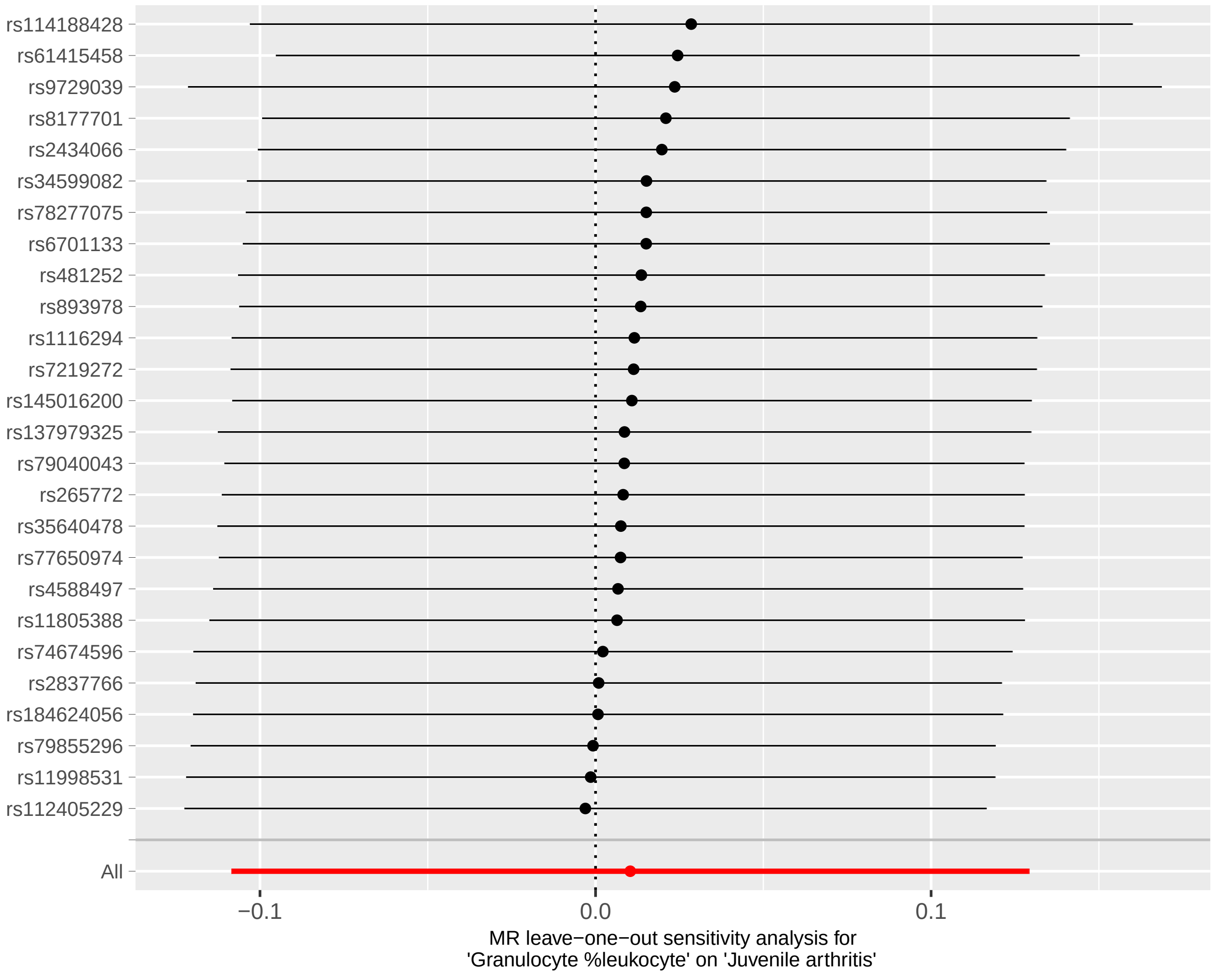
rs114188428
rs61415458
rs9729039
rs8177701
rs2434066
rs34599082
rs78277075
rs6701133
rs481252
rs893978
rs1116294
rs7219272
rs145016200
rs137979325
rs79040043
rs265772
rs35640478
rs77650974
rs4588497
rs11805388
rs74674596
rs2837766
rs184624056
rs79855296
rs11998531
rs112405229
All
−0.1
0.0
0.1
MR leave−one−out sensitivity analysis for
'Granulocyte %leukocyte' on 'Juvenile arthritis'

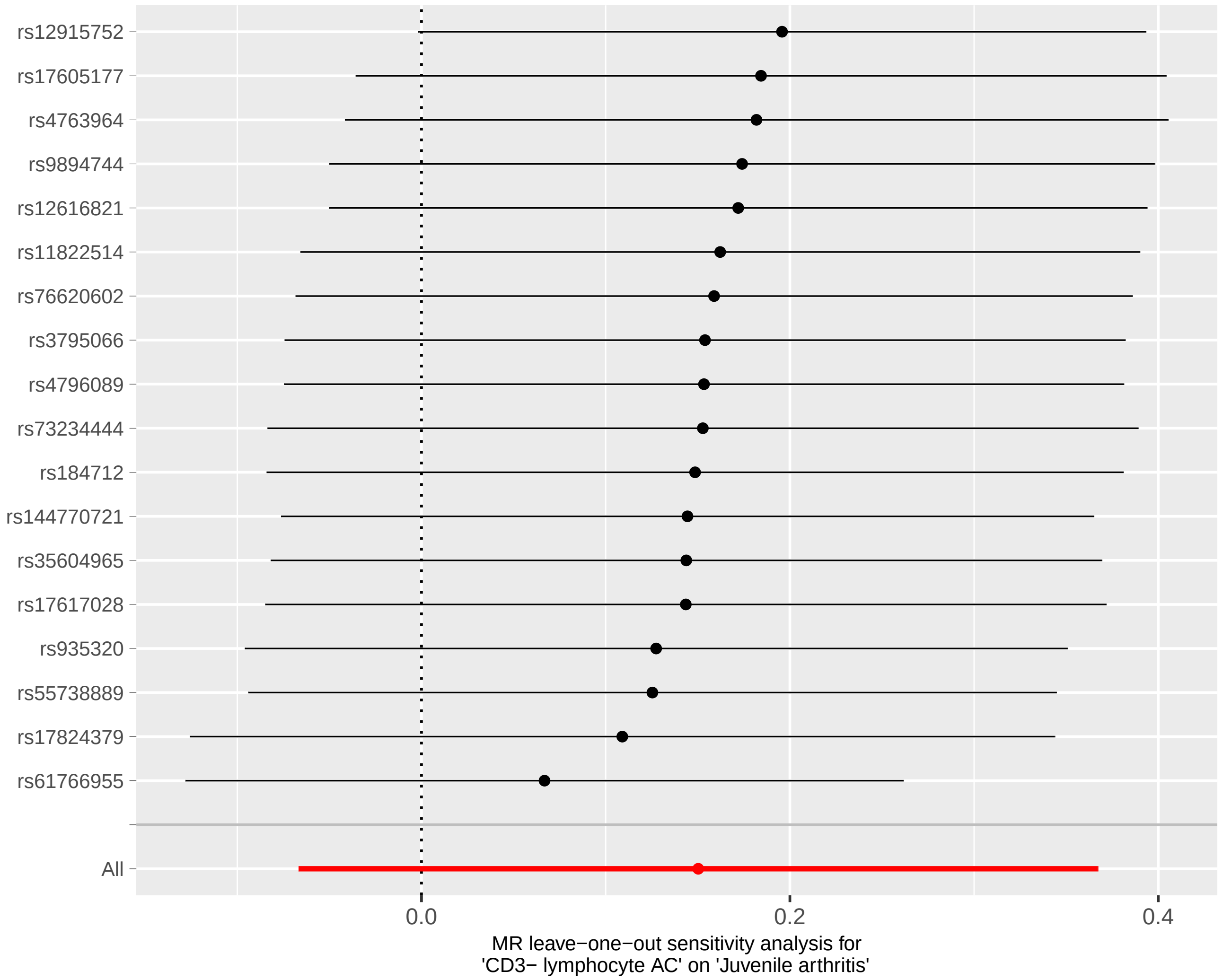

rs12915752
rs17605177
rs4763964
rs9894744
rs12616821
rs11822514
rs76620602
rs3795066
rs4796089
rs73234444
rs184712
rs144770721
rs35604965
rs17617028
rs935320
rs55738889
rs17824379
rs61766955
All
0.0
0.2
0.4
MR leave−one−out sensitivity analysis for
'CD3− lymphocyte AC' on 'Juvenile arthritis'

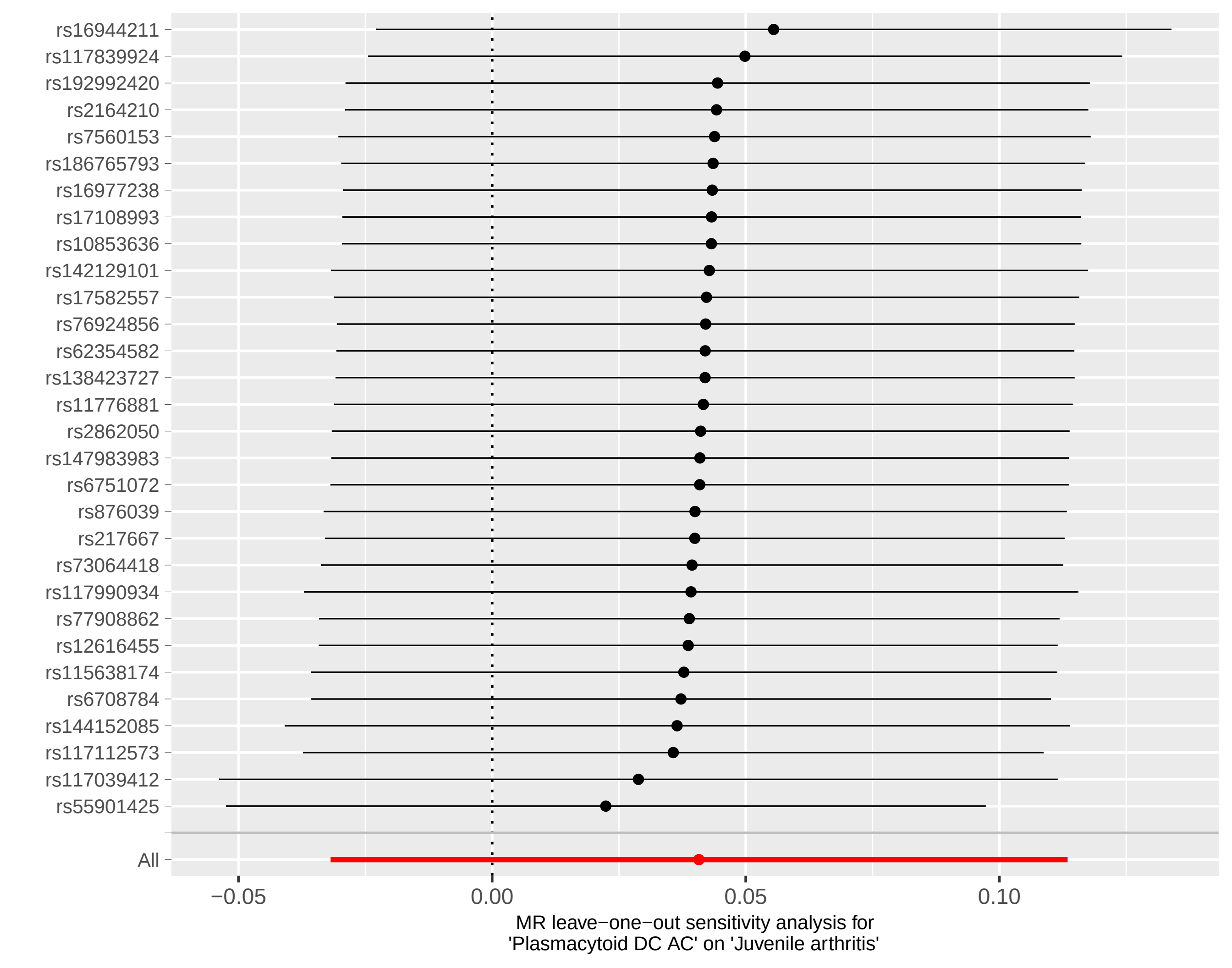
rs16944211
rs117839924
rs192992420
rs2164210
rs7560153
rs186765793
rs16977238
rs17108993
rs10853636
rs142129101
rs17582557
rs76924856
rs62354582
rs138423727
rs11776881
rs2862050
rs147983983
rs6751072
rs876039
rs217667
rs73064418
rs117990934
rs77908862
rs12616455
rs115638174
rs6708784
rs144152085
rs117112573
rs117039412
rs55901425
All
−0.05
0.00
0.05
0.10
MR leave−one−out sensitivity analysis for
'Plasmacytoid DC AC' on 'Juvenile arthritis'

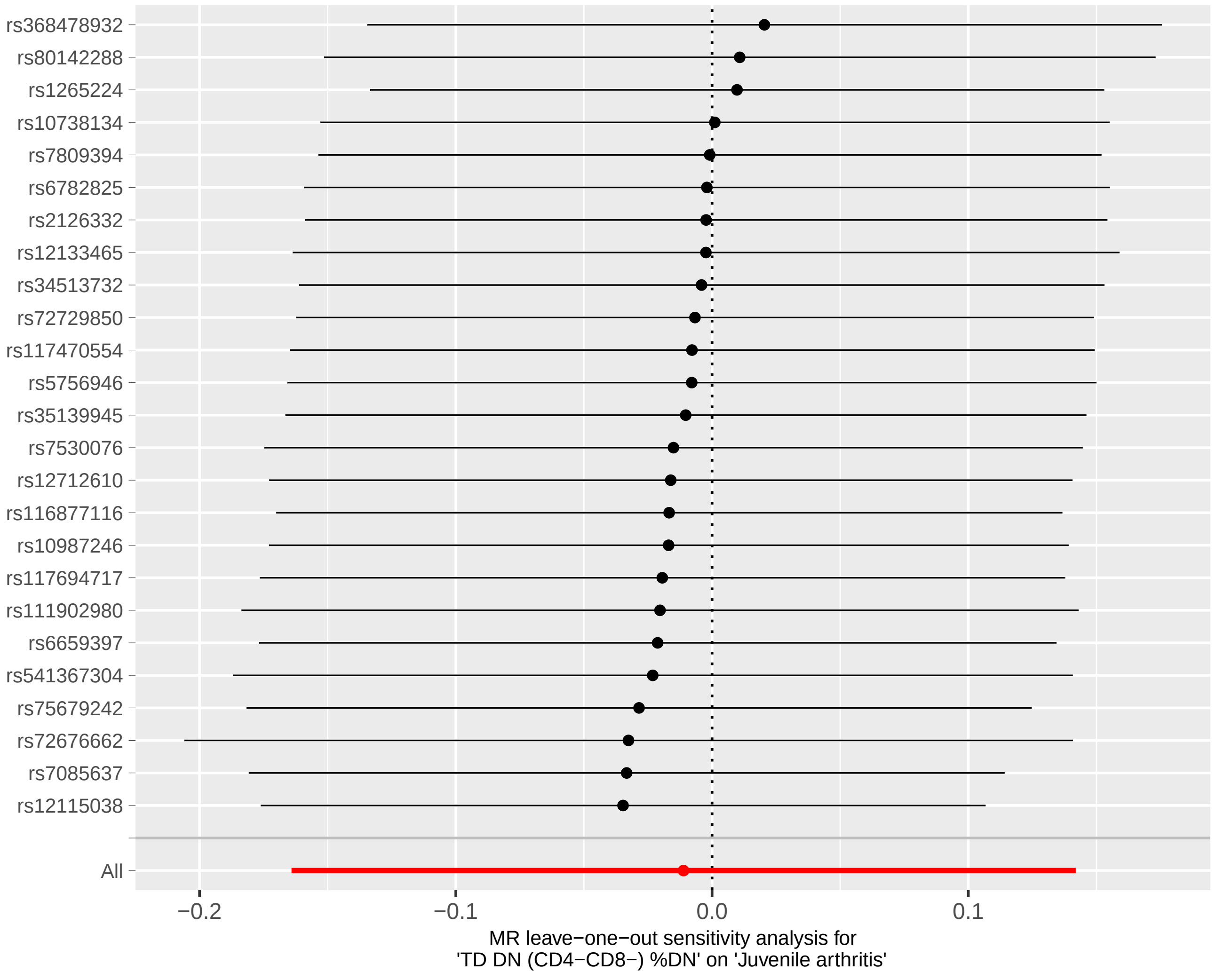
rs368478932
rs80142288
rs1265224
rs10738134
rs7809394
rs6782825
rs2126332
rs12133465
rs34513732
rs72729850
rs117470554
rs5756946
rs35139945
rs7530076
rs12712610
rs116877116
rs10987246
rs117694717
rs111902980
rs6659397
rs541367304
rs75679242
rs72676662
rs7085637
rs12115038
All
−0.2
−0.1
0.0
0.1
MR leave−one−out sensitivity analysis for
'TD DN (CD4−CD8−) %DN' on 'Juvenile arthritis'

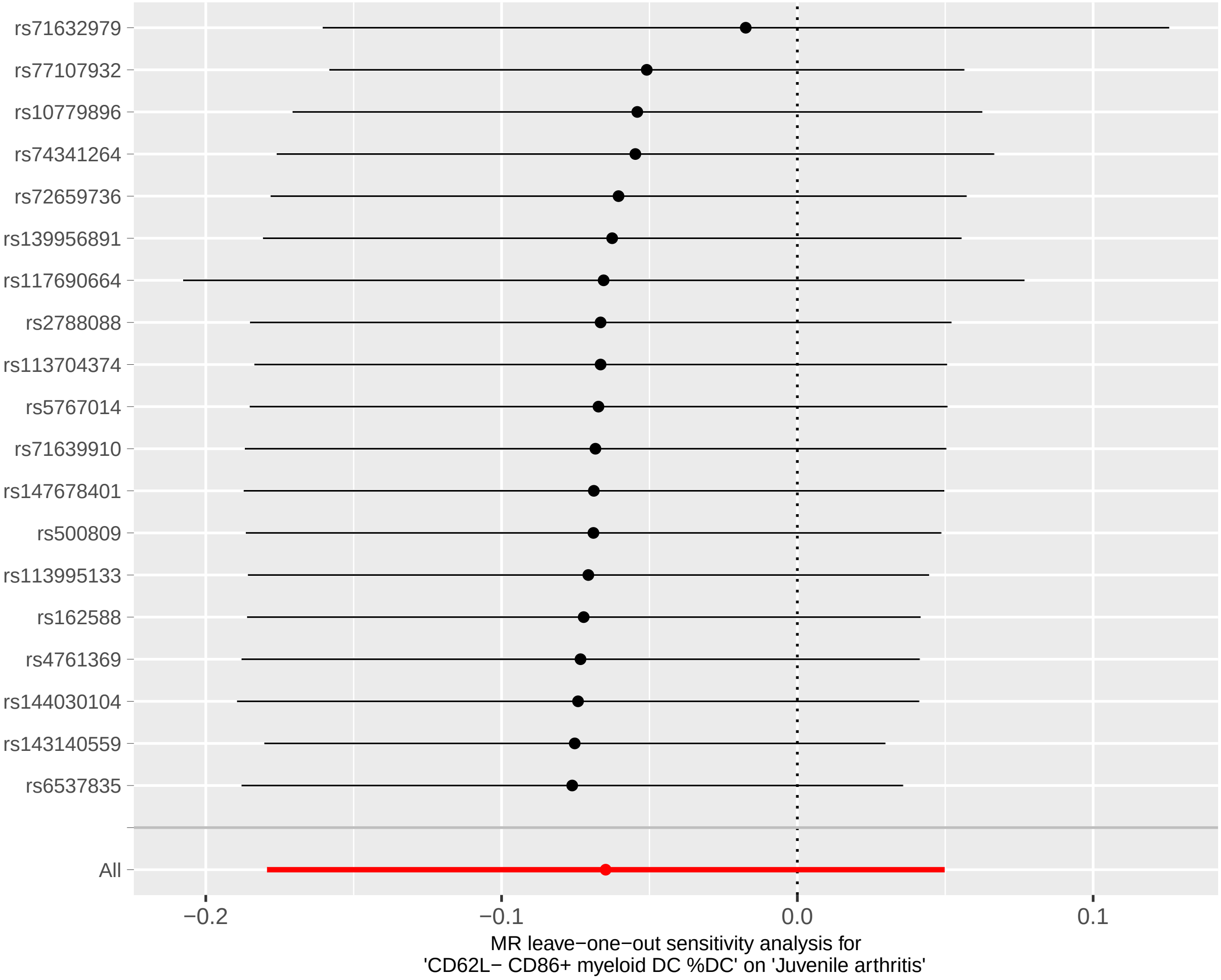
rs71632979
rs77107932
rs10779896
rs74341264
rs72659736
rs139956891
rs117690664
rs2788088
rs113704374
rs5767014
rs71639910
rs147678401
rs500809
rs113995133
rs162588
rs4761369
rs144030104
rs143140559
rs6537835
All
−0.2
−0.1
0.0
0.1
MR leave−one−out sensitivity analysis for
'CD62L− CD86+ myeloid DC %DC' on 'Juvenile arthritis'

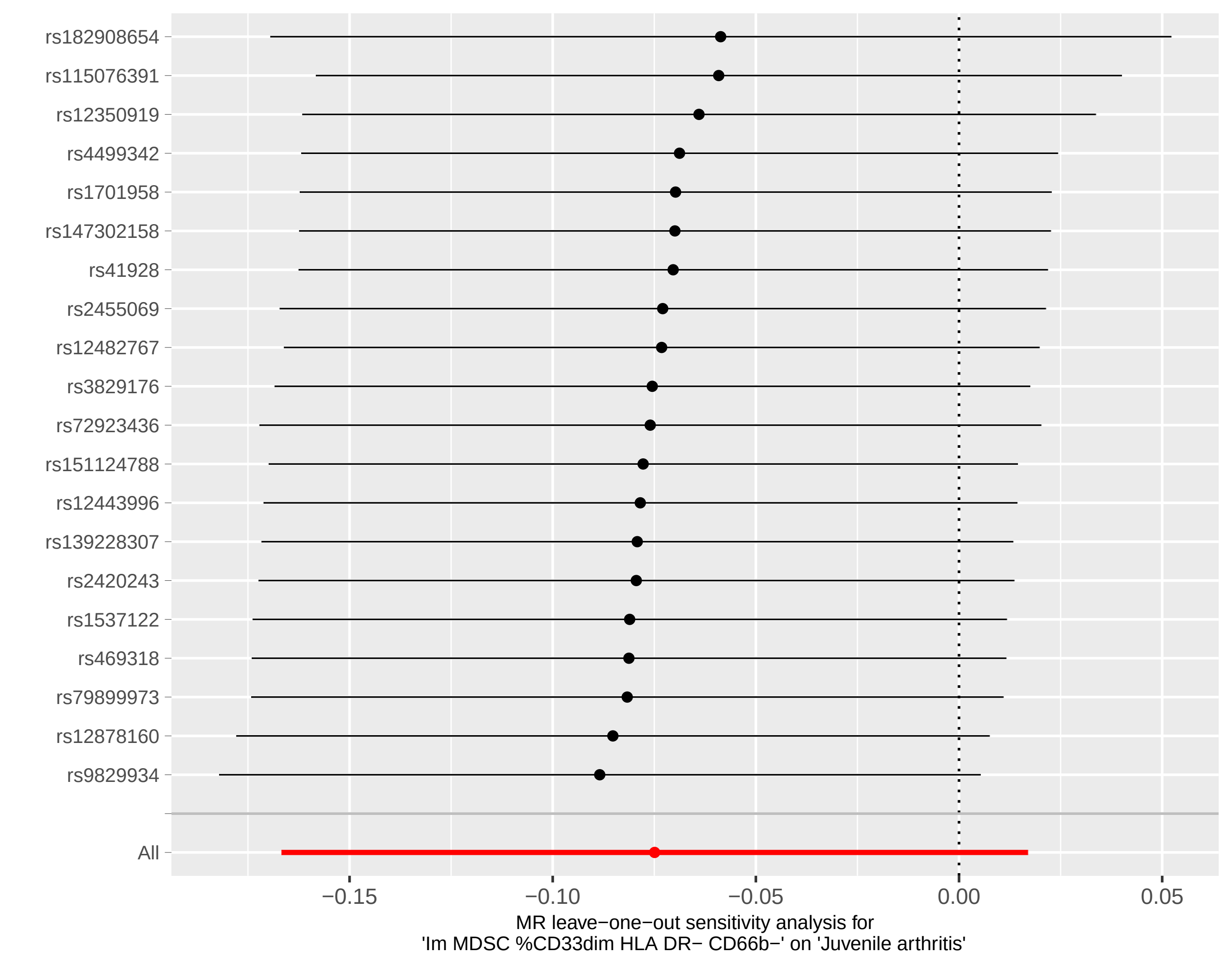
rs182908654
rs115076391
rs12350919
rs4499342
rs1701958
rs147302158
rs41928
rs2455069
rs12482767
rs3829176
rs72923436
rs151124788
rs12443996
rs139228307
rs2420243
rs1537122
rs469318
rs79899973
rs12878160
rs9829934
All
−0.15
−0.10
−0.05
0.00
0.05
MR leave−one−out sensitivity analysis for
'Im MDSC %CD33dim HLA DR− CD66b−' on 'Juvenile arthritis'

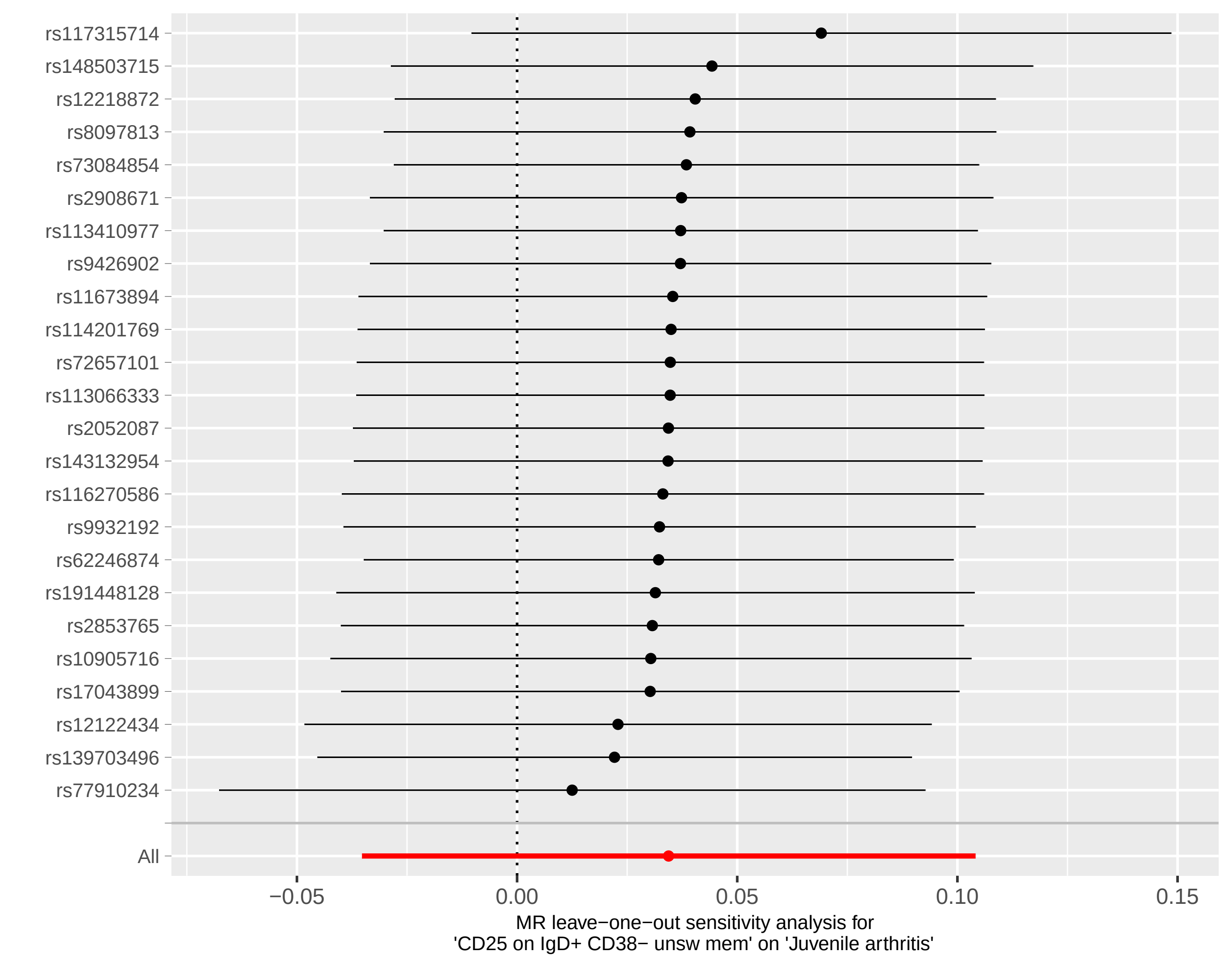

rs117315714
rs148503715
rs12218872
rs8097813
rs73084854
rs2908671
rs113410977
rs9426902
rs11673894
rs114201769
rs72657101
rs113066333
rs2052087
rs143132954
rs116270586
rs9932192
rs62246874
rs191448128
rs2853765
rs10905716
rs17043899
rs12122434
rs139703496
rs77910234
All
−0.05
0.00
0.05
0.10
0.15
MR leave−one−out sensitivity analysis for
'CD25 on IgD+ CD38− unsw mem' on 'Juvenile arthritis'

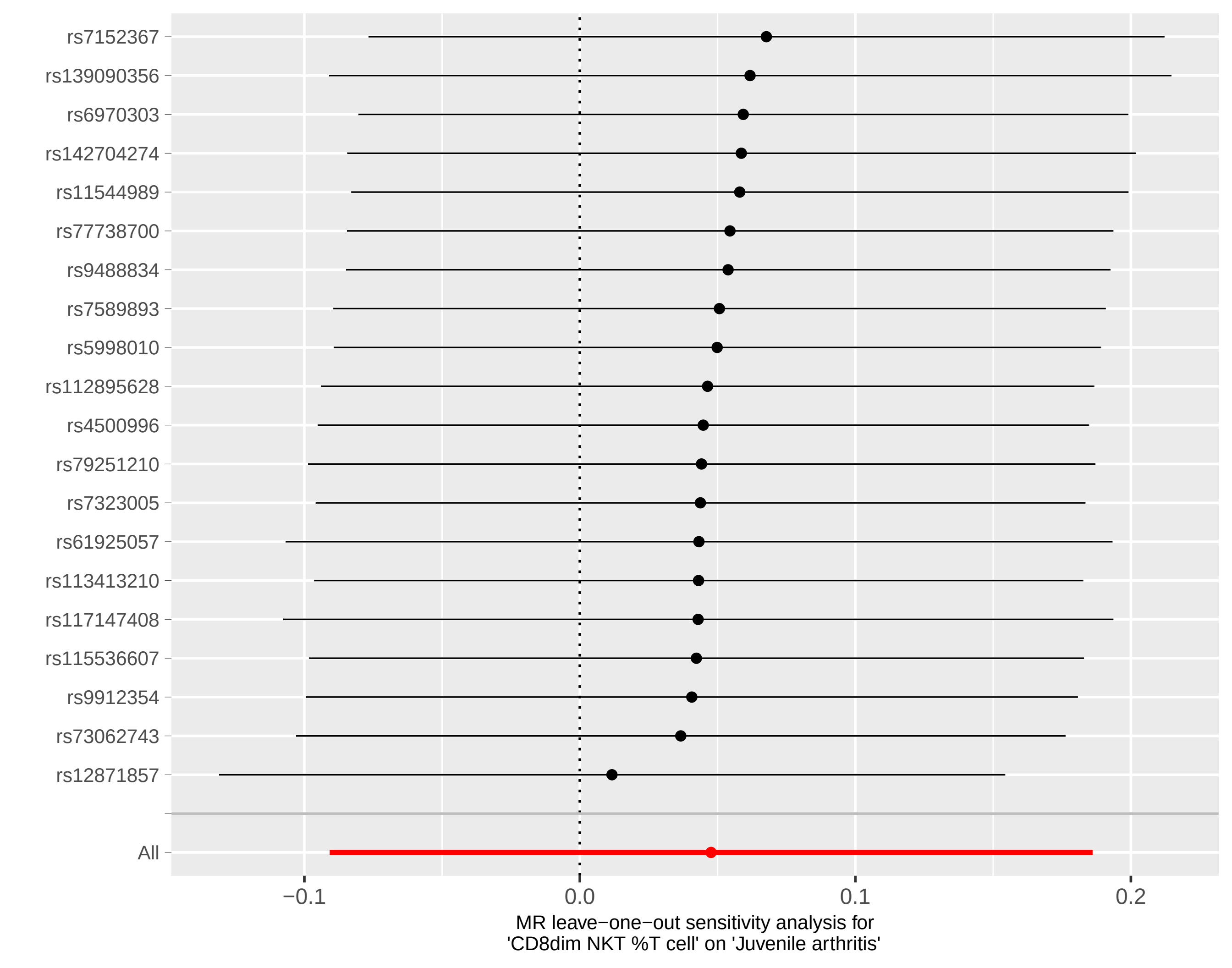

rs7152367
rs139090356
rs6970303
rs142704274
rs11544989
rs77738700
rs9488834
rs7589893
rs5998010
rs112895628
rs4500996
rs79251210
rs7323005
rs61925057
rs113413210
rs117147408
rs115536607
rs9912354
rs73062743
rs12871857
All
−0.1
0.0
0.1
0.2
MR leave−one−out sensitivity analysis for
'CD8dim NKT %T cell' on 'Juvenile arthritis'

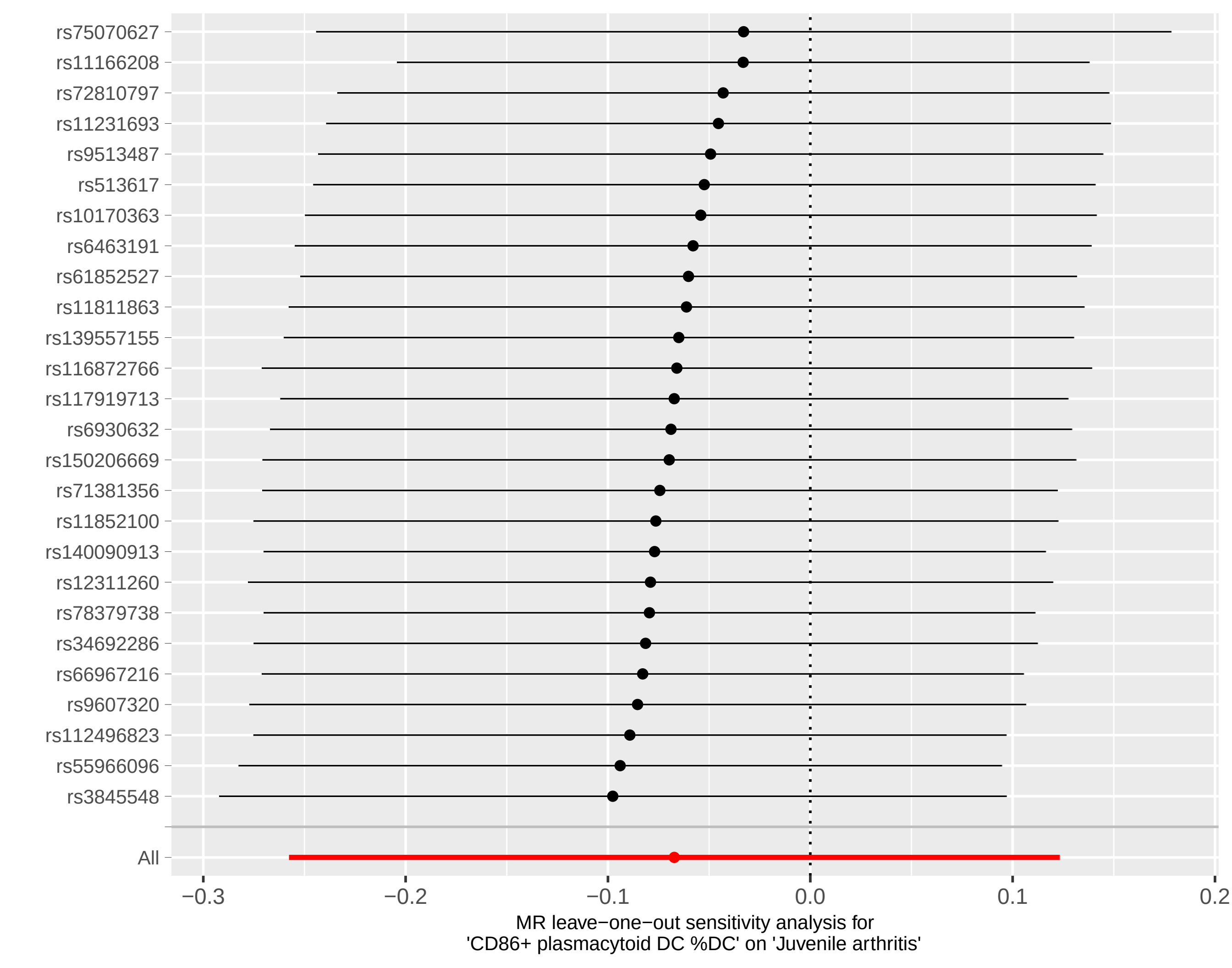
rs75070627
rs11166208
rs72810797
rs11231693
rs9513487
rs513617
rs10170363
rs6463191
rs61852527
rs11811863
rs139557155
rs116872766
rs117919713
rs6930632
rs150206669
rs71381356
rs11852100
rs140090913
rs12311260
rs78379738
rs34692286
rs66967216
rs9607320
rs112496823
rs55966096
rs3845548
All
−0.3
−0.2
−0.1
0.0
0.1
0.2
MR leave−one−out sensitivity analysis for
'CD86+ plasmacytoid DC %DC' on 'Juvenile arthritis'

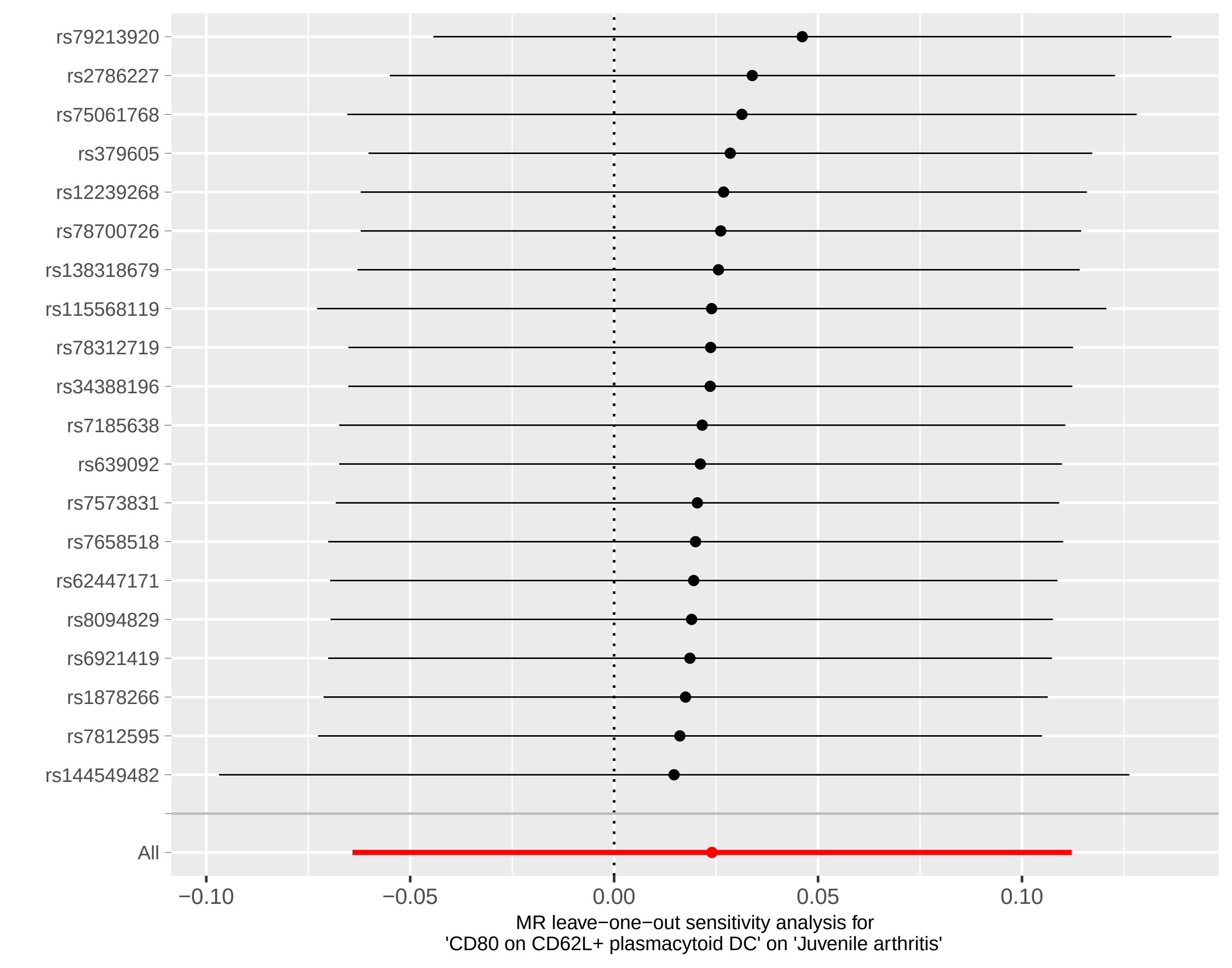
rs79213920
rs2786227
rs75061768
rs379605
rs12239268
rs78700726
rs138318679
rs115568119
rs78312719
rs34388196
rs7185638
rs639092
rs7573831
rs7658518
rs62447171
rs8094829
rs6921419
rs1878266
rs7812595
rs144549482
All
−0.10
−0.05
0.00
0.05
0.10
MR leave−one−out sensitivity analysis for
'CD80 on CD62L+ plasmacytoid DC' on 'Juvenile arthritis'

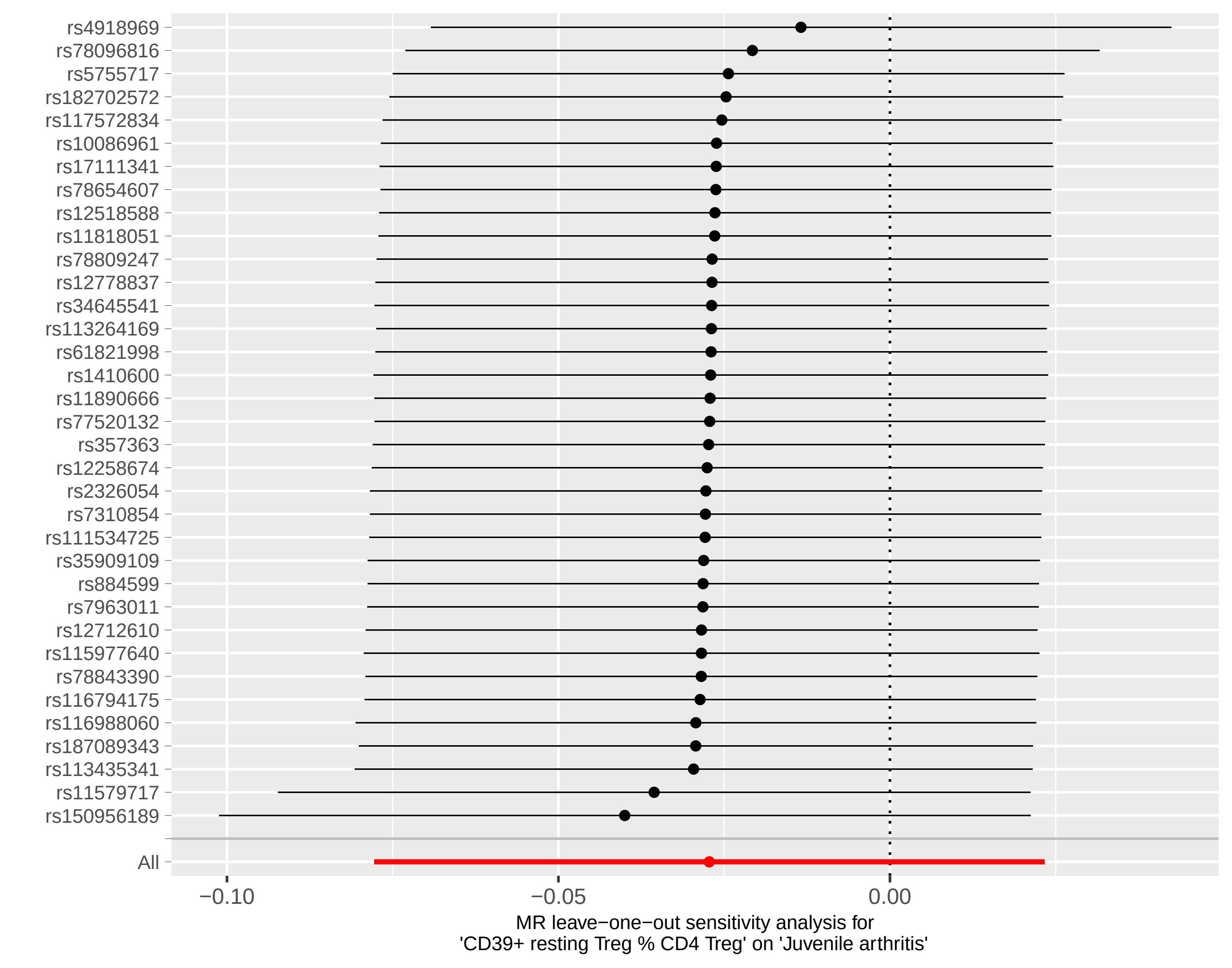

rs4918969
rs78096816
rs5755717
rs182702572
rs117572834
rs10086961
rs17111341
rs78654607
rs12518588
rs11818051
rs78809247
rs12778837
rs34645541
rs113264169
rs61821998
rs1410600
rs11890666
rs77520132
rs357363
rs12258674
rs2326054
rs7310854
rs111534725
rs35909109
rs884599
rs7963011
rs12712610
rs115977640
rs78843390
rs116794175
rs116988060
rs187089343
rs113435341
rs11579717
rs150956189
All
−0.10
−0.05
0.00
MR leave−one−out sensitivity analysis for
'CD39+ resting Treg % CD4 Treg' on 'Juvenile arthritis'

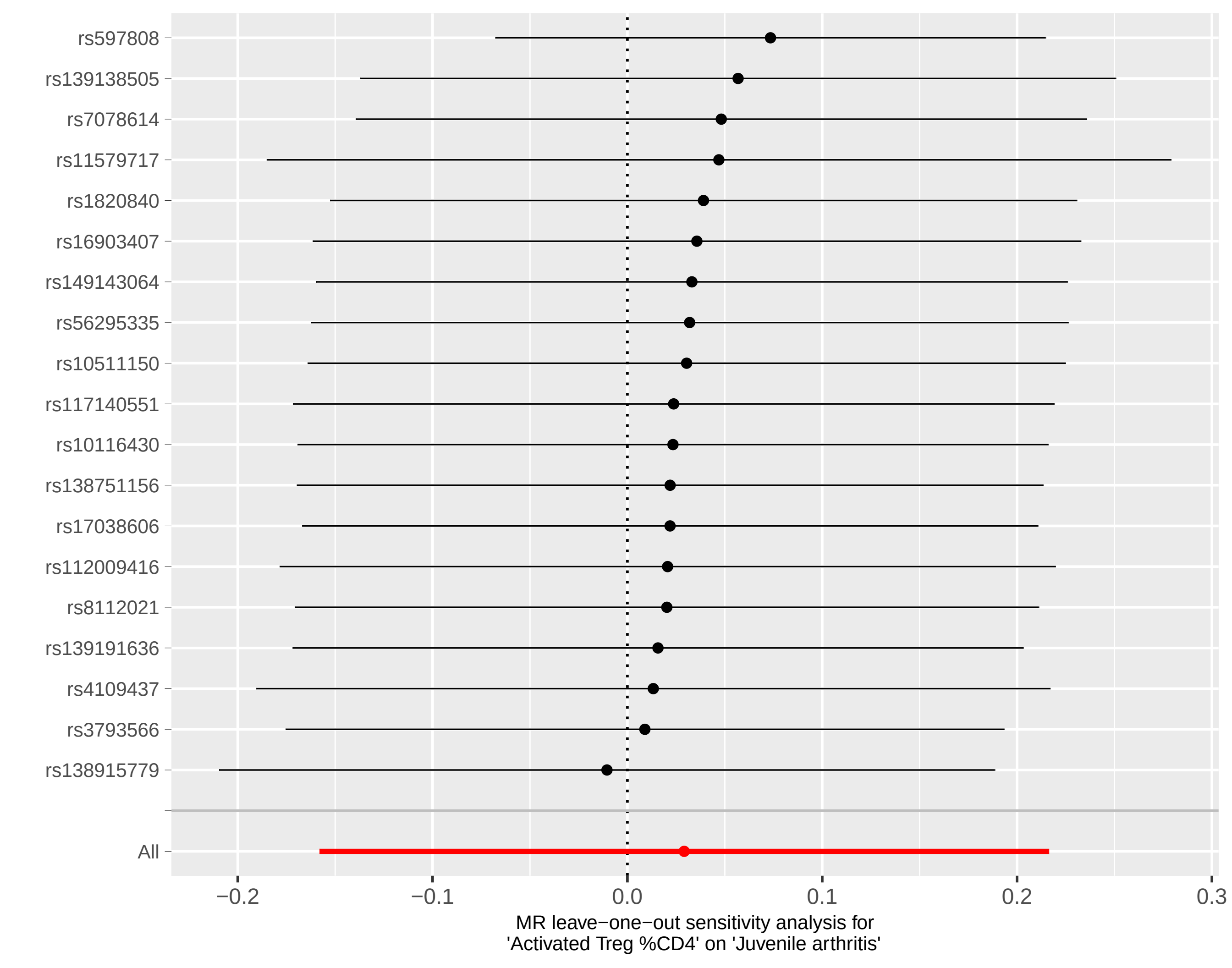

rs597808
rs139138505
rs7078614
rs11579717
rs1820840
rs16903407
rs149143064
rs56295335
rs10511150
rs117140551
rs10116430
rs138751156
rs17038606
rs112009416
rs8112021
rs139191636
rs4109437
rs3793566
rs138915779
All
−0.2
−0.1
0.0
0.1
0.2
0.3
MR leave−one−out sensitivity analysis for
'Activated Treg %CD4' on 'Juvenile arthritis'

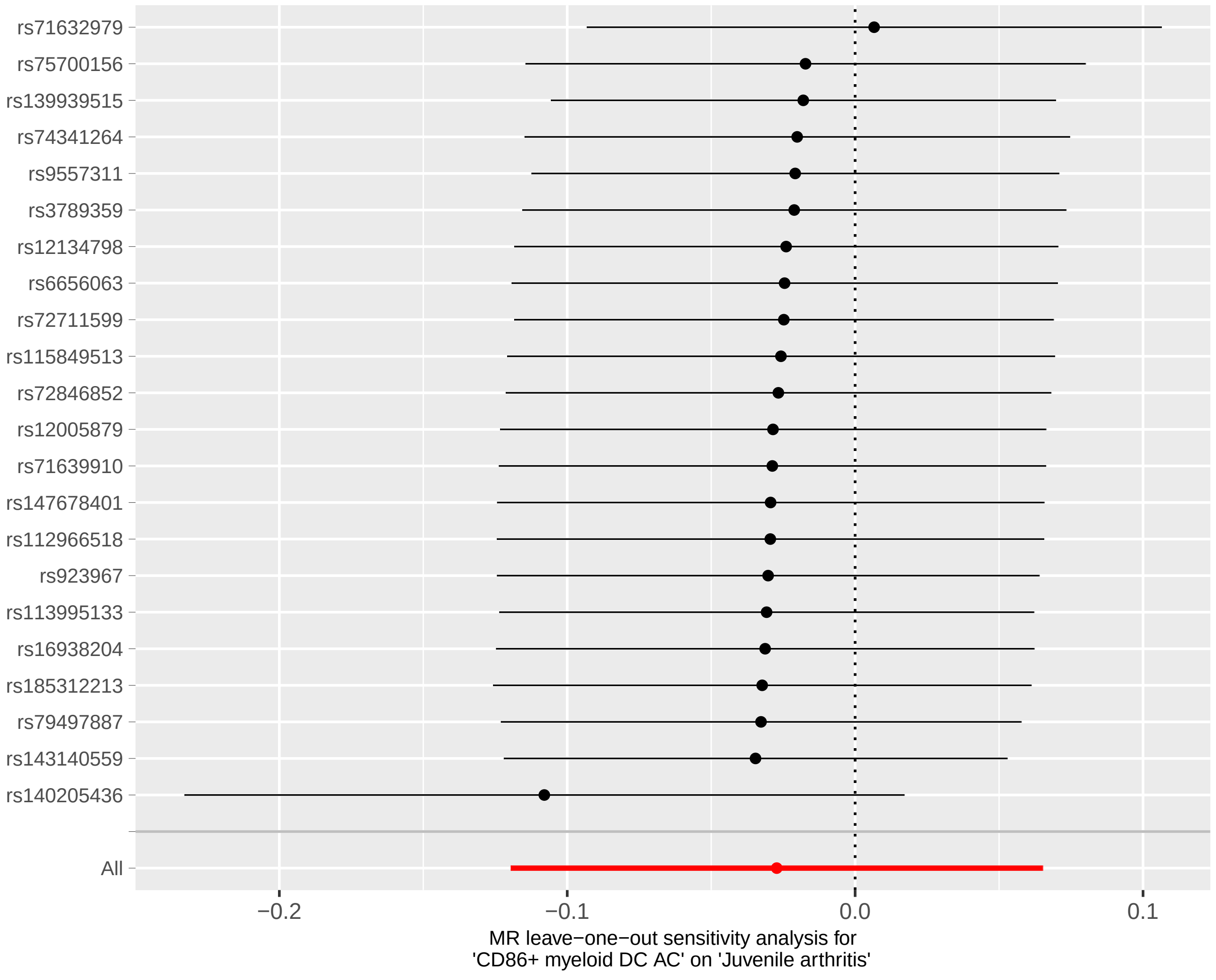
rs71632979
rs75700156
rs139939515
rs74341264
rs9557311
rs3789359
rs12134798
rs6656063
rs72711599
rs115849513
rs72846852
rs12005879
rs71639910
rs147678401
rs112966518
rs923967
rs113995133
rs16938204
rs185312213
rs79497887
rs143140559
rs140205436
All
−0.2
−0.1
0.0
0.1
MR leave−one−out sensitivity analysis for
'CD86+ myeloid DC AC' on 'Juvenile arthritis'

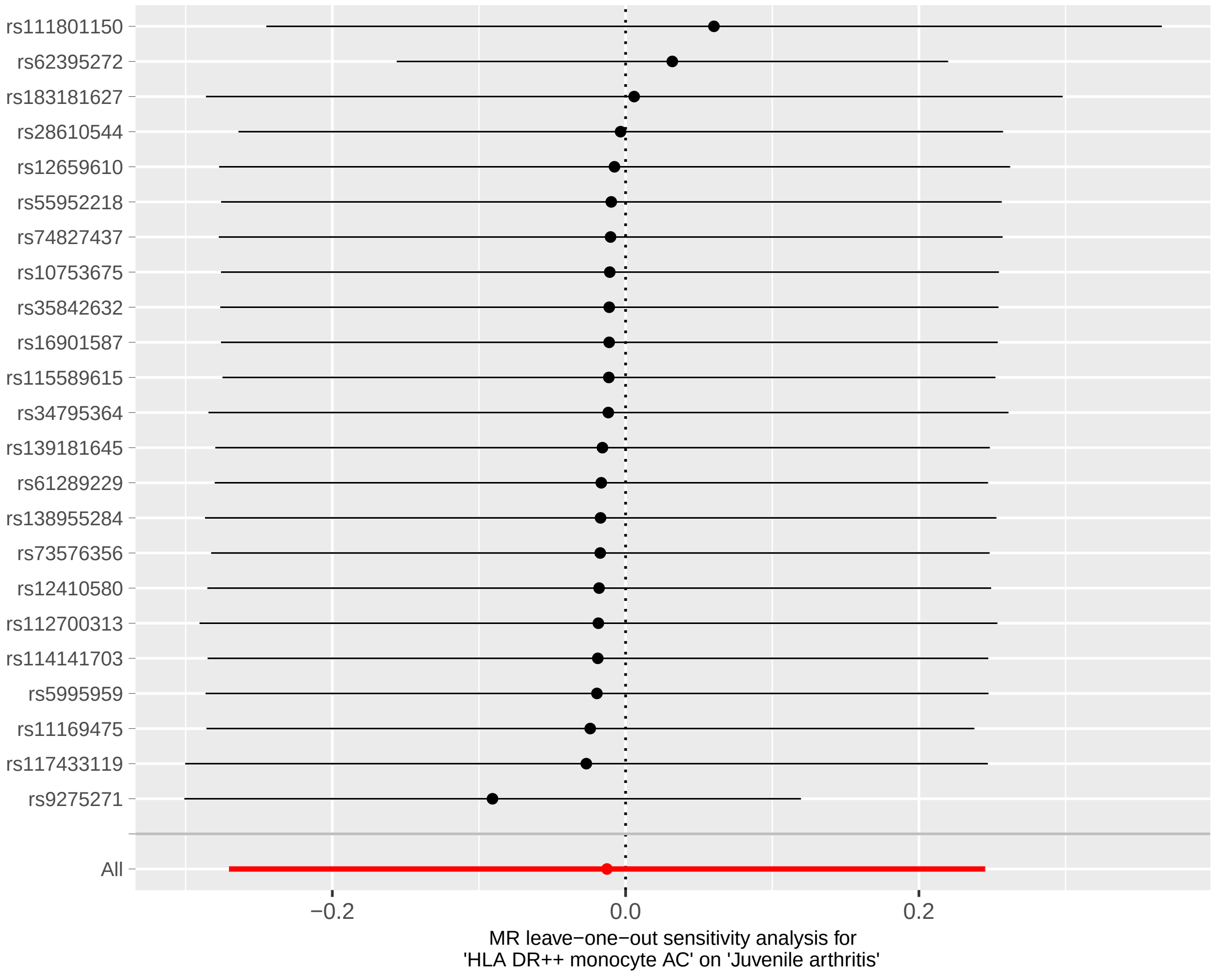

rs111801150
rs62395272
rs183181627
rs28610544
rs12659610
rs55952218
rs74827437
rs10753675
rs35842632
rs16901587
rs115589615
rs34795364
rs139181645
rs61289229
rs138955284
rs73576356
rs12410580
rs112700313
rs114141703
rs5995959
rs11169475
rs117433119
rs9275271
All
−0.2
0.0
0.2
MR leave−one−out sensitivity analysis for
'HLA DR++ monocyte AC' on 'Juvenile arthritis'

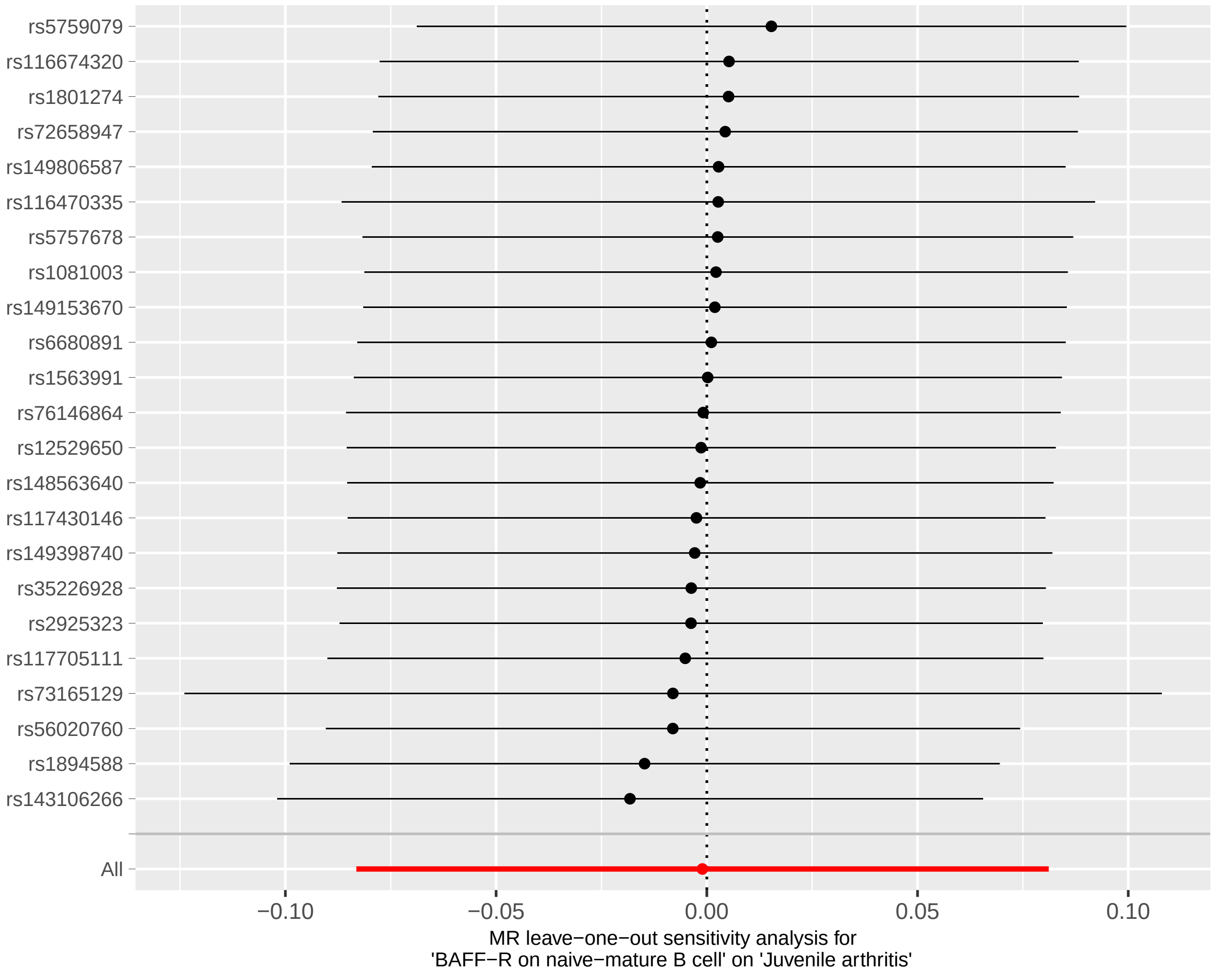

rs5759079
rs116674320
rs1801274
rs72658947
rs149806587
rs116470335
rs5757678
rs1081003
rs149153670
rs6680891
rs1563991
rs76146864
rs12529650
rs148563640
rs117430146
rs149398740
rs35226928
rs2925323
rs117705111
rs73165129
rs56020760
rs1894588
rs143106266
All
−0.10
−0.05
0.00
0.05
0.10
MR leave−one−out sensitivity analysis for
'BAFF−R on naive−mature B cell' on 'Juvenile arthritis'

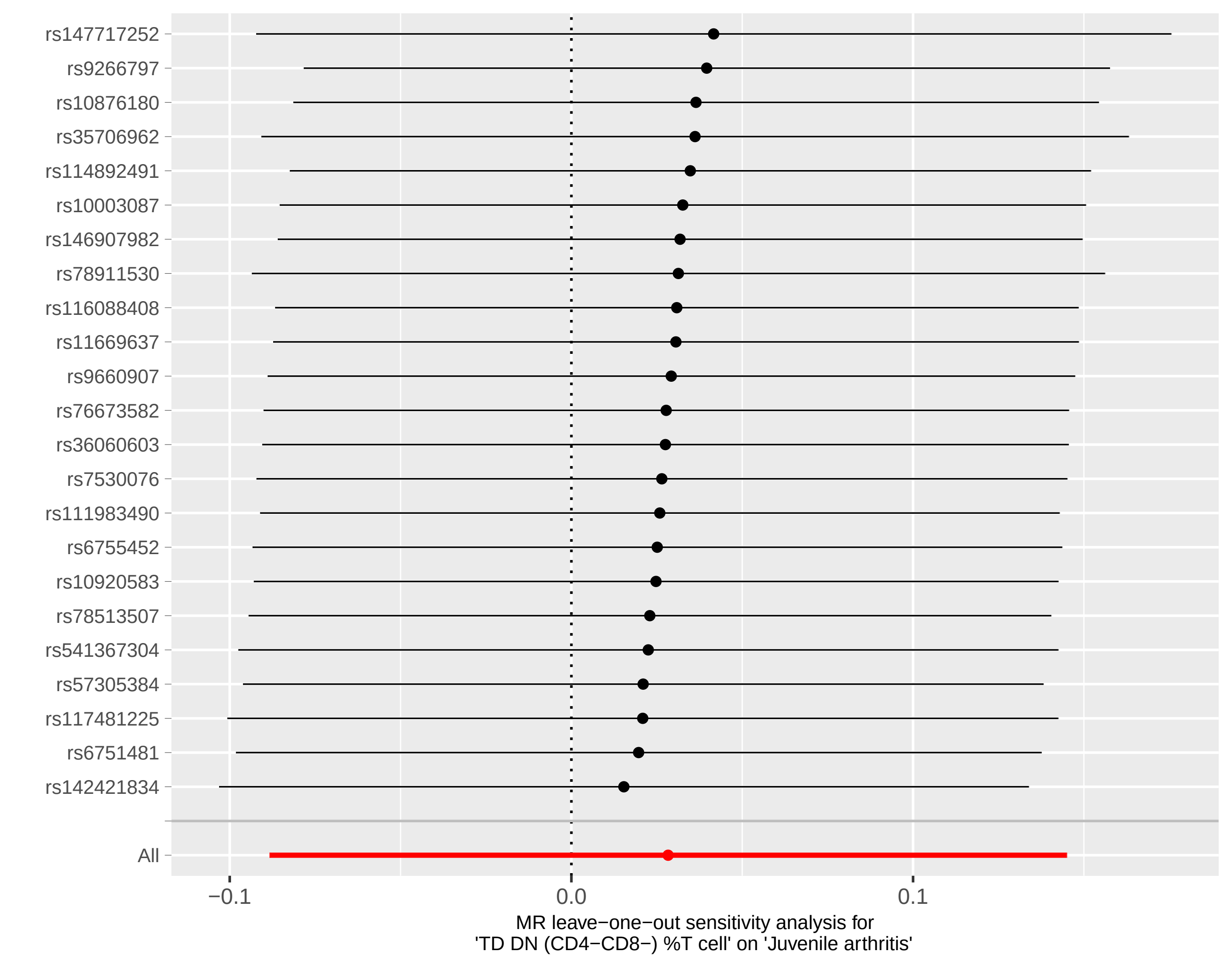
rs147717252
rs9266797
rs10876180
rs35706962
rs114892491
rs10003087
rs146907982
rs78911530
rs116088408
rs11669637
rs9660907
rs76673582
rs36060603
rs7530076
rs111983490
rs6755452
rs10920583
rs78513507
rs541367304
rs57305384
rs117481225
rs6751481
rs142421834
All
−0.1
0.0
0.1
MR leave−one−out sensitivity analysis for
'TD DN (CD4−CD8−) %T cell' on 'Juvenile arthritis'

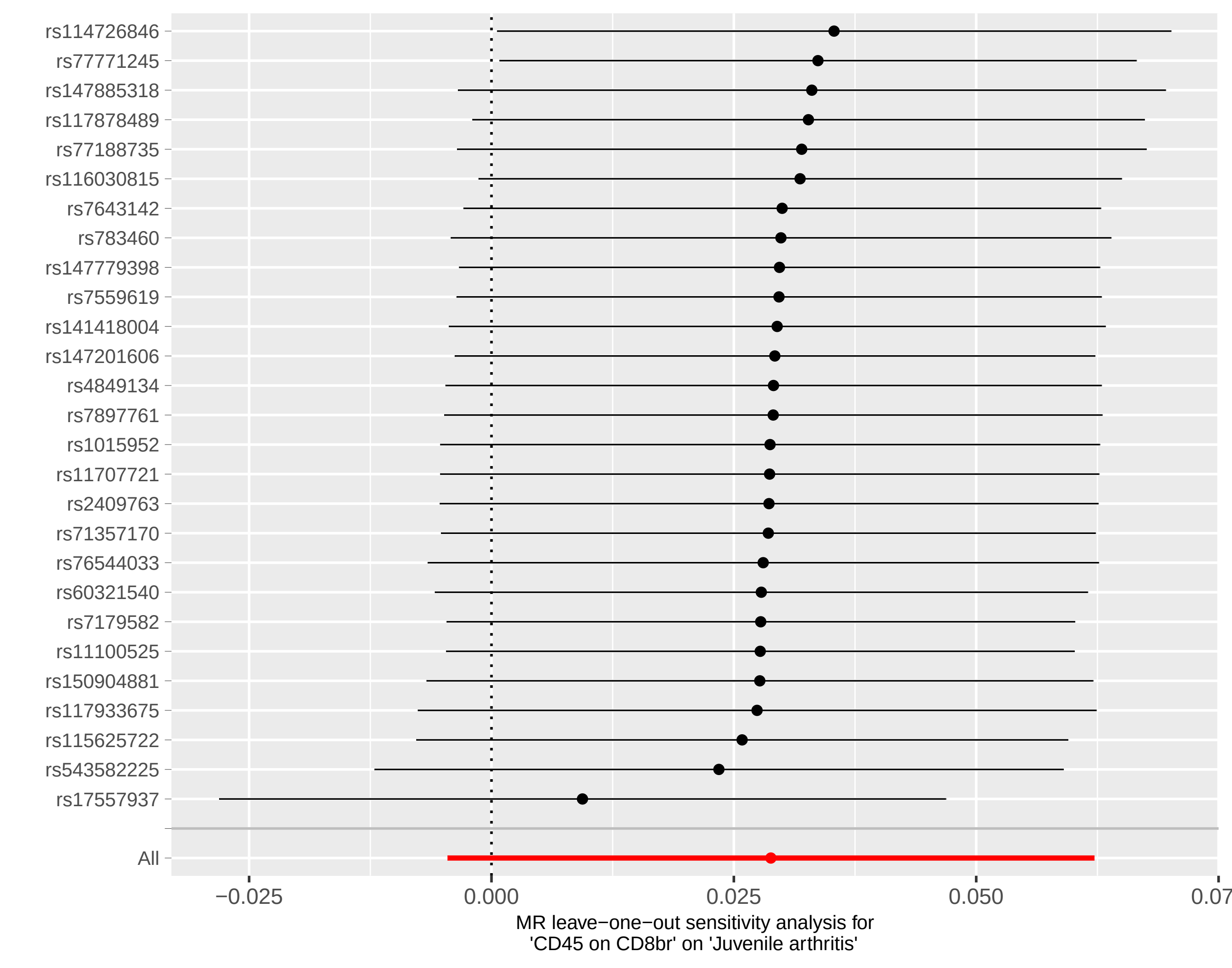

rs114726846
rs77771245
rs147885318
rs117878489
rs77188735
rs116030815
rs7643142
rs783460
rs147779398
rs7559619
rs141418004
rs147201606
rs4849134
rs7897761
rs1015952
rs11707721
rs2409763
rs71357170
rs76544033
rs60321540
rs7179582
rs11100525
rs150904881
rs117933675
rs115625722
rs543582225
rs17557937
All
−0.025
0.000
0.025
0.050
0.07
MR leave−one−out sensitivity analysis for
'CD45 on CD8br' on 'Juvenile arthritis'

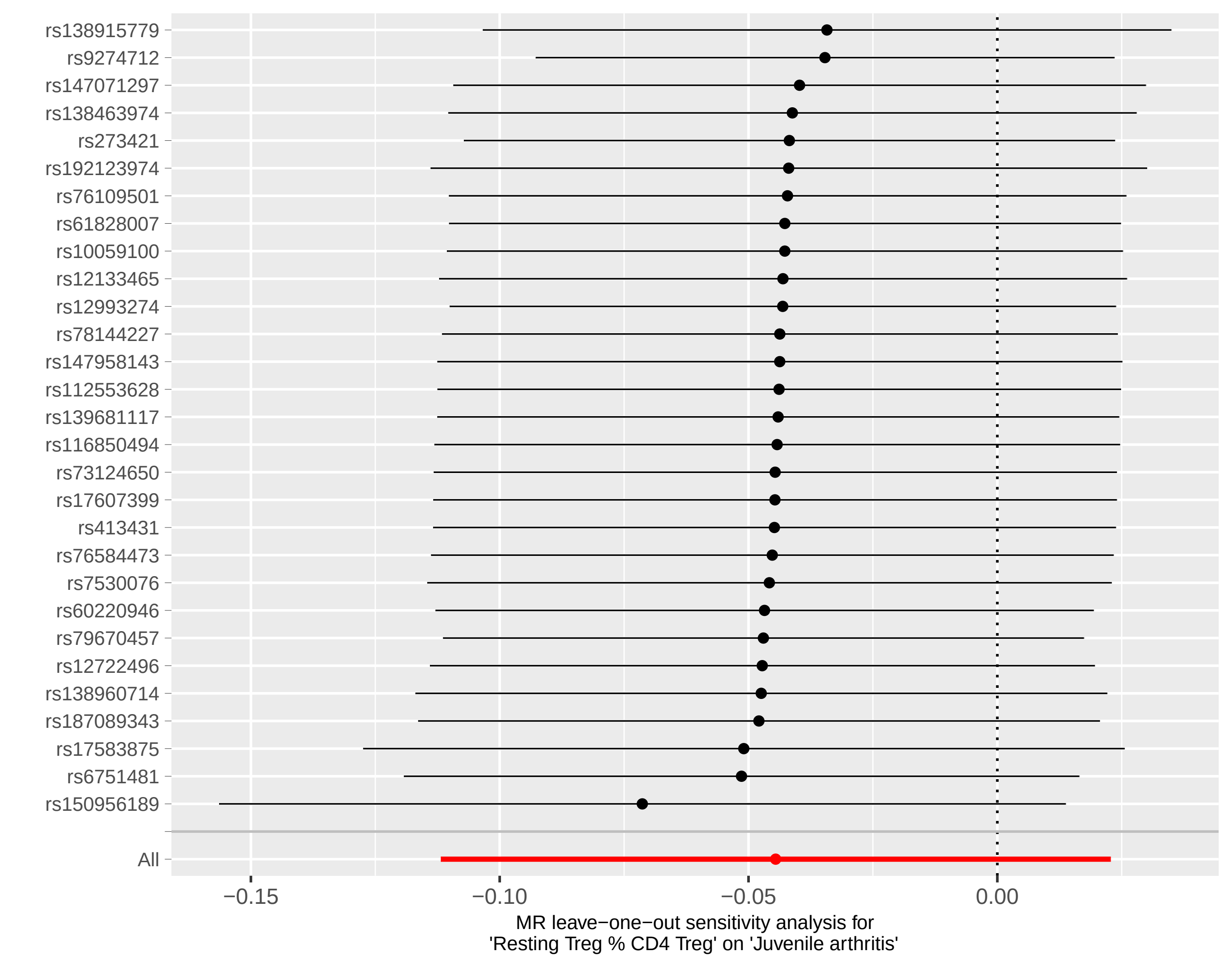
rs138915779
rs9274712
rs147071297
rs138463974
rs273421
rs192123974
rs76109501
rs61828007
rs10059100
rs12133465
rs12993274
rs78144227
rs147958143
rs112553628
rs139681117
rs116850494
rs73124650
rs17607399
rs413431
rs76584473
rs7530076
rs60220946
rs79670457
rs12722496
rs138960714
rs187089343
rs17583875
rs6751481
rs150956189
All
−0.15
−0.10
−0.05
0.00
MR leave−one−out sensitivity analysis for
'Resting Treg % CD4 Treg' on 'Juvenile arthritis'

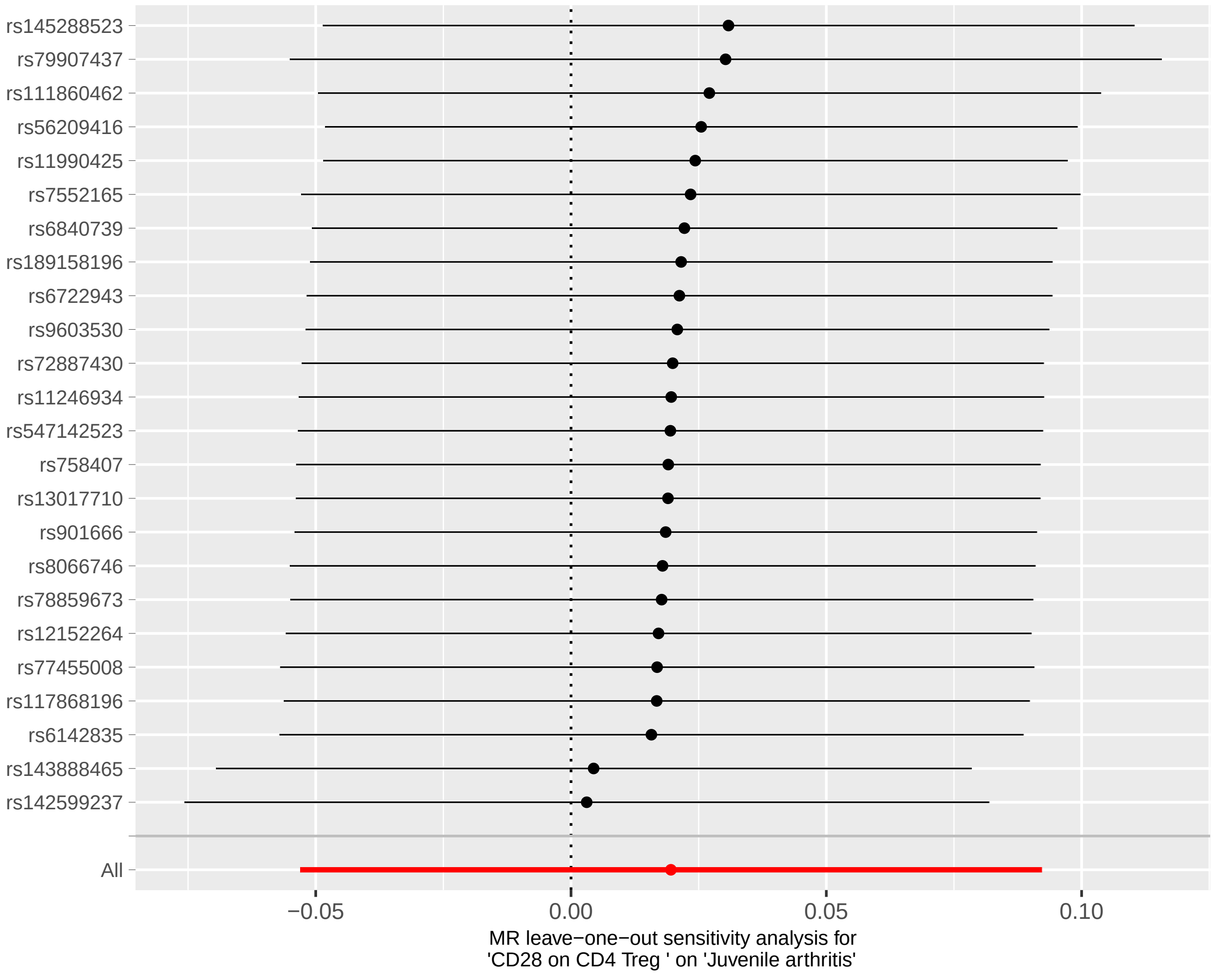

rs145288523
rs79907437
rs111860462
rs56209416
rs11990425
rs7552165
rs6840739
rs189158196
rs6722943
rs9603530
rs72887430
rs11246934
rs547142523
rs758407
rs13017710
rs901666
rs8066746
rs78859673
rs12152264
rs77455008
rs117868196
rs6142835
rs143888465
rs142599237
All
−0.05
0.00
0.05
0.10
MR leave−one−out sensitivity analysis for
'CD28 on CD4 Treg ' on 'Juvenile arthritis'

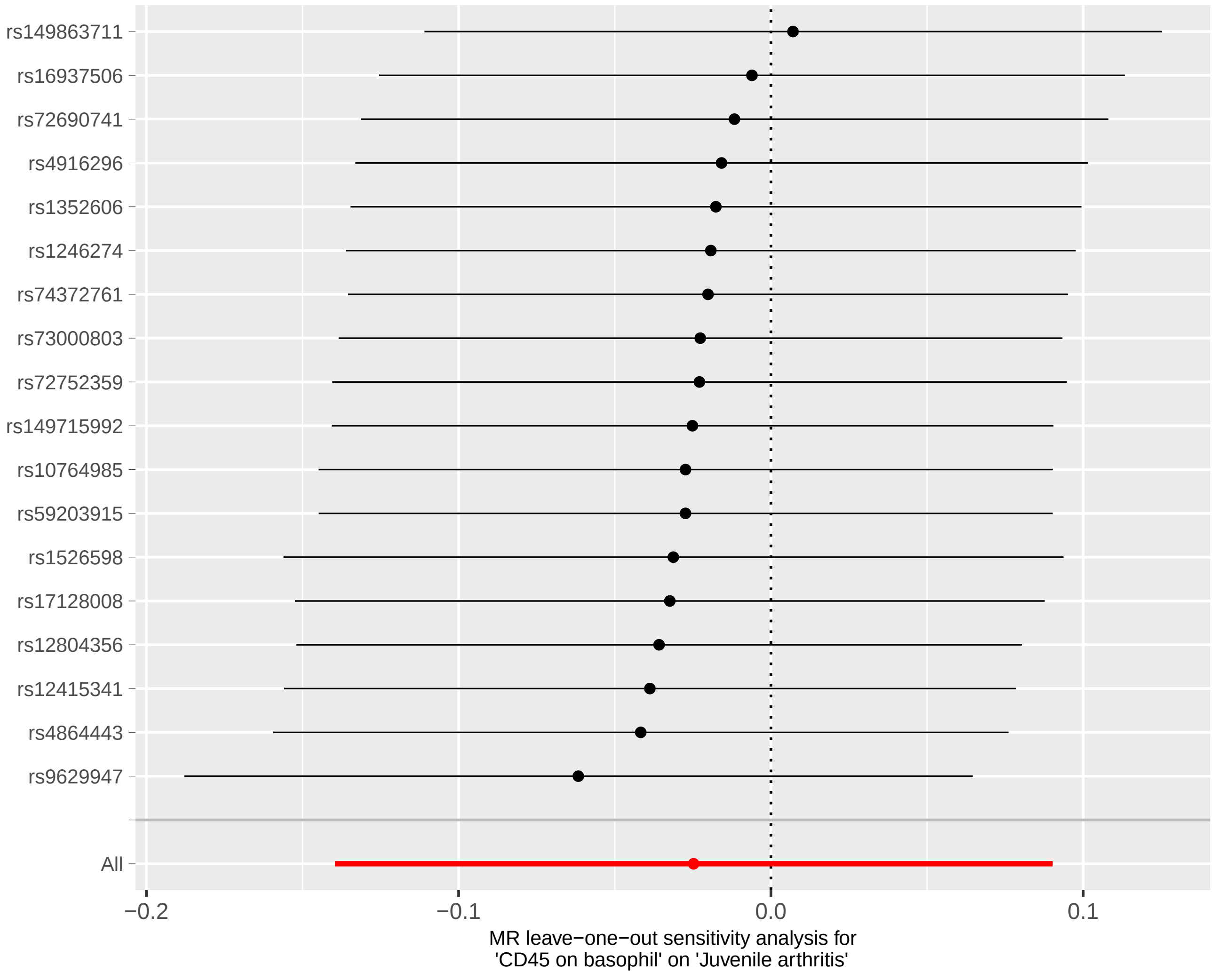
rs149863711
rs16937506
rs72690741
rs4916296
rs1352606
rs1246274
rs74372761
rs73000803
rs72752359
rs149715992
rs10764985
rs59203915
rs1526598
rs17128008
rs12804356
rs12415341
rs4864443
rs9629947
All
−0.2
−0.1
0.0
0.1
MR leave−one−out sensitivity analysis for
'CD45 on basophil' on 'Juvenile arthritis'

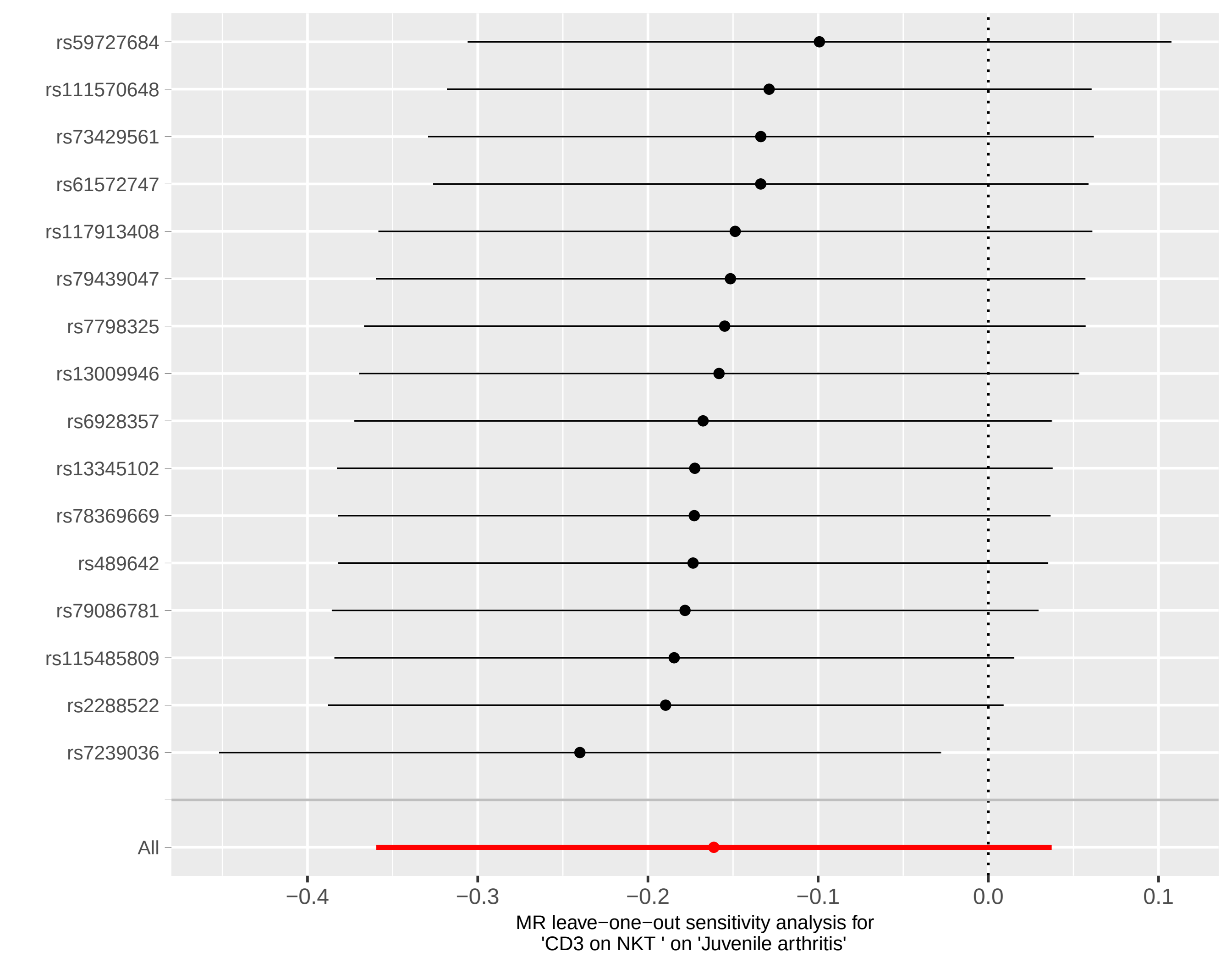

rs59727684
rs111570648
rs73429561
rs61572747
rs117913408
rs79439047
rs7798325
rs13009946
rs6928357
rs13345102
rs78369669
rs489642
rs79086781
rs115485809
rs2288522
rs7239036
All
−0.4
−0.3
−0.2
−0.1
0.0
0.1
MR leave−one−out sensitivity analysis for
'CD3 on NKT ' on 'Juvenile arthritis'

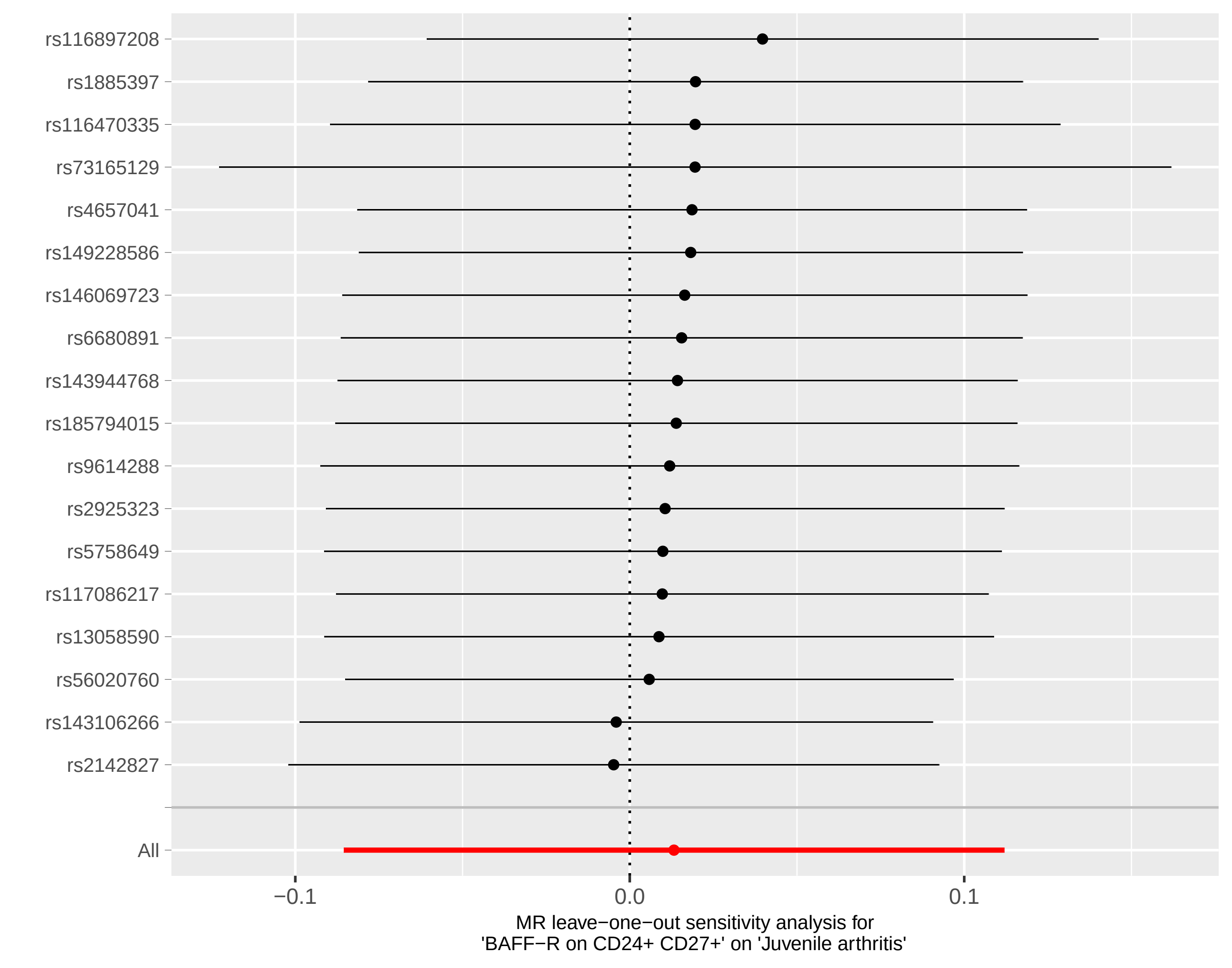

rs116897208
rs1885397
rs116470335
rs73165129
rs4657041
rs149228586
rs146069723
rs6680891
rs143944768
rs185794015
rs9614288
rs2925323
rs5758649
rs117086217
rs13058590
rs56020760
rs143106266
rs2142827
All
−0.1
0.0
0.1
MR leave−one−out sensitivity analysis for
'BAFF−R on CD24+ CD27+' on 'Juvenile arthritis'

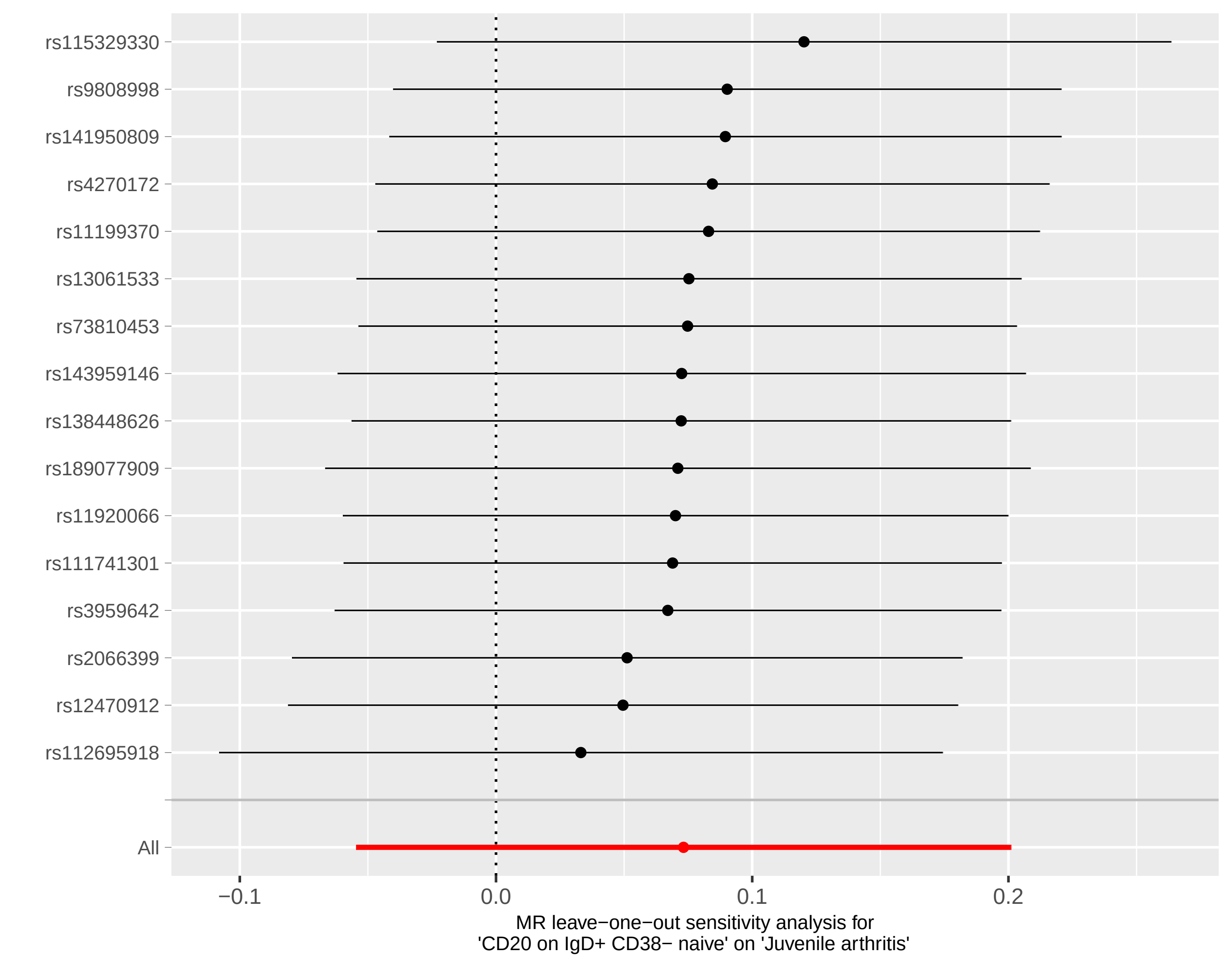

rs115329330
rs9808998
rs141950809
rs4270172
rs11199370
rs13061533
rs73810453
rs143959146
rs138448626
rs189077909
rs11920066
rs111741301
rs3959642
rs2066399
rs12470912
rs112695918
All
−0.1
0.0
0.1
0.2
MR leave−one−out sensitivity analysis for
'CD20 on IgD+ CD38− naive' on 'Juvenile arthritis'

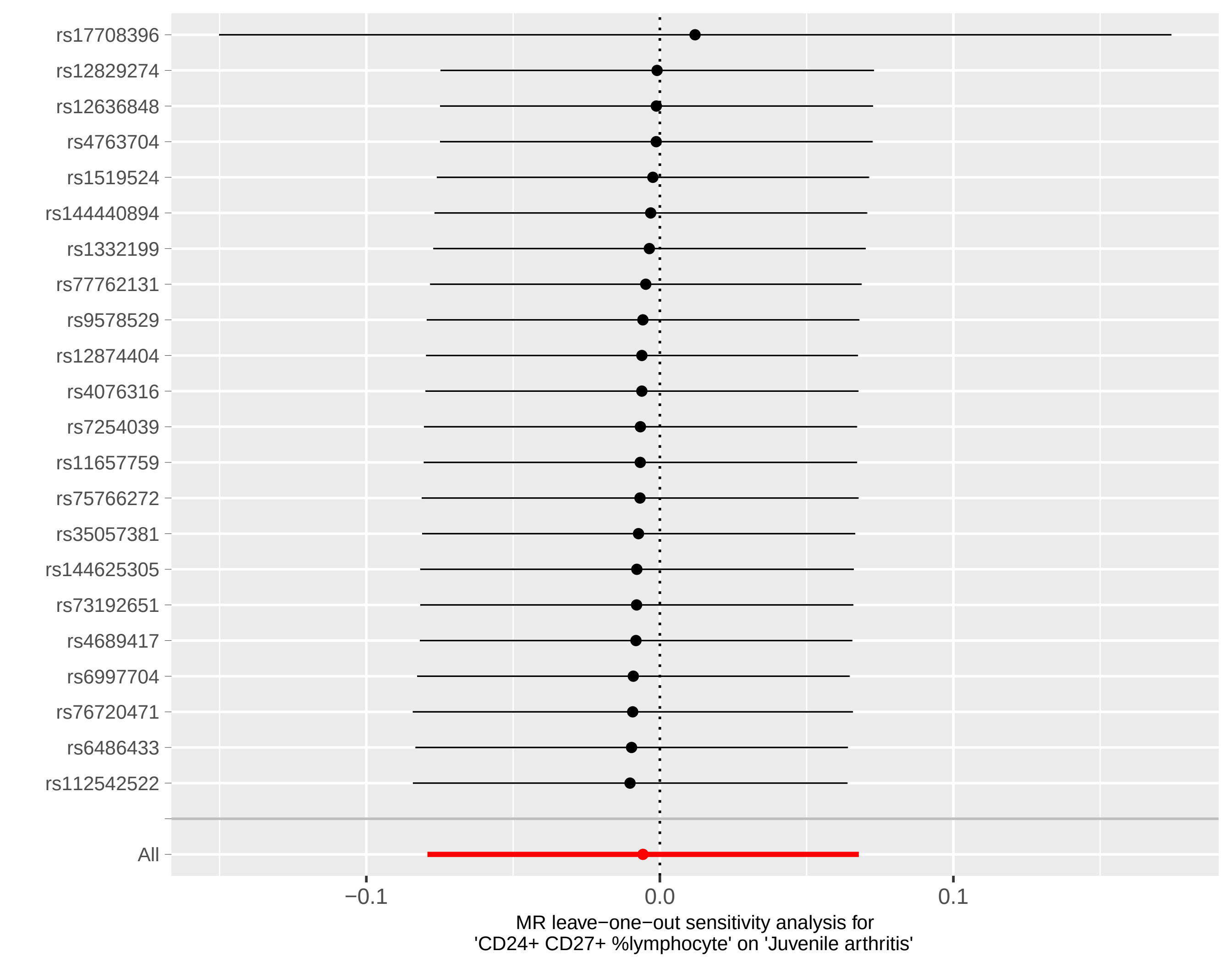
rs17708396
rs12829274
rs12636848
rs4763704
rs1519524
rs144440894
rs1332199
rs77762131
rs9578529
rs12874404
rs4076316
rs7254039
rs11657759
rs75766272
rs35057381
rs144625305
rs73192651
rs4689417
rs6997704
rs76720471
rs6486433
rs112542522
All
−0.1
0.0
0.1
MR leave−one−out sensitivity analysis for
'CD24+ CD27+ %lymphocyte' on 'Juvenile arthritis'

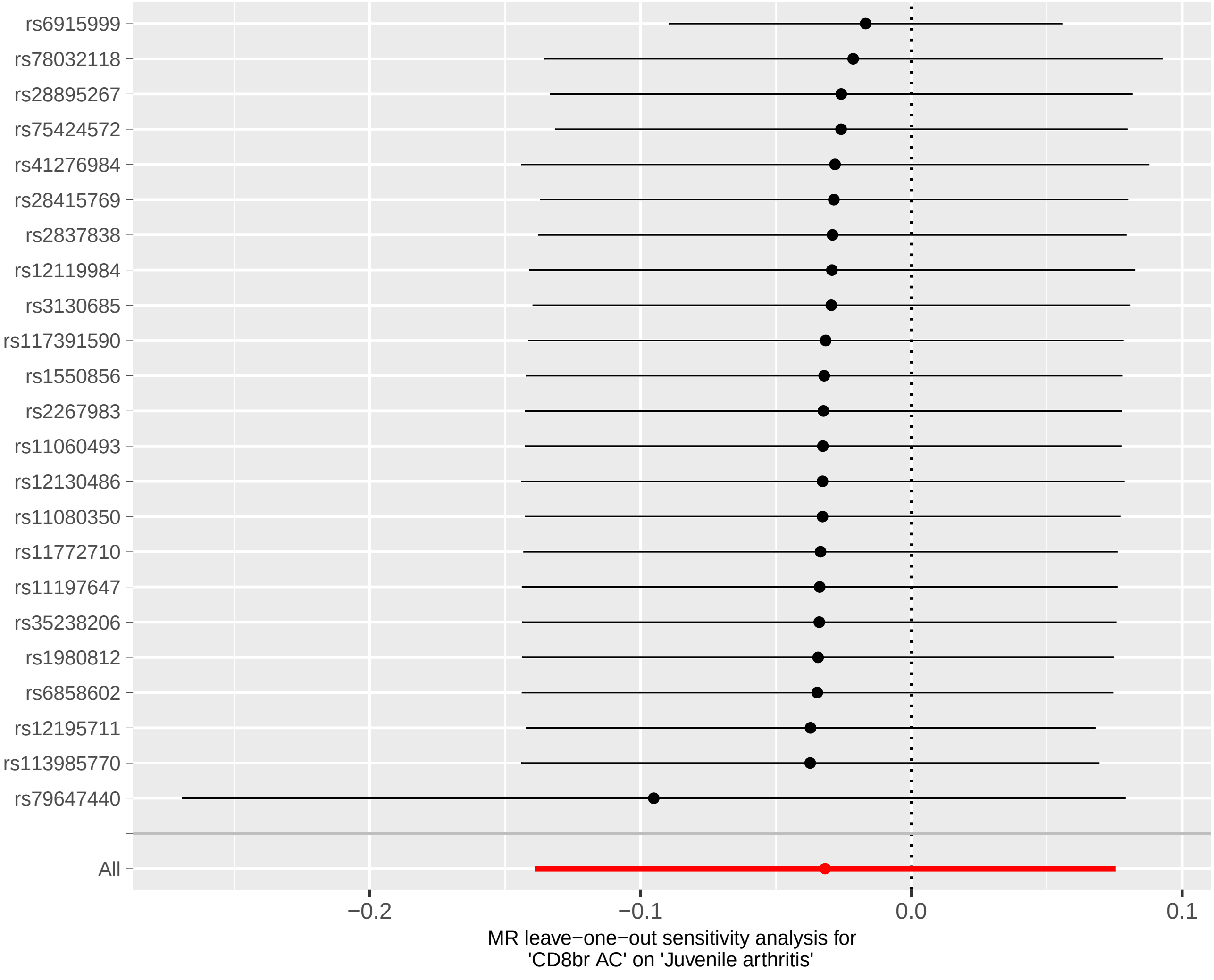
rs6915999
rs78032118
rs28895267
rs75424572
rs41276984
rs28415769
rs2837838
rs12119984
rs3130685
rs117391590
rs1550856
rs2267983
rs11060493
rs12130486
rs11080350
rs11772710
rs11197647
rs35238206
rs1980812
rs6858602
rs12195711
rs113985770
rs79647440
All
−0.2
−0.1
0.0
0.1
MR leave−one−out sensitivity analysis for
'CD8br AC' on 'Juvenile arthritis'

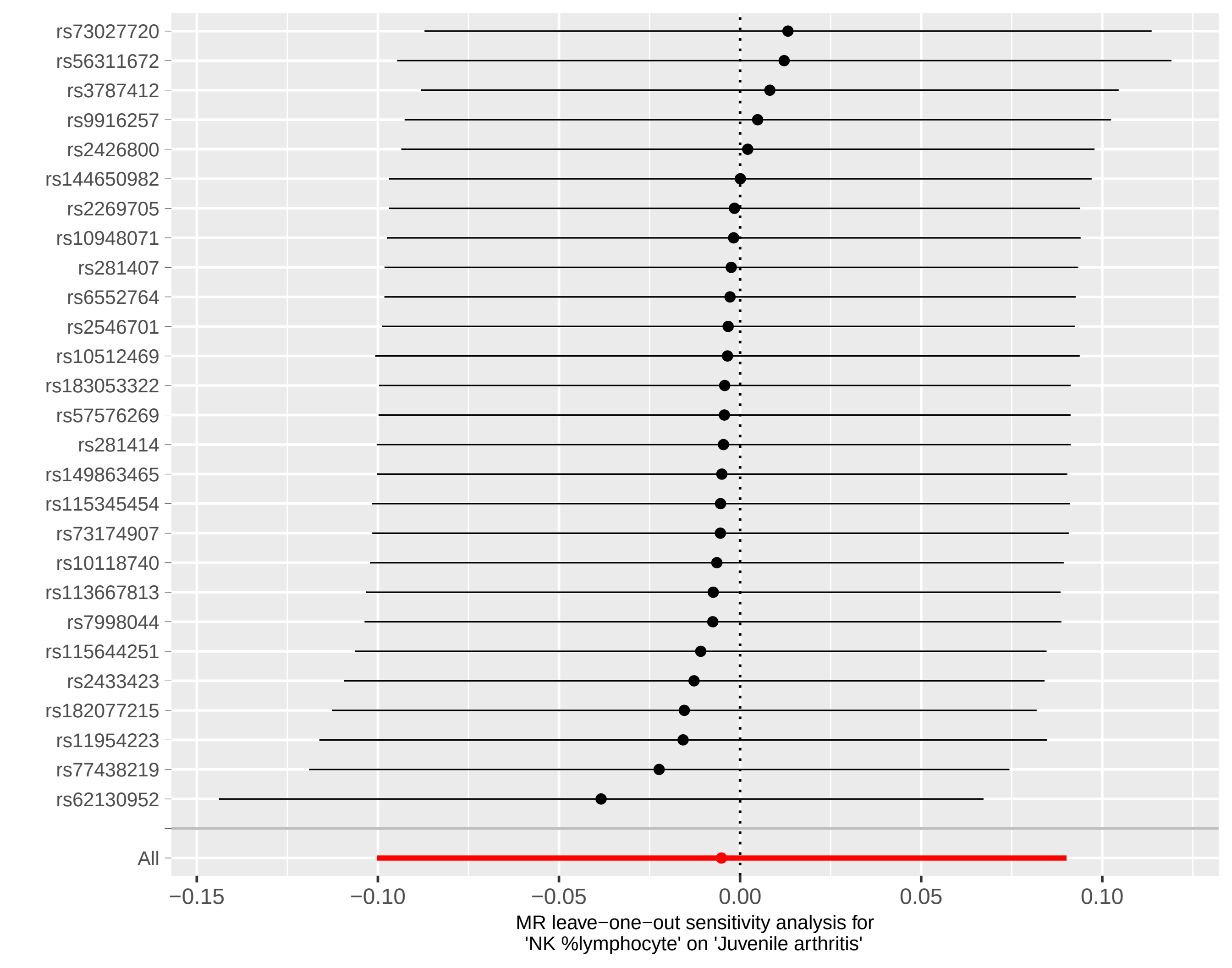
rs73027720
rs56311672
rs3787412
rs9916257
rs2426800
rs144650982
rs2269705
rs10948071
rs281407
rs6552764
rs2546701
rs10512469
rs183053322
rs57576269
rs281414
rs149863465
rs115345454
rs73174907
rs10118740
rs113667813
rs7998044
rs115644251
rs2433423
rs182077215
rs11954223
rs77438219
rs62130952
All
−0.15
−0.10
−0.05
0.00
0.05
0.10
MR leave−one−out sensitivity analysis for
'NK %lymphocyte' on 'Juvenile arthritis'

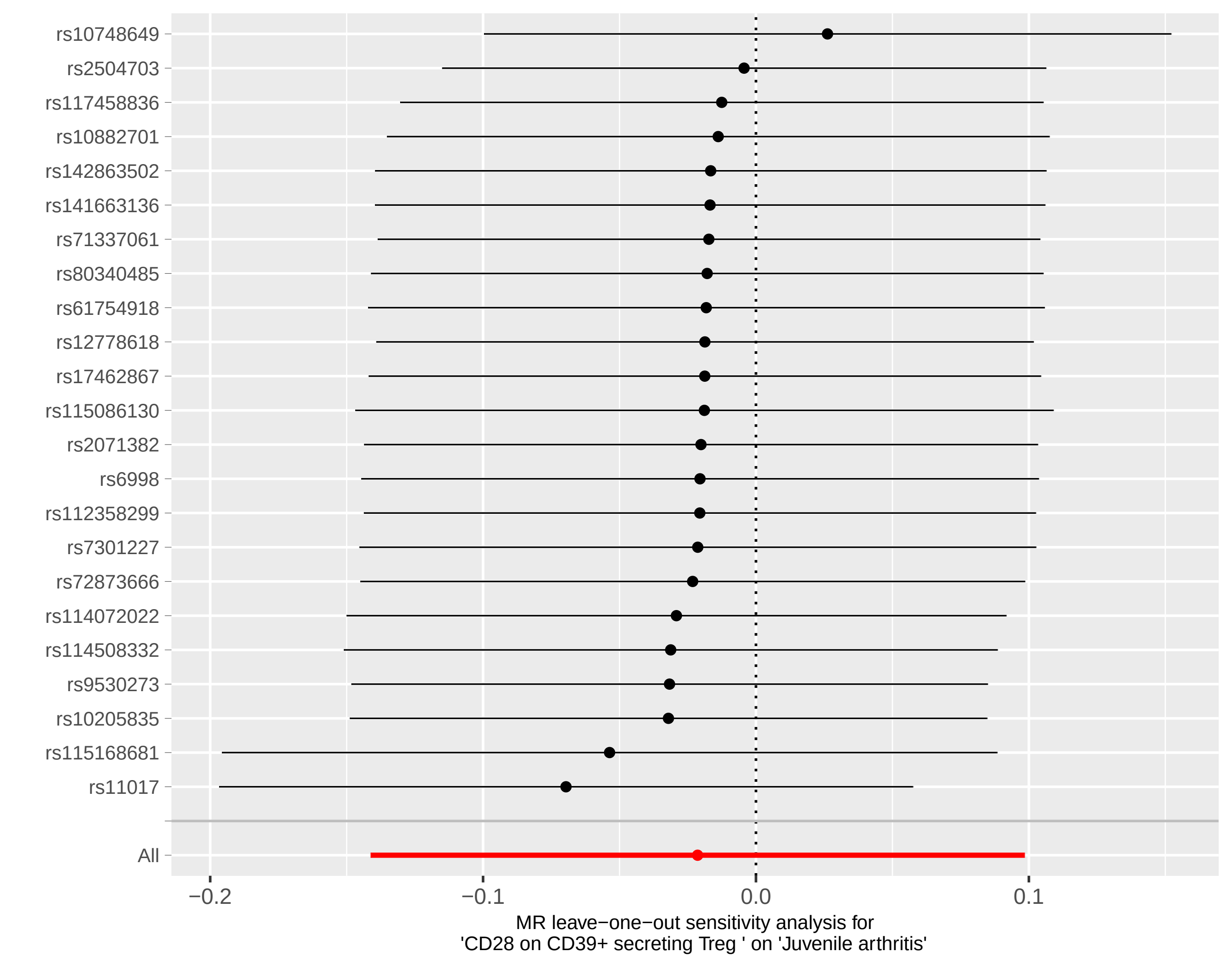
rs10748649
rs2504703
rs117458836
rs10882701
rs142863502
rs141663136
rs71337061
rs80340485
rs61754918
rs12778618
rs17462867
rs115086130
rs2071382
rs6998
rs112358299
rs7301227
rs72873666
rs114072022
rs114508332
rs9530273
rs10205835
rs115168681
rs11017
All
−0.2
−0.1
0.0
0.1
MR leave−one−out sensitivity analysis for
'CD28 on CD39+ secreting Treg ' on 'Juvenile arthritis'

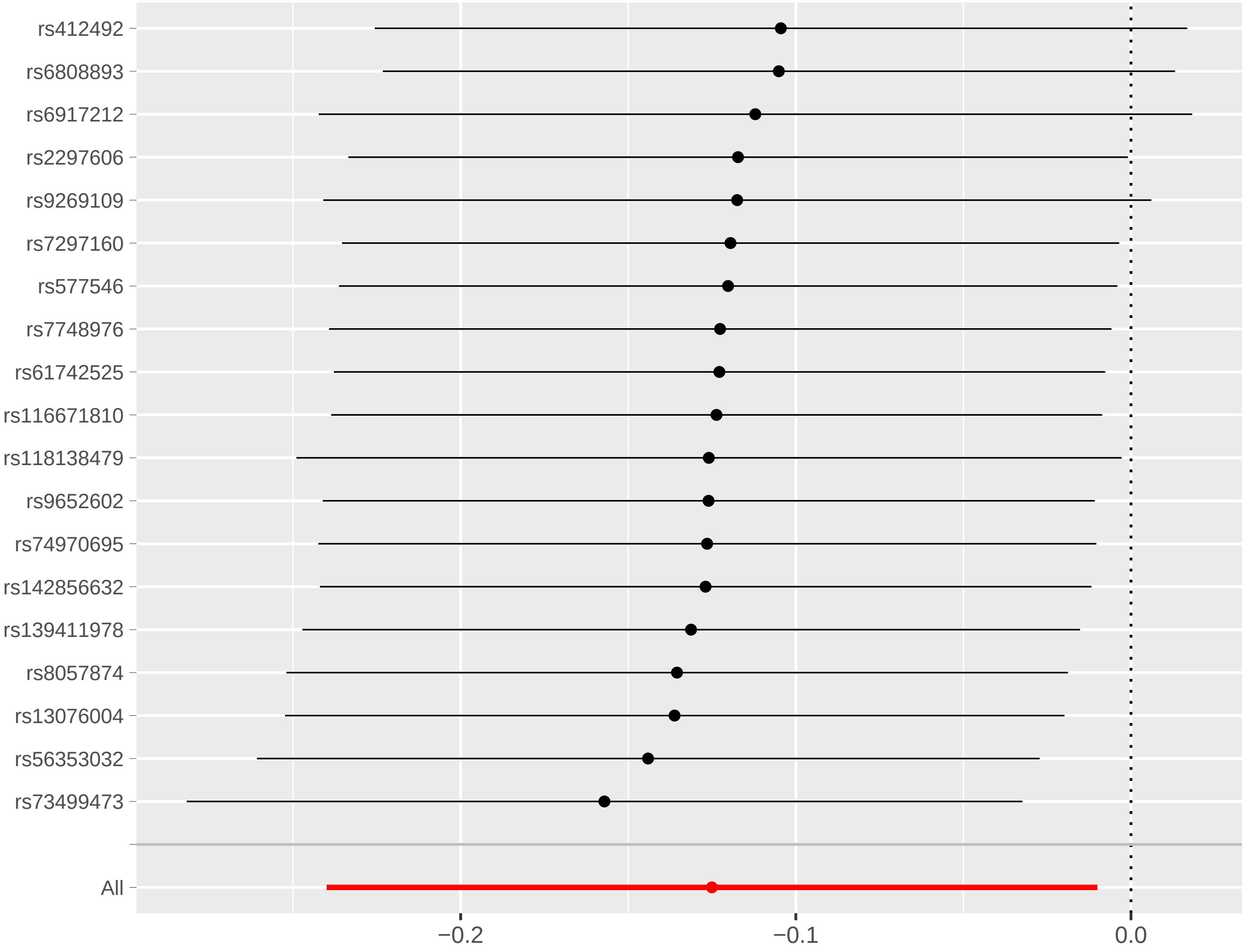

MR leave−one−out sensitivity analysis for
'HLA DR on CD14− CD16+ monocyte' on 'Juvenile arthritis'

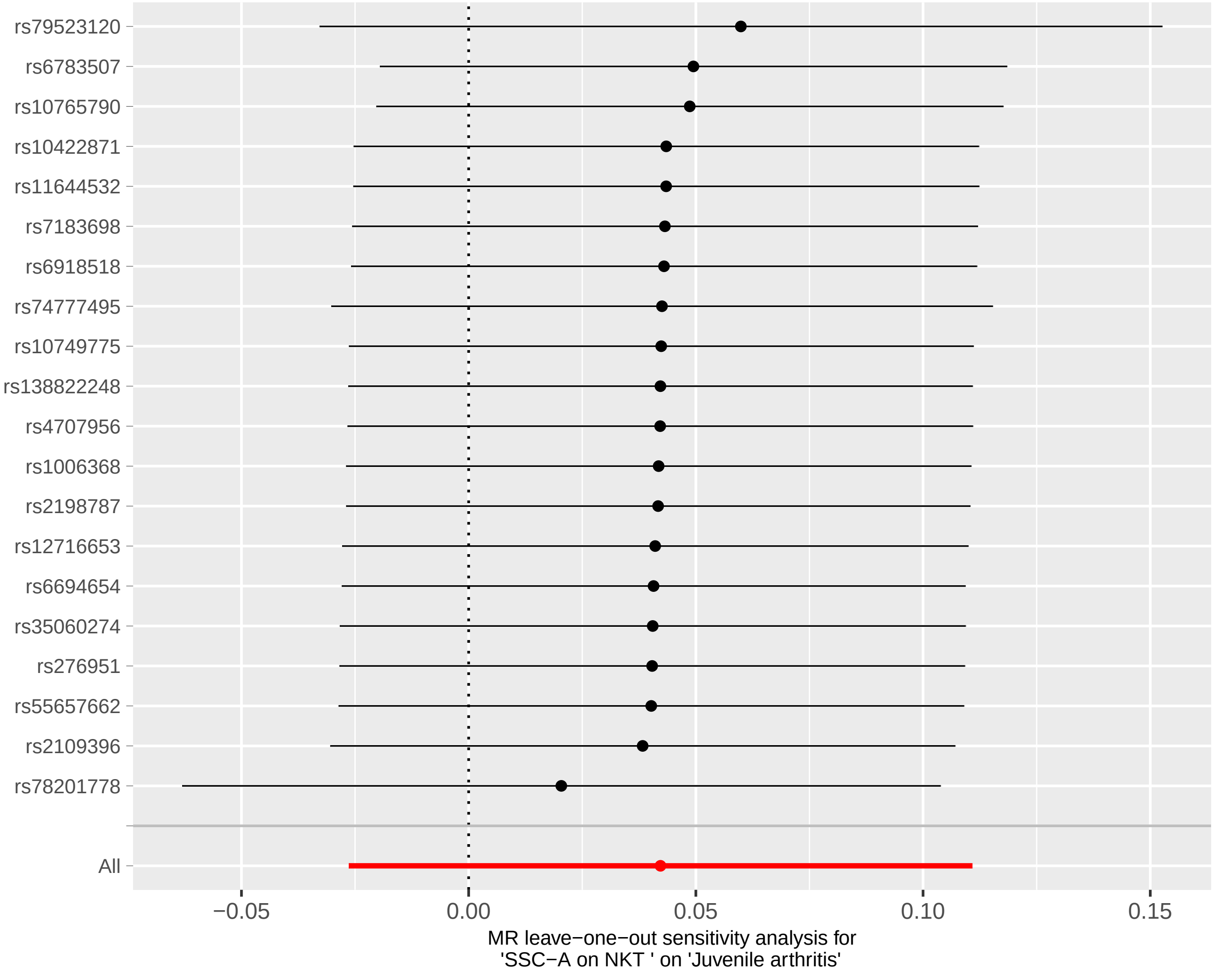
rs79523120
rs6783507
rs10765790
rs10422871
rs11644532
rs7183698
rs6918518
rs74777495
rs10749775
rs138822248
rs4707956
rs1006368
rs2198787
rs12716653
rs6694654
rs35060274
rs276951
rs55657662
rs2109396
rs78201778
All
−0.05
0.00
0.05
0.10
0.15
MR leave−one−out sensitivity analysis for
'SSC−A on NKT ' on 'Juvenile arthritis'

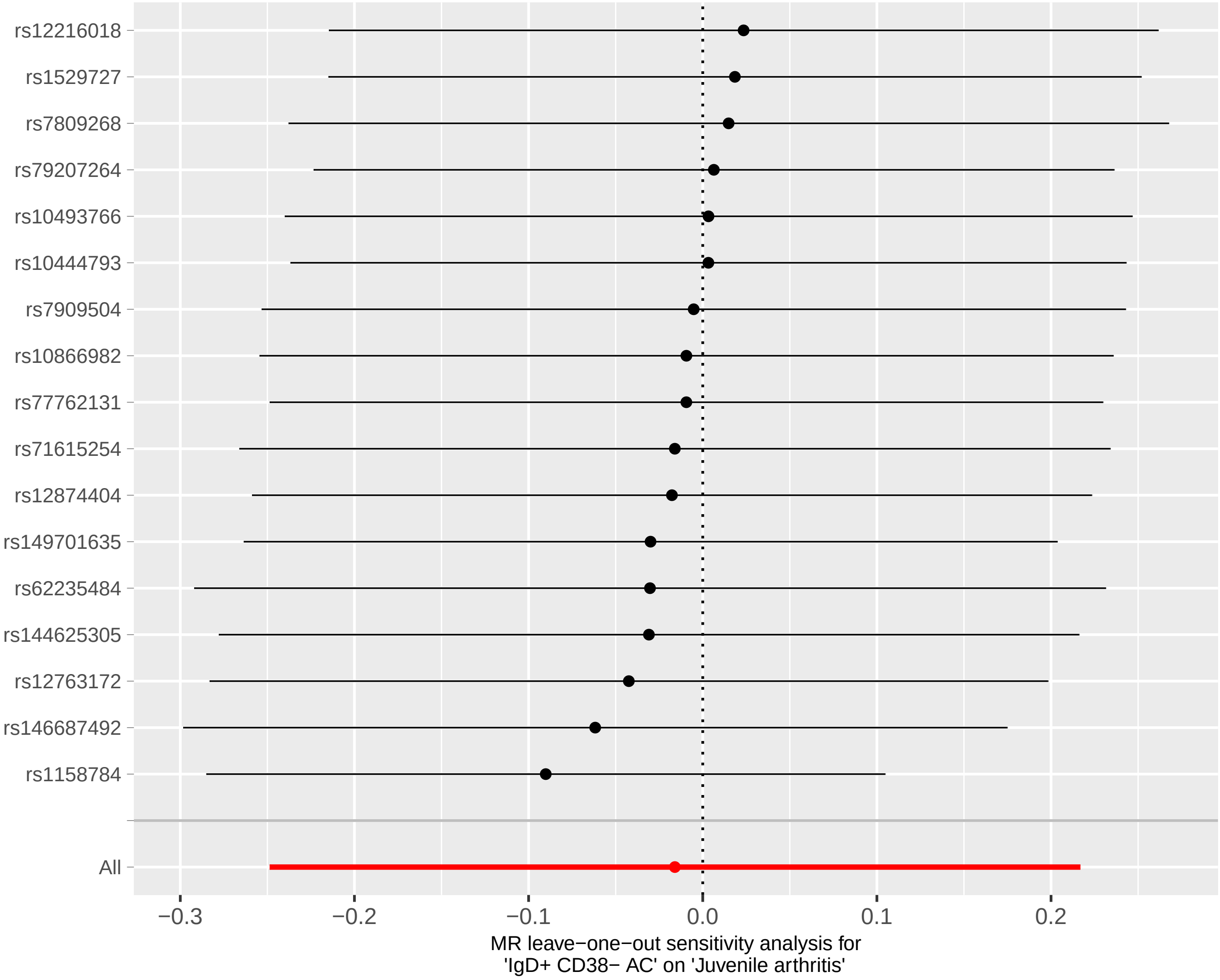
rs12216018
rs1529727
rs7809268
rs79207264
rs10493766
rs10444793
rs7909504
rs10866982
rs77762131
rs71615254
rs12874404
rs149701635
rs62235484
rs144625305
rs12763172
rs146687492
rs1158784
All
−0.3
−0.2
−0.1
0.0
0.1
0.2
MR leave−one−out sensitivity analysis for
'IgD+ CD38− AC' on 'Juvenile arthritis'

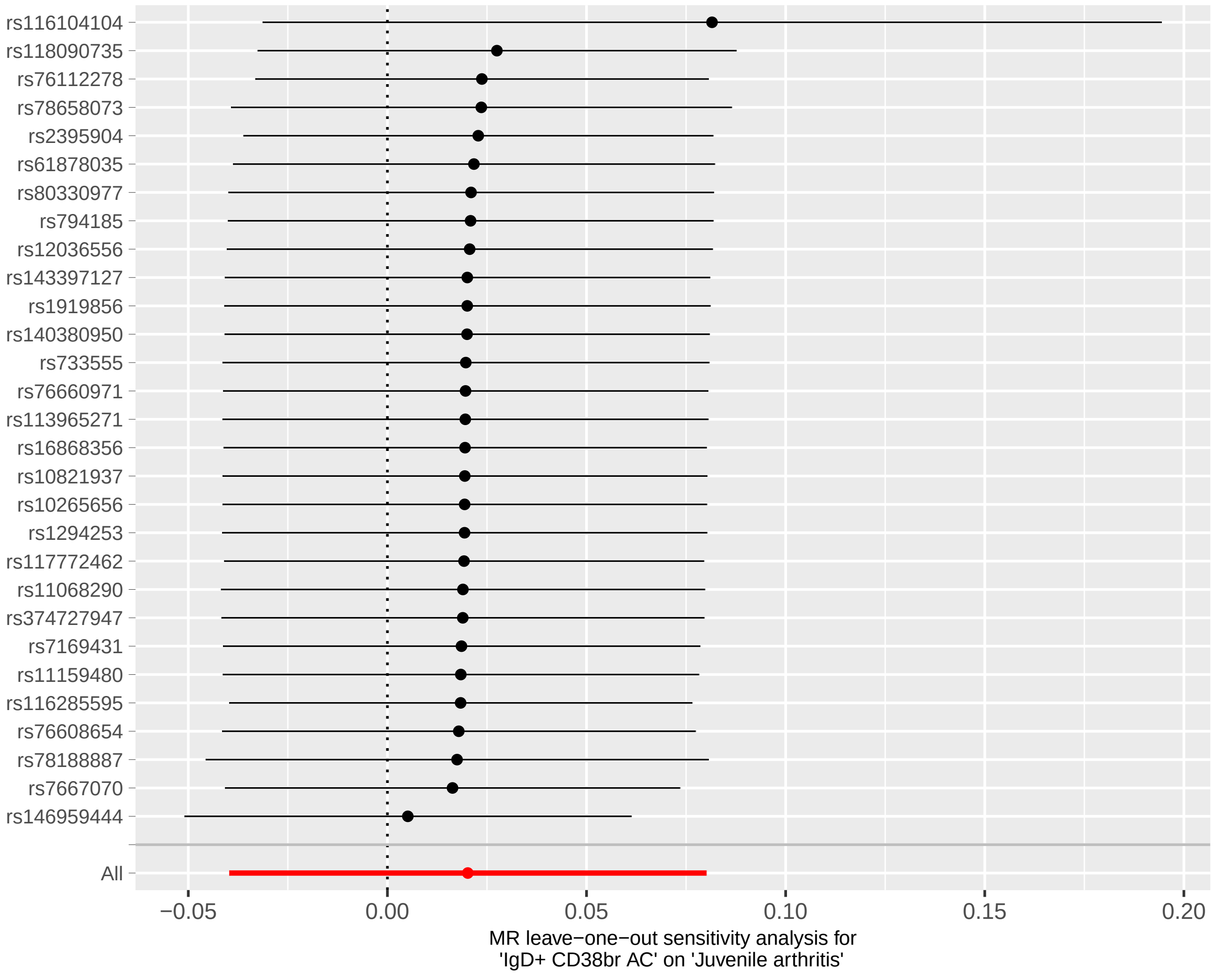

rs116104104
rs118090735
rs76112278
rs78658073
rs2395904
rs61878035
rs80330977
rs794185
rs12036556
rs143397127
rs1919856
rs140380950
rs733555
rs76660971
rs113965271
rs16868356
rs10821937
rs10265656
rs1294253
rs117772462
rs11068290
rs374727947
rs7169431
rs11159480
rs116285595
rs76608654
rs78188887
rs7667070
rs146959444
All
−0.05
0.00
0.05
0.10
0.15
0.20
MR leave−one−out sensitivity analysis for
'IgD+ CD38br AC' on 'Juvenile arthritis'

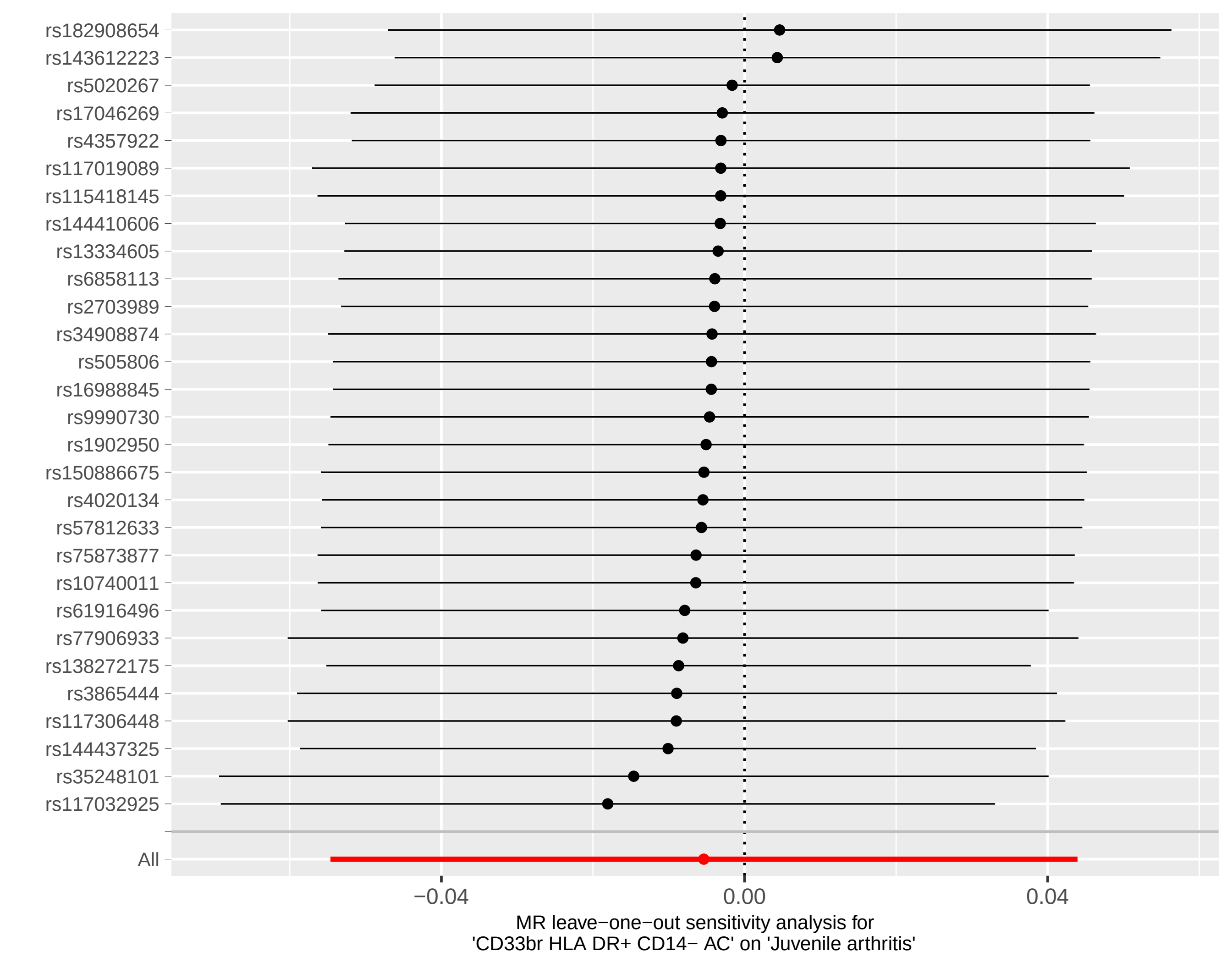

rs182908654
rs143612223
rs5020267
rs17046269
rs4357922
rs117019089
rs115418145
rs144410606
rs13334605
rs6858113
rs2703989
rs34908874
rs505806
rs16988845
rs9990730
rs1902950
rs150886675
rs4020134
rs57812633
rs75873877
rs10740011
rs61916496
rs77906933
rs138272175
rs3865444
rs117306448
rs144437325
rs35248101
rs117032925
All
−0.04
0.00
0.04
MR leave−one−out sensitivity analysis for
'CD33br HLA DR+ CD14− AC' on 'Juvenile arthritis'

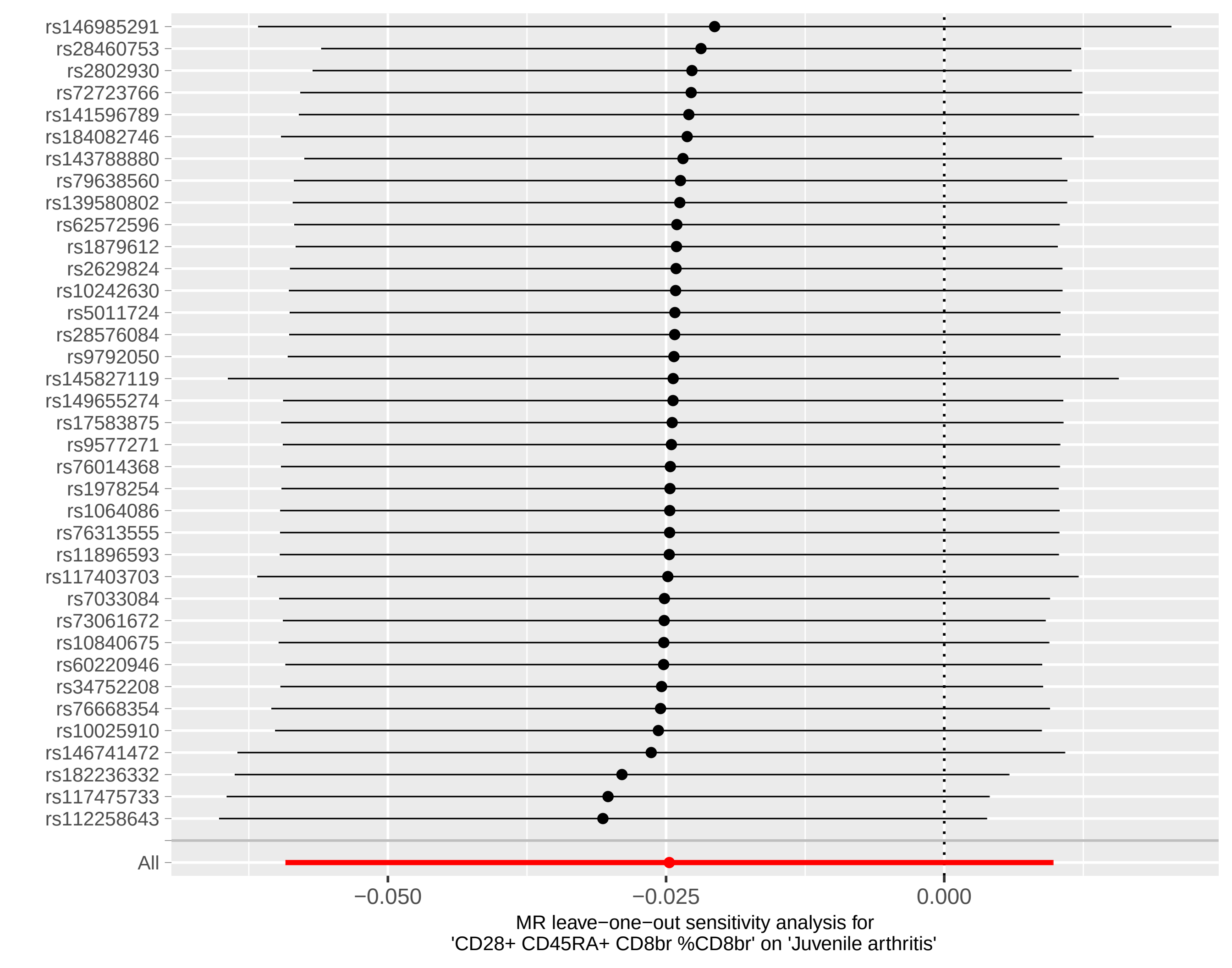

rs146985291
rs28460753
rs2802930
rs72723766
rs141596789
rs184082746
rs143788880
rs79638560
rs139580802
rs62572596
rs1879612
rs2629824
rs10242630
rs5011724
rs28576084
rs9792050
rs145827119
rs149655274
rs17583875
rs9577271
rs76014368
rs1978254
rs1064086
rs76313555
rs11896593
rs117403703
rs7033084
rs73061672
rs10840675
rs60220946
rs34752208
rs76668354
rs10025910
rs146741472
rs182236332
rs117475733
rs112258643
All
−0.050
−0.025
0.000
MR leave−one−out sensitivity analysis for
'CD28+ CD45RA+ CD8br %CD8br' on 'Juvenile arthritis'

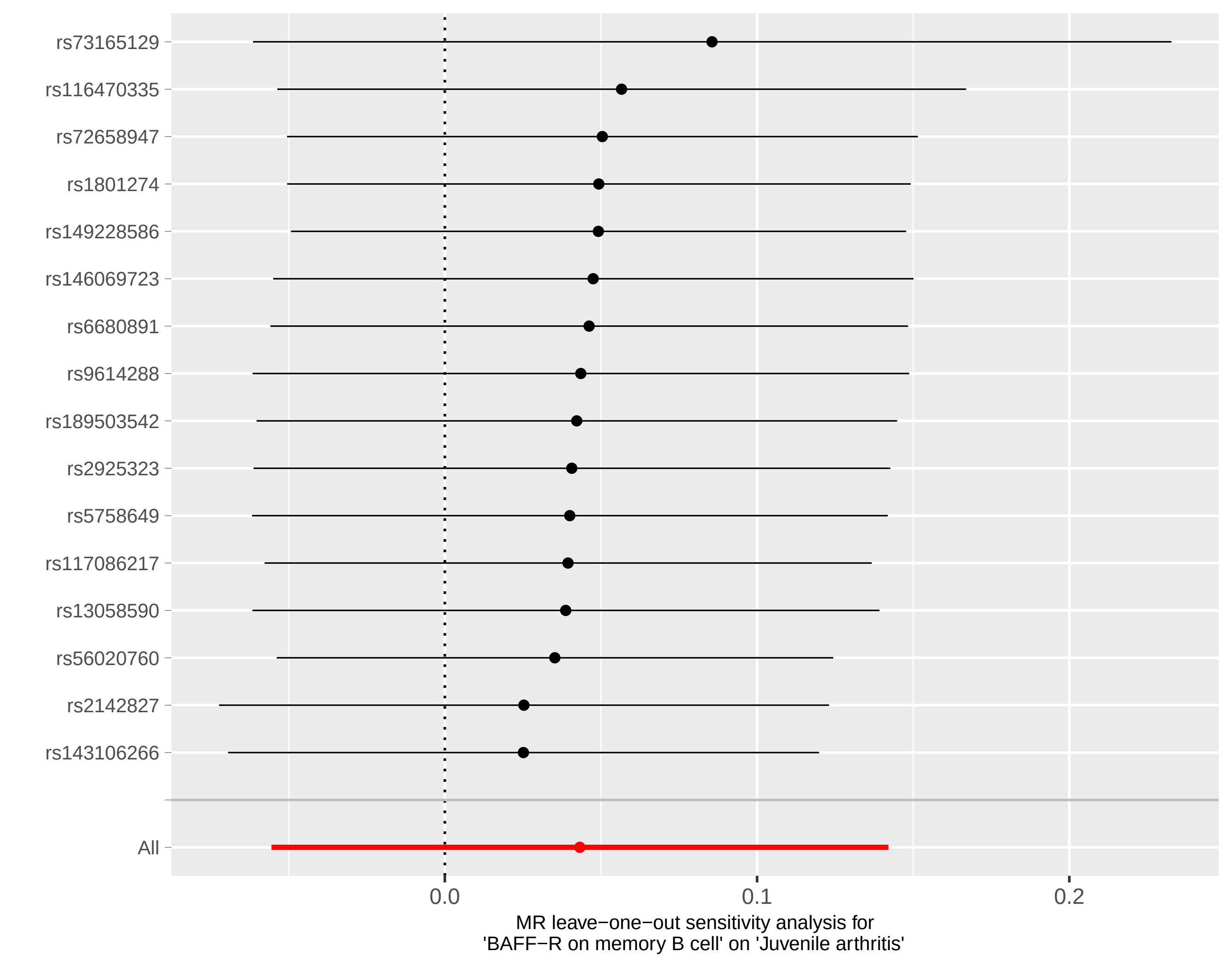
rs73165129
rs116470335
rs72658947
rs1801274
rs149228586
rs146069723
rs6680891
rs9614288
rs189503542
rs2925323
rs5758649
rs117086217
rs13058590
rs56020760
rs2142827
rs143106266
All
0.0
0.1
0.2
MR leave−one−out sensitivity analysis for
'BAFF−R on memory B cell' on 'Juvenile arthritis'

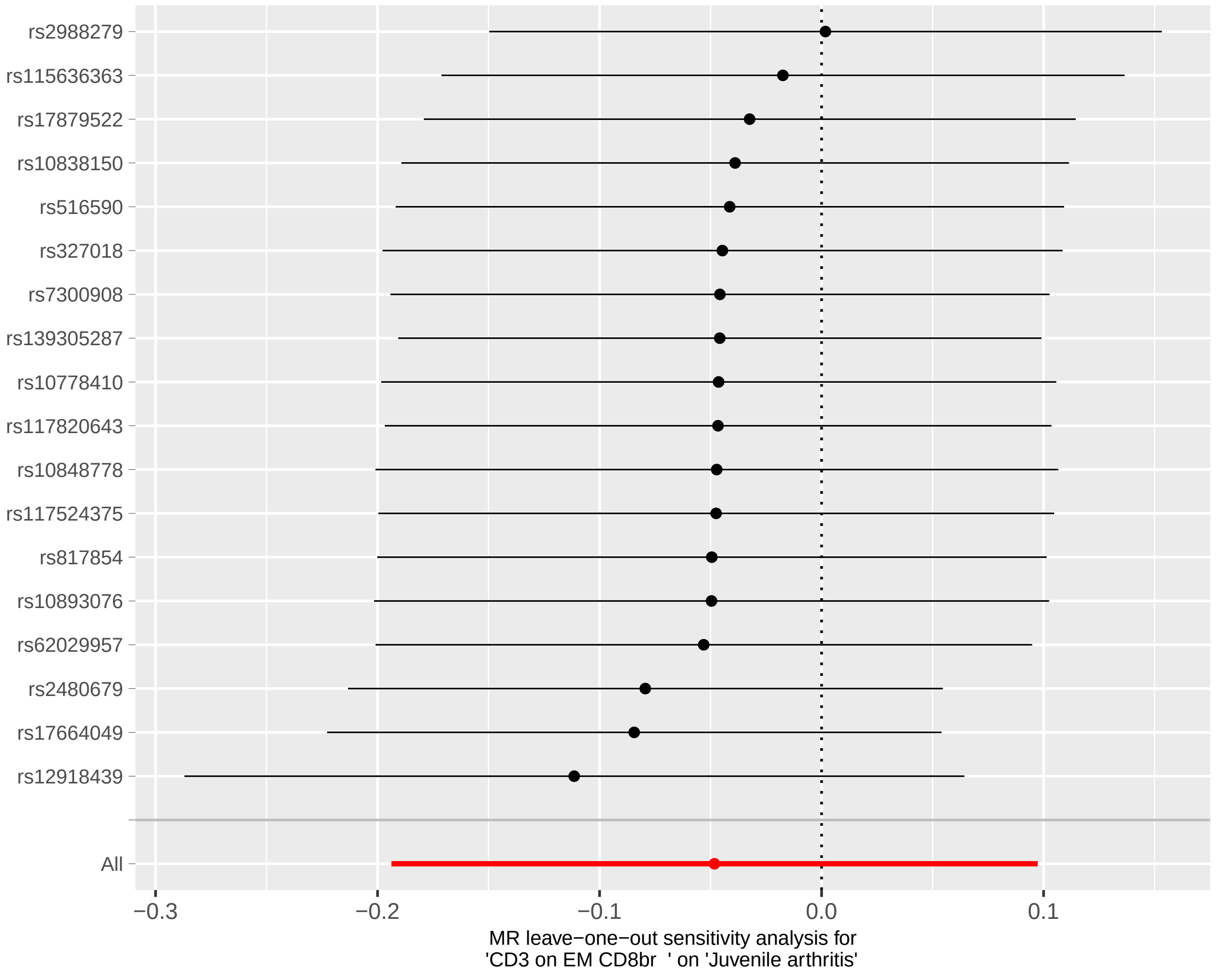
rs2988279
rs115636363
rs17879522
rs10838150
rs516590
rs327018
rs7300908
rs139305287
rs10778410
rs117820643
rs10848778
rs117524375
rs817854
rs10893076
rs62029957
rs2480679
rs17664049
rs12918439
All
−0.3
−0.2
−0.1
0.0
0.1
MR leave−one−out sensitivity analysis for
'CD3 on EM CD8br ' on 'Juvenile arthritis'

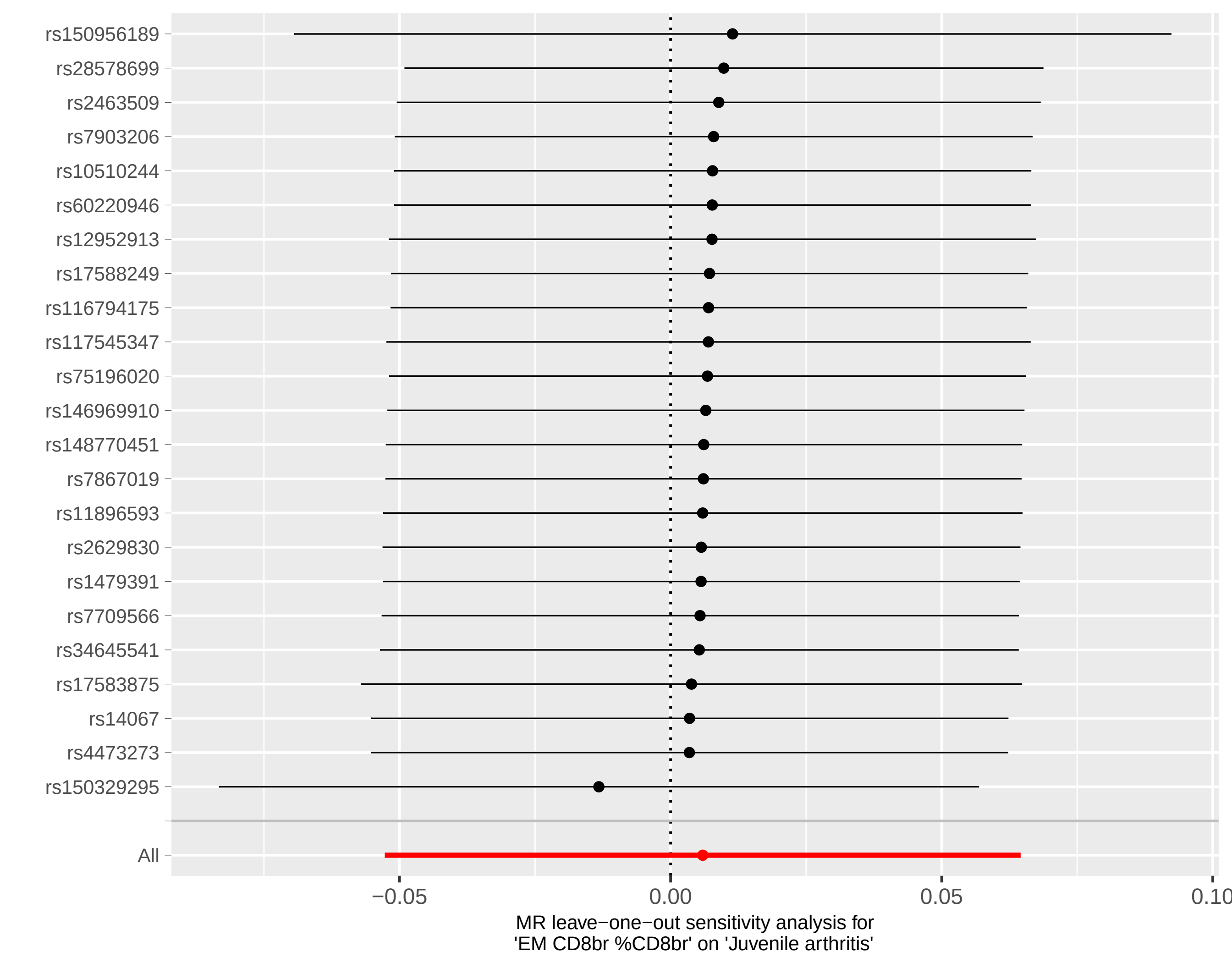
rs150956189
rs28578699
rs2463509
rs7903206
rs10510244
rs60220946
rs12952913
rs17588249
rs116794175
rs117545347
rs75196020
rs146969910
rs148770451
rs7867019
rs11896593
rs2629830
rs1479391
rs7709566
rs34645541
rs17583875
rs14067
rs4473273
rs150329295
All
−0.05
0.00
0.05
0.10
MR leave−one−out sensitivity analysis for
'EM CD8br %CD8br' on 'Juvenile arthritis'

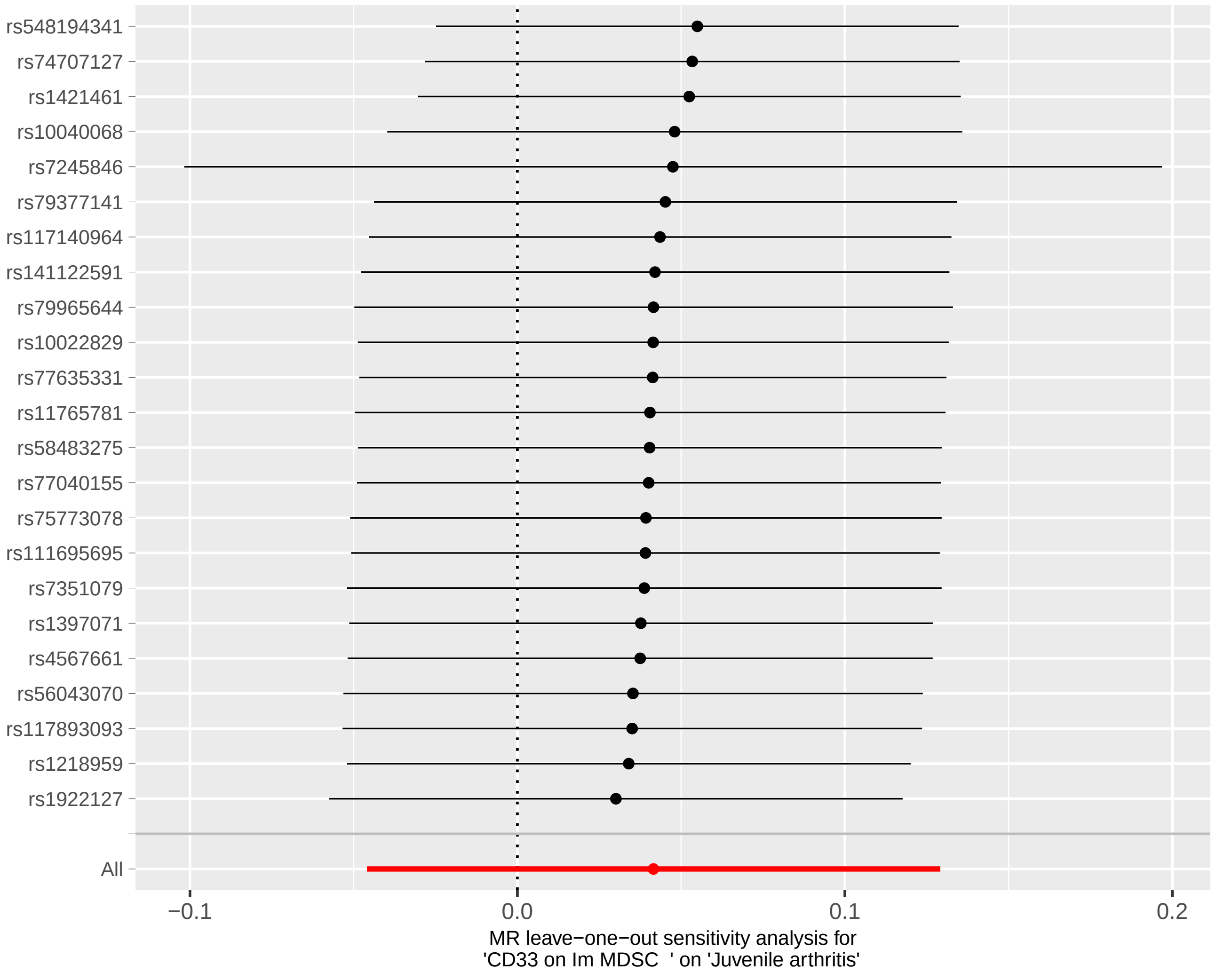
rs548194341
rs74707127
rs1421461
rs10040068
rs7245846
rs79377141
rs117140964
rs141122591
rs79965644
rs10022829
rs77635331
rs11765781
rs58483275
rs77040155
rs75773078
rs111695695
rs7351079
rs1397071
rs4567661
rs56043070
rs117893093
rs1218959
rs1922127
All
−0.1
0.0
0.1
0.2
MR leave−one−out sensitivity analysis for
'CD33 on Im MDSC ' on 'Juvenile arthritis'

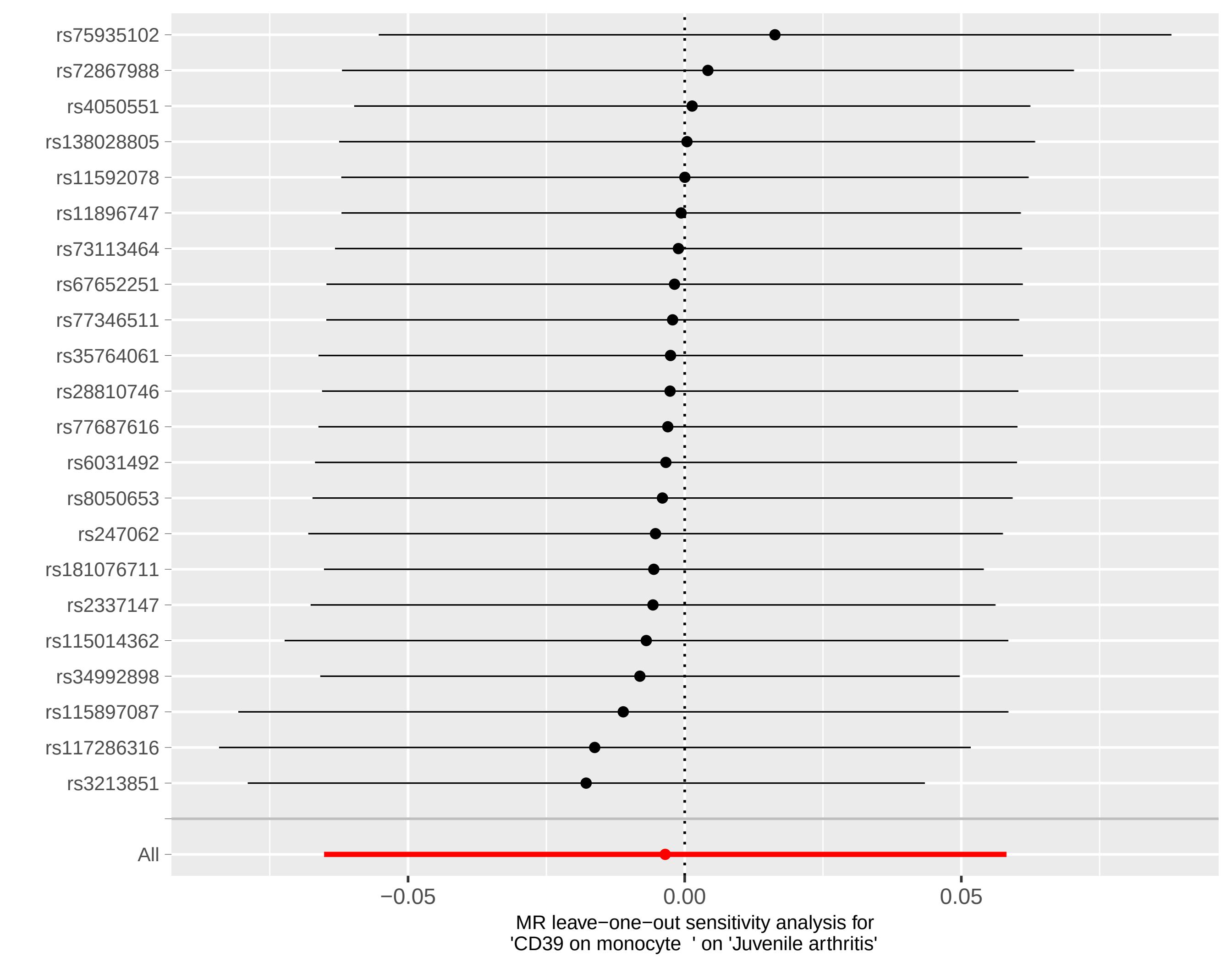

rs75935102
rs72867988
rs4050551
rs138028805
rs11592078
rs11896747
rs73113464
rs67652251
rs77346511
rs35764061
rs28810746
rs77687616
rs6031492
rs8050653
rs247062
rs181076711
rs2337147
rs115014362
rs34992898
rs115897087
rs117286316
rs3213851
All
−0.05
0.00
0.05
MR leave−one−out sensitivity analysis for
'CD39 on monocyte ' on 'Juvenile arthritis'

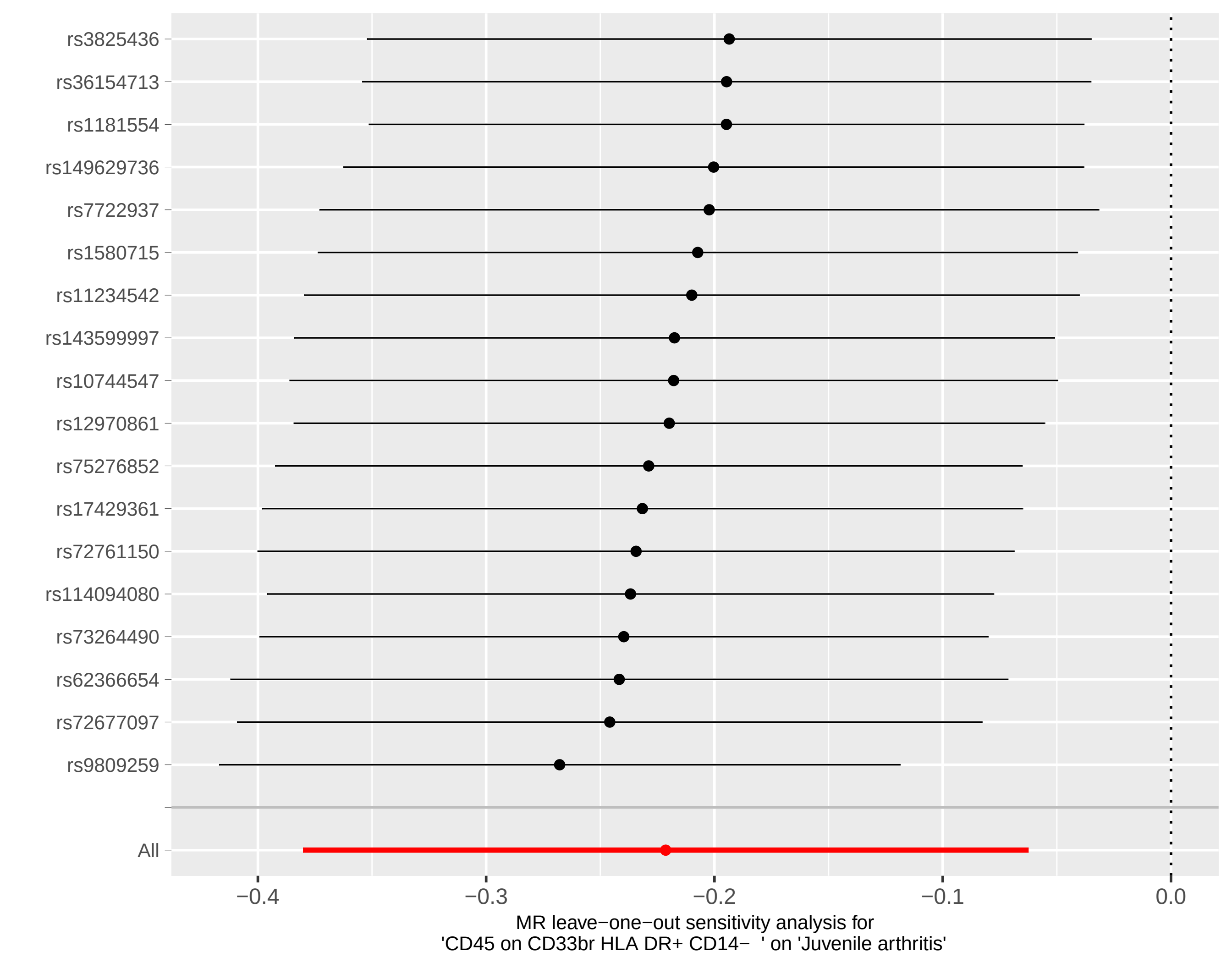
rs3825436
rs36154713
rs1181554
rs149629736
rs7722937
rs1580715
rs11234542
rs143599997
rs10744547
rs12970861
rs75276852
rs17429361
rs72761150
rs114094080
rs73264490
rs62366654
rs72677097
rs9809259
All
−0.4
−0.3
−0.2
−0.1
0.0
MR leave−one−out sensitivity analysis for
'CD45 on CD33br HLA DR+ CD14− ' on 'Juvenile arthritis'

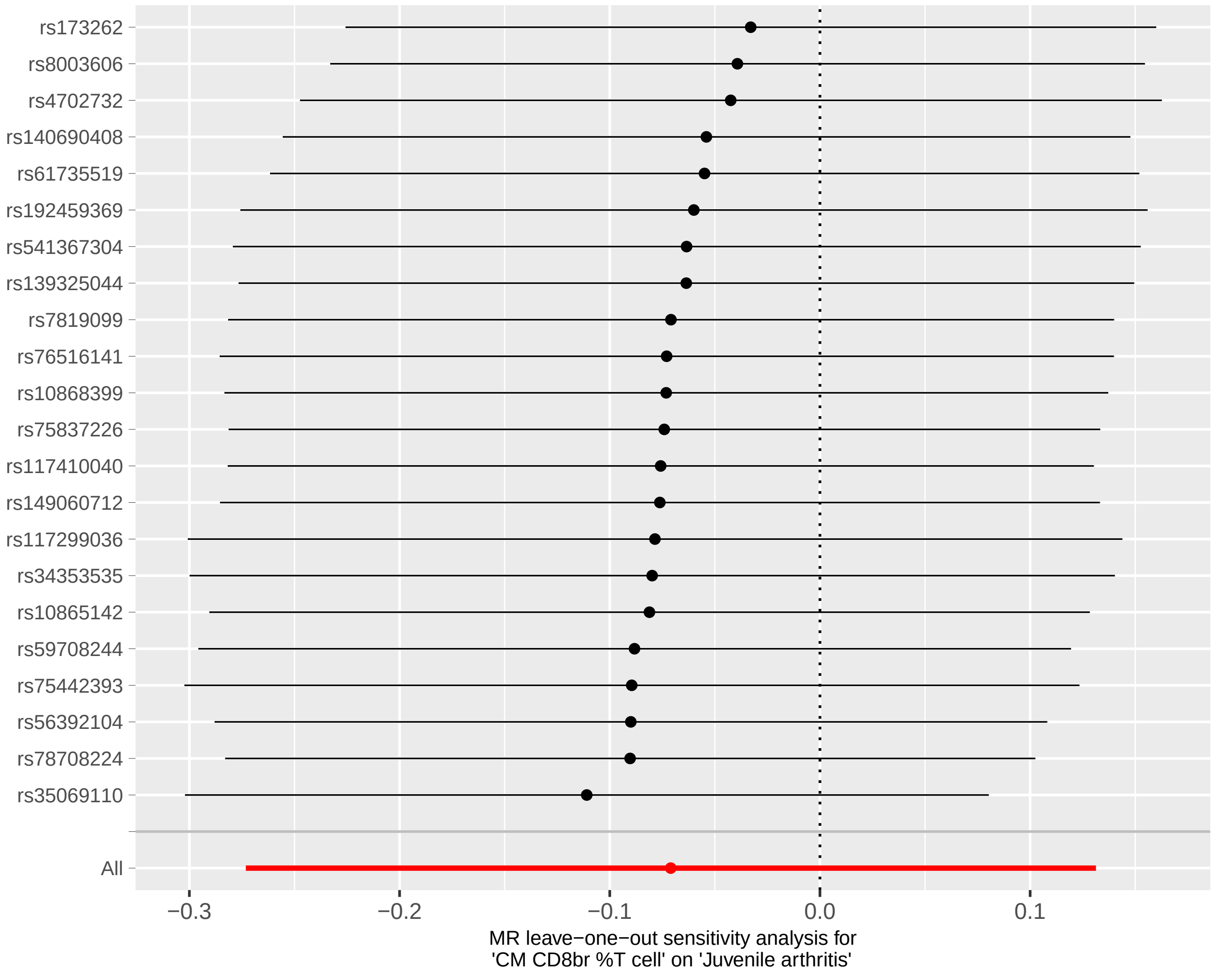

rs173262
rs8003606
rs4702732
rs140690408
rs61735519
rs192459369
rs541367304
rs139325044
rs7819099
rs76516141
rs10868399
rs75837226
rs117410040
rs149060712
rs117299036
rs34353535
rs10865142
rs59708244
rs75442393
rs56392104
rs78708224
rs35069110
All
−0.3
−0.2
−0.1
0.0
0.1
MR leave−one−out sensitivity analysis for
'CM CD8br %T cell' on 'Juvenile arthritis'

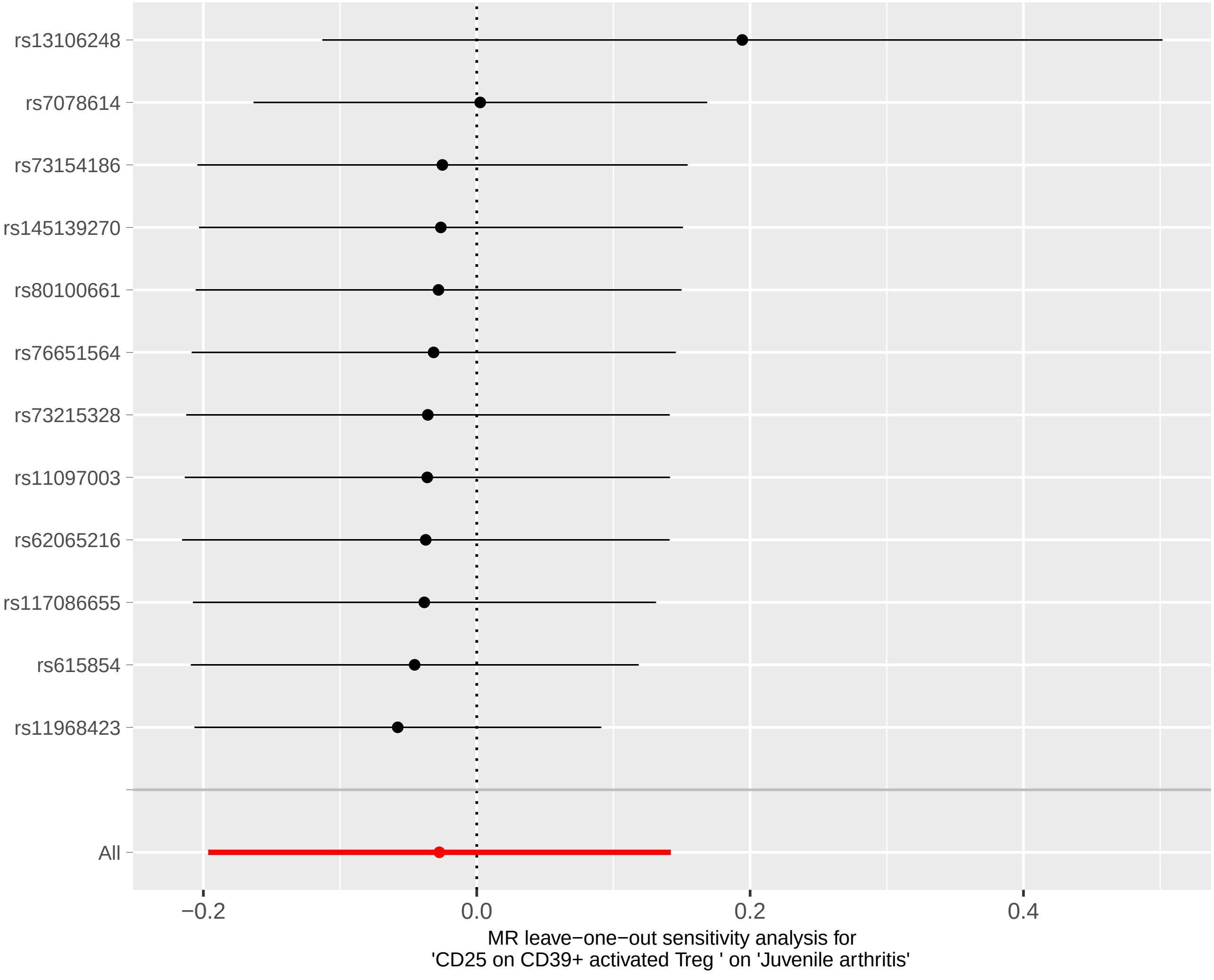

rs13106248
rs7078614
rs73154186
rs145139270
rs80100661
rs76651564
rs73215328
rs11097003
rs62065216
rs117086655
rs615854
rs11968423
All
−0.2
0.0
0.2
0.4
MR leave−one−out sensitivity analysis for
'CD25 on CD39+ activated Treg ' on 'Juvenile arthritis'

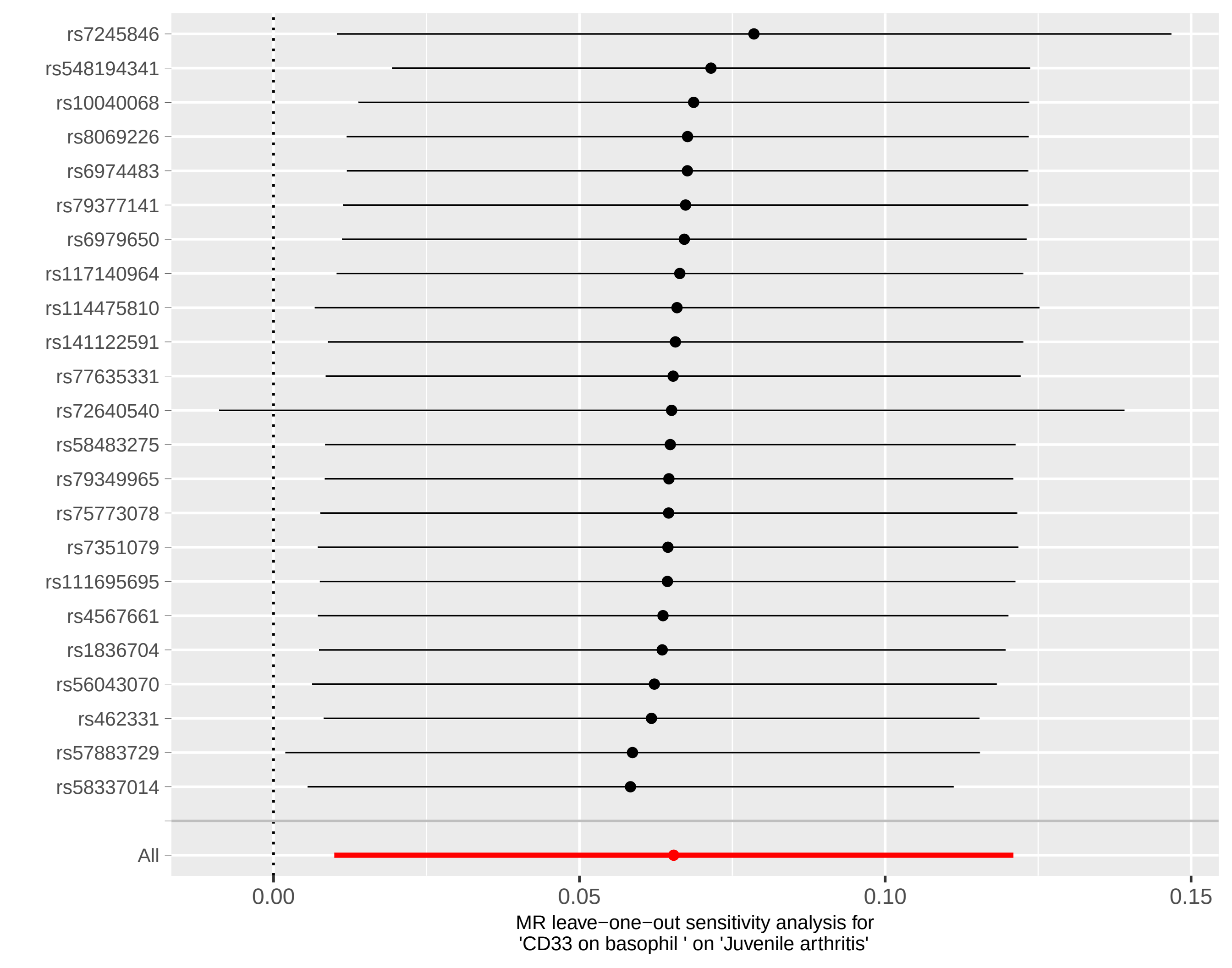
rs7245846
rs548194341
rs10040068
rs8069226
rs6974483
rs79377141
rs6979650
rs117140964
rs114475810
rs141122591
rs77635331
rs72640540
rs58483275
rs79349965
rs75773078
rs7351079
rs111695695
rs4567661
rs1836704
rs56043070
rs462331
rs57883729
rs58337014
All
0.00
0.05
0.10
0.15
MR leave−one−out sensitivity analysis for
'CD33 on basophil ' on 'Juvenile arthritis'

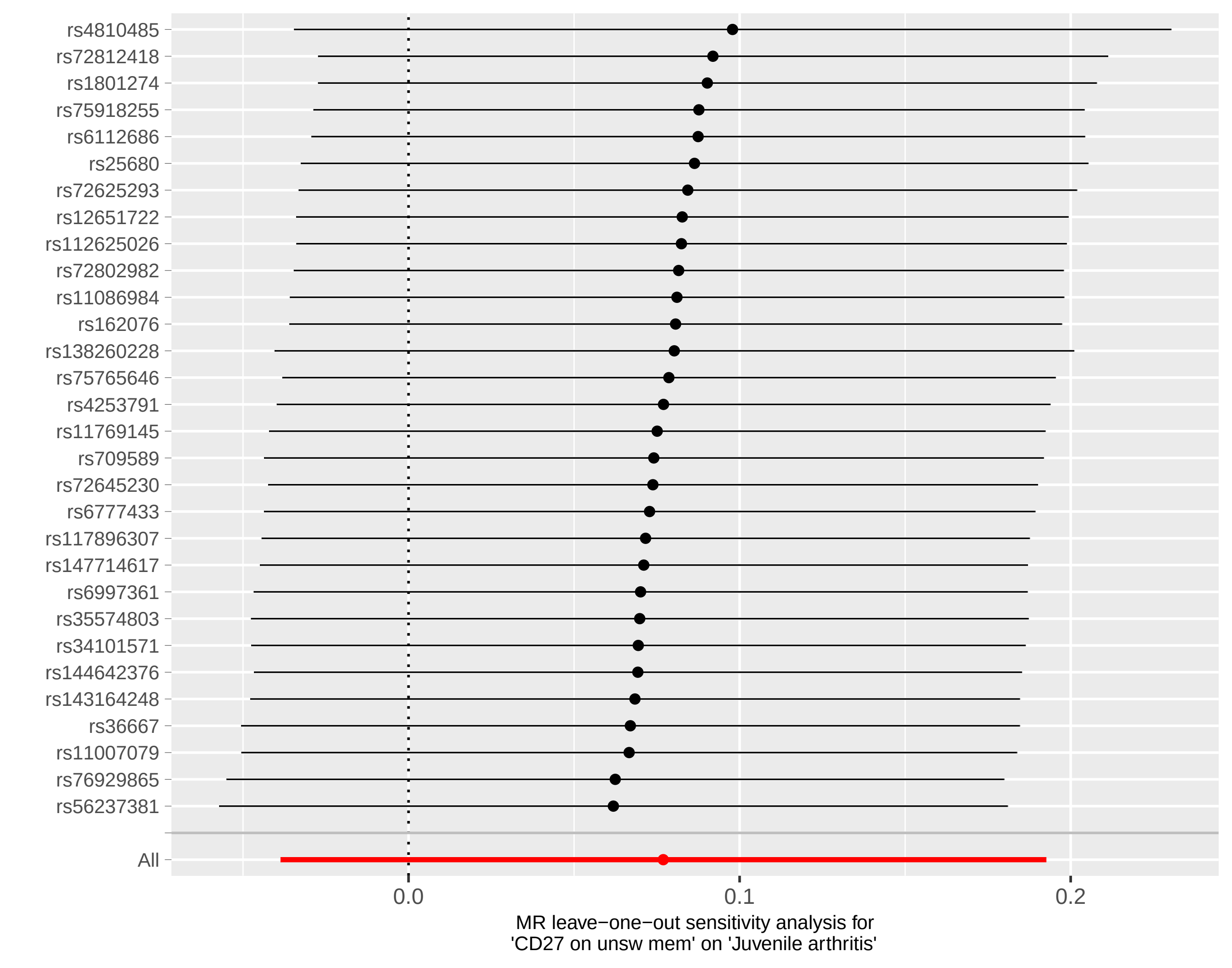
rs4810485
rs72812418
rs1801274
rs75918255
rs6112686
rs25680
rs72625293
rs12651722
rs112625026
rs72802982
rs11086984
rs162076
rs138260228
rs75765646
rs4253791
rs11769145
rs709589
rs72645230
rs6777433
rs117896307
rs147714617
rs6997361
rs35574803
rs34101571
rs144642376
rs143164248
rs36667
rs11007079
rs76929865
rs56237381
All
0.0
0.1
0.2
MR leave−one−out sensitivity analysis for
'CD27 on unsw mem' on 'Juvenile arthritis'

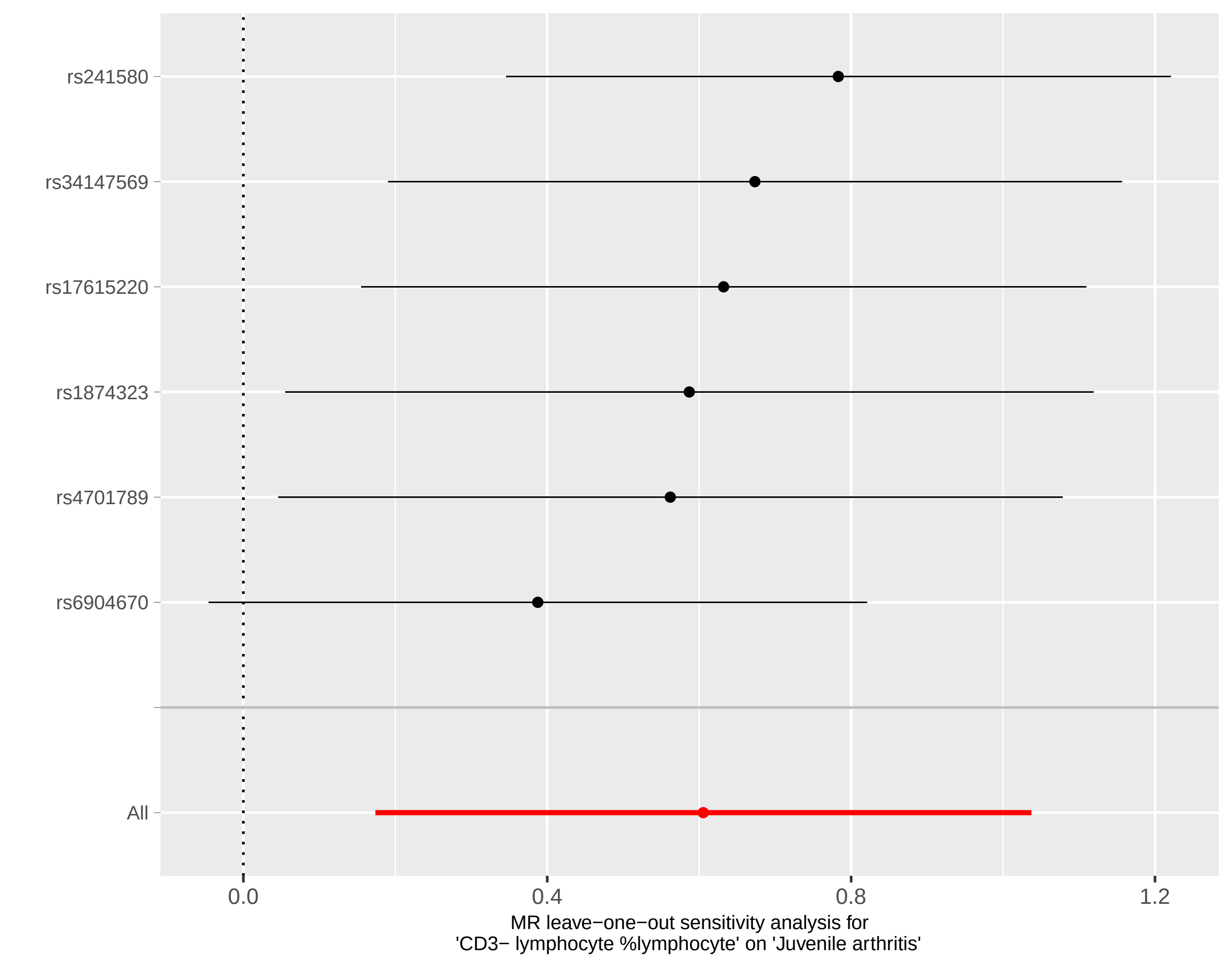

rs241580
rs34147569
rs17615220
rs1874323
rs4701789
rs6904670
All
0.0
0.4
0.8
1.2
MR leave−one−out sensitivity analysis for
'CD3− lymphocyte %lymphocyte' on 'Juvenile arthritis'

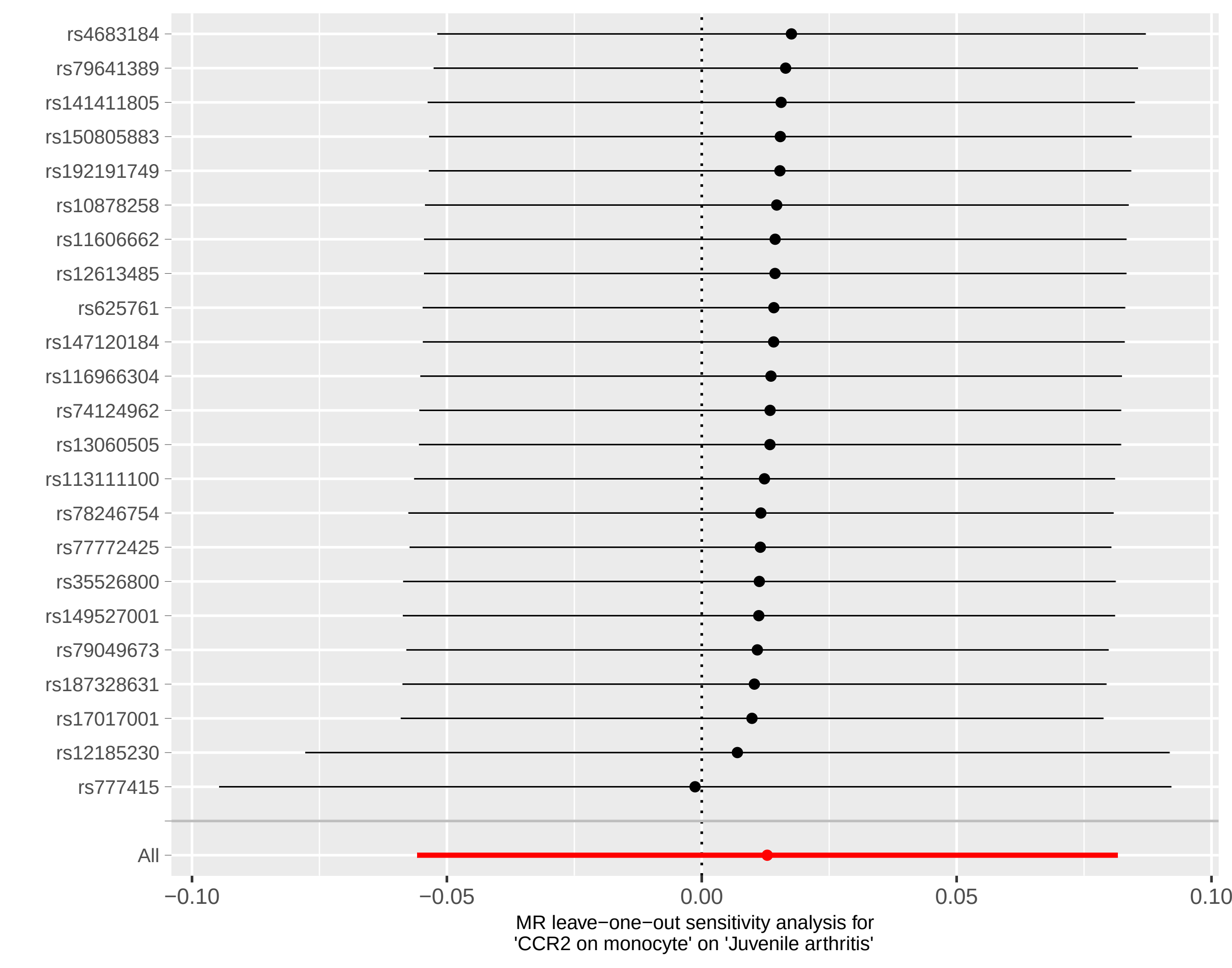
rs4683184
rs79641389
rs141411805
rs150805883
rs192191749
rs10878258
rs11606662
rs12613485
rs625761
rs147120184
rs116966304
rs74124962
rs13060505
rs113111100
rs78246754
rs77772425
rs35526800
rs149527001
rs79049673
rs187328631
rs17017001
rs12185230
rs777415
All
−0.10
−0.05
0.00
0.05
0.10
MR leave−one−out sensitivity analysis for
'CCR2 on monocyte' on 'Juvenile arthritis'

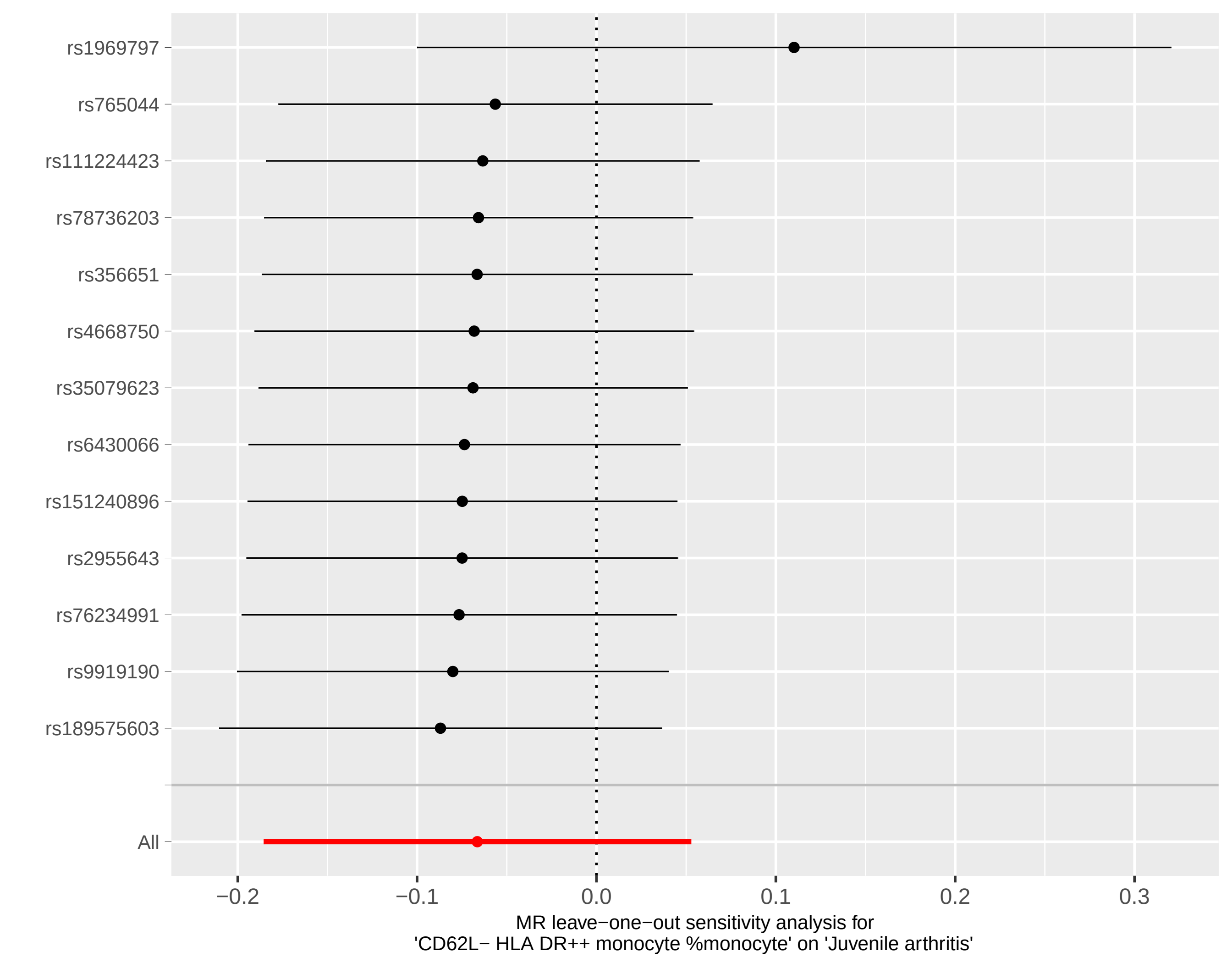
rs1969797
rs765044
rs111224423
rs78736203
rs356651
rs4668750
rs35079623
rs6430066
rs151240896
rs2955643
rs76234991
rs9919190
rs189575603
All
−0.2
−0.1
0.0
0.1
0.2
0.3
MR leave−one−out sensitivity analysis for
'CD62L− HLA DR++ monocyte %monocyte' on 'Juvenile arthritis'

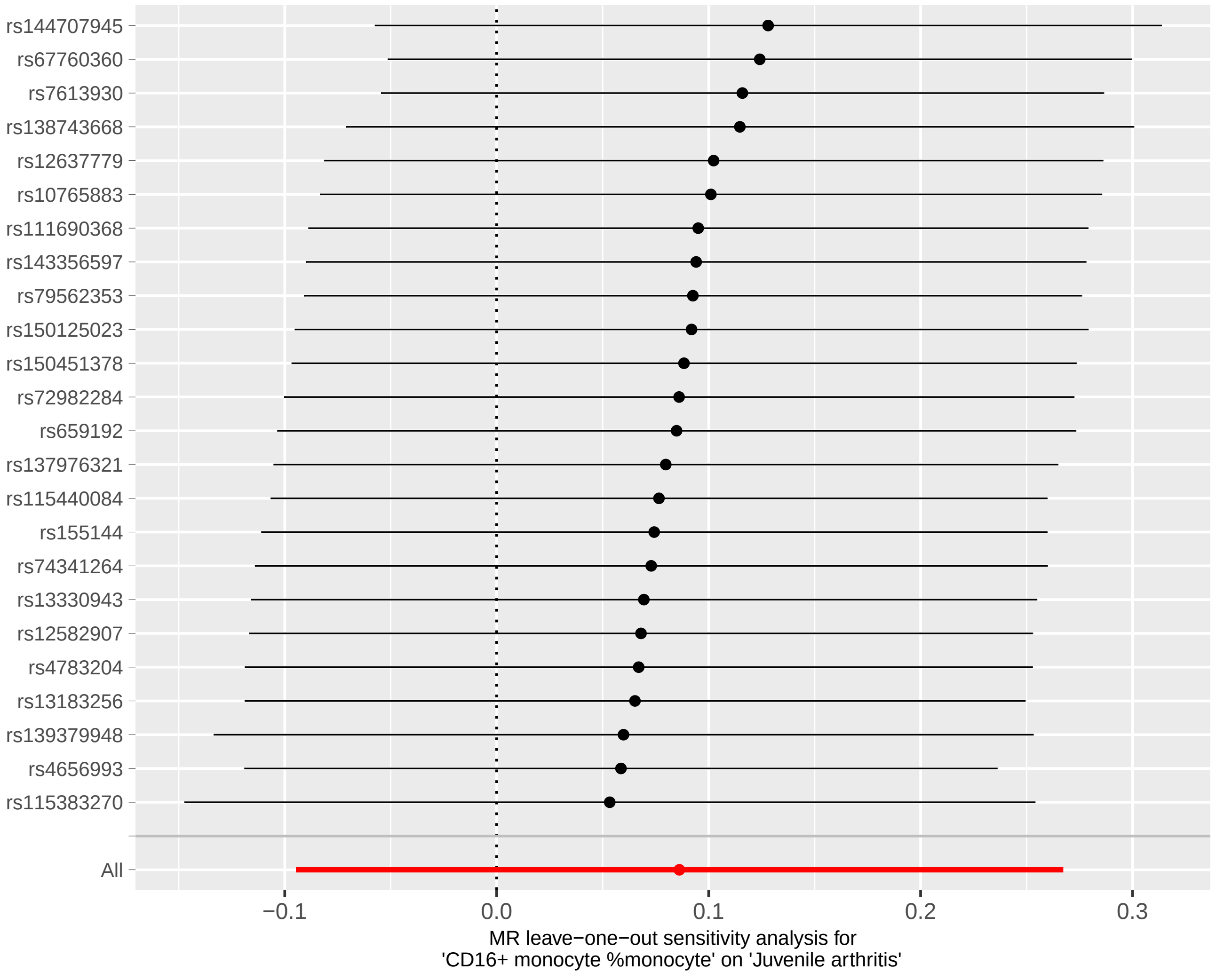
rs144707945
rs67760360
rs7613930
rs138743668
rs12637779
rs10765883
rs111690368
rs143356597
rs79562353
rs150125023
rs150451378
rs72982284
rs659192
rs137976321
rs115440084
rs155144
rs74341264
rs13330943
rs12582907
rs4783204
rs13183256
rs139379948
rs4656993
rs115383270
All
−0.1
0.0
0.1
0.2
0.3
MR leave−one−out sensitivity analysis for
'CD16+ monocyte %monocyte' on 'Juvenile arthritis'

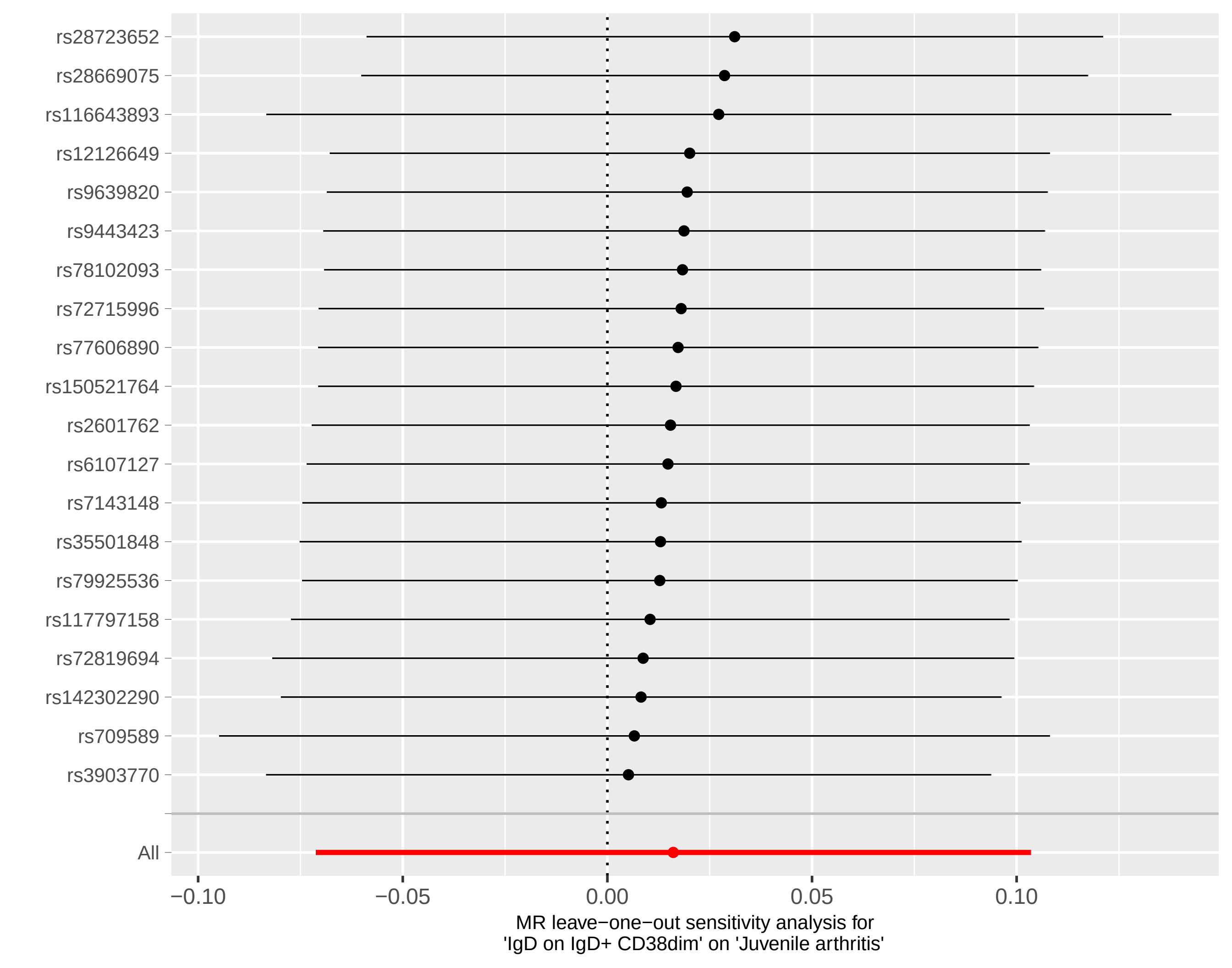
rs28723652
rs28669075
rs116643893
rs12126649
rs9639820
rs9443423
rs78102093
rs72715996
rs77606890
rs150521764
rs2601762
rs6107127
rs7143148
rs35501848
rs79925536
rs117797158
rs72819694
rs142302290
rs709589
rs3903770
All
−0.10
−0.05
0.00
0.05
0.10
MR leave−one−out sensitivity analysis for
'IgD on IgD+ CD38dim' on 'Juvenile arthritis'

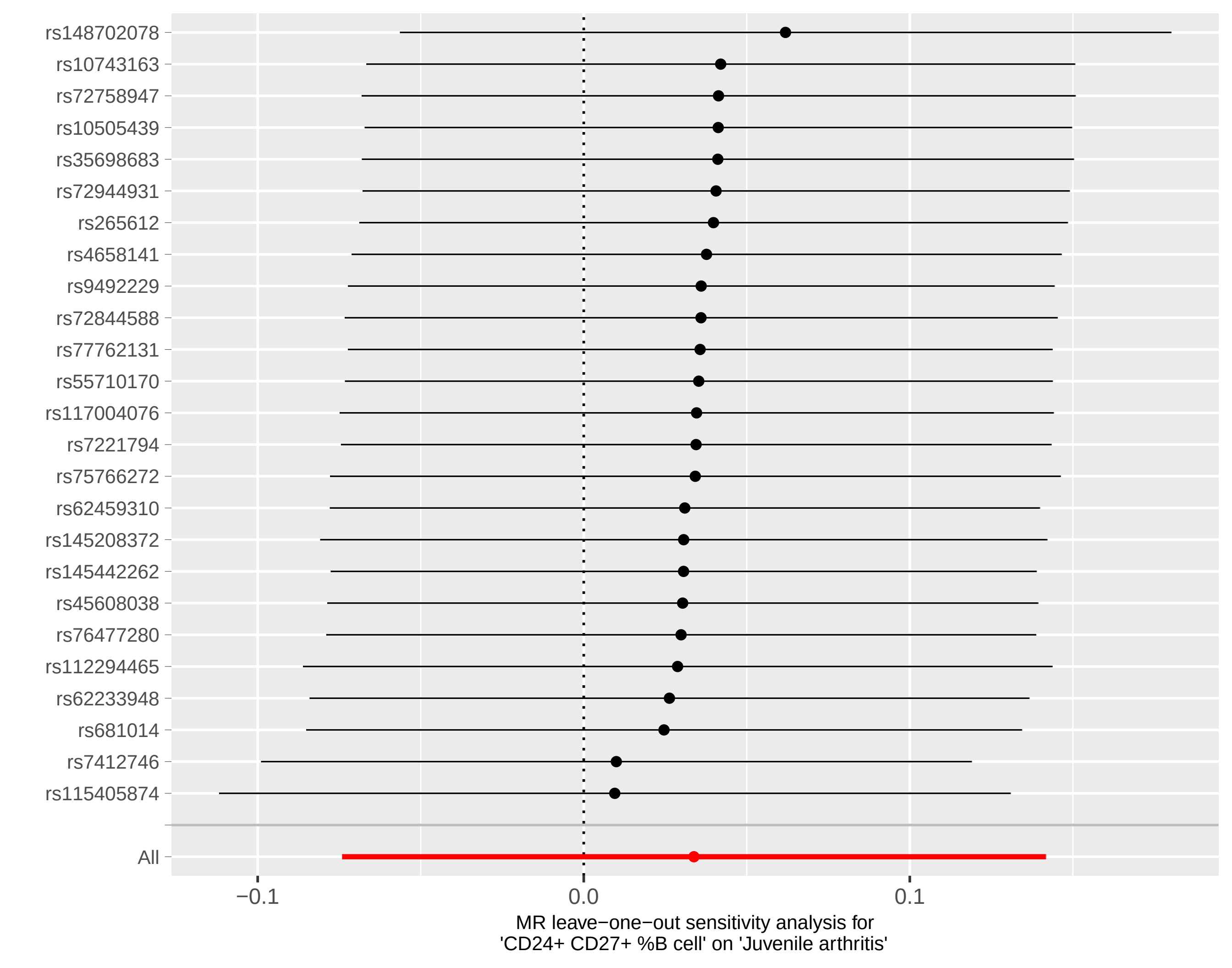
rs148702078
rs10743163
rs72758947
rs10505439
rs35698683
rs72944931
rs265612
rs4658141
rs9492229
rs72844588
rs77762131
rs55710170
rs117004076
rs7221794
rs75766272
rs62459310
rs145208372
rs145442262
rs45608038
rs76477280
rs112294465
rs62233948
rs681014
rs7412746
rs115405874
All
−0.1
0.0
0.1
MR leave−one−out sensitivity analysis for
'CD24+ CD27+ %B cell' on 'Juvenile arthritis'

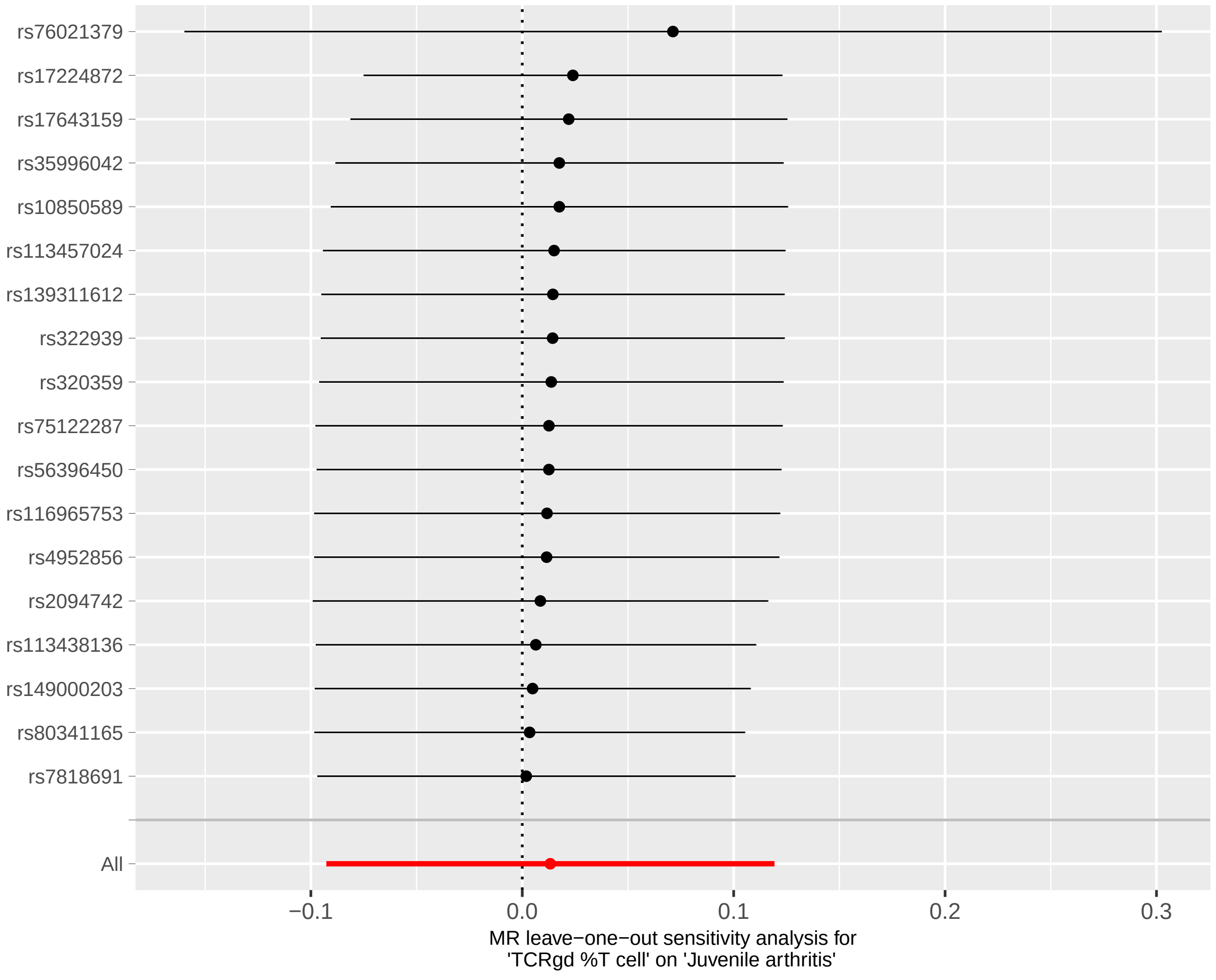
rs76021379
rs17224872
rs17643159
rs35996042
rs10850589
rs113457024
rs139311612
rs322939
rs320359
rs75122287
rs56396450
rs116965753
rs4952856
rs2094742
rs113438136
rs149000203
rs80341165
rs7818691
All
−0.1
0.0
0.1
0.2
0.3
MR leave−one−out sensitivity analysis for
'TCRgd %T cell' on 'Juvenile arthritis'

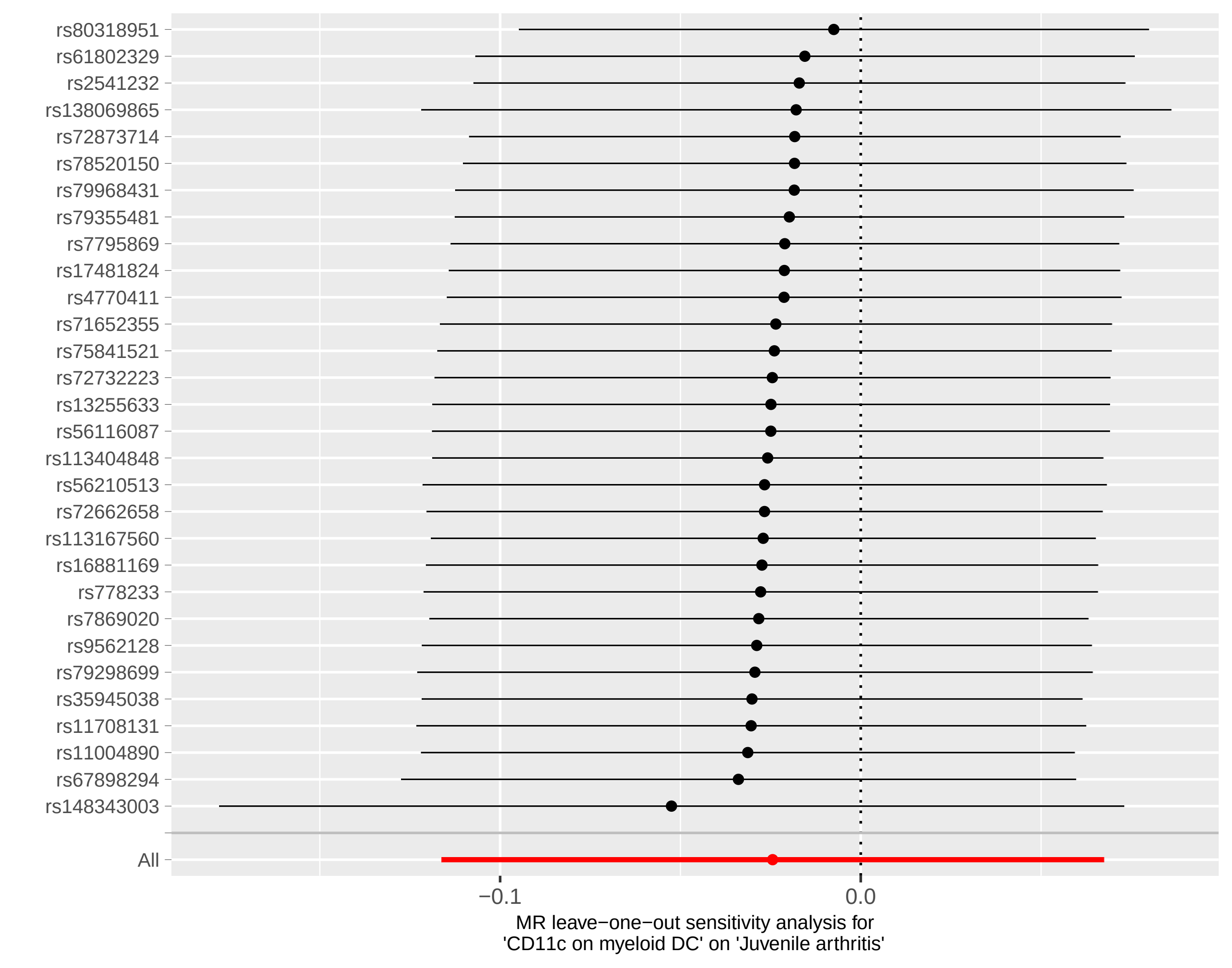
rs80318951
rs61802329
rs2541232
rs138069865
rs72873714
rs78520150
rs79968431
rs79355481
rs7795869
rs17481824
rs4770411
rs71652355
rs75841521
rs72732223
rs13255633
rs56116087
rs113404848
rs56210513
rs72662658
rs113167560
rs16881169
rs778233
rs7869020
rs9562128
rs79298699
rs35945038
rs11708131
rs11004890
rs67898294
rs148343003
All
−0.1
0.0
MR leave−one−out sensitivity analysis for
'CD11c on myeloid DC' on 'Juvenile arthritis'

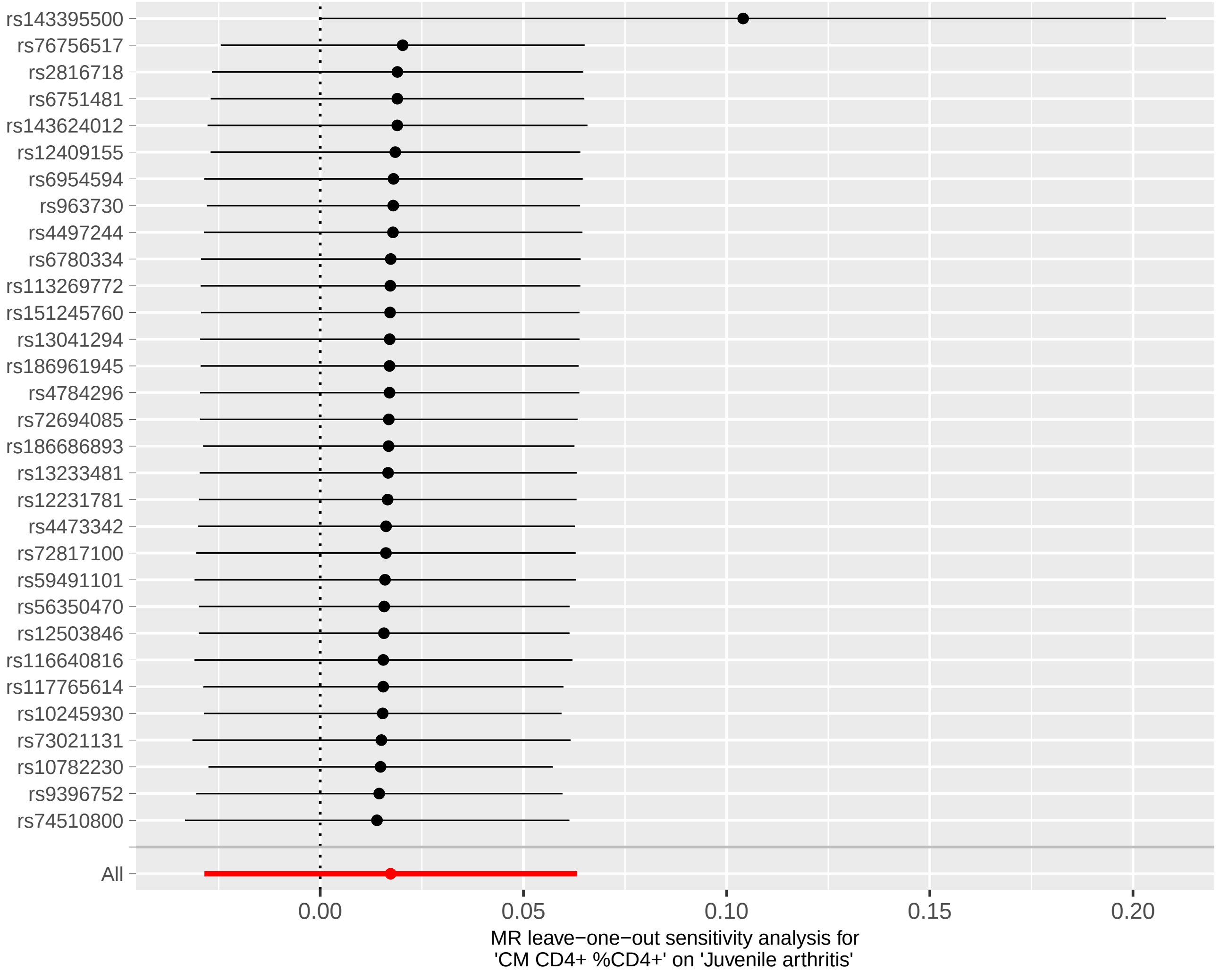

rs143395500
rs76756517
rs2816718
rs6751481
rs143624012
rs12409155
rs6954594
rs963730
rs4497244
rs6780334
rs113269772
rs151245760
rs13041294
rs186961945
rs4784296
rs72694085
rs186686893
rs13233481
rs12231781
rs4473342
rs72817100
rs59491101
rs56350470
rs12503846
rs116640816
rs117765614
rs10245930
rs73021131
rs10782230
rs9396752
rs74510800
All
0.00
0.05
0.10
0.15
0.20
MR leave−one−out sensitivity analysis for
'CM CD4+ %CD4+' on 'Juvenile arthritis'

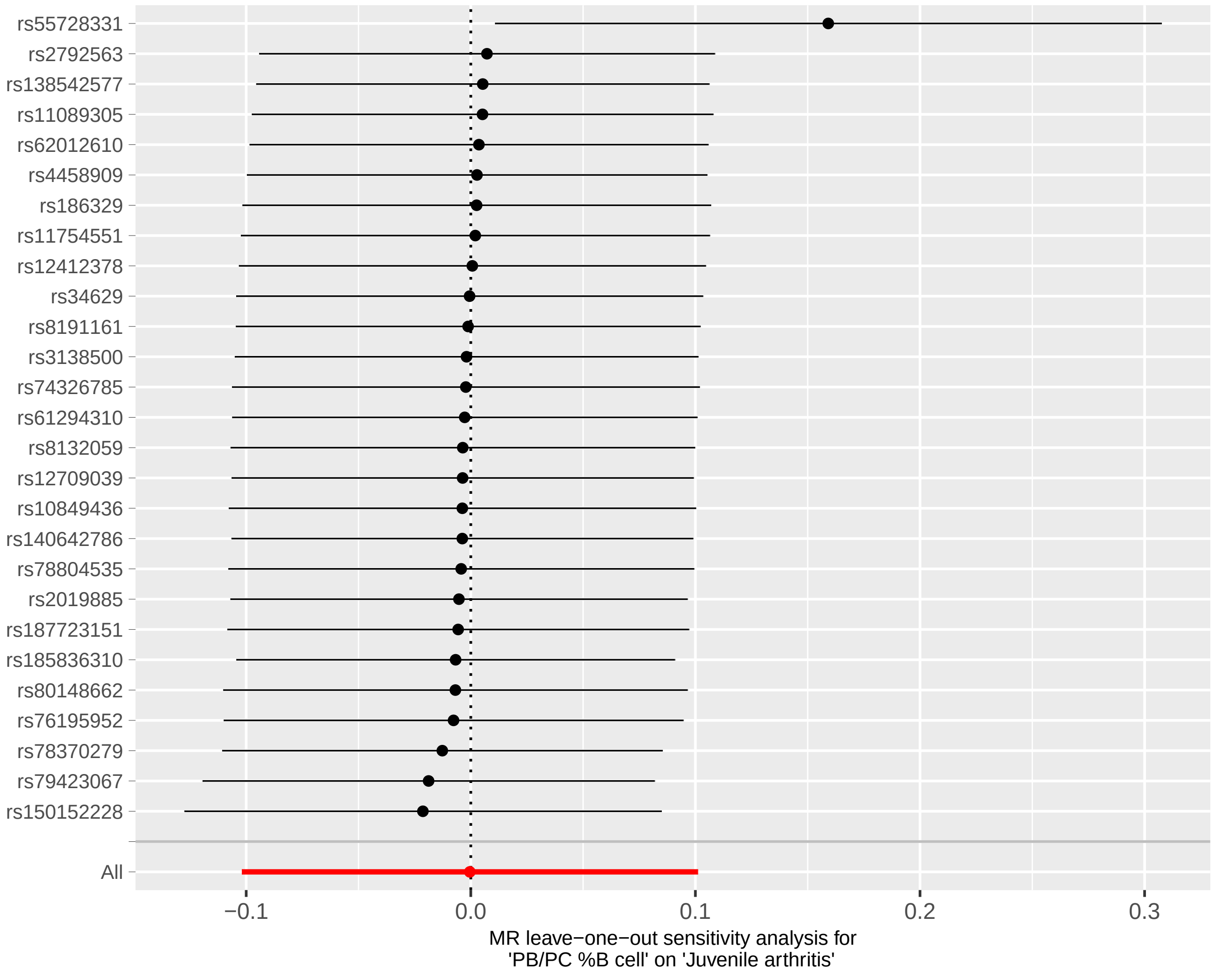

rs55728331
rs2792563
rs138542577
rs11089305
rs62012610
rs4458909
rs186329
rs11754551
rs12412378
rs34629
rs8191161
rs3138500
rs74326785
rs61294310
rs8132059
rs12709039
rs10849436
rs140642786
rs78804535
rs2019885
rs187723151
rs185836310
rs80148662
rs76195952
rs78370279
rs79423067
rs150152228
All
−0.1
0.0
0.1
0.2
0.3
MR leave−one−out sensitivity analysis for
'PB/PC %B cell' on 'Juvenile arthritis'

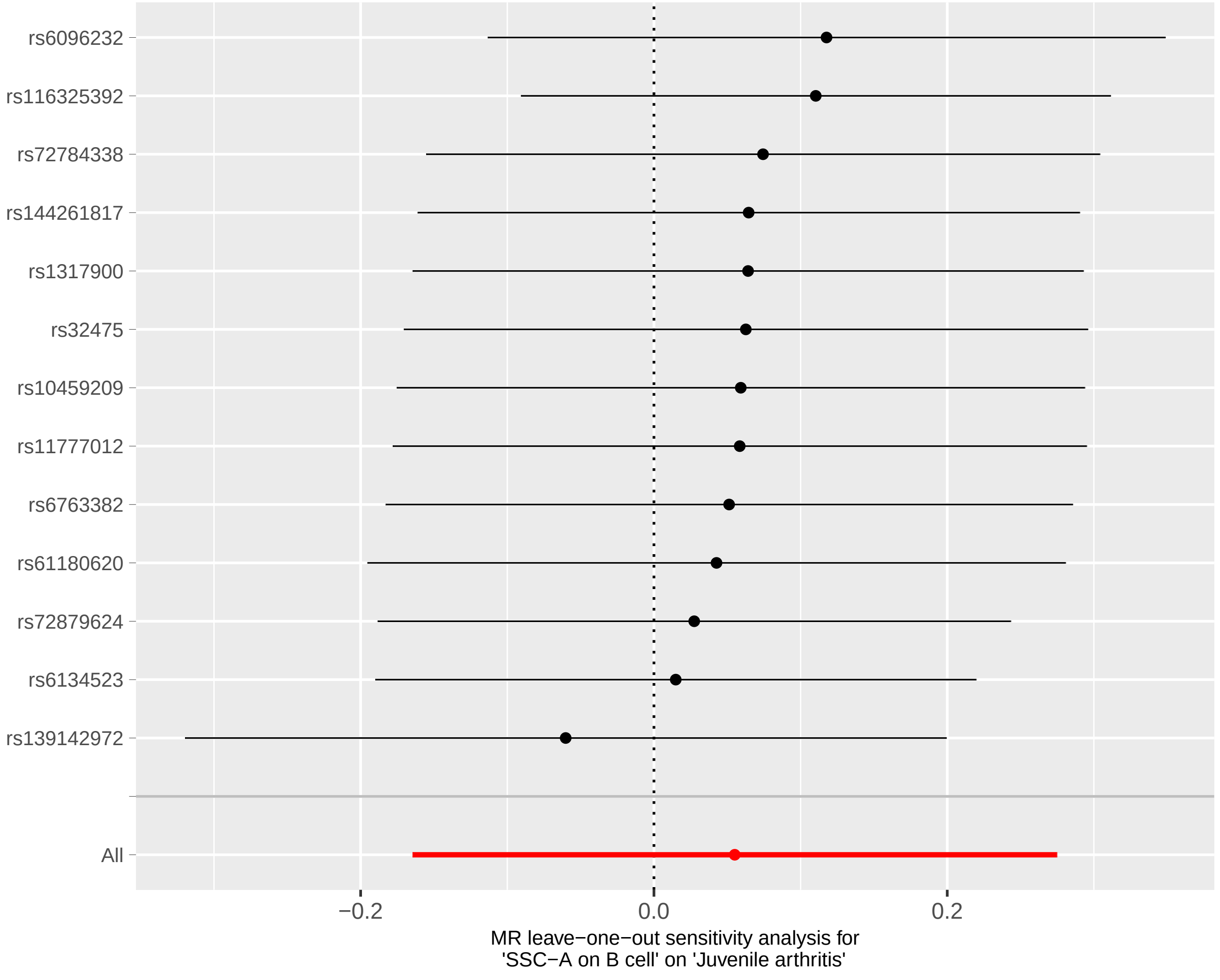
rs6096232
rs116325392
rs72784338
rs144261817
rs1317900
rs32475
rs10459209
rs11777012
rs6763382
rs61180620
rs72879624
rs6134523
rs139142972
All
−0.2
0.0
0.2
MR leave−one−out sensitivity analysis for
'SSC−A on B cell' on 'Juvenile arthritis'

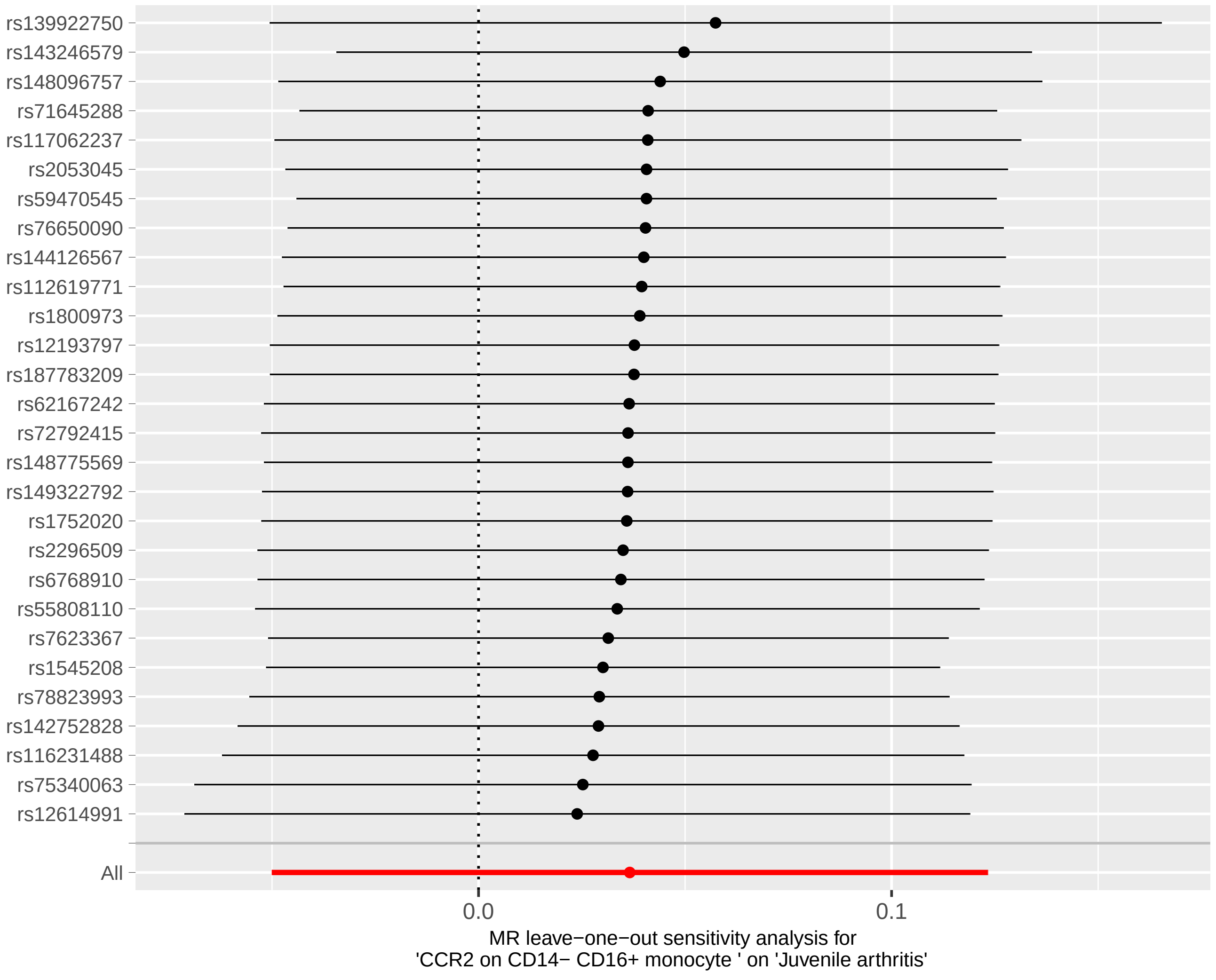
rs139922750
rs143246579
rs148096757
rs71645288
rs117062237
rs2053045
rs59470545
rs76650090
rs144126567
rs112619771
rs1800973
rs12193797
rs187783209
rs62167242
rs72792415
rs148775569
rs149322792
rs1752020
rs2296509
rs6768910
rs55808110
rs7623367
rs1545208
rs78823993
rs142752828
rs116231488
rs75340063
rs12614991
All
0.0
0.1
MR leave−one−out sensitivity analysis for
'CCR2 on CD14− CD16+ monocyte ' on 'Juvenile arthritis'

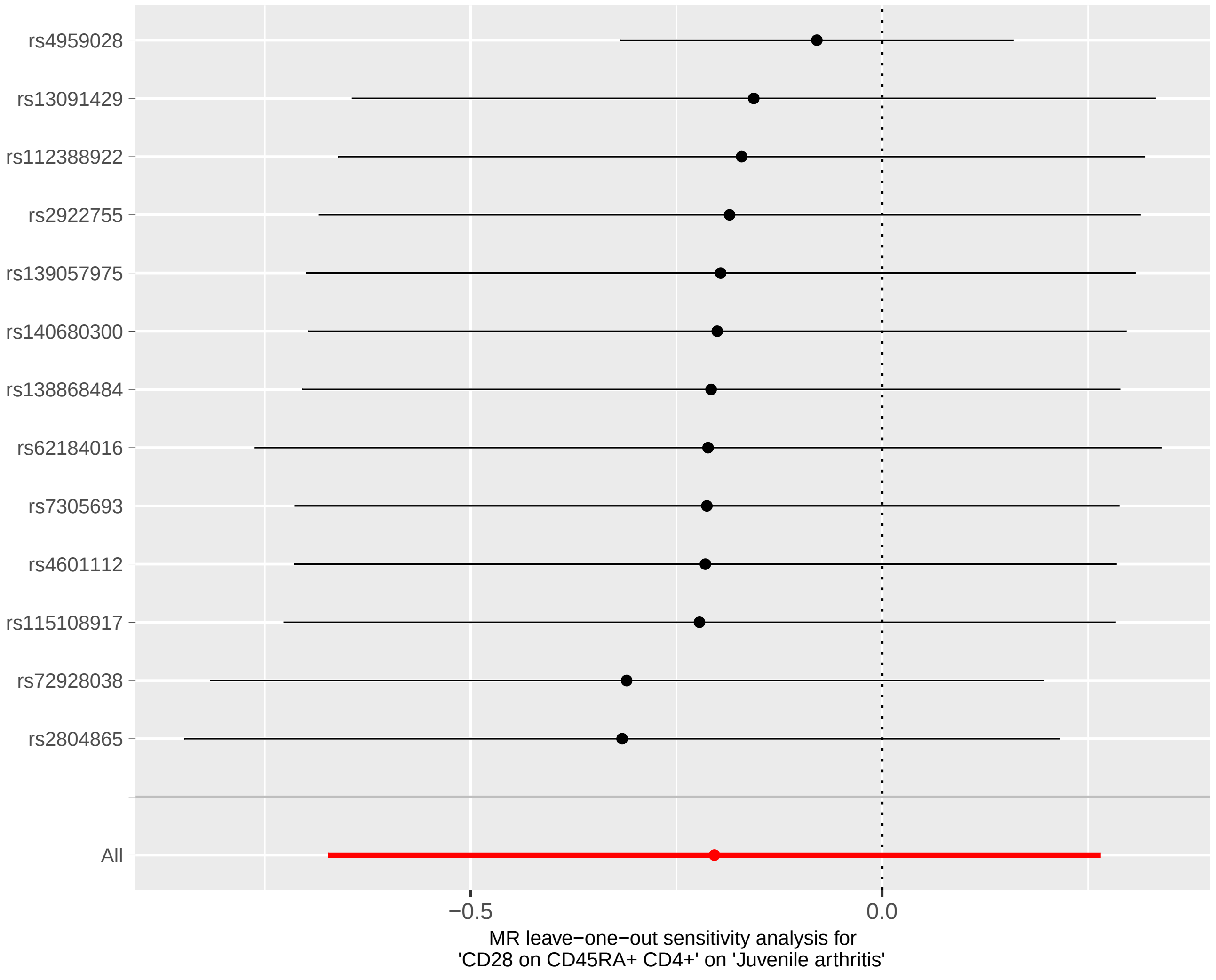
rs4959028
rs13091429
rs112388922
rs2922755
rs139057975
rs140680300
rs138868484
rs62184016
rs7305693
rs4601112
rs115108917
rs72928038
rs2804865
All
−0.5
0.0
MR leave−one−out sensitivity analysis for
'CD28 on CD45RA+ CD4+' on 'Juvenile arthritis'

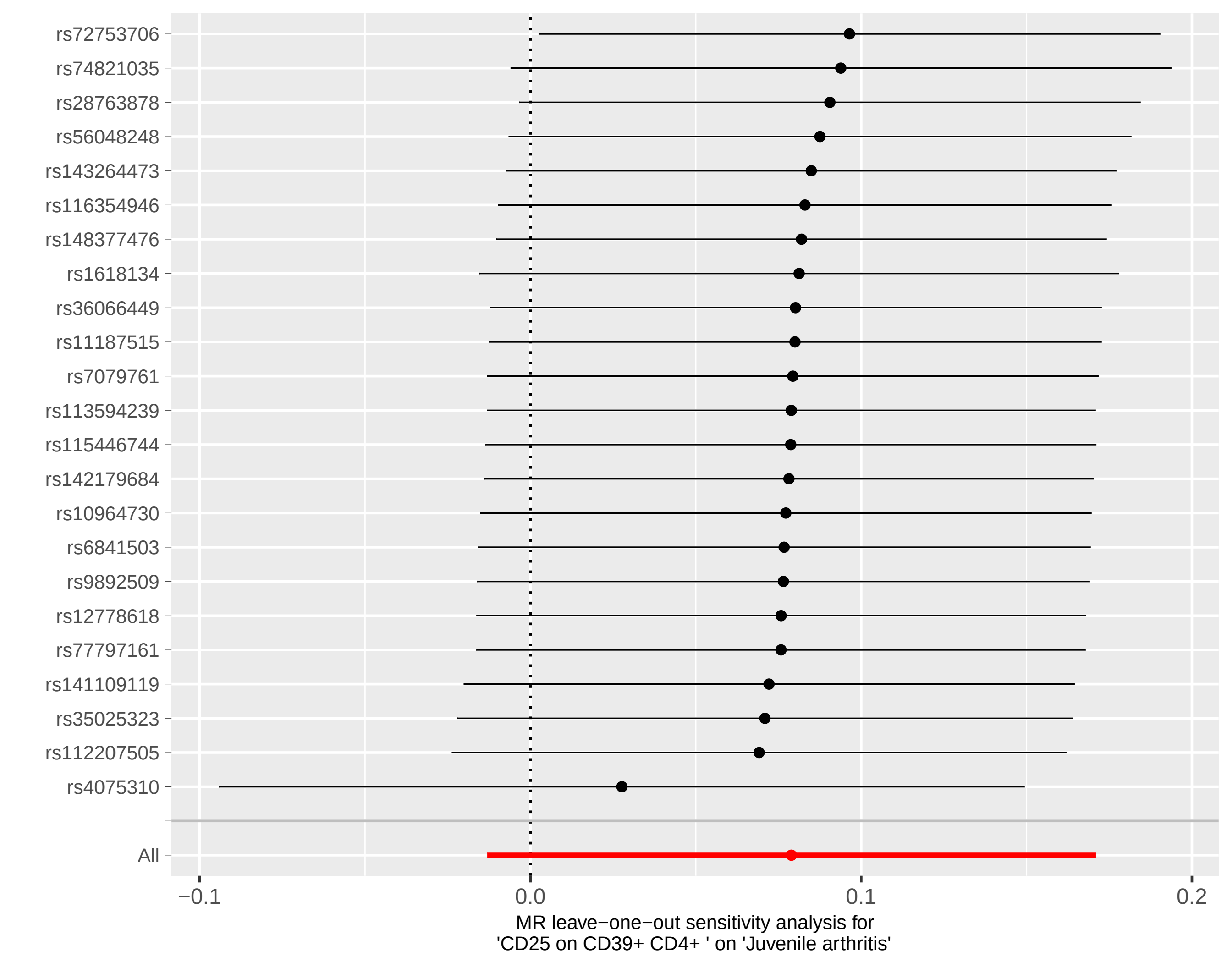
rs72753706
rs74821035
rs28763878
rs56048248
rs143264473
rs116354946
rs148377476
rs1618134
rs36066449
rs11187515
rs7079761
rs113594239
rs115446744
rs142179684
rs10964730
rs6841503
rs9892509
rs12778618
rs77797161
rs141109119
rs35025323
rs112207505
rs4075310
All
−0.1
0.0
0.1
0.2
MR leave−one−out sensitivity analysis for
'CD25 on CD39+ CD4+ ' on 'Juvenile arthritis'

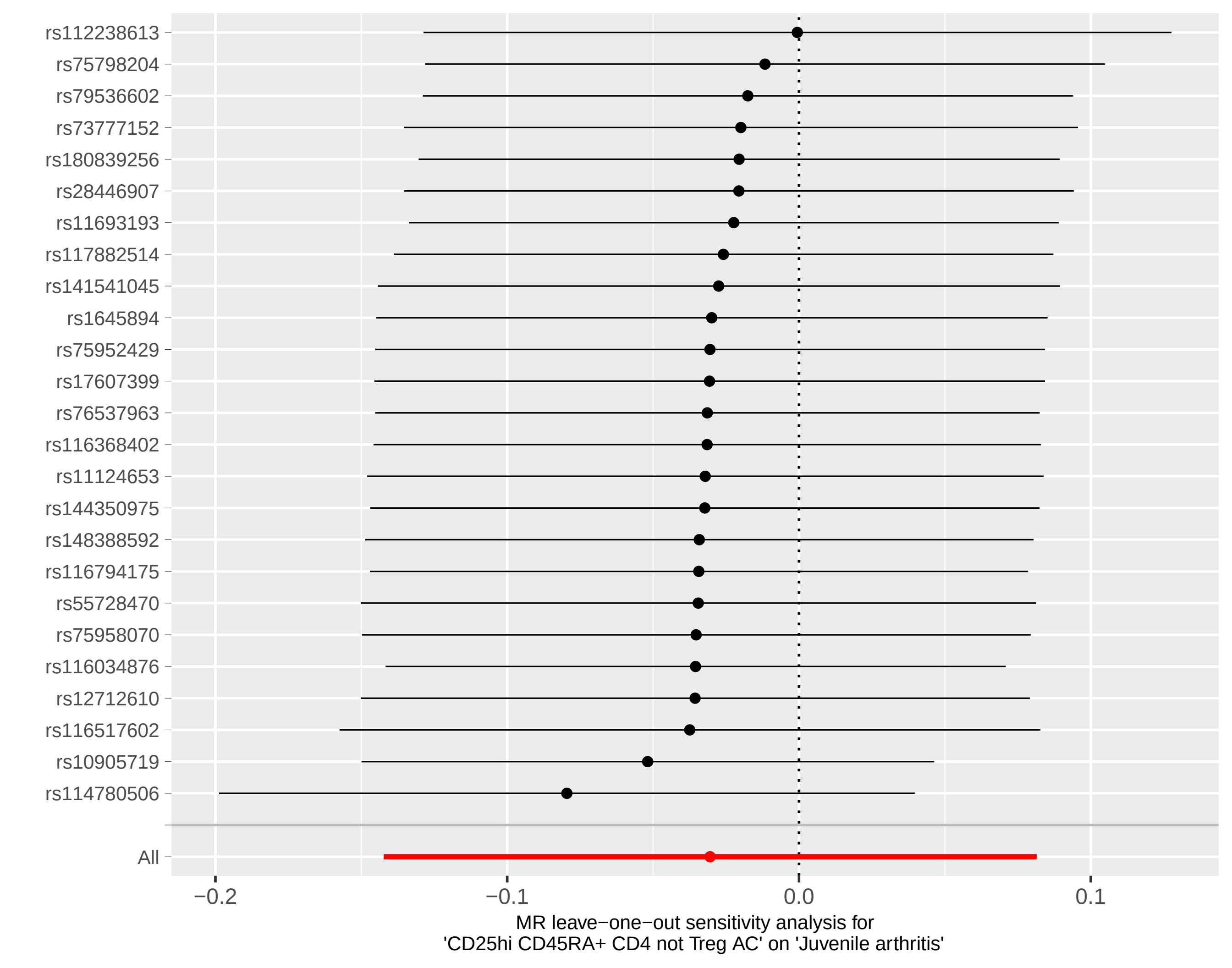

rs112238613
rs75798204
rs79536602
rs73777152
rs180839256
rs28446907
rs11693193
rs117882514
rs141541045
rs1645894
rs75952429
rs17607399
rs76537963
rs116368402
rs11124653
rs144350975
rs148388592
rs116794175
rs55728470
rs75958070
rs116034876
rs12712610
rs116517602
rs10905719
rs114780506
All
−0.2
−0.1
0.0
0.1
MR leave−one−out sensitivity analysis for
'CD25hi CD45RA+ CD4 not Treg AC' on 'Juvenile arthritis'

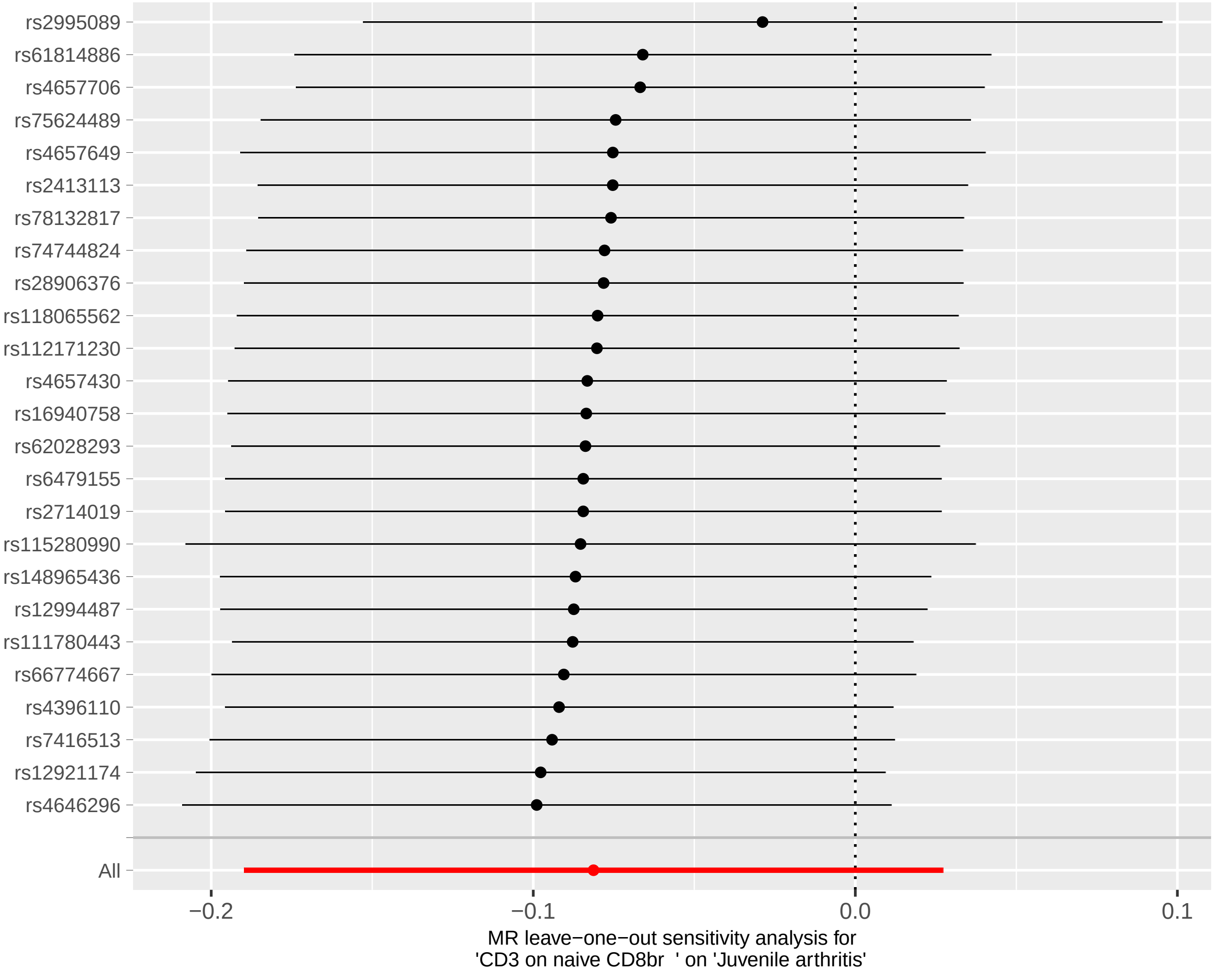
rs2995089
rs61814886
rs4657706
rs75624489
rs4657649
rs2413113
rs78132817
rs74744824
rs28906376
rs118065562
rs112171230
rs4657430
rs16940758
rs62028293
rs6479155
rs2714019
rs115280990
rs148965436
rs12994487
rs111780443
rs66774667
rs4396110
rs7416513
rs12921174
rs4646296
All
−0.2
−0.1
0.0
0.1
MR leave−one−out sensitivity analysis for
'CD3 on naive CD8br ' on 'Juvenile arthritis'

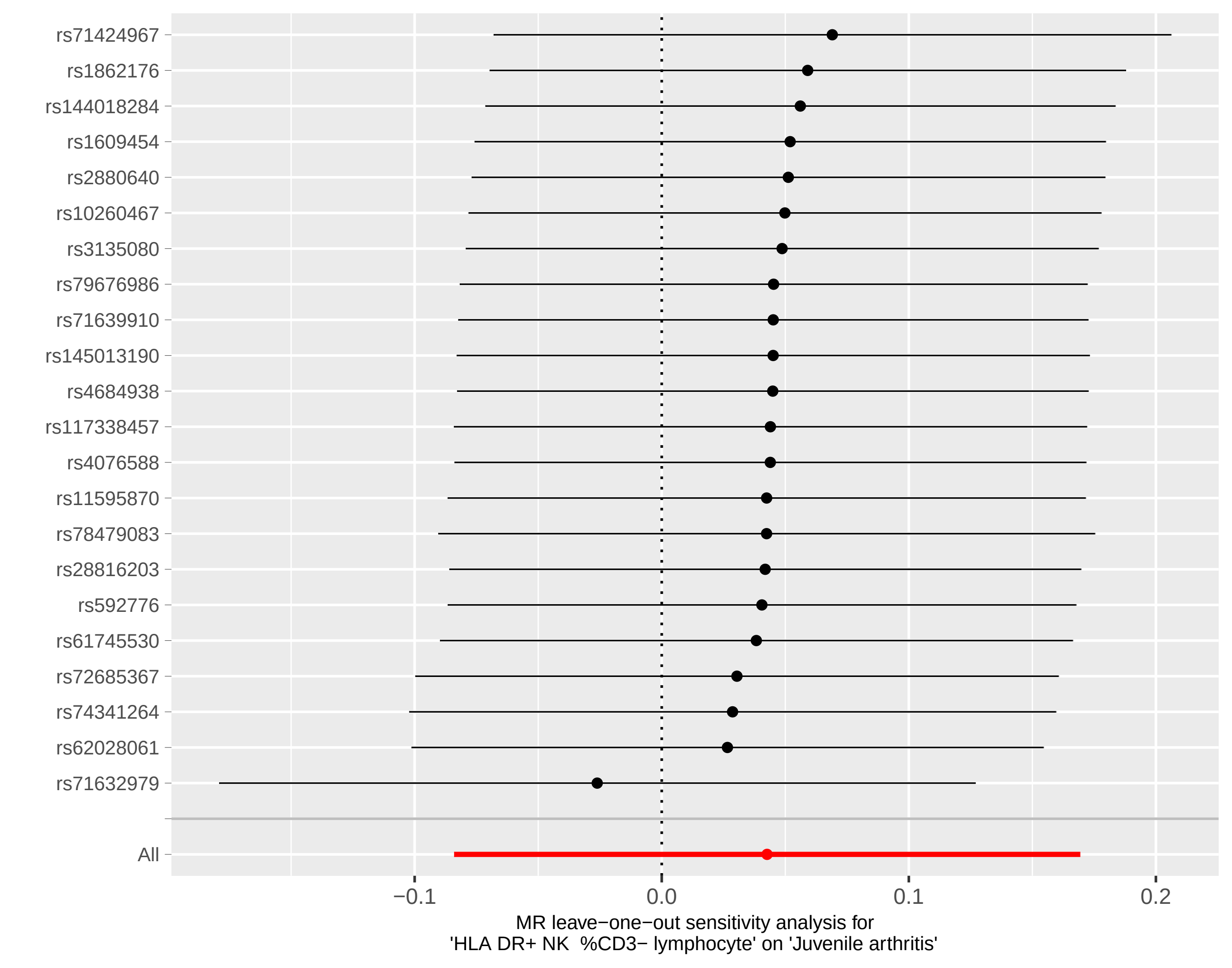
rs71424967
rs1862176
rs144018284
rs1609454
rs2880640
rs10260467
rs3135080
rs79676986
rs71639910
rs145013190
rs4684938
rs117338457
rs4076588
rs11595870
rs78479083
rs28816203
rs592776
rs61745530
rs72685367
rs74341264
rs62028061
rs71632979
All
−0.1
0.0
0.1
0.2
MR leave−one−out sensitivity analysis for
'HLA DR+ NK %CD3− lymphocyte' on 'Juvenile arthritis'

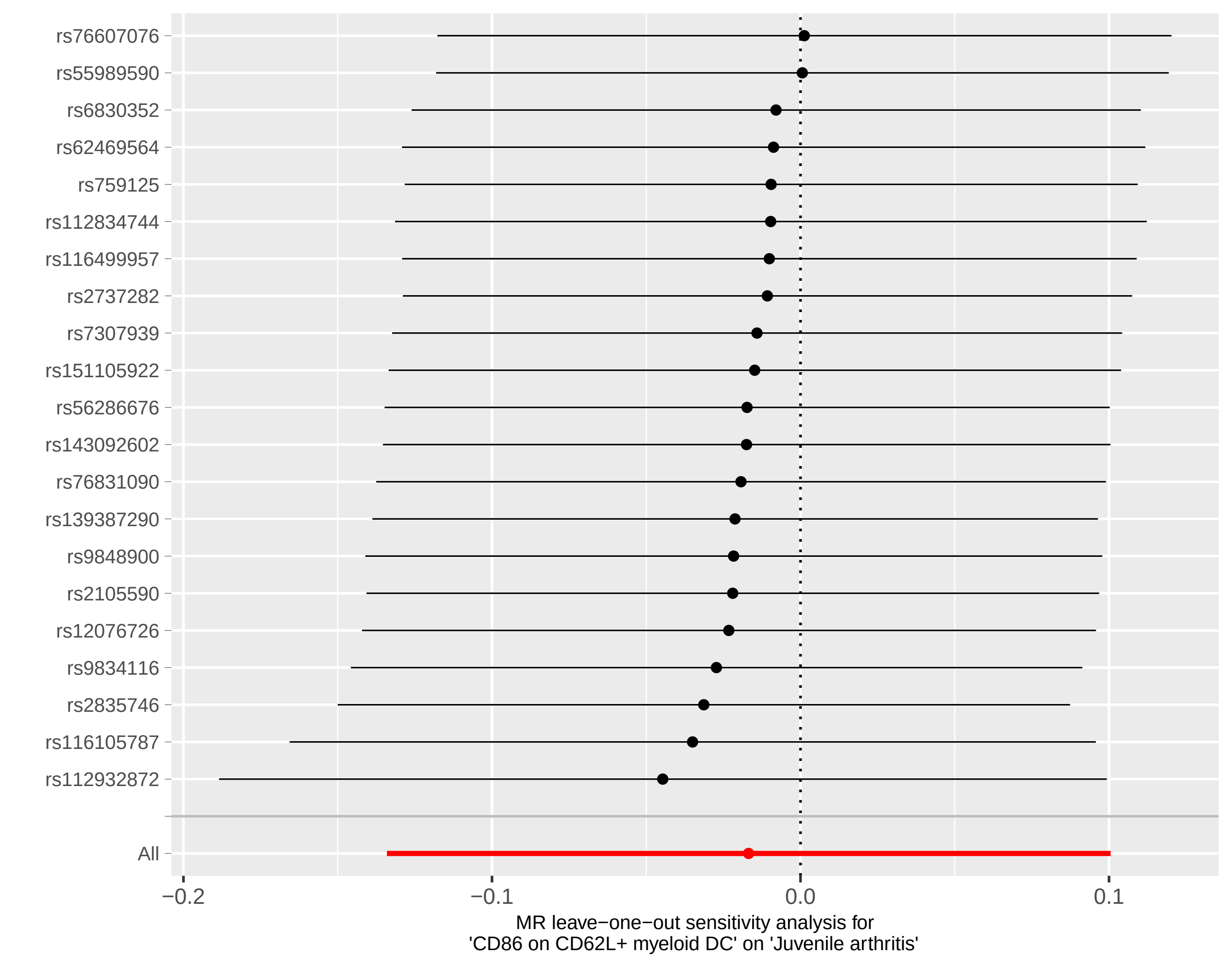
rs76607076
rs55989590
rs6830352
rs62469564
rs759125
rs112834744
rs116499957
rs2737282
rs7307939
rs151105922
rs56286676
rs143092602
rs76831090
rs139387290
rs9848900
rs2105590
rs12076726
rs9834116
rs2835746
rs116105787
rs112932872
All
−0.2
−0.1
0.0
0.1
MR leave−one−out sensitivity analysis for
'CD86 on CD62L+ myeloid DC' on 'Juvenile arthritis'

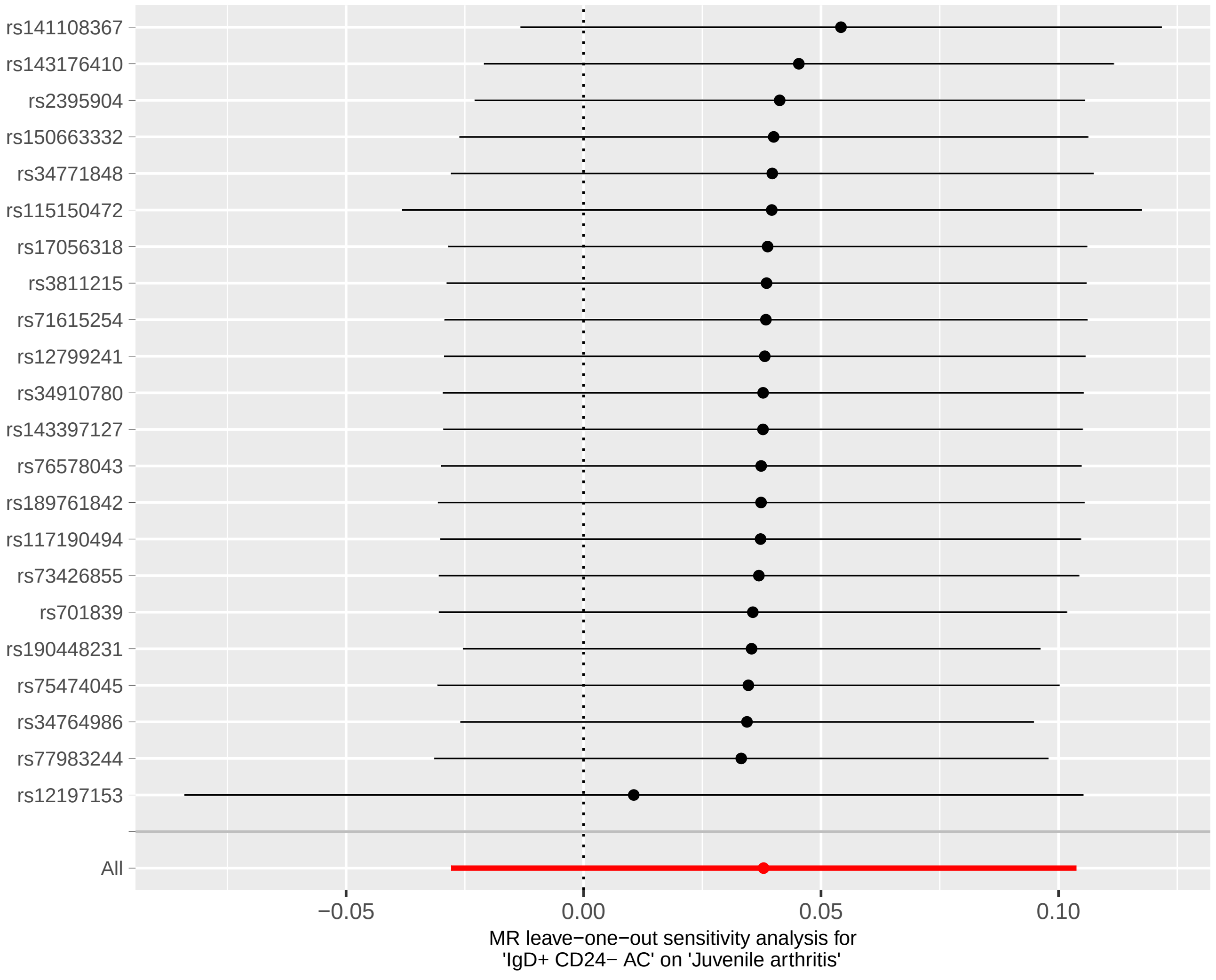

rs141108367
rs143176410
rs2395904
rs150663332
rs34771848
rs115150472
rs17056318
rs3811215
rs71615254
rs12799241
rs34910780
rs143397127
rs76578043
rs189761842
rs117190494
rs73426855
rs701839
rs190448231
rs75474045
rs34764986
rs77983244
rs12197153
All
−0.05
0.00
0.05
0.10
MR leave−one−out sensitivity analysis for
'IgD+ CD24− AC' on 'Juvenile arthritis'

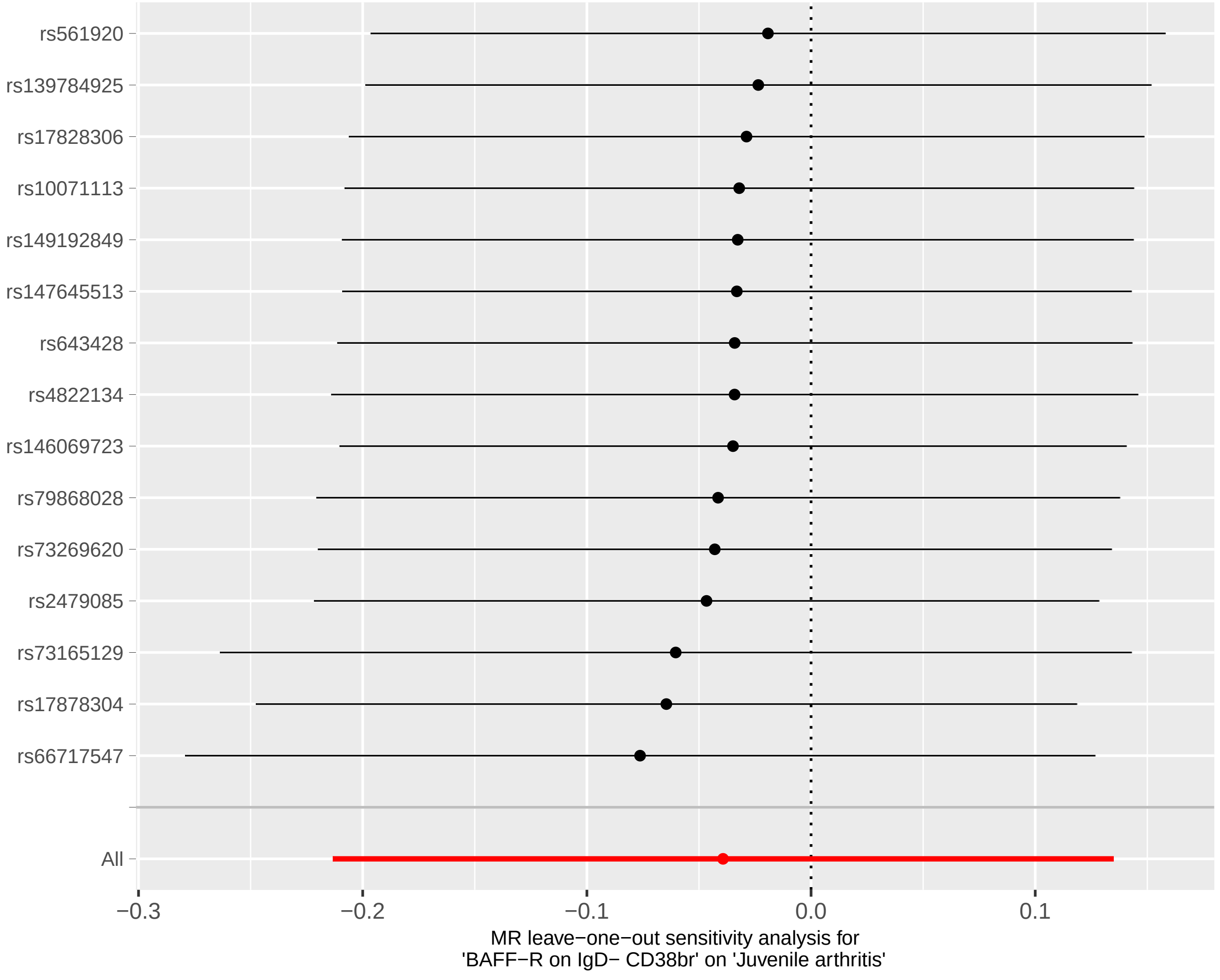
rs561920
rs139784925
rs17828306
rs10071113
rs149192849
rs147645513
rs643428
rs4822134
rs146069723
rs79868028
rs73269620
rs2479085
rs73165129
rs17878304
rs66717547
All
−0.3
−0.2
−0.1
0.0
0.1
MR leave−one−out sensitivity analysis for
'BAFF−R on IgD− CD38br' on 'Juvenile arthritis'

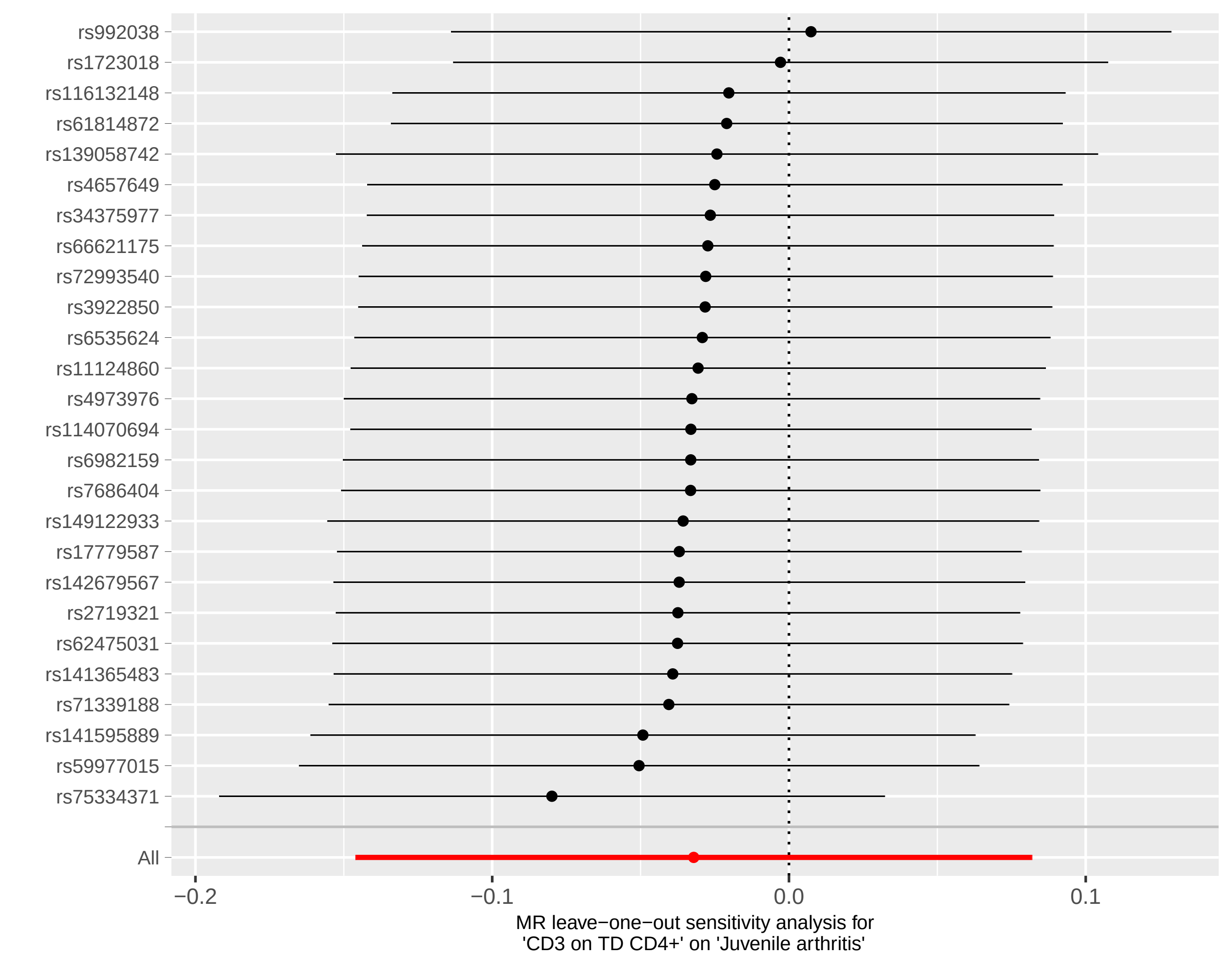
rs992038
rs1723018
rs116132148
rs61814872
rs139058742
rs4657649
rs34375977
rs66621175
rs72993540
rs3922850
rs6535624
rs11124860
rs4973976
rs114070694
rs6982159
rs7686404
rs149122933
rs17779587
rs142679567
rs2719321
rs62475031
rs141365483
rs71339188
rs141595889
rs59977015
rs75334371
All
−0.2
−0.1
0.0
0.1
MR leave−one−out sensitivity analysis for
'CD3 on TD CD4+' on 'Juvenile arthritis'

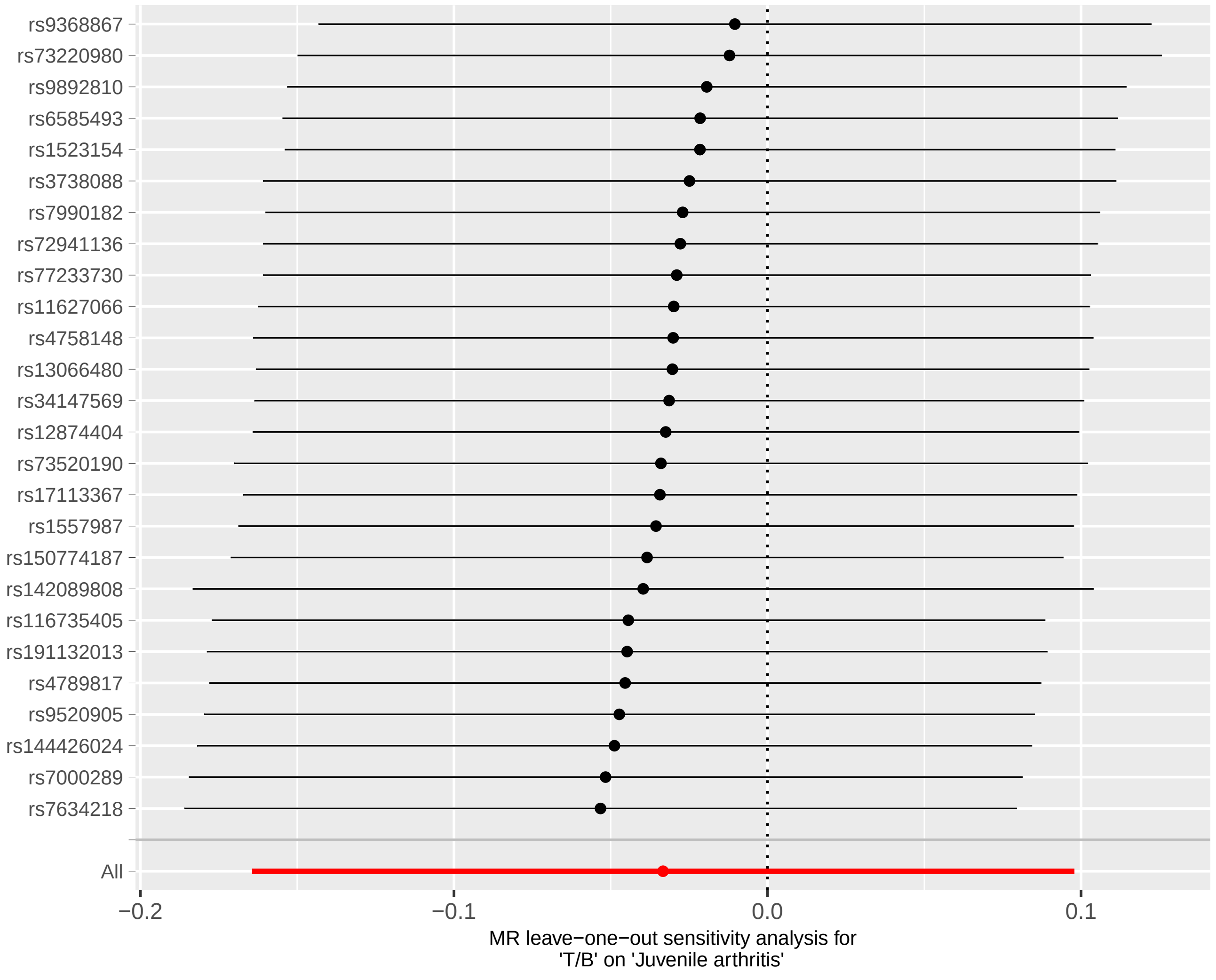
rs9368867
rs73220980
rs9892810
rs6585493
rs1523154
rs3738088
rs7990182
rs72941136
rs77233730
rs11627066
rs4758148
rs13066480
rs34147569
rs12874404
rs73520190
rs17113367
rs1557987
rs150774187
rs142089808
rs116735405
rs191132013
rs4789817
rs9520905
rs144426024
rs7000289
rs7634218
All
−0.2
−0.1
0.0
0.1
MR leave−one−out sensitivity analysis for
'T/B' on 'Juvenile arthritis'

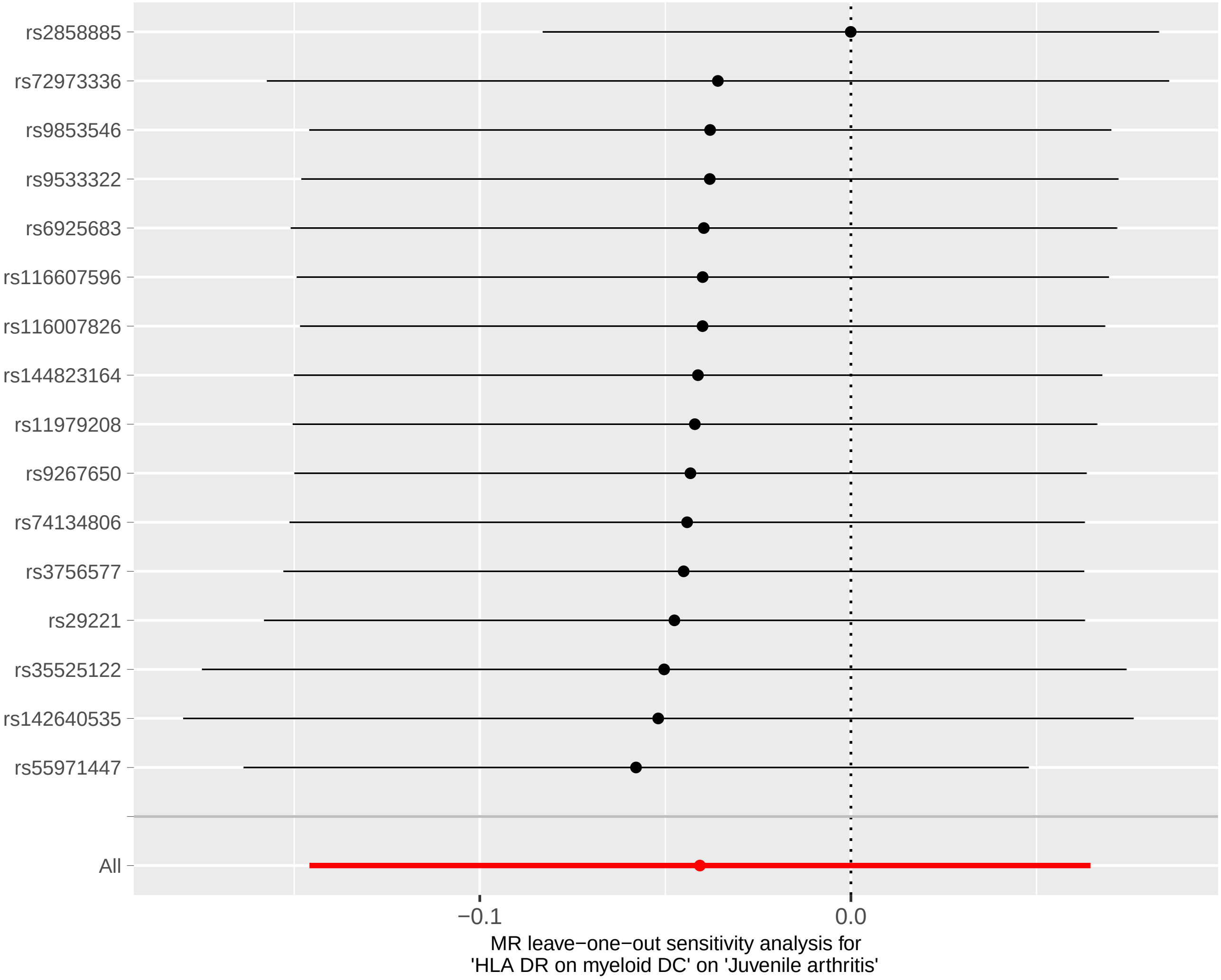
rs2858885
rs72973336
rs9853546
rs9533322
rs6925683
rs116607596
rs116007826
rs144823164
rs11979208
rs9267650
rs74134806
rs3756577
rs29221
rs35525122
rs142640535
rs55971447
All
−0.1
0.0
MR leave−one−out sensitivity analysis for
'HLA DR on myeloid DC' on 'Juvenile arthritis'

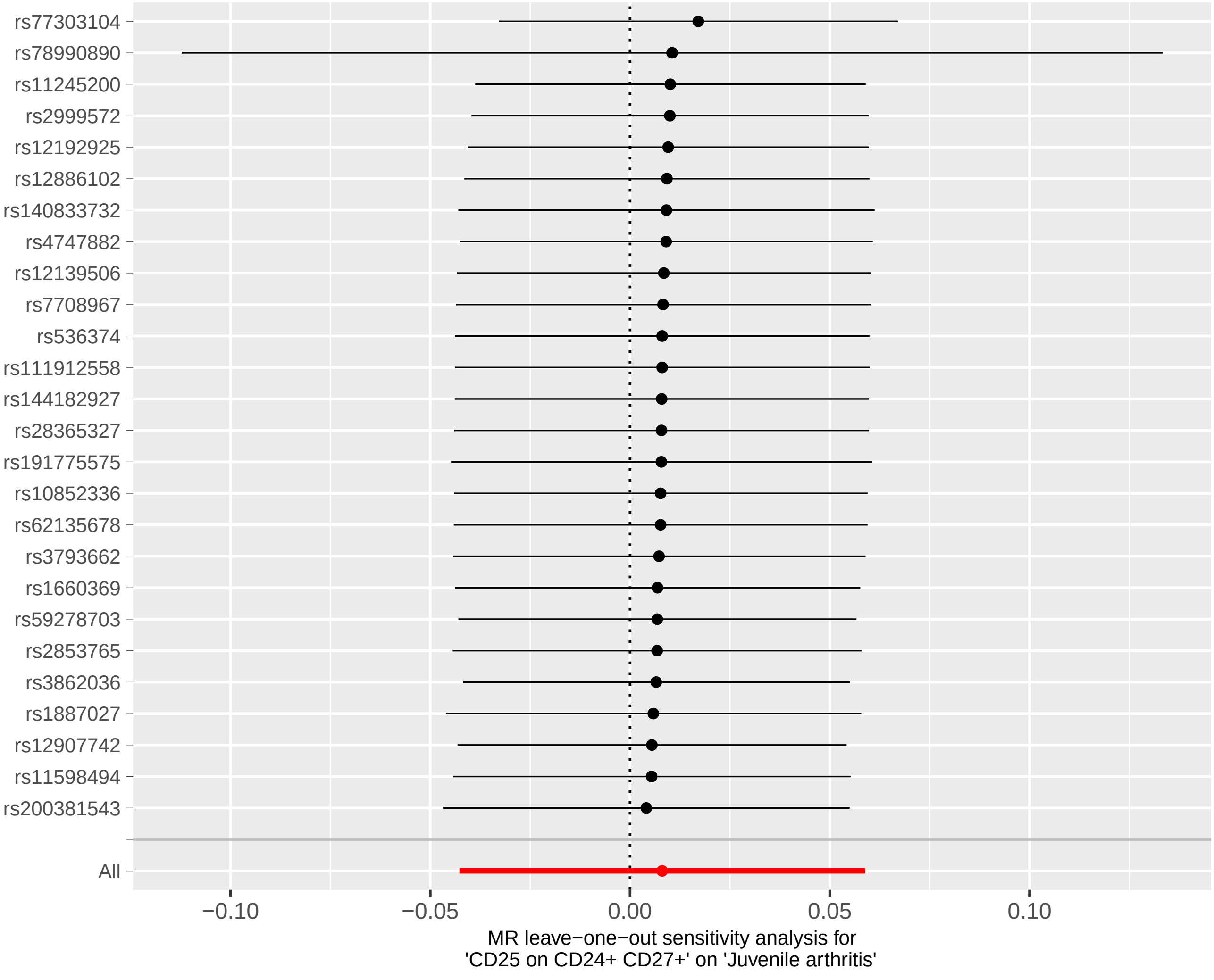

rs77303104
rs78990890
rs11245200
rs2999572
rs12192925
rs12886102
rs140833732
rs4747882
rs12139506
rs7708967
rs536374
rs111912558
rs144182927
rs28365327
rs191775575
rs10852336
rs62135678
rs3793662
rs1660369
rs59278703
rs2853765
rs3862036
rs1887027
rs12907742
rs11598494
rs200381543
All
−0.10
−0.05
0.00
0.05
0.10
MR leave−one−out sensitivity analysis for
'CD25 on CD24+ CD27+' on 'Juvenile arthritis'

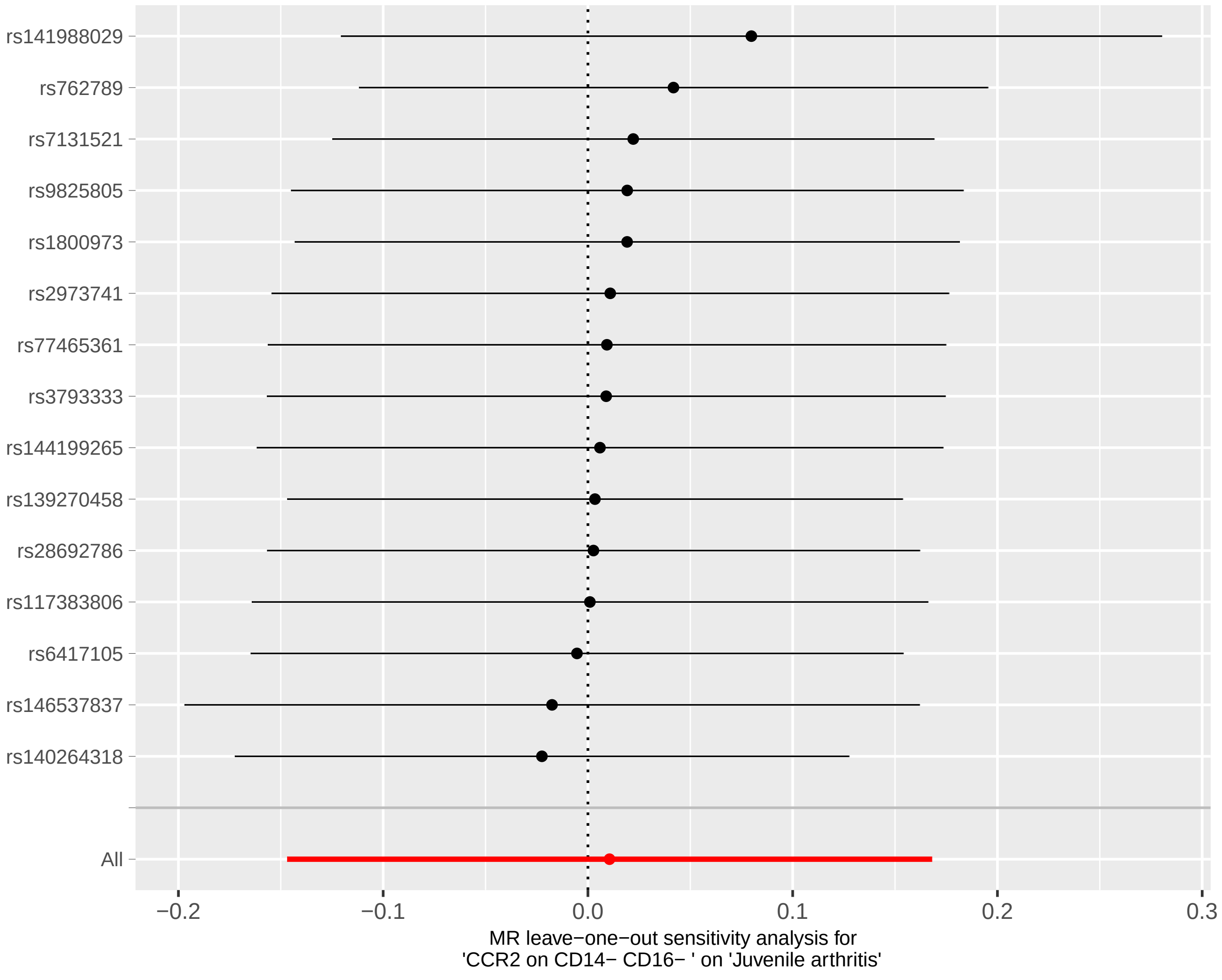

rs141988029
rs762789
rs7131521
rs9825805
rs1800973
rs2973741
rs77465361
rs3793333
rs144199265
rs139270458
rs28692786
rs117383806
rs6417105
rs146537837
rs140264318
All
−0.2
−0.1
0.0
0.1
0.2
0.3
MR leave−one−out sensitivity analysis for
'CCR2 on CD14− CD16− ' on 'Juvenile arthritis'

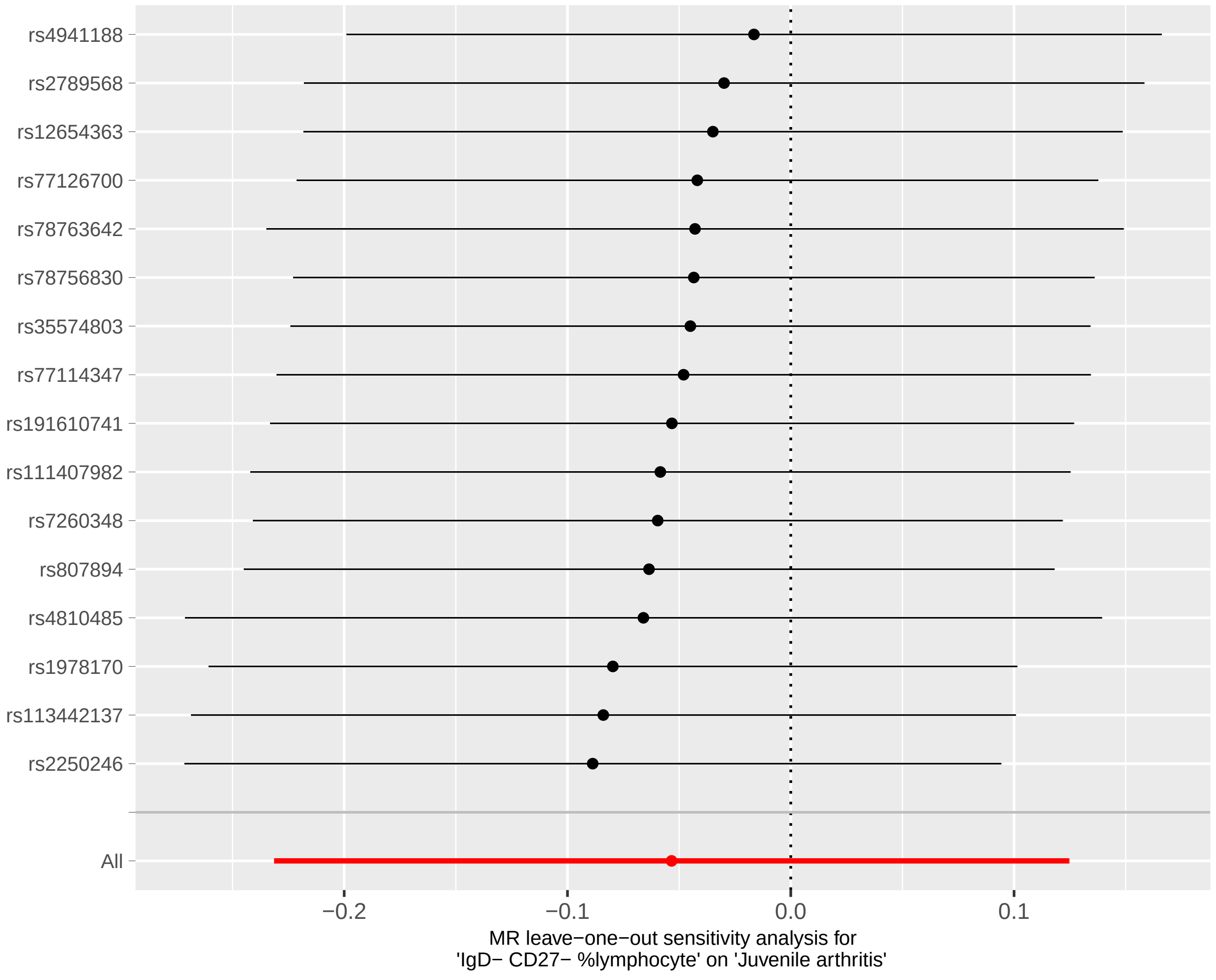
rs4941188
rs2789568
rs12654363
rs77126700
rs78763642
rs78756830
rs35574803
rs77114347
rs191610741
rs111407982
rs7260348
rs807894
rs4810485
rs1978170
rs113442137
rs2250246
All
−0.2
−0.1
0.0
0.1
MR leave−one−out sensitivity analysis for
'IgD− CD27− %lymphocyte' on 'Juvenile arthritis'

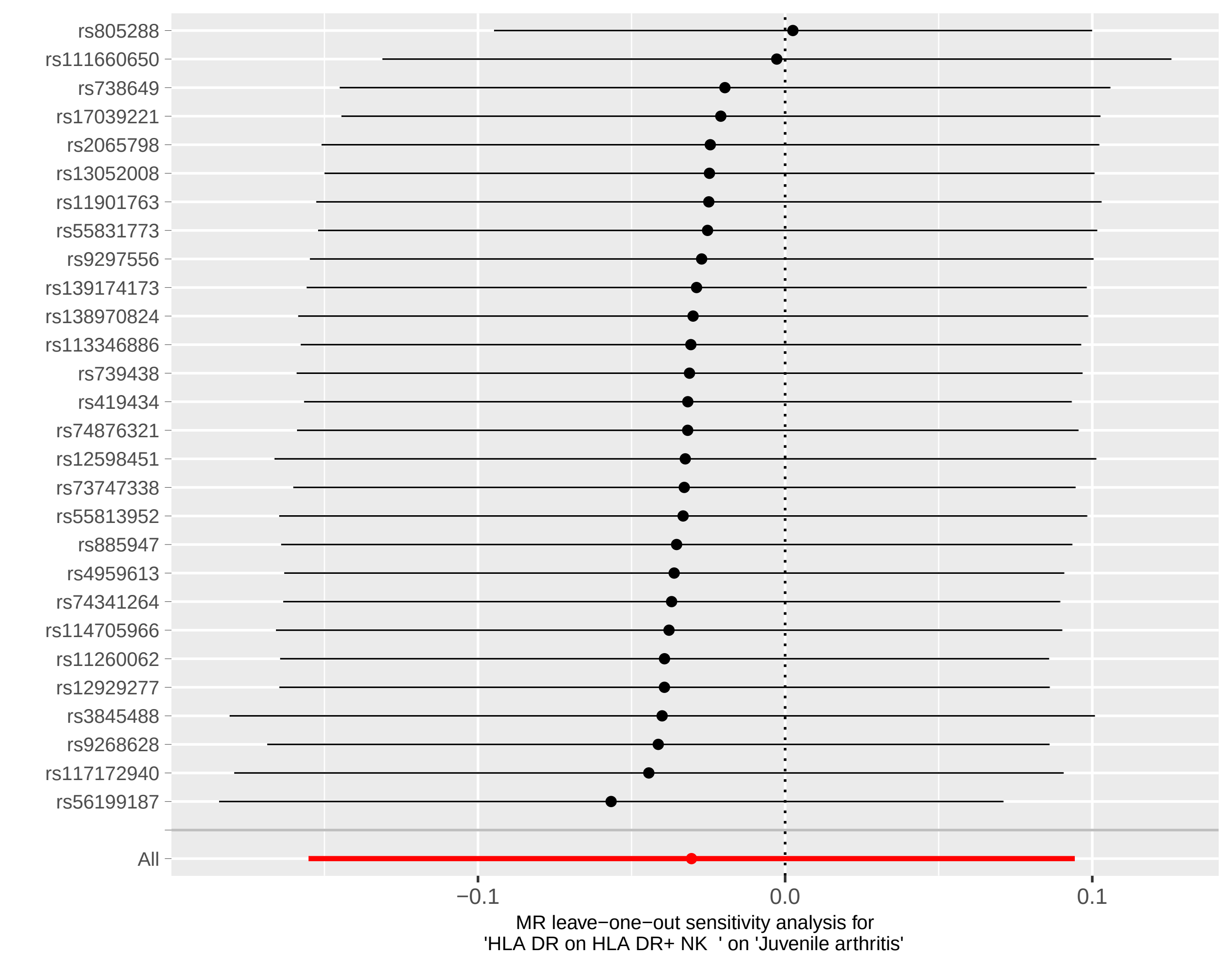

rs805288
rs111660650
rs738649
rs17039221
rs2065798
rs13052008
rs11901763
rs55831773
rs9297556
rs139174173
rs138970824
rs113346886
rs739438
rs419434
rs74876321
rs12598451
rs73747338
rs55813952
rs885947
rs4959613
rs74341264
rs114705966
rs11260062
rs12929277
rs3845488
rs9268628
rs117172940
rs56199187
All
−0.1
0.0
0.1
MR leave−one−out sensitivity analysis for
'HLA DR on HLA DR+ NK ' on 'Juvenile arthritis'

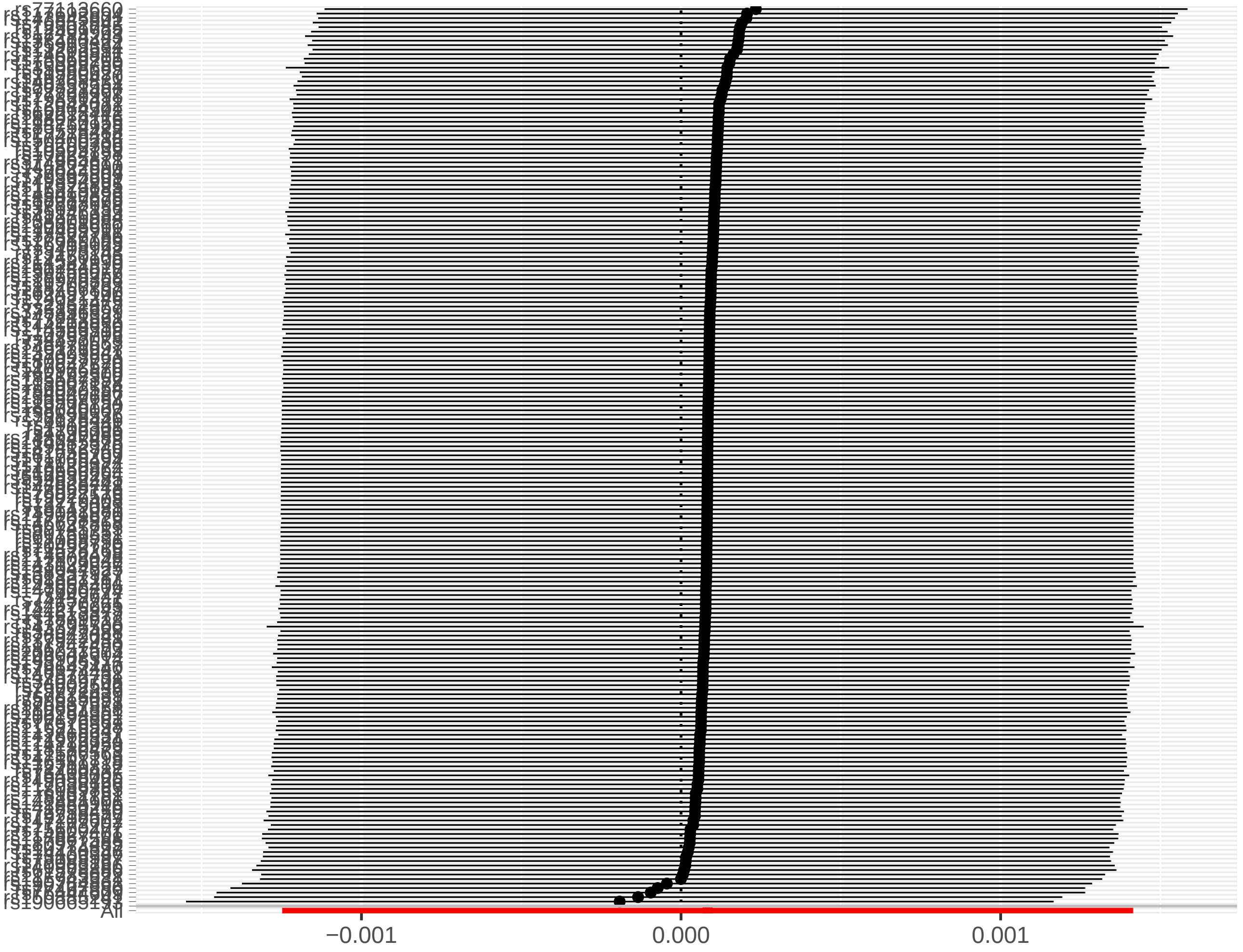

MR leave−one−out sensitivity analysis for
'CD45RA+ CD28− CD8br %T cell' on 'Juvenile arthritis'

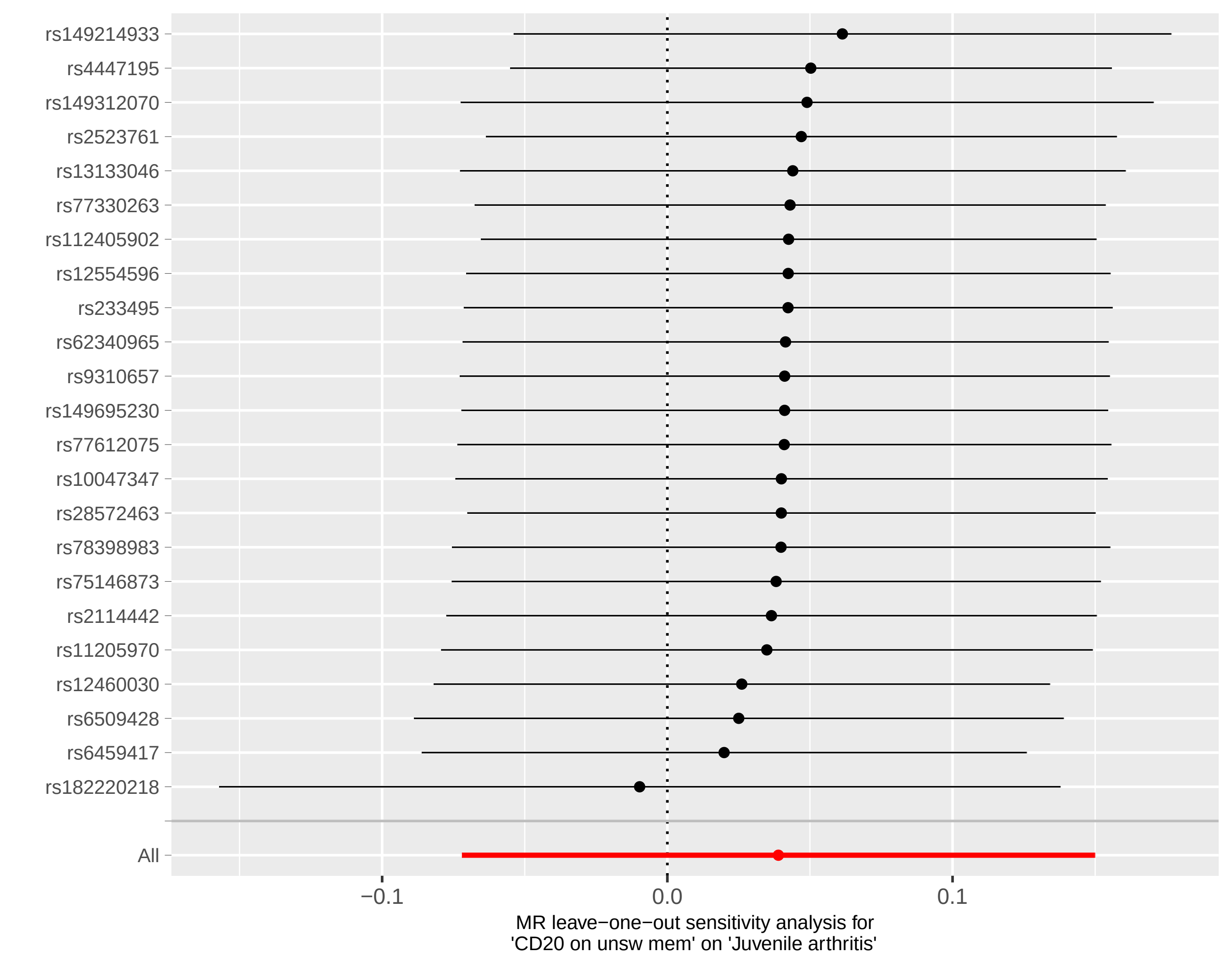
rs149214933
rs4447195
rs149312070
rs2523761
rs13133046
rs77330263
rs112405902
rs12554596
rs233495
rs62340965
rs9310657
rs149695230
rs77612075
rs10047347
rs28572463
rs78398983
rs75146873
rs2114442
rs11205970
rs12460030
rs6509428
rs6459417
rs182220218
All
−0.1
0.0
0.1
MR leave−one−out sensitivity analysis for
'CD20 on unsw mem' on 'Juvenile arthritis'

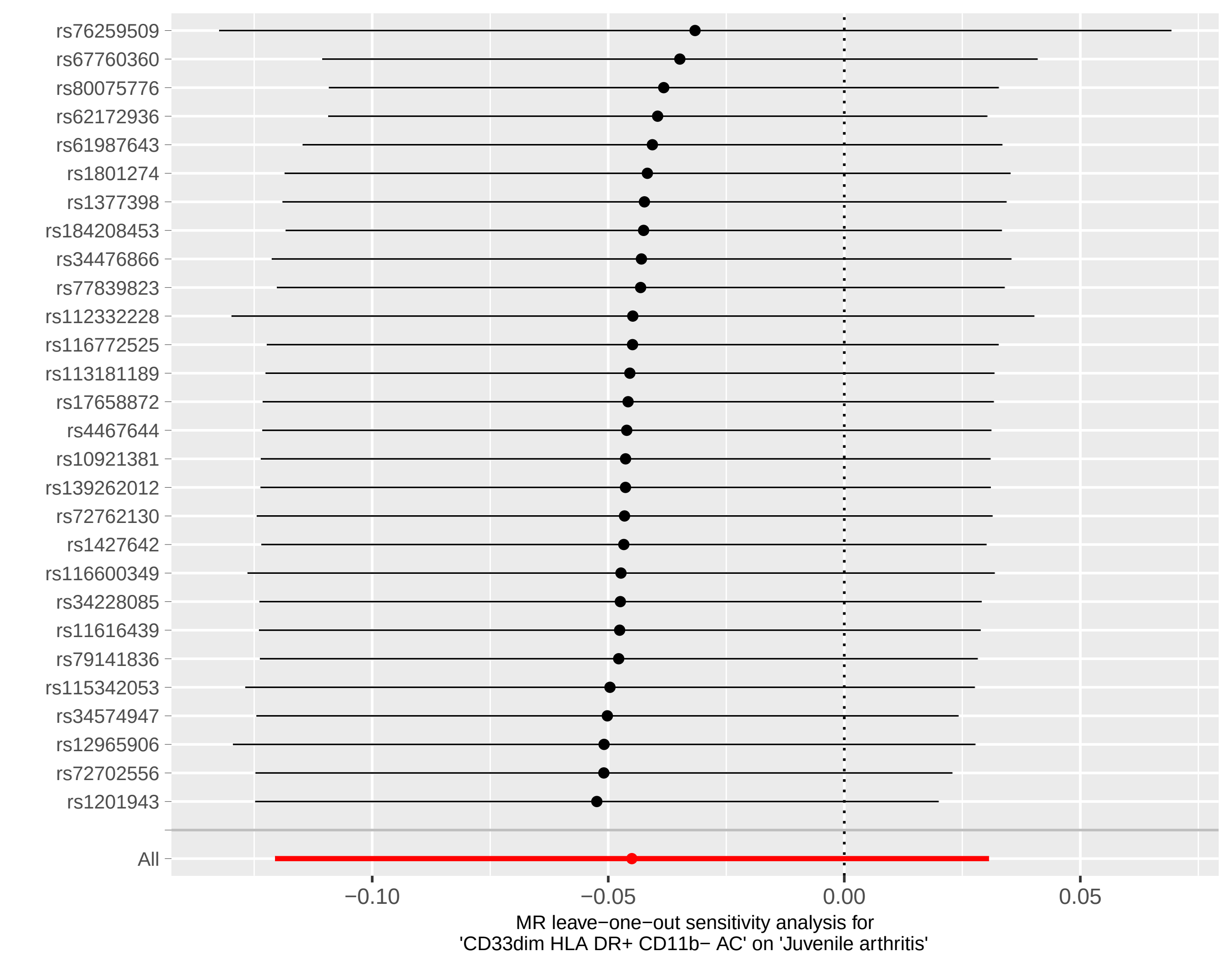
rs76259509
rs67760360
rs80075776
rs62172936
rs61987643
rs1801274
rs1377398
rs184208453
rs34476866
rs77839823
rs112332228
rs116772525
rs113181189
rs17658872
rs4467644
rs10921381
rs139262012
rs72762130
rs1427642
rs116600349
rs34228085
rs11616439
rs79141836
rs115342053
rs34574947
rs12965906
rs72702556
rs1201943
All
−0.10
−0.05
0.00
0.05
MR leave−one−out sensitivity analysis for
'CD33dim HLA DR+ CD11b− AC' on 'Juvenile arthritis'

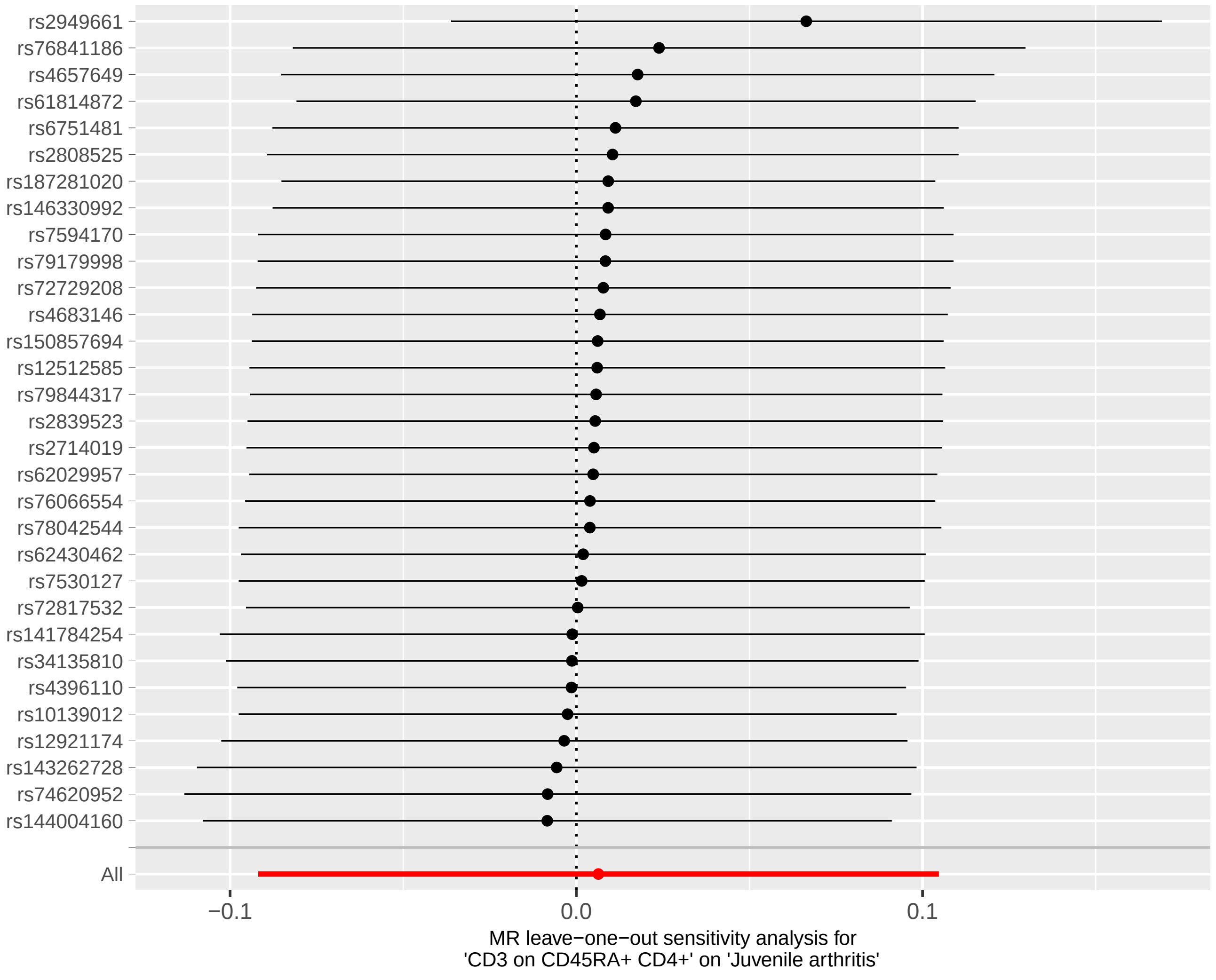
rs2949661
rs76841186
rs4657649
rs61814872
rs6751481
rs2808525
rs187281020
rs146330992
rs7594170
rs79179998
rs72729208
rs4683146
rs150857694
rs12512585
rs79844317
rs2839523
rs2714019
rs62029957
rs76066554
rs78042544
rs62430462
rs7530127
rs72817532
rs141784254
rs34135810
rs4396110
rs10139012
rs12921174
rs143262728
rs74620952
rs144004160
All
−0.1
0.0
0.1
MR leave−one−out sensitivity analysis for
'CD3 on CD45RA+ CD4+' on 'Juvenile arthritis'

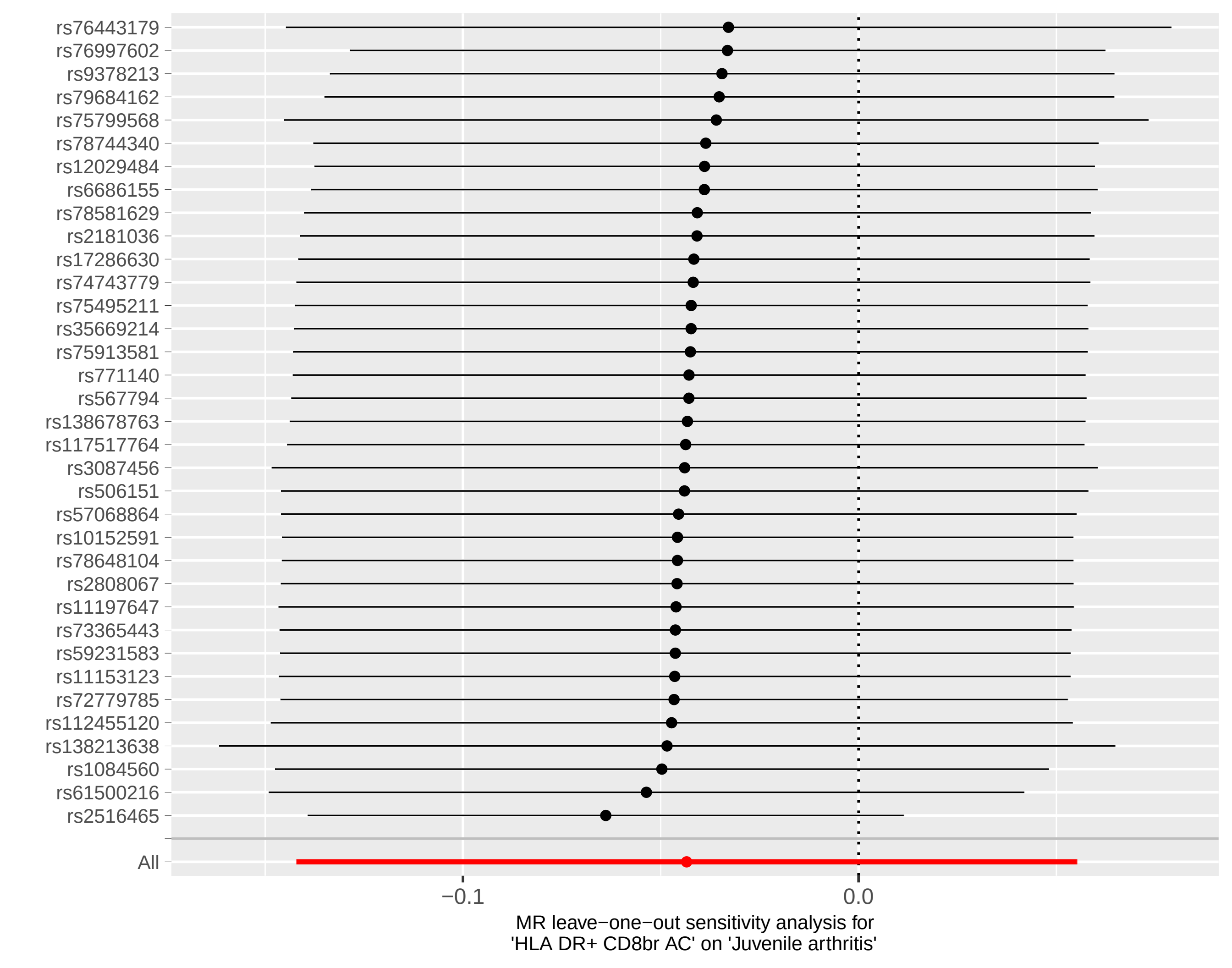
rs76443179
rs76997602
rs9378213
rs79684162
rs75799568
rs78744340
rs12029484
rs6686155
rs78581629
rs2181036
rs17286630
rs74743779
rs75495211
rs35669214
rs75913581
rs771140
rs567794
rs138678763
rs117517764
rs3087456
rs506151
rs57068864
rs10152591
rs78648104
rs2808067
rs11197647
rs73365443
rs59231583
rs11153123
rs72779785
rs112455120
rs138213638
rs1084560
rs61500216
rs2516465
All
−0.1
0.0
MR leave−one−out sensitivity analysis for
'HLA DR+ CD8br AC' on 'Juvenile arthritis'

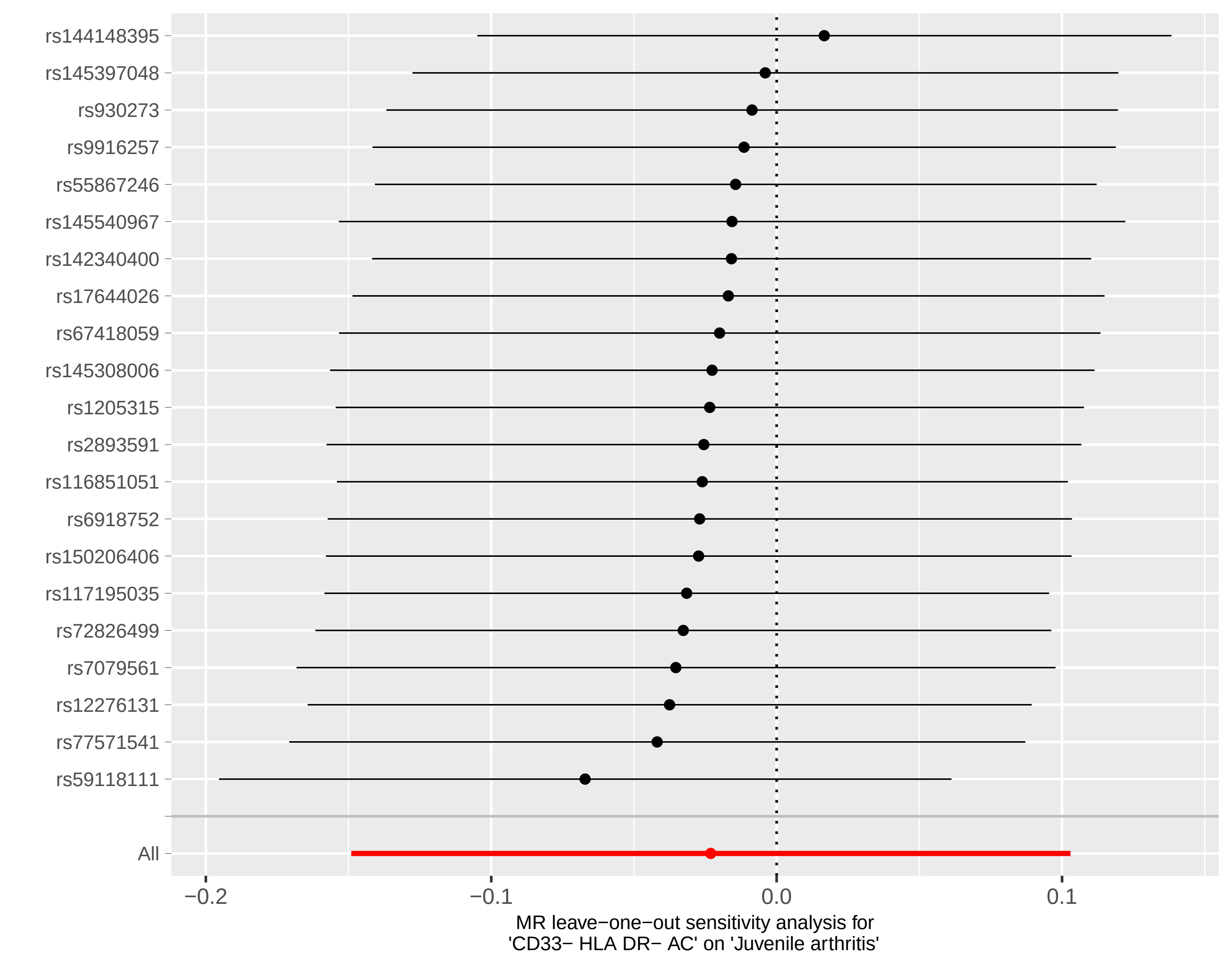
rs144148395
rs145397048
rs930273
rs9916257
rs55867246
rs145540967
rs142340400
rs17644026
rs67418059
rs145308006
rs1205315
rs2893591
rs116851051
rs6918752
rs150206406
rs117195035
rs72826499
rs7079561
rs12276131
rs77571541
rs59118111
All
−0.2
−0.1
0.0
0.1
MR leave−one−out sensitivity analysis for
'CD33− HLA DR− AC' on 'Juvenile arthritis'

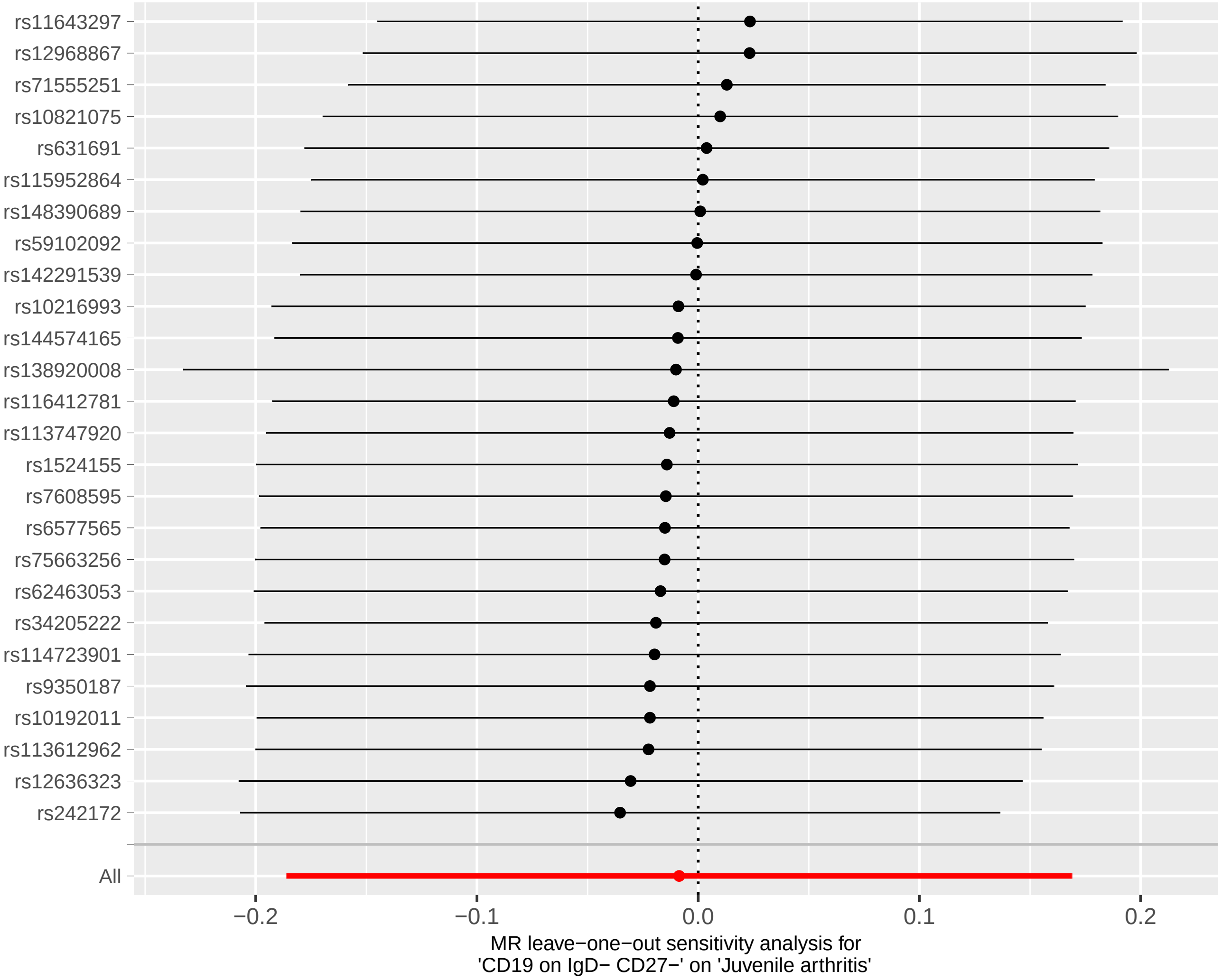
rs11643297
rs12968867
rs71555251
rs10821075
rs631691
rs115952864
rs148390689
rs59102092
rs142291539
rs10216993
rs144574165
rs138920008
rs116412781
rs113747920
rs1524155
rs7608595
rs6577565
rs75663256
rs62463053
rs34205222
rs114723901
rs9350187
rs10192011
rs113612962
rs12636323
rs242172
All
−0.2
−0.1
0.0
0.1
0.2
MR leave−one−out sensitivity analysis for
'CD19 on IgD− CD27−' on 'Juvenile arthritis'

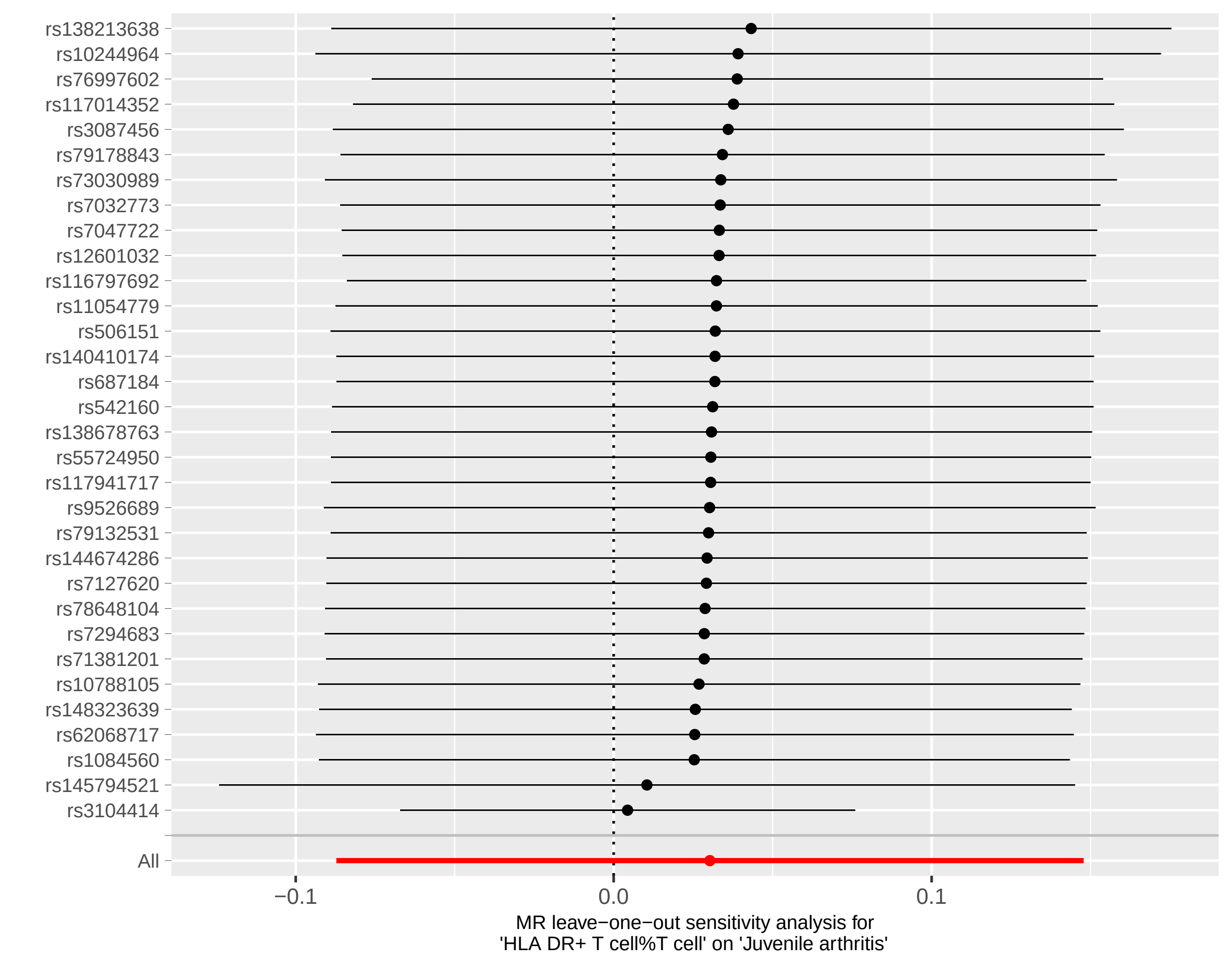

rs138213638
rs10244964
rs76997602
rs117014352
rs3087456
rs79178843
rs73030989
rs7032773
rs7047722
rs12601032
rs116797692
rs11054779
rs506151
rs140410174
rs687184
rs542160
rs138678763
rs55724950
rs117941717
rs9526689
rs79132531
rs144674286
rs7127620
rs78648104
rs7294683
rs71381201
rs10788105
rs148323639
rs62068717
rs1084560
rs145794521
rs3104414
All
−0.1
0.0
0.1
MR leave−one−out sensitivity analysis for
'HLA DR+ T cell%T cell' on 'Juvenile arthritis'

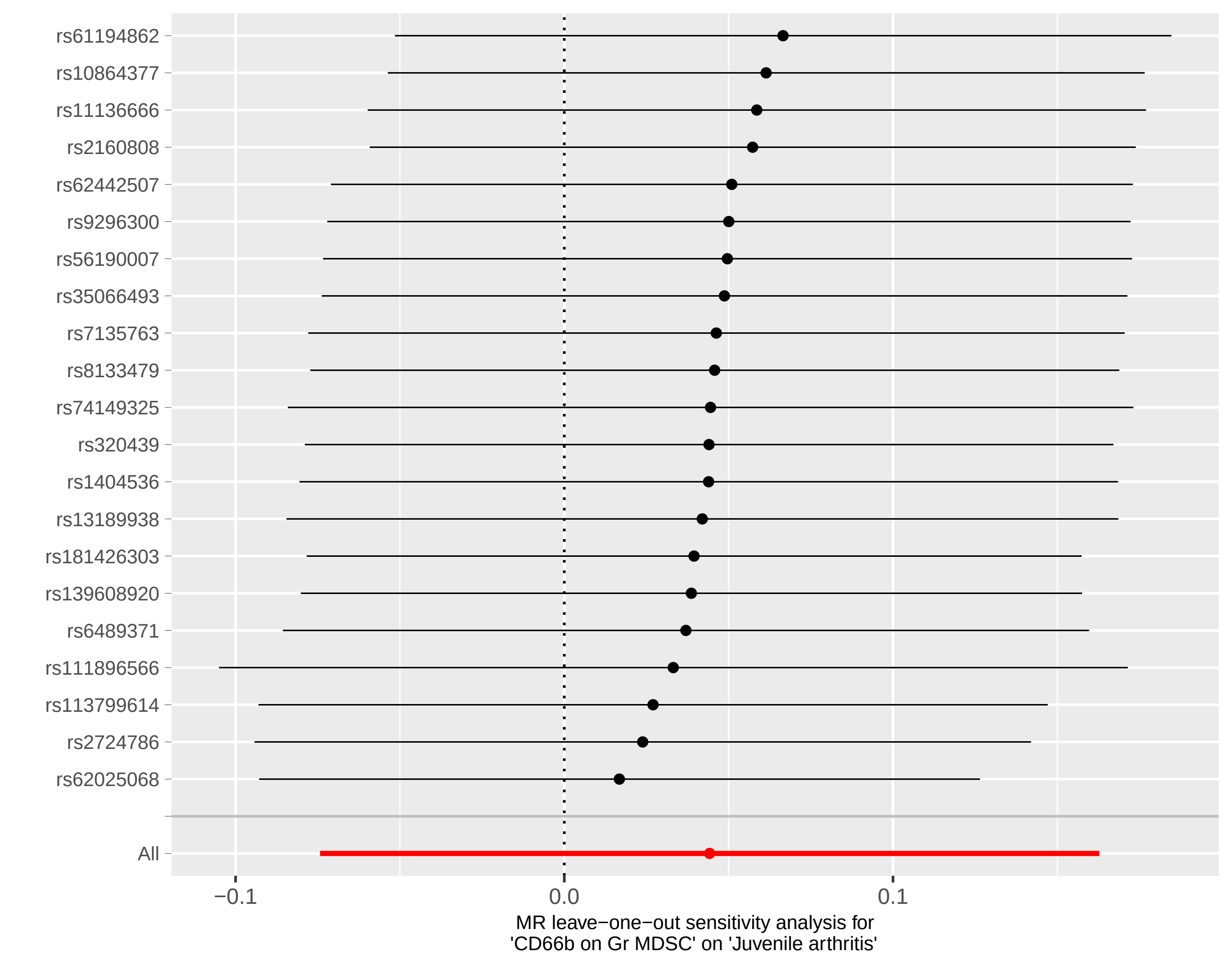
rs61194862
rs10864377
rs11136666
rs2160808
rs62442507
rs9296300
rs56190007
rs35066493
rs7135763
rs8133479
rs74149325
rs320439
rs1404536
rs13189938
rs181426303
rs139608920
rs6489371
rs111896566
rs113799614
rs2724786
rs62025068
All
−0.1
0.0
0.1
MR leave−one−out sensitivity analysis for
'CD66b on Gr MDSC' on 'Juvenile arthritis'

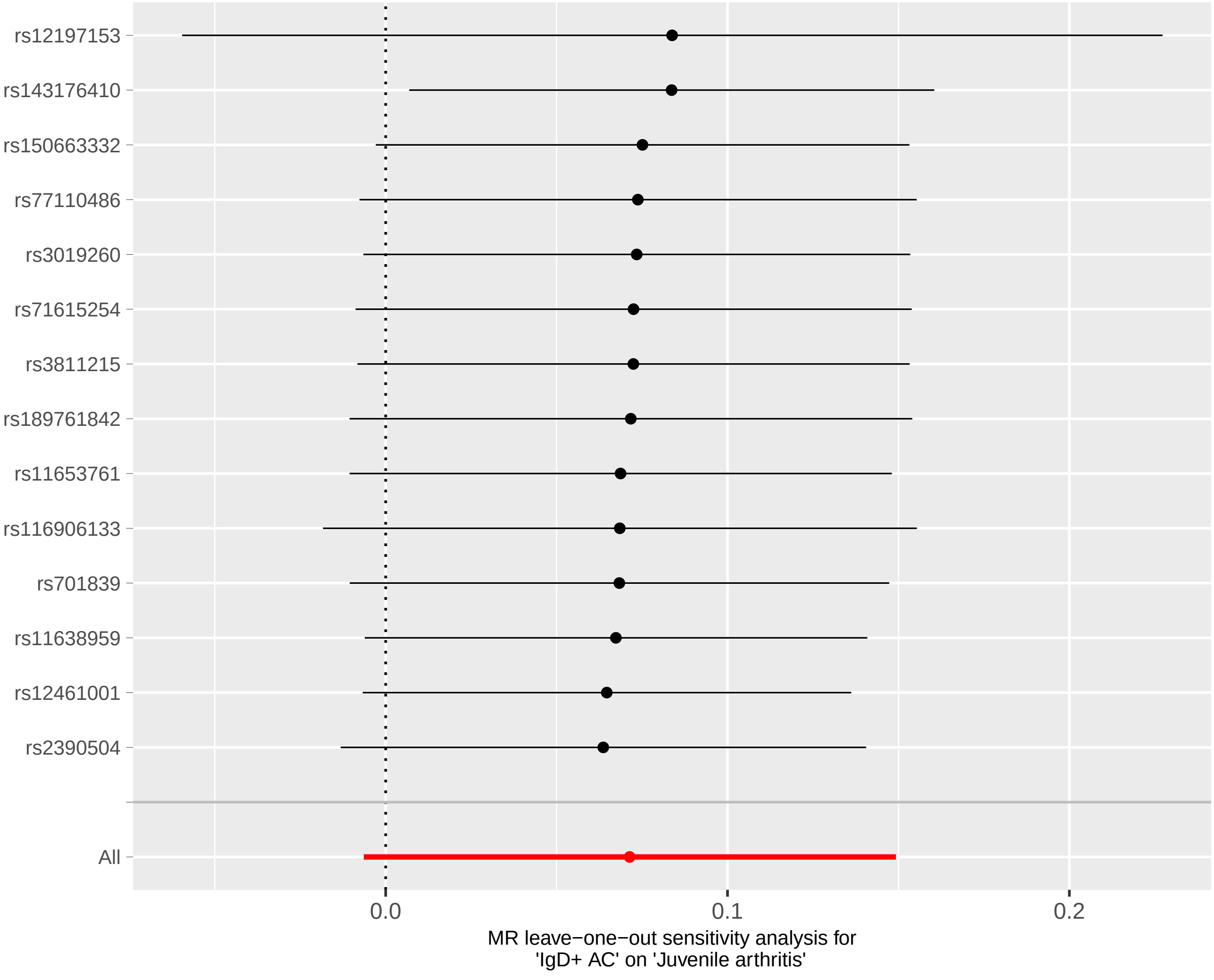

rs12197153
rs143176410
rs150663332
rs77110486
rs3019260
rs71615254
rs3811215
rs189761842
rs11653761
rs116906133
rs701839
rs11638959
rs12461001
rs2390504
All
0.0
0.1
0.2
MR leave−one−out sensitivity analysis for
'IgD+ AC' on 'Juvenile arthritis'

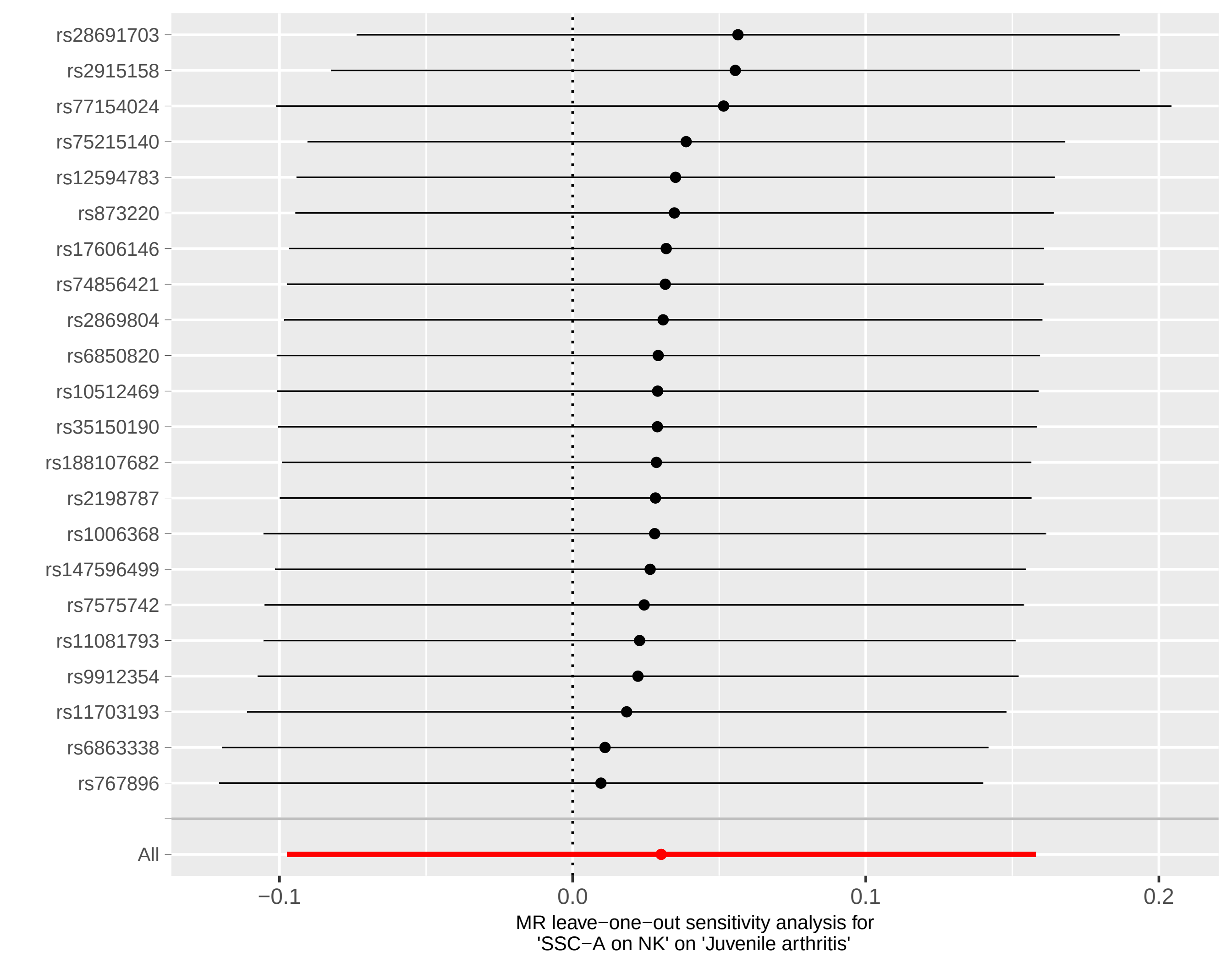
rs28691703
rs2915158
rs77154024
rs75215140
rs12594783
rs873220
rs17606146
rs74856421
rs2869804
rs6850820
rs10512469
rs35150190
rs188107682
rs2198787
rs1006368
rs147596499
rs7575742
rs11081793
rs9912354
rs11703193
rs6863338
rs767896
All
−0.1
0.0
0.1
0.2
MR leave−one−out sensitivity analysis for
'SSC−A on NK' on 'Juvenile arthritis'

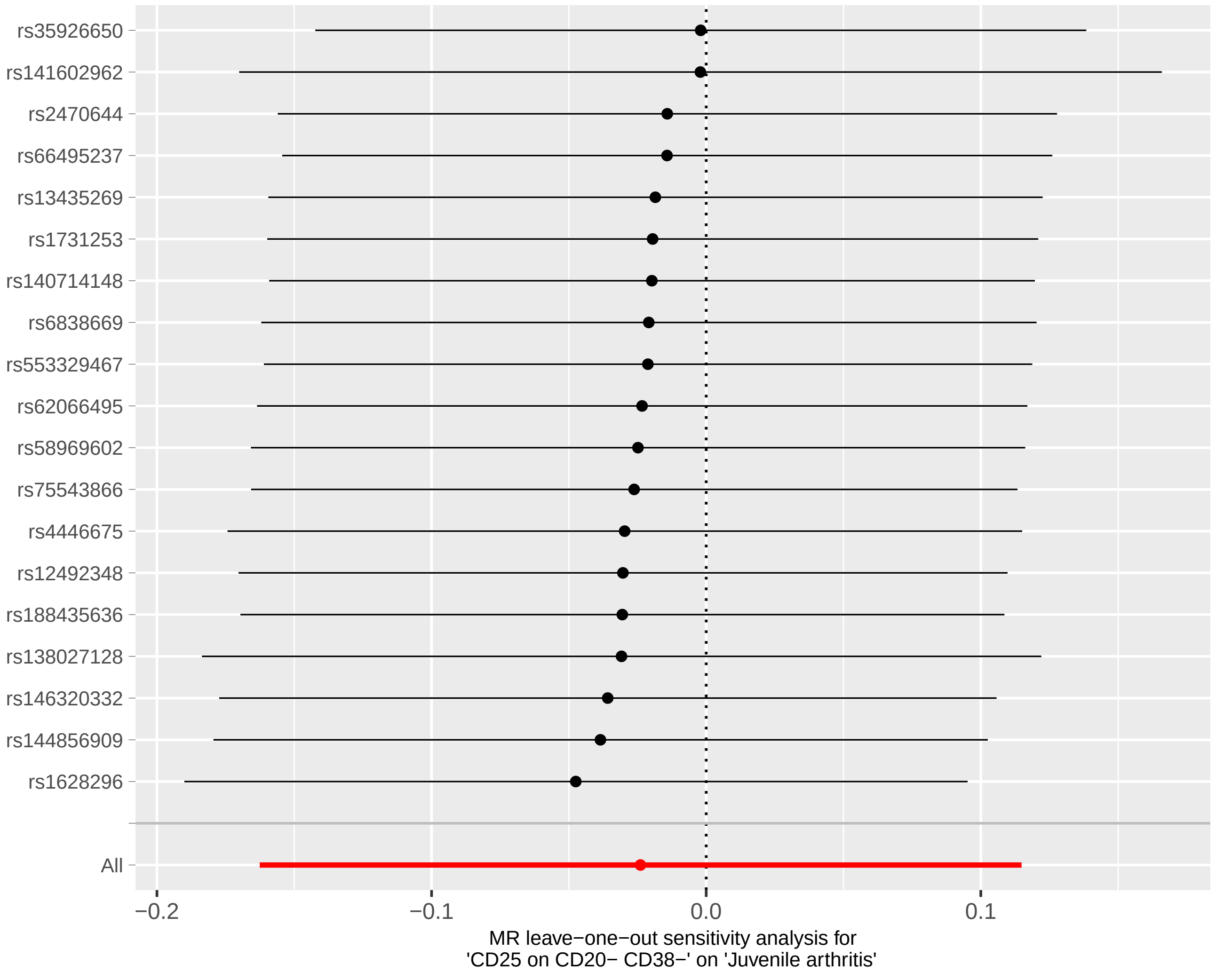
rs35926650
rs141602962
rs2470644
rs66495237
rs13435269
rs1731253
rs140714148
rs6838669
rs553329467
rs62066495
rs58969602
rs75543866
rs4446675
rs12492348
rs188435636
rs138027128
rs146320332
rs144856909
rs1628296
All
−0.2
−0.1
0.0
0.1
MR leave−one−out sensitivity analysis for
'CD25 on CD20− CD38−' on 'Juvenile arthritis'

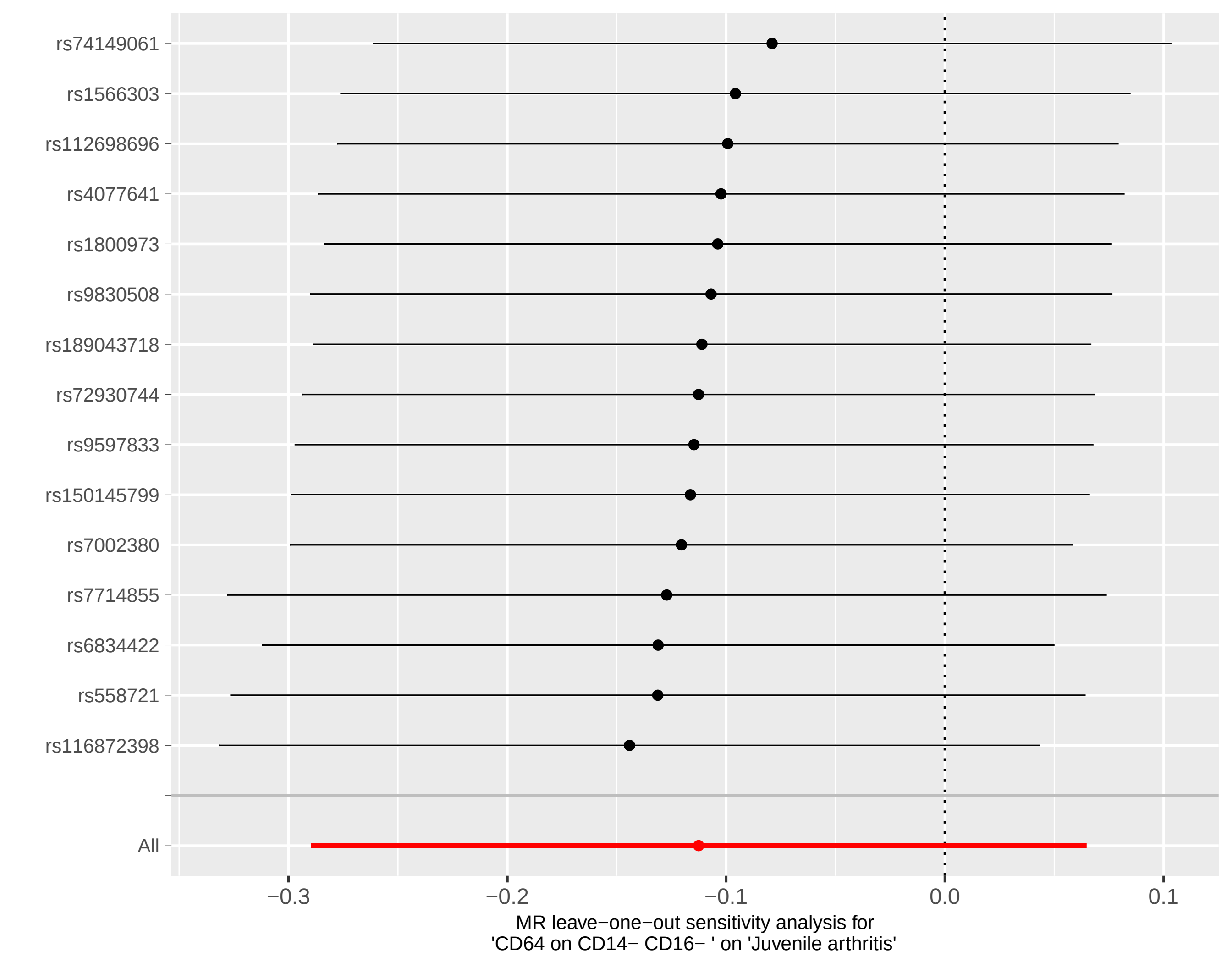

rs74149061
rs1566303
rs112698696
rs4077641
rs1800973
rs9830508
rs189043718
rs72930744
rs9597833
rs150145799
rs7002380
rs7714855
rs6834422
rs558721
rs116872398
All
−0.3
−0.2
−0.1
0.0
0.1
MR leave−one−out sensitivity analysis for
'CD64 on CD14− CD16− ' on 'Juvenile arthritis'

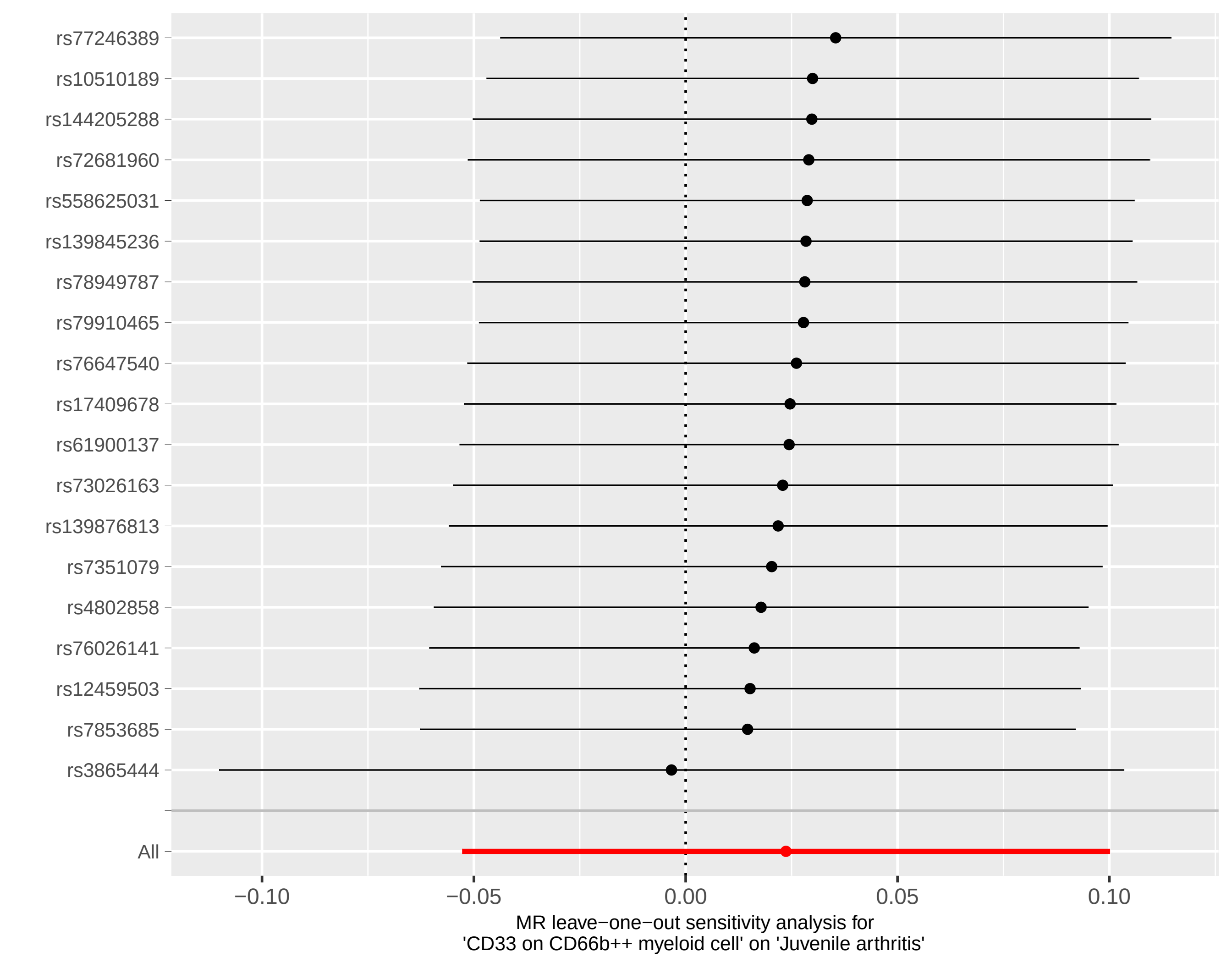
rs77246389
rs10510189
rs144205288
rs72681960
rs558625031
rs139845236
rs78949787
rs79910465
rs76647540
rs17409678
rs61900137
rs73026163
rs139876813
rs7351079
rs4802858
rs76026141
rs12459503
rs7853685
rs3865444
All
−0.10
−0.05
0.00
0.05
0.10
MR leave−one−out sensitivity analysis for
'CD33 on CD66b++ myeloid cell' on 'Juvenile arthritis'

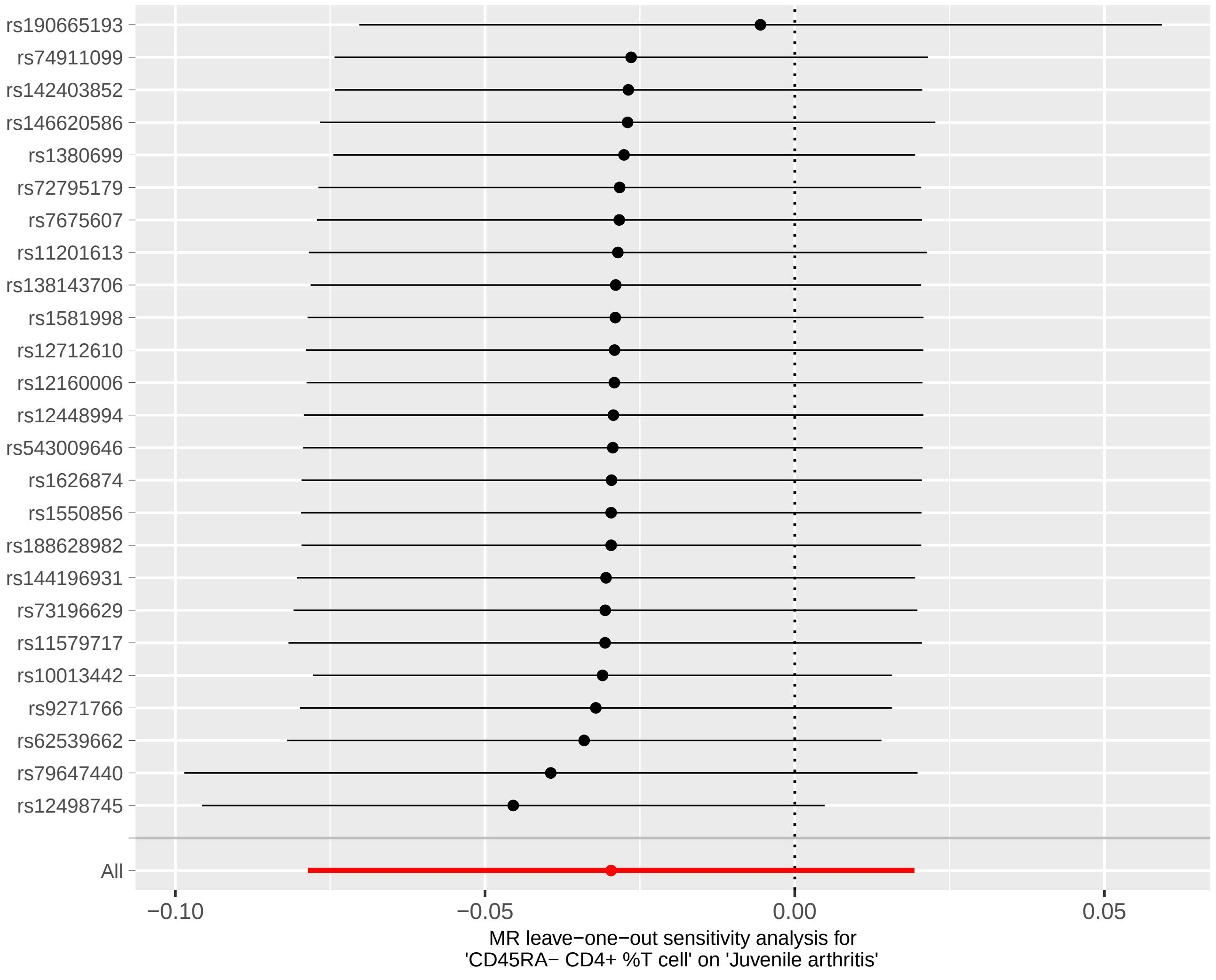
rs190665193
rs74911099
rs142403852
rs146620586
rs1380699
rs72795179
rs7675607
rs11201613
rs138143706
rs1581998
rs12712610
rs12160006
rs12448994
rs543009646
rs1626874
rs1550856
rs188628982
rs144196931
rs73196629
rs11579717
rs10013442
rs9271766
rs62539662
rs79647440
rs12498745
All
−0.10
−0.05
0.00
0.05
MR leave−one−out sensitivity analysis for
'CD45RA− CD4+ %T cell' on 'Juvenile arthritis'

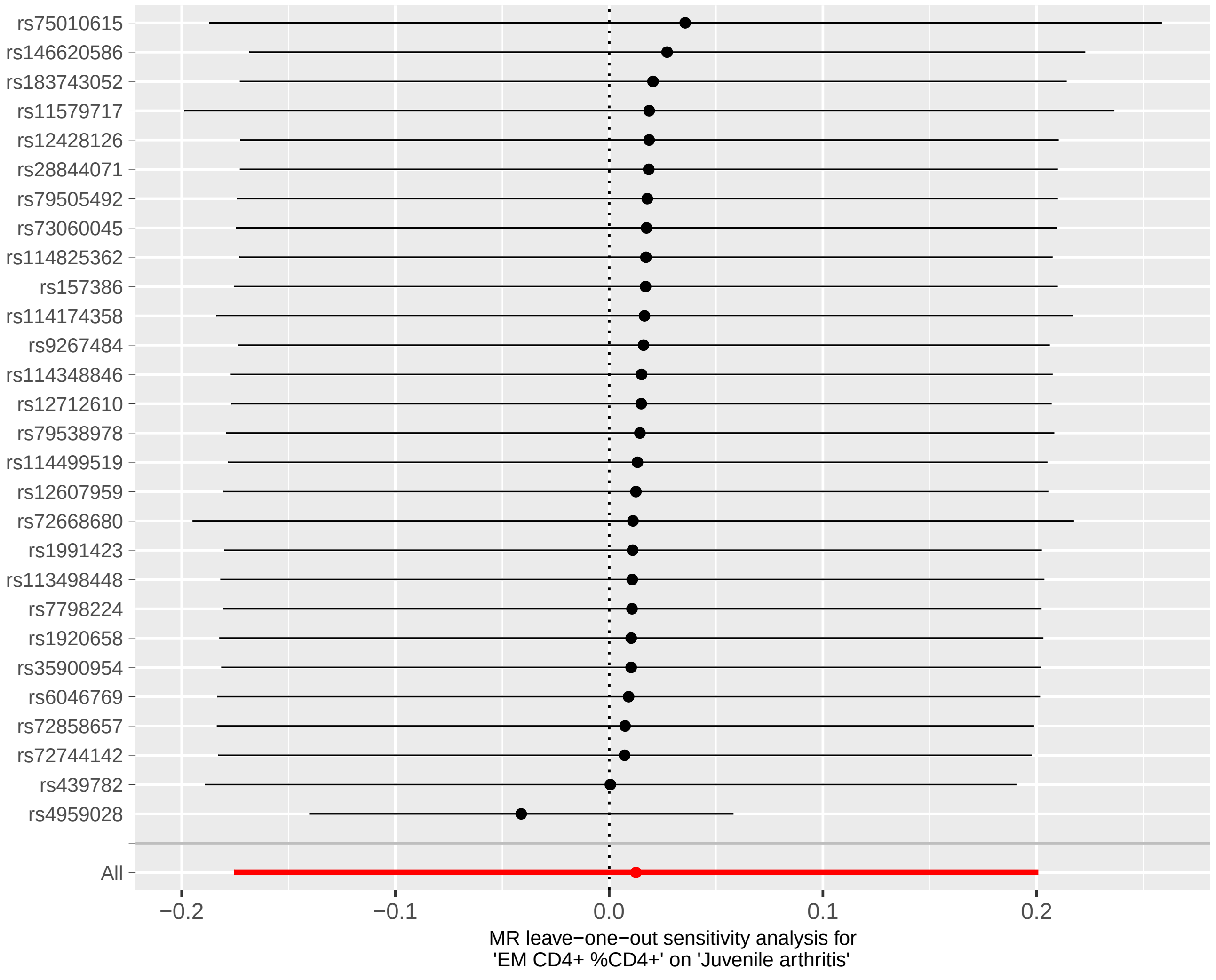
rs75010615
rs146620586
rs183743052
rs11579717
rs12428126
rs28844071
rs79505492
rs73060045
rs114825362
rs157386
rs114174358
rs9267484
rs114348846
rs12712610
rs79538978
rs114499519
rs12607959
rs72668680
rs1991423
rs113498448
rs7798224
rs1920658
rs35900954
rs6046769
rs72858657
rs72744142
rs439782
rs4959028
All
−0.2
−0.1
0.0
0.1
0.2
MR leave−one−out sensitivity analysis for
'EM CD4+ %CD4+' on 'Juvenile arthritis'

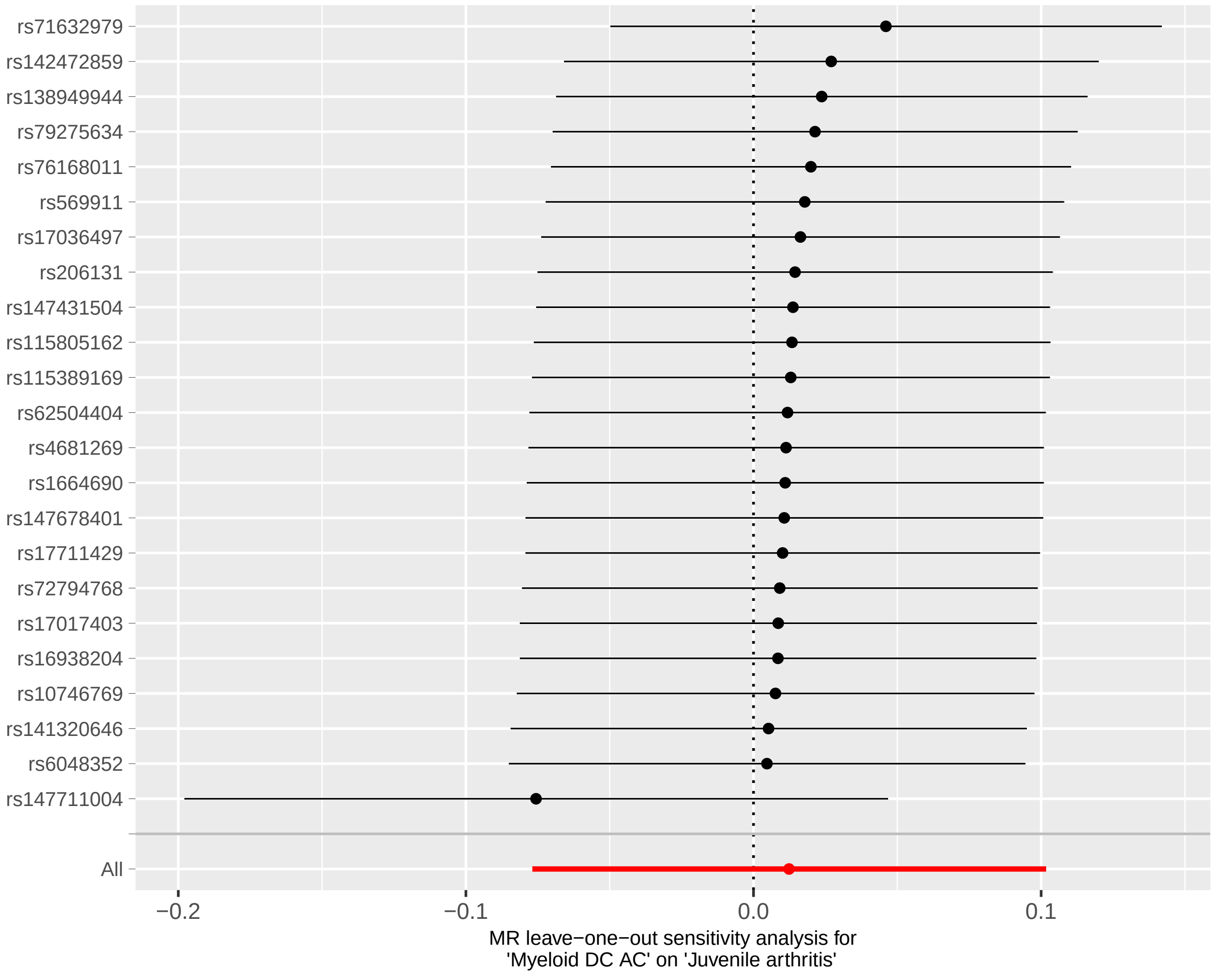
rs71632979
rs142472859
rs138949944
rs79275634
rs76168011
rs569911
rs17036497
rs206131
rs147431504
rs115805162
rs115389169
rs62504404
rs4681269
rs1664690
rs147678401
rs17711429
rs72794768
rs17017403
rs16938204
rs10746769
rs141320646
rs6048352
rs147711004
All
−0.2
−0.1
0.0
0.1
MR leave−one−out sensitivity analysis for
'Myeloid DC AC' on 'Juvenile arthritis'

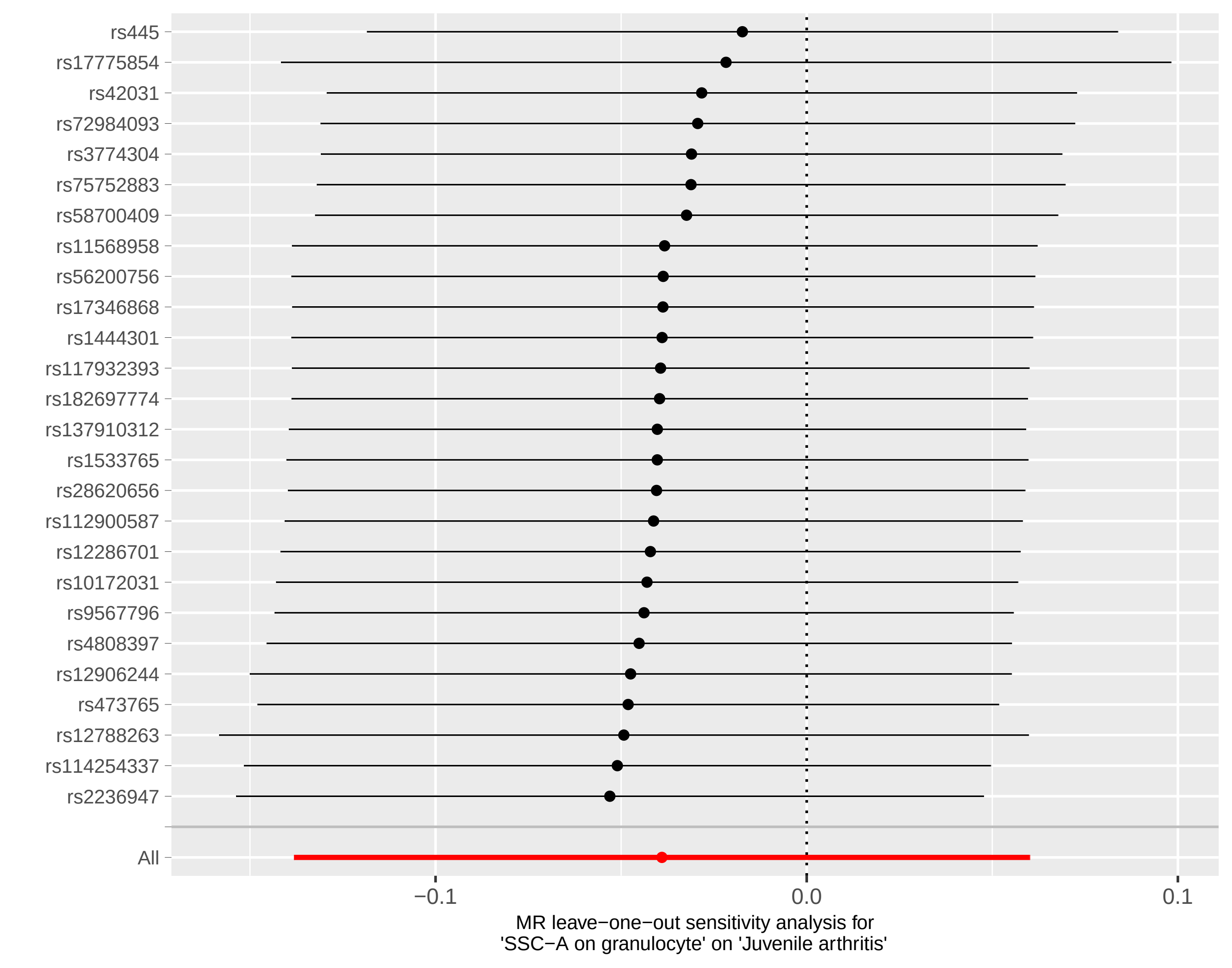

rs445
rs17775854
rs42031
rs72984093
rs3774304
rs75752883
rs58700409
rs11568958
rs56200756
rs17346868
rs1444301
rs117932393
rs182697774
rs137910312
rs1533765
rs28620656
rs112900587
rs12286701
rs10172031
rs9567796
rs4808397
rs12906244
rs473765
rs12788263
rs114254337
rs2236947
All
−0.1
0.0
0.1
MR leave−one−out sensitivity analysis for
'SSC−A on granulocyte' on 'Juvenile arthritis'

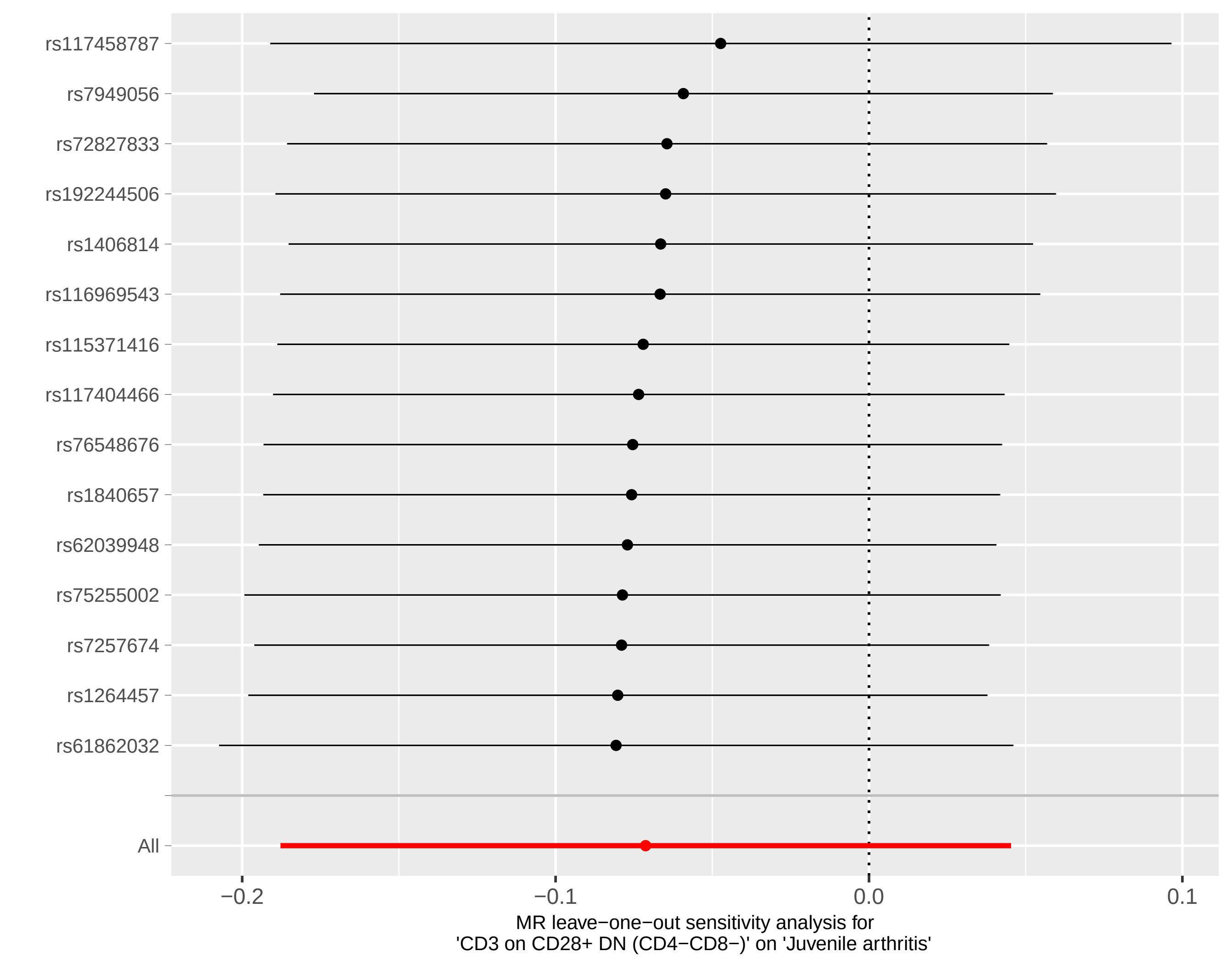
rs117458787
rs7949056
rs72827833
rs192244506
rs1406814
rs116969543
rs115371416
rs117404466
rs76548676
rs1840657
rs62039948
rs75255002
rs7257674
rs1264457
rs61862032
All
−0.2
−0.1
0.0
0.1
MR leave−one−out sensitivity analysis for
'CD3 on CD28+ DN (CD4−CD8−)' on 'Juvenile arthritis'

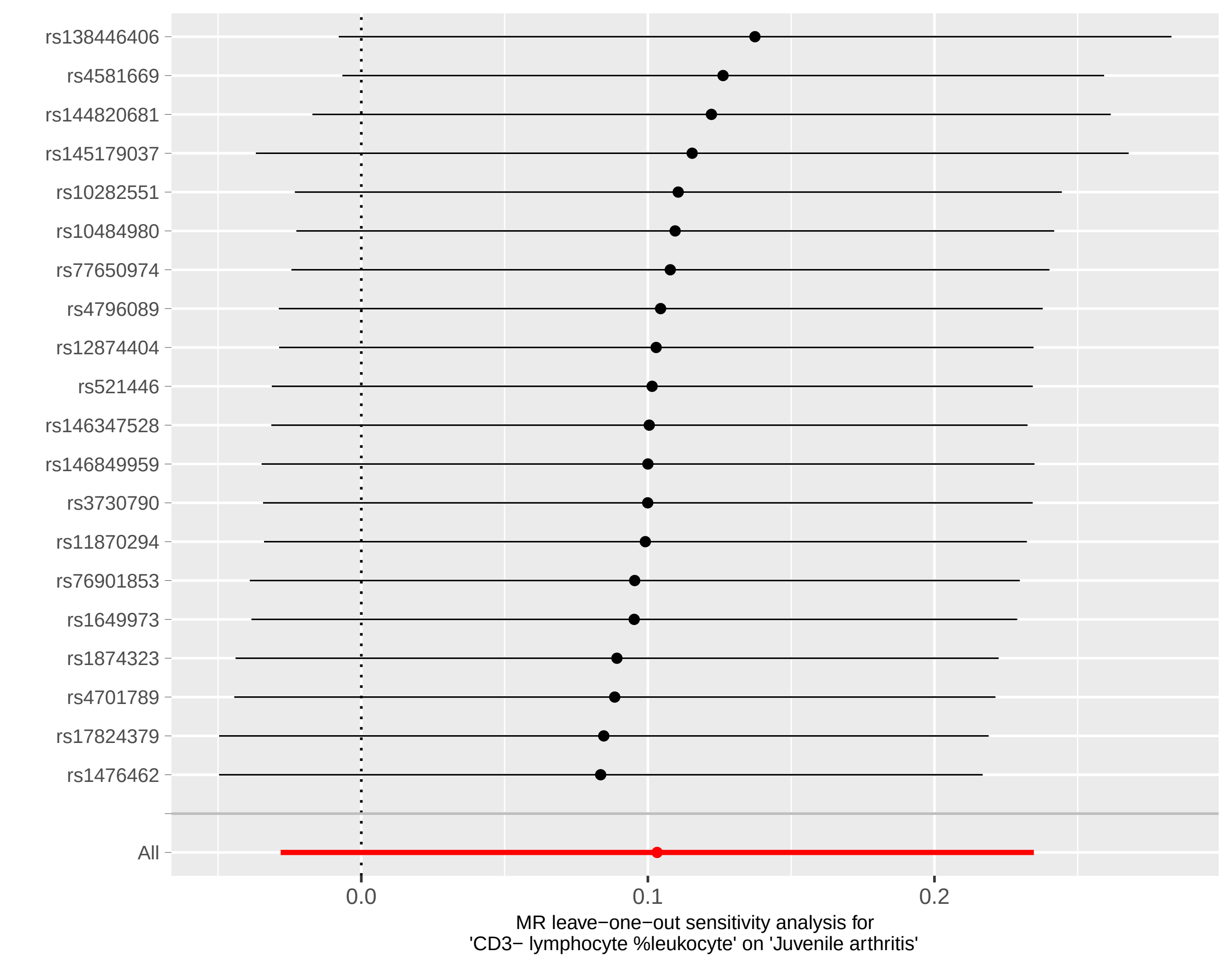
rs138446406
rs4581669
rs144820681
rs145179037
rs10282551
rs10484980
rs77650974
rs4796089
rs12874404
rs521446
rs146347528
rs146849959
rs3730790
rs11870294
rs76901853
rs1649973
rs1874323
rs4701789
rs17824379
rs1476462
All
0.0
0.1
0.2
MR leave−one−out sensitivity analysis for
'CD3− lymphocyte %leukocyte' on 'Juvenile arthritis'

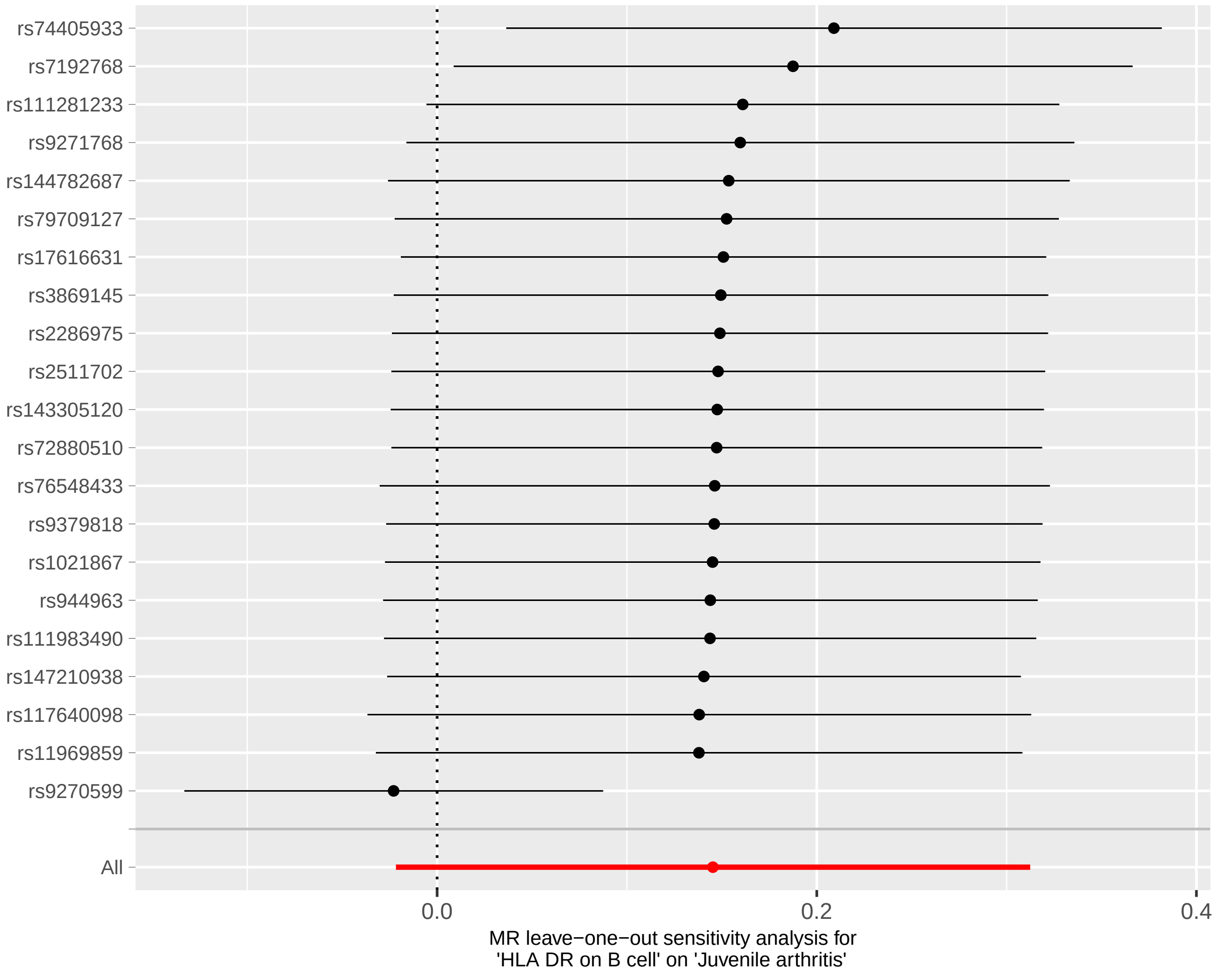
rs74405933
rs7192768
rs111281233
rs9271768
rs144782687
rs79709127
rs17616631
rs3869145
rs2286975
rs2511702
rs143305120
rs72880510
rs76548433
rs9379818
rs1021867
rs944963
rs111983490
rs147210938
rs117640098
rs11969859
rs9270599
All
0.0
0.2
0.4
MR leave−one−out sensitivity analysis for
'HLA DR on B cell' on 'Juvenile arthritis'

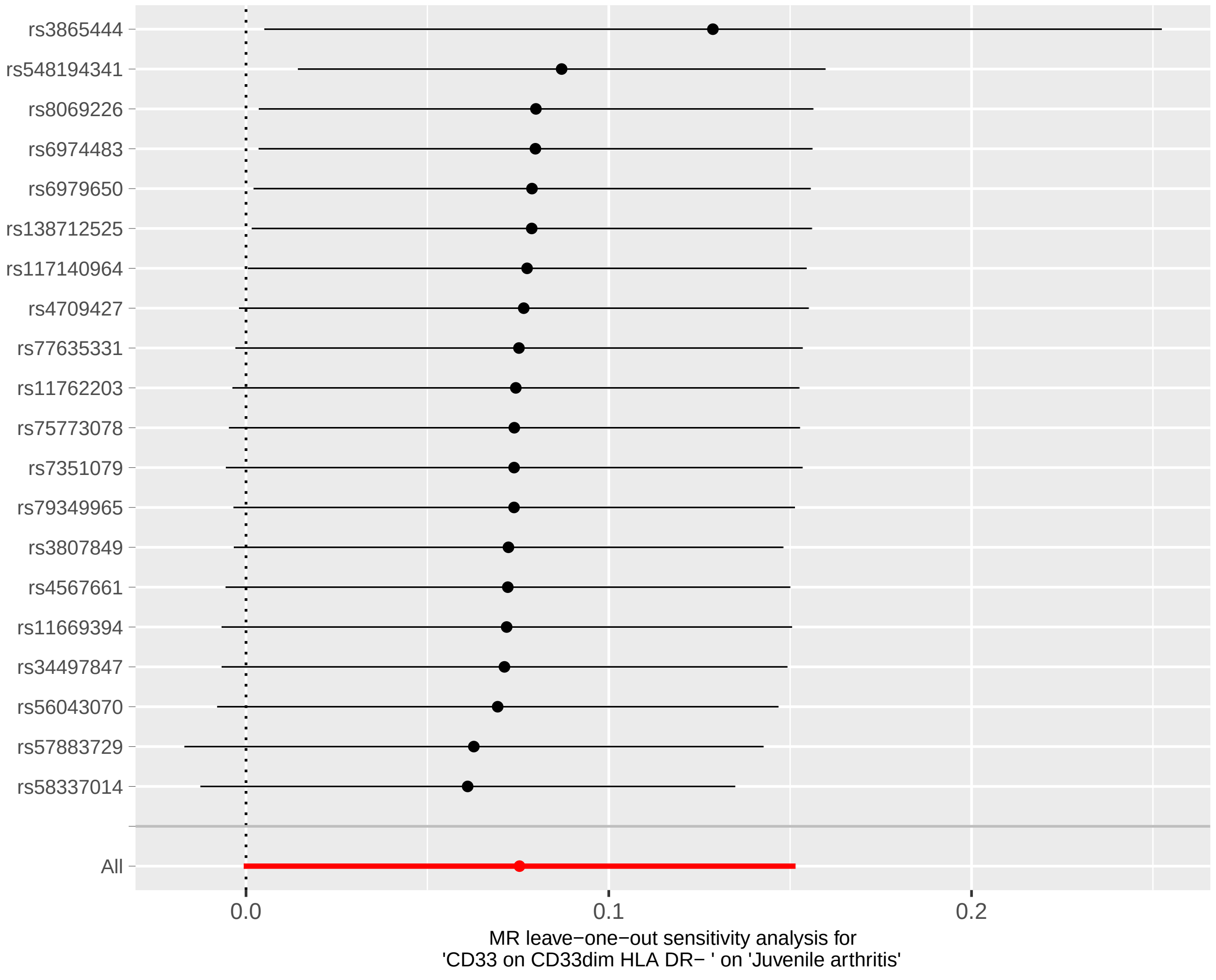

rs3865444
rs548194341
rs8069226
rs6974483
rs6979650
rs138712525
rs117140964
rs4709427
rs77635331
rs11762203
rs75773078
rs7351079
rs79349965
rs3807849
rs4567661
rs11669394
rs34497847
rs56043070
rs57883729
rs58337014
All
0.0
0.1
0.2
MR leave−one−out sensitivity analysis for
'CD33 on CD33dim HLA DR− ' on 'Juvenile arthritis'

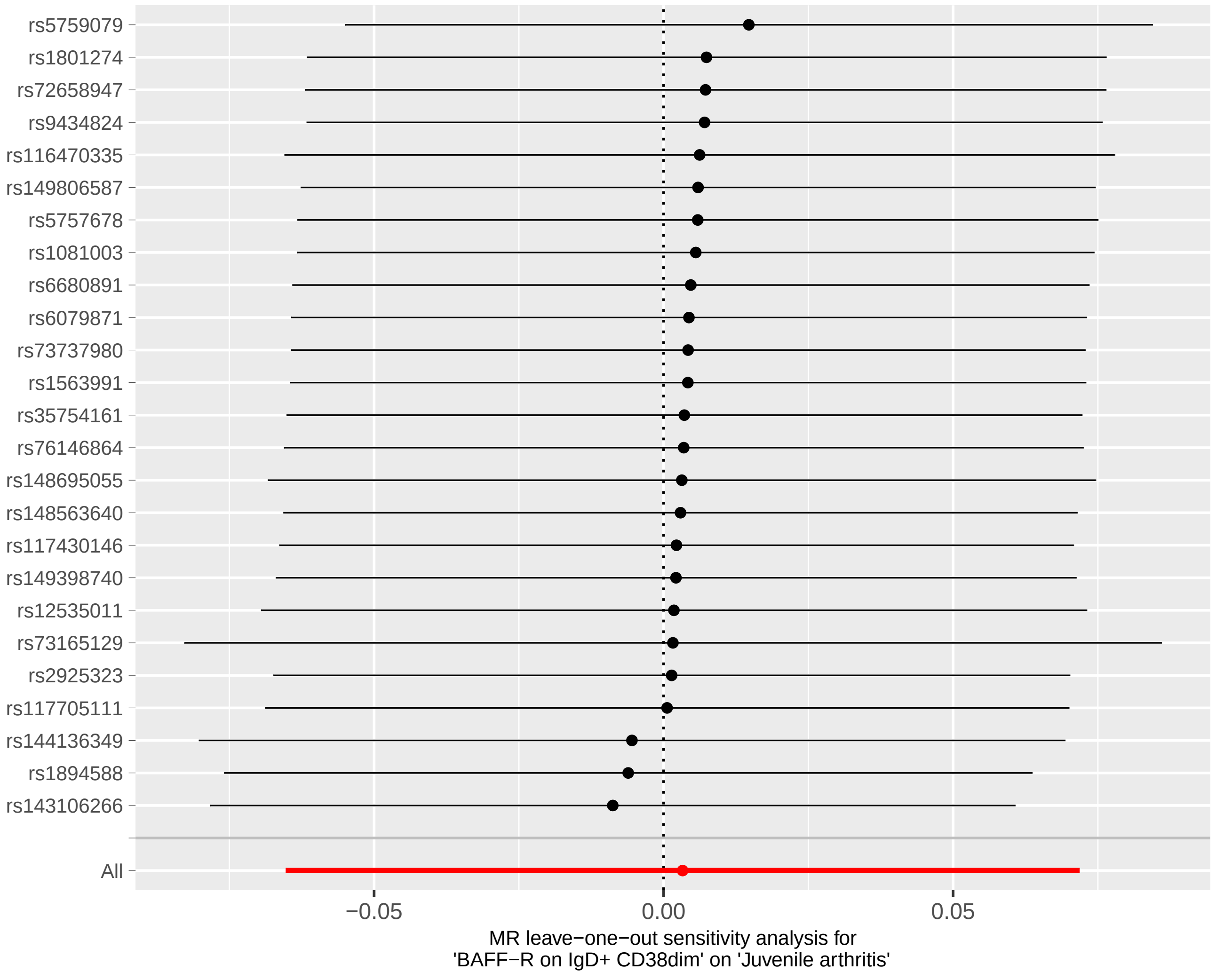

rs5759079
rs1801274
rs72658947
rs9434824
rs116470335
rs149806587
rs5757678
rs1081003
rs6680891
rs6079871
rs73737980
rs1563991
rs35754161
rs76146864
rs148695055
rs148563640
rs117430146
rs149398740
rs12535011
rs73165129
rs2925323
rs117705111
rs144136349
rs1894588
rs143106266
All
−0.05
0.00
0.05
MR leave−one−out sensitivity analysis for
'BAFF−R on IgD+ CD38dim' on 'Juvenile arthritis'

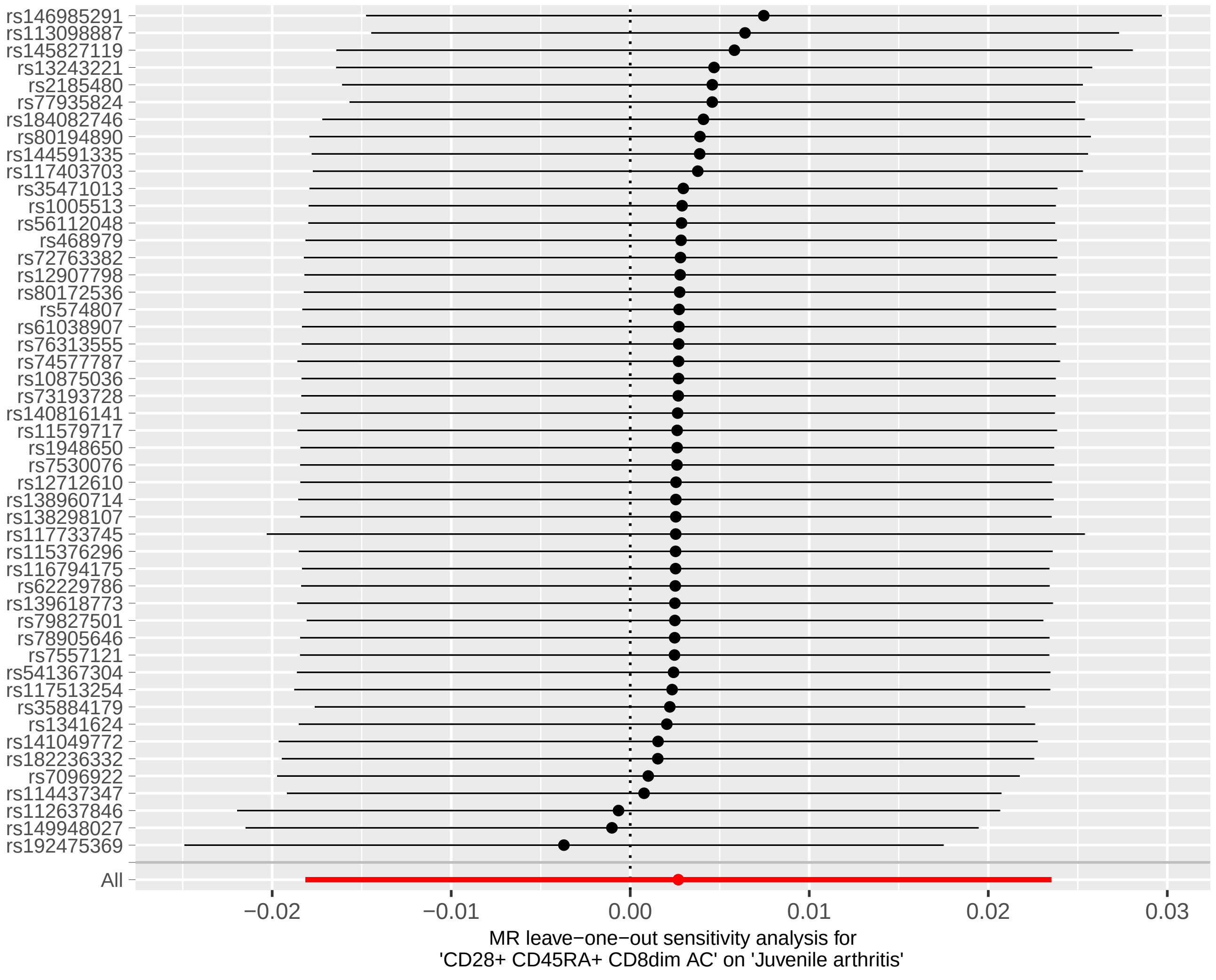
rs146985291
rs113098887
rs145827119
rs13243221
rs2185480
rs77935824
rs184082746
rs80194890
rs144591335
rs117403703
rs35471013
rs1005513
rs56112048
rs468979
rs72763382
rs12907798
rs80172536
rs574807
rs61038907
rs76313555
rs74577787
rs10875036
rs73193728
rs140816141
rs11579717
rs1948650
rs7530076
rs12712610
rs138960714
rs138298107
rs117733745
rs115376296
rs116794175
rs62229786
rs139618773
rs79827501
rs78905646
rs7557121
rs541367304
rs117513254
rs35884179
rs1341624
rs141049772
rs182236332
rs7096922
rs114437347
rs112637846
rs149948027
rs192475369
All
−0.02
−0.01
0.00
0.01
0.02
0.03
MR leave−one−out sensitivity analysis for
'CD28+ CD45RA+ CD8dim AC' on 'Juvenile arthritis'

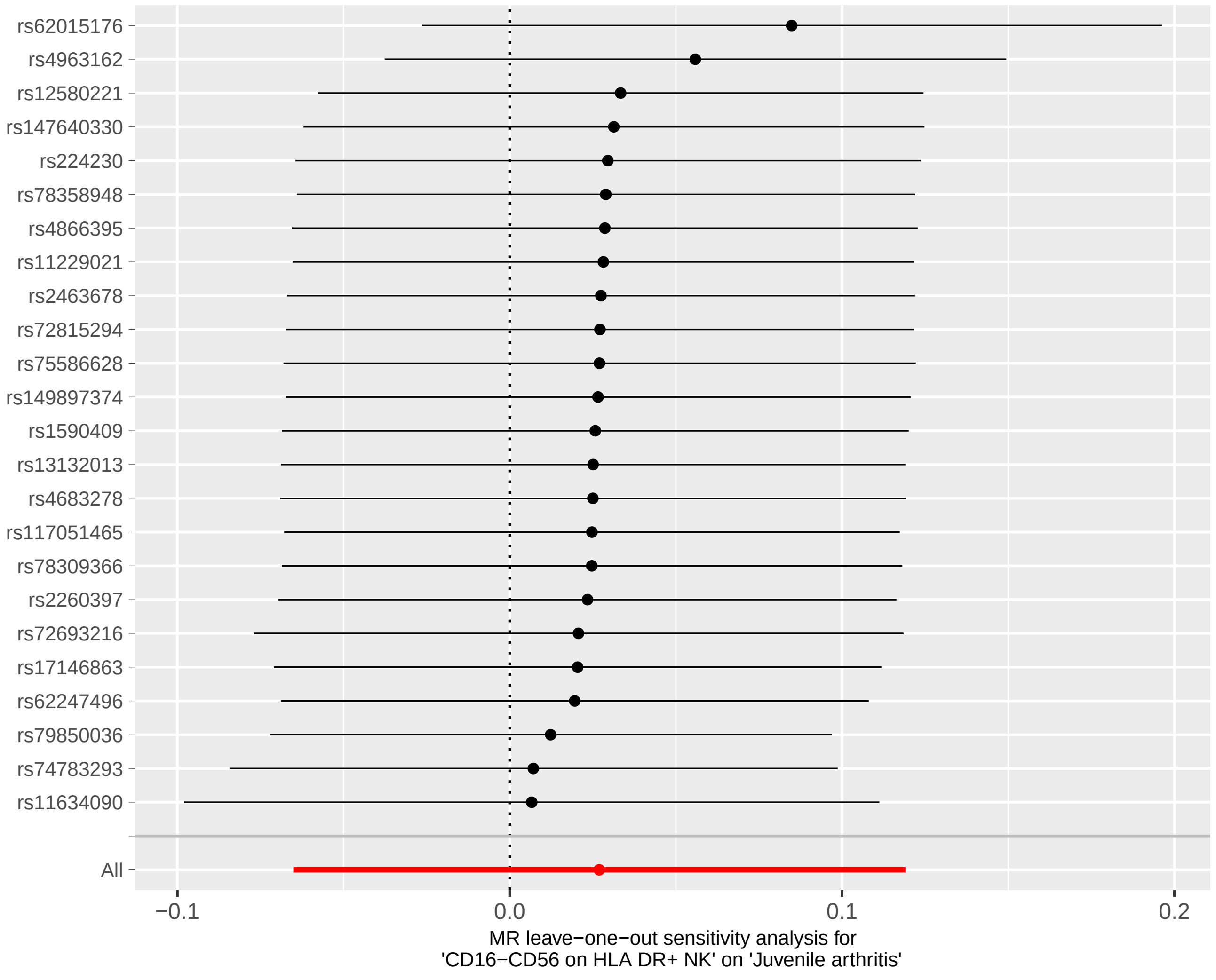
rs62015176
rs4963162
rs12580221
rs147640330
rs224230
rs78358948
rs4866395
rs11229021
rs2463678
rs72815294
rs75586628
rs149897374
rs1590409
rs13132013
rs4683278
rs117051465
rs78309366
rs2260397
rs72693216
rs17146863
rs62247496
rs79850036
rs74783293
rs11634090
All
−0.1
0.0
0.1
0.2
MR leave−one−out sensitivity analysis for
'CD16−CD56 on HLA DR+ NK' on 'Juvenile arthritis'

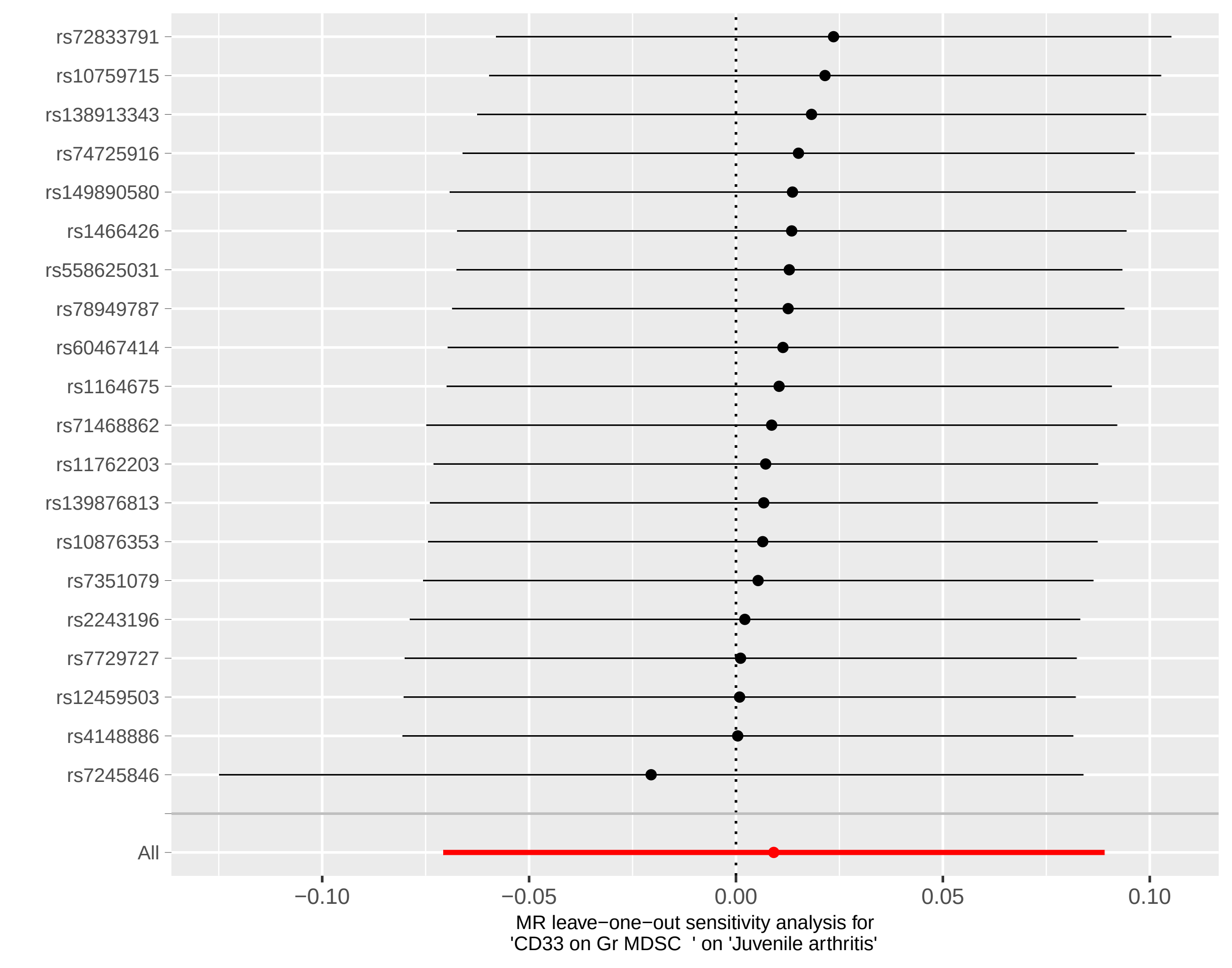

rs72833791
rs10759715
rs138913343
rs74725916
rs149890580
rs1466426
rs558625031
rs78949787
rs60467414
rs1164675
rs71468862
rs11762203
rs139876813
rs10876353
rs7351079
rs2243196
rs7729727
rs12459503
rs4148886
rs7245846
All
−0.10
−0.05
0.00
0.05
0.10
MR leave−one−out sensitivity analysis for
'CD33 on Gr MDSC ' on 'Juvenile arthritis'

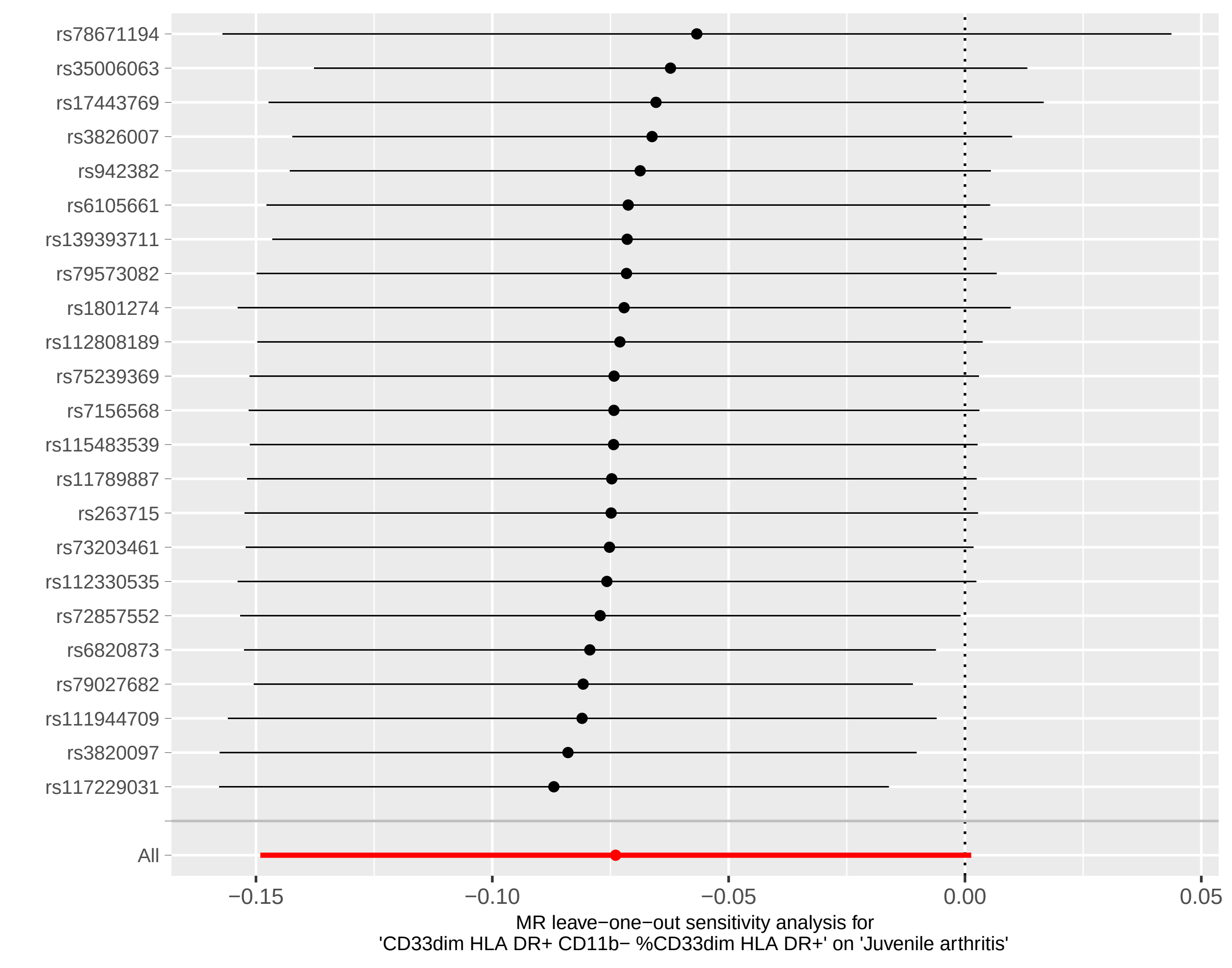

rs78671194
rs35006063
rs17443769
rs3826007
rs942382
rs6105661
rs139393711
rs79573082
rs1801274
rs112808189
rs75239369
rs7156568
rs115483539
rs11789887
rs263715
rs73203461
rs112330535
rs72857552
rs6820873
rs79027682
rs111944709
rs3820097
rs117229031
All
−0.15
−0.10
−0.05
0.00
0.05
MR leave−one−out sensitivity analysis for
'CD33dim HLA DR+ CD11b− %CD33dim HLA DR+' on 'Juvenile arthritis'

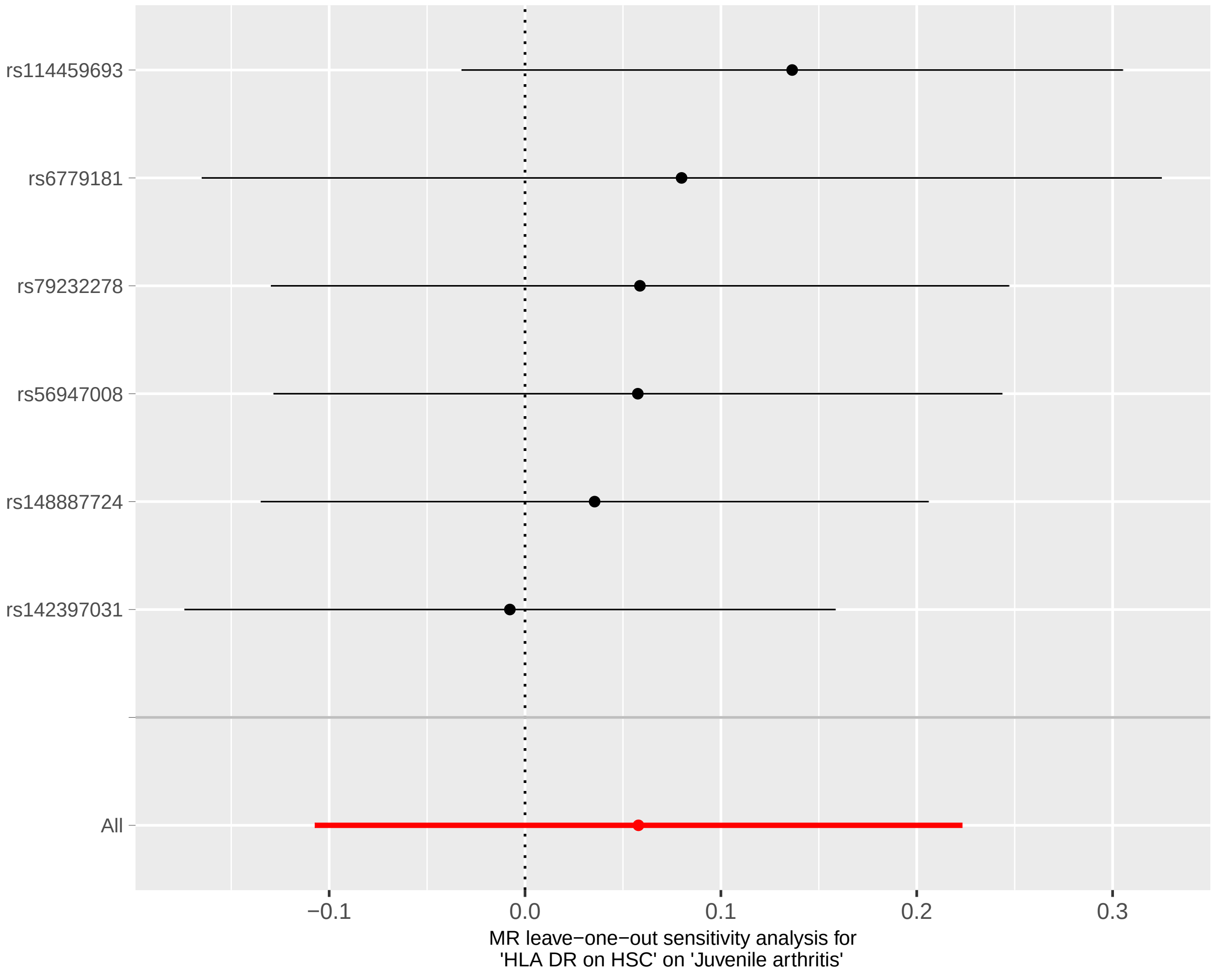
rs114459693
rs6779181
rs79232278
rs56947008
rs148887724
rs142397031
All
−0.1
0.0
0.1
0.2
0.3
MR leave−one−out sensitivity analysis for
'HLA DR on HSC' on 'Juvenile arthritis'

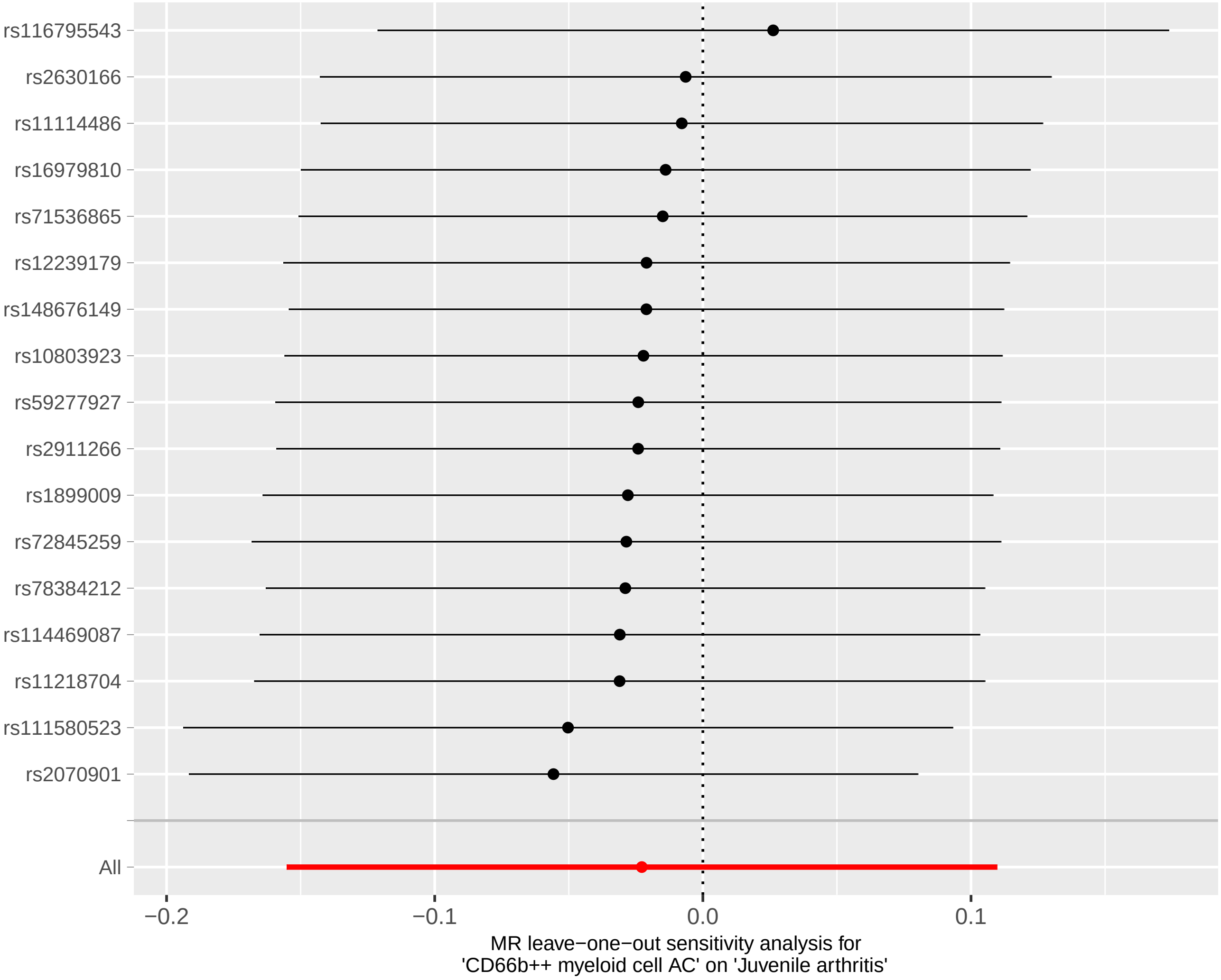

rs116795543
rs2630166
rs11114486
rs16979810
rs71536865
rs12239179
rs148676149
rs10803923
rs59277927
rs2911266
rs1899009
rs72845259
rs78384212
rs114469087
rs11218704
rs111580523
rs2070901
All
−0.2
−0.1
0.0
0.1
MR leave−one−out sensitivity analysis for
'CD66b++ myeloid cell AC' on 'Juvenile arthritis'

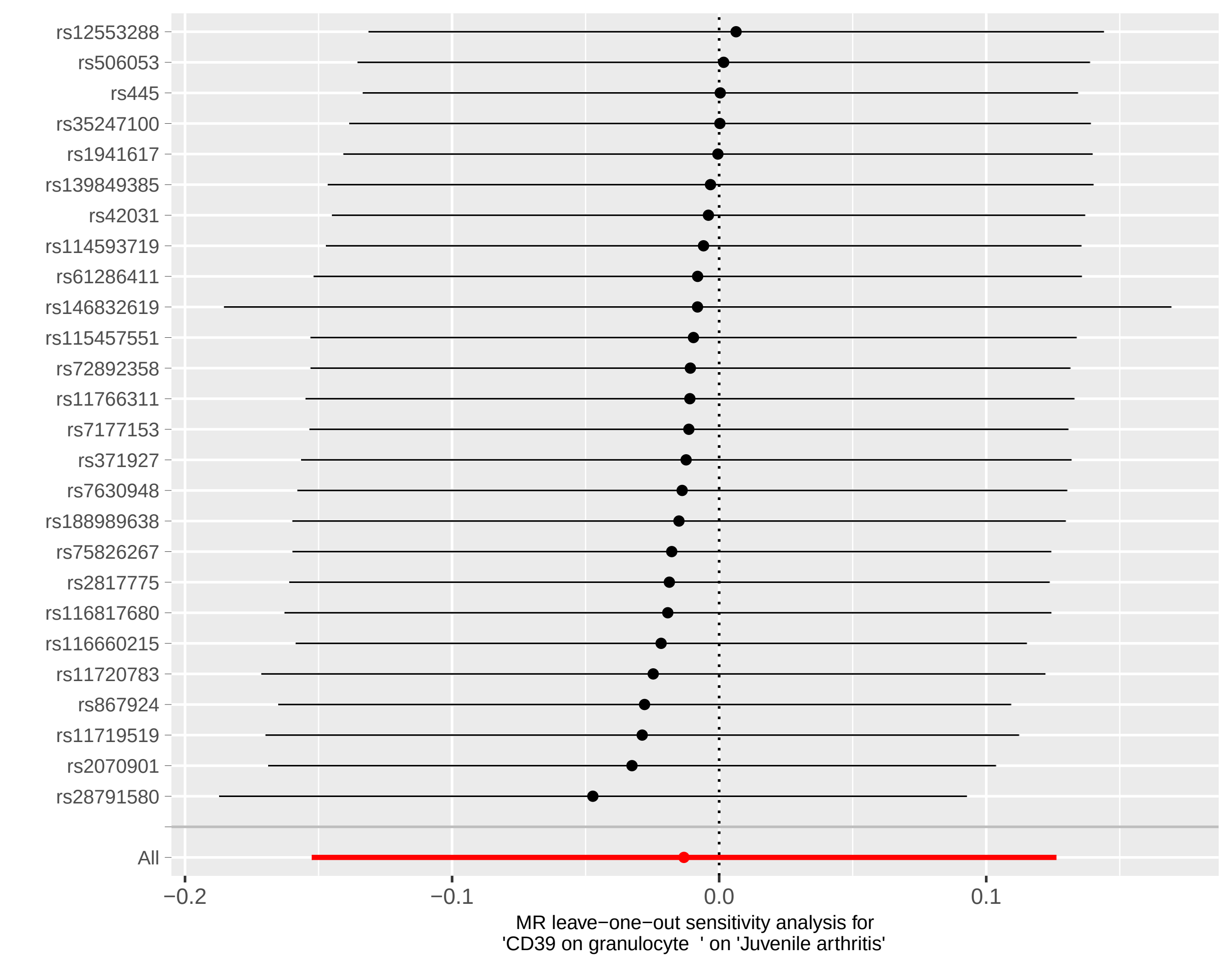
rs12553288
rs506053
rs445
rs35247100
rs1941617
rs139849385
rs42031
rs114593719
rs61286411
rs146832619
rs115457551
rs72892358
rs11766311
rs7177153
rs371927
rs7630948
rs188989638
rs75826267
rs2817775
rs116817680
rs116660215
rs11720783
rs867924
rs11719519
rs2070901
rs28791580
All
−0.2
−0.1
0.0
0.1
MR leave−one−out sensitivity analysis for
'CD39 on granulocyte ' on 'Juvenile arthritis'

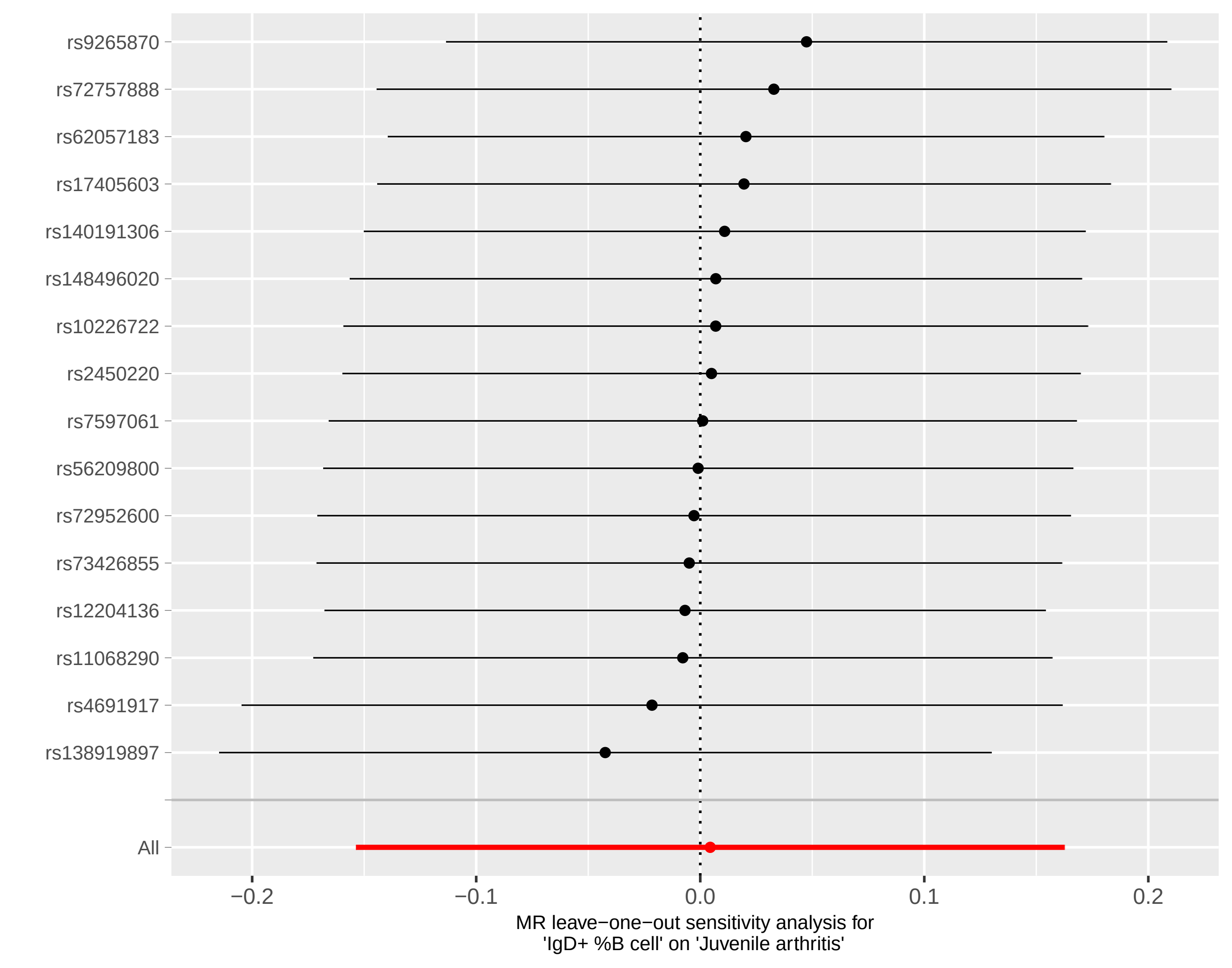

rs9265870
rs72757888
rs62057183
rs17405603
rs140191306
rs148496020
rs10226722
rs2450220
rs7597061
rs56209800
rs72952600
rs73426855
rs12204136
rs11068290
rs4691917
rs138919897
All
−0.2
−0.1
0.0
0.1
0.2
MR leave−one−out sensitivity analysis for
'IgD+ %B cell' on 'Juvenile arthritis'

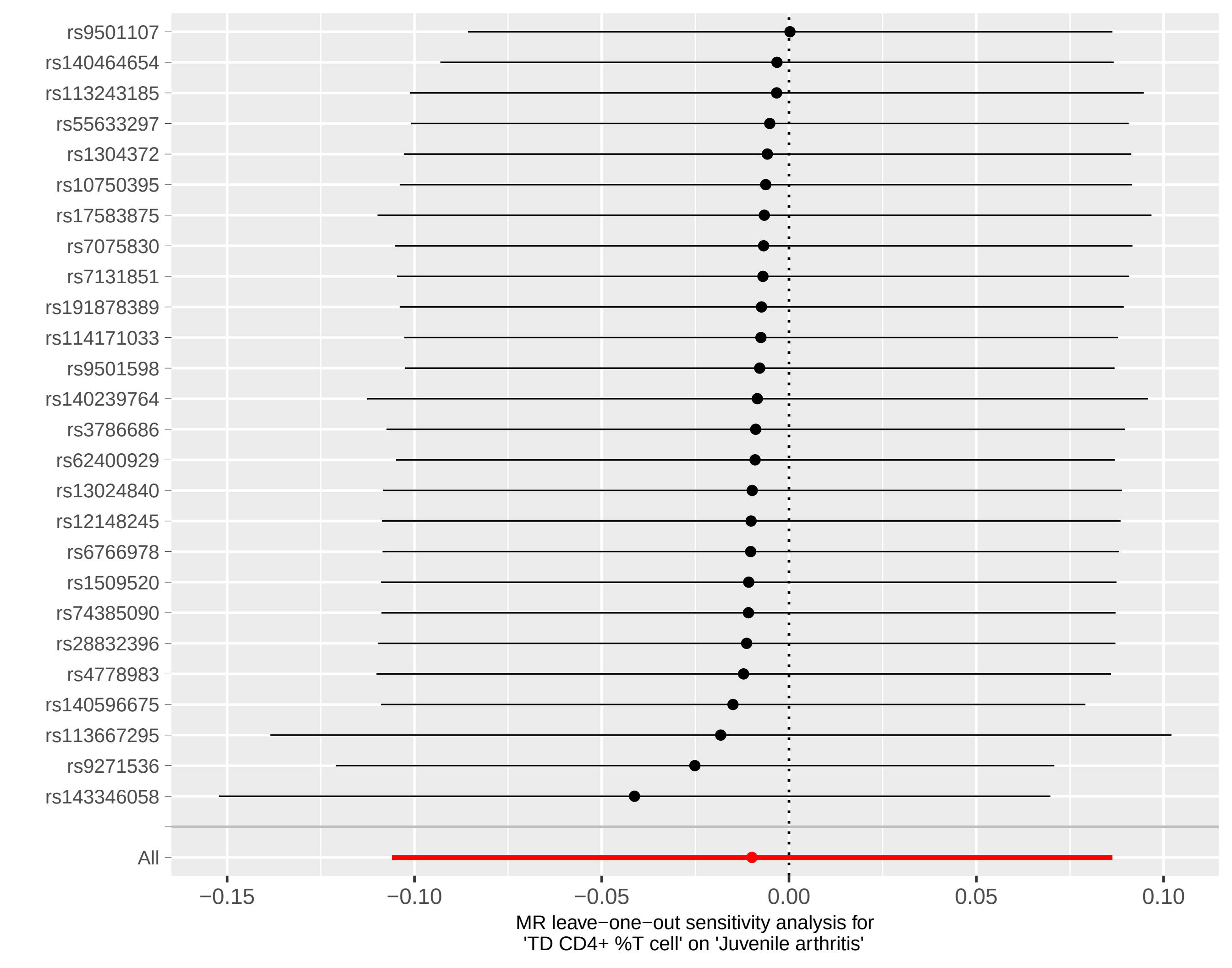

rs9501107
rs140464654
rs113243185
rs55633297
rs1304372
rs10750395
rs17583875
rs7075830
rs7131851
rs191878389
rs114171033
rs9501598
rs140239764
rs3786686
rs62400929
rs13024840
rs12148245
rs6766978
rs1509520
rs74385090
rs28832396
rs4778983
rs140596675
rs113667295
rs9271536
rs143346058
All
−0.15
−0.10
−0.05
0.00
0.05
0.10
MR leave−one−out sensitivity analysis for
'TD CD4+ %T cell' on 'Juvenile arthritis'

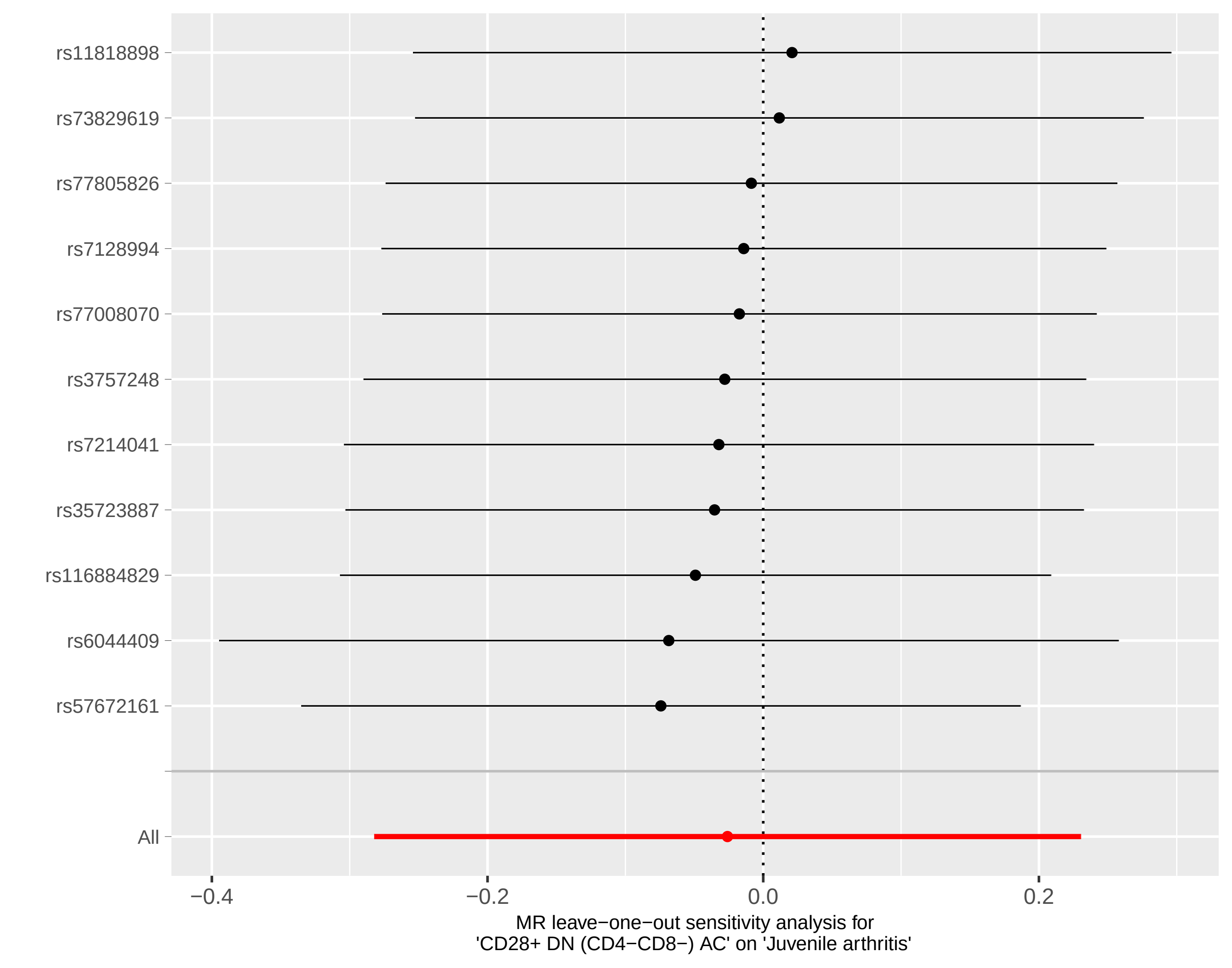

rs11818898
rs73829619
rs77805826
rs7128994
rs77008070
rs3757248
rs7214041
rs35723887
rs116884829
rs6044409
rs57672161
All
−0.4
−0.2
0.0
0.2
MR leave−one−out sensitivity analysis for
'CD28+ DN (CD4−CD8−) AC' on 'Juvenile arthritis'

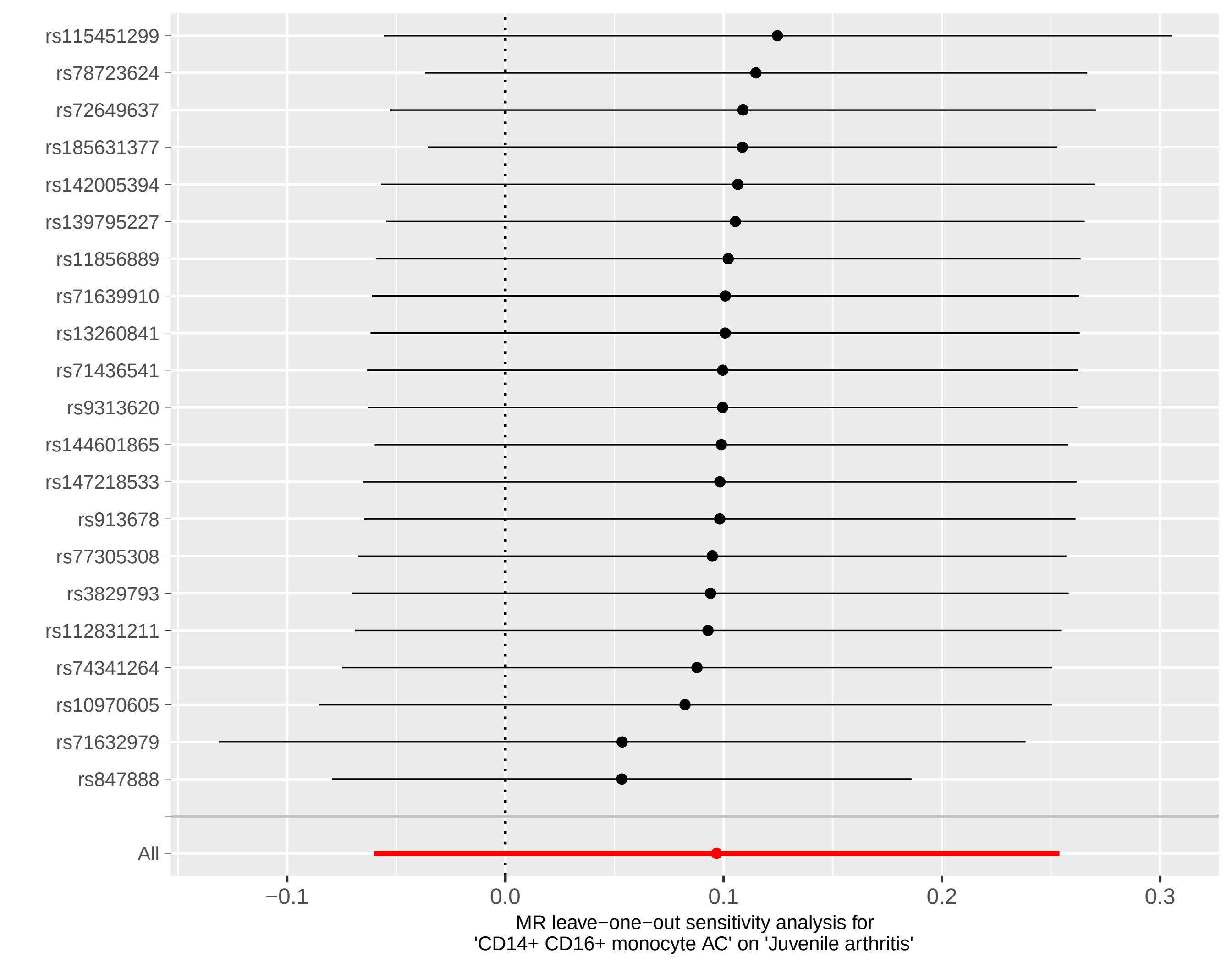
rs115451299
rs78723624
rs72649637
rs185631377
rs142005394
rs139795227
rs11856889
rs71639910
rs13260841
rs71436541
rs9313620
rs144601865
rs147218533
rs913678
rs77305308
rs3829793
rs112831211
rs74341264
rs10970605
rs71632979
rs847888
All
−0.1
0.0
0.1
0.2
0.3
MR leave−one−out sensitivity analysis for
'CD14+ CD16+ monocyte AC' on 'Juvenile arthritis'

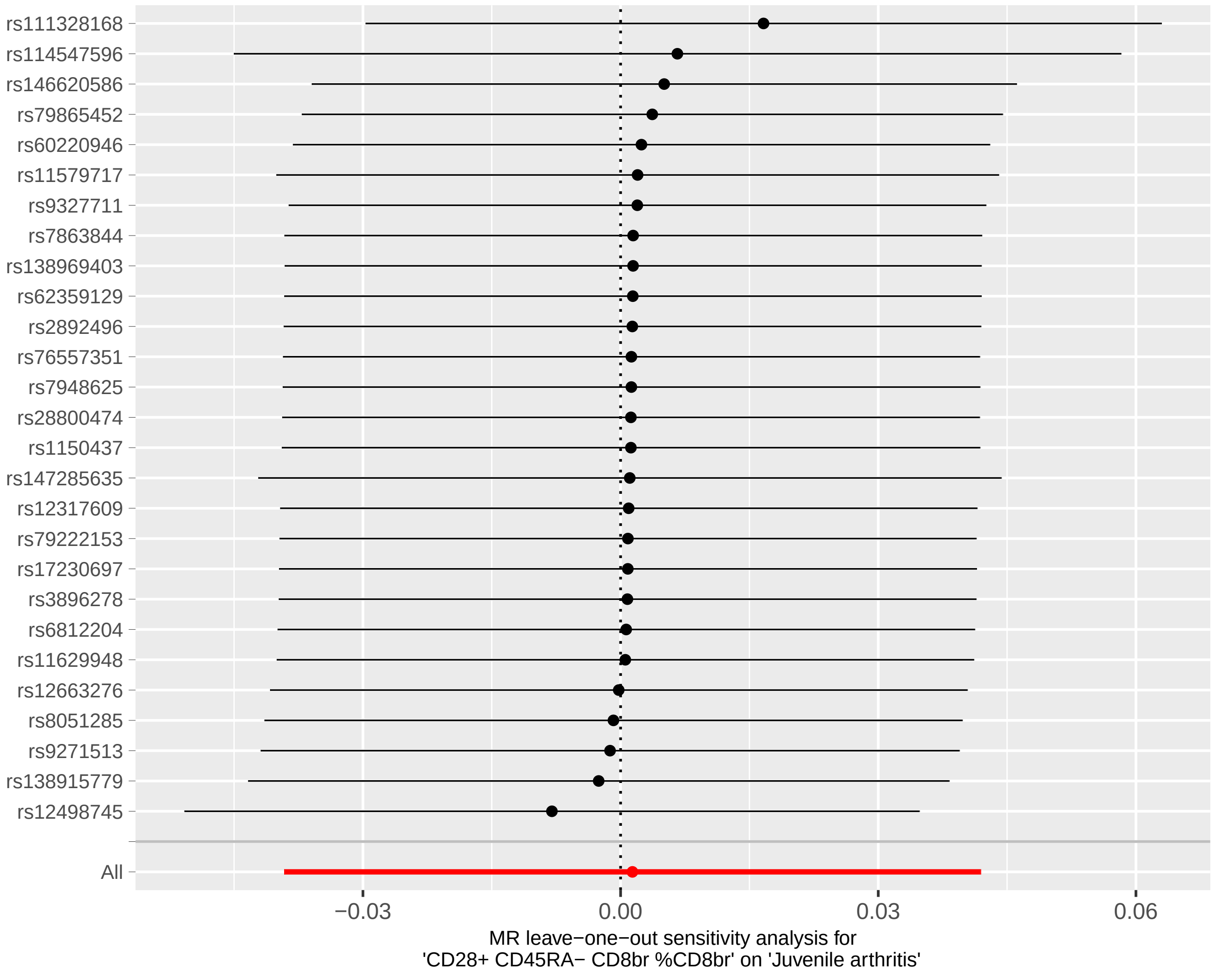
rs111328168
rs114547596
rs146620586
rs79865452
rs60220946
rs11579717
rs9327711
rs7863844
rs138969403
rs62359129
rs2892496
rs76557351
rs7948625
rs28800474
rs1150437
rs147285635
rs12317609
rs79222153
rs17230697
rs3896278
rs6812204
rs11629948
rs12663276
rs8051285
rs9271513
rs138915779
rs12498745
All
−0.03
0.00
0.03
0.06
MR leave−one−out sensitivity analysis for
'CD28+ CD45RA− CD8br %CD8br' on 'Juvenile arthritis'

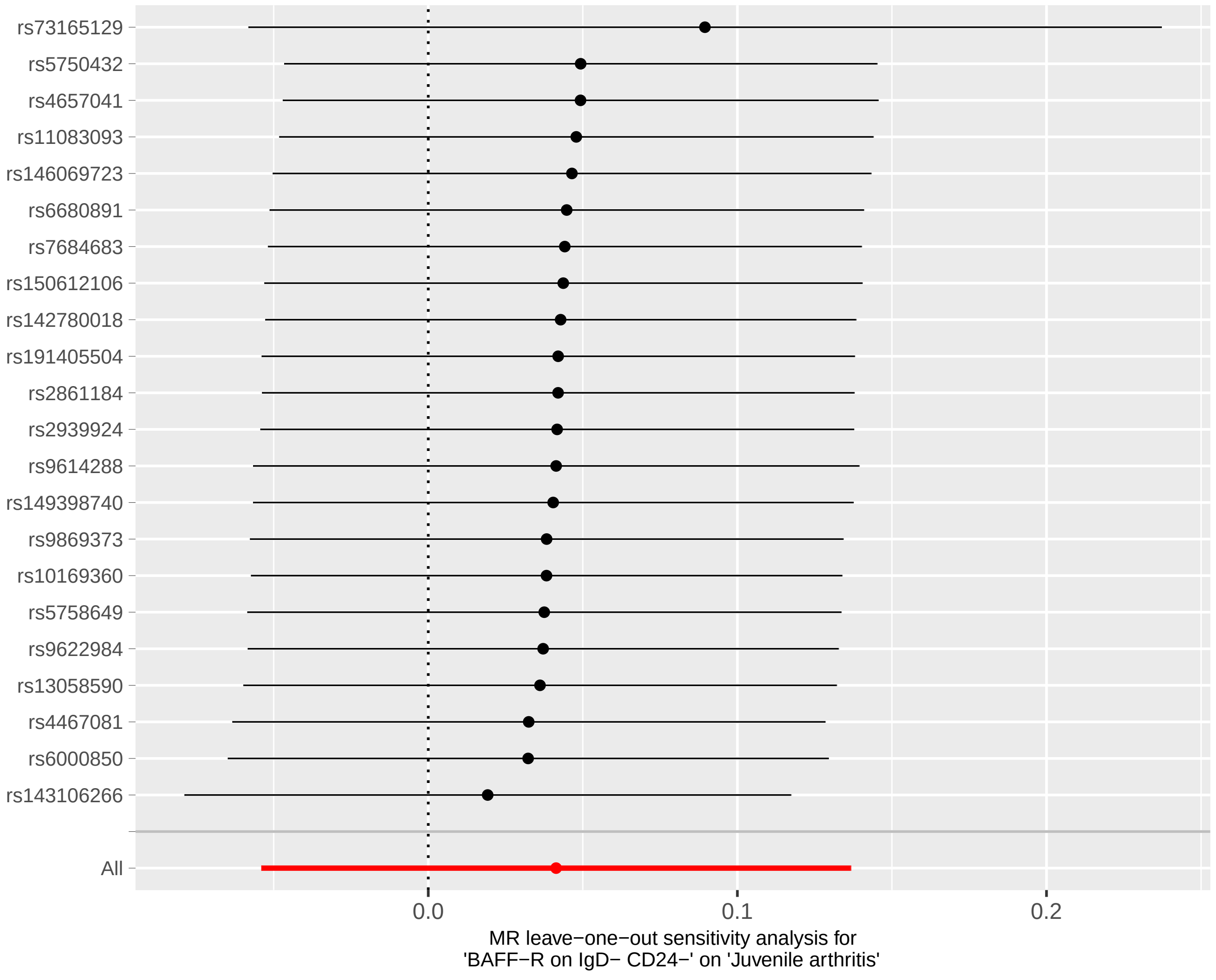
rs73165129
rs5750432
rs4657041
rs11083093
rs146069723
rs6680891
rs7684683
rs150612106
rs142780018
rs191405504
rs2861184
rs2939924
rs9614288
rs149398740
rs9869373
rs10169360
rs5758649
rs9622984
rs13058590
rs4467081
rs6000850
rs143106266
All
0.0
0.1
0.2
MR leave−one−out sensitivity analysis for
'BAFF−R on IgD− CD24−' on 'Juvenile arthritis'

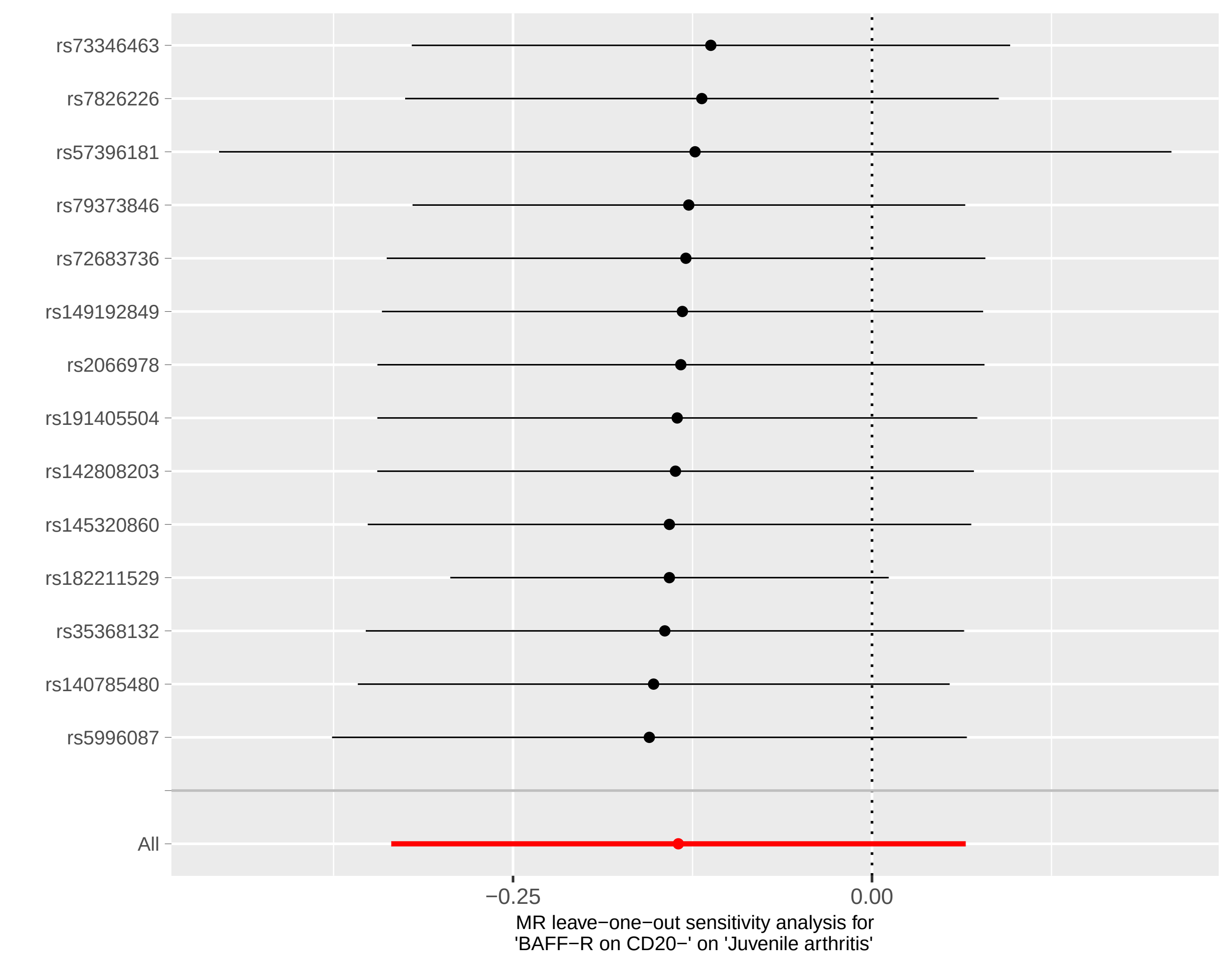
rs73346463
rs7826226
rs57396181
rs79373846
rs72683736
rs149192849
rs2066978
rs191405504
rs142808203
rs145320860
rs182211529
rs35368132
rs140785480
rs5996087
All
−0.25
0.00
MR leave−one−out sensitivity analysis for
'BAFF−R on CD20−' on 'Juvenile arthritis'

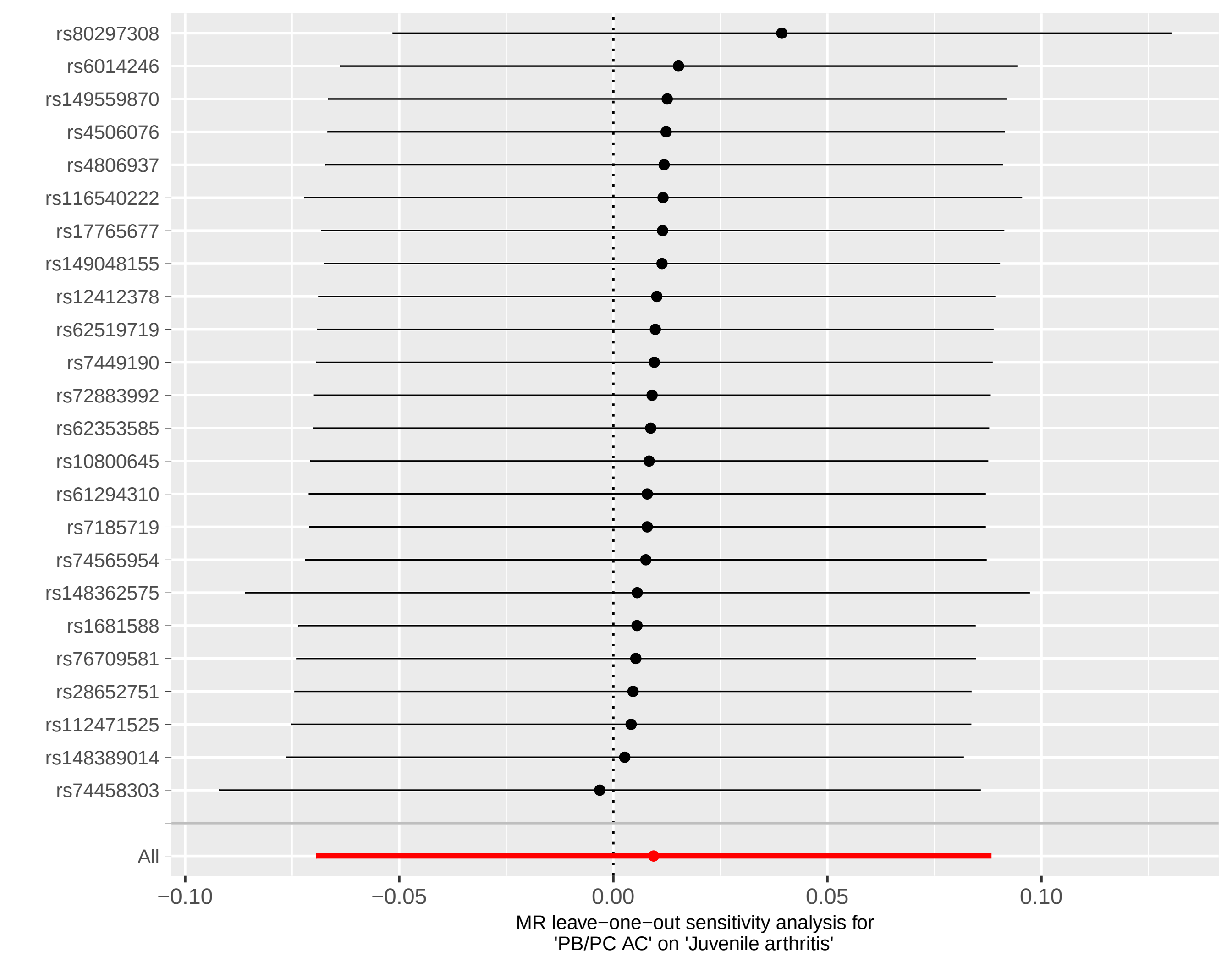
rs80297308
rs6014246
rs149559870
rs4506076
rs4806937
rs116540222
rs17765677
rs149048155
rs12412378
rs62519719
rs7449190
rs72883992
rs62353585
rs10800645
rs61294310
rs7185719
rs74565954
rs148362575
rs1681588
rs76709581
rs28652751
rs112471525
rs148389014
rs74458303
All
−0.10
−0.05
0.00
0.05
0.10
MR leave−one−out sensitivity analysis for
'PB/PC AC' on 'Juvenile arthritis'

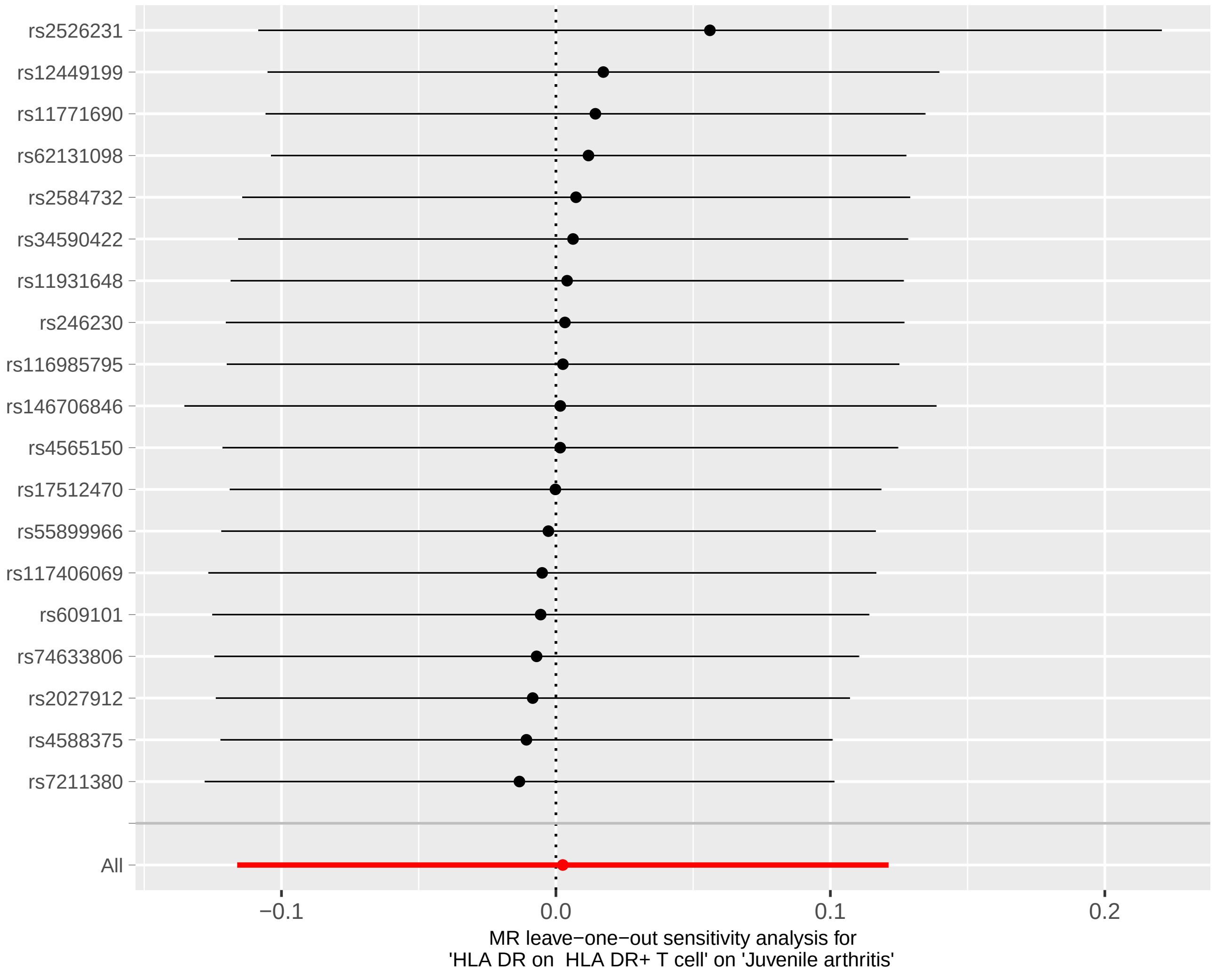
rs2526231
rs12449199
rs11771690
rs62131098
rs2584732
rs34590422
rs11931648
rs246230
rs116985795
rs146706846
rs4565150
rs17512470
rs55899966
rs117406069
rs609101
rs74633806
rs2027912
rs4588375
rs7211380
All
−0.1
0.0
0.1
0.2
MR leave−one−out sensitivity analysis for
'HLA DR on HLA DR+ T cell' on 'Juvenile arthritis'

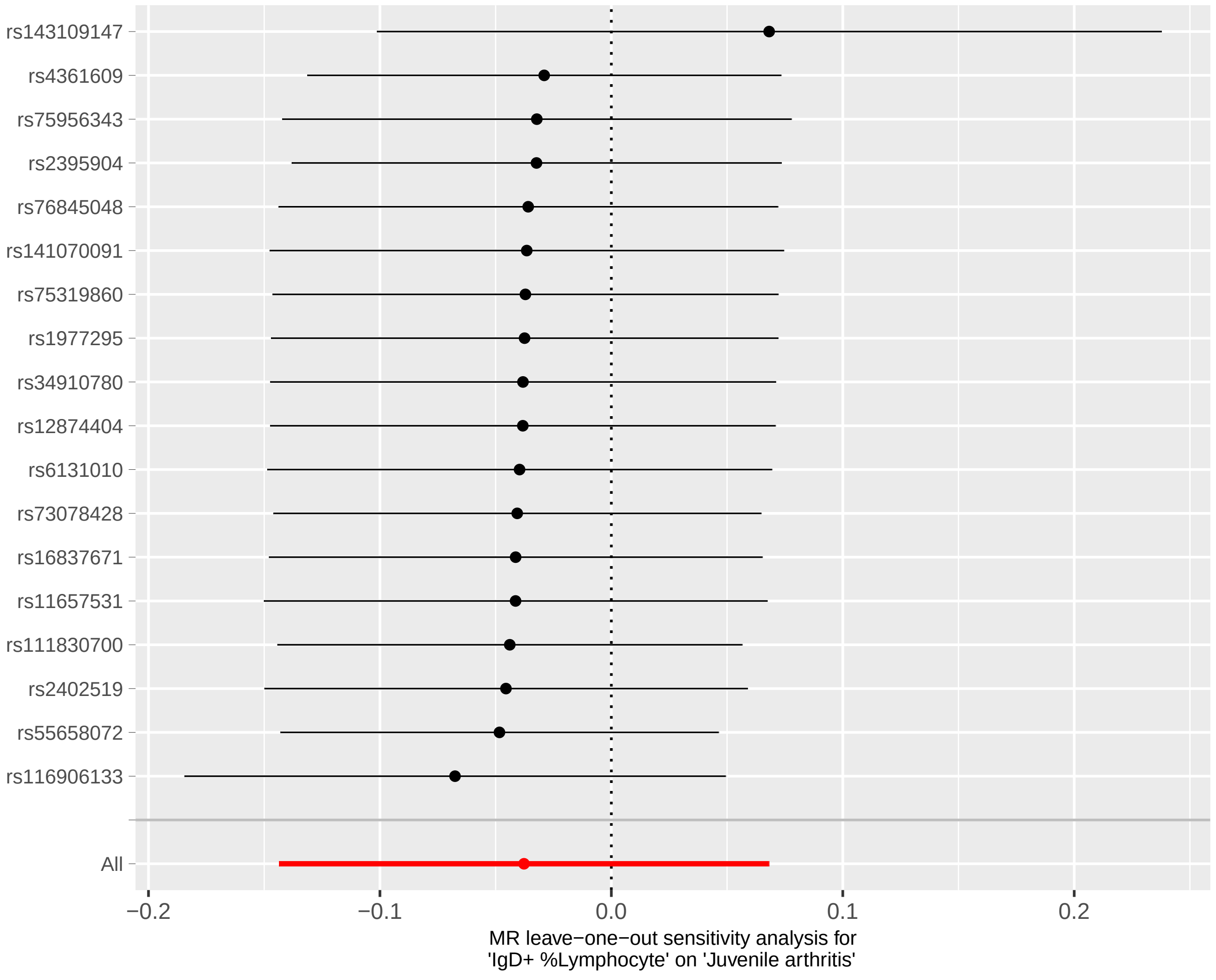

rs143109147
rs4361609
rs75956343
rs2395904
rs76845048
rs141070091
rs75319860
rs1977295
rs34910780
rs12874404
rs6131010
rs73078428
rs16837671
rs11657531
rs111830700
rs2402519
rs55658072
rs116906133
All
−0.2
−0.1
0.0
0.1
0.2
MR leave−one−out sensitivity analysis for
'IgD+ %Lymphocyte' on 'Juvenile arthritis'

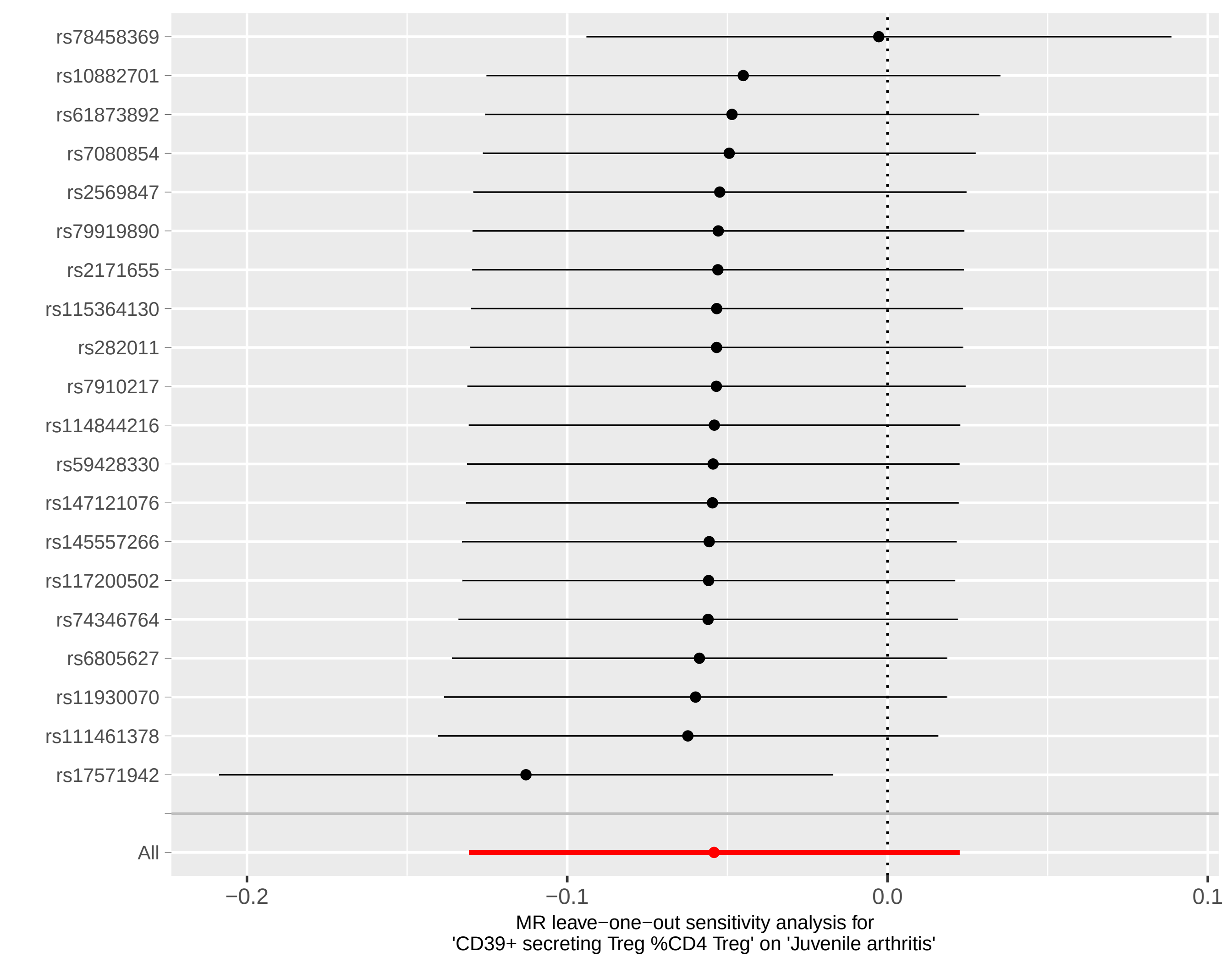
rs78458369
rs10882701
rs61873892
rs7080854
rs2569847
rs79919890
rs2171655
rs115364130
rs282011
rs7910217
rs114844216
rs59428330
rs147121076
rs145557266
rs117200502
rs74346764
rs6805627
rs11930070
rs111461378
rs17571942
All
−0.2
−0.1
0.0
0.1
MR leave−one−out sensitivity analysis for
'CD39+ secreting Treg %CD4 Treg' on 'Juvenile arthritis'

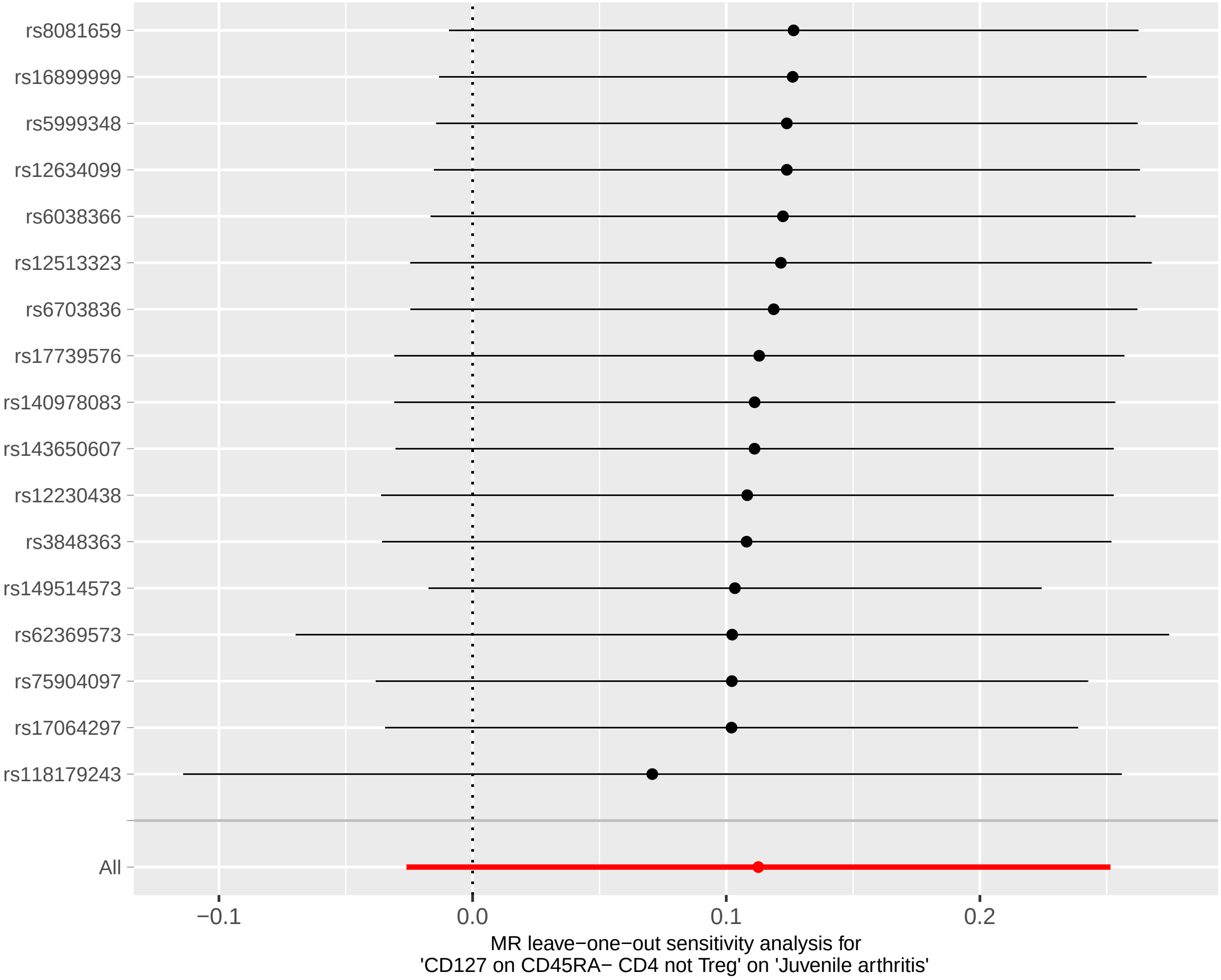
rs8081659
rs16899999
rs5999348
rs12634099
rs6038366
rs12513323
rs6703836
rs17739576
rs140978083
rs143650607
rs12230438
rs3848363
rs149514573
rs62369573
rs75904097
rs17064297
rs118179243
All
−0.1
0.0
0.1
0.2
MR leave−one−out sensitivity analysis for
'CD127 on CD45RA− CD4 not Treg' on 'Juvenile arthritis'

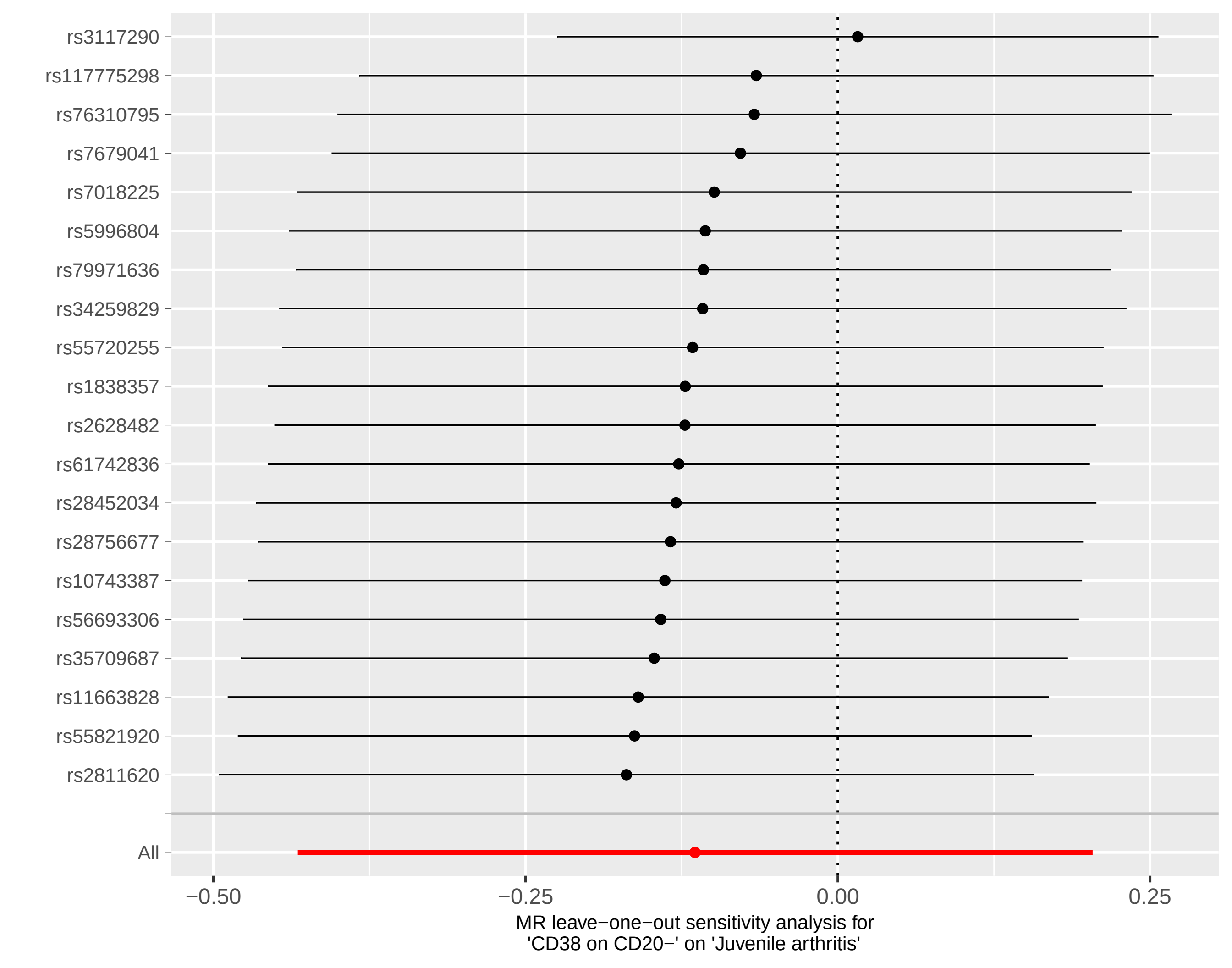
rs3117290
rs117775298
rs76310795
rs7679041
rs7018225
rs5996804
rs79971636
rs34259829
rs55720255
rs1838357
rs2628482
rs61742836
rs28452034
rs28756677
rs10743387
rs56693306
rs35709687
rs11663828
rs55821920
rs2811620
All
−0.50
−0.25
0.00
0.25
MR leave−one−out sensitivity analysis for
'CD38 on CD20−' on 'Juvenile arthritis'

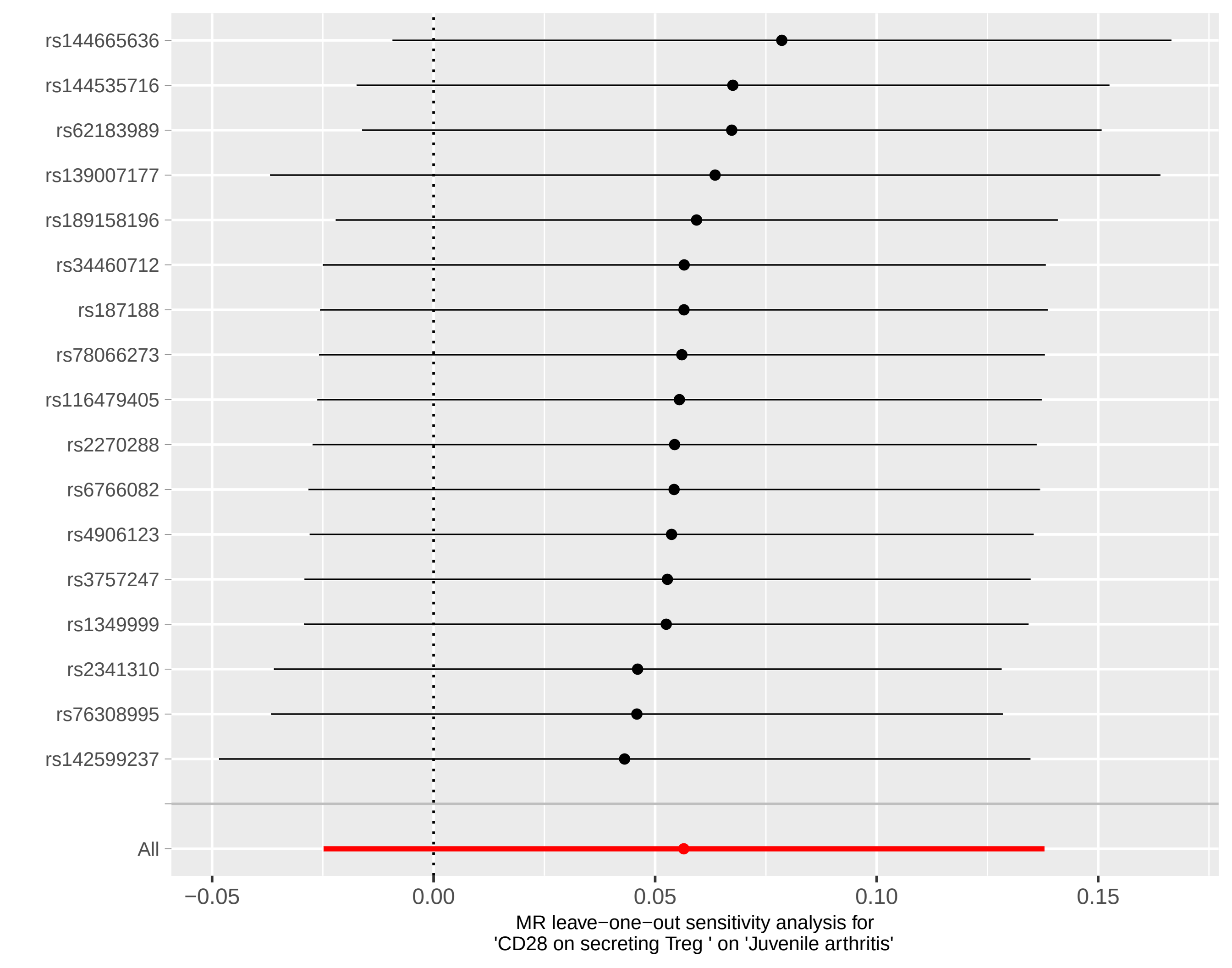
rs144665636
rs144535716
rs62183989
rs139007177
rs189158196
rs34460712
rs187188
rs78066273
rs116479405
rs2270288
rs6766082
rs4906123
rs3757247
rs1349999
rs2341310
rs76308995
rs142599237
All
−0.05
0.00
0.05
0.10
0.15
MR leave−one−out sensitivity analysis for
'CD28 on secreting Treg ' on 'Juvenile arthritis'

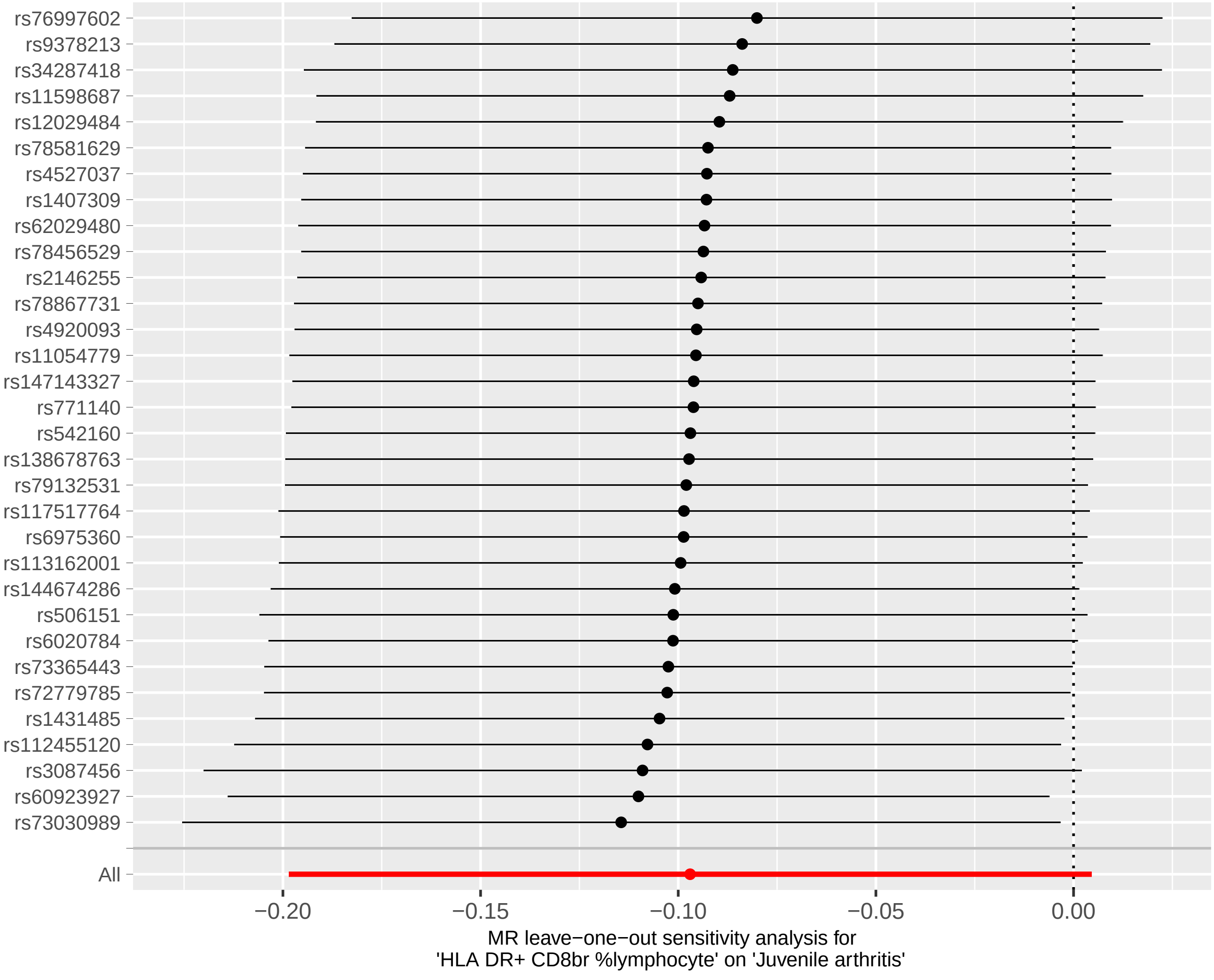

rs76997602
rs9378213
rs34287418
rs11598687
rs12029484
rs78581629
rs4527037
rs1407309
rs62029480
rs78456529
rs2146255
rs78867731
rs4920093
rs11054779
rs147143327
rs771140
rs542160
rs138678763
rs79132531
rs117517764
rs6975360
rs113162001
rs144674286
rs506151
rs6020784
rs73365443
rs72779785
rs1431485
rs112455120
rs3087456
rs60923927
rs73030989
All
−0.20
−0.15
−0.10
−0.05
0.00
MR leave−one−out sensitivity analysis for
'HLA DR+ CD8br %lymphocyte' on 'Juvenile arthritis'

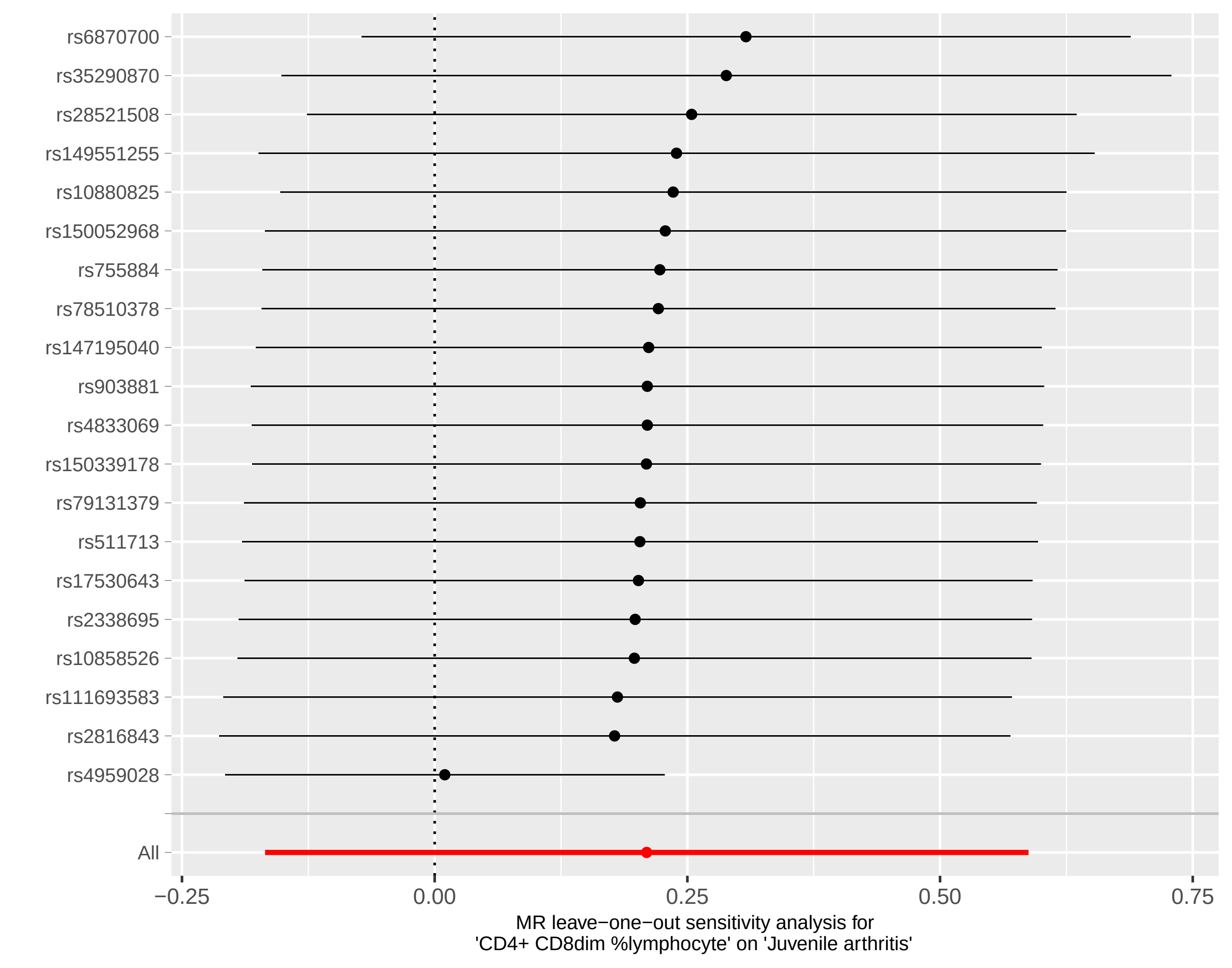

rs6870700
rs35290870
rs28521508
rs149551255
rs10880825
rs150052968
rs755884
rs78510378
rs147195040
rs903881
rs4833069
rs150339178
rs79131379
rs511713
rs17530643
rs2338695
rs10858526
rs111693583
rs2816843
rs4959028
All
−0.25
0.00
0.25
0.50
0.75
MR leave−one−out sensitivity analysis for
'CD4+ CD8dim %lymphocyte' on 'Juvenile arthritis'

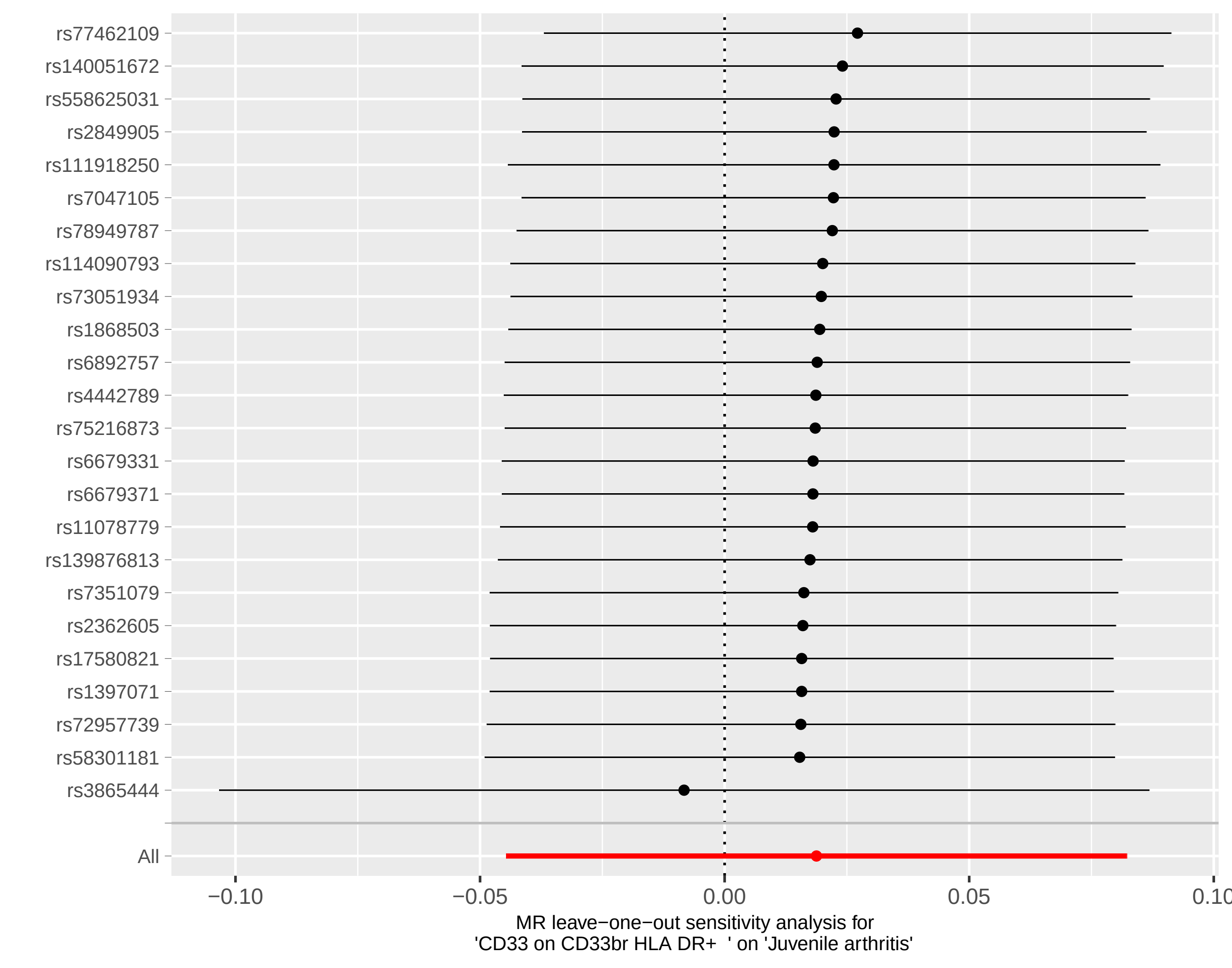
rs77462109
rs140051672
rs558625031
rs2849905
rs111918250
rs7047105
rs78949787
rs114090793
rs73051934
rs1868503
rs6892757
rs4442789
rs75216873
rs6679331
rs6679371
rs11078779
rs139876813
rs7351079
rs2362605
rs17580821
rs1397071
rs72957739
rs58301181
rs3865444
All
−0.10
−0.05
0.00
0.05
0.10
MR leave−one−out sensitivity analysis for
'CD33 on CD33br HLA DR+ ' on 'Juvenile arthritis'

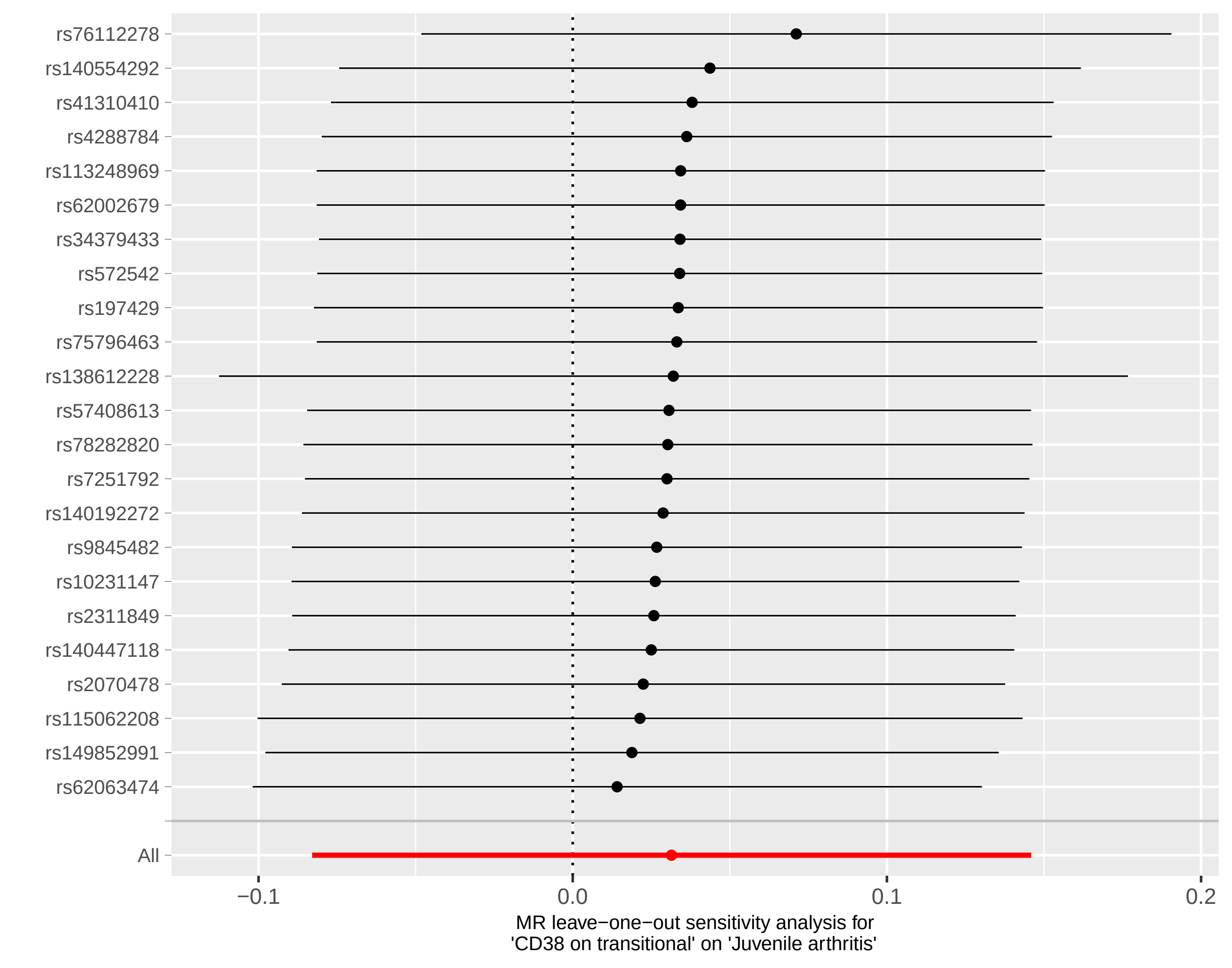

rs76112278
rs140554292
rs41310410
rs4288784
rs113248969
rs62002679
rs34379433
rs572542
rs197429
rs75796463
rs138612228
rs57408613
rs78282820
rs7251792
rs140192272
rs9845482
rs10231147
rs2311849
rs140447118
rs2070478
rs115062208
rs149852991
rs62063474
All
−0.1
0.0
0.1
0.2
MR leave−one−out sensitivity analysis for
'CD38 on transitional' on 'Juvenile arthritis'

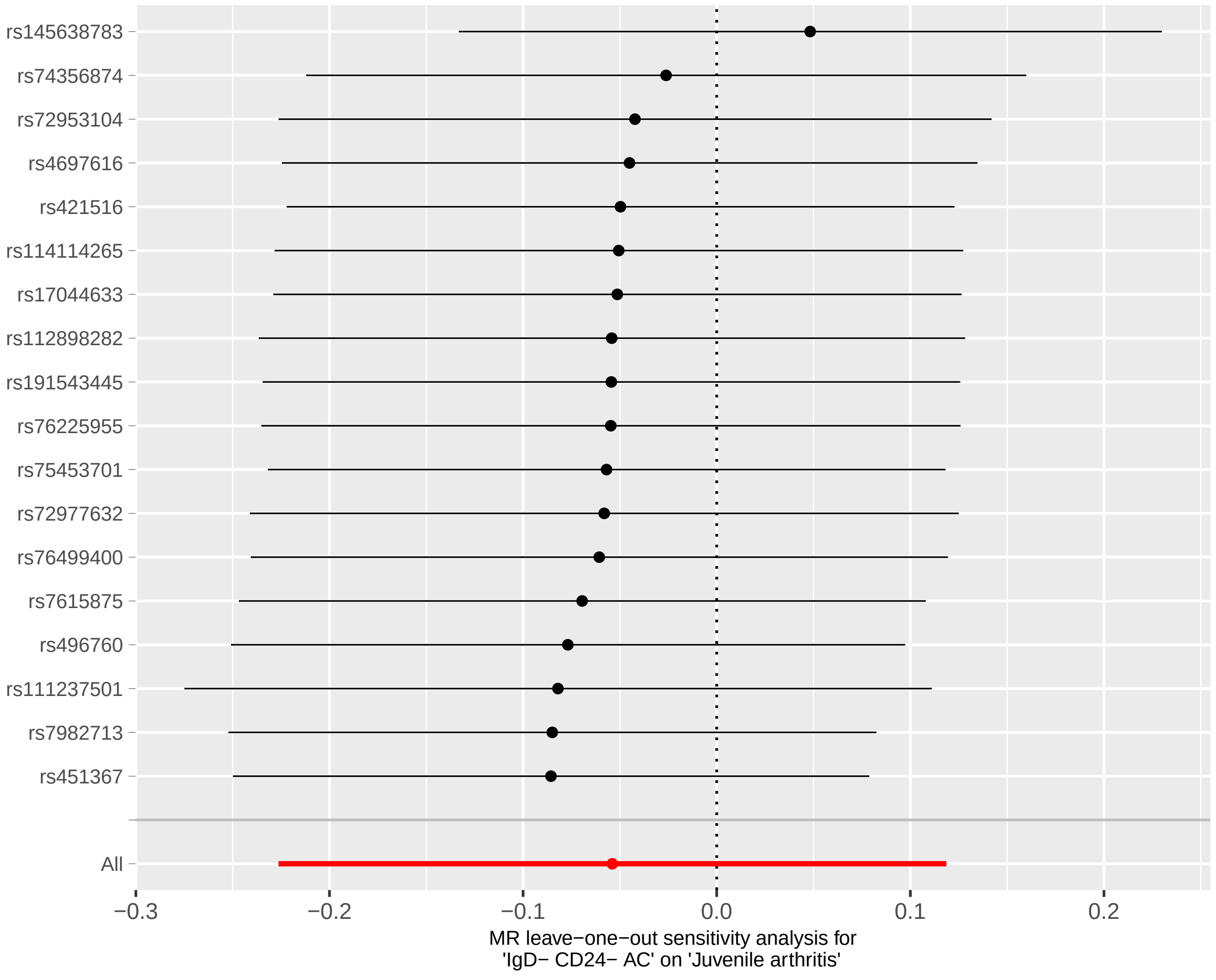

rs145638783
rs74356874
rs72953104
rs4697616
rs421516
rs114114265
rs17044633
rs112898282
rs191543445
rs76225955
rs75453701
rs72977632
rs76499400
rs7615875
rs496760
rs111237501
rs7982713
rs451367
All
−0.3
−0.2
−0.1
0.0
0.1
0.2
MR leave−one−out sensitivity analysis for
'IgD− CD24− AC' on 'Juvenile arthritis'

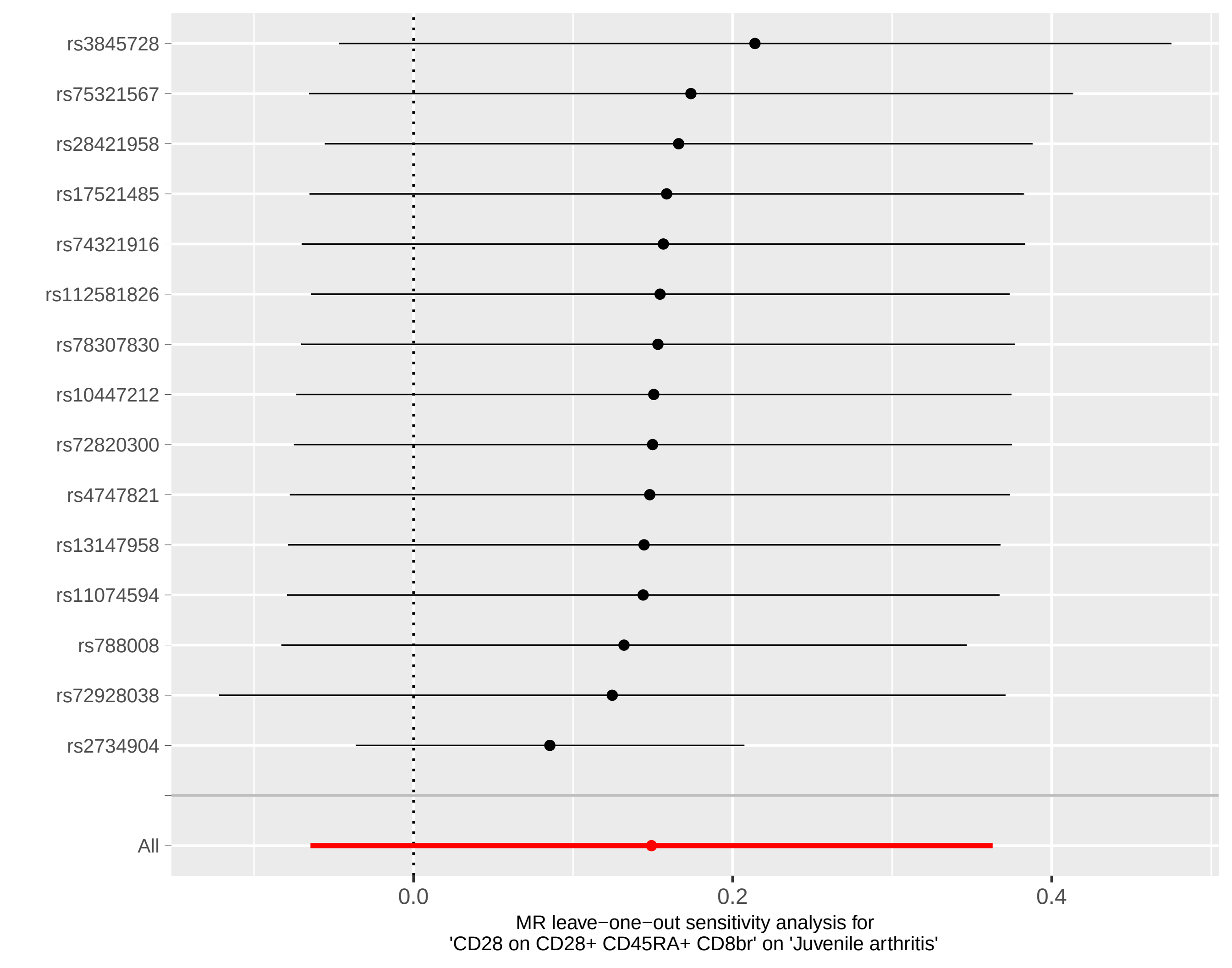

rs3845728
rs75321567
rs28421958
rs17521485
rs74321916
rs112581826
rs78307830
rs10447212
rs72820300
rs4747821
rs13147958
rs11074594
rs788008
rs72928038
rs2734904
All
0.0
0.2
0.4
MR leave−one−out sensitivity analysis for
'CD28 on CD28+ CD45RA+ CD8br' on 'Juvenile arthritis'

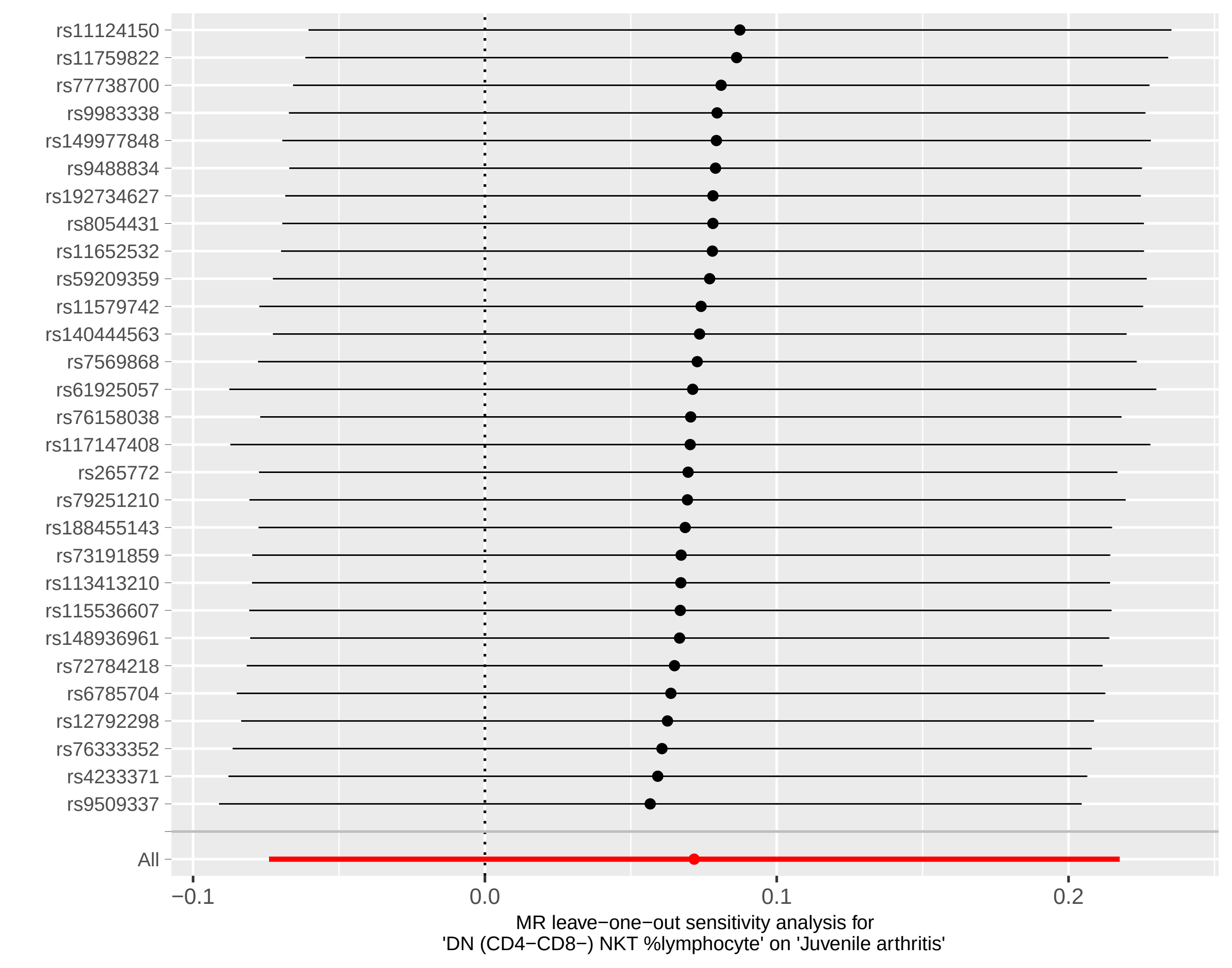
rs11124150
rs11759822
rs77738700
rs9983338
rs149977848
rs9488834
rs192734627
rs8054431
rs11652532
rs59209359
rs11579742
rs140444563
rs7569868
rs61925057
rs76158038
rs117147408
rs265772
rs79251210
rs188455143
rs73191859
rs113413210
rs115536607
rs148936961
rs72784218
rs6785704
rs12792298
rs76333352
rs4233371
rs9509337
All
−0.1
0.0
0.1
0.2
MR leave−one−out sensitivity analysis for
'DN (CD4−CD8−) NKT %lymphocyte' on 'Juvenile arthritis'

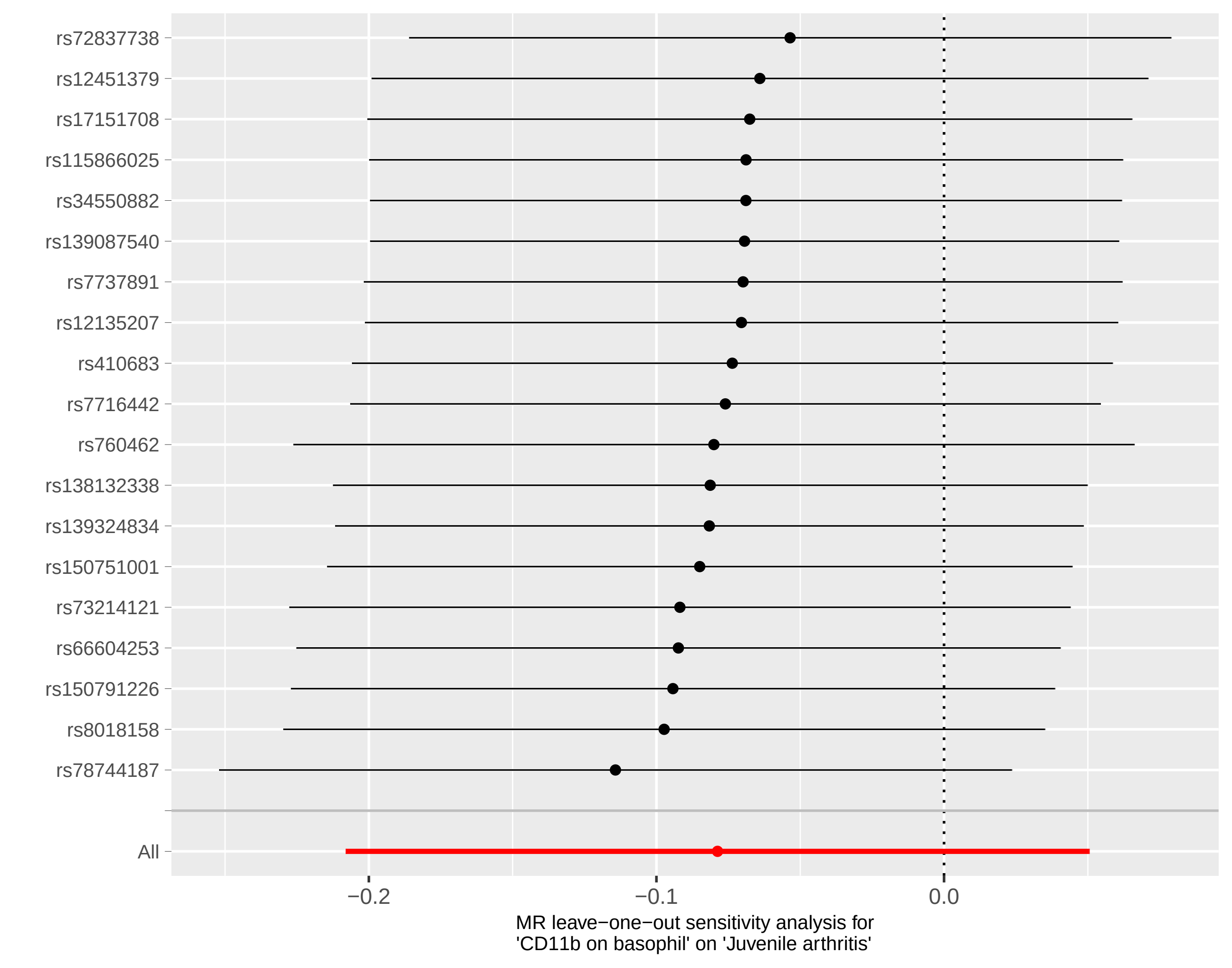
rs72837738
rs12451379
rs17151708
rs115866025
rs34550882
rs139087540
rs7737891
rs12135207
rs410683
rs7716442
rs760462
rs138132338
rs139324834
rs150751001
rs73214121
rs66604253
rs150791226
rs8018158
rs78744187
All
−0.2
−0.1
0.0
MR leave−one−out sensitivity analysis for
'CD11b on basophil' on 'Juvenile arthritis'

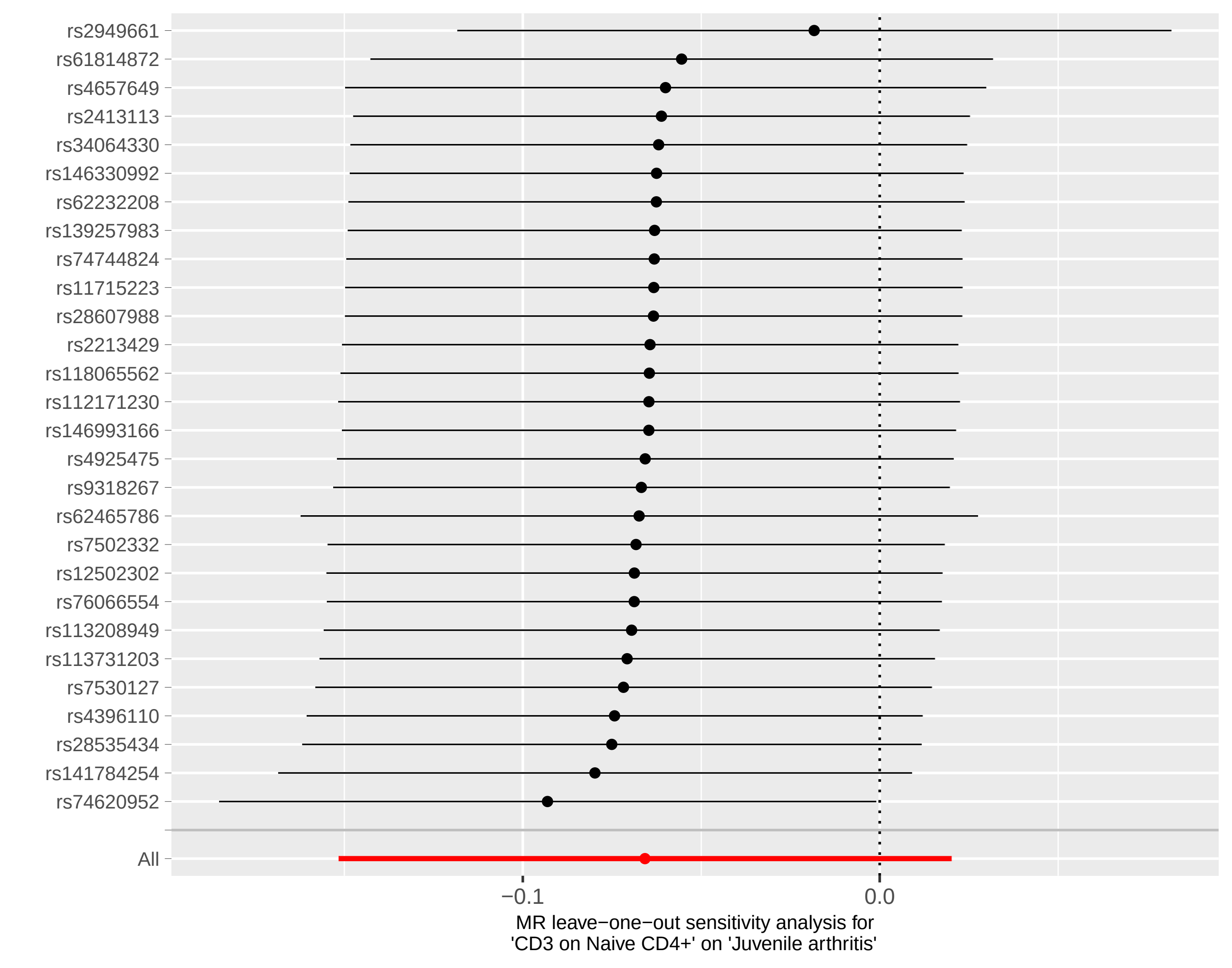

rs2949661
rs61814872
rs4657649
rs2413113
rs34064330
rs146330992
rs62232208
rs139257983
rs74744824
rs11715223
rs28607988
rs2213429
rs118065562
rs112171230
rs146993166
rs4925475
rs9318267
rs62465786
rs7502332
rs12502302
rs76066554
rs113208949
rs113731203
rs7530127
rs4396110
rs28535434
rs141784254
rs74620952
All
−0.1
0.0
MR leave−one−out sensitivity analysis for
'CD3 on Naive CD4+' on 'Juvenile arthritis'

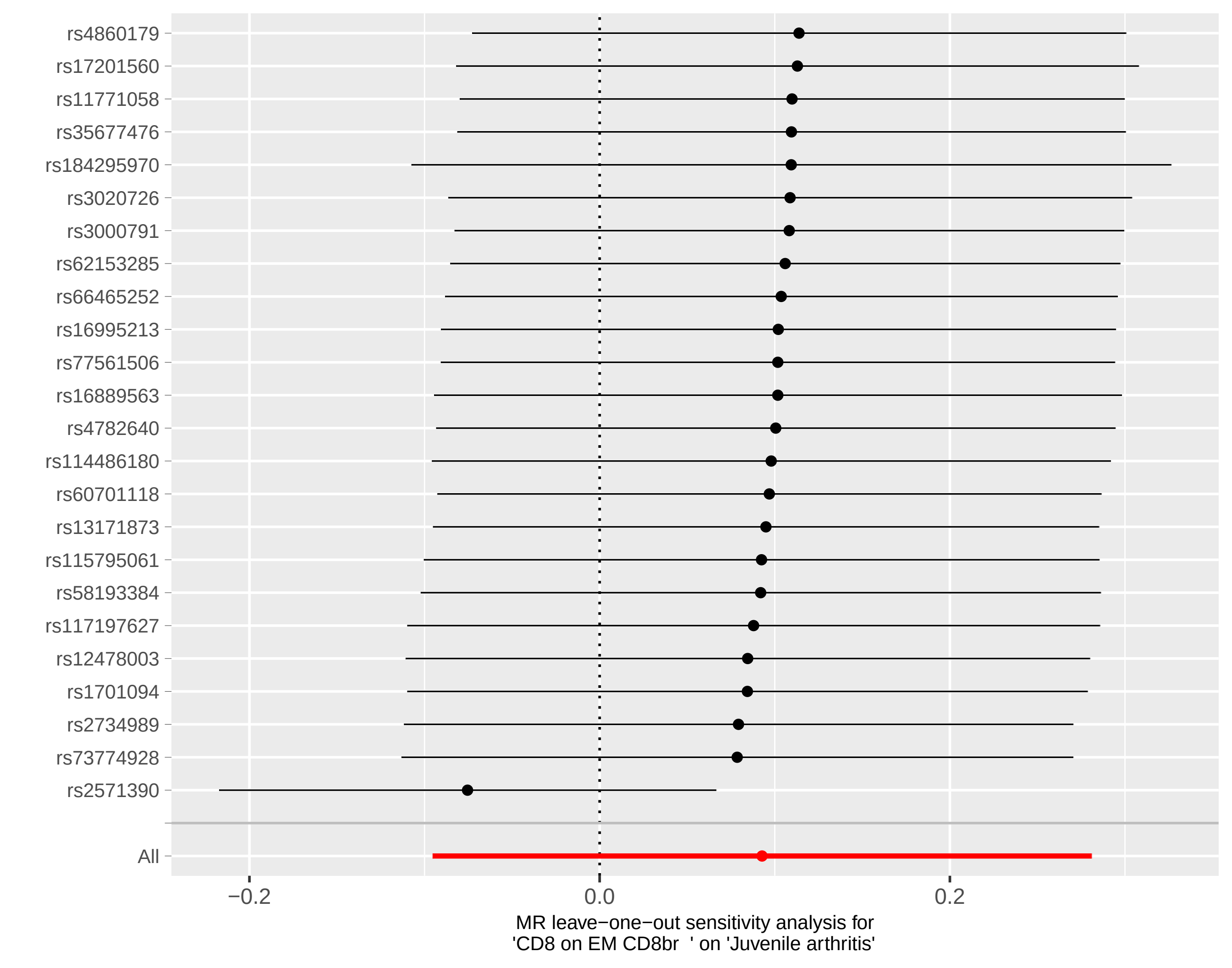
rs4860179
rs17201560
rs11771058
rs35677476
rs184295970
rs3020726
rs3000791
rs62153285
rs66465252
rs16995213
rs77561506
rs16889563
rs4782640
rs114486180
rs60701118
rs13171873
rs115795061
rs58193384
rs117197627
rs12478003
rs1701094
rs2734989
rs73774928
rs2571390
All
−0.2
0.0
0.2
MR leave−one−out sensitivity analysis for
'CD8 on EM CD8br ' on 'Juvenile arthritis'

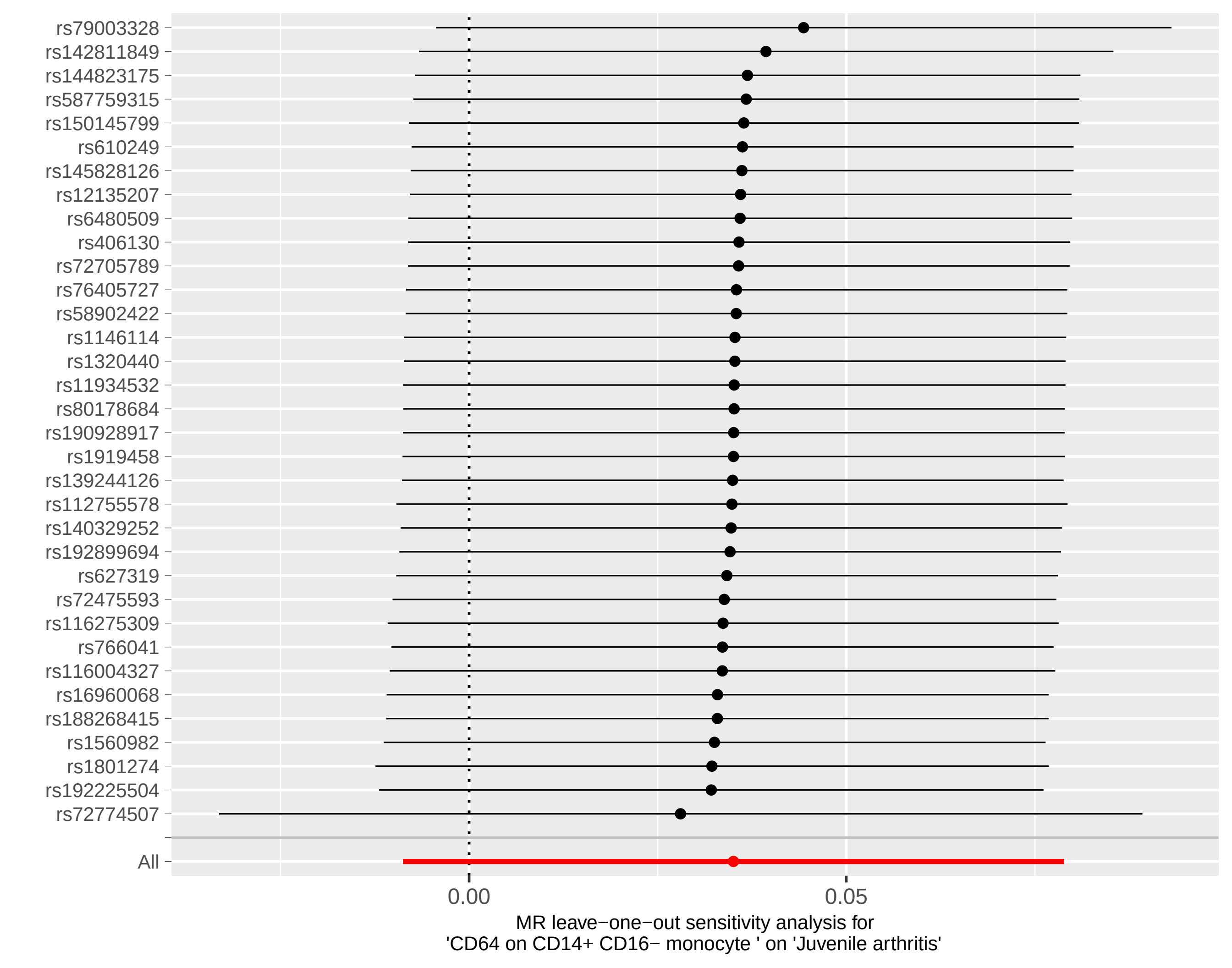
rs79003328
rs142811849
rs144823175
rs587759315
rs150145799
rs610249
rs145828126
rs12135207
rs6480509
rs406130
rs72705789
rs76405727
rs58902422
rs1146114
rs1320440
rs11934532
rs80178684
rs190928917
rs1919458
rs139244126
rs112755578
rs140329252
rs192899694
rs627319
rs72475593
rs116275309
rs766041
rs116004327
rs16960068
rs188268415
rs1560982
rs1801274
rs192225504
rs72774507
All
0.00
0.05
MR leave−one−out sensitivity analysis for
'CD64 on CD14+ CD16− monocyte ' on 'Juvenile arthritis'

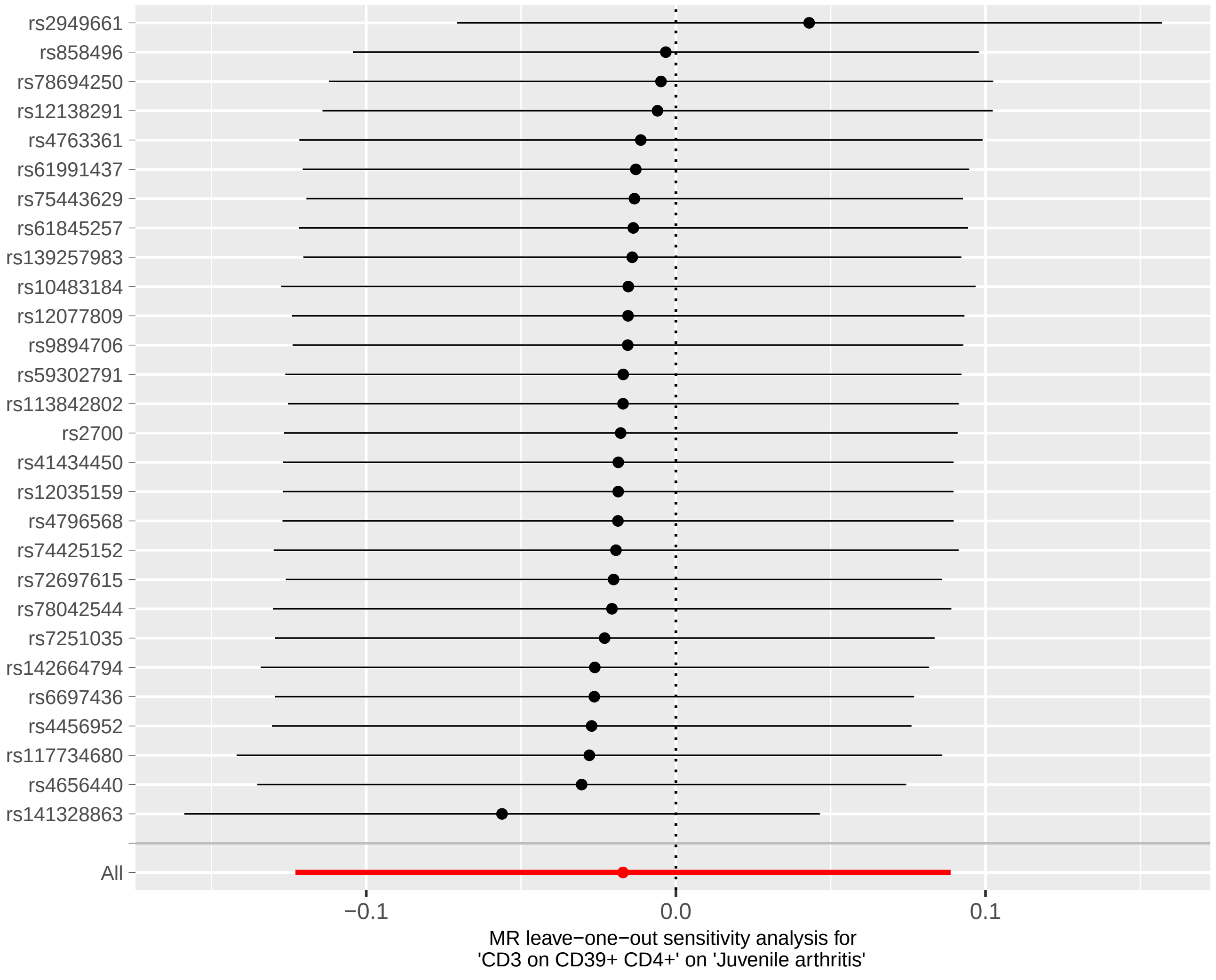
rs2949661
rs858496
rs78694250
rs12138291
rs4763361
rs61991437
rs75443629
rs61845257
rs139257983
rs10483184
rs12077809
rs9894706
rs59302791
rs113842802
rs2700
rs41434450
rs12035159
rs4796568
rs74425152
rs72697615
rs78042544
rs7251035
rs142664794
rs6697436
rs4456952
rs117734680
rs4656440
rs141328863
All
−0.1
0.0
0.1
MR leave−one−out sensitivity analysis for
'CD3 on CD39+ CD4+' on 'Juvenile arthritis'

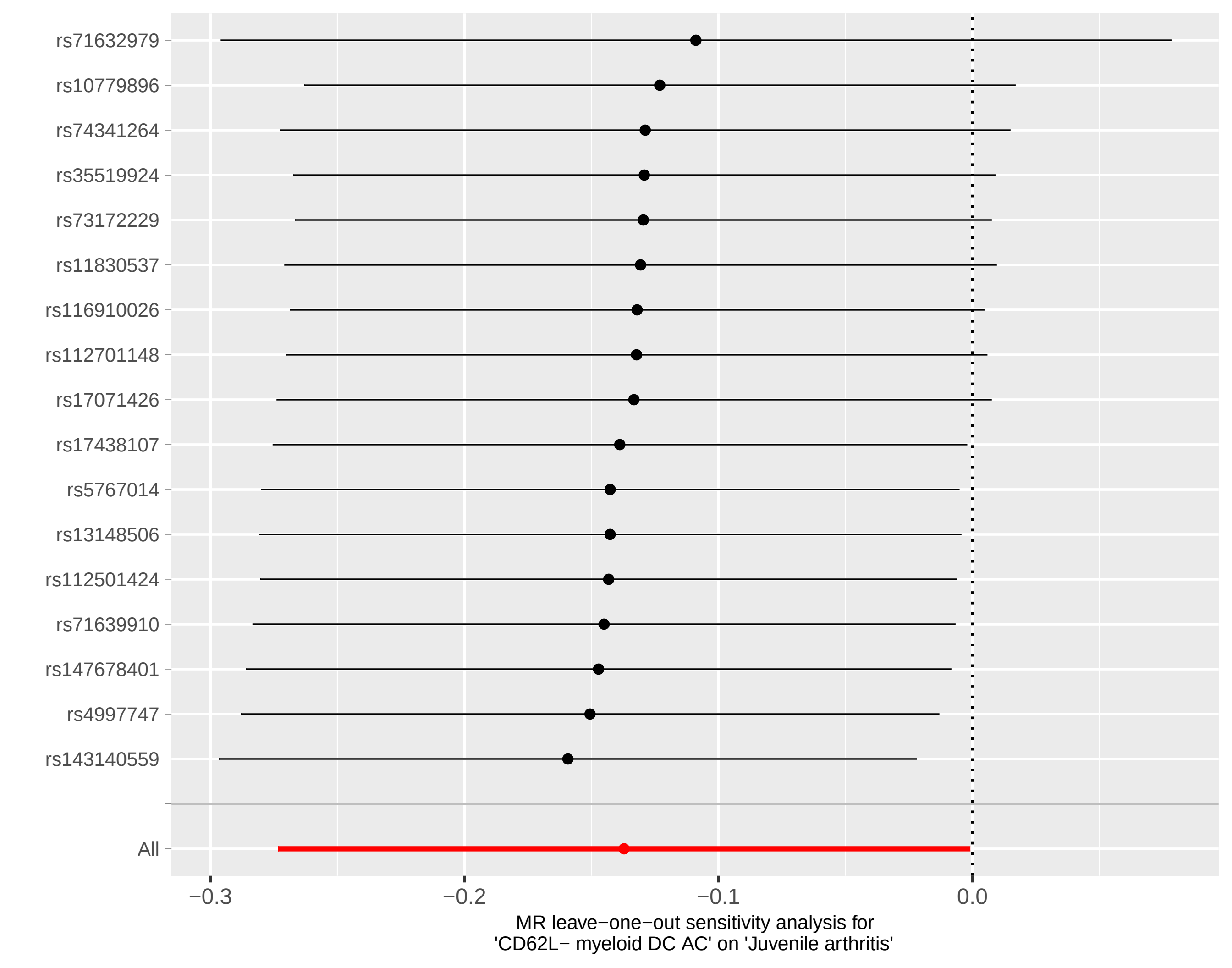
rs71632979
rs10779896
rs74341264
rs35519924
rs73172229
rs11830537
rs116910026
rs112701148
rs17071426
rs17438107
rs5767014
rs13148506
rs112501424
rs71639910
rs147678401
rs4997747
rs143140559
All
−0.3
−0.2
−0.1
0.0
MR leave−one−out sensitivity analysis for
'CD62L− myeloid DC AC' on 'Juvenile arthritis'

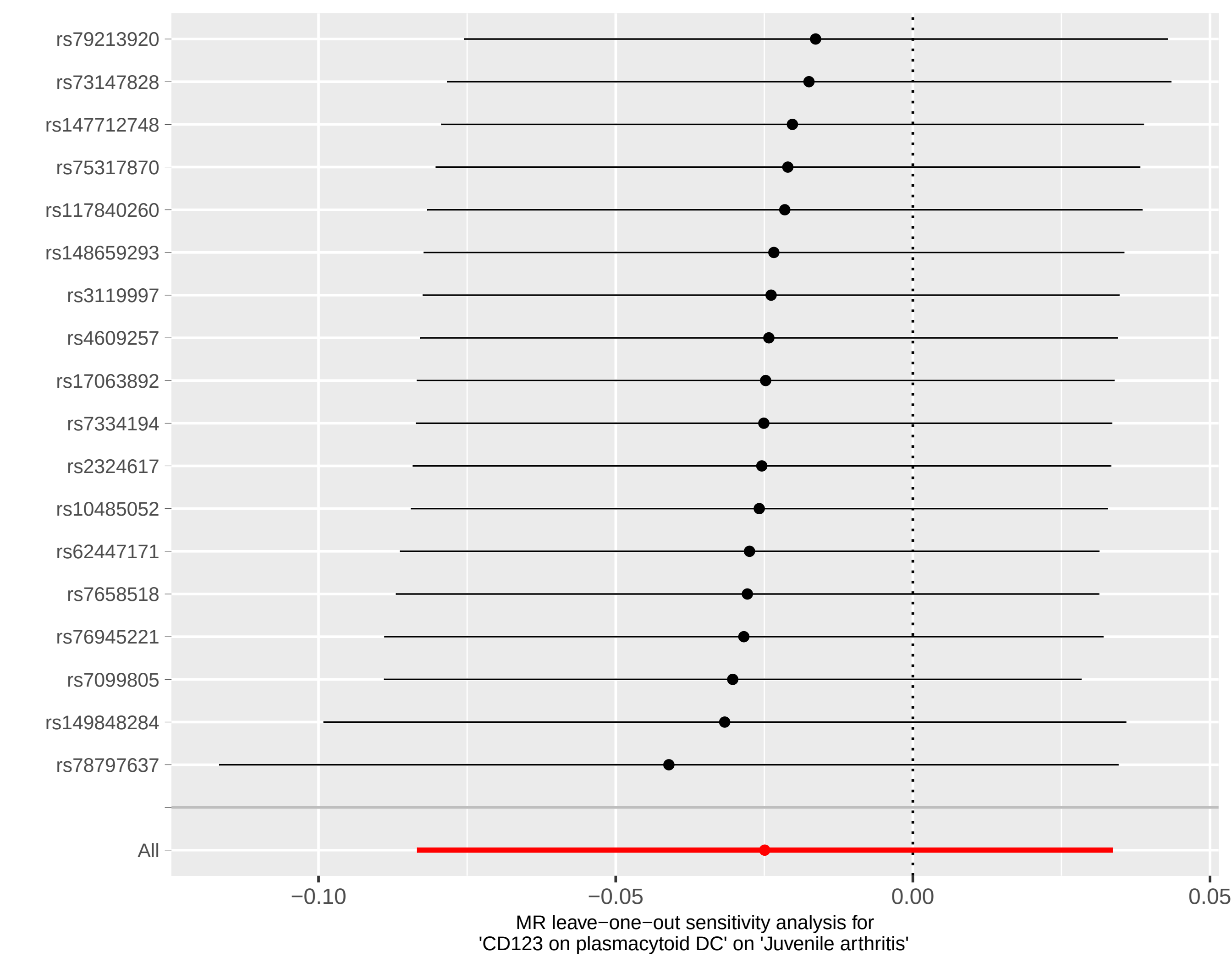
rs79213920
rs73147828
rs147712748
rs75317870
rs117840260
rs148659293
rs3119997
rs4609257
rs17063892
rs7334194
rs2324617
rs10485052
rs62447171
rs7658518
rs76945221
rs7099805
rs149848284
rs78797637
All
−0.10
−0.05
0.00
0.05
MR leave−one−out sensitivity analysis for
'CD123 on plasmacytoid DC' on 'Juvenile arthritis'

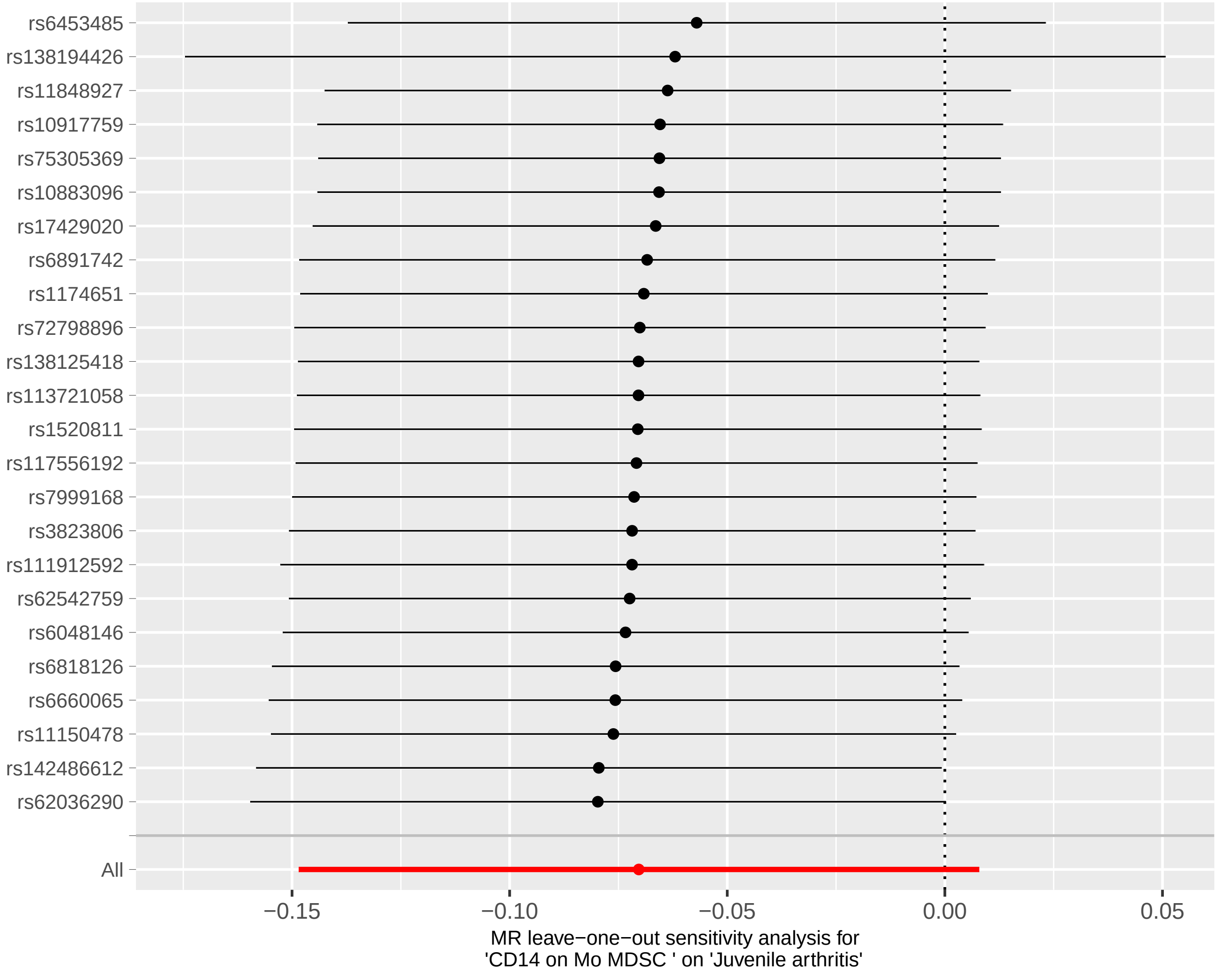
rs6453485
rs138194426
rs11848927
rs10917759
rs75305369
rs10883096
rs17429020
rs6891742
rs1174651
rs72798896
rs138125418
rs113721058
rs1520811
rs117556192
rs7999168
rs3823806
rs111912592
rs62542759
rs6048146
rs6818126
rs6660065
rs11150478
rs142486612
rs62036290
All
−0.15
−0.10
−0.05
0.00
0.05
MR leave−one−out sensitivity analysis for
'CD14 on Mo MDSC ' on 'Juvenile arthritis'

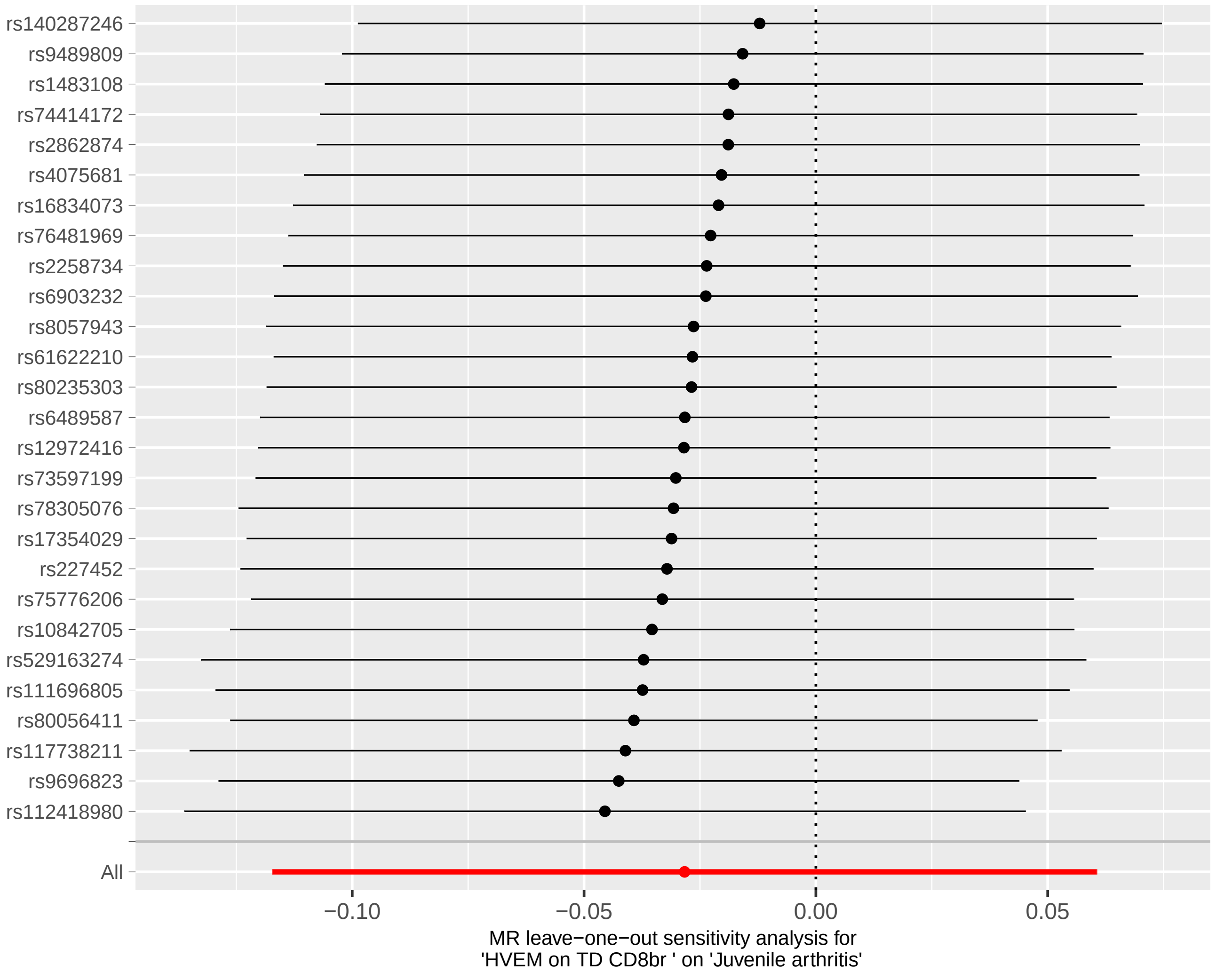
rs140287246
rs9489809
rs1483108
rs74414172
rs2862874
rs4075681
rs16834073
rs76481969
rs2258734
rs6903232
rs8057943
rs61622210
rs80235303
rs6489587
rs12972416
rs73597199
rs78305076
rs17354029
rs227452
rs75776206
rs10842705
rs529163274
rs111696805
rs80056411
rs117738211
rs9696823
rs112418980
All
−0.10
−0.05
0.00
0.05
MR leave−one−out sensitivity analysis for
'HVEM on TD CD8br ' on 'Juvenile arthritis'

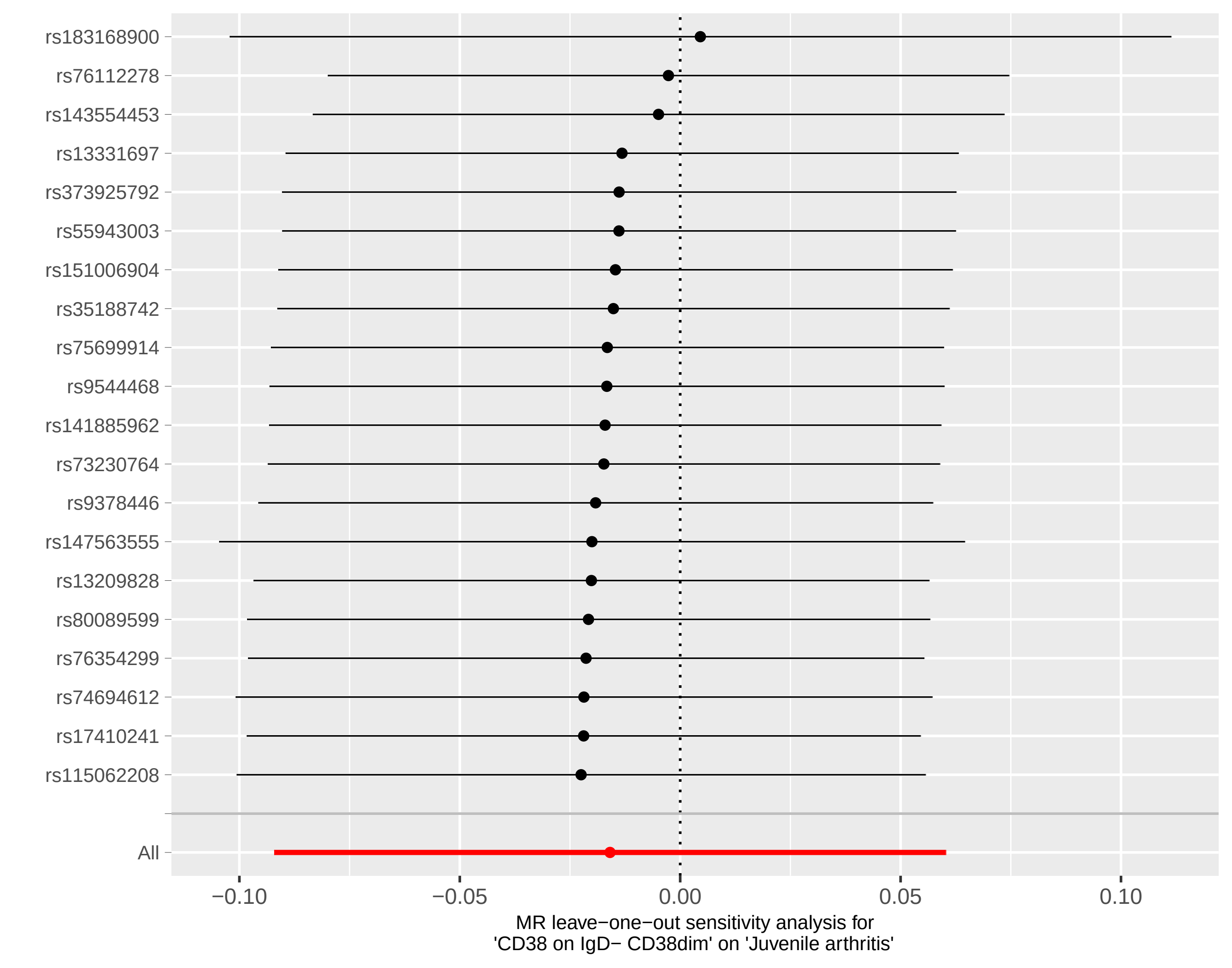
rs183168900
rs76112278
rs143554453
rs13331697
rs373925792
rs55943003
rs151006904
rs35188742
rs75699914
rs9544468
rs141885962
rs73230764
rs9378446
rs147563555
rs13209828
rs80089599
rs76354299
rs74694612
rs17410241
rs115062208
All
−0.10
−0.05
0.00
0.05
0.10
MR leave−one−out sensitivity analysis for
'CD38 on IgD− CD38dim' on 'Juvenile arthritis'

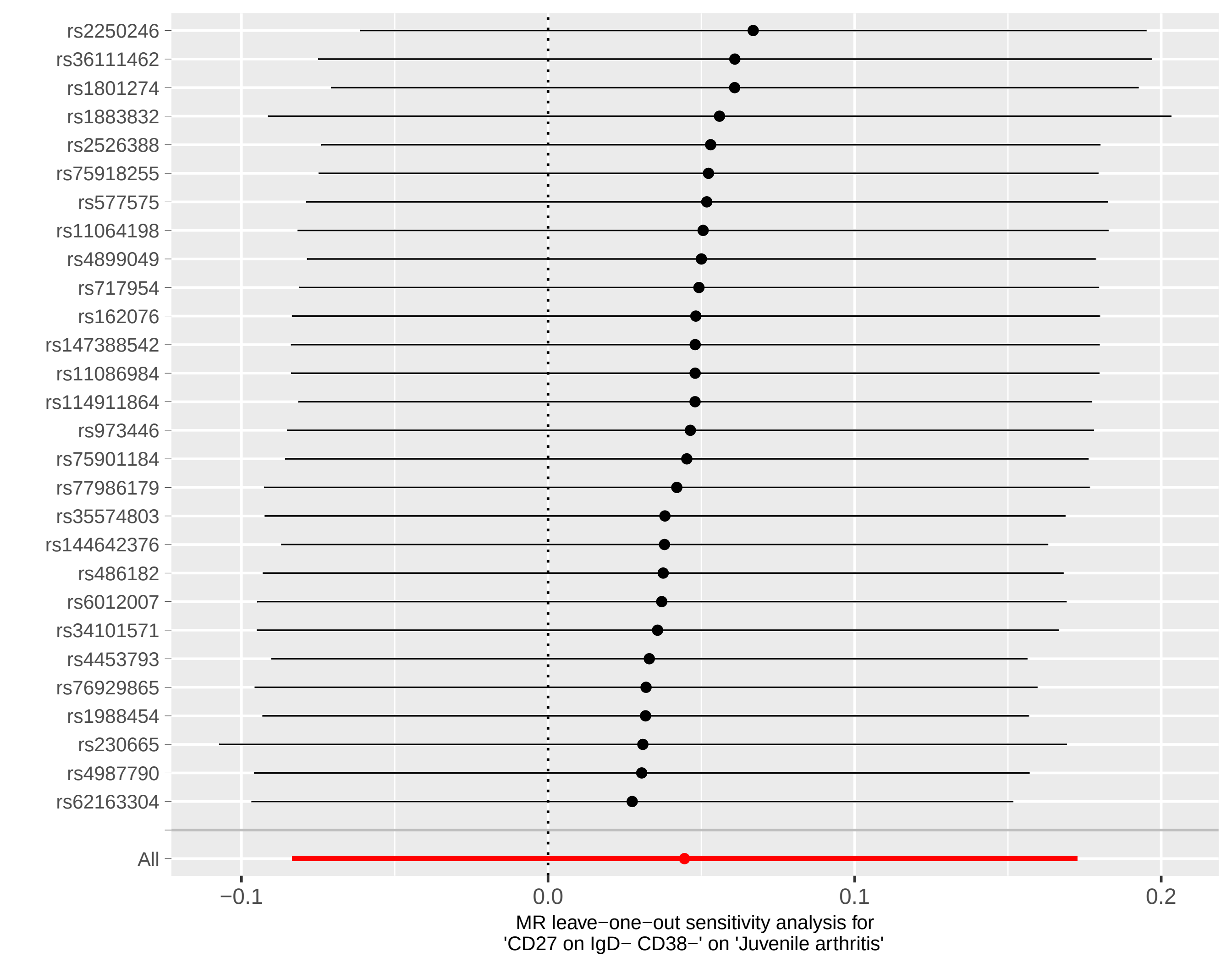
rs2250246
rs36111462
rs1801274
rs1883832
rs2526388
rs75918255
rs577575
rs11064198
rs4899049
rs717954
rs162076
rs147388542
rs11086984
rs114911864
rs973446
rs75901184
rs77986179
rs35574803
rs144642376
rs486182
rs6012007
rs34101571
rs4453793
rs76929865
rs1988454
rs230665
rs4987790
rs62163304
All
−0.1
0.0
0.1
0.2
MR leave−one−out sensitivity analysis for
'CD27 on IgD− CD38−' on 'Juvenile arthritis'

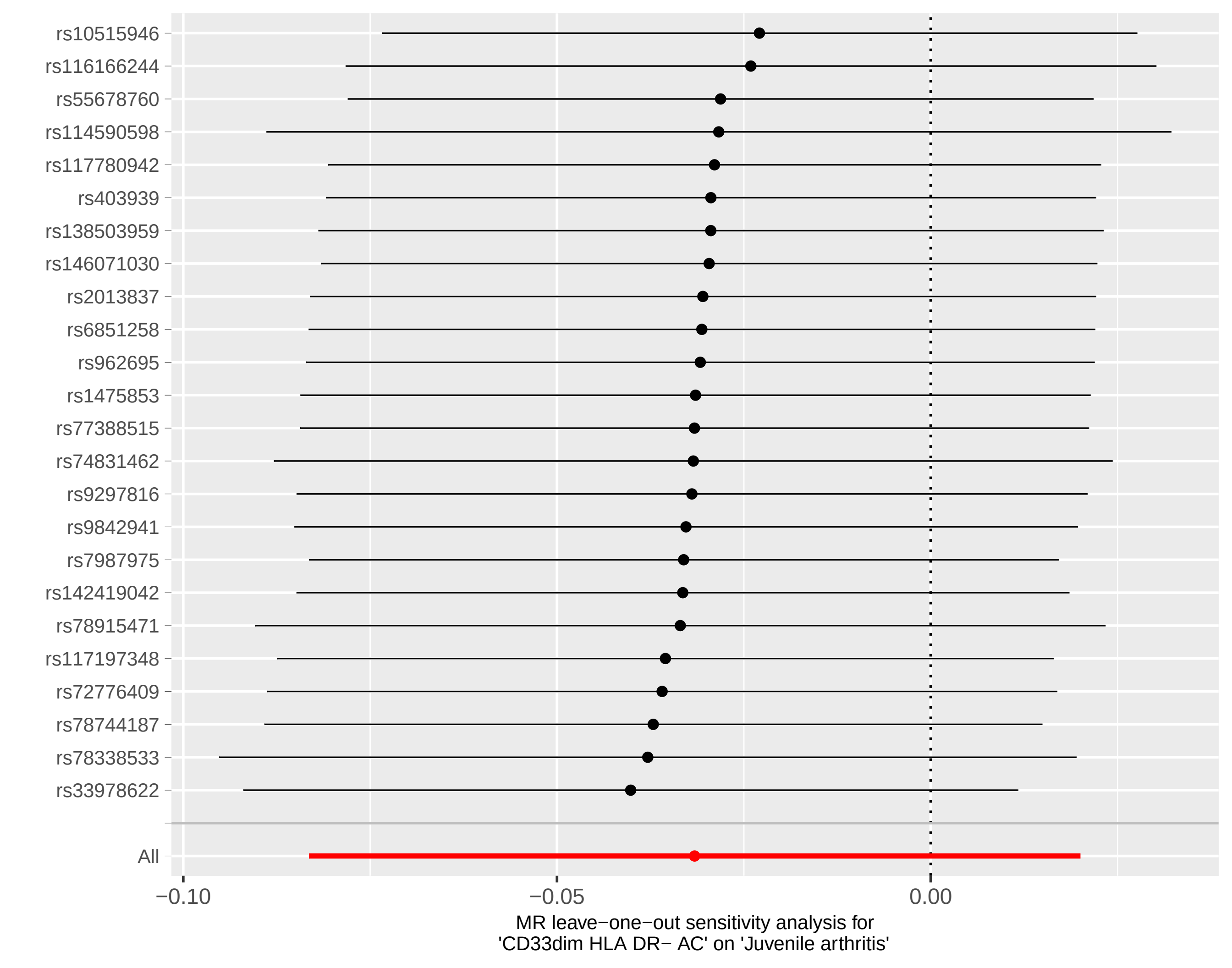
rs10515946
rs116166244
rs55678760
rs114590598
rs117780942
rs403939
rs138503959
rs146071030
rs2013837
rs6851258
rs962695
rs1475853
rs77388515
rs74831462
rs9297816
rs9842941
rs7987975
rs142419042
rs78915471
rs117197348
rs72776409
rs78744187
rs78338533
rs33978622
All
−0.10
−0.05
0.00
MR leave−one−out sensitivity analysis for
'CD33dim HLA DR− AC' on 'Juvenile arthritis'

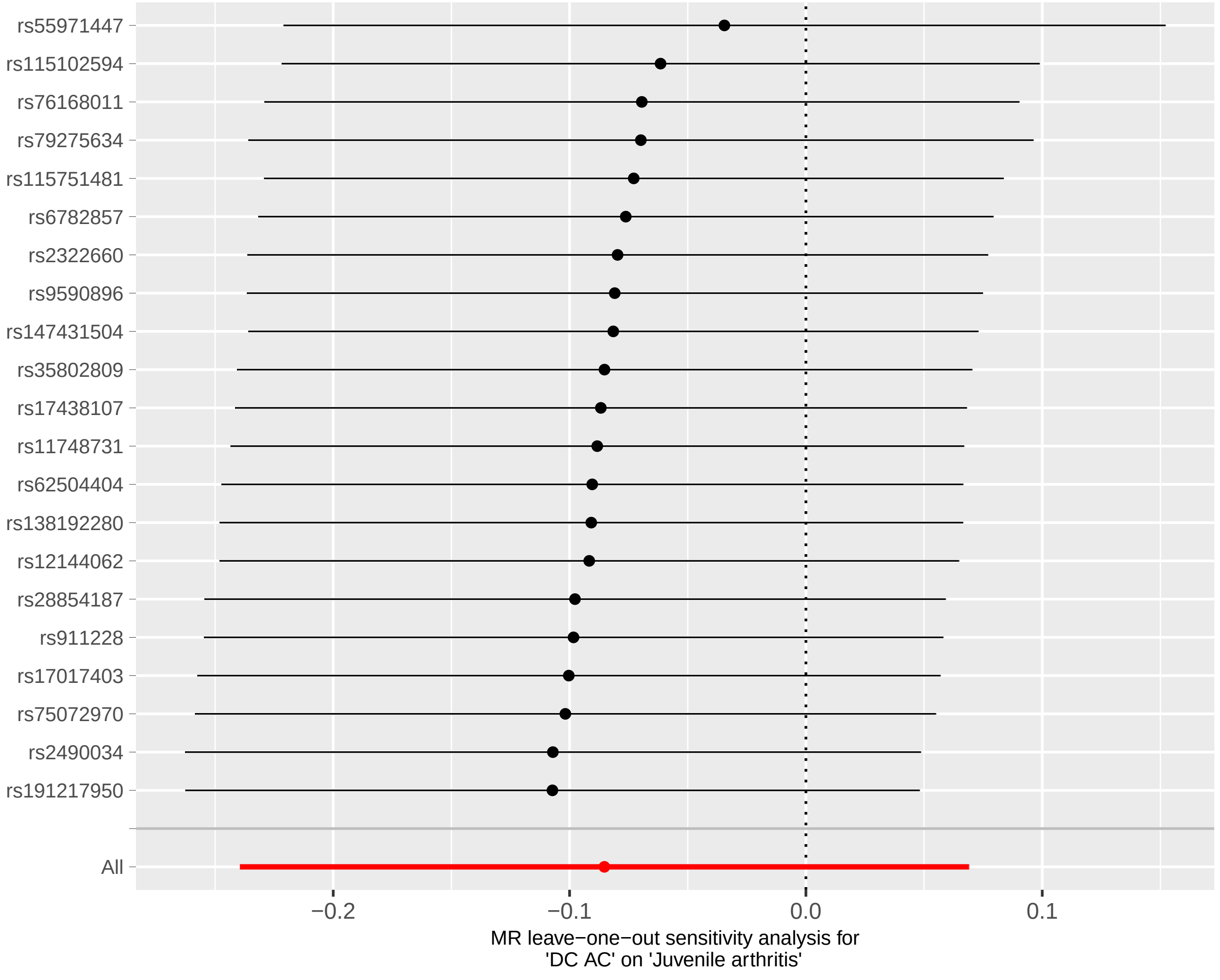

rs55971447
rs115102594
rs76168011
rs79275634
rs115751481
rs6782857
rs2322660
rs9590896
rs147431504
rs35802809
rs17438107
rs11748731
rs62504404
rs138192280
rs12144062
rs28854187
rs911228
rs17017403
rs75072970
rs2490034
rs191217950
All
−0.2
−0.1
0.0
0.1
MR leave−one−out sensitivity analysis for
'DC AC' on 'Juvenile arthritis'

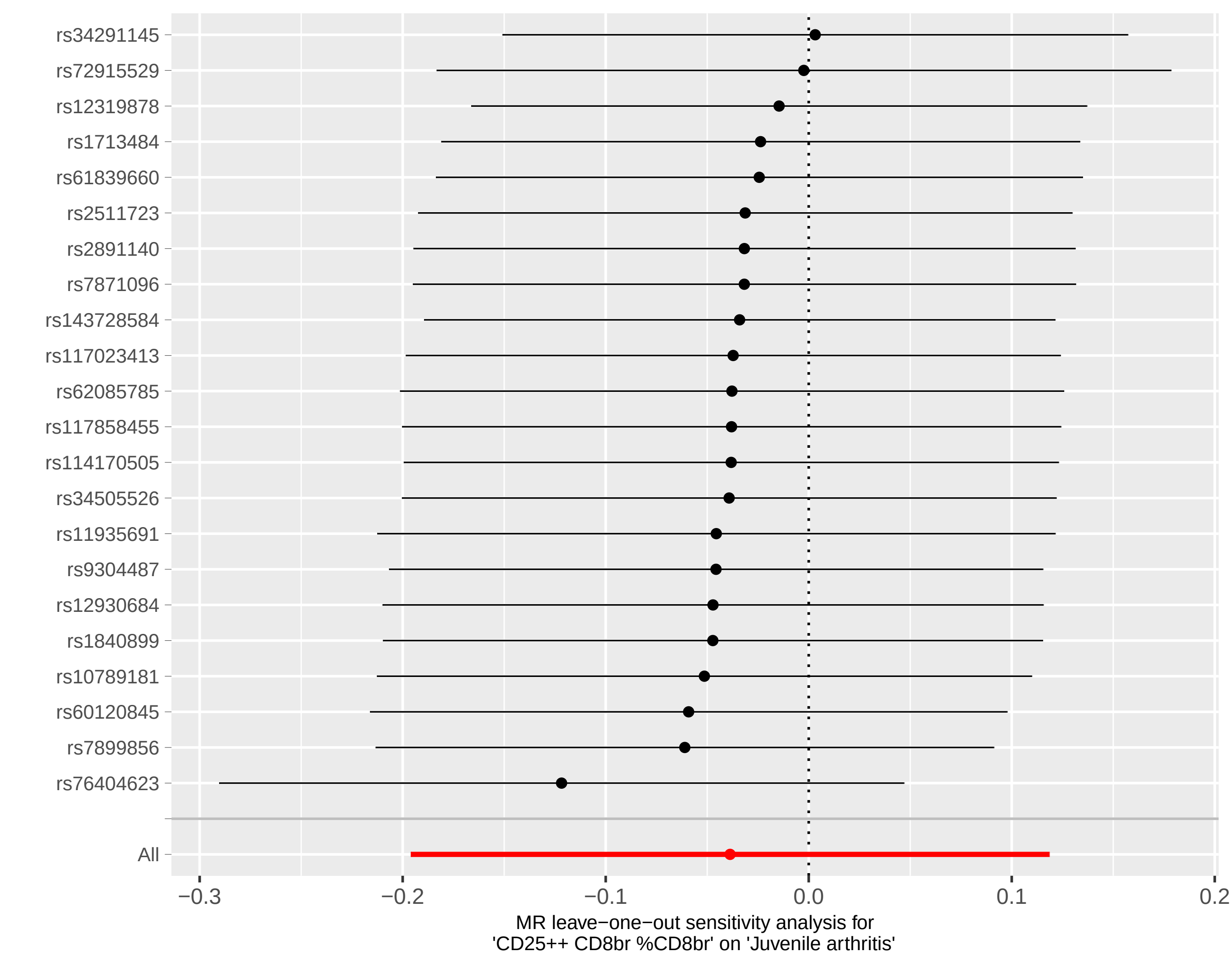

rs34291145
rs72915529
rs12319878
rs1713484
rs61839660
rs2511723
rs2891140
rs7871096
rs143728584
rs117023413
rs62085785
rs117858455
rs114170505
rs34505526
rs11935691
rs9304487
rs12930684
rs1840899
rs10789181
rs60120845
rs7899856
rs76404623
All
−0.3
−0.2
−0.1
0.0
0.1
0.2
MR leave−one−out sensitivity analysis for
'CD25++ CD8br %CD8br' on 'Juvenile arthritis'

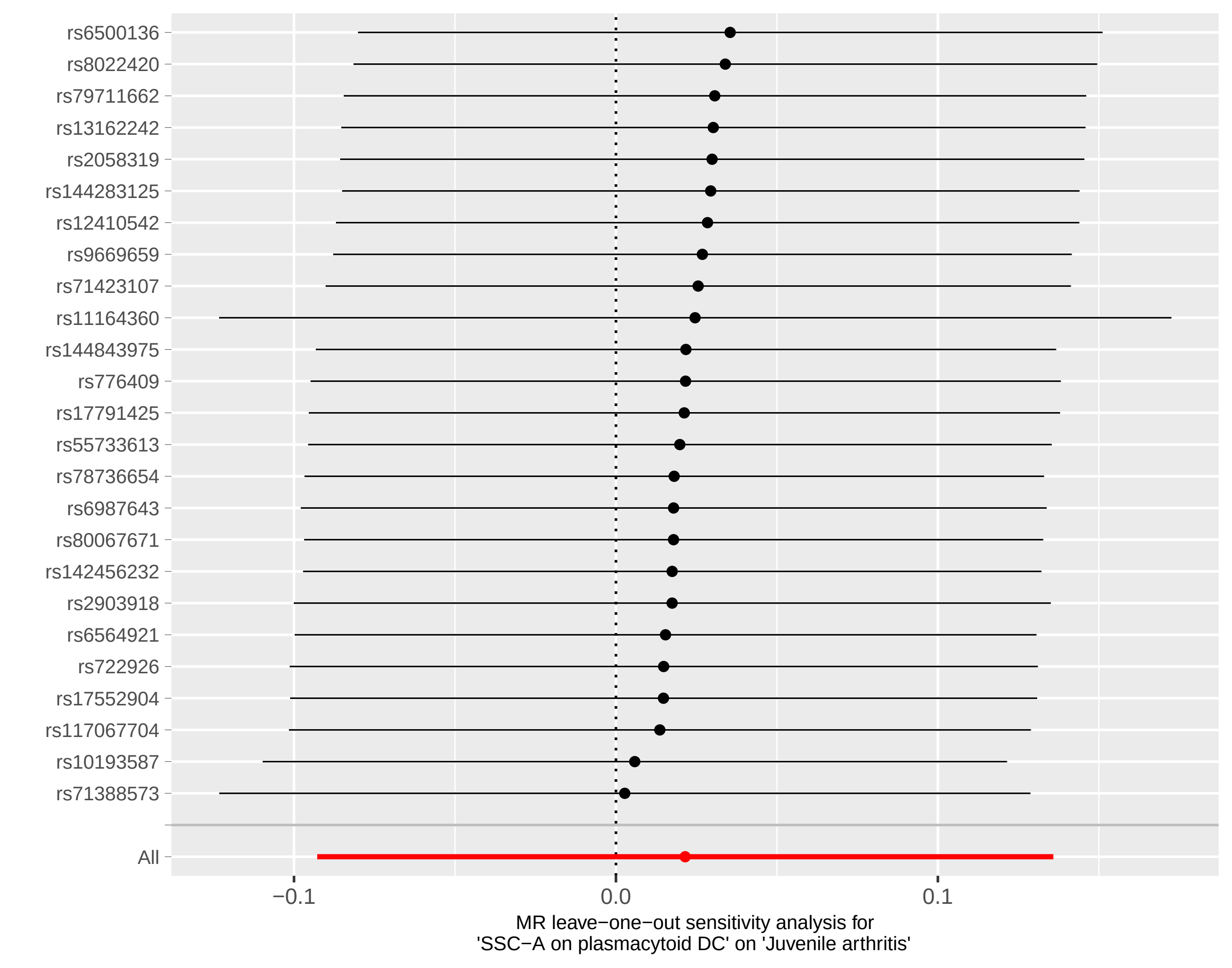
rs6500136
rs8022420
rs79711662
rs13162242
rs2058319
rs144283125
rs12410542
rs9669659
rs71423107
rs11164360
rs144843975
rs776409
rs17791425
rs55733613
rs78736654
rs6987643
rs80067671
rs142456232
rs2903918
rs6564921
rs722926
rs17552904
rs117067704
rs10193587
rs71388573
All
−0.1
0.0
0.1
MR leave−one−out sensitivity analysis for
'SSC−A on plasmacytoid DC' on 'Juvenile arthritis'

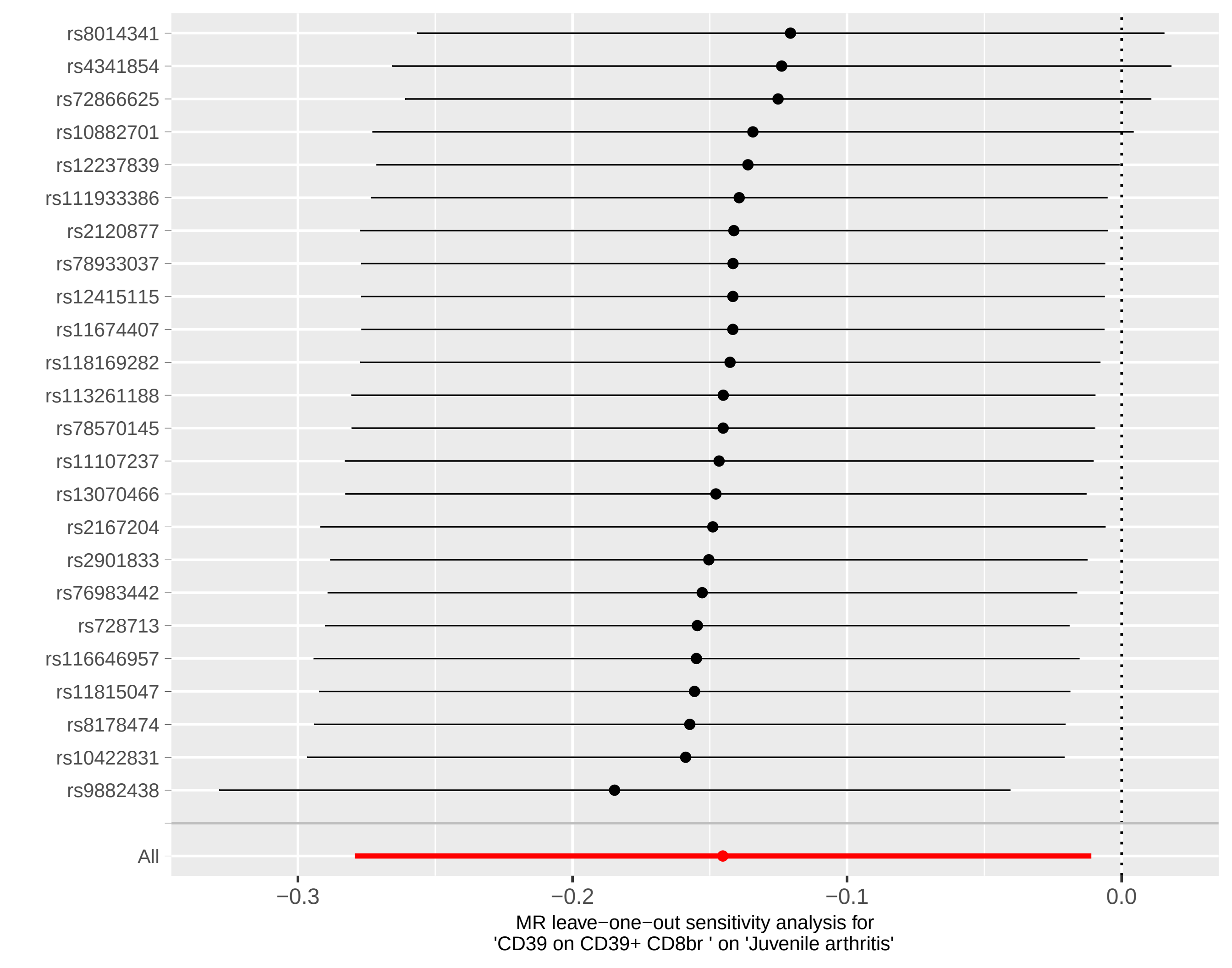
rs8014341
rs4341854
rs72866625
rs10882701
rs12237839
rs111933386
rs2120877
rs78933037
rs12415115
rs11674407
rs118169282
rs113261188
rs78570145
rs11107237
rs13070466
rs2167204
rs2901833
rs76983442
rs728713
rs116646957
rs11815047
rs8178474
rs10422831
rs9882438
All
−0.3
−0.2
−0.1
0.0
MR leave−one−out sensitivity analysis for
'CD39 on CD39+ CD8br ' on 'Juvenile arthritis'

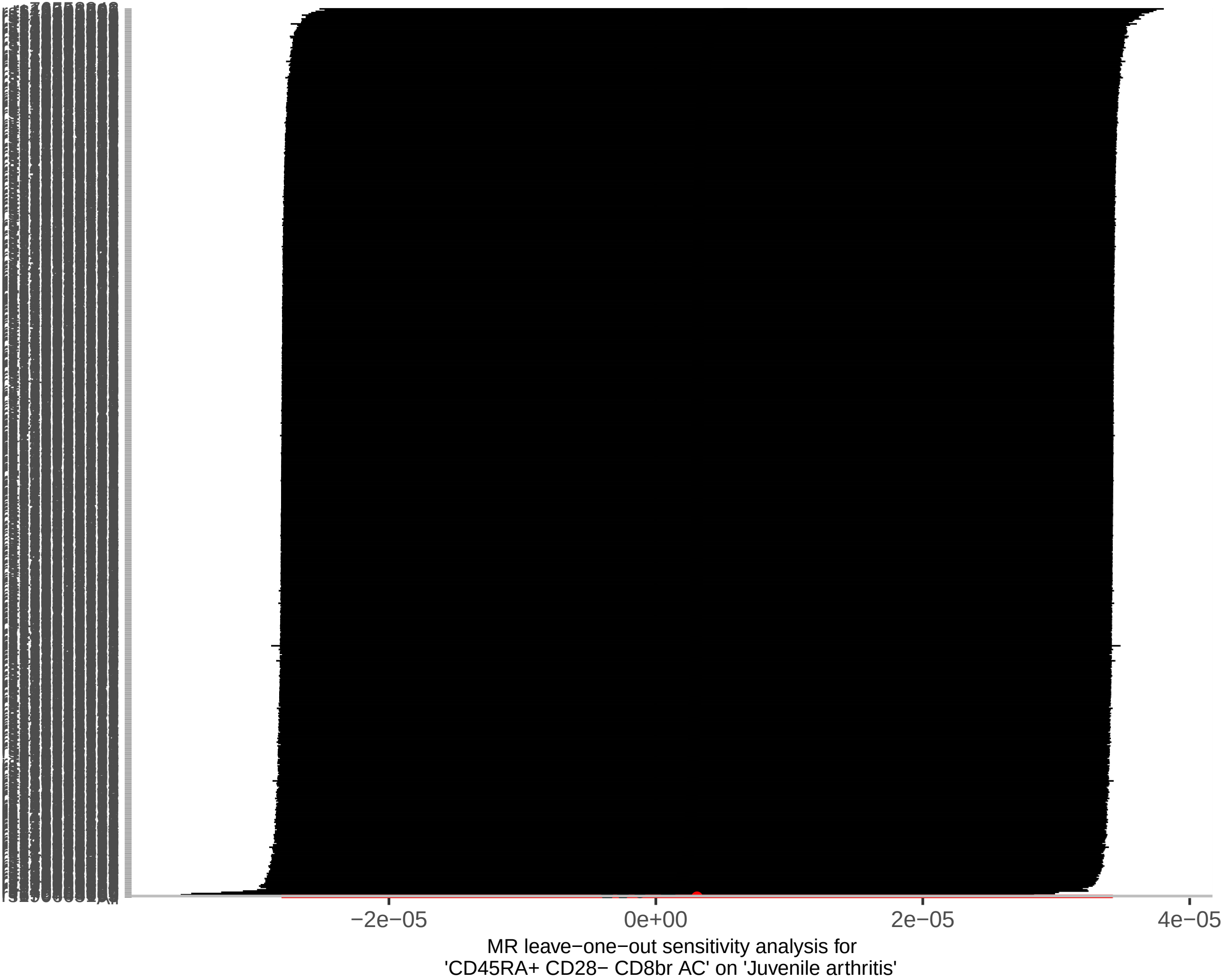

−2e−05
0e+00
2e−05
4e−05
MR leave−one−out sensitivity analysis for
'CD45RA+ CD28− CD8br AC' on 'Juvenile arthritis'

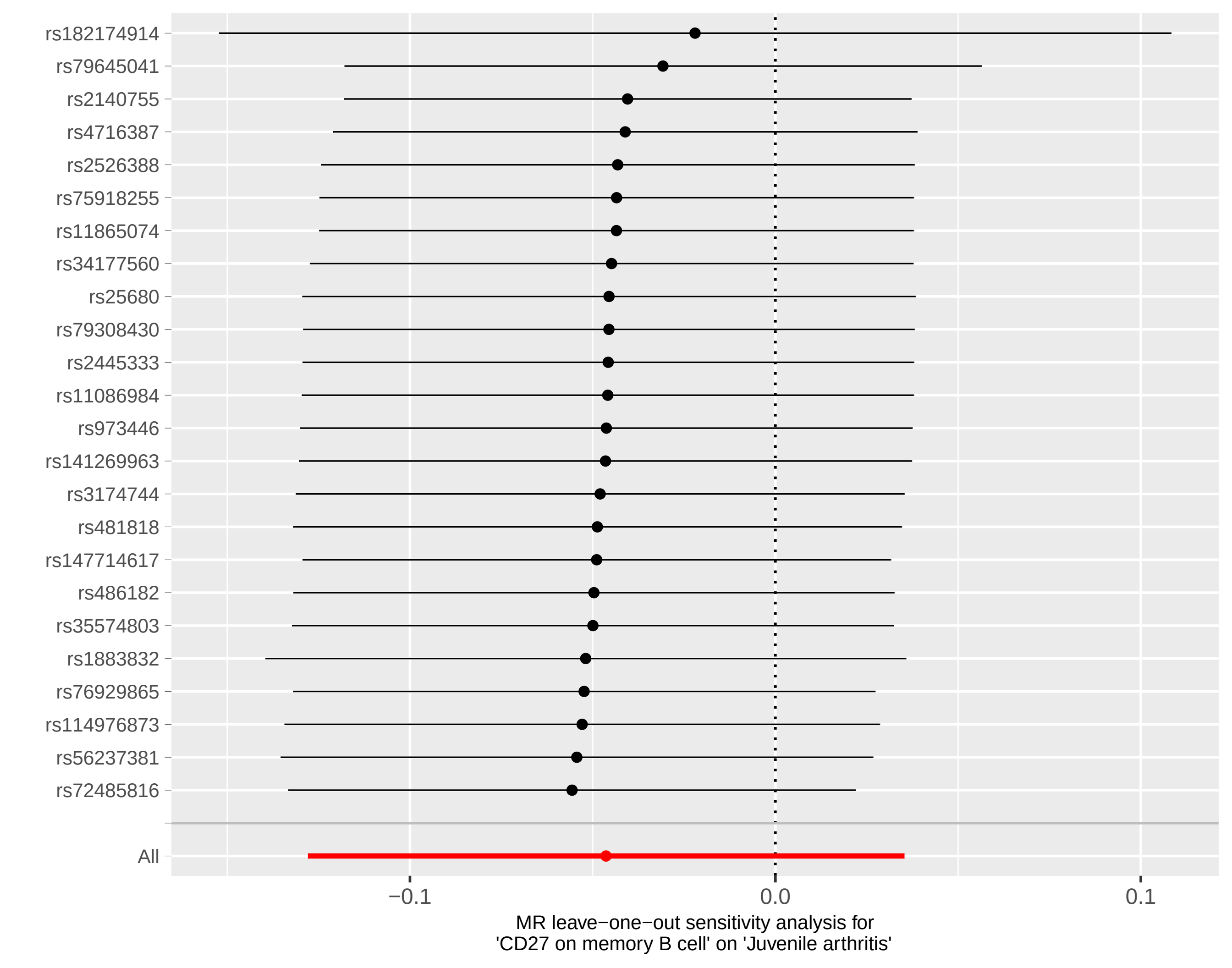
rs182174914
rs79645041
rs2140755
rs4716387
rs2526388
rs75918255
rs11865074
rs34177560
rs25680
rs79308430
rs2445333
rs11086984
rs973446
rs141269963
rs3174744
rs481818
rs147714617
rs486182
rs35574803
rs1883832
rs76929865
rs114976873
rs56237381
rs72485816
All
−0.1
0.0
0.1
MR leave−one−out sensitivity analysis for
'CD27 on memory B cell' on 'Juvenile arthritis'

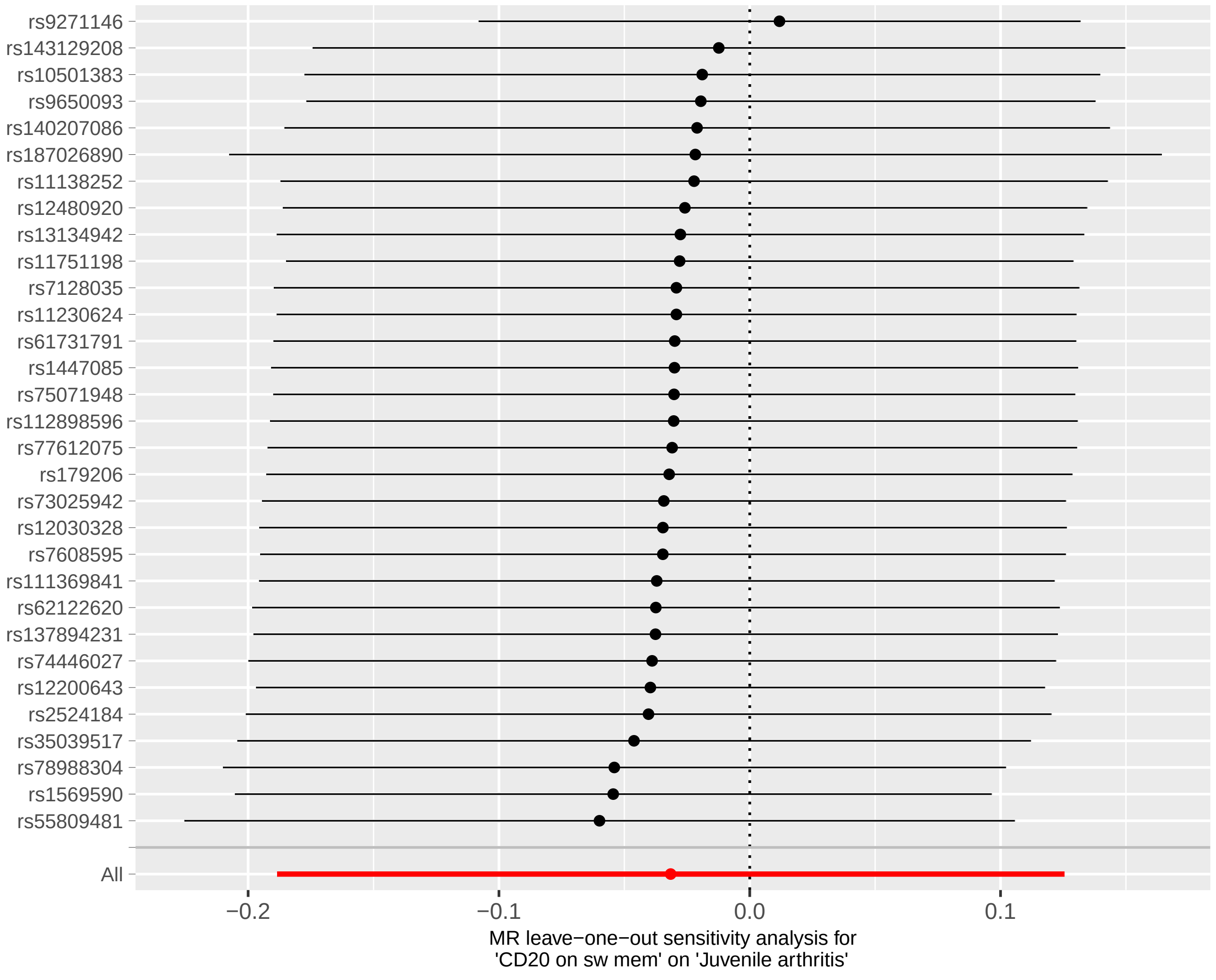
rs9271146
rs143129208
rs10501383
rs9650093
rs140207086
rs187026890
rs11138252
rs12480920
rs13134942
rs11751198
rs7128035
rs11230624
rs61731791
rs1447085
rs75071948
rs112898596
rs77612075
rs179206
rs73025942
rs12030328
rs7608595
rs111369841
rs62122620
rs137894231
rs74446027
rs12200643
rs2524184
rs35039517
rs78988304
rs1569590
rs55809481
All
−0.2
−0.1
0.0
0.1
MR leave−one−out sensitivity analysis for
'CD20 on sw mem' on 'Juvenile arthritis'

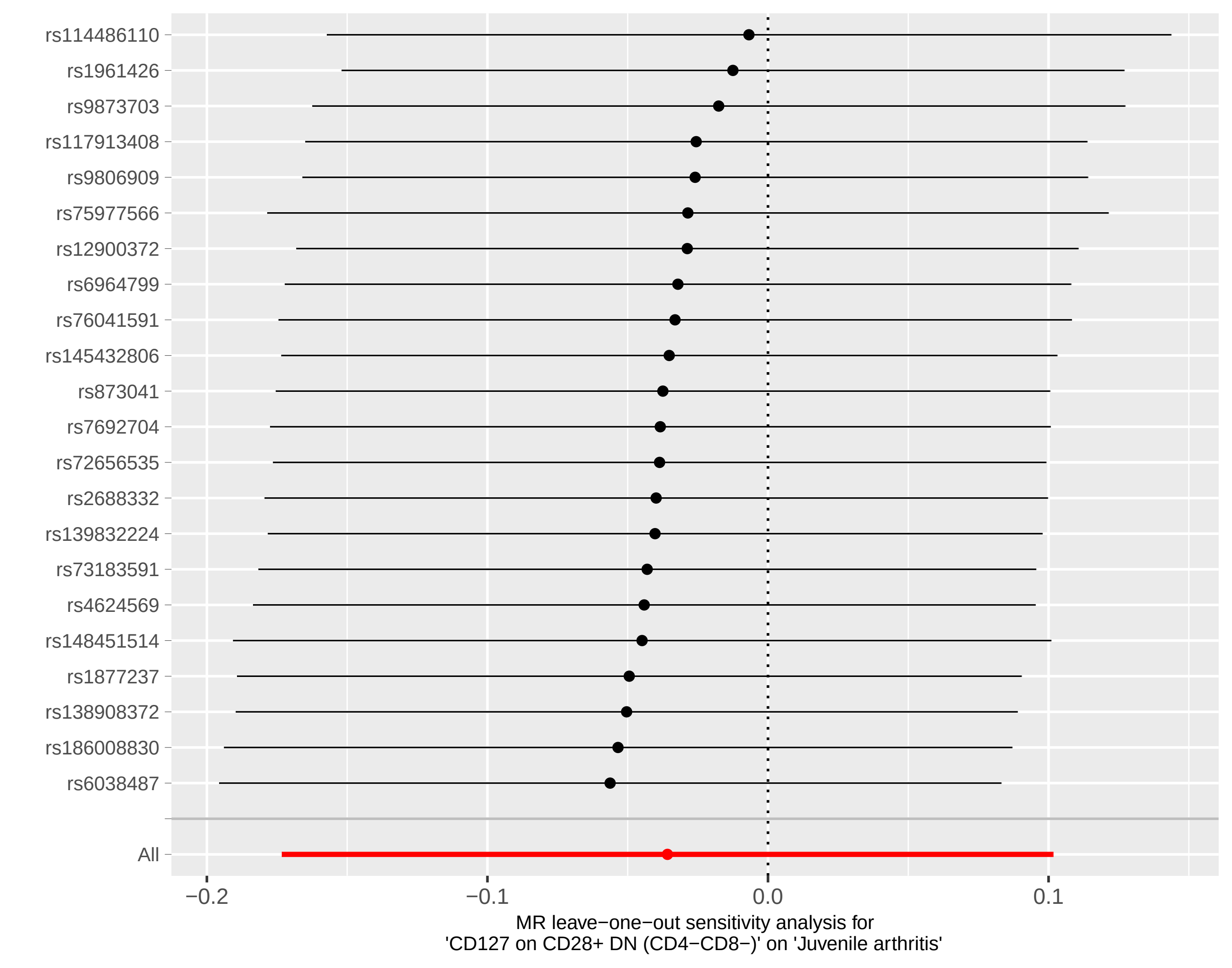

rs114486110
rs1961426
rs9873703
rs117913408
rs9806909
rs75977566
rs12900372
rs6964799
rs76041591
rs145432806
rs873041
rs7692704
rs72656535
rs2688332
rs139832224
rs73183591
rs4624569
rs148451514
rs1877237
rs138908372
rs186008830
rs6038487
All
−0.2
−0.1
0.0
0.1
MR leave−one−out sensitivity analysis for
'CD127 on CD28+ DN (CD4−CD8−)' on 'Juvenile arthritis'

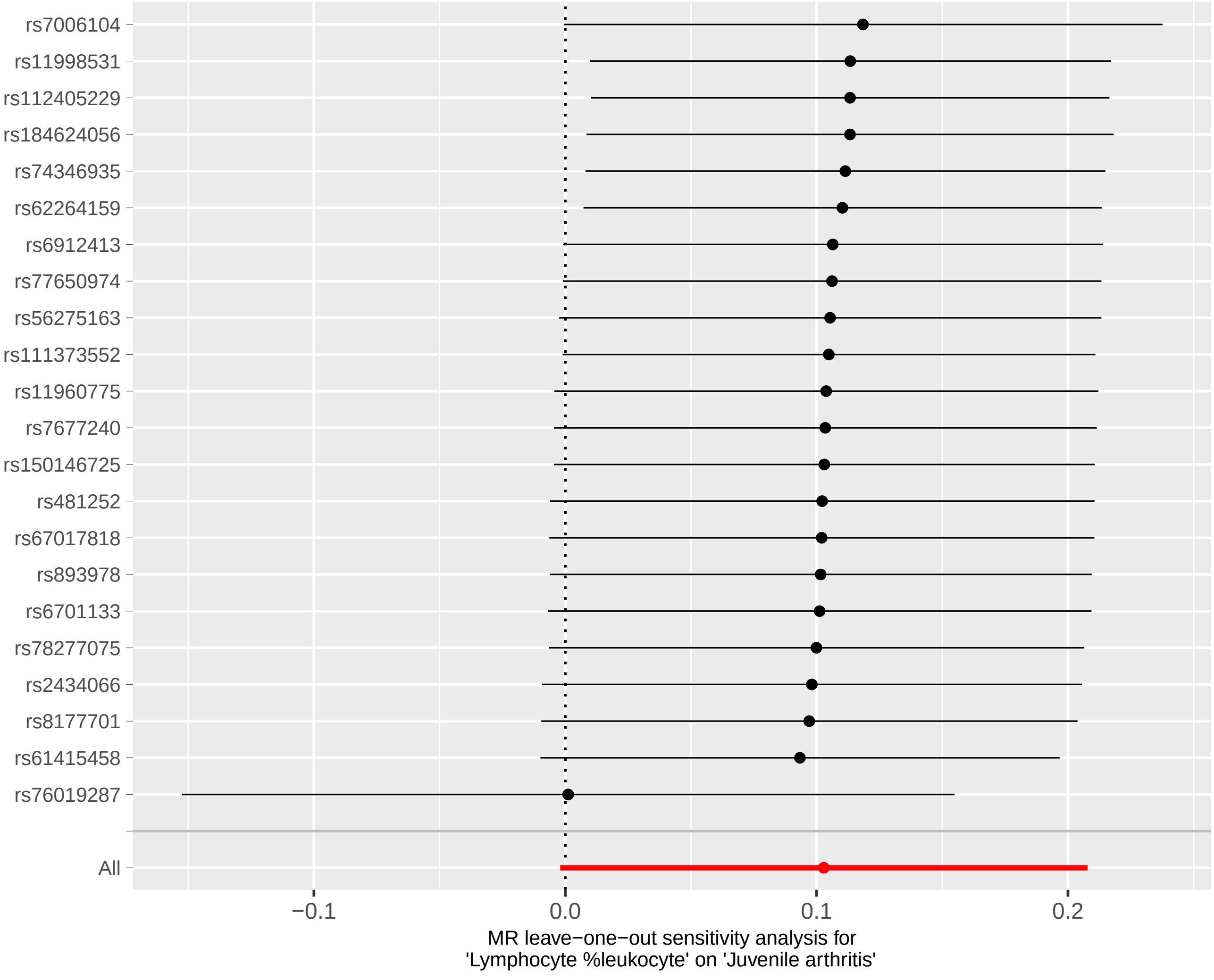
rs7006104
rs11998531
rs112405229
rs184624056
rs74346935
rs62264159
rs6912413
rs77650974
rs56275163
rs111373552
rs11960775
rs7677240
rs150146725
rs481252
rs67017818
rs893978
rs6701133
rs78277075
rs2434066
rs8177701
rs61415458
rs76019287
All
−0.1
0.0
0.1
0.2
MR leave−one−out sensitivity analysis for
'Lymphocyte %leukocyte' on 'Juvenile arthritis'

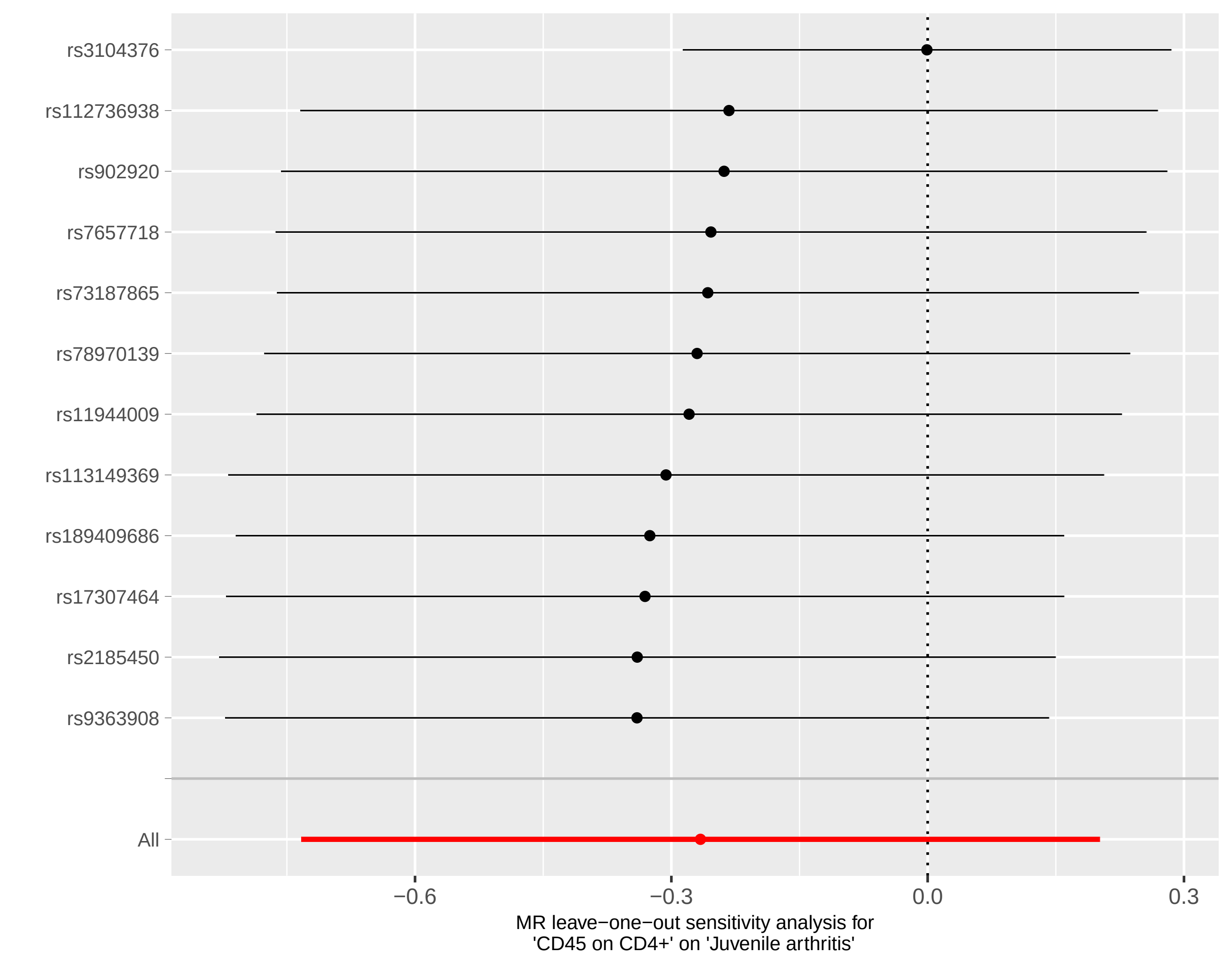
rs3104376
rs112736938
rs902920
rs7657718
rs73187865
rs78970139
rs11944009
rs113149369
rs189409686
rs17307464
rs2185450
rs9363908
All
−0.6
−0.3
0.0
0.3
MR leave−one−out sensitivity analysis for
'CD45 on CD4+' on 'Juvenile arthritis'

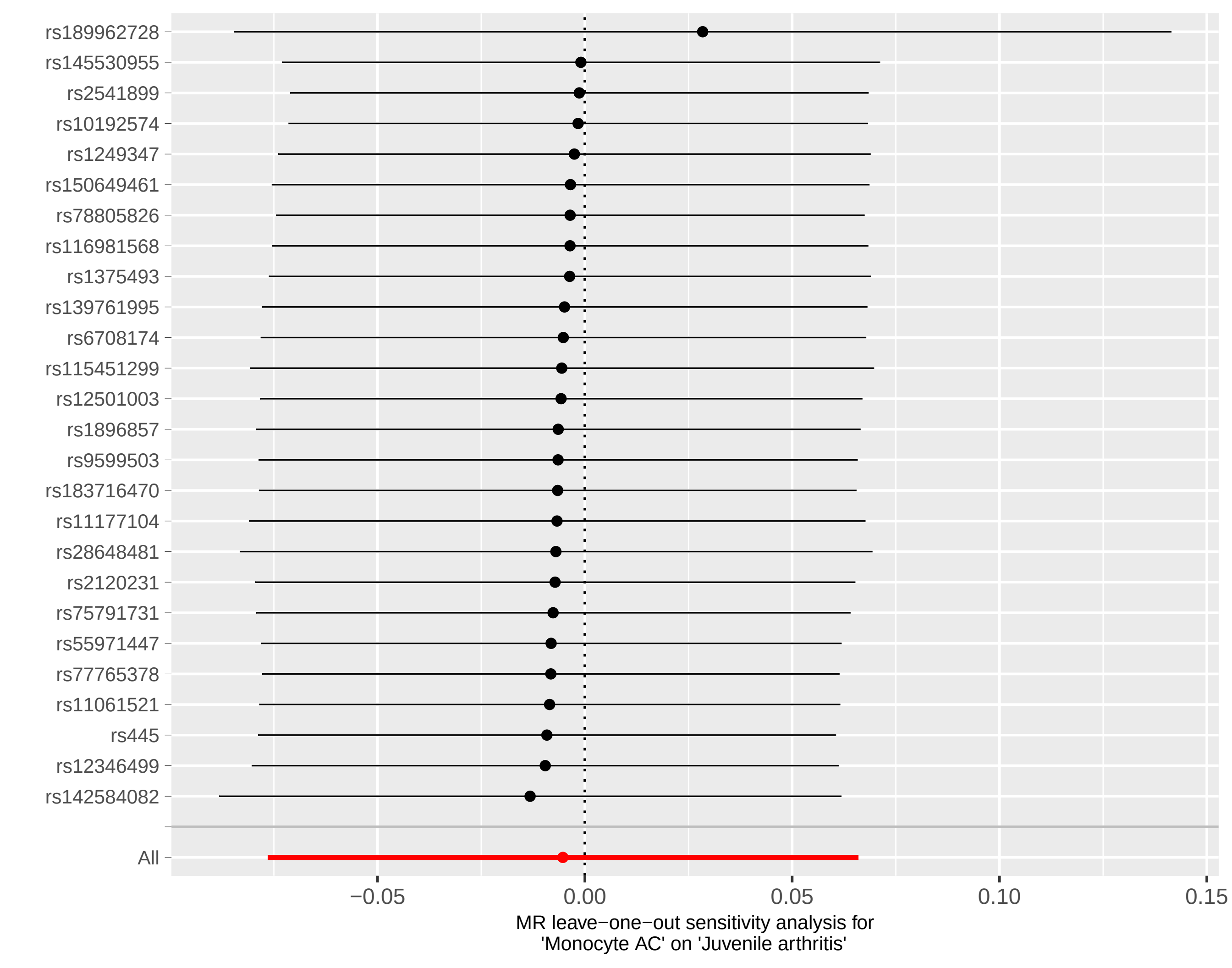

rs189962728
rs145530955
rs2541899
rs10192574
rs1249347
rs150649461
rs78805826
rs116981568
rs1375493
rs139761995
rs6708174
rs115451299
rs12501003
rs1896857
rs9599503
rs183716470
rs11177104
rs28648481
rs2120231
rs75791731
rs55971447
rs77765378
rs11061521
rs445
rs12346499
rs142584082
All
−0.05
0.00
0.05
0.10
0.15
MR leave−one−out sensitivity analysis for
'Monocyte AC' on 'Juvenile arthritis'

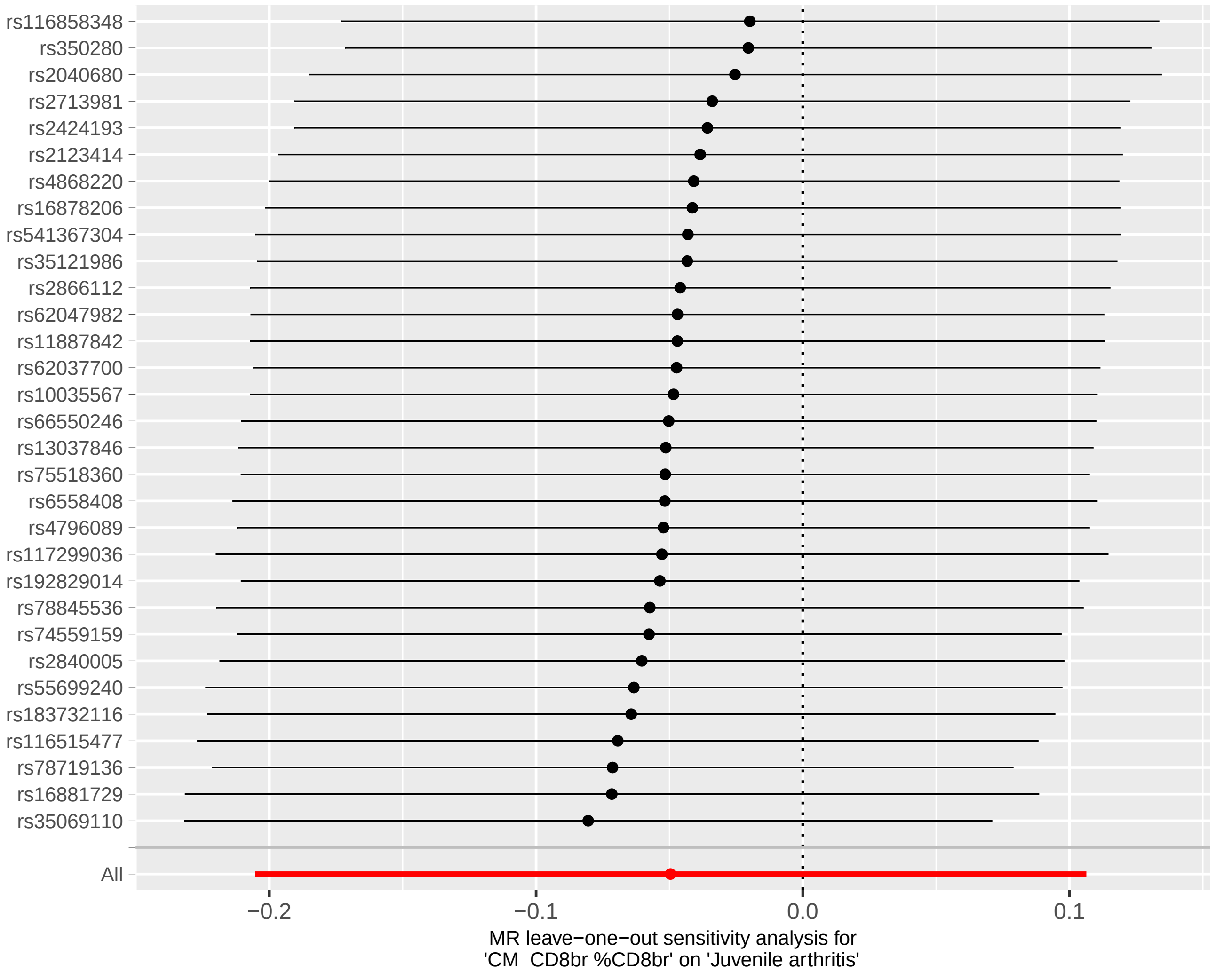
rs116858348
rs350280
rs2040680
rs2713981
rs2424193
rs2123414
rs4868220
rs16878206
rs541367304
rs35121986
rs2866112
rs62047982
rs11887842
rs62037700
rs10035567
rs66550246
rs13037846
rs75518360
rs6558408
rs4796089
rs117299036
rs192829014
rs78845536
rs74559159
rs2840005
rs55699240
rs183732116
rs116515477
rs78719136
rs16881729
rs35069110
All
−0.2
−0.1
0.0
0.1
MR leave−one−out sensitivity analysis for
'CM CD8br %CD8br' on 'Juvenile arthritis'

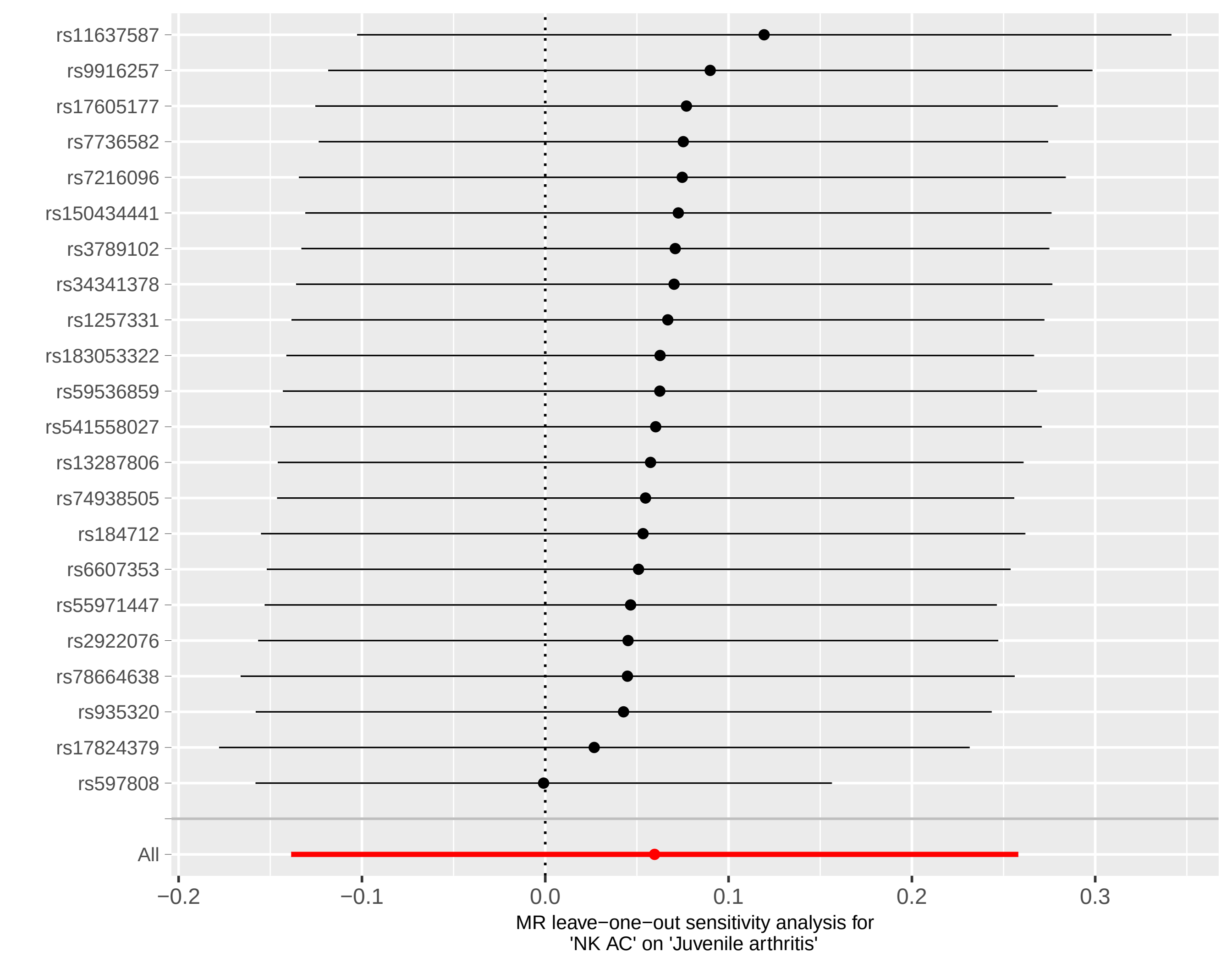

rs11637587
rs9916257
rs17605177
rs7736582
rs7216096
rs150434441
rs3789102
rs34341378
rs1257331
rs183053322
rs59536859
rs541558027
rs13287806
rs74938505
rs184712
rs6607353
rs55971447
rs2922076
rs78664638
rs935320
rs17824379
rs597808
All
−0.2
−0.1
0.0
0.1
0.2
0.3
MR leave−one−out sensitivity analysis for
'NK AC' on 'Juvenile arthritis'

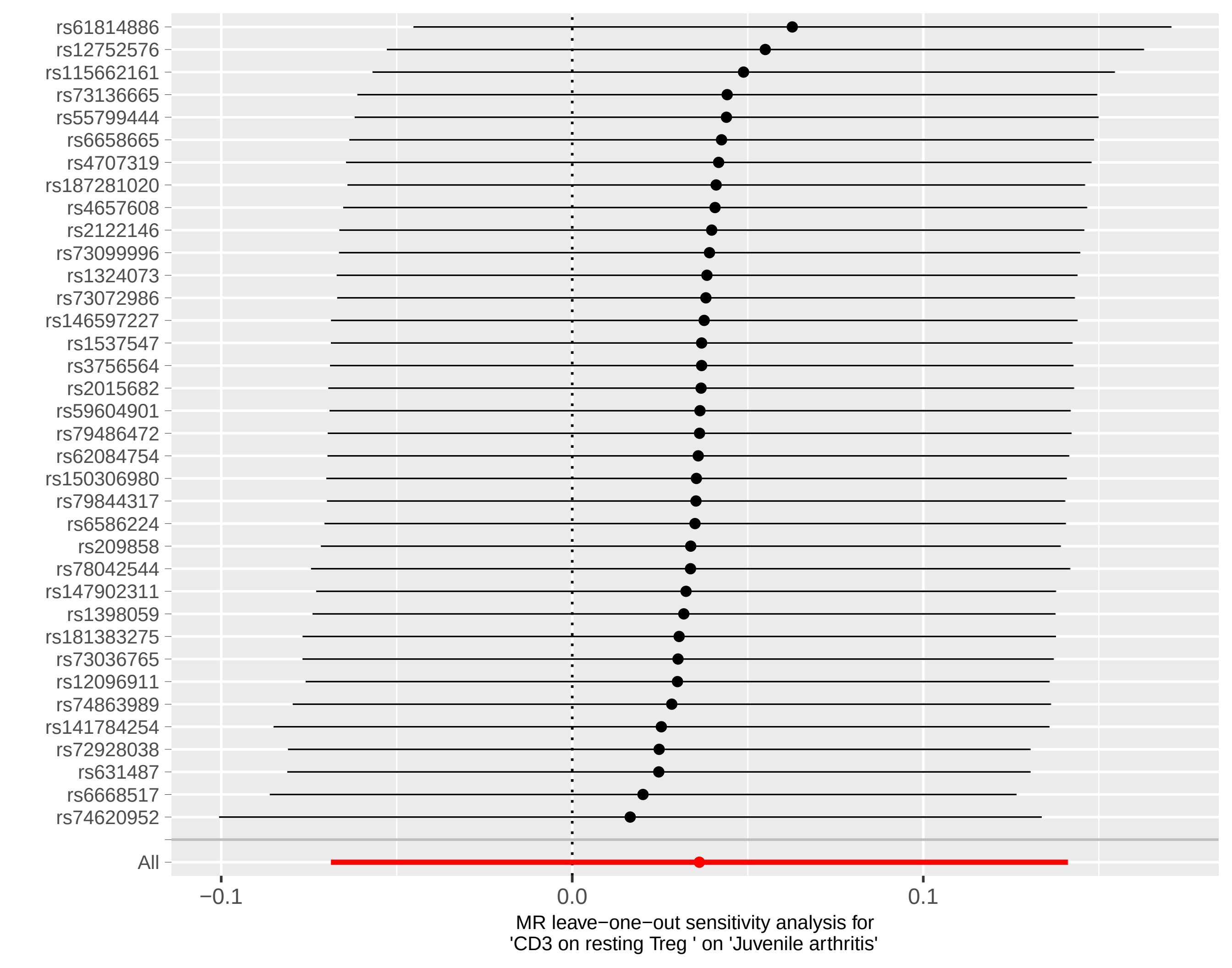
rs61814886
rs12752576
rs115662161
rs73136665
rs55799444
rs6658665
rs4707319
rs187281020
rs4657608
rs2122146
rs73099996
rs1324073
rs73072986
rs146597227
rs1537547
rs3756564
rs2015682
rs59604901
rs79486472
rs62084754
rs150306980
rs79844317
rs6586224
rs209858
rs78042544
rs147902311
rs1398059
rs181383275
rs73036765
rs12096911
rs74863989
rs141784254
rs72928038
rs631487
rs6668517
rs74620952
All
−0.1
0.0
0.1
MR leave−one−out sensitivity analysis for
'CD3 on resting Treg ' on 'Juvenile arthritis'

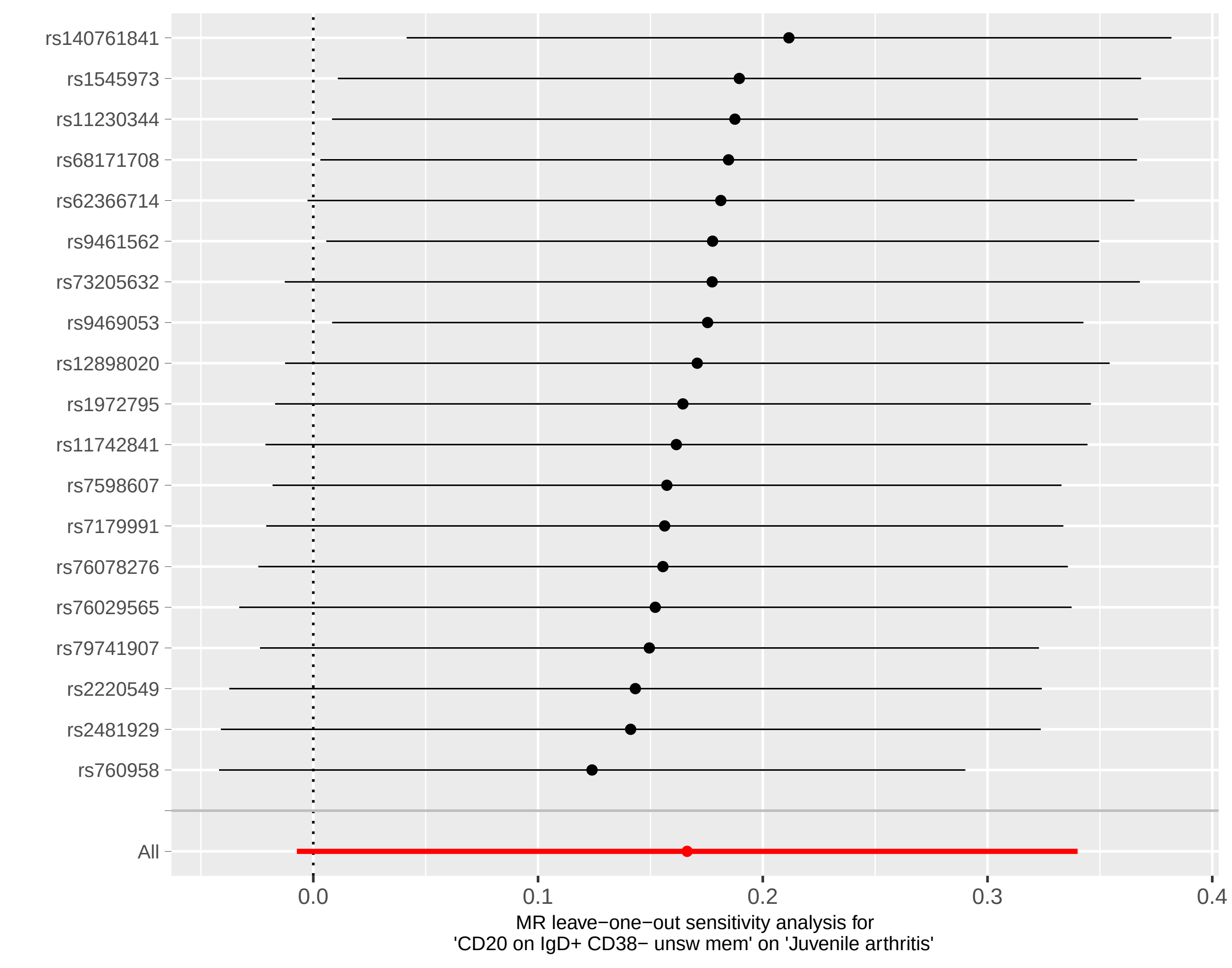
rs140761841
rs1545973
rs11230344
rs68171708
rs62366714
rs9461562
rs73205632
rs9469053
rs12898020
rs1972795
rs11742841
rs7598607
rs7179991
rs76078276
rs76029565
rs79741907
rs2220549
rs2481929
rs760958
All
0.0
0.1
0.2
0.3
0.4
MR leave−one−out sensitivity analysis for
'CD20 on IgD+ CD38− unsw mem' on 'Juvenile arthritis'

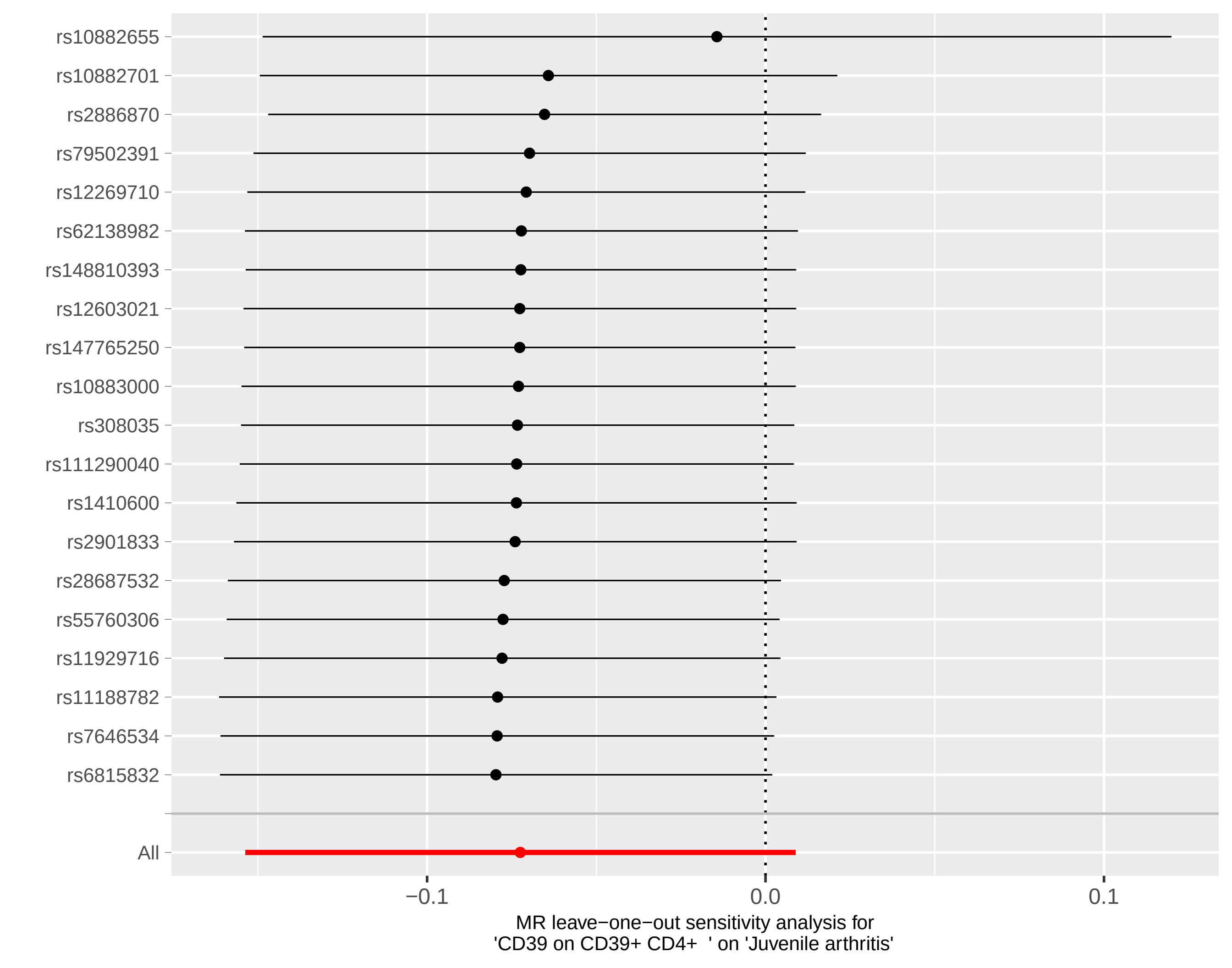
rs10882655
rs10882701
rs2886870
rs79502391
rs12269710
rs62138982
rs148810393
rs12603021
rs147765250
rs10883000
rs308035
rs111290040
rs1410600
rs2901833
rs28687532
rs55760306
rs11929716
rs11188782
rs7646534
rs6815832
All
−0.1
0.0
0.1
MR leave−one−out sensitivity analysis for
'CD39 on CD39+ CD4+ ' on 'Juvenile arthritis'

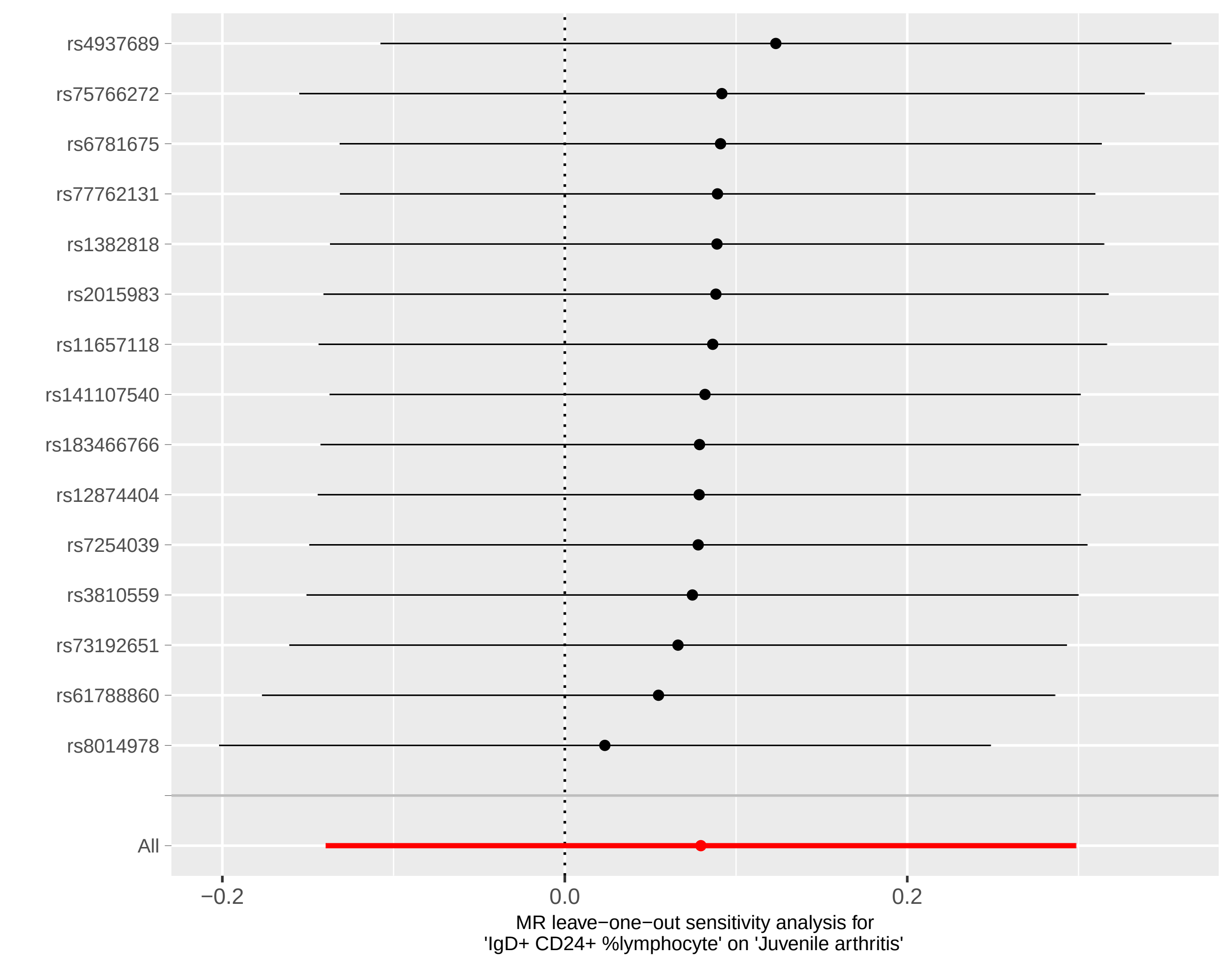
rs4937689
rs75766272
rs6781675
rs77762131
rs1382818
rs2015983
rs11657118
rs141107540
rs183466766
rs12874404
rs7254039
rs3810559
rs73192651
rs61788860
rs8014978
All
−0.2
0.0
0.2
MR leave−one−out sensitivity analysis for
'IgD+ CD24+ %lymphocyte' on 'Juvenile arthritis'

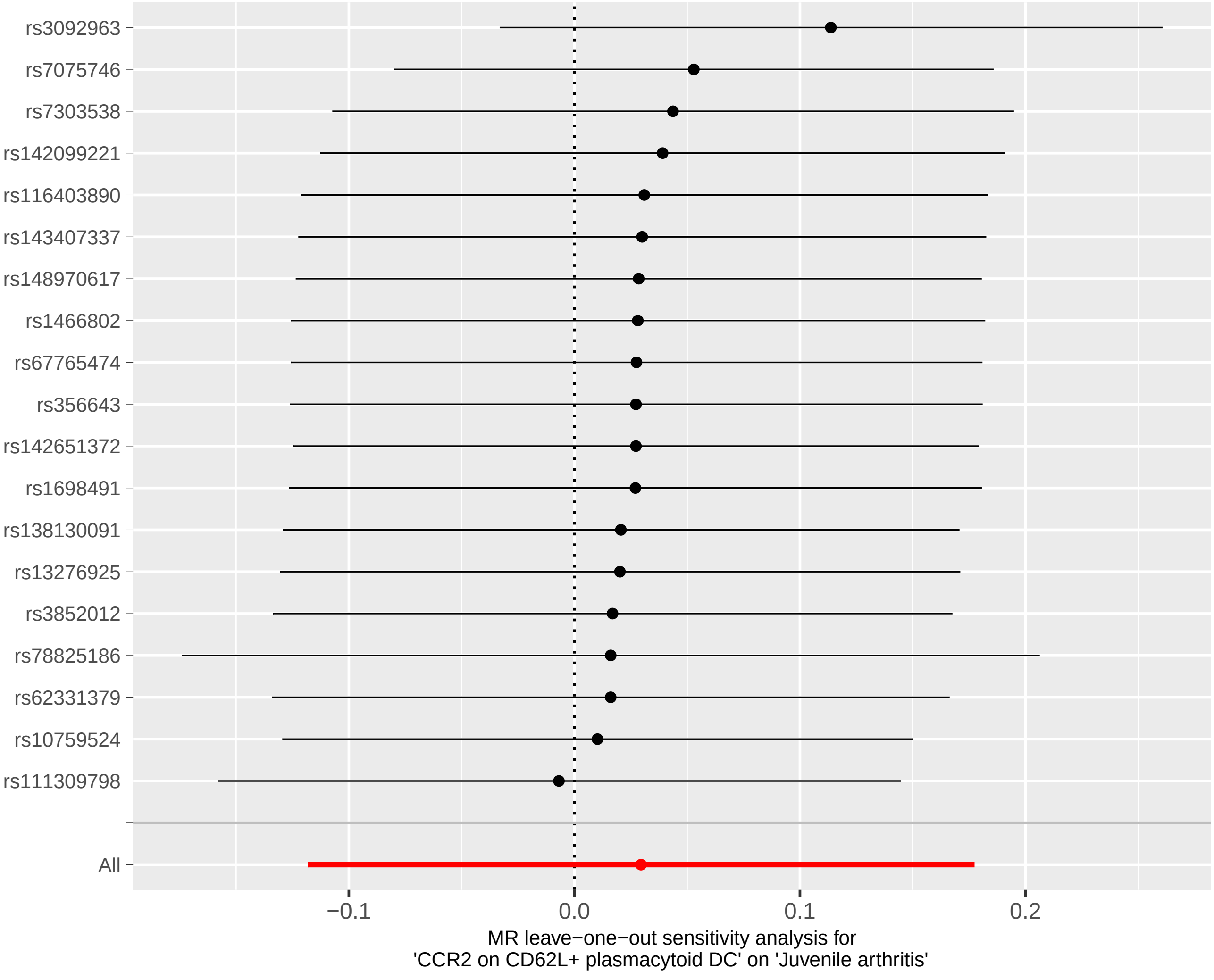
rs3092963
rs7075746
rs7303538
rs142099221
rs116403890
rs143407337
rs148970617
rs1466802
rs67765474
rs356643
rs142651372
rs1698491
rs138130091
rs13276925
rs3852012
rs78825186
rs62331379
rs10759524
rs111309798
All
−0.1
0.0
0.1
0.2
MR leave−one−out sensitivity analysis for
'CCR2 on CD62L+ plasmacytoid DC' on 'Juvenile arthritis'

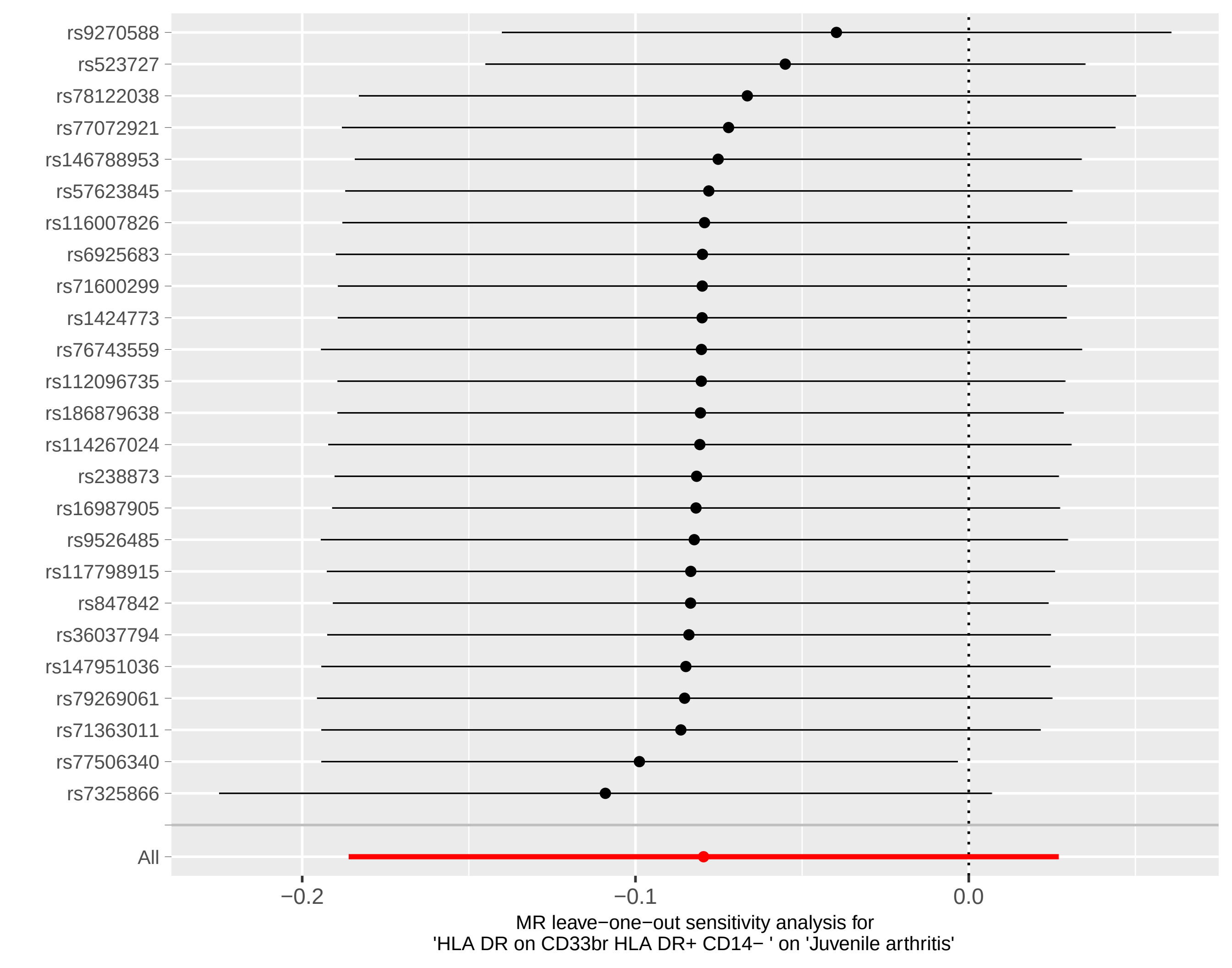
rs9270588
rs523727
rs78122038
rs77072921
rs146788953
rs57623845
rs116007826
rs6925683
rs71600299
rs1424773
rs76743559
rs112096735
rs186879638
rs114267024
rs238873
rs16987905
rs9526485
rs117798915
rs847842
rs36037794
rs147951036
rs79269061
rs71363011
rs77506340
rs7325866
All
−0.2
−0.1
0.0
MR leave−one−out sensitivity analysis for
'HLA DR on CD33br HLA DR+ CD14− ' on 'Juvenile arthritis'

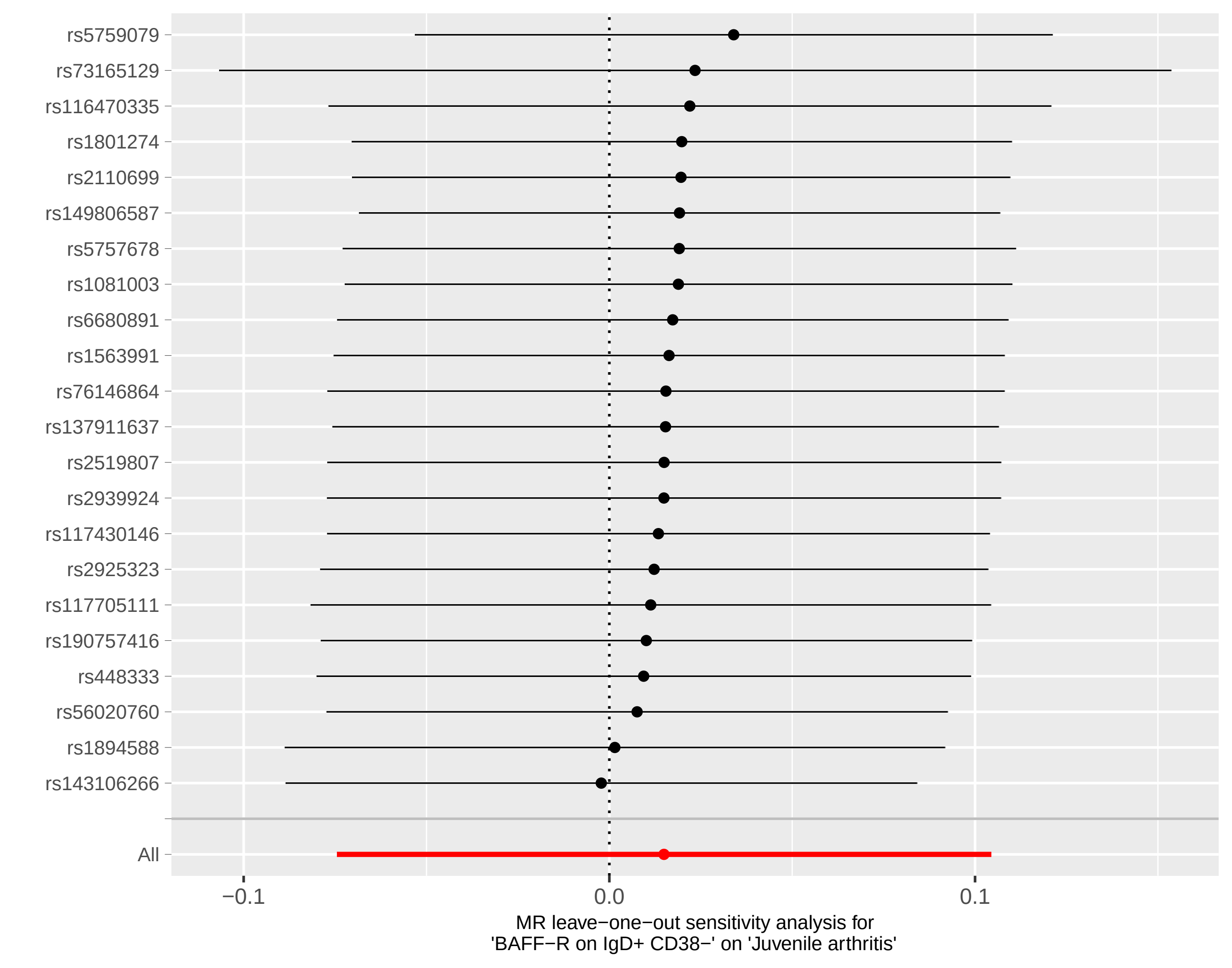
rs5759079
rs73165129
rs116470335
rs1801274
rs2110699
rs149806587
rs5757678
rs1081003
rs6680891
rs1563991
rs76146864
rs137911637
rs2519807
rs2939924
rs117430146
rs2925323
rs117705111
rs190757416
rs448333
rs56020760
rs1894588
rs143106266
All
−0.1
0.0
0.1
MR leave−one−out sensitivity analysis for
'BAFF−R on IgD+ CD38−' on 'Juvenile arthritis'

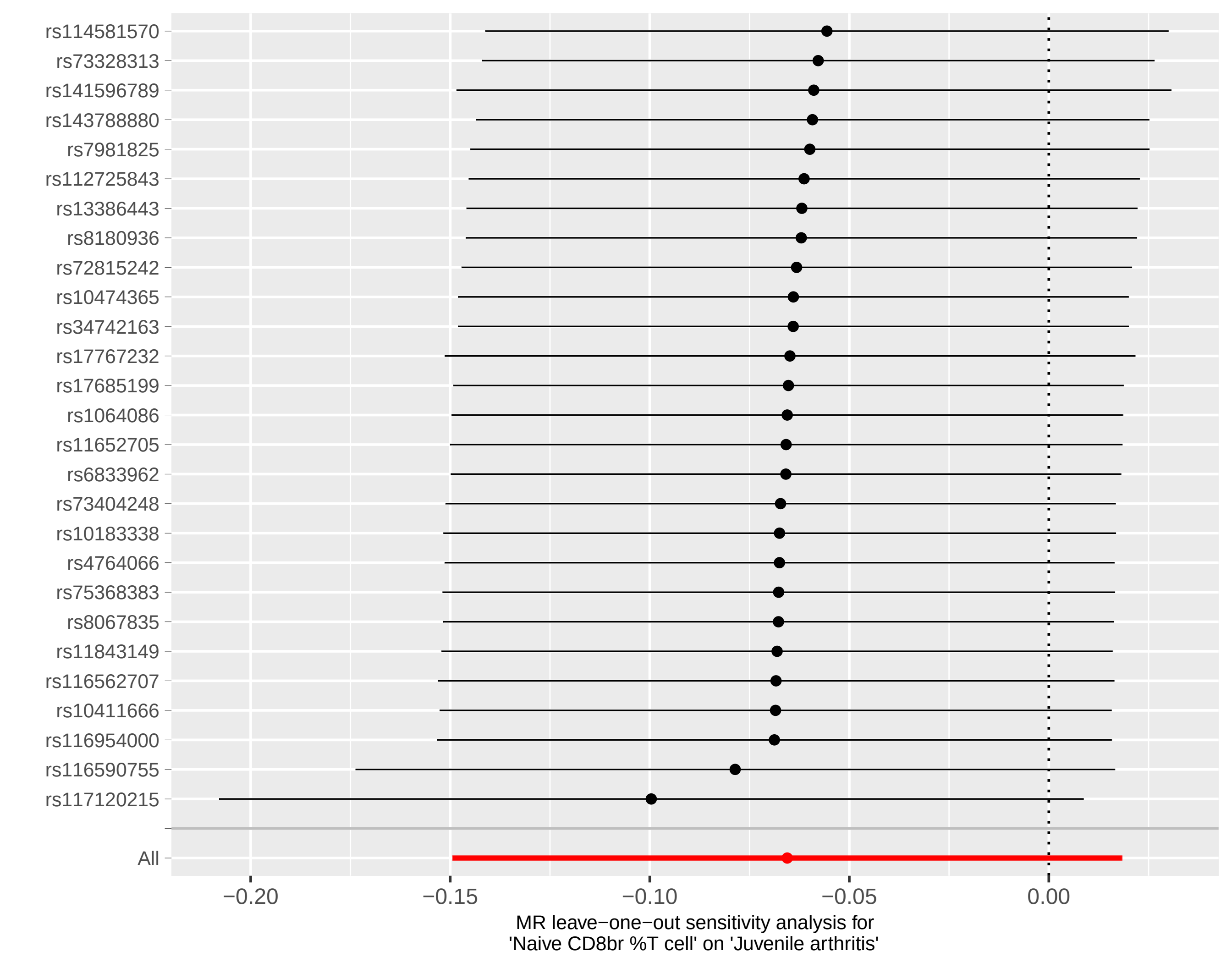
rs114581570
rs73328313
rs141596789
rs143788880
rs7981825
rs112725843
rs13386443
rs8180936
rs72815242
rs10474365
rs34742163
rs17767232
rs17685199
rs1064086
rs11652705
rs6833962
rs73404248
rs10183338
rs4764066
rs75368383
rs8067835
rs11843149
rs116562707
rs10411666
rs116954000
rs116590755
rs117120215
All
−0.20
−0.15
−0.10
−0.05
0.00
MR leave−one−out sensitivity analysis for
'Naive CD8br %T cell' on 'Juvenile arthritis'

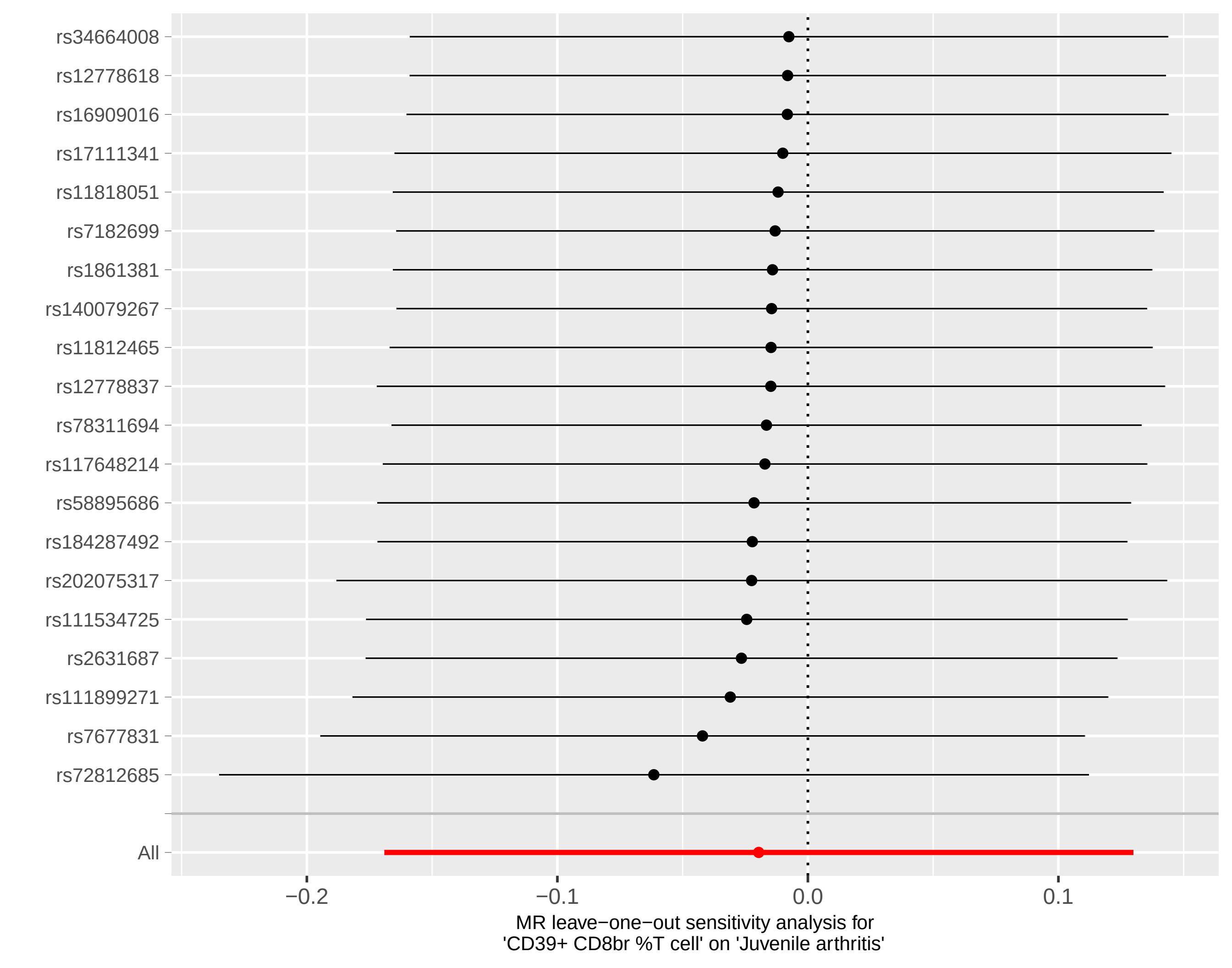

rs34664008
rs12778618
rs16909016
rs17111341
rs11818051
rs7182699
rs1861381
rs140079267
rs11812465
rs12778837
rs78311694
rs117648214
rs58895686
rs184287492
rs202075317
rs111534725
rs2631687
rs111899271
rs7677831
rs72812685
All
−0.2
−0.1
0.0
0.1
MR leave−one−out sensitivity analysis for
'CD39+ CD8br %T cell' on 'Juvenile arthritis'

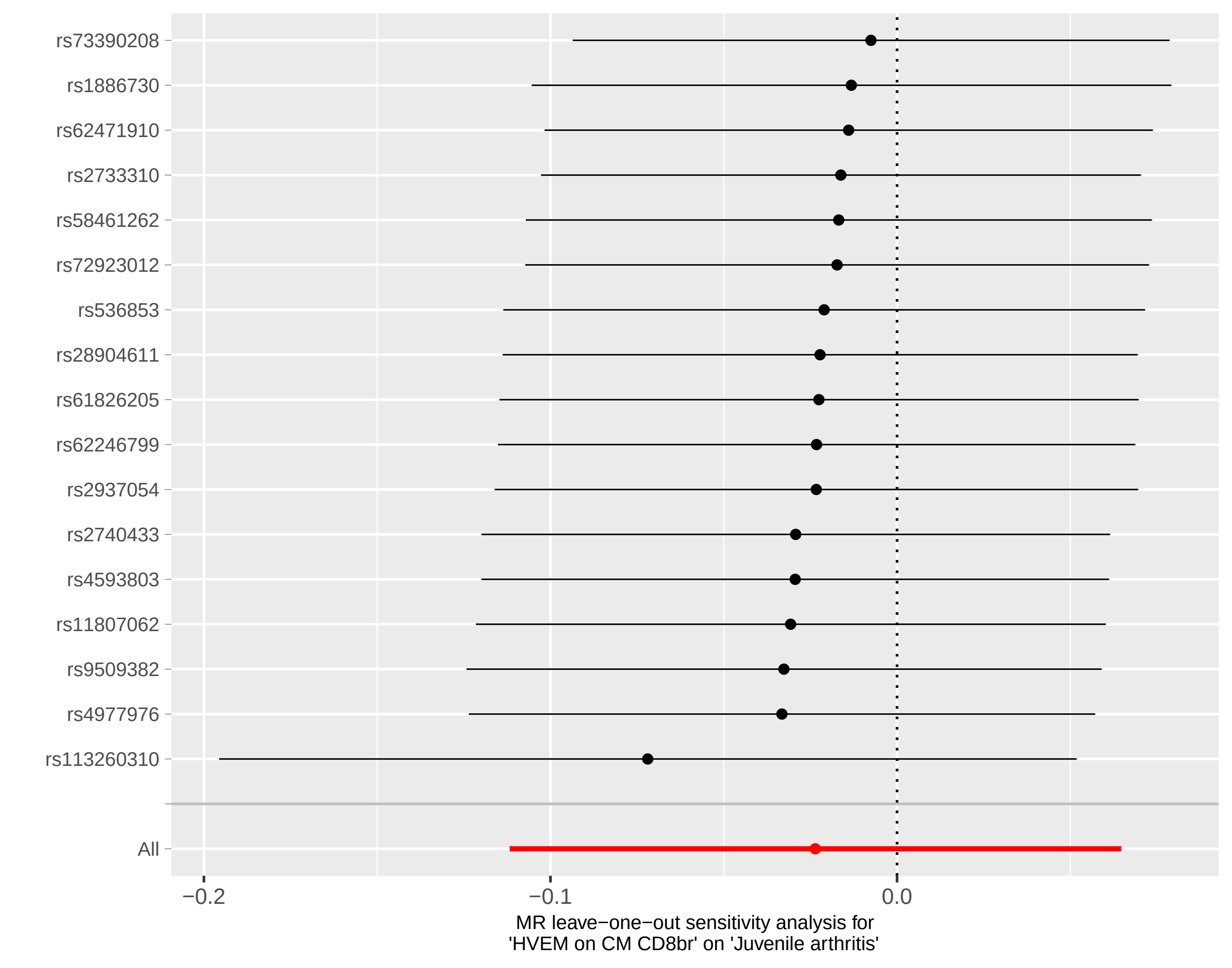
rs73390208
rs1886730
rs62471910
rs2733310
rs58461262
rs72923012
rs536853
rs28904611
rs61826205
rs62246799
rs2937054
rs2740433
rs4593803
rs11807062
rs9509382
rs4977976
rs113260310
All
−0.2
−0.1
0.0
MR leave−one−out sensitivity analysis for
'HVEM on CM CD8br' on 'Juvenile arthritis'

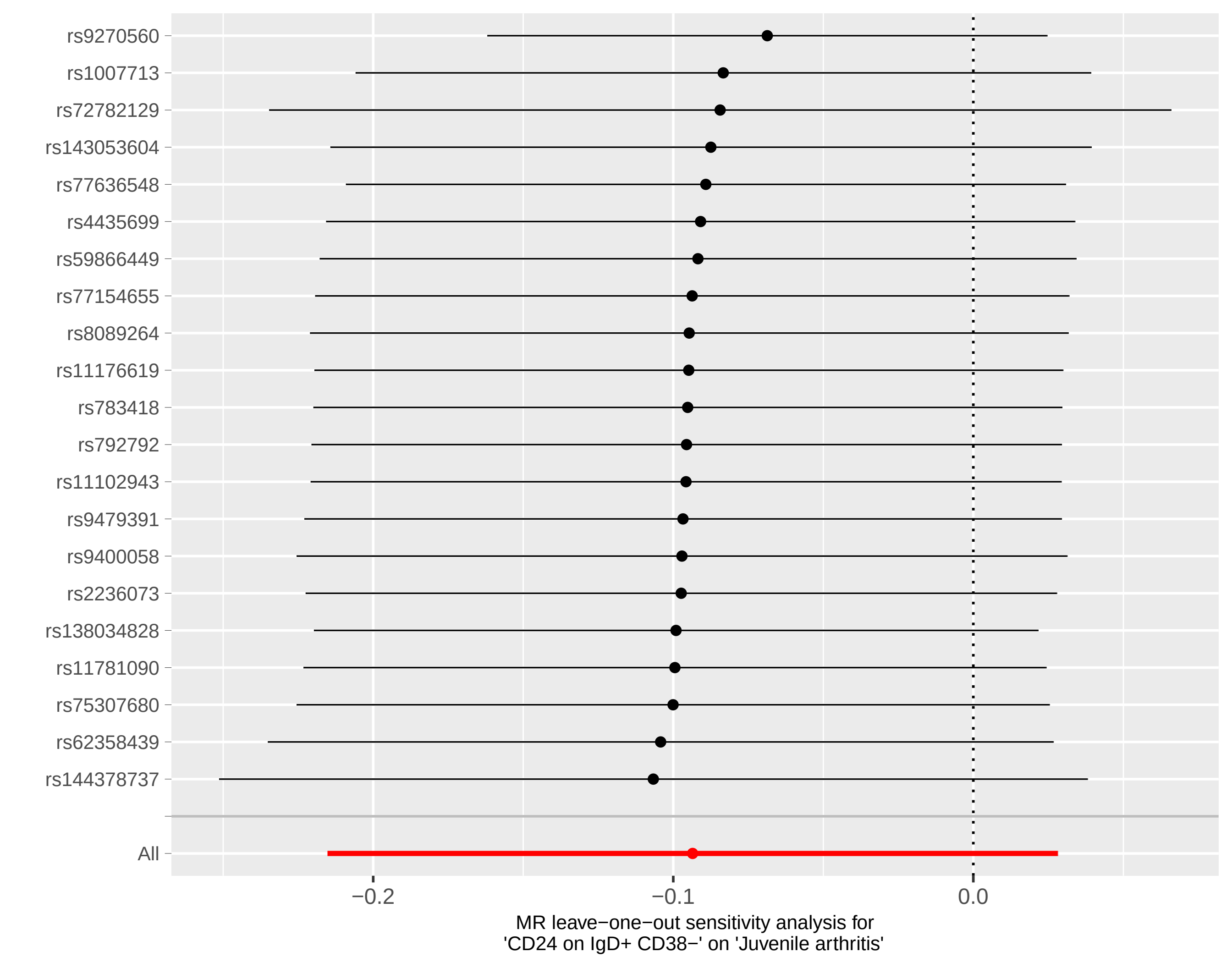
rs9270560
rs1007713
rs72782129
rs143053604
rs77636548
rs4435699
rs59866449
rs77154655
rs8089264
rs11176619
rs783418
rs792792
rs11102943
rs9479391
rs9400058
rs2236073
rs138034828
rs11781090
rs75307680
rs62358439
rs144378737
All
−0.2
−0.1
0.0
MR leave−one−out sensitivity analysis for
'CD24 on IgD+ CD38−' on 'Juvenile arthritis'

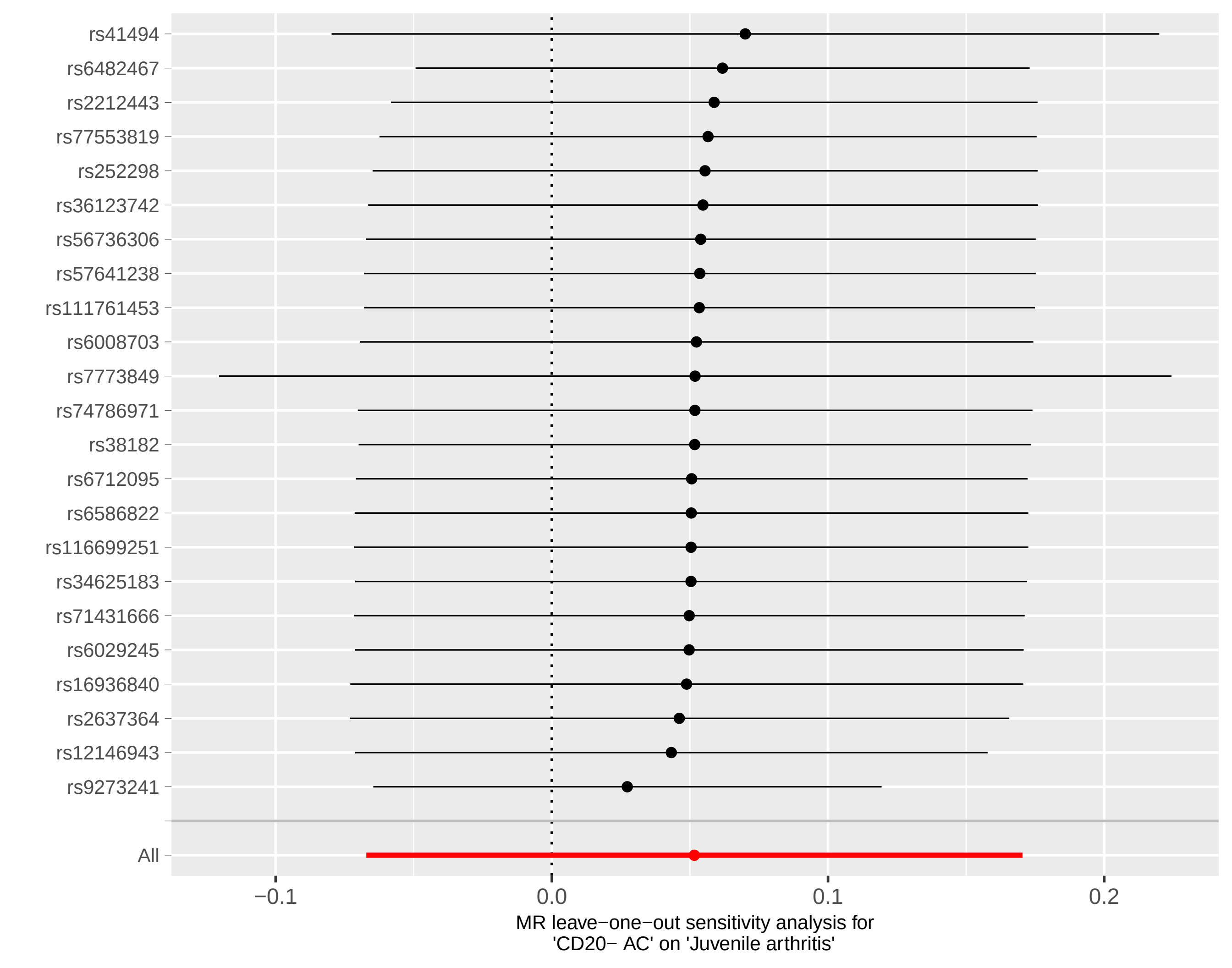
rs41494
rs6482467
rs2212443
rs77553819
rs252298
rs36123742
rs56736306
rs57641238
rs111761453
rs6008703
rs7773849
rs74786971
rs38182
rs6712095
rs6586822
rs116699251
rs34625183
rs71431666
rs6029245
rs16936840
rs2637364
rs12146943
rs9273241
All
−0.1
0.0
0.1
0.2
MR leave−one−out sensitivity analysis for
'CD20− AC' on 'Juvenile arthritis'

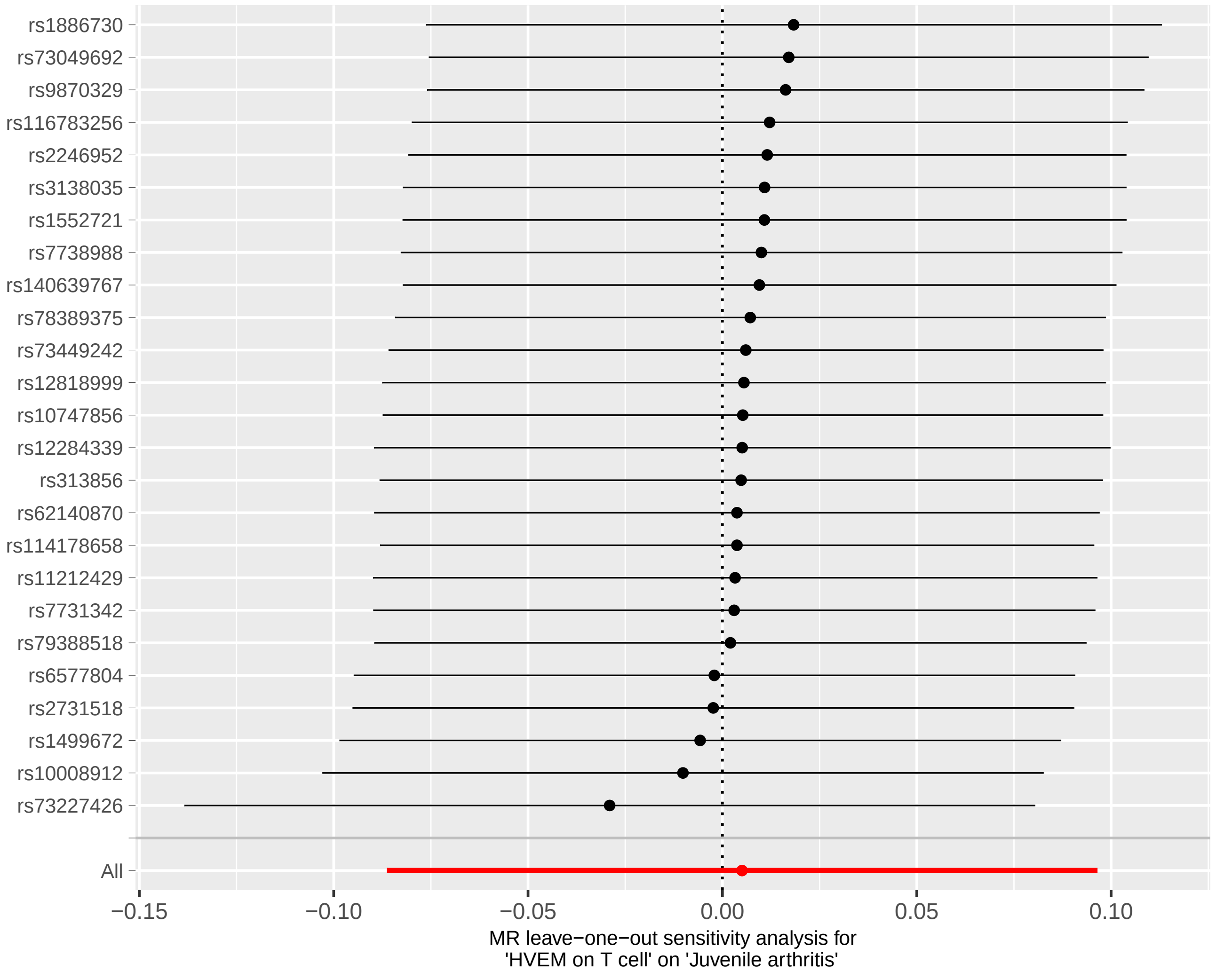

rs1886730
rs73049692
rs9870329
rs116783256
rs2246952
rs3138035
rs1552721
rs7738988
rs140639767
rs78389375
rs73449242
rs12818999
rs10747856
rs12284339
rs313856
rs62140870
rs114178658
rs11212429
rs7731342
rs79388518
rs6577804
rs2731518
rs1499672
rs10008912
rs73227426
All
−0.15
−0.10
−0.05
0.00
0.05
0.10
MR leave−one−out sensitivity analysis for
'HVEM on T cell' on 'Juvenile arthritis'

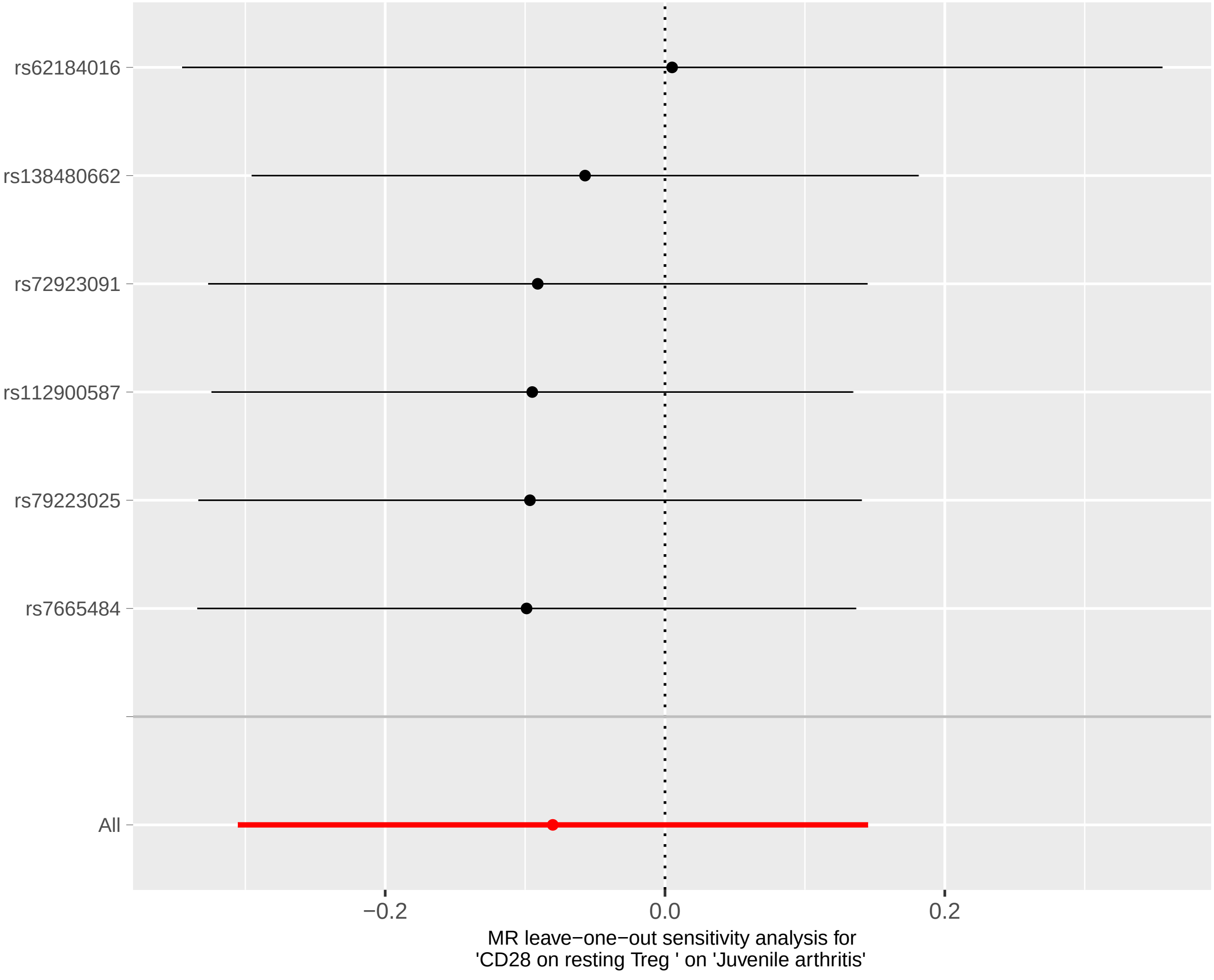

rs62184016
rs138480662
rs72923091
rs112900587
rs79223025
rs7665484
All
−0.2
0.0
0.2
MR leave−one−out sensitivity analysis for
'CD28 on resting Treg ' on 'Juvenile arthritis'

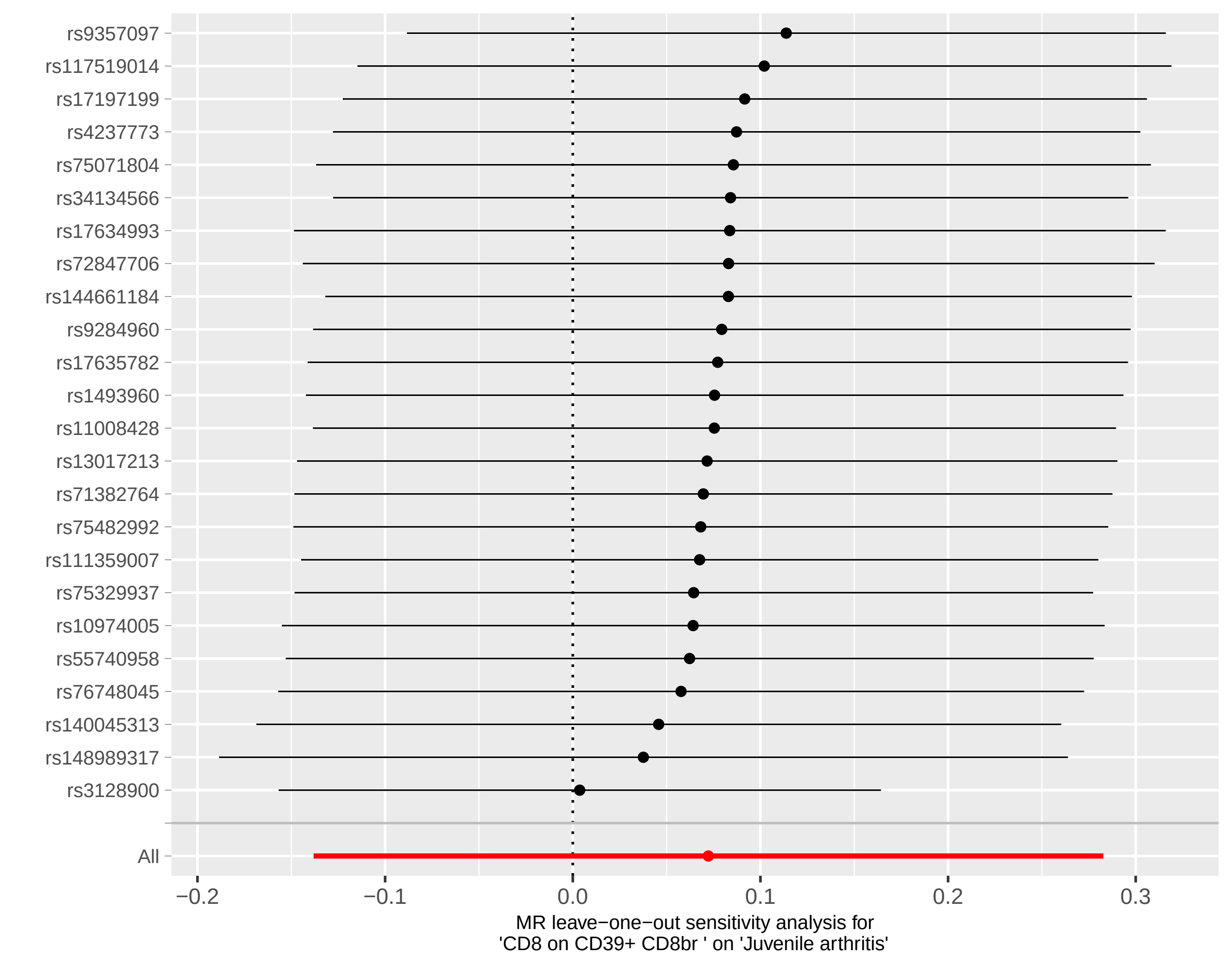
rs9357097
rs117519014
rs17197199
rs4237773
rs75071804
rs34134566
rs17634993
rs72847706
rs144661184
rs9284960
rs17635782
rs1493960
rs11008428
rs13017213
rs71382764
rs75482992
rs111359007
rs75329937
rs10974005
rs55740958
rs76748045
rs140045313
rs148989317
rs3128900
All
−0.2
−0.1
0.0
0.1
0.2
0.3
MR leave−one−out sensitivity analysis for
'CD8 on CD39+ CD8br ' on 'Juvenile arthritis'

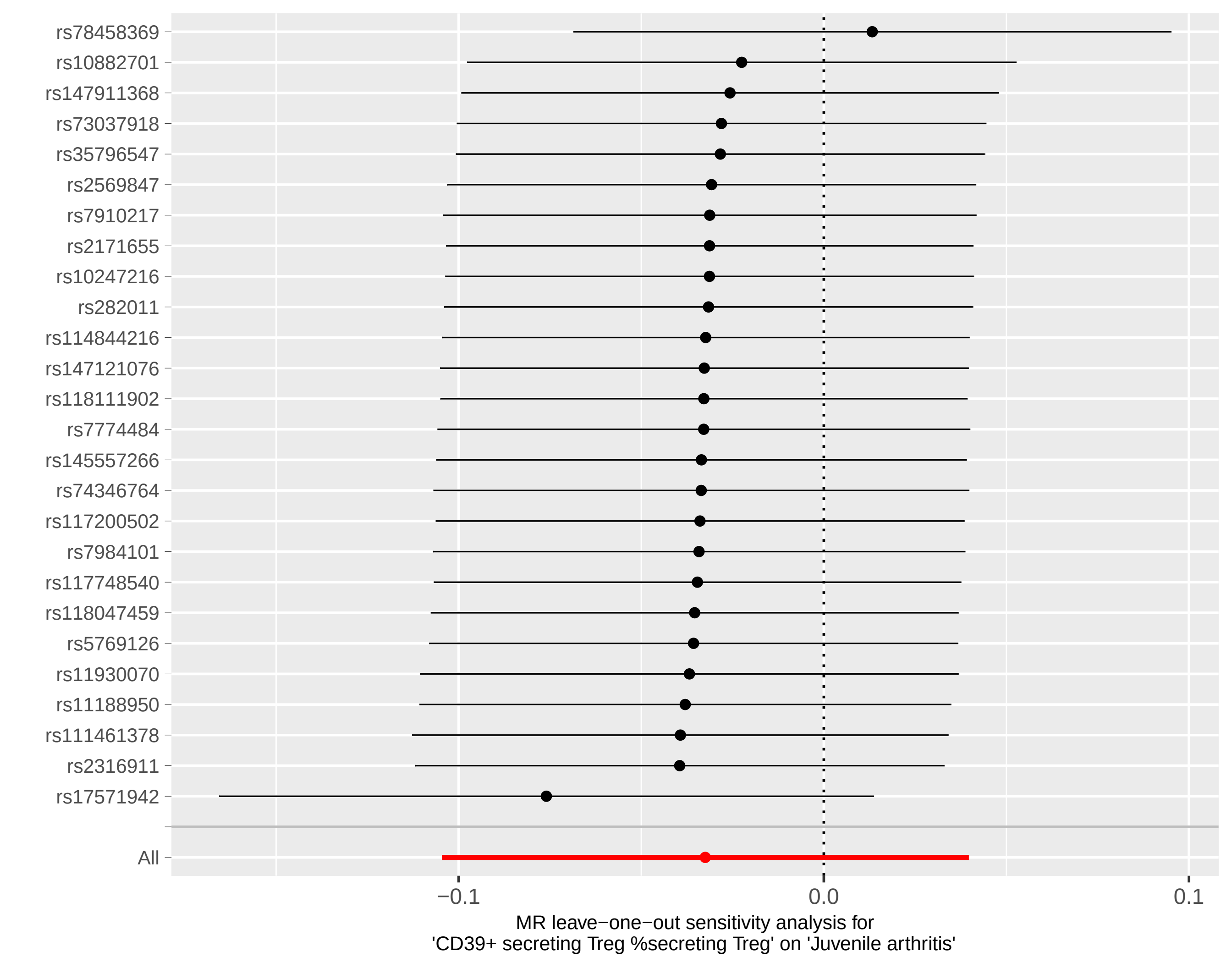

rs78458369
rs10882701
rs147911368
rs73037918
rs35796547
rs2569847
rs7910217
rs2171655
rs10247216
rs282011
rs114844216
rs147121076
rs118111902
rs7774484
rs145557266
rs74346764
rs117200502
rs7984101
rs117748540
rs118047459
rs5769126
rs11930070
rs11188950
rs111461378
rs2316911
rs17571942
All
−0.1
0.0
0.1
MR leave−one−out sensitivity analysis for
'CD39+ secreting Treg %secreting Treg' on 'Juvenile arthritis'

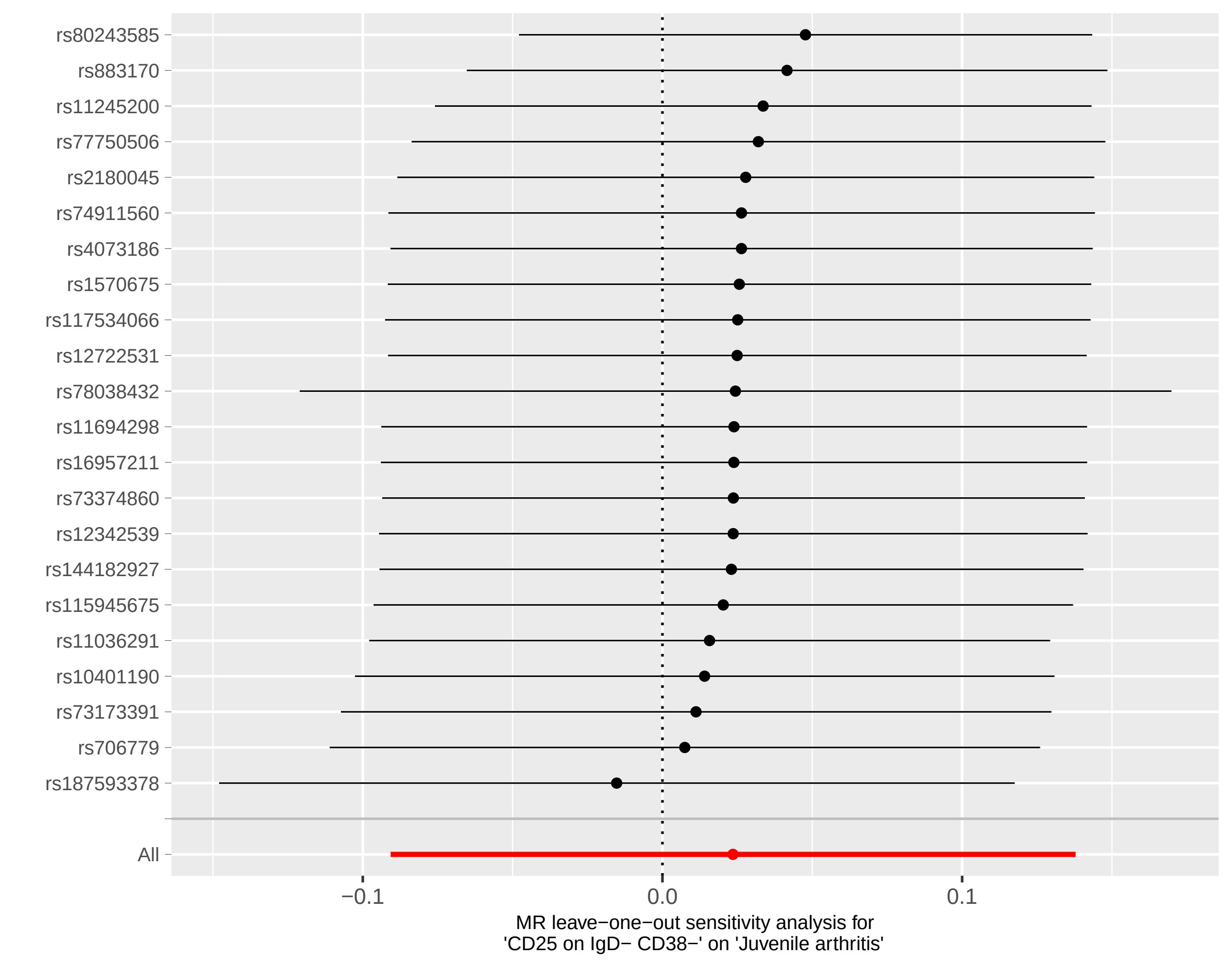
rs80243585
rs883170
rs11245200
rs77750506
rs2180045
rs74911560
rs4073186
rs1570675
rs117534066
rs12722531
rs78038432
rs11694298
rs16957211
rs73374860
rs12342539
rs144182927
rs115945675
rs11036291
rs10401190
rs73173391
rs706779
rs187593378
All
−0.1
0.0
0.1
MR leave−one−out sensitivity analysis for
'CD25 on IgD− CD38−' on 'Juvenile arthritis'

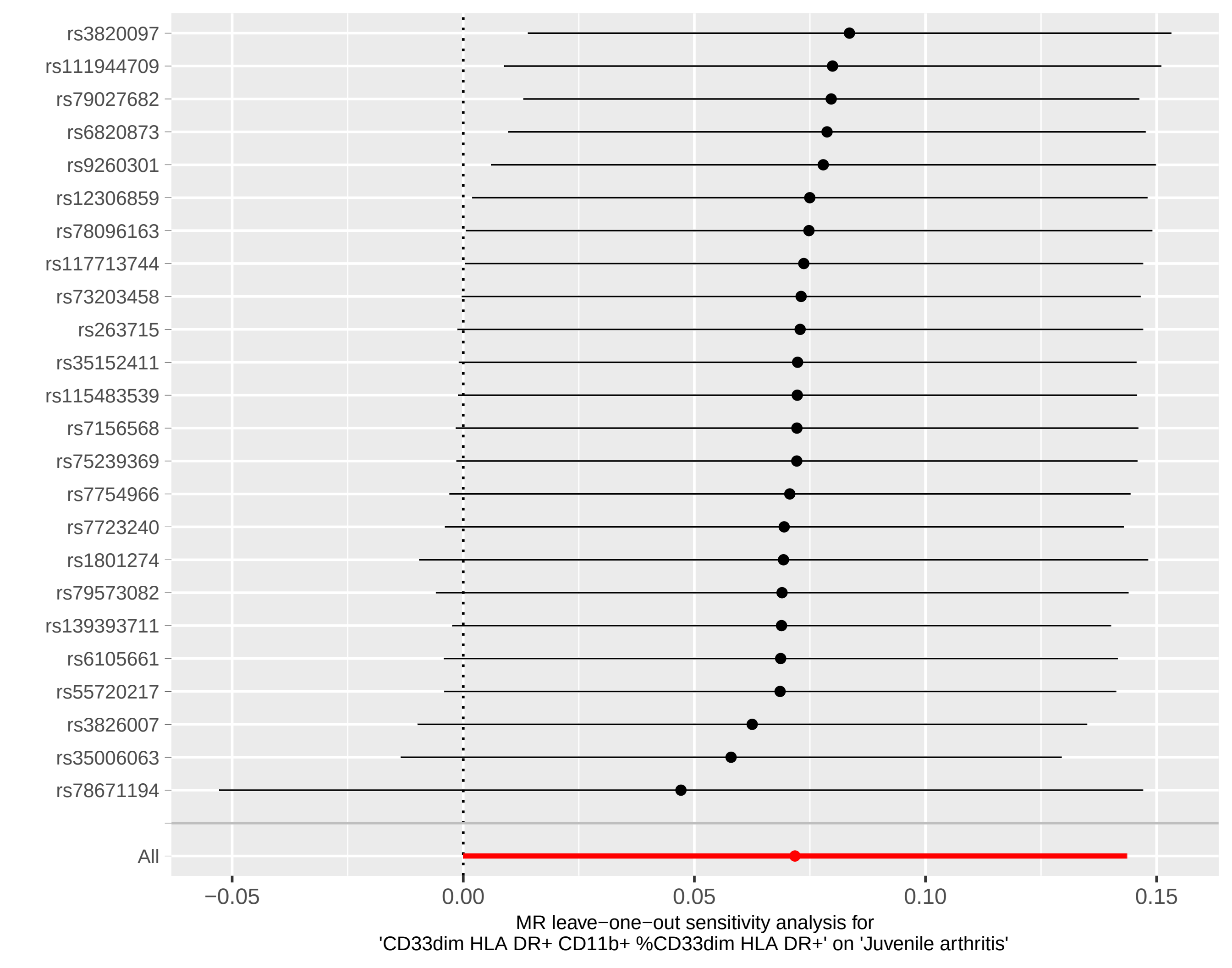

rs3820097
rs111944709
rs79027682
rs6820873
rs9260301
rs12306859
rs78096163
rs117713744
rs73203458
rs263715
rs35152411
rs115483539
rs7156568
rs75239369
rs7754966
rs7723240
rs1801274
rs79573082
rs139393711
rs6105661
rs55720217
rs3826007
rs35006063
rs78671194
All
−0.05
0.00
0.05
0.10
0.15
MR leave−one−out sensitivity analysis for
'CD33dim HLA DR+ CD11b+ %CD33dim HLA DR+' on 'Juvenile arthritis'

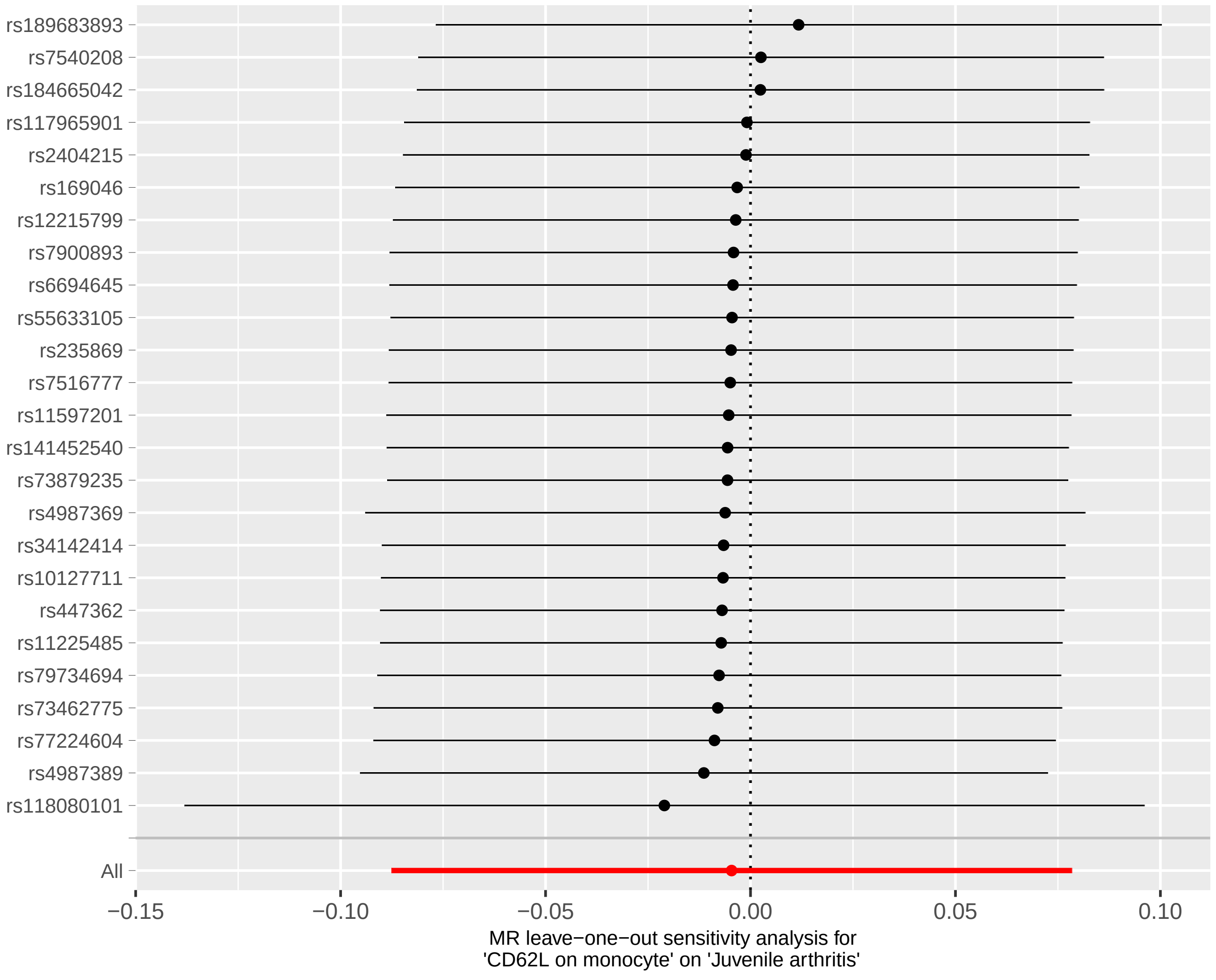

rs189683893
rs7540208
rs184665042
rs117965901
rs2404215
rs169046
rs12215799
rs7900893
rs6694645
rs55633105
rs235869
rs7516777
rs11597201
rs141452540
rs73879235
rs4987369
rs34142414
rs10127711
rs447362
rs11225485
rs79734694
rs73462775
rs77224604
rs4987389
rs118080101
All
−0.15
−0.10
−0.05
0.00
0.05
0.10
MR leave−one−out sensitivity analysis for
'CD62L on monocyte' on 'Juvenile arthritis'

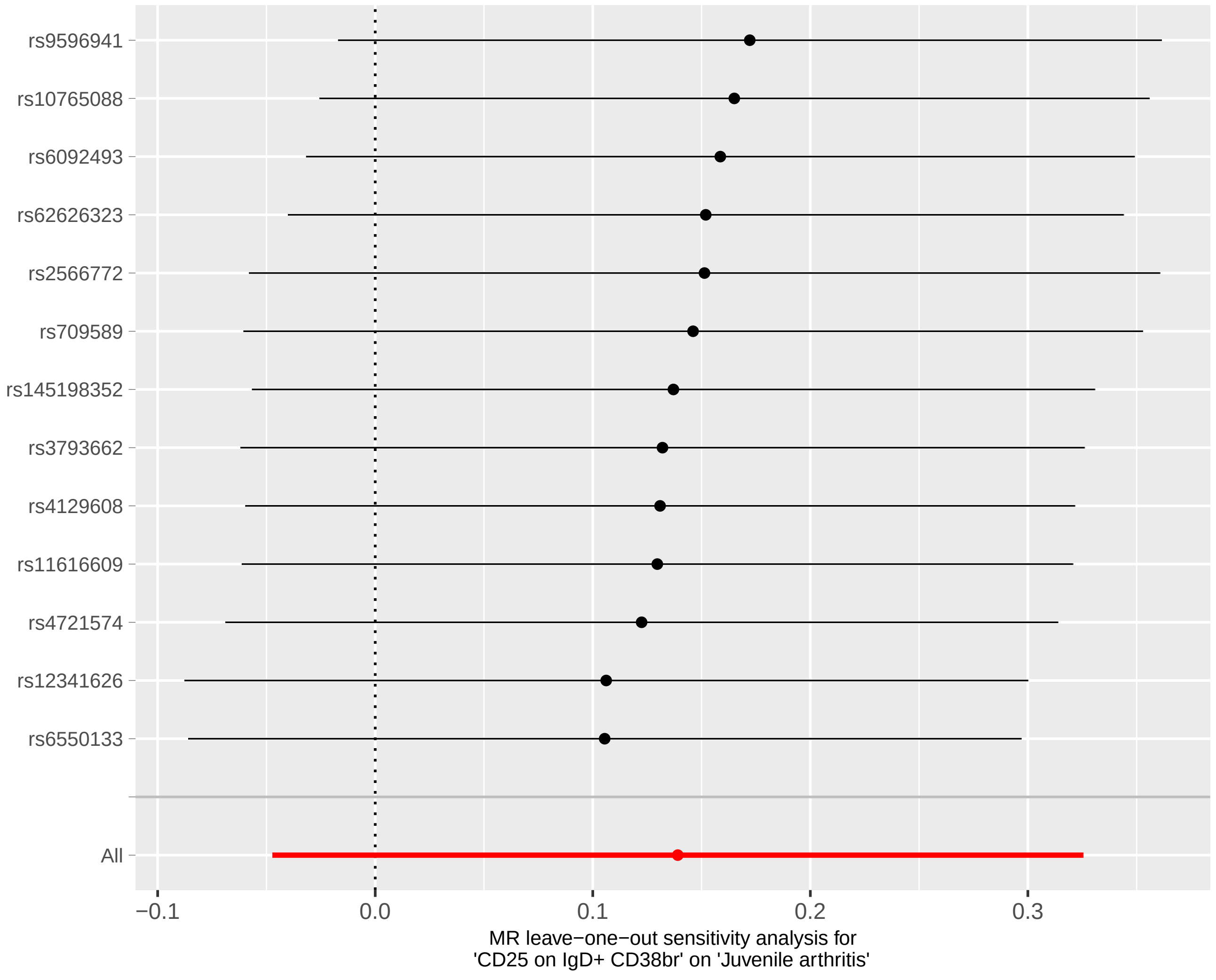
rs9596941
rs10765088
rs6092493
rs62626323
rs2566772
rs709589
rs145198352
rs3793662
rs4129608
rs11616609
rs4721574
rs12341626
rs6550133
All
−0.1
0.0
0.1
0.2
0.3
MR leave−one−out sensitivity analysis for
'CD25 on IgD+ CD38br' on 'Juvenile arthritis'

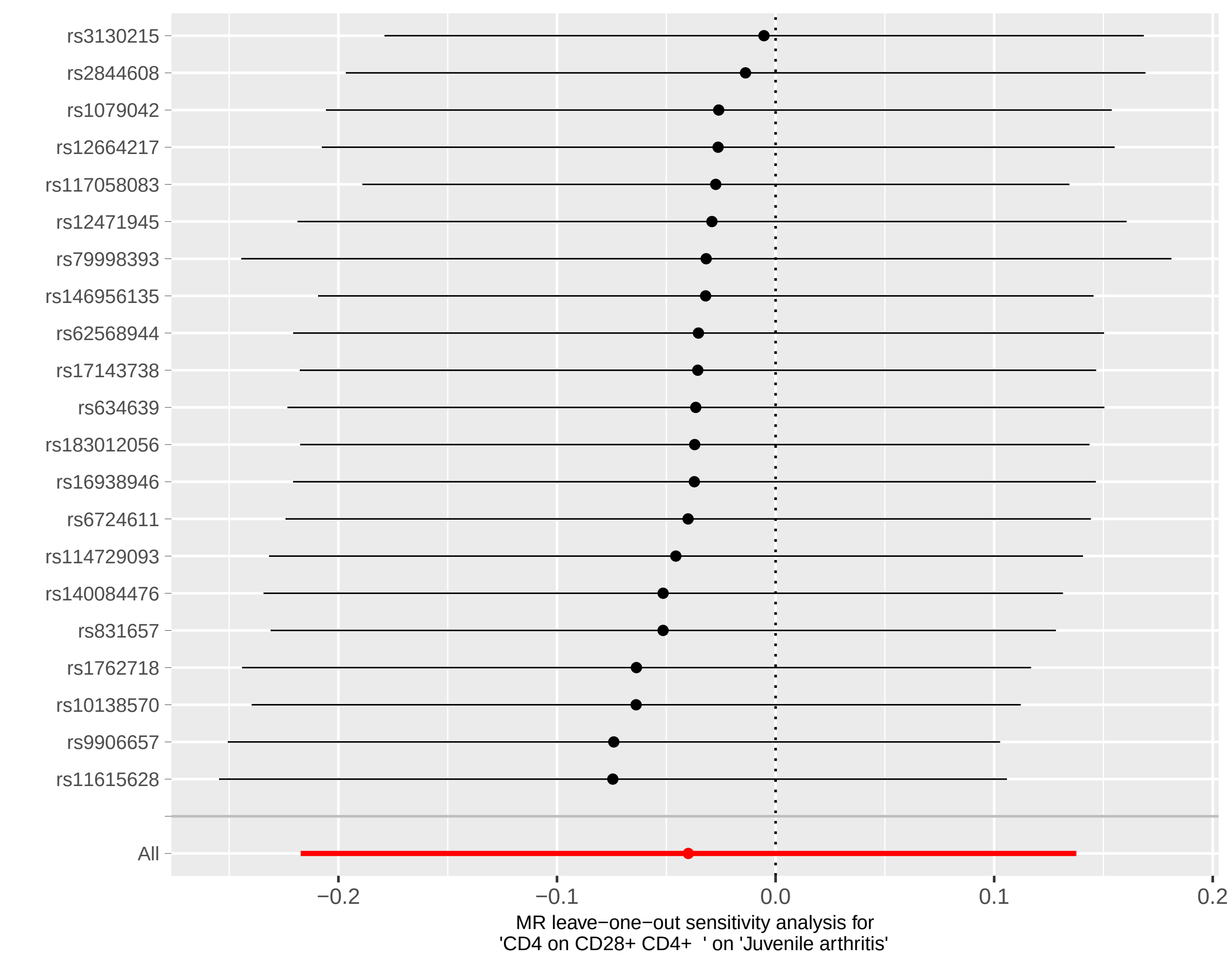
rs3130215
rs2844608
rs1079042
rs12664217
rs117058083
rs12471945
rs79998393
rs146956135
rs62568944
rs17143738
rs634639
rs183012056
rs16938946
rs6724611
rs114729093
rs140084476
rs831657
rs1762718
rs10138570
rs9906657
rs11615628
All
−0.2
−0.1
0.0
0.1
0.2
MR leave−one−out sensitivity analysis for
'CD4 on CD28+ CD4+ ' on 'Juvenile arthritis'

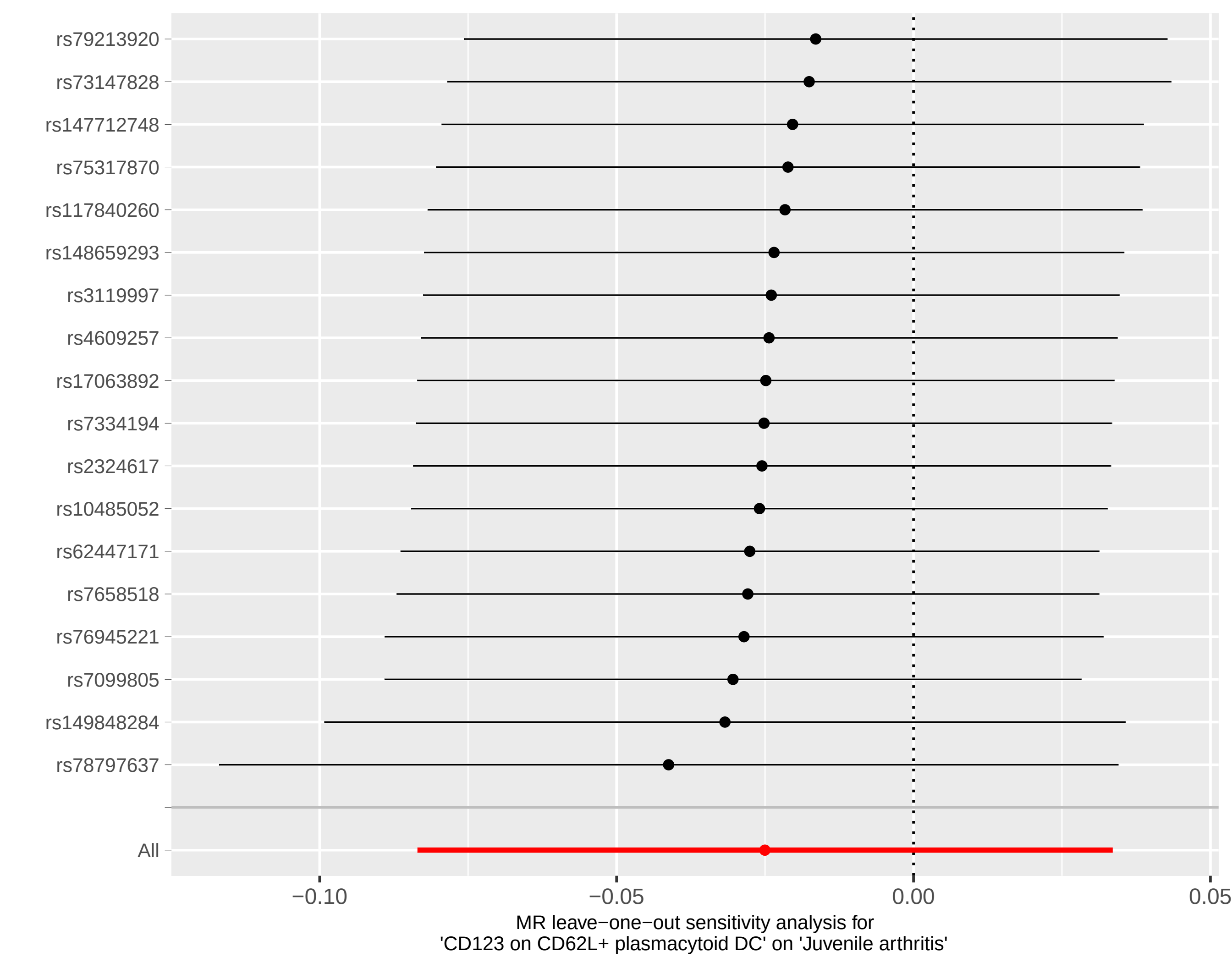
rs79213920
rs73147828
rs147712748
rs75317870
rs117840260
rs148659293
rs3119997
rs4609257
rs17063892
rs7334194
rs2324617
rs10485052
rs62447171
rs7658518
rs76945221
rs7099805
rs149848284
rs78797637
All
−0.10
−0.05
0.00
0.05
MR leave−one−out sensitivity analysis for
'CD123 on CD62L+ plasmacytoid DC' on 'Juvenile arthritis'

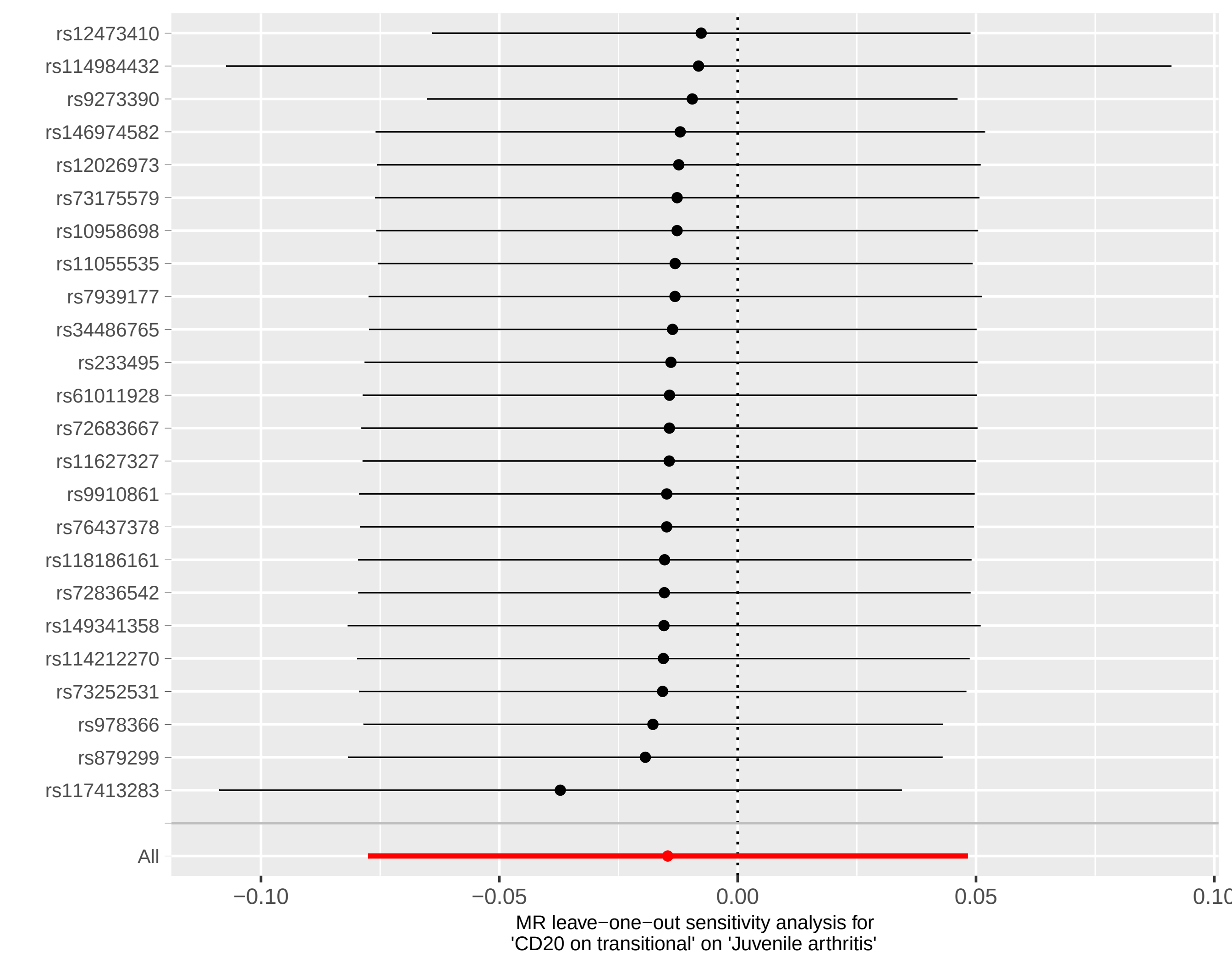
rs12473410
rs114984432
rs9273390
rs146974582
rs12026973
rs73175579
rs10958698
rs11055535
rs7939177
rs34486765
rs233495
rs61011928
rs72683667
rs11627327
rs9910861
rs76437378
rs118186161
rs72836542
rs149341358
rs114212270
rs73252531
rs978366
rs879299
rs117413283
All
−0.10
−0.05
0.00
0.05
0.10
MR leave−one−out sensitivity analysis for
'CD20 on transitional' on 'Juvenile arthritis'

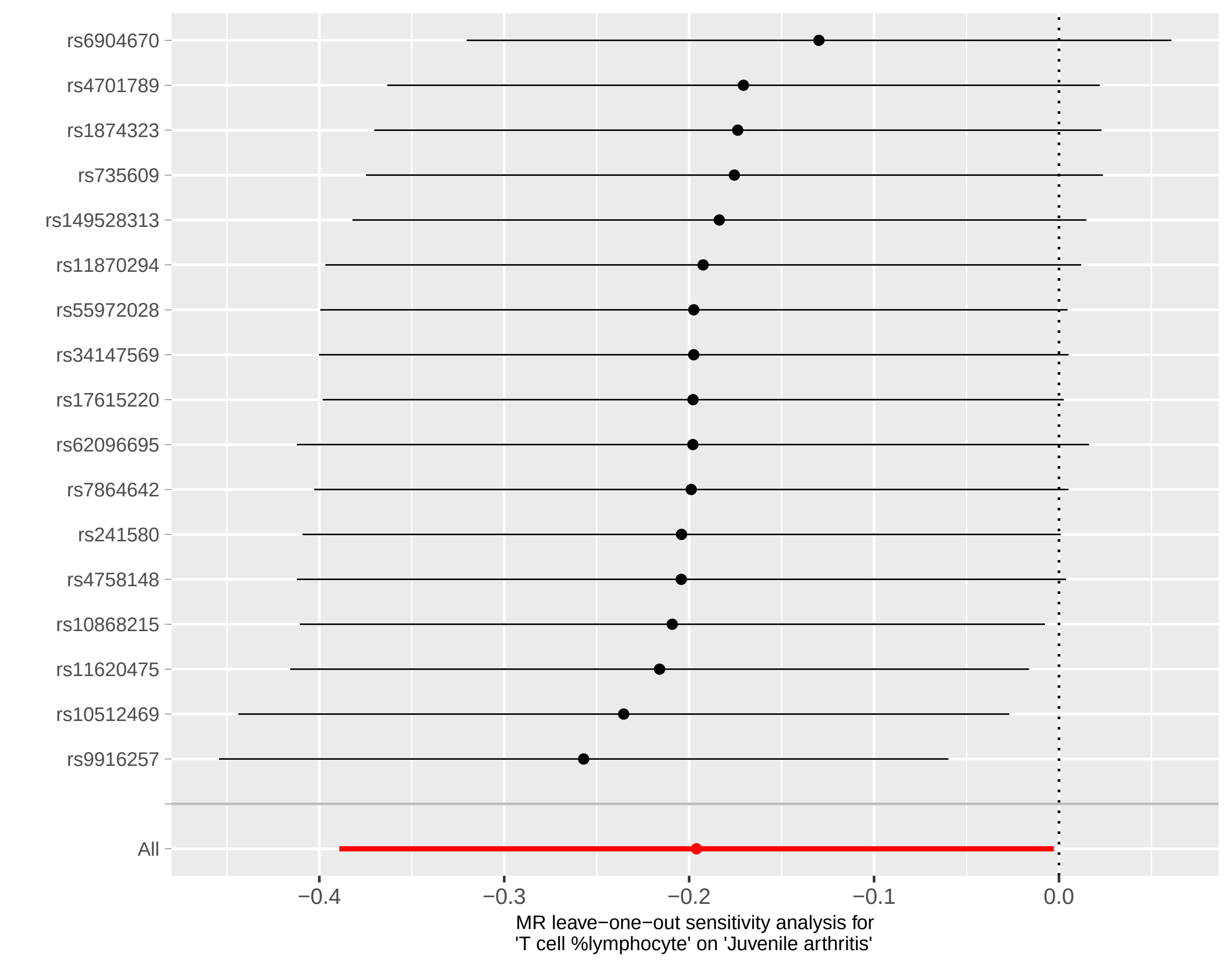
rs6904670
rs4701789
rs1874323
rs735609
rs149528313
rs11870294
rs55972028
rs34147569
rs17615220
rs62096695
rs7864642
rs241580
rs4758148
rs10868215
rs11620475
rs10512469
rs9916257
All
−0.4
−0.3
−0.2
−0.1
0.0
MR leave−one−out sensitivity analysis for
'T cell %lymphocyte' on 'Juvenile arthritis'

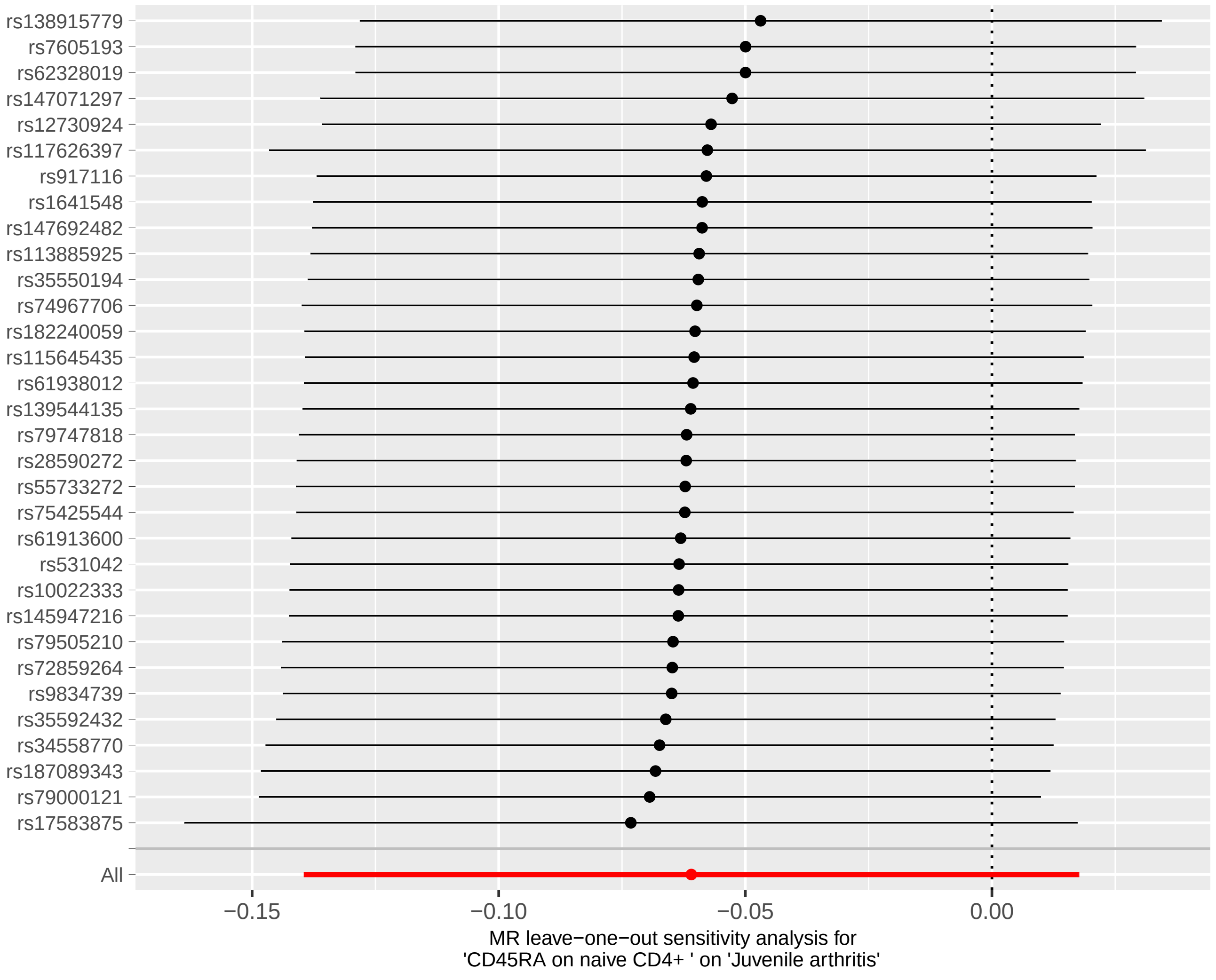

rs138915779
rs7605193
rs62328019
rs147071297
rs12730924
rs117626397
rs917116
rs1641548
rs147692482
rs113885925
rs35550194
rs74967706
rs182240059
rs115645435
rs61938012
rs139544135
rs79747818
rs28590272
rs55733272
rs75425544
rs61913600
rs531042
rs10022333
rs145947216
rs79505210
rs72859264
rs9834739
rs35592432
rs34558770
rs187089343
rs79000121
rs17583875
All
−0.15
−0.10
−0.05
0.00
MR leave−one−out sensitivity analysis for
'CD45RA on naive CD4+ ' on 'Juvenile arthritis'

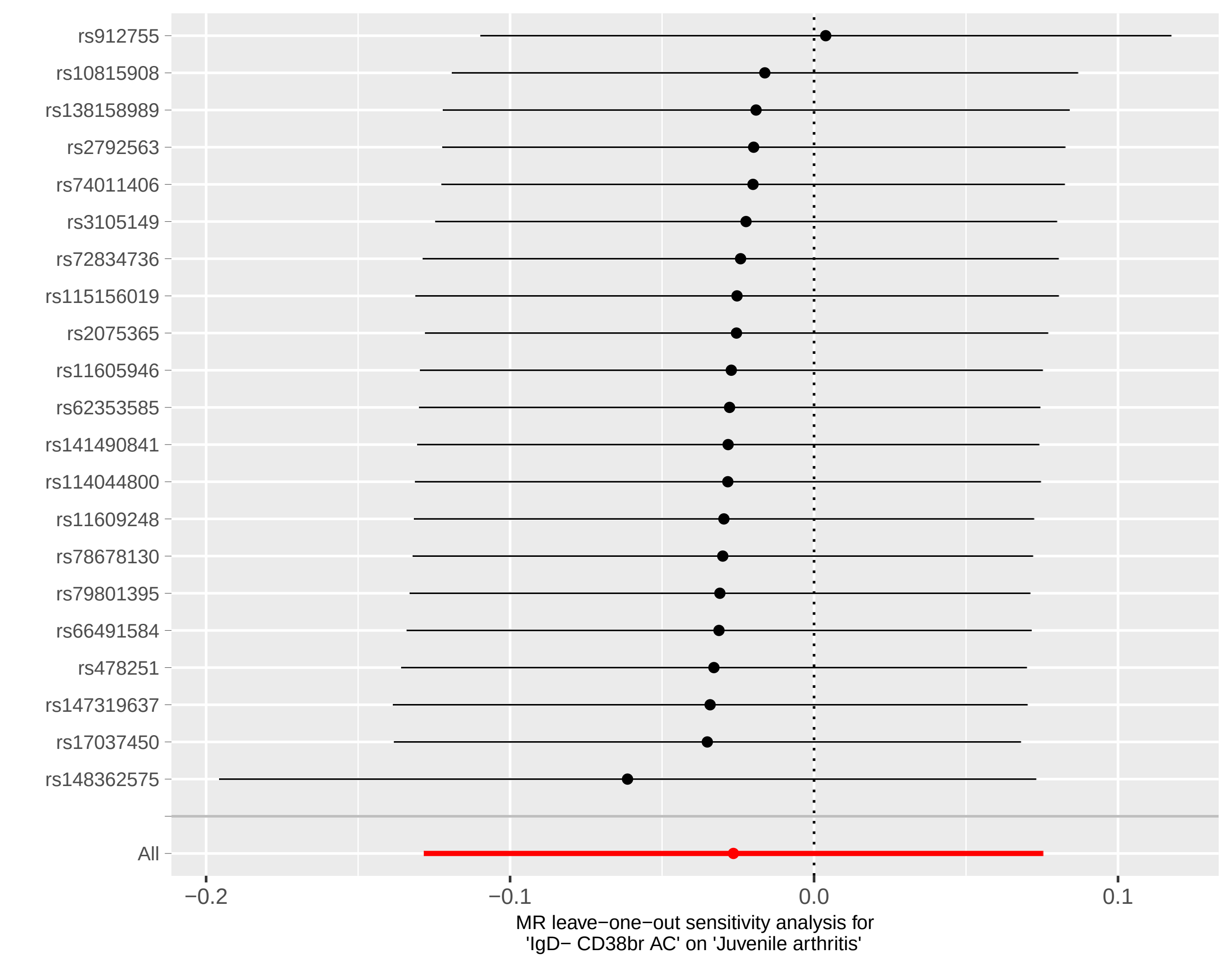
rs912755
rs10815908
rs138158989
rs2792563
rs74011406
rs3105149
rs72834736
rs115156019
rs2075365
rs11605946
rs62353585
rs141490841
rs114044800
rs11609248
rs78678130
rs79801395
rs66491584
rs478251
rs147319637
rs17037450
rs148362575
All
−0.2
−0.1
0.0
0.1
MR leave−one−out sensitivity analysis for
'IgD− CD38br AC' on 'Juvenile arthritis'

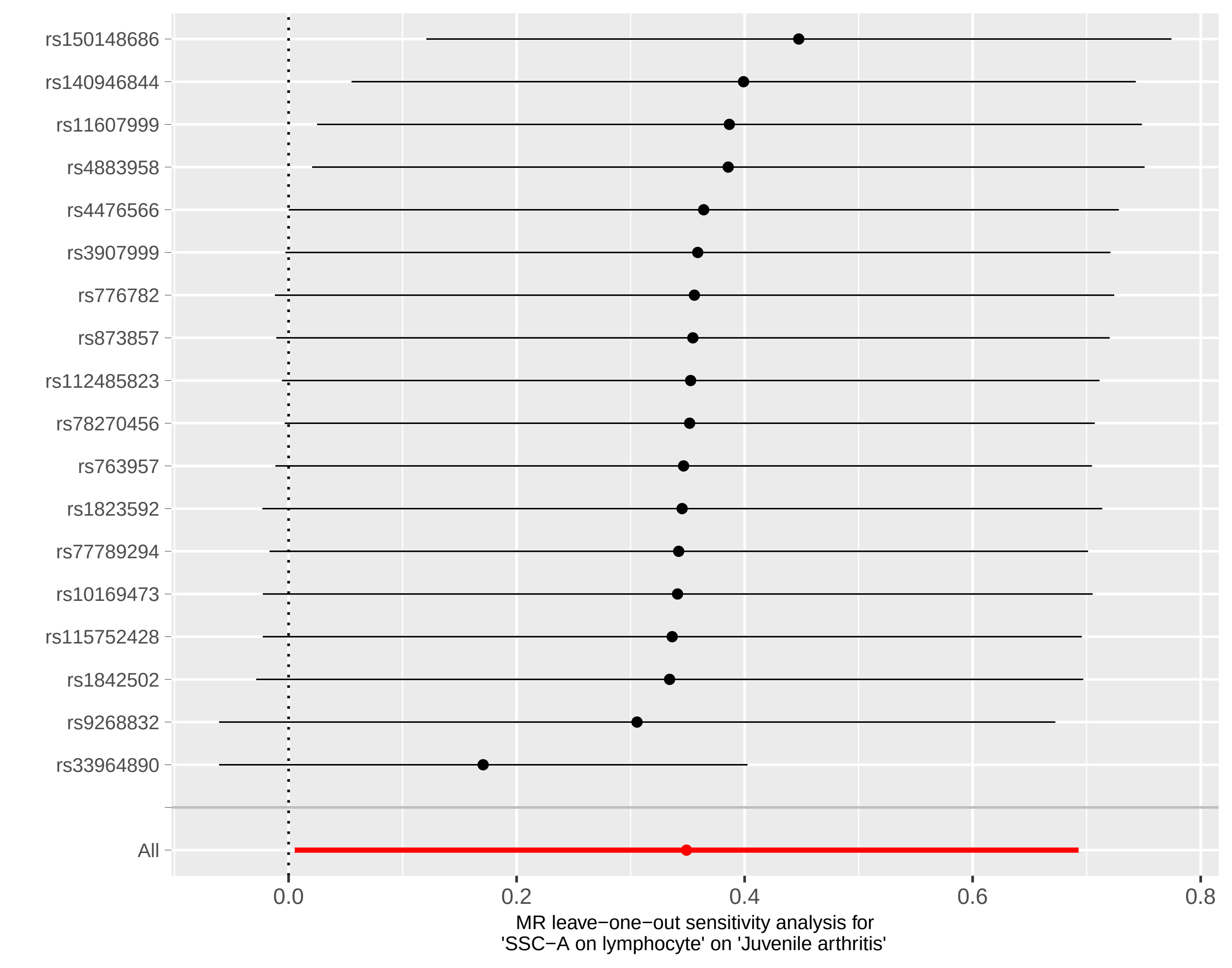
rs150148686
rs140946844
rs11607999
rs4883958
rs4476566
rs3907999
rs776782
rs873857
rs112485823
rs78270456
rs763957
rs1823592
rs77789294
rs10169473
rs115752428
rs1842502
rs9268832
rs33964890
All
0.0
0.2
0.4
0.6
0.8
MR leave−one−out sensitivity analysis for
'SSC−A on lymphocyte' on 'Juvenile arthritis'

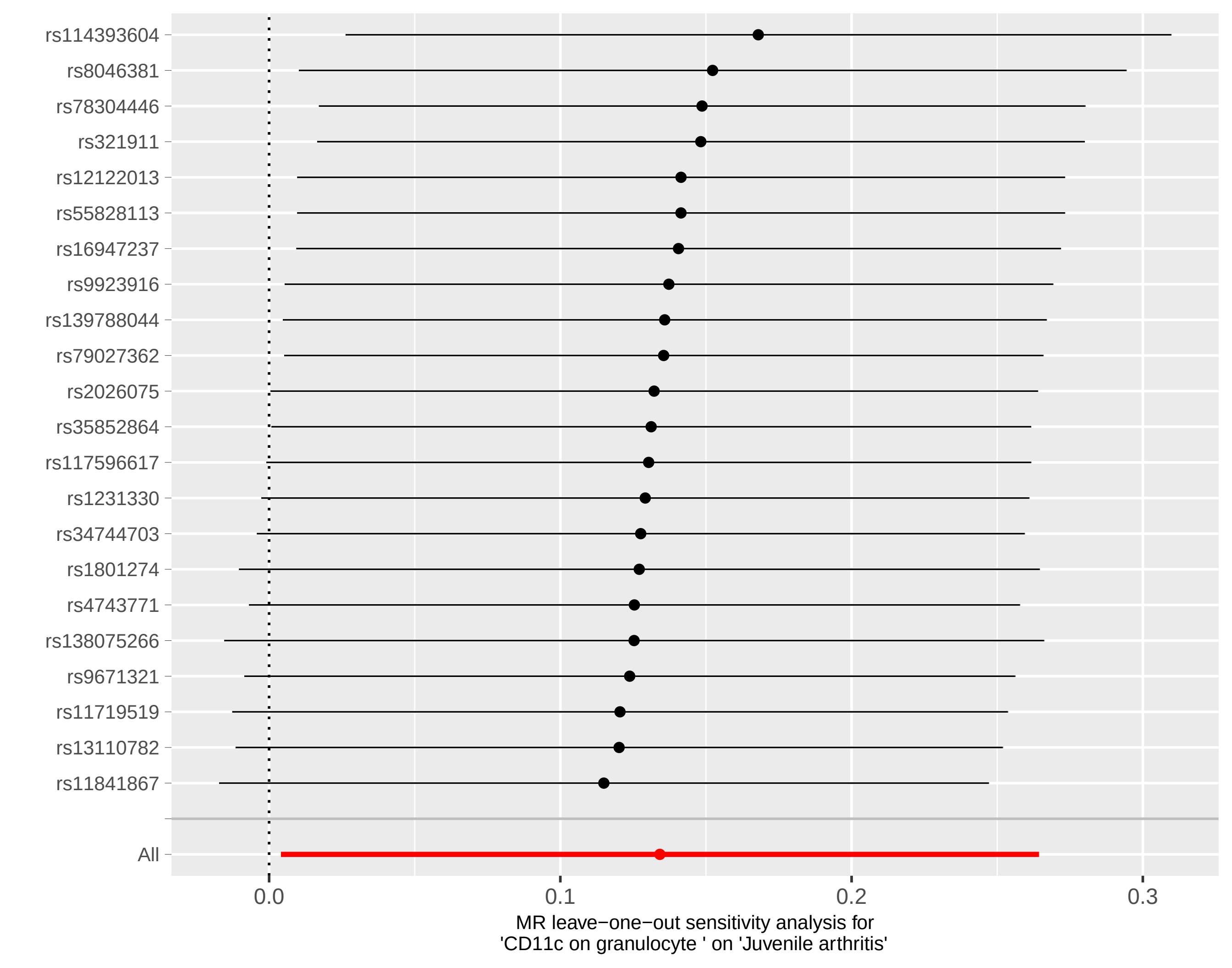
rs114393604
rs8046381
rs78304446
rs321911
rs12122013
rs55828113
rs16947237
rs9923916
rs139788044
rs79027362
rs2026075
rs35852864
rs117596617
rs1231330
rs34744703
rs1801274
rs4743771
rs138075266
rs9671321
rs11719519
rs13110782
rs11841867
All
0.0
0.1
0.2
0.3
MR leave−one−out sensitivity analysis for
'CD11c on granulocyte ' on 'Juvenile arthritis'

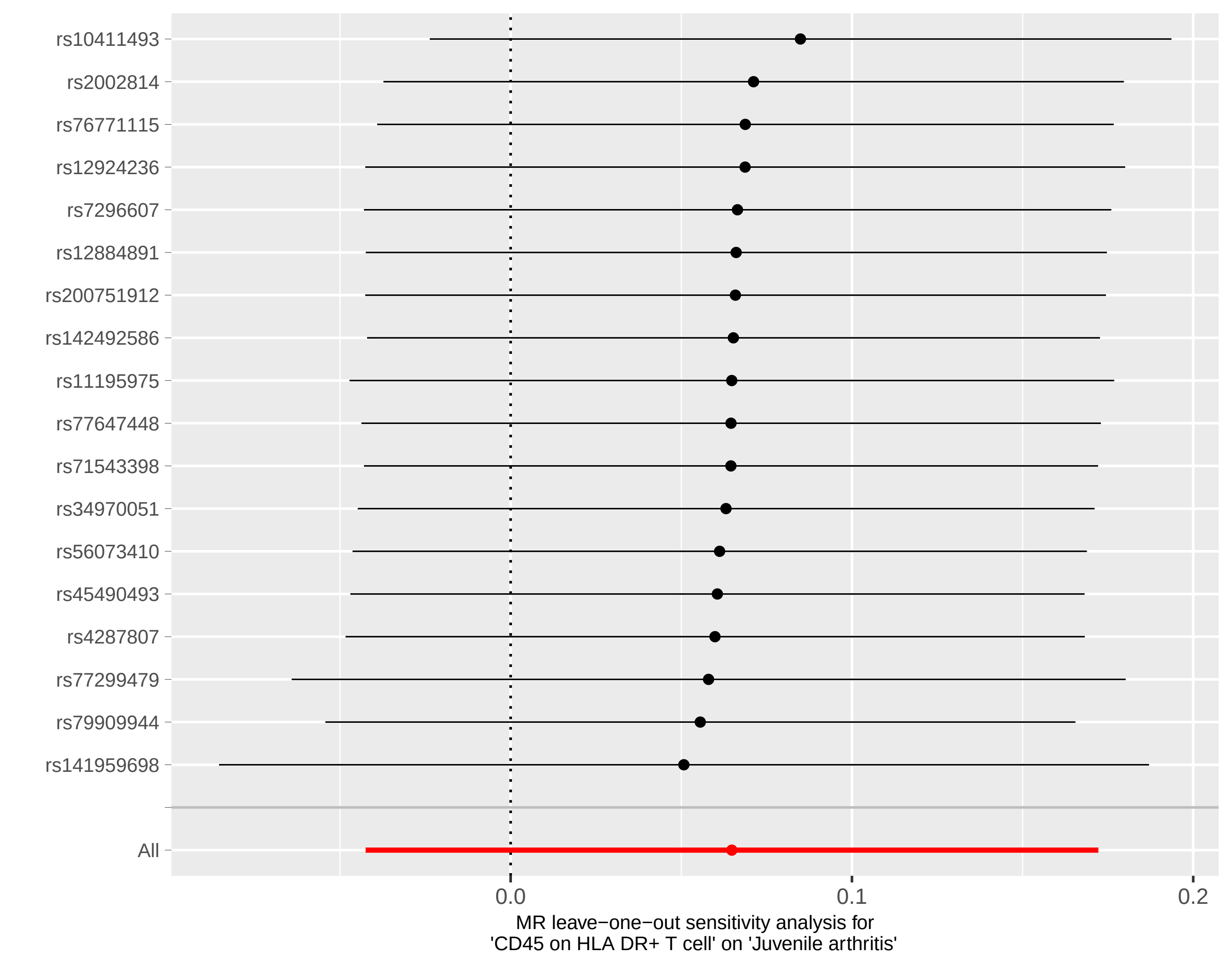

rs10411493
rs2002814
rs76771115
rs12924236
rs7296607
rs12884891
rs200751912
rs142492586
rs11195975
rs77647448
rs71543398
rs34970051
rs56073410
rs45490493
rs4287807
rs77299479
rs79909944
rs141959698
All
0.0
0.1
0.2
MR leave−one−out sensitivity analysis for
'CD45 on HLA DR+ T cell' on 'Juvenile arthritis'

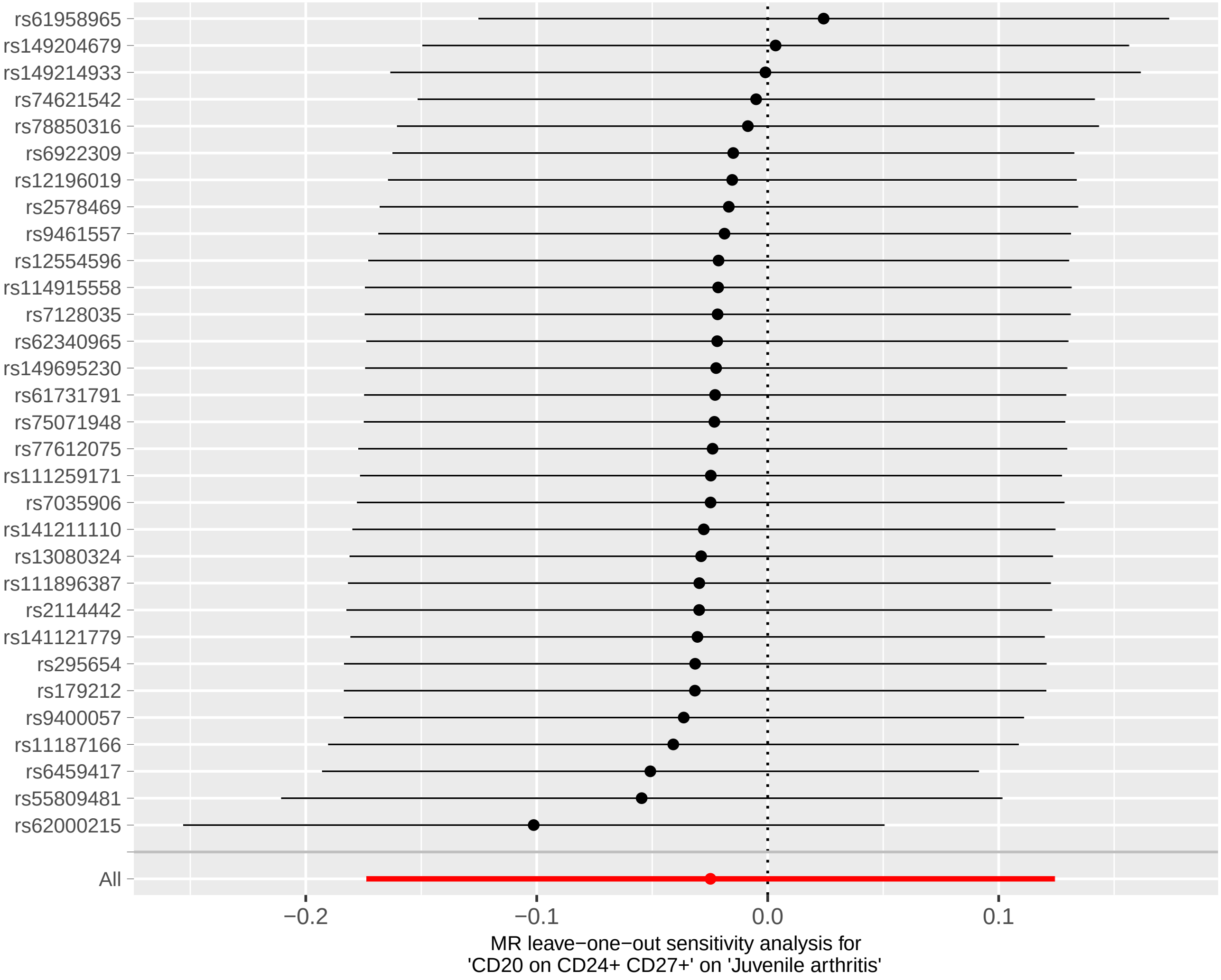

rs61958965
rs149204679
rs149214933
rs74621542
rs78850316
rs6922309
rs12196019
rs2578469
rs9461557
rs12554596
rs114915558
rs7128035
rs62340965
rs149695230
rs61731791
rs75071948
rs77612075
rs111259171
rs7035906
rs141211110
rs13080324
rs111896387
rs2114442
rs141121779
rs295654
rs179212
rs9400057
rs11187166
rs6459417
rs55809481
rs62000215
All
−0.2
−0.1
0.0
0.1
MR leave−one−out sensitivity analysis for
'CD20 on CD24+ CD27+' on 'Juvenile arthritis'

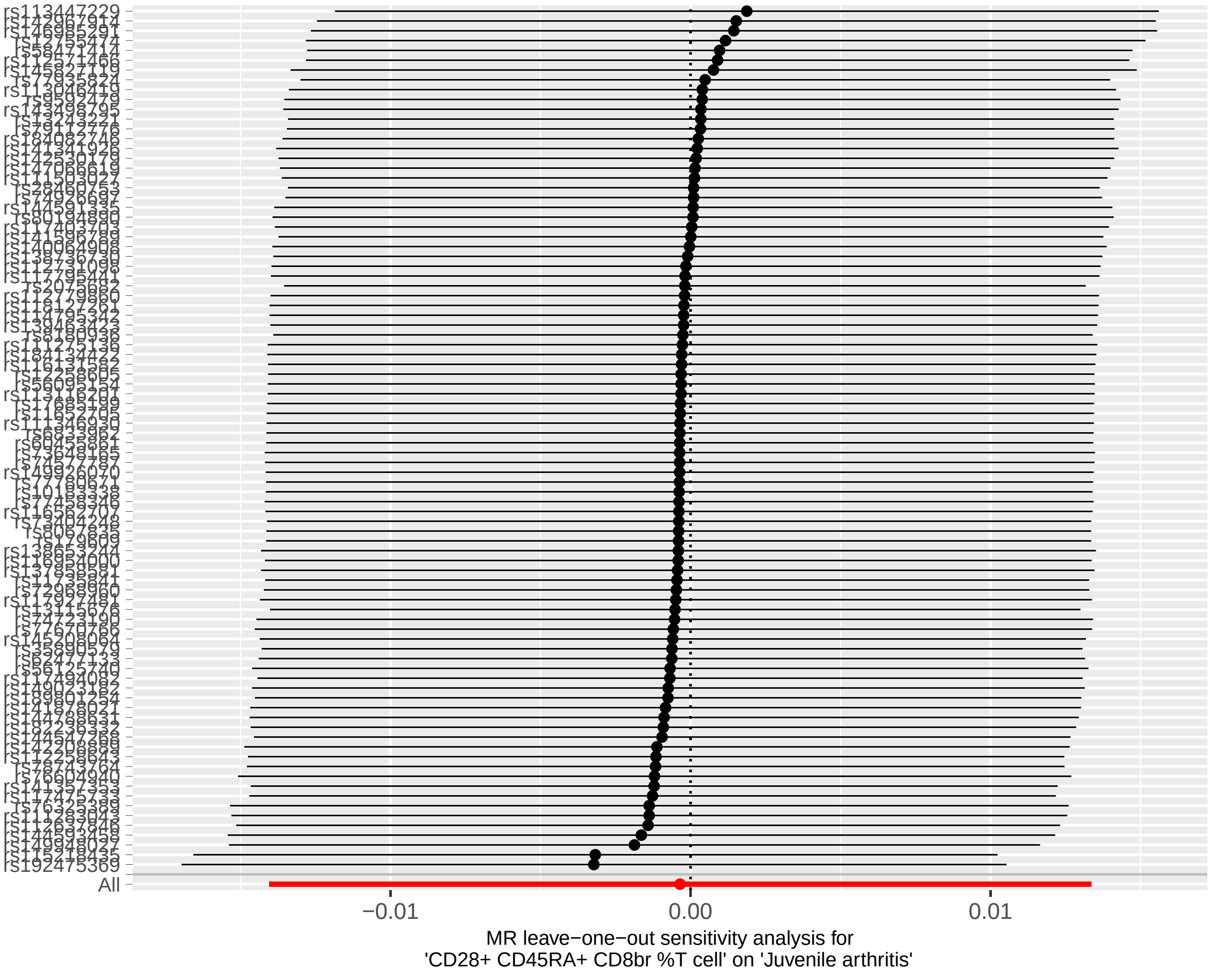
rs113447229
rs192475369
All
−0.01
0.00
0.01
MR leave−one−out sensitivity analysis for
'CD28+ CD45RA+ CD8br %T cell' on 'Juvenile arthritis'

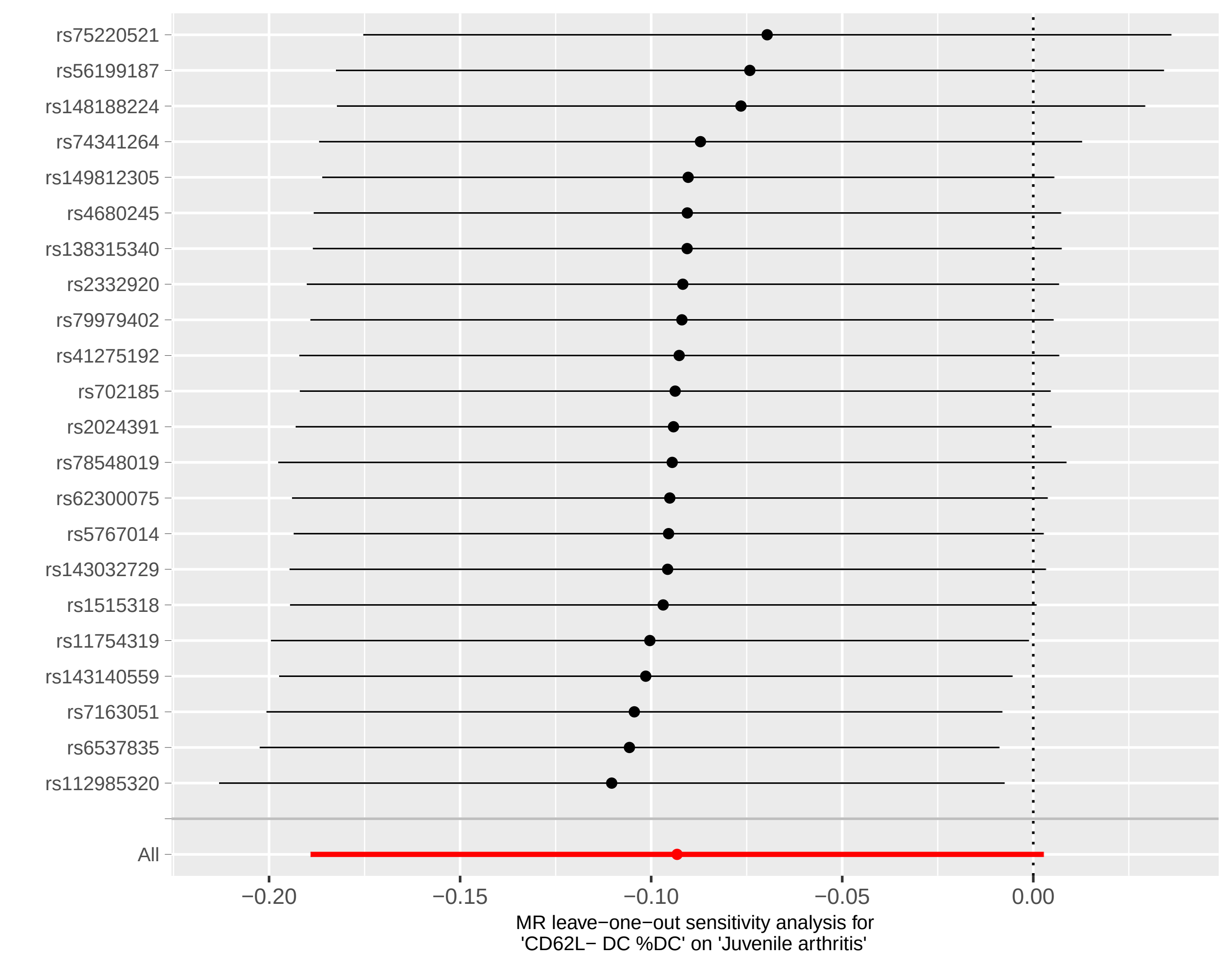

rs75220521
rs56199187
rs148188224
rs74341264
rs149812305
rs4680245
rs138315340
rs2332920
rs79979402
rs41275192
rs702185
rs2024391
rs78548019
rs62300075
rs5767014
rs143032729
rs1515318
rs11754319
rs143140559
rs7163051
rs6537835
rs112985320
All
−0.20
−0.15
−0.10
−0.05
0.00
MR leave−one−out sensitivity analysis for
'CD62L− DC %DC' on 'Juvenile arthritis'

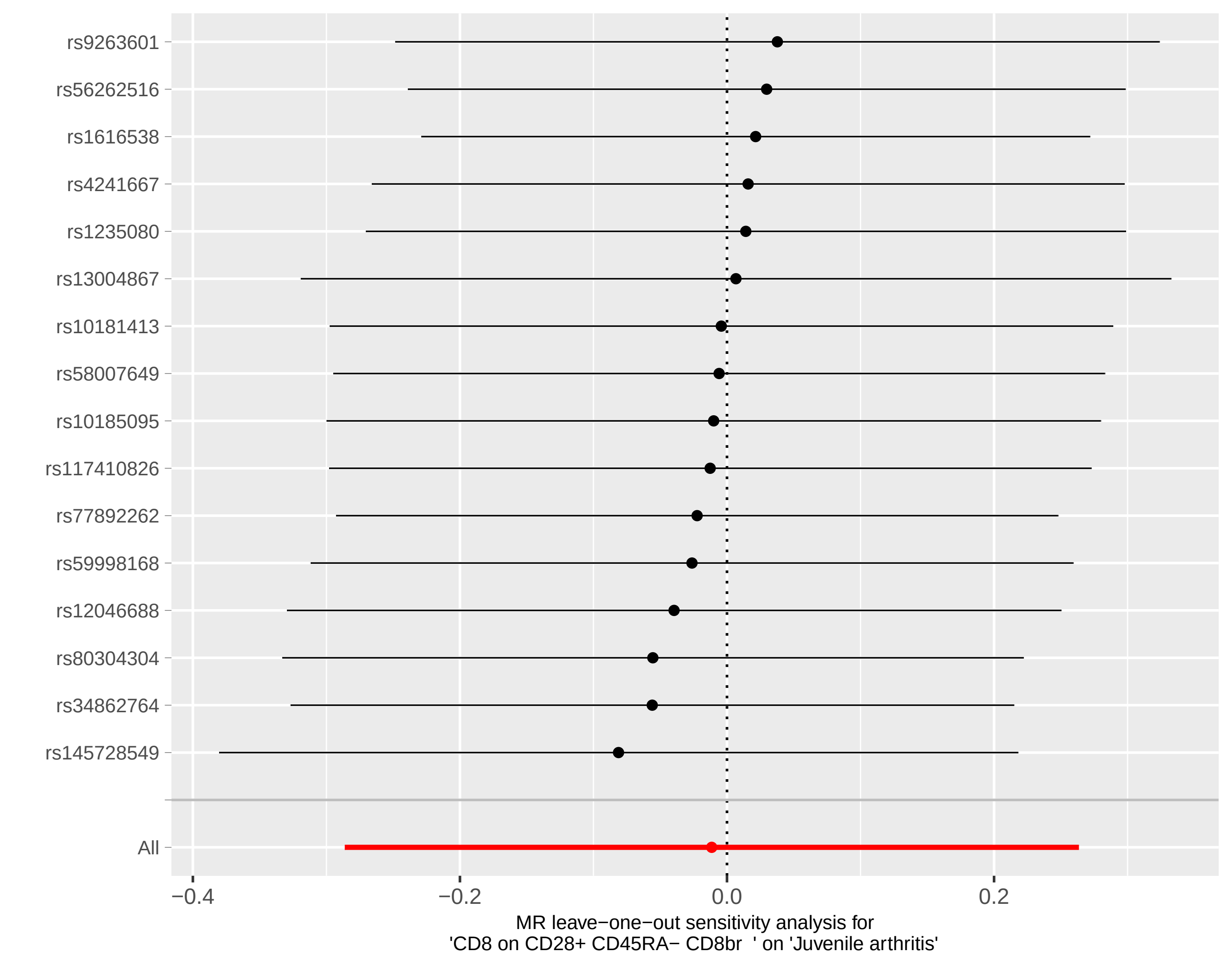
rs9263601
rs56262516
rs1616538
rs4241667
rs1235080
rs13004867
rs10181413
rs58007649
rs10185095
rs117410826
rs77892262
rs59998168
rs12046688
rs80304304
rs34862764
rs145728549
All
−0.4
−0.2
0.0
0.2
MR leave−one−out sensitivity analysis for
'CD8 on CD28+ CD45RA− CD8br ' on 'Juvenile arthritis'

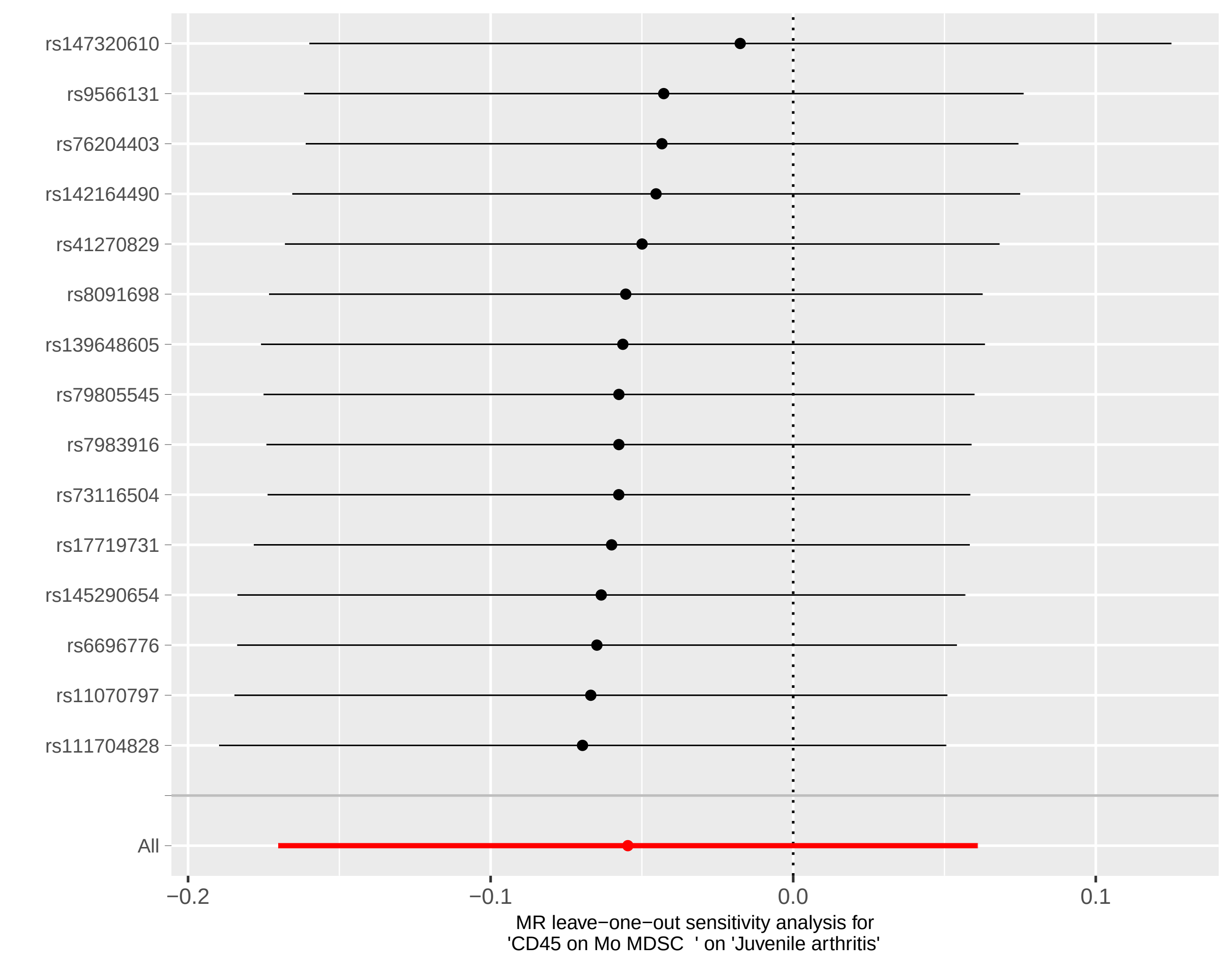
rs147320610
rs9566131
rs76204403
rs142164490
rs41270829
rs8091698
rs139648605
rs79805545
rs7983916
rs73116504
rs17719731
rs145290654
rs6696776
rs11070797
rs111704828
All
−0.2
−0.1
0.0
0.1
MR leave−one−out sensitivity analysis for
'CD45 on Mo MDSC ' on 'Juvenile arthritis'

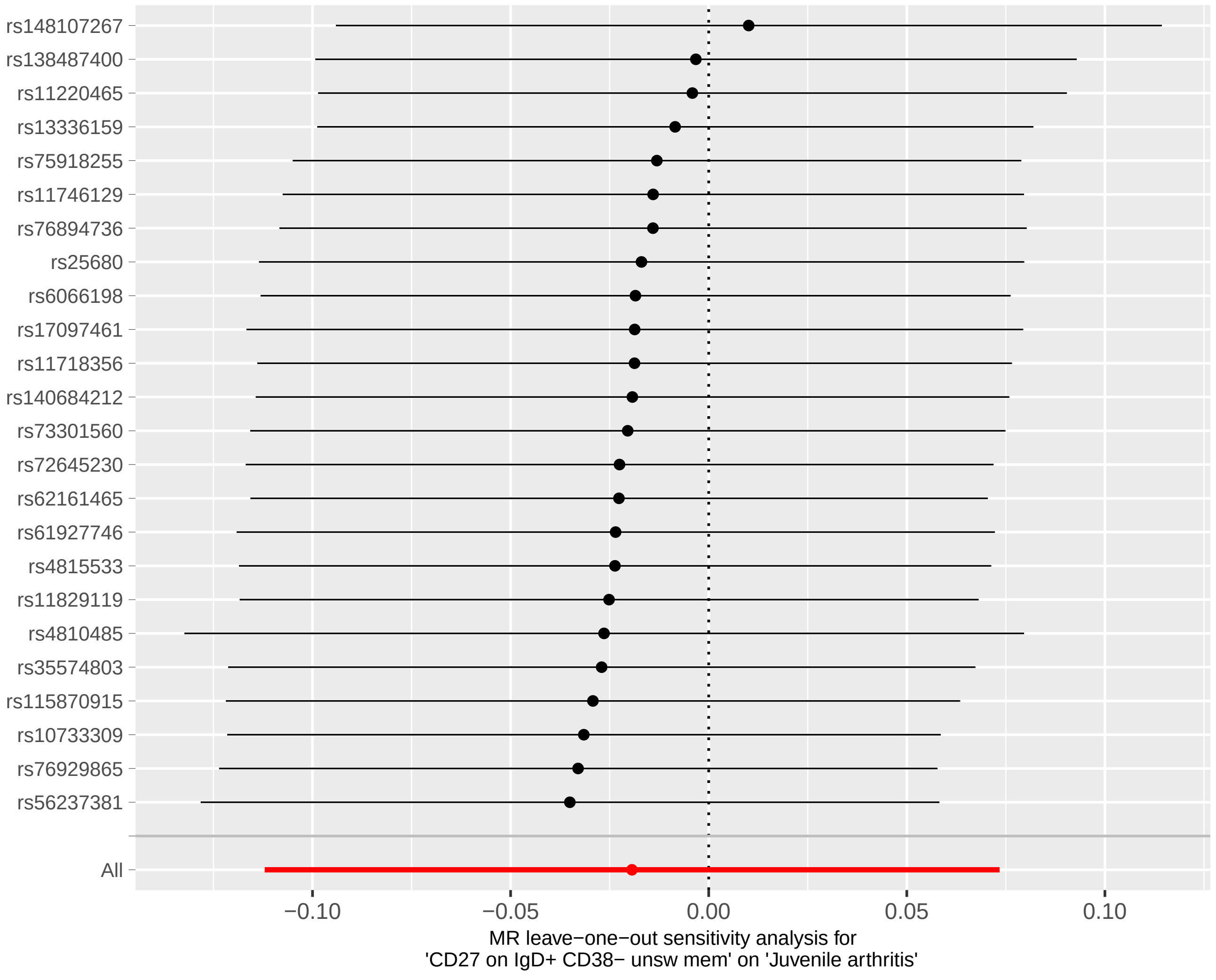
rs148107267
rs138487400
rs11220465
rs13336159
rs75918255
rs11746129
rs76894736
rs25680
rs6066198
rs17097461
rs11718356
rs140684212
rs73301560
rs72645230
rs62161465
rs61927746
rs4815533
rs11829119
rs4810485
rs35574803
rs115870915
rs10733309
rs76929865
rs56237381
All
−0.10
−0.05
0.00
0.05
0.10
MR leave−one−out sensitivity analysis for
'CD27 on IgD+ CD38− unsw mem' on 'Juvenile arthritis'

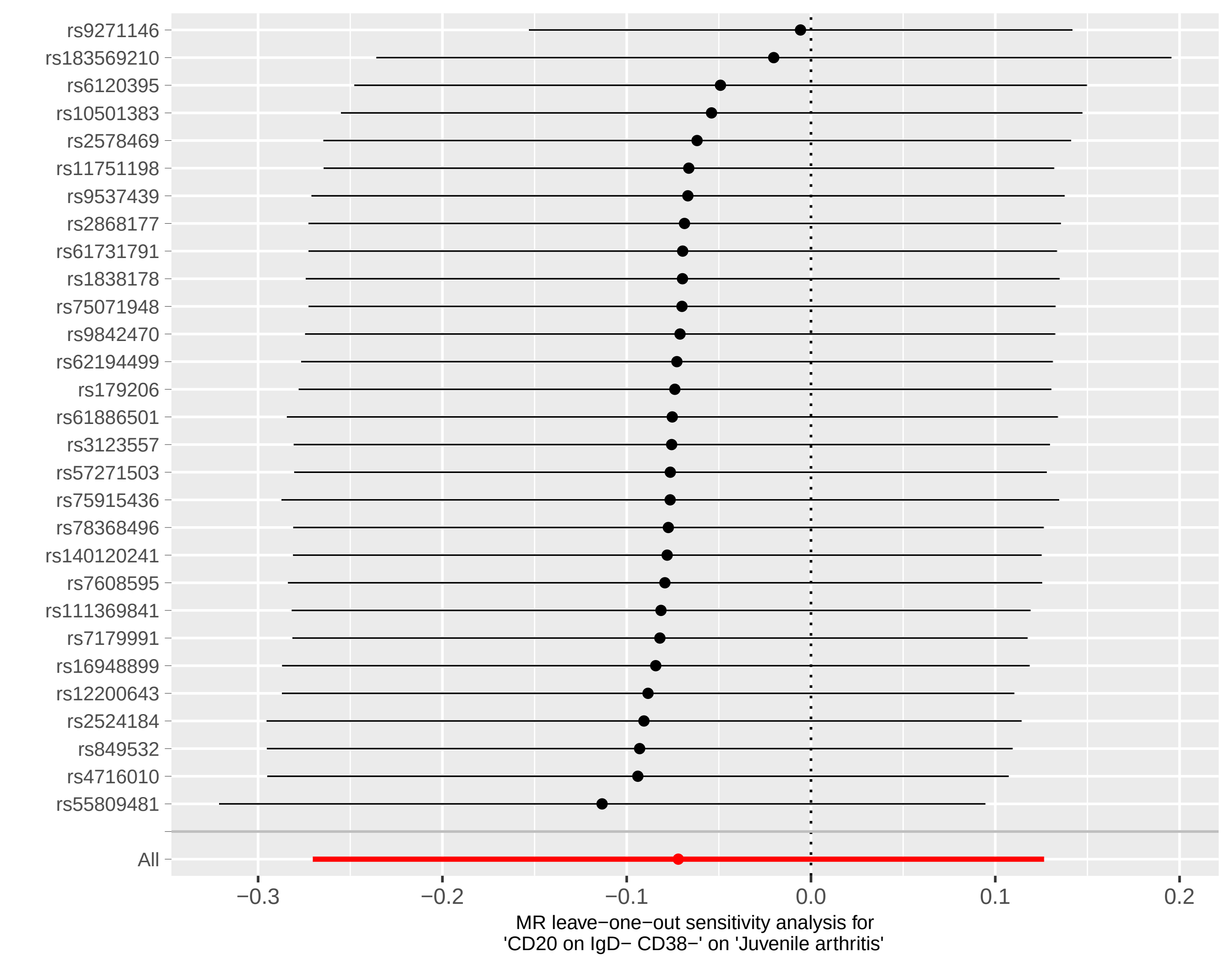
rs9271146
rs183569210
rs6120395
rs10501383
rs2578469
rs11751198
rs9537439
rs2868177
rs61731791
rs1838178
rs75071948
rs9842470
rs62194499
rs179206
rs61886501
rs3123557
rs57271503
rs75915436
rs78368496
rs140120241
rs7608595
rs111369841
rs7179991
rs16948899
rs12200643
rs2524184
rs849532
rs4716010
rs55809481
All
−0.3
−0.2
−0.1
0.0
0.1
0.2
MR leave−one−out sensitivity analysis for
'CD20 on IgD− CD38−' on 'Juvenile arthritis'

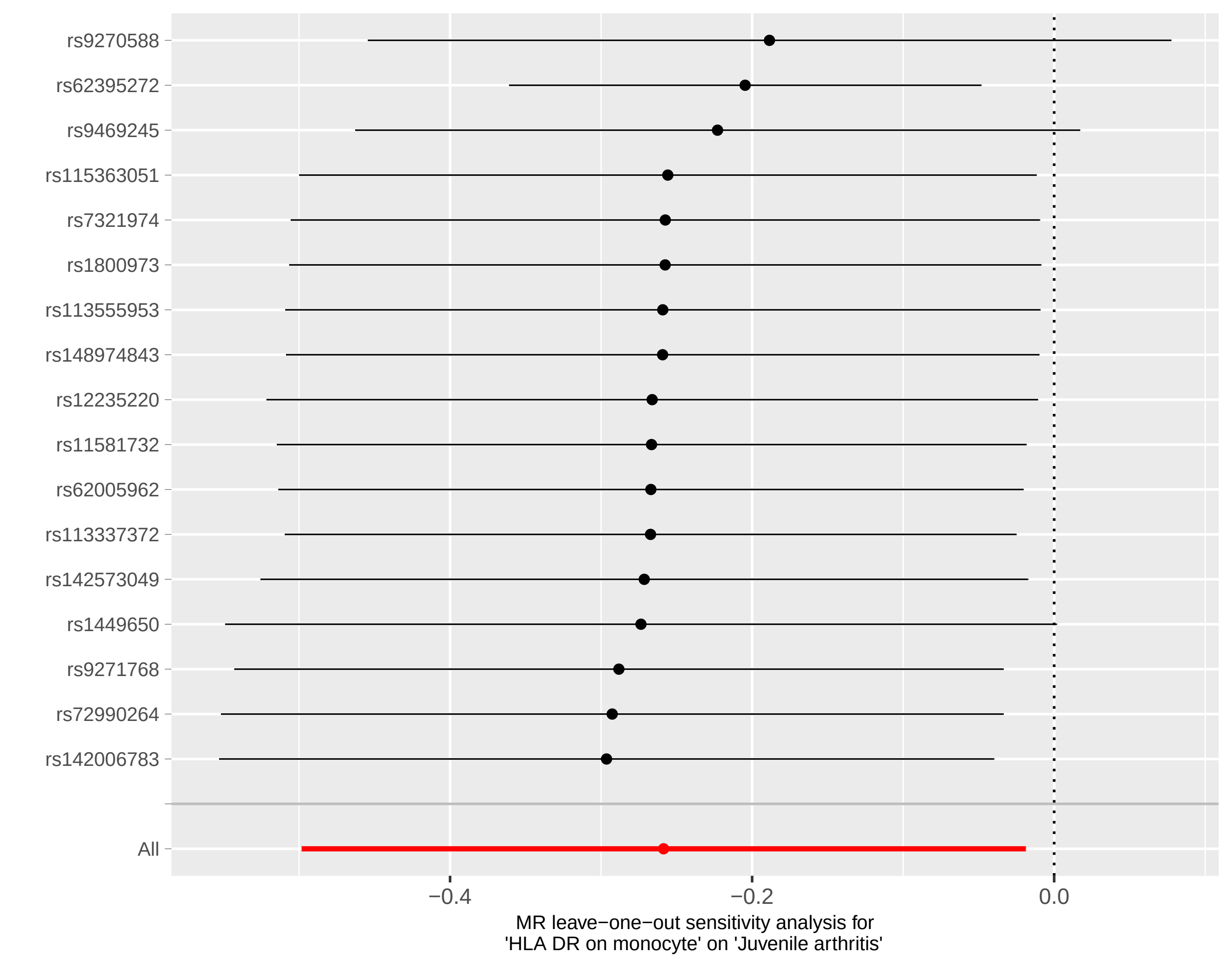
rs9270588
rs62395272
rs9469245
rs115363051
rs7321974
rs1800973
rs113555953
rs148974843
rs12235220
rs11581732
rs62005962
rs113337372
rs142573049
rs1449650
rs9271768
rs72990264
rs142006783
All
−0.4
−0.2
0.0
MR leave−one−out sensitivity analysis for
'HLA DR on monocyte' on 'Juvenile arthritis'

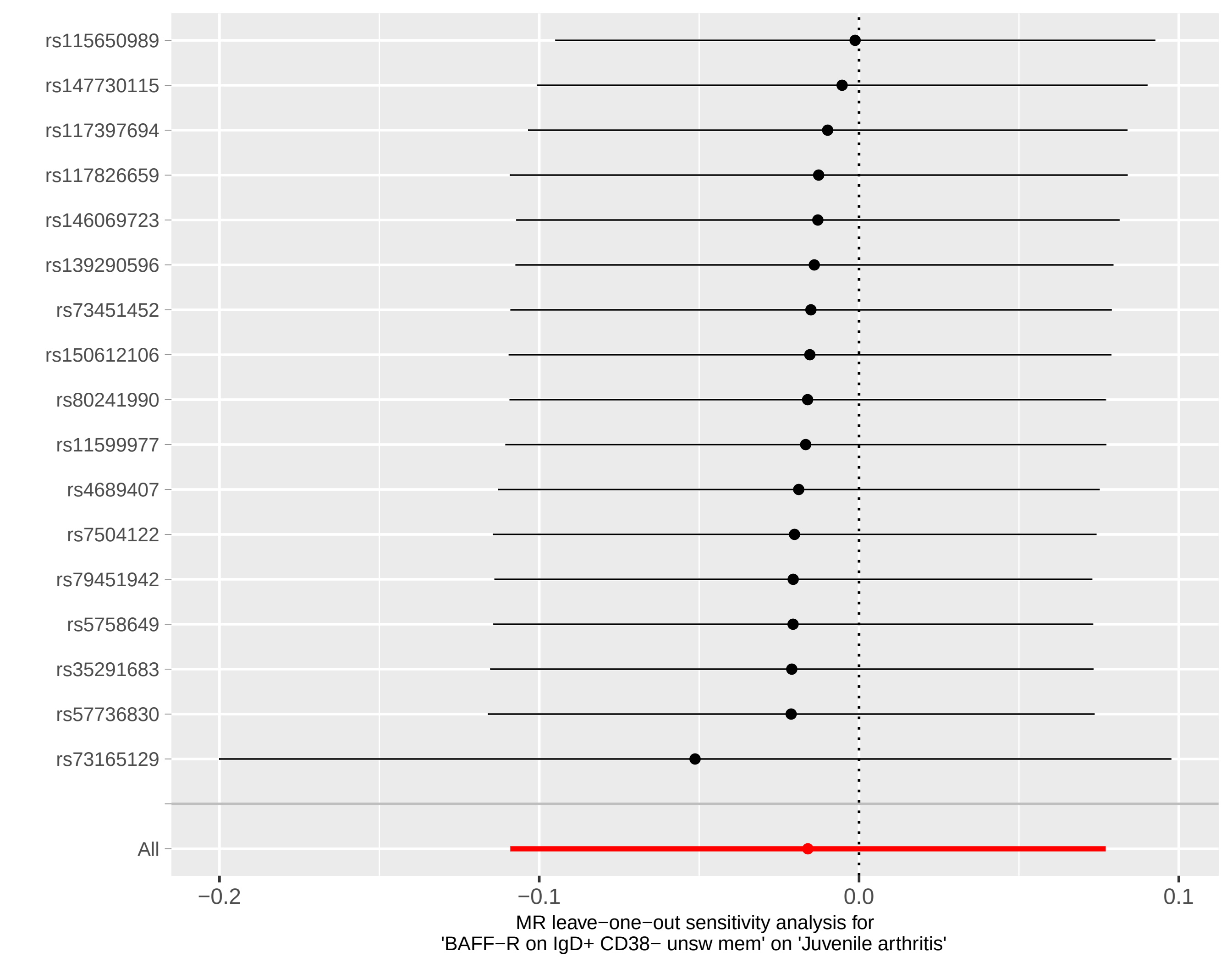

rs115650989
rs147730115
rs117397694
rs117826659
rs146069723
rs139290596
rs73451452
rs150612106
rs80241990
rs11599977
rs4689407
rs7504122
rs79451942
rs5758649
rs35291683
rs57736830
rs73165129
All
−0.2
−0.1
0.0
0.1
MR leave−one−out sensitivity analysis for
'BAFF−R on IgD+ CD38− unsw mem' on 'Juvenile arthritis'

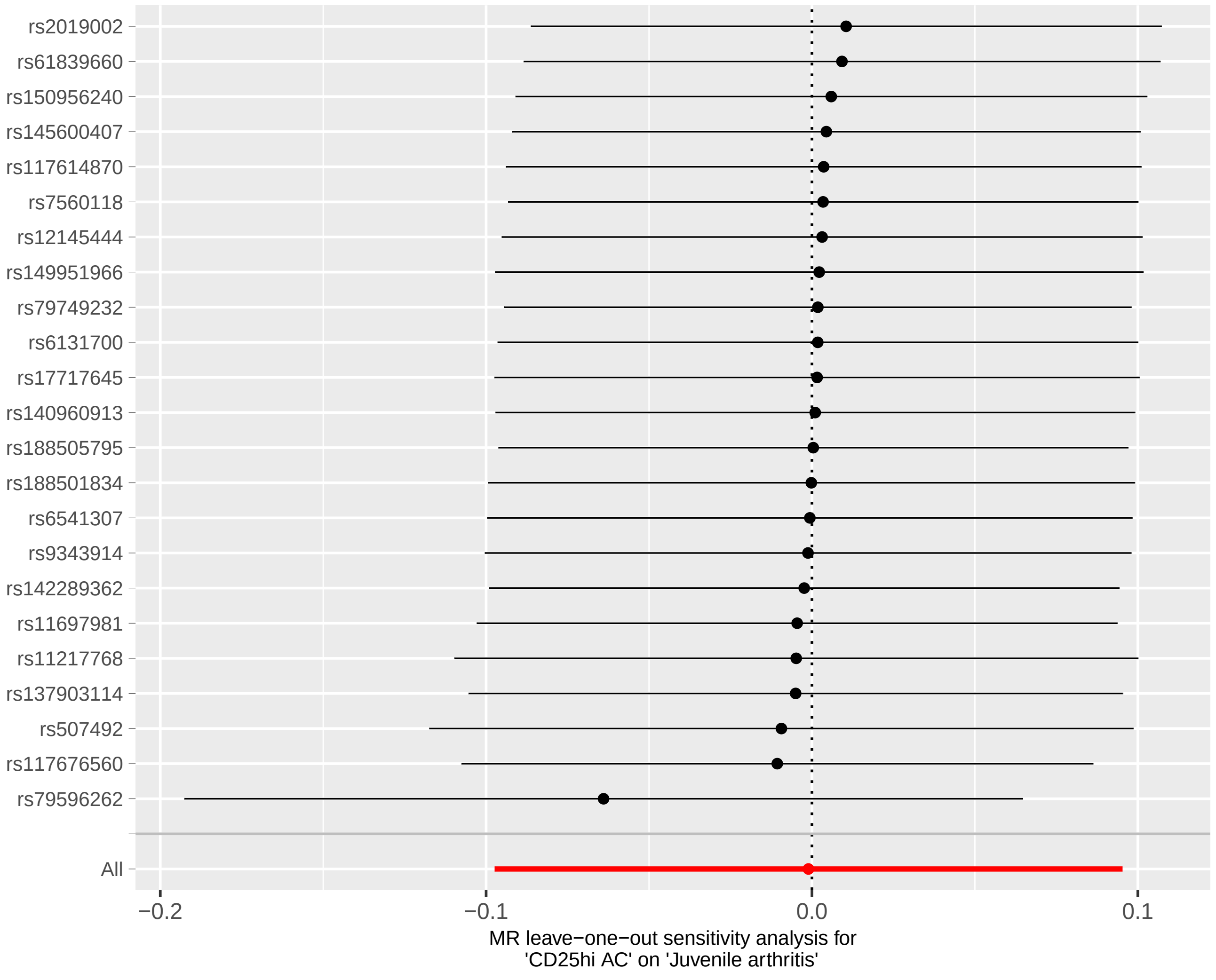
rs2019002
rs61839660
rs150956240
rs145600407
rs117614870
rs7560118
rs12145444
rs149951966
rs79749232
rs6131700
rs17717645
rs140960913
rs188505795
rs188501834
rs6541307
rs9343914
rs142289362
rs11697981
rs11217768
rs137903114
rs507492
rs117676560
rs79596262
All
−0.2
−0.1
0.0
0.1
MR leave−one−out sensitivity analysis for
'CD25hi AC' on 'Juvenile arthritis'

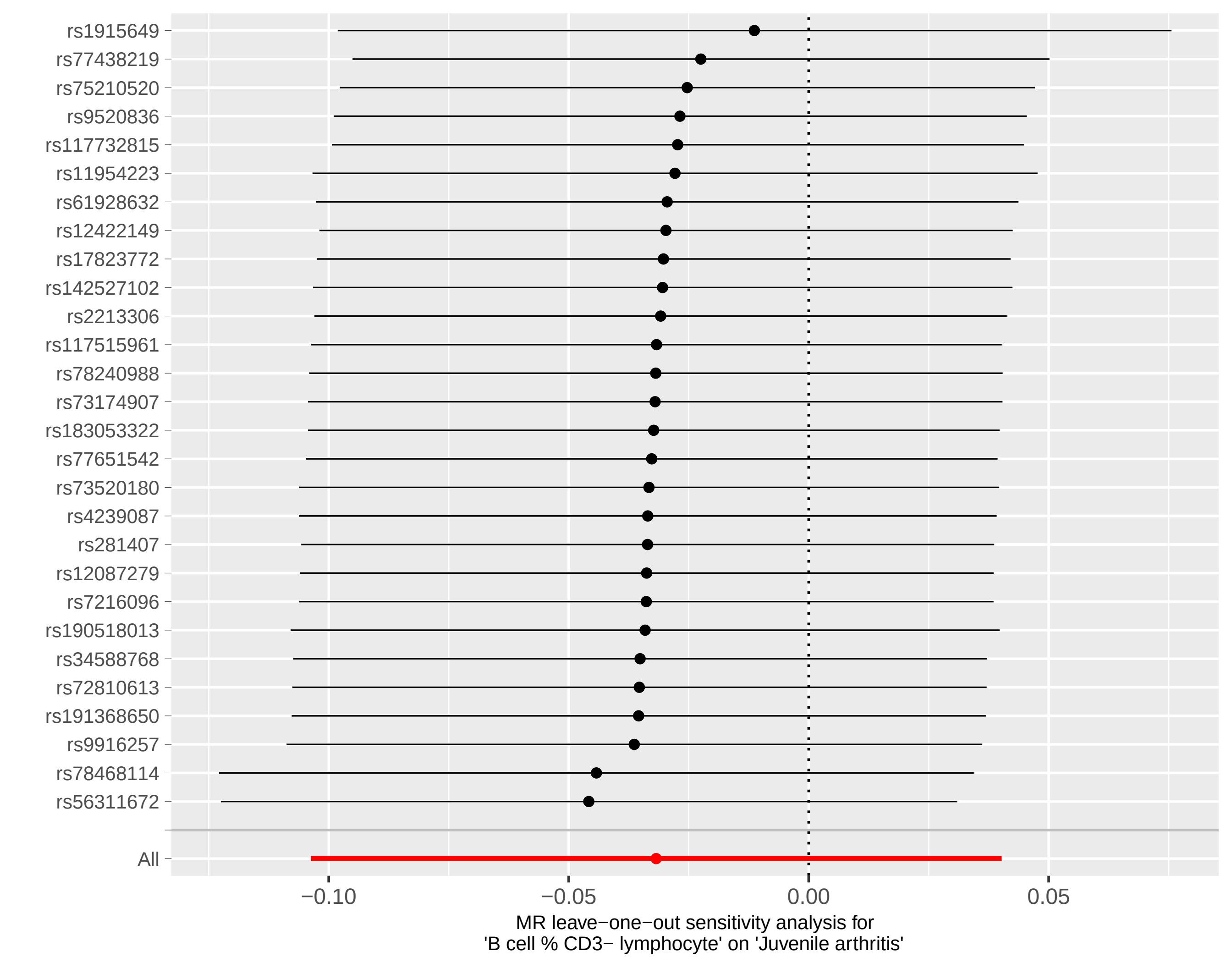
rs1915649
rs77438219
rs75210520
rs9520836
rs117732815
rs11954223
rs61928632
rs12422149
rs17823772
rs142527102
rs2213306
rs117515961
rs78240988
rs73174907
rs183053322
rs77651542
rs73520180
rs4239087
rs281407
rs12087279
rs7216096
rs190518013
rs34588768
rs72810613
rs191368650
rs9916257
rs78468114
rs56311672
All
−0.10
−0.05
0.00
0.05
MR leave−one−out sensitivity analysis for
'B cell % CD3− lymphocyte' on 'Juvenile arthritis'

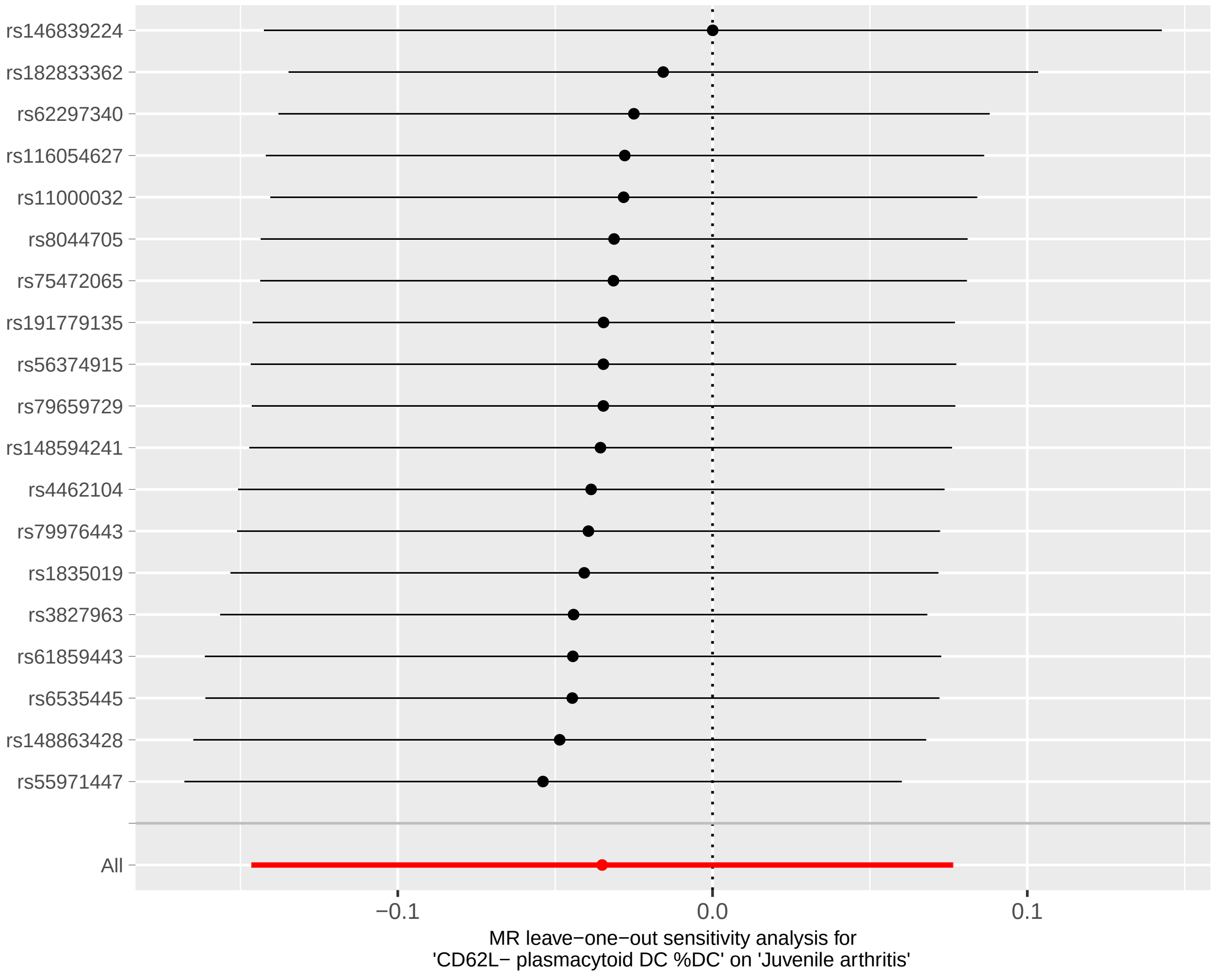
rs146839224
rs182833362
rs62297340
rs116054627
rs11000032
rs8044705
rs75472065
rs191779135
rs56374915
rs79659729
rs148594241
rs4462104
rs79976443
rs1835019
rs3827963
rs61859443
rs6535445
rs148863428
rs55971447
All
−0.1
0.0
0.1
MR leave−one−out sensitivity analysis for
'CD62L− plasmacytoid DC %DC' on 'Juvenile arthritis'

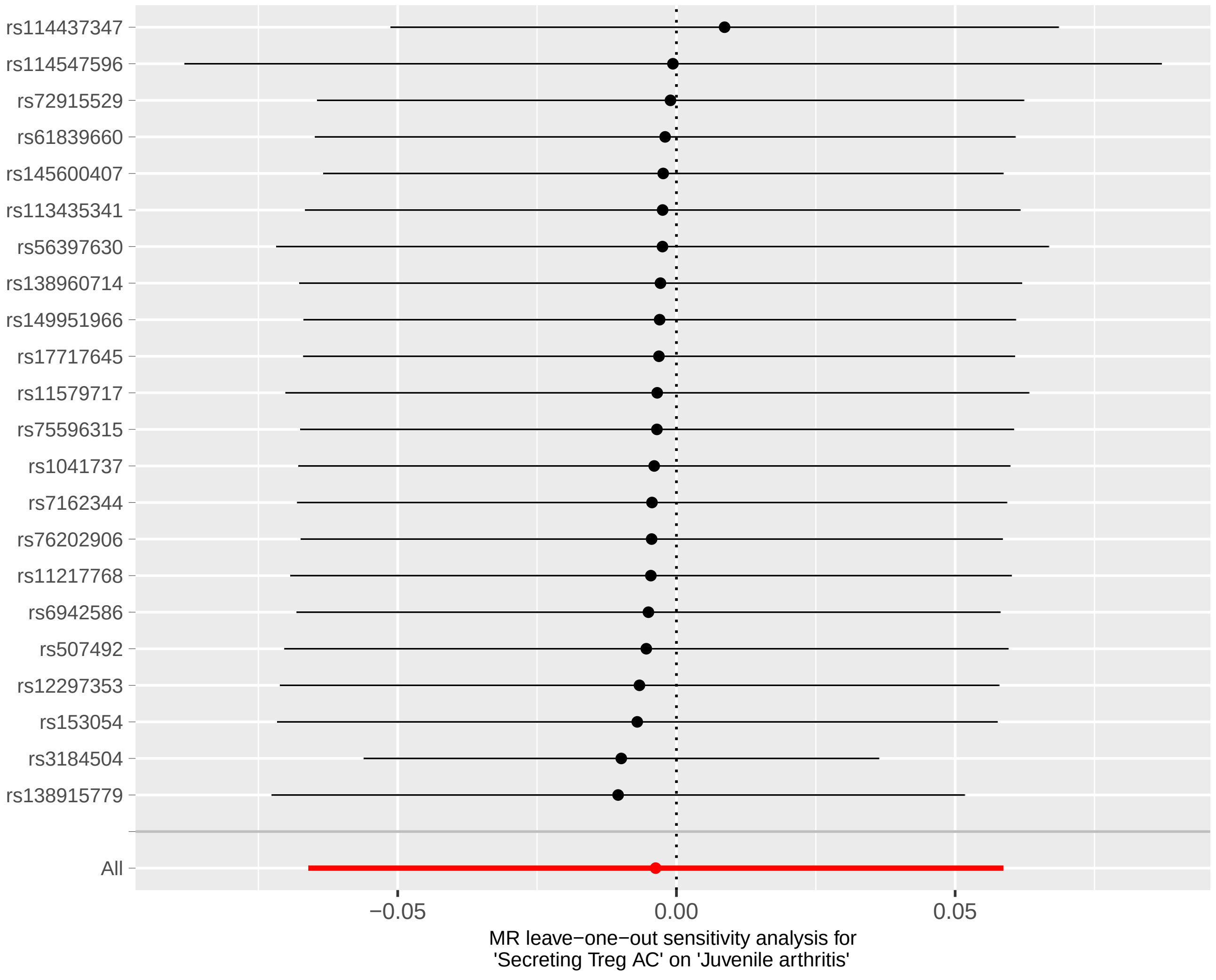
rs114437347
rs114547596
rs72915529
rs61839660
rs145600407
rs113435341
rs56397630
rs138960714
rs149951966
rs17717645
rs11579717
rs75596315
rs1041737
rs7162344
rs76202906
rs11217768
rs6942586
rs507492
rs12297353
rs153054
rs3184504
rs138915779
All
−0.05
0.00
0.05
MR leave−one−out sensitivity analysis for
'Secreting Treg AC' on 'Juvenile arthritis'

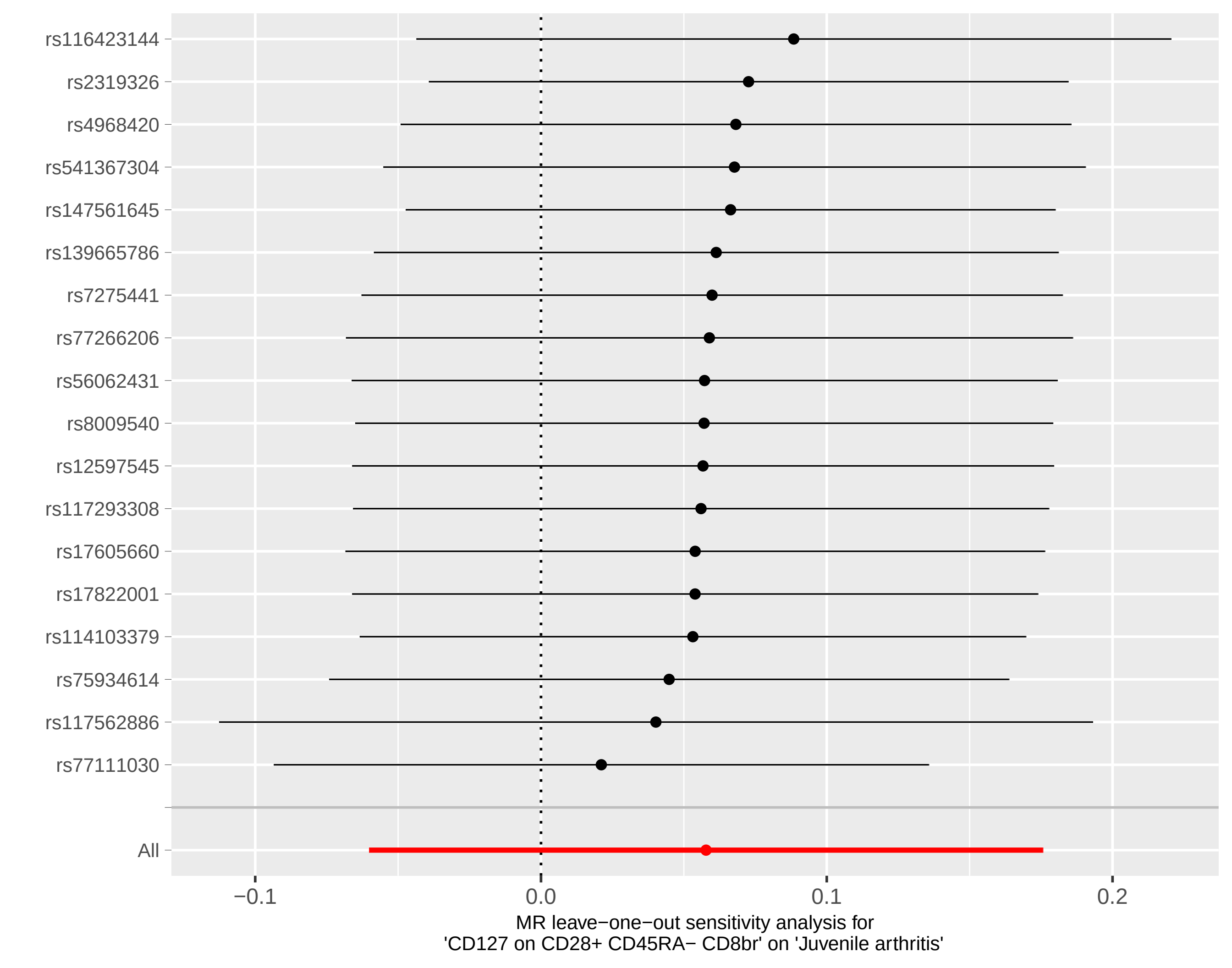
rs116423144
rs2319326
rs4968420
rs541367304
rs147561645
rs139665786
rs7275441
rs77266206
rs56062431
rs8009540
rs12597545
rs117293308
rs17605660
rs17822001
rs114103379
rs75934614
rs117562886
rs77111030
All
−0.1
0.0
0.1
0.2
MR leave−one−out sensitivity analysis for
'CD127 on CD28+ CD45RA− CD8br' on 'Juvenile arthritis'

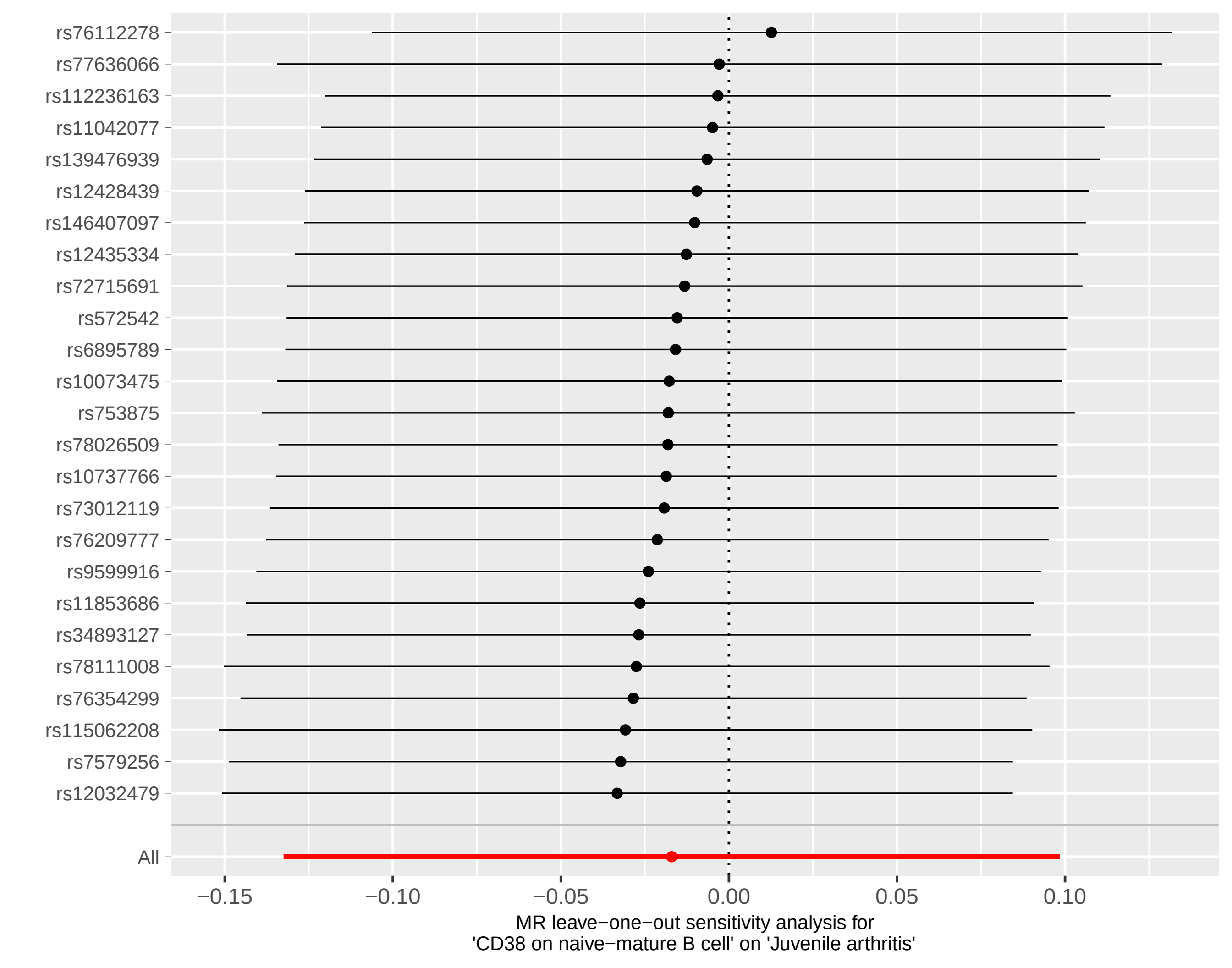
rs76112278
rs77636066
rs112236163
rs11042077
rs139476939
rs12428439
rs146407097
rs12435334
rs72715691
rs572542
rs6895789
rs10073475
rs753875
rs78026509
rs10737766
rs73012119
rs76209777
rs9599916
rs11853686
rs34893127
rs78111008
rs76354299
rs115062208
rs7579256
rs12032479
All
−0.15
−0.10
−0.05
0.00
0.05
0.10
MR leave−one−out sensitivity analysis for
'CD38 on naive−mature B cell' on 'Juvenile arthritis'

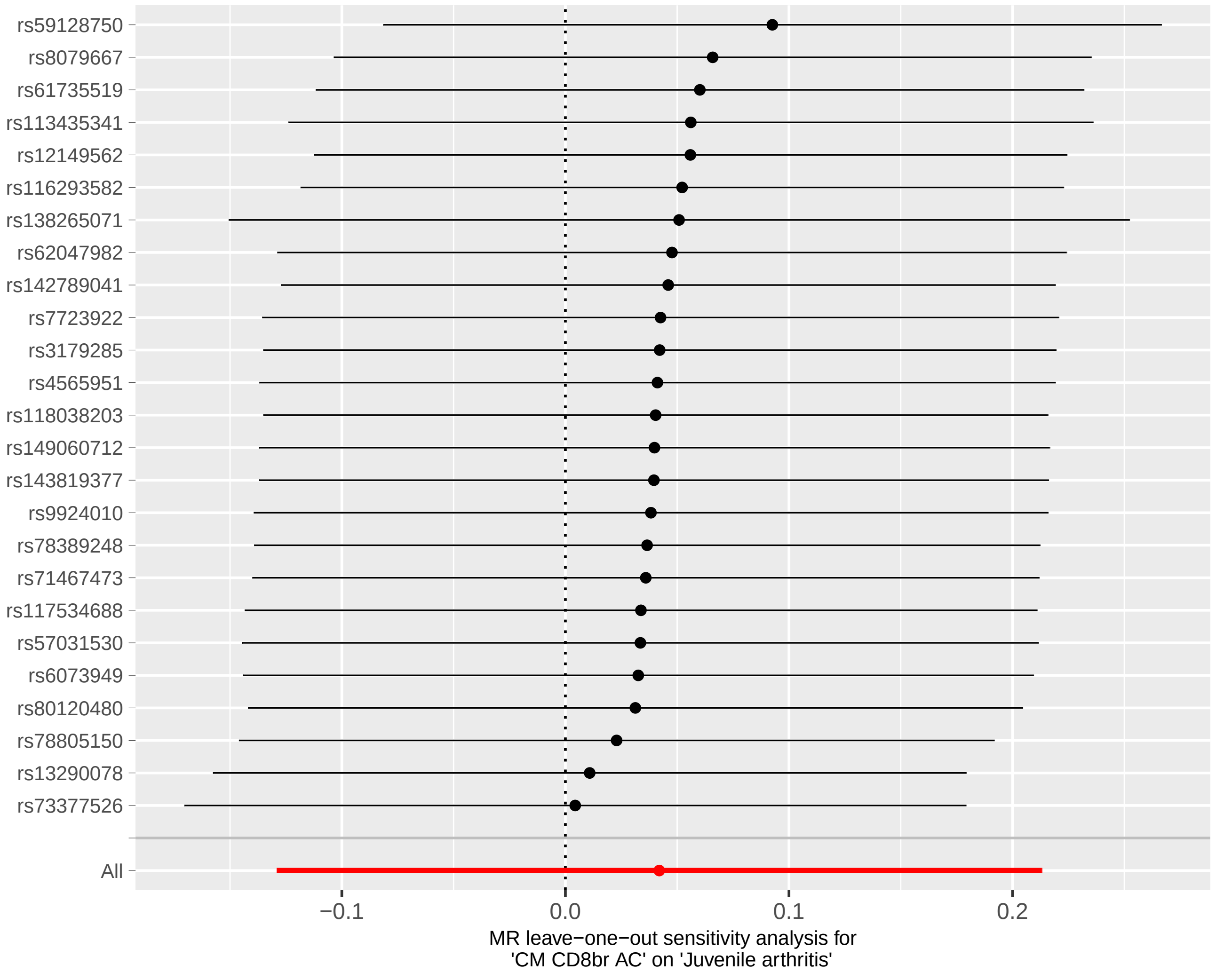

rs59128750
rs8079667
rs61735519
rs113435341
rs12149562
rs116293582
rs138265071
rs62047982
rs142789041
rs7723922
rs3179285
rs4565951
rs118038203
rs149060712
rs143819377
rs9924010
rs78389248
rs71467473
rs117534688
rs57031530
rs6073949
rs80120480
rs78805150
rs13290078
rs73377526
All
−0.1
0.0
0.1
0.2
MR leave−one−out sensitivity analysis for
'CM CD8br AC' on 'Juvenile arthritis'

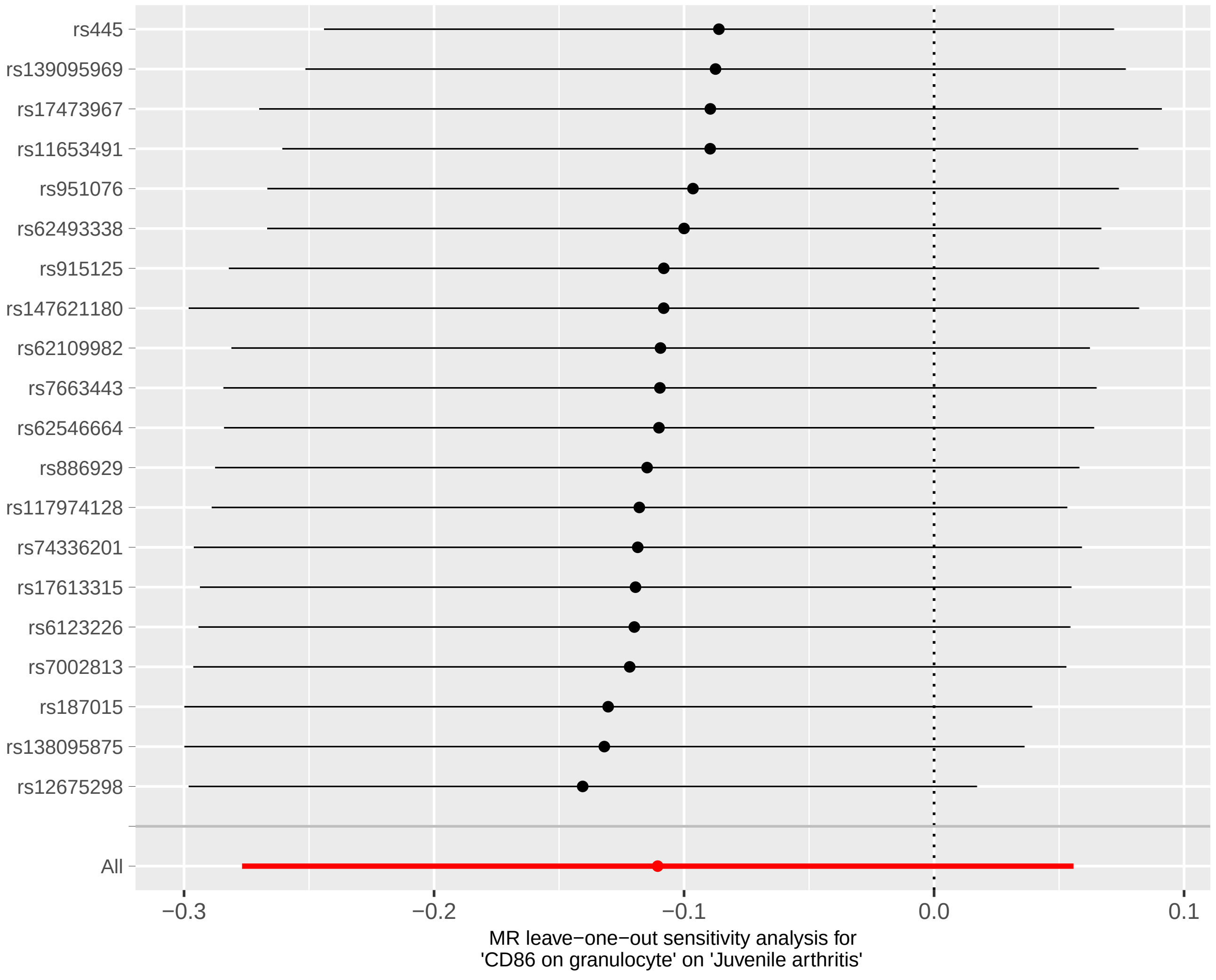

rs445
rs139095969
rs17473967
rs11653491
rs951076
rs62493338
rs915125
rs147621180
rs62109982
rs7663443
rs62546664
rs886929
rs117974128
rs74336201
rs17613315
rs6123226
rs7002813
rs187015
rs138095875
rs12675298
All
−0.3
−0.2
−0.1
0.0
0.1
MR leave−one−out sensitivity analysis for
'CD86 on granulocyte' on 'Juvenile arthritis'

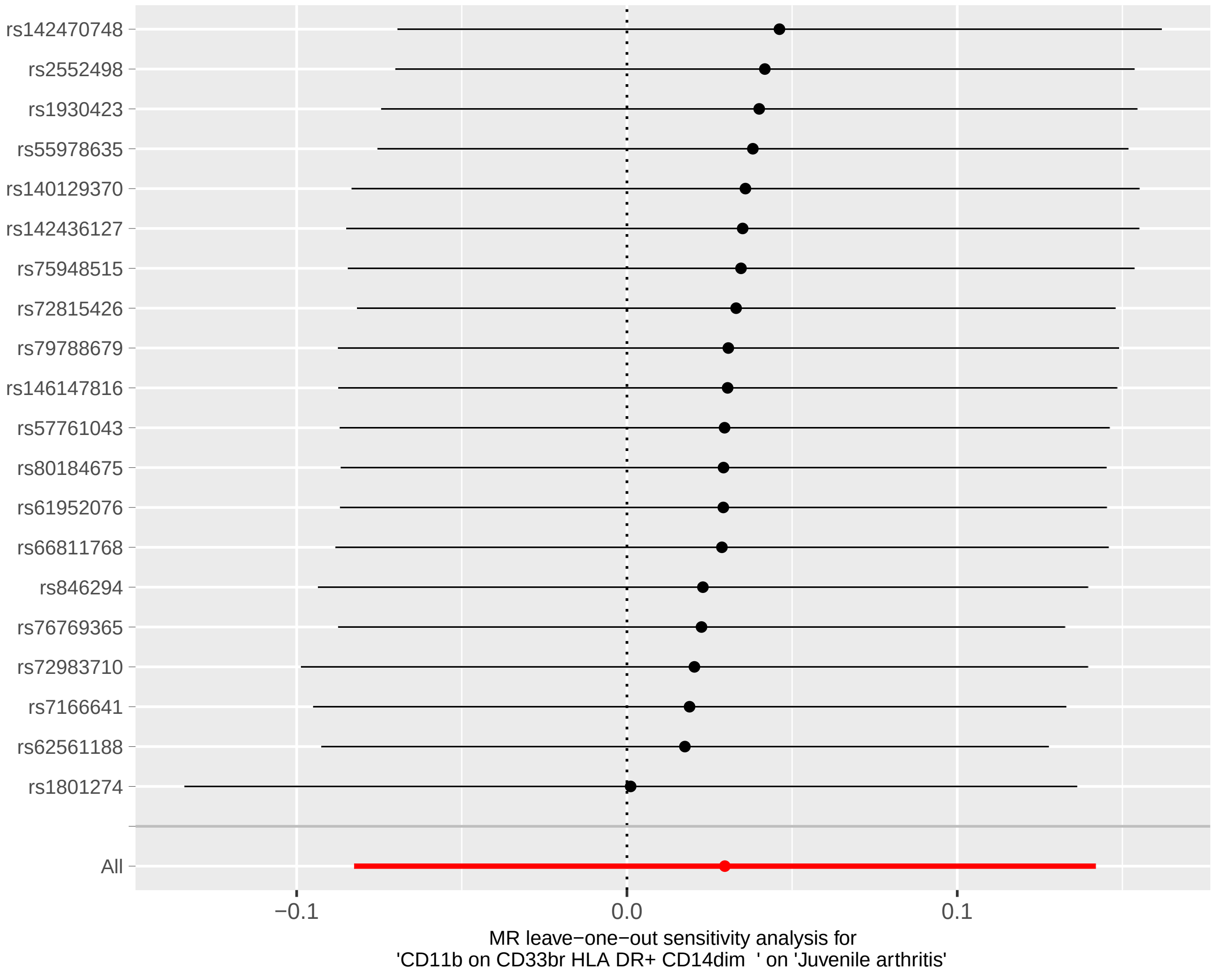

rs142470748
rs2552498
rs1930423
rs55978635
rs140129370
rs142436127
rs75948515
rs72815426
rs79788679
rs146147816
rs57761043
rs80184675
rs61952076
rs66811768
rs846294
rs76769365
rs72983710
rs7166641
rs62561188
rs1801274
All
−0.1
0.0
0.1
MR leave−one−out sensitivity analysis for
'CD11b on CD33br HLA DR+ CD14dim ' on 'Juvenile arthritis'

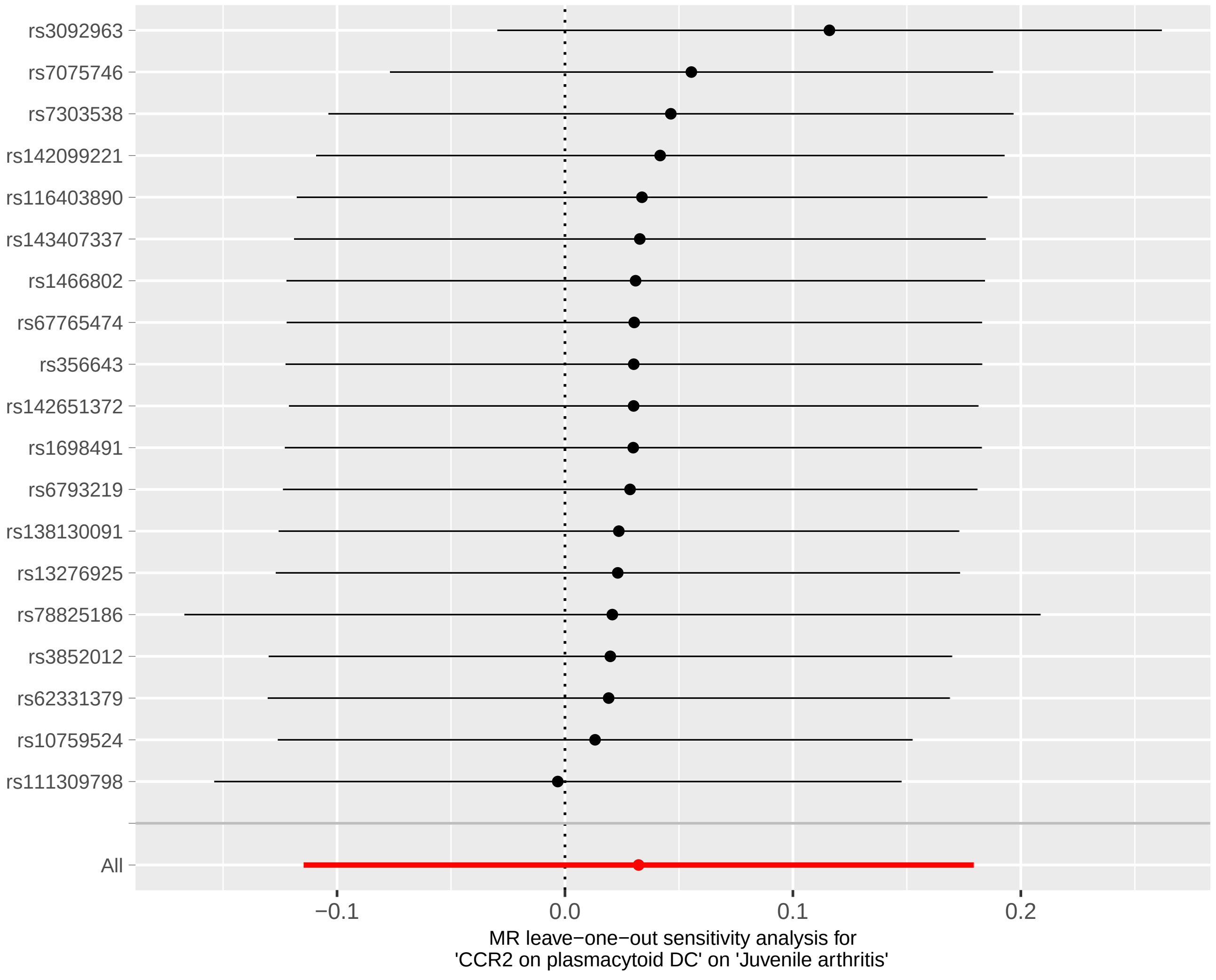
rs3092963
rs7075746
rs7303538
rs142099221
rs116403890
rs143407337
rs1466802
rs67765474
rs356643
rs142651372
rs1698491
rs6793219
rs138130091
rs13276925
rs78825186
rs3852012
rs62331379
rs10759524
rs111309798
All
−0.1
0.0
0.1
0.2
MR leave−one−out sensitivity analysis for
'CCR2 on plasmacytoid DC' on 'Juvenile arthritis'

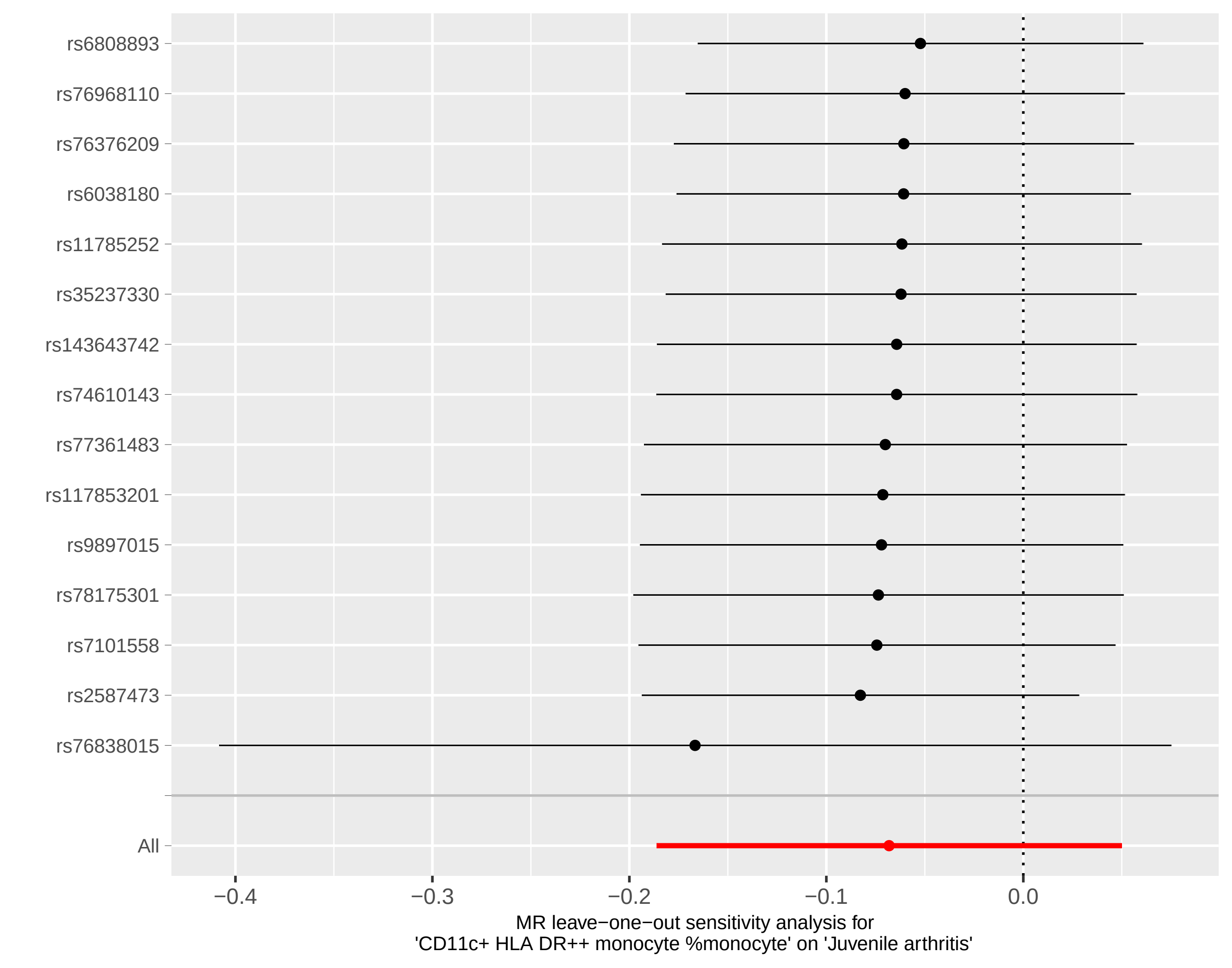
rs6808893
rs76968110
rs76376209
rs6038180
rs11785252
rs35237330
rs143643742
rs74610143
rs77361483
rs117853201
rs9897015
rs78175301
rs7101558
rs2587473
rs76838015
All
−0.4
−0.3
−0.2
−0.1
0.0
MR leave−one−out sensitivity analysis for
'CD11c+ HLA DR++ monocyte %monocyte' on 'Juvenile arthritis'

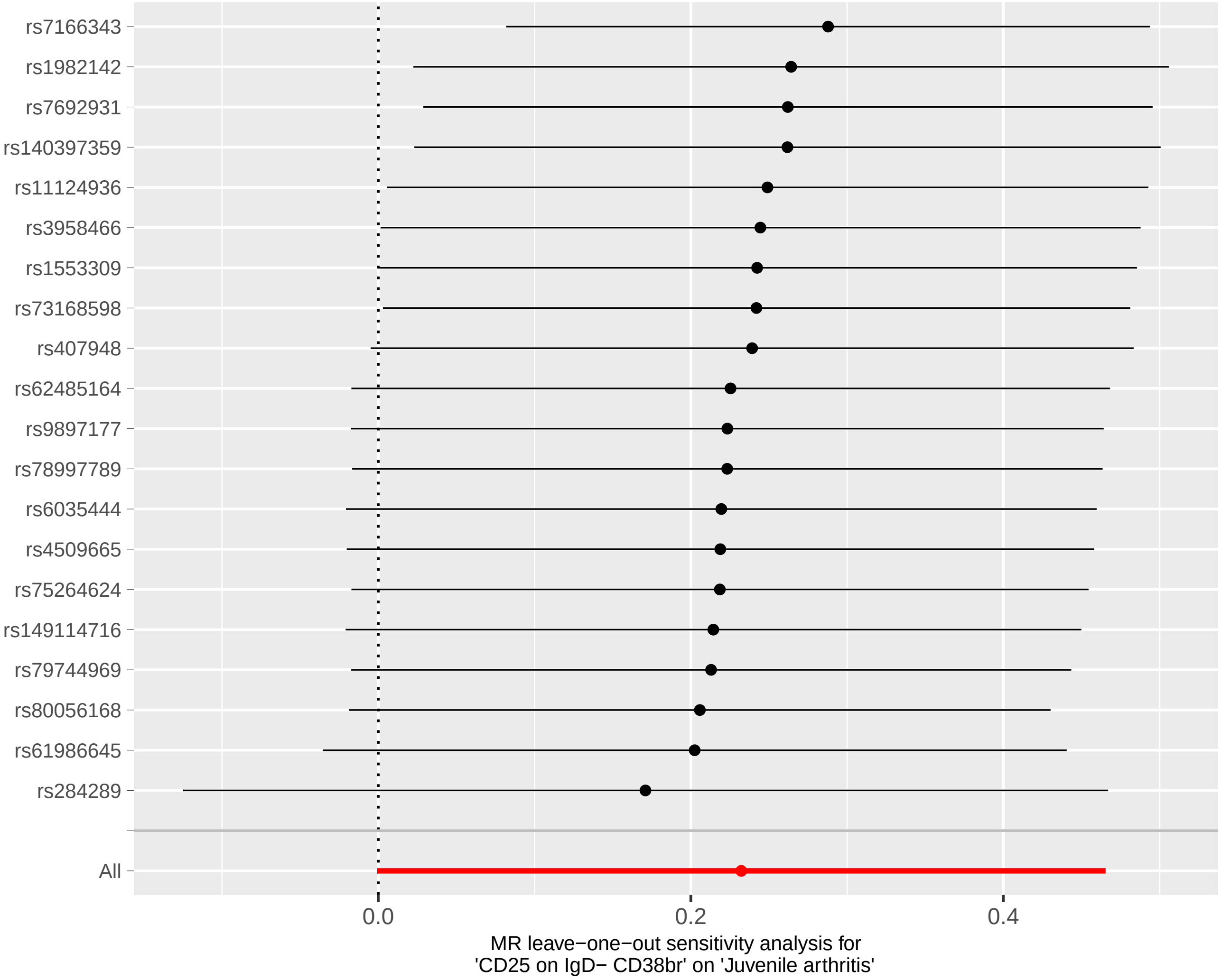

rs7166343
rs1982142
rs7692931
rs140397359
rs11124936
rs3958466
rs1553309
rs73168598
rs407948
rs62485164
rs9897177
rs78997789
rs6035444
rs4509665
rs75264624
rs149114716
rs79744969
rs80056168
rs61986645
rs284289
All
0.0
0.2
0.4
MR leave−one−out sensitivity analysis for
'CD25 on IgD− CD38br' on 'Juvenile arthritis'

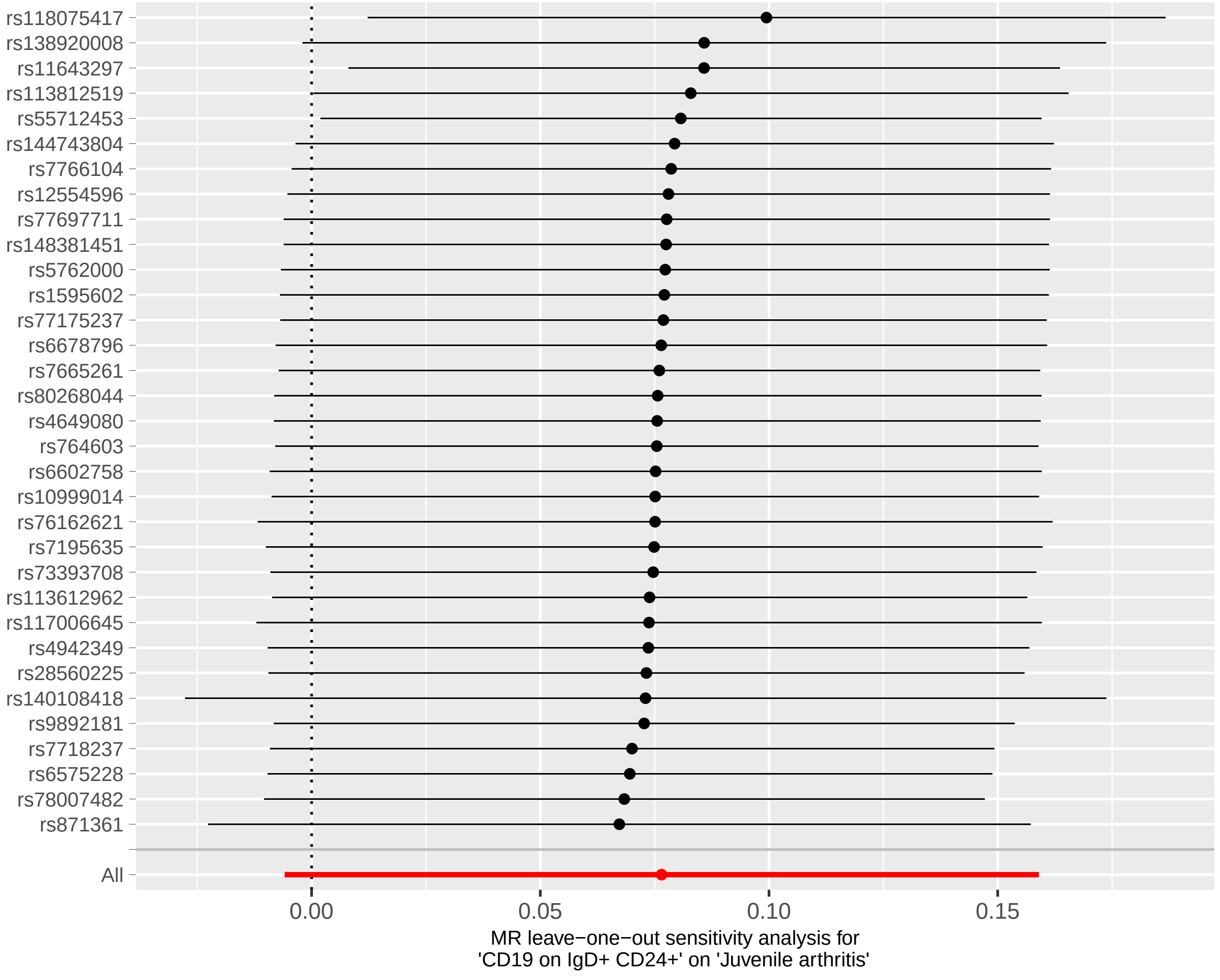

rs118075417
rs138920008
rs11643297
rs113812519
rs55712453
rs144743804
rs7766104
rs12554596
rs77697711
rs148381451
rs5762000
rs1595602
rs77175237
rs6678796
rs7665261
rs80268044
rs4649080
rs764603
rs6602758
rs10999014
rs76162621
rs7195635
rs73393708
rs113612962
rs117006645
rs4942349
rs28560225
rs140108418
rs9892181
rs7718237
rs6575228
rs78007482
rs871361
All
0.00
0.05
0.10
0.15
MR leave−one−out sensitivity analysis for
'CD19 on IgD+ CD24+' on 'Juvenile arthritis'

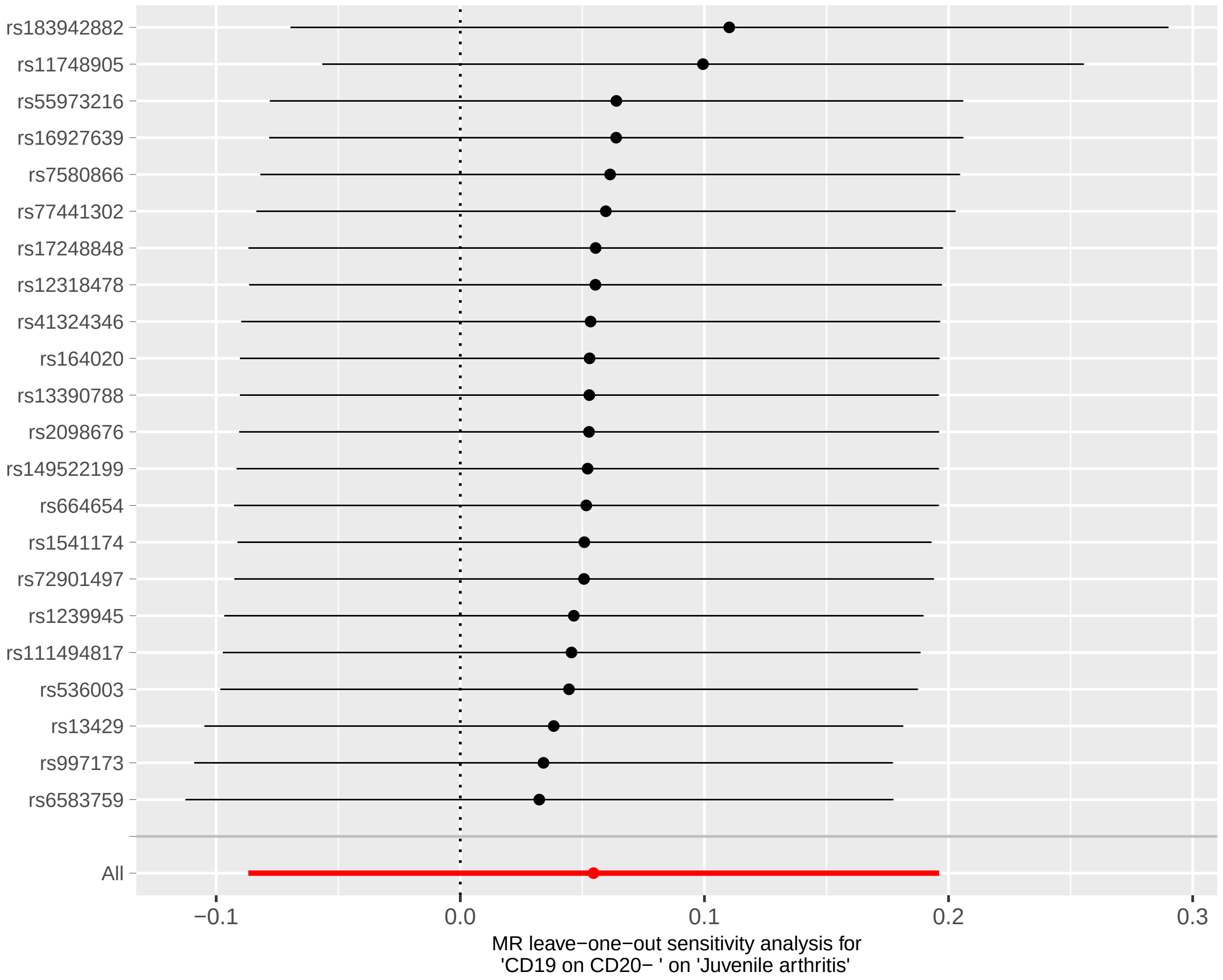
rs183942882
rs11748905
rs55973216
rs16927639
rs7580866
rs77441302
rs17248848
rs12318478
rs41324346
rs164020
rs13390788
rs2098676
rs149522199
rs664654
rs1541174
rs72901497
rs1239945
rs111494817
rs536003
rs13429
rs997173
rs6583759
All
−0.1
0.0
0.1
0.2
0.3
MR leave−one−out sensitivity analysis for
'CD19 on CD20− ' on 'Juvenile arthritis'

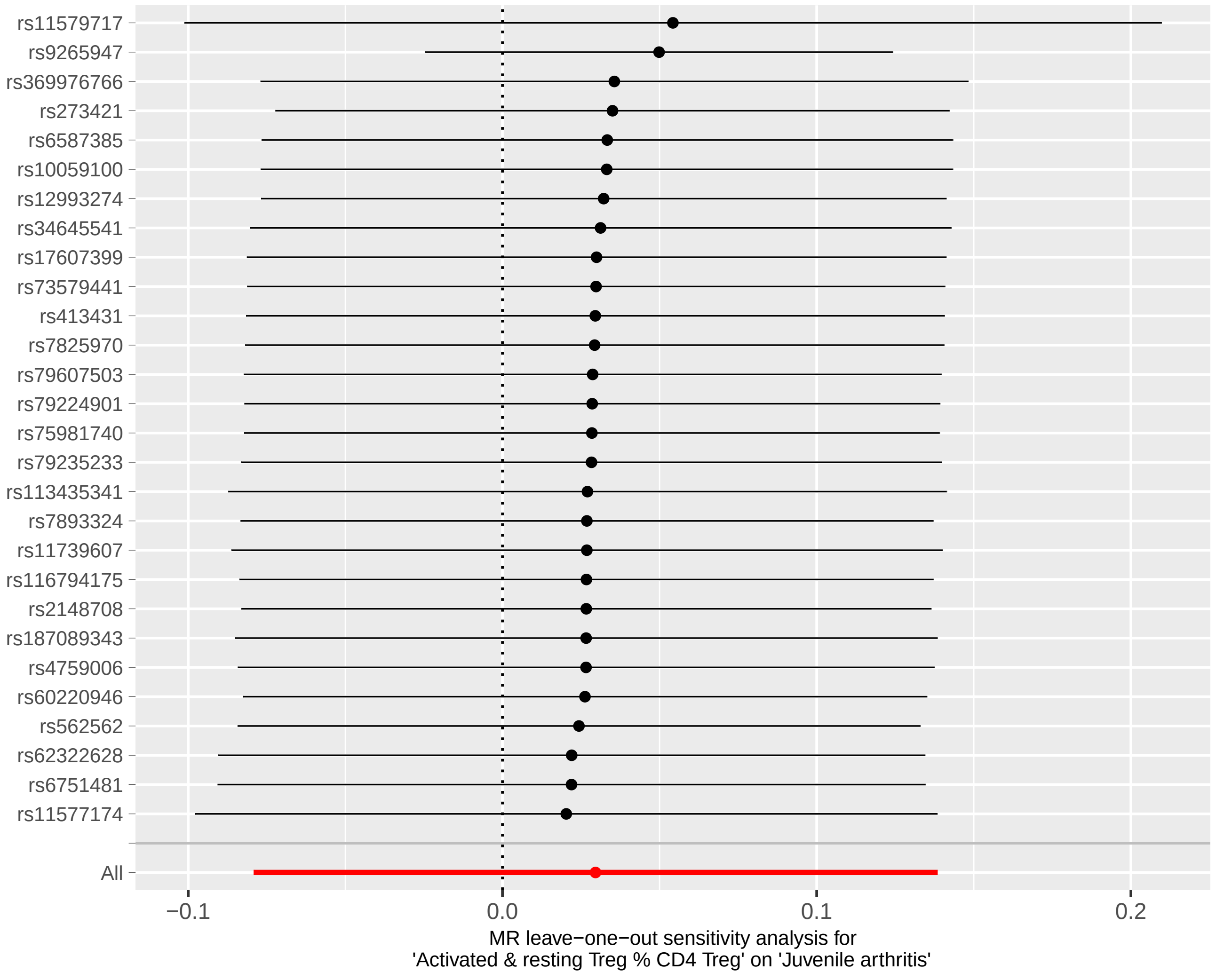

rs11579717
rs9265947
rs369976766
rs273421
rs6587385
rs10059100
rs12993274
rs34645541
rs17607399
rs73579441
rs413431
rs7825970
rs79607503
rs79224901
rs75981740
rs79235233
rs113435341
rs7893324
rs11739607
rs116794175
rs2148708
rs187089343
rs4759006
rs60220946
rs562562
rs62322628
rs6751481
rs11577174
All
−0.1
0.0
0.1
0.2
MR leave−one−out sensitivity analysis for
'Activated & resting Treg % CD4 Treg' on 'Juvenile arthritis'

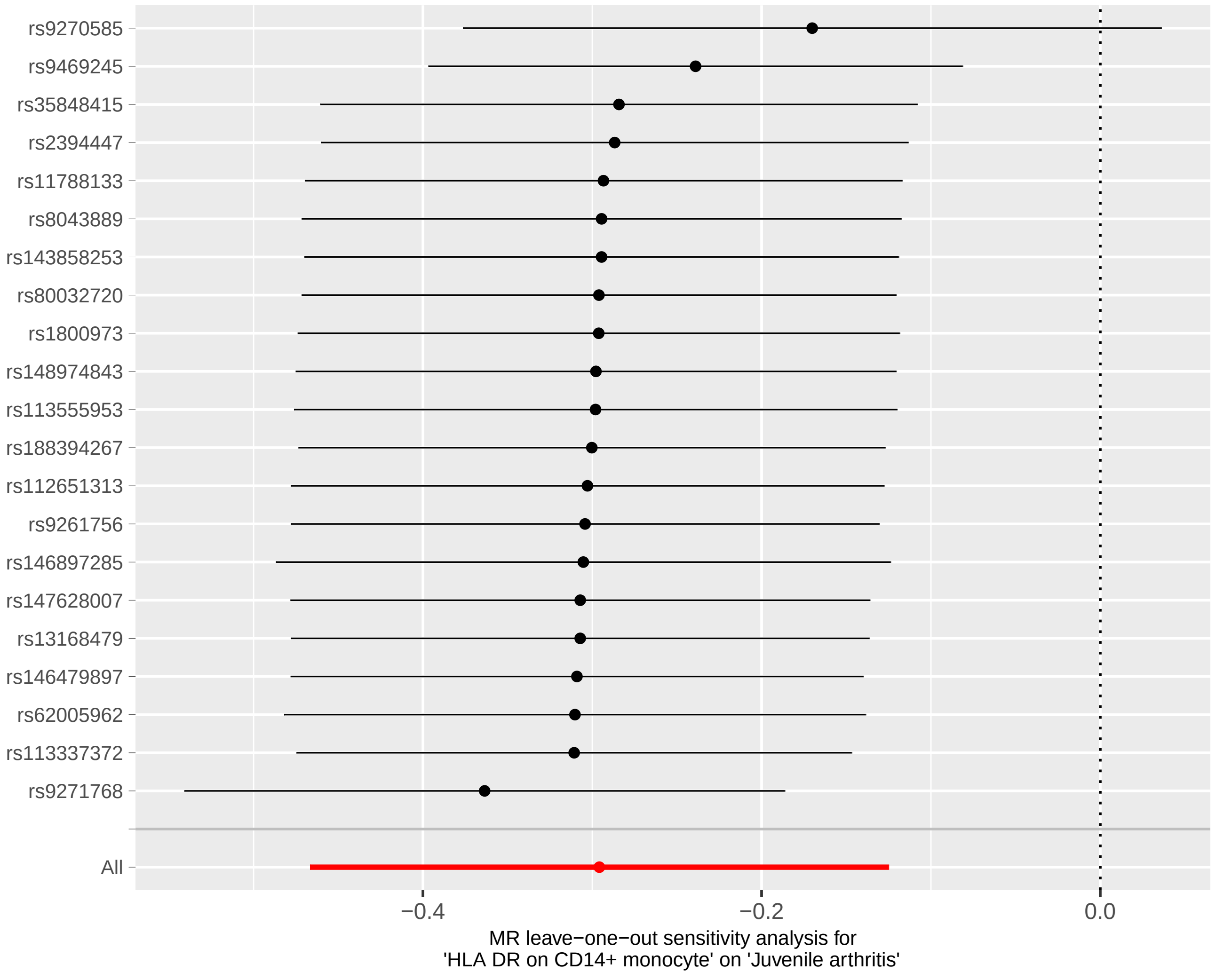

rs9270585
rs9469245
rs35848415
rs2394447
rs11788133
rs8043889
rs143858253
rs80032720
rs1800973
rs148974843
rs113555953
rs188394267
rs112651313
rs9261756
rs146897285
rs147628007
rs13168479
rs146479897
rs62005962
rs113337372
rs9271768
All
−0.4
−0.2
0.0
MR leave−one−out sensitivity analysis for
'HLA DR on CD14+ monocyte' on 'Juvenile arthritis'

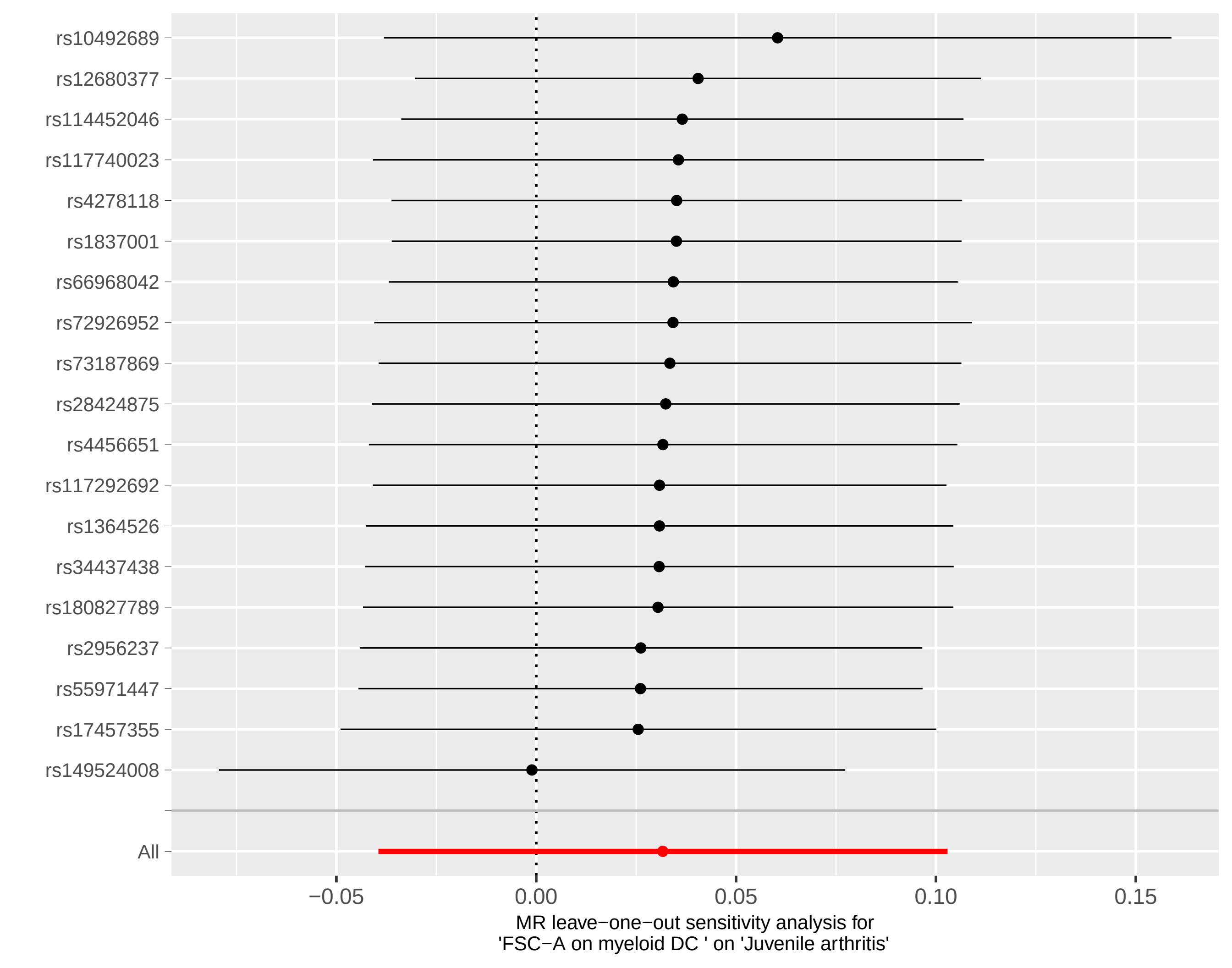
rs10492689
rs12680377
rs114452046
rs117740023
rs4278118
rs1837001
rs66968042
rs72926952
rs73187869
rs28424875
rs4456651
rs117292692
rs1364526
rs34437438
rs180827789
rs2956237
rs55971447
rs17457355
rs149524008
All
−0.05
0.00
0.05
0.10
0.15
MR leave−one−out sensitivity analysis for
'FSC−A on myeloid DC ' on 'Juvenile arthritis'

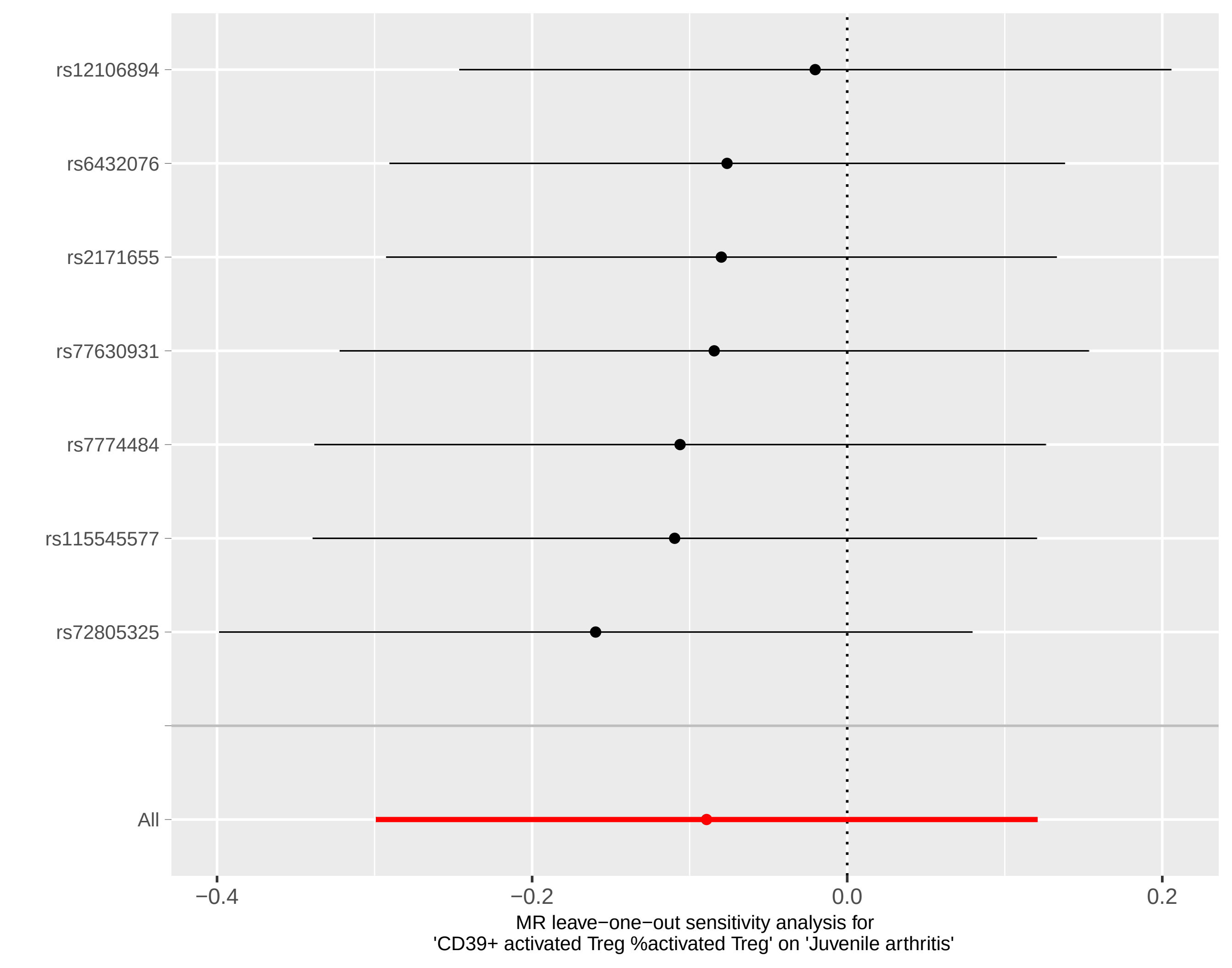
rs12106894
rs6432076
rs2171655
rs77630931
rs7774484
rs115545577
rs72805325
All
−0.4
−0.2
0.0
0.2
MR leave−one−out sensitivity analysis for
'CD39+ activated Treg %activated Treg' on 'Juvenile arthritis'

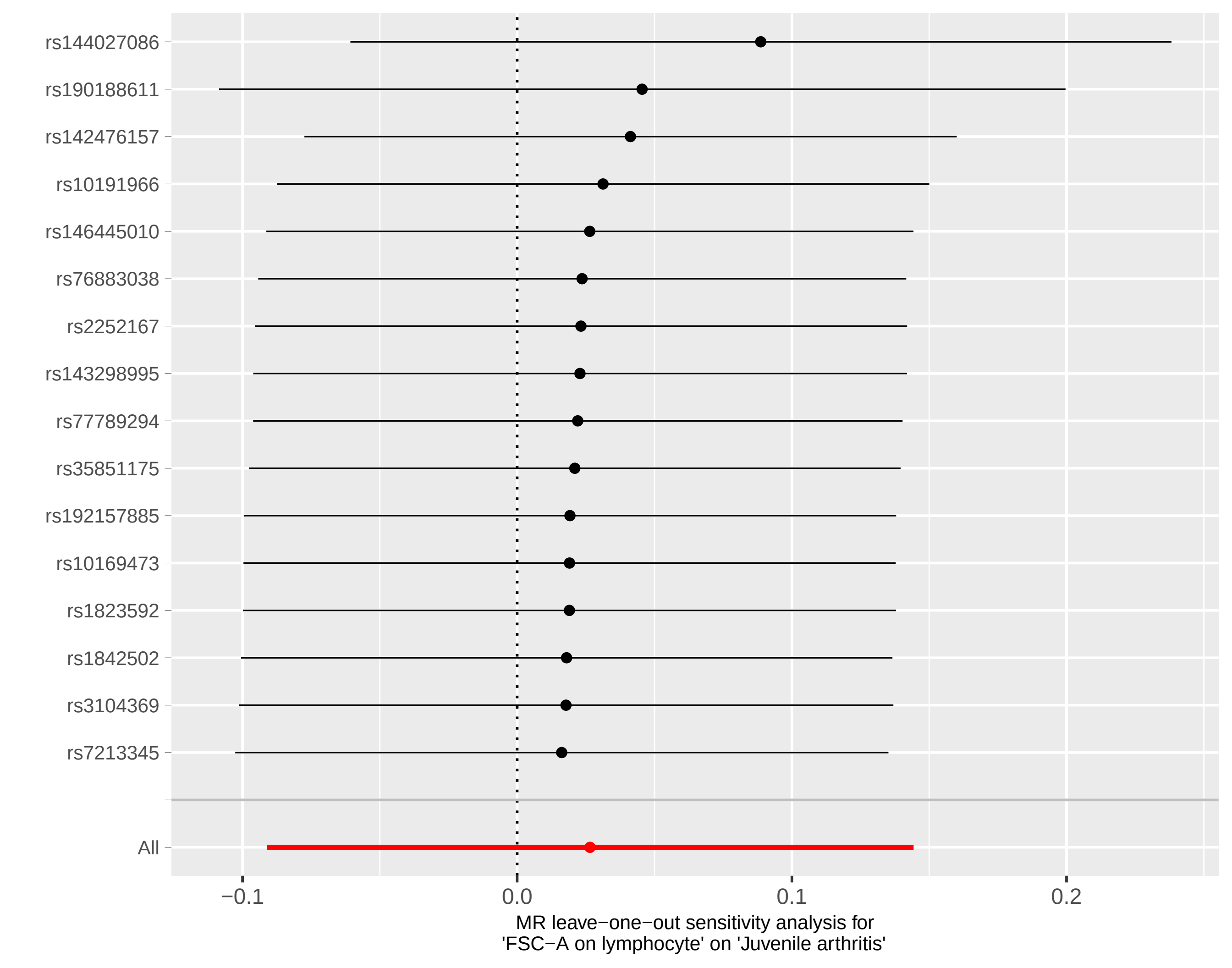

rs144027086
rs190188611
rs142476157
rs10191966
rs146445010
rs76883038
rs2252167
rs143298995
rs77789294
rs35851175
rs192157885
rs10169473
rs1823592
rs1842502
rs3104369
rs7213345
All
−0.1
0.0
0.1
0.2
MR leave−one−out sensitivity analysis for
'FSC−A on lymphocyte' on 'Juvenile arthritis'

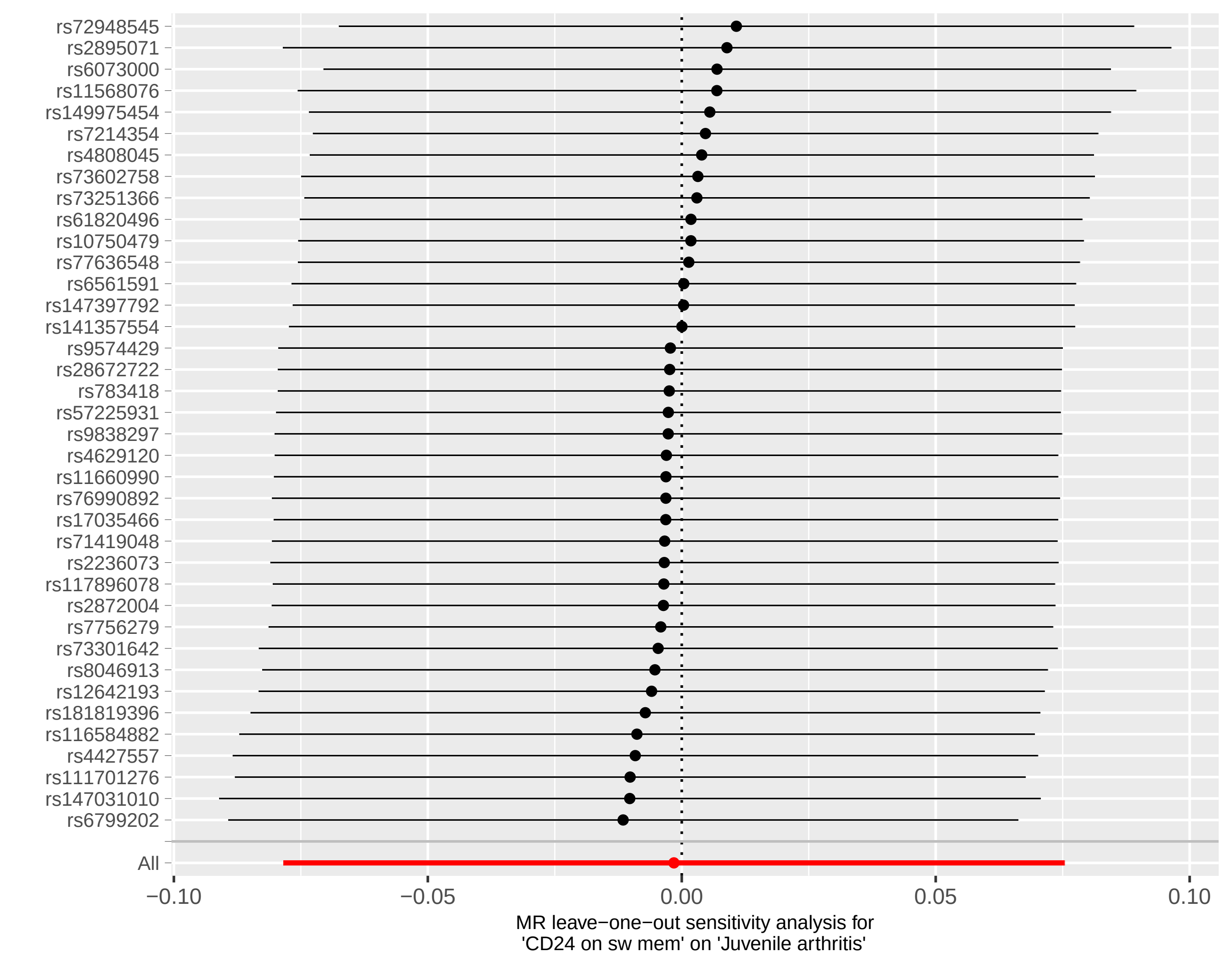
rs72948545
rs2895071
rs6073000
rs11568076
rs149975454
rs7214354
rs4808045
rs73602758
rs73251366
rs61820496
rs10750479
rs77636548
rs6561591
rs147397792
rs141357554
rs9574429
rs28672722
rs783418
rs57225931
rs9838297
rs4629120
rs11660990
rs76990892
rs17035466
rs71419048
rs2236073
rs117896078
rs2872004
rs7756279
rs73301642
rs8046913
rs12642193
rs181819396
rs116584882
rs4427557
rs111701276
rs147031010
rs6799202
All
−0.10
−0.05
0.00
0.05
0.10
MR leave−one−out sensitivity analysis for
'CD24 on sw mem' on 'Juvenile arthritis'

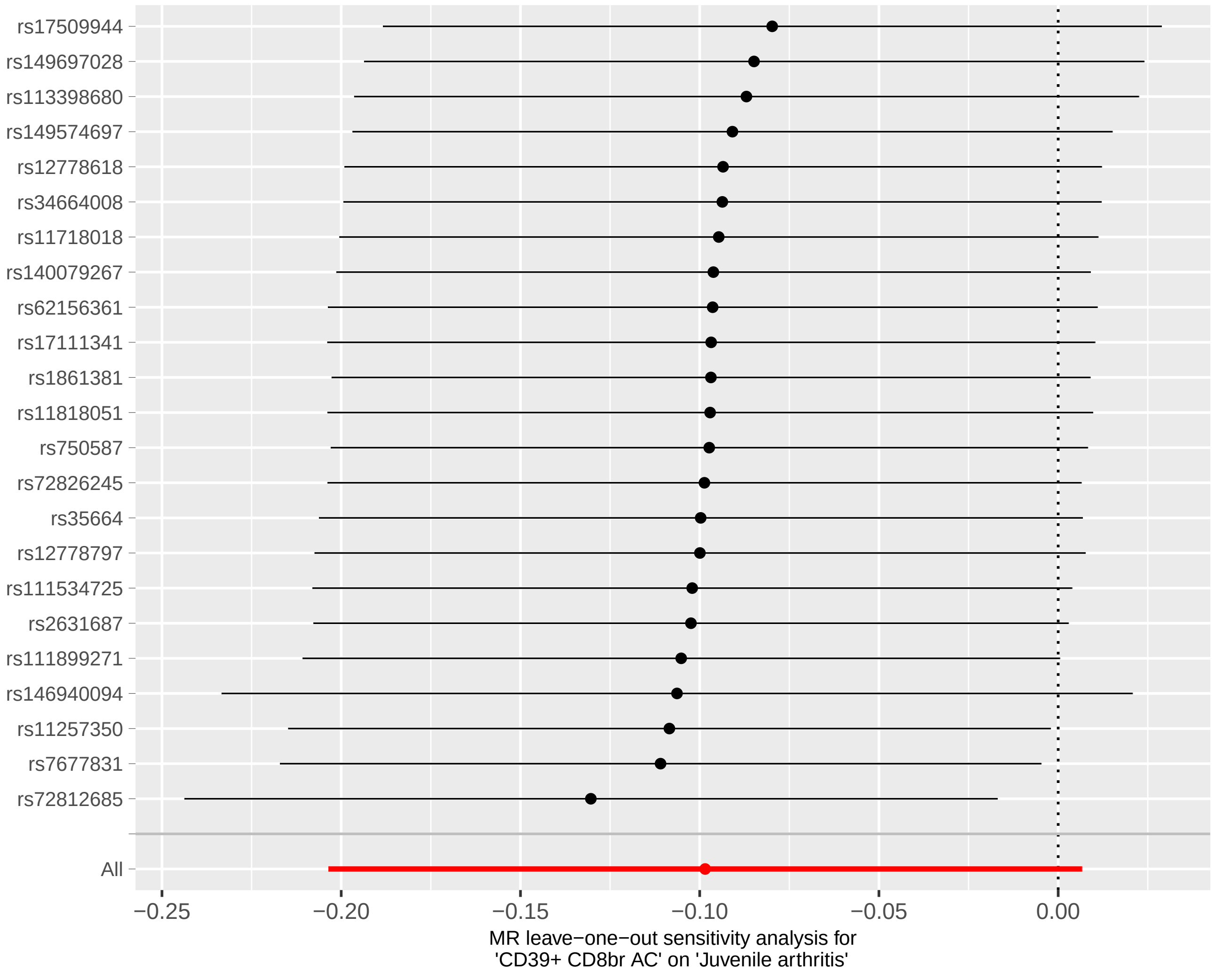
rs17509944
rs149697028
rs113398680
rs149574697
rs12778618
rs34664008
rs11718018
rs140079267
rs62156361
rs17111341
rs1861381
rs11818051
rs750587
rs72826245
rs35664
rs12778797
rs111534725
rs2631687
rs111899271
rs146940094
rs11257350
rs7677831
rs72812685
All
−0.25
−0.20
−0.15
−0.10
−0.05
0.00
MR leave−one−out sensitivity analysis for
'CD39+ CD8br AC' on 'Juvenile arthritis'

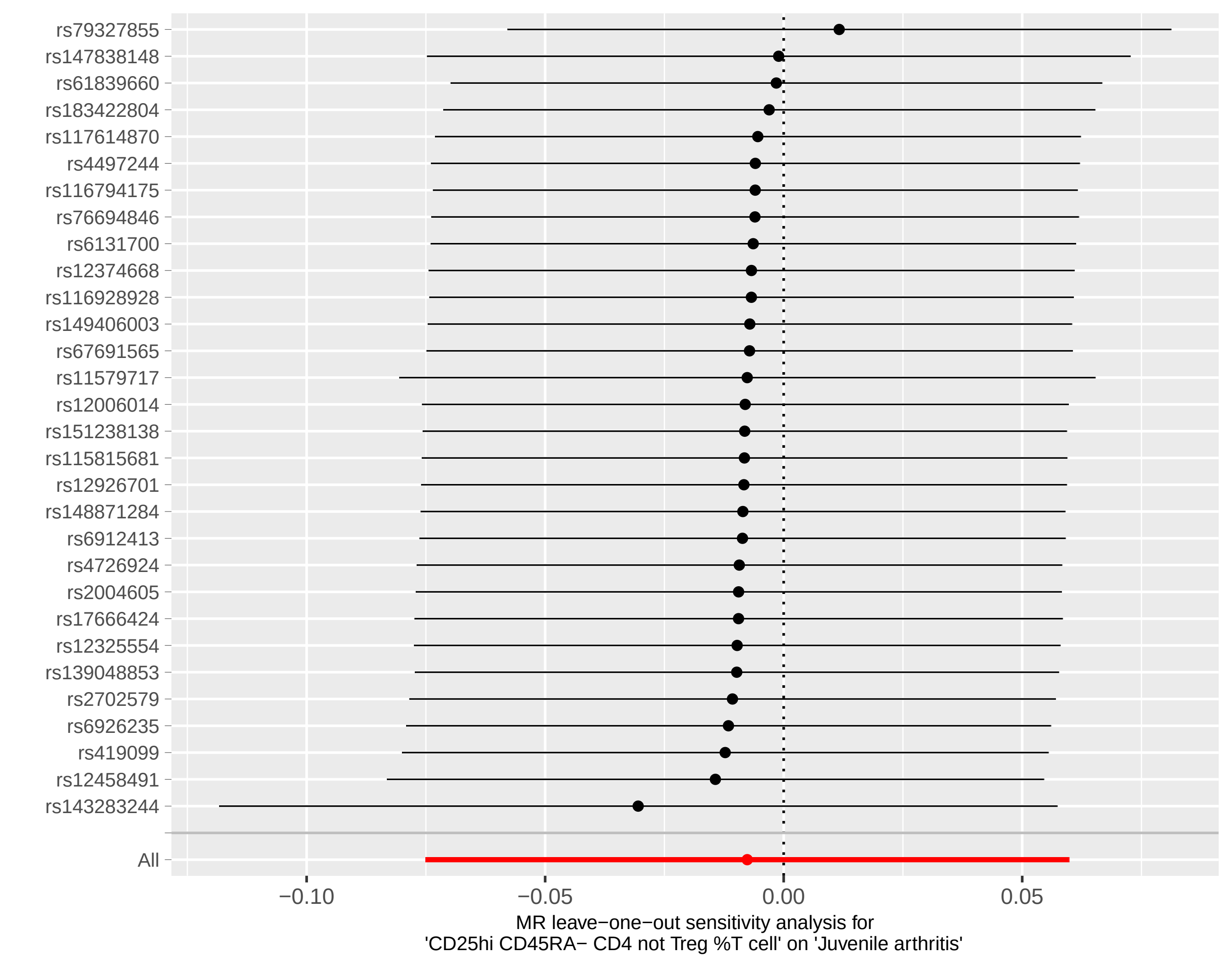
rs79327855
rs147838148
rs61839660
rs183422804
rs117614870
rs4497244
rs116794175
rs76694846
rs6131700
rs12374668
rs116928928
rs149406003
rs67691565
rs11579717
rs12006014
rs151238138
rs115815681
rs12926701
rs148871284
rs6912413
rs4726924
rs2004605
rs17666424
rs12325554
rs139048853
rs2702579
rs6926235
rs419099
rs12458491
rs143283244
All
−0.10
−0.05
0.00
0.05
MR leave−one−out sensitivity analysis for
'CD25hi CD45RA− CD4 not Treg %T cell' on 'Juvenile arthritis'

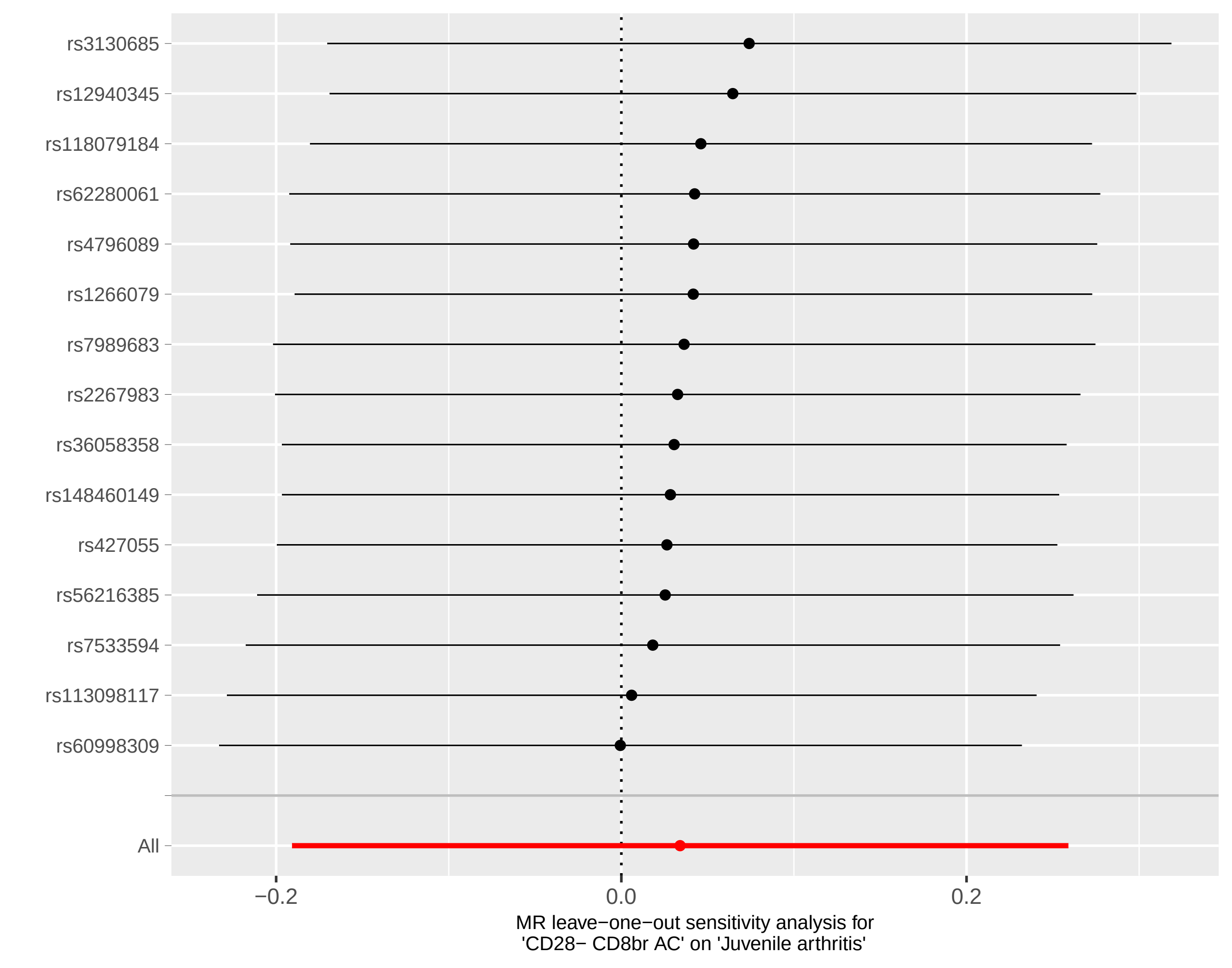

rs3130685
rs12940345
rs118079184
rs62280061
rs4796089
rs1266079
rs7989683
rs2267983
rs36058358
rs148460149
rs427055
rs56216385
rs7533594
rs113098117
rs60998309
All
−0.2
0.0
0.2
MR leave−one−out sensitivity analysis for
'CD28− CD8br AC' on 'Juvenile arthritis'

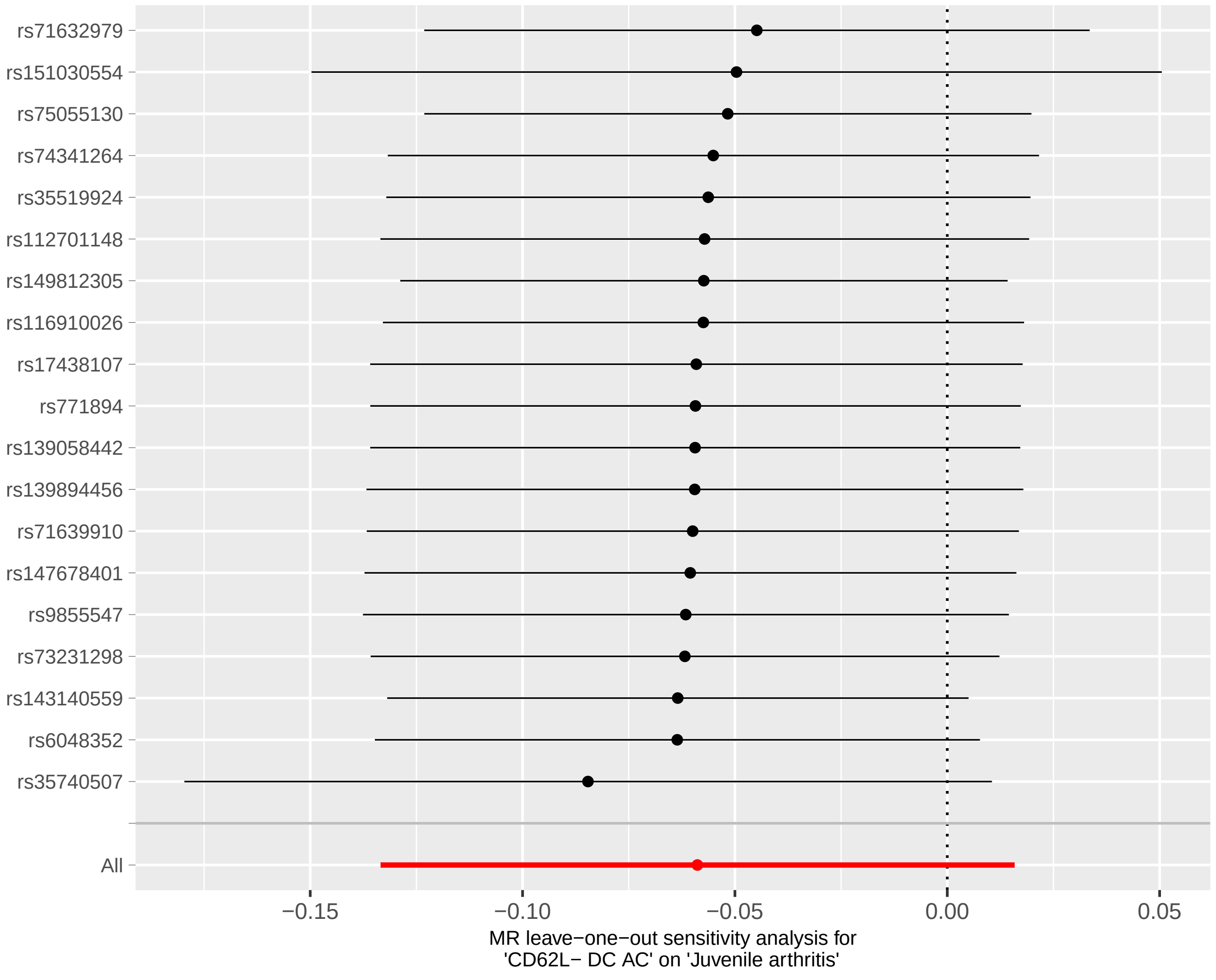
rs71632979
rs151030554
rs75055130
rs74341264
rs35519924
rs112701148
rs149812305
rs116910026
rs17438107
rs771894
rs139058442
rs139894456
rs71639910
rs147678401
rs9855547
rs73231298
rs143140559
rs6048352
rs35740507
All
−0.15
−0.10
−0.05
0.00
0.05
MR leave−one−out sensitivity analysis for
'CD62L− DC AC' on 'Juvenile arthritis'

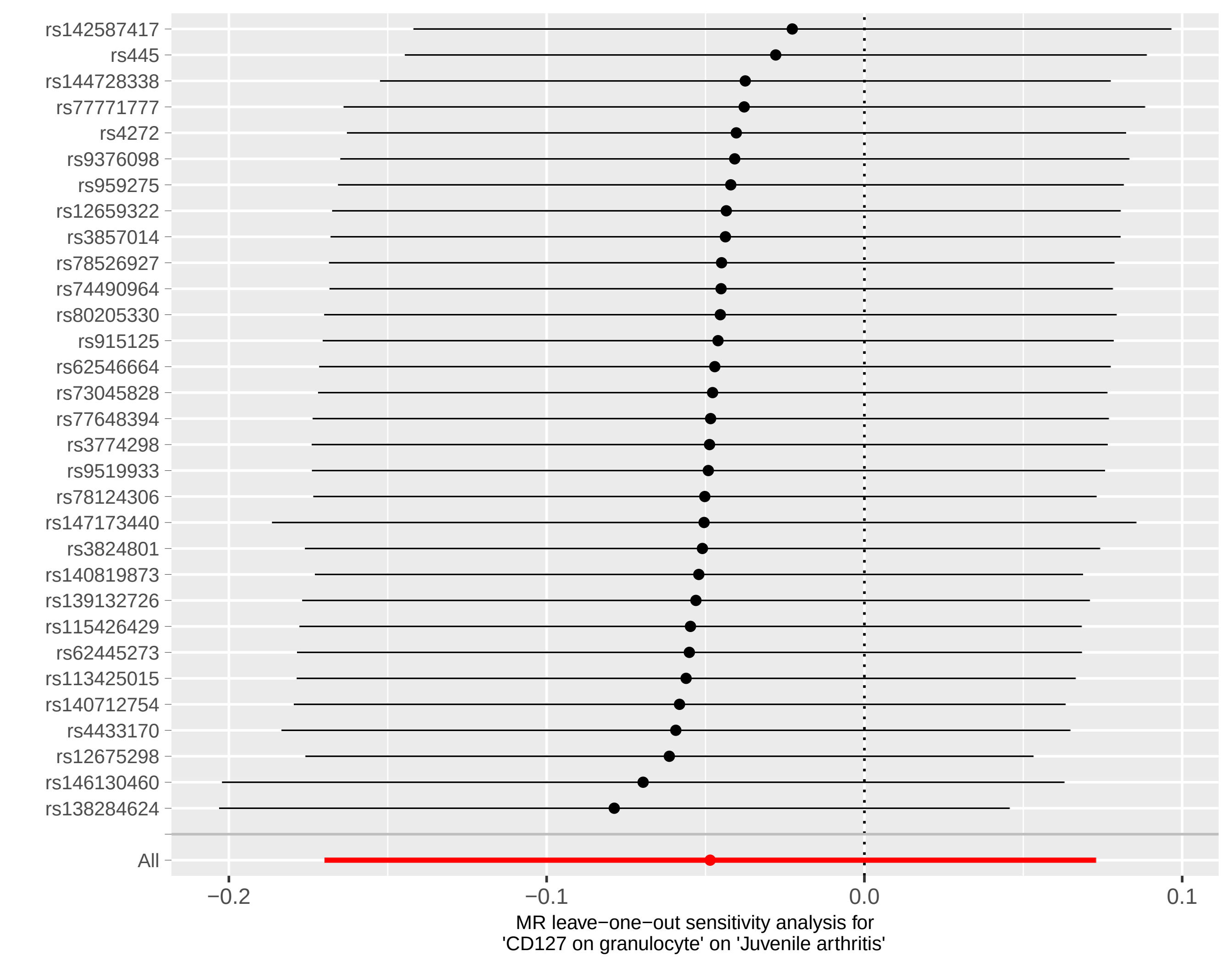

rs142587417
rs445
rs144728338
rs77771777
rs4272
rs9376098
rs959275
rs12659322
rs3857014
rs78526927
rs74490964
rs80205330
rs915125
rs62546664
rs73045828
rs77648394
rs3774298
rs9519933
rs78124306
rs147173440
rs3824801
rs140819873
rs139132726
rs115426429
rs62445273
rs113425015
rs140712754
rs4433170
rs12675298
rs146130460
rs138284624
All
−0.2
−0.1
0.0
0.1
MR leave−one−out sensitivity analysis for
'CD127 on granulocyte' on 'Juvenile arthritis'

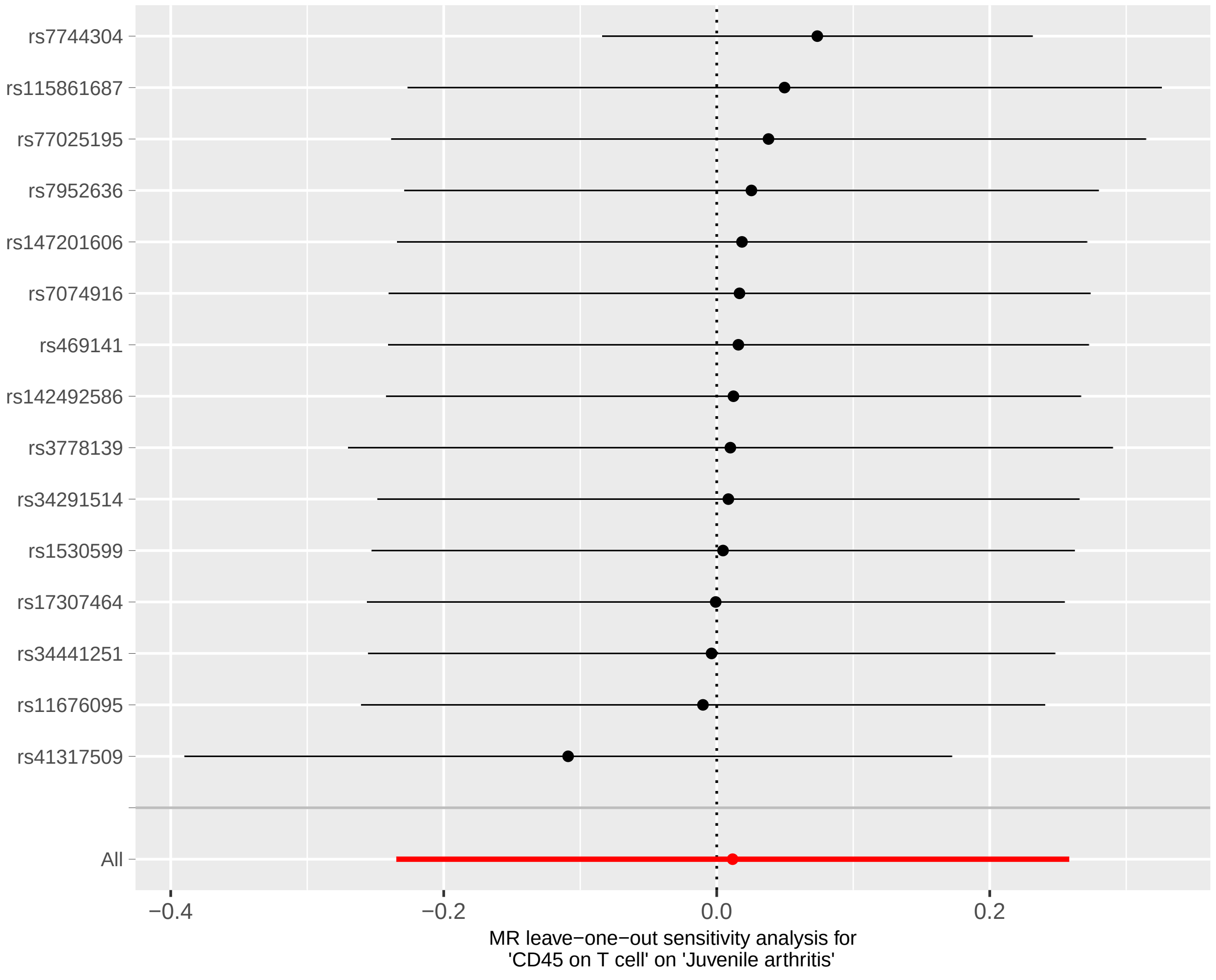
rs7744304
rs115861687
rs77025195
rs7952636
rs147201606
rs7074916
rs469141
rs142492586
rs3778139
rs34291514
rs1530599
rs17307464
rs34441251
rs11676095
rs41317509
All
−0.4
−0.2
0.0
0.2
MR leave−one−out sensitivity analysis for
'CD45 on T cell' on 'Juvenile arthritis'

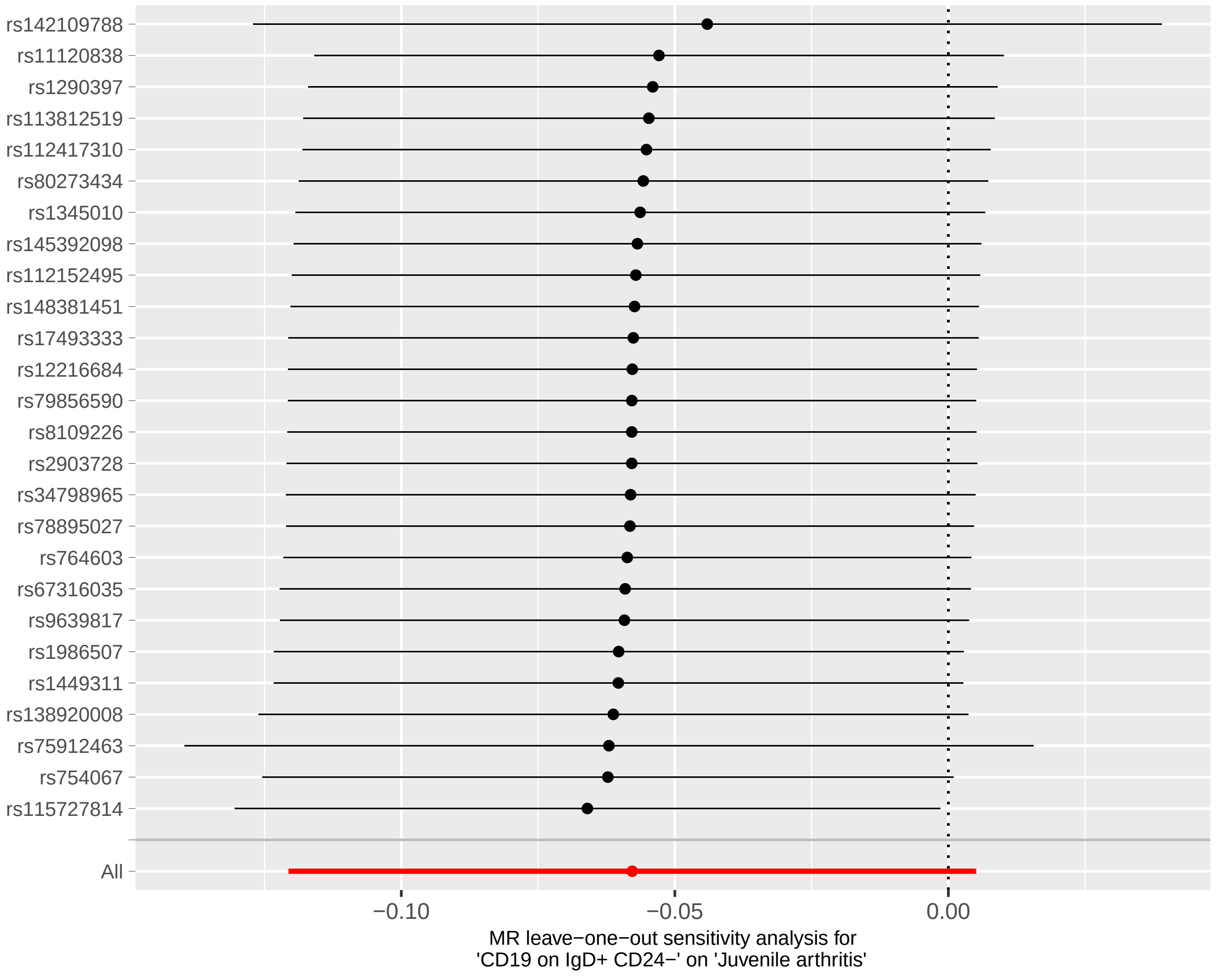

rs142109788
rs11120838
rs1290397
rs113812519
rs112417310
rs80273434
rs1345010
rs145392098
rs112152495
rs148381451
rs17493333
rs12216684
rs79856590
rs8109226
rs2903728
rs34798965
rs78895027
rs764603
rs67316035
rs9639817
rs1986507
rs1449311
rs138920008
rs75912463
rs754067
rs115727814
All
−0.10
−0.05
0.00
MR leave−one−out sensitivity analysis for
'CD19 on IgD+ CD24−' on 'Juvenile arthritis'

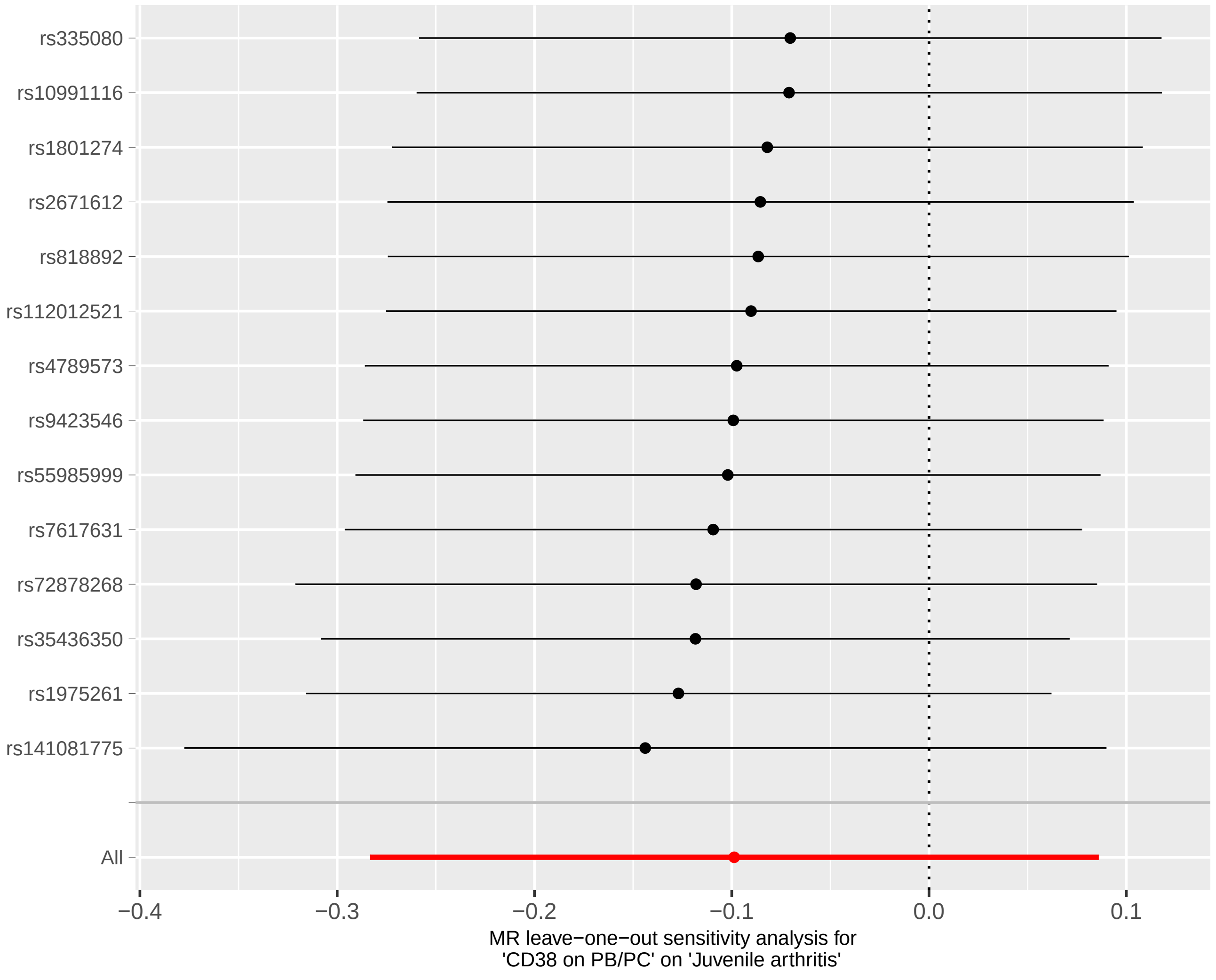

rs335080
rs10991116
rs1801274
rs2671612
rs818892
rs112012521
rs4789573
rs9423546
rs55985999
rs7617631
rs72878268
rs35436350
rs1975261
rs141081775
All
−0.4
−0.3
−0.2
−0.1
0.0
0.1
MR leave−one−out sensitivity analysis for
'CD38 on PB/PC' on 'Juvenile arthritis'

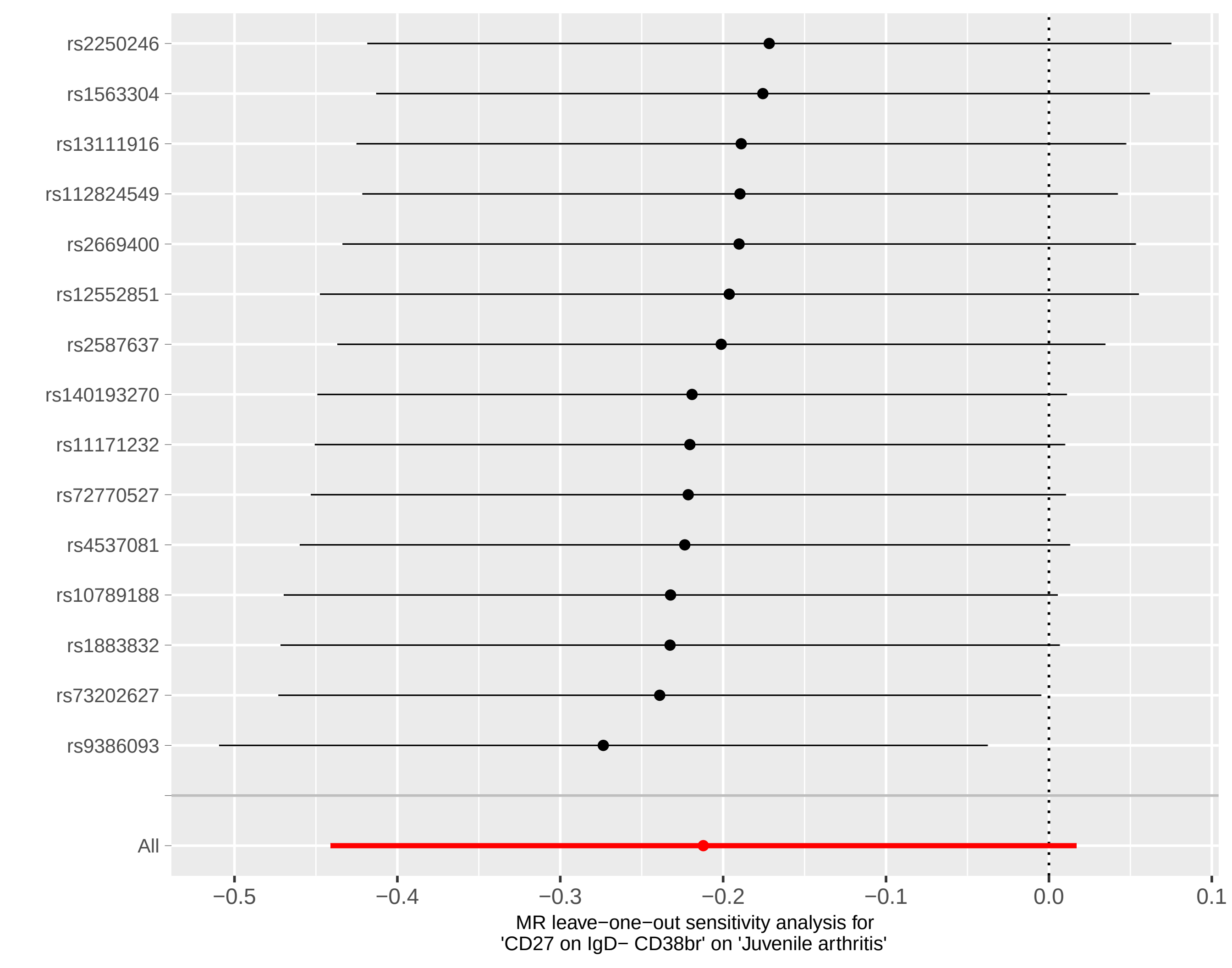
rs2250246
rs1563304
rs13111916
rs112824549
rs2669400
rs12552851
rs2587637
rs140193270
rs11171232
rs72770527
rs4537081
rs10789188
rs1883832
rs73202627
rs9386093
All
−0.5
−0.4
−0.3
−0.2
−0.1
0.0
0.1
MR leave−one−out sensitivity analysis for
'CD27 on IgD− CD38br' on 'Juvenile arthritis'

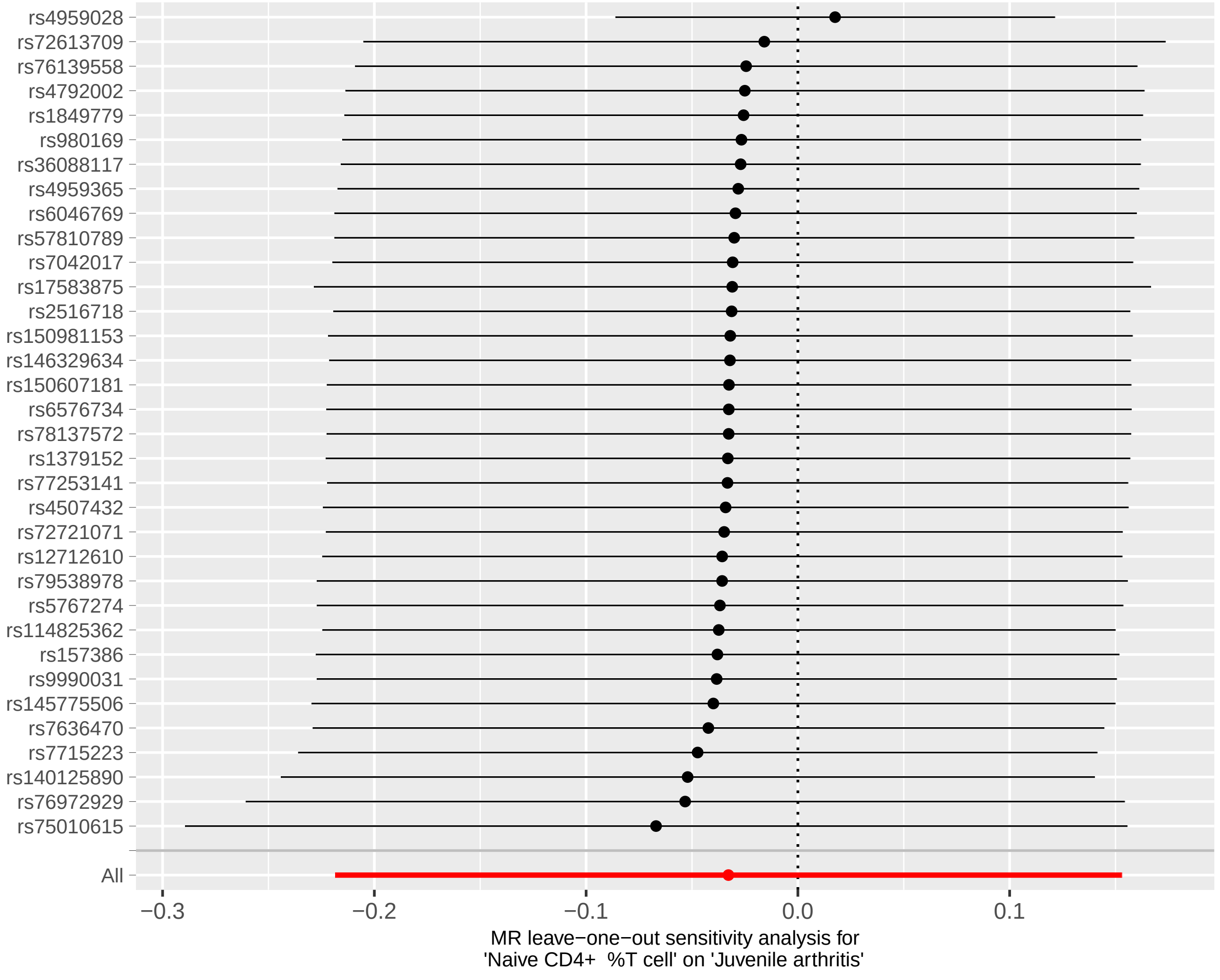
rs4959028
rs72613709
rs76139558
rs4792002
rs1849779
rs980169
rs36088117
rs4959365
rs6046769
rs57810789
rs7042017
rs17583875
rs2516718
rs150981153
rs146329634
rs150607181
rs6576734
rs78137572
rs1379152
rs77253141
rs4507432
rs72721071
rs12712610
rs79538978
rs5767274
rs114825362
rs157386
rs9990031
rs145775506
rs7636470
rs7715223
rs140125890
rs76972929
rs75010615
All
−0.3
−0.2
−0.1
0.0
0.1
MR leave−one−out sensitivity analysis for
'Naive CD4+ %T cell' on 'Juvenile arthritis'

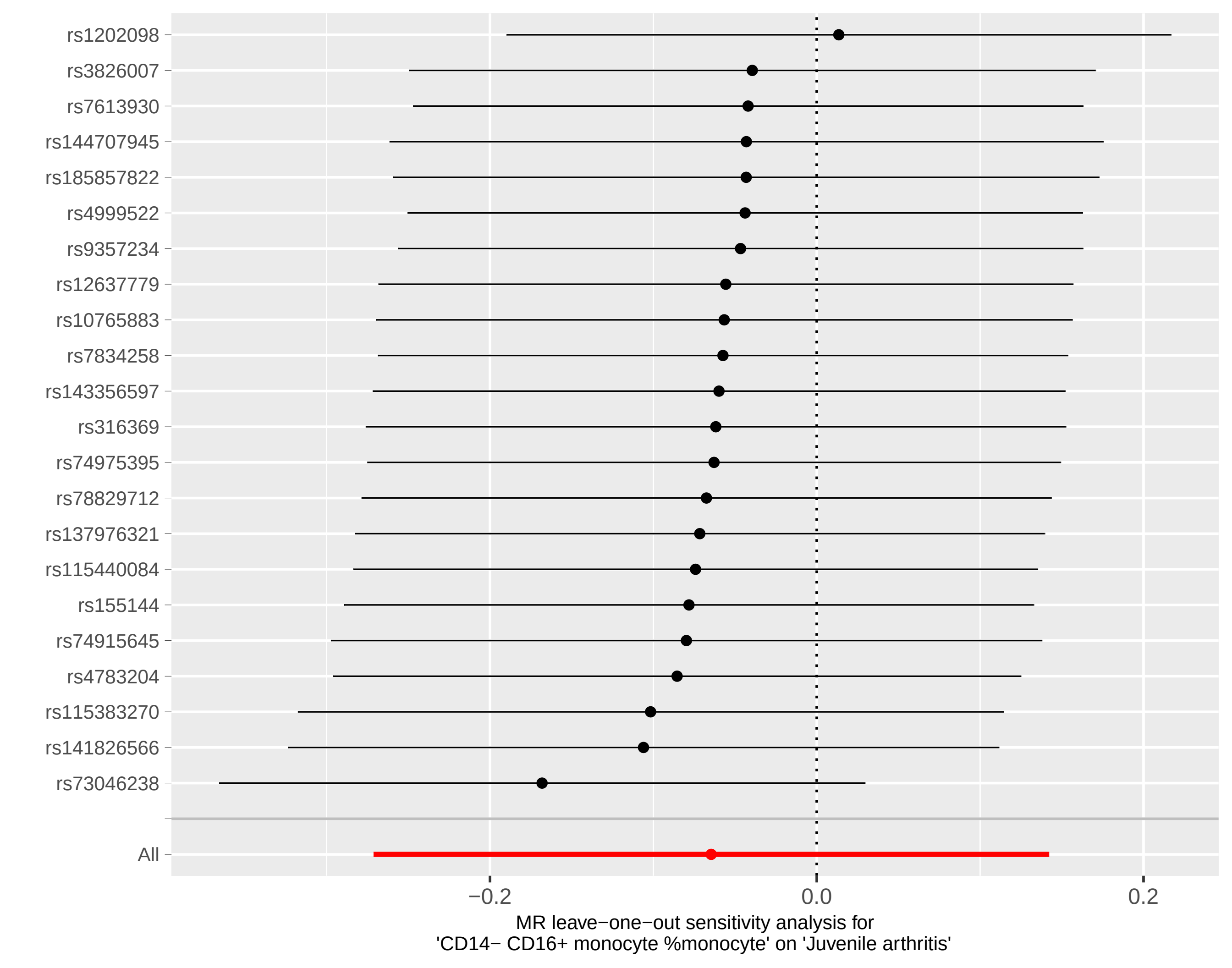

rs1202098
rs3826007
rs7613930
rs144707945
rs185857822
rs4999522
rs9357234
rs12637779
rs10765883
rs7834258
rs143356597
rs316369
rs74975395
rs78829712
rs137976321
rs115440084
rs155144
rs74915645
rs4783204
rs115383270
rs141826566
rs73046238
All
−0.2
0.0
0.2
MR leave−one−out sensitivity analysis for
'CD14− CD16+ monocyte %monocyte' on 'Juvenile arthritis'

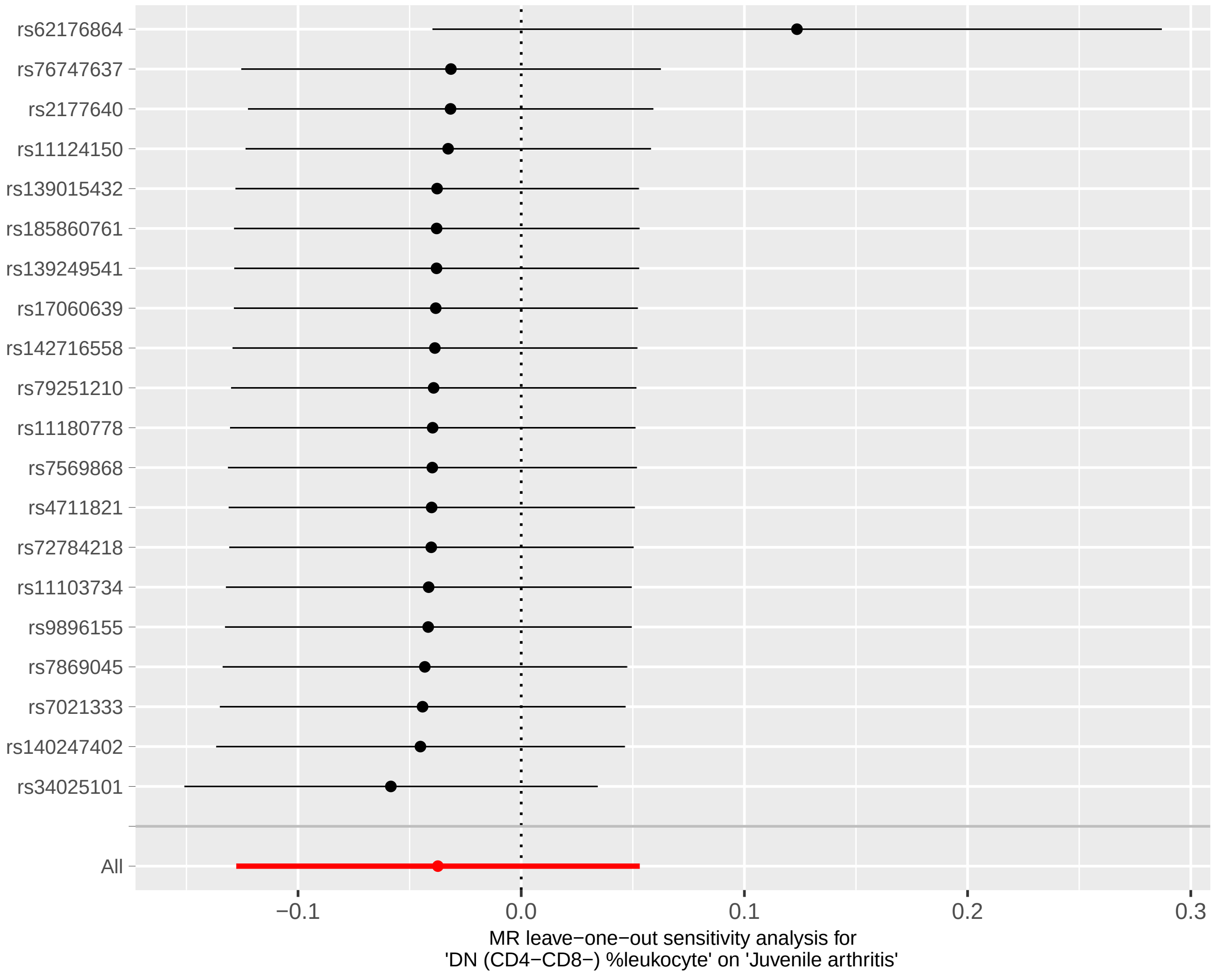
rs62176864
rs76747637
rs2177640
rs11124150
rs139015432
rs185860761
rs139249541
rs17060639
rs142716558
rs79251210
rs11180778
rs7569868
rs4711821
rs72784218
rs11103734
rs9896155
rs7869045
rs7021333
rs140247402
rs34025101
All
−0.1
0.0
0.1
0.2
0.3
MR leave−one−out sensitivity analysis for
'DN (CD4−CD8−) %leukocyte' on 'Juvenile arthritis'

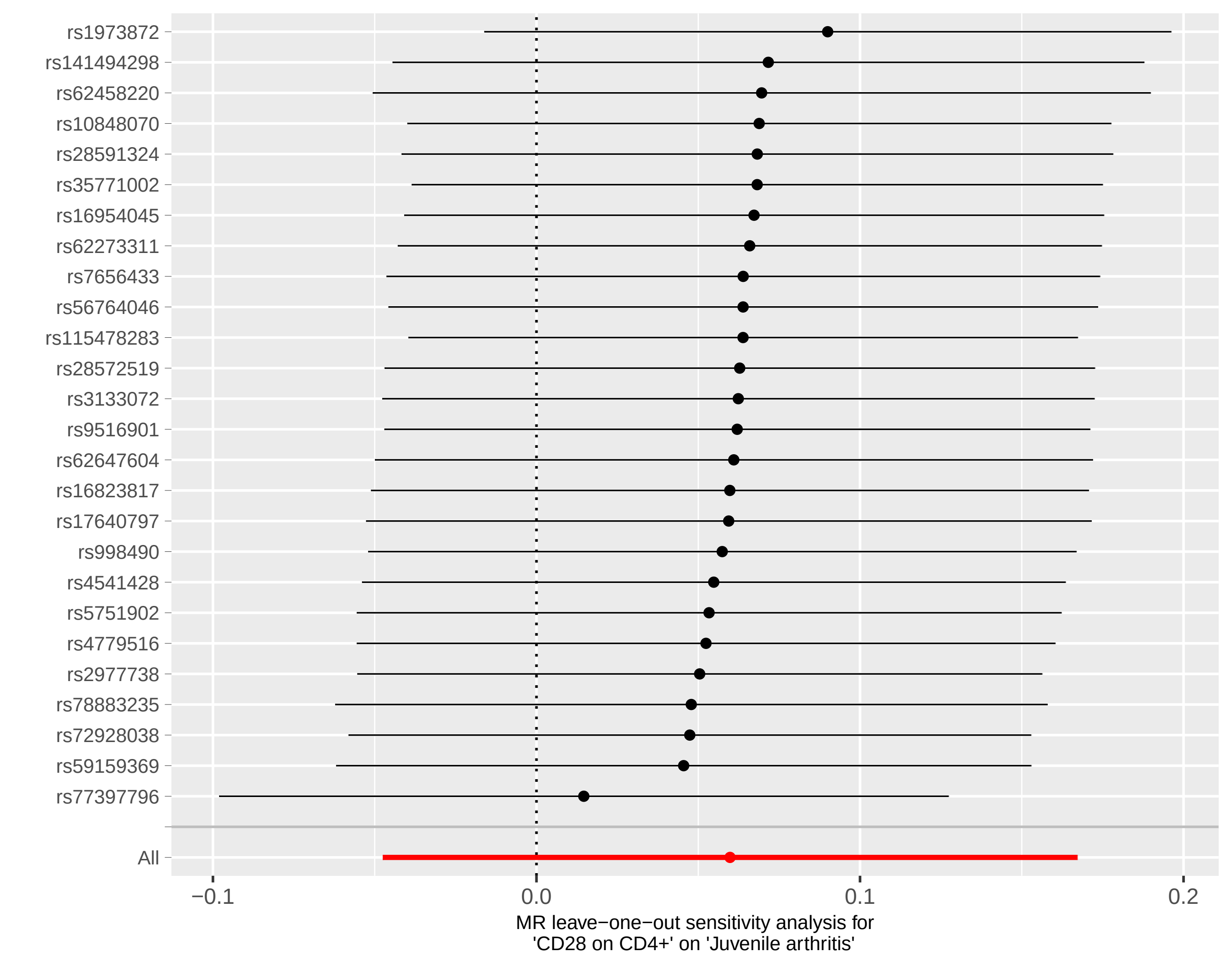
rs1973872
rs141494298
rs62458220
rs10848070
rs28591324
rs35771002
rs16954045
rs62273311
rs7656433
rs56764046
rs115478283
rs28572519
rs3133072
rs9516901
rs62647604
rs16823817
rs17640797
rs998490
rs4541428
rs5751902
rs4779516
rs2977738
rs78883235
rs72928038
rs59159369
rs77397796
All
−0.1
0.0
0.1
0.2
MR leave−one−out sensitivity analysis for
'CD28 on CD4+' on 'Juvenile arthritis'

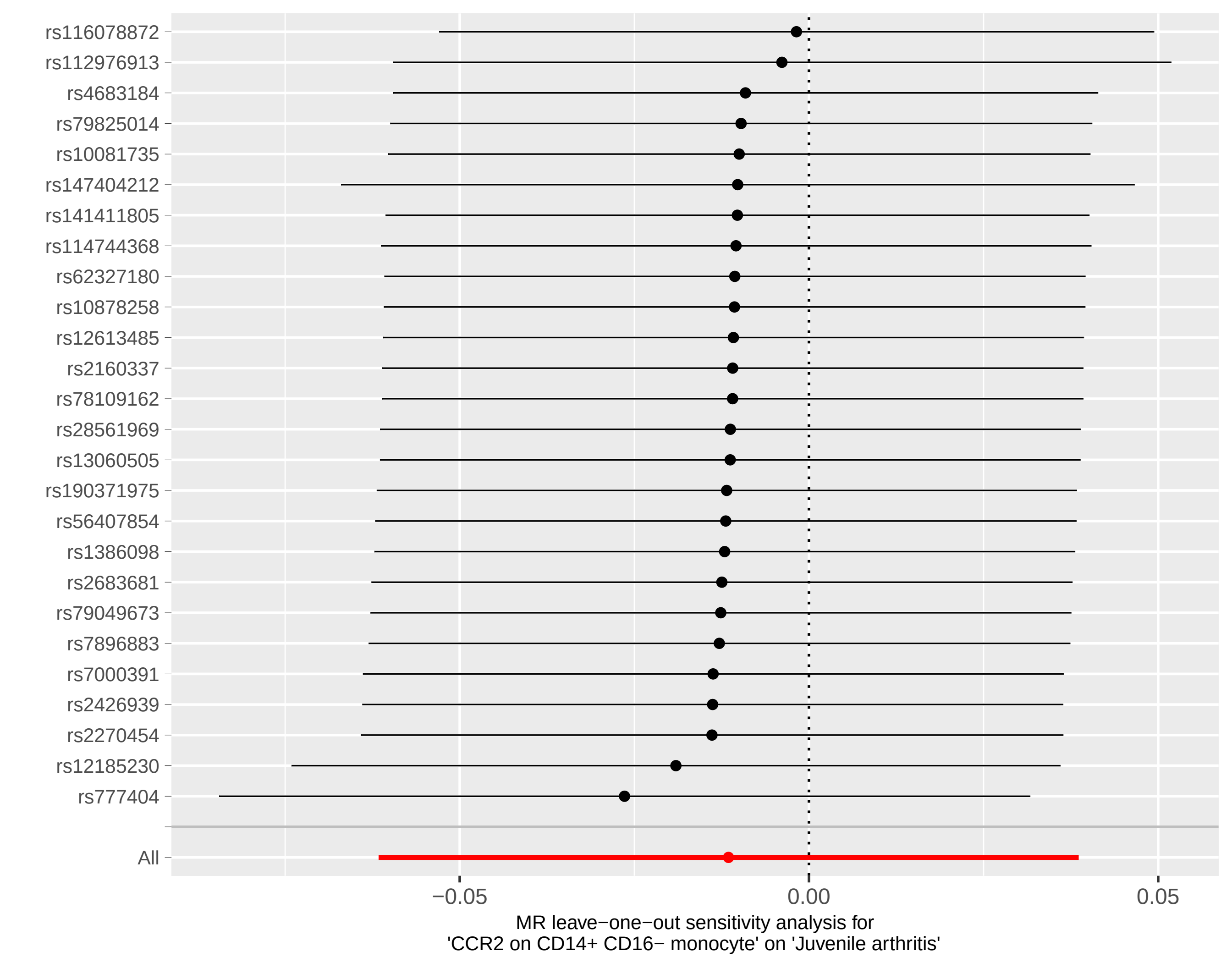
rs116078872
rs112976913
rs4683184
rs79825014
rs10081735
rs147404212
rs141411805
rs114744368
rs62327180
rs10878258
rs12613485
rs2160337
rs78109162
rs28561969
rs13060505
rs190371975
rs56407854
rs1386098
rs2683681
rs79049673
rs7896883
rs7000391
rs2426939
rs2270454
rs12185230
rs777404
All
−0.05
0.00
0.05
MR leave−one−out sensitivity analysis for
'CCR2 on CD14+ CD16− monocyte' on 'Juvenile arthritis'

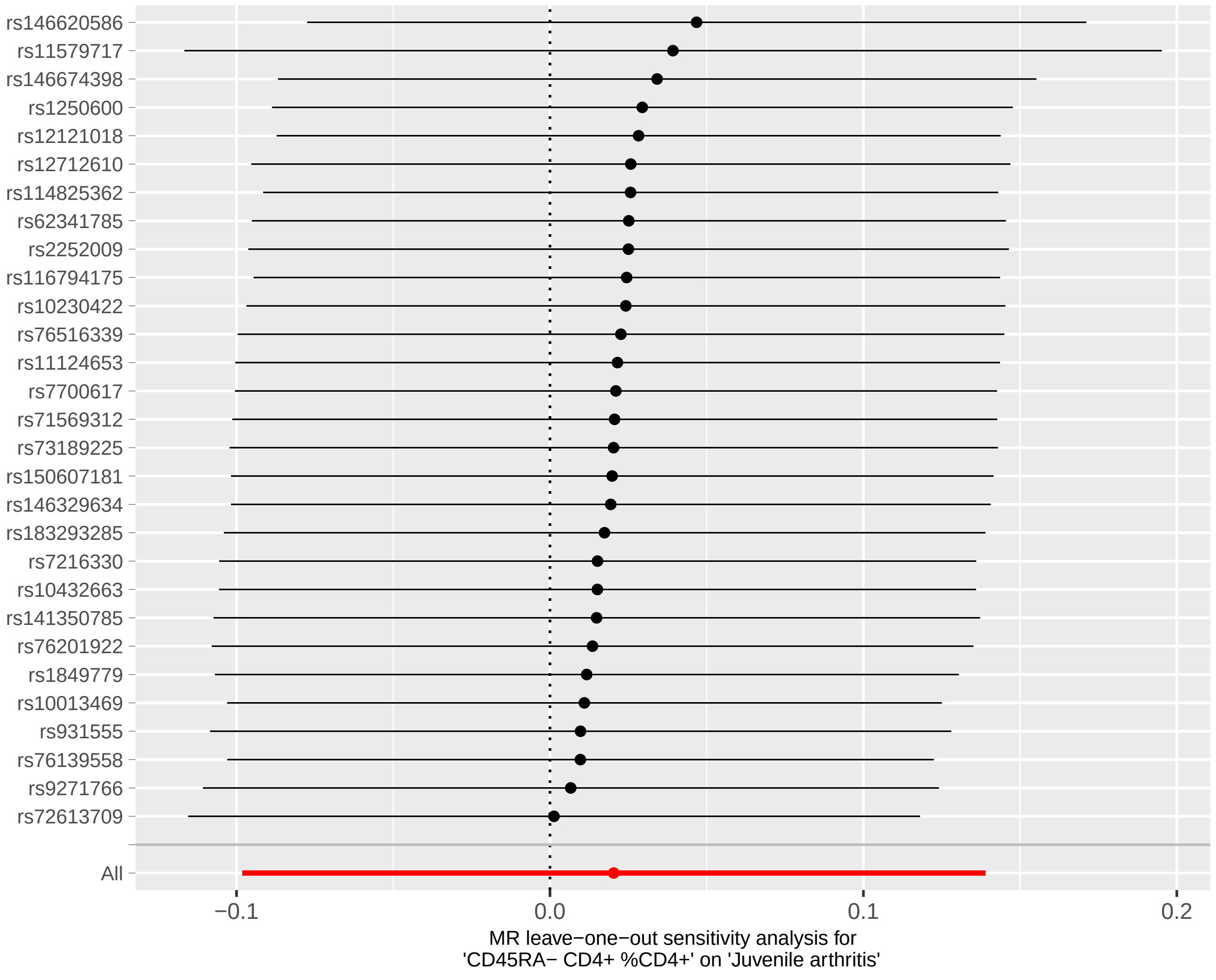

rs146620586
rs11579717
rs146674398
rs1250600
rs12121018
rs12712610
rs114825362
rs62341785
rs2252009
rs116794175
rs10230422
rs76516339
rs11124653
rs7700617
rs71569312
rs73189225
rs150607181
rs146329634
rs183293285
rs7216330
rs10432663
rs141350785
rs76201922
rs1849779
rs10013469
rs931555
rs76139558
rs9271766
rs72613709
All
−0.1
0.0
0.1
0.2
MR leave−one−out sensitivity analysis for
'CD45RA− CD4+ %CD4+' on 'Juvenile arthritis'

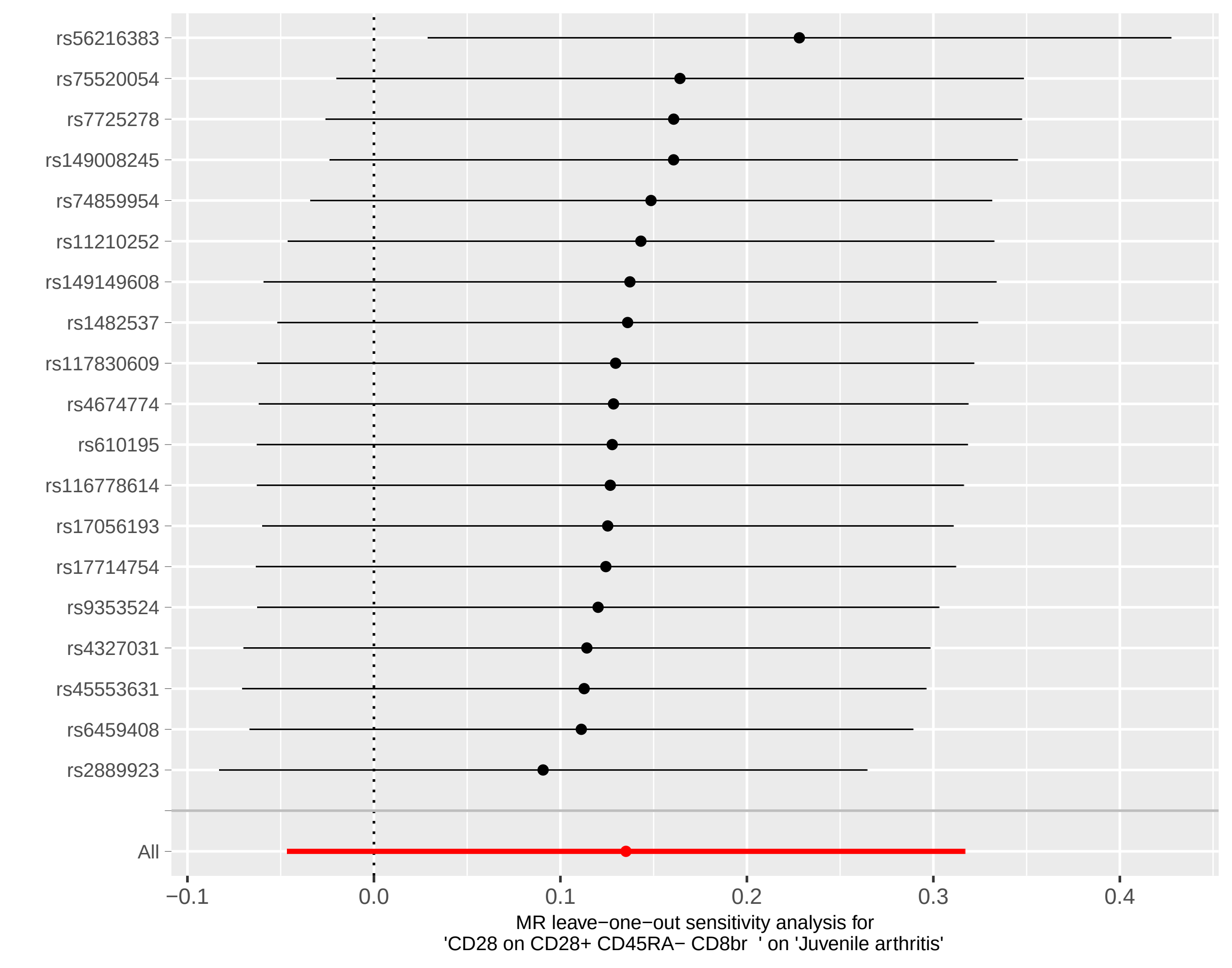
rs56216383
rs75520054
rs7725278
rs149008245
rs74859954
rs11210252
rs149149608
rs1482537
rs117830609
rs4674774
rs610195
rs116778614
rs17056193
rs17714754
rs9353524
rs4327031
rs45553631
rs6459408
rs2889923
All
−0.1
0.0
0.1
0.2
0.3
0.4
MR leave−one−out sensitivity analysis for
'CD28 on CD28+ CD45RA− CD8br ' on 'Juvenile arthritis'

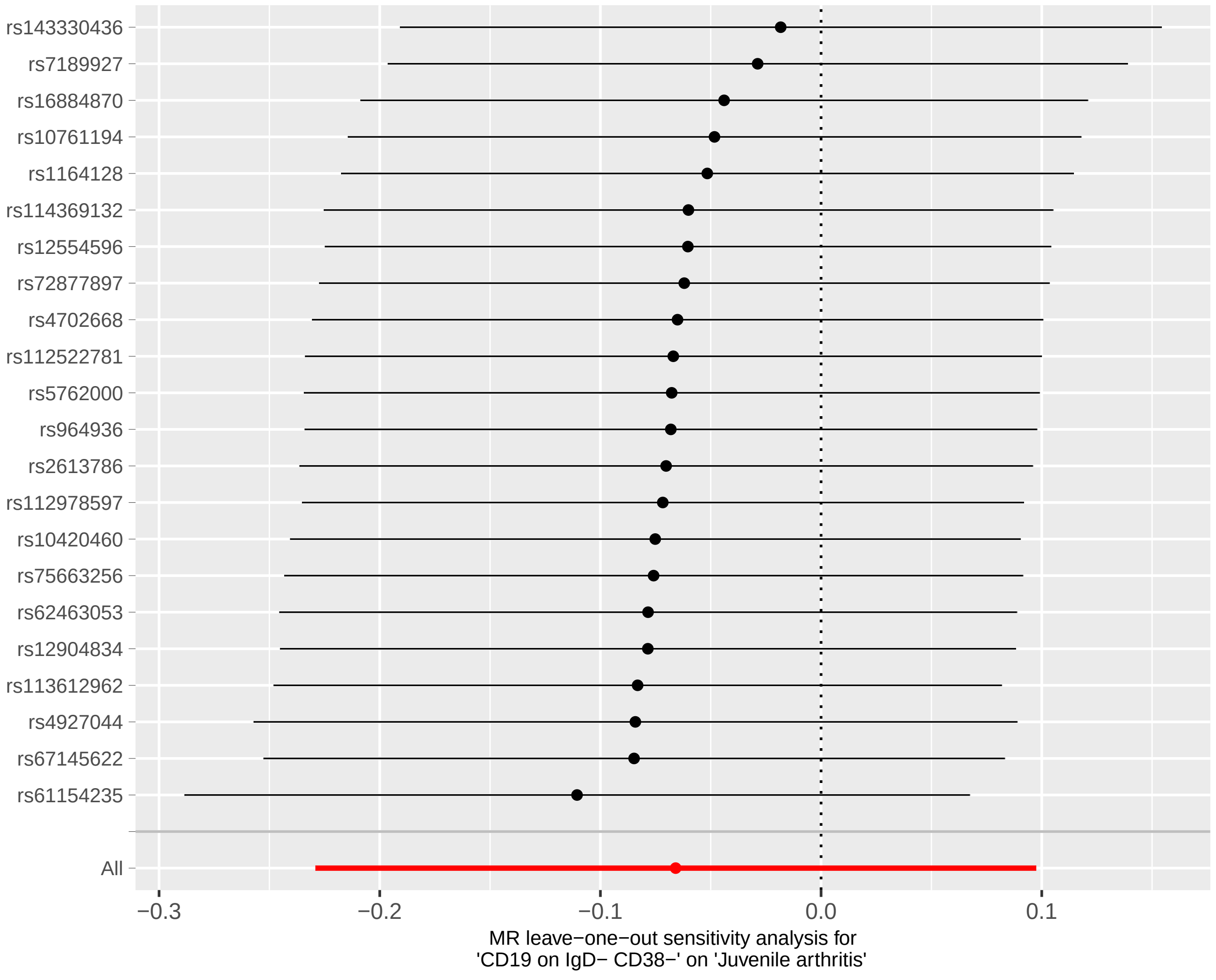
rs143330436
rs7189927
rs16884870
rs10761194
rs1164128
rs114369132
rs12554596
rs72877897
rs4702668
rs112522781
rs5762000
rs964936
rs2613786
rs112978597
rs10420460
rs75663256
rs62463053
rs12904834
rs113612962
rs4927044
rs67145622
rs61154235
All
−0.3
−0.2
−0.1
0.0
0.1
MR leave−one−out sensitivity analysis for
'CD19 on IgD− CD38−' on 'Juvenile arthritis'

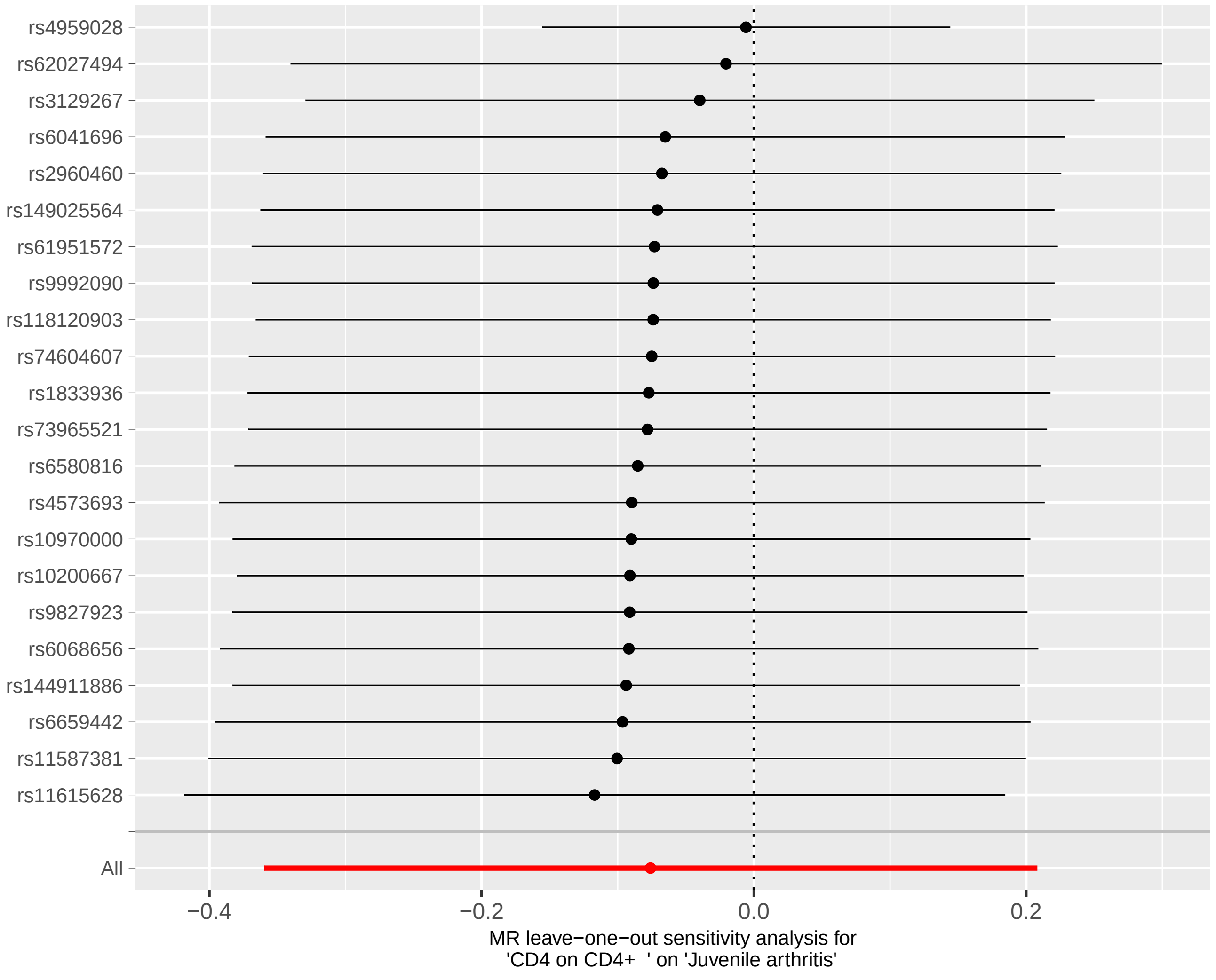
rs4959028
rs62027494
rs3129267
rs6041696
rs2960460
rs149025564
rs61951572
rs9992090
rs118120903
rs74604607
rs1833936
rs73965521
rs6580816
rs4573693
rs10970000
rs10200667
rs9827923
rs6068656
rs144911886
rs6659442
rs11587381
rs11615628
All
−0.4
−0.2
0.0
0.2
MR leave−one−out sensitivity analysis for
'CD4 on CD4+ ' on 'Juvenile arthritis'

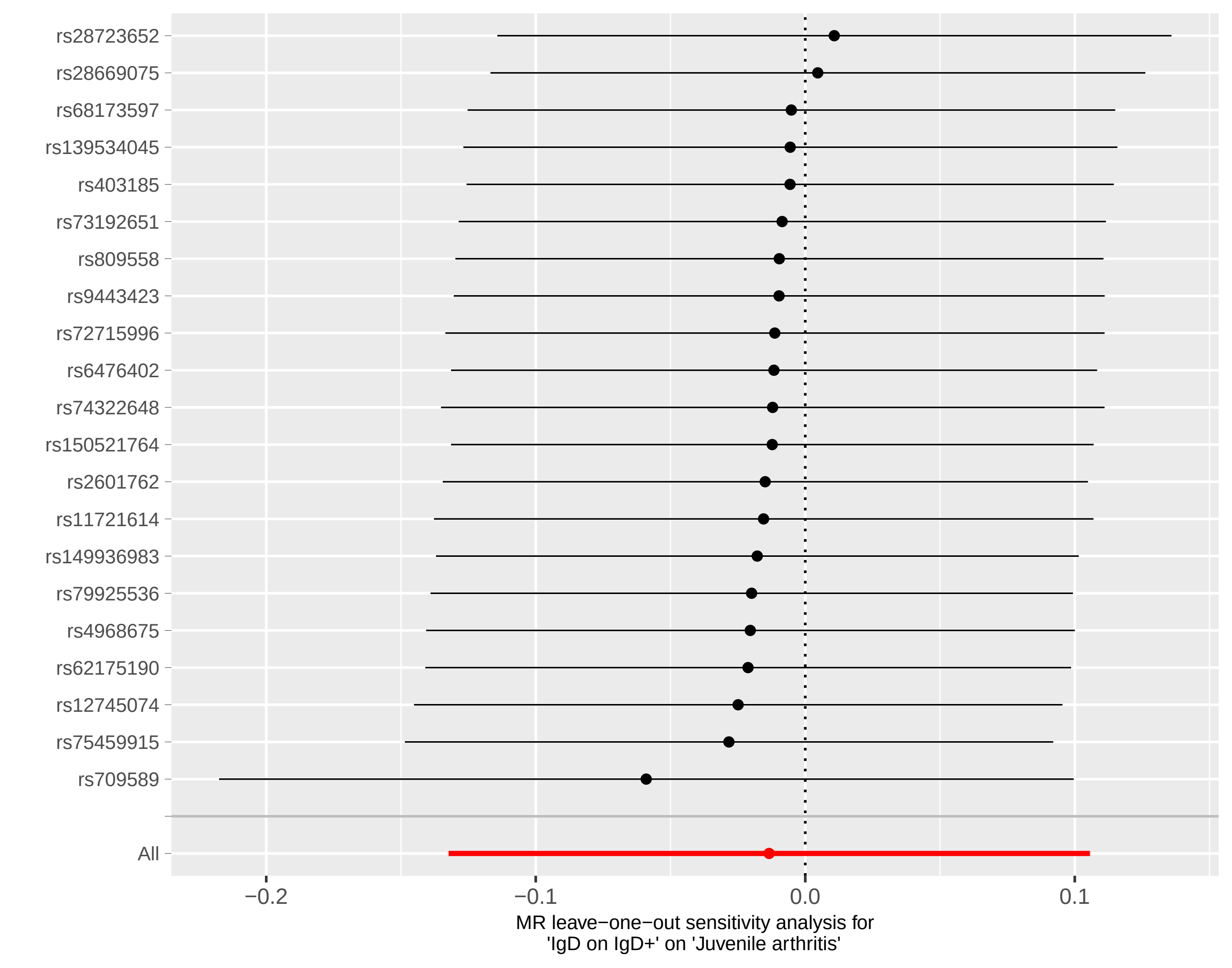

rs28723652
rs28669075
rs68173597
rs139534045
rs403185
rs73192651
rs809558
rs9443423
rs72715996
rs6476402
rs74322648
rs150521764
rs2601762
rs11721614
rs149936983
rs79925536
rs4968675
rs62175190
rs12745074
rs75459915
rs709589
All
−0.2
−0.1
0.0
0.1
MR leave−one−out sensitivity analysis for
'IgD on IgD+' on 'Juvenile arthritis'

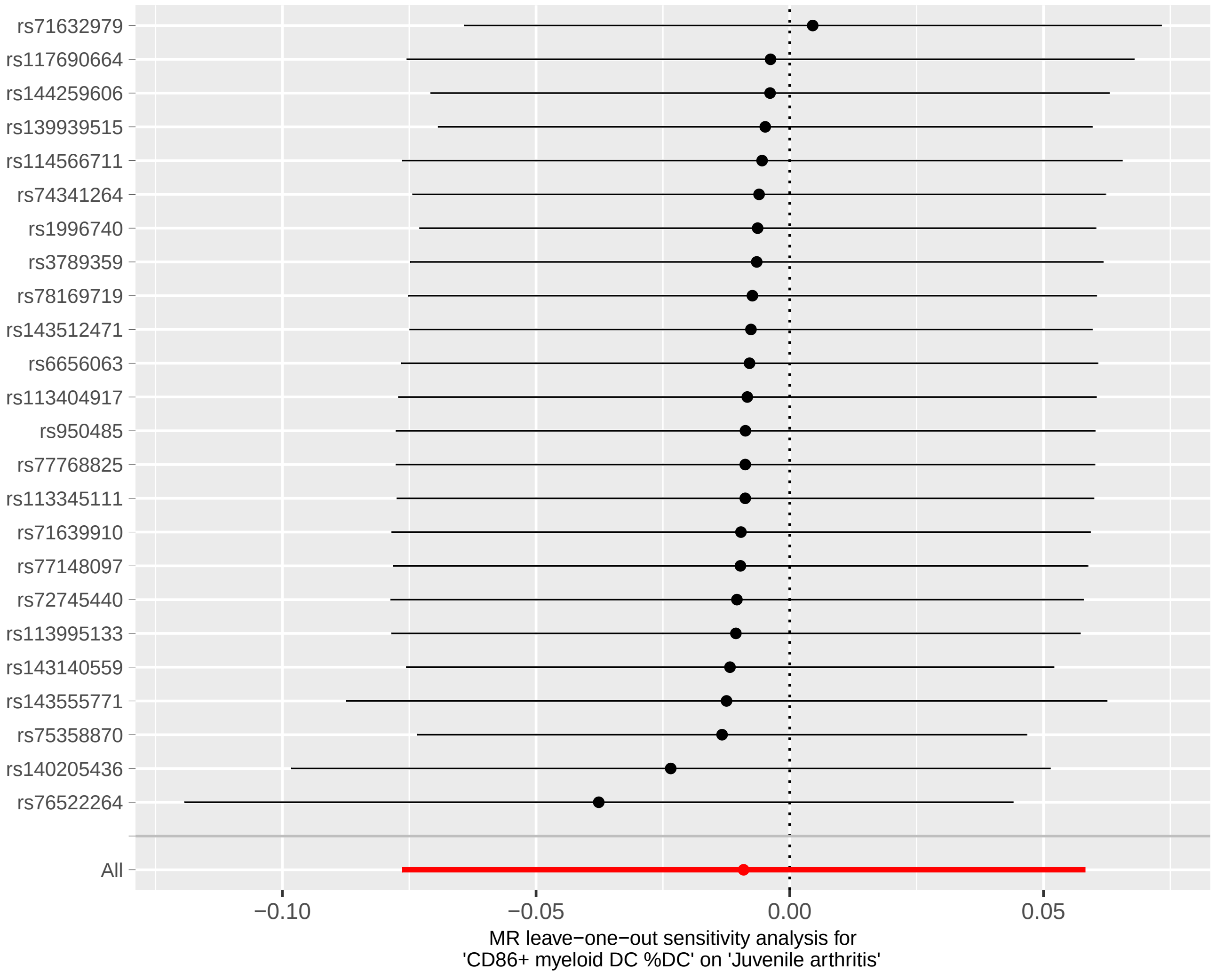
rs71632979
rs117690664
rs144259606
rs139939515
rs114566711
rs74341264
rs1996740
rs3789359
rs78169719
rs143512471
rs6656063
rs113404917
rs950485
rs77768825
rs113345111
rs71639910
rs77148097
rs72745440
rs113995133
rs143140559
rs143555771
rs75358870
rs140205436
rs76522264
All
−0.10
−0.05
0.00
0.05
MR leave−one−out sensitivity analysis for
'CD86+ myeloid DC %DC' on 'Juvenile arthritis'

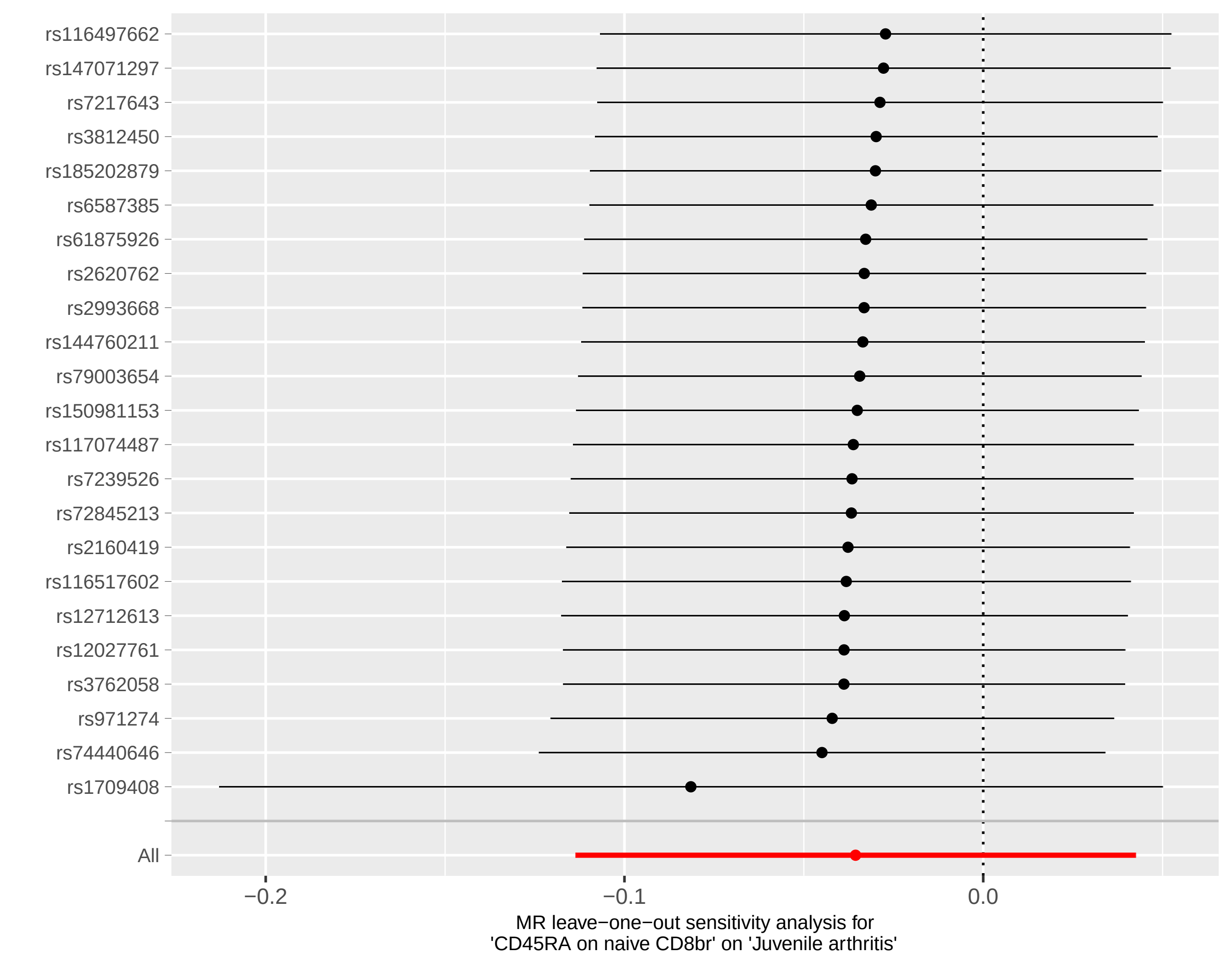
rs116497662
rs147071297
rs7217643
rs3812450
rs185202879
rs6587385
rs61875926
rs2620762
rs2993668
rs144760211
rs79003654
rs150981153
rs117074487
rs7239526
rs72845213
rs2160419
rs116517602
rs12712613
rs12027761
rs3762058
rs971274
rs74440646
rs1709408
All
−0.2
−0.1
0.0
MR leave−one−out sensitivity analysis for
'CD45RA on naive CD8br' on 'Juvenile arthritis'

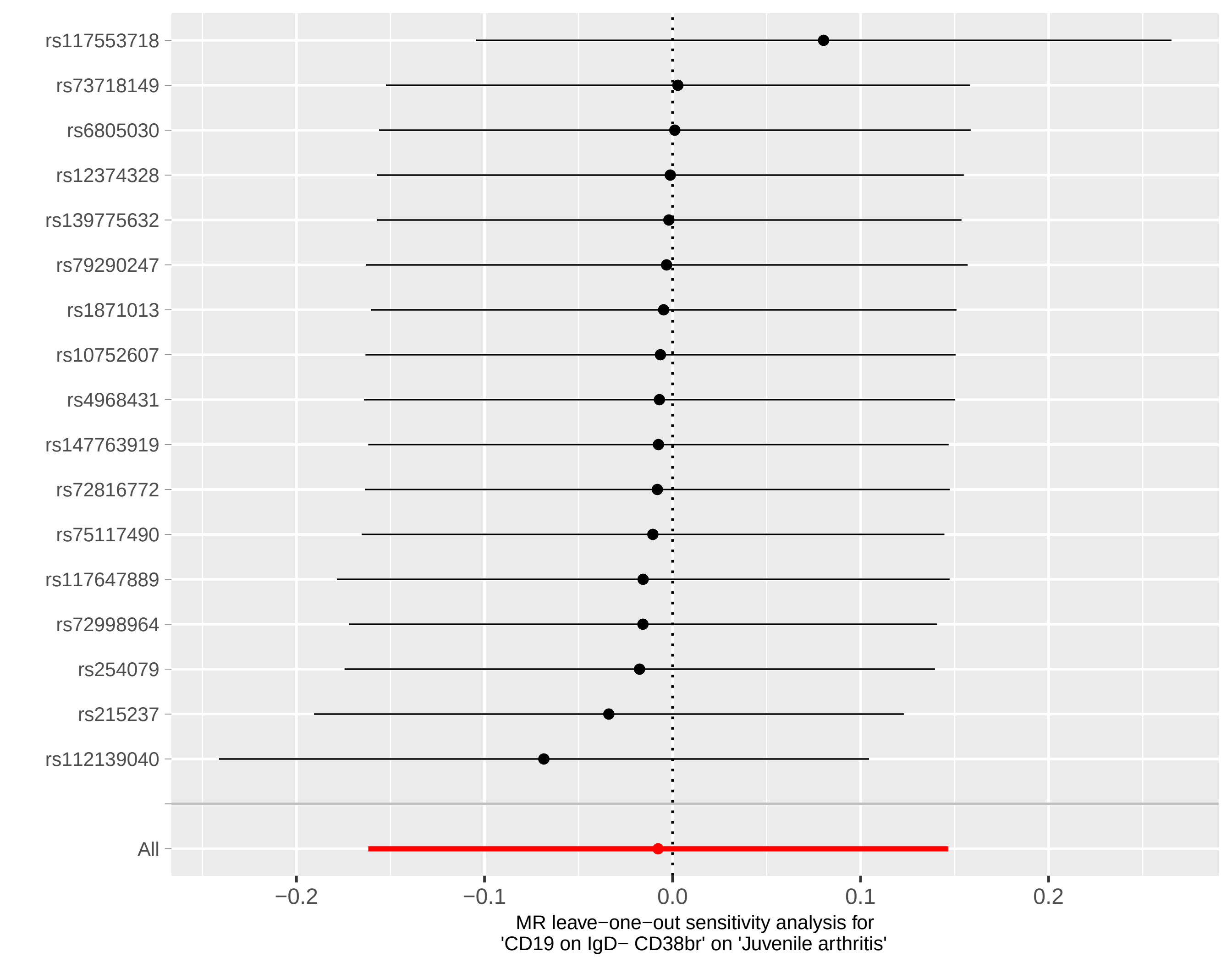

rs117553718
rs73718149
rs6805030
rs12374328
rs139775632
rs79290247
rs1871013
rs10752607
rs4968431
rs147763919
rs72816772
rs75117490
rs117647889
rs72998964
rs254079
rs215237
rs112139040
All
−0.2
−0.1
0.0
0.1
0.2
MR leave−one−out sensitivity analysis for
'CD19 on IgD− CD38br' on 'Juvenile arthritis'

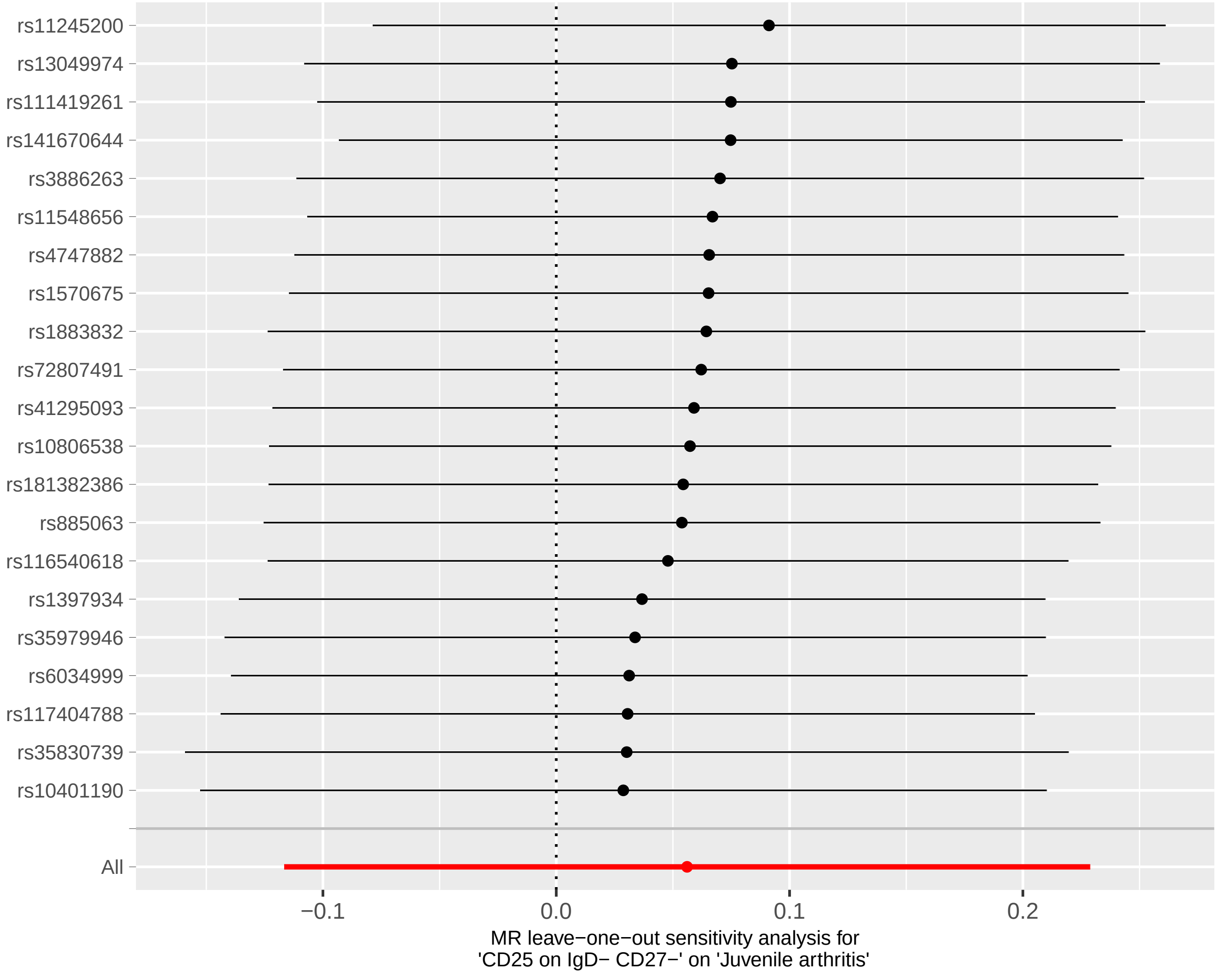

rs11245200
rs13049974
rs111419261
rs141670644
rs3886263
rs11548656
rs4747882
rs1570675
rs1883832
rs72807491
rs41295093
rs10806538
rs181382386
rs885063
rs116540618
rs1397934
rs35979946
rs6034999
rs117404788
rs35830739
rs10401190
All
−0.1
0.0
0.1
0.2
MR leave−one−out sensitivity analysis for
'CD25 on IgD− CD27−' on 'Juvenile arthritis'

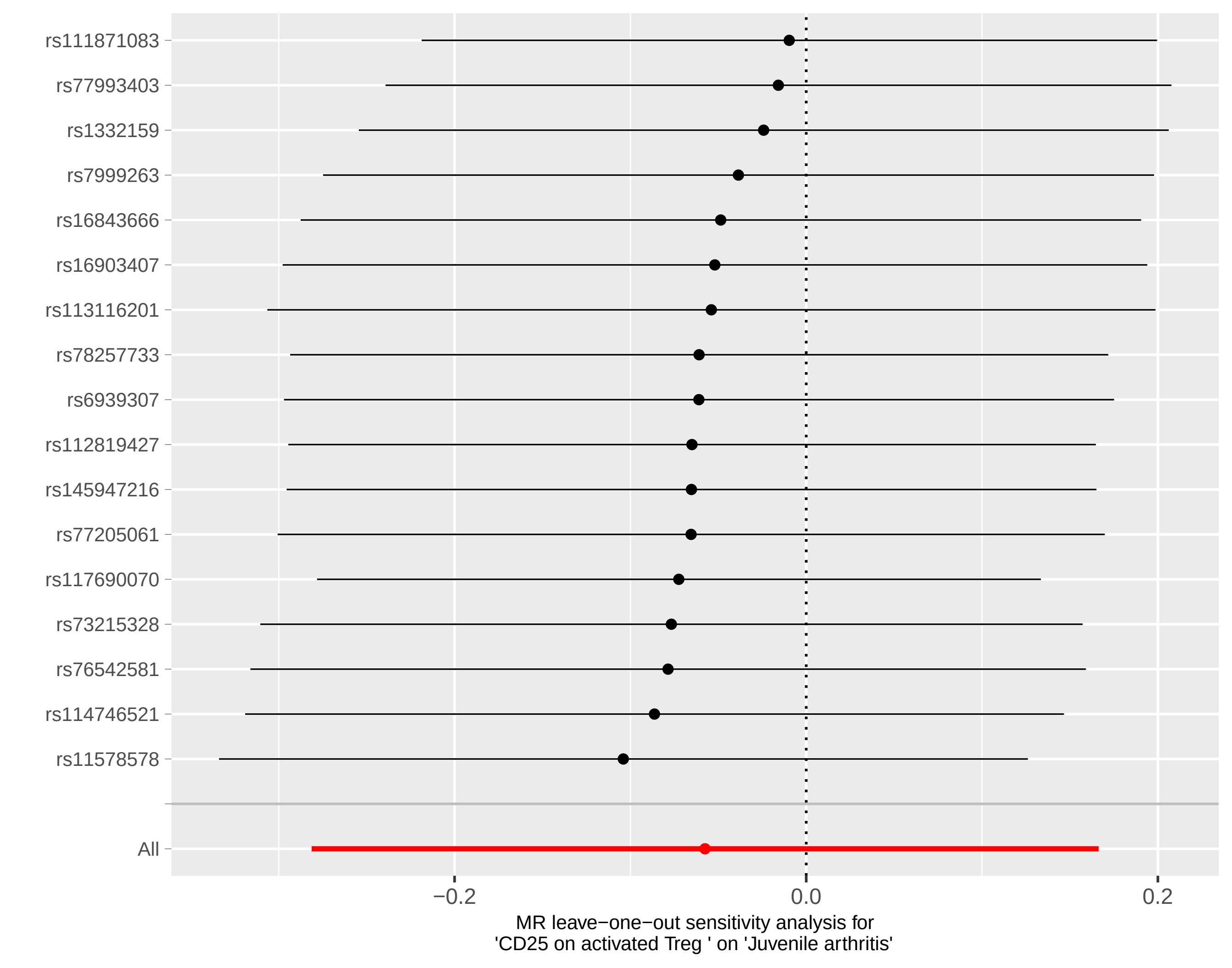
rs111871083
rs77993403
rs1332159
rs7999263
rs16843666
rs16903407
rs113116201
rs78257733
rs6939307
rs112819427
rs145947216
rs77205061
rs117690070
rs73215328
rs76542581
rs114746521
rs11578578
All
−0.2
0.0
0.2
MR leave−one−out sensitivity analysis for
'CD25 on activated Treg ' on 'Juvenile arthritis'

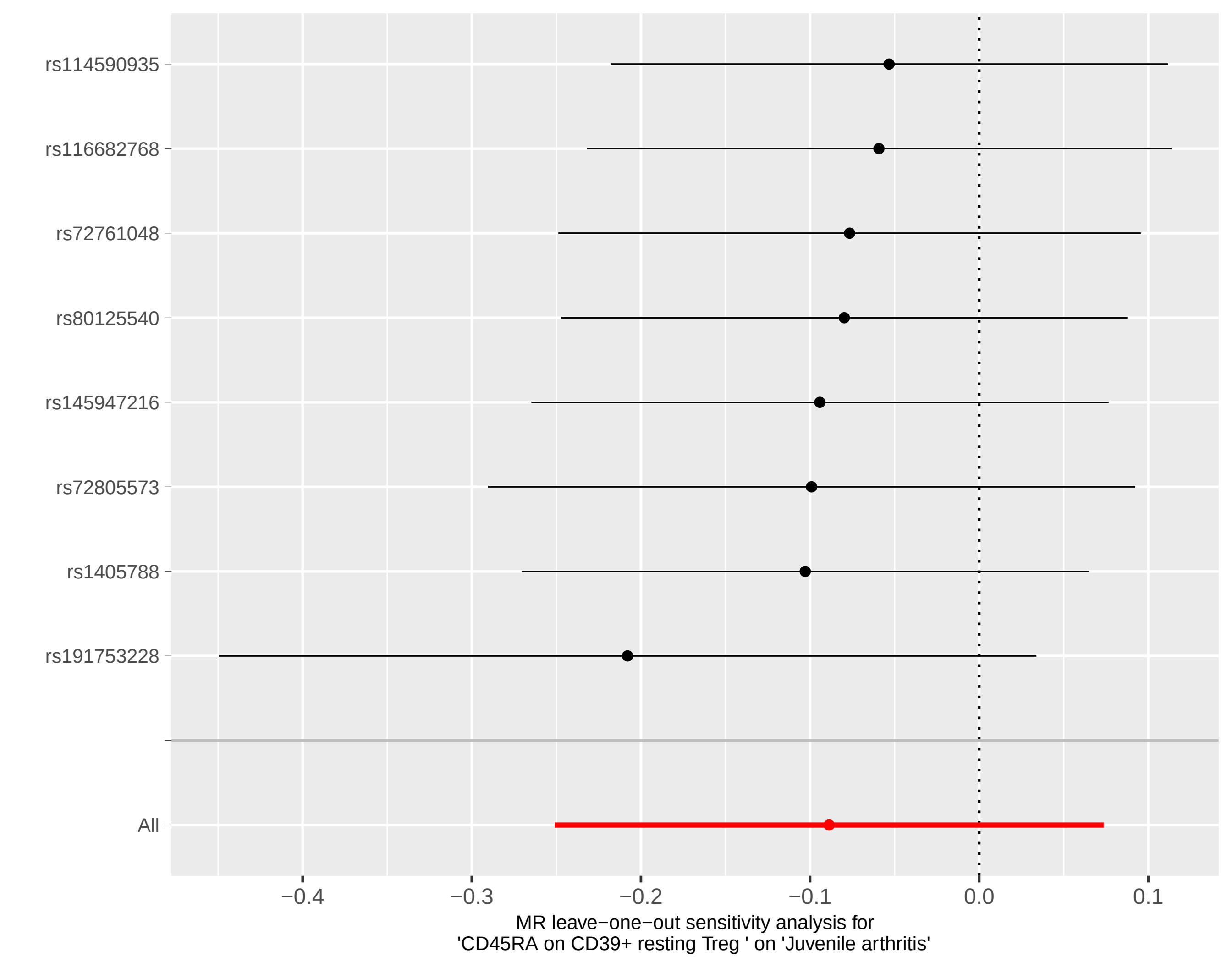

rs114590935
rs116682768
rs72761048
rs80125540
rs145947216
rs72805573
rs1405788
rs191753228
All
−0.4
−0.3
−0.2
−0.1
0.0
0.1
MR leave−one−out sensitivity analysis for
'CD45RA on CD39+ resting Treg ' on 'Juvenile arthritis'

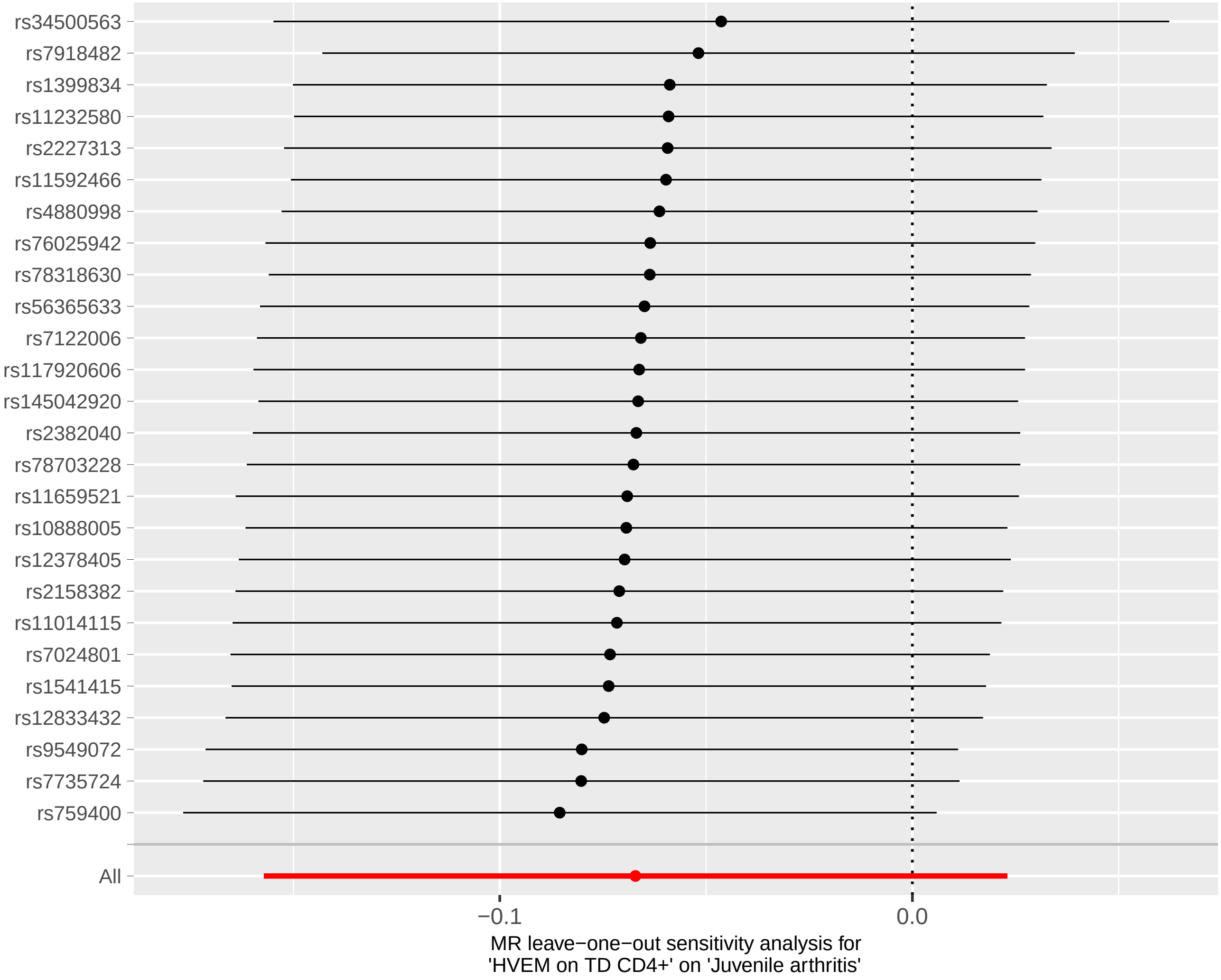

rs34500563
rs7918482
rs1399834
rs11232580
rs2227313
rs11592466
rs4880998
rs76025942
rs78318630
rs56365633
rs7122006
rs117920606
rs145042920
rs2382040
rs78703228
rs11659521
rs10888005
rs12378405
rs2158382
rs11014115
rs7024801
rs1541415
rs12833432
rs9549072
rs7735724
rs759400
All
−0.1
0.0
MR leave−one−out sensitivity analysis for
'HVEM on TD CD4+' on 'Juvenile arthritis'

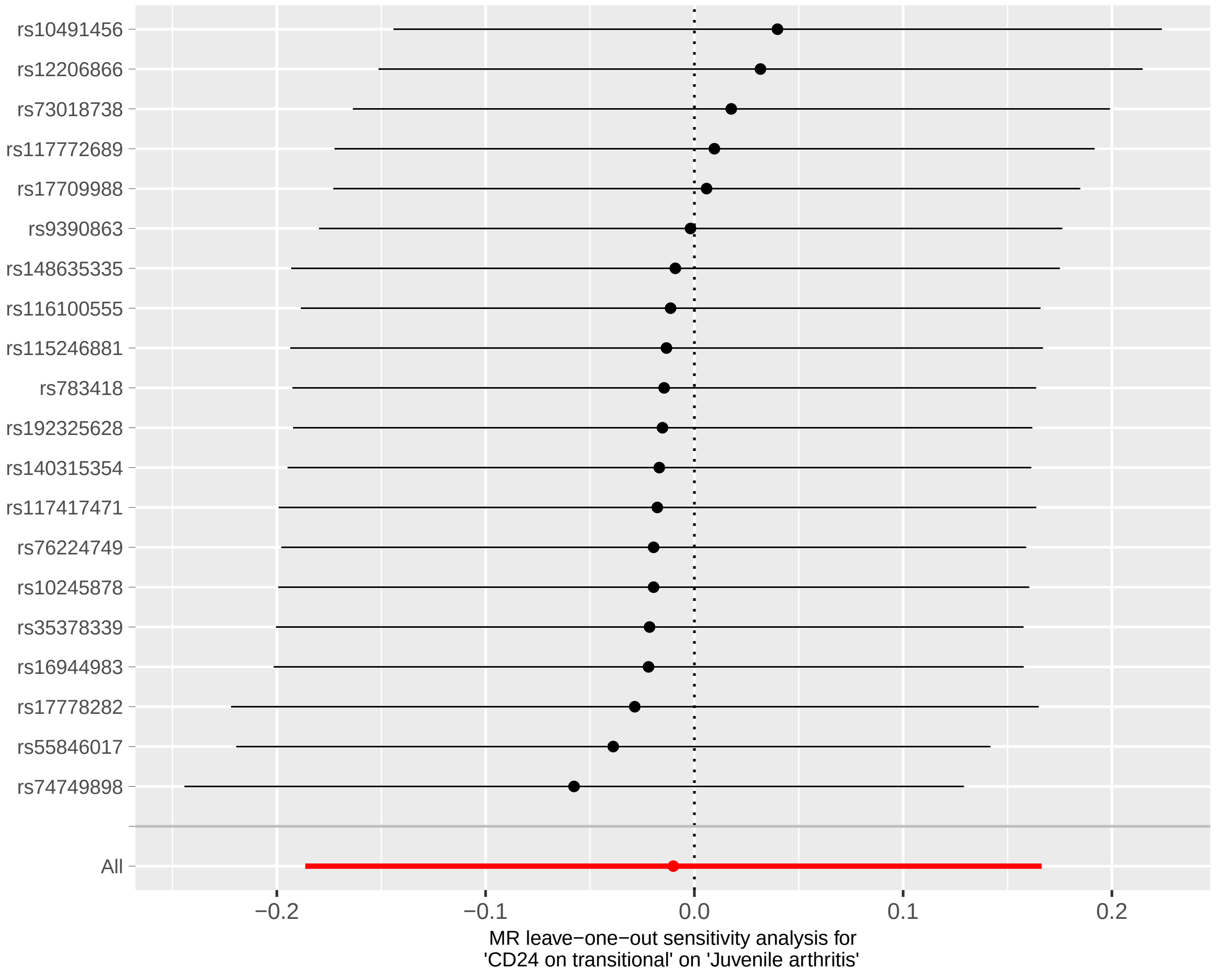
rs10491456
rs12206866
rs73018738
rs117772689
rs17709988
rs9390863
rs148635335
rs116100555
rs115246881
rs783418
rs192325628
rs140315354
rs117417471
rs76224749
rs10245878
rs35378339
rs16944983
rs17778282
rs55846017
rs74749898
All
−0.2
−0.1
0.0
0.1
0.2
MR leave−one−out sensitivity analysis for
'CD24 on transitional' on 'Juvenile arthritis'

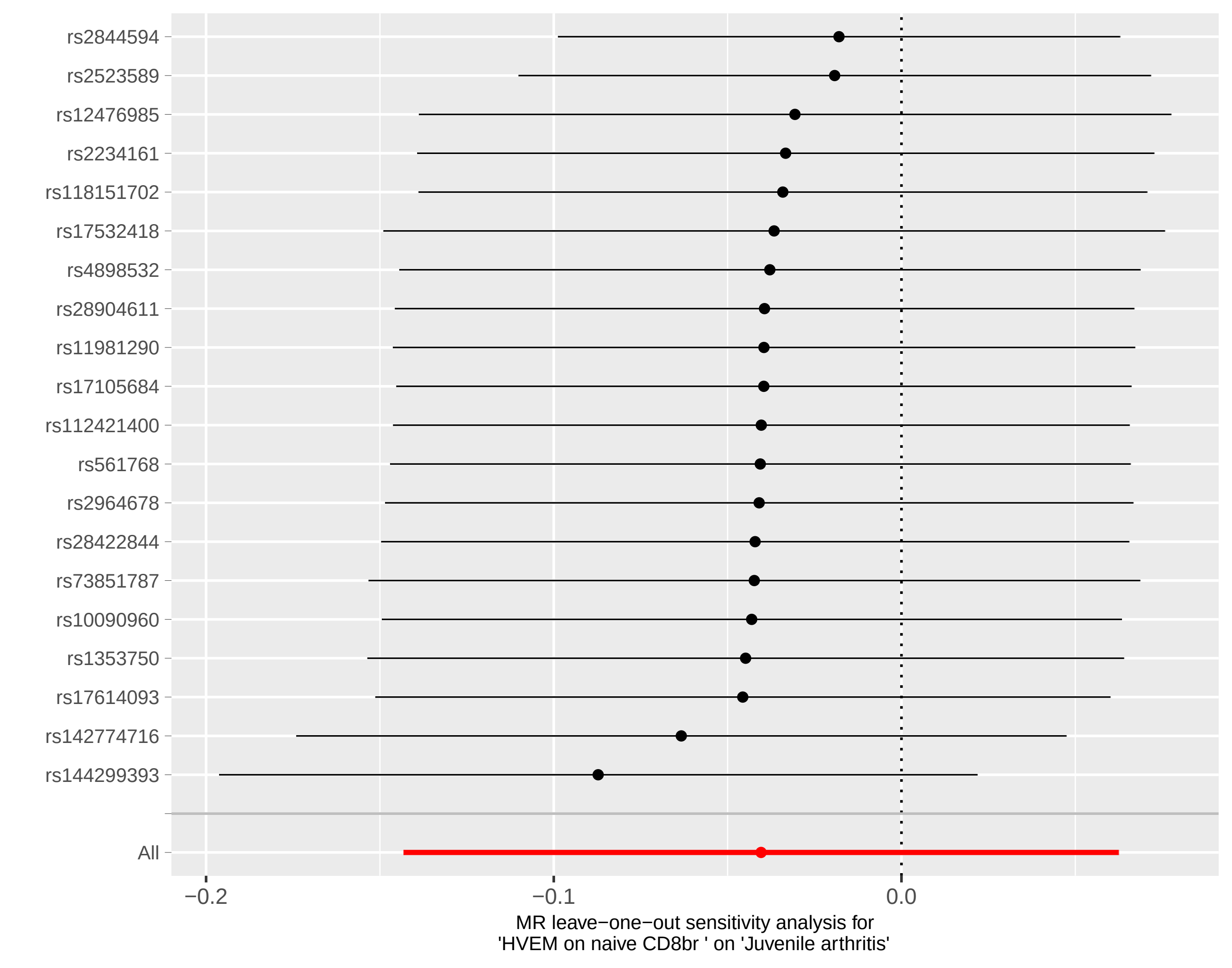

rs2844594
rs2523589
rs12476985
rs2234161
rs118151702
rs17532418
rs4898532
rs28904611
rs11981290
rs17105684
rs112421400
rs561768
rs2964678
rs28422844
rs73851787
rs10090960
rs1353750
rs17614093
rs142774716
rs144299393
All
−0.2
−0.1
0.0
MR leave−one−out sensitivity analysis for
'HVEM on naive CD8br ' on 'Juvenile arthritis'

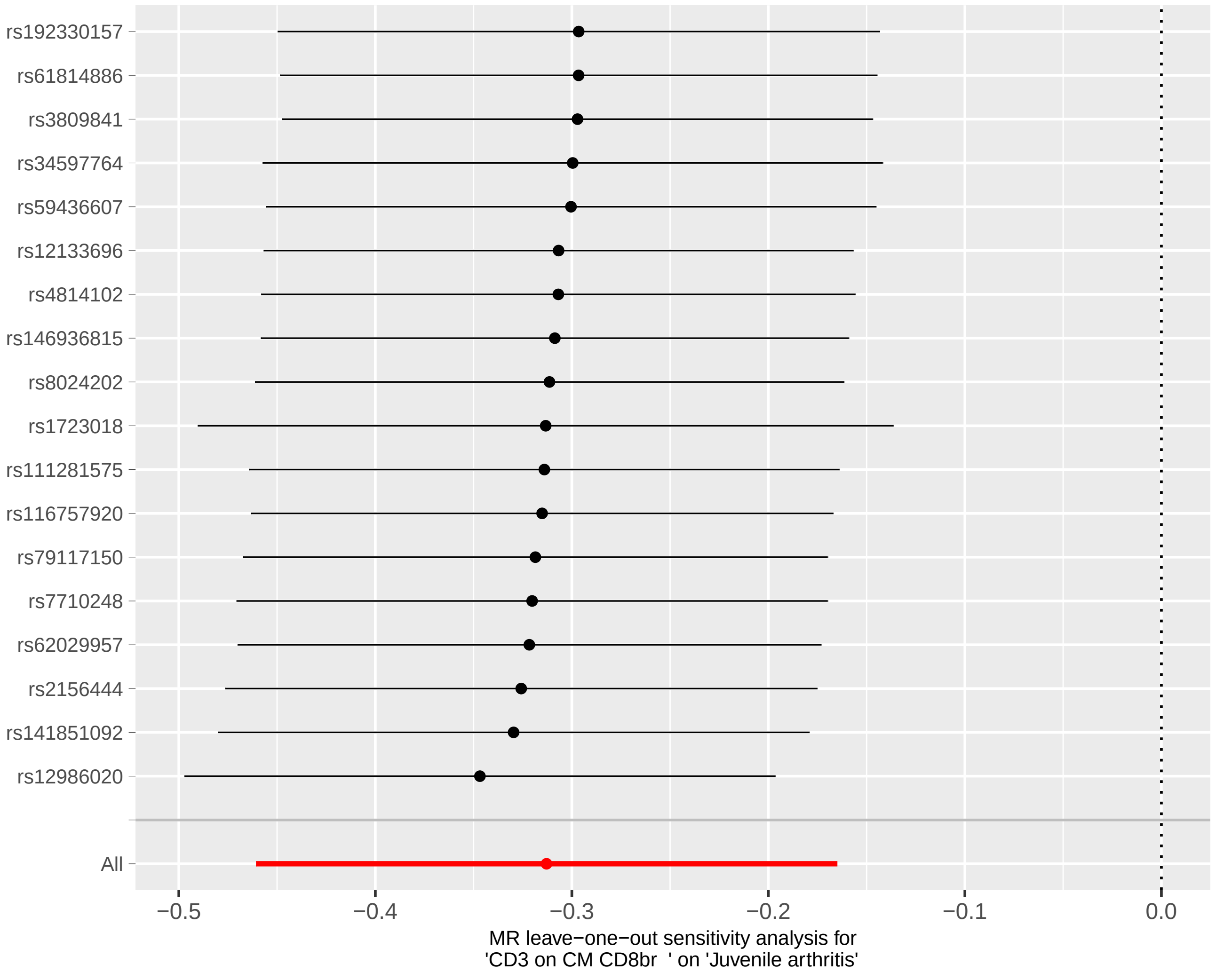

rs192330157
rs61814886
rs3809841
rs34597764
rs59436607
rs12133696
rs4814102
rs146936815
rs8024202
rs1723018
rs111281575
rs116757920
rs79117150
rs7710248
rs62029957
rs2156444
rs141851092
rs12986020
All
−0.5
−0.4
−0.3
−0.2
−0.1
0.0
MR leave−one−out sensitivity analysis for
'CD3 on CM CD8br ' on 'Juvenile arthritis'

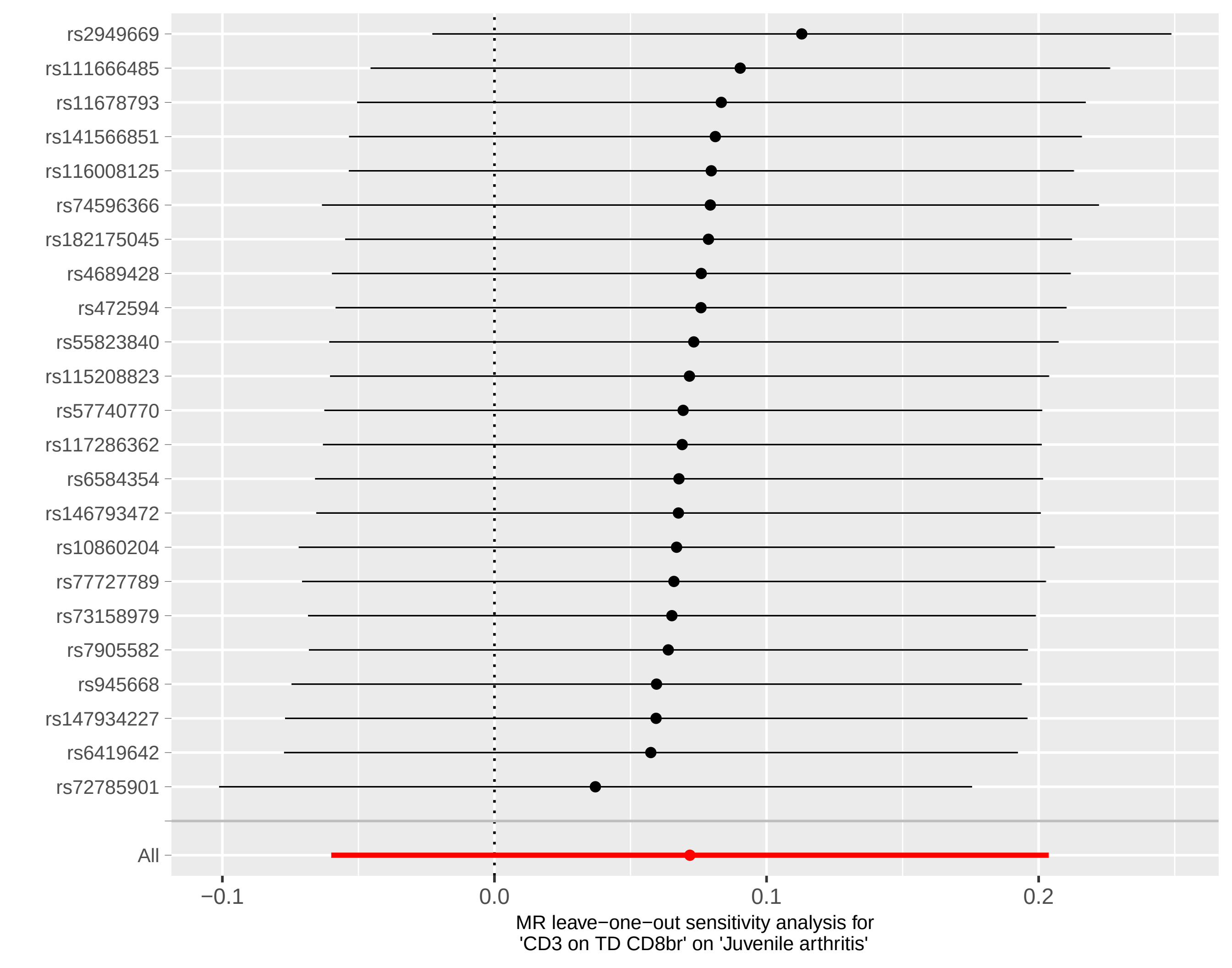
rs2949669
rs111666485
rs11678793
rs141566851
rs116008125
rs74596366
rs182175045
rs4689428
rs472594
rs55823840
rs115208823
rs57740770
rs117286362
rs6584354
rs146793472
rs10860204
rs77727789
rs73158979
rs7905582
rs945668
rs147934227
rs6419642
rs72785901
All
−0.1
0.0
0.1
0.2
MR leave−one−out sensitivity analysis for
'CD3 on TD CD8br' on 'Juvenile arthritis'

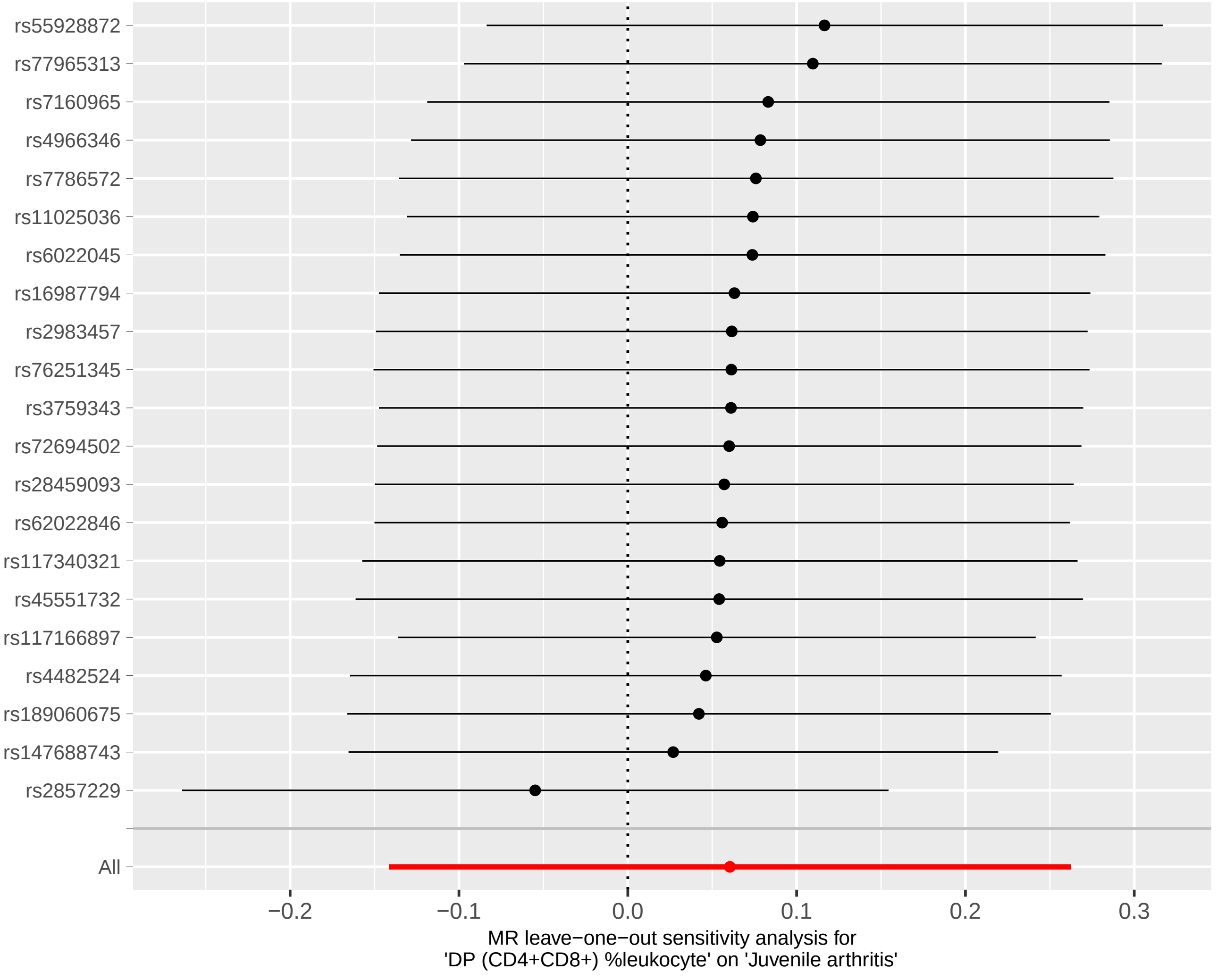
rs55928872
rs77965313
rs7160965
rs4966346
rs7786572
rs11025036
rs6022045
rs16987794
rs2983457
rs76251345
rs3759343
rs72694502
rs28459093
rs62022846
rs117340321
rs45551732
rs117166897
rs4482524
rs189060675
rs147688743
rs2857229
All
−0.2
−0.1
0.0
0.1
0.2
0.3
MR leave−one−out sensitivity analysis for
'DP (CD4+CD8+) %leukocyte' on 'Juvenile arthritis'

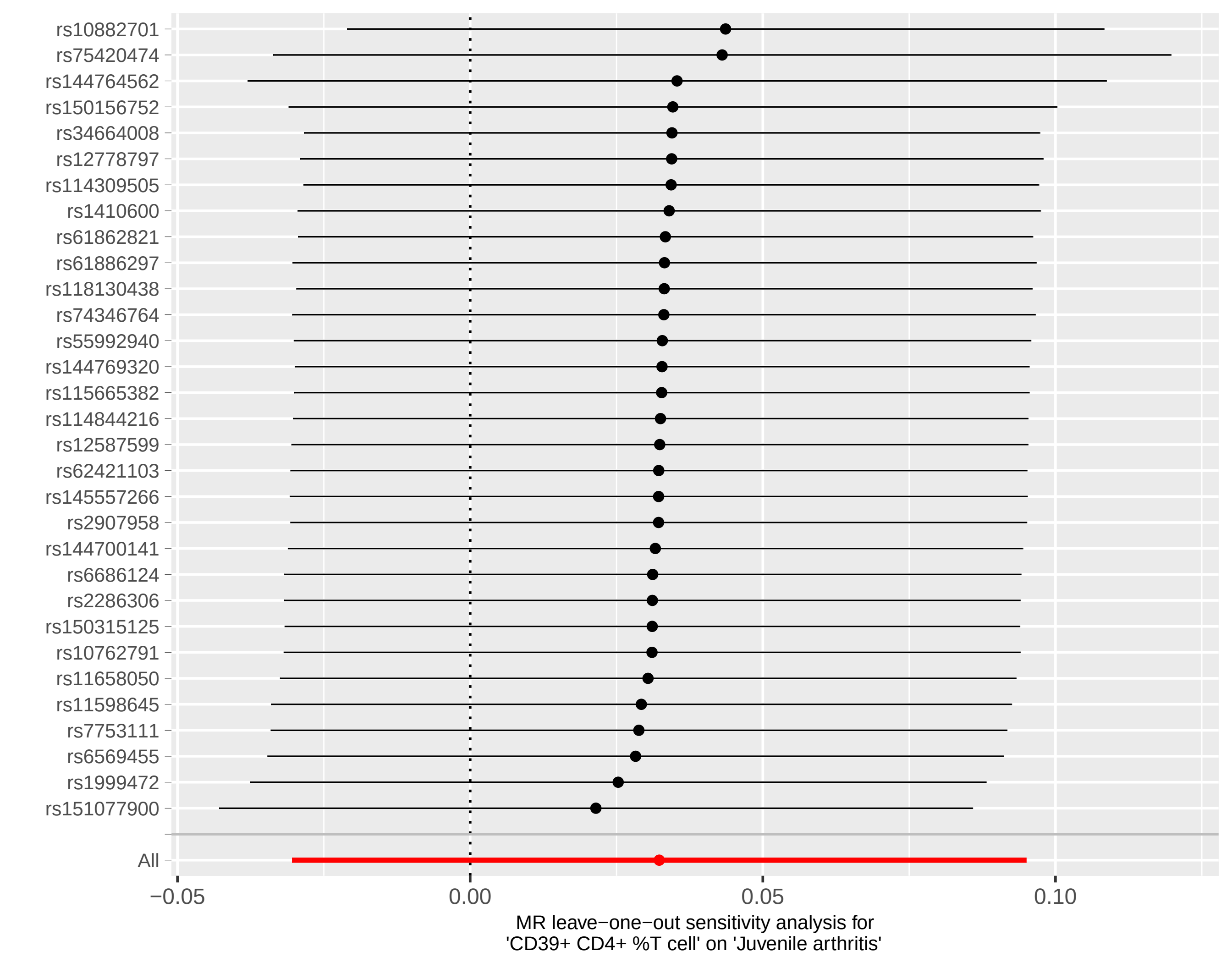
rs10882701
rs75420474
rs144764562
rs150156752
rs34664008
rs12778797
rs114309505
rs1410600
rs61862821
rs61886297
rs118130438
rs74346764
rs55992940
rs144769320
rs115665382
rs114844216
rs12587599
rs62421103
rs145557266
rs2907958
rs144700141
rs6686124
rs2286306
rs150315125
rs10762791
rs11658050
rs11598645
rs7753111
rs6569455
rs1999472
rs151077900
All
−0.05
0.00
0.05
0.10
MR leave−one−out sensitivity analysis for
'CD39+ CD4+ %T cell' on 'Juvenile arthritis'

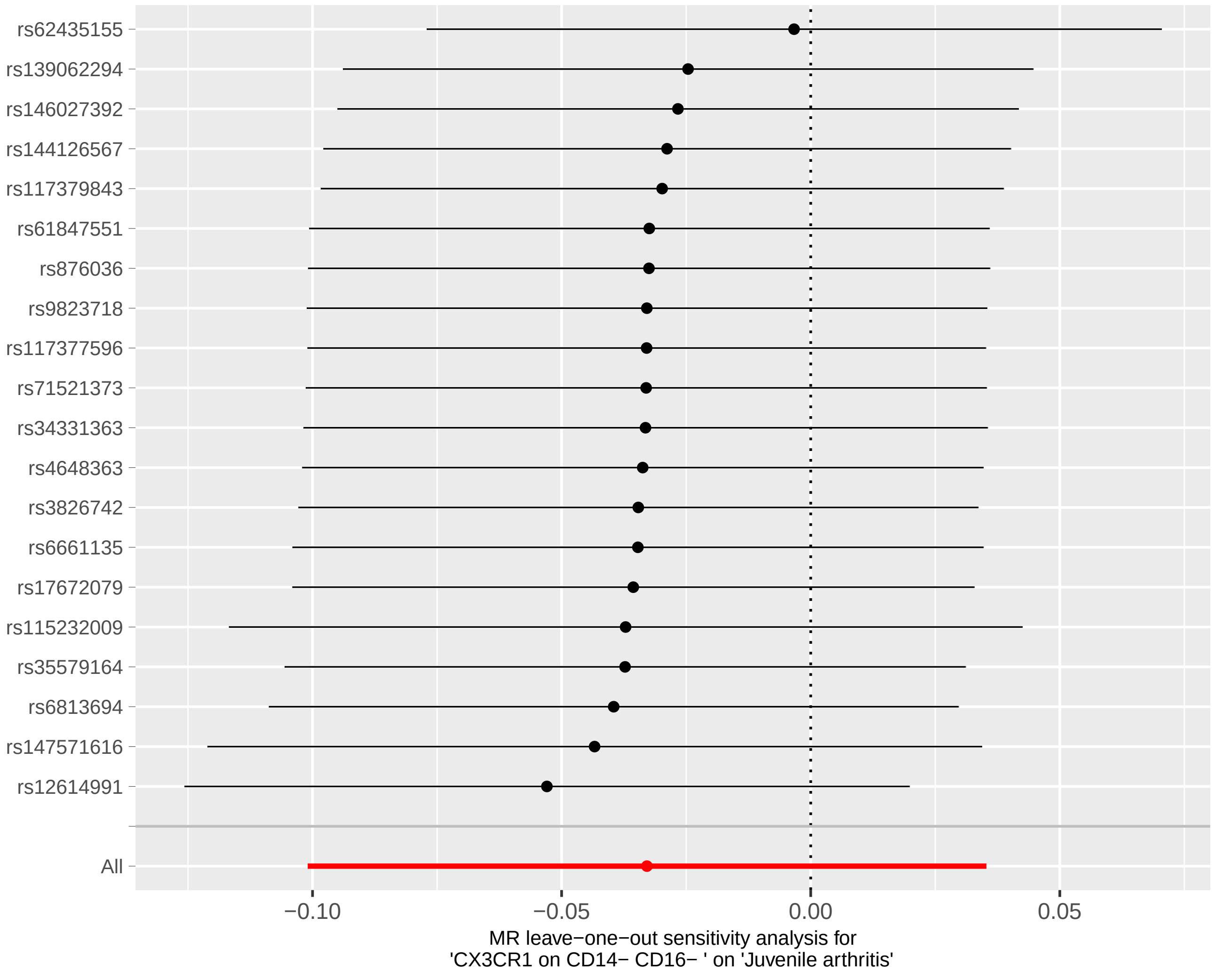
rs62435155
rs139062294
rs146027392
rs144126567
rs117379843
rs61847551
rs876036
rs9823718
rs117377596
rs71521373
rs34331363
rs4648363
rs3826742
rs6661135
rs17672079
rs115232009
rs35579164
rs6813694
rs147571616
rs12614991
All
−0.10
−0.05
0.00
0.05
MR leave−one−out sensitivity analysis for
'CX3CR1 on CD14− CD16− ' on 'Juvenile arthritis'

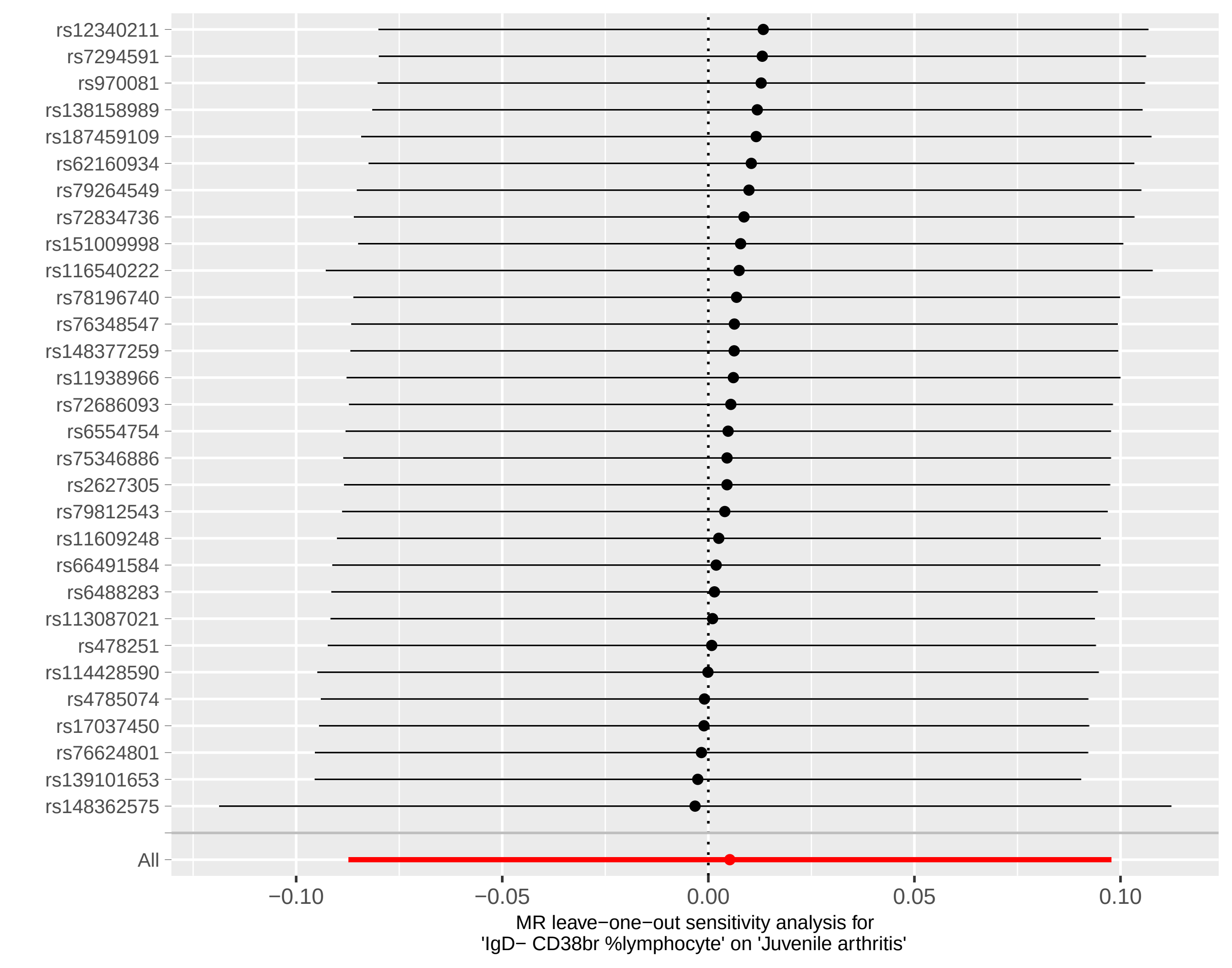

rs12340211
rs7294591
rs970081
rs138158989
rs187459109
rs62160934
rs79264549
rs72834736
rs151009998
rs116540222
rs78196740
rs76348547
rs148377259
rs11938966
rs72686093
rs6554754
rs75346886
rs2627305
rs79812543
rs11609248
rs66491584
rs6488283
rs113087021
rs478251
rs114428590
rs4785074
rs17037450
rs76624801
rs139101653
rs148362575
All
−0.10
−0.05
0.00
0.05
0.10
MR leave−one−out sensitivity analysis for
'IgD− CD38br %lymphocyte' on 'Juvenile arthritis'

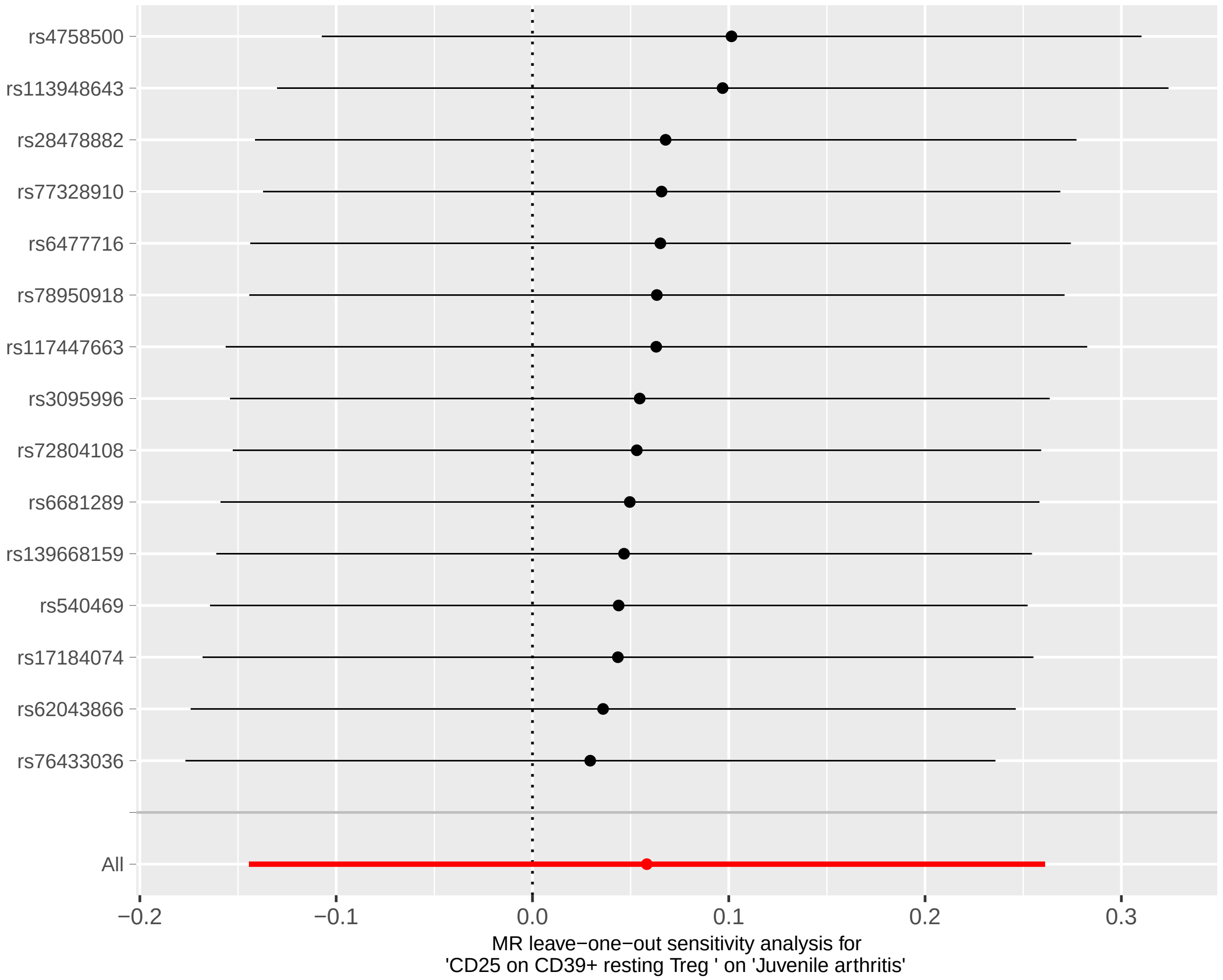
rs4758500
rs113948643
rs28478882
rs77328910
rs6477716
rs78950918
rs117447663
rs3095996
rs72804108
rs6681289
rs139668159
rs540469
rs17184074
rs62043866
rs76433036
All
−0.2
−0.1
0.0
0.1
0.2
0.3
MR leave−one−out sensitivity analysis for
'CD25 on CD39+ resting Treg ' on 'Juvenile arthritis'

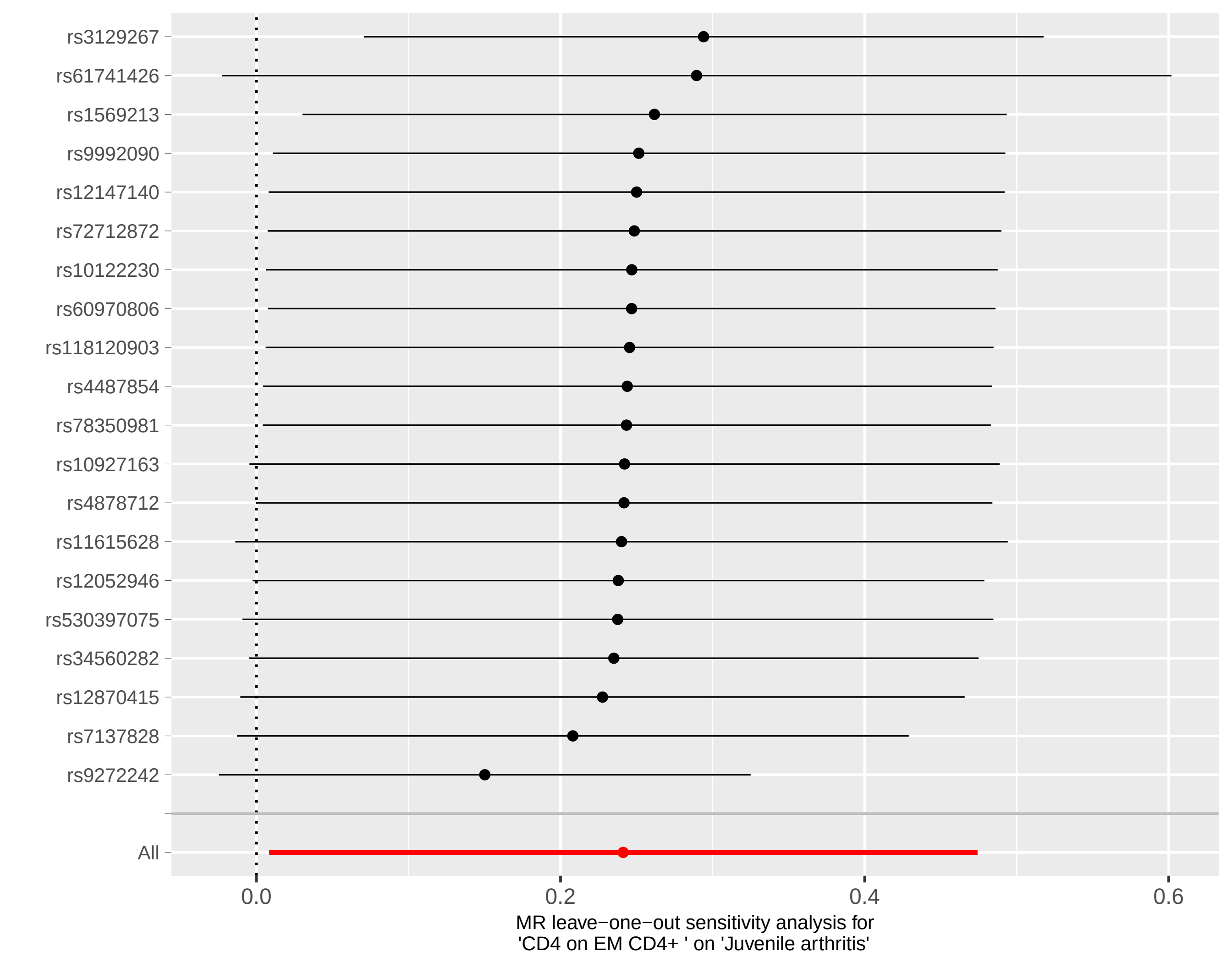
rs3129267
rs61741426
rs1569213
rs9992090
rs12147140
rs72712872
rs10122230
rs60970806
rs118120903
rs4487854
rs78350981
rs10927163
rs4878712
rs11615628
rs12052946
rs530397075
rs34560282
rs12870415
rs7137828
rs9272242
All
0.0
0.2
0.4
0.6
MR leave−one−out sensitivity analysis for
'CD4 on EM CD4+ ' on 'Juvenile arthritis'

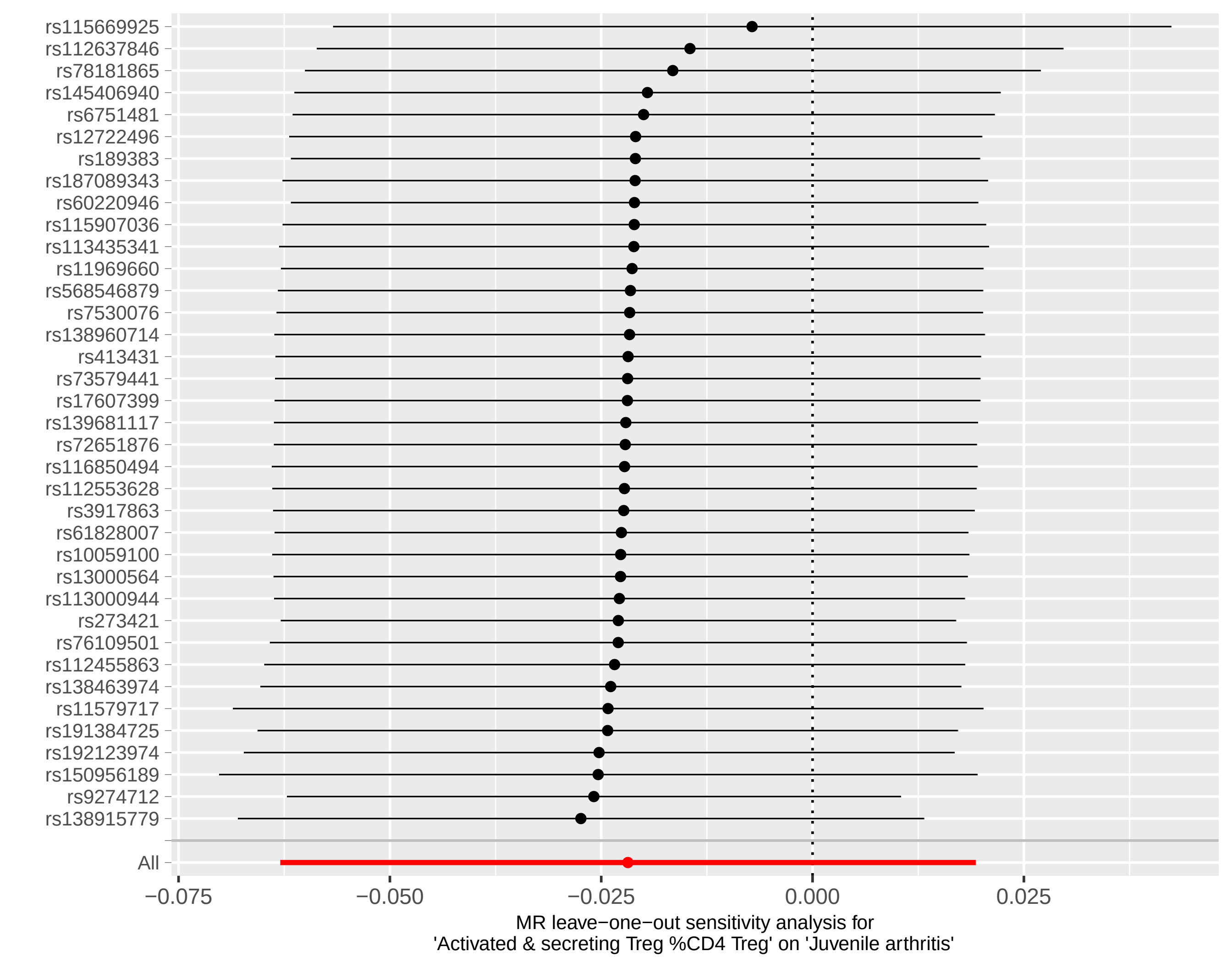

rs115669925
rs112637846
rs78181865
rs145406940
rs6751481
rs12722496
rs189383
rs187089343
rs60220946
rs115907036
rs113435341
rs11969660
rs568546879
rs7530076
rs138960714
rs413431
rs73579441
rs17607399
rs139681117
rs72651876
rs116850494
rs112553628
rs3917863
rs61828007
rs10059100
rs13000564
rs113000944
rs273421
rs76109501
rs112455863
rs138463974
rs11579717
rs191384725
rs192123974
rs150956189
rs9274712
rs138915779
All
−0.075
−0.050
−0.025
0.000
0.025
MR leave−one−out sensitivity analysis for
'Activated & secreting Treg %CD4 Treg' on 'Juvenile arthritis'

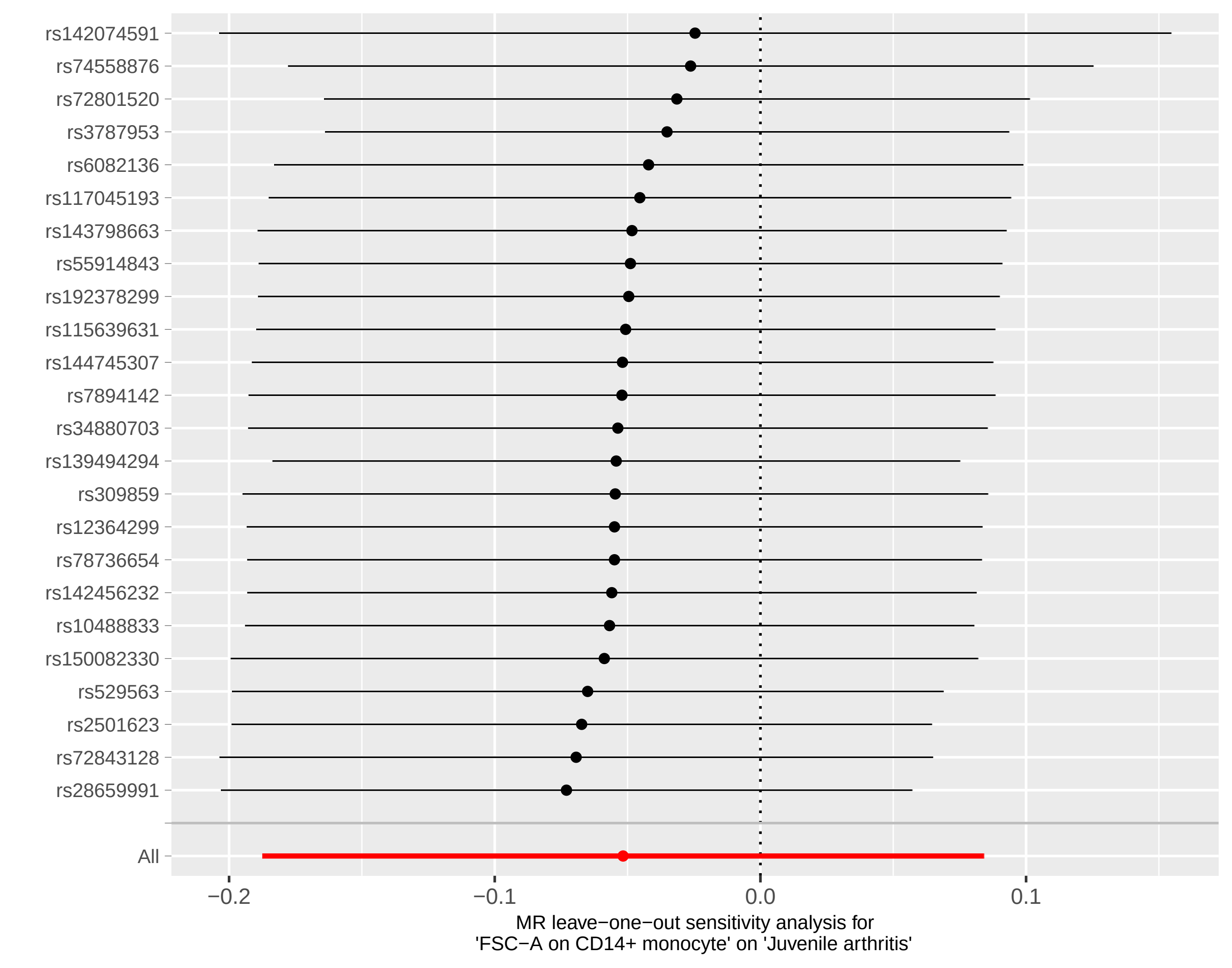
rs142074591
rs74558876
rs72801520
rs3787953
rs6082136
rs117045193
rs143798663
rs55914843
rs192378299
rs115639631
rs144745307
rs7894142
rs34880703
rs139494294
rs309859
rs12364299
rs78736654
rs142456232
rs10488833
rs150082330
rs529563
rs2501623
rs72843128
rs28659991
All
−0.2
−0.1
0.0
0.1
MR leave−one−out sensitivity analysis for
'FSC−A on CD14+ monocyte' on 'Juvenile arthritis'

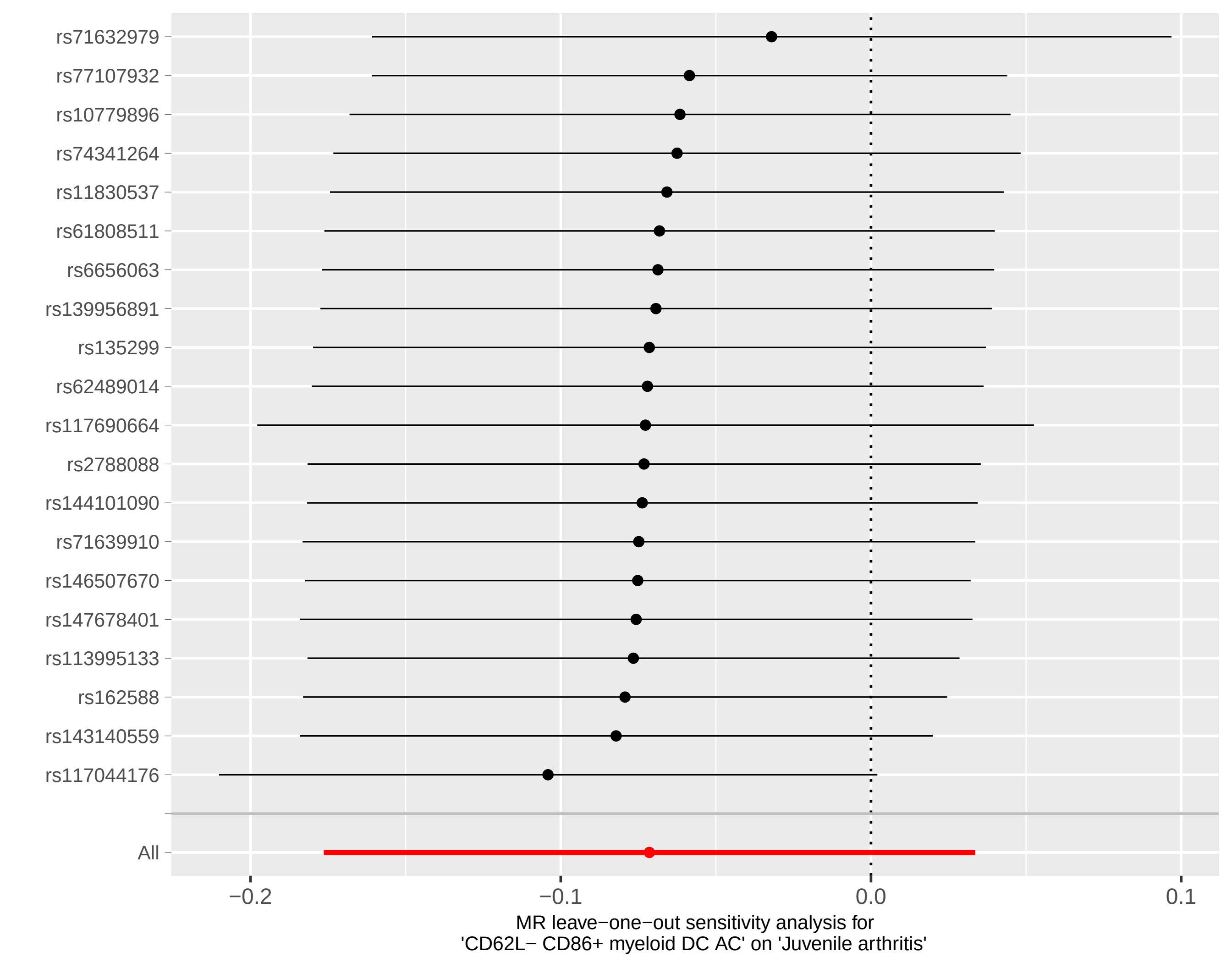
rs71632979
rs77107932
rs10779896
rs74341264
rs11830537
rs61808511
rs6656063
rs139956891
rs135299
rs62489014
rs117690664
rs2788088
rs144101090
rs71639910
rs146507670
rs147678401
rs113995133
rs162588
rs143140559
rs117044176
All
−0.2
−0.1
0.0
0.1
MR leave−one−out sensitivity analysis for
'CD62L− CD86+ myeloid DC AC' on 'Juvenile arthritis'

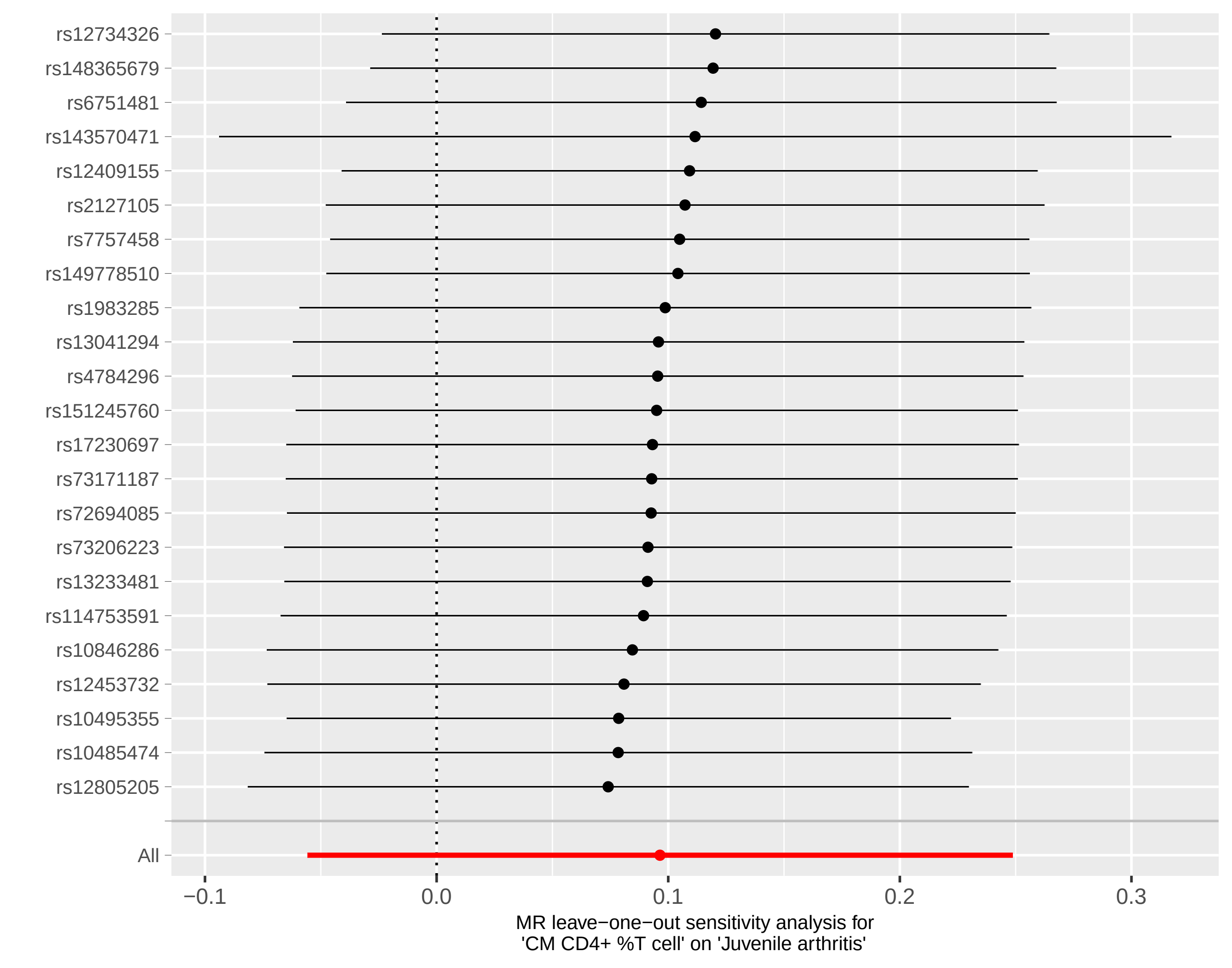
rs12734326
rs148365679
rs6751481
rs143570471
rs12409155
rs2127105
rs7757458
rs149778510
rs1983285
rs13041294
rs4784296
rs151245760
rs17230697
rs73171187
rs72694085
rs73206223
rs13233481
rs114753591
rs10846286
rs12453732
rs10495355
rs10485474
rs12805205
All
−0.1
0.0
0.1
0.2
0.3
MR leave−one−out sensitivity analysis for
'CM CD4+ %T cell' on 'Juvenile arthritis'

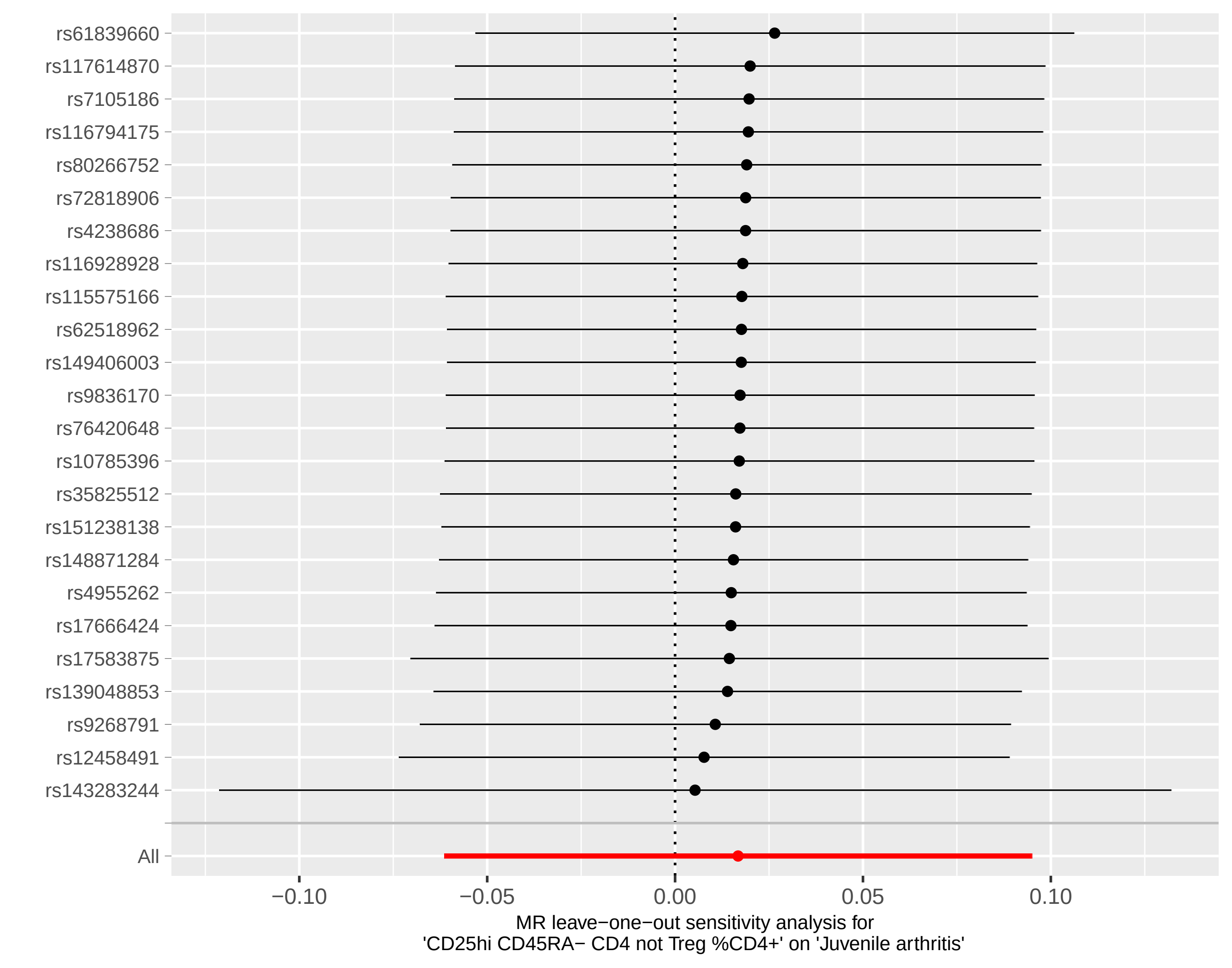

rs61839660
rs117614870
rs7105186
rs116794175
rs80266752
rs72818906
rs4238686
rs116928928
rs115575166
rs62518962
rs149406003
rs9836170
rs76420648
rs10785396
rs35825512
rs151238138
rs148871284
rs4955262
rs17666424
rs17583875
rs139048853
rs9268791
rs12458491
rs143283244
All
−0.10
−0.05
0.00
0.05
0.10
MR leave−one−out sensitivity analysis for
'CD25hi CD45RA− CD4 not Treg %CD4+' on 'Juvenile arthritis'

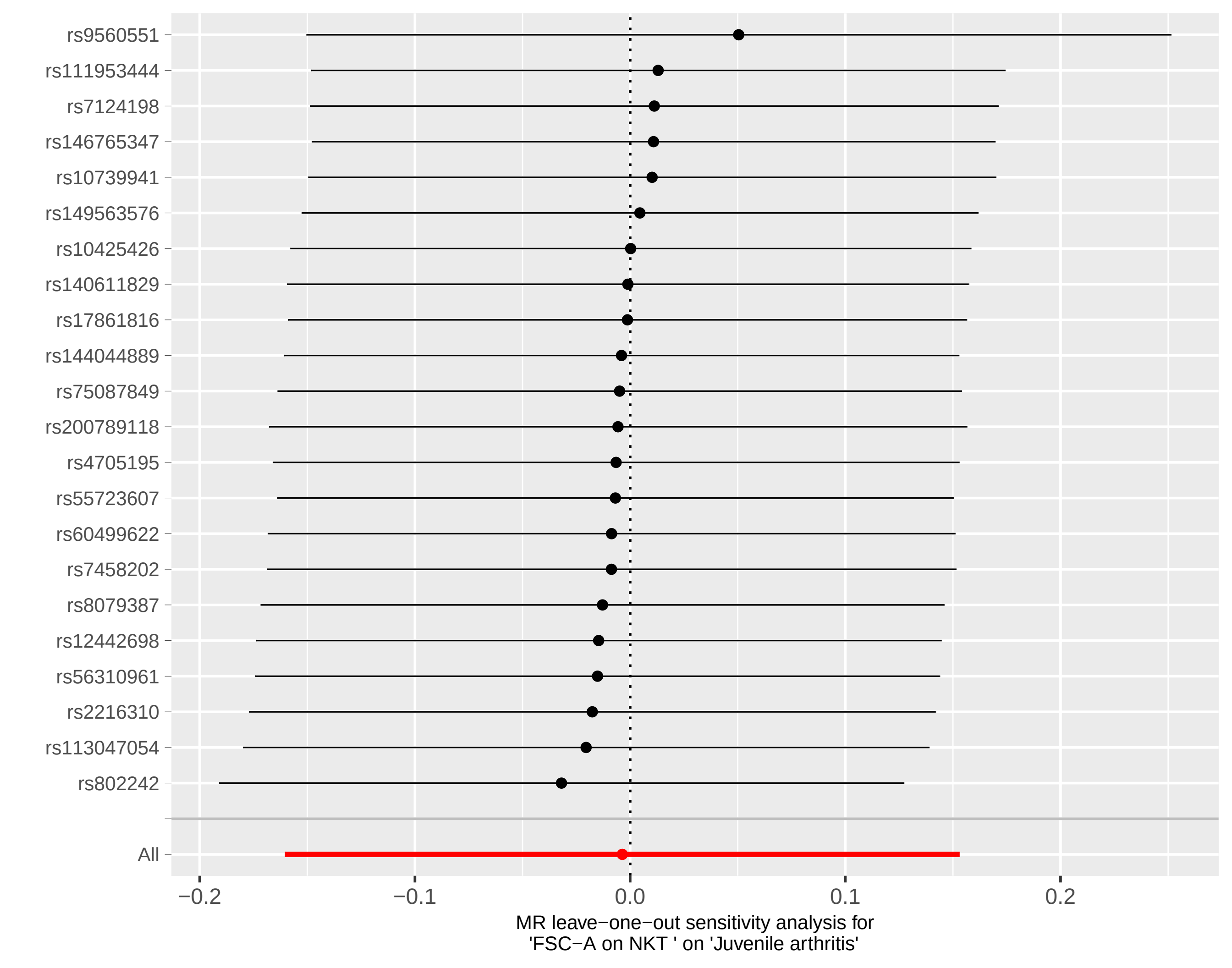

rs9560551
rs111953444
rs7124198
rs146765347
rs10739941
rs149563576
rs10425426
rs140611829
rs17861816
rs144044889
rs75087849
rs200789118
rs4705195
rs55723607
rs60499622
rs7458202
rs8079387
rs12442698
rs56310961
rs2216310
rs113047054
rs802242
All
−0.2
−0.1
0.0
0.1
0.2
MR leave−one−out sensitivity analysis for
'FSC−A on NKT ' on 'Juvenile arthritis'

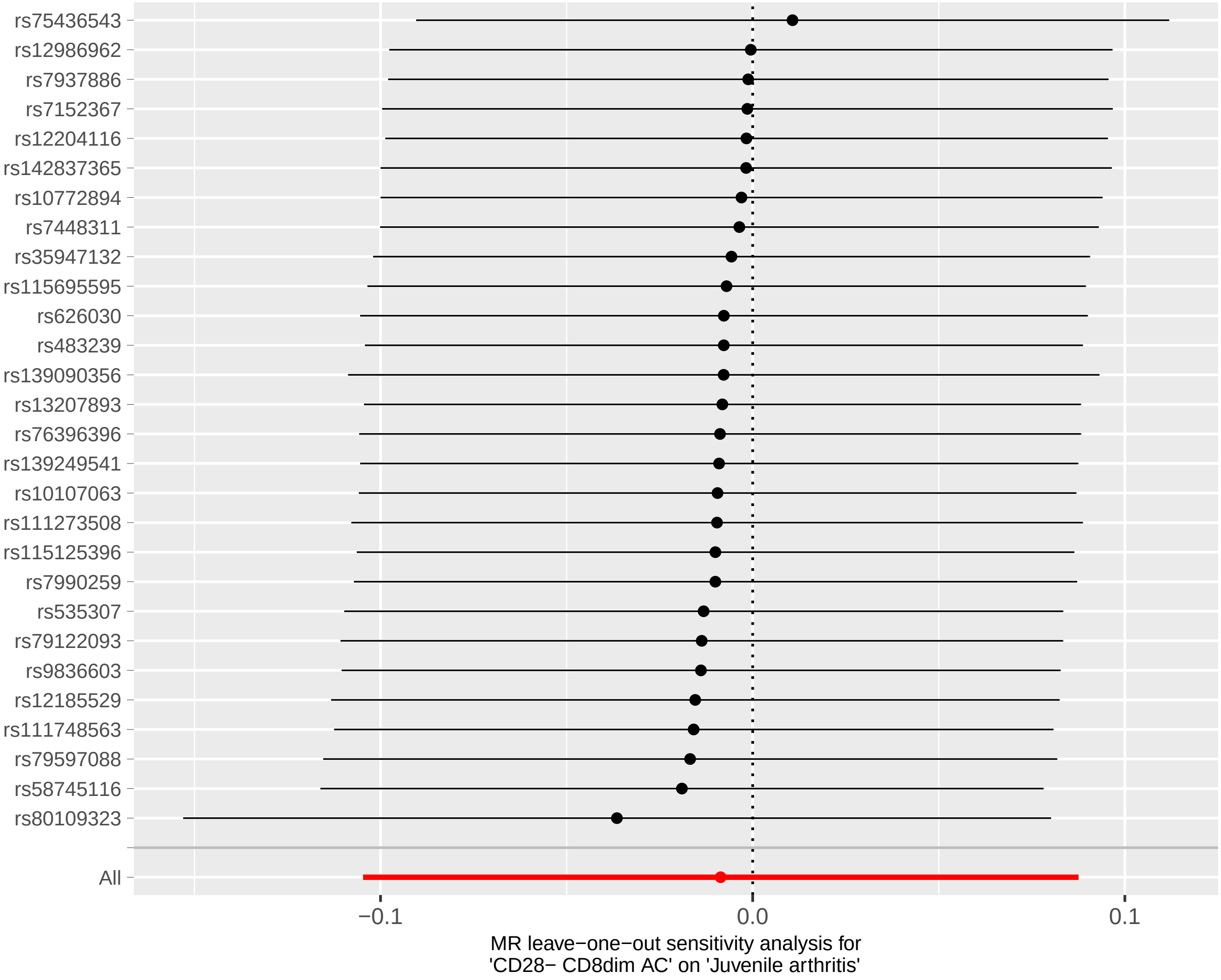
rs75436543
rs12986962
rs7937886
rs7152367
rs12204116
rs142837365
rs10772894
rs7448311
rs35947132
rs115695595
rs626030
rs483239
rs139090356
rs13207893
rs76396396
rs139249541
rs10107063
rs111273508
rs115125396
rs7990259
rs535307
rs79122093
rs9836603
rs12185529
rs111748563
rs79597088
rs58745116
rs80109323
All
−0.1
0.0
0.1
MR leave−one−out sensitivity analysis for
'CD28− CD8dim AC' on 'Juvenile arthritis'

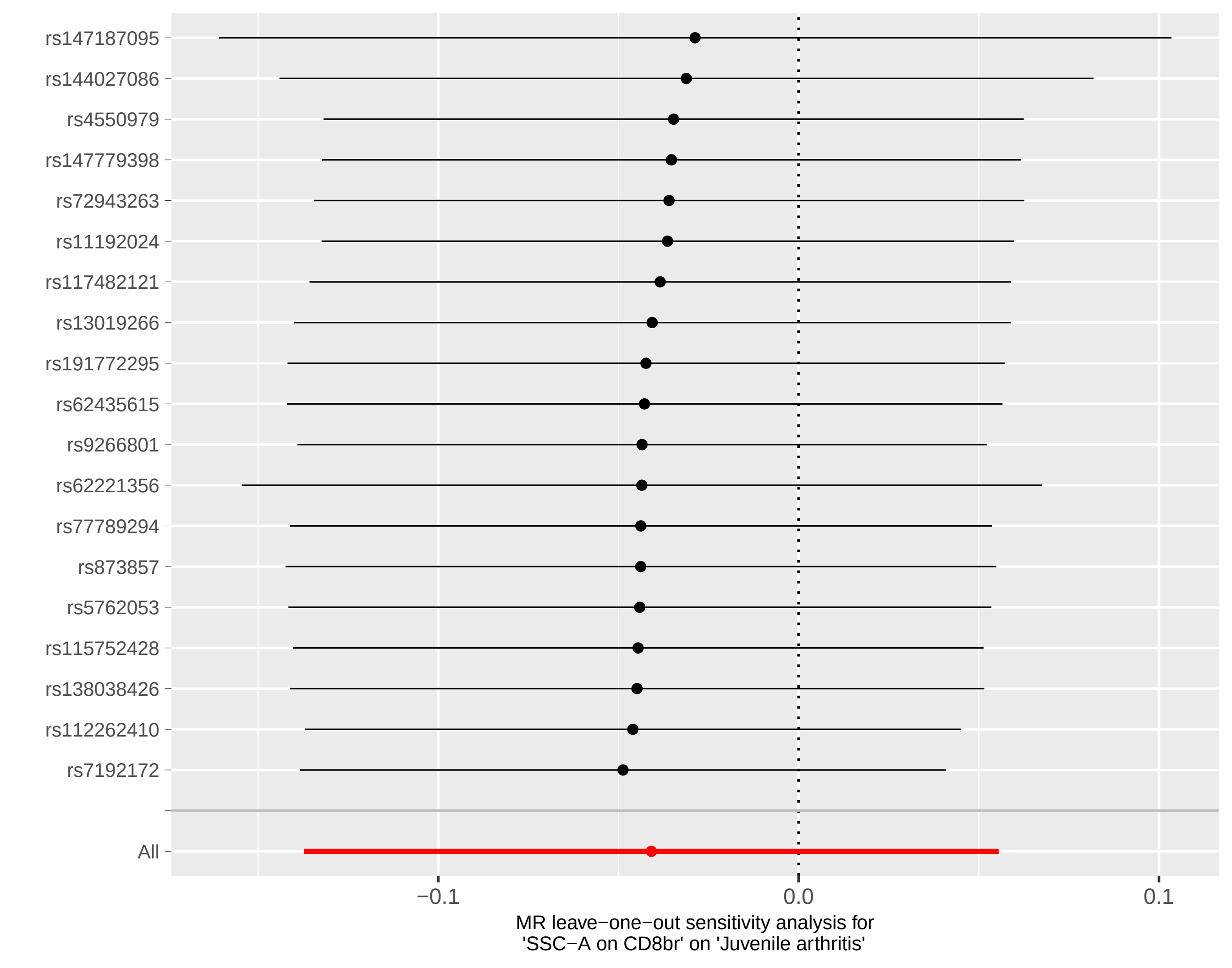

rs147187095
rs144027086
rs4550979
rs147779398
rs72943263
rs11192024
rs117482121
rs13019266
rs191772295
rs62435615
rs9266801
rs62221356
rs77789294
rs873857
rs5762053
rs115752428
rs138038426
rs112262410
rs7192172
All
−0.1
0.0
0.1
MR leave−one−out sensitivity analysis for
'SSC−A on CD8br' on 'Juvenile arthritis'

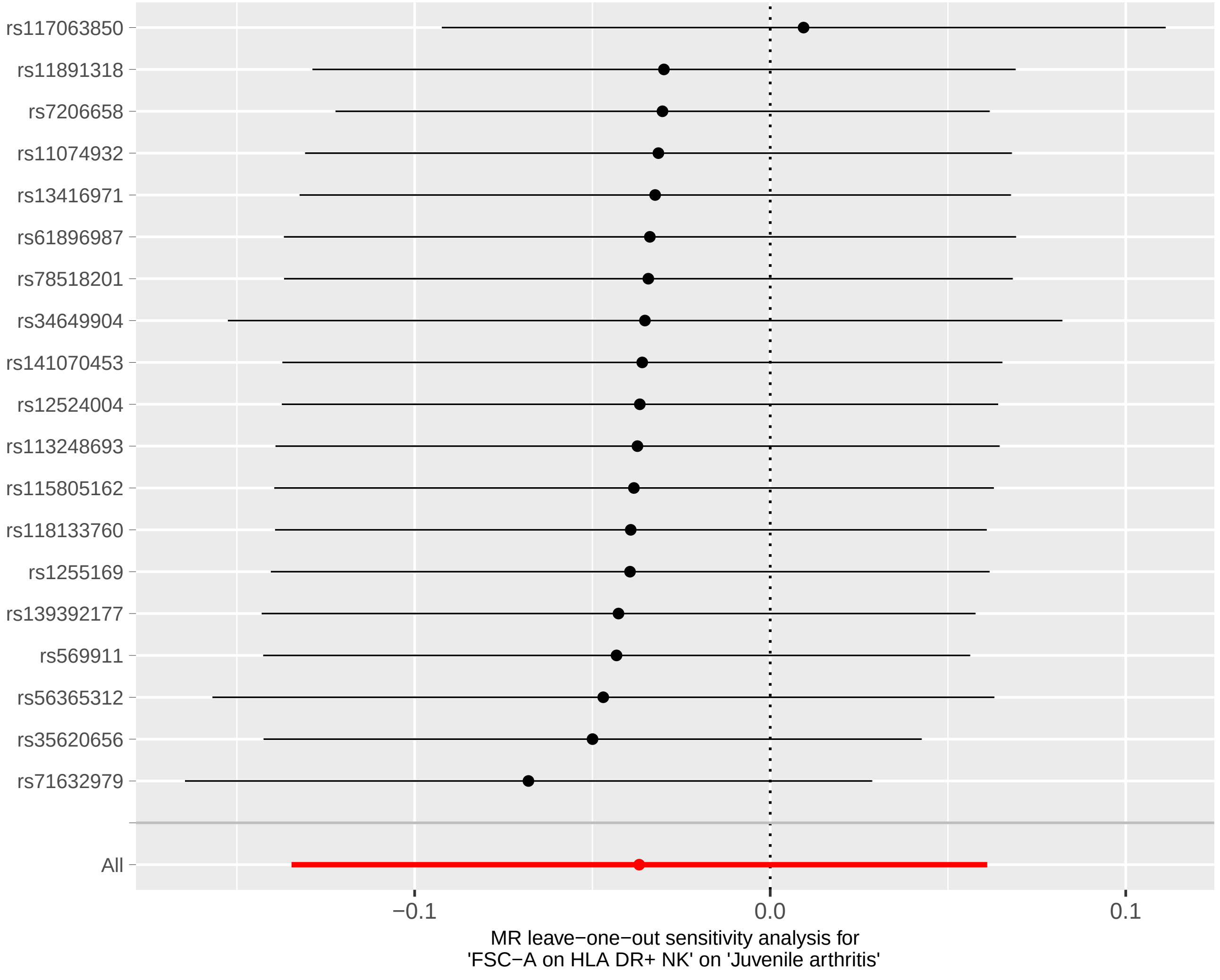
rs117063850
rs11891318
rs7206658
rs11074932
rs13416971
rs61896987
rs78518201
rs34649904
rs141070453
rs12524004
rs113248693
rs115805162
rs118133760
rs1255169
rs139392177
rs569911
rs56365312
rs35620656
rs71632979
All
−0.1
0.0
0.1
MR leave−one−out sensitivity analysis for
'FSC−A on HLA DR+ NK' on 'Juvenile arthritis'

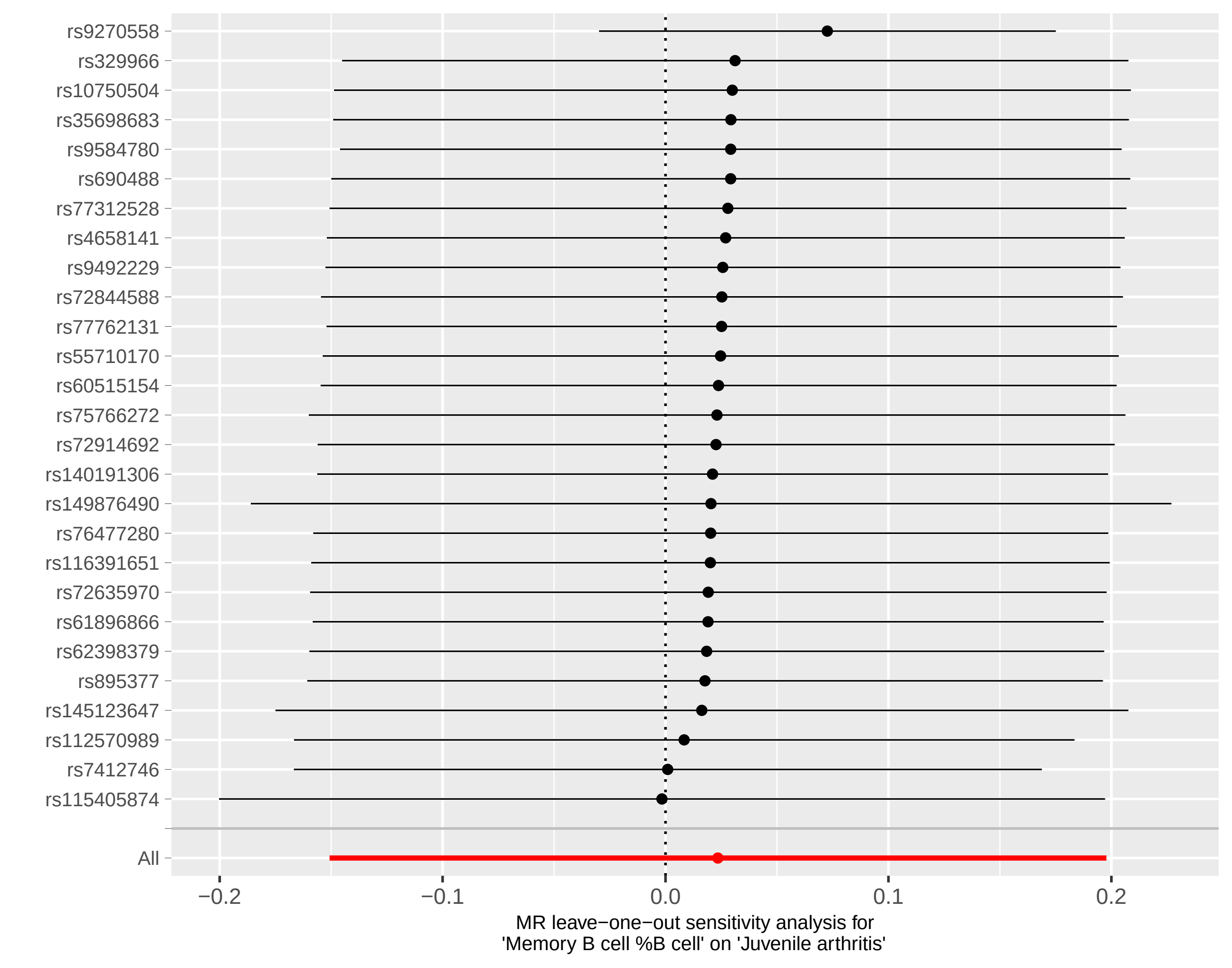
rs9270558
rs329966
rs10750504
rs35698683
rs9584780
rs690488
rs77312528
rs4658141
rs9492229
rs72844588
rs77762131
rs55710170
rs60515154
rs75766272
rs72914692
rs140191306
rs149876490
rs76477280
rs116391651
rs72635970
rs61896866
rs62398379
rs895377
rs145123647
rs112570989
rs7412746
rs115405874
All
−0.2
−0.1
0.0
0.1
0.2
MR leave−one−out sensitivity analysis for
'Memory B cell %B cell' on 'Juvenile arthritis'

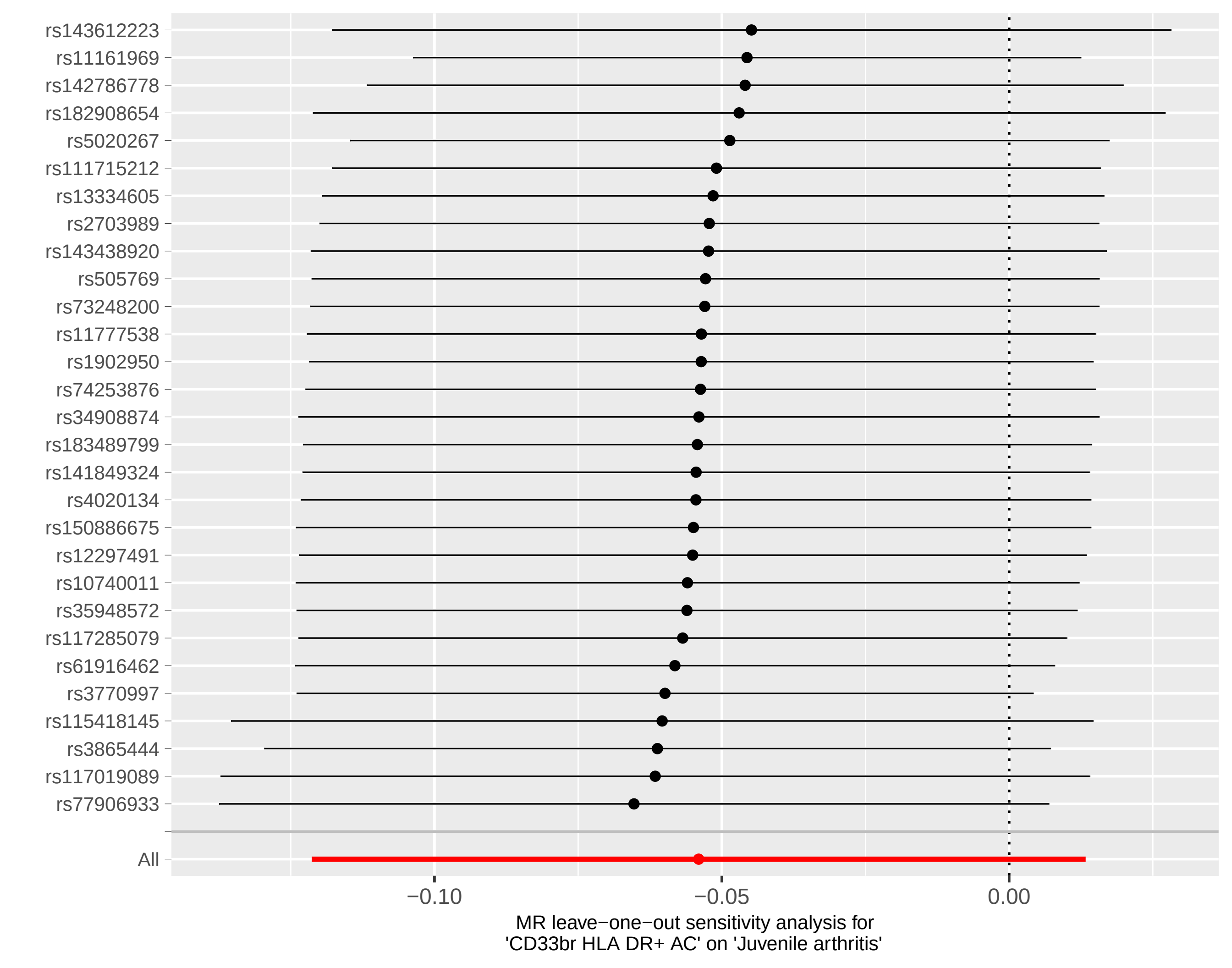

rs143612223
rs11161969
rs142786778
rs182908654
rs5020267
rs111715212
rs13334605
rs2703989
rs143438920
rs505769
rs73248200
rs11777538
rs1902950
rs74253876
rs34908874
rs183489799
rs141849324
rs4020134
rs150886675
rs12297491
rs10740011
rs35948572
rs117285079
rs61916462
rs3770997
rs115418145
rs3865444
rs117019089
rs77906933
All
−0.10
−0.05
0.00
MR leave−one−out sensitivity analysis for
'CD33br HLA DR+ AC' on 'Juvenile arthritis'

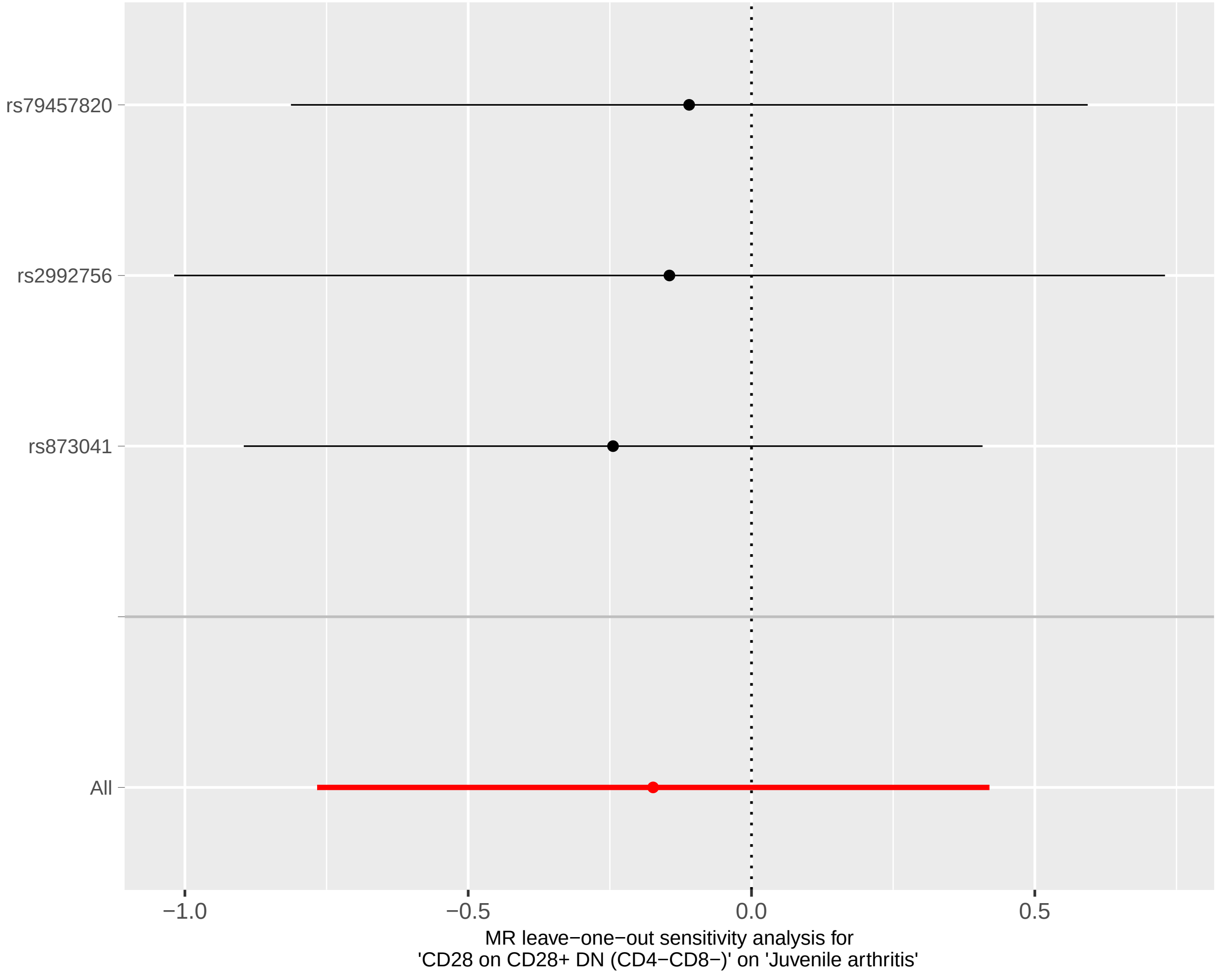
rs79457820
rs2992756
rs873041
All
−1.0
−0.5
0.0
0.5
MR leave−one−out sensitivity analysis for
'CD28 on CD28+ DN (CD4−CD8−)' on 'Juvenile arthritis'

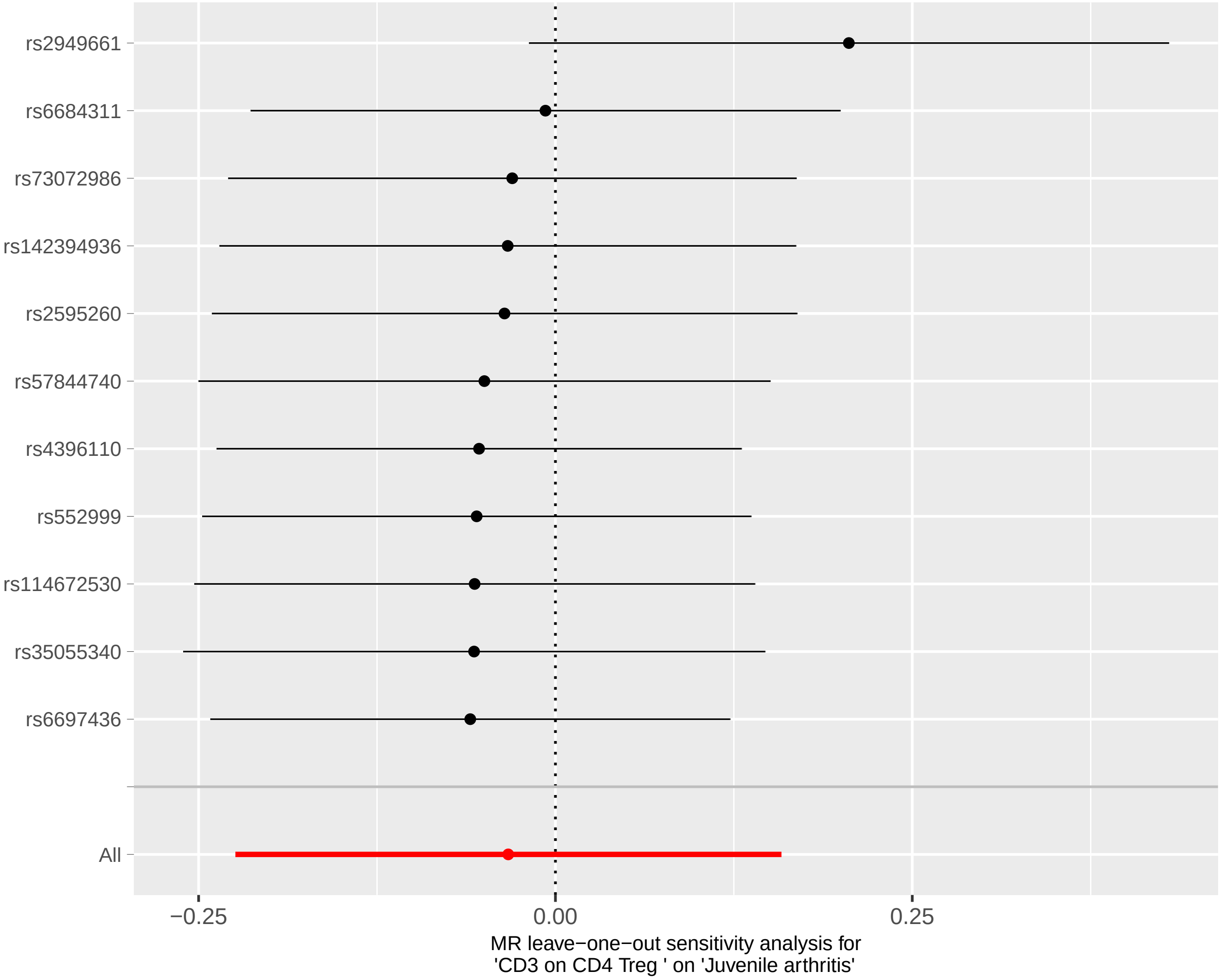
rs2949661
rs6684311
rs73072986
rs142394936
rs2595260
rs57844740
rs4396110
rs552999
rs114672530
rs35055340
rs6697436
All
−0.25
0.00
0.25
MR leave−one−out sensitivity analysis for
'CD3 on CD4 Treg ' on 'Juvenile arthritis'

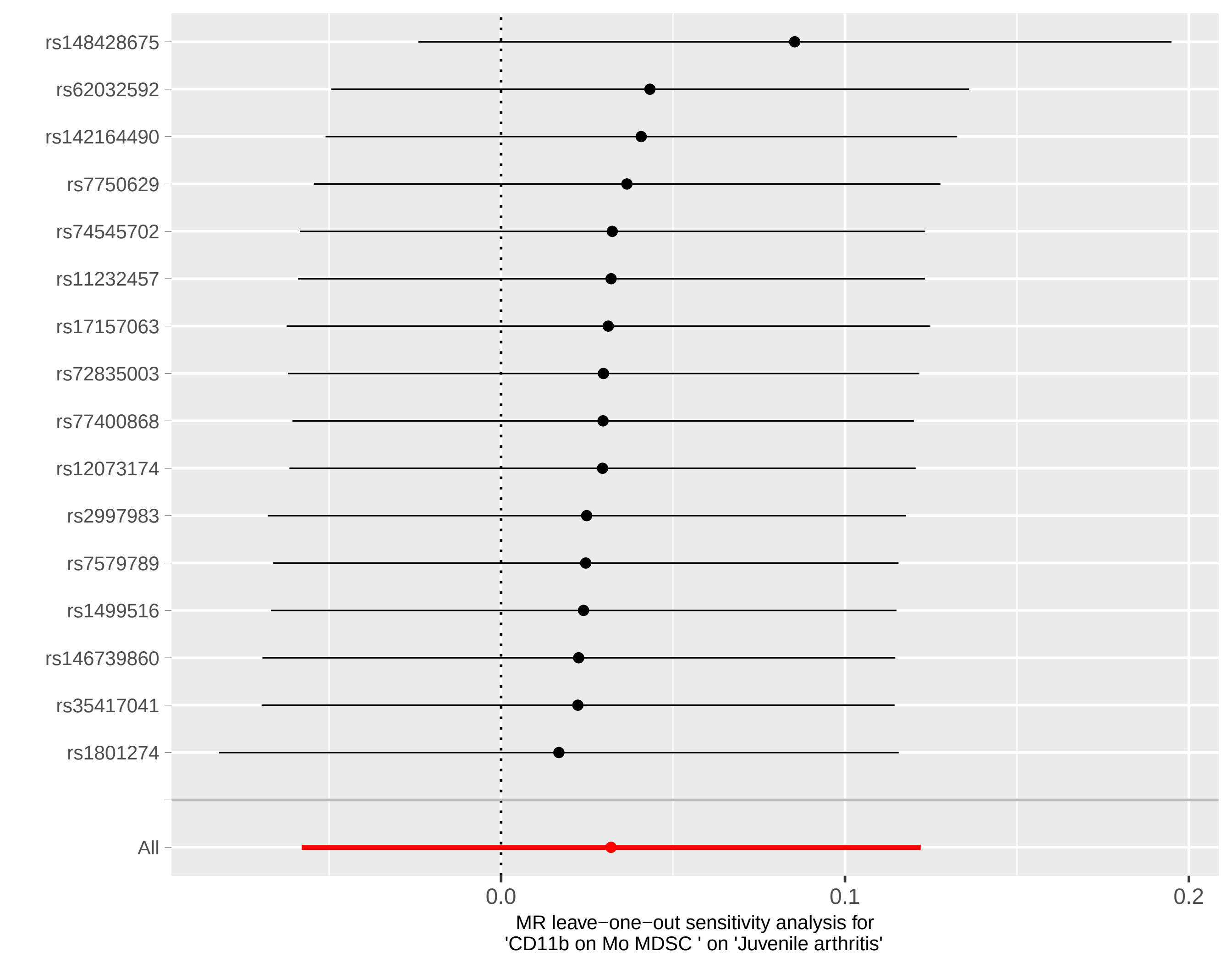

rs148428675
rs62032592
rs142164490
rs7750629
rs74545702
rs11232457
rs17157063
rs72835003
rs77400868
rs12073174
rs2997983
rs7579789
rs1499516
rs146739860
rs35417041
rs1801274
All
0.0
0.1
0.2
MR leave−one−out sensitivity analysis for
'CD11b on Mo MDSC ' on 'Juvenile arthritis'

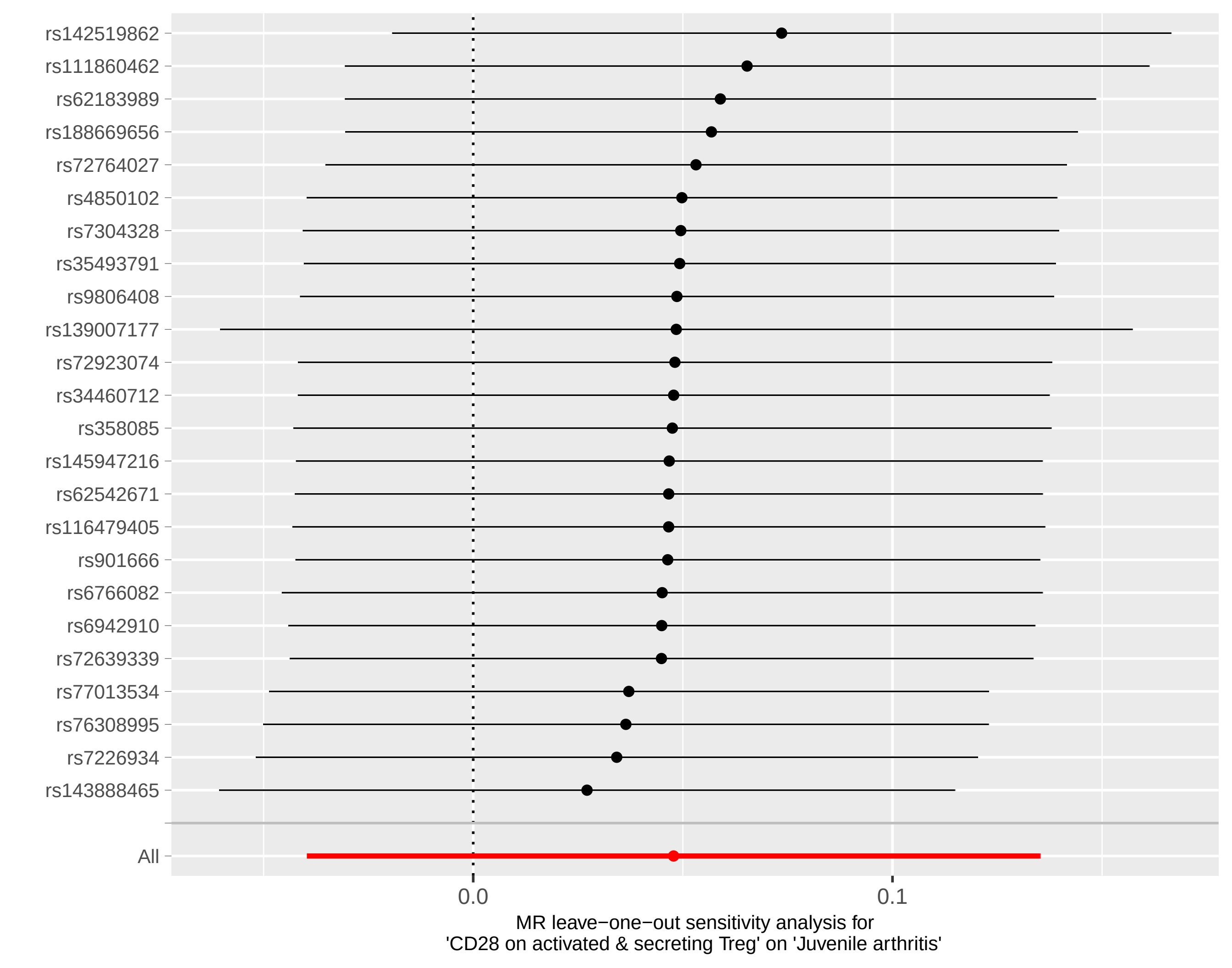

rs142519862
rs111860462
rs62183989
rs188669656
rs72764027
rs4850102
rs7304328
rs35493791
rs9806408
rs139007177
rs72923074
rs34460712
rs358085
rs145947216
rs62542671
rs116479405
rs901666
rs6766082
rs6942910
rs72639339
rs77013534
rs76308995
rs7226934
rs143888465
All
0.0
0.1
MR leave−one−out sensitivity analysis for
'CD28 on activated & secreting Treg' on 'Juvenile arthritis'

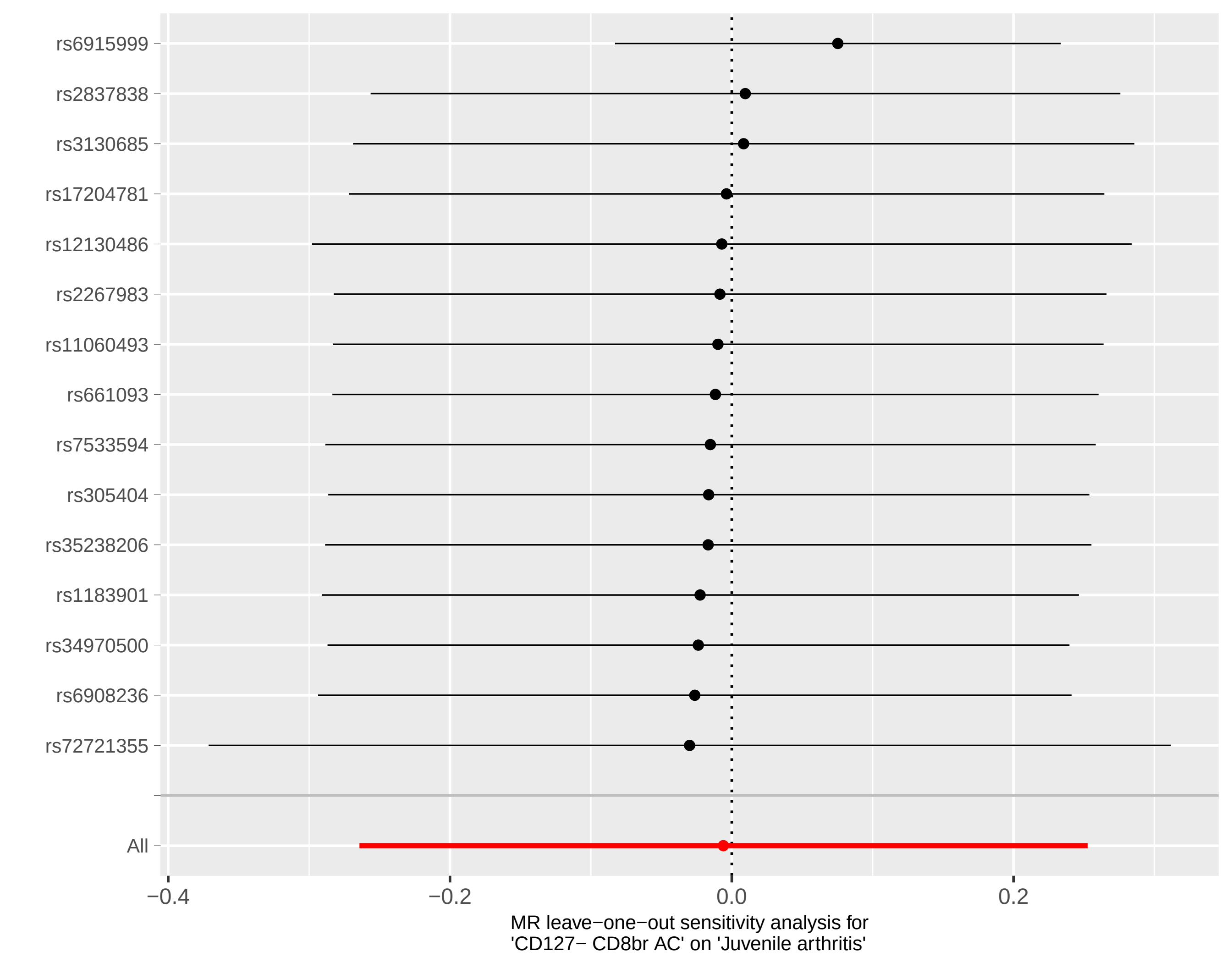
rs6915999
rs2837838
rs3130685
rs17204781
rs12130486
rs2267983
rs11060493
rs661093
rs7533594
rs305404
rs35238206
rs1183901
rs34970500
rs6908236
rs72721355
All
−0.4
−0.2
0.0
0.2
MR leave−one−out sensitivity analysis for
'CD127− CD8br AC' on 'Juvenile arthritis'

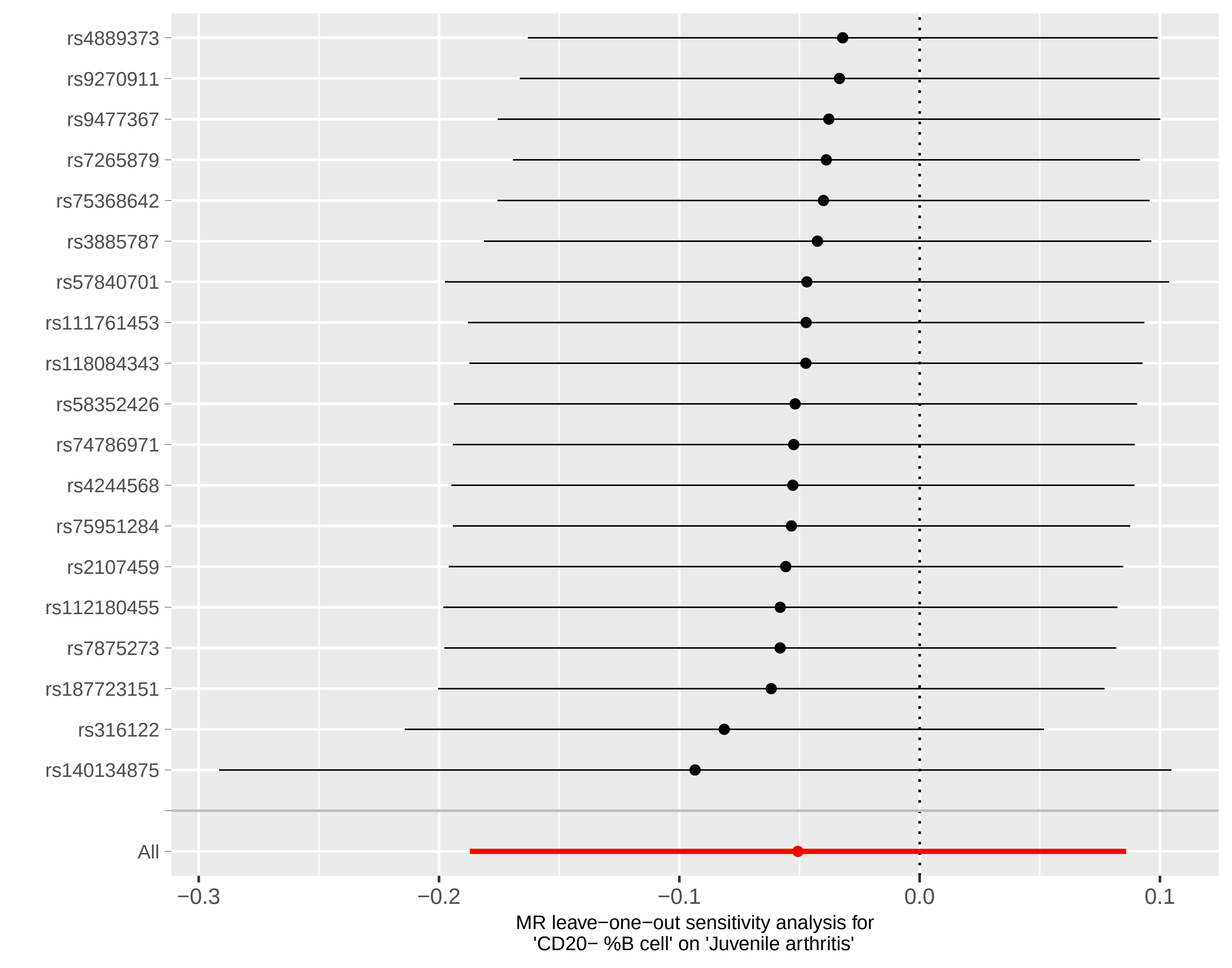
rs4889373
rs9270911
rs9477367
rs7265879
rs75368642
rs3885787
rs57840701
rs111761453
rs118084343
rs58352426
rs74786971
rs4244568
rs75951284
rs2107459
rs112180455
rs7875273
rs187723151
rs316122
rs140134875
All
−0.3
−0.2
−0.1
0.0
0.1
MR leave−one−out sensitivity analysis for
'CD20− %B cell' on 'Juvenile arthritis'

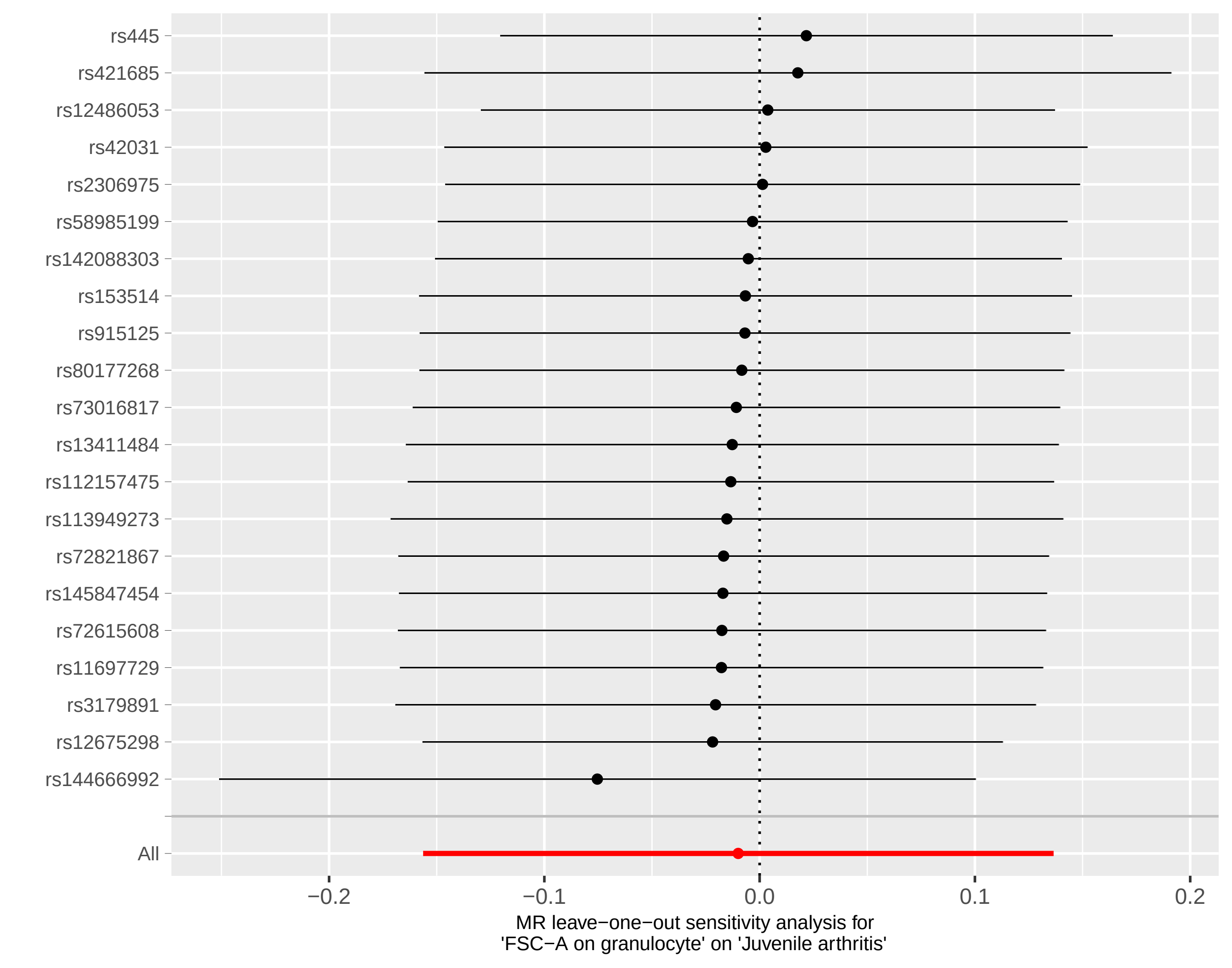
rs445
rs421685
rs12486053
rs42031
rs2306975
rs58985199
rs142088303
rs153514
rs915125
rs80177268
rs73016817
rs13411484
rs112157475
rs113949273
rs72821867
rs145847454
rs72615608
rs11697729
rs3179891
rs12675298
rs144666992
All
−0.2
−0.1
0.0
0.1
0.2
MR leave−one−out sensitivity analysis for
'FSC−A on granulocyte' on 'Juvenile arthritis'

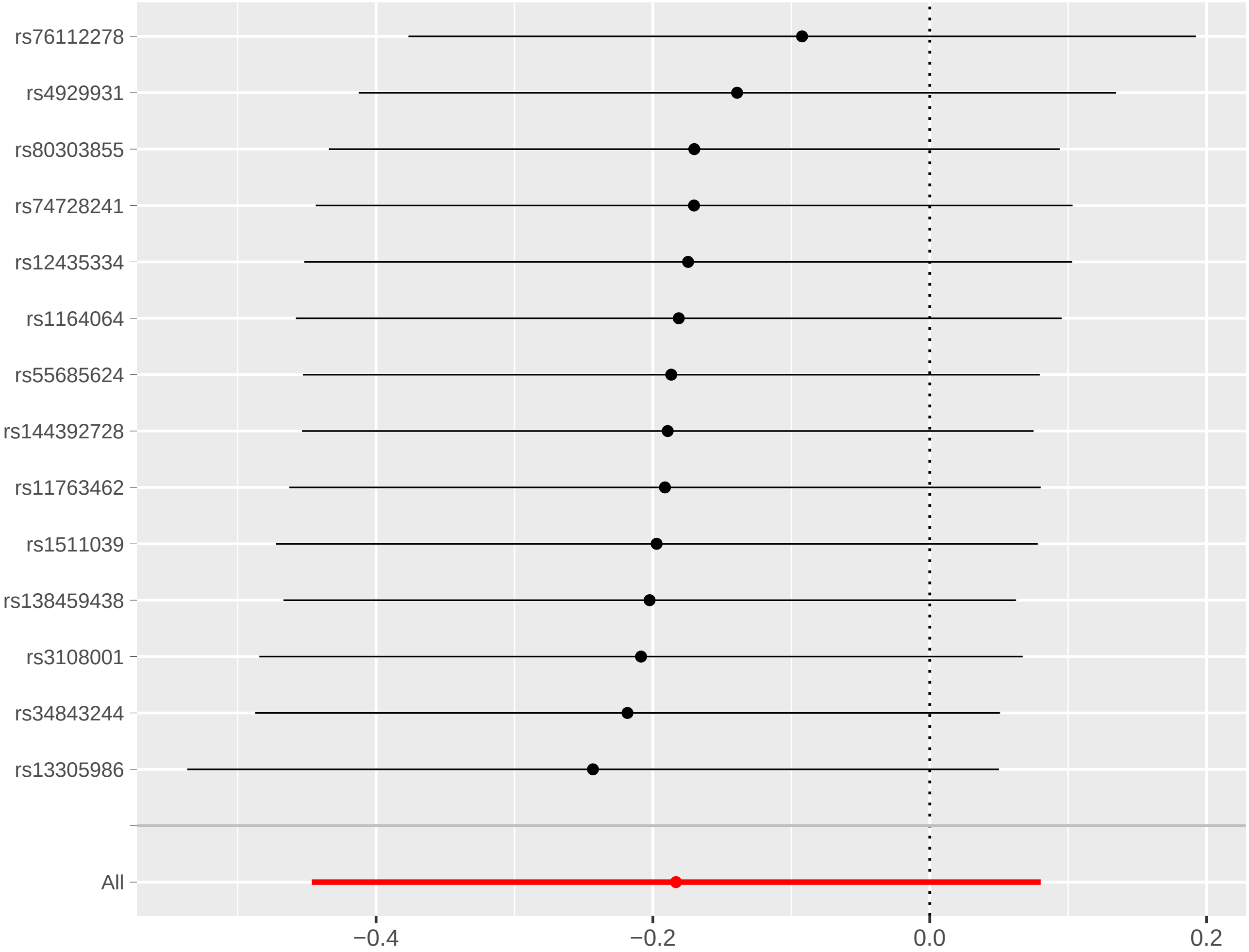

MR leave−one−out sensitivity analysis for
'CD38 on IgD+' on 'Juvenile arthritis'

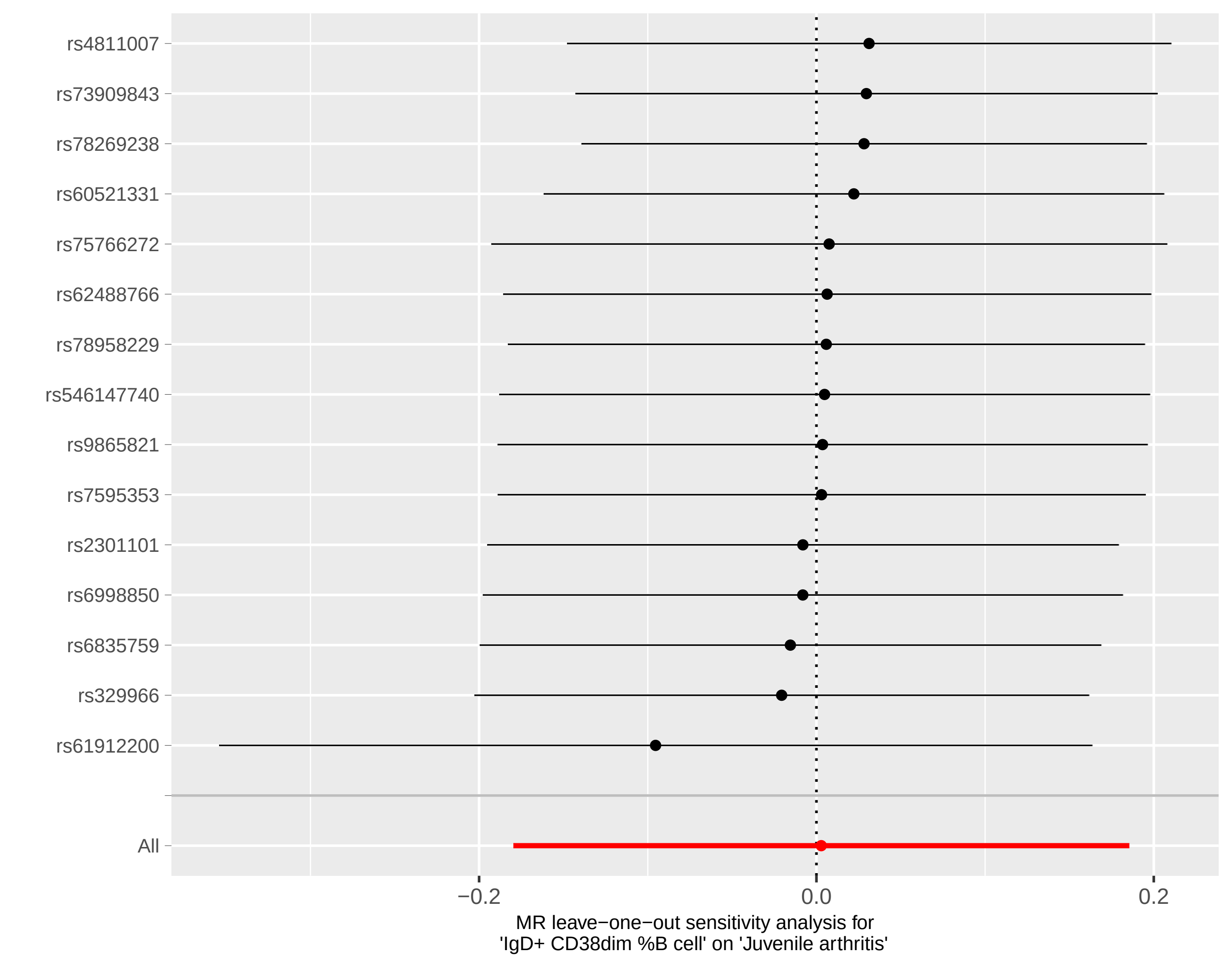
rs4811007
rs73909843
rs78269238
rs60521331
rs75766272
rs62488766
rs78958229
rs546147740
rs9865821
rs7595353
rs2301101
rs6998850
rs6835759
rs329966
rs61912200
All
−0.2
0.0
0.2
MR leave−one−out sensitivity analysis for
'IgD+ CD38dim %B cell' on 'Juvenile arthritis'

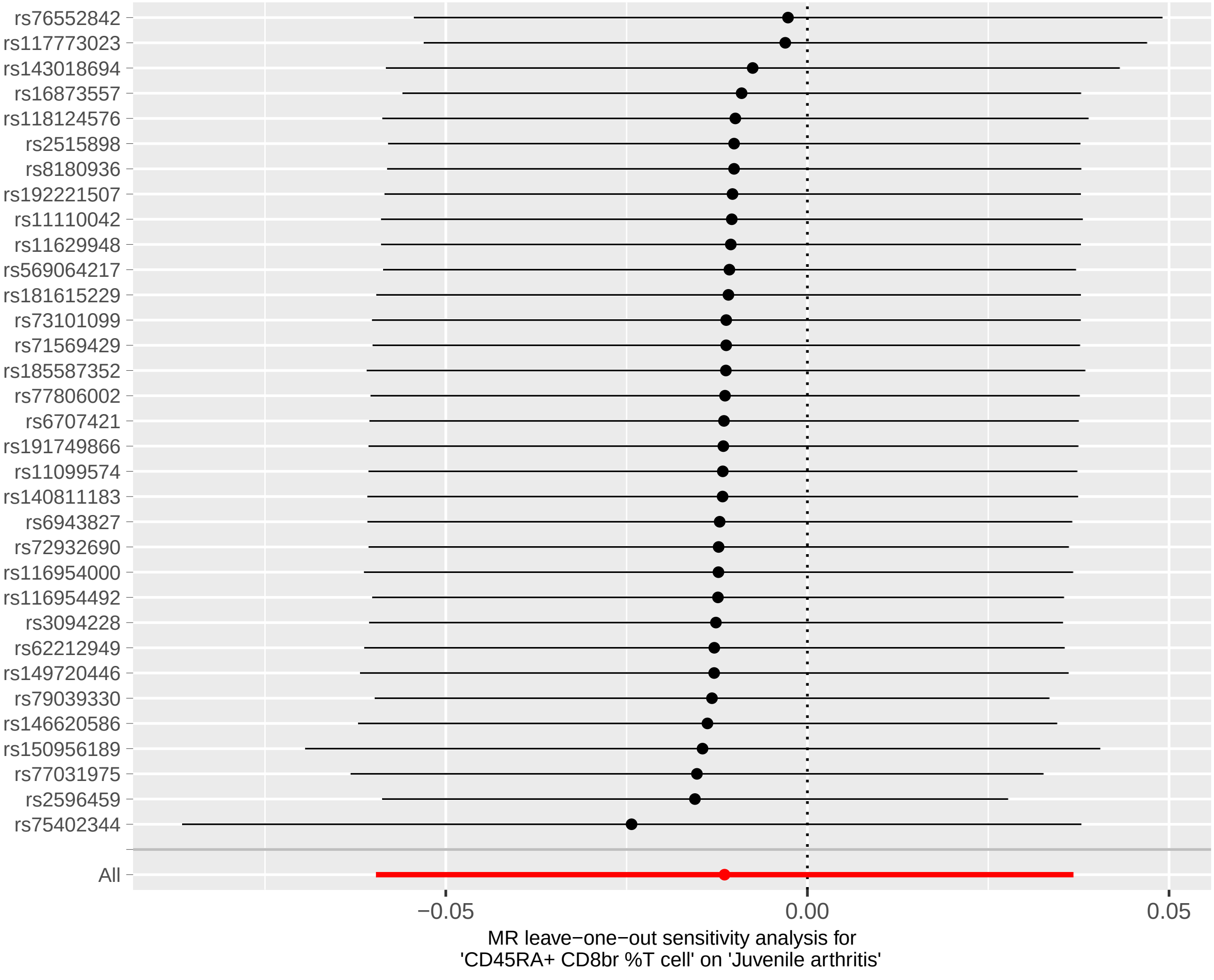

rs76552842
rs117773023
rs143018694
rs16873557
rs118124576
rs2515898
rs8180936
rs192221507
rs11110042
rs11629948
rs569064217
rs181615229
rs73101099
rs71569429
rs185587352
rs77806002
rs6707421
rs191749866
rs11099574
rs140811183
rs6943827
rs72932690
rs116954000
rs116954492
rs3094228
rs62212949
rs149720446
rs79039330
rs146620586
rs150956189
rs77031975
rs2596459
rs75402344
All
−0.05
0.00
0.05
MR leave−one−out sensitivity analysis for
'CD45RA+ CD8br %T cell' on 'Juvenile arthritis'

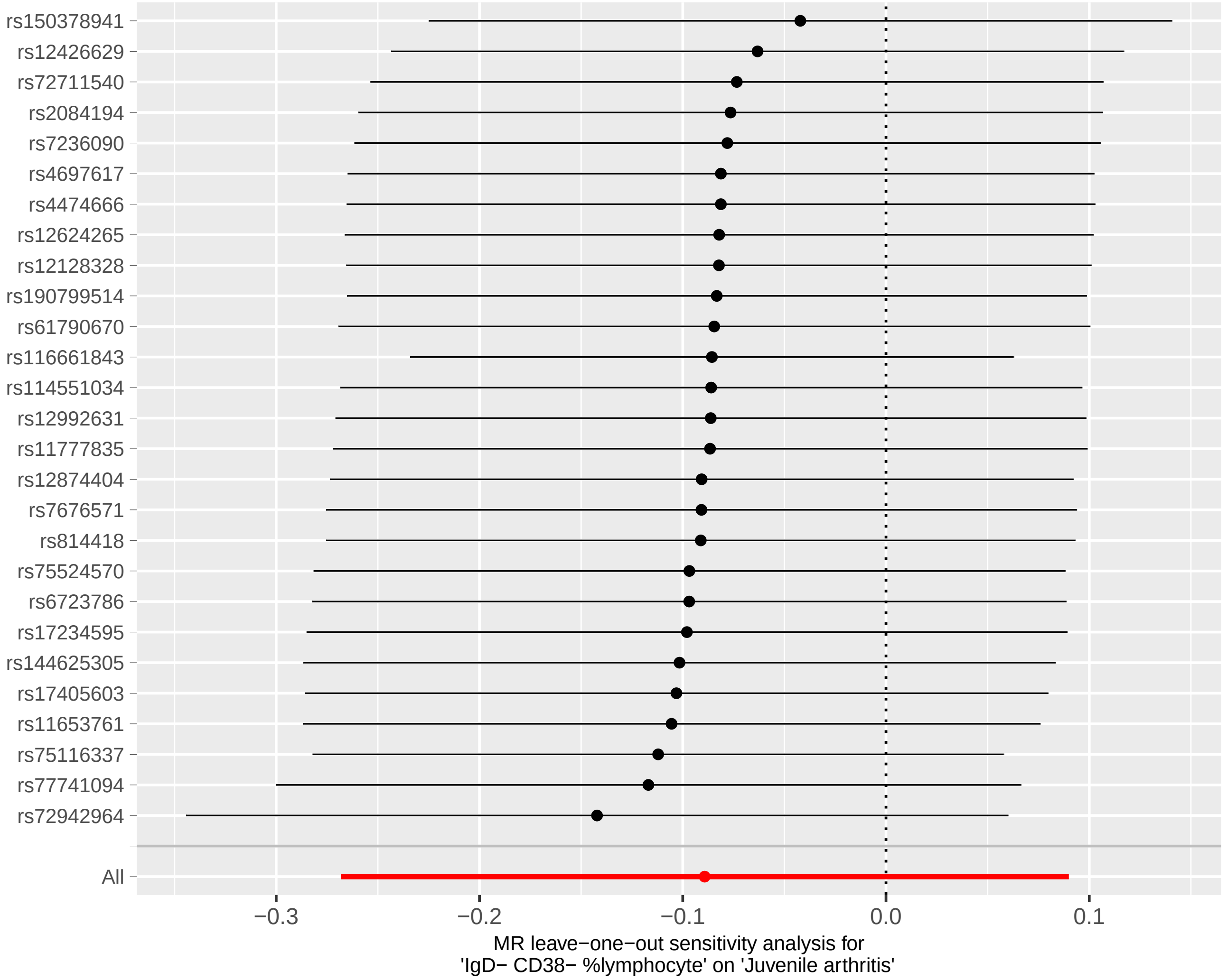
rs150378941
rs12426629
rs72711540
rs2084194
rs7236090
rs4697617
rs4474666
rs12624265
rs12128328
rs190799514
rs61790670
rs116661843
rs114551034
rs12992631
rs11777835
rs12874404
rs7676571
rs814418
rs75524570
rs6723786
rs17234595
rs144625305
rs17405603
rs11653761
rs75116337
rs77741094
rs72942964
All
−0.3
−0.2
−0.1
0.0
0.1
MR leave−one−out sensitivity analysis for
'IgD− CD38− %lymphocyte' on 'Juvenile arthritis'

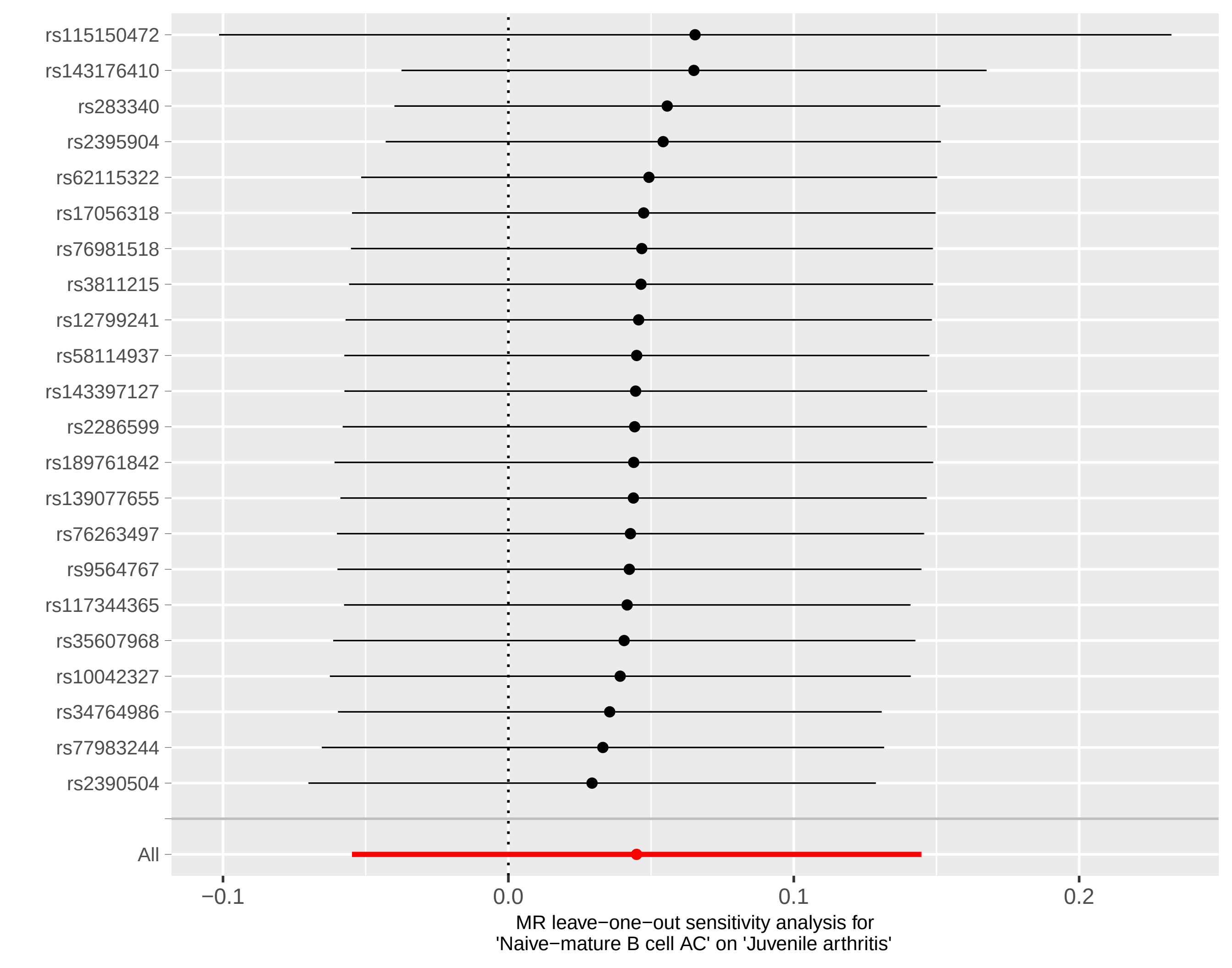
rs115150472
rs143176410
rs283340
rs2395904
rs62115322
rs17056318
rs76981518
rs3811215
rs12799241
rs58114937
rs143397127
rs2286599
rs189761842
rs139077655
rs76263497
rs9564767
rs117344365
rs35607968
rs10042327
rs34764986
rs77983244
rs2390504
All
−0.1
0.0
0.1
0.2
MR leave−one−out sensitivity analysis for
'Naive−mature B cell AC' on 'Juvenile arthritis'

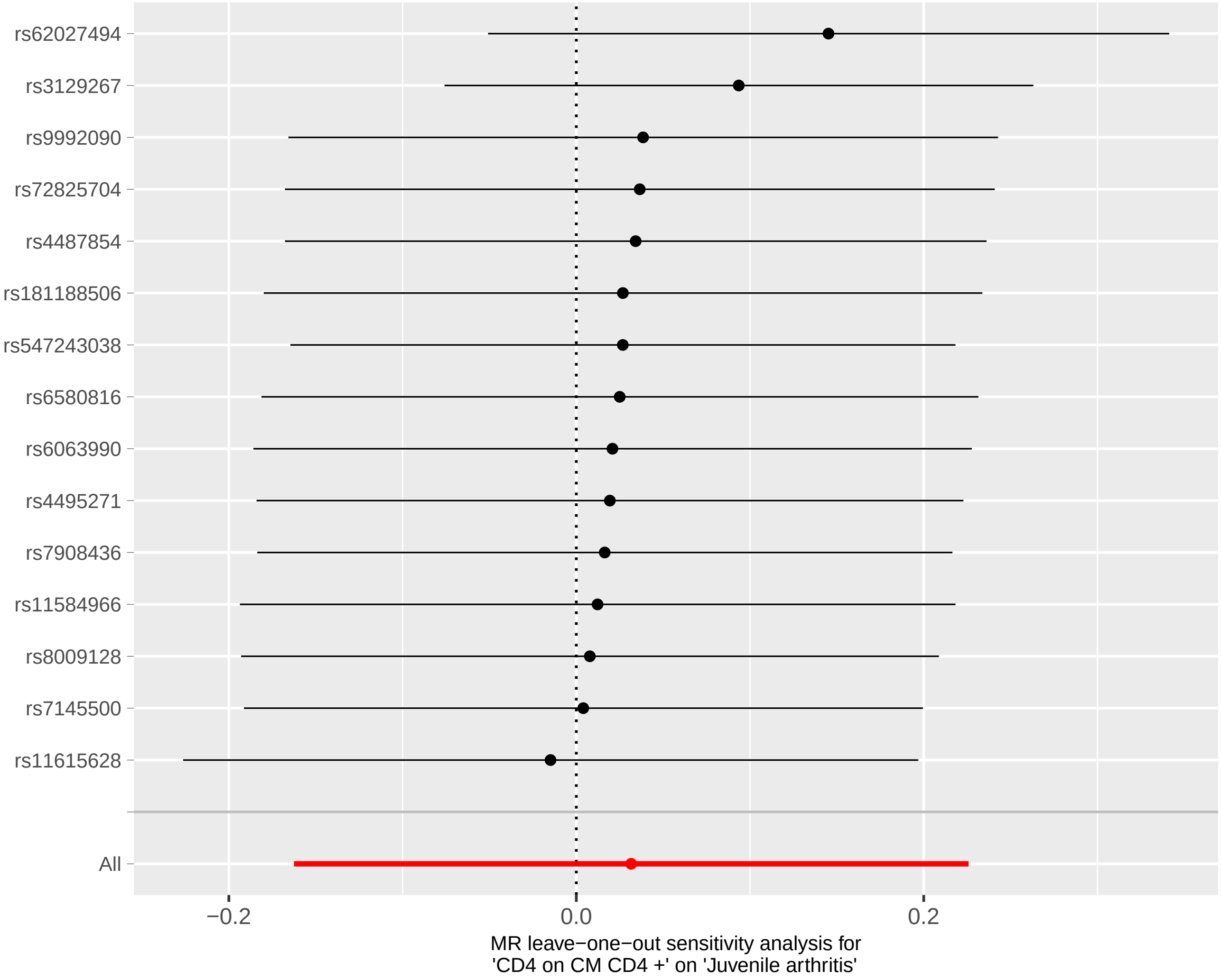

rs62027494
rs3129267
rs9992090
rs72825704
rs4487854
rs181188506
rs547243038
rs6580816
rs6063990
rs4495271
rs7908436
rs11584966
rs8009128
rs7145500
rs11615628
All
−0.2
0.0
0.2
MR leave−one−out sensitivity analysis for
'CD4 on CM CD4 +' on 'Juvenile arthritis'

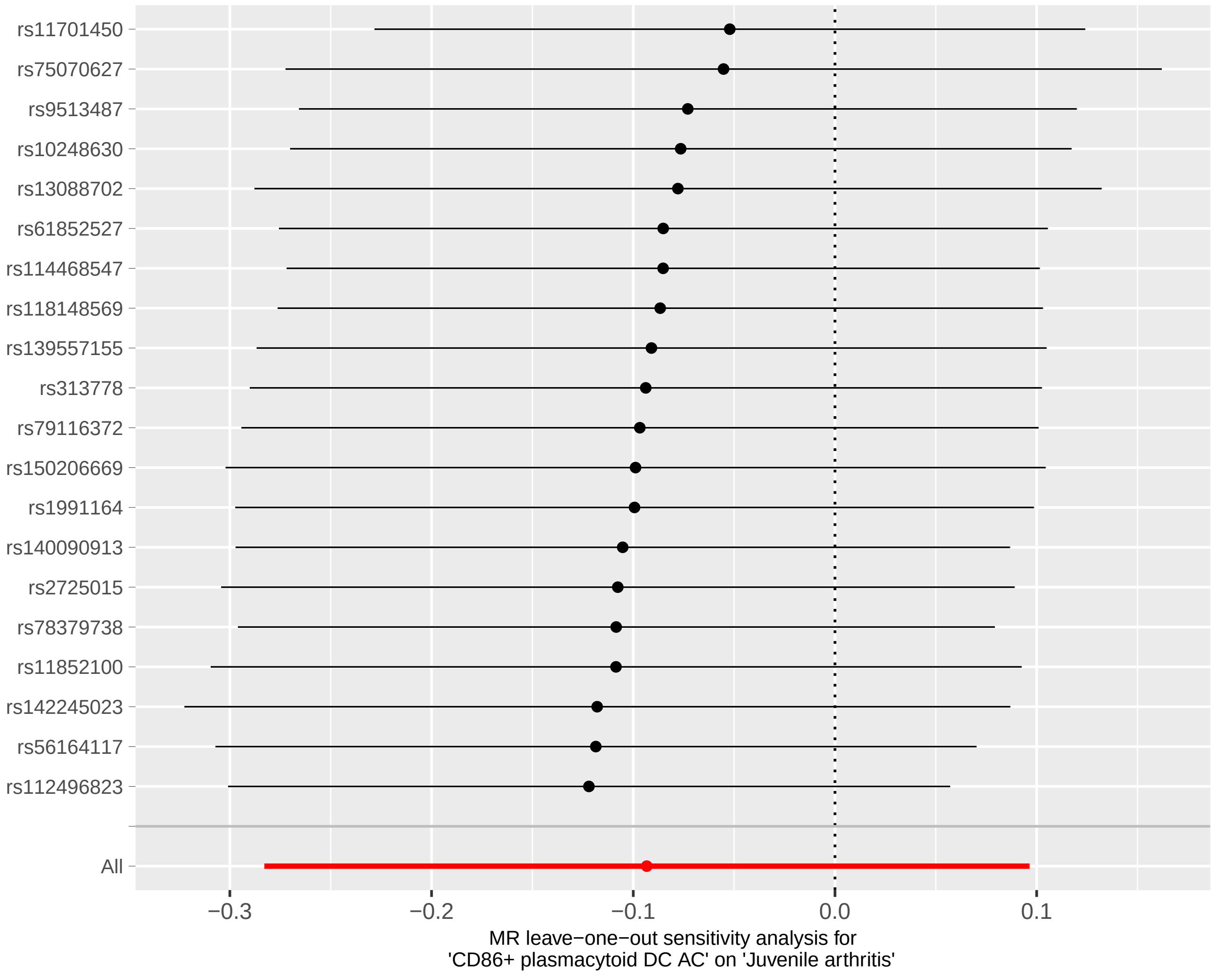

rs11701450
rs75070627
rs9513487
rs10248630
rs13088702
rs61852527
rs114468547
rs118148569
rs139557155
rs313778
rs79116372
rs150206669
rs1991164
rs140090913
rs2725015
rs78379738
rs11852100
rs142245023
rs56164117
rs112496823
All
−0.3
−0.2
−0.1
0.0
0.1
MR leave−one−out sensitivity analysis for
'CD86+ plasmacytoid DC AC' on 'Juvenile arthritis'

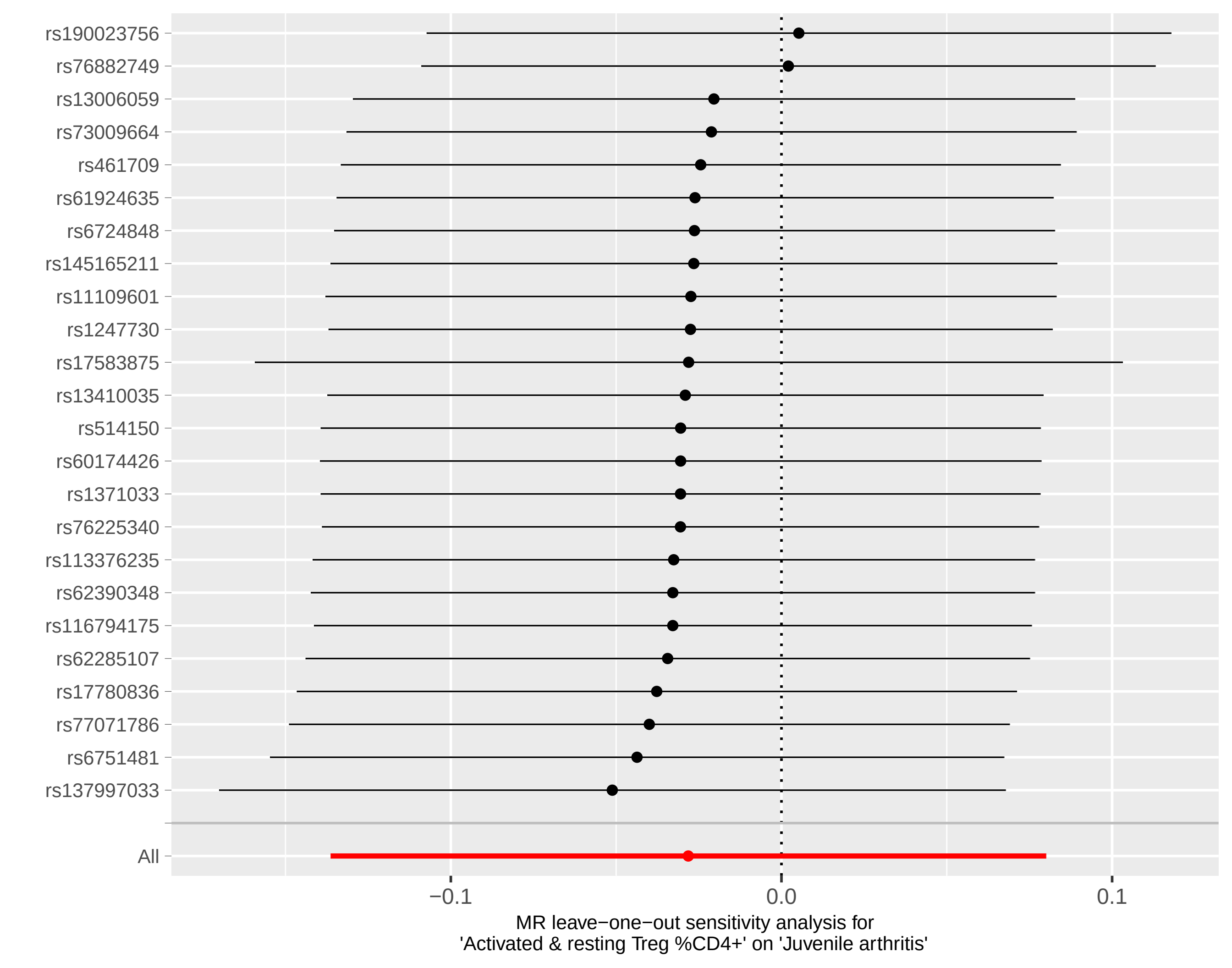

rs190023756
rs76882749
rs13006059
rs73009664
rs461709
rs61924635
rs6724848
rs145165211
rs11109601
rs1247730
rs17583875
rs13410035
rs514150
rs60174426
rs1371033
rs76225340
rs113376235
rs62390348
rs116794175
rs62285107
rs17780836
rs77071786
rs6751481
rs137997033
All
−0.1
0.0
0.1
MR leave−one−out sensitivity analysis for
'Activated & resting Treg %CD4+' on 'Juvenile arthritis'

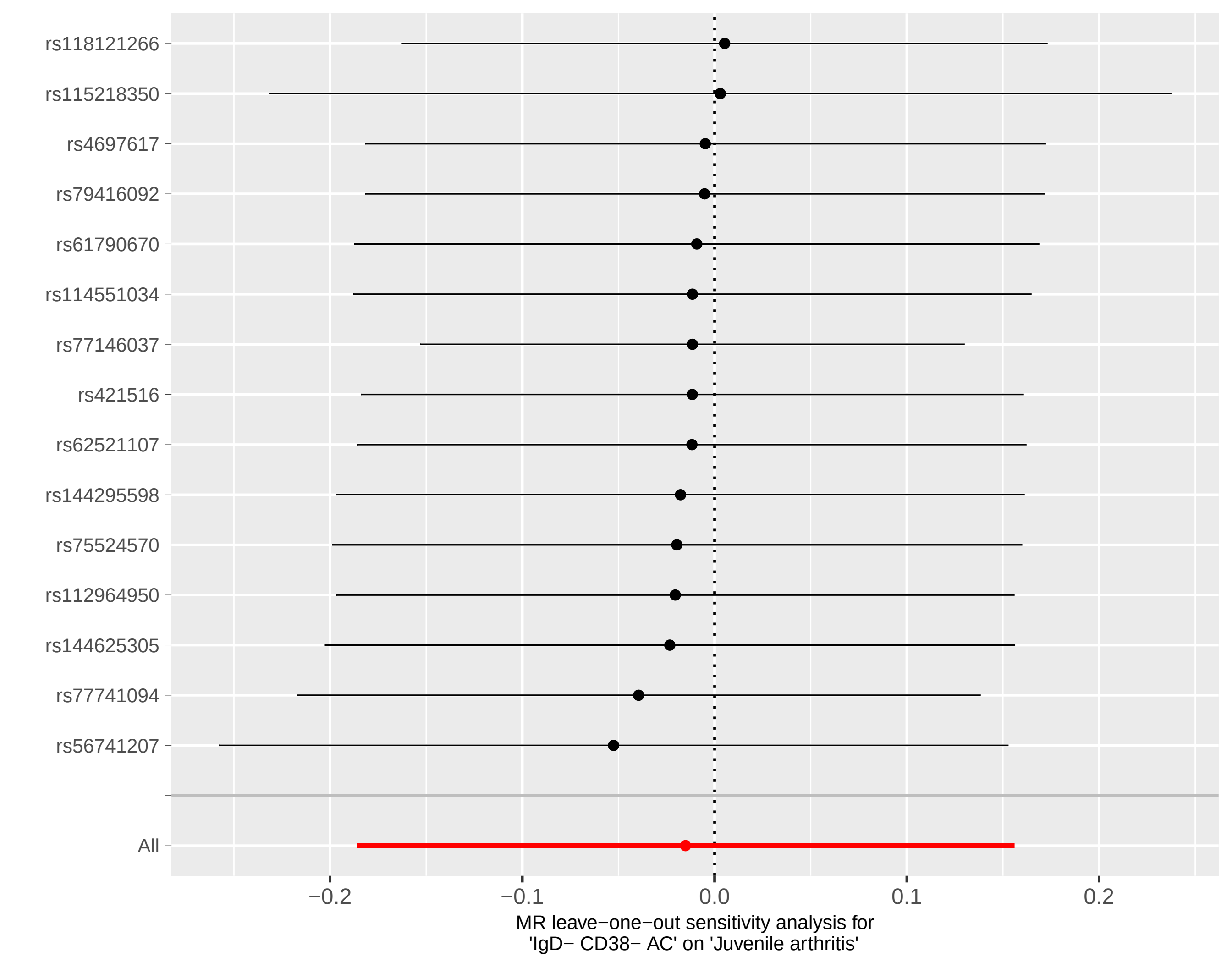
rs118121266
rs115218350
rs4697617
rs79416092
rs61790670
rs114551034
rs77146037
rs421516
rs62521107
rs144295598
rs75524570
rs112964950
rs144625305
rs77741094
rs56741207
All
−0.2
−0.1
0.0
0.1
0.2
MR leave−one−out sensitivity analysis for
'IgD− CD38− AC' on 'Juvenile arthritis'

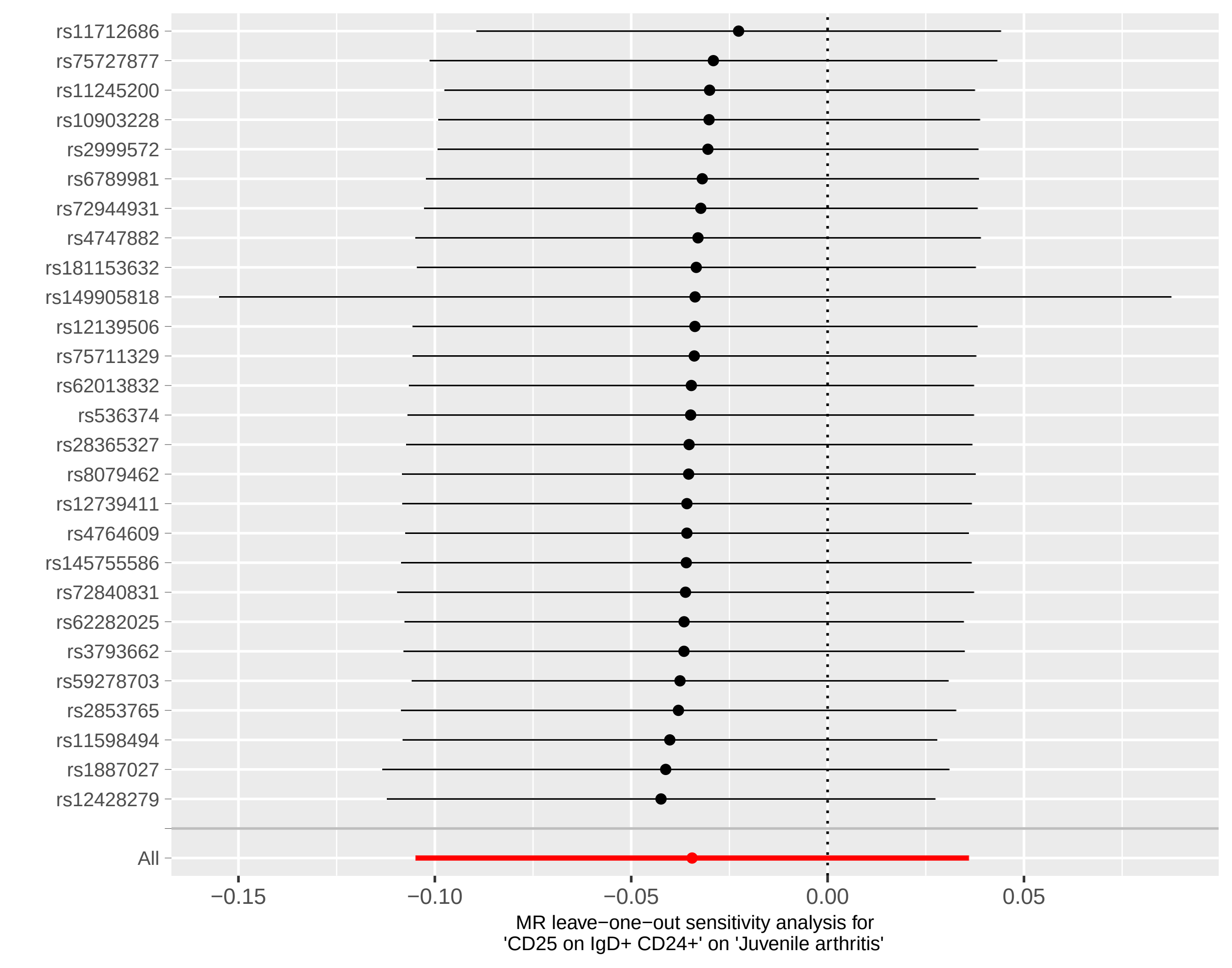
rs11712686
rs75727877
rs11245200
rs10903228
rs2999572
rs6789981
rs72944931
rs4747882
rs181153632
rs149905818
rs12139506
rs75711329
rs62013832
rs536374
rs28365327
rs8079462
rs12739411
rs4764609
rs145755586
rs72840831
rs62282025
rs3793662
rs59278703
rs2853765
rs11598494
rs1887027
rs12428279
All
−0.15
−0.10
−0.05
0.00
0.05
MR leave−one−out sensitivity analysis for
'CD25 on IgD+ CD24+' on 'Juvenile arthritis'

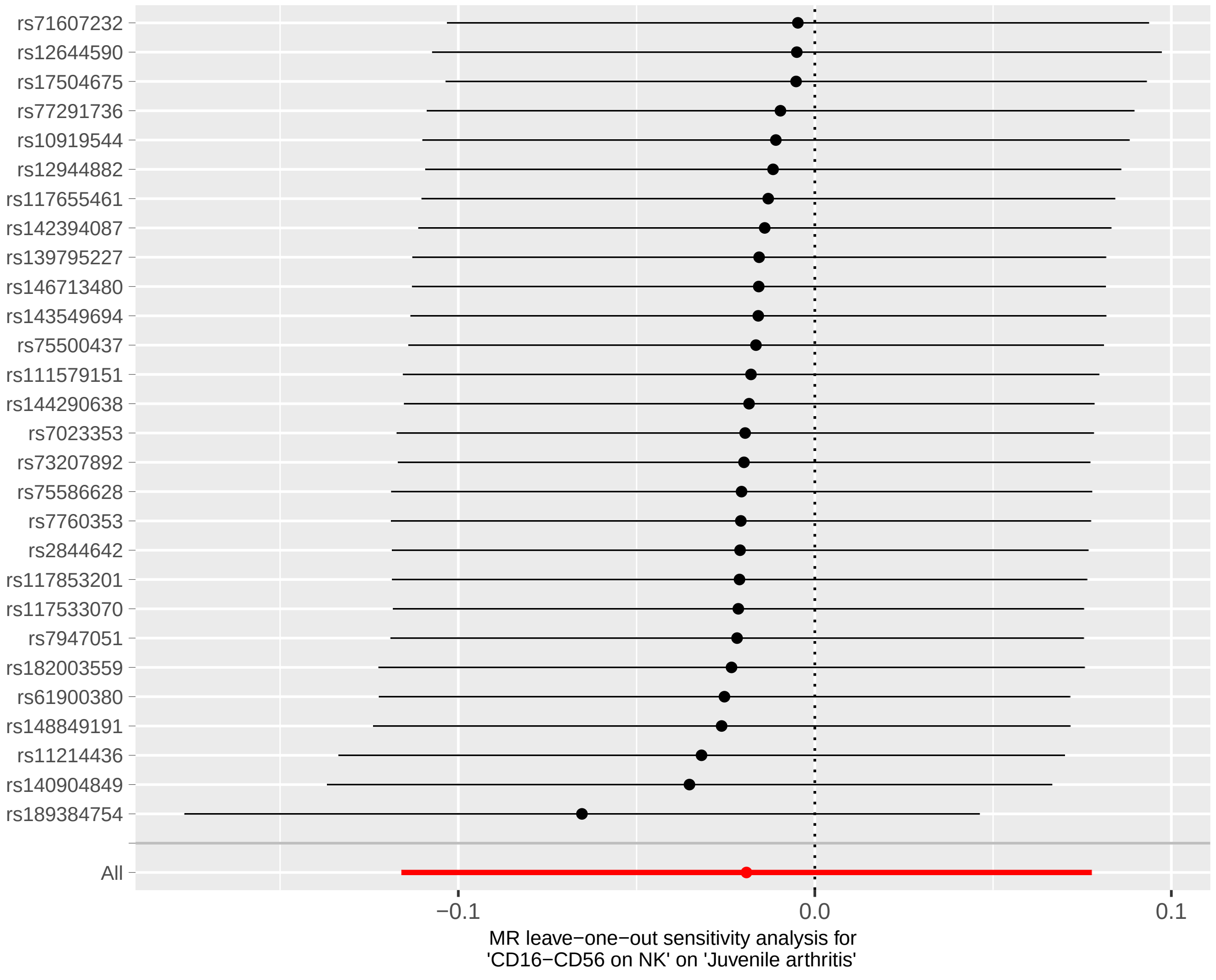

rs71607232
rs12644590
rs17504675
rs77291736
rs10919544
rs12944882
rs117655461
rs142394087
rs139795227
rs146713480
rs143549694
rs75500437
rs111579151
rs144290638
rs7023353
rs73207892
rs75586628
rs7760353
rs2844642
rs117853201
rs117533070
rs7947051
rs182003559
rs61900380
rs148849191
rs11214436
rs140904849
rs189384754
All
−0.1
0.0
0.1
MR leave−one−out sensitivity analysis for
'CD16−CD56 on NK' on 'Juvenile arthritis'

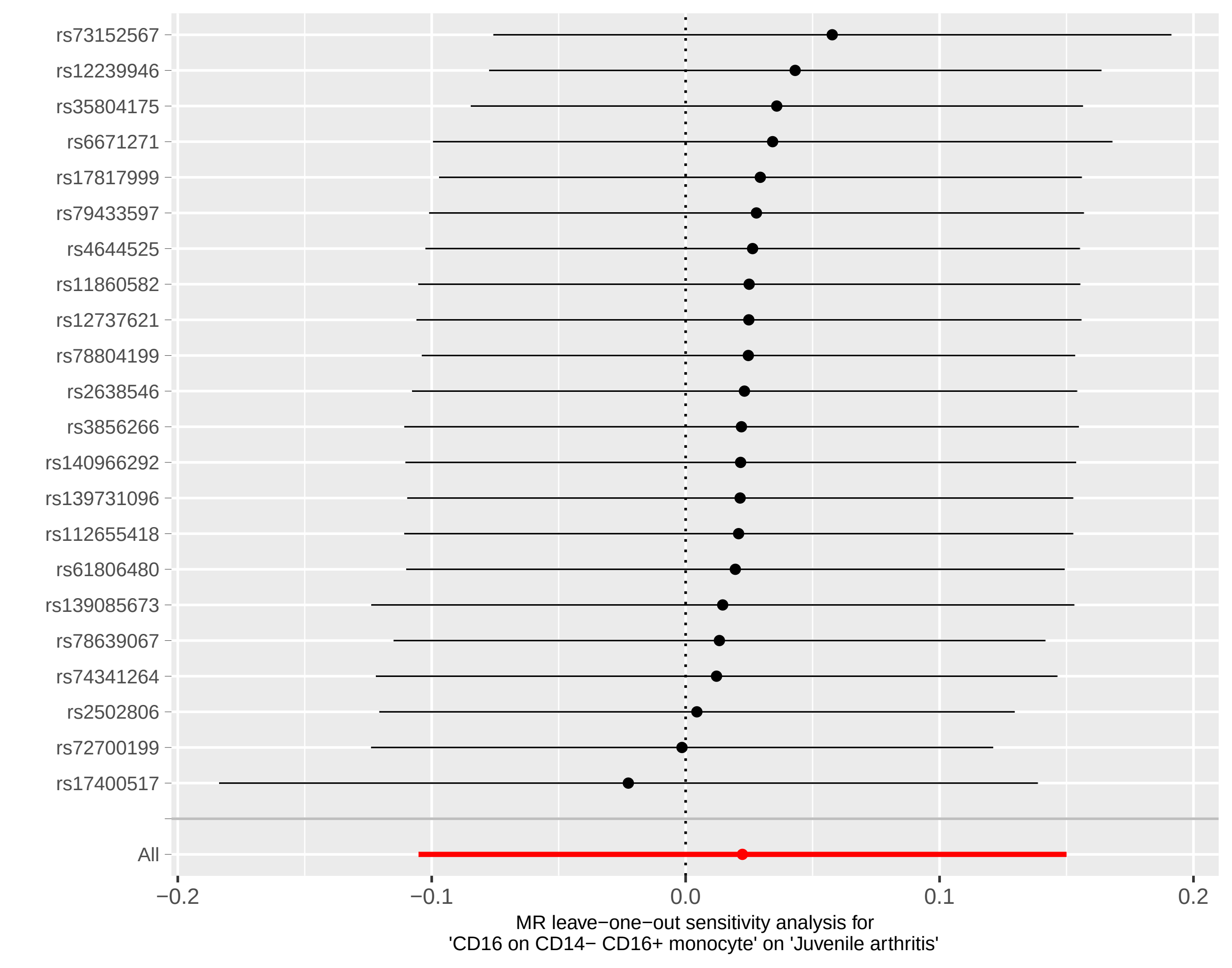
rs73152567
rs12239946
rs35804175
rs6671271
rs17817999
rs79433597
rs4644525
rs11860582
rs12737621
rs78804199
rs2638546
rs3856266
rs140966292
rs139731096
rs112655418
rs61806480
rs139085673
rs78639067
rs74341264
rs2502806
rs72700199
rs17400517
All
−0.2
−0.1
0.0
0.1
0.2
MR leave−one−out sensitivity analysis for
'CD16 on CD14− CD16+ monocyte' on 'Juvenile arthritis'

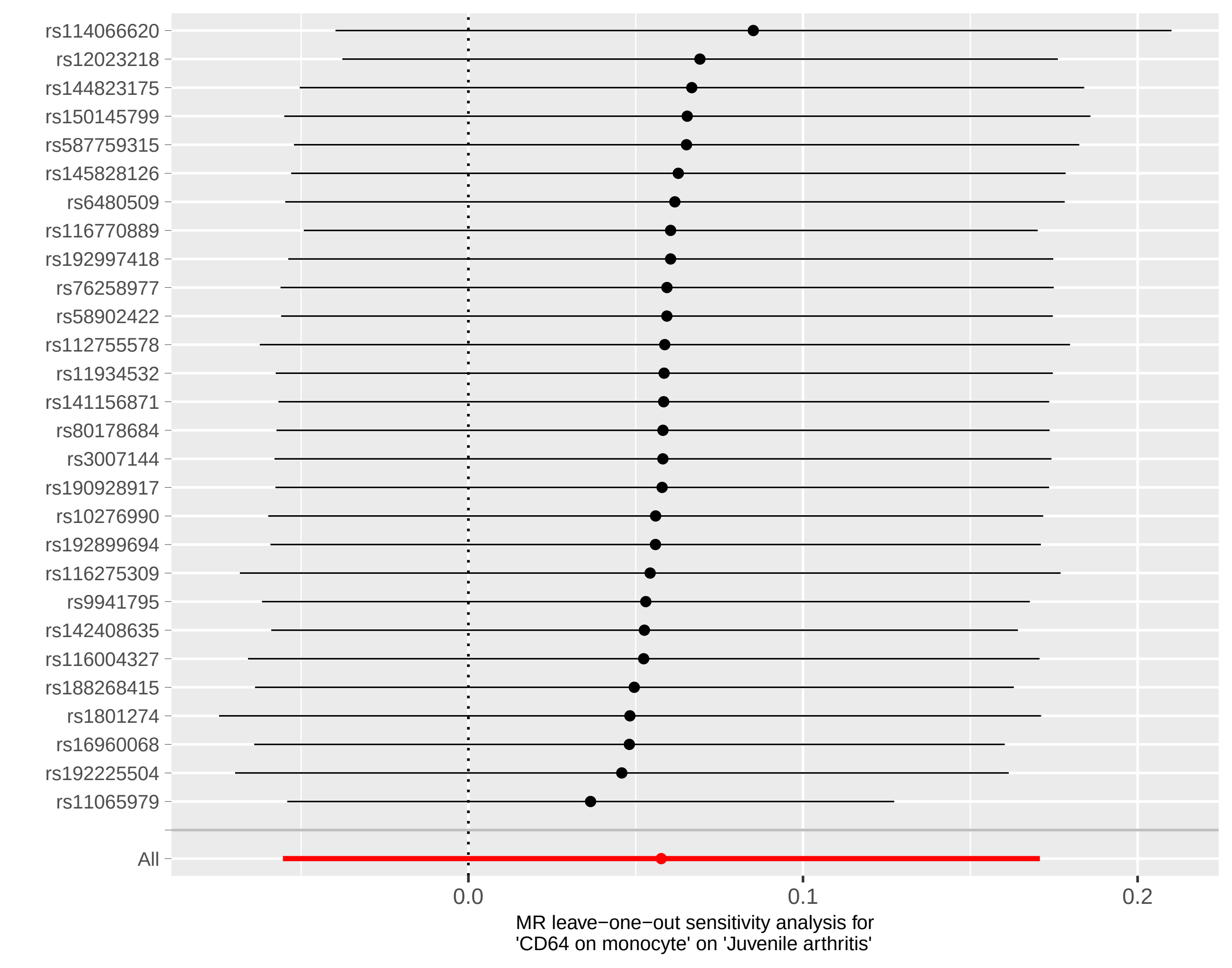
rs114066620
rs12023218
rs144823175
rs150145799
rs587759315
rs145828126
rs6480509
rs116770889
rs192997418
rs76258977
rs58902422
rs112755578
rs11934532
rs141156871
rs80178684
rs3007144
rs190928917
rs10276990
rs192899694
rs116275309
rs9941795
rs142408635
rs116004327
rs188268415
rs1801274
rs16960068
rs192225504
rs11065979
All
0.0
0.1
0.2
MR leave−one−out sensitivity analysis for
'CD64 on monocyte' on 'Juvenile arthritis'

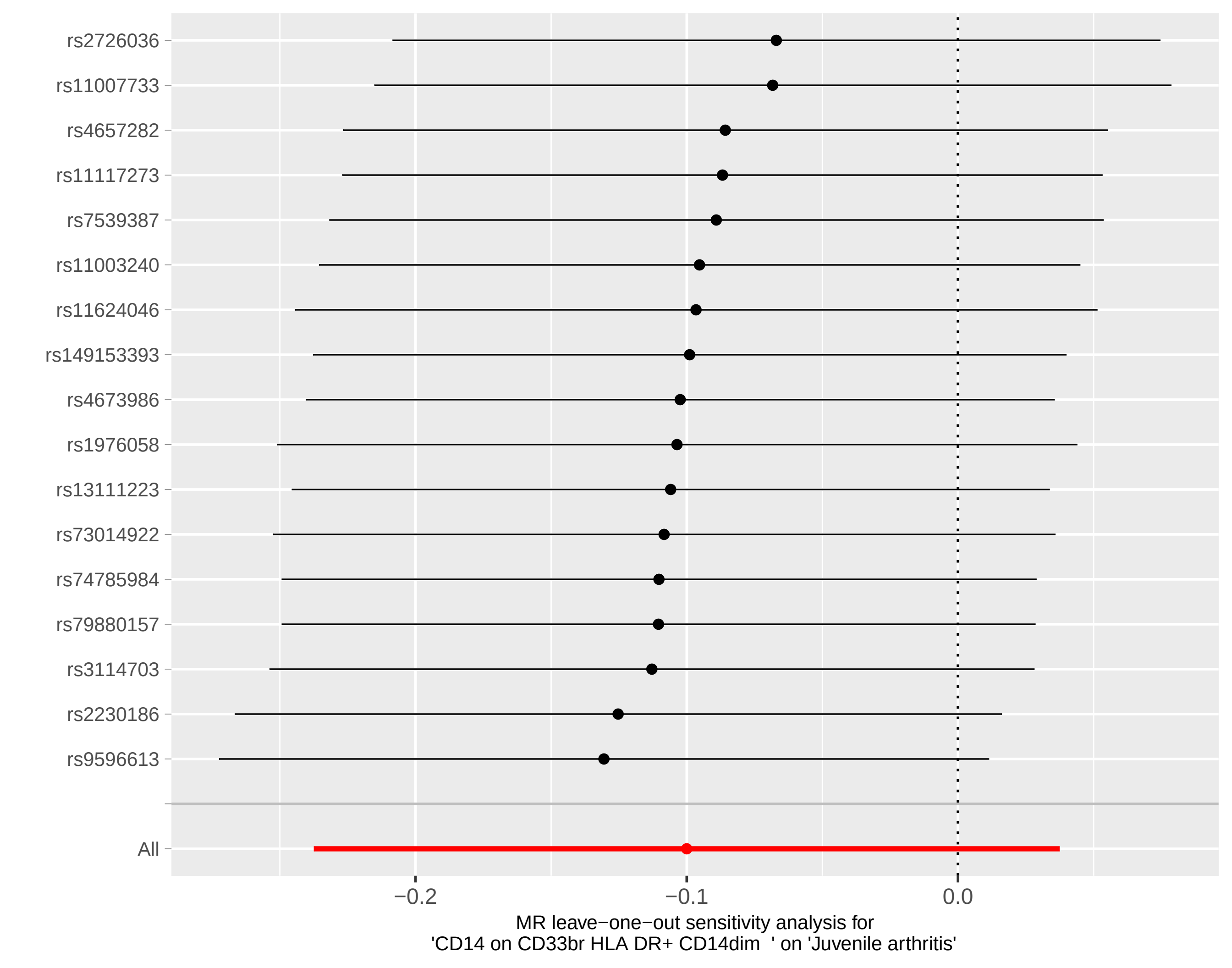

rs2726036
rs11007733
rs4657282
rs11117273
rs7539387
rs11003240
rs11624046
rs149153393
rs4673986
rs1976058
rs13111223
rs73014922
rs74785984
rs79880157
rs3114703
rs2230186
rs9596613
All
−0.2
−0.1
0.0
MR leave−one−out sensitivity analysis for
'CD14 on CD33br HLA DR+ CD14dim ' on 'Juvenile arthritis'

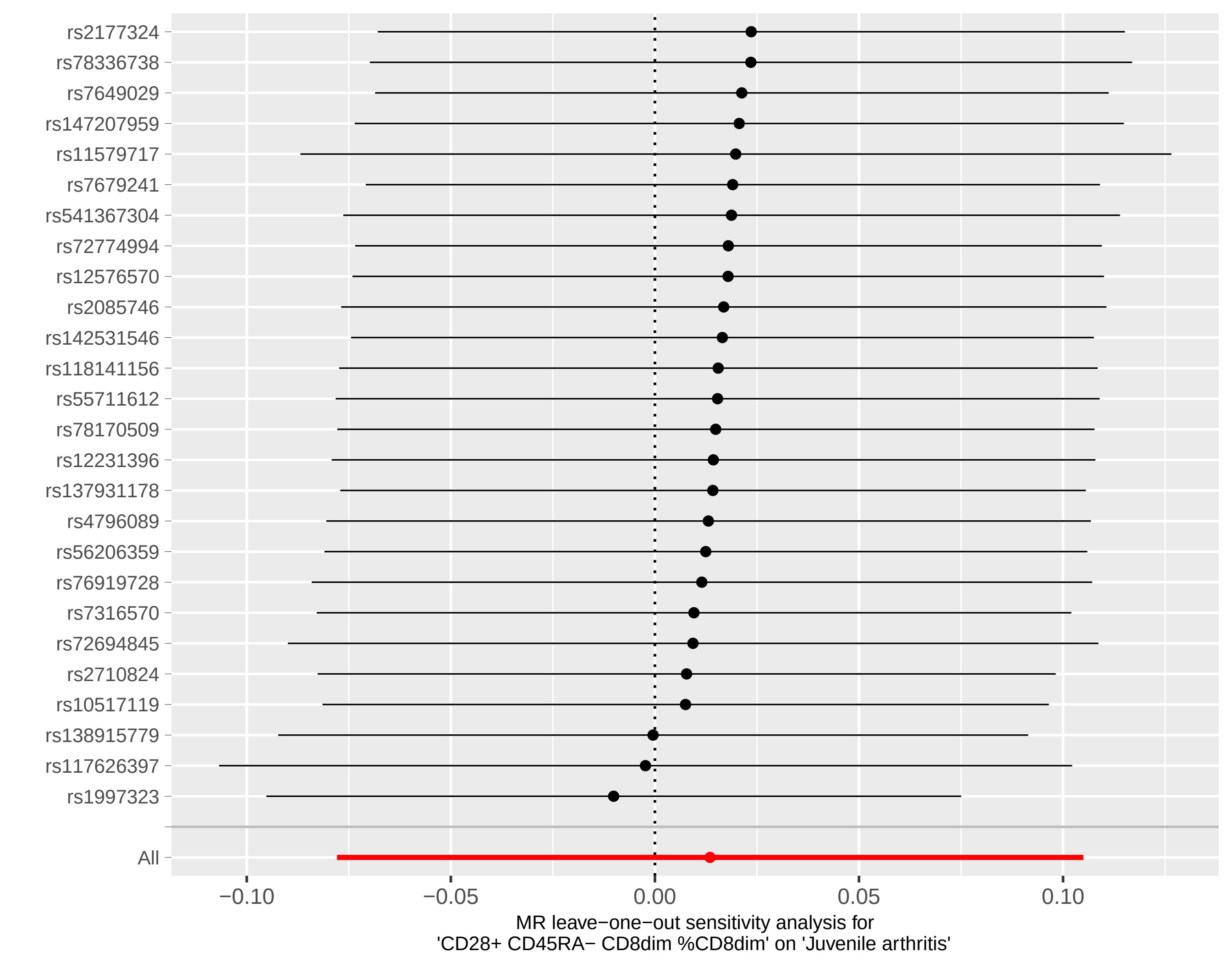

rs2177324
rs78336738
rs7649029
rs147207959
rs11579717
rs7679241
rs541367304
rs72774994
rs12576570
rs2085746
rs142531546
rs118141156
rs55711612
rs78170509
rs12231396
rs137931178
rs4796089
rs56206359
rs76919728
rs7316570
rs72694845
rs2710824
rs10517119
rs138915779
rs117626397
rs1997323
All
−0.10
−0.05
0.00
0.05
0.10
MR leave−one−out sensitivity analysis for
'CD28+ CD45RA− CD8dim %CD8dim' on 'Juvenile arthritis'

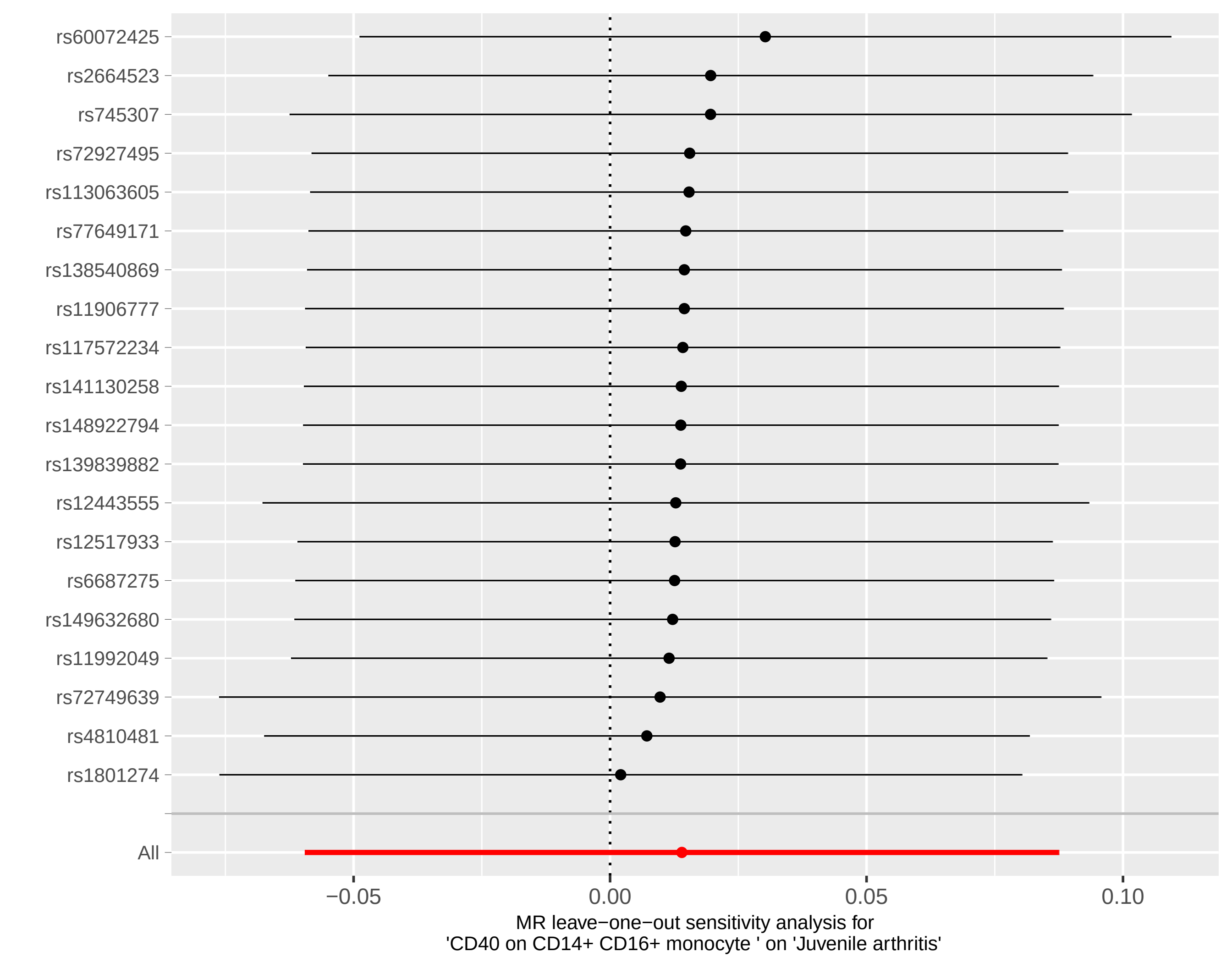
rs60072425
rs2664523
rs745307
rs72927495
rs113063605
rs77649171
rs138540869
rs11906777
rs117572234
rs141130258
rs148922794
rs139839882
rs12443555
rs12517933
rs6687275
rs149632680
rs11992049
rs72749639
rs4810481
rs1801274
All
−0.05
0.00
0.05
0.10
MR leave−one−out sensitivity analysis for
'CD40 on CD14+ CD16+ monocyte ' on 'Juvenile arthritis'

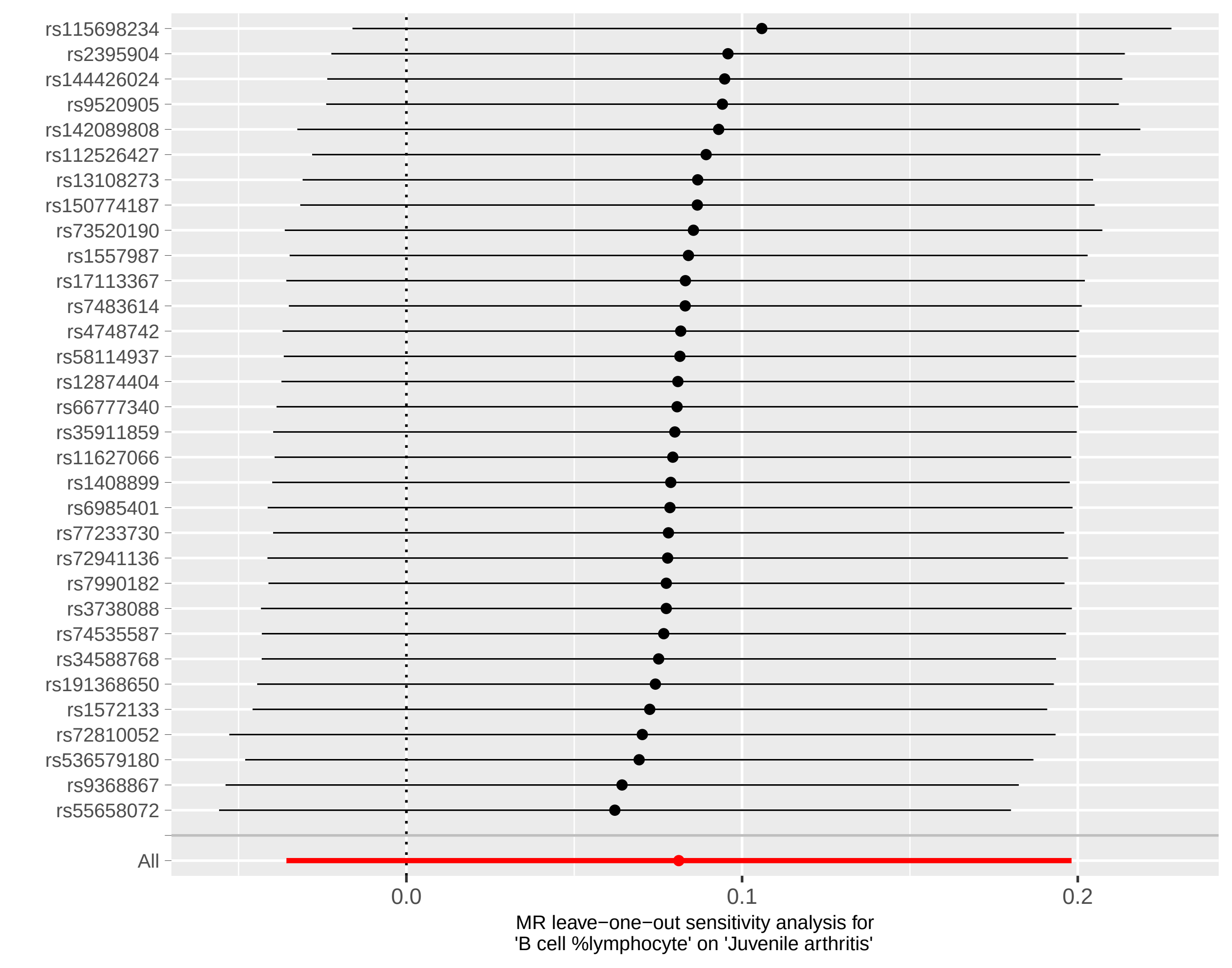
rs115698234
rs2395904
rs144426024
rs9520905
rs142089808
rs112526427
rs13108273
rs150774187
rs73520190
rs1557987
rs17113367
rs7483614
rs4748742
rs58114937
rs12874404
rs66777340
rs35911859
rs11627066
rs1408899
rs6985401
rs77233730
rs72941136
rs7990182
rs3738088
rs74535587
rs34588768
rs191368650
rs1572133
rs72810052
rs536579180
rs9368867
rs55658072
All
0.0
0.1
0.2
MR leave−one−out sensitivity analysis for
'B cell %lymphocyte' on 'Juvenile arthritis'

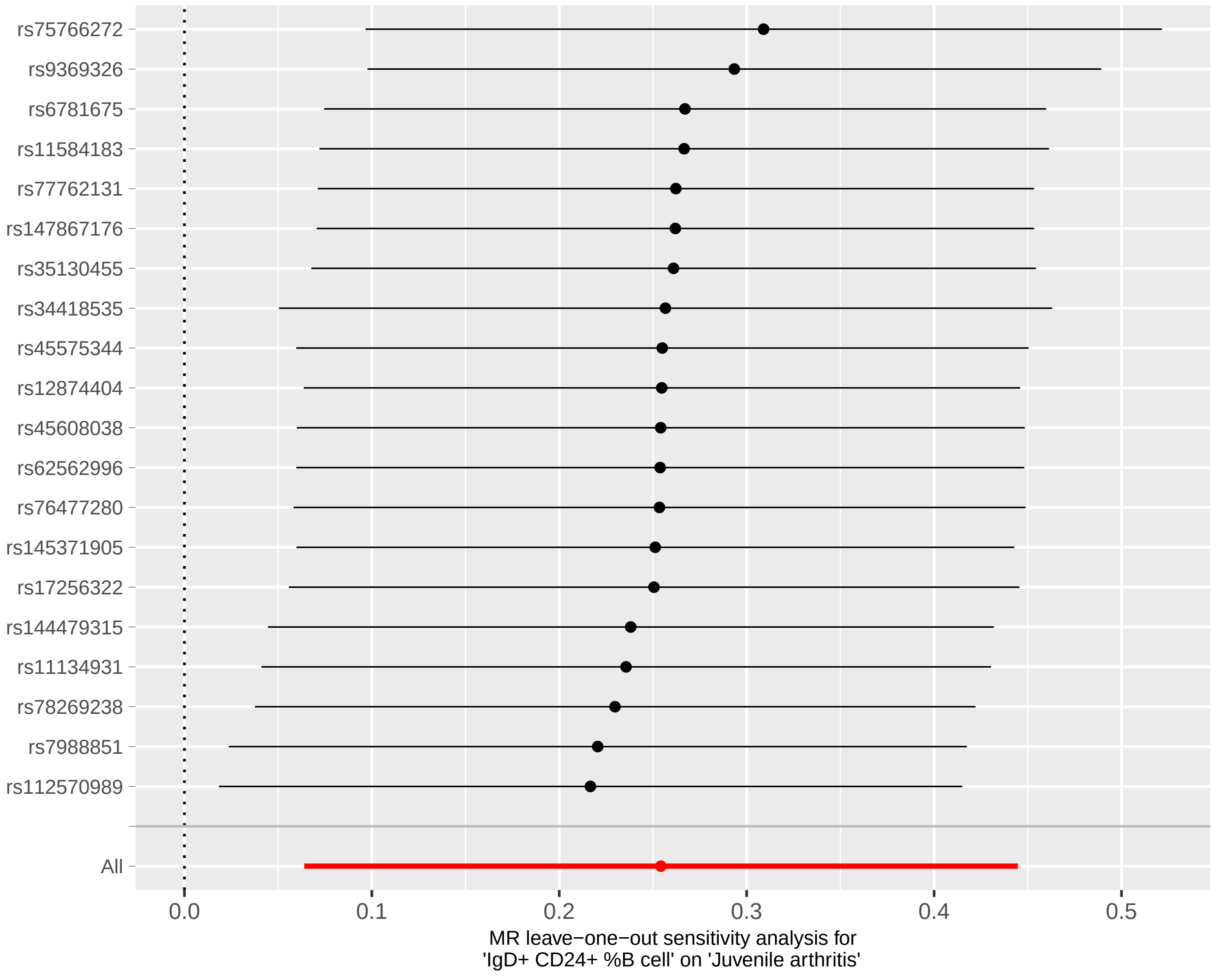

rs75766272
rs9369326
rs6781675
rs11584183
rs77762131
rs147867176
rs35130455
rs34418535
rs45575344
rs12874404
rs45608038
rs62562996
rs76477280
rs145371905
rs17256322
rs144479315
rs11134931
rs78269238
rs7988851
rs112570989
All
0.0
0.1
0.2
0.3
0.4
0.5
MR leave−one−out sensitivity analysis for
'IgD+ CD24+ %B cell' on 'Juvenile arthritis'

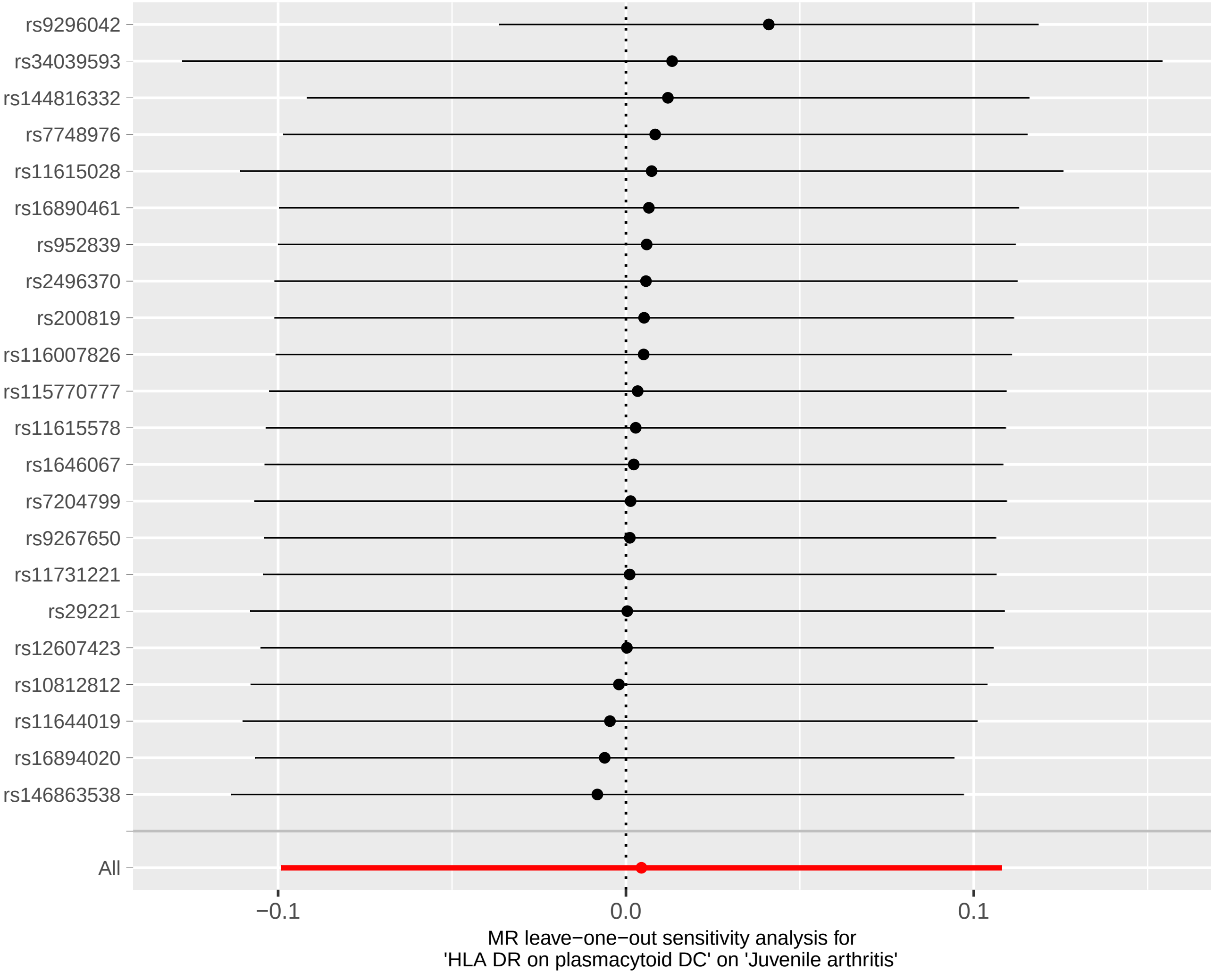

rs9296042
rs34039593
rs144816332
rs7748976
rs11615028
rs16890461
rs952839
rs2496370
rs200819
rs116007826
rs115770777
rs11615578
rs1646067
rs7204799
rs9267650
rs11731221
rs29221
rs12607423
rs10812812
rs11644019
rs16894020
rs146863538
All
−0.1
0.0
0.1
MR leave−one−out sensitivity analysis for
'HLA DR on plasmacytoid DC' on 'Juvenile arthritis'

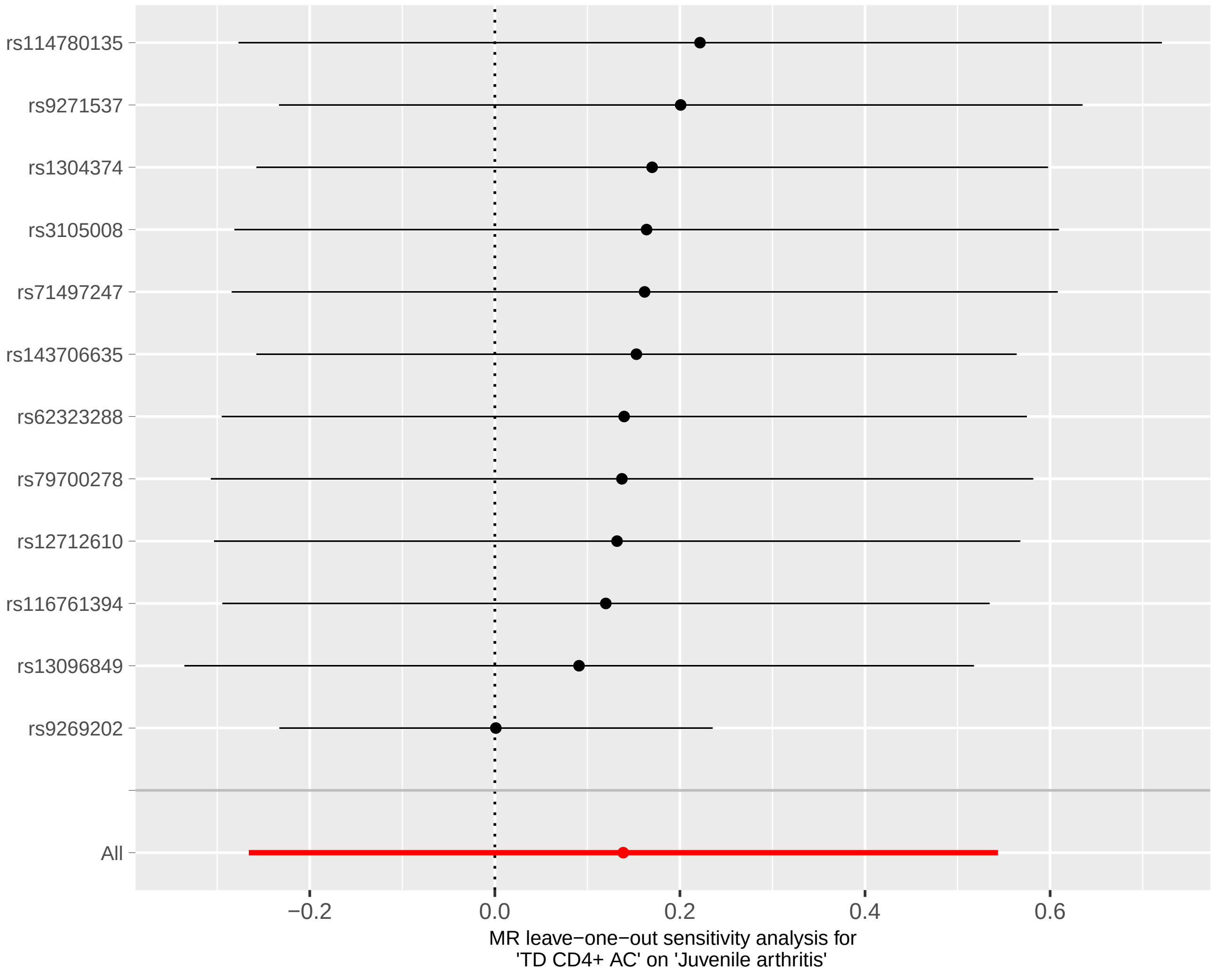

rs114780135
rs9271537
rs1304374
rs3105008
rs71497247
rs143706635
rs62323288
rs79700278
rs12712610
rs116761394
rs13096849
rs9269202
All
−0.2
0.0
0.2
0.4
0.6
MR leave−one−out sensitivity analysis for
'TD CD4+ AC' on 'Juvenile arthritis'

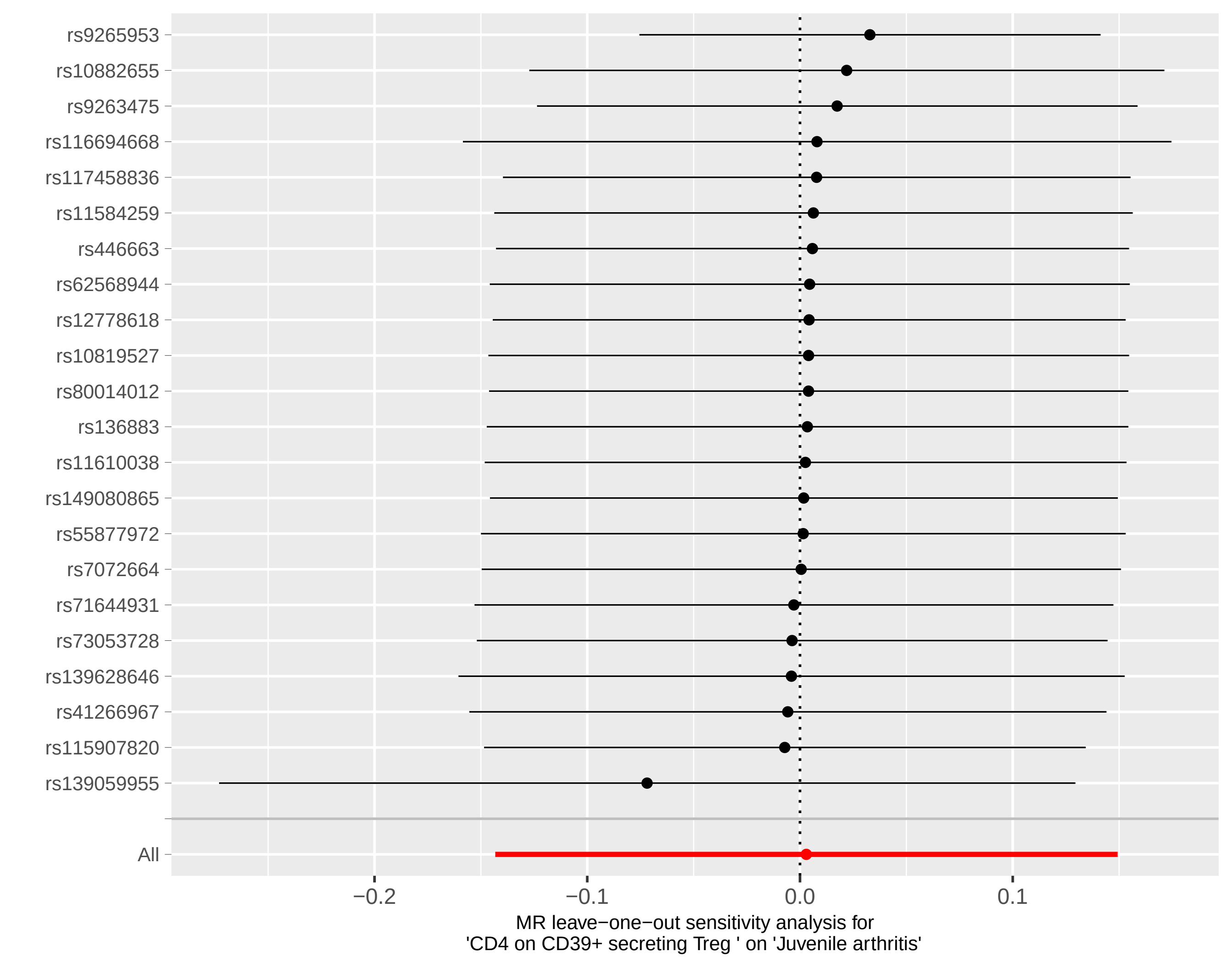

rs9265953
rs10882655
rs9263475
rs116694668
rs117458836
rs11584259
rs446663
rs62568944
rs12778618
rs10819527
rs80014012
rs136883
rs11610038
rs149080865
rs55877972
rs7072664
rs71644931
rs73053728
rs139628646
rs41266967
rs115907820
rs139059955
All
−0.2
−0.1
0.0
0.1
MR leave−one−out sensitivity analysis for
'CD4 on CD39+ secreting Treg ' on 'Juvenile arthritis'

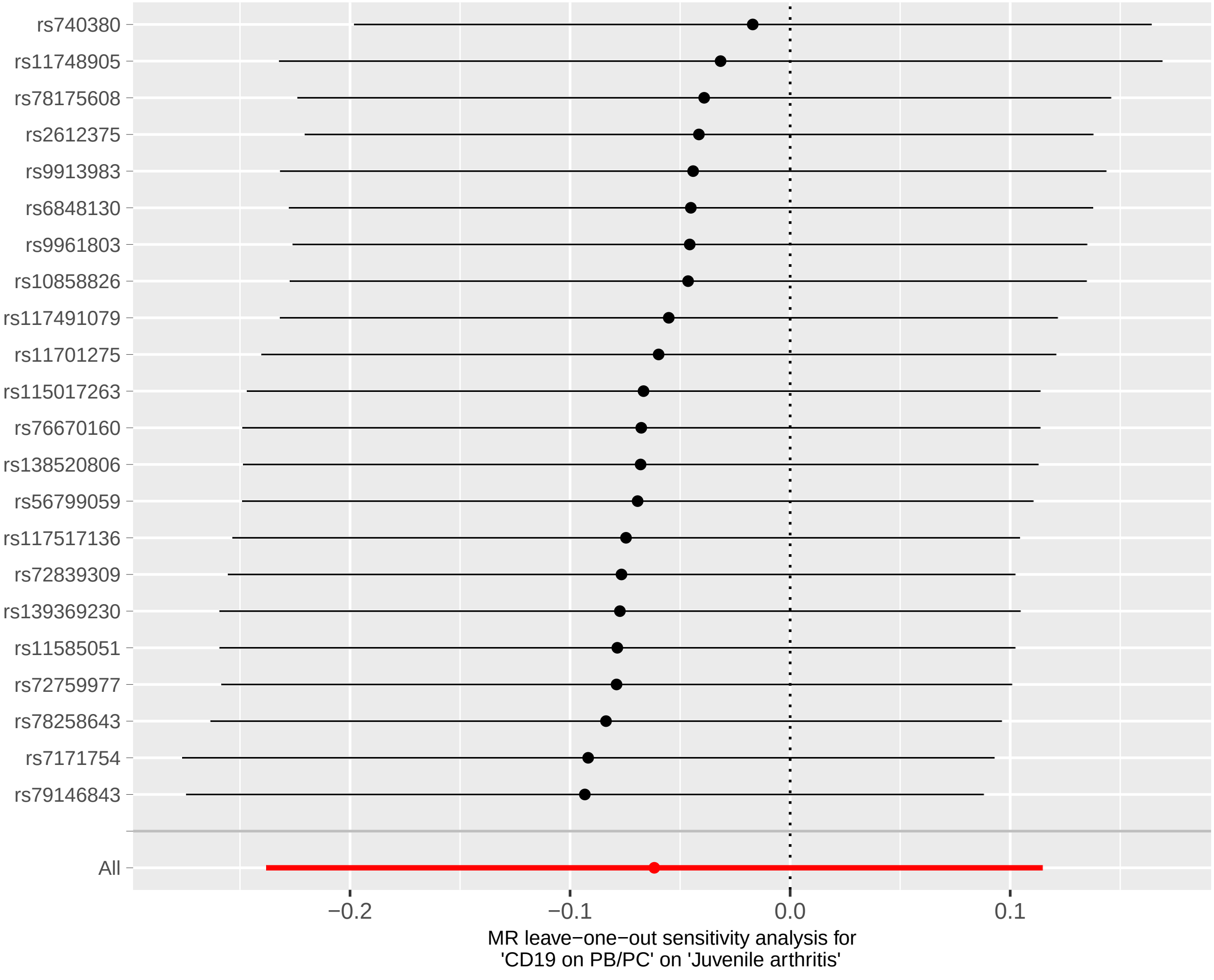

rs740380
rs11748905
rs78175608
rs2612375
rs9913983
rs6848130
rs9961803
rs10858826
rs117491079
rs11701275
rs115017263
rs76670160
rs138520806
rs56799059
rs117517136
rs72839309
rs139369230
rs11585051
rs72759977
rs78258643
rs7171754
rs79146843
All
−0.2
−0.1
0.0
0.1
MR leave−one−out sensitivity analysis for
'CD19 on PB/PC' on 'Juvenile arthritis'

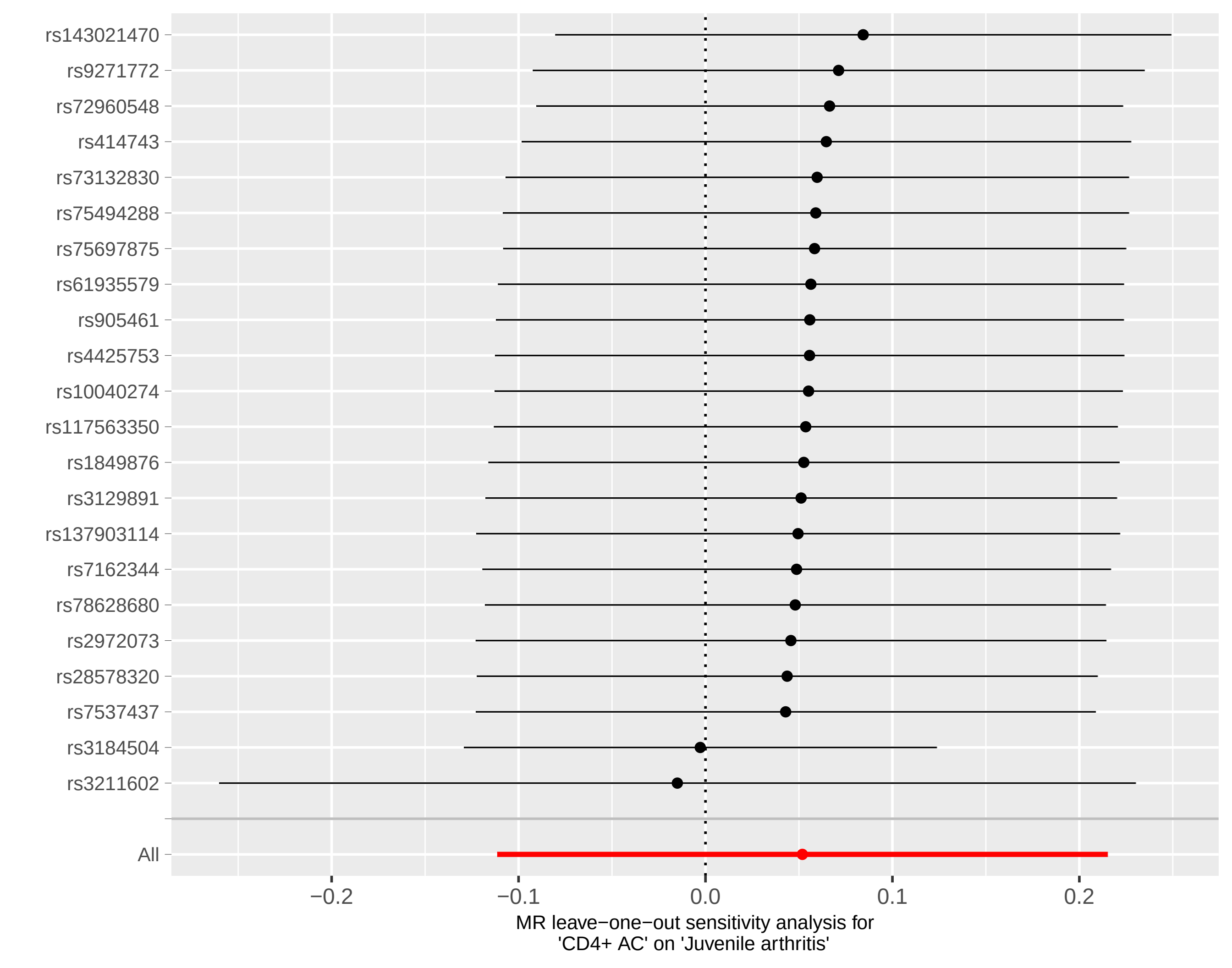

rs143021470
rs9271772
rs72960548
rs414743
rs73132830
rs75494288
rs75697875
rs61935579
rs905461
rs4425753
rs10040274
rs117563350
rs1849876
rs3129891
rs137903114
rs7162344
rs78628680
rs2972073
rs28578320
rs7537437
rs3184504
rs3211602
All
−0.2
−0.1
0.0
0.1
0.2
MR leave−one−out sensitivity analysis for
'CD4+ AC' on 'Juvenile arthritis'

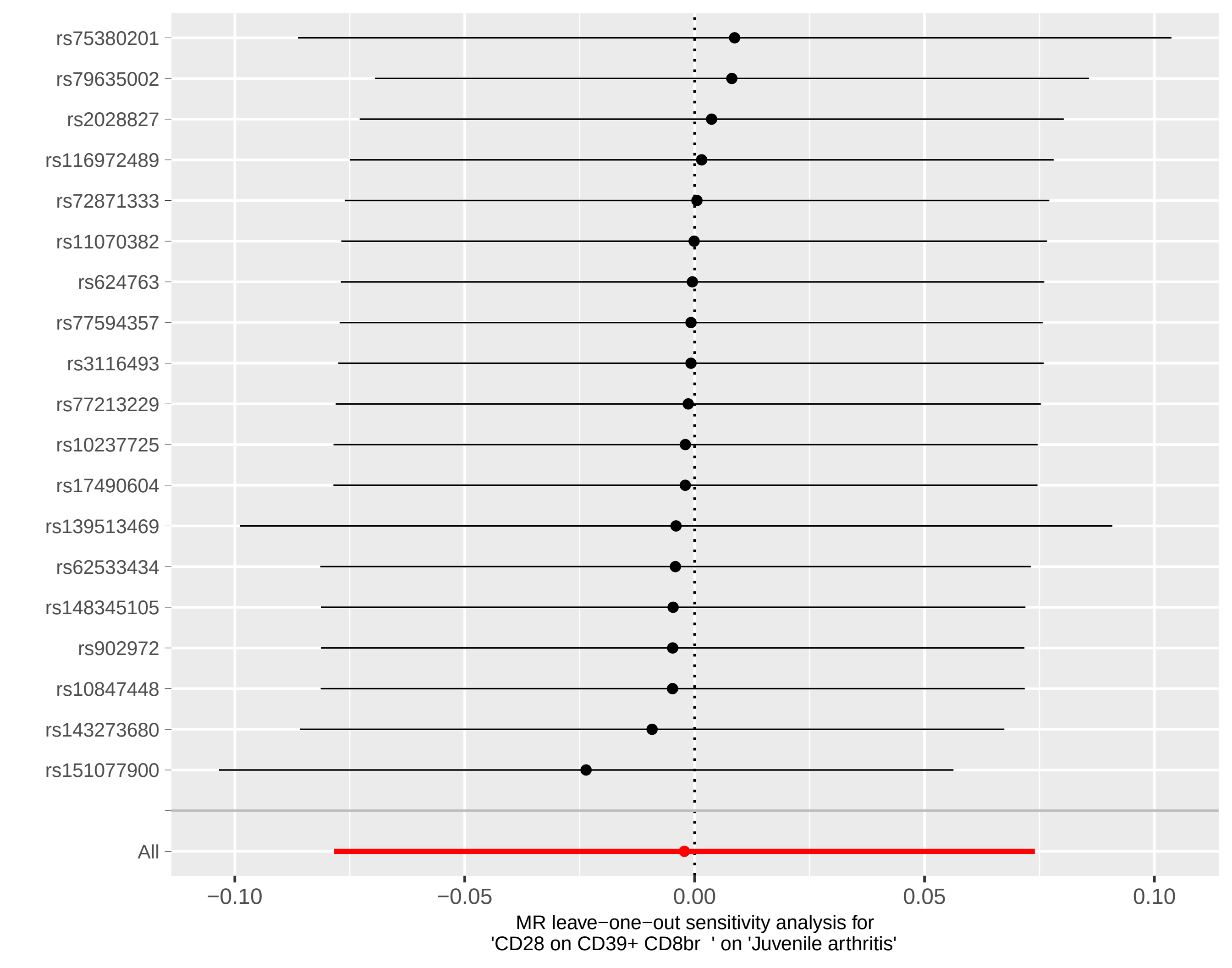

rs75380201
rs79635002
rs2028827
rs116972489
rs72871333
rs11070382
rs624763
rs77594357
rs3116493
rs77213229
rs10237725
rs17490604
rs139513469
rs62533434
rs148345105
rs902972
rs10847448
rs143273680
rs151077900
All
−0.10
−0.05
0.00
0.05
0.10
MR leave−one−out sensitivity analysis for
'CD28 on CD39+ CD8br ' on 'Juvenile arthritis'

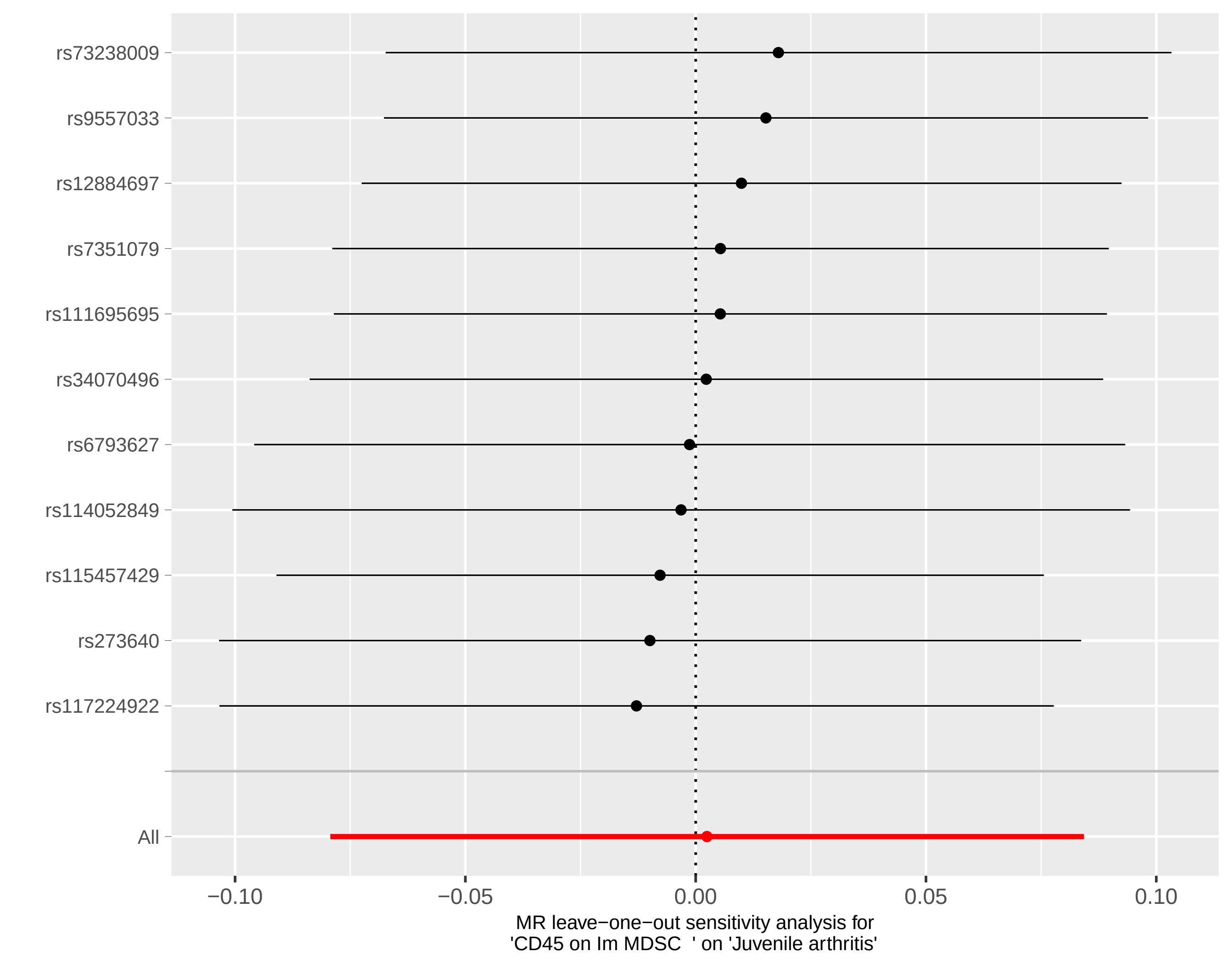
rs73238009
rs9557033
rs12884697
rs7351079
rs111695695
rs34070496
rs6793627
rs114052849
rs115457429
rs273640
rs117224922
All
−0.10
−0.05
0.00
0.05
0.10
MR leave−one−out sensitivity analysis for
'CD45 on Im MDSC ' on 'Juvenile arthritis'

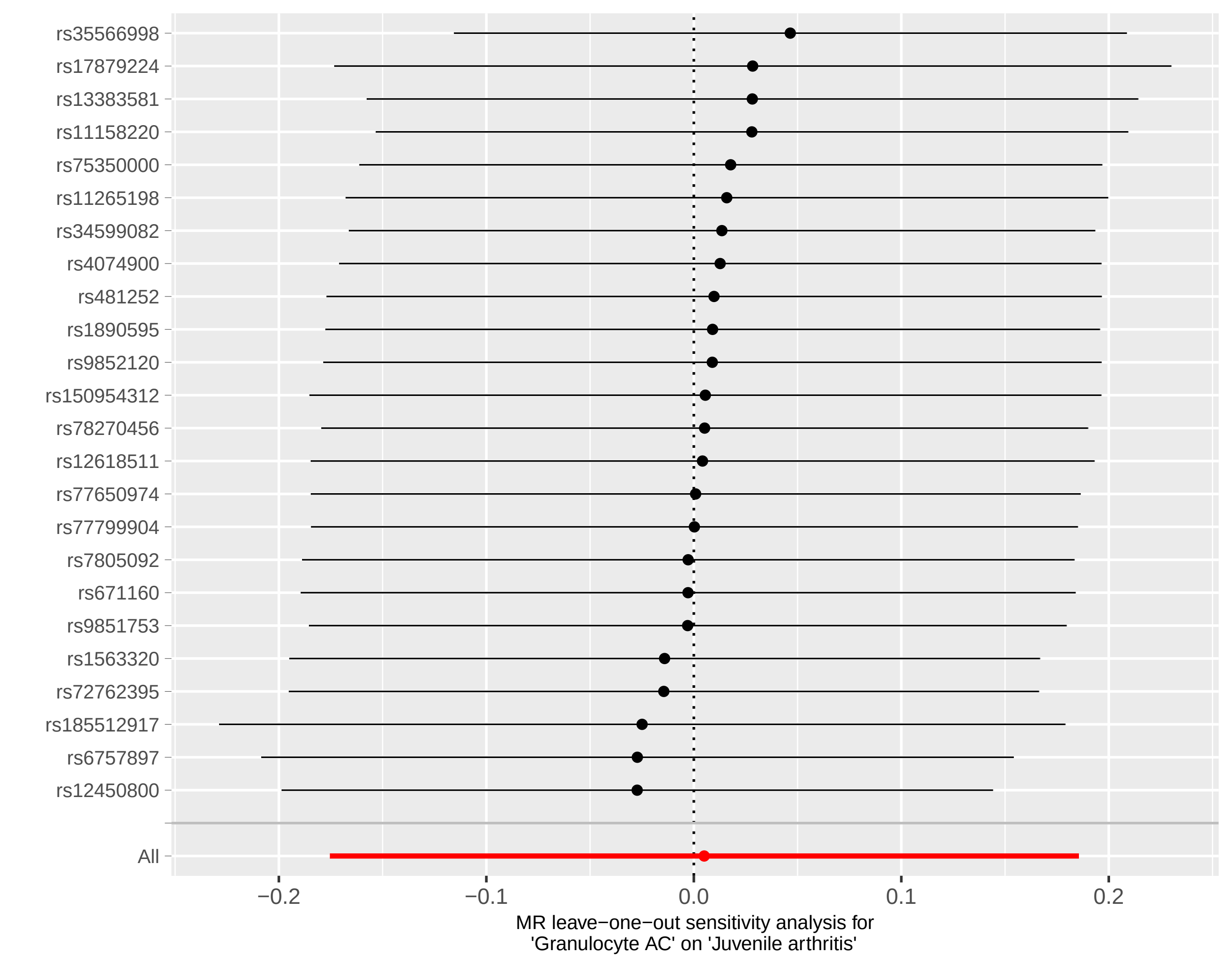
rs35566998
rs17879224
rs13383581
rs11158220
rs75350000
rs11265198
rs34599082
rs4074900
rs481252
rs1890595
rs9852120
rs150954312
rs78270456
rs12618511
rs77650974
rs77799904
rs7805092
rs671160
rs9851753
rs1563320
rs72762395
rs185512917
rs6757897
rs12450800
All
−0.2
−0.1
0.0
0.1
0.2
MR leave−one−out sensitivity analysis for
'Granulocyte AC' on 'Juvenile arthritis'

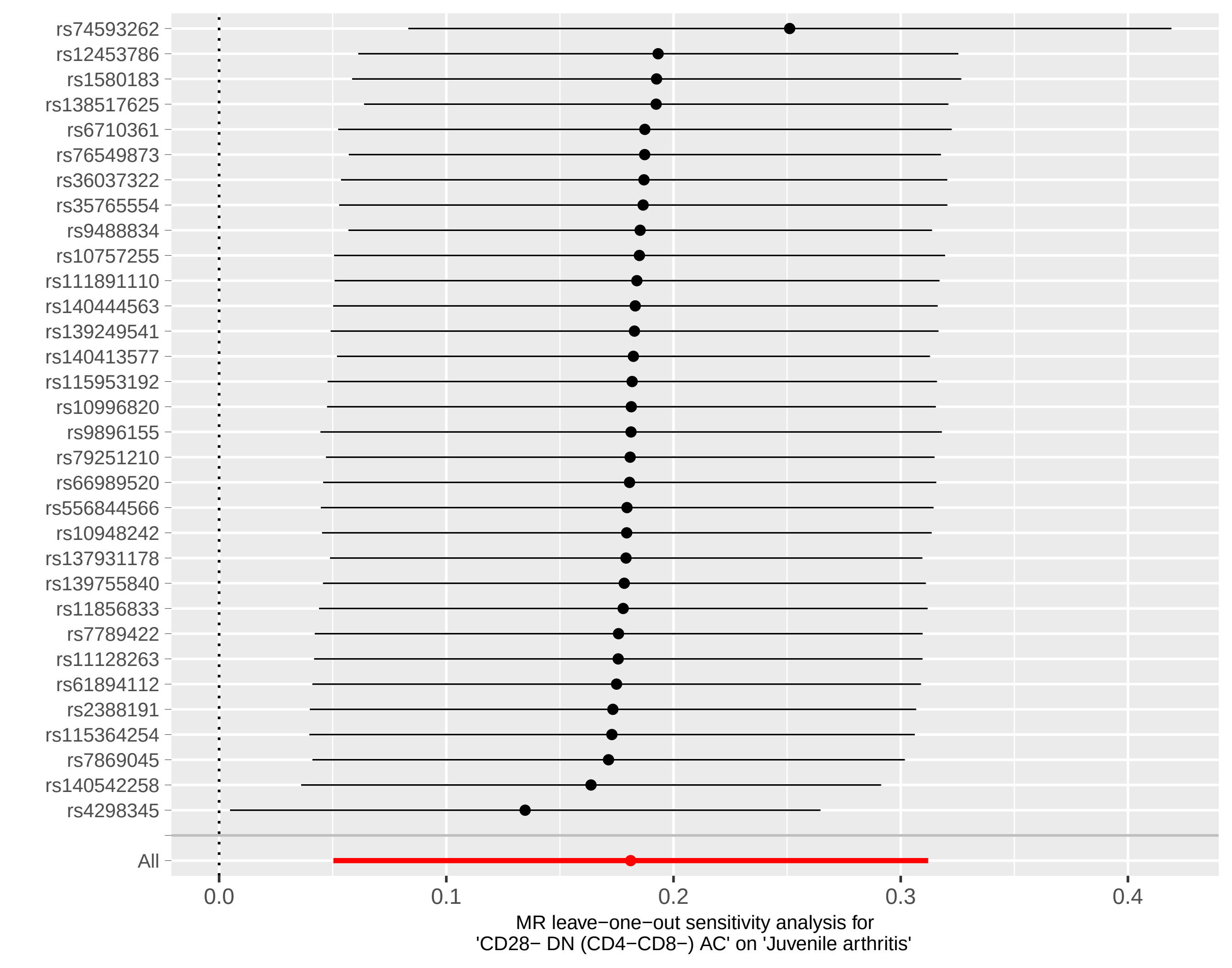
rs74593262
rs12453786
rs1580183
rs138517625
rs6710361
rs76549873
rs36037322
rs35765554
rs9488834
rs10757255
rs111891110
rs140444563
rs139249541
rs140413577
rs115953192
rs10996820
rs9896155
rs79251210
rs66989520
rs556844566
rs10948242
rs137931178
rs139755840
rs11856833
rs7789422
rs11128263
rs61894112
rs2388191
rs115364254
rs7869045
rs140542258
rs4298345
All
0.0
0.1
0.2
0.3
0.4
MR leave−one−out sensitivity analysis for
'CD28− DN (CD4−CD8−) AC' on 'Juvenile arthritis'

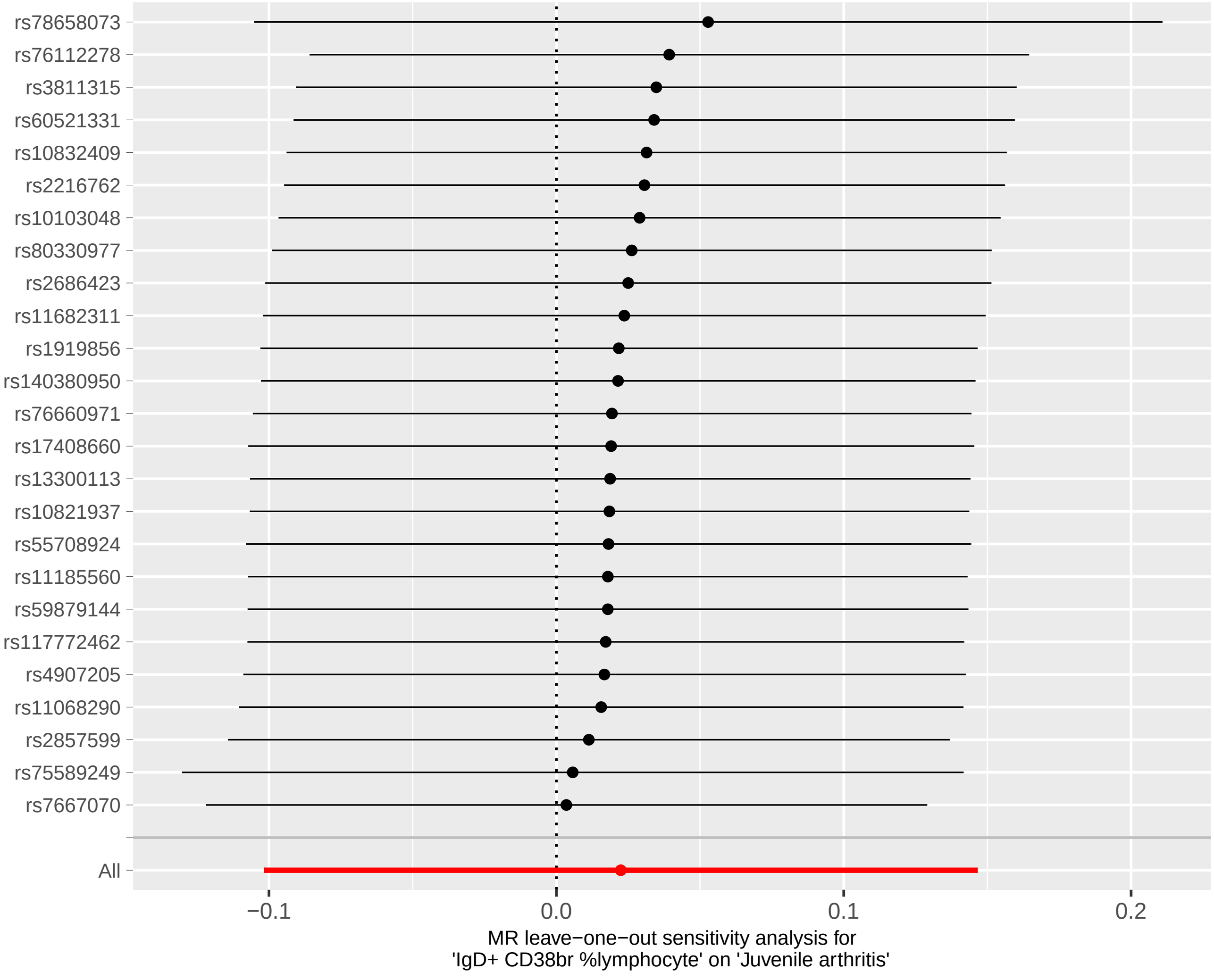
rs78658073
rs76112278
rs3811315
rs60521331
rs10832409
rs2216762
rs10103048
rs80330977
rs2686423
rs11682311
rs1919856
rs140380950
rs76660971
rs17408660
rs13300113
rs10821937
rs55708924
rs11185560
rs59879144
rs117772462
rs4907205
rs11068290
rs2857599
rs75589249
rs7667070
All
−0.1
0.0
0.1
0.2
MR leave−one−out sensitivity analysis for
'IgD+ CD38br %lymphocyte' on 'Juvenile arthritis'

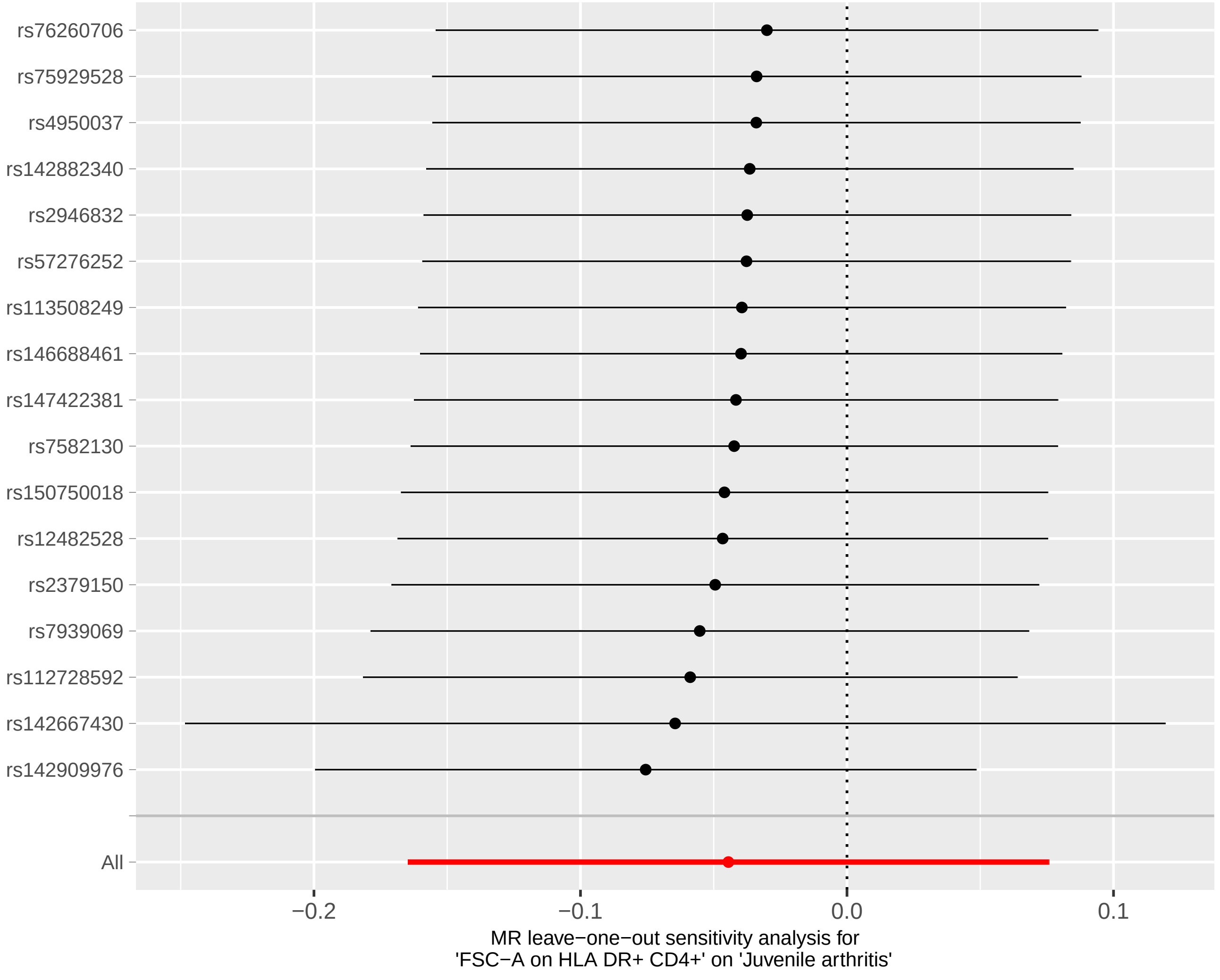

rs76260706
rs75929528
rs4950037
rs142882340
rs2946832
rs57276252
rs113508249
rs146688461
rs147422381
rs7582130
rs150750018
rs12482528
rs2379150
rs7939069
rs112728592
rs142667430
rs142909976
All
−0.2
−0.1
0.0
0.1
MR leave−one−out sensitivity analysis for
'FSC−A on HLA DR+ CD4+' on 'Juvenile arthritis'

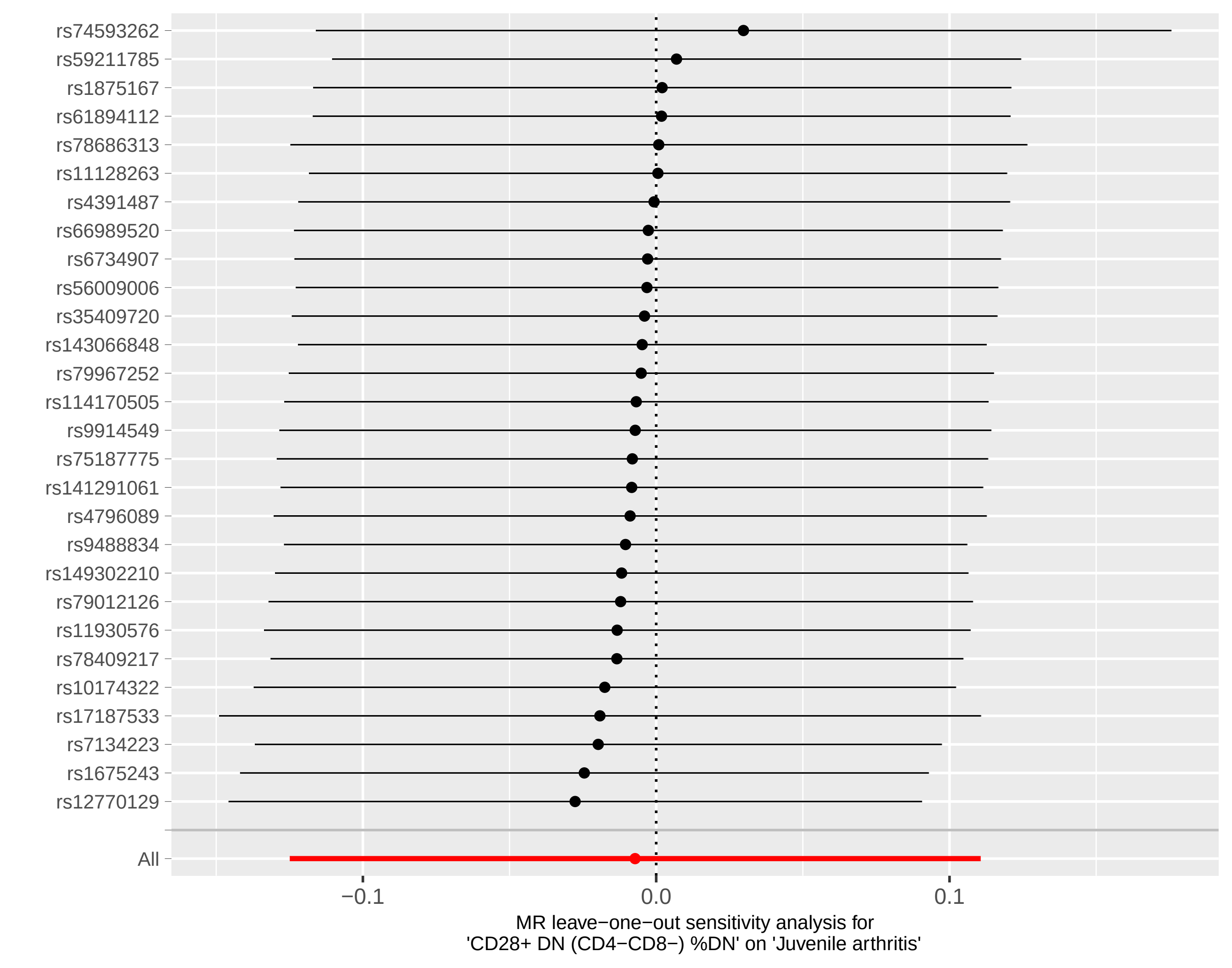
rs74593262
rs59211785
rs1875167
rs61894112
rs78686313
rs11128263
rs4391487
rs66989520
rs6734907
rs56009006
rs35409720
rs143066848
rs79967252
rs114170505
rs9914549
rs75187775
rs141291061
rs4796089
rs9488834
rs149302210
rs79012126
rs11930576
rs78409217
rs10174322
rs17187533
rs7134223
rs1675243
rs12770129
All
−0.1
0.0
0.1
MR leave−one−out sensitivity analysis for
'CD28+ DN (CD4−CD8−) %DN' on 'Juvenile arthritis'

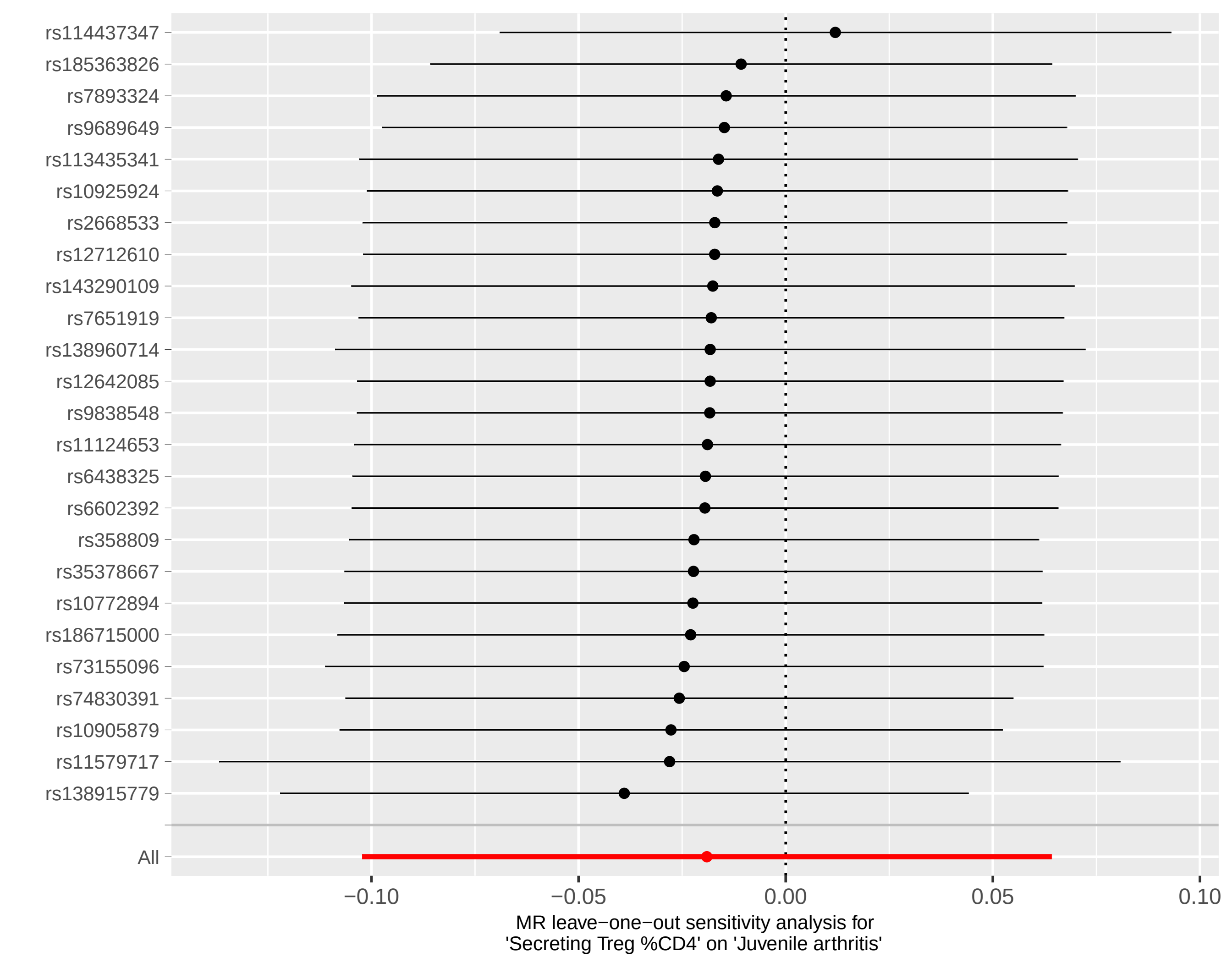
rs114437347
rs185363826
rs7893324
rs9689649
rs113435341
rs10925924
rs2668533
rs12712610
rs143290109
rs7651919
rs138960714
rs12642085
rs9838548
rs11124653
rs6438325
rs6602392
rs358809
rs35378667
rs10772894
rs186715000
rs73155096
rs74830391
rs10905879
rs11579717
rs138915779
All
−0.10
−0.05
0.00
0.05
0.10
MR leave−one−out sensitivity analysis for
'Secreting Treg %CD4' on 'Juvenile arthritis'

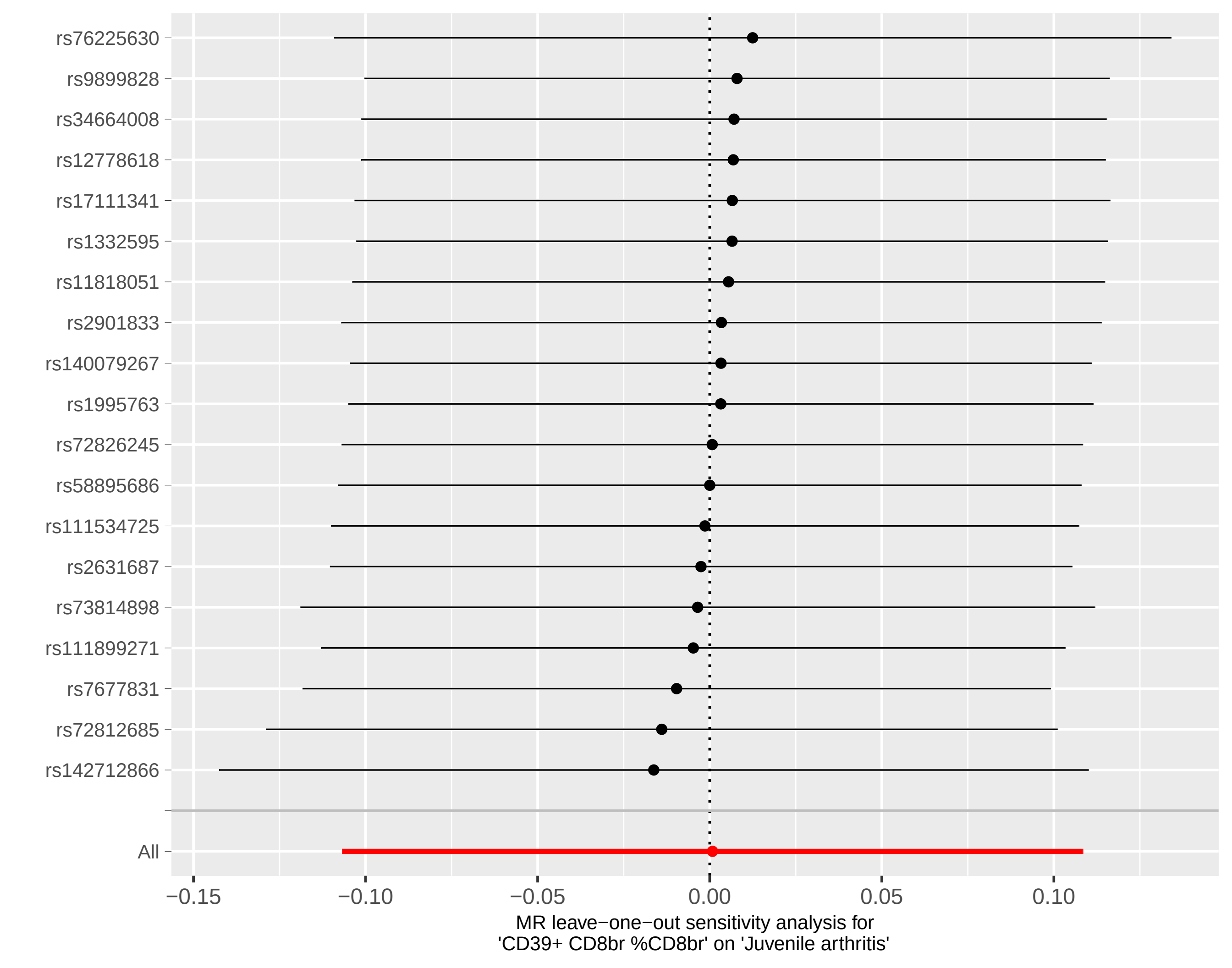
rs76225630
rs9899828
rs34664008
rs12778618
rs17111341
rs1332595
rs11818051
rs2901833
rs140079267
rs1995763
rs72826245
rs58895686
rs111534725
rs2631687
rs73814898
rs111899271
rs7677831
rs72812685
rs142712866
All
−0.15
−0.10
−0.05
0.00
0.05
0.10
MR leave−one−out sensitivity analysis for
'CD39+ CD8br %CD8br' on 'Juvenile arthritis'

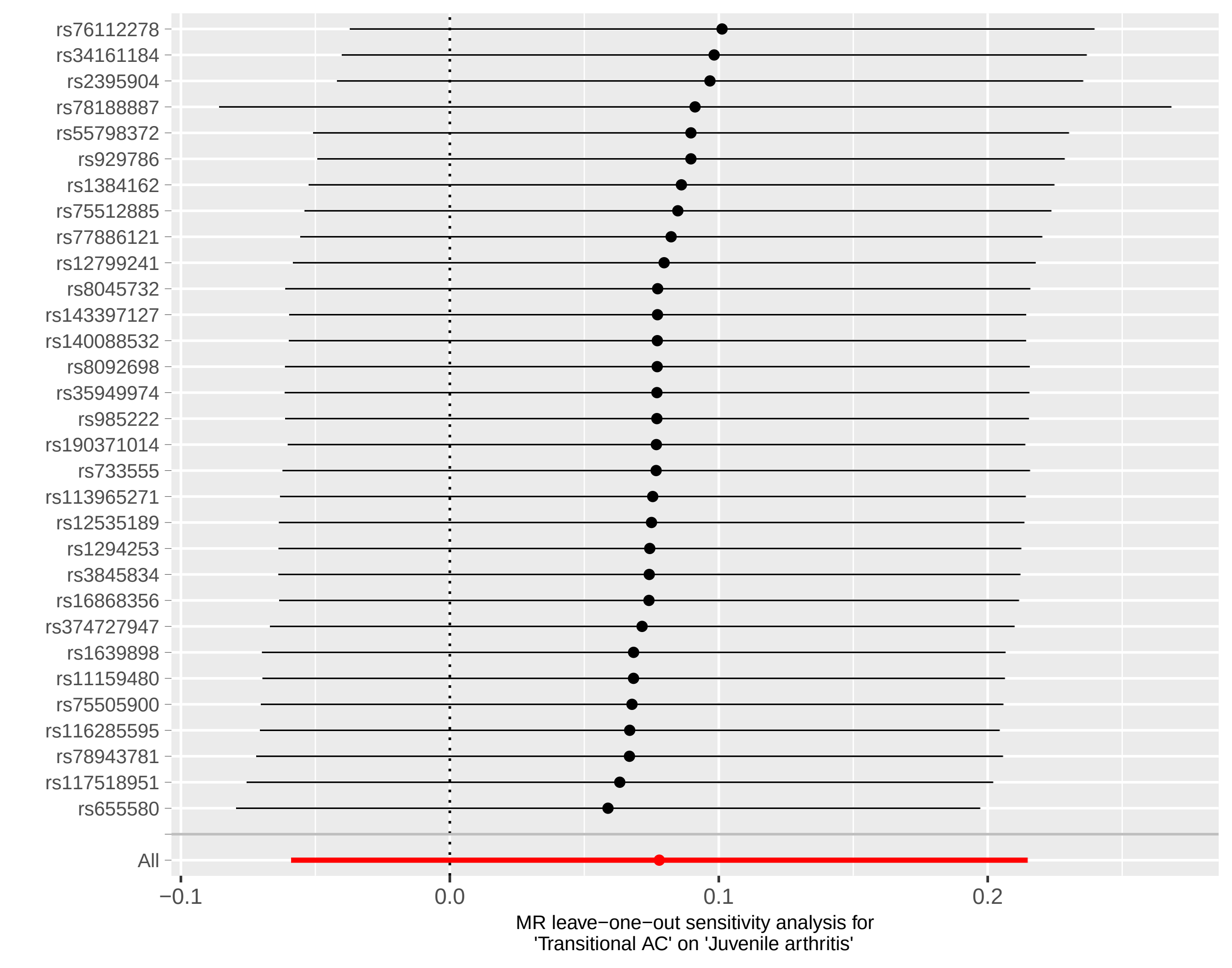

rs76112278
rs34161184
rs2395904
rs78188887
rs55798372
rs929786
rs1384162
rs75512885
rs77886121
rs12799241
rs8045732
rs143397127
rs140088532
rs8092698
rs35949974
rs985222
rs190371014
rs733555
rs113965271
rs12535189
rs1294253
rs3845834
rs16868356
rs374727947
rs1639898
rs11159480
rs75505900
rs116285595
rs78943781
rs117518951
rs655580
All
−0.1
0.0
0.1
0.2
MR leave−one−out sensitivity analysis for
'Transitional AC' on 'Juvenile arthritis'

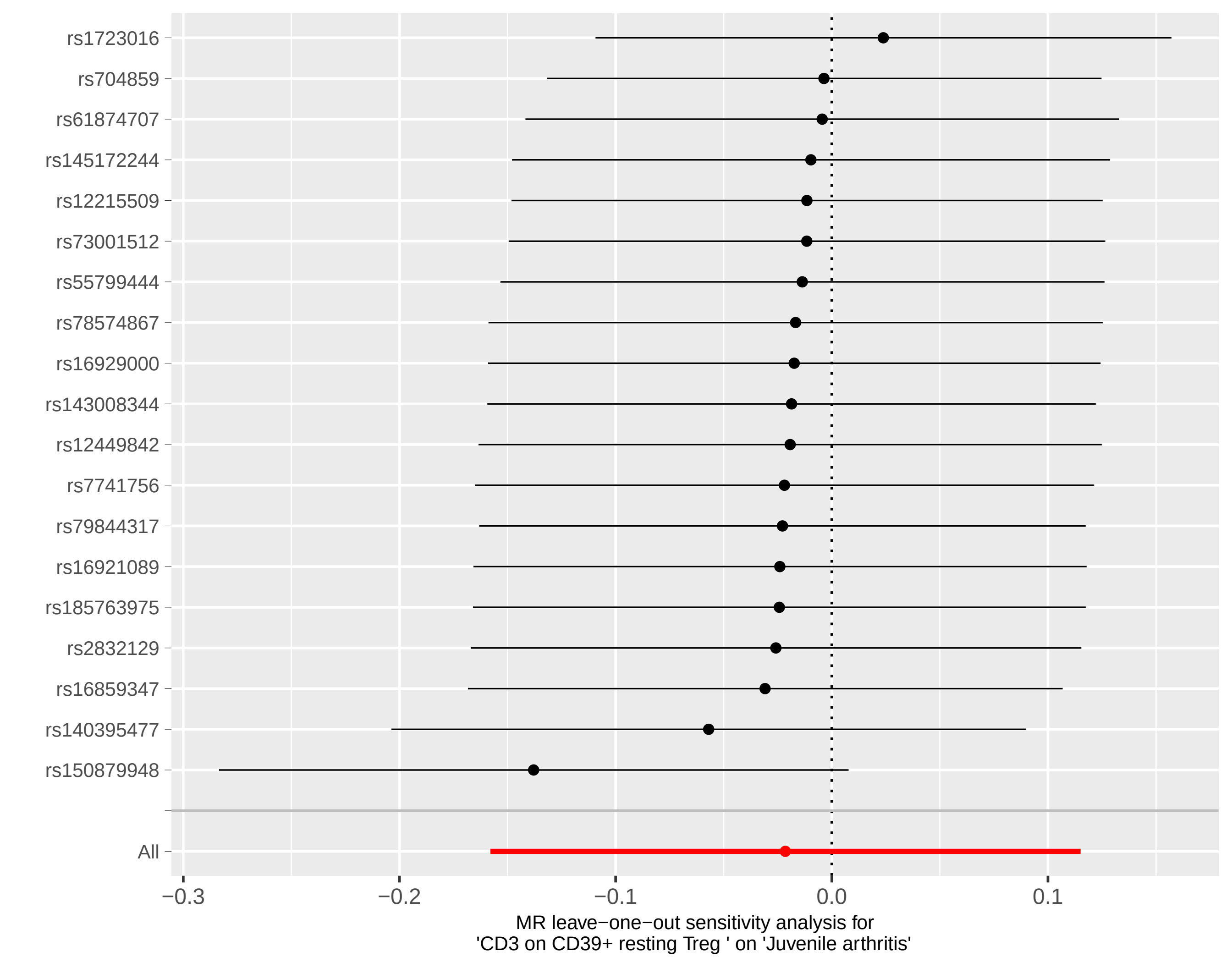

rs1723016
rs704859
rs61874707
rs145172244
rs12215509
rs73001512
rs55799444
rs78574867
rs16929000
rs143008344
rs12449842
rs7741756
rs79844317
rs16921089
rs185763975
rs2832129
rs16859347
rs140395477
rs150879948
All
−0.3
−0.2
−0.1
0.0
0.1
MR leave−one−out sensitivity analysis for
'CD3 on CD39+ resting Treg ' on 'Juvenile arthritis'

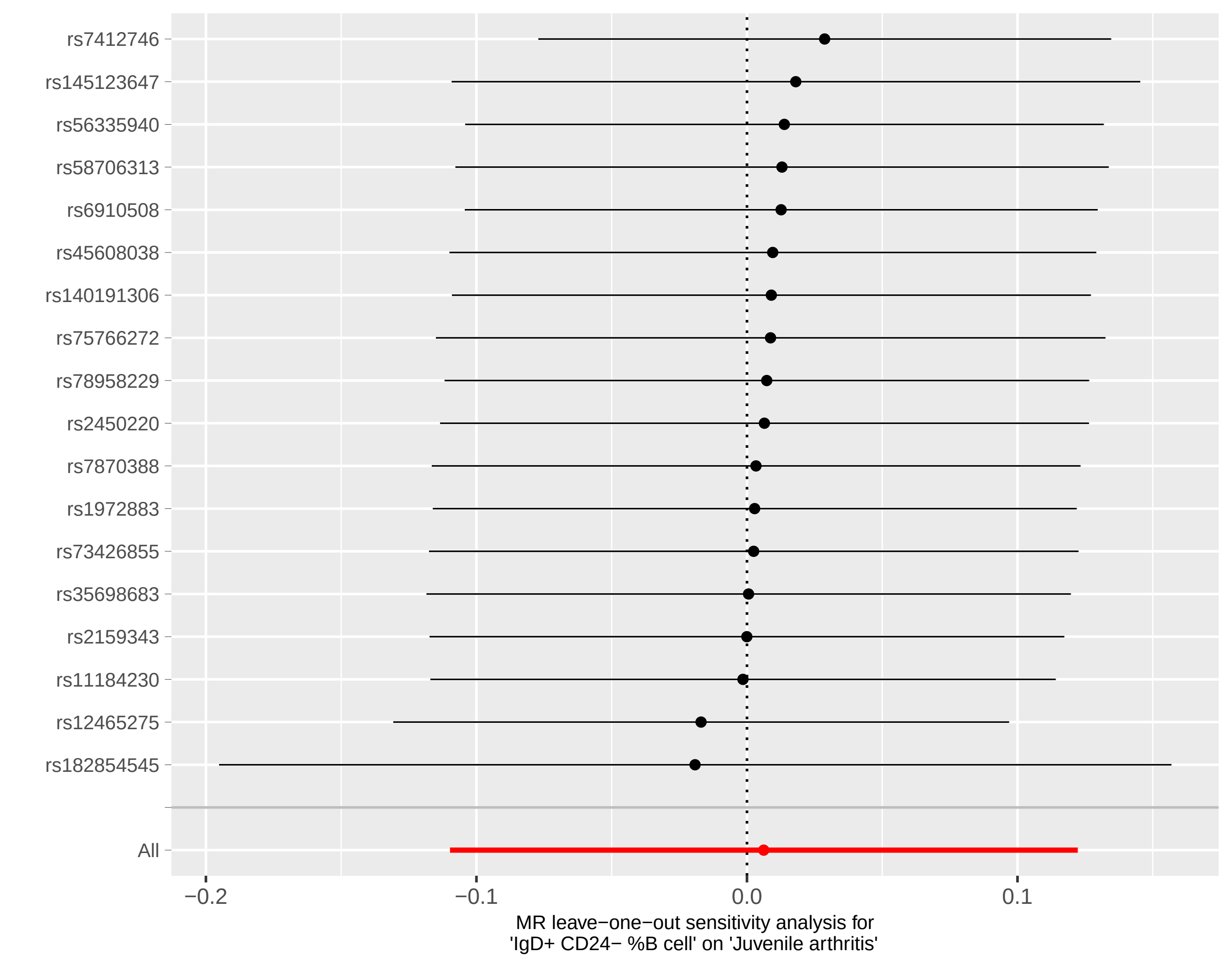
rs7412746
rs145123647
rs56335940
rs58706313
rs6910508
rs45608038
rs140191306
rs75766272
rs78958229
rs2450220
rs7870388
rs1972883
rs73426855
rs35698683
rs2159343
rs11184230
rs12465275
rs182854545
All
−0.2
−0.1
0.0
0.1
MR leave−one−out sensitivity analysis for
'IgD+ CD24− %B cell' on 'Juvenile arthritis'

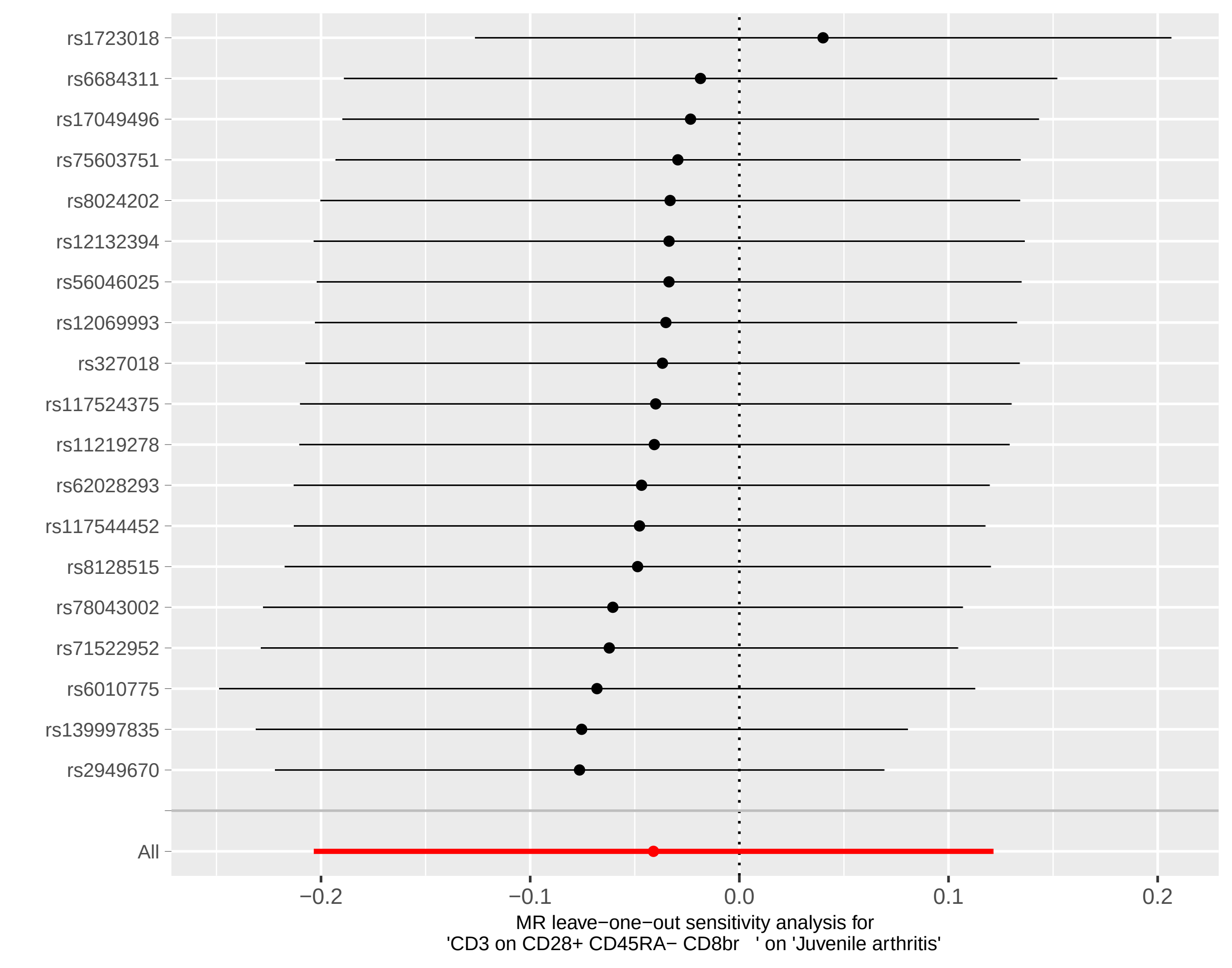

rs1723018
rs6684311
rs17049496
rs75603751
rs8024202
rs12132394
rs56046025
rs12069993
rs327018
rs117524375
rs11219278
rs62028293
rs117544452
rs8128515
rs78043002
rs71522952
rs6010775
rs139997835
rs2949670
All
−0.2
−0.1
0.0
0.1
0.2
MR leave−one−out sensitivity analysis for
'CD3 on CD28+ CD45RA− CD8br ' on 'Juvenile arthritis'

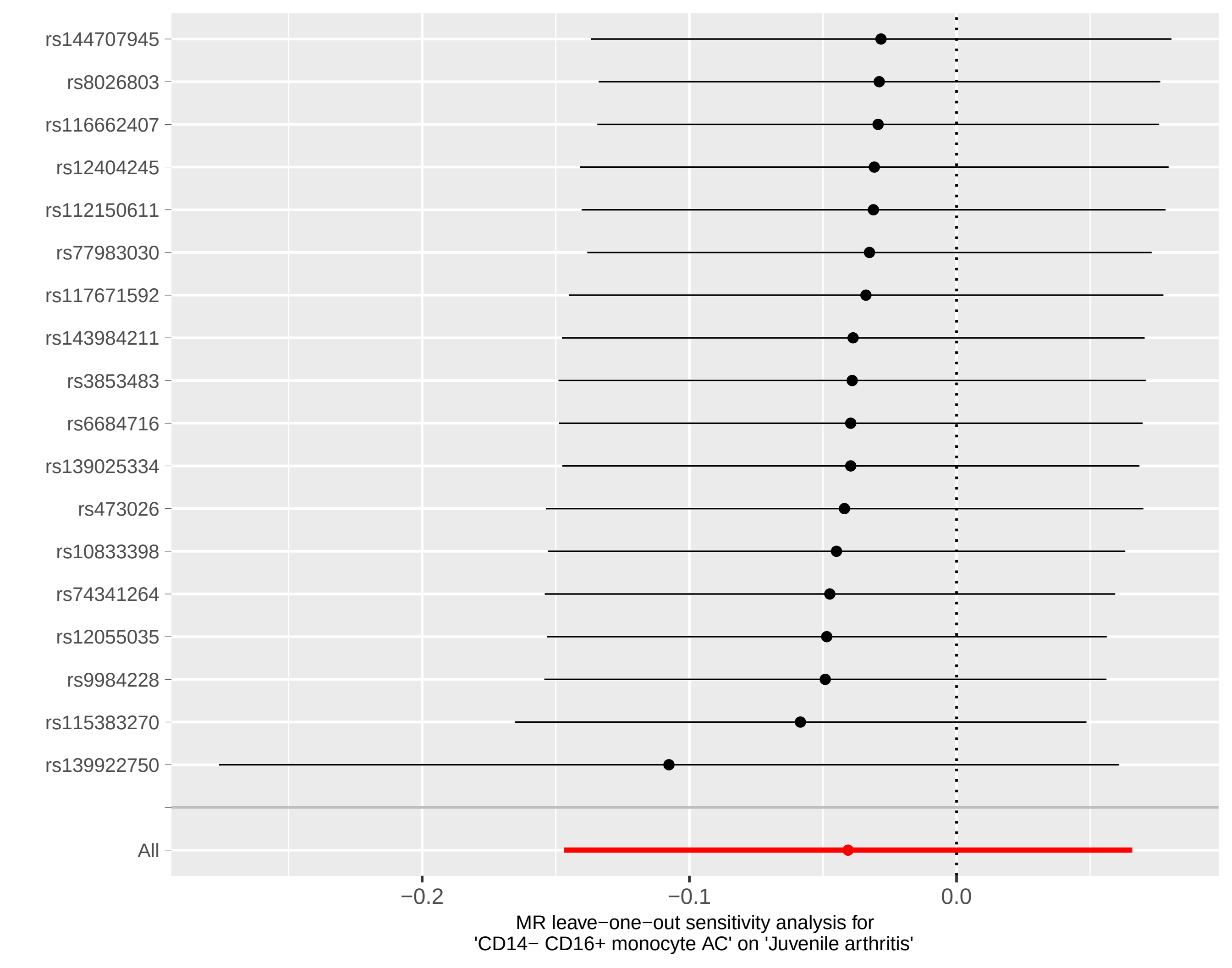
rs144707945
rs8026803
rs116662407
rs12404245
rs112150611
rs77983030
rs117671592
rs143984211
rs3853483
rs6684716
rs139025334
rs473026
rs10833398
rs74341264
rs12055035
rs9984228
rs115383270
rs139922750
All
−0.2
−0.1
0.0
MR leave−one−out sensitivity analysis for
'CD14− CD16+ monocyte AC' on 'Juvenile arthritis'

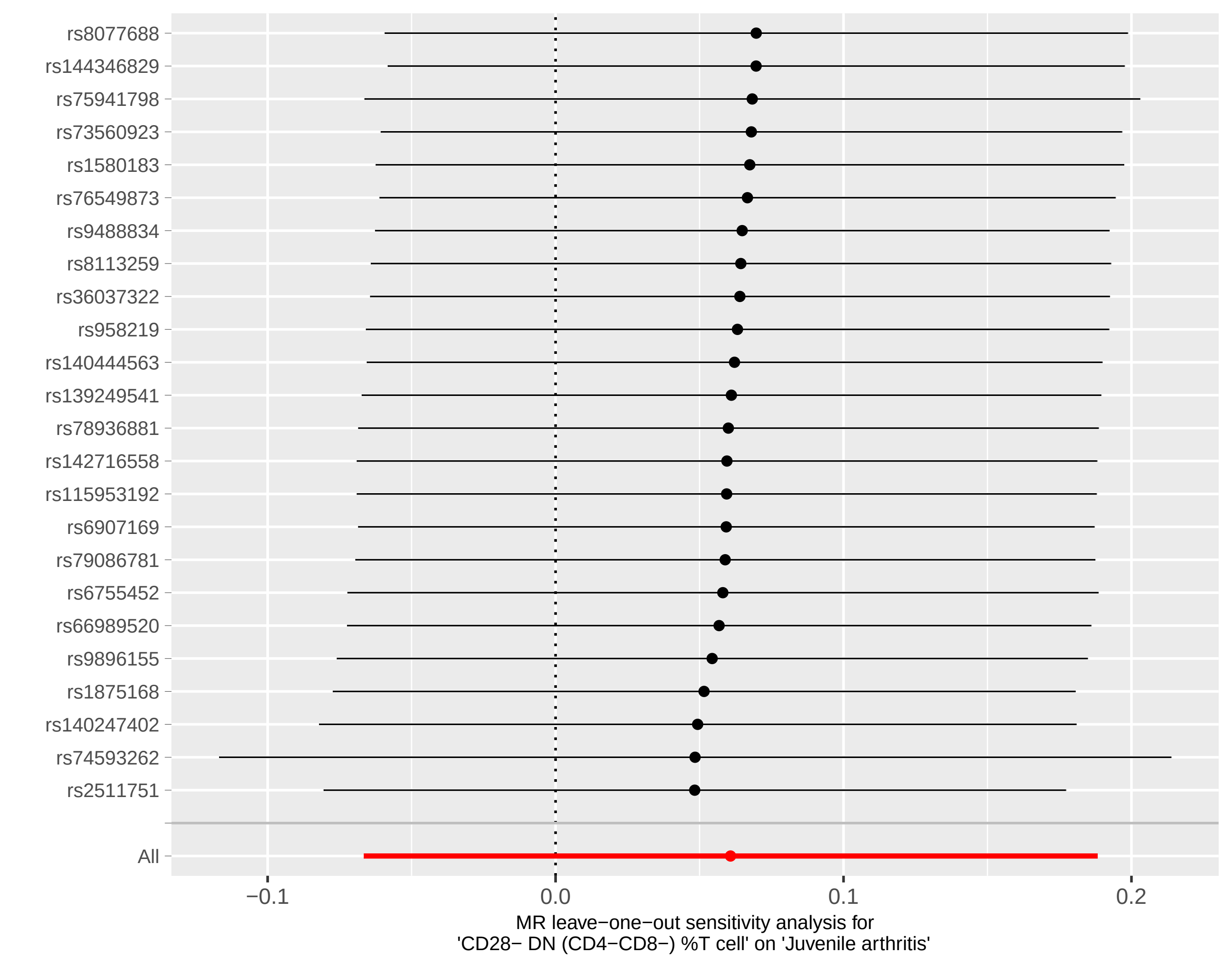
rs8077688
rs144346829
rs75941798
rs73560923
rs1580183
rs76549873
rs9488834
rs8113259
rs36037322
rs958219
rs140444563
rs139249541
rs78936881
rs142716558
rs115953192
rs6907169
rs79086781
rs6755452
rs66989520
rs9896155
rs1875168
rs140247402
rs74593262
rs2511751
All
−0.1
0.0
0.1
0.2
MR leave−one−out sensitivity analysis for
'CD28− DN (CD4−CD8−) %T cell' on 'Juvenile arthritis'

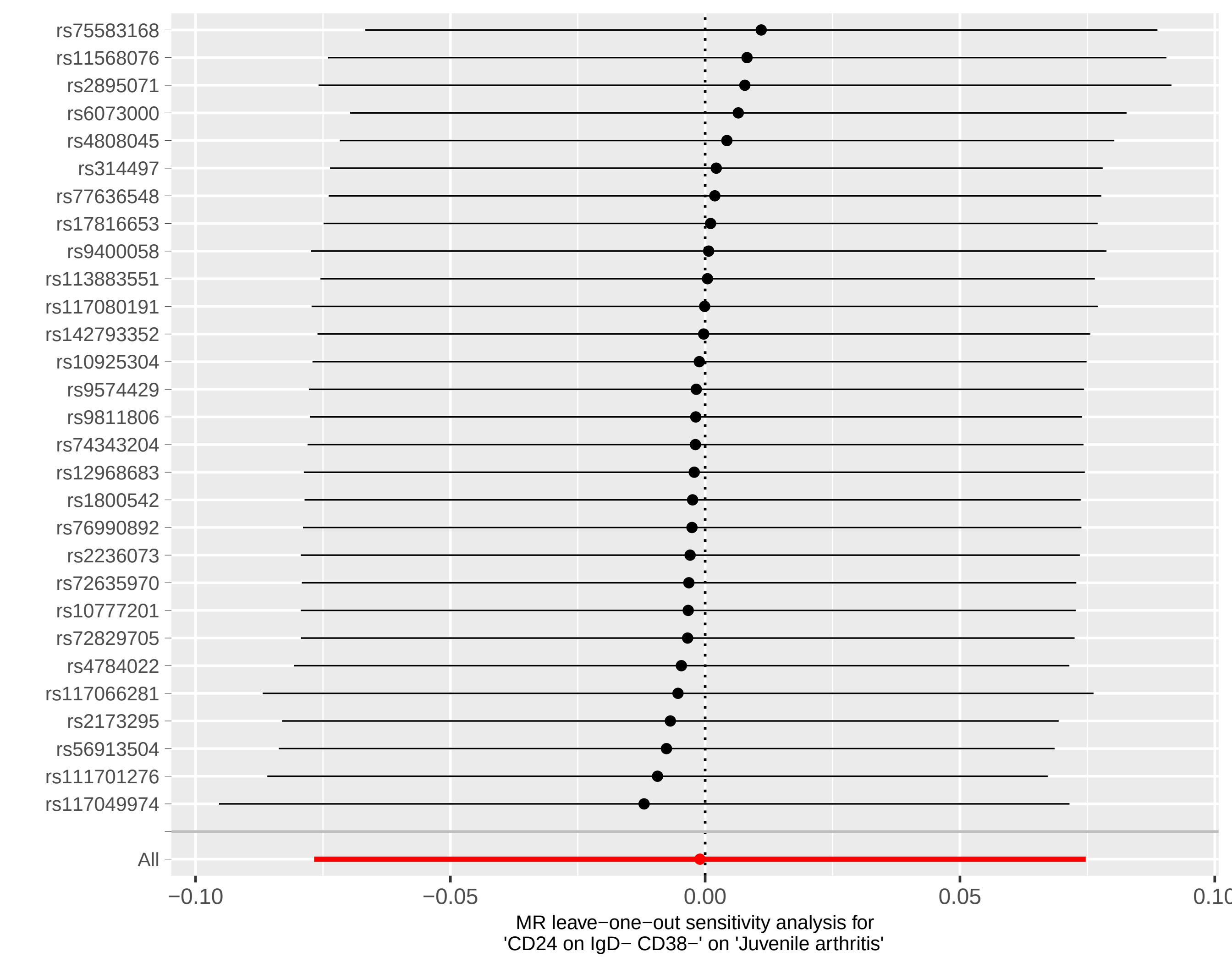

rs75583168
rs11568076
rs2895071
rs6073000
rs4808045
rs314497
rs77636548
rs17816653
rs9400058
rs113883551
rs117080191
rs142793352
rs10925304
rs9574429
rs9811806
rs74343204
rs12968683
rs1800542
rs76990892
rs2236073
rs72635970
rs10777201
rs72829705
rs4784022
rs117066281
rs2173295
rs56913504
rs111701276
rs117049974
All
−0.10
−0.05
0.00
0.05
MR leave−one−out sensitivity analysis for
'CD24 on IgD− CD38−' on 'Juvenile arthritis'

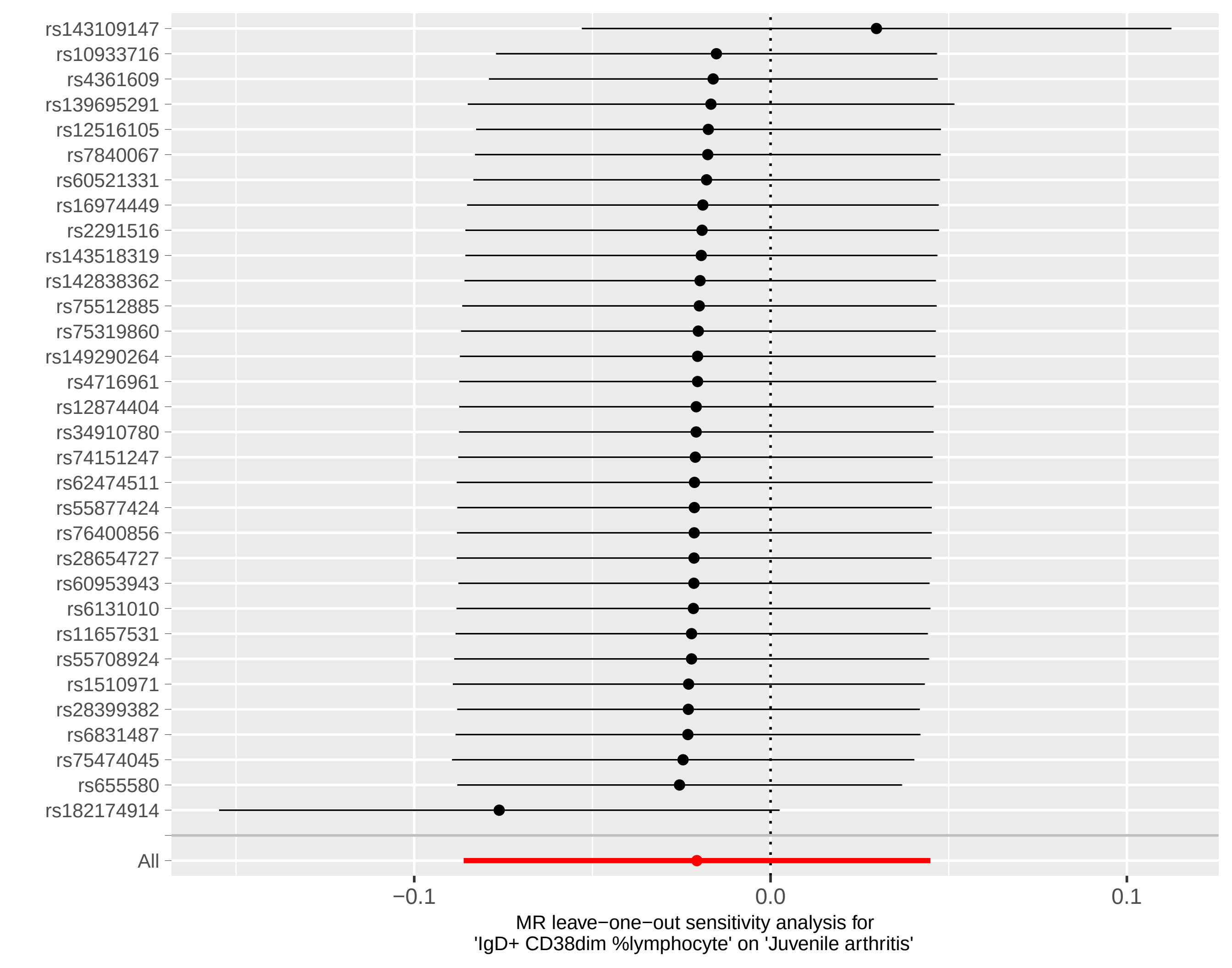
rs143109147
rs10933716
rs4361609
rs139695291
rs12516105
rs7840067
rs60521331
rs16974449
rs2291516
rs143518319
rs142838362
rs75512885
rs75319860
rs149290264
rs4716961
rs12874404
rs34910780
rs74151247
rs62474511
rs55877424
rs76400856
rs28654727
rs60953943
rs6131010
rs11657531
rs55708924
rs1510971
rs28399382
rs6831487
rs75474045
rs655580
rs182174914
All
−0.1
0.0
0.1
MR leave−one−out sensitivity analysis for
'IgD+ CD38dim %lymphocyte' on 'Juvenile arthritis'

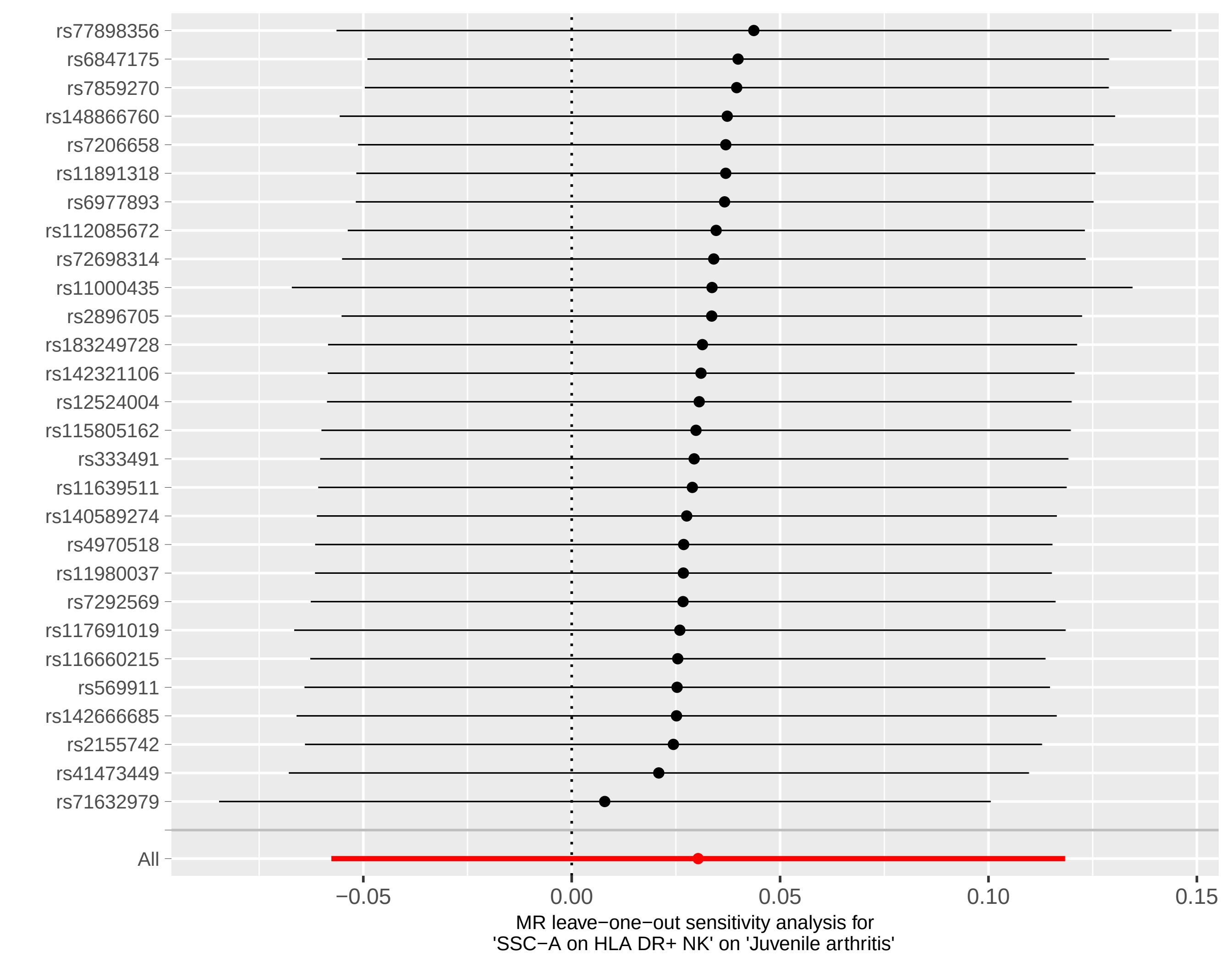
rs77898356
rs6847175
rs7859270
rs148866760
rs7206658
rs11891318
rs6977893
rs112085672
rs72698314
rs11000435
rs2896705
rs183249728
rs142321106
rs12524004
rs115805162
rs333491
rs11639511
rs140589274
rs4970518
rs11980037
rs7292569
rs117691019
rs116660215
rs569911
rs142666685
rs2155742
rs41473449
rs71632979
All
−0.05
0.00
0.05
0.10
0.15
MR leave−one−out sensitivity analysis for
'SSC−A on HLA DR+ NK' on 'Juvenile arthritis'

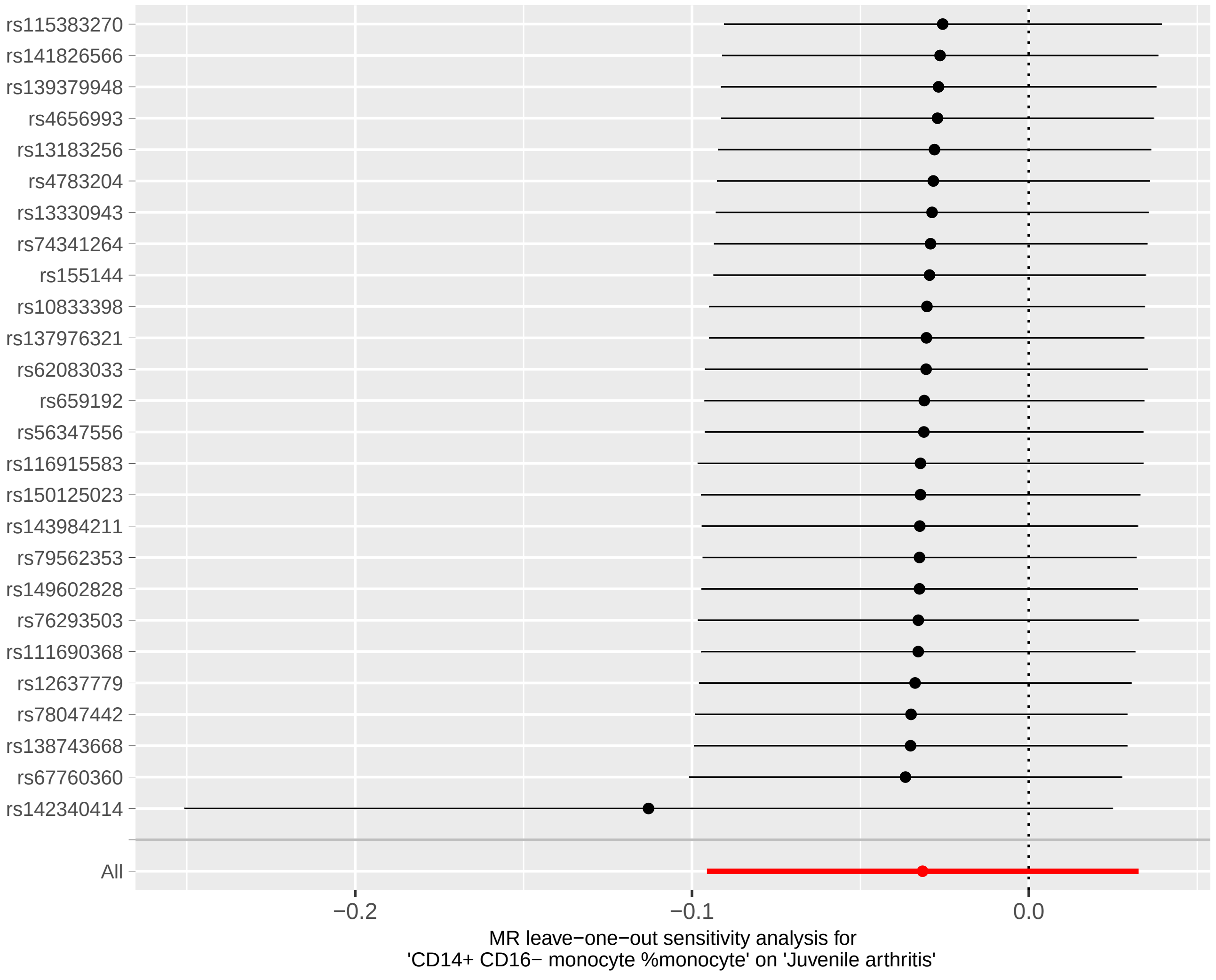

rs115383270
rs141826566
rs139379948
rs4656993
rs13183256
rs4783204
rs13330943
rs74341264
rs155144
rs10833398
rs137976321
rs62083033
rs659192
rs56347556
rs116915583
rs150125023
rs143984211
rs79562353
rs149602828
rs76293503
rs111690368
rs12637779
rs78047442
rs138743668
rs67760360
rs142340414
All
−0.2
−0.1
0.0
MR leave−one−out sensitivity analysis for
'CD14+ CD16− monocyte %monocyte' on 'Juvenile arthritis'

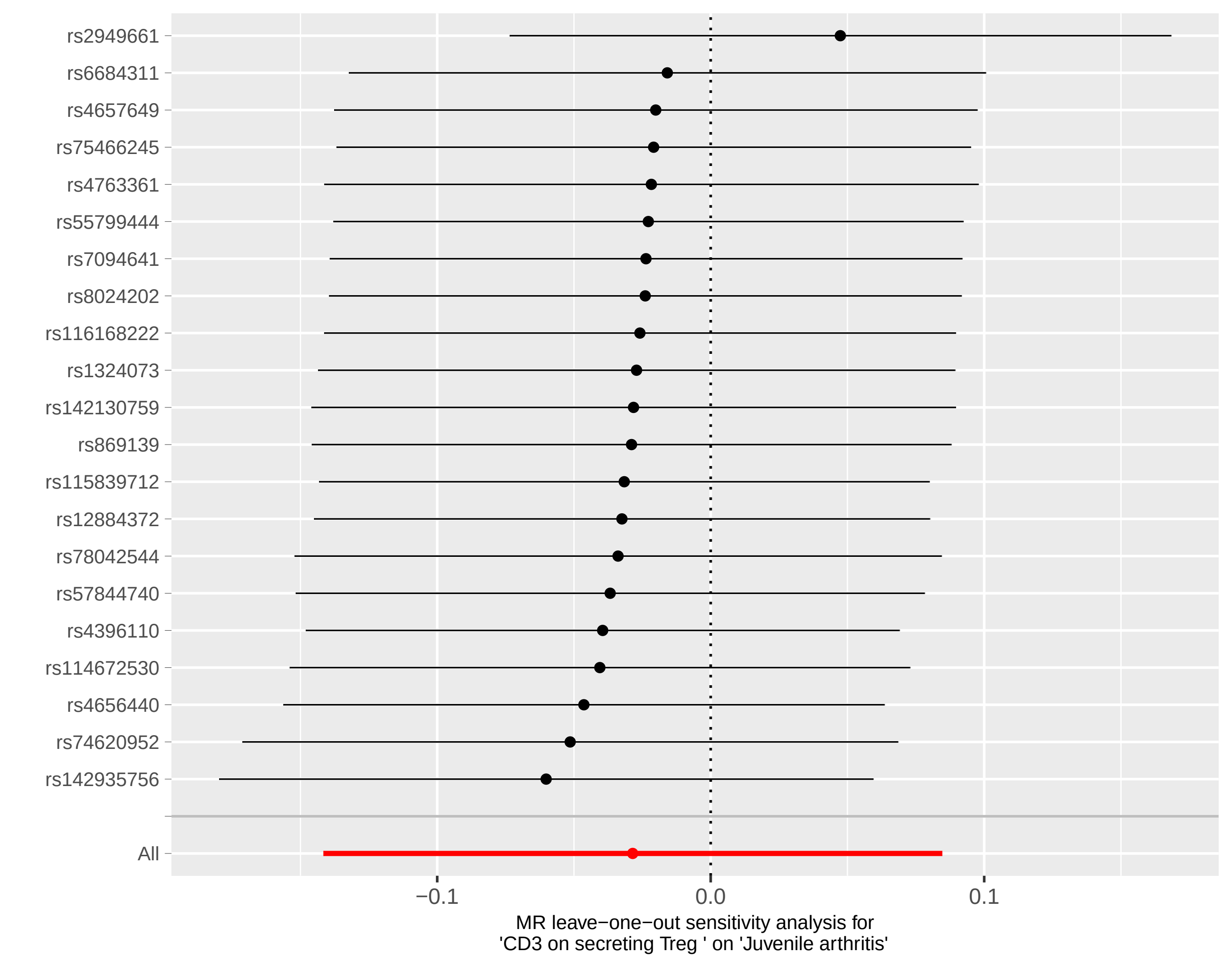
rs2949661
rs6684311
rs4657649
rs75466245
rs4763361
rs55799444
rs7094641
rs8024202
rs116168222
rs1324073
rs142130759
rs869139
rs115839712
rs12884372
rs78042544
rs57844740
rs4396110
rs114672530
rs4656440
rs74620952
rs142935756
All
−0.1
0.0
0.1
MR leave−one−out sensitivity analysis for
'CD3 on secreting Treg ' on 'Juvenile arthritis'

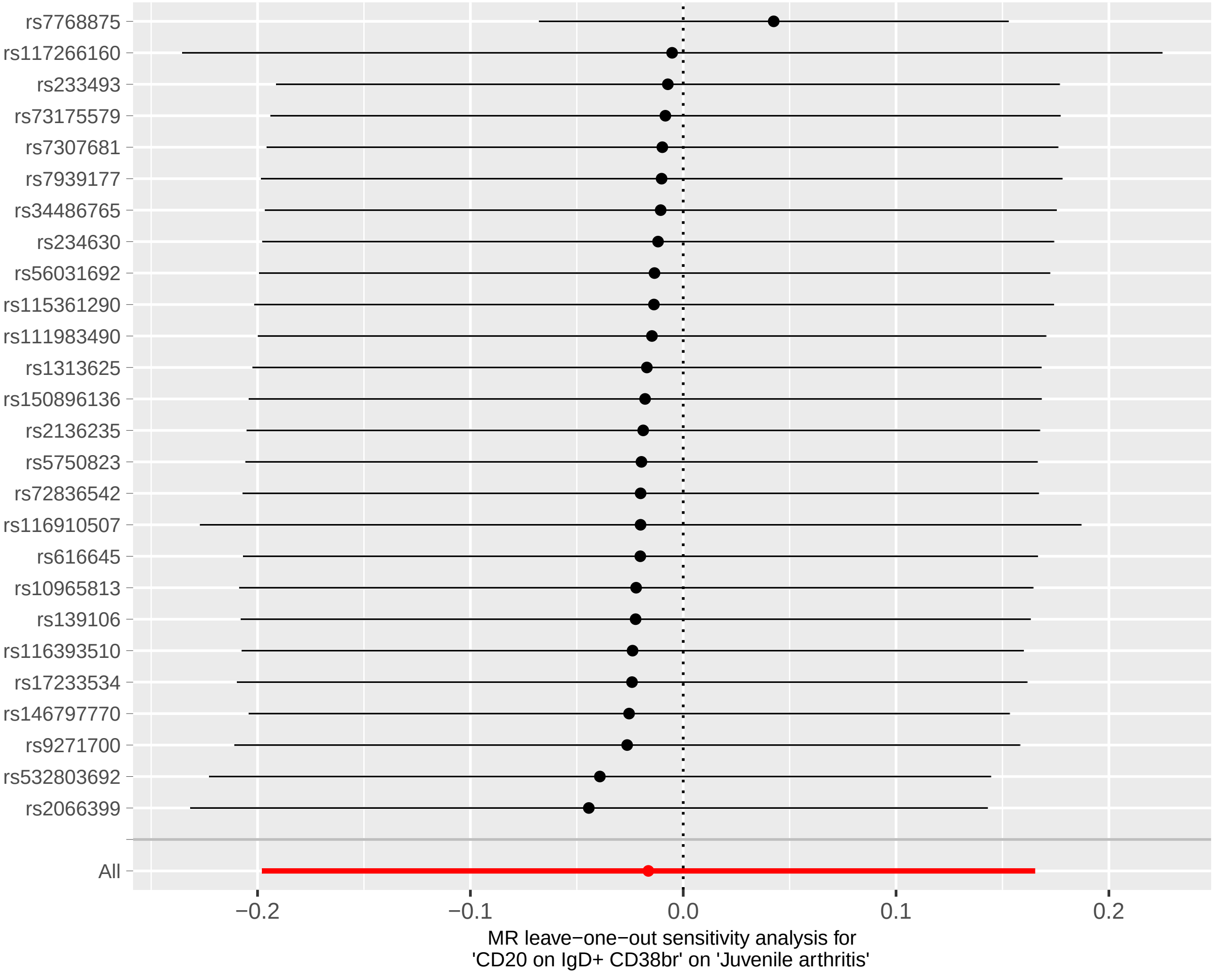
rs7768875
rs117266160
rs233493
rs73175579
rs7307681
rs7939177
rs34486765
rs234630
rs56031692
rs115361290
rs111983490
rs1313625
rs150896136
rs2136235
rs5750823
rs72836542
rs116910507
rs616645
rs10965813
rs139106
rs116393510
rs17233534
rs146797770
rs9271700
rs532803692
rs2066399
All
−0.2
−0.1
0.0
0.1
0.2
MR leave−one−out sensitivity analysis for
'CD20 on IgD+ CD38br' on 'Juvenile arthritis'

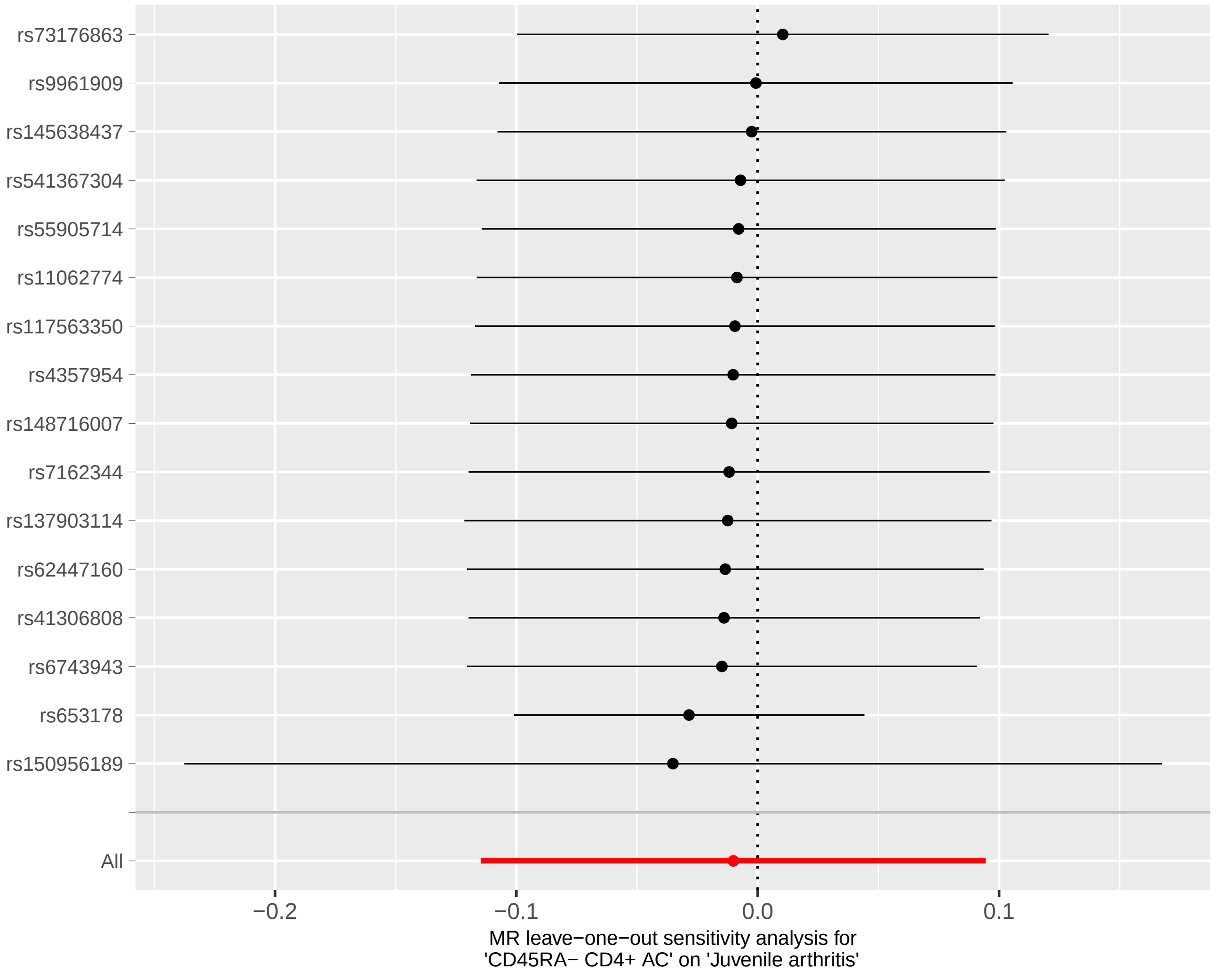
rs73176863
rs9961909
rs145638437
rs541367304
rs55905714
rs11062774
rs117563350
rs4357954
rs148716007
rs7162344
rs137903114
rs62447160
rs41306808
rs6743943
rs653178
rs150956189
All
−0.2
−0.1
0.0
0.1
MR leave−one−out sensitivity analysis for
'CD45RA− CD4+ AC' on 'Juvenile arthritis'

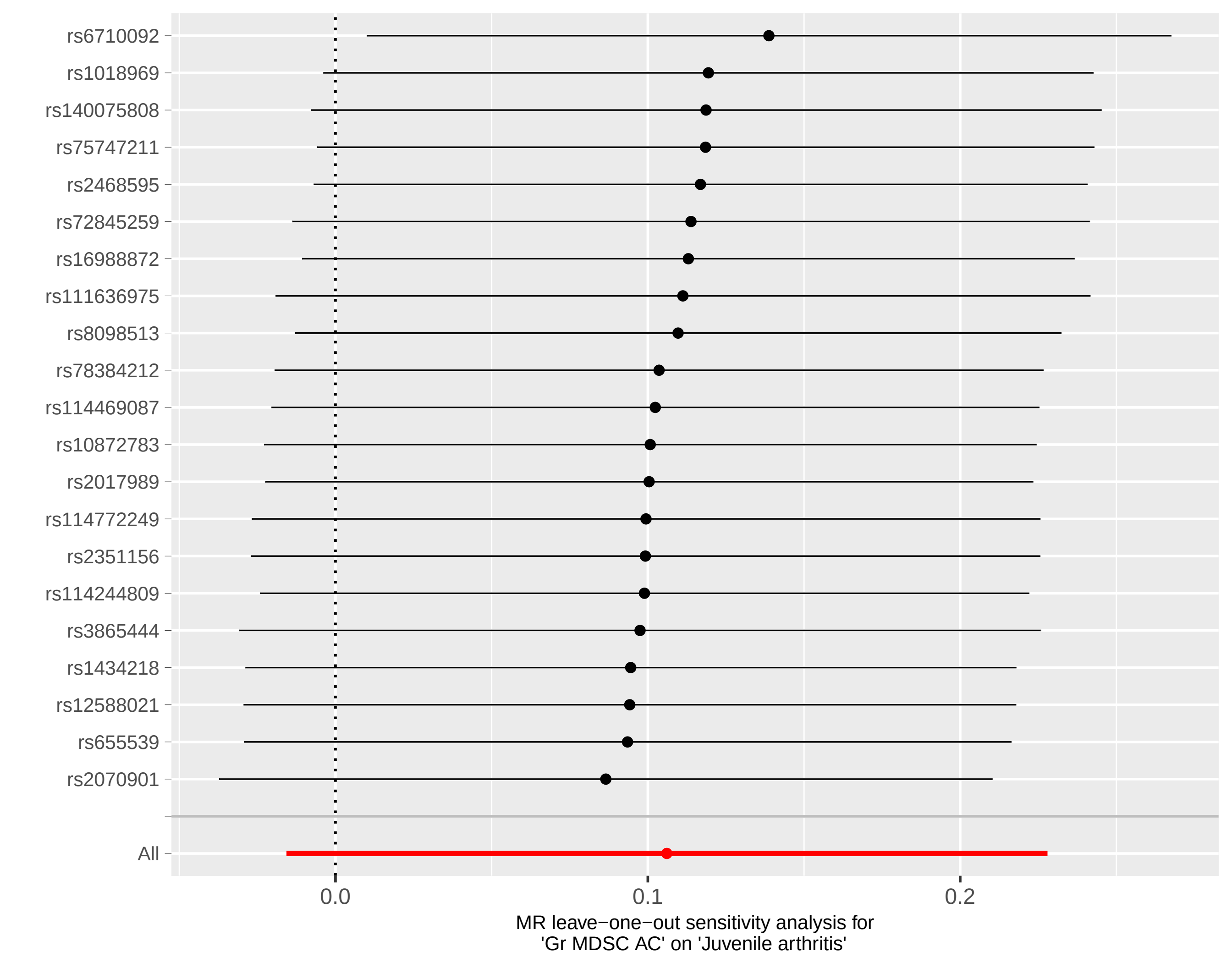
rs6710092
rs1018969
rs140075808
rs75747211
rs2468595
rs72845259
rs16988872
rs111636975
rs8098513
rs78384212
rs114469087
rs10872783
rs2017989
rs114772249
rs2351156
rs114244809
rs3865444
rs1434218
rs12588021
rs655539
rs2070901
All
0.0
0.1
0.2
MR leave−one−out sensitivity analysis for
'Gr MDSC AC' on 'Juvenile arthritis'

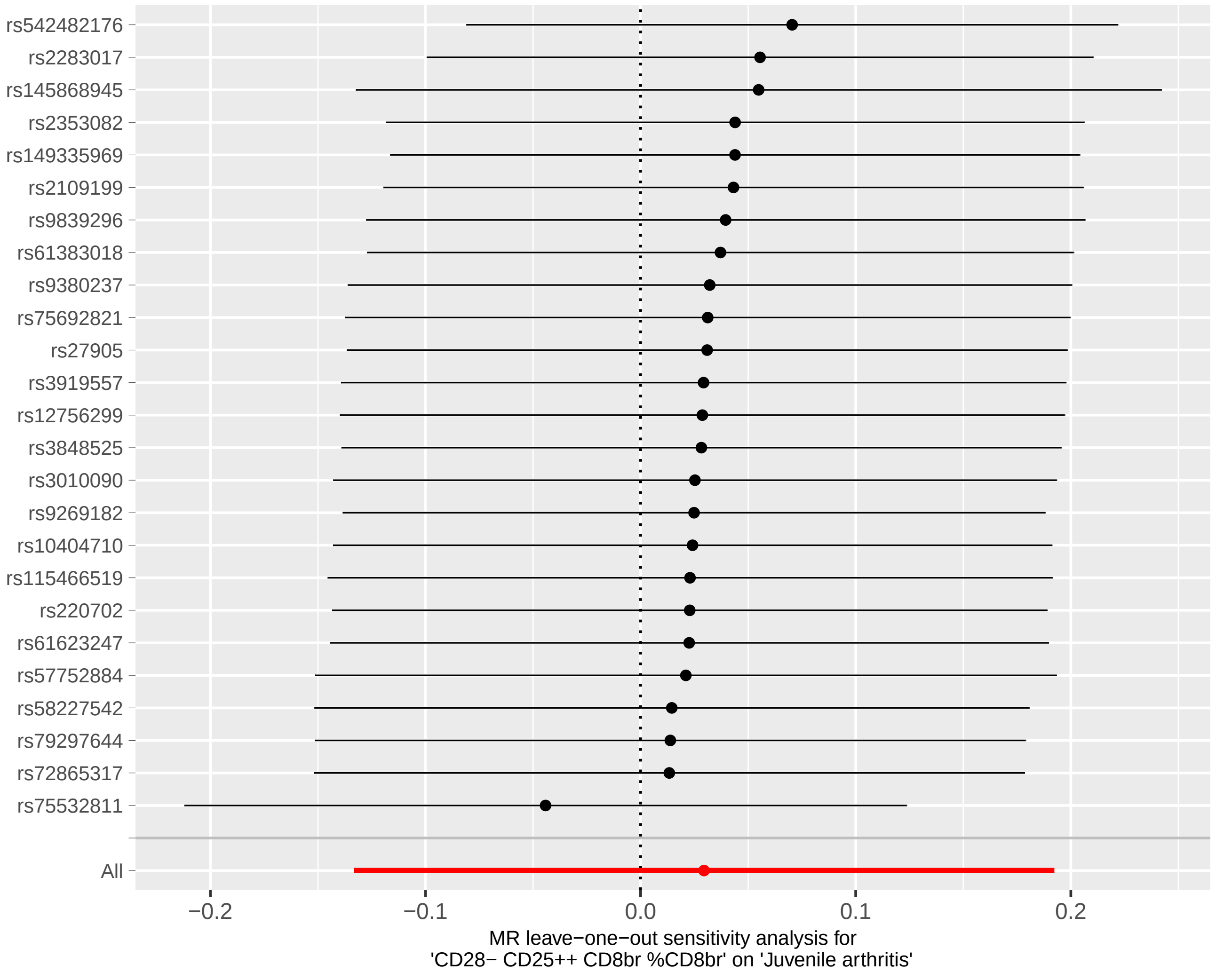

rs542482176
rs2283017
rs145868945
rs2353082
rs149335969
rs2109199
rs9839296
rs61383018
rs9380237
rs75692821
rs27905
rs3919557
rs12756299
rs3848525
rs3010090
rs9269182
rs10404710
rs115466519
rs220702
rs61623247
rs57752884
rs58227542
rs79297644
rs72865317
rs75532811
All
−0.2
−0.1
0.0
0.1
0.2
MR leave−one−out sensitivity analysis for
'CD28− CD25++ CD8br %CD8br' on 'Juvenile arthritis'

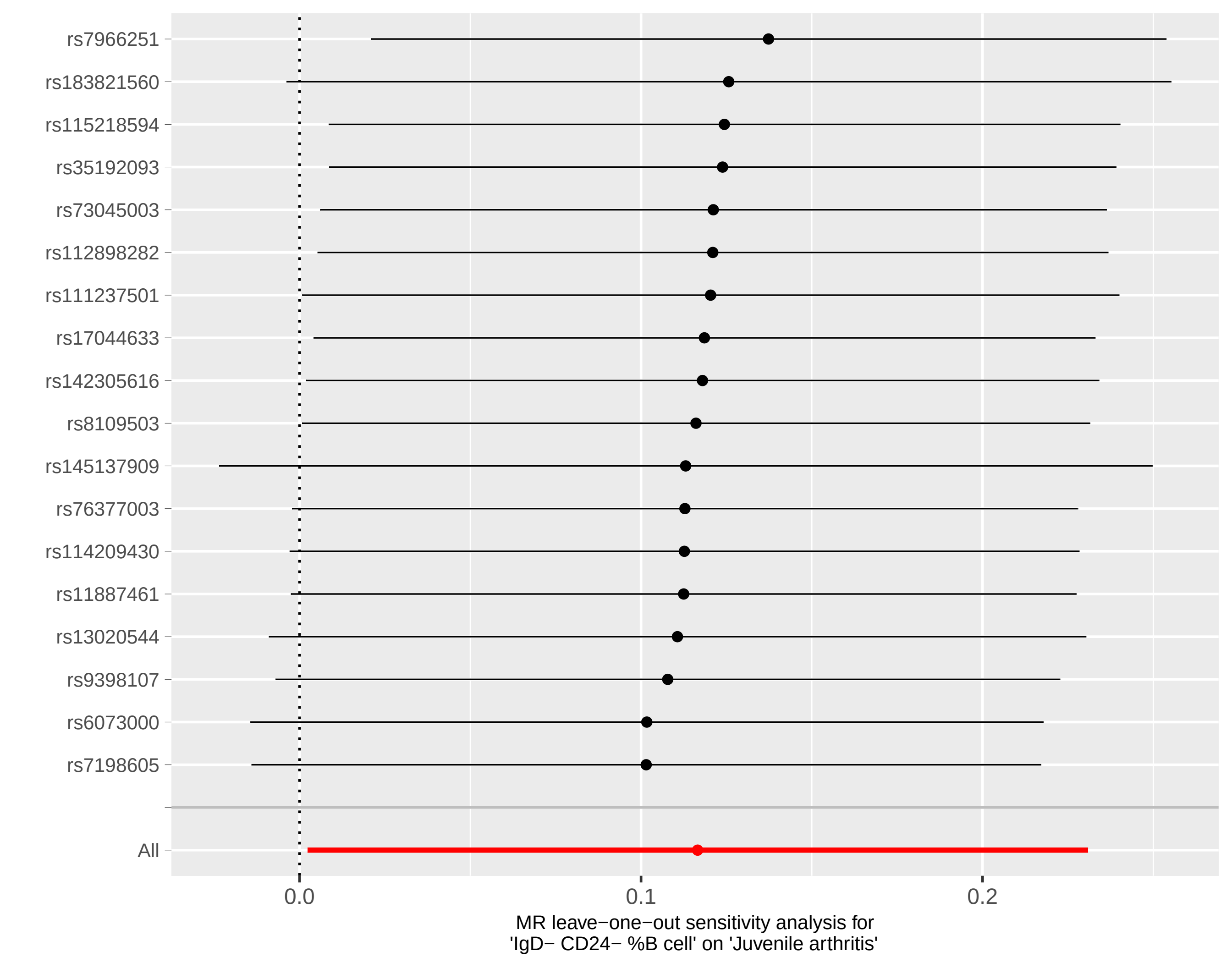
rs7966251
rs183821560
rs115218594
rs35192093
rs73045003
rs112898282
rs111237501
rs17044633
rs142305616
rs8109503
rs145137909
rs76377003
rs114209430
rs11887461
rs13020544
rs9398107
rs6073000
rs7198605
All
0.0
0.1
0.2
MR leave−one−out sensitivity analysis for
'IgD− CD24− %B cell' on 'Juvenile arthritis'

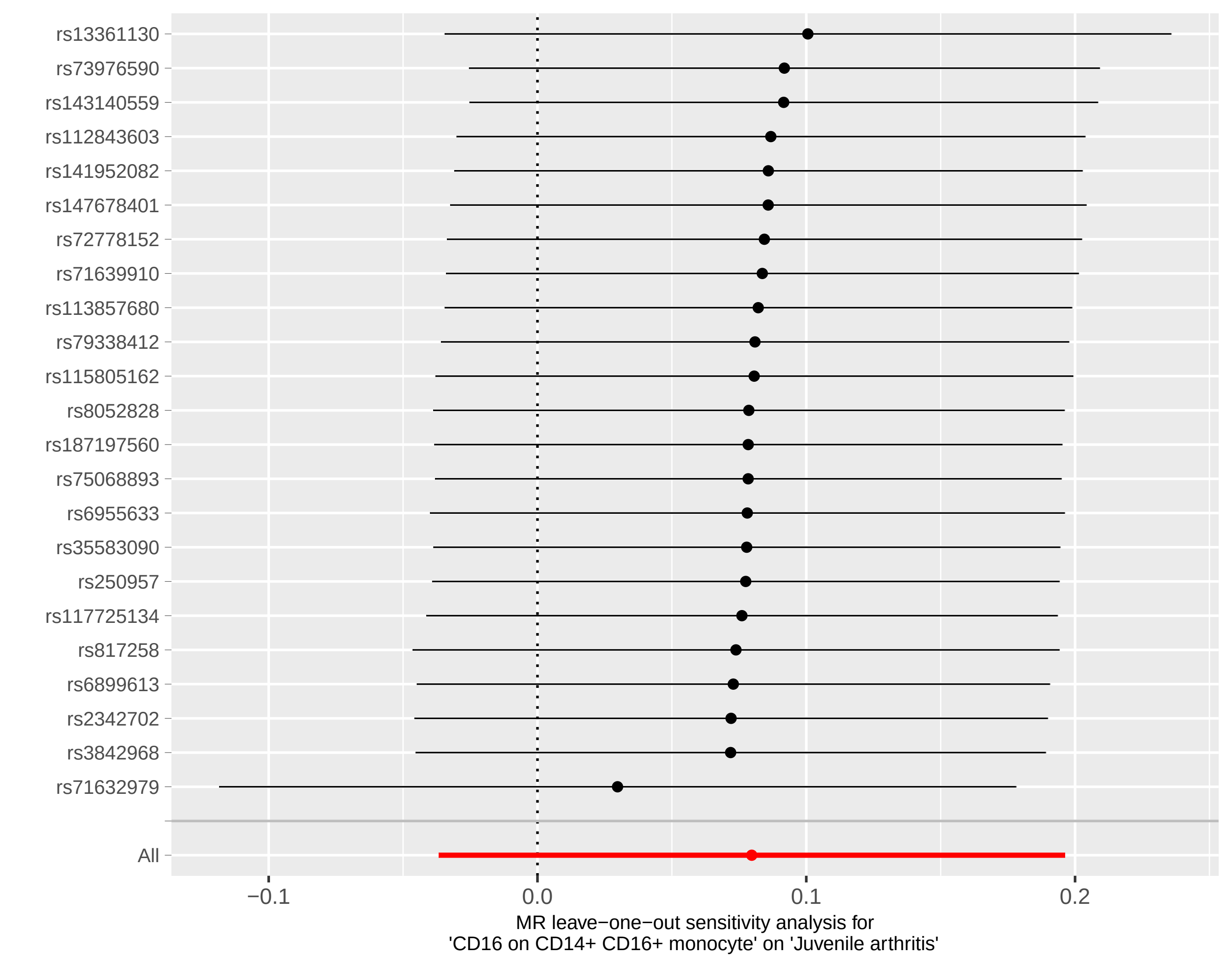
rs13361130
rs73976590
rs143140559
rs112843603
rs141952082
rs147678401
rs72778152
rs71639910
rs113857680
rs79338412
rs115805162
rs8052828
rs187197560
rs75068893
rs6955633
rs35583090
rs250957
rs117725134
rs817258
rs6899613
rs2342702
rs3842968
rs71632979
All
−0.1
0.0
0.1
0.2
MR leave−one−out sensitivity analysis for
'CD16 on CD14+ CD16+ monocyte' on 'Juvenile arthritis'

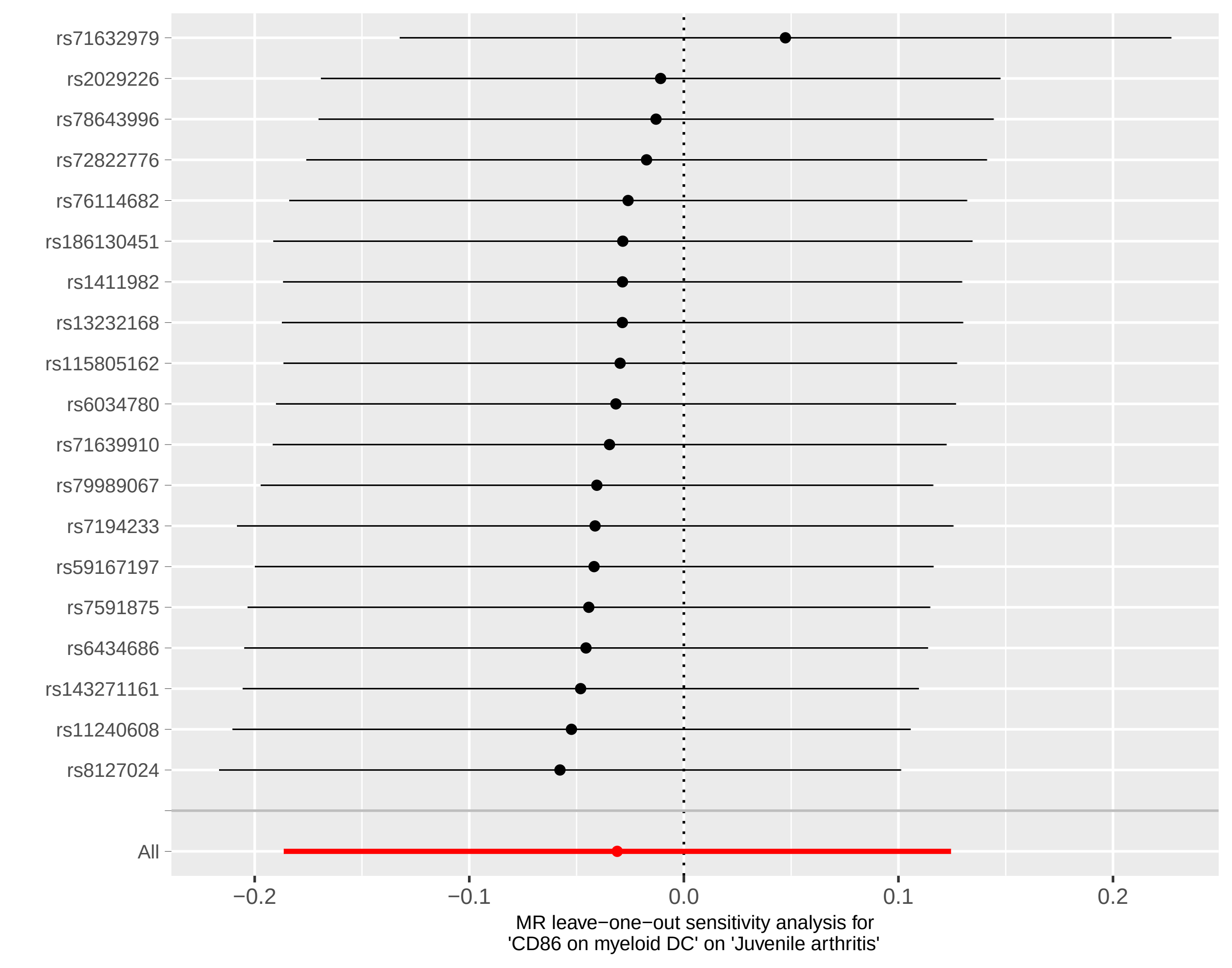
rs71632979
rs2029226
rs78643996
rs72822776
rs76114682
rs186130451
rs1411982
rs13232168
rs115805162
rs6034780
rs71639910
rs79989067
rs7194233
rs59167197
rs7591875
rs6434686
rs143271161
rs11240608
rs8127024
All
−0.2
−0.1
0.0
0.1
0.2
MR leave−one−out sensitivity analysis for
'CD86 on myeloid DC' on 'Juvenile arthritis'

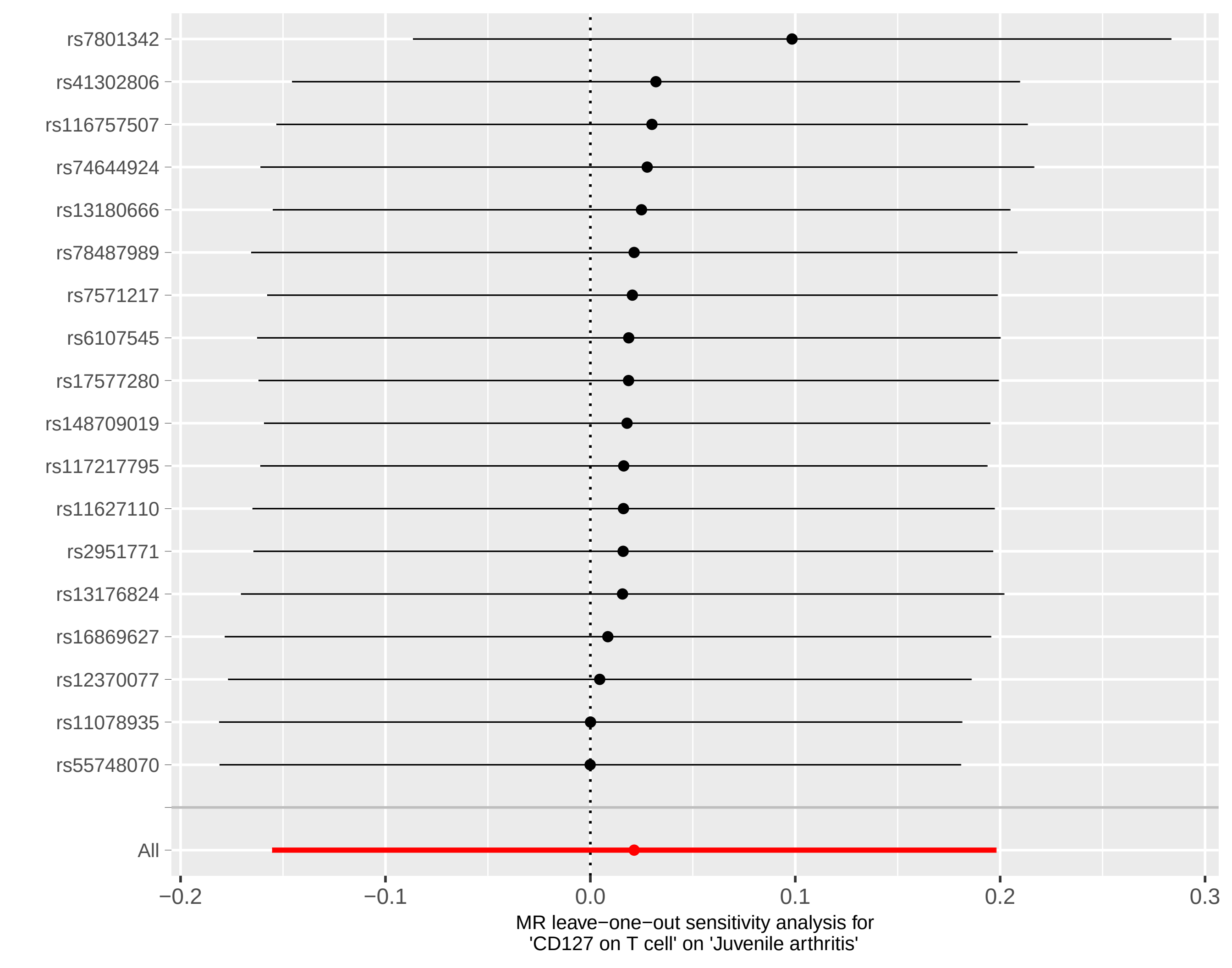

rs7801342
rs41302806
rs116757507
rs74644924
rs13180666
rs78487989
rs7571217
rs6107545
rs17577280
rs148709019
rs117217795
rs11627110
rs2951771
rs13176824
rs16869627
rs12370077
rs11078935
rs55748070
All
−0.2
−0.1
0.0
0.1
0.2
0.3
MR leave−one−out sensitivity analysis for
'CD127 on T cell' on 'Juvenile arthritis'

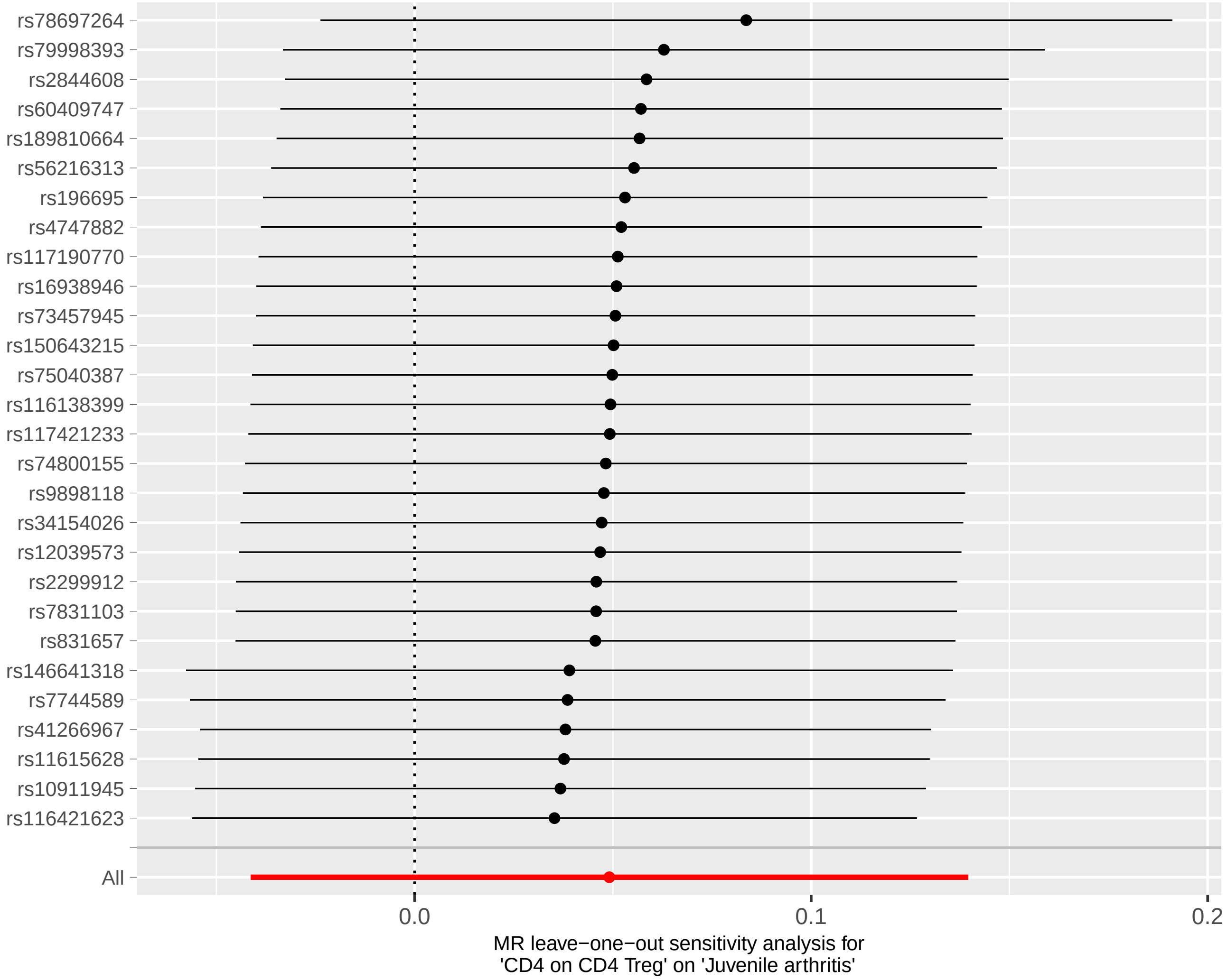
rs78697264
rs79998393
rs2844608
rs60409747
rs189810664
rs56216313
rs196695
rs4747882
rs117190770
rs16938946
rs73457945
rs150643215
rs75040387
rs116138399
rs117421233
rs74800155
rs9898118
rs34154026
rs12039573
rs2299912
rs7831103
rs831657
rs146641318
rs7744589
rs41266967
rs11615628
rs10911945
rs116421623
All
0.0
0.1
0.2
MR leave−one−out sensitivity analysis for
'CD4 on CD4 Treg' on 'Juvenile arthritis'

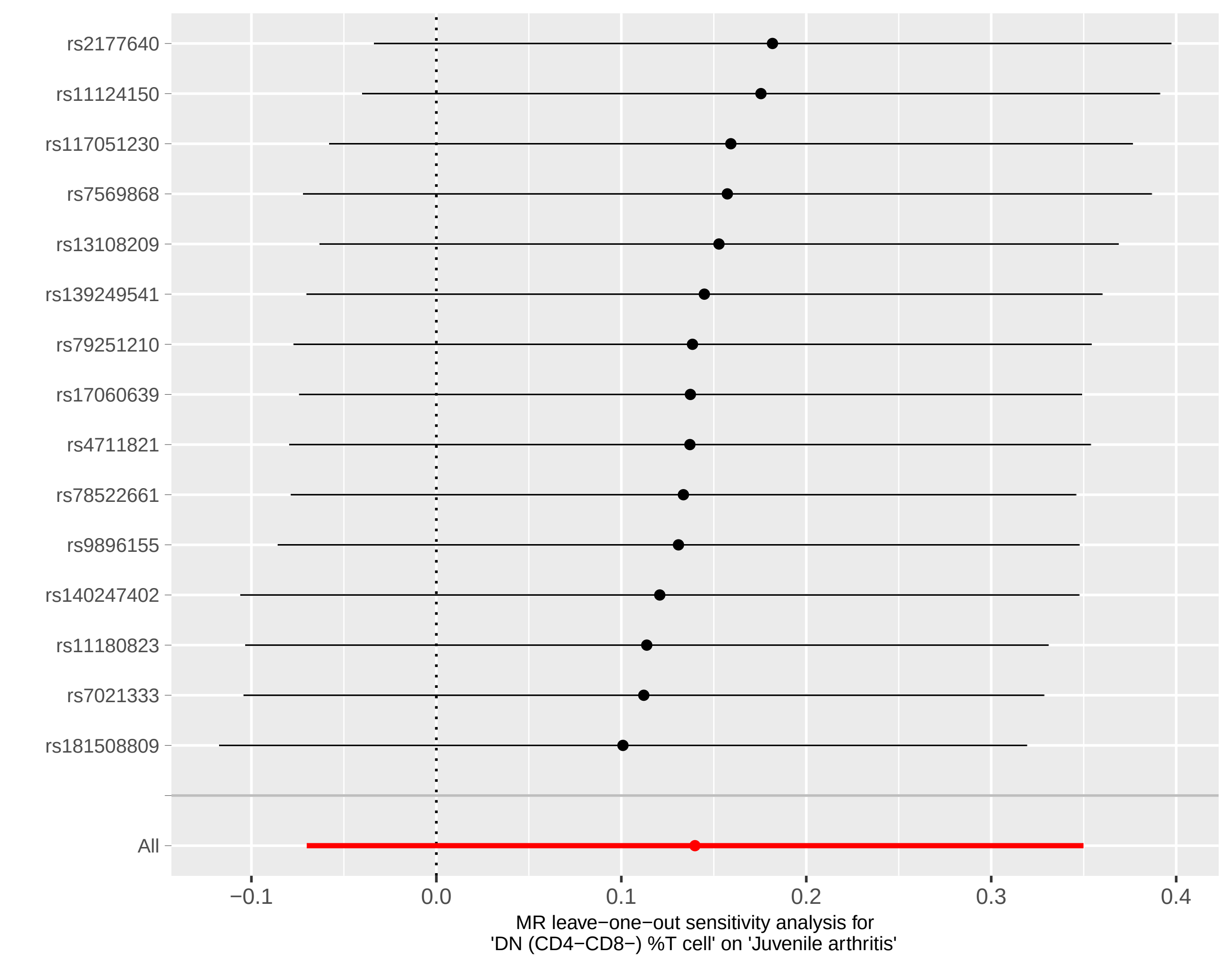
rs2177640
rs11124150
rs117051230
rs7569868
rs13108209
rs139249541
rs79251210
rs17060639
rs4711821
rs78522661
rs9896155
rs140247402
rs11180823
rs7021333
rs181508809
All
−0.1
0.0
0.1
0.2
0.3
0.4
MR leave−one−out sensitivity analysis for
'DN (CD4−CD8−) %T cell' on 'Juvenile arthritis'

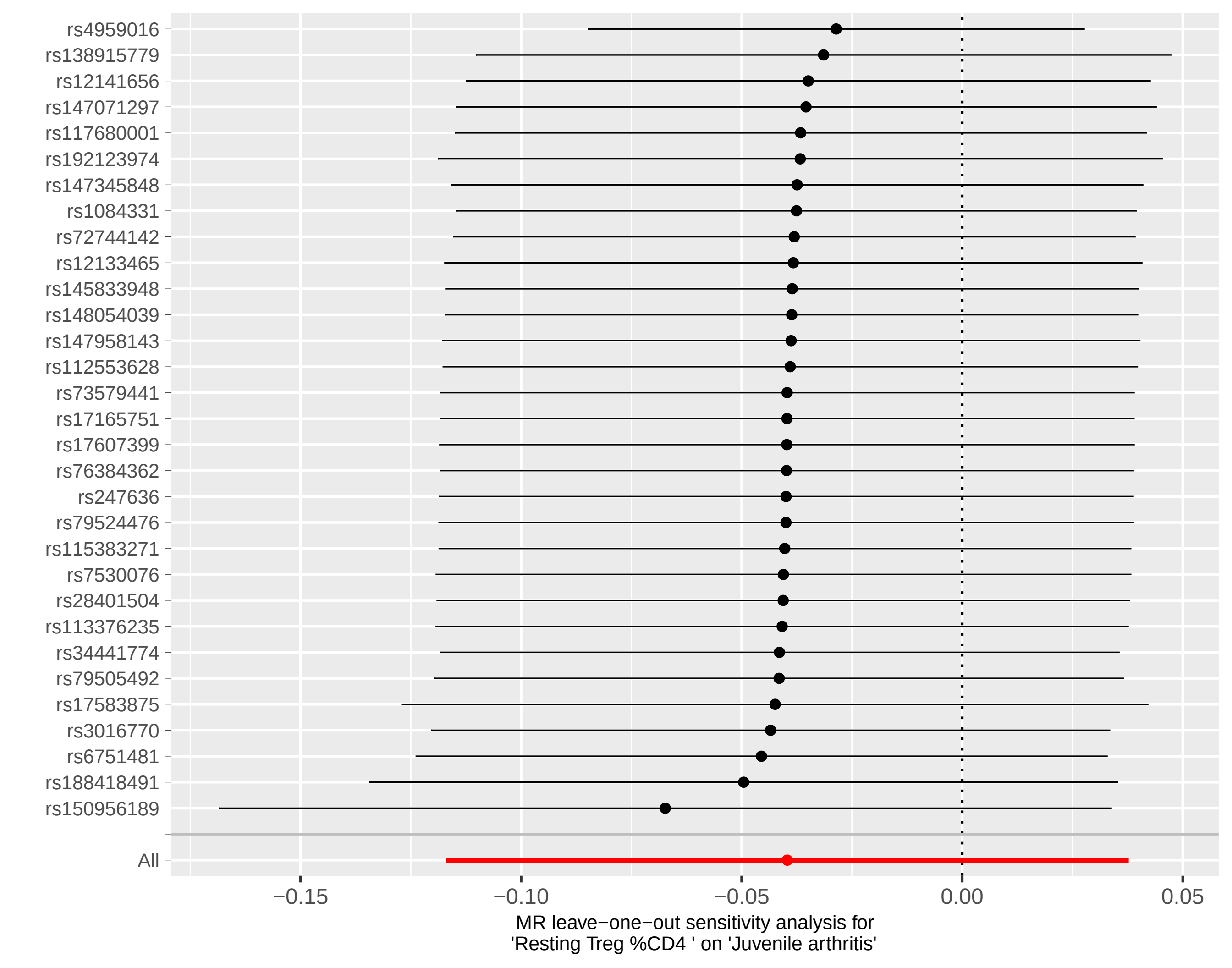
rs4959016
rs138915779
rs12141656
rs147071297
rs117680001
rs192123974
rs147345848
rs1084331
rs72744142
rs12133465
rs145833948
rs148054039
rs147958143
rs112553628
rs73579441
rs17165751
rs17607399
rs76384362
rs247636
rs79524476
rs115383271
rs7530076
rs28401504
rs113376235
rs34441774
rs79505492
rs17583875
rs3016770
rs6751481
rs188418491
rs150956189
All
−0.15
−0.10
−0.05
0.00
0.05
MR leave−one−out sensitivity analysis for
'Resting Treg %CD4 ' on 'Juvenile arthritis'

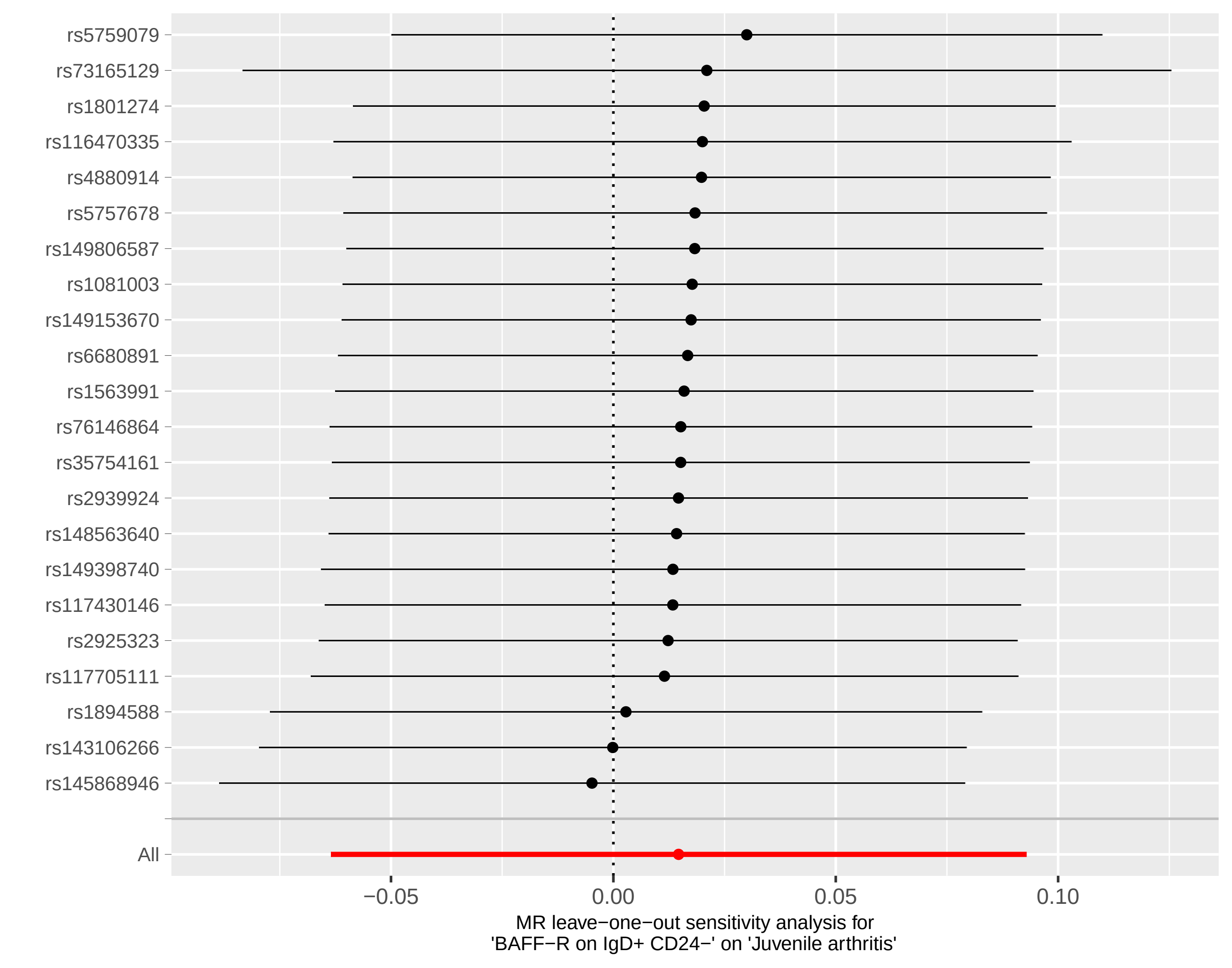
rs5759079
rs73165129
rs1801274
rs116470335
rs4880914
rs5757678
rs149806587
rs1081003
rs149153670
rs6680891
rs1563991
rs76146864
rs35754161
rs2939924
rs148563640
rs149398740
rs117430146
rs2925323
rs117705111
rs1894588
rs143106266
rs145868946
All
−0.05
0.00
0.05
0.10
MR leave−one−out sensitivity analysis for
'BAFF−R on IgD+ CD24−' on 'Juvenile arthritis'

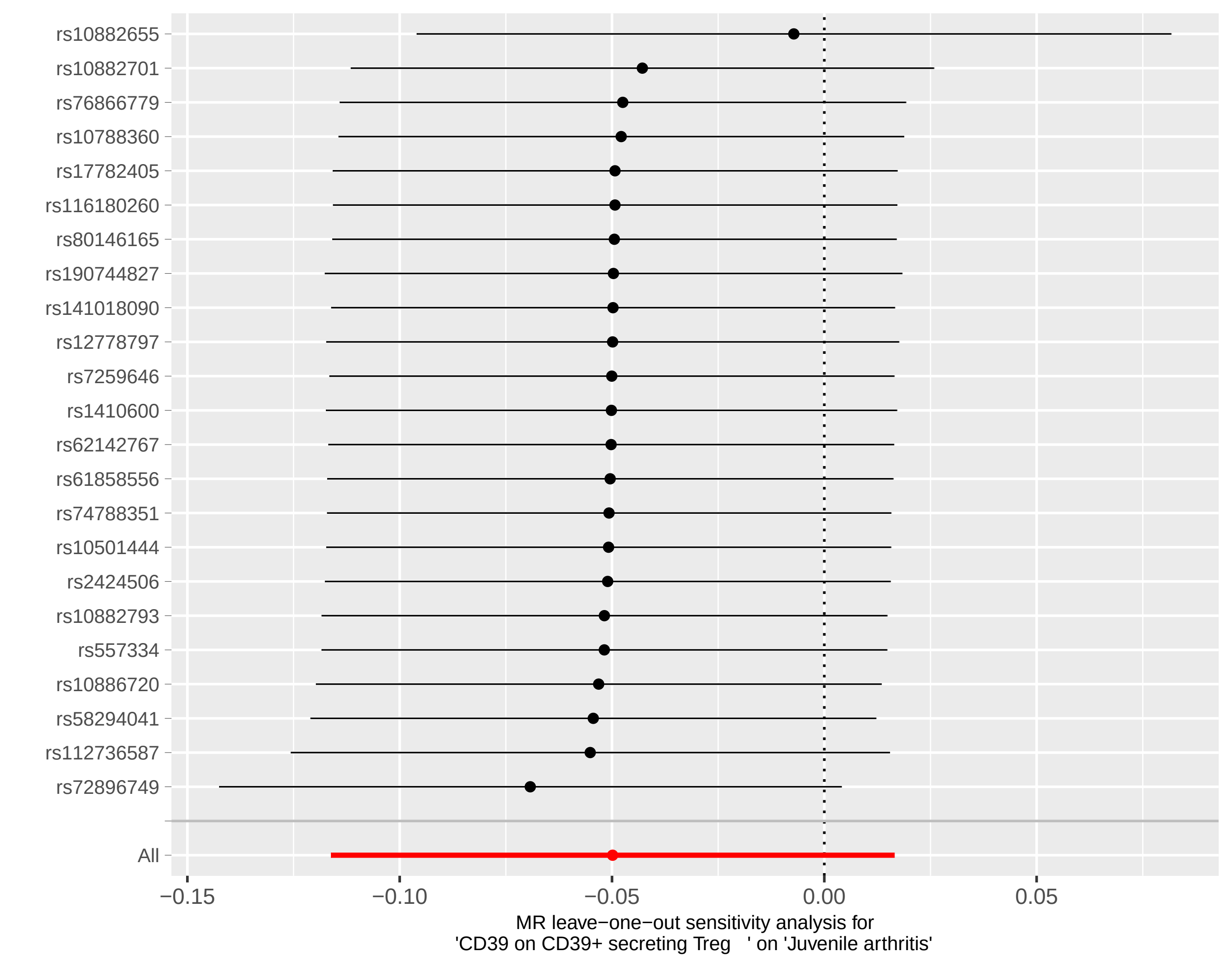

rs10882655
rs10882701
rs76866779
rs10788360
rs17782405
rs116180260
rs80146165
rs190744827
rs141018090
rs12778797
rs7259646
rs1410600
rs62142767
rs61858556
rs74788351
rs10501444
rs2424506
rs10882793
rs557334
rs10886720
rs58294041
rs112736587
rs72896749
All
−0.15
−0.10
−0.05
0.00
0.05
MR leave−one−out sensitivity analysis for
'CD39 on CD39+ secreting Treg ' on 'Juvenile arthritis'

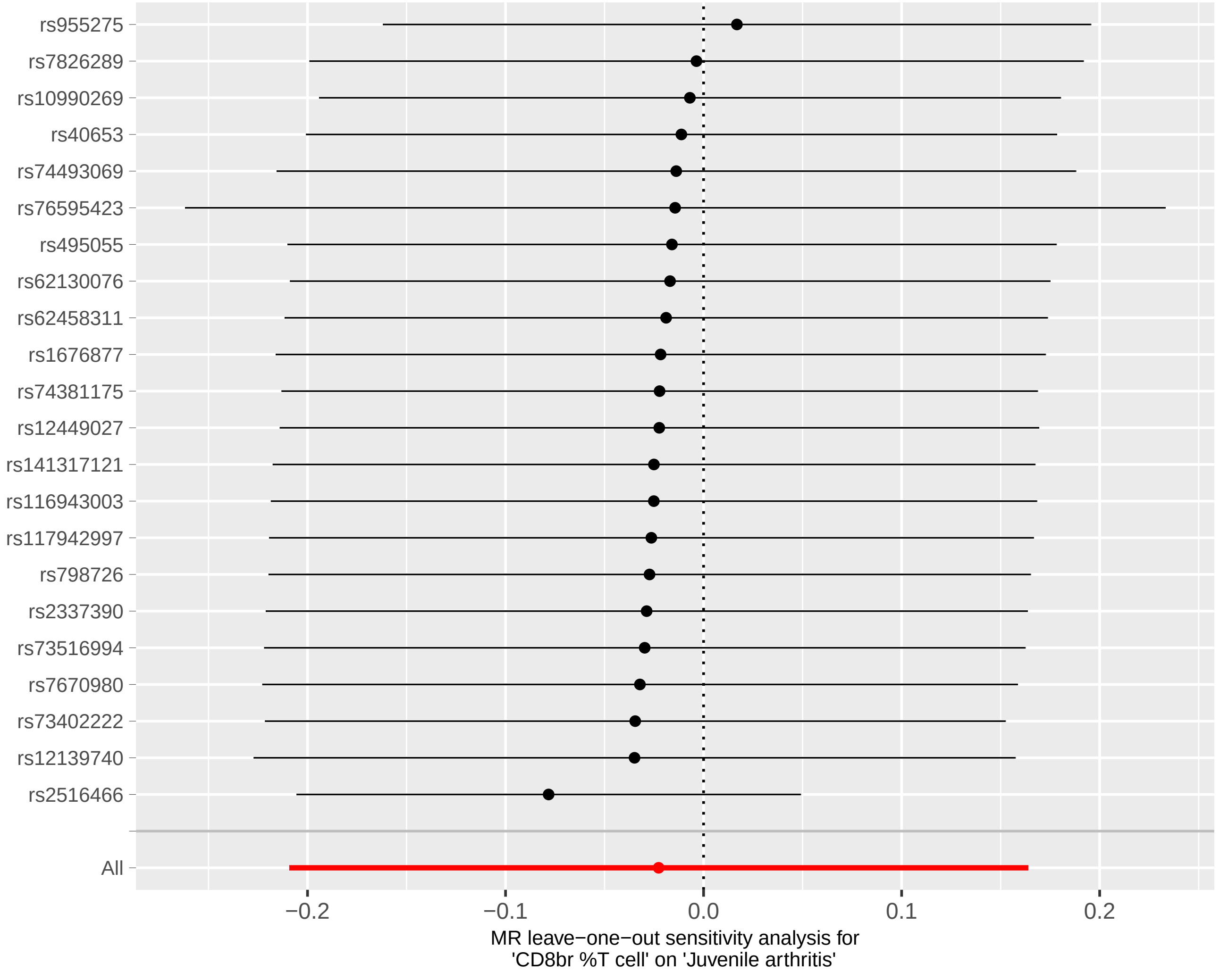

rs955275
rs7826289
rs10990269
rs40653
rs74493069
rs76595423
rs495055
rs62130076
rs62458311
rs1676877
rs74381175
rs12449027
rs141317121
rs116943003
rs117942997
rs798726
rs2337390
rs73516994
rs7670980
rs73402222
rs12139740
rs2516466
All
−0.2
−0.1
0.0
0.1
0.2
MR leave−one−out sensitivity analysis for
'CD8br %T cell' on 'Juvenile arthritis'

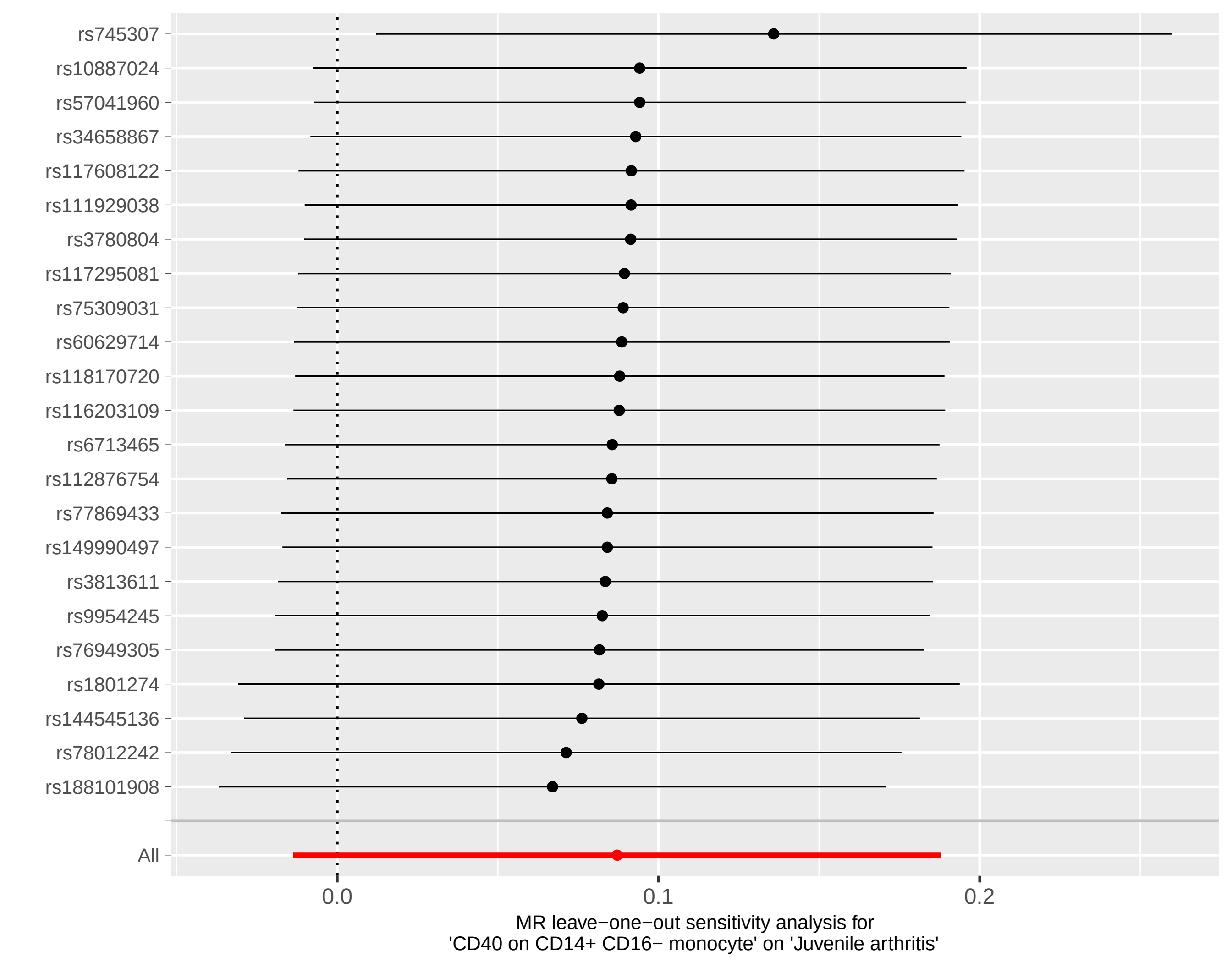
rs745307
rs10887024
rs57041960
rs34658867
rs117608122
rs111929038
rs3780804
rs117295081
rs75309031
rs60629714
rs118170720
rs116203109
rs6713465
rs112876754
rs77869433
rs149990497
rs3813611
rs9954245
rs76949305
rs1801274
rs144545136
rs78012242
rs188101908
All
0.0
0.1
0.2
MR leave−one−out sensitivity analysis for
'CD40 on CD14+ CD16− monocyte' on 'Juvenile arthritis'

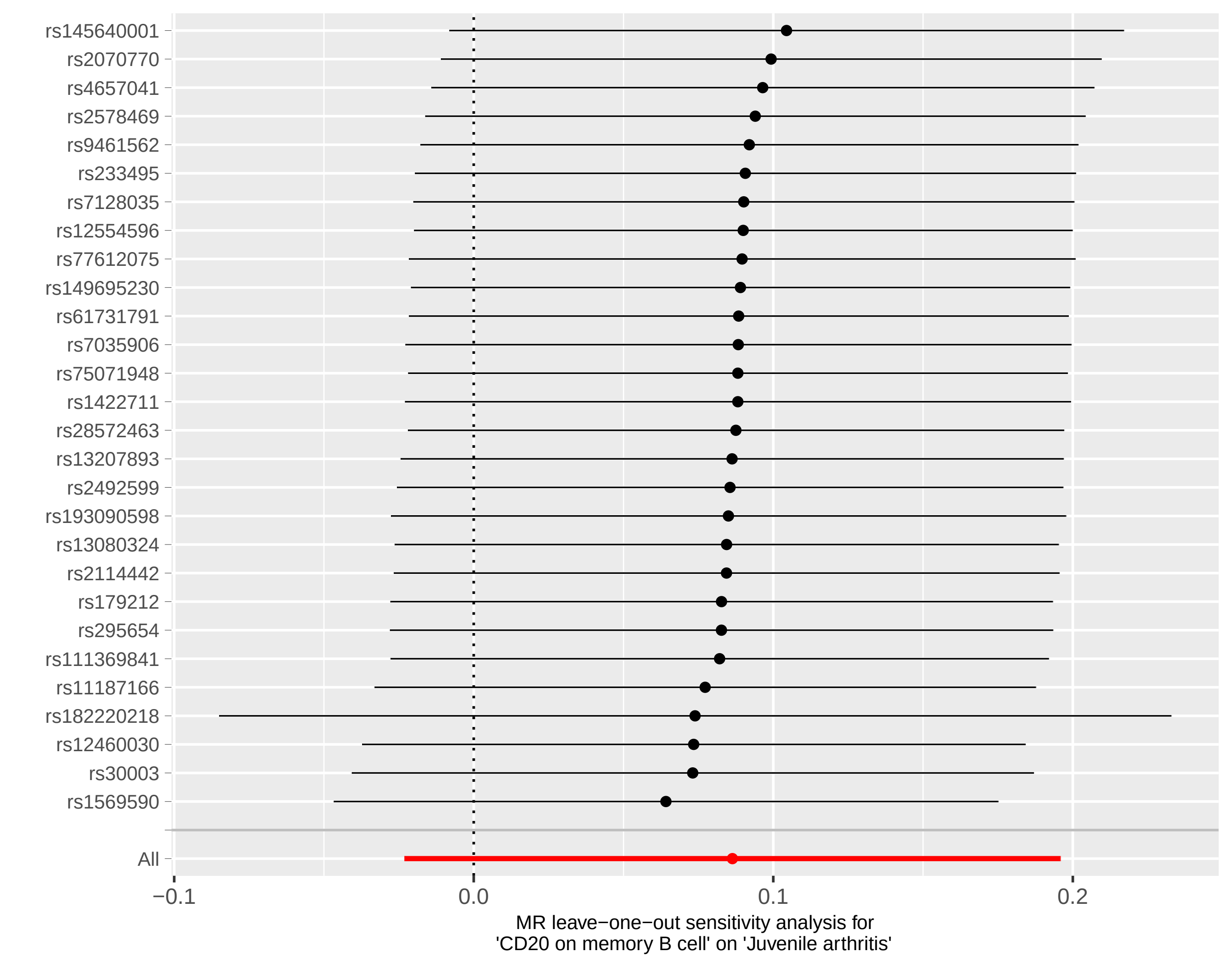

rs145640001
rs2070770
rs4657041
rs2578469
rs9461562
rs233495
rs7128035
rs12554596
rs77612075
rs149695230
rs61731791
rs7035906
rs75071948
rs1422711
rs28572463
rs13207893
rs2492599
rs193090598
rs13080324
rs2114442
rs179212
rs295654
rs111369841
rs11187166
rs182220218
rs12460030
rs30003
rs1569590
All
−0.1
0.0
0.1
0.2
MR leave−one−out sensitivity analysis for
'CD20 on memory B cell' on 'Juvenile arthritis'

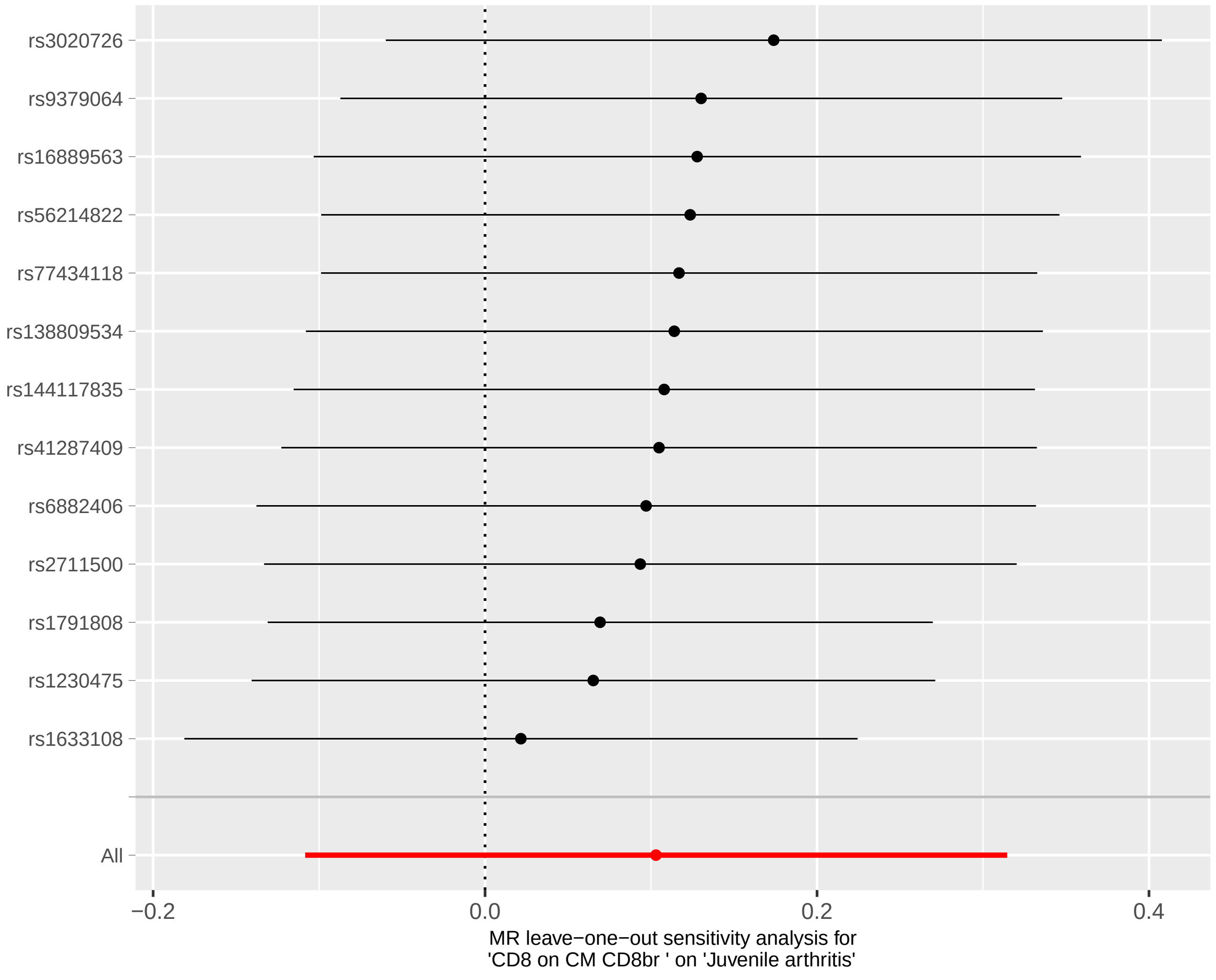
rs3020726
rs9379064
rs16889563
rs56214822
rs77434118
rs138809534
rs144117835
rs41287409
rs6882406
rs2711500
rs1791808
rs1230475
rs1633108
All
−0.2
0.0
0.2
0.4
MR leave−one−out sensitivity analysis for
'CD8 on CM CD8br ' on 'Juvenile arthritis'

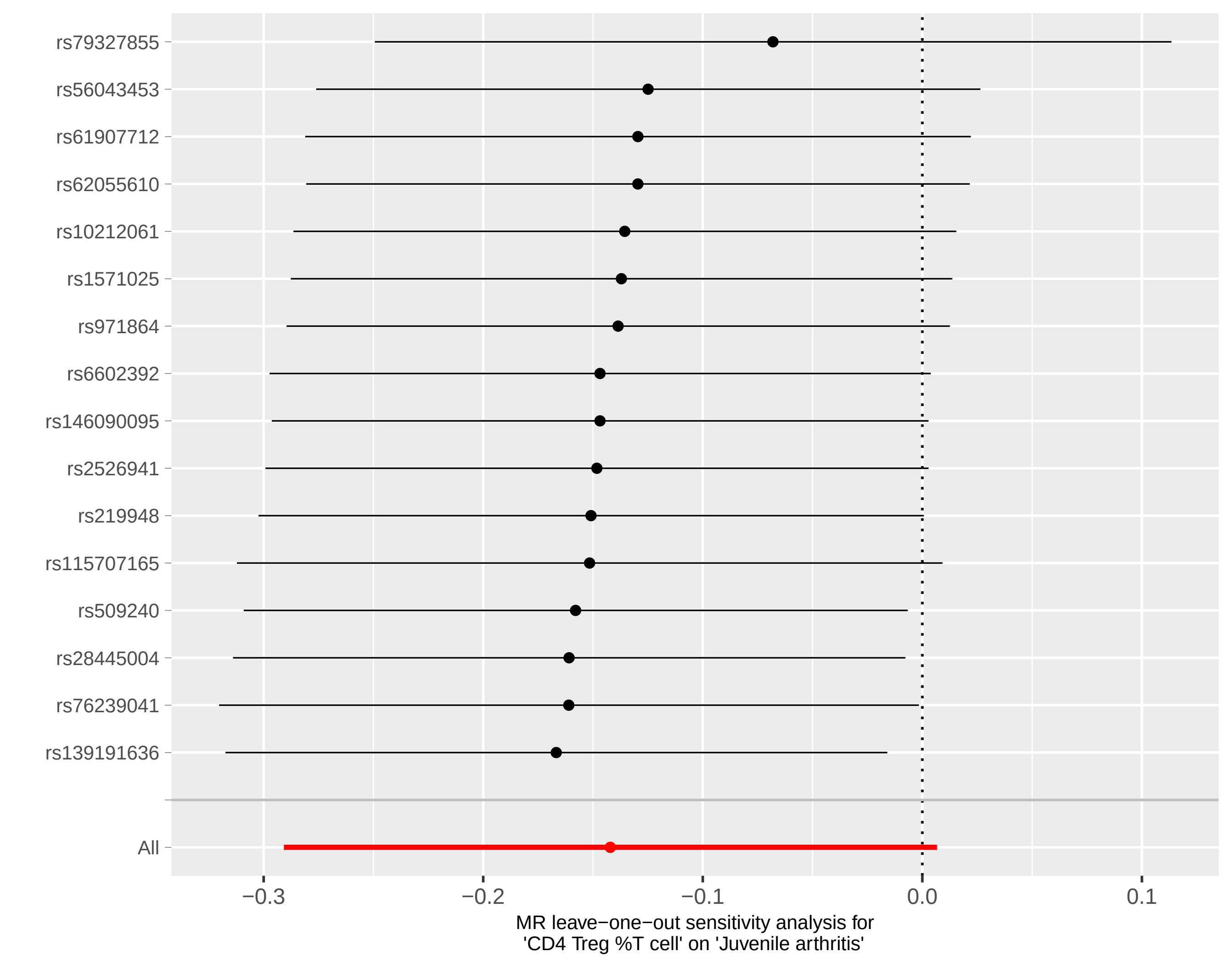
rs79327855
rs56043453
rs61907712
rs62055610
rs10212061
rs1571025
rs971864
rs6602392
rs146090095
rs2526941
rs219948
rs115707165
rs509240
rs28445004
rs76239041
rs139191636
All
−0.3
−0.2
−0.1
0.0
0.1
MR leave−one−out sensitivity analysis for
'CD4 Treg %T cell' on 'Juvenile arthritis'

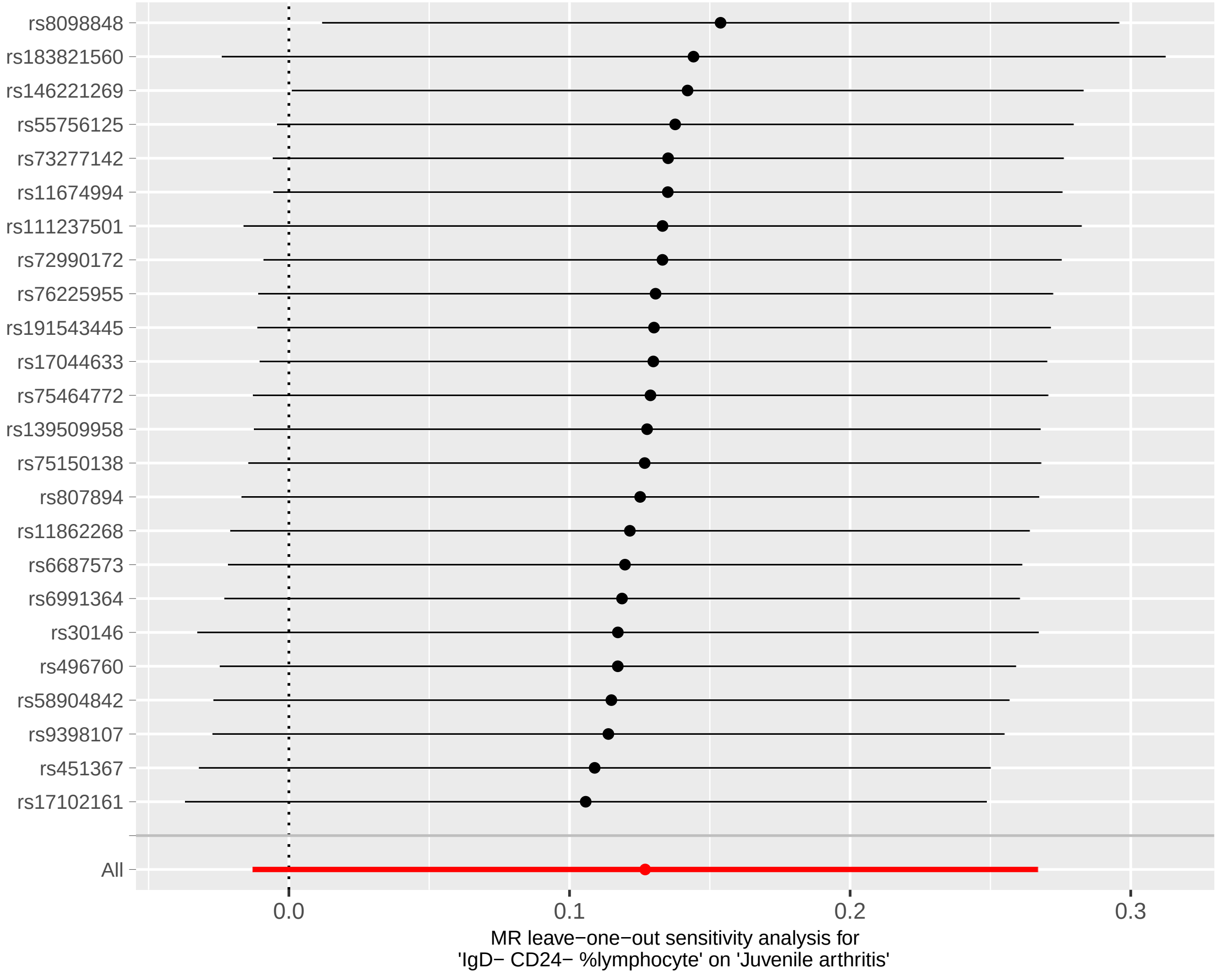
rs8098848
rs183821560
rs146221269
rs55756125
rs73277142
rs11674994
rs111237501
rs72990172
rs76225955
rs191543445
rs17044633
rs75464772
rs139509958
rs75150138
rs807894
rs11862268
rs6687573
rs6991364
rs30146
rs496760
rs58904842
rs9398107
rs451367
rs17102161
All
0.0
0.1
0.2
0.3
MR leave−one−out sensitivity analysis for
'IgD− CD24− %lymphocyte' on 'Juvenile arthritis'

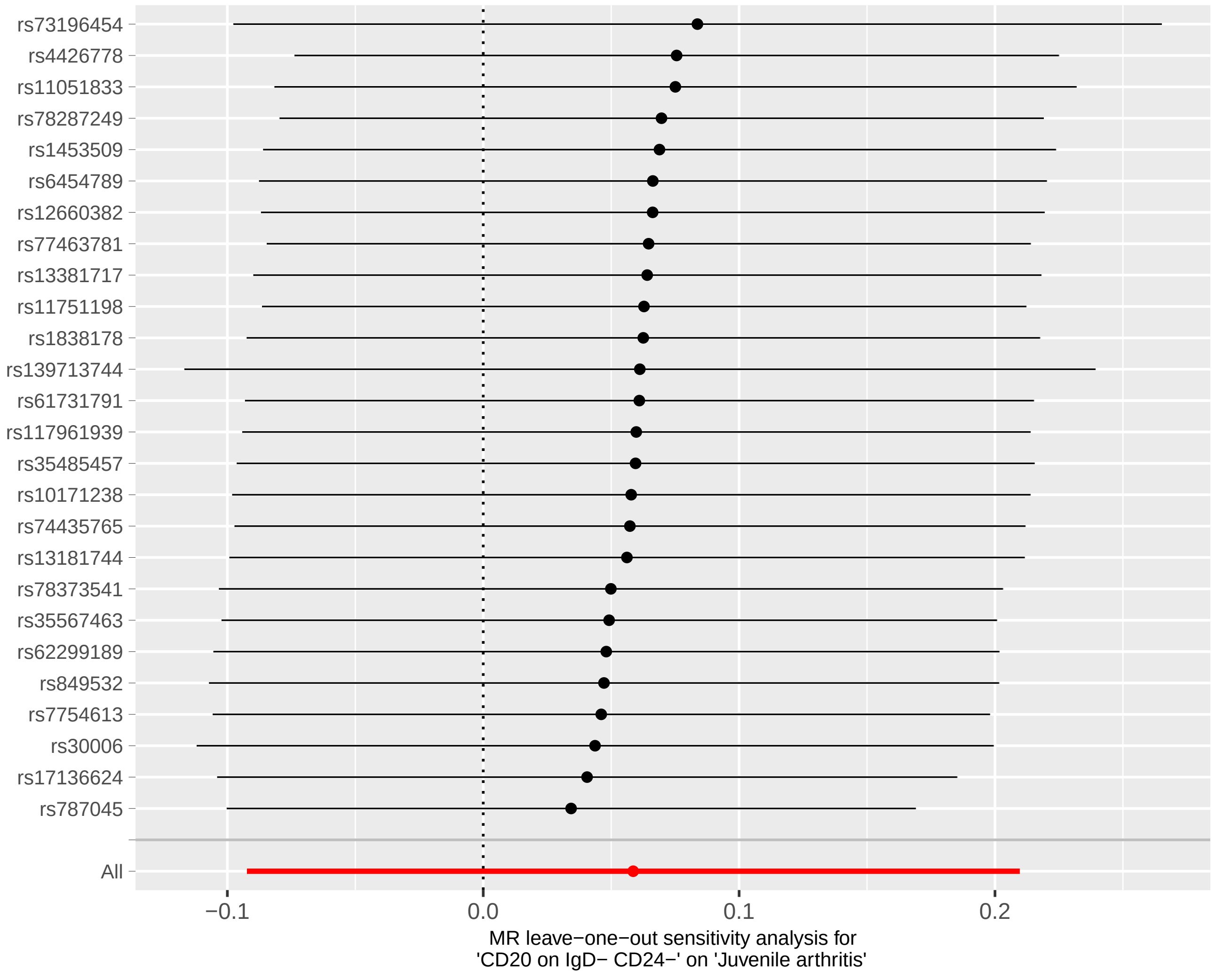
rs73196454
rs4426778
rs11051833
rs78287249
rs1453509
rs6454789
rs12660382
rs77463781
rs13381717
rs11751198
rs1838178
rs139713744
rs61731791
rs117961939
rs35485457
rs10171238
rs74435765
rs13181744
rs78373541
rs35567463
rs62299189
rs849532
rs7754613
rs30006
rs17136624
rs787045
All
−0.1
0.0
0.1
0.2
MR leave−one−out sensitivity analysis for
'CD20 on IgD− CD24−' on 'Juvenile arthritis'

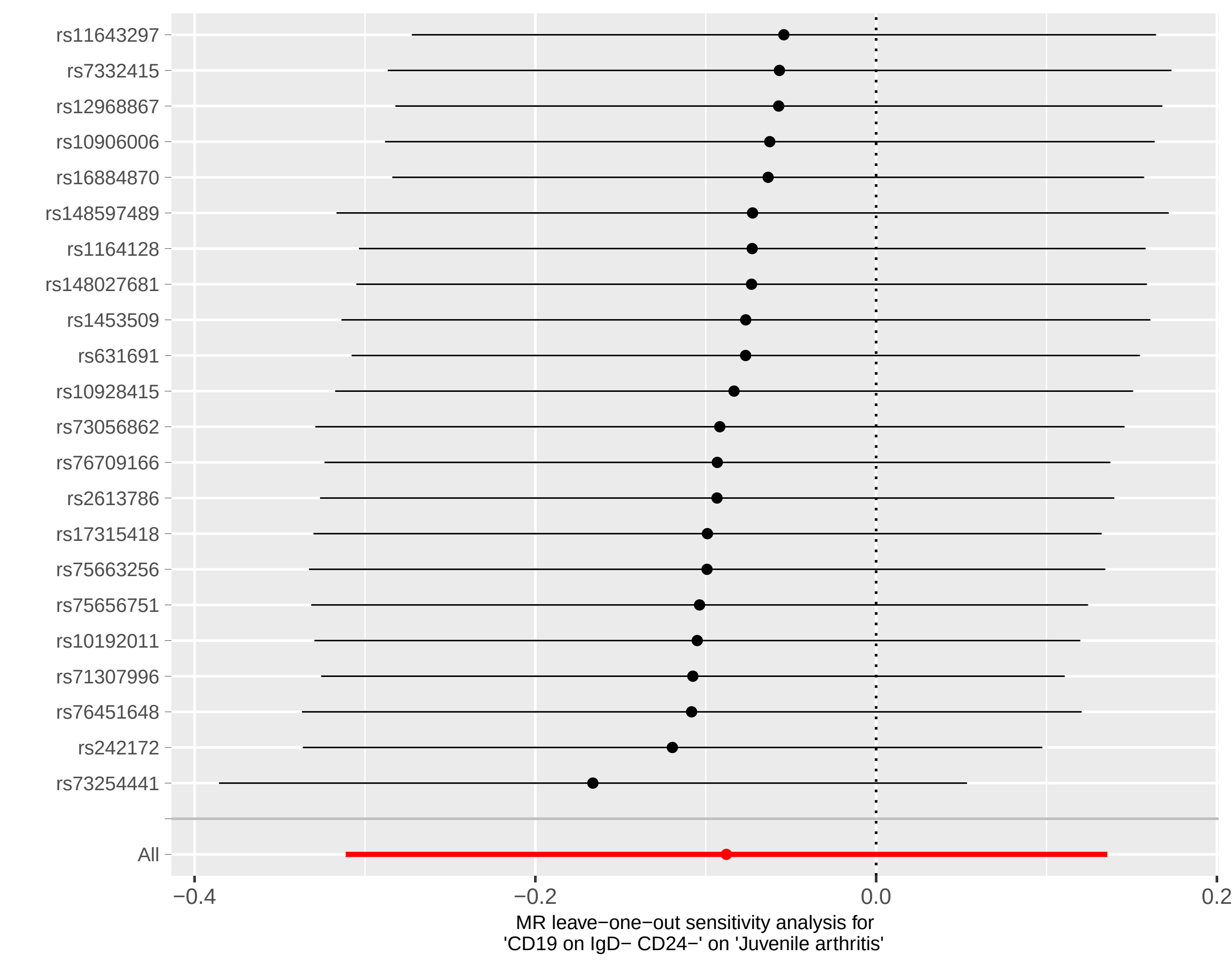
rs11643297
rs7332415
rs12968867
rs10906006
rs16884870
rs148597489
rs1164128
rs148027681
rs1453509
rs631691
rs10928415
rs73056862
rs76709166
rs2613786
rs17315418
rs75663256
rs75656751
rs10192011
rs71307996
rs76451648
rs242172
rs73254441
All
−0.4
−0.2
0.0
0.2
MR leave−one−out sensitivity analysis for
'CD19 on IgD− CD24−' on 'Juvenile arthritis'

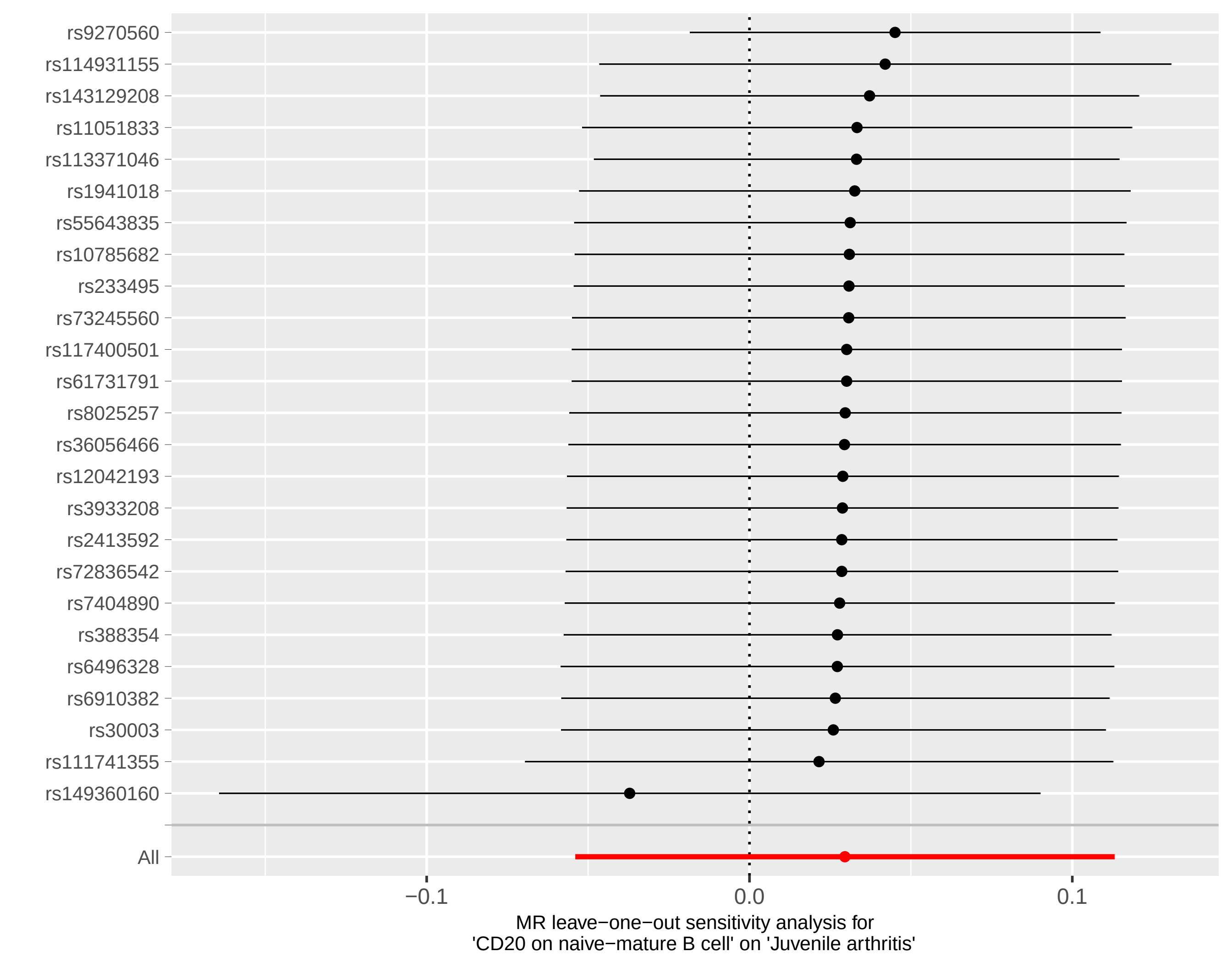
rs9270560
rs114931155
rs143129208
rs11051833
rs113371046
rs1941018
rs55643835
rs10785682
rs233495
rs73245560
rs117400501
rs61731791
rs8025257
rs36056466
rs12042193
rs3933208
rs2413592
rs72836542
rs7404890
rs388354
rs6496328
rs6910382
rs30003
rs111741355
rs149360160
All
−0.1
0.0
0.1
MR leave−one−out sensitivity analysis for
'CD20 on naive−mature B cell' on 'Juvenile arthritis'

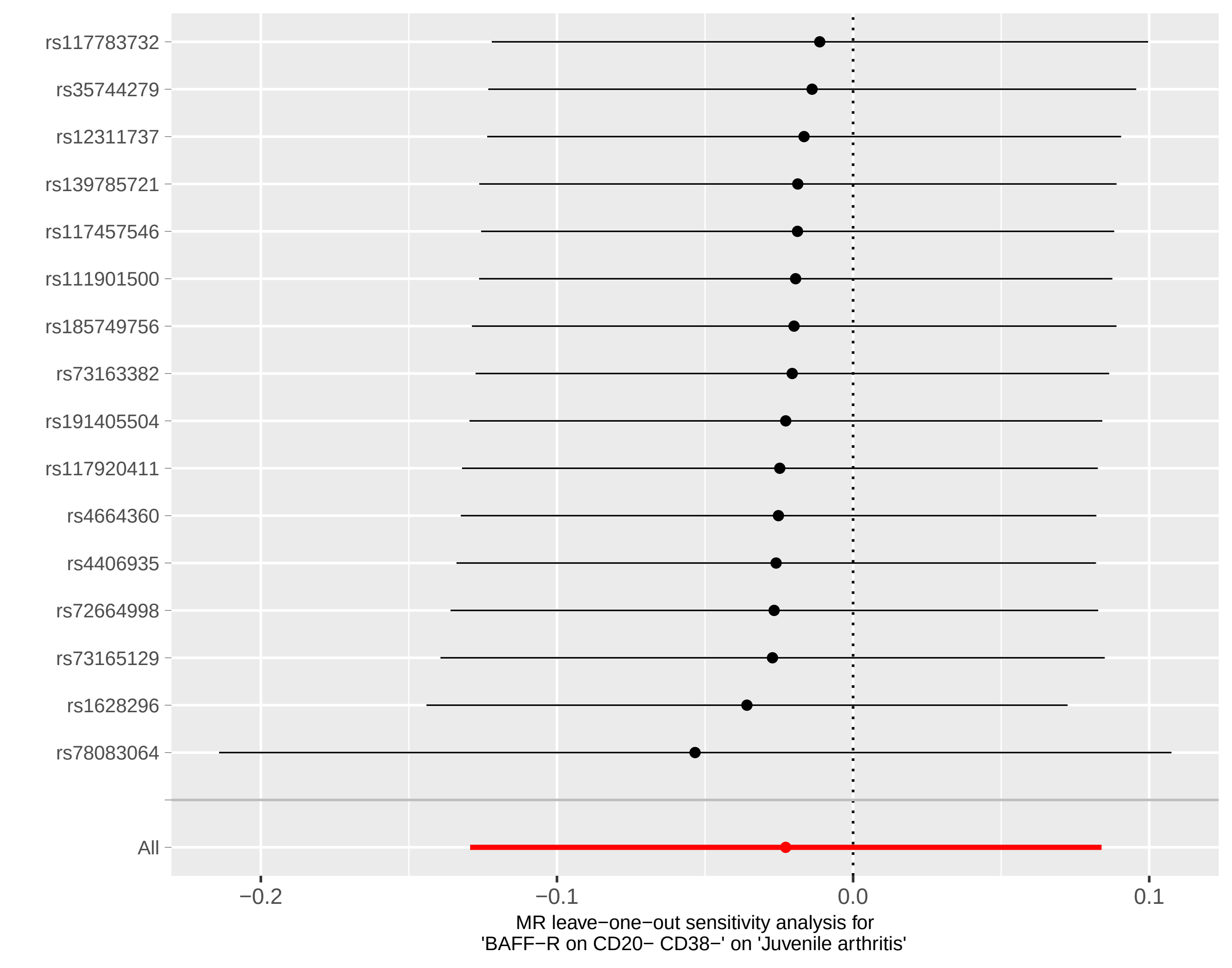
rs117783732
rs35744279
rs12311737
rs139785721
rs117457546
rs111901500
rs185749756
rs73163382
rs191405504
rs117920411
rs4664360
rs4406935
rs72664998
rs73165129
rs1628296
rs78083064
All
−0.2
−0.1
0.0
0.1
MR leave−one−out sensitivity analysis for
'BAFF−R on CD20− CD38−' on 'Juvenile arthritis'

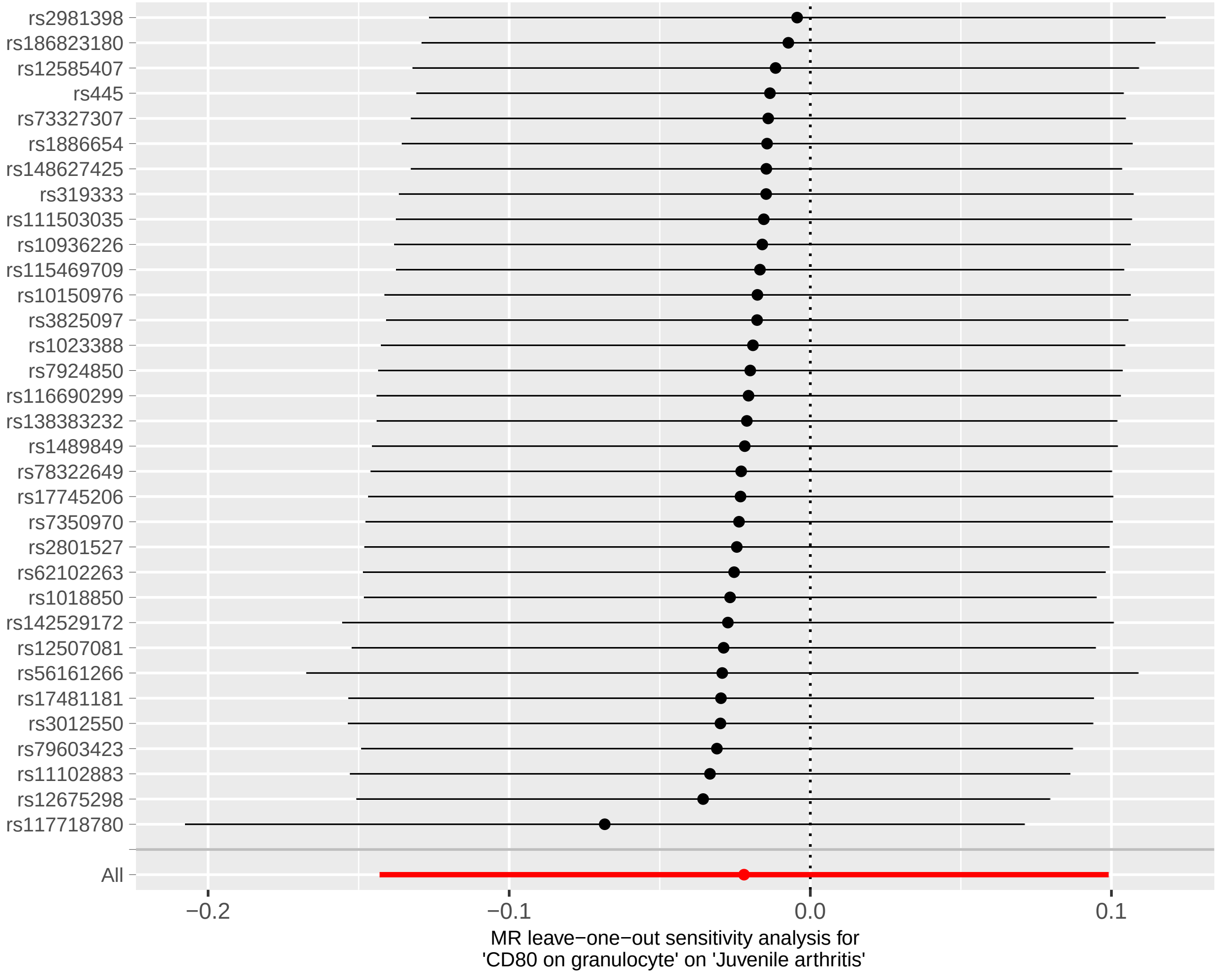
rs2981398
rs186823180
rs12585407
rs445
rs73327307
rs1886654
rs148627425
rs319333
rs111503035
rs10936226
rs115469709
rs10150976
rs3825097
rs1023388
rs7924850
rs116690299
rs138383232
rs1489849
rs78322649
rs17745206
rs7350970
rs2801527
rs62102263
rs1018850
rs142529172
rs12507081
rs56161266
rs17481181
rs3012550
rs79603423
rs11102883
rs12675298
rs117718780
All
−0.2
−0.1
0.0
0.1
MR leave−one−out sensitivity analysis for
'CD80 on granulocyte' on 'Juvenile arthritis'

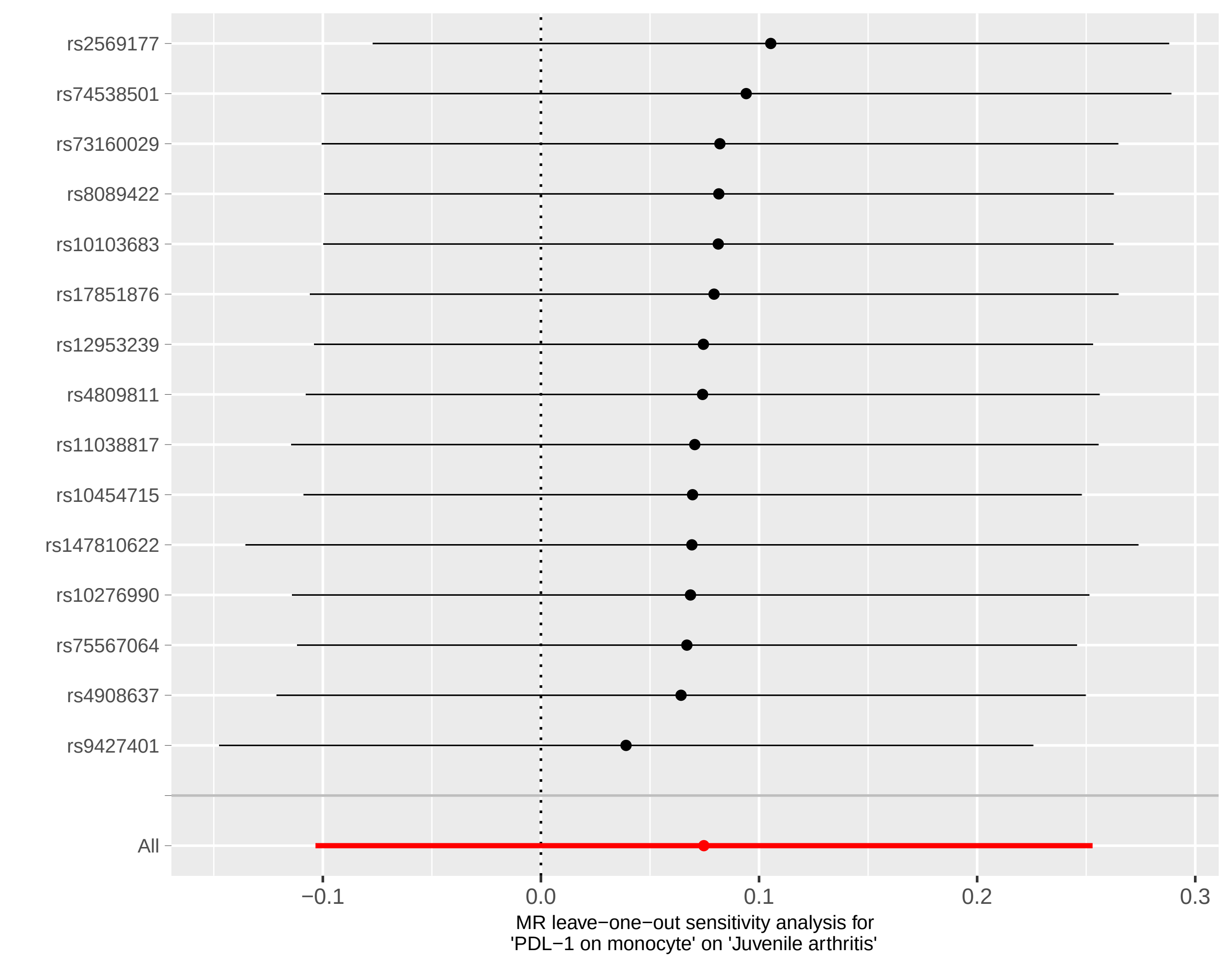
rs2569177
rs74538501
rs73160029
rs8089422
rs10103683
rs17851876
rs12953239
rs4809811
rs11038817
rs10454715
rs147810622
rs10276990
rs75567064
rs4908637
rs9427401
All
−0.1
0.0
0.1
0.2
0.3
MR leave−one−out sensitivity analysis for
'PDL−1 on monocyte' on 'Juvenile arthritis'

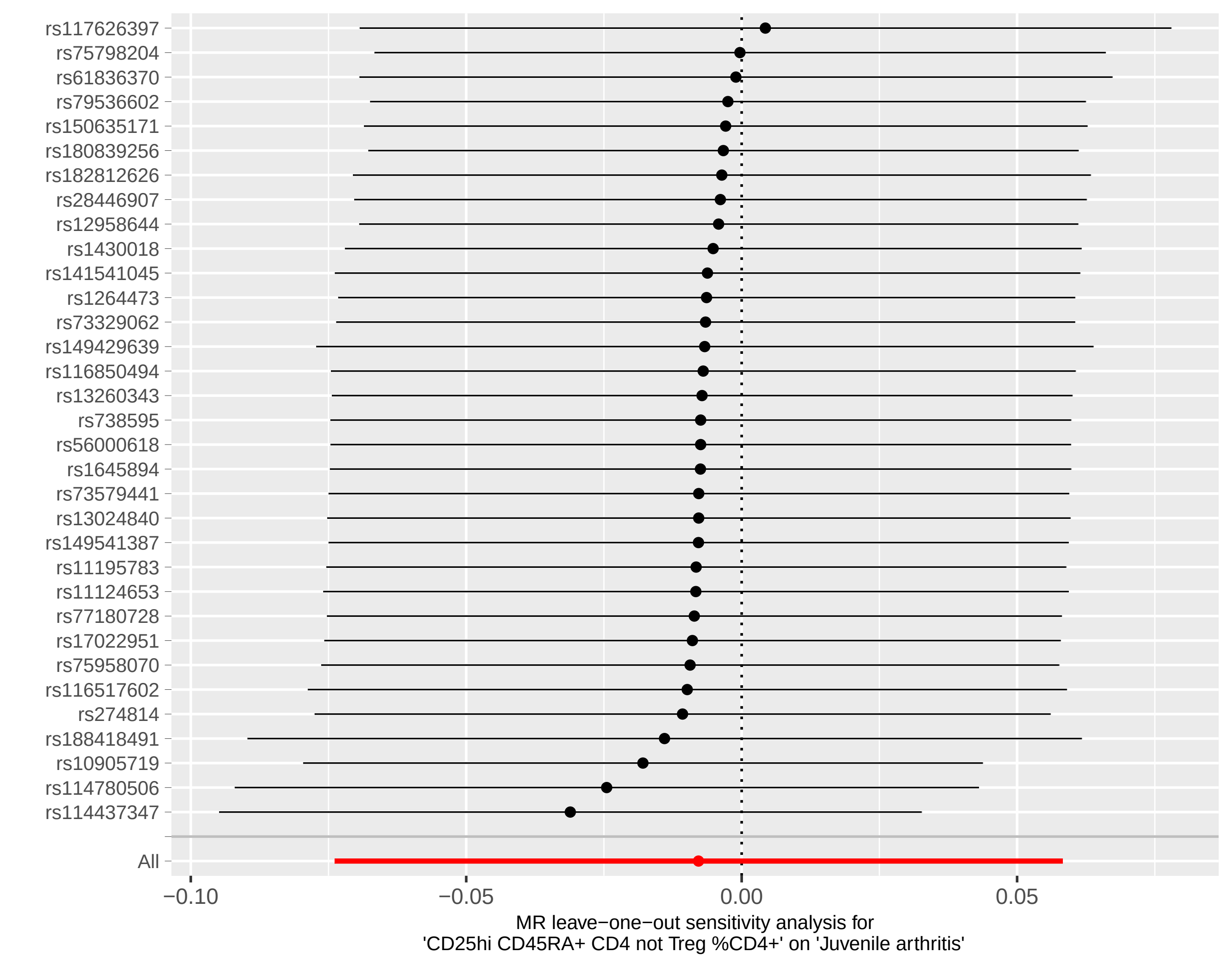
rs117626397
rs75798204
rs61836370
rs79536602
rs150635171
rs180839256
rs182812626
rs28446907
rs12958644
rs1430018
rs141541045
rs1264473
rs73329062
rs149429639
rs116850494
rs13260343
rs738595
rs56000618
rs1645894
rs73579441
rs13024840
rs149541387
rs11195783
rs11124653
rs77180728
rs17022951
rs75958070
rs116517602
rs274814
rs188418491
rs10905719
rs114780506
rs114437347
All
−0.10
−0.05
0.00
0.05
MR leave−one−out sensitivity analysis for
'CD25hi CD45RA+ CD4 not Treg %CD4+' on 'Juvenile arthritis'

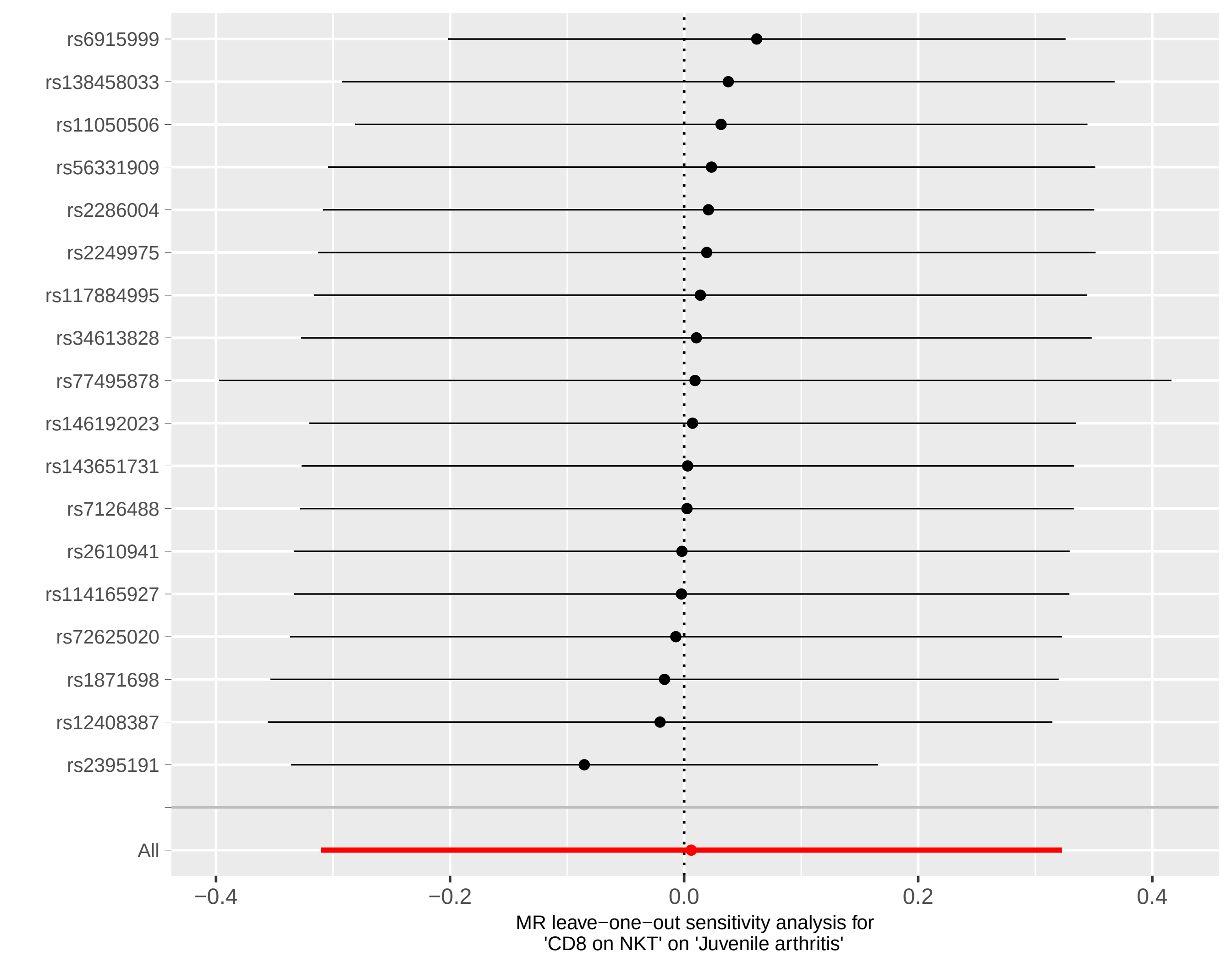

rs6915999
rs138458033
rs11050506
rs56331909
rs2286004
rs2249975
rs117884995
rs34613828
rs77495878
rs146192023
rs143651731
rs7126488
rs2610941
rs114165927
rs72625020
rs1871698
rs12408387
rs2395191
All
−0.4
−0.2
0.0
0.2
0.4
MR leave−one−out sensitivity analysis for
'CD8 on NKT' on 'Juvenile arthritis'

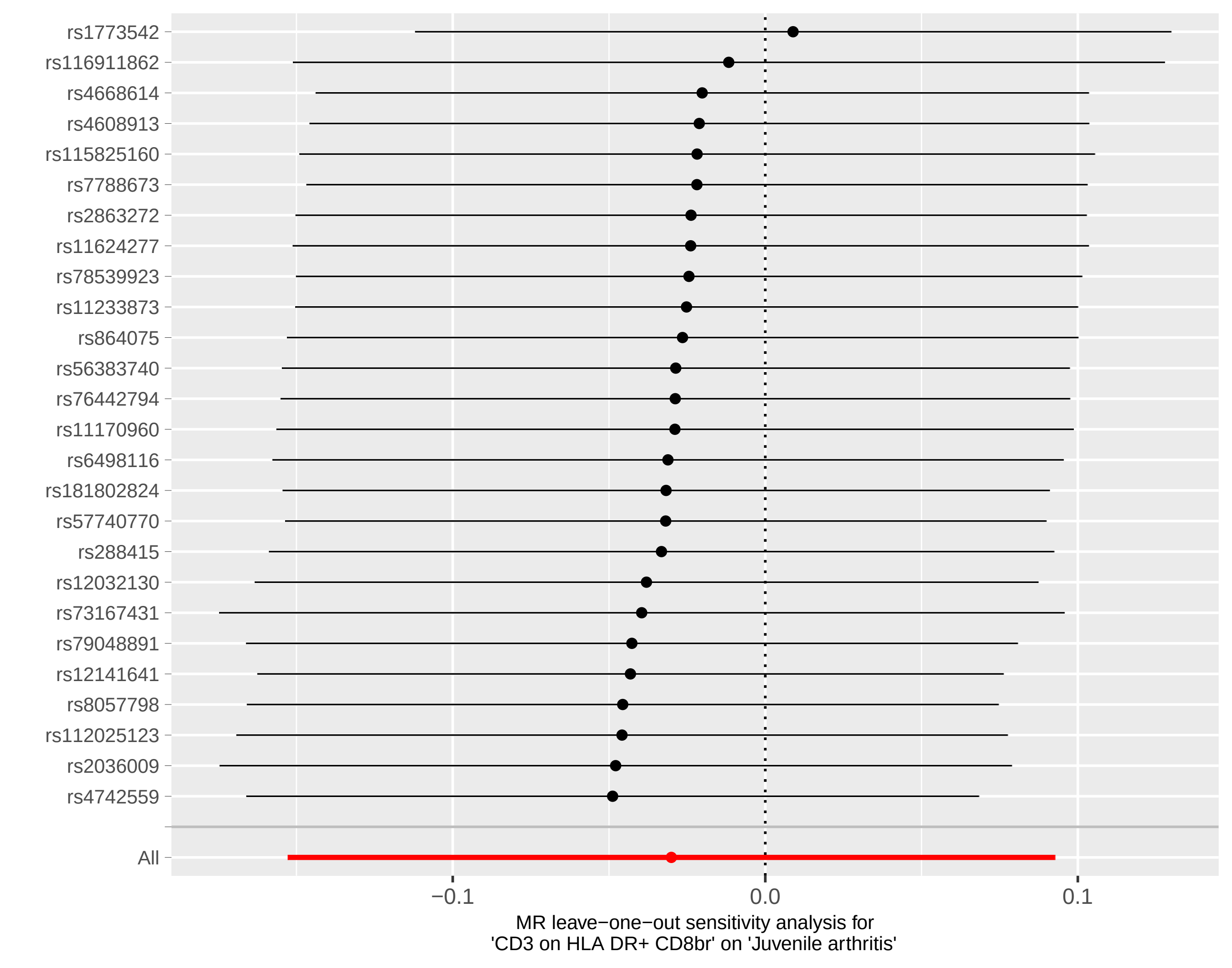
rs1773542
rs116911862
rs4668614
rs4608913
rs115825160
rs7788673
rs2863272
rs11624277
rs78539923
rs11233873
rs864075
rs56383740
rs76442794
rs11170960
rs6498116
rs181802824
rs57740770
rs288415
rs12032130
rs73167431
rs79048891
rs12141641
rs8057798
rs112025123
rs2036009
rs4742559
All
−0.1
0.0
0.1
MR leave−one−out sensitivity analysis for
'CD3 on HLA DR+ CD8br' on 'Juvenile arthritis'

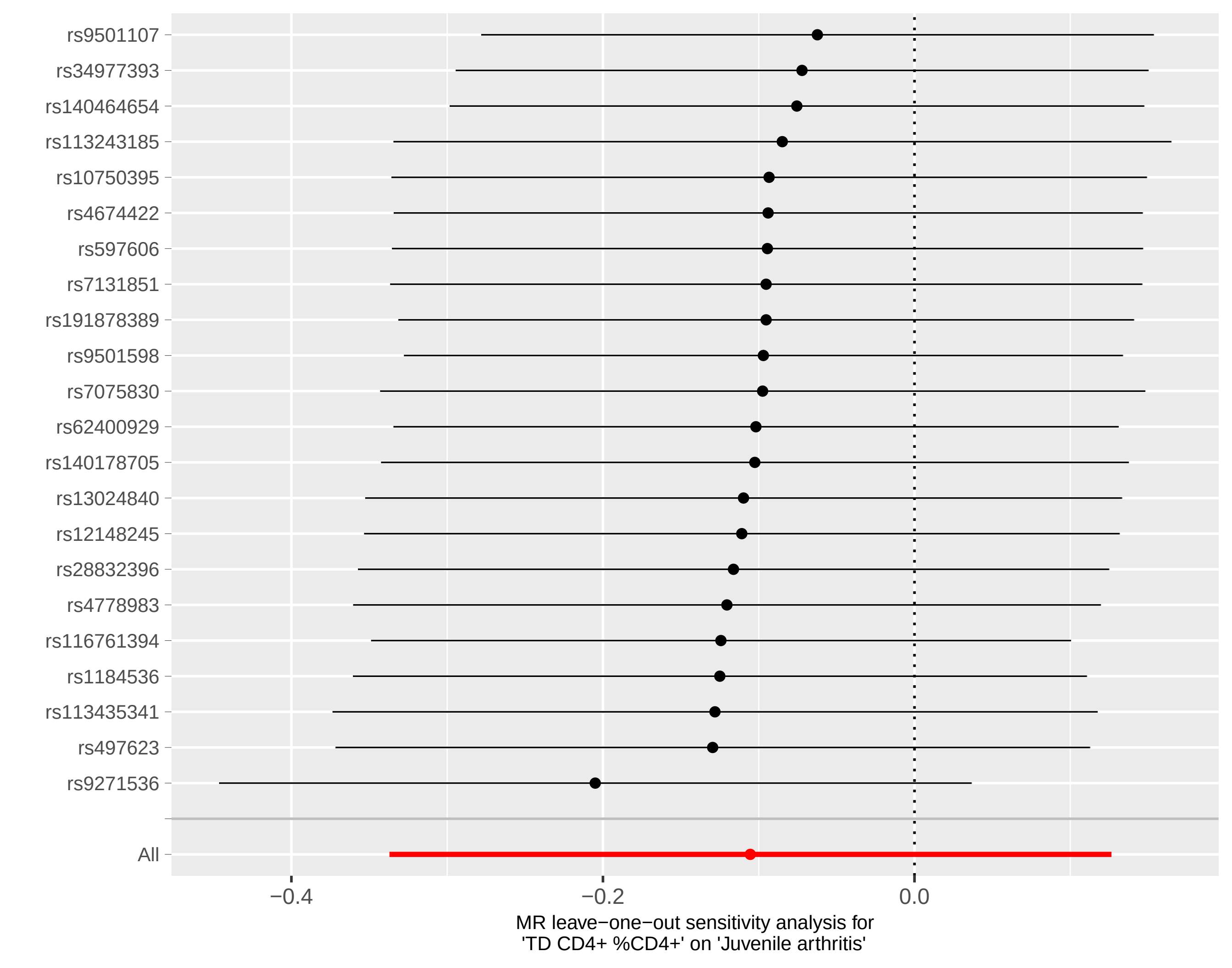
rs9501107
rs34977393
rs140464654
rs113243185
rs10750395
rs4674422
rs597606
rs7131851
rs191878389
rs9501598
rs7075830
rs62400929
rs140178705
rs13024840
rs12148245
rs28832396
rs4778983
rs116761394
rs1184536
rs113435341
rs497623
rs9271536
All
−0.4
−0.2
0.0
MR leave−one−out sensitivity analysis for
'TD CD4+ %CD4+' on 'Juvenile arthritis'

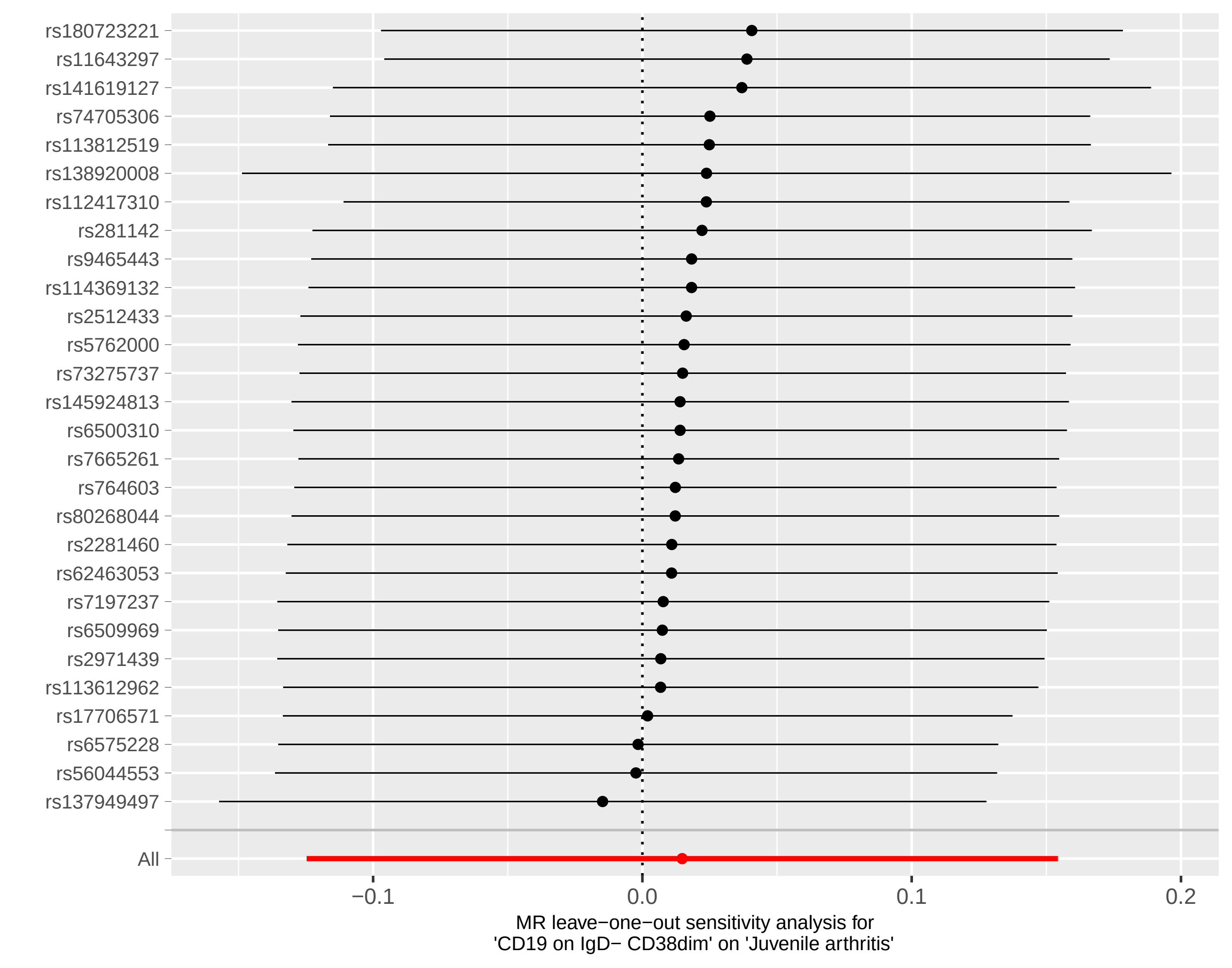
rs180723221
rs11643297
rs141619127
rs74705306
rs113812519
rs138920008
rs112417310
rs281142
rs9465443
rs114369132
rs2512433
rs5762000
rs73275737
rs145924813
rs6500310
rs7665261
rs764603
rs80268044
rs2281460
rs62463053
rs7197237
rs6509969
rs2971439
rs113612962
rs17706571
rs6575228
rs56044553
rs137949497
All
−0.1
0.0
0.1
0.2
MR leave−one−out sensitivity analysis for
'CD19 on IgD− CD38dim' on 'Juvenile arthritis'

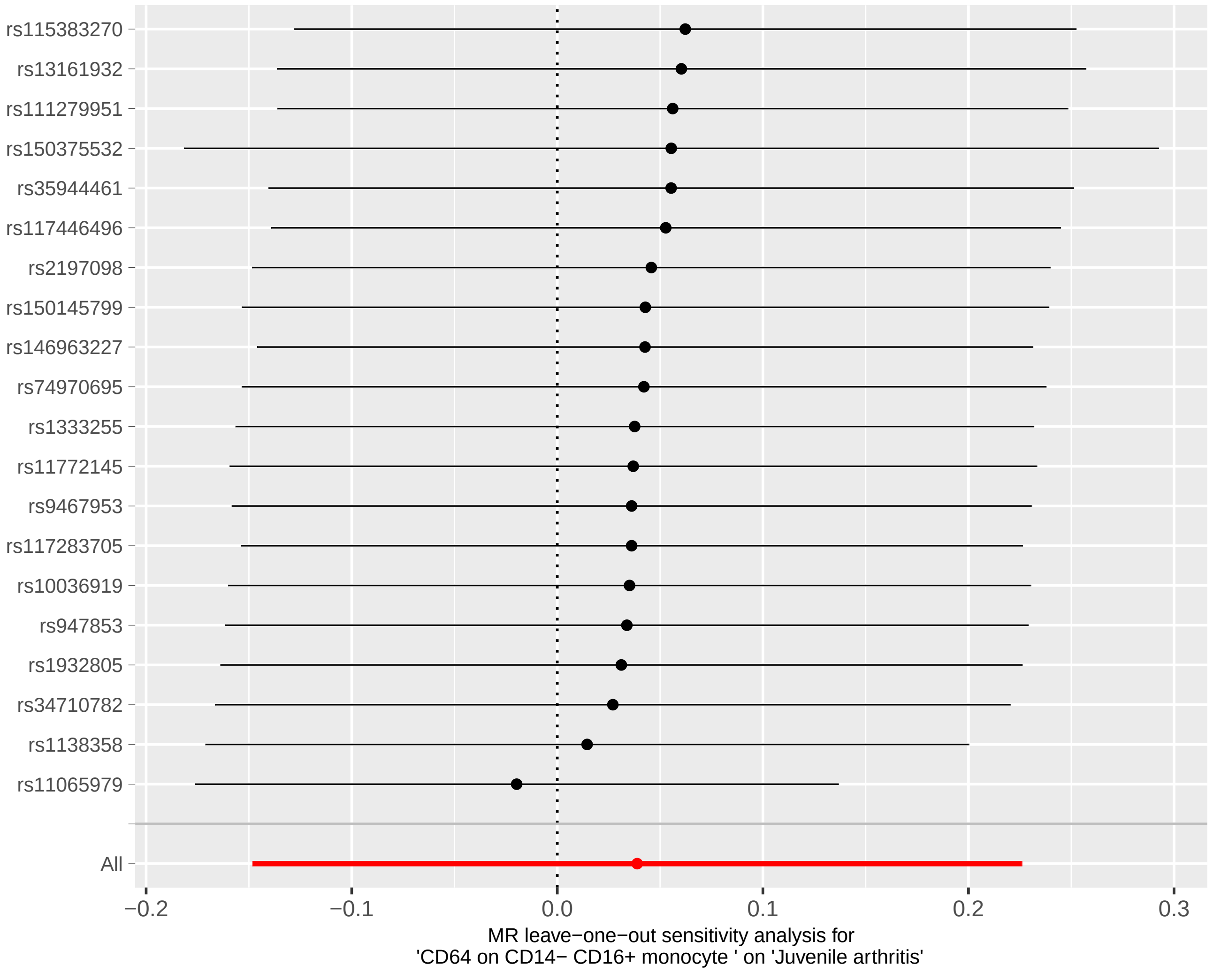

rs115383270
rs13161932
rs111279951
rs150375532
rs35944461
rs117446496
rs2197098
rs150145799
rs146963227
rs74970695
rs1333255
rs11772145
rs9467953
rs117283705
rs10036919
rs947853
rs1932805
rs34710782
rs1138358
rs11065979
All
−0.2
−0.1
0.0
0.1
0.2
0.3
MR leave−one−out sensitivity analysis for
'CD64 on CD14− CD16+ monocyte ' on 'Juvenile arthritis'

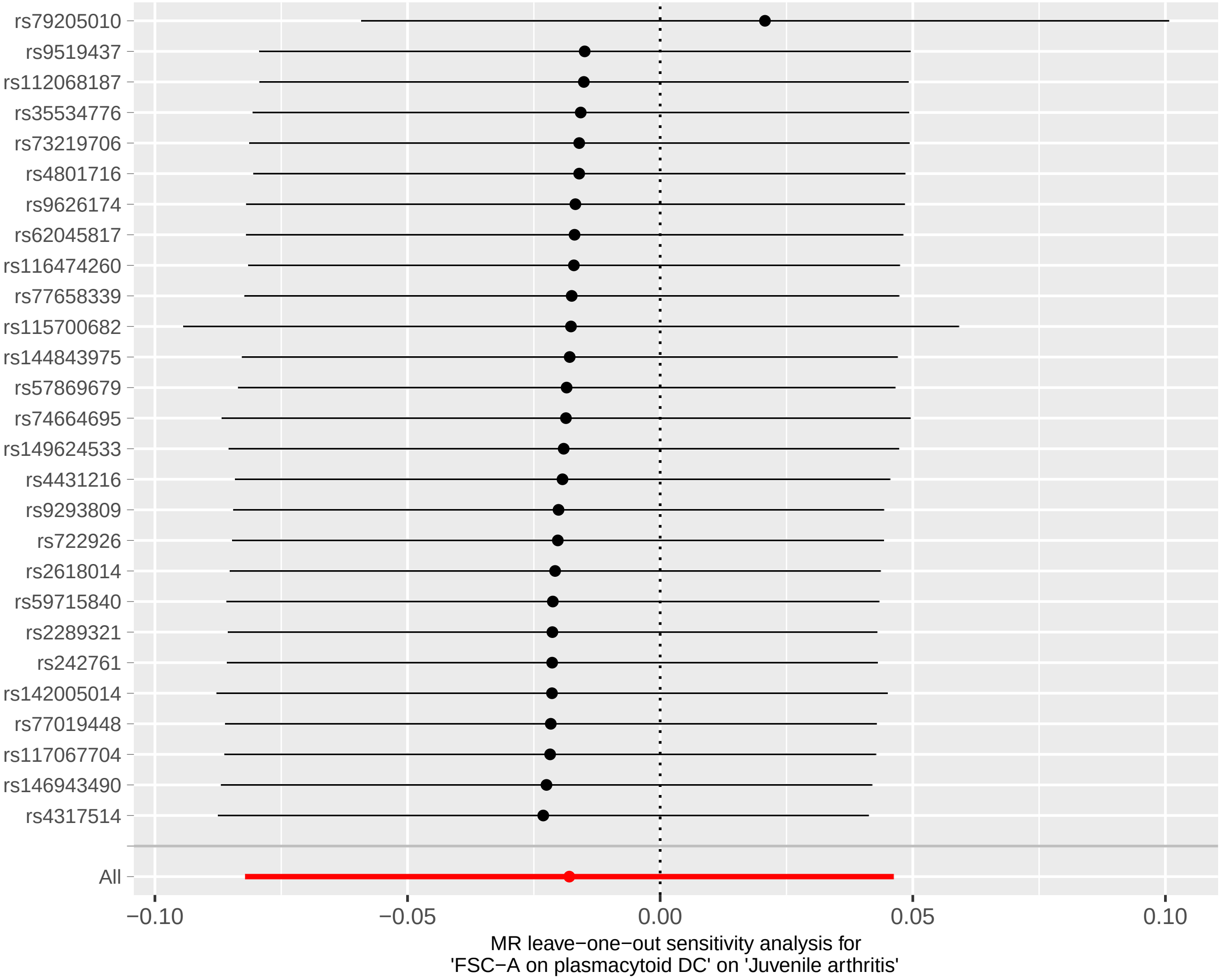
rs79205010
rs9519437
rs112068187
rs35534776
rs73219706
rs4801716
rs9626174
rs62045817
rs116474260
rs77658339
rs115700682
rs144843975
rs57869679
rs74664695
rs149624533
rs4431216
rs9293809
rs722926
rs2618014
rs59715840
rs2289321
rs242761
rs142005014
rs77019448
rs117067704
rs146943490
rs4317514
All
−0.10
−0.05
0.00
0.05
0.10
MR leave−one−out sensitivity analysis for
'FSC−A on plasmacytoid DC' on 'Juvenile arthritis'

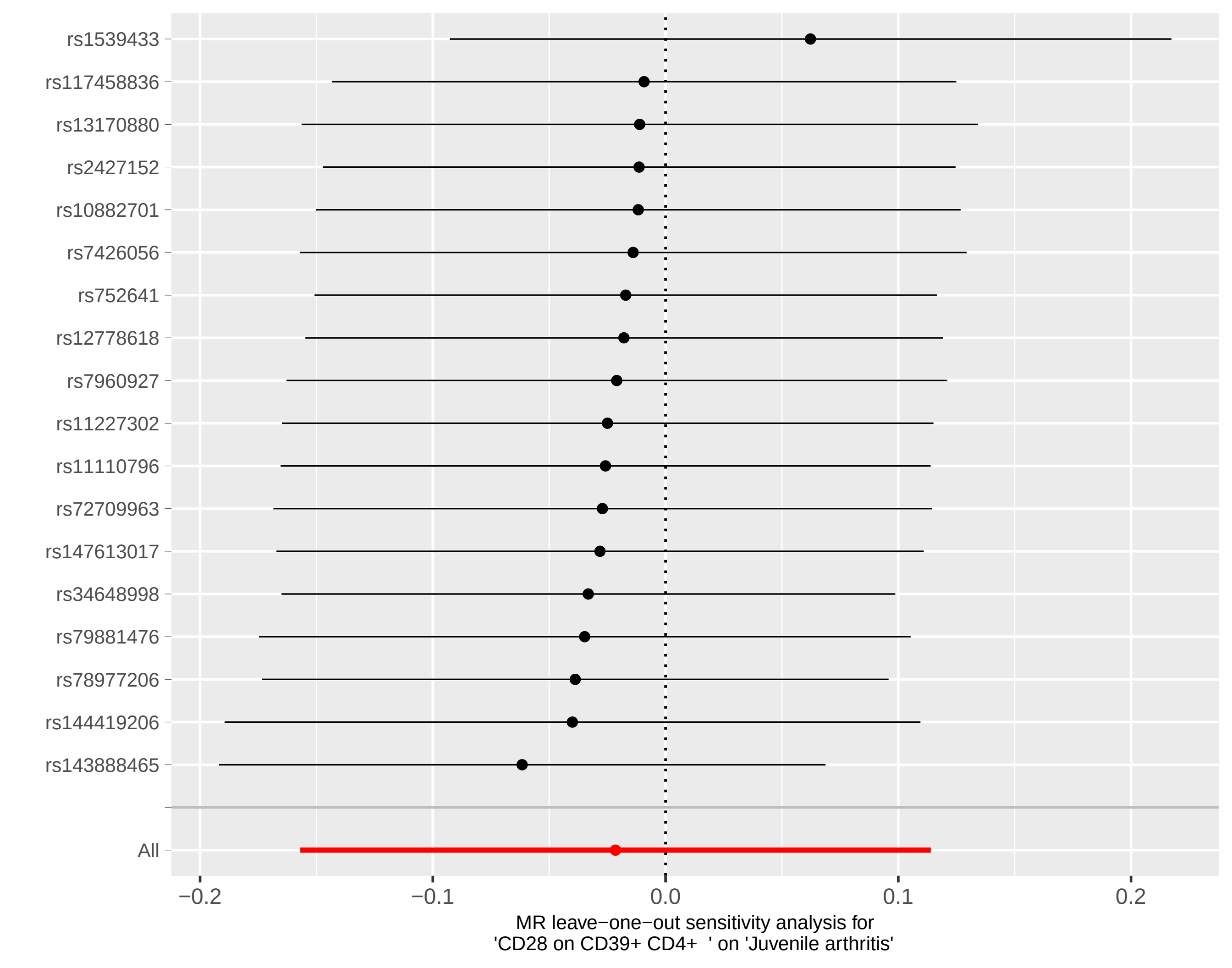

rs1539433
rs117458836
rs13170880
rs2427152
rs10882701
rs7426056
rs752641
rs12778618
rs7960927
rs11227302
rs11110796
rs72709963
rs147613017
rs34648998
rs79881476
rs78977206
rs144419206
rs143888465
All
−0.2
−0.1
0.0
0.1
0.2
MR leave−one−out sensitivity analysis for
'CD28 on CD39+ CD4+ ' on 'Juvenile arthritis'

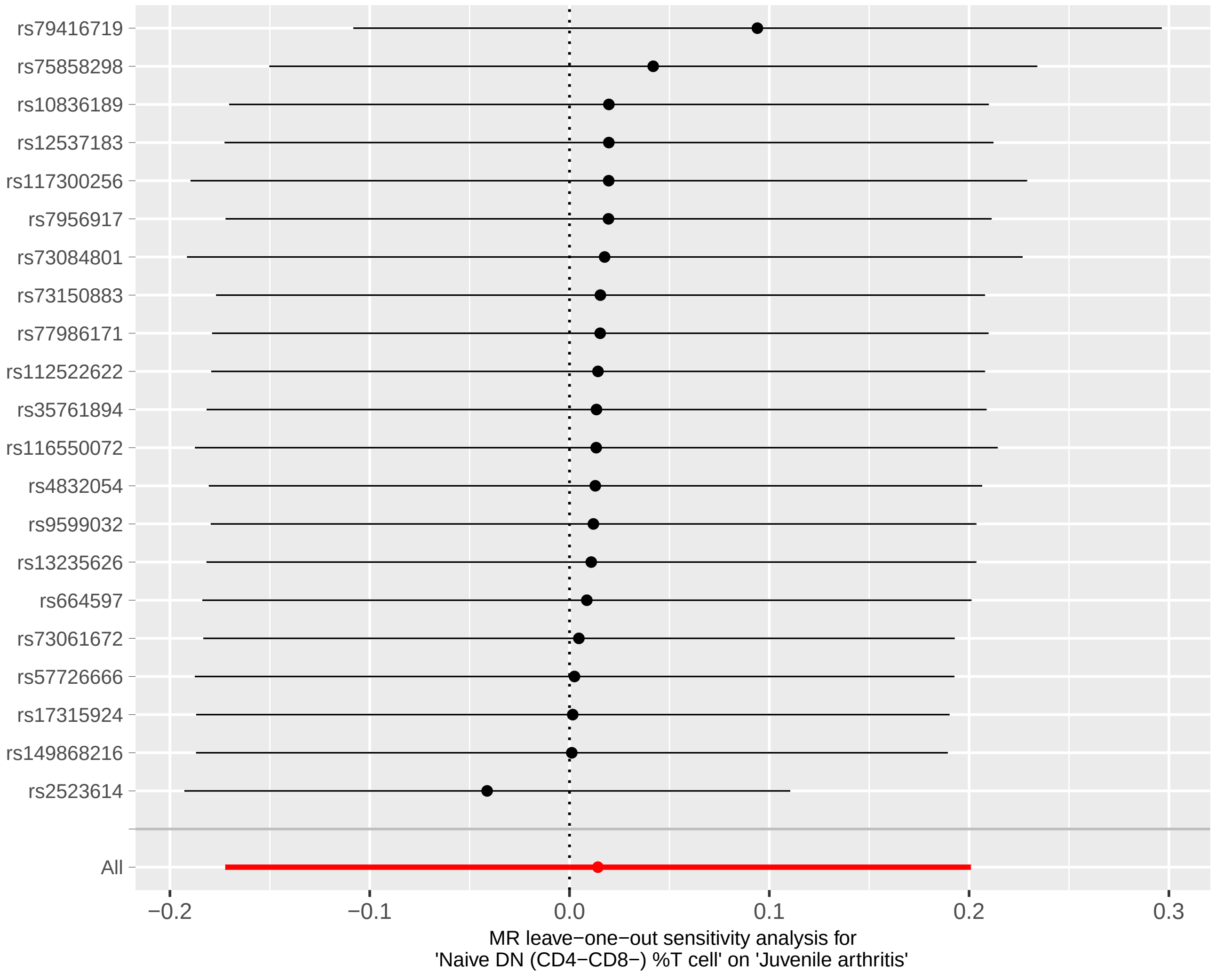
rs79416719
rs75858298
rs10836189
rs12537183
rs117300256
rs7956917
rs73084801
rs73150883
rs77986171
rs112522622
rs35761894
rs116550072
rs4832054
rs9599032
rs13235626
rs664597
rs73061672
rs57726666
rs17315924
rs149868216
rs2523614
All
−0.2
−0.1
0.0
0.1
0.2
0.3
MR leave−one−out sensitivity analysis for
'Naive DN (CD4−CD8−) %T cell' on 'Juvenile arthritis'

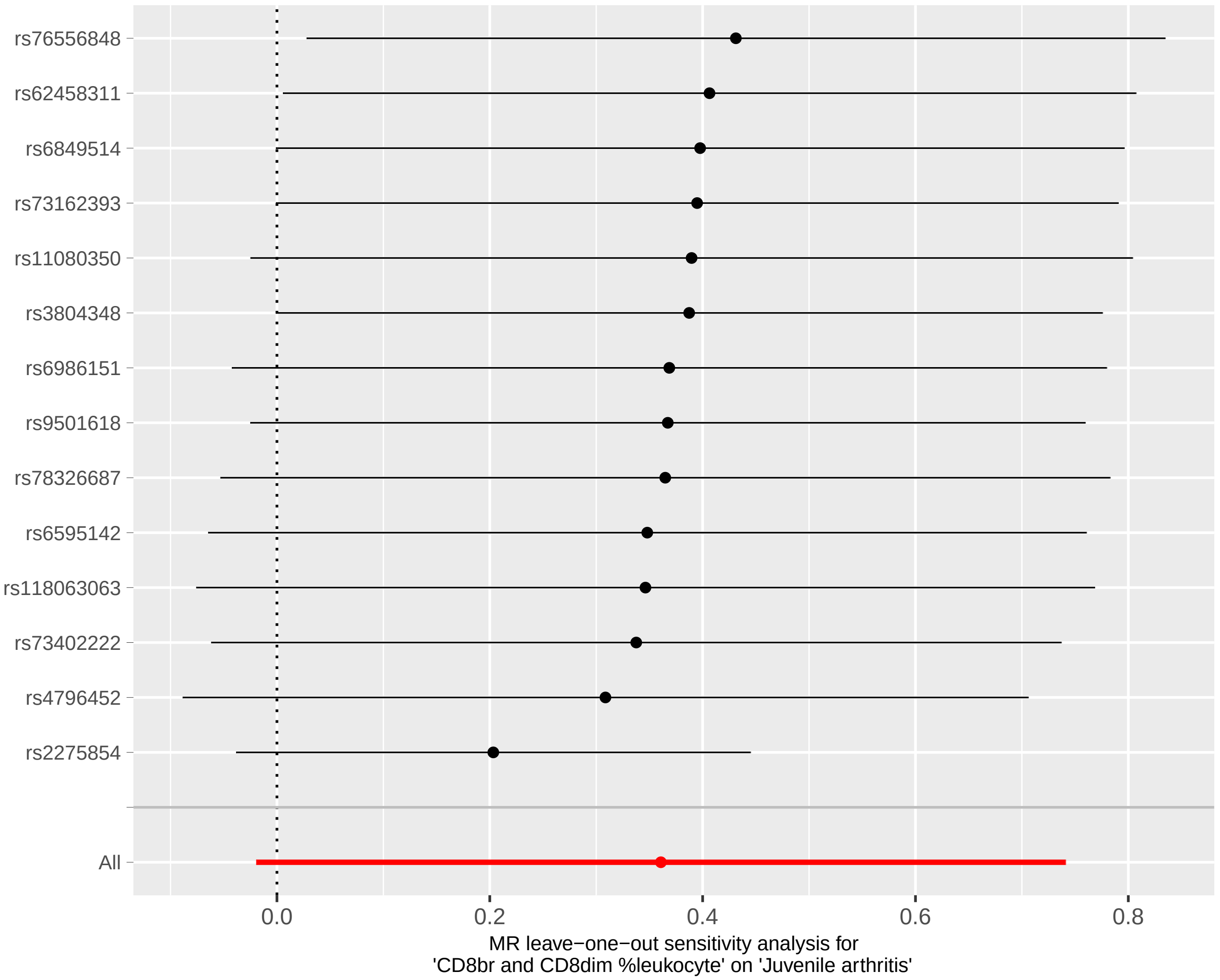
rs76556848
rs62458311
rs6849514
rs73162393
rs11080350
rs3804348
rs6986151
rs9501618
rs78326687
rs6595142
rs118063063
rs73402222
rs4796452
rs2275854
All
0.0
0.2
0.4
0.6
0.8
MR leave−one−out sensitivity analysis for
'CD8br and CD8dim %leukocyte' on 'Juvenile arthritis'

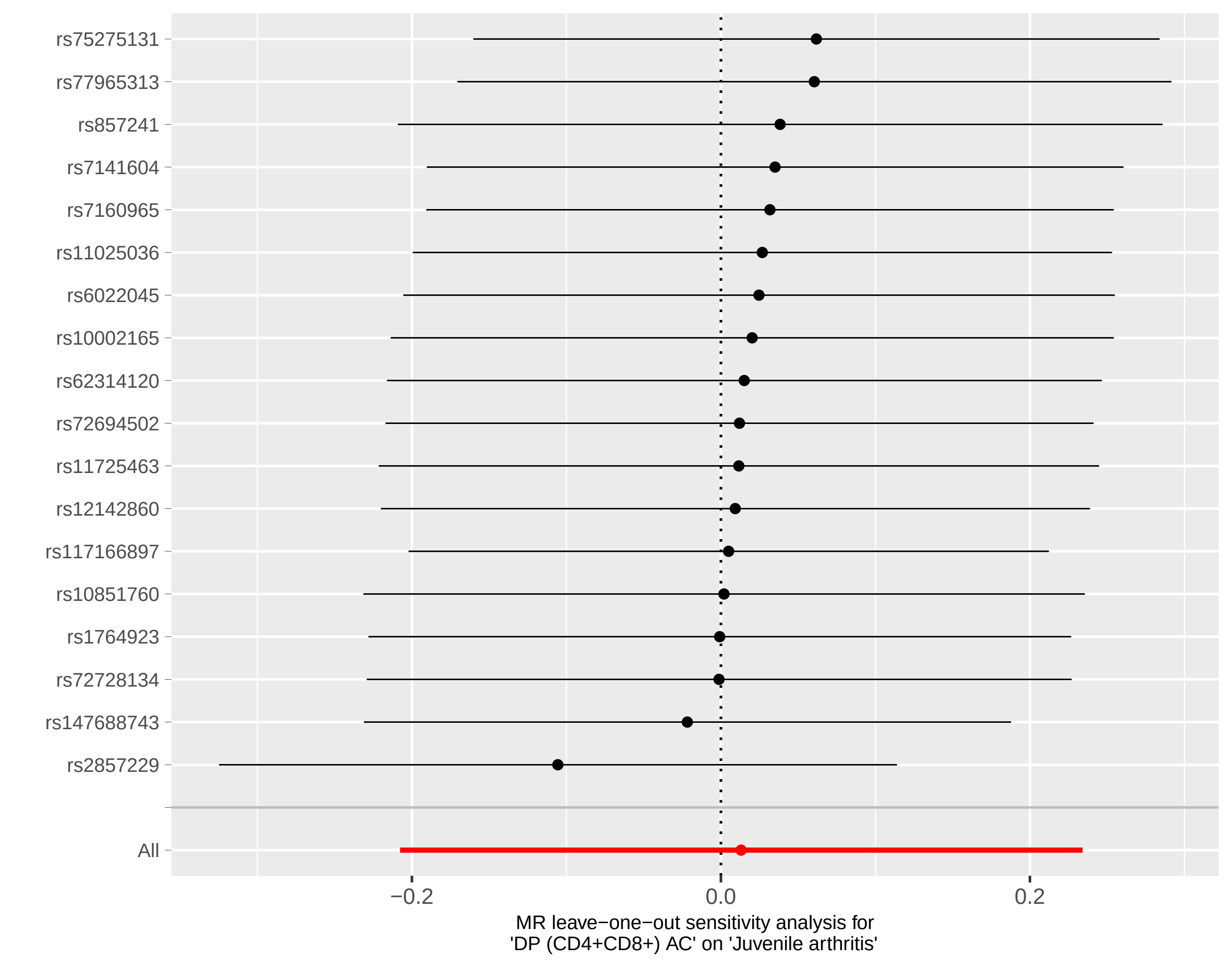

rs75275131
rs77965313
rs857241
rs7141604
rs7160965
rs11025036
rs6022045
rs10002165
rs62314120
rs72694502
rs11725463
rs12142860
rs117166897
rs10851760
rs1764923
rs72728134
rs147688743
rs2857229
All
−0.2
0.0
0.2
MR leave−one−out sensitivity analysis for
'DP (CD4+CD8+) AC' on 'Juvenile arthritis'

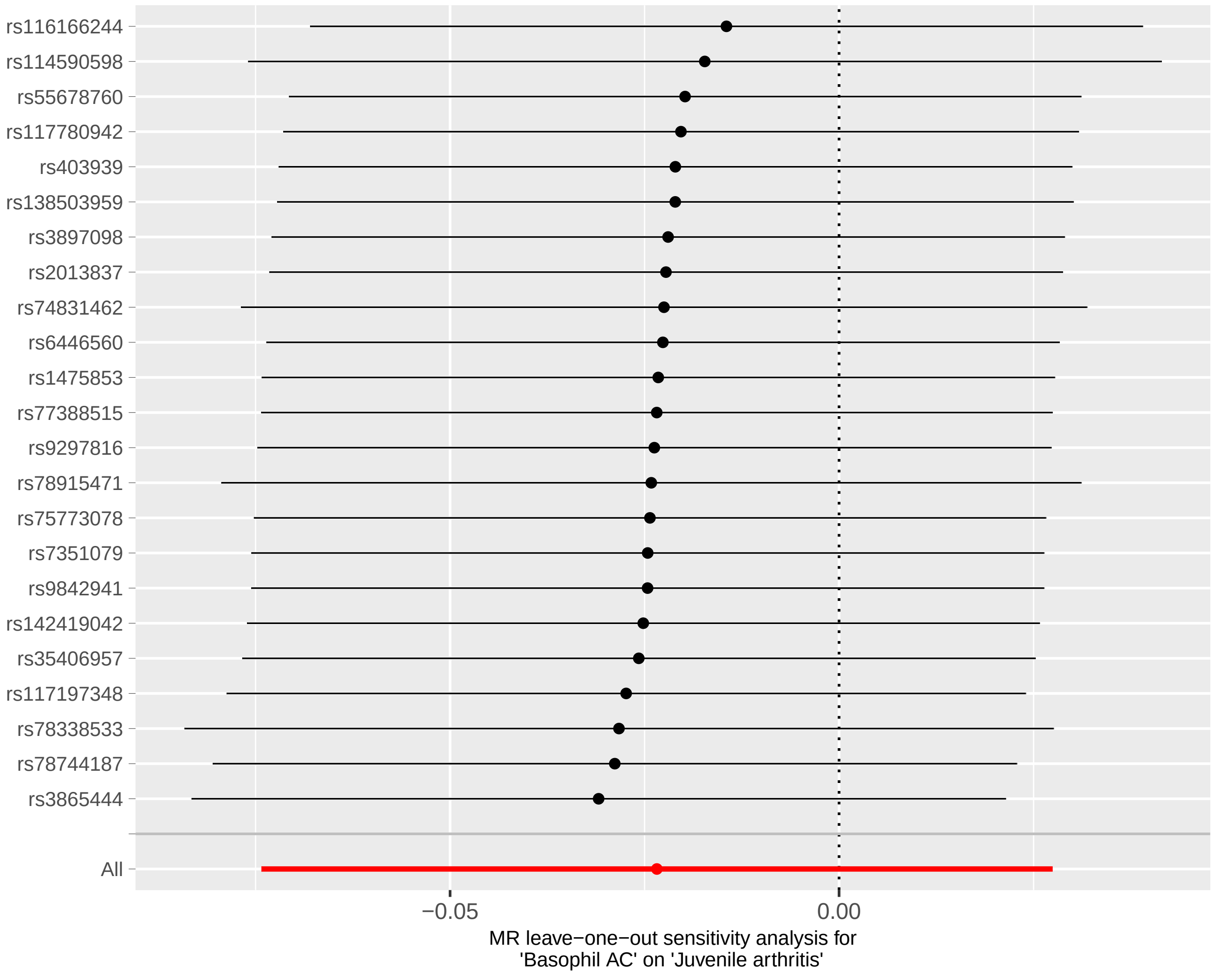
rs116166244
rs114590598
rs55678760
rs117780942
rs403939
rs138503959
rs3897098
rs2013837
rs74831462
rs6446560
rs1475853
rs77388515
rs9297816
rs78915471
rs75773078
rs7351079
rs9842941
rs142419042
rs35406957
rs117197348
rs78338533
rs78744187
rs3865444
All
−0.05
0.00
MR leave−one−out sensitivity analysis for
'Basophil AC' on 'Juvenile arthritis'

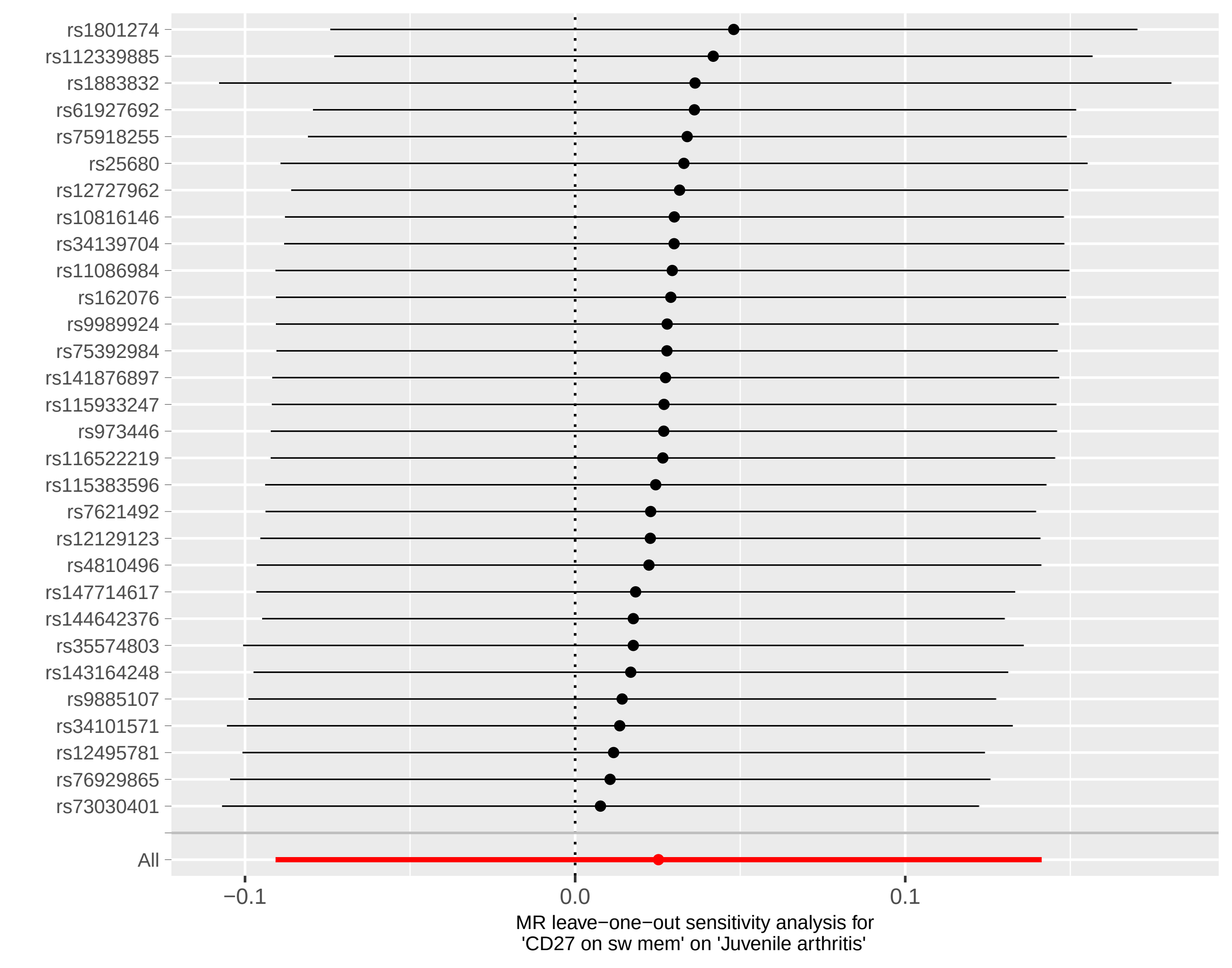

rs1801274
rs112339885
rs1883832
rs61927692
rs75918255
rs25680
rs12727962
rs10816146
rs34139704
rs11086984
rs162076
rs9989924
rs75392984
rs141876897
rs115933247
rs973446
rs116522219
rs115383596
rs7621492
rs12129123
rs4810496
rs147714617
rs144642376
rs35574803
rs143164248
rs9885107
rs34101571
rs12495781
rs76929865
rs73030401
All
−0.1
0.0
0.1
MR leave−one−out sensitivity analysis for
'CD27 on sw mem' on 'Juvenile arthritis'

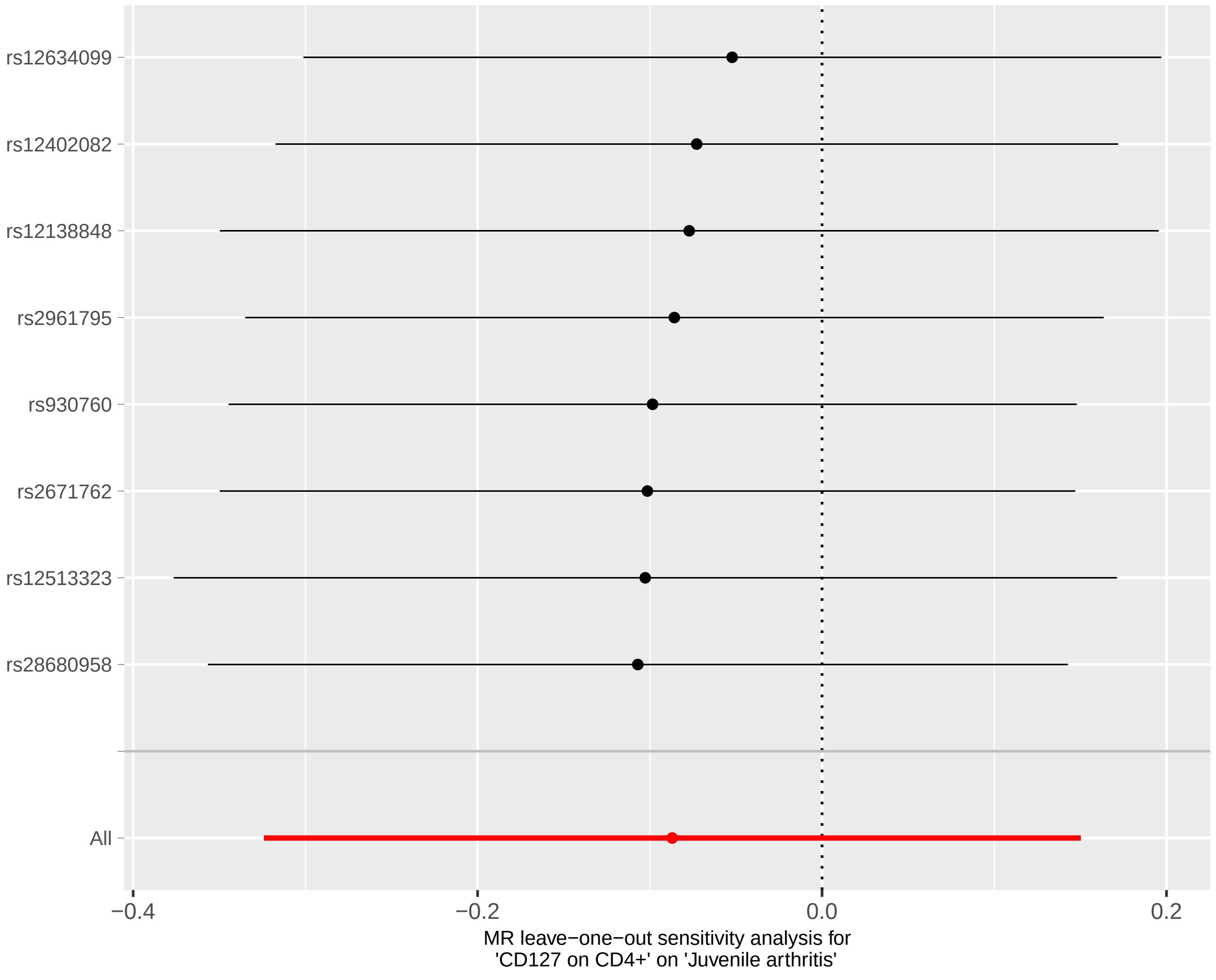
rs12634099
rs12402082
rs12138848
rs2961795
rs930760
rs2671762
rs12513323
rs28680958
All
−0.4
−0.2
0.0
0.2
MR leave−one−out sensitivity analysis for
'CD127 on CD4+' on 'Juvenile arthritis'

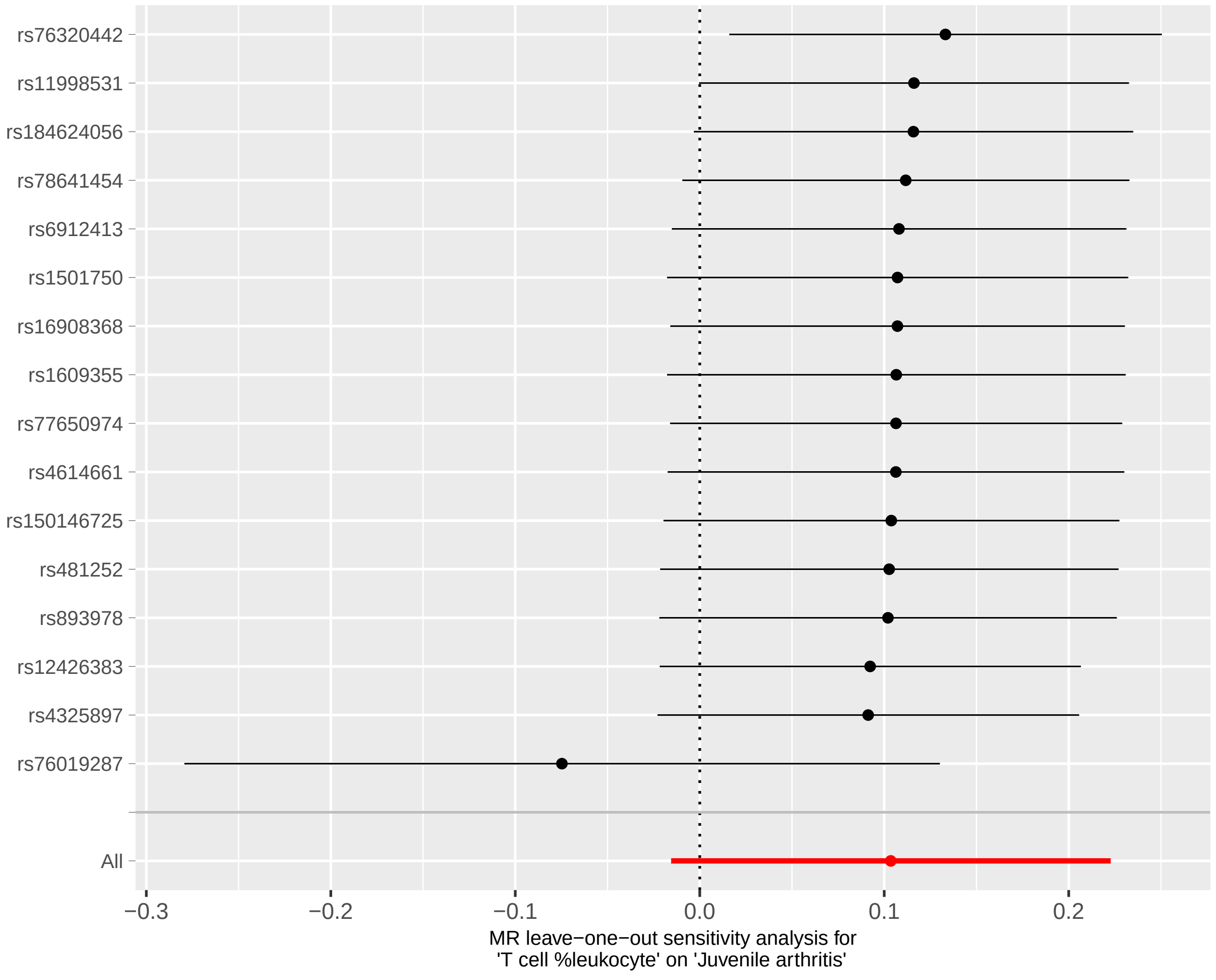

rs76320442
rs11998531
rs184624056
rs78641454
rs6912413
rs1501750
rs16908368
rs1609355
rs77650974
rs4614661
rs150146725
rs481252
rs893978
rs12426383
rs4325897
rs76019287
All
−0.3
−0.2
−0.1
0.0
0.1
0.2
MR leave−one−out sensitivity analysis for
'T cell %leukocyte' on 'Juvenile arthritis'

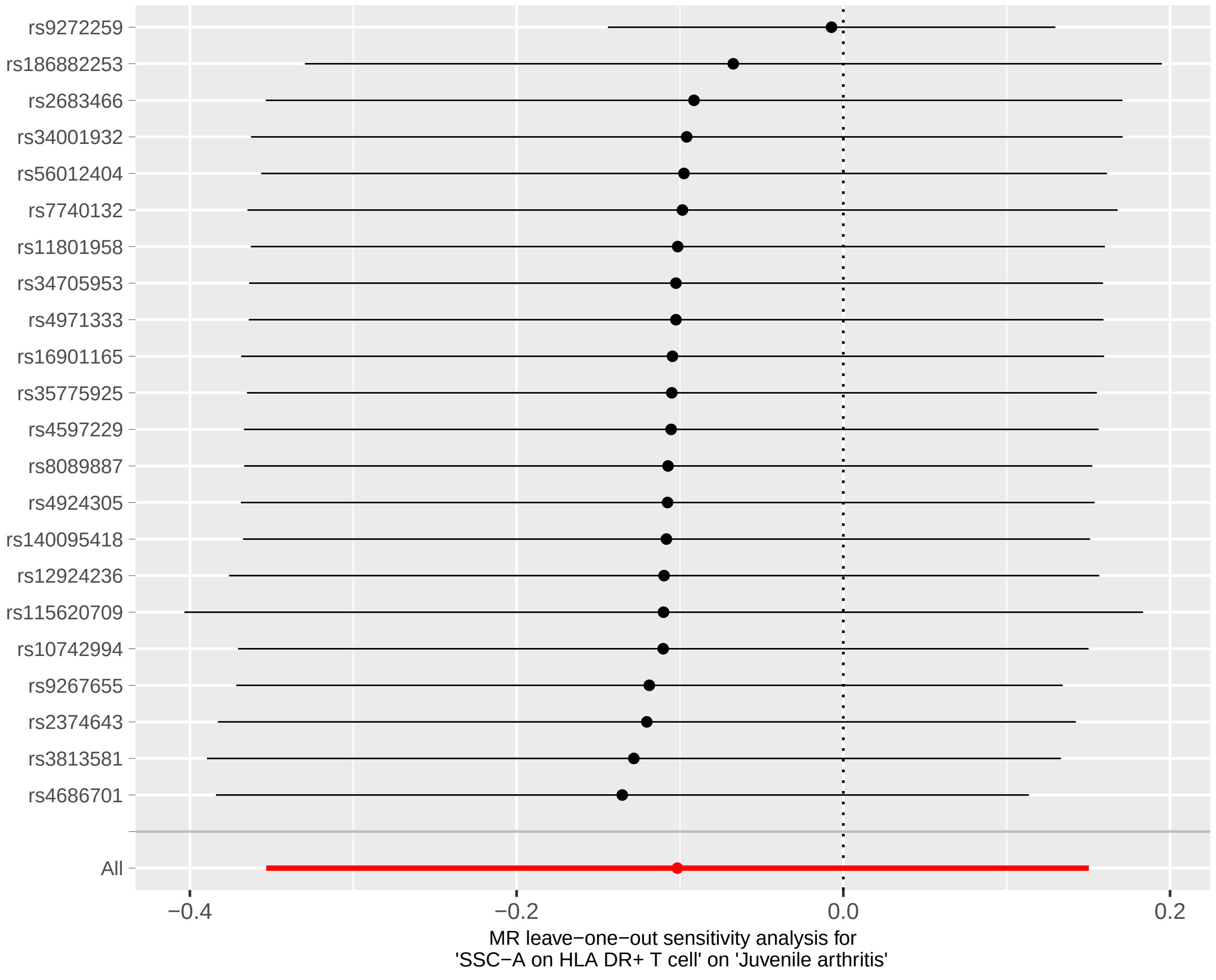

rs9272259
rs186882253
rs2683466
rs34001932
rs56012404
rs7740132
rs11801958
rs34705953
rs4971333
rs16901165
rs35775925
rs4597229
rs8089887
rs4924305
rs140095418
rs12924236
rs115620709
rs10742994
rs9267655
rs2374643
rs3813581
rs4686701
All
−0.4
−0.2
0.0
0.2
MR leave−one−out sensitivity analysis for
'SSC−A on HLA DR+ T cell' on 'Juvenile arthritis'

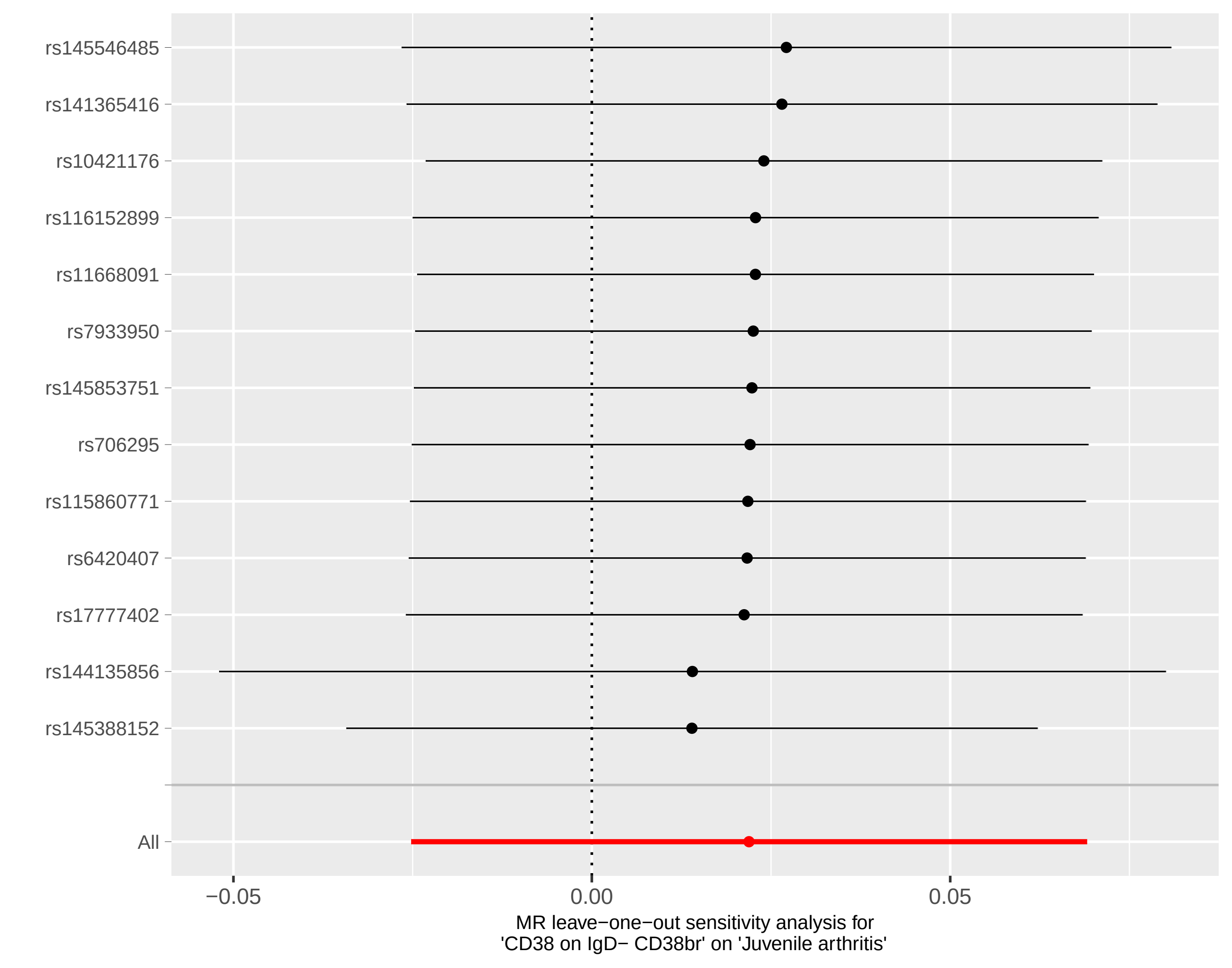
rs145546485
rs141365416
rs10421176
rs116152899
rs11668091
rs7933950
rs145853751
rs706295
rs115860771
rs6420407
rs17777402
rs144135856
rs145388152
All
−0.05
0.00
0.05
MR leave−one−out sensitivity analysis for
'CD38 on IgD− CD38br' on 'Juvenile arthritis'

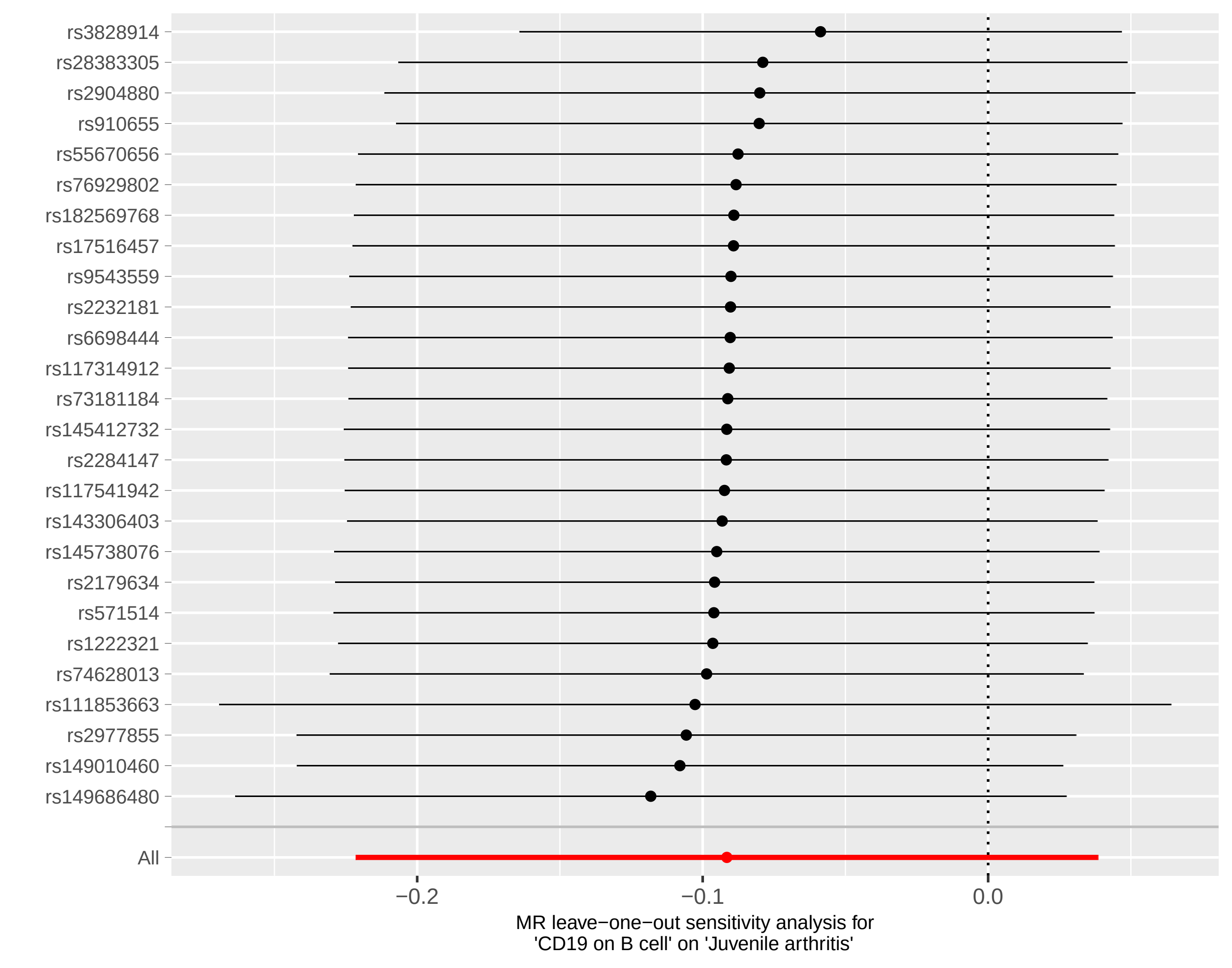
rs3828914
rs28383305
rs2904880
rs910655
rs55670656
rs76929802
rs182569768
rs17516457
rs9543559
rs2232181
rs6698444
rs117314912
rs73181184
rs145412732
rs2284147
rs117541942
rs143306403
rs145738076
rs2179634
rs571514
rs1222321
rs74628013
rs111853663
rs2977855
rs149010460
rs149686480
All
−0.2
−0.1
0.0
MR leave−one−out sensitivity analysis for
'CD19 on B cell' on 'Juvenile arthritis'

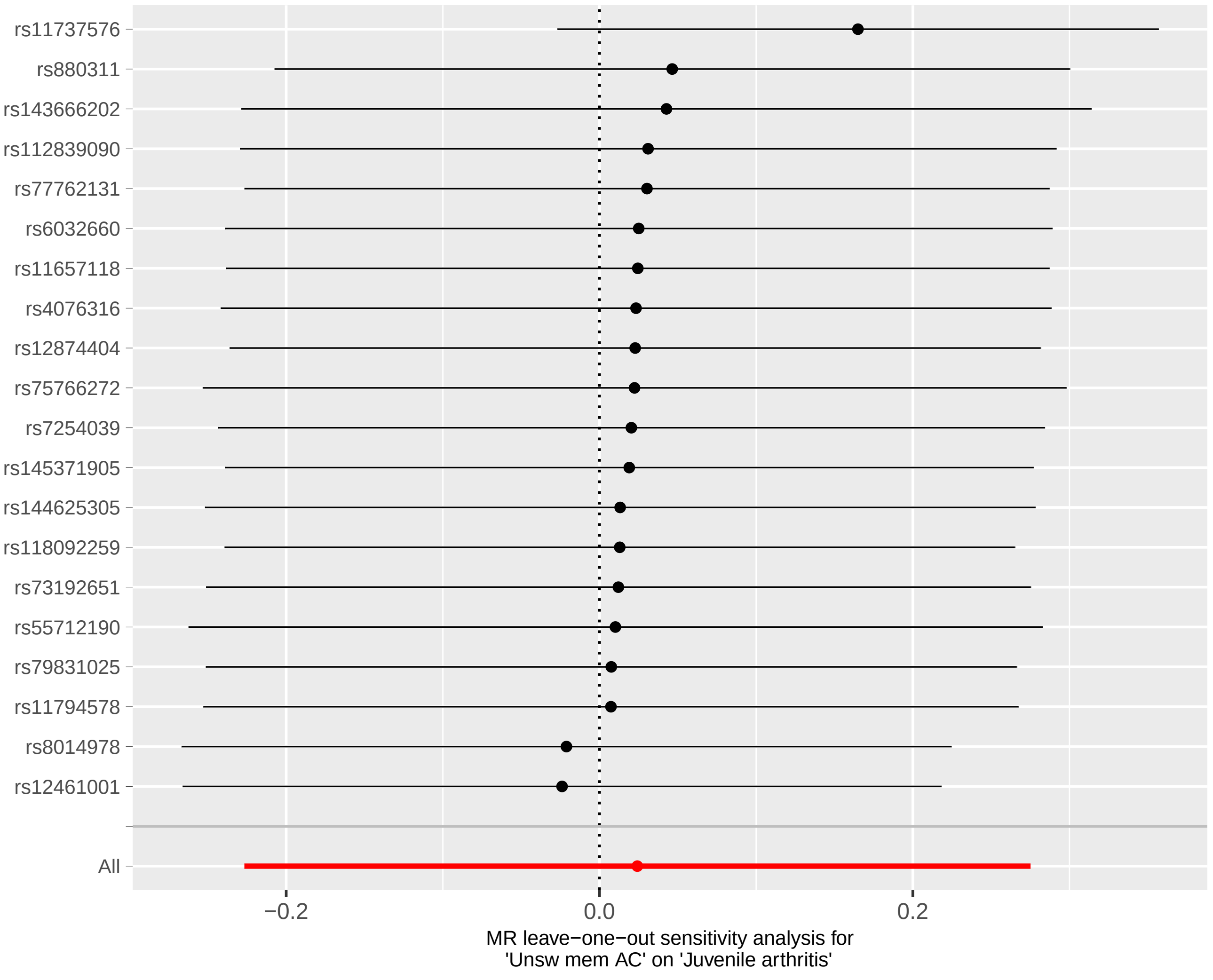

rs11737576
rs880311
rs143666202
rs112839090
rs77762131
rs6032660
rs11657118
rs4076316
rs12874404
rs75766272
rs7254039
rs145371905
rs144625305
rs118092259
rs73192651
rs55712190
rs79831025
rs11794578
rs8014978
rs12461001
All
−0.2
0.0
0.2
MR leave−one−out sensitivity analysis for
'Unsw mem AC' on 'Juvenile arthritis'

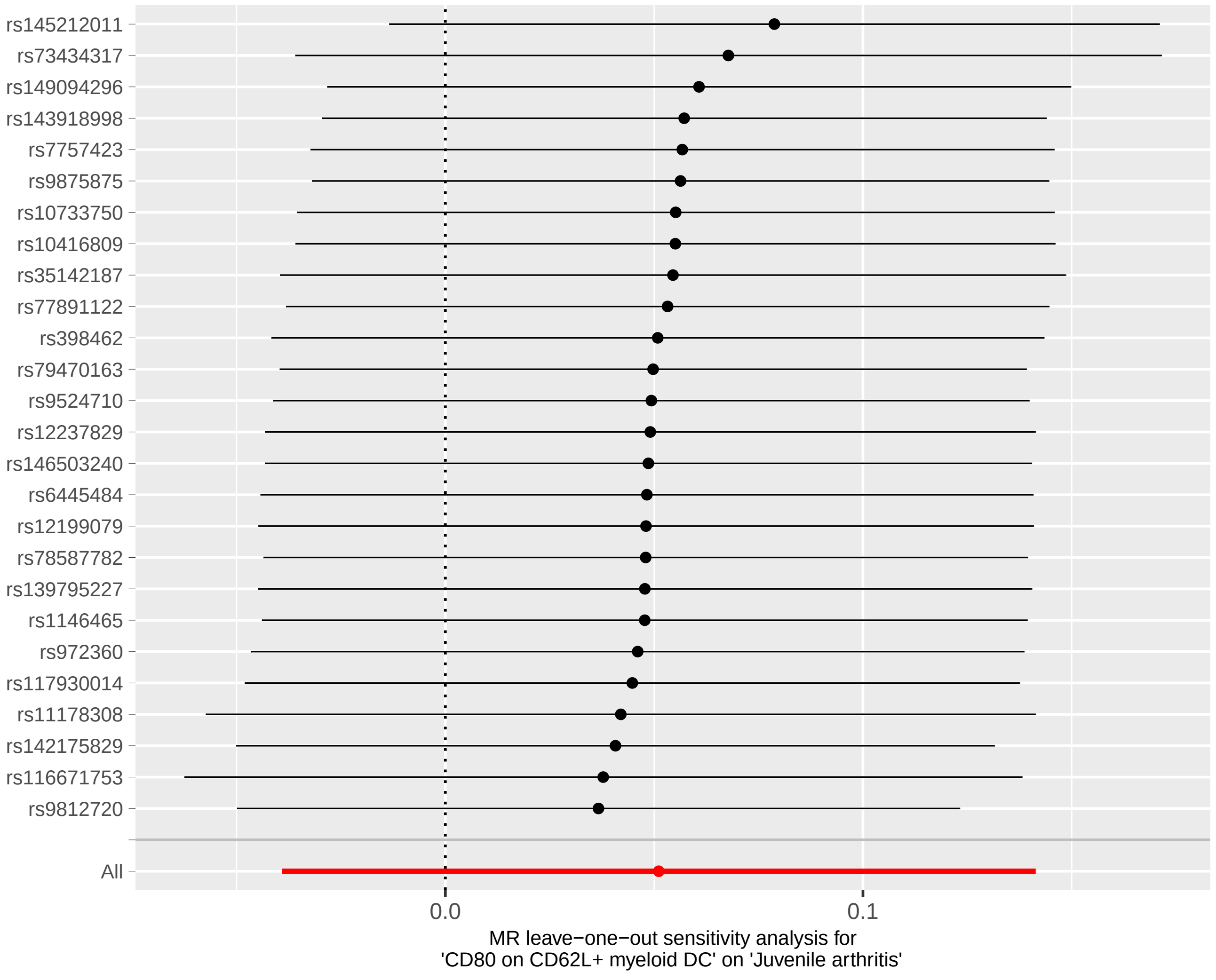
rs145212011
rs73434317
rs149094296
rs143918998
rs7757423
rs9875875
rs10733750
rs10416809
rs35142187
rs77891122
rs398462
rs79470163
rs9524710
rs12237829
rs146503240
rs6445484
rs12199079
rs78587782
rs139795227
rs1146465
rs972360
rs117930014
rs11178308
rs142175829
rs116671753
rs9812720
All
0.0
0.1
MR leave−one−out sensitivity analysis for
'CD80 on CD62L+ myeloid DC' on 'Juvenile arthritis'

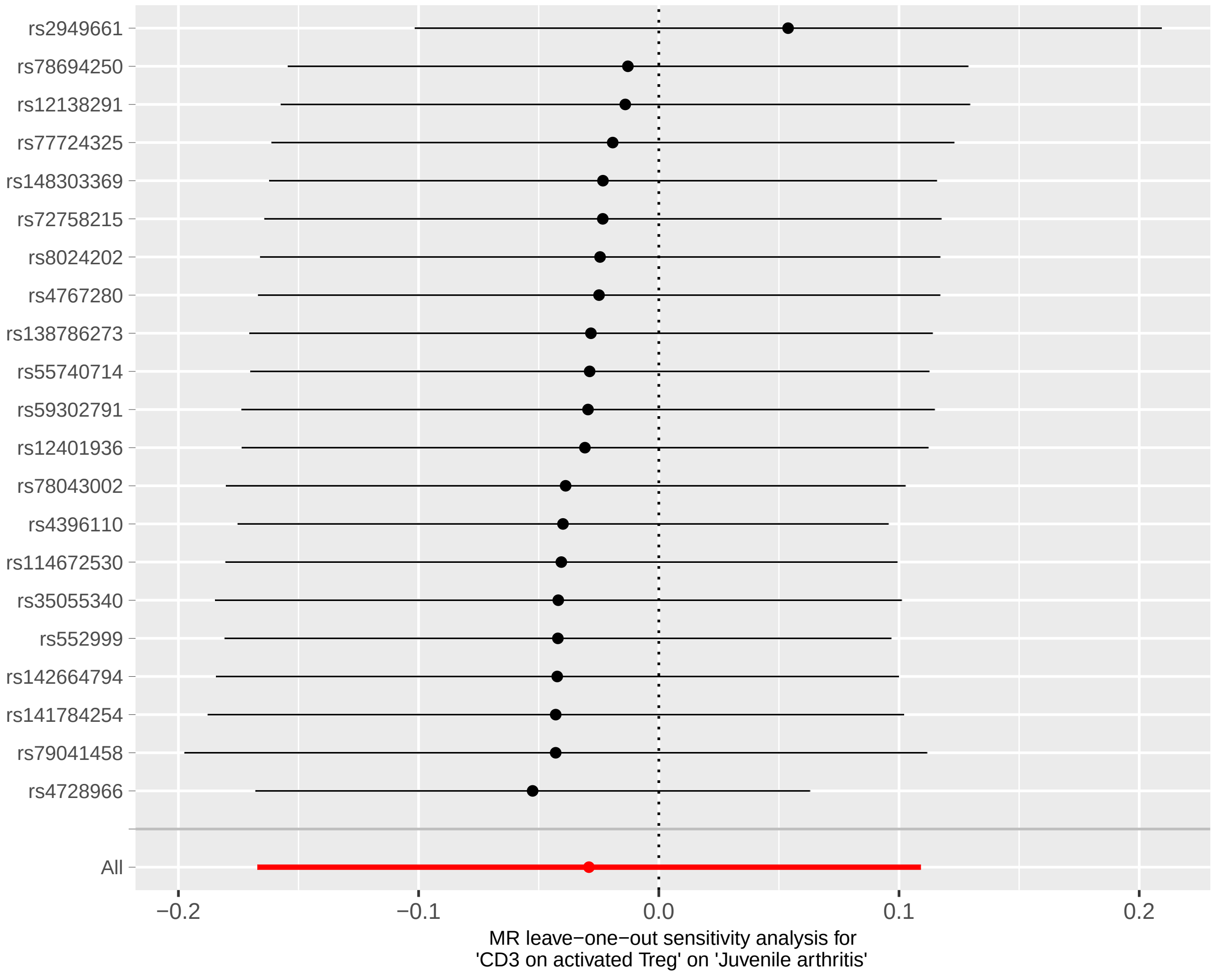
rs2949661
rs78694250
rs12138291
rs77724325
rs148303369
rs72758215
rs8024202
rs4767280
rs138786273
rs55740714
rs59302791
rs12401936
rs78043002
rs4396110
rs114672530
rs35055340
rs552999
rs142664794
rs141784254
rs79041458
rs4728966
All
−0.2
−0.1
0.0
0.1
0.2
MR leave−one−out sensitivity analysis for
'CD3 on activated Treg' on 'Juvenile arthritis'

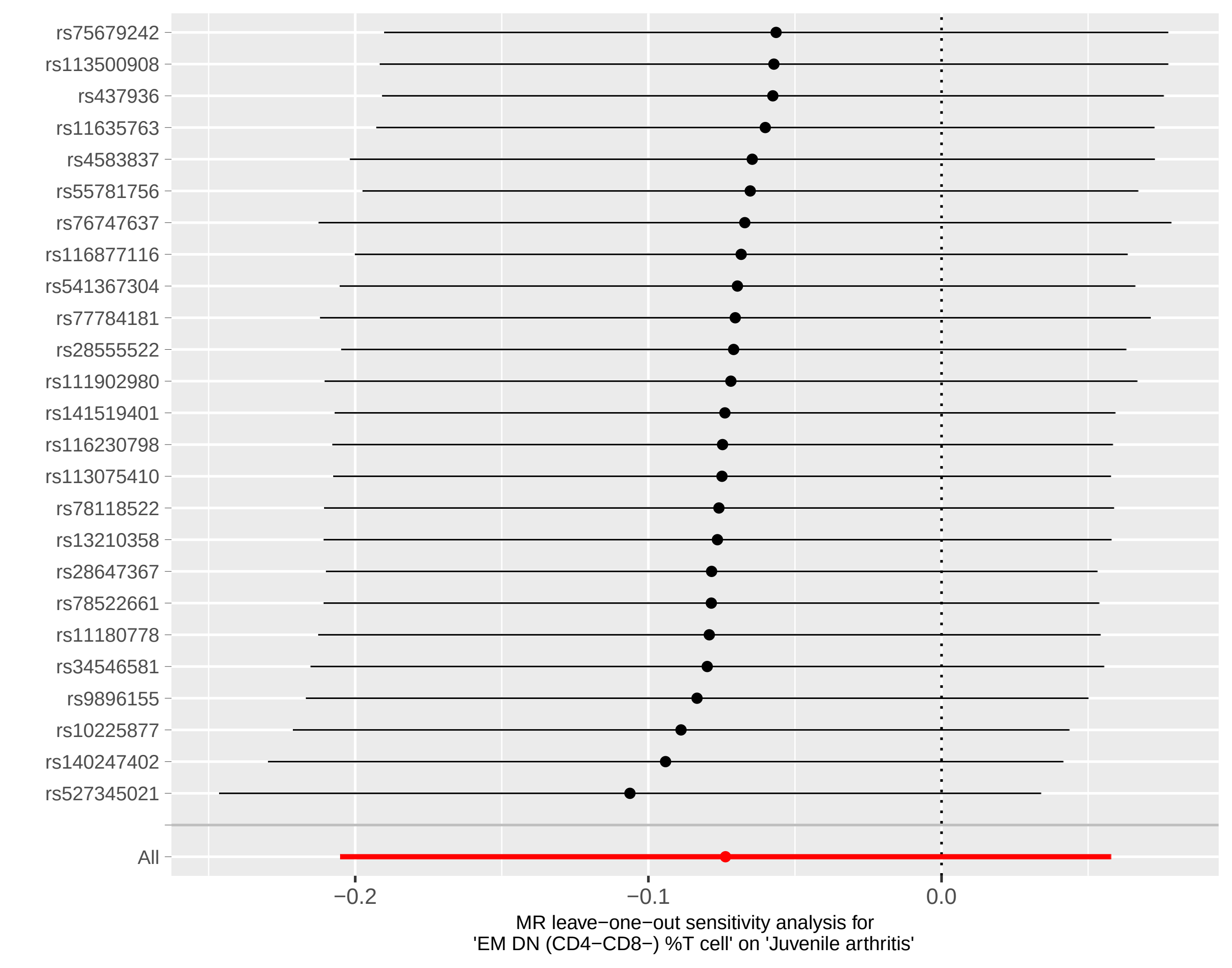
rs75679242
rs113500908
rs437936
rs11635763
rs4583837
rs55781756
rs76747637
rs116877116
rs541367304
rs77784181
rs28555522
rs111902980
rs141519401
rs116230798
rs113075410
rs78118522
rs13210358
rs28647367
rs78522661
rs11180778
rs34546581
rs9896155
rs10225877
rs140247402
rs527345021
All
−0.2
−0.1
0.0
MR leave−one−out sensitivity analysis for
'EM DN (CD4−CD8−) %T cell' on 'Juvenile arthritis'

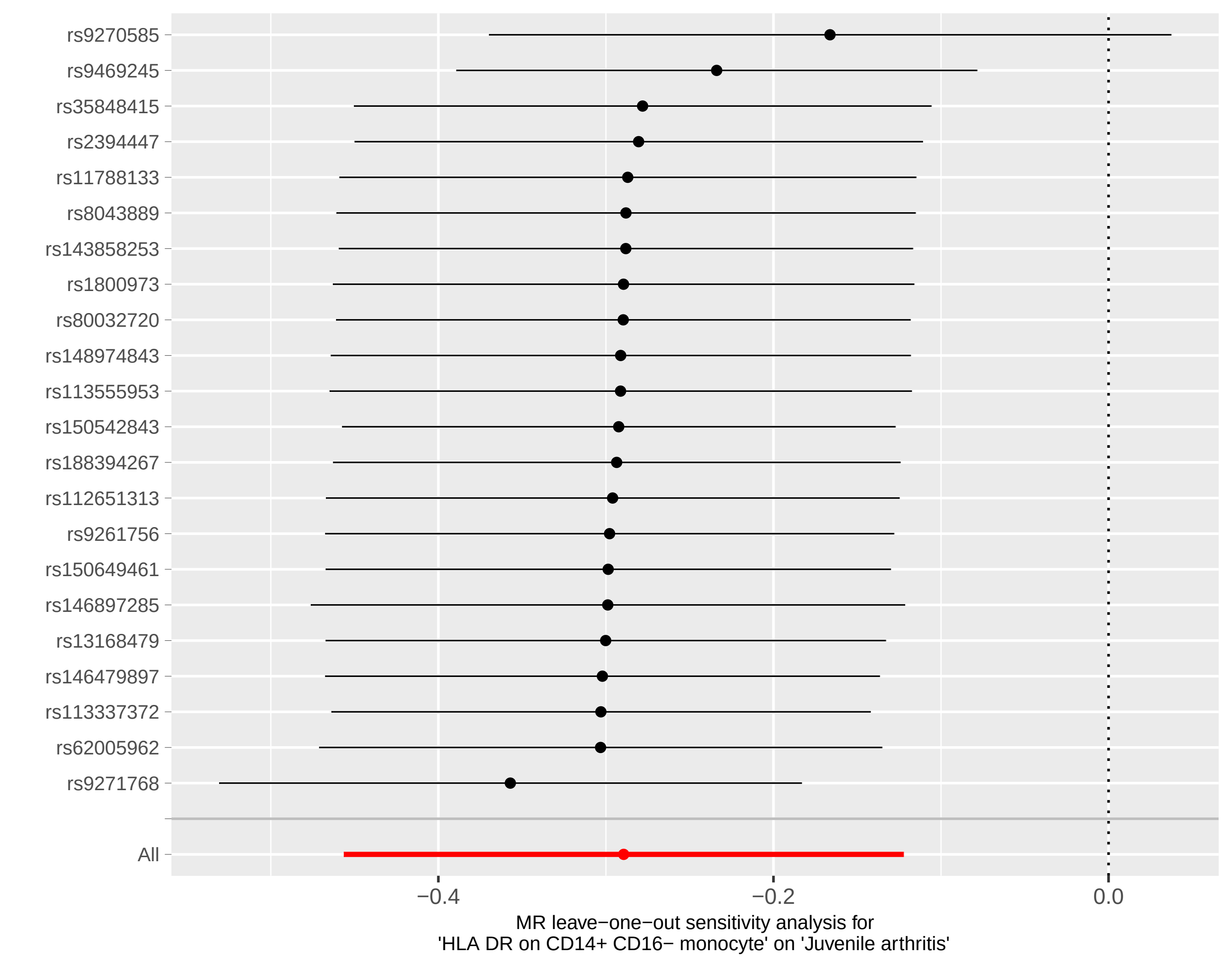

rs9270585
rs9469245
rs35848415
rs2394447
rs11788133
rs8043889
rs143858253
rs1800973
rs80032720
rs148974843
rs113555953
rs150542843
rs188394267
rs112651313
rs9261756
rs150649461
rs146897285
rs13168479
rs146479897
rs113337372
rs62005962
rs9271768
All
−0.4
−0.2
0.0
MR leave−one−out sensitivity analysis for
'HLA DR on CD14+ CD16− monocyte' on 'Juvenile arthritis'

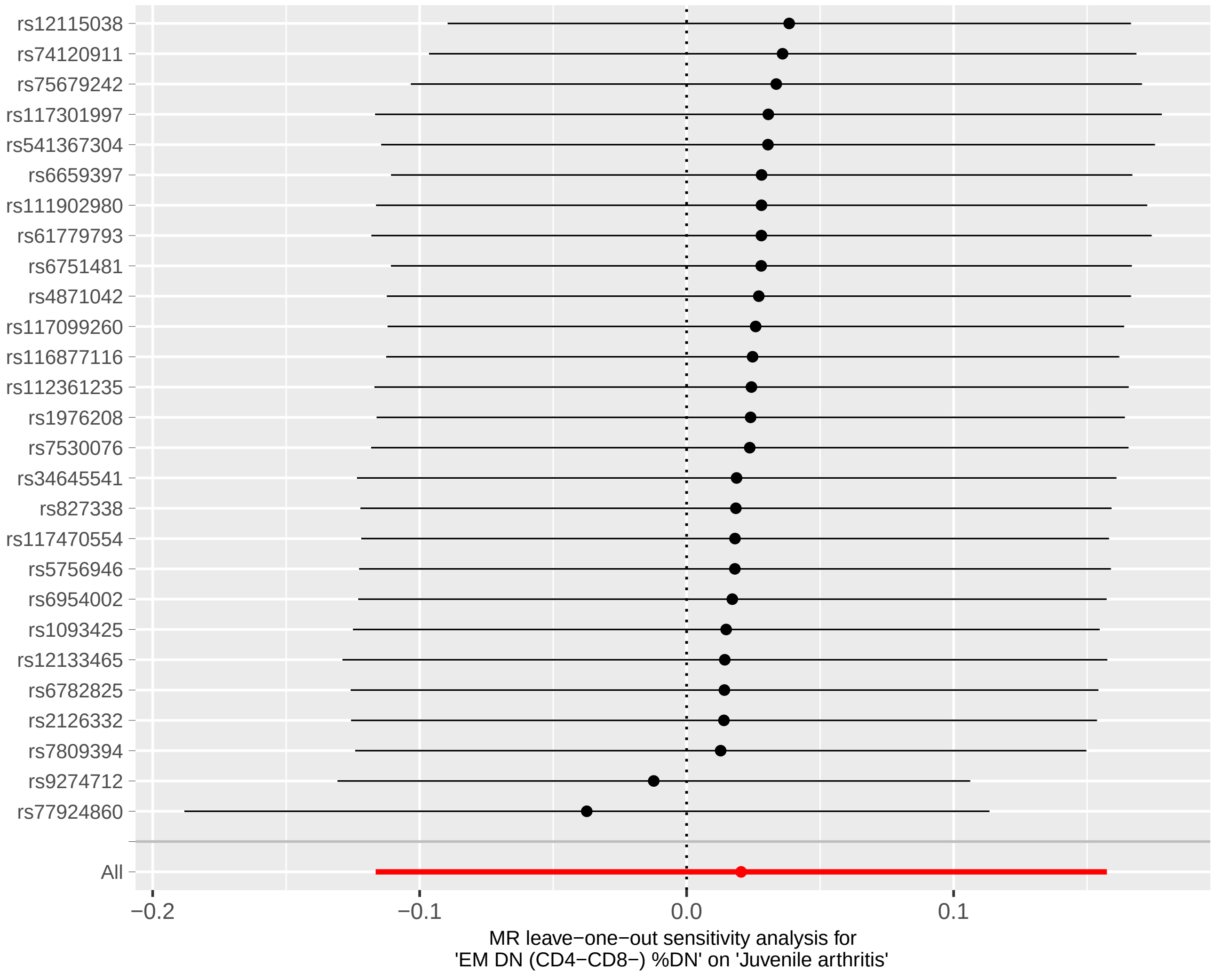
rs12115038
rs74120911
rs75679242
rs117301997
rs541367304
rs6659397
rs111902980
rs61779793
rs6751481
rs4871042
rs117099260
rs116877116
rs112361235
rs1976208
rs7530076
rs34645541
rs827338
rs117470554
rs5756946
rs6954002
rs1093425
rs12133465
rs6782825
rs2126332
rs7809394
rs9274712
rs77924860
All
−0.2
−0.1
0.0
0.1
MR leave−one−out sensitivity analysis for
'EM DN (CD4−CD8−) %DN' on 'Juvenile arthritis'

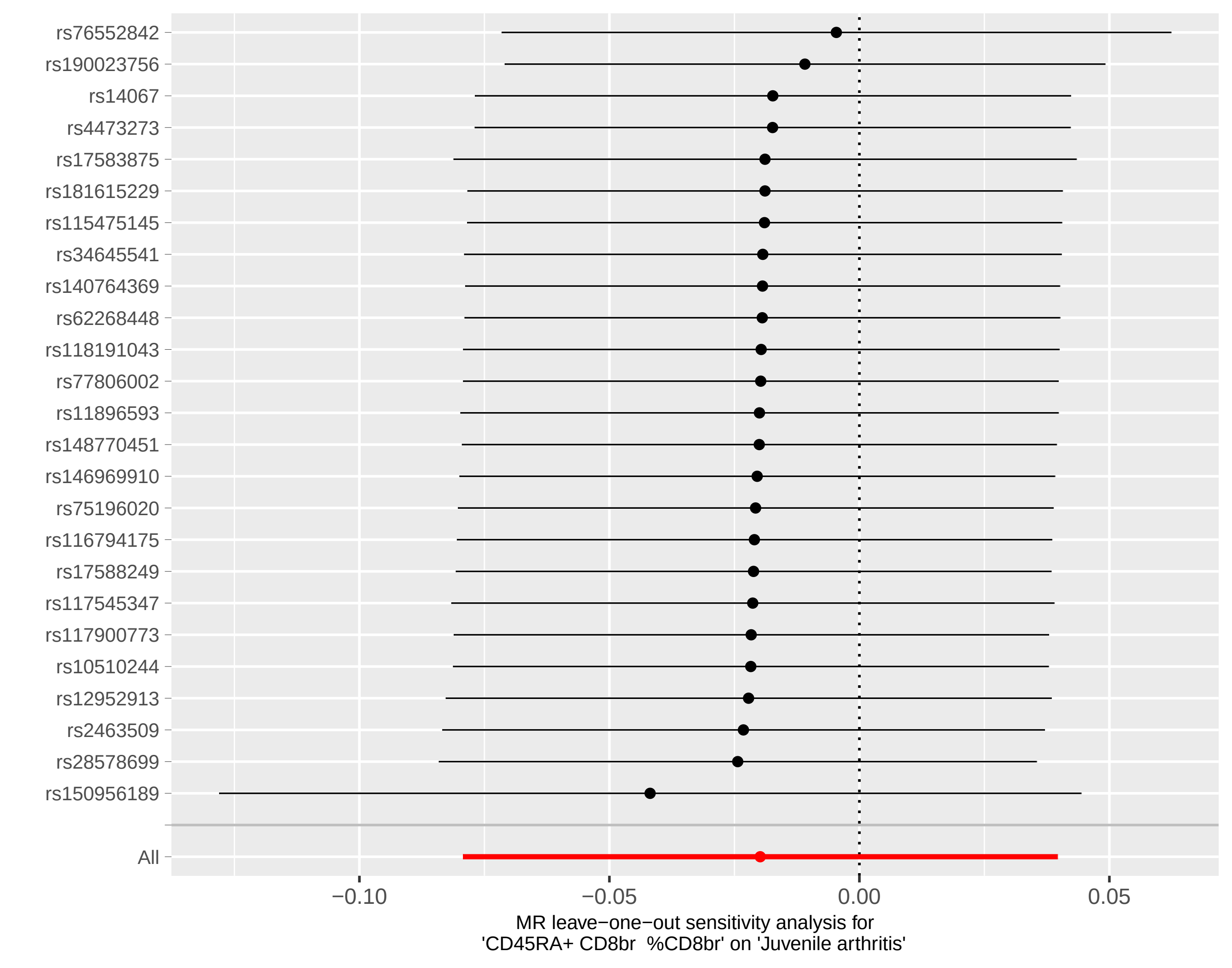
rs76552842
rs190023756
rs14067
rs4473273
rs17583875
rs181615229
rs115475145
rs34645541
rs140764369
rs62268448
rs118191043
rs77806002
rs11896593
rs148770451
rs146969910
rs75196020
rs116794175
rs17588249
rs117545347
rs117900773
rs10510244
rs12952913
rs2463509
rs28578699
rs150956189
All
−0.10
−0.05
0.00
0.05
MR leave−one−out sensitivity analysis for
'CD45RA+ CD8br %CD8br' on 'Juvenile arthritis'

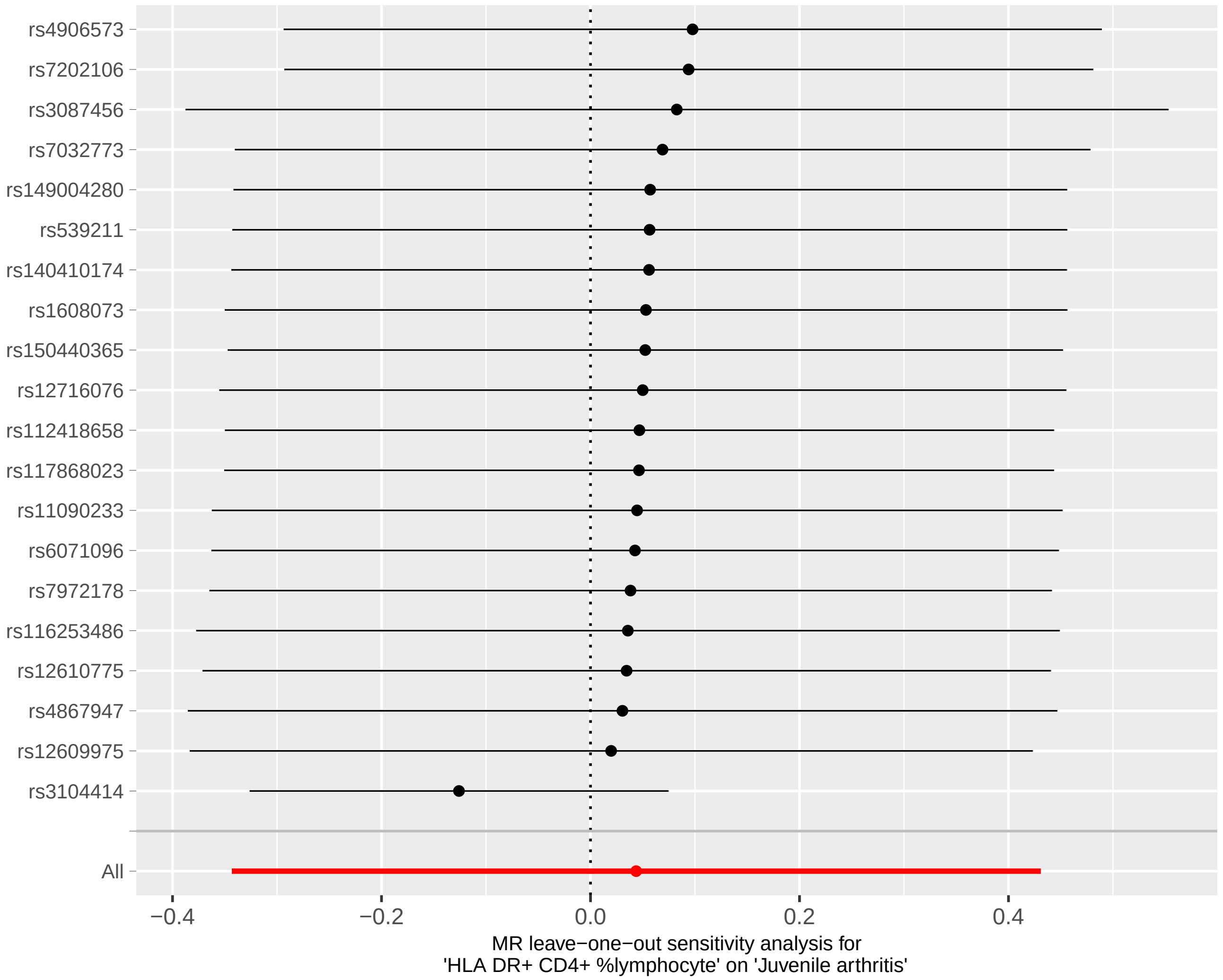
rs4906573
rs7202106
rs3087456
rs7032773
rs149004280
rs539211
rs140410174
rs1608073
rs150440365
rs12716076
rs112418658
rs117868023
rs11090233
rs6071096
rs7972178
rs116253486
rs12610775
rs4867947
rs12609975
rs3104414
All
−0.4
−0.2
0.0
0.2
0.4
MR leave−one−out sensitivity analysis for
'HLA DR+ CD4+ %lymphocyte' on 'Juvenile arthritis'

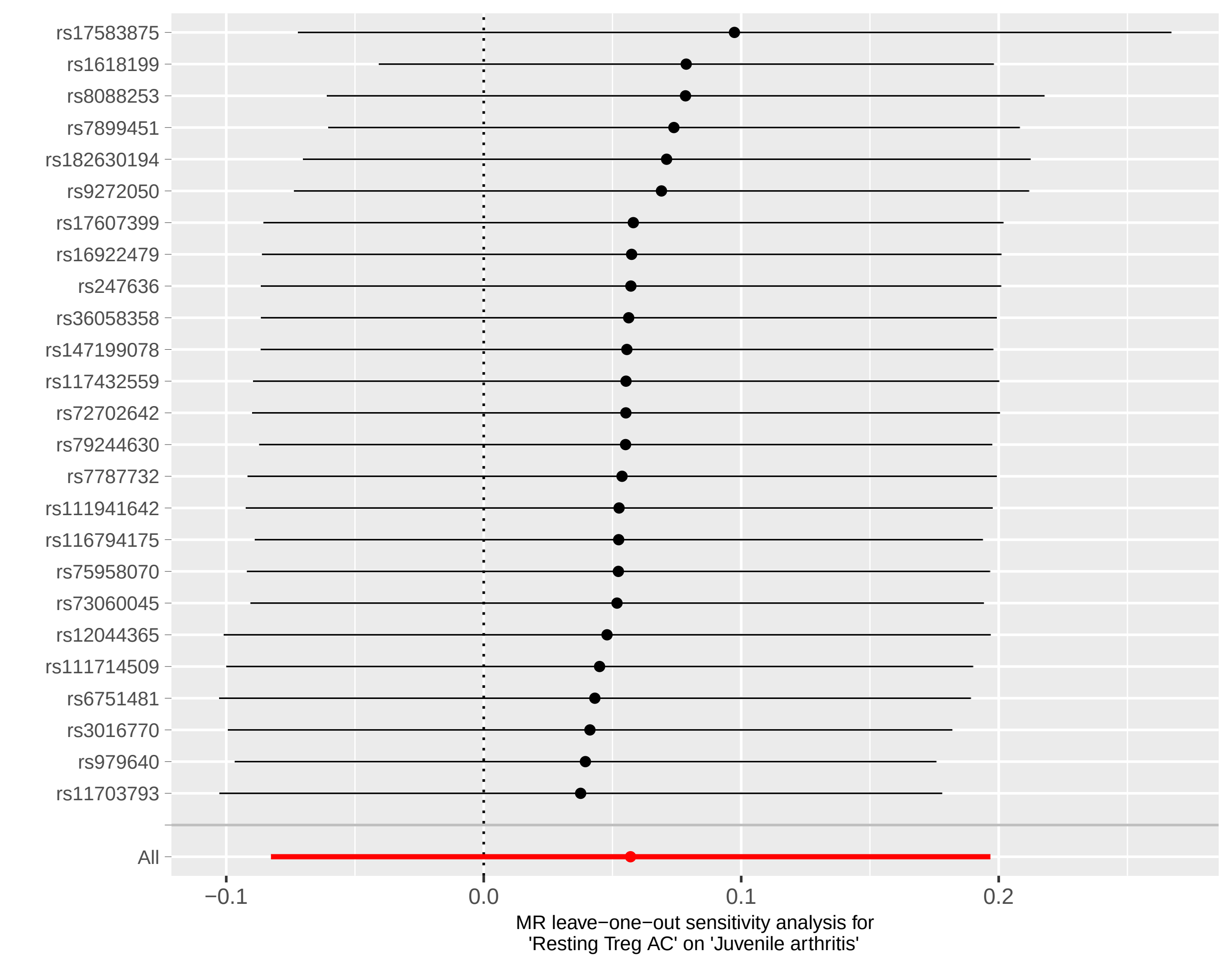
rs17583875
rs1618199
rs8088253
rs7899451
rs182630194
rs9272050
rs17607399
rs16922479
rs247636
rs36058358
rs147199078
rs117432559
rs72702642
rs79244630
rs7787732
rs111941642
rs116794175
rs75958070
rs73060045
rs12044365
rs111714509
rs6751481
rs3016770
rs979640
rs11703793
All
−0.1
0.0
0.1
0.2
MR leave−one−out sensitivity analysis for
'Resting Treg AC' on 'Juvenile arthritis'

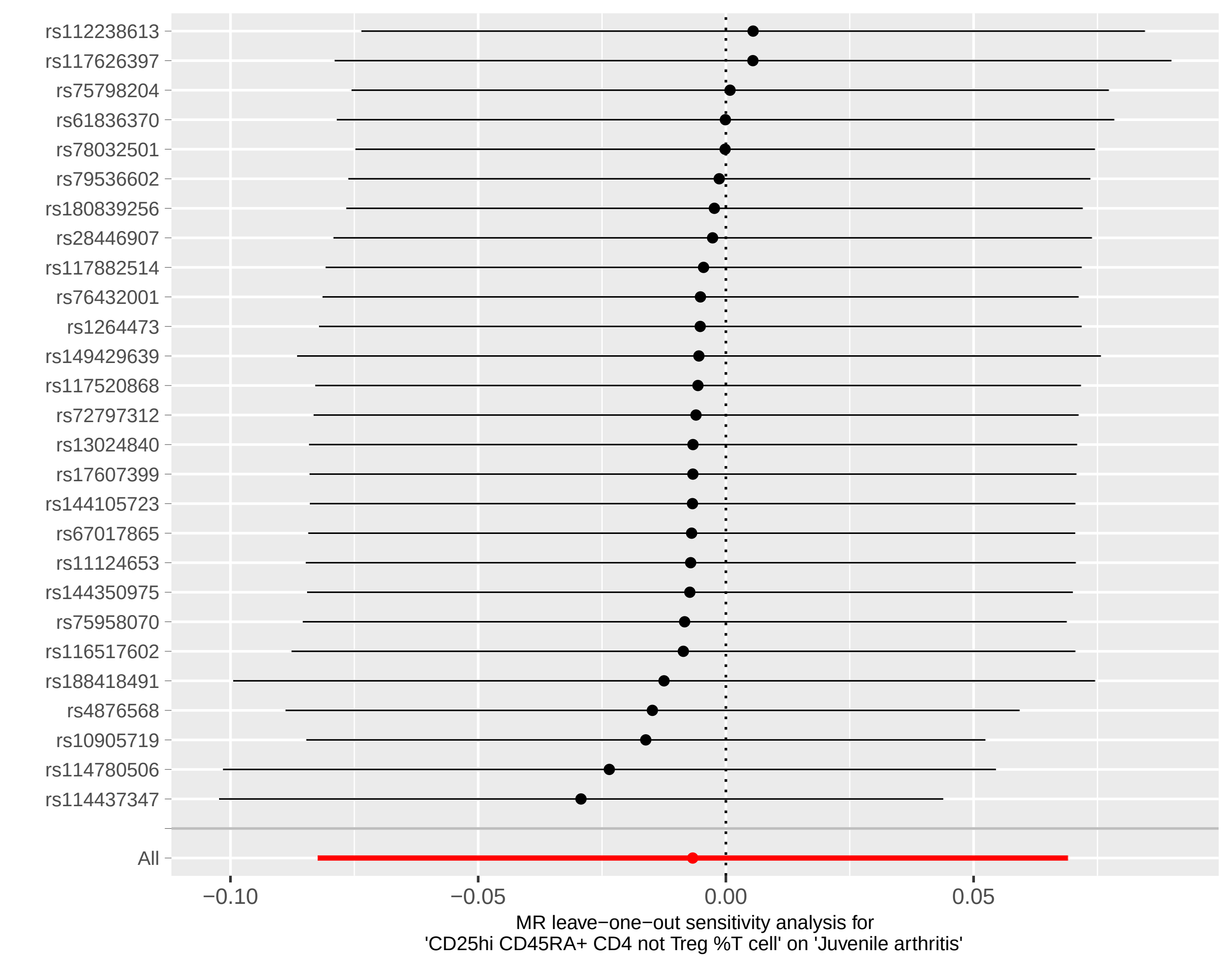

rs112238613
rs117626397
rs75798204
rs61836370
rs78032501
rs79536602
rs180839256
rs28446907
rs117882514
rs76432001
rs1264473
rs149429639
rs117520868
rs72797312
rs13024840
rs17607399
rs144105723
rs67017865
rs11124653
rs144350975
rs75958070
rs116517602
rs188418491
rs4876568
rs10905719
rs114780506
rs114437347
All
−0.10
−0.05
0.00
0.05
MR leave−one−out sensitivity analysis for
'CD25hi CD45RA+ CD4 not Treg %T cell' on 'Juvenile arthritis'

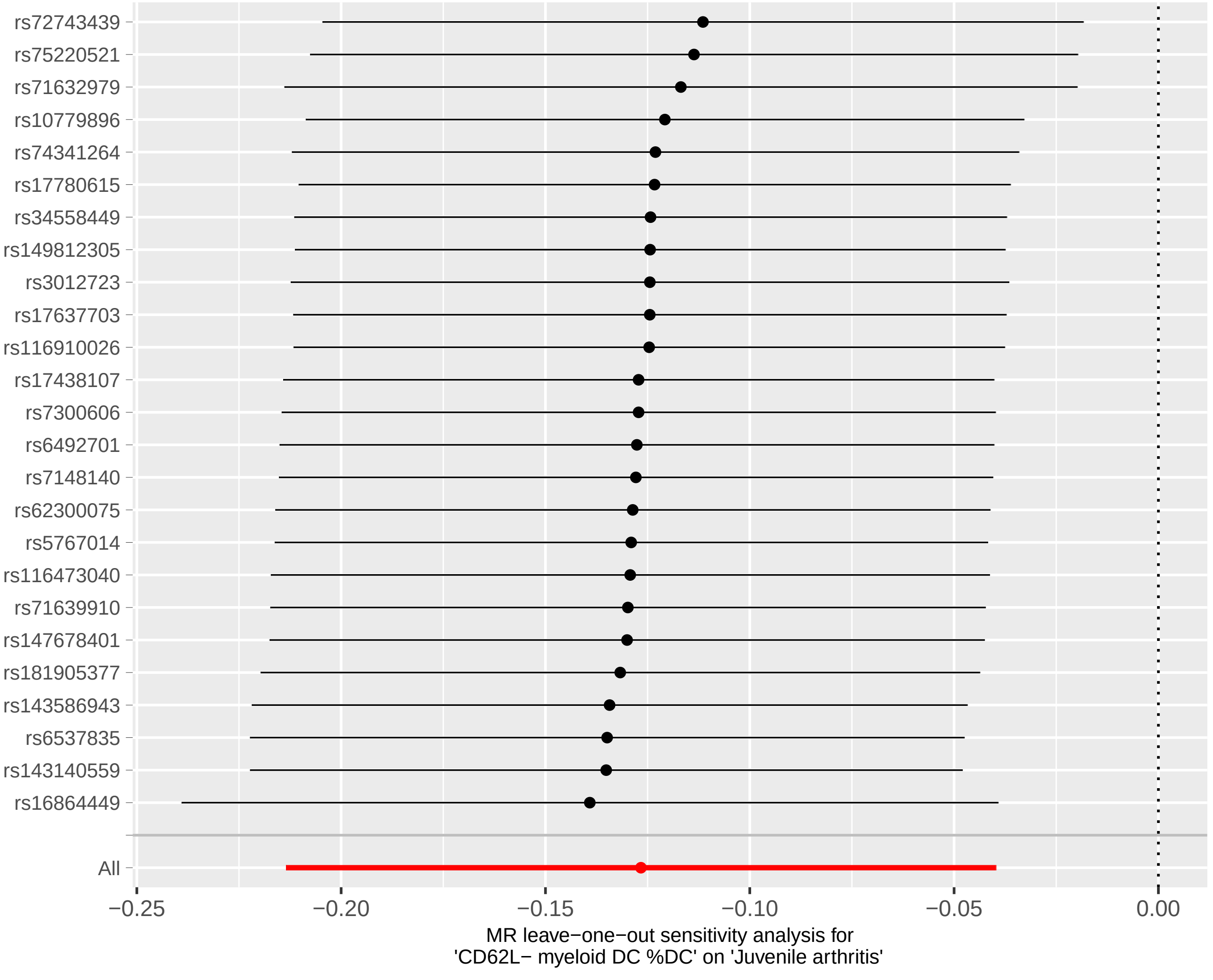

rs72743439
rs75220521
rs71632979
rs10779896
rs74341264
rs17780615
rs34558449
rs149812305
rs3012723
rs17637703
rs116910026
rs17438107
rs7300606
rs6492701
rs7148140
rs62300075
rs5767014
rs116473040
rs71639910
rs147678401
rs181905377
rs143586943
rs6537835
rs143140559
rs16864449
All
−0.25
−0.20
−0.15
−0.10
−0.05
0.00
MR leave−one−out sensitivity analysis for
'CD62L− myeloid DC %DC' on 'Juvenile arthritis'

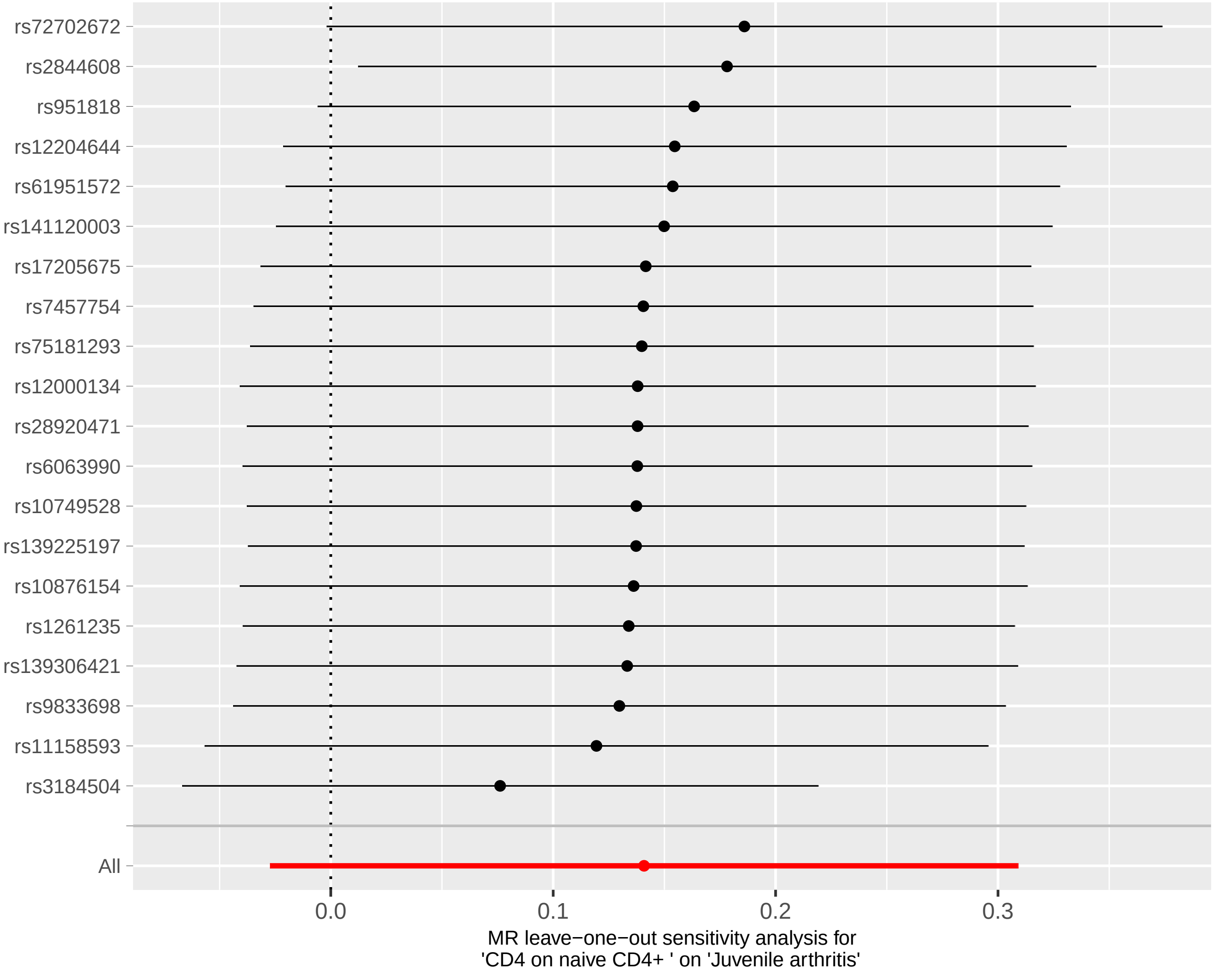

rs72702672
rs2844608
rs951818
rs12204644
rs61951572
rs141120003
rs17205675
rs7457754
rs75181293
rs12000134
rs28920471
rs6063990
rs10749528
rs139225197
rs10876154
rs1261235
rs139306421
rs9833698
rs11158593
rs3184504
All
0.0
0.1
0.2
0.3
MR leave−one−out sensitivity analysis for
'CD4 on naive CD4+ ' on 'Juvenile arthritis'

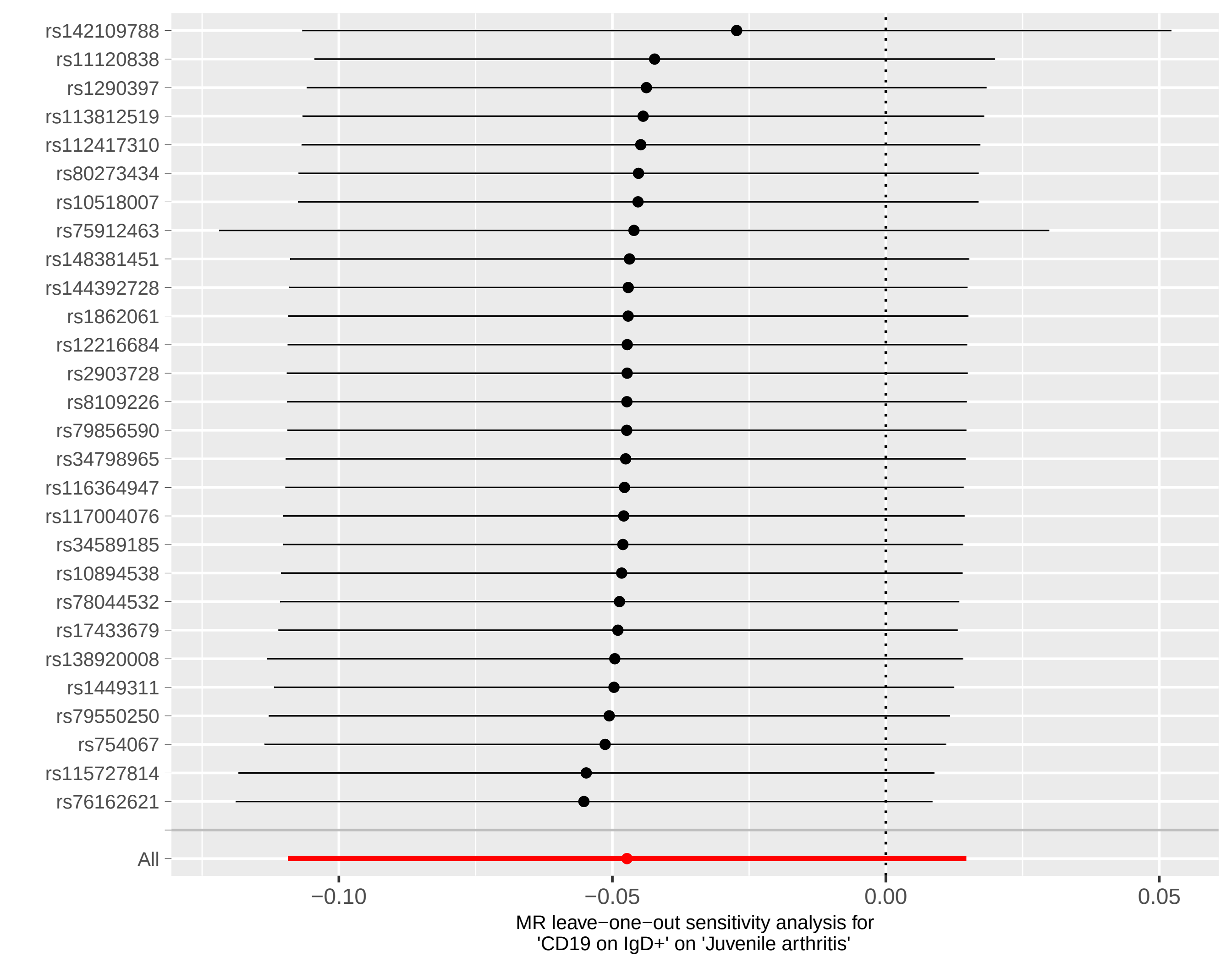
rs142109788
rs11120838
rs1290397
rs113812519
rs112417310
rs80273434
rs10518007
rs75912463
rs148381451
rs144392728
rs1862061
rs12216684
rs2903728
rs8109226
rs79856590
rs34798965
rs116364947
rs117004076
rs34589185
rs10894538
rs78044532
rs17433679
rs138920008
rs1449311
rs79550250
rs754067
rs115727814
rs76162621
All
−0.10
−0.05
0.00
0.05
MR leave−one−out sensitivity analysis for
'CD19 on IgD+' on 'Juvenile arthritis'

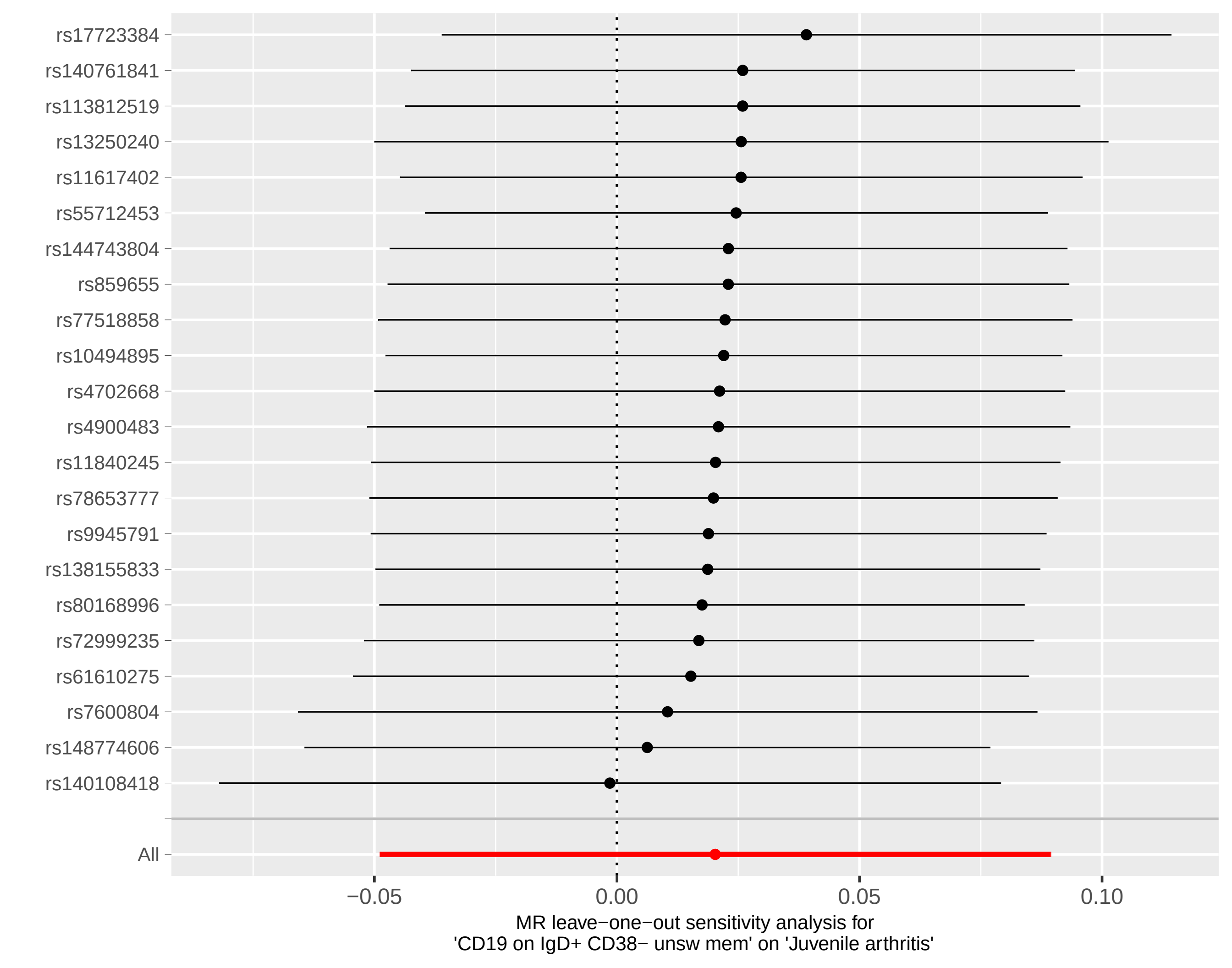
rs17723384
rs140761841
rs113812519
rs13250240
rs11617402
rs55712453
rs144743804
rs859655
rs77518858
rs10494895
rs4702668
rs4900483
rs11840245
rs78653777
rs9945791
rs138155833
rs80168996
rs72999235
rs61610275
rs7600804
rs148774606
rs140108418
All
−0.05
0.00
0.05
0.10
MR leave−one−out sensitivity analysis for
'CD19 on IgD+ CD38− unsw mem' on 'Juvenile arthritis'

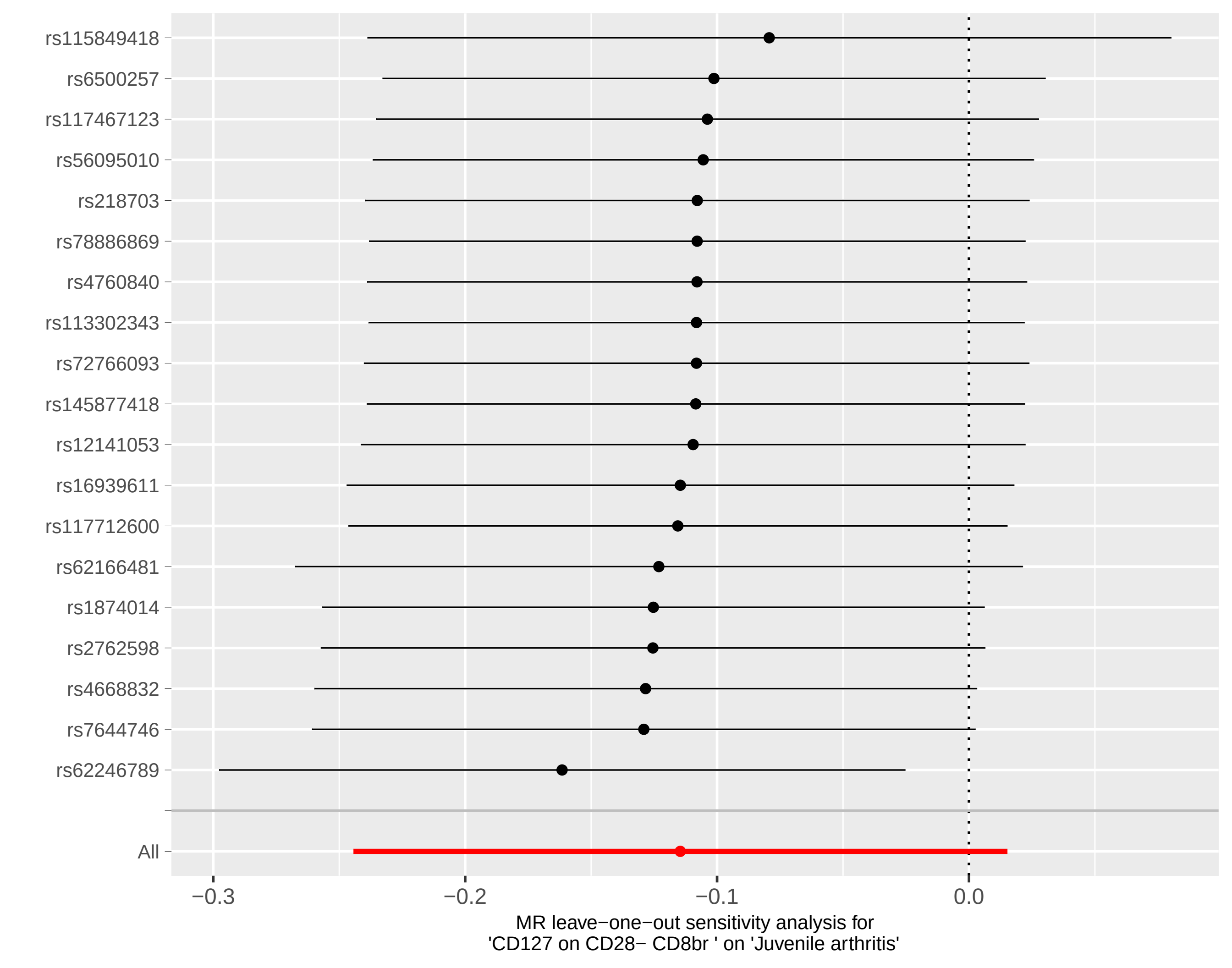

rs115849418
rs6500257
rs117467123
rs56095010
rs218703
rs78886869
rs4760840
rs113302343
rs72766093
rs145877418
rs12141053
rs16939611
rs117712600
rs62166481
rs1874014
rs2762598
rs4668832
rs7644746
rs62246789
All
−0.3
−0.2
−0.1
0.0
MR leave−one−out sensitivity analysis for
'CD127 on CD28− CD8br ' on 'Juvenile arthritis'

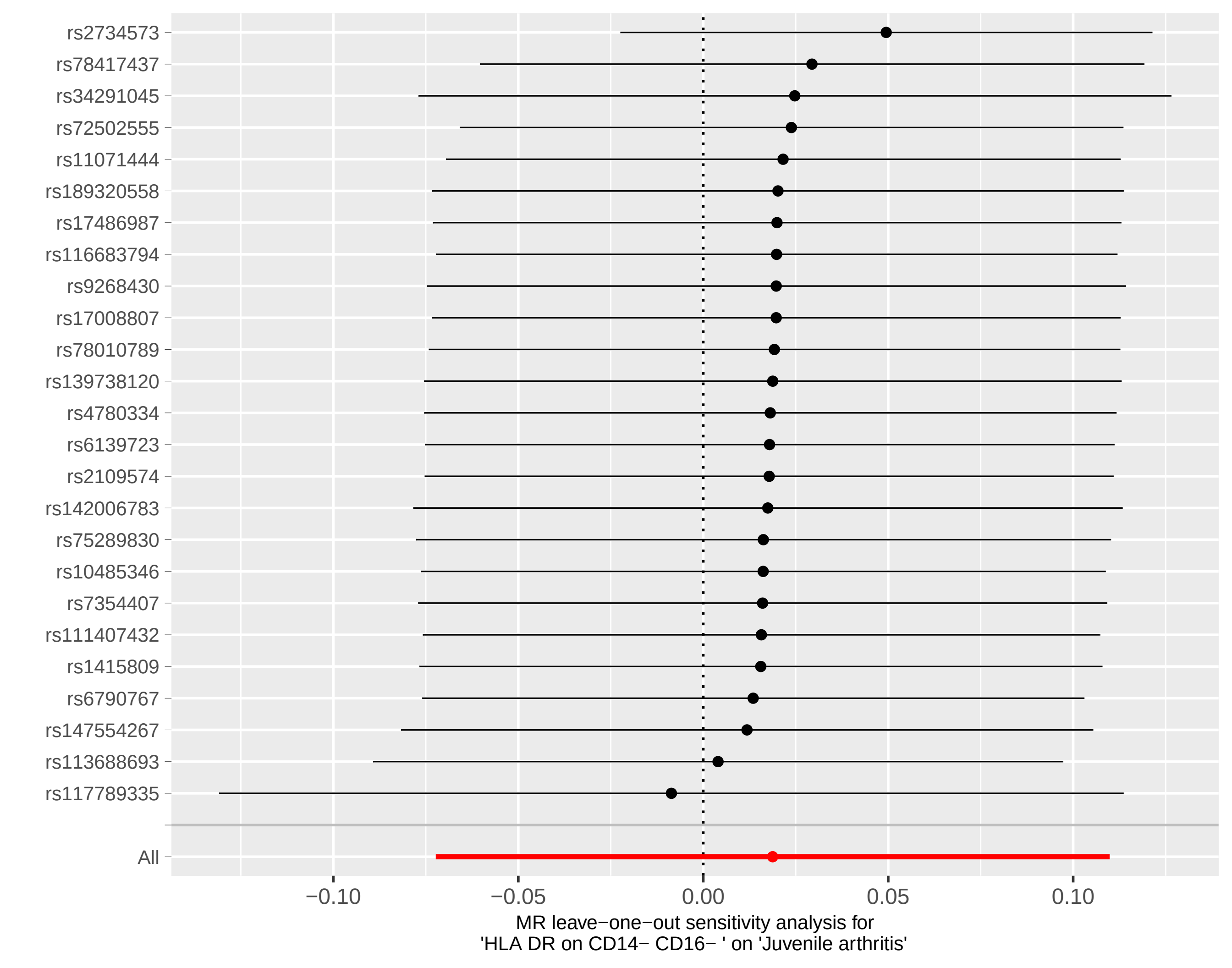
rs2734573
rs78417437
rs34291045
rs72502555
rs11071444
rs189320558
rs17486987
rs116683794
rs9268430
rs17008807
rs78010789
rs139738120
rs4780334
rs6139723
rs2109574
rs142006783
rs75289830
rs10485346
rs7354407
rs111407432
rs1415809
rs6790767
rs147554267
rs113688693
rs117789335
All
−0.10
−0.05
0.00
0.05
0.10
MR leave−one−out sensitivity analysis for
'HLA DR on CD14− CD16− ' on 'Juvenile arthritis'

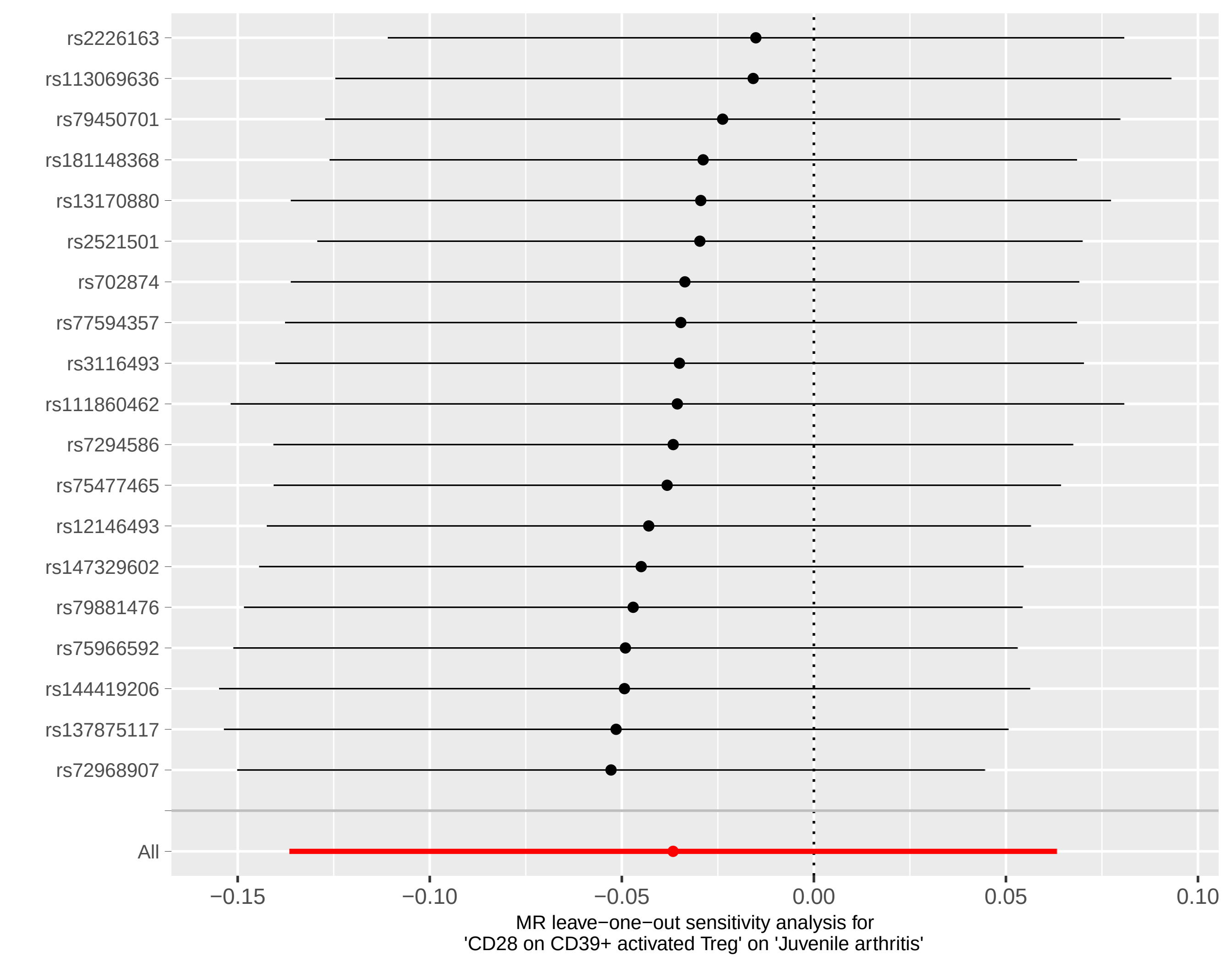
rs2226163
rs113069636
rs79450701
rs181148368
rs13170880
rs2521501
rs702874
rs77594357
rs3116493
rs111860462
rs7294586
rs75477465
rs12146493
rs147329602
rs79881476
rs75966592
rs144419206
rs137875117
rs72968907
All
−0.15
−0.10
−0.05
0.00
0.05
0.10
MR leave−one−out sensitivity analysis for
'CD28 on CD39+ activated Treg' on 'Juvenile arthritis'

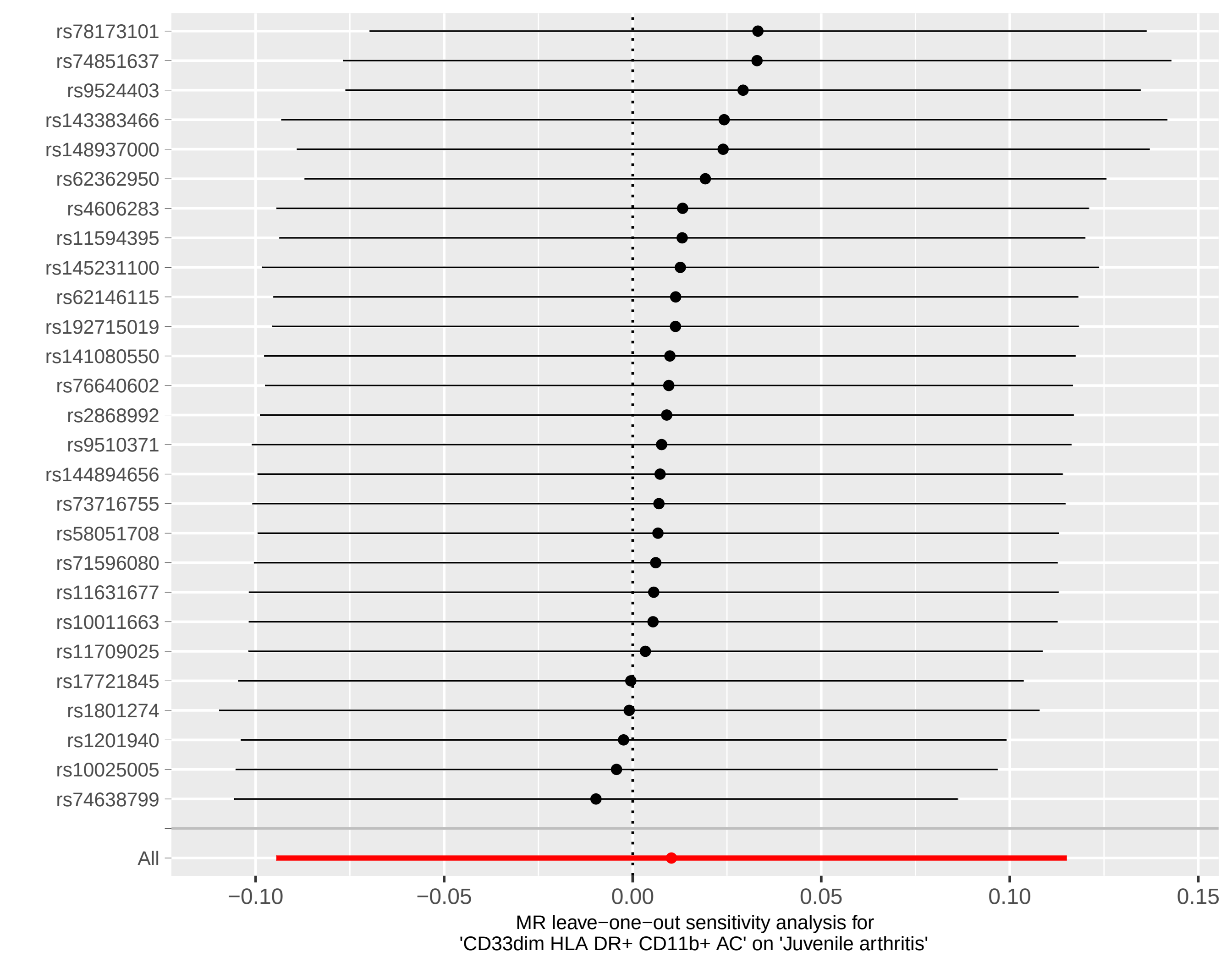
rs78173101
rs74851637
rs9524403
rs143383466
rs148937000
rs62362950
rs4606283
rs11594395
rs145231100
rs62146115
rs192715019
rs141080550
rs76640602
rs2868992
rs9510371
rs144894656
rs73716755
rs58051708
rs71596080
rs11631677
rs10011663
rs11709025
rs17721845
rs1801274
rs1201940
rs10025005
rs74638799
All
−0.10
−0.05
0.00
0.05
0.10
0.15
MR leave−one−out sensitivity analysis for
'CD33dim HLA DR+ CD11b+ AC' on 'Juvenile arthritis'

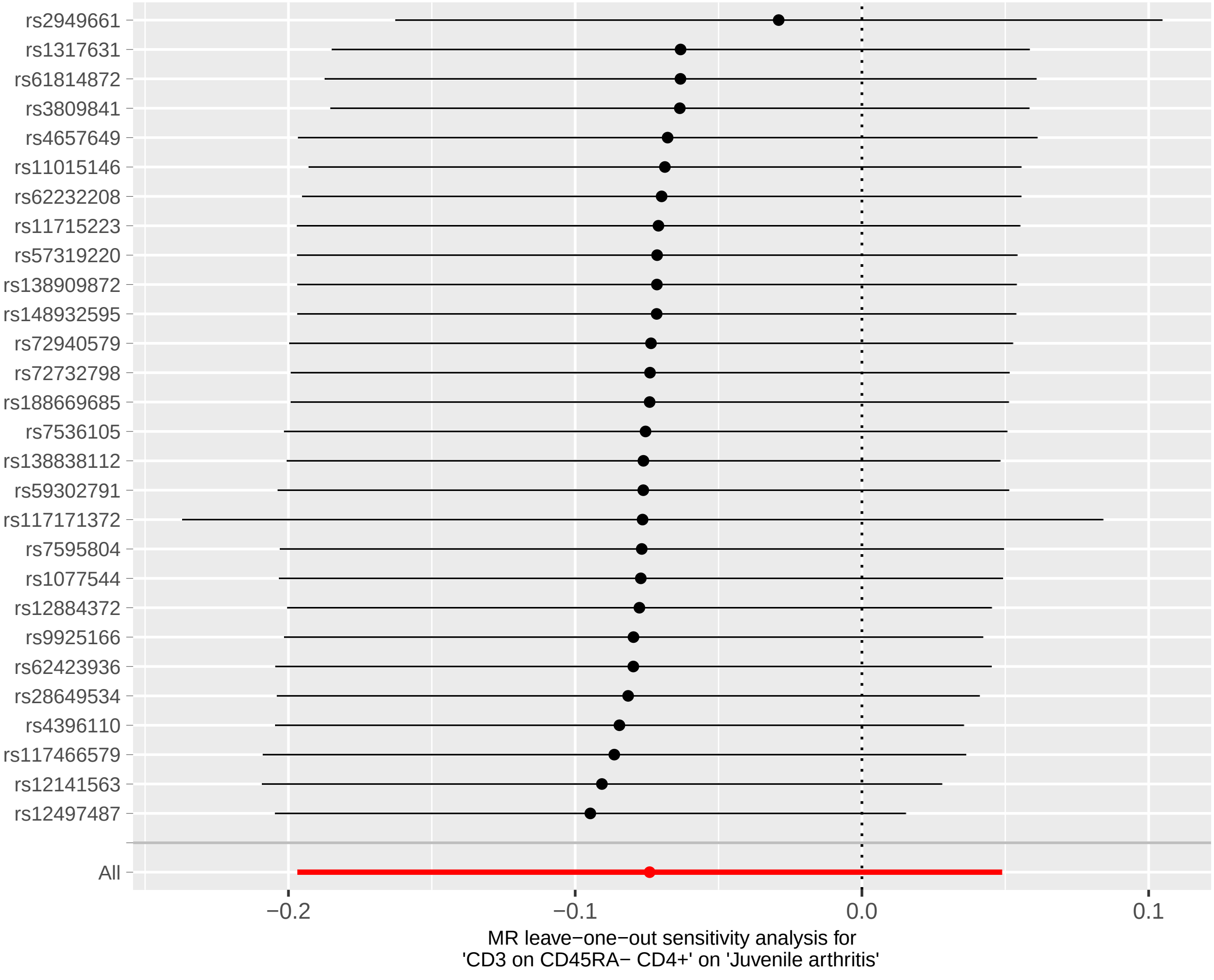
rs2949661
rs1317631
rs61814872
rs3809841
rs4657649
rs11015146
rs62232208
rs11715223
rs57319220
rs138909872
rs148932595
rs72940579
rs72732798
rs188669685
rs7536105
rs138838112
rs59302791
rs117171372
rs7595804
rs1077544
rs12884372
rs9925166
rs62423936
rs28649534
rs4396110
rs117466579
rs12141563
rs12497487
All
−0.2
−0.1
0.0
0.1
MR leave−one−out sensitivity analysis for
'CD3 on CD45RA− CD4+' on 'Juvenile arthritis'

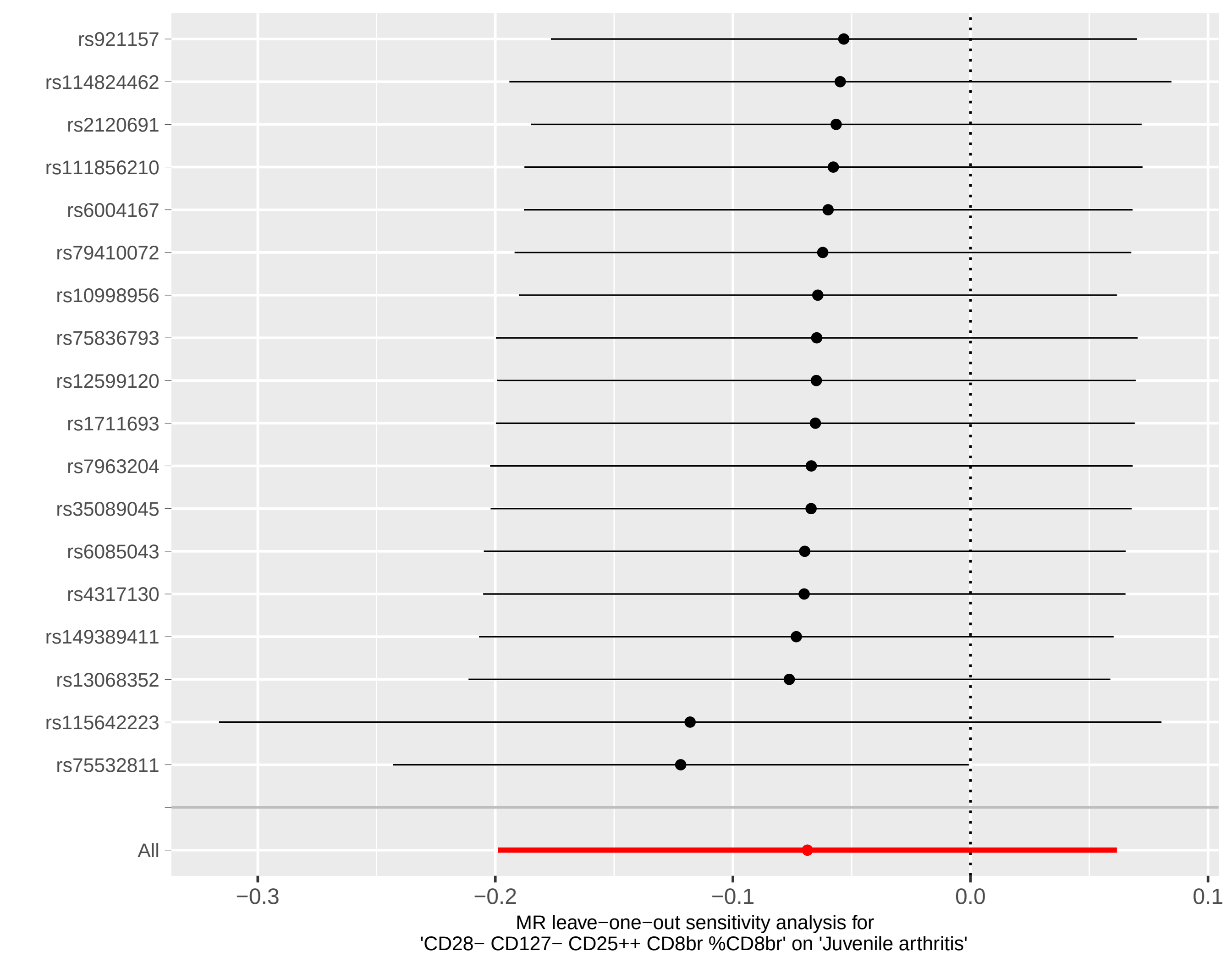
rs921157
rs114824462
rs2120691
rs111856210
rs6004167
rs79410072
rs10998956
rs75836793
rs12599120
rs1711693
rs7963204
rs35089045
rs6085043
rs4317130
rs149389411
rs13068352
rs115642223
rs75532811
All
−0.3
−0.2
−0.1
0.0
0.1
MR leave−one−out sensitivity analysis for
'CD28− CD127− CD25++ CD8br %CD8br' on 'Juvenile arthritis'

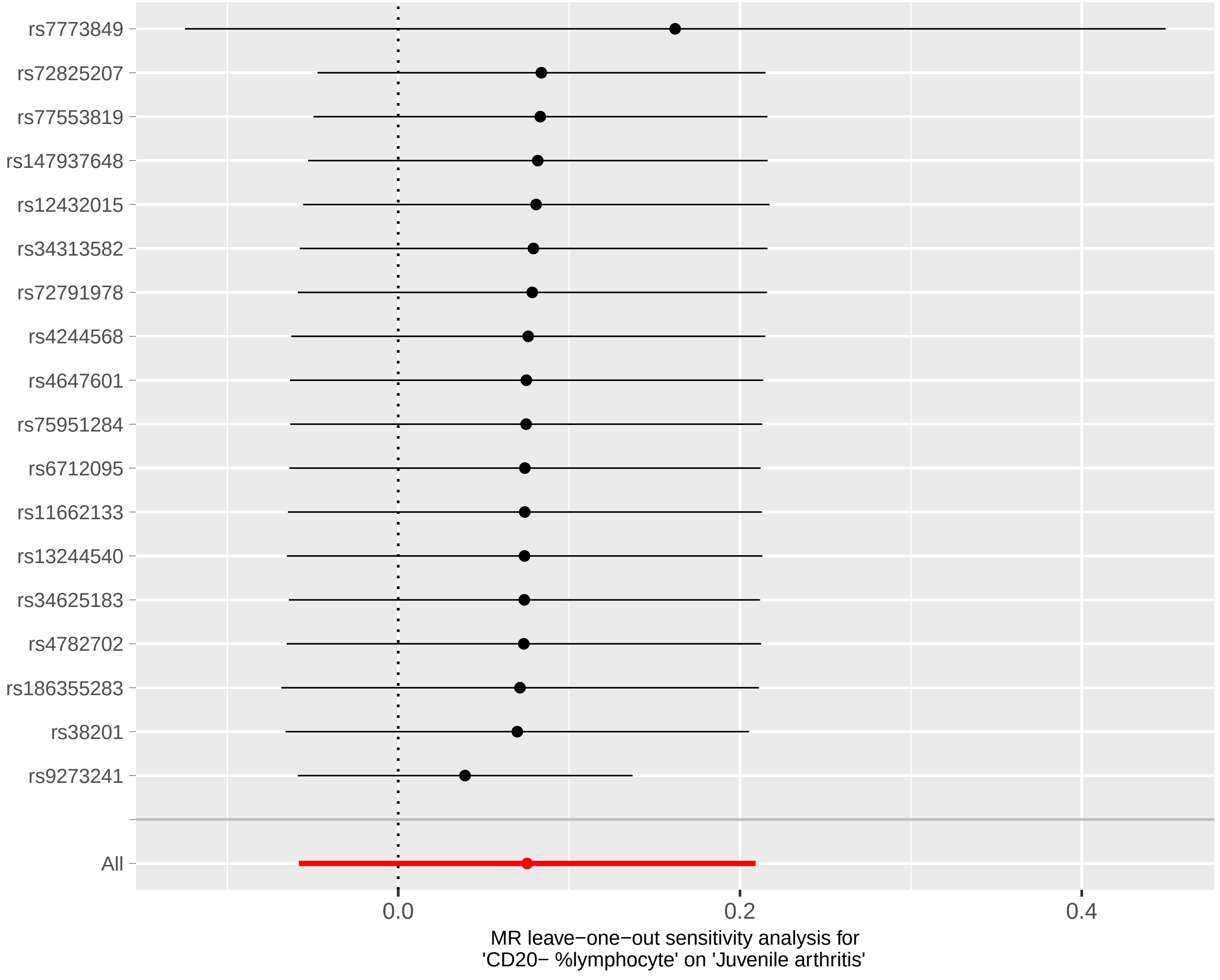

rs7773849
rs72825207
rs77553819
rs147937648
rs12432015
rs34313582
rs72791978
rs4244568
rs4647601
rs75951284
rs6712095
rs11662133
rs13244540
rs34625183
rs4782702
rs186355283
rs38201
rs9273241
All
0.0
0.2
0.4
MR leave−one−out sensitivity analysis for
'CD20− %lymphocyte' on 'Juvenile arthritis'

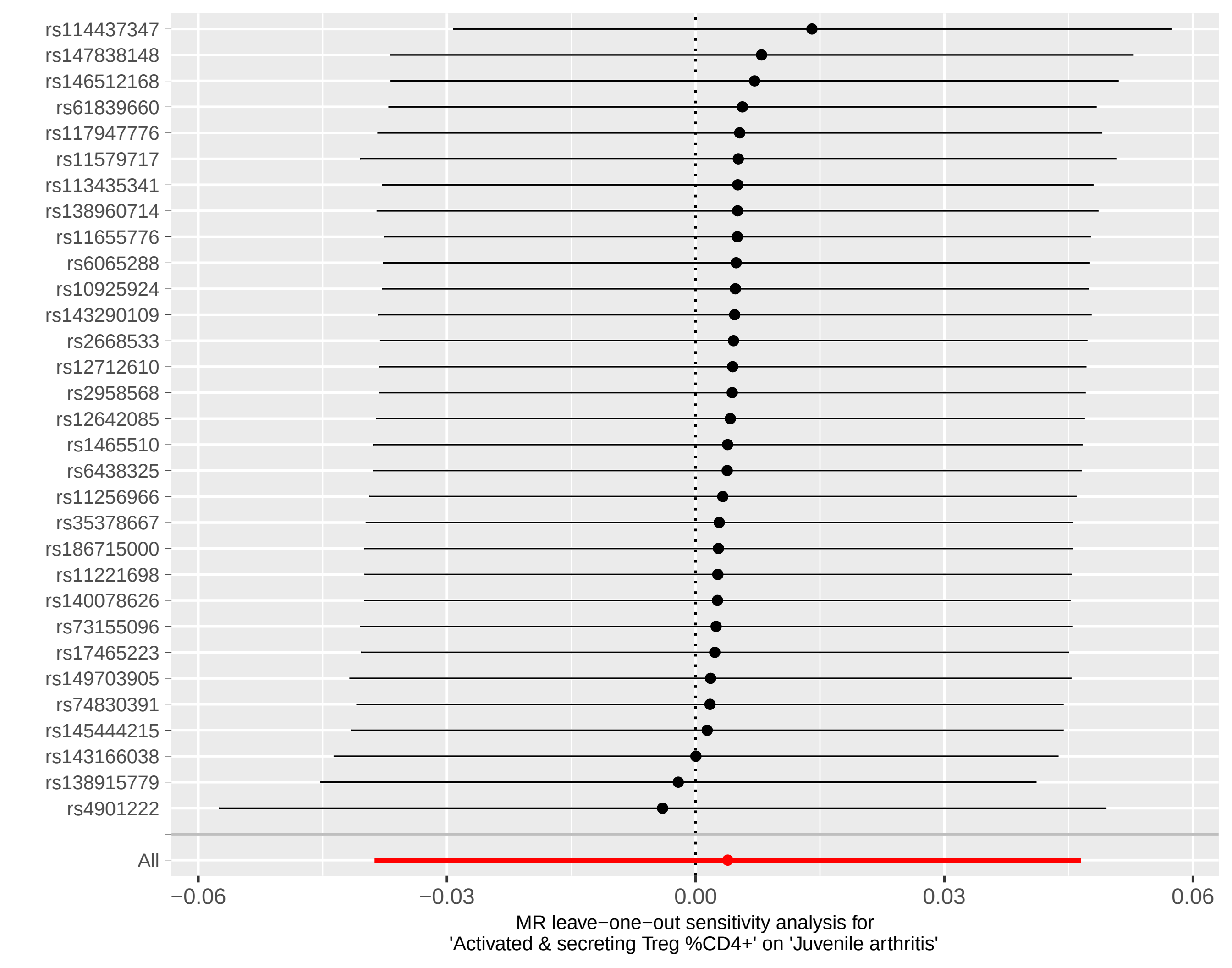
rs114437347
rs147838148
rs146512168
rs61839660
rs117947776
rs11579717
rs113435341
rs138960714
rs11655776
rs6065288
rs10925924
rs143290109
rs2668533
rs12712610
rs2958568
rs12642085
rs1465510
rs6438325
rs11256966
rs35378667
rs186715000
rs11221698
rs140078626
rs73155096
rs17465223
rs149703905
rs74830391
rs145444215
rs143166038
rs138915779
rs4901222
All
−0.06
−0.03
0.00
0.03
0.06
MR leave−one−out sensitivity analysis for
'Activated & secreting Treg %CD4+' on 'Juvenile arthritis'

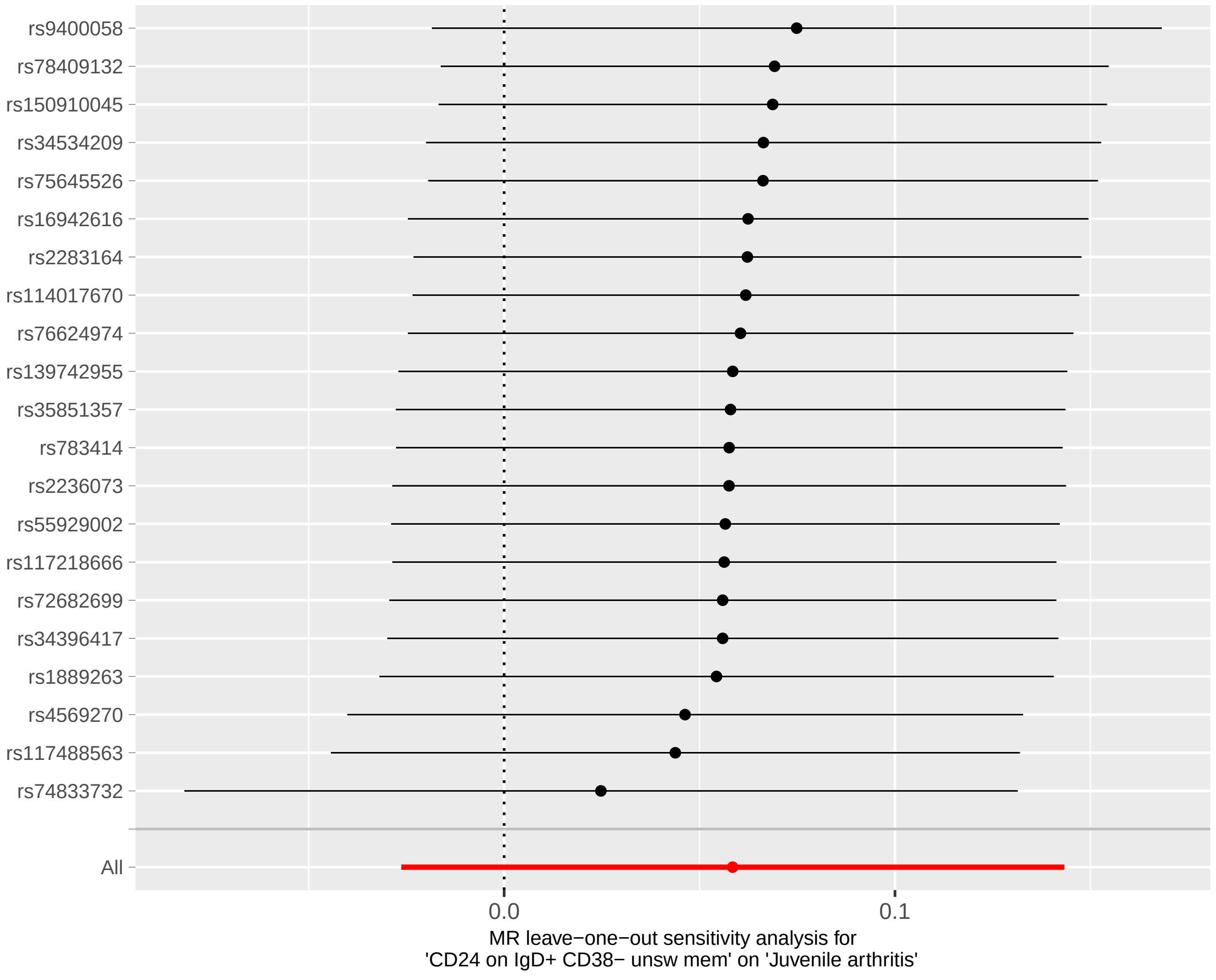

rs9400058
rs78409132
rs150910045
rs34534209
rs75645526
rs16942616
rs2283164
rs114017670
rs76624974
rs139742955
rs35851357
rs783414
rs2236073
rs55929002
rs117218666
rs72682699
rs34396417
rs1889263
rs4569270
rs117488563
rs74833732
All
0.0
0.1
MR leave−one−out sensitivity analysis for
'CD24 on IgD+ CD38− unsw mem' on 'Juvenile arthritis'

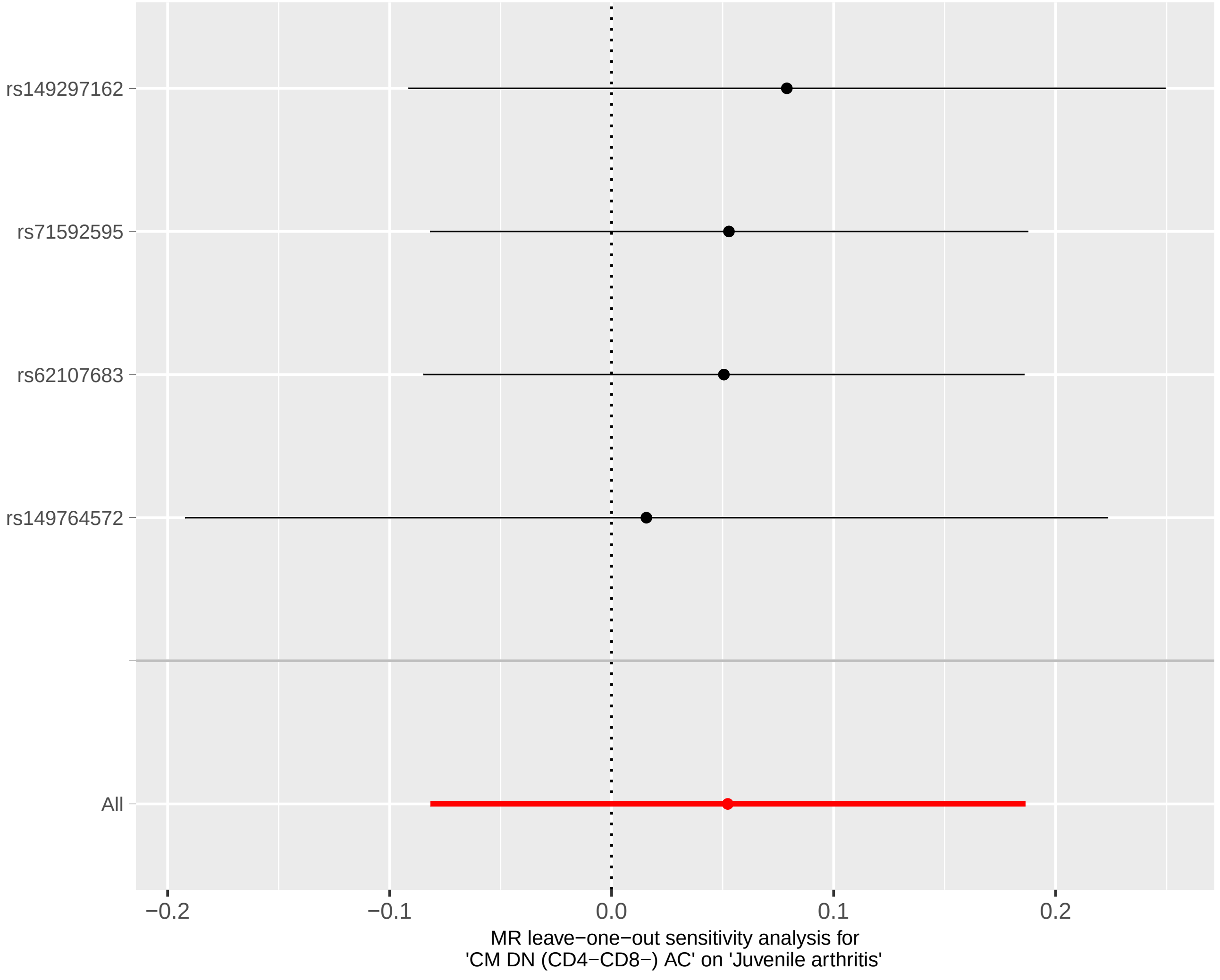

rs149297162
rs71592595
rs62107683
rs149764572
All
−0.2
−0.1
0.0
0.1
0.2
MR leave−one−out sensitivity analysis for
'CM DN (CD4−CD8−) AC' on 'Juvenile arthritis'

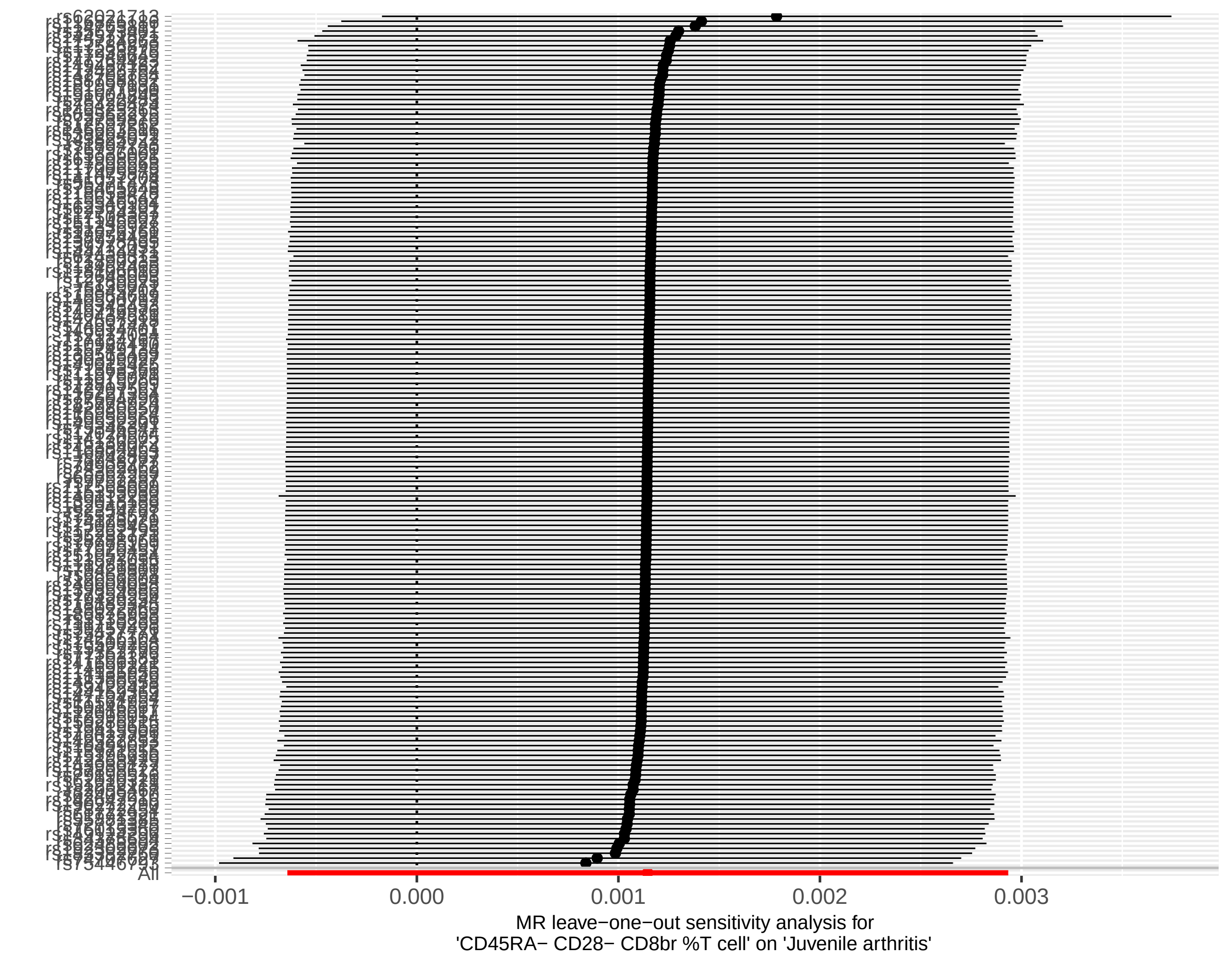

rs62021712
rs75446703
All
−0.001
0.000
0.001
0.002
0.003
MR leave−one−out sensitivity analysis for
'CD45RA− CD28− CD8br %T cell' on 'Juvenile arthritis'

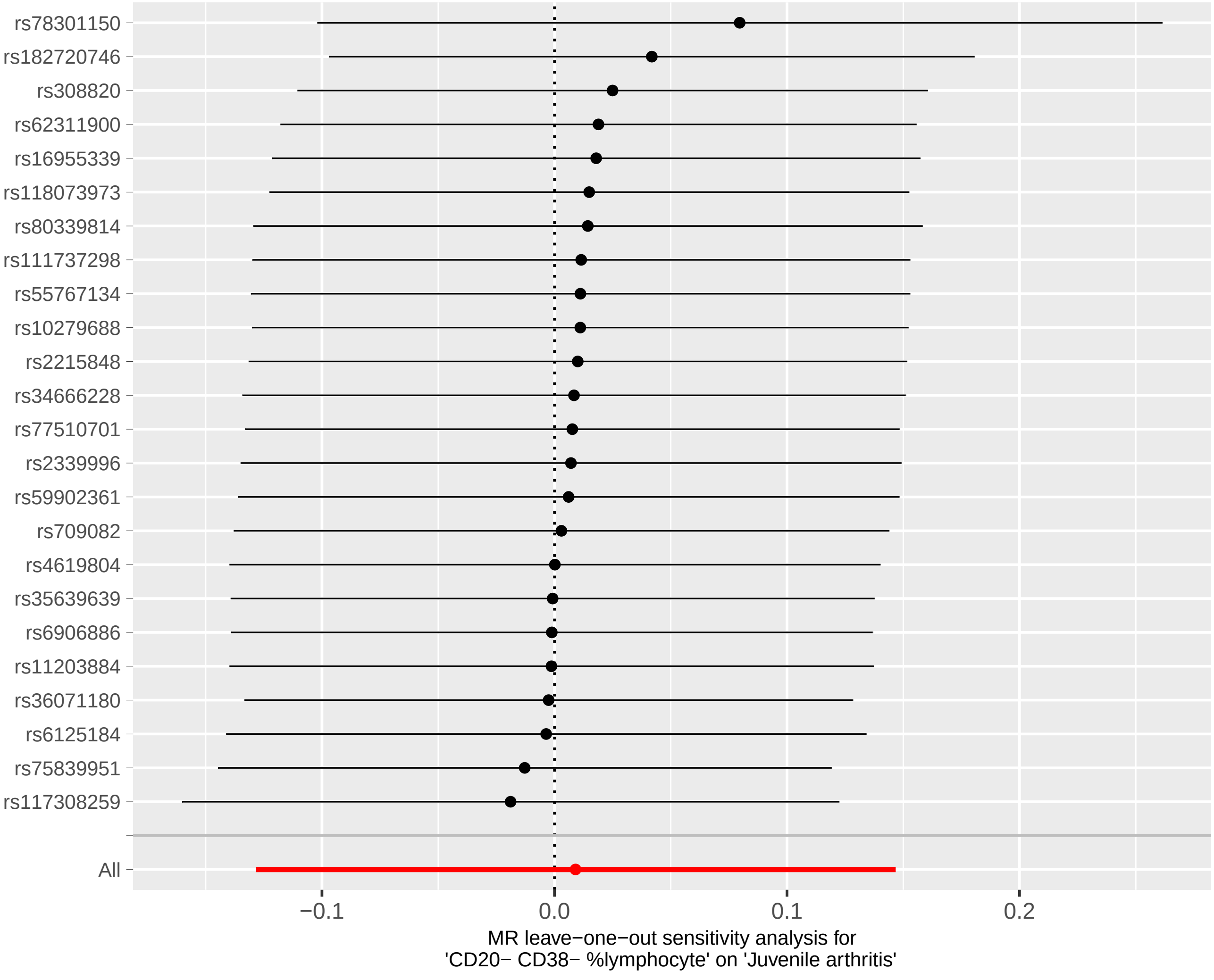

rs78301150
rs182720746
rs308820
rs62311900
rs16955339
rs118073973
rs80339814
rs111737298
rs55767134
rs10279688
rs2215848
rs34666228
rs77510701
rs2339996
rs59902361
rs709082
rs4619804
rs35639639
rs6906886
rs11203884
rs36071180
rs6125184
rs75839951
rs117308259
All
−0.1
0.0
0.1
0.2
MR leave−one−out sensitivity analysis for
'CD20− CD38− %lymphocyte' on 'Juvenile arthritis'

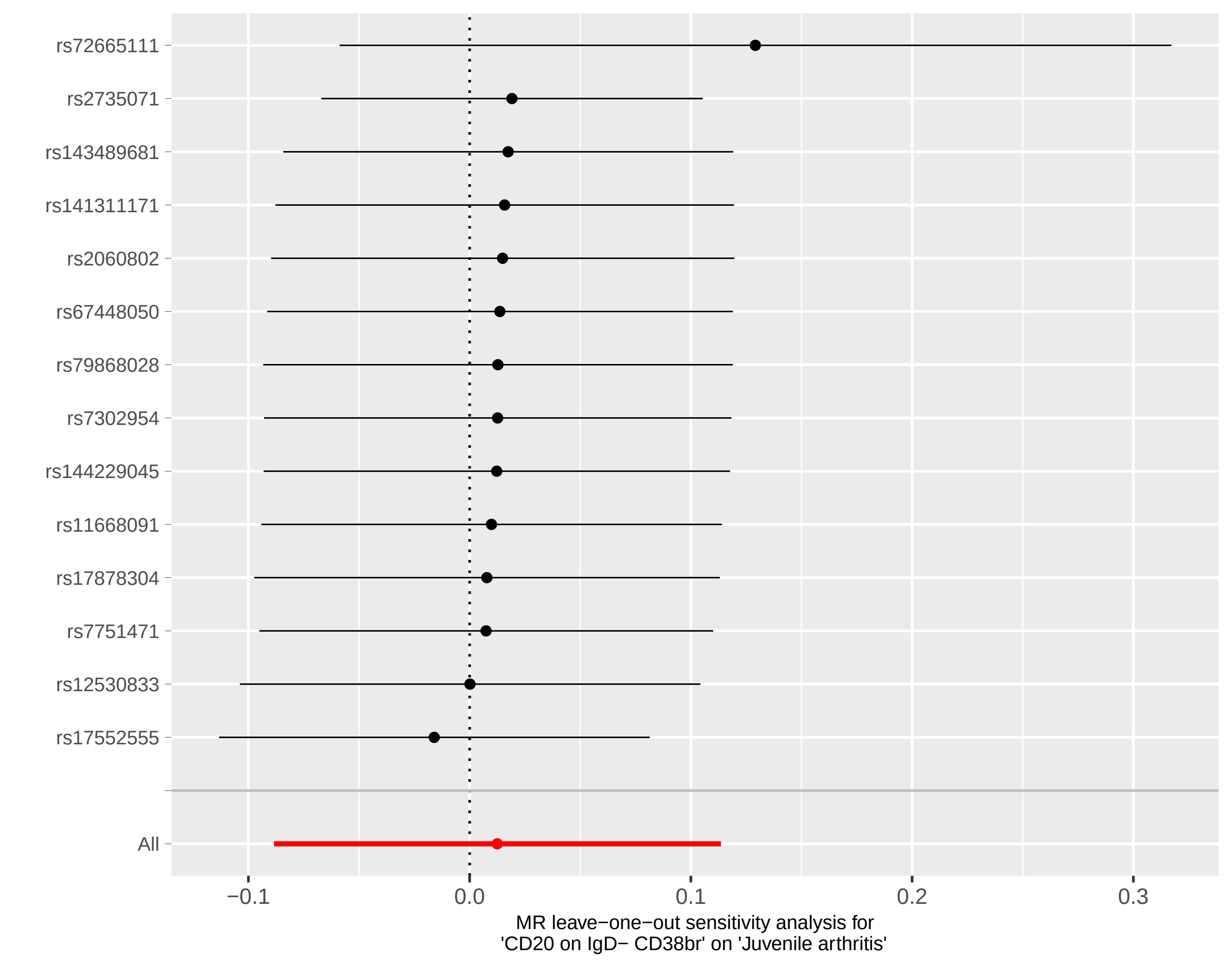
rs72665111
rs2735071
rs143489681
rs141311171
rs2060802
rs67448050
rs79868028
rs7302954
rs144229045
rs11668091
rs17878304
rs7751471
rs12530833
rs17552555
All
−0.1
0.0
0.1
0.2
0.3
MR leave−one−out sensitivity analysis for
'CD20 on IgD− CD38br' on 'Juvenile arthritis'

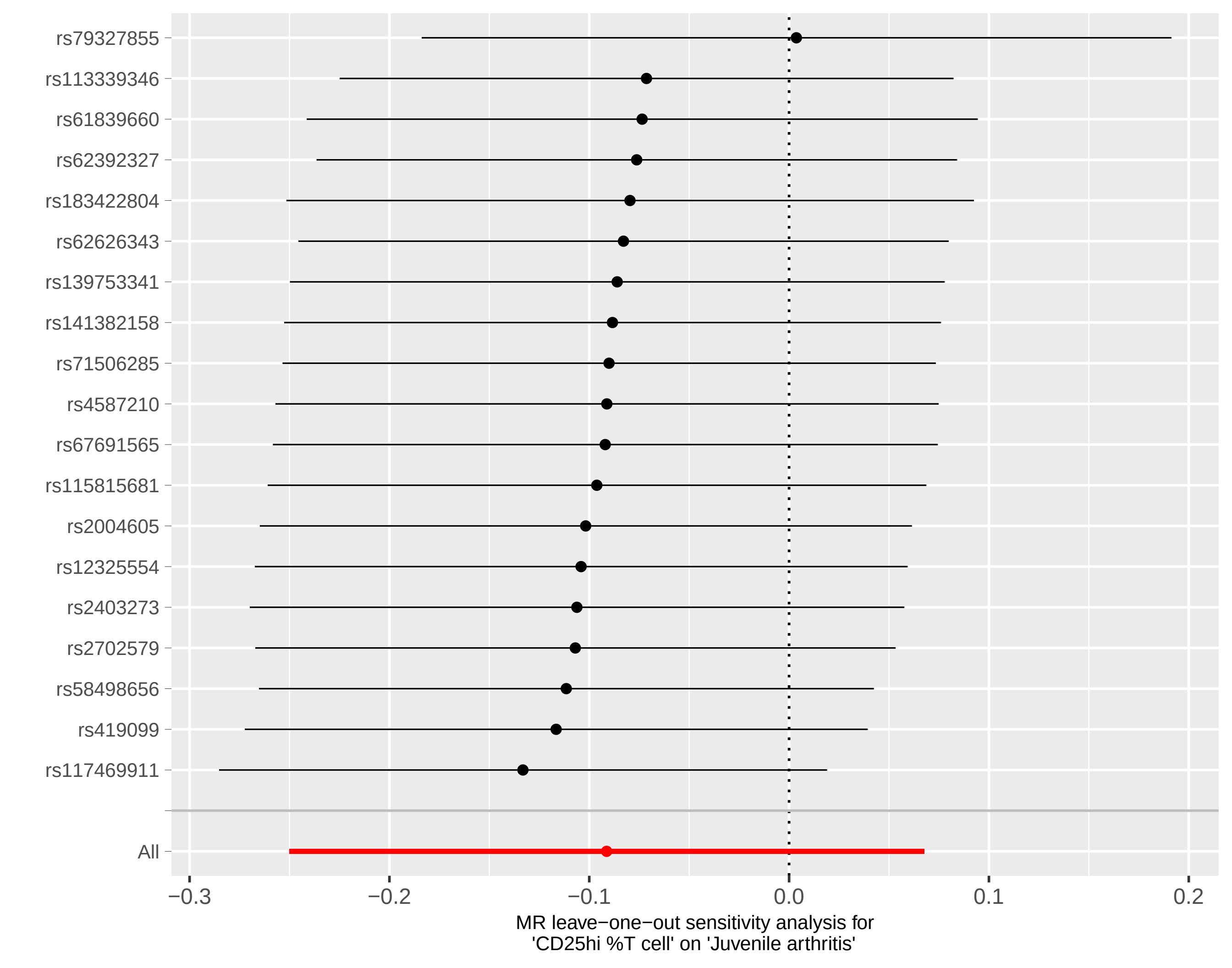
rs79327855
rs113339346
rs61839660
rs62392327
rs183422804
rs62626343
rs139753341
rs141382158
rs71506285
rs4587210
rs67691565
rs115815681
rs2004605
rs12325554
rs2403273
rs2702579
rs58498656
rs419099
rs117469911
All
−0.3
−0.2
−0.1
0.0
0.1
0.2
MR leave−one−out sensitivity analysis for
'CD25hi %T cell' on 'Juvenile arthritis'

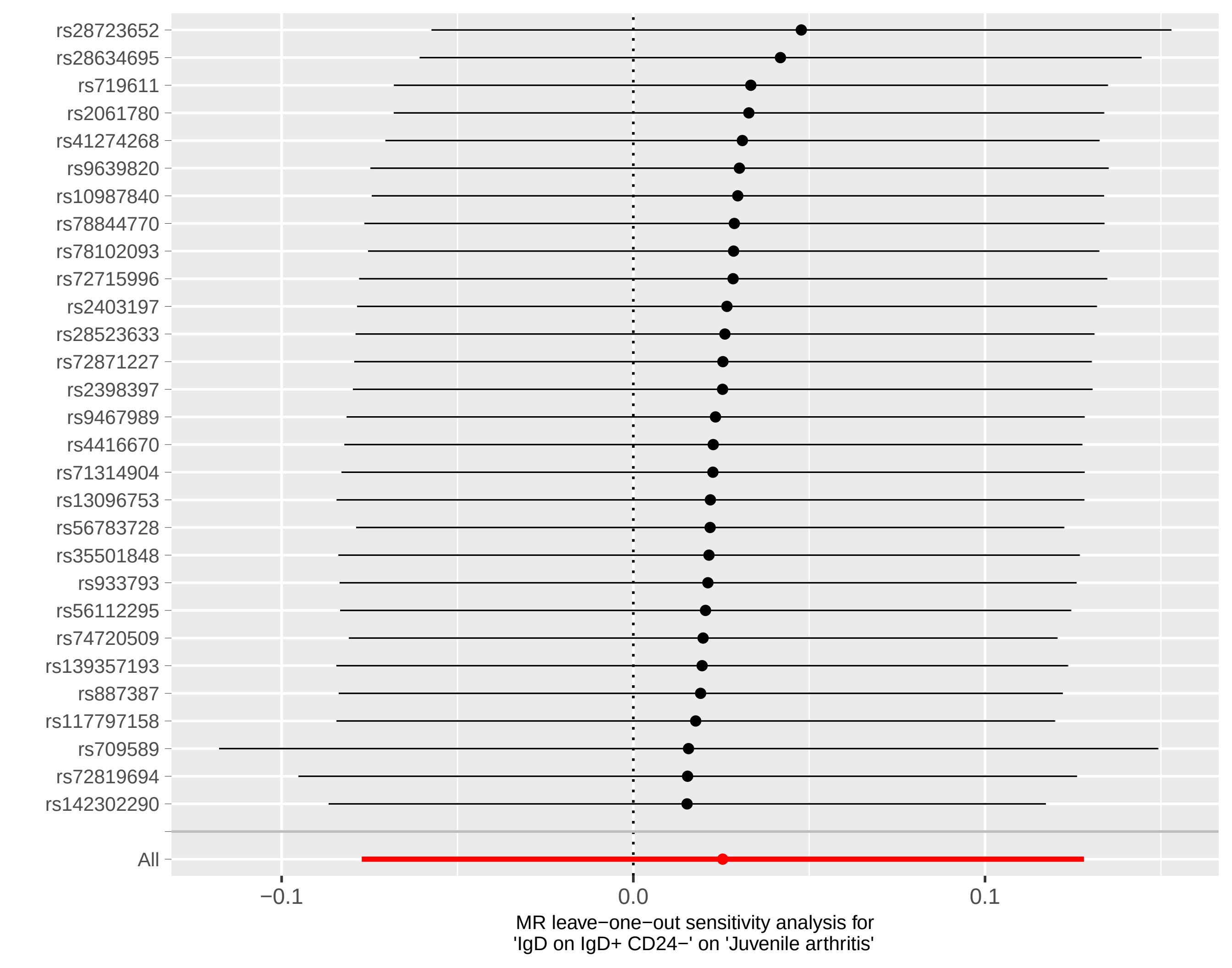
rs28723652
rs28634695
rs719611
rs2061780
rs41274268
rs9639820
rs10987840
rs78844770
rs78102093
rs72715996
rs2403197
rs28523633
rs72871227
rs2398397
rs9467989
rs4416670
rs71314904
rs13096753
rs56783728
rs35501848
rs933793
rs56112295
rs74720509
rs139357193
rs887387
rs117797158
rs709589
rs72819694
rs142302290
All
−0.1
0.0
0.1
MR leave−one−out sensitivity analysis for
'IgD on IgD+ CD24−' on 'Juvenile arthritis'

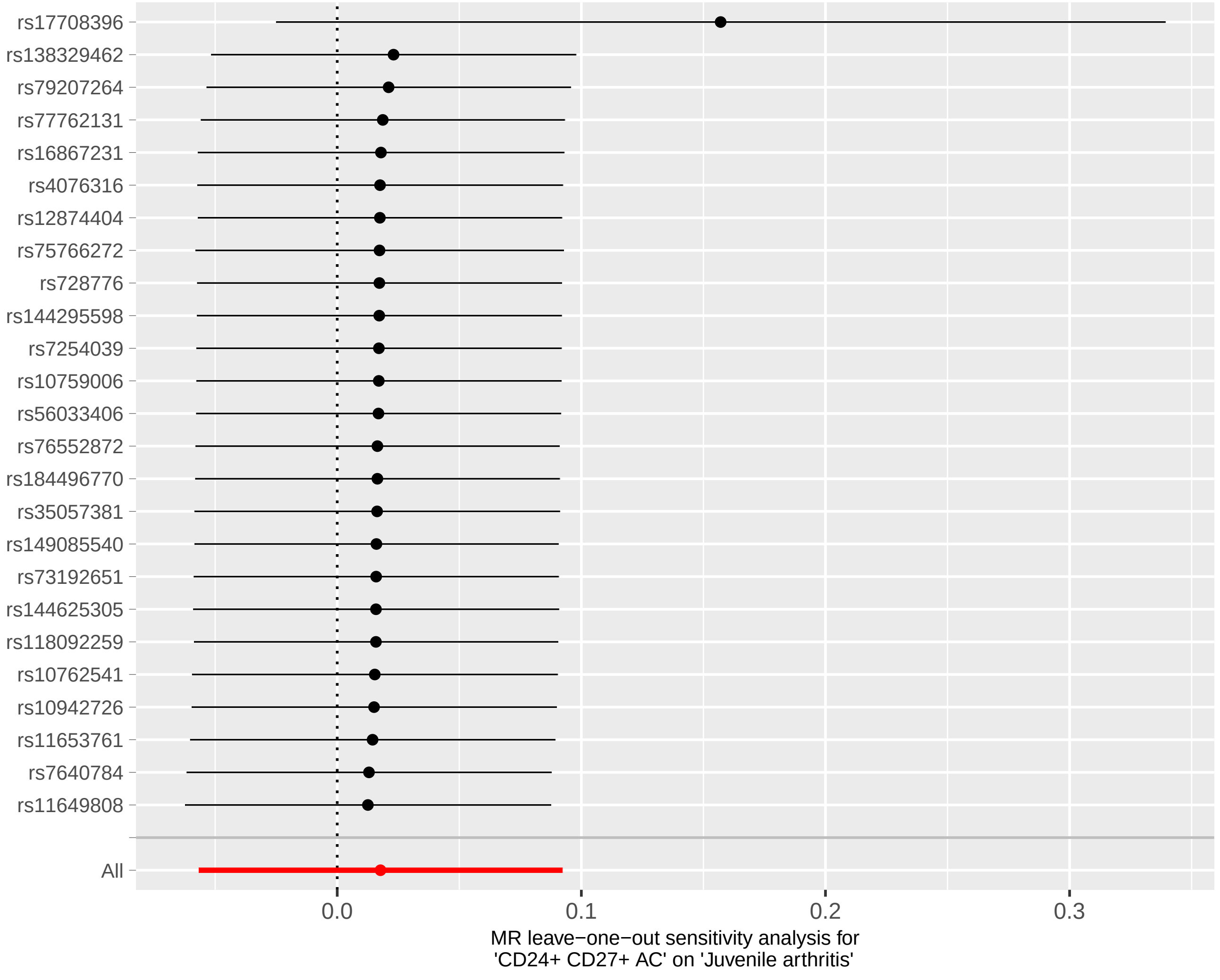
rs17708396
rs138329462
rs79207264
rs77762131
rs16867231
rs4076316
rs12874404
rs75766272
rs728776
rs144295598
rs7254039
rs10759006
rs56033406
rs76552872
rs184496770
rs35057381
rs149085540
rs73192651
rs144625305
rs118092259
rs10762541
rs10942726
rs11653761
rs7640784
rs11649808
All
0.0
0.1
0.2
0.3
MR leave−one−out sensitivity analysis for
'CD24+ CD27+ AC' on 'Juvenile arthritis'

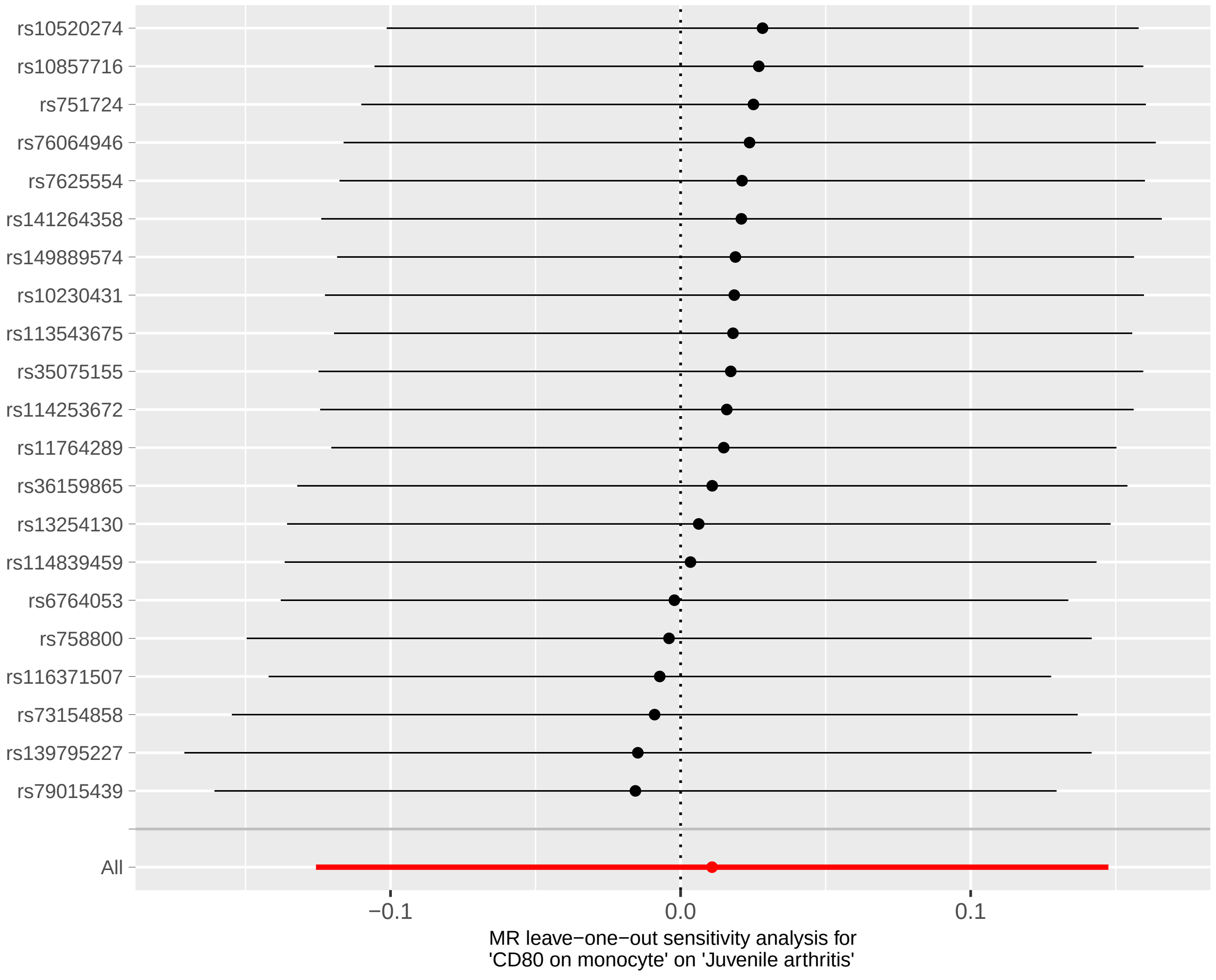

rs10520274
rs10857716
rs751724
rs76064946
rs7625554
rs141264358
rs149889574
rs10230431
rs113543675
rs35075155
rs114253672
rs11764289
rs36159865
rs13254130
rs114839459
rs6764053
rs758800
rs116371507
rs73154858
rs139795227
rs79015439
All
−0.1
0.0
0.1
MR leave−one−out sensitivity analysis for
'CD80 on monocyte' on 'Juvenile arthritis'

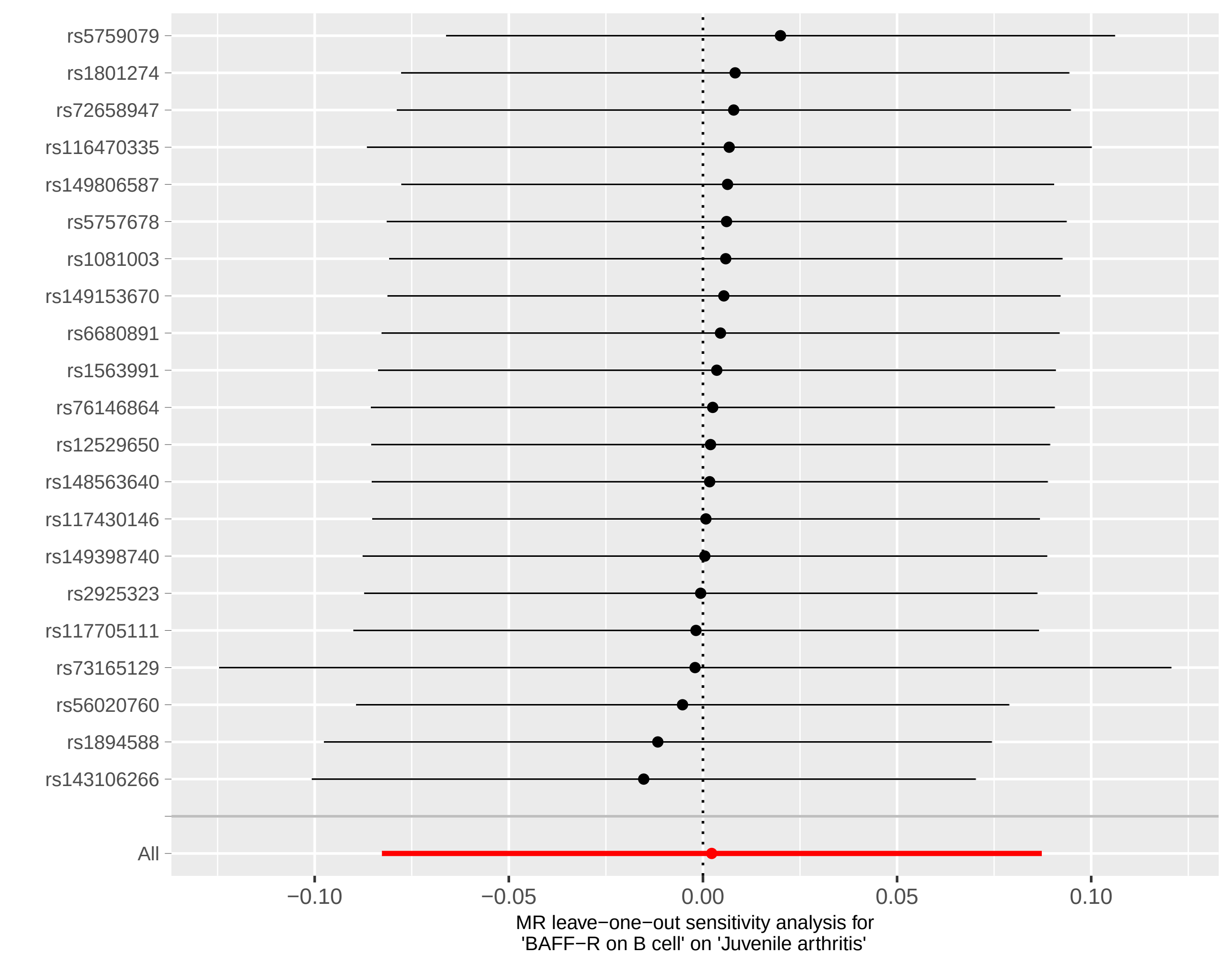
rs5759079
rs1801274
rs72658947
rs116470335
rs149806587
rs5757678
rs1081003
rs149153670
rs6680891
rs1563991
rs76146864
rs12529650
rs148563640
rs117430146
rs149398740
rs2925323
rs117705111
rs73165129
rs56020760
rs1894588
rs143106266
All
−0.10
−0.05
0.00
0.05
0.10
MR leave−one−out sensitivity analysis for
'BAFF−R on B cell' on 'Juvenile arthritis'

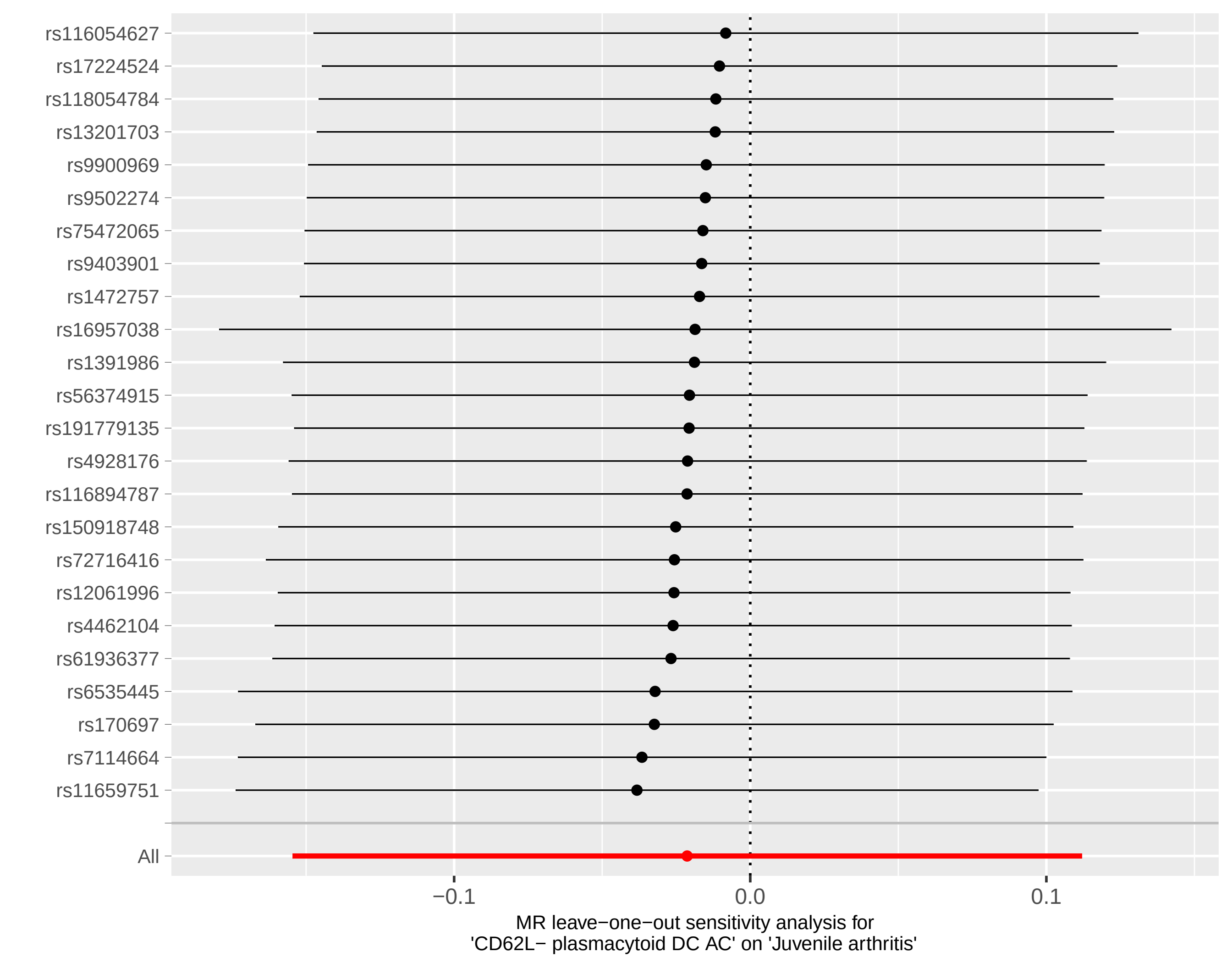
rs116054627
rs17224524
rs118054784
rs13201703
rs9900969
rs9502274
rs75472065
rs9403901
rs1472757
rs16957038
rs1391986
rs56374915
rs191779135
rs4928176
rs116894787
rs150918748
rs72716416
rs12061996
rs4462104
rs61936377
rs6535445
rs170697
rs7114664
rs11659751
All
−0.1
0.0
0.1
MR leave−one−out sensitivity analysis for
'CD62L− plasmacytoid DC AC' on 'Juvenile arthritis'

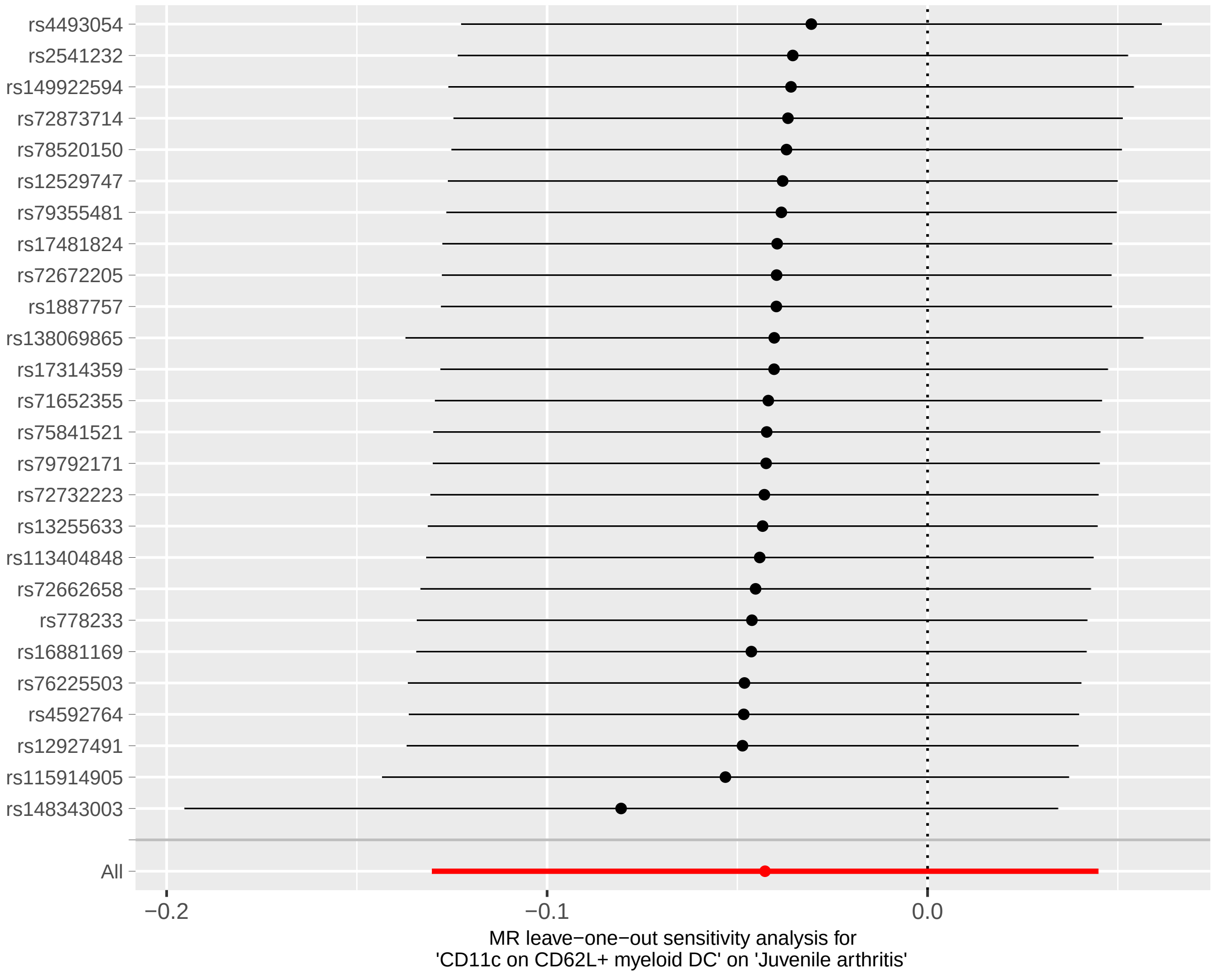

rs4493054
rs2541232
rs149922594
rs72873714
rs78520150
rs12529747
rs79355481
rs17481824
rs72672205
rs1887757
rs138069865
rs17314359
rs71652355
rs75841521
rs79792171
rs72732223
rs13255633
rs113404848
rs72662658
rs778233
rs16881169
rs76225503
rs4592764
rs12927491
rs115914905
rs148343003
All
−0.2
−0.1
0.0
MR leave−one−out sensitivity analysis for
'CD11c on CD62L+ myeloid DC' on 'Juvenile arthritis'

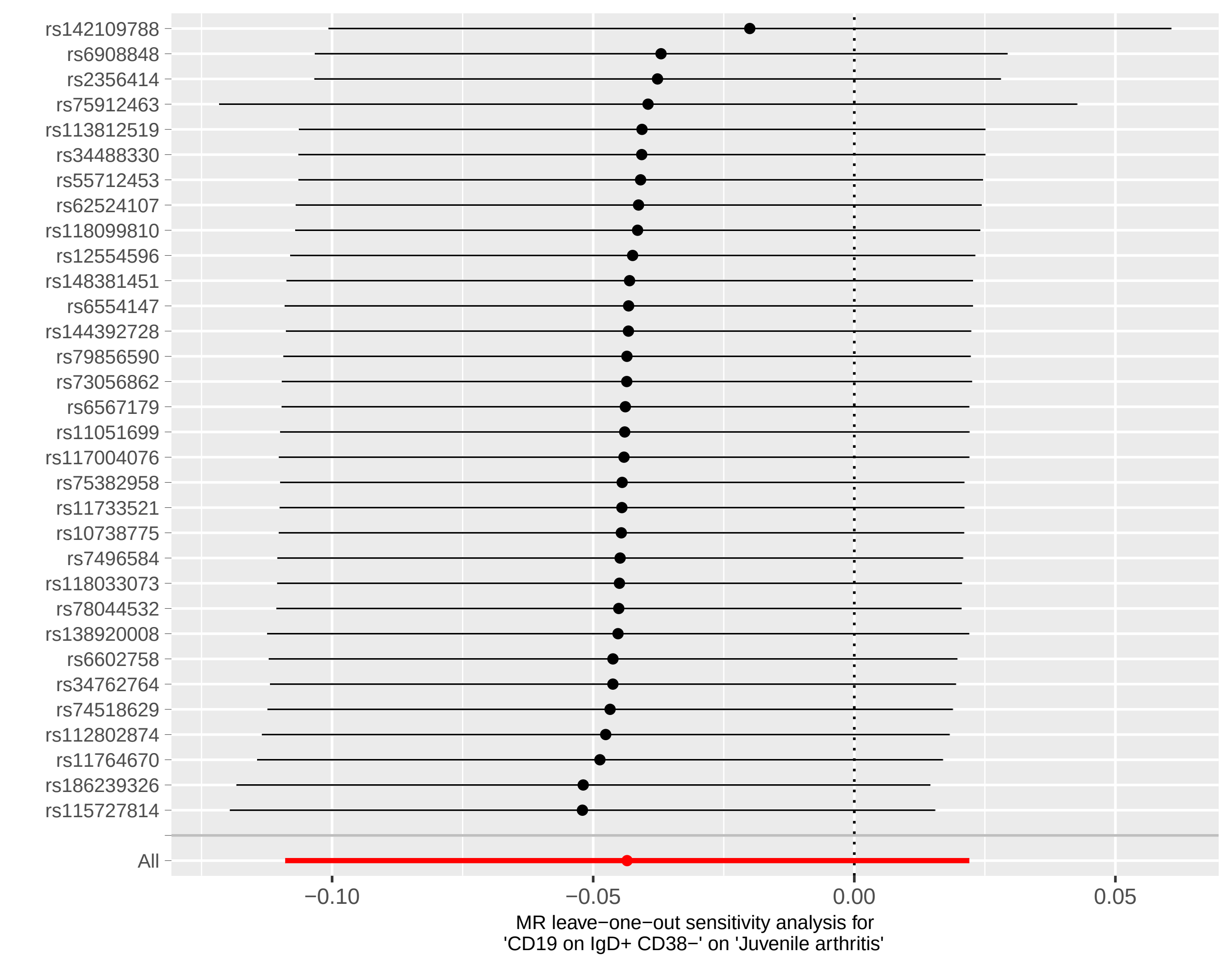
rs142109788
rs6908848
rs2356414
rs75912463
rs113812519
rs34488330
rs55712453
rs62524107
rs118099810
rs12554596
rs148381451
rs6554147
rs144392728
rs79856590
rs73056862
rs6567179
rs11051699
rs117004076
rs75382958
rs11733521
rs10738775
rs7496584
rs118033073
rs78044532
rs138920008
rs6602758
rs34762764
rs74518629
rs112802874
rs11764670
rs186239326
rs115727814
All
−0.10
−0.05
0.00
0.05
MR leave−one−out sensitivity analysis for
'CD19 on IgD+ CD38−' on 'Juvenile arthritis'

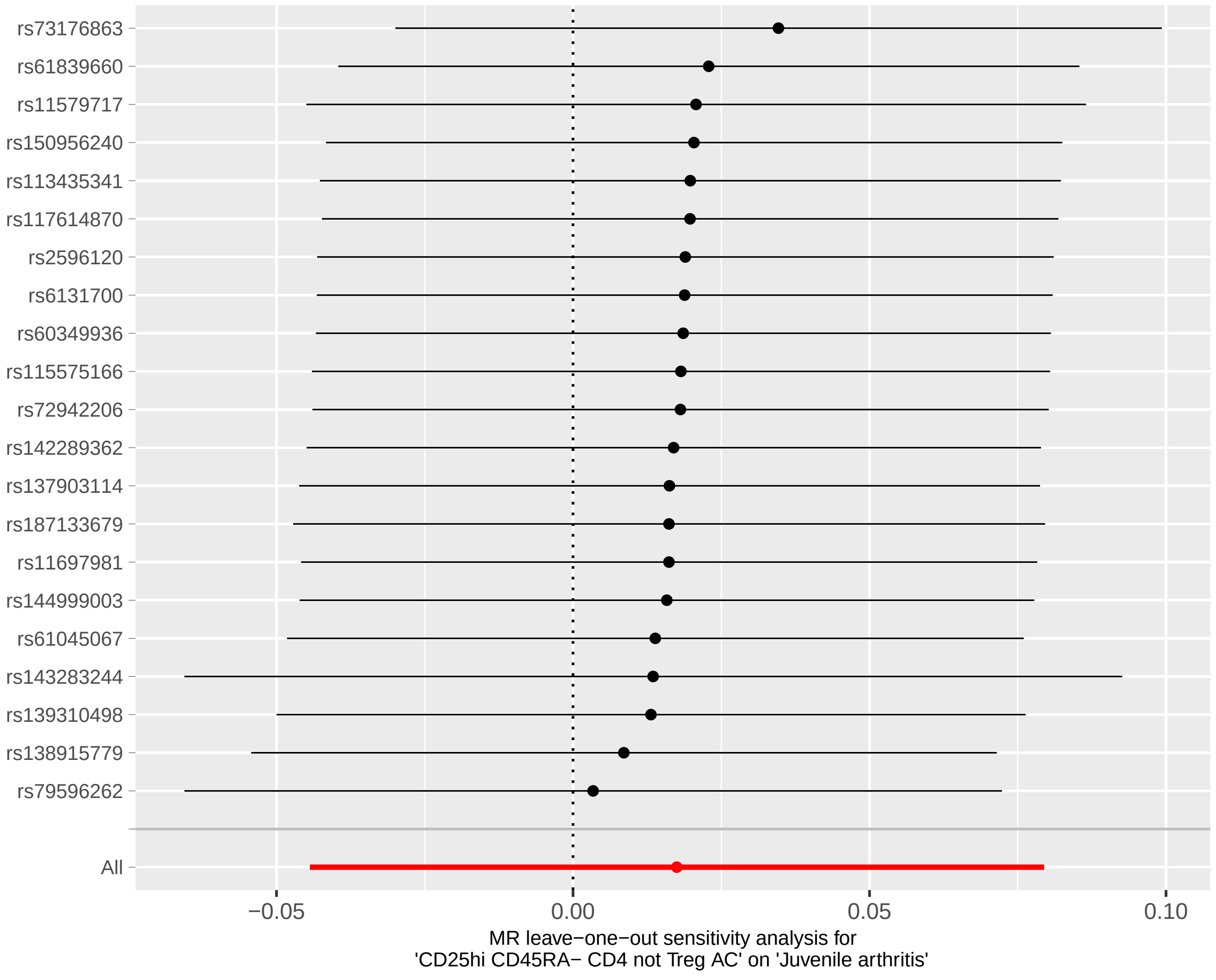
rs73176863
rs61839660
rs11579717
rs150956240
rs113435341
rs117614870
rs2596120
rs6131700
rs60349936
rs115575166
rs72942206
rs142289362
rs137903114
rs187133679
rs11697981
rs144999003
rs61045067
rs143283244
rs139310498
rs138915779
rs79596262
All
−0.05
0.00
0.05
0.10
MR leave−one−out sensitivity analysis for
'CD25hi CD45RA− CD4 not Treg AC' on 'Juvenile arthritis'

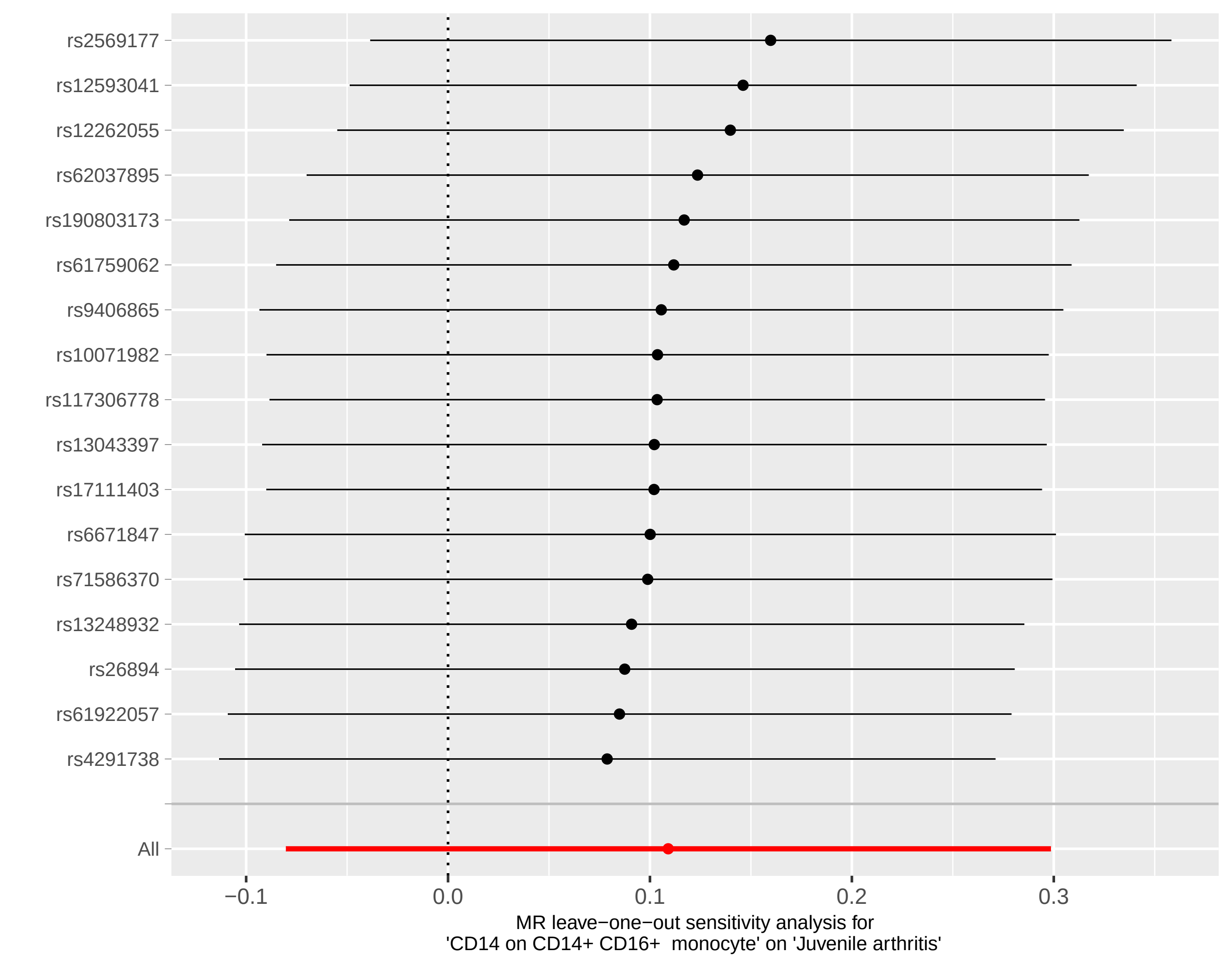
rs2569177
rs12593041
rs12262055
rs62037895
rs190803173
rs61759062
rs9406865
rs10071982
rs117306778
rs13043397
rs17111403
rs6671847
rs71586370
rs13248932
rs26894
rs61922057
rs4291738
All
−0.1
0.0
0.1
0.2
0.3
MR leave−one−out sensitivity analysis for
'CD14 on CD14+ CD16+ monocyte' on 'Juvenile arthritis'

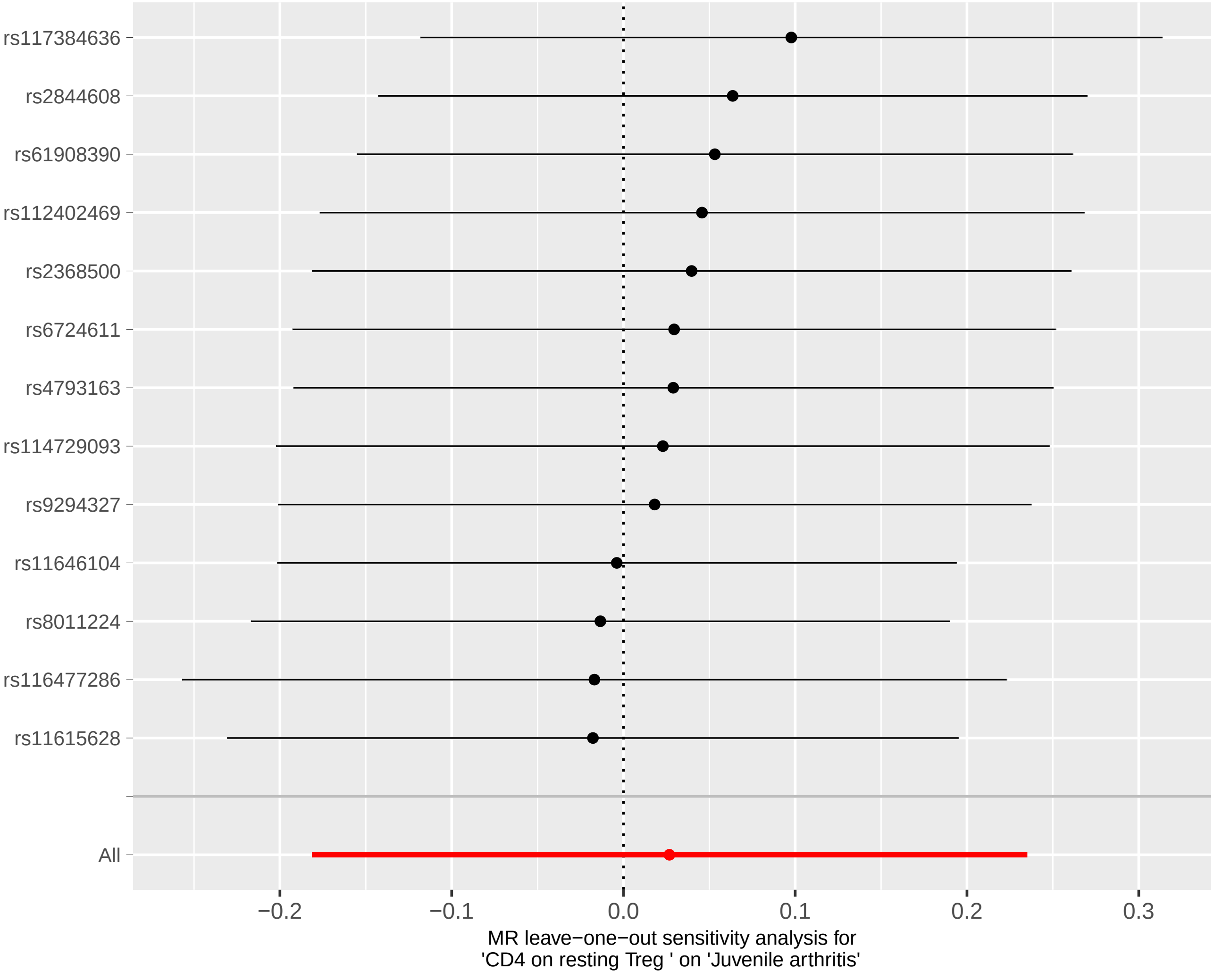
rs117384636
rs2844608
rs61908390
rs112402469
rs2368500
rs6724611
rs4793163
rs114729093
rs9294327
rs11646104
rs8011224
rs116477286
rs11615628
All
−0.2
−0.1
0.0
0.1
0.2
0.3
MR leave−one−out sensitivity analysis for
'CD4 on resting Treg ' on 'Juvenile arthritis'

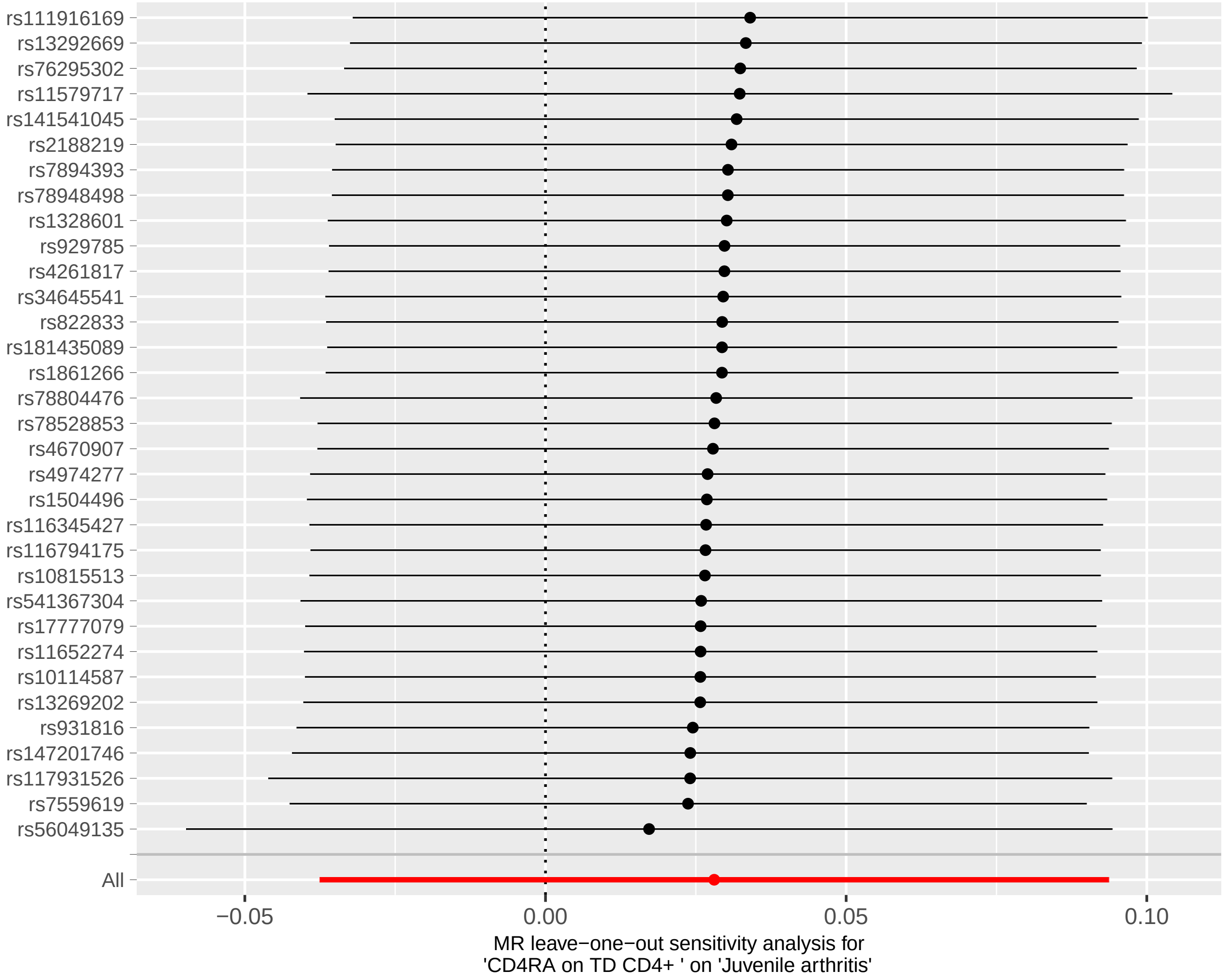
rs111916169
rs13292669
rs76295302
rs11579717
rs141541045
rs2188219
rs7894393
rs78948498
rs1328601
rs929785
rs4261817
rs34645541
rs822833
rs181435089
rs1861266
rs78804476
rs78528853
rs4670907
rs4974277
rs1504496
rs116345427
rs116794175
rs10815513
rs541367304
rs17777079
rs11652274
rs10114587
rs13269202
rs931816
rs147201746
rs117931526
rs7559619
rs56049135
All
−0.05
0.00
0.05
0.10
MR leave−one−out sensitivity analysis for
'CD4RA on TD CD4+ ' on 'Juvenile arthritis'

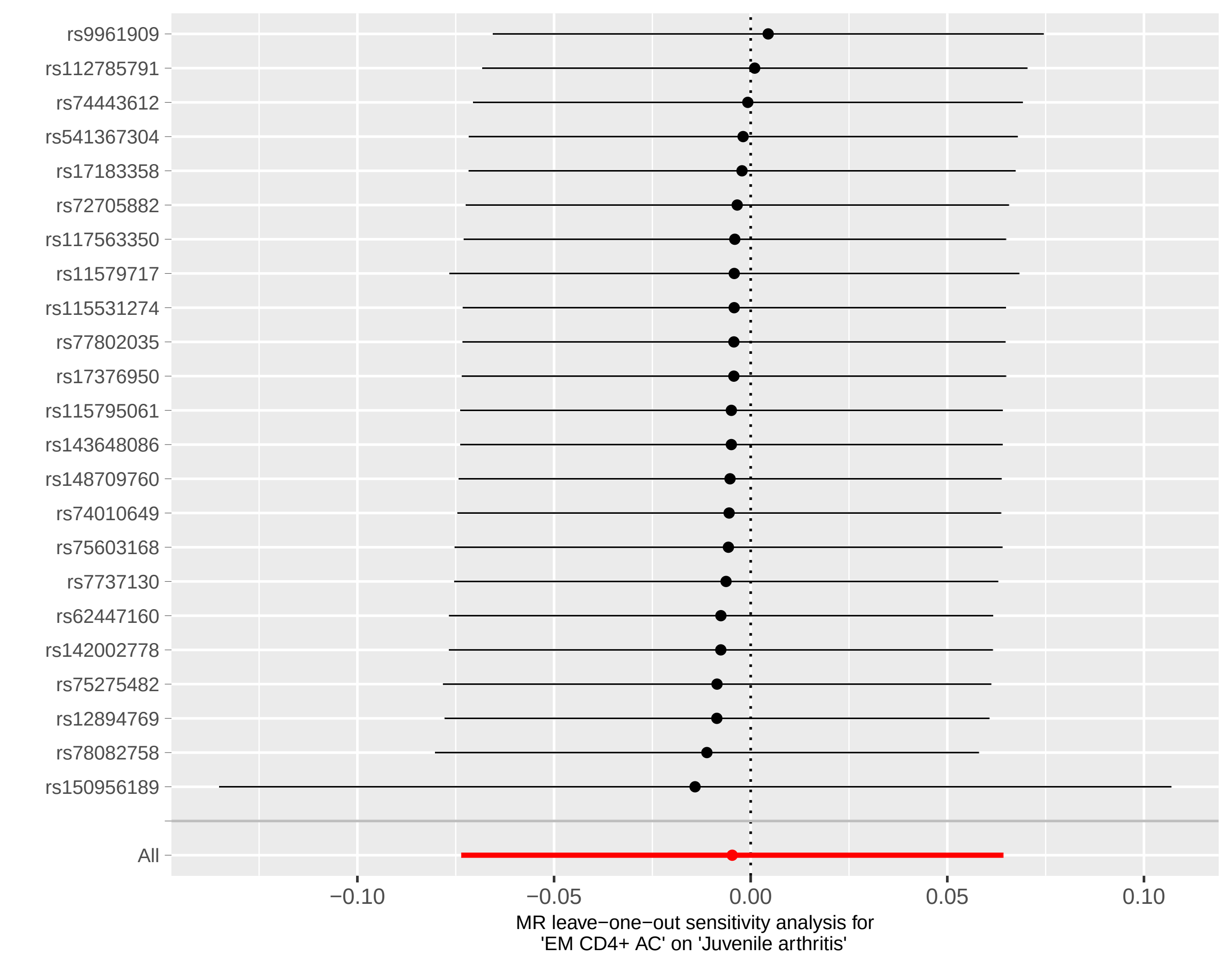
rs9961909
rs112785791
rs74443612
rs541367304
rs17183358
rs72705882
rs117563350
rs11579717
rs115531274
rs77802035
rs17376950
rs115795061
rs143648086
rs148709760
rs74010649
rs75603168
rs7737130
rs62447160
rs142002778
rs75275482
rs12894769
rs78082758
rs150956189
All
−0.10
−0.05
0.00
0.05
0.10
MR leave−one−out sensitivity analysis for
'EM CD4+ AC' on 'Juvenile arthritis'

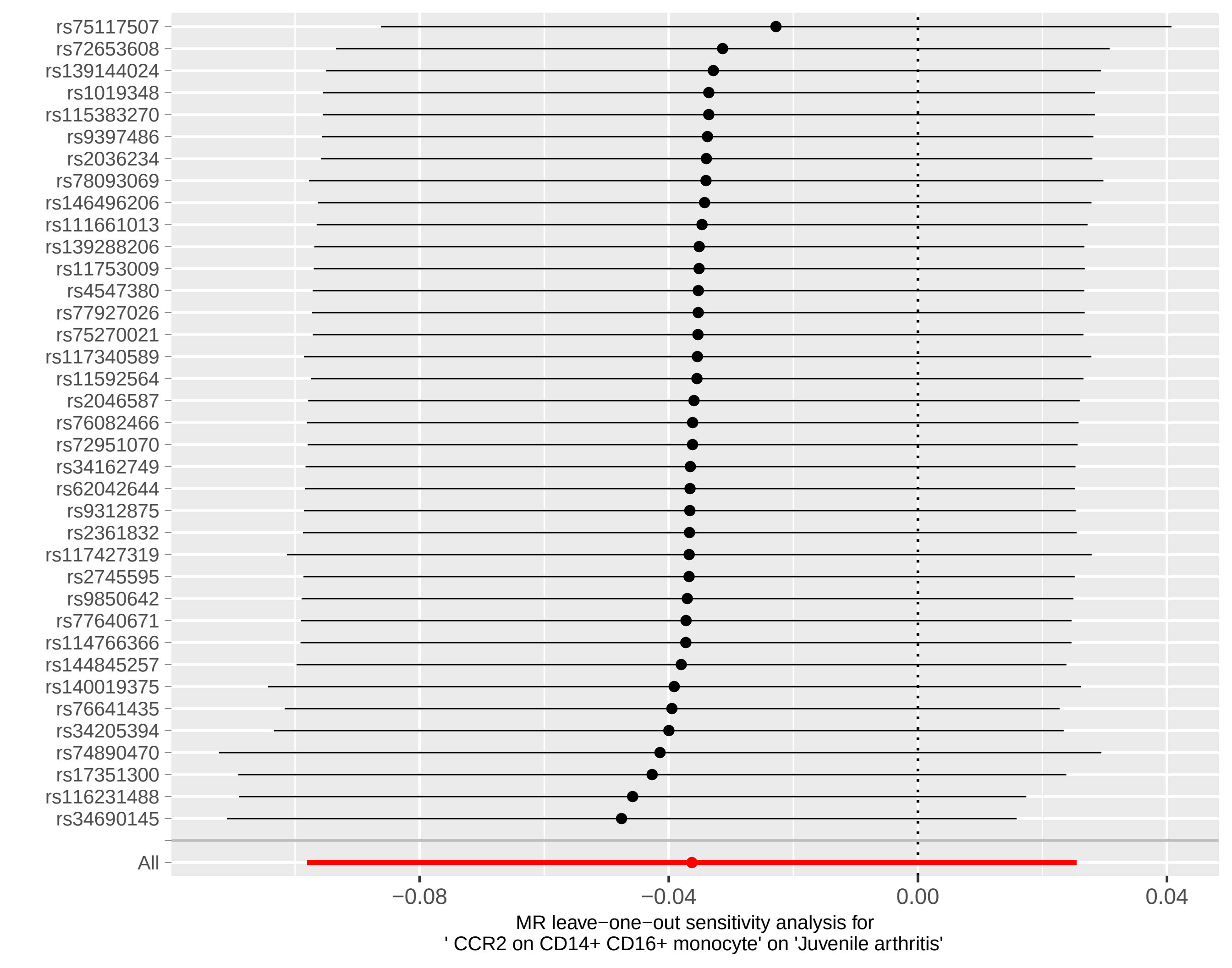
rs75117507
rs72653608
rs139144024
rs1019348
rs115383270
rs9397486
rs2036234
rs78093069
rs146496206
rs111661013
rs139288206
rs11753009
rs4547380
rs77927026
rs75270021
rs117340589
rs11592564
rs2046587
rs76082466
rs72951070
rs34162749
rs62042644
rs9312875
rs2361832
rs117427319
rs2745595
rs9850642
rs77640671
rs114766366
rs144845257
rs140019375
rs76641435
rs34205394
rs74890470
rs17351300
rs116231488
rs34690145
All
−0.08
−0.04
0.00
0.04
MR leave−one−out sensitivity analysis for
' CCR2 on CD14+ CD16+ monocyte' on 'Juvenile arthritis'

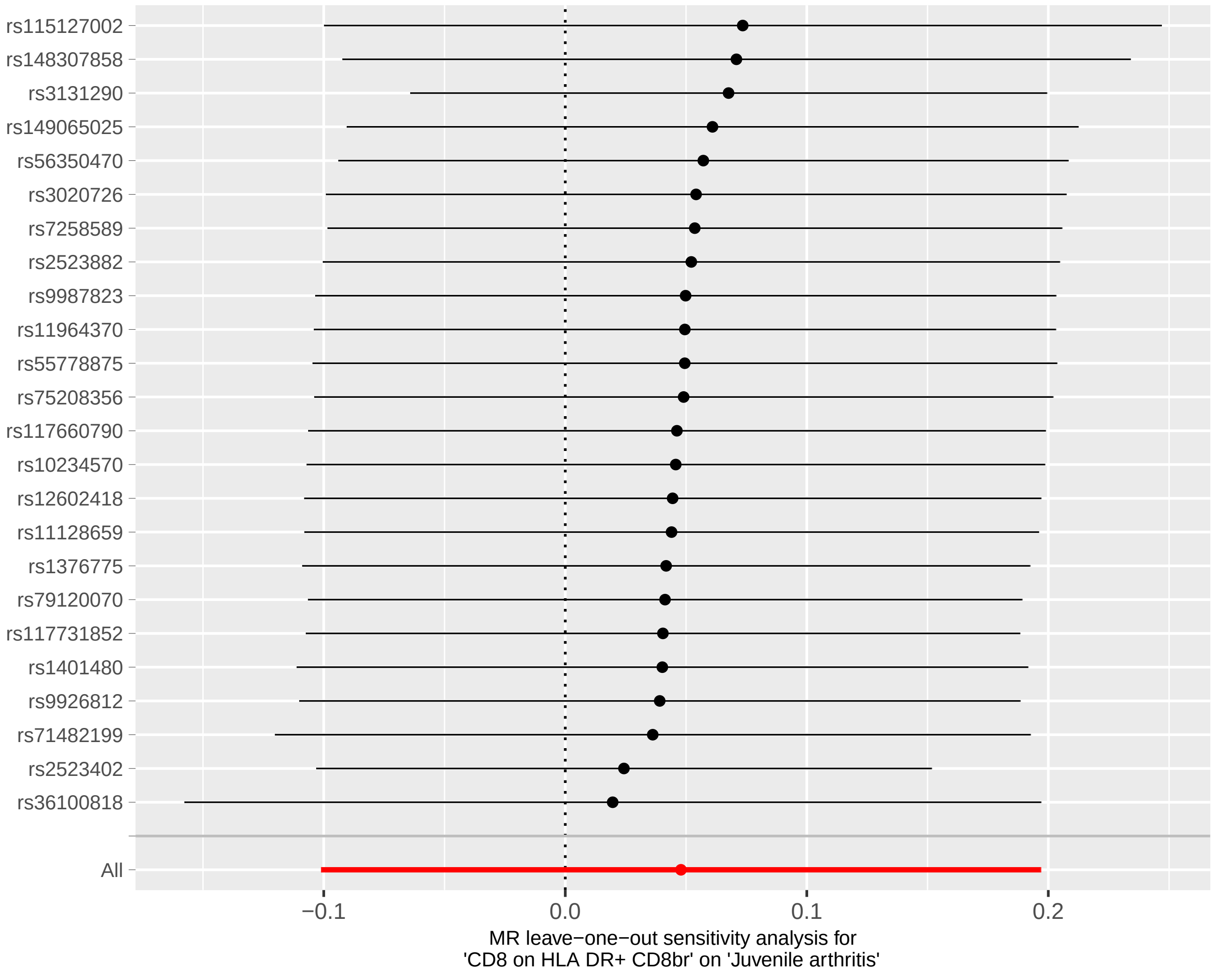
rs115127002
rs148307858
rs3131290
rs149065025
rs56350470
rs3020726
rs7258589
rs2523882
rs9987823
rs11964370
rs55778875
rs75208356
rs117660790
rs10234570
rs12602418
rs11128659
rs1376775
rs79120070
rs117731852
rs1401480
rs9926812
rs71482199
rs2523402
rs36100818
All
−0.1
0.0
0.1
0.2
MR leave−one−out sensitivity analysis for
'CD8 on HLA DR+ CD8br' on 'Juvenile arthritis'

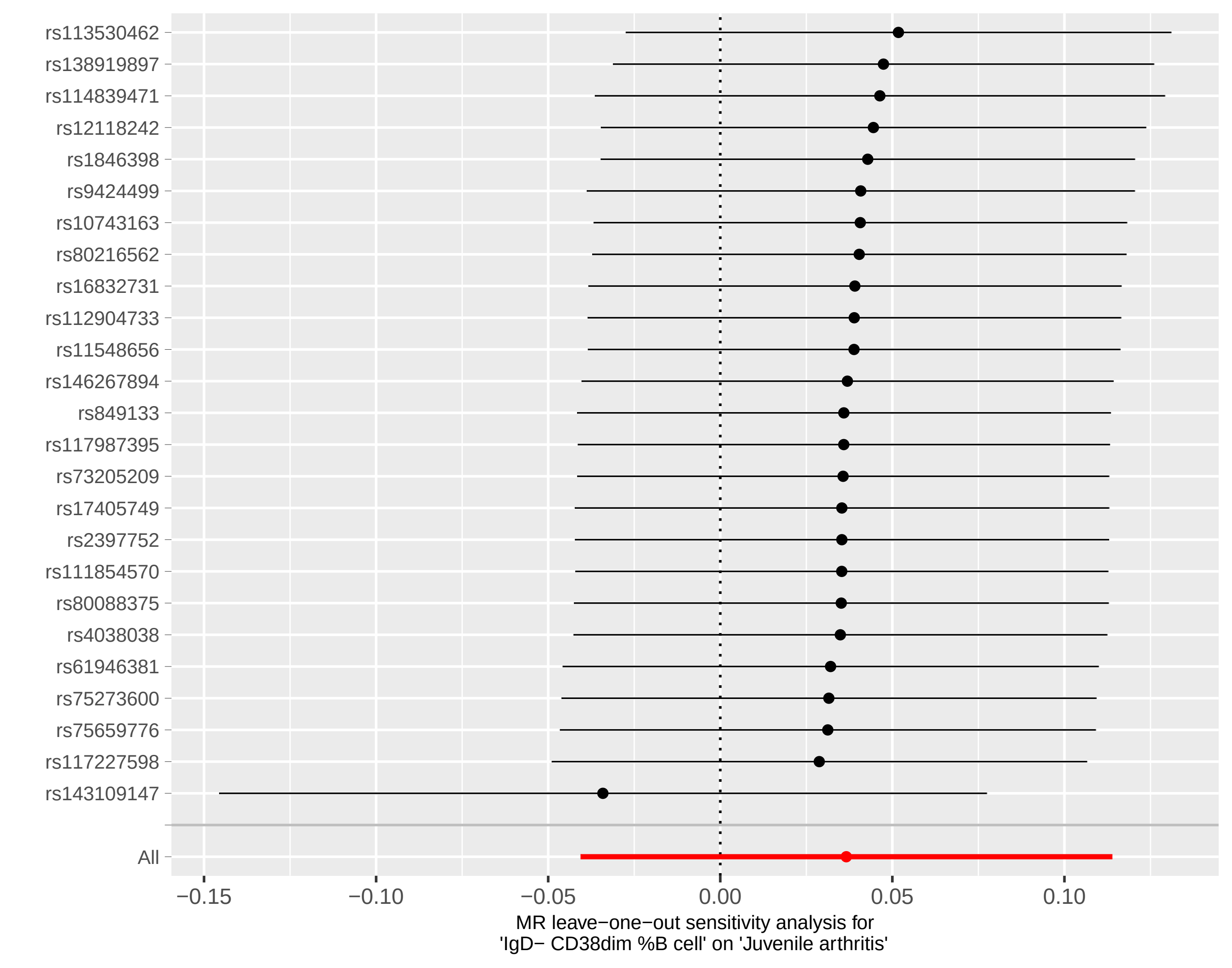
rs113530462
rs138919897
rs114839471
rs12118242
rs1846398
rs9424499
rs10743163
rs80216562
rs16832731
rs112904733
rs11548656
rs146267894
rs849133
rs117987395
rs73205209
rs17405749
rs2397752
rs111854570
rs80088375
rs4038038
rs61946381
rs75273600
rs75659776
rs117227598
rs143109147
All
−0.15
−0.10
−0.05
0.00
0.05
0.10
MR leave−one−out sensitivity analysis for
'IgD− CD38dim %B cell' on 'Juvenile arthritis'

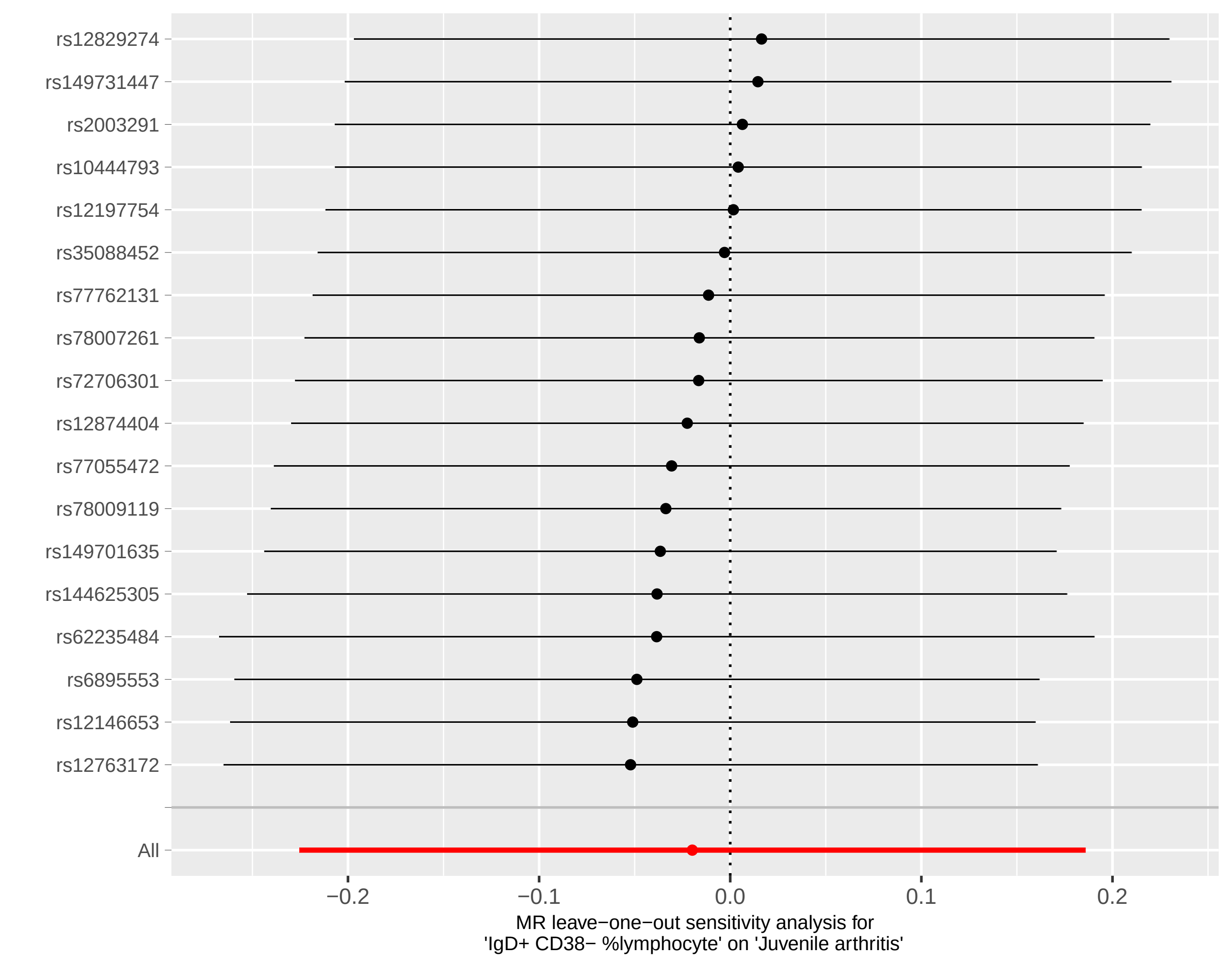
rs12829274
rs149731447
rs2003291
rs10444793
rs12197754
rs35088452
rs77762131
rs78007261
rs72706301
rs12874404
rs77055472
rs78009119
rs149701635
rs144625305
rs62235484
rs6895553
rs12146653
rs12763172
All
−0.2
−0.1
0.0
0.1
0.2
MR leave−one−out sensitivity analysis for
'IgD+ CD38− %lymphocyte' on 'Juvenile arthritis'

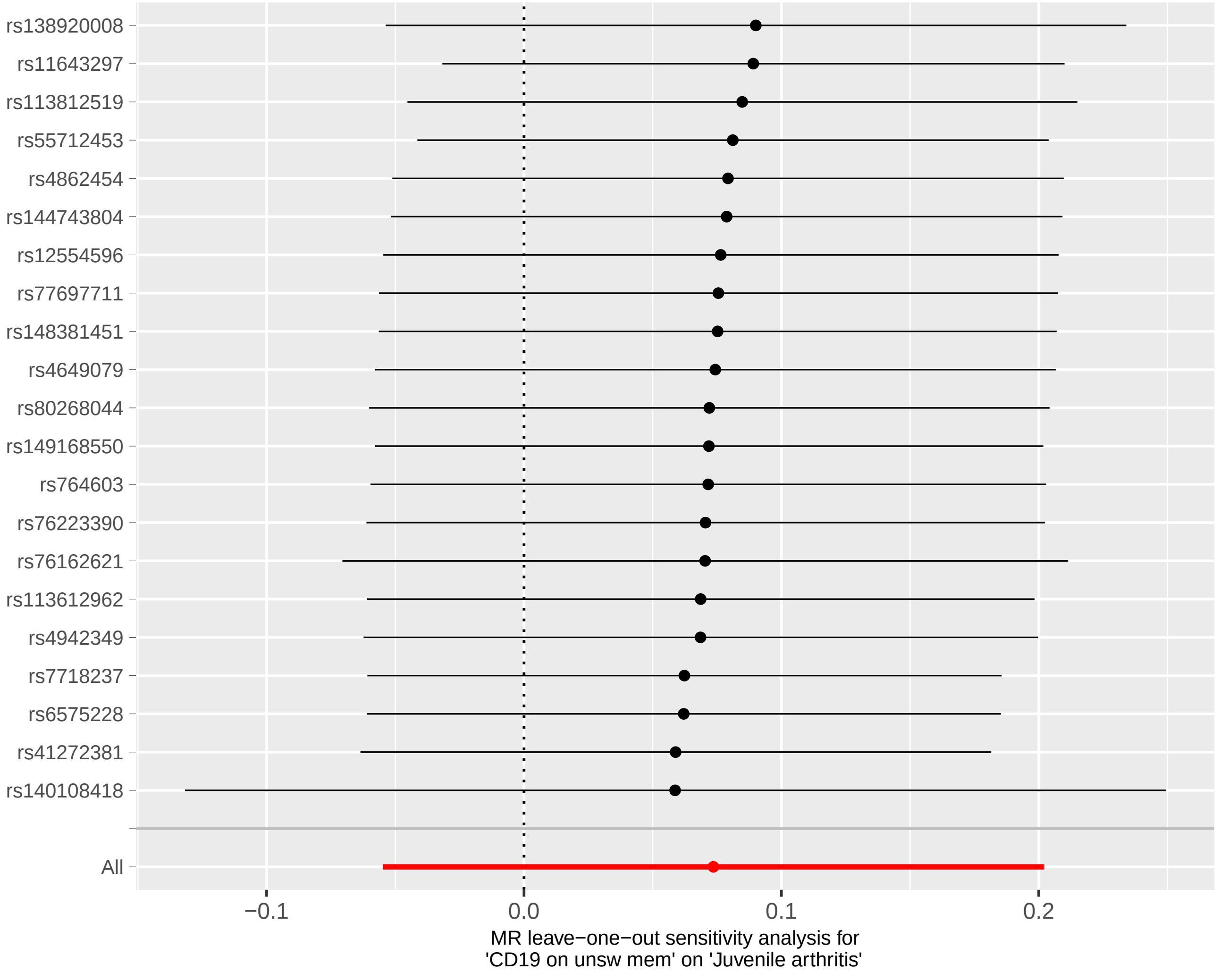
rs138920008
rs11643297
rs113812519
rs55712453
rs4862454
rs144743804
rs12554596
rs77697711
rs148381451
rs4649079
rs80268044
rs149168550
rs764603
rs76223390
rs76162621
rs113612962
rs4942349
rs7718237
rs6575228
rs41272381
rs140108418
All
−0.1
0.0
0.1
0.2
MR leave−one−out sensitivity analysis for
'CD19 on unsw mem' on 'Juvenile arthritis'

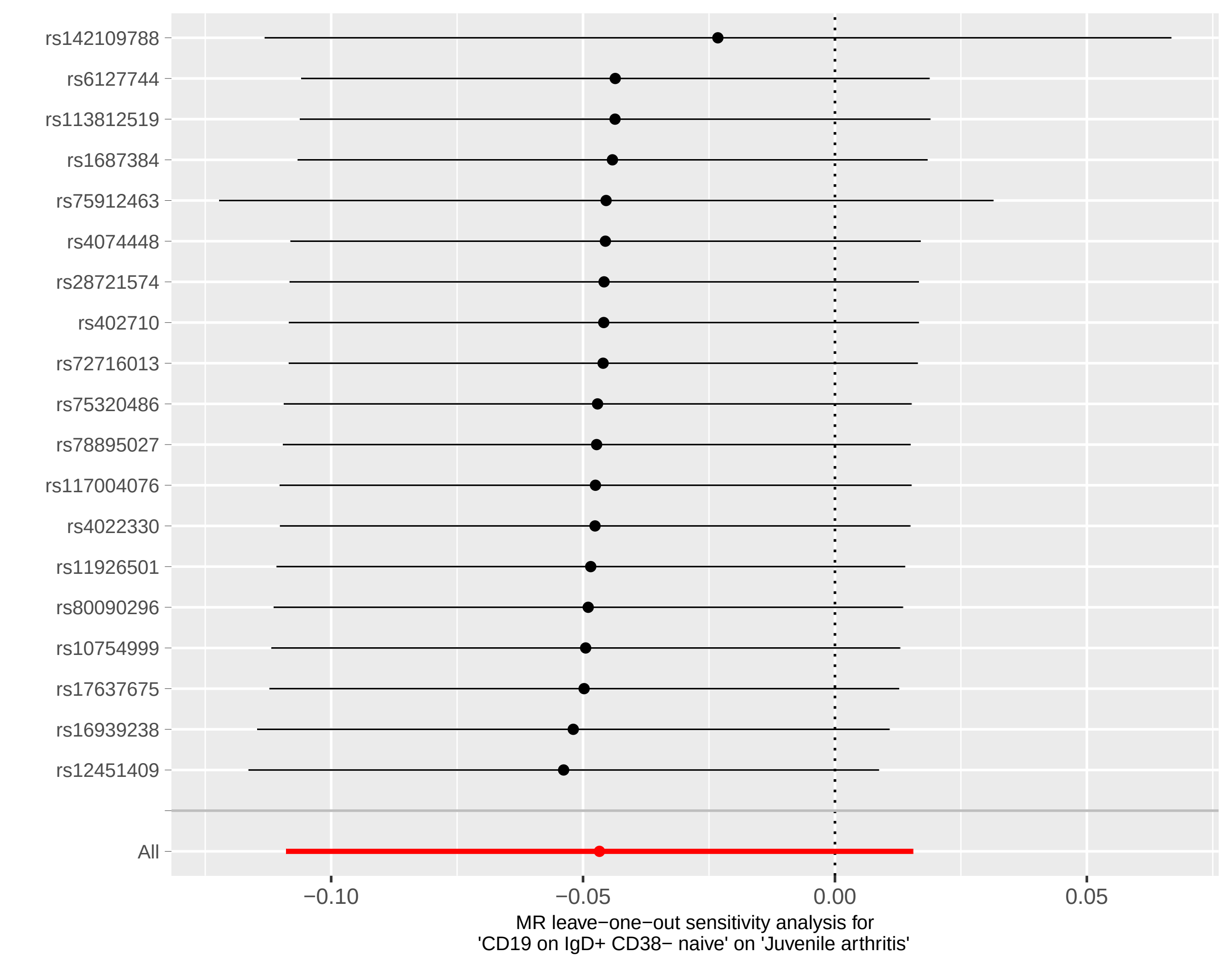

rs142109788
rs6127744
rs113812519
rs1687384
rs75912463
rs4074448
rs28721574
rs402710
rs72716013
rs75320486
rs78895027
rs117004076
rs4022330
rs11926501
rs80090296
rs10754999
rs17637675
rs16939238
rs12451409
All
−0.10
−0.05
0.00
0.05
MR leave−one−out sensitivity analysis for
'CD19 on IgD+ CD38− naive' on 'Juvenile arthritis'

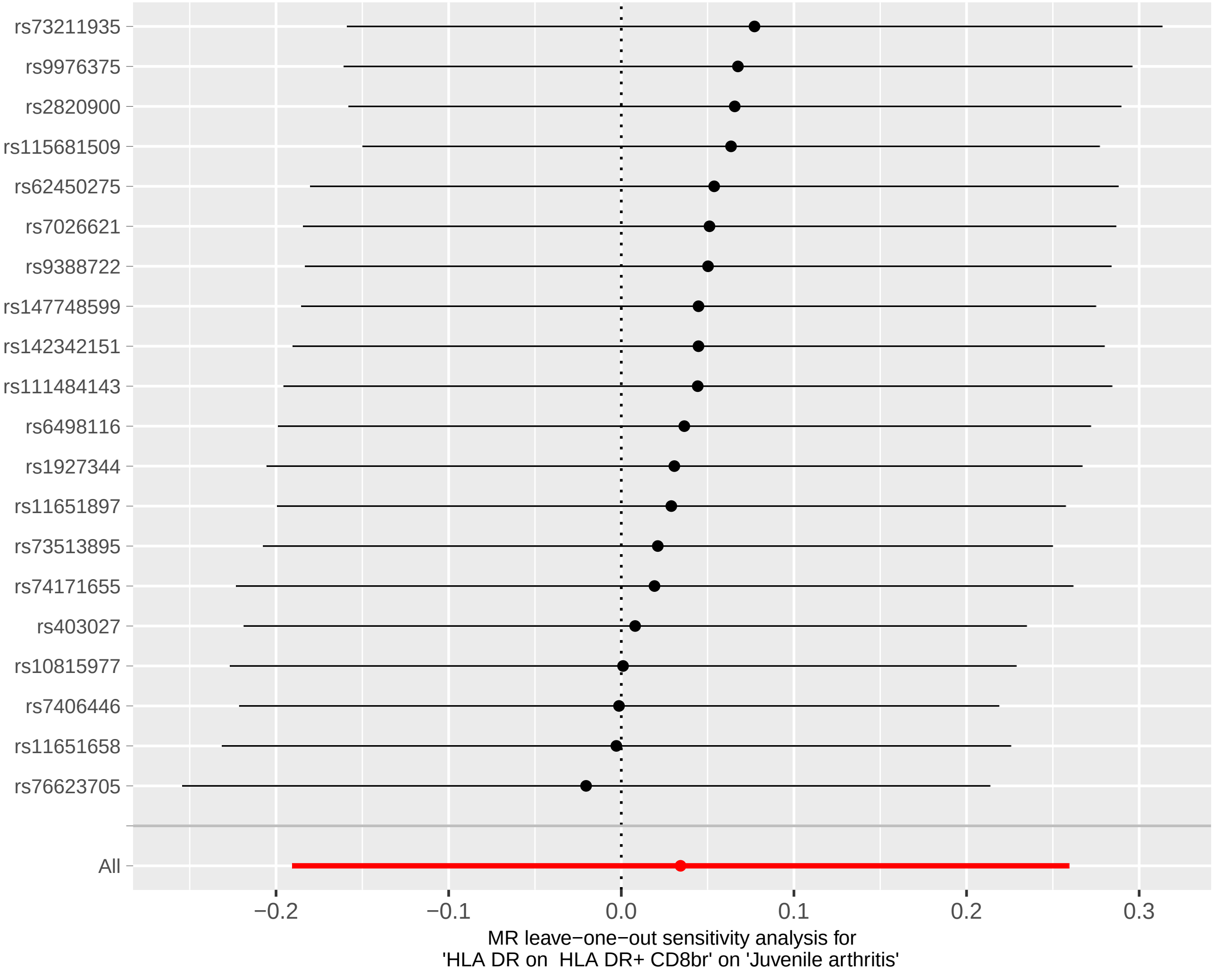
rs73211935
rs9976375
rs2820900
rs115681509
rs62450275
rs7026621
rs9388722
rs147748599
rs142342151
rs111484143
rs6498116
rs1927344
rs11651897
rs73513895
rs74171655
rs403027
rs10815977
rs7406446
rs11651658
rs76623705
All
−0.2
−0.1
0.0
0.1
0.2
0.3
MR leave−one−out sensitivity analysis for
'HLA DR on HLA DR+ CD8br' on 'Juvenile arthritis'

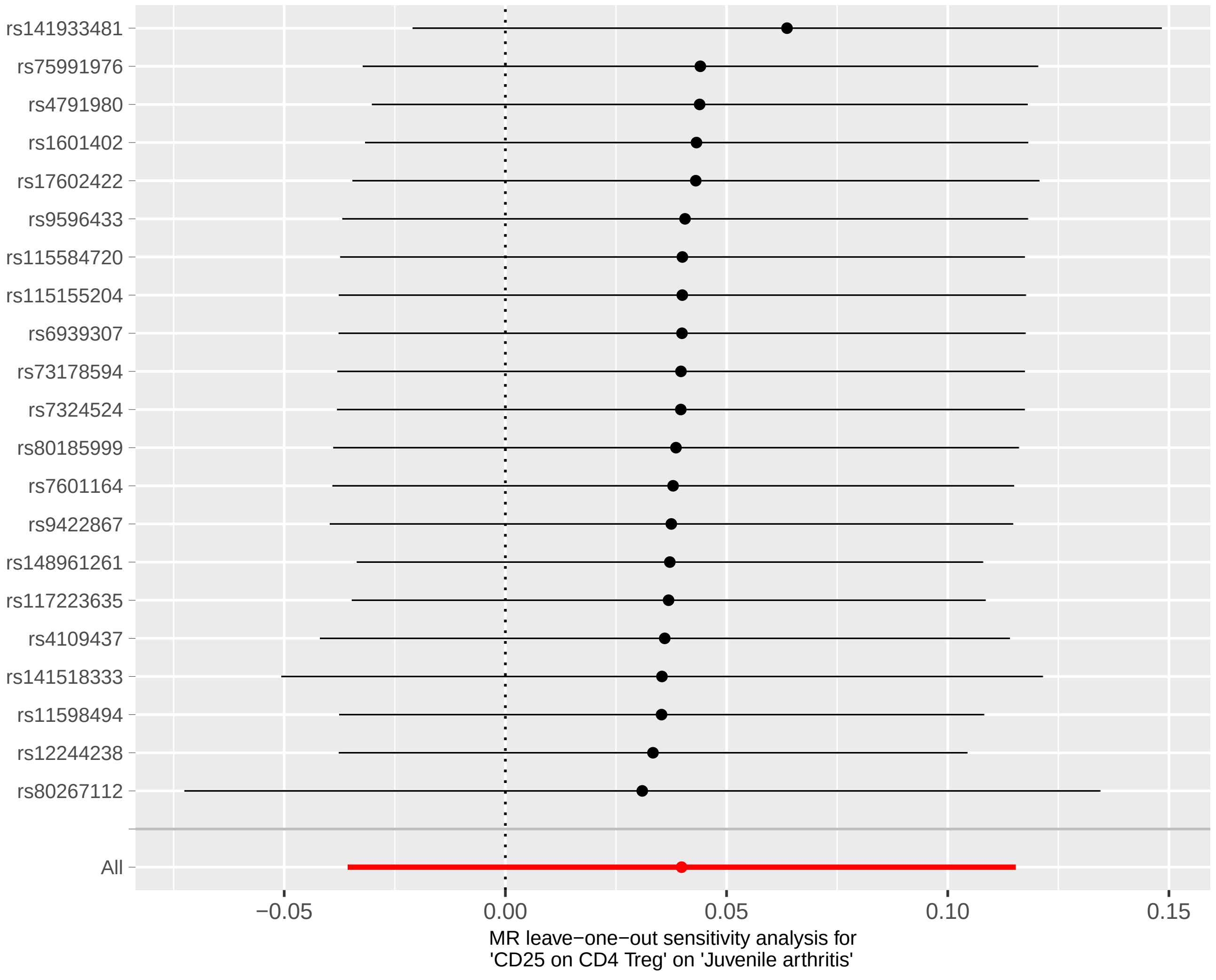

rs141933481
rs75991976
rs4791980
rs1601402
rs17602422
rs9596433
rs115584720
rs115155204
rs6939307
rs73178594
rs7324524
rs80185999
rs7601164
rs9422867
rs148961261
rs117223635
rs4109437
rs141518333
rs11598494
rs12244238
rs80267112
All
−0.05
0.00
0.05
0.10
0.15
MR leave−one−out sensitivity analysis for
'CD25 on CD4 Treg' on 'Juvenile arthritis'

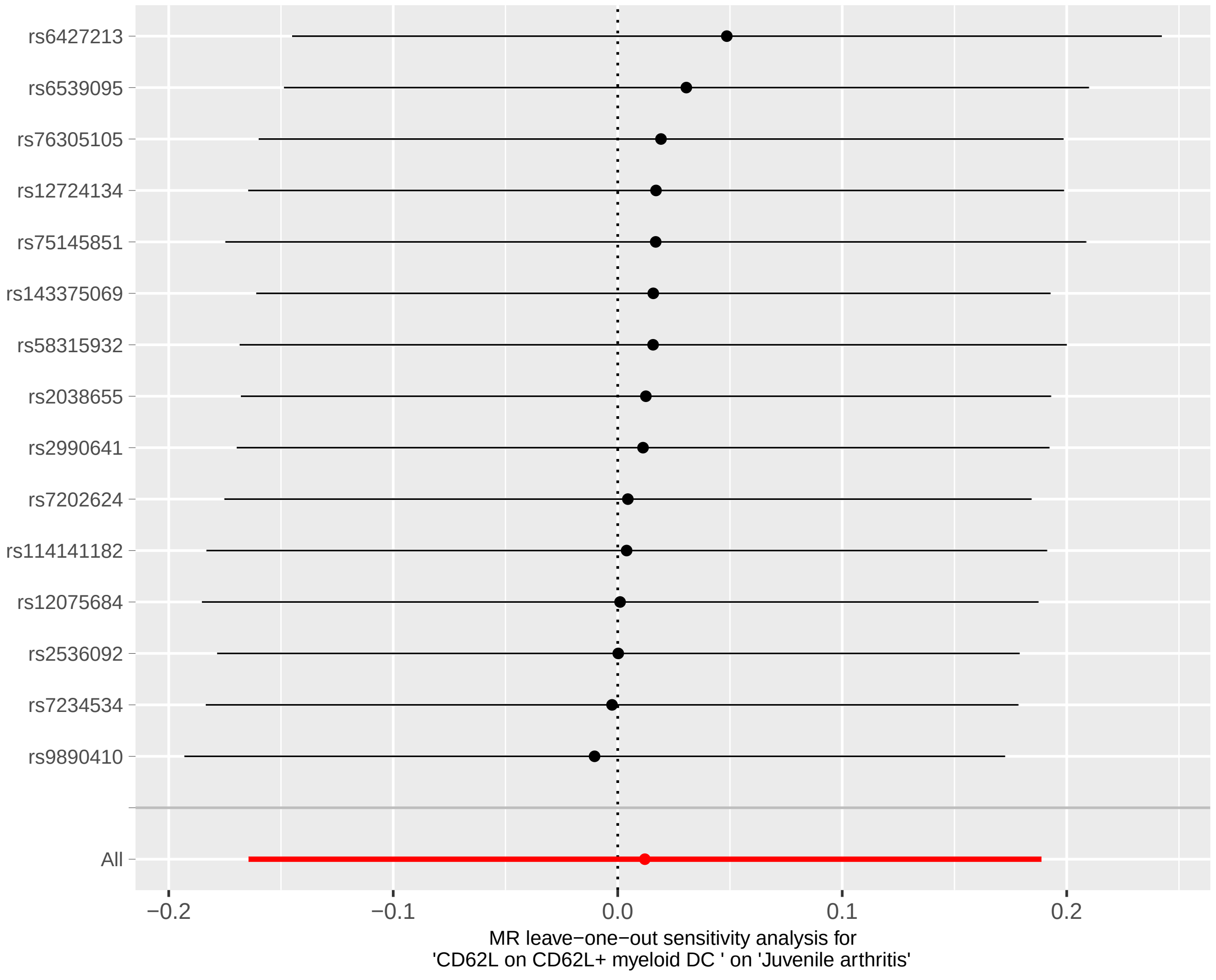

rs6427213
rs6539095
rs76305105
rs12724134
rs75145851
rs143375069
rs58315932
rs2038655
rs2990641
rs7202624
rs114141182
rs12075684
rs2536092
rs7234534
rs9890410
All
−0.2
−0.1
0.0
0.1
0.2
MR leave−one−out sensitivity analysis for
'CD62L on CD62L+ myeloid DC ' on 'Juvenile arthritis'

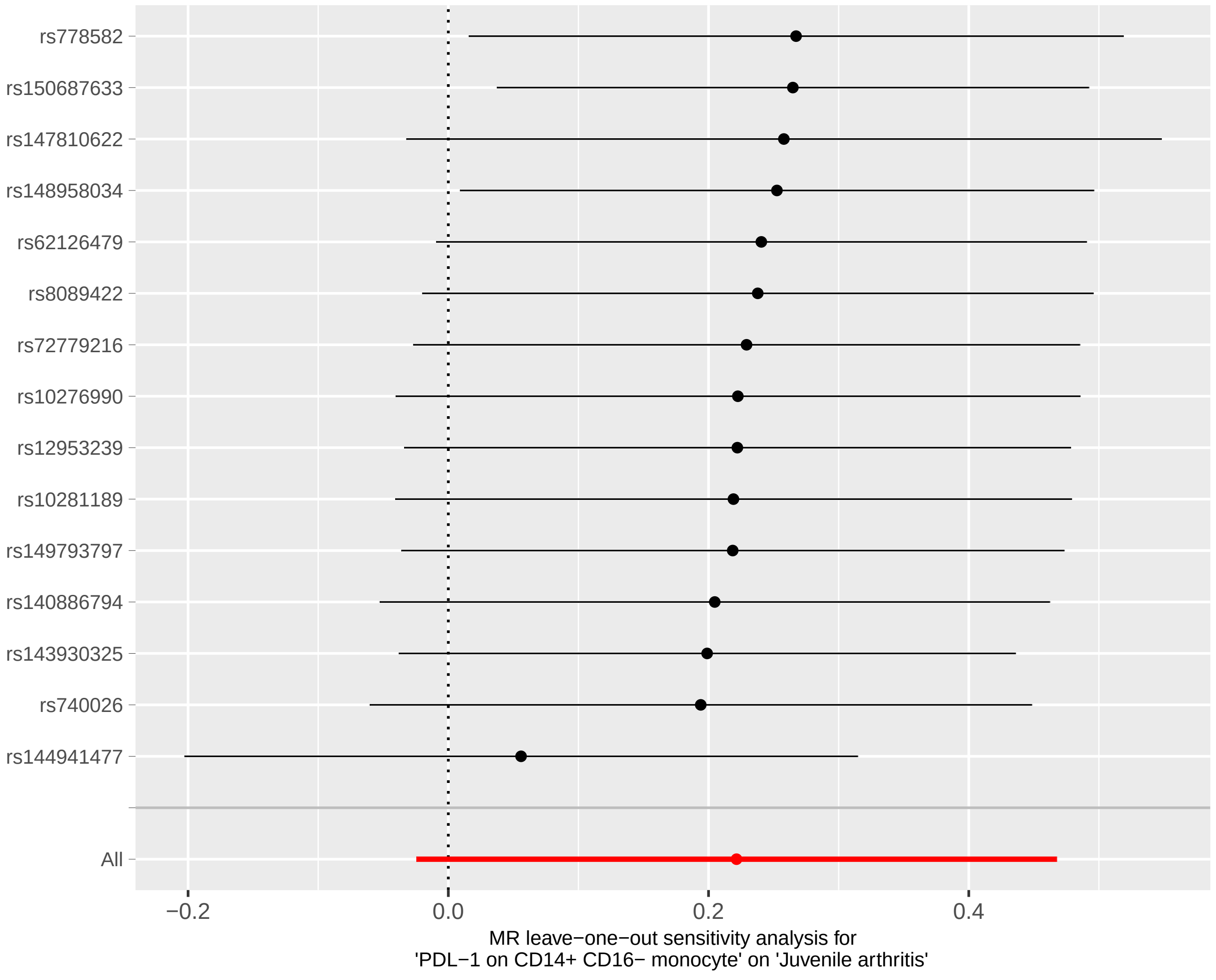
rs778582
rs150687633
rs147810622
rs148958034
rs62126479
rs8089422
rs72779216
rs10276990
rs12953239
rs10281189
rs149793797
rs140886794
rs143930325
rs740026
rs144941477
All
−0.2
0.0
0.2
0.4
MR leave−one−out sensitivity analysis for
'PDL−1 on CD14+ CD16− monocyte' on 'Juvenile arthritis'

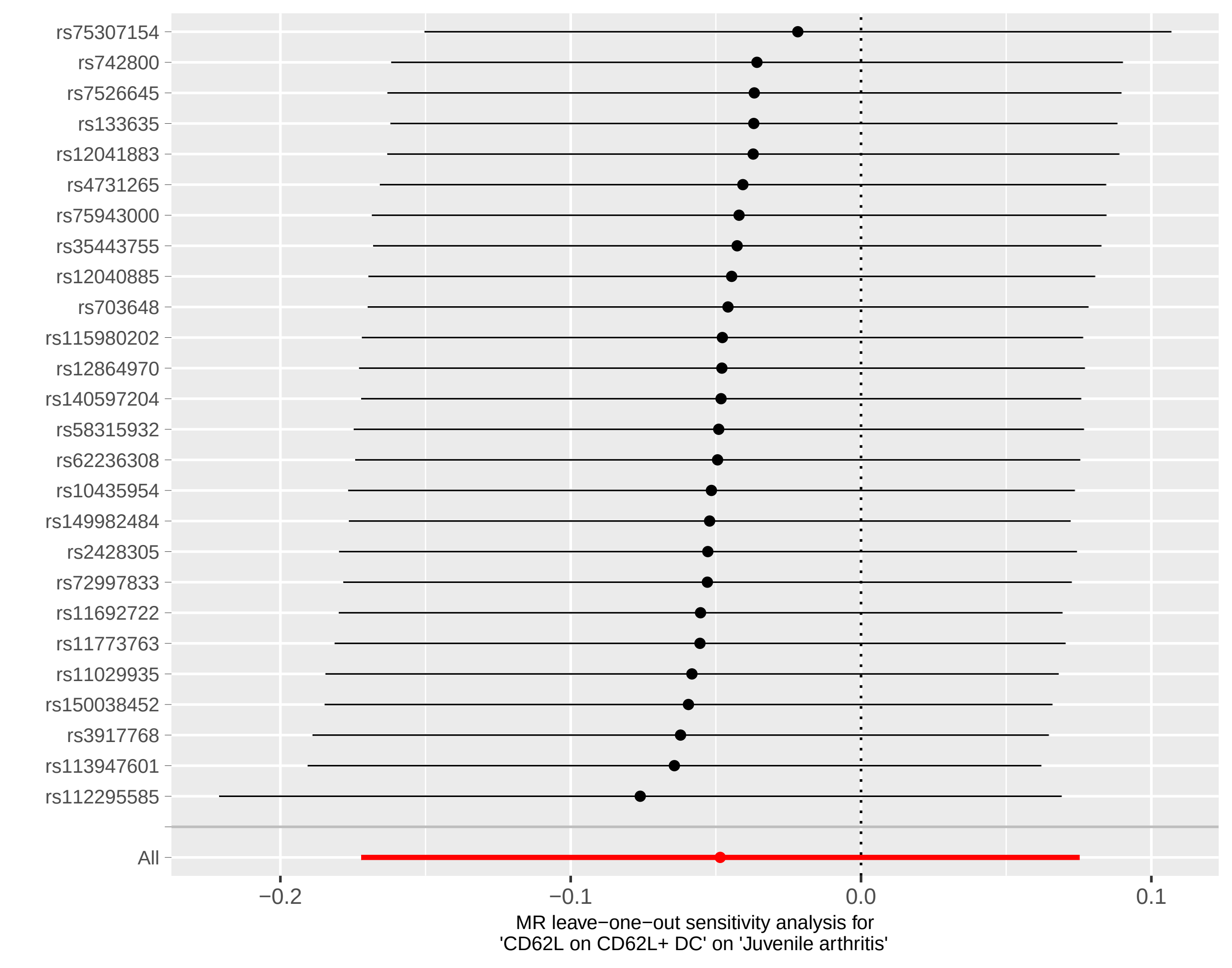

rs75307154
rs742800
rs7526645
rs133635
rs12041883
rs4731265
rs75943000
rs35443755
rs12040885
rs703648
rs115980202
rs12864970
rs140597204
rs58315932
rs62236308
rs10435954
rs149982484
rs2428305
rs72997833
rs11692722
rs11773763
rs11029935
rs150038452
rs3917768
rs113947601
rs112295585
All
−0.2
−0.1
0.0
0.1
MR leave−one−out sensitivity analysis for
'CD62L on CD62L+ DC' on 'Juvenile arthritis'

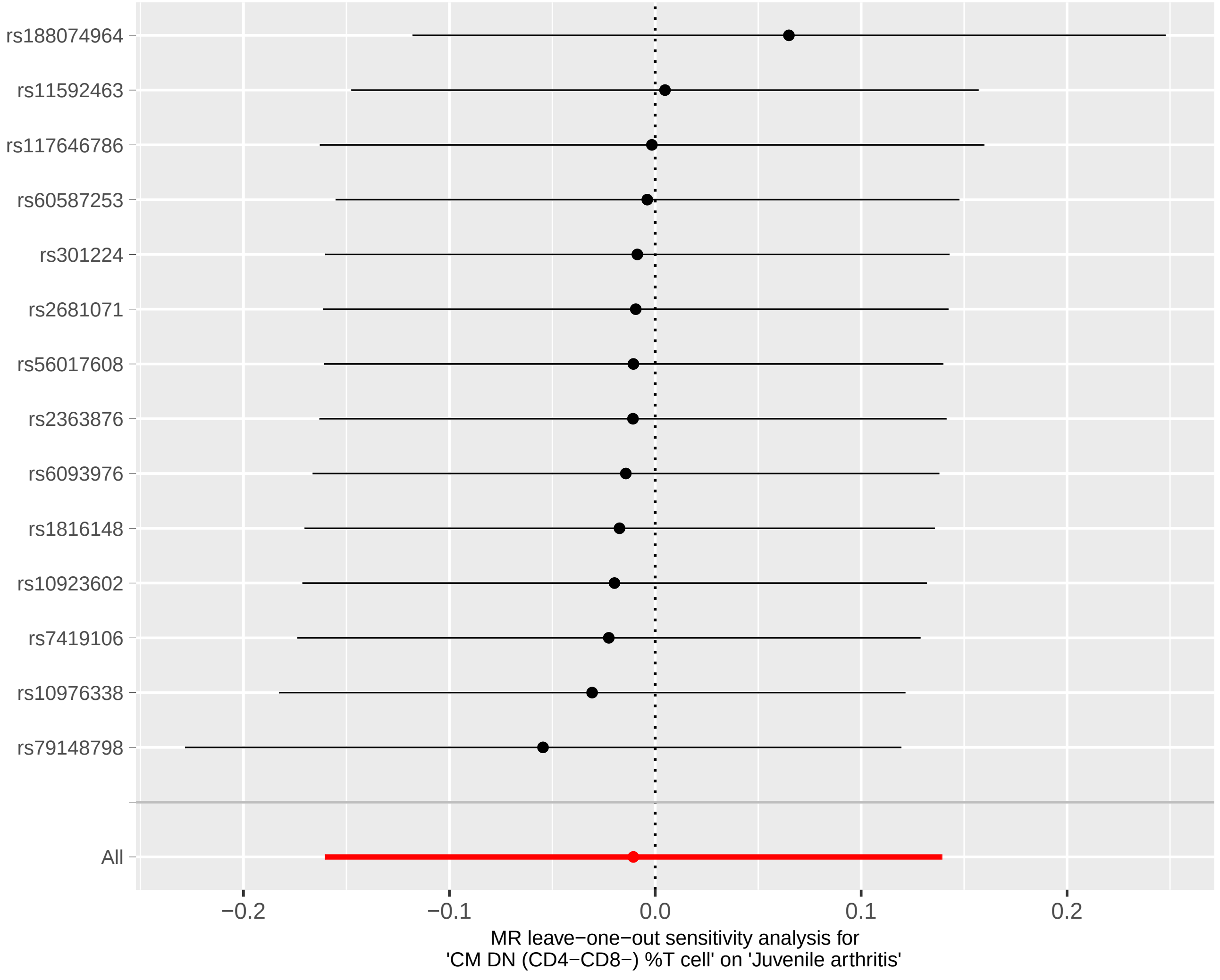
rs188074964
rs11592463
rs117646786
rs60587253
rs301224
rs2681071
rs56017608
rs2363876
rs6093976
rs1816148
rs10923602
rs7419106
rs10976338
rs79148798
All
−0.2
−0.1
0.0
0.1
0.2
MR leave−one−out sensitivity analysis for
'CM DN (CD4−CD8−) %T cell' on 'Juvenile arthritis'

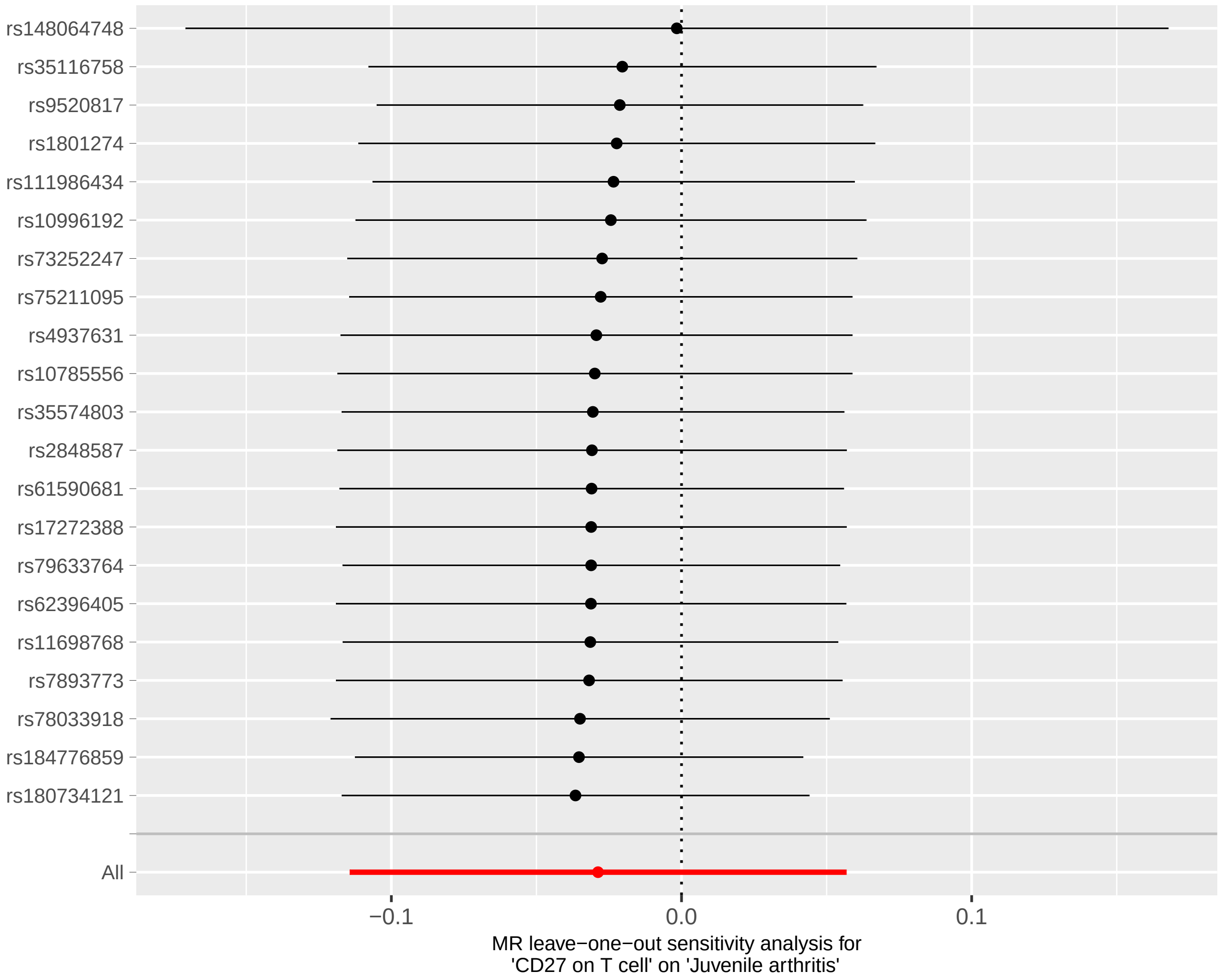
rs148064748
rs35116758
rs9520817
rs1801274
rs111986434
rs10996192
rs73252247
rs75211095
rs4937631
rs10785556
rs35574803
rs2848587
rs61590681
rs17272388
rs79633764
rs62396405
rs11698768
rs7893773
rs78033918
rs184776859
rs180734121
All
−0.1
0.0
0.1
MR leave−one−out sensitivity analysis for
'CD27 on T cell' on 'Juvenile arthritis'

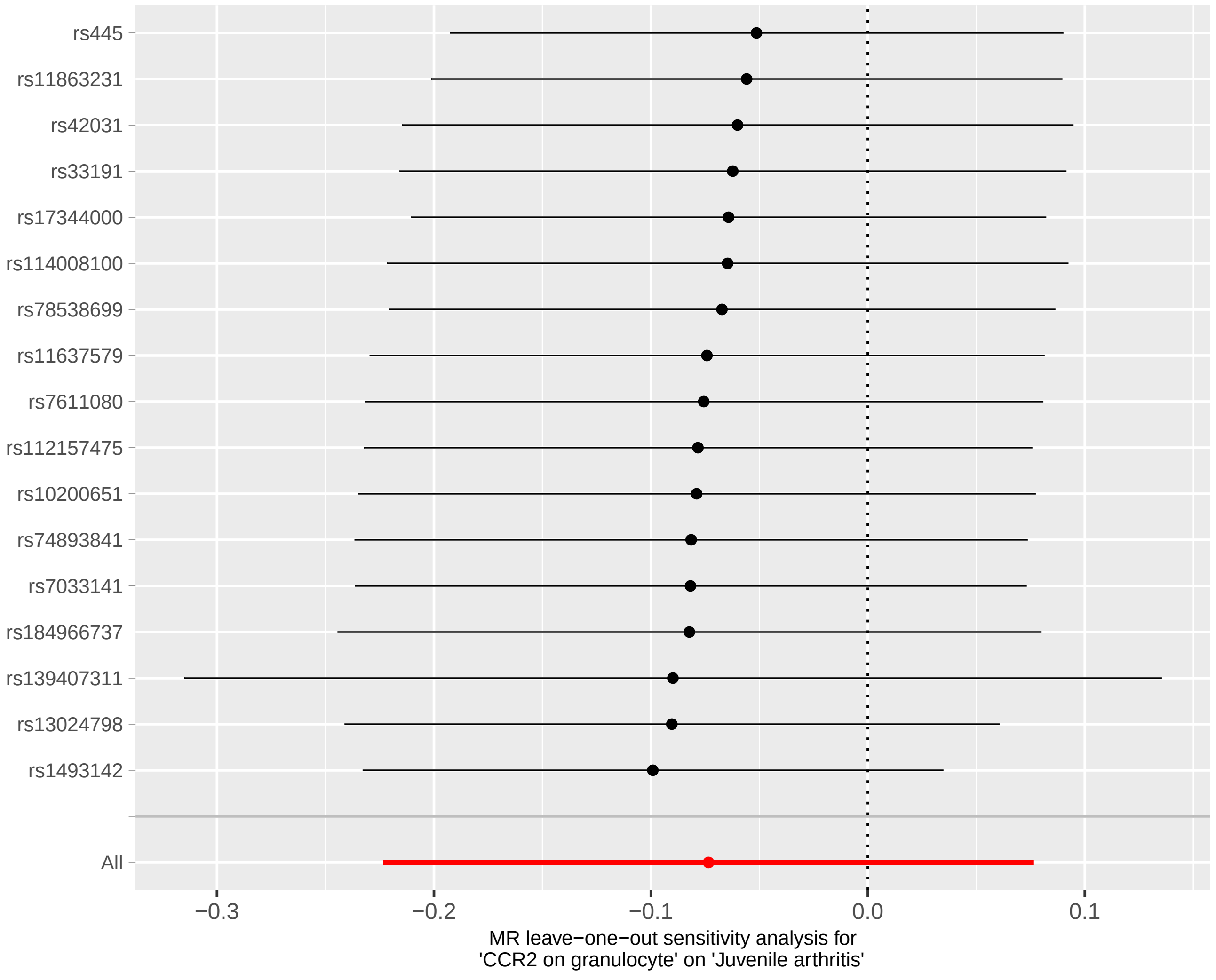
rs445
rs11863231
rs42031
rs33191
rs17344000
rs114008100
rs78538699
rs11637579
rs7611080
rs112157475
rs10200651
rs74893841
rs7033141
rs184966737
rs139407311
rs13024798
rs1493142
All
−0.3
−0.2
−0.1
0.0
0.1
MR leave−one−out sensitivity analysis for
'CCR2 on granulocyte' on 'Juvenile arthritis'

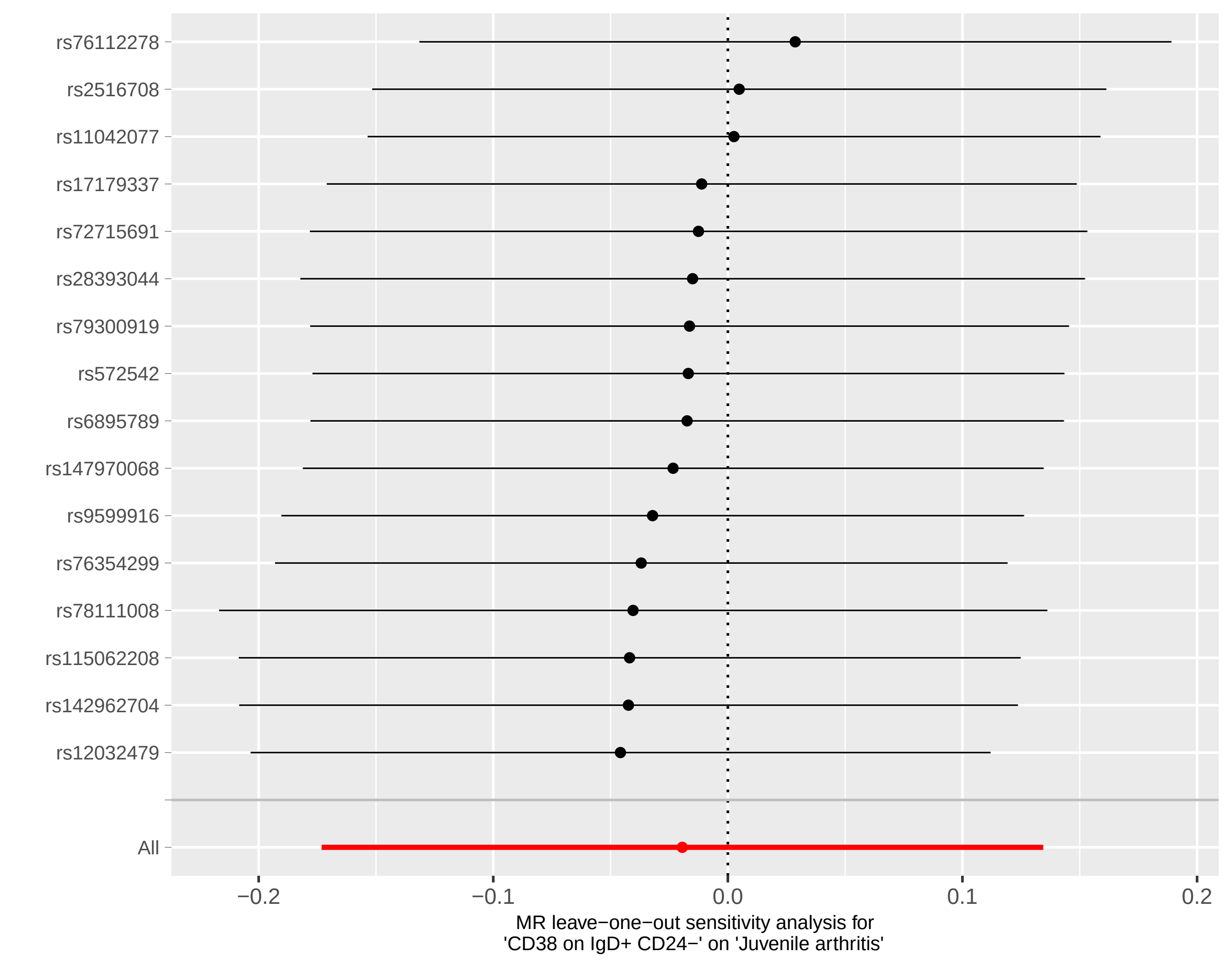
rs76112278
rs2516708
rs11042077
rs17179337
rs72715691
rs28393044
rs79300919
rs572542
rs6895789
rs147970068
rs9599916
rs76354299
rs78111008
rs115062208
rs142962704
rs12032479
All
−0.2
−0.1
0.0
0.1
0.2
MR leave−one−out sensitivity analysis for
'CD38 on IgD+ CD24−' on 'Juvenile arthritis'

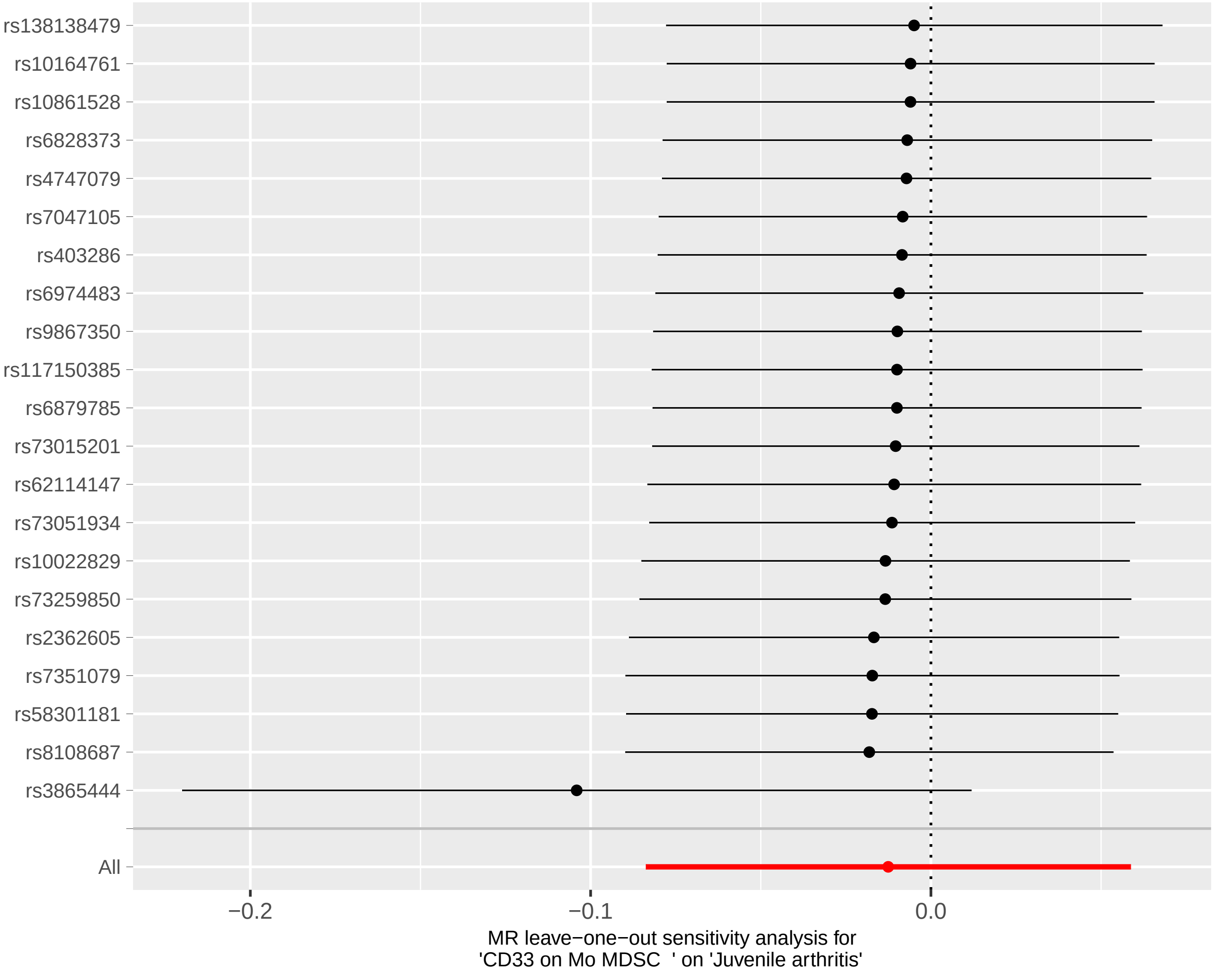
rs138138479
rs10164761
rs10861528
rs6828373
rs4747079
rs7047105
rs403286
rs6974483
rs9867350
rs117150385
rs6879785
rs73015201
rs62114147
rs73051934
rs10022829
rs73259850
rs2362605
rs7351079
rs58301181
rs8108687
rs3865444
All
−0.2
−0.1
0.0
MR leave−one−out sensitivity analysis for
'CD33 on Mo MDSC ' on 'Juvenile arthritis'

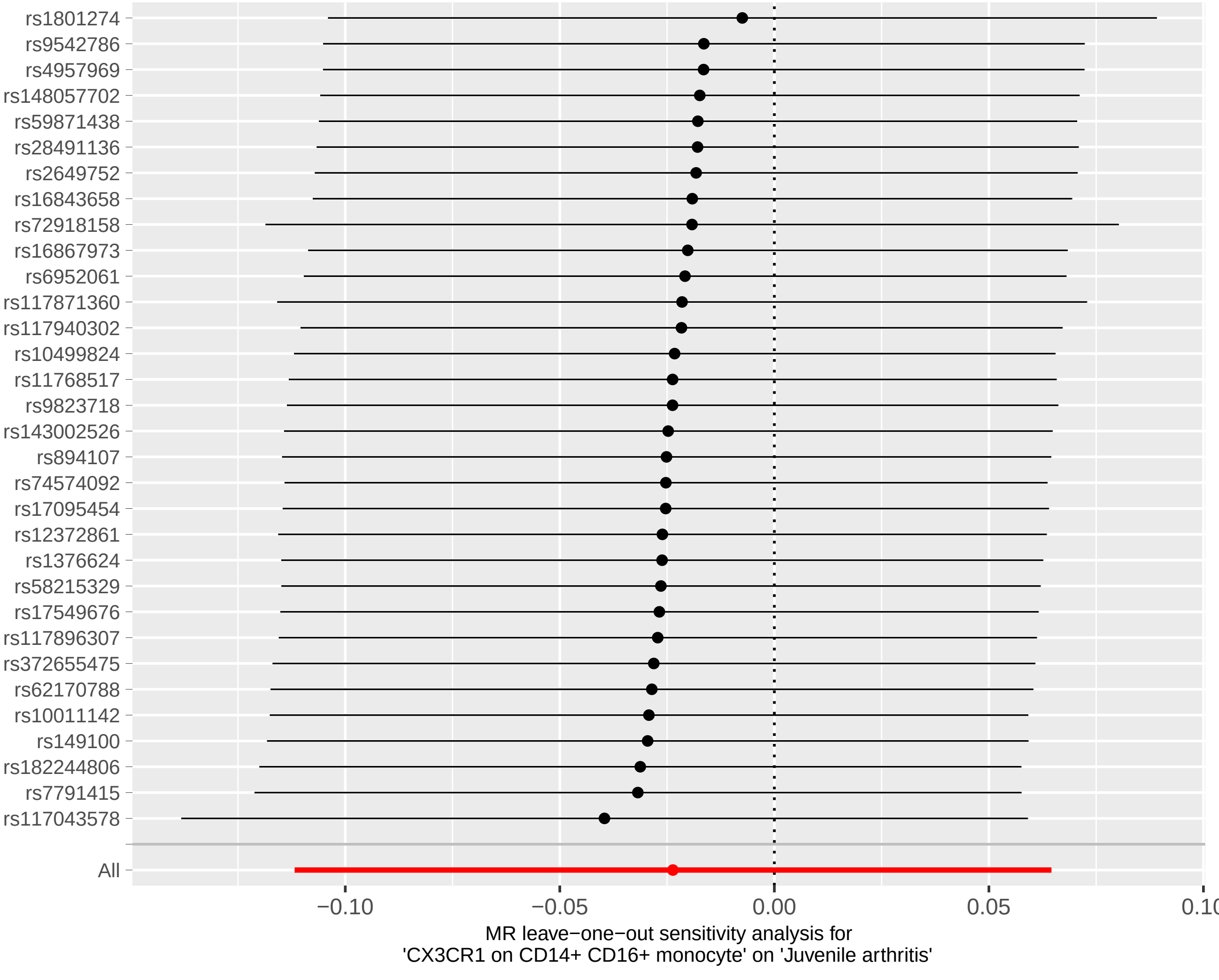
rs1801274
rs9542786
rs4957969
rs148057702
rs59871438
rs28491136
rs2649752
rs16843658
rs72918158
rs16867973
rs6952061
rs117871360
rs117940302
rs10499824
rs11768517
rs9823718
rs143002526
rs894107
rs74574092
rs17095454
rs12372861
rs1376624
rs58215329
rs17549676
rs117896307
rs372655475
rs62170788
rs10011142
rs149100
rs182244806
rs7791415
rs117043578
All
−0.10
−0.05
0.00
0.05
0.10
MR leave−one−out sensitivity analysis for
'CX3CR1 on CD14+ CD16+ monocyte' on 'Juvenile arthritis'

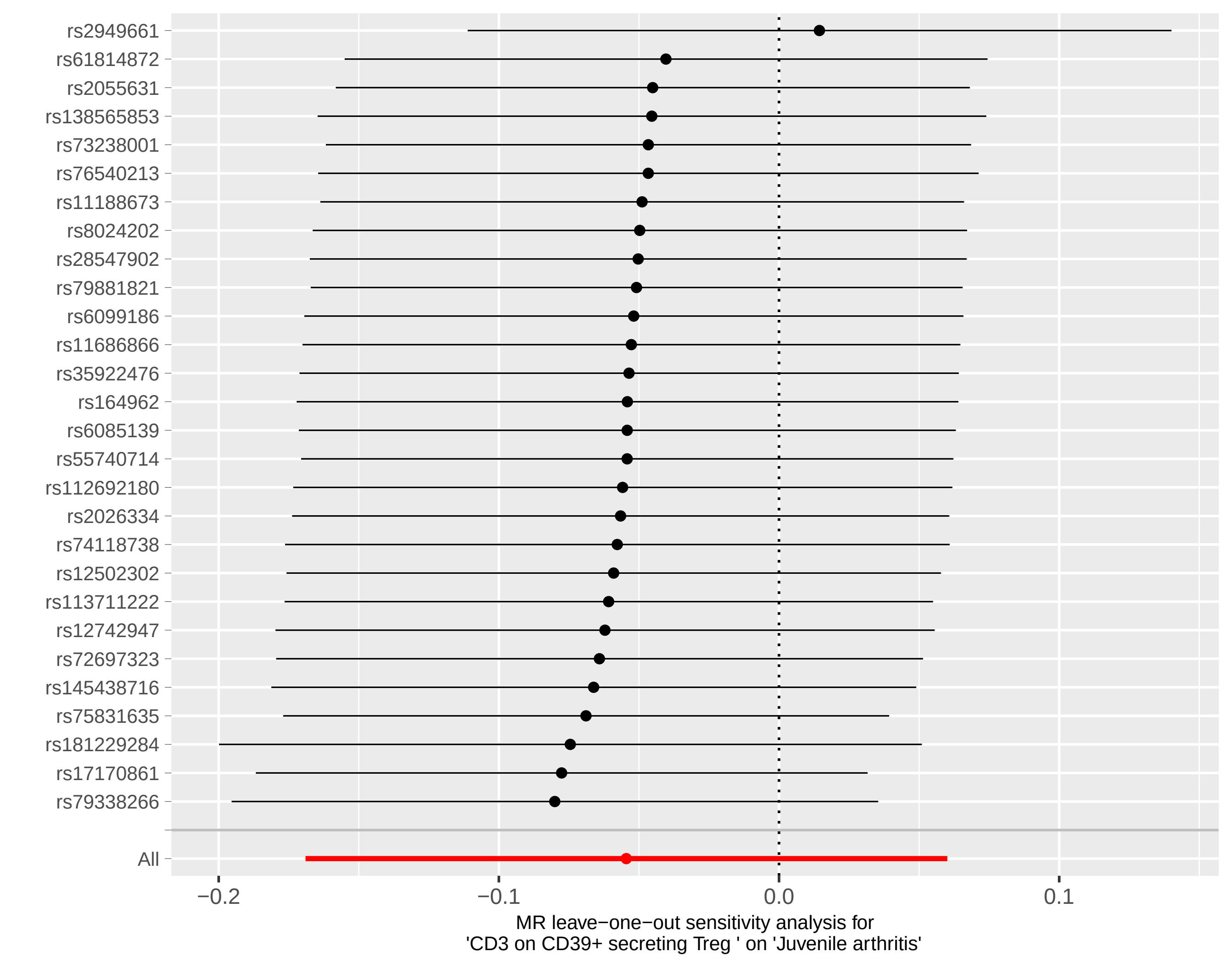
rs2949661
rs61814872
rs2055631
rs138565853
rs73238001
rs76540213
rs11188673
rs8024202
rs28547902
rs79881821
rs6099186
rs11686866
rs35922476
rs164962
rs6085139
rs55740714
rs112692180
rs2026334
rs74118738
rs12502302
rs113711222
rs12742947
rs72697323
rs145438716
rs75831635
rs181229284
rs17170861
rs79338266
All
−0.2
−0.1
0.0
0.1
MR leave−one−out sensitivity analysis for
'CD3 on CD39+ secreting Treg ' on 'Juvenile arthritis'

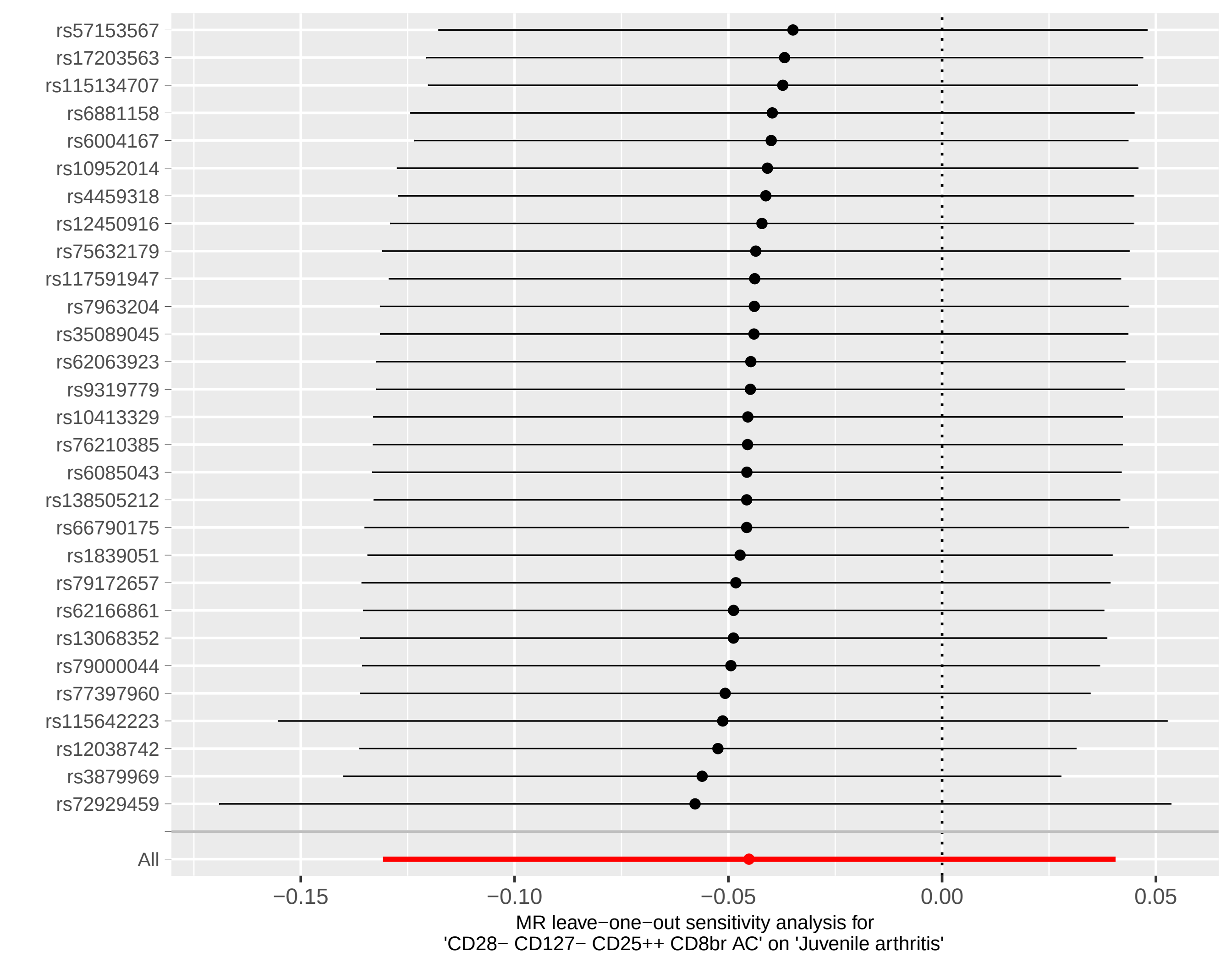
rs57153567
rs17203563
rs115134707
rs6881158
rs6004167
rs10952014
rs4459318
rs12450916
rs75632179
rs117591947
rs7963204
rs35089045
rs62063923
rs9319779
rs10413329
rs76210385
rs6085043
rs138505212
rs66790175
rs1839051
rs79172657
rs62166861
rs13068352
rs79000044
rs77397960
rs115642223
rs12038742
rs3879969
rs72929459
All
−0.15
−0.10
−0.05
0.00
0.05
MR leave−one−out sensitivity analysis for
'CD28− CD127− CD25++ CD8br AC' on 'Juvenile arthritis'

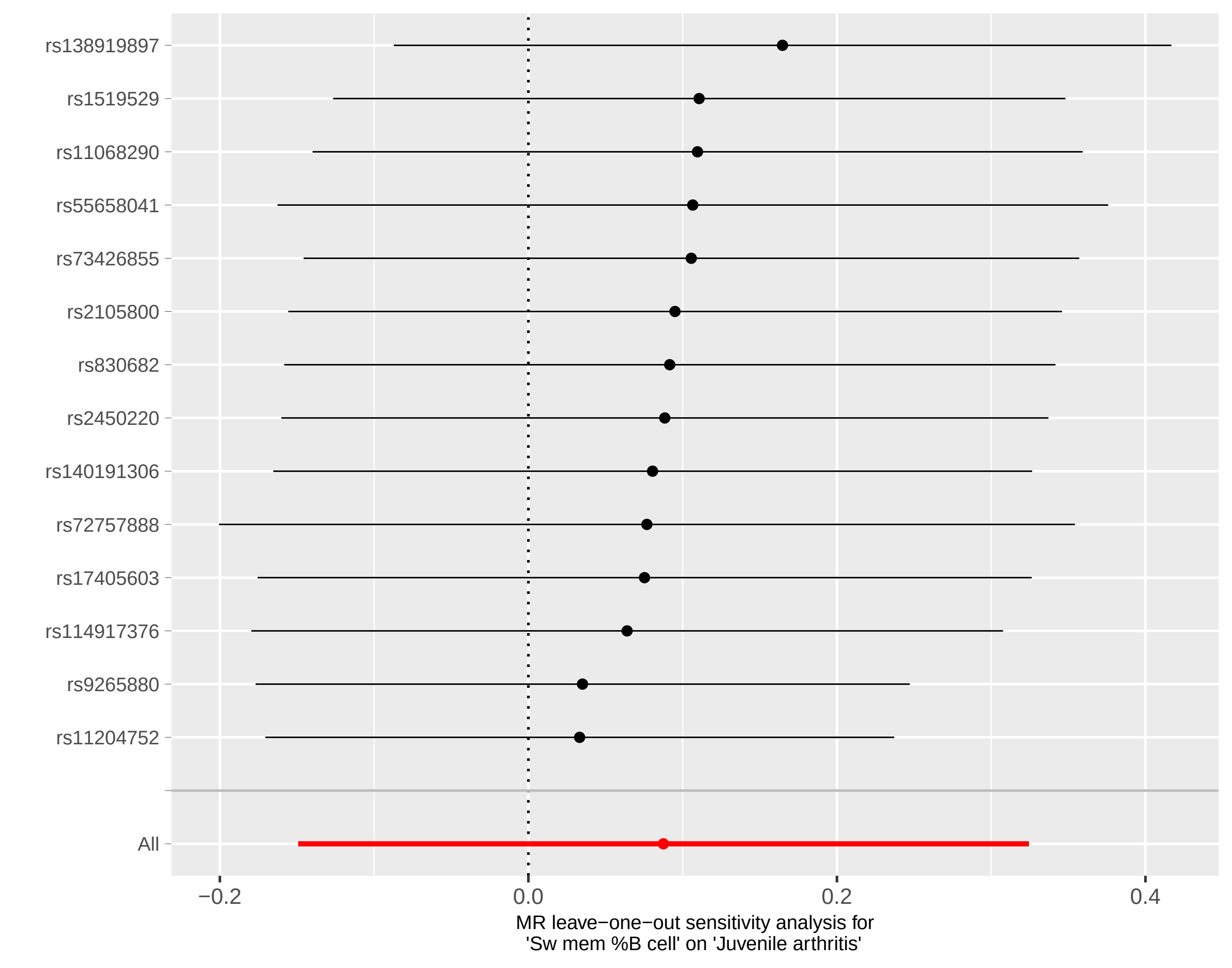

rs138919897
rs1519529
rs11068290
rs55658041
rs73426855
rs2105800
rs830682
rs2450220
rs140191306
rs72757888
rs17405603
rs114917376
rs9265880
rs11204752
All
−0.2
0.0
0.2
0.4
MR leave−one−out sensitivity analysis for
'Sw mem %B cell' on 'Juvenile arthritis'

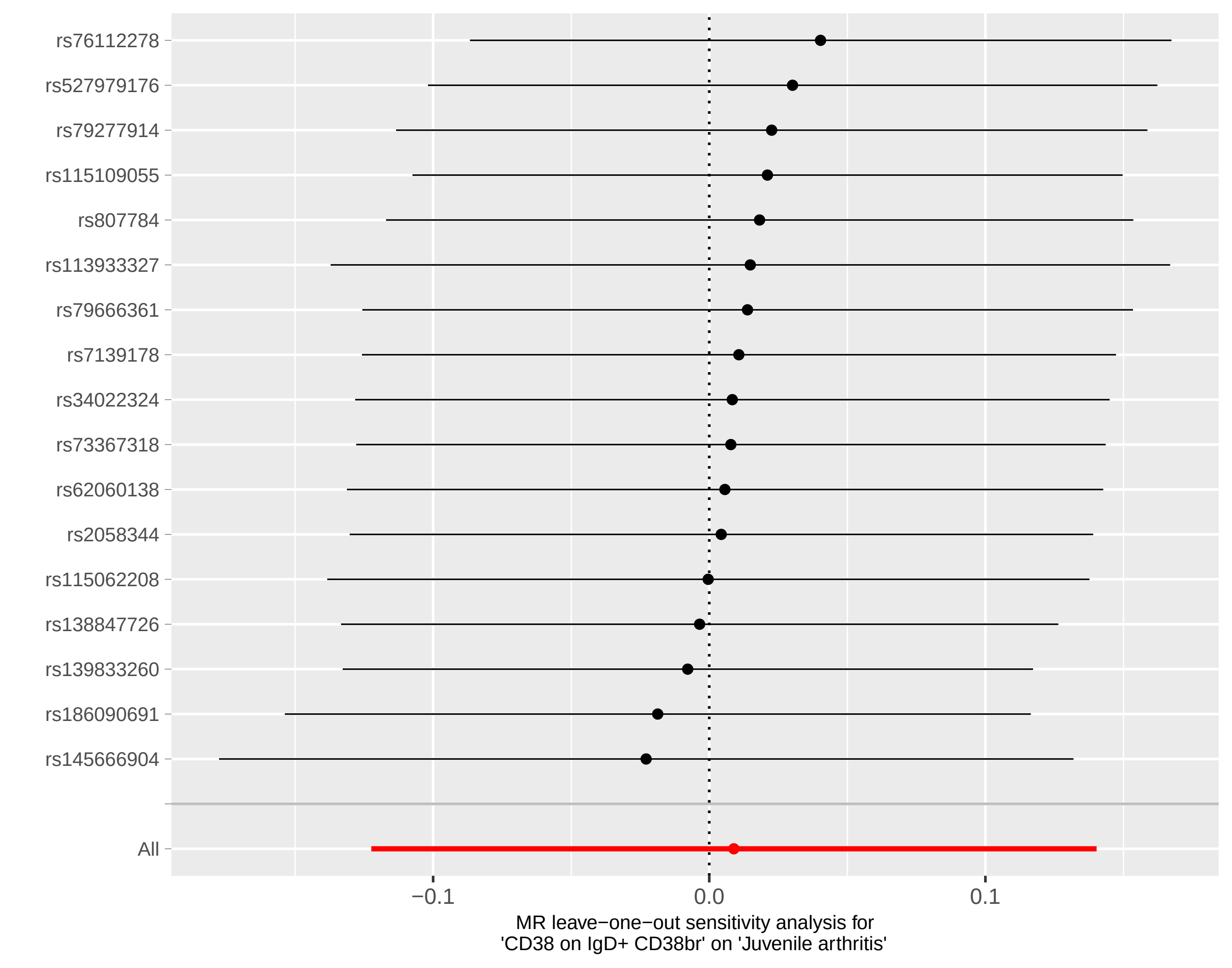
rs76112278
rs527979176
rs79277914
rs115109055
rs807784
rs113933327
rs79666361
rs7139178
rs34022324
rs73367318
rs62060138
rs2058344
rs115062208
rs138847726
rs139833260
rs186090691
rs145666904
All
−0.1
0.0
0.1
MR leave−one−out sensitivity analysis for
'CD38 on IgD+ CD38br' on 'Juvenile arthritis'

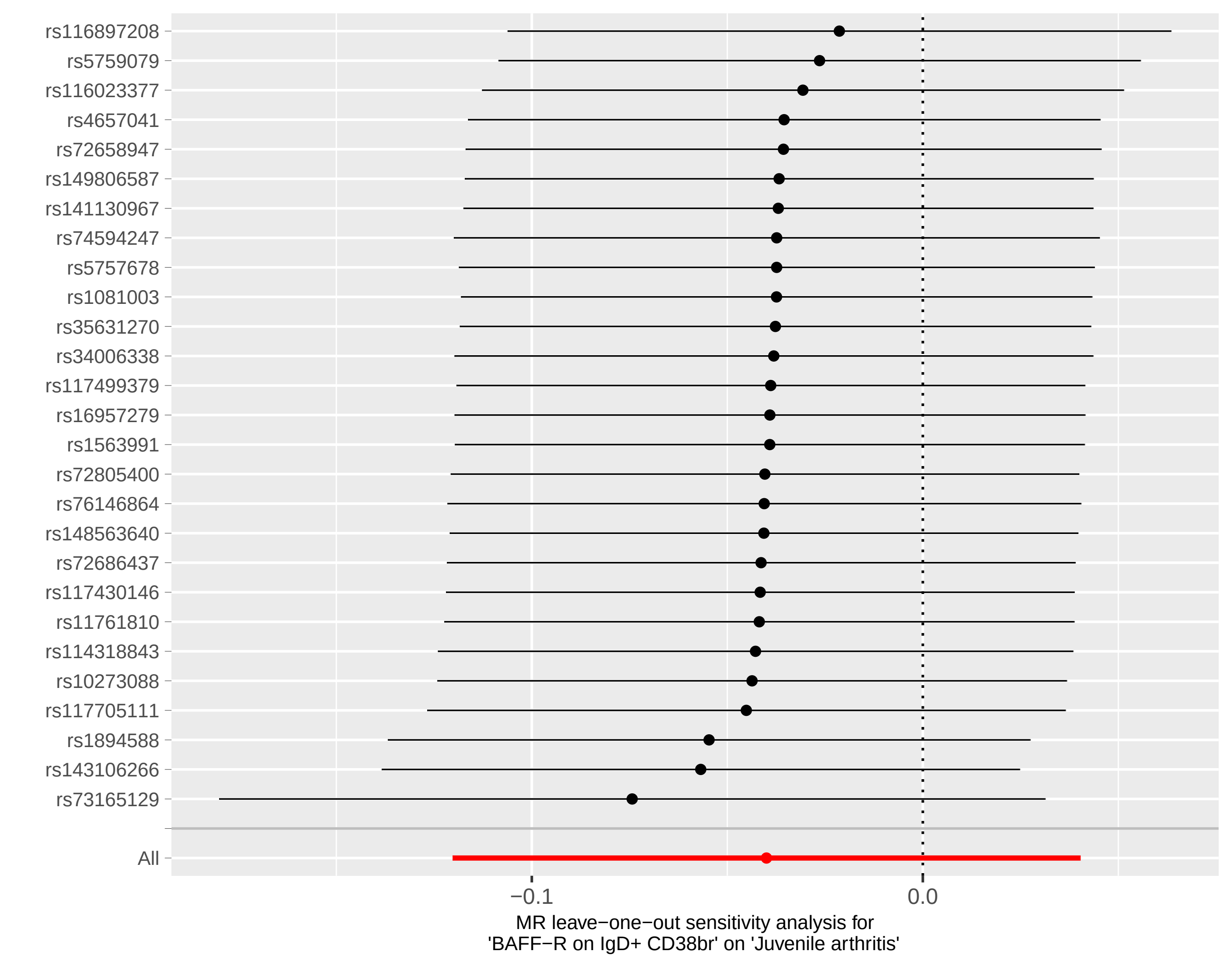
rs116897208
rs5759079
rs116023377
rs4657041
rs72658947
rs149806587
rs141130967
rs74594247
rs5757678
rs1081003
rs35631270
rs34006338
rs117499379
rs16957279
rs1563991
rs72805400
rs76146864
rs148563640
rs72686437
rs117430146
rs11761810
rs114318843
rs10273088
rs117705111
rs1894588
rs143106266
rs73165129
All
−0.1
0.0
MR leave−one−out sensitivity analysis for
'BAFF−R on IgD+ CD38br' on 'Juvenile arthritis'

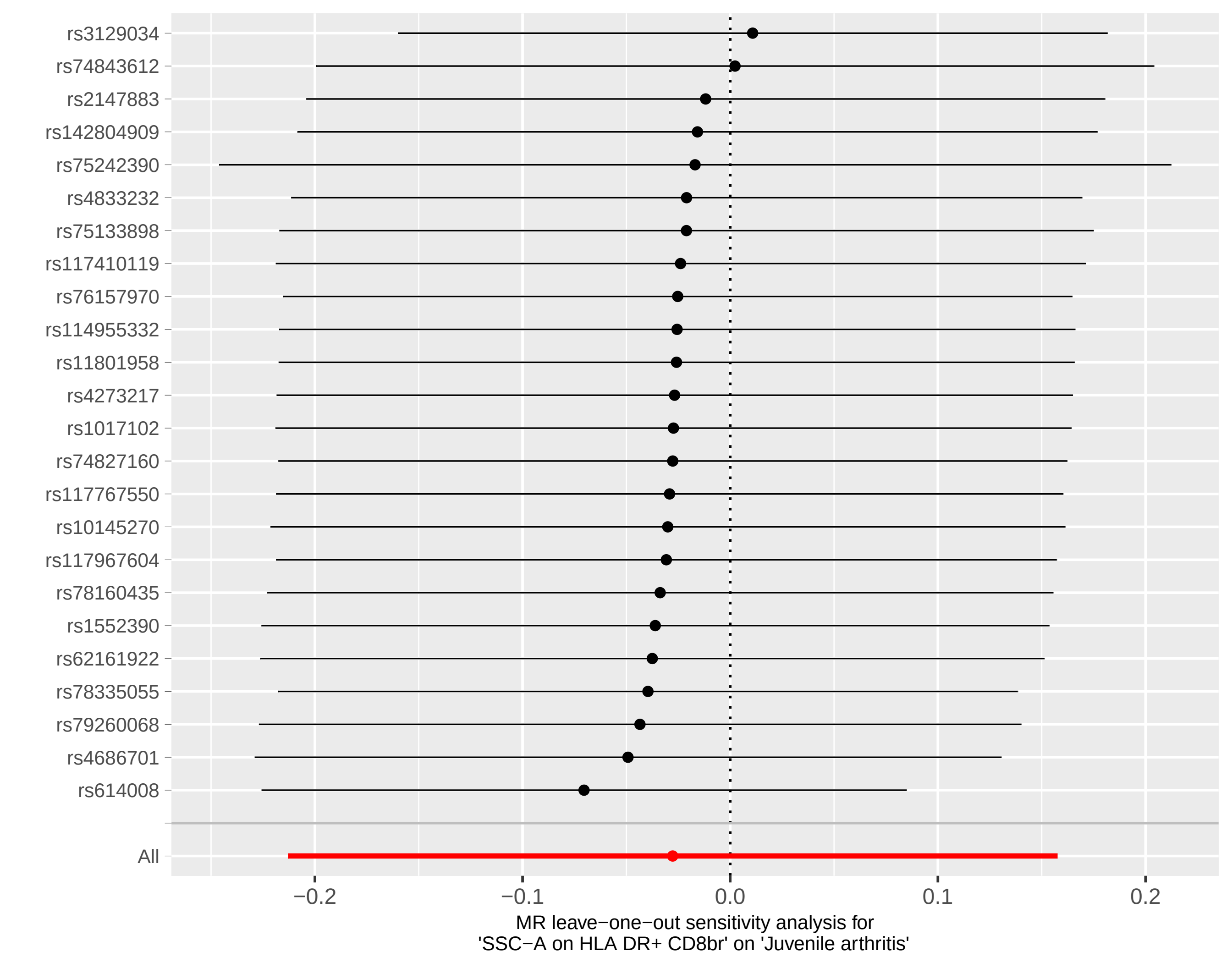
rs3129034
rs74843612
rs2147883
rs142804909
rs75242390
rs4833232
rs75133898
rs117410119
rs76157970
rs114955332
rs11801958
rs4273217
rs1017102
rs74827160
rs117767550
rs10145270
rs117967604
rs78160435
rs1552390
rs62161922
rs78335055
rs79260068
rs4686701
rs614008
All
−0.2
−0.1
0.0
0.1
0.2
MR leave−one−out sensitivity analysis for
'SSC−A on HLA DR+ CD8br' on 'Juvenile arthritis'

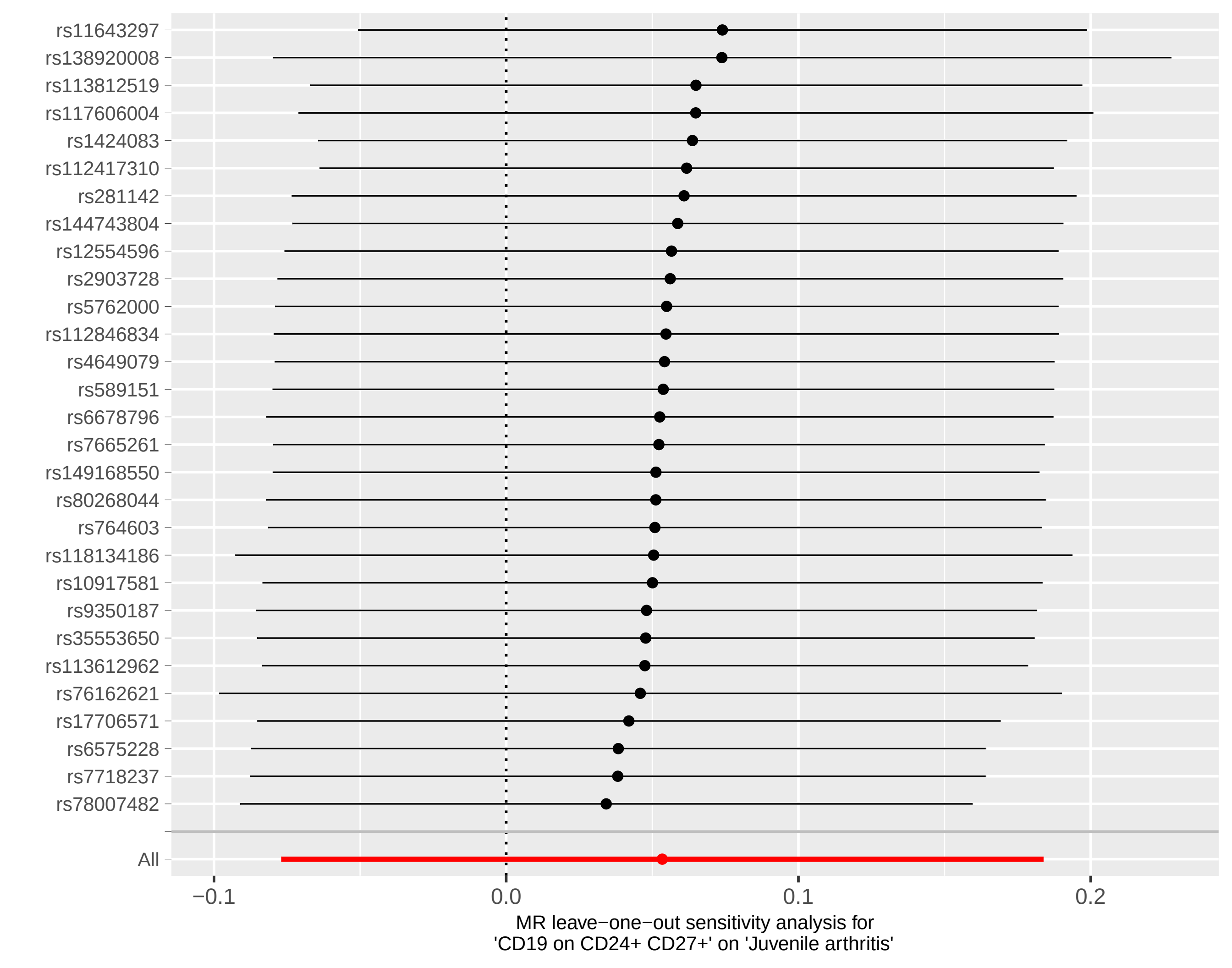
rs11643297
rs138920008
rs113812519
rs117606004
rs1424083
rs112417310
rs281142
rs144743804
rs12554596
rs2903728
rs5762000
rs112846834
rs4649079
rs589151
rs6678796
rs7665261
rs149168550
rs80268044
rs764603
rs118134186
rs10917581
rs9350187
rs35553650
rs113612962
rs76162621
rs17706571
rs6575228
rs7718237
rs78007482
All
−0.1
0.0
0.1
0.2
MR leave−one−out sensitivity analysis for
'CD19 on CD24+ CD27+' on 'Juvenile arthritis'

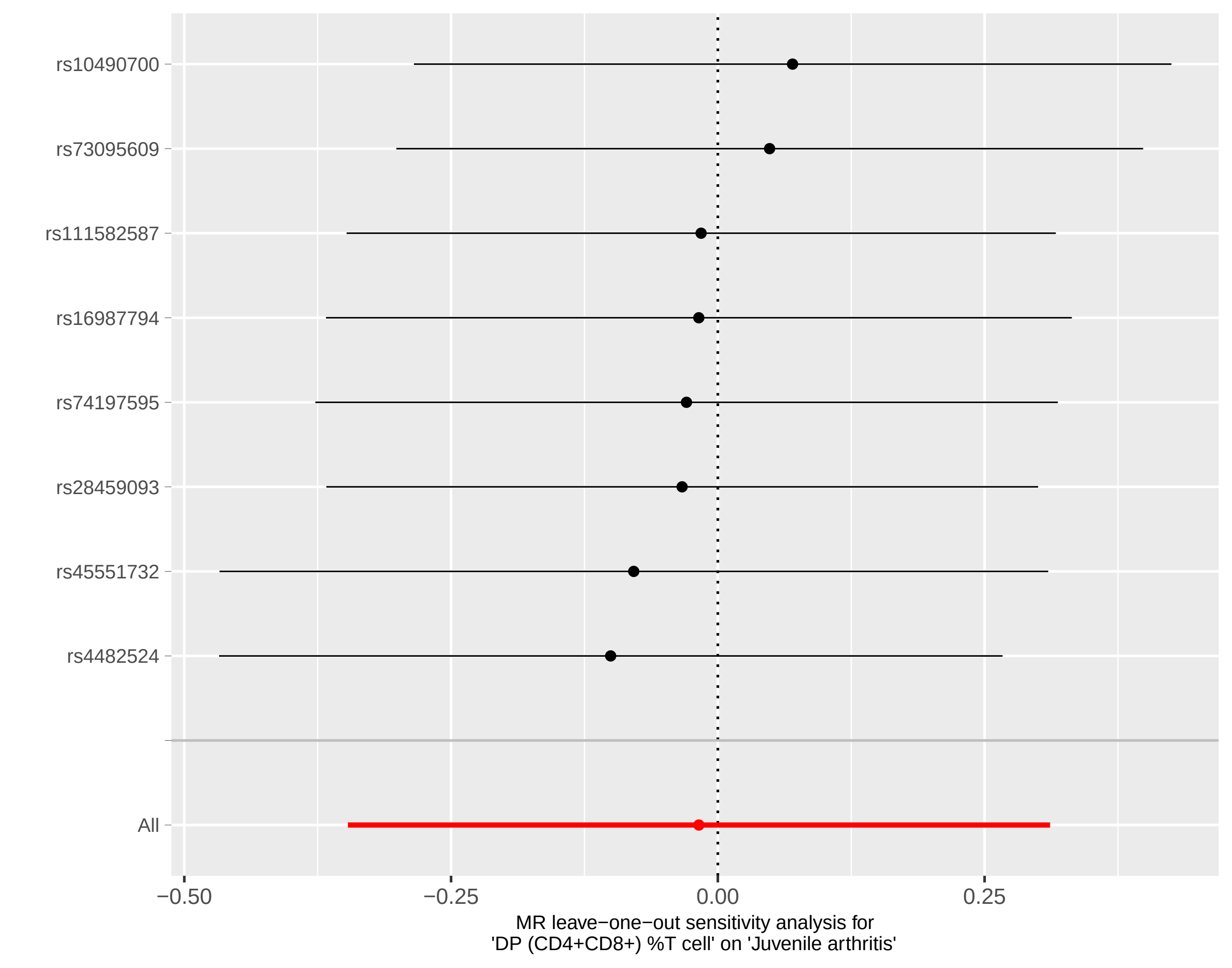
rs10490700
rs73095609
rs111582587
rs16987794
rs74197595
rs28459093
rs45551732
rs4482524
All
−0.50
−0.25
0.00
0.25
MR leave−one−out sensitivity analysis for
'DP (CD4+CD8+) %T cell' on 'Juvenile arthritis'

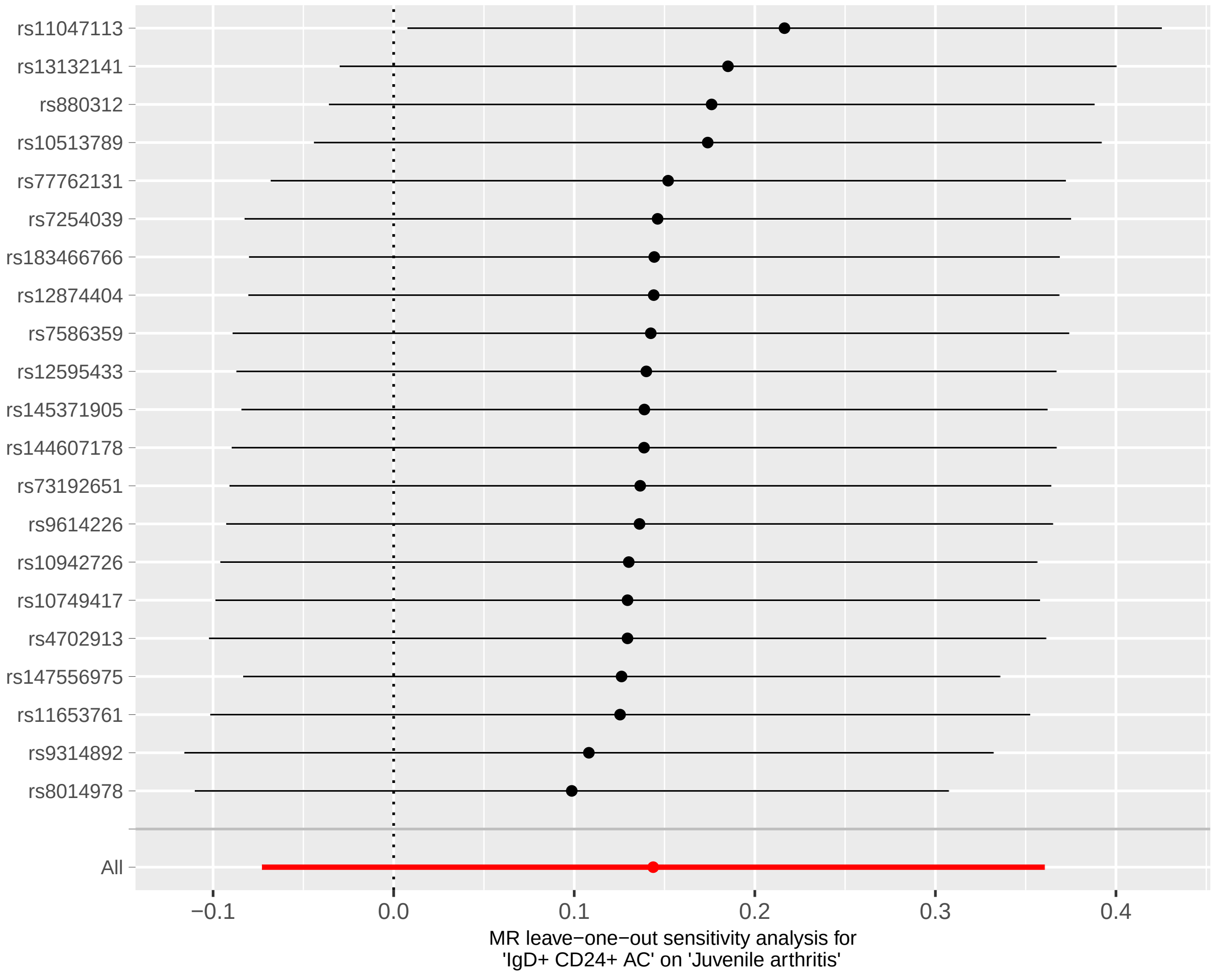
rs11047113
rs13132141
rs880312
rs10513789
rs77762131
rs7254039
rs183466766
rs12874404
rs7586359
rs12595433
rs145371905
rs144607178
rs73192651
rs9614226
rs10942726
rs10749417
rs4702913
rs147556975
rs11653761
rs9314892
rs8014978
All
−0.1
0.0
0.1
0.2
0.3
0.4
MR leave−one−out sensitivity analysis for
'IgD+ CD24+ AC' on 'Juvenile arthritis'

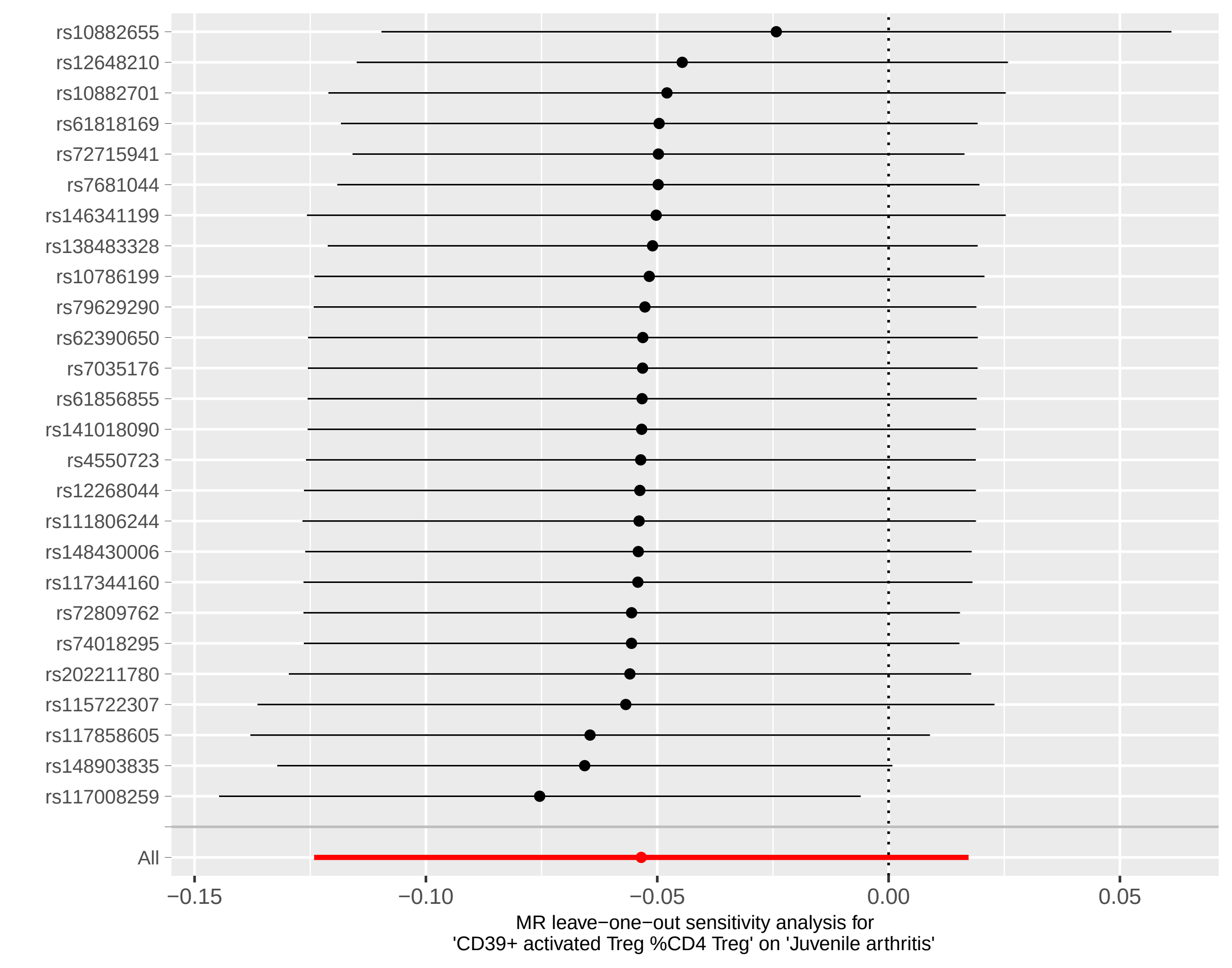
rs10882655
rs12648210
rs10882701
rs61818169
rs72715941
rs7681044
rs146341199
rs138483328
rs10786199
rs79629290
rs62390650
rs7035176
rs61856855
rs141018090
rs4550723
rs12268044
rs111806244
rs148430006
rs117344160
rs72809762
rs74018295
rs202211780
rs115722307
rs117858605
rs148903835
rs117008259
All
−0.15
−0.10
−0.05
0.00
0.05
MR leave−one−out sensitivity analysis for
'CD39+ activated Treg %CD4 Treg' on 'Juvenile arthritis'

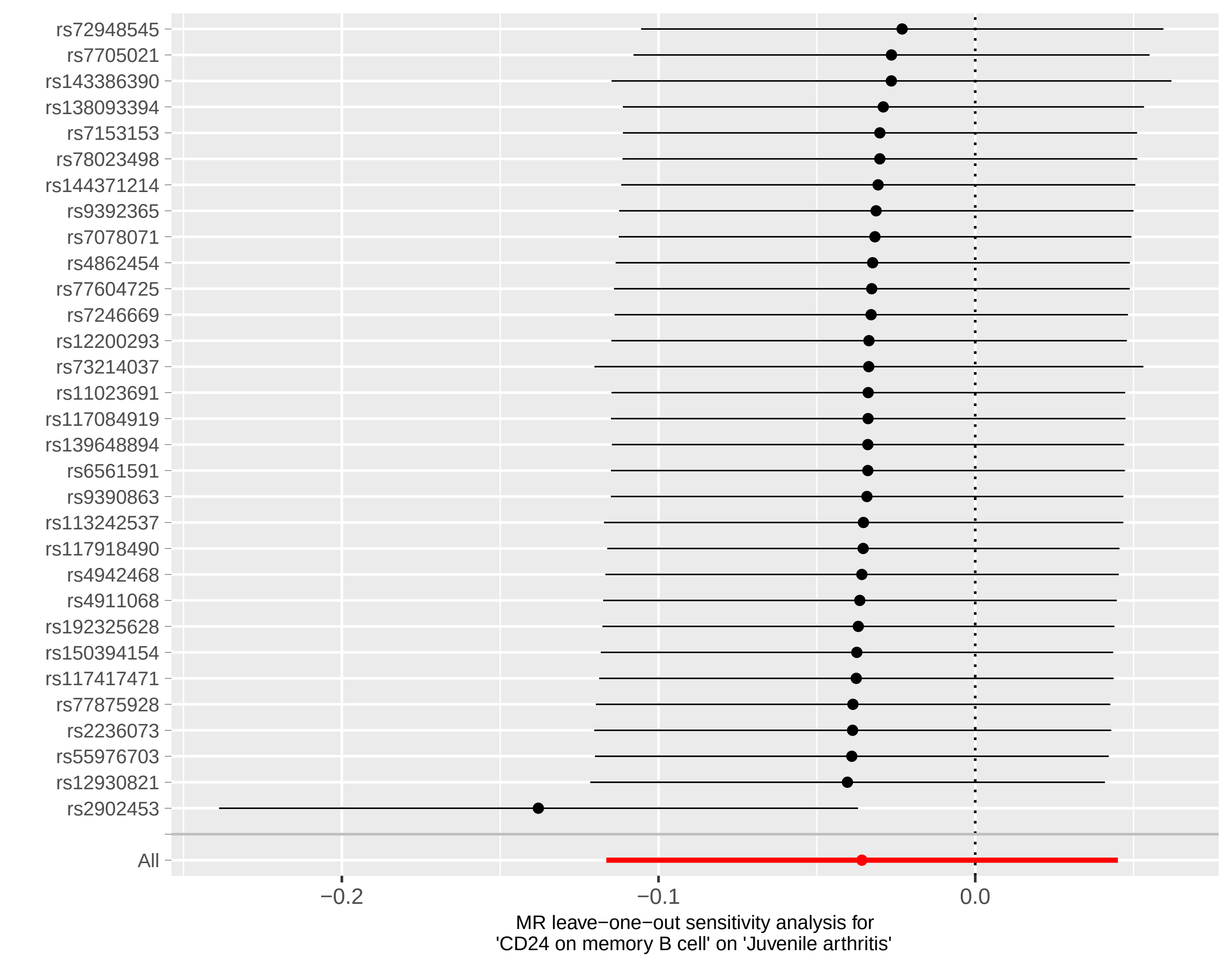

rs72948545
rs7705021
rs143386390
rs138093394
rs7153153
rs78023498
rs144371214
rs9392365
rs7078071
rs4862454
rs77604725
rs7246669
rs12200293
rs73214037
rs11023691
rs117084919
rs139648894
rs6561591
rs9390863
rs113242537
rs117918490
rs4942468
rs4911068
rs192325628
rs150394154
rs117417471
rs77875928
rs2236073
rs55976703
rs12930821
rs2902453
All
−0.2
−0.1
0.0
MR leave−one−out sensitivity analysis for
'CD24 on memory B cell' on 'Juvenile arthritis'

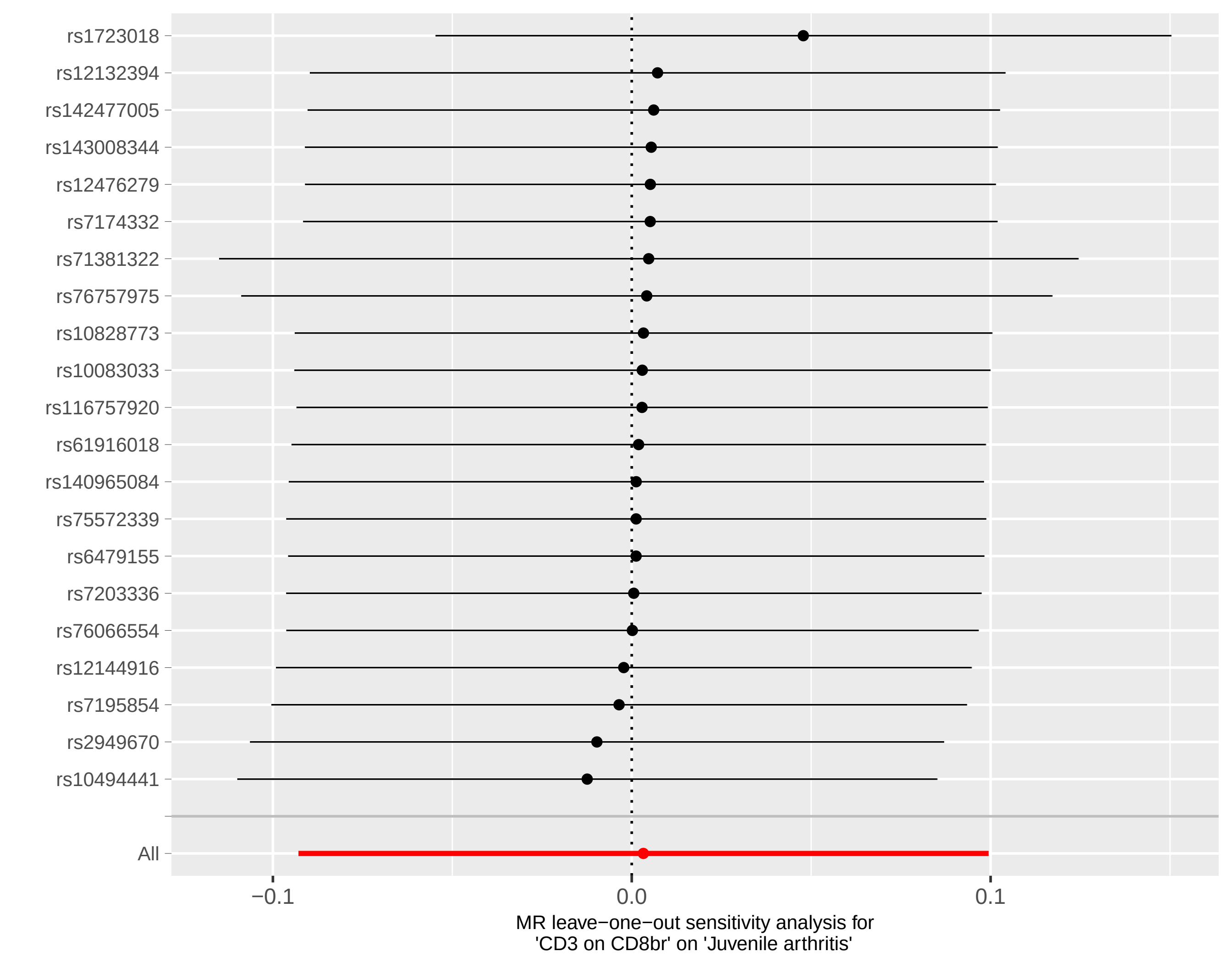
rs1723018
rs12132394
rs142477005
rs143008344
rs12476279
rs7174332
rs71381322
rs76757975
rs10828773
rs10083033
rs116757920
rs61916018
rs140965084
rs75572339
rs6479155
rs7203336
rs76066554
rs12144916
rs7195854
rs2949670
rs10494441
All
−0.1
0.0
0.1
MR leave−one−out sensitivity analysis for
'CD3 on CD8br' on 'Juvenile arthritis'

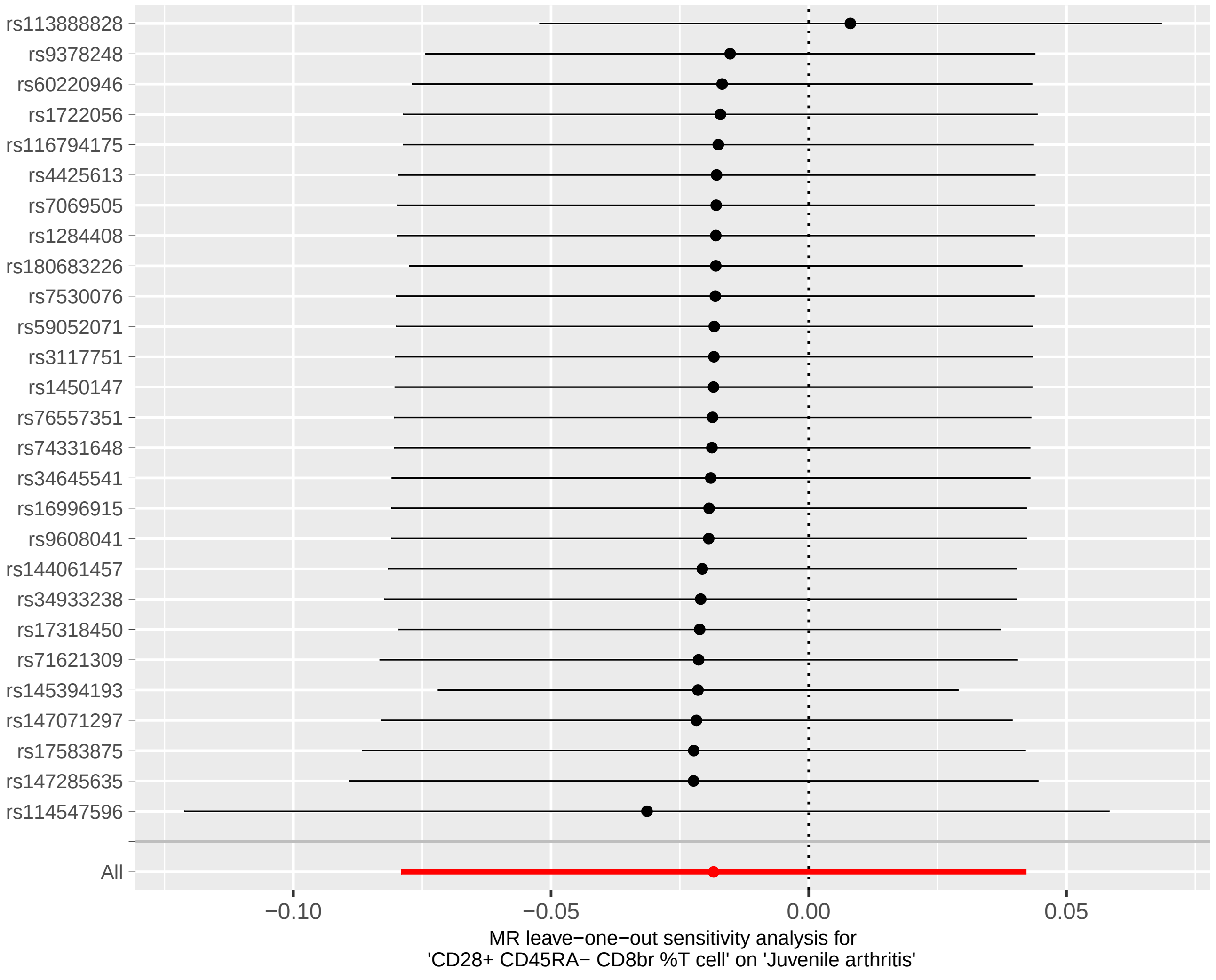

rs113888828
rs9378248
rs60220946
rs1722056
rs116794175
rs4425613
rs7069505
rs1284408
rs180683226
rs7530076
rs59052071
rs3117751
rs1450147
rs76557351
rs74331648
rs34645541
rs16996915
rs9608041
rs144061457
rs34933238
rs17318450
rs71621309
rs145394193
rs147071297
rs17583875
rs147285635
rs114547596
All
−0.10
−0.05
0.00
0.05
MR leave−one−out sensitivity analysis for
'CD28+ CD45RA− CD8br %T cell' on 'Juvenile arthritis'

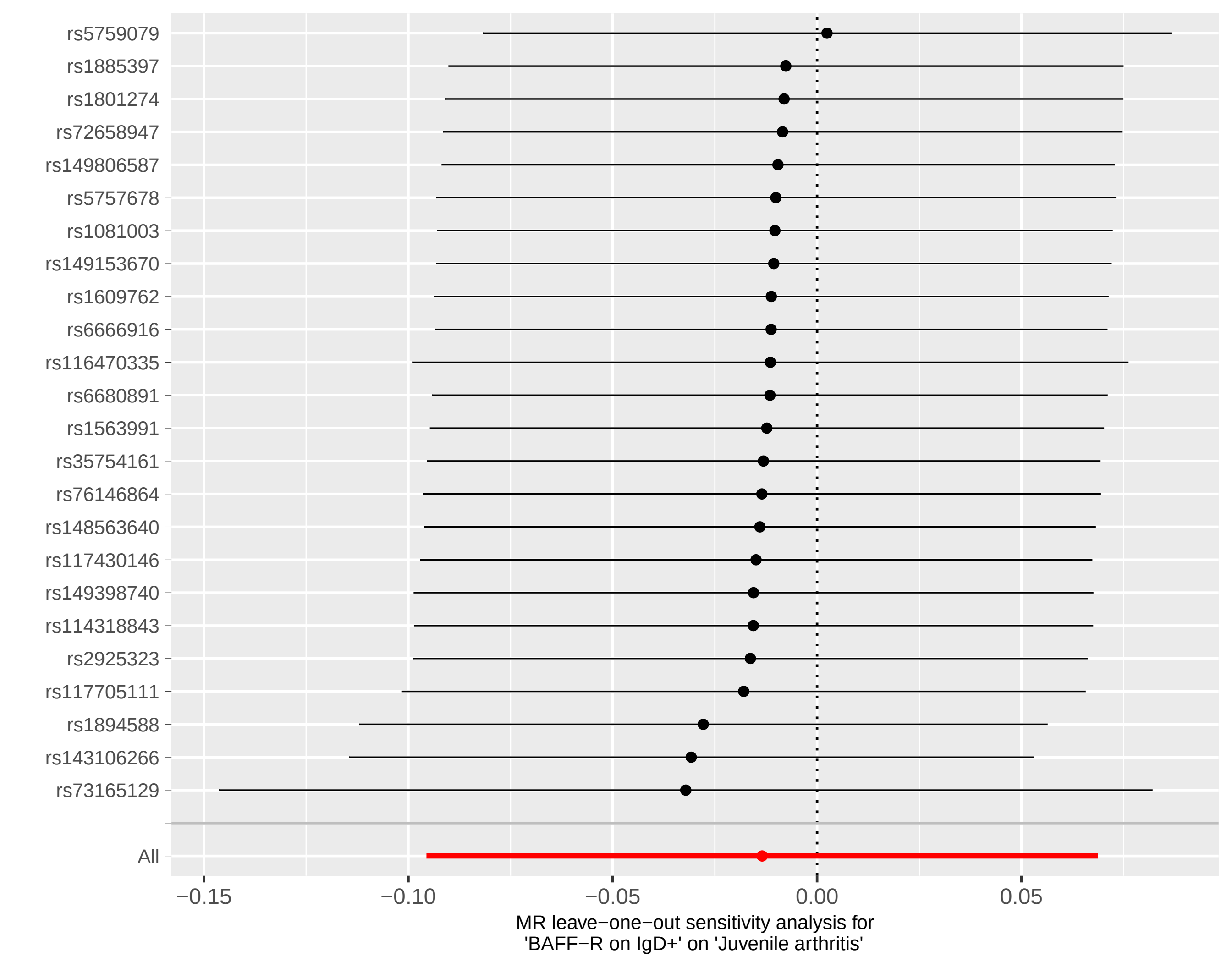

rs5759079
rs1885397
rs1801274
rs72658947
rs149806587
rs5757678
rs1081003
rs149153670
rs1609762
rs6666916
rs116470335
rs6680891
rs1563991
rs35754161
rs76146864
rs148563640
rs117430146
rs149398740
rs114318843
rs2925323
rs117705111
rs1894588
rs143106266
rs73165129
All
−0.15
−0.10
−0.05
0.00
0.05
MR leave−one−out sensitivity analysis for
'BAFF−R on IgD+' on 'Juvenile arthritis'

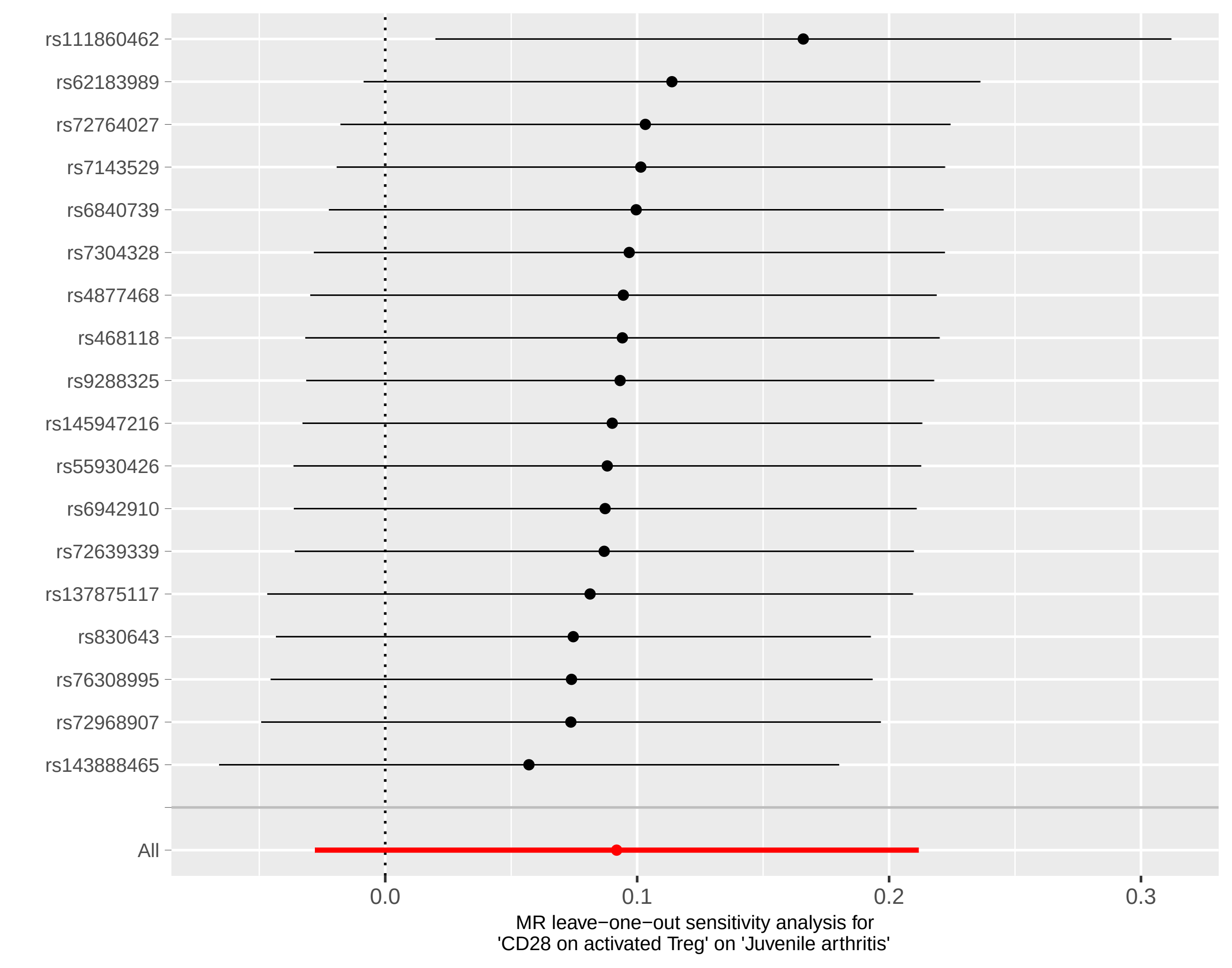
rs111860462
rs62183989
rs72764027
rs7143529
rs6840739
rs7304328
rs4877468
rs468118
rs9288325
rs145947216
rs55930426
rs6942910
rs72639339
rs137875117
rs830643
rs76308995
rs72968907
rs143888465
All
0.0
0.1
0.2
0.3
MR leave−one−out sensitivity analysis for
'CD28 on activated Treg' on 'Juvenile arthritis'

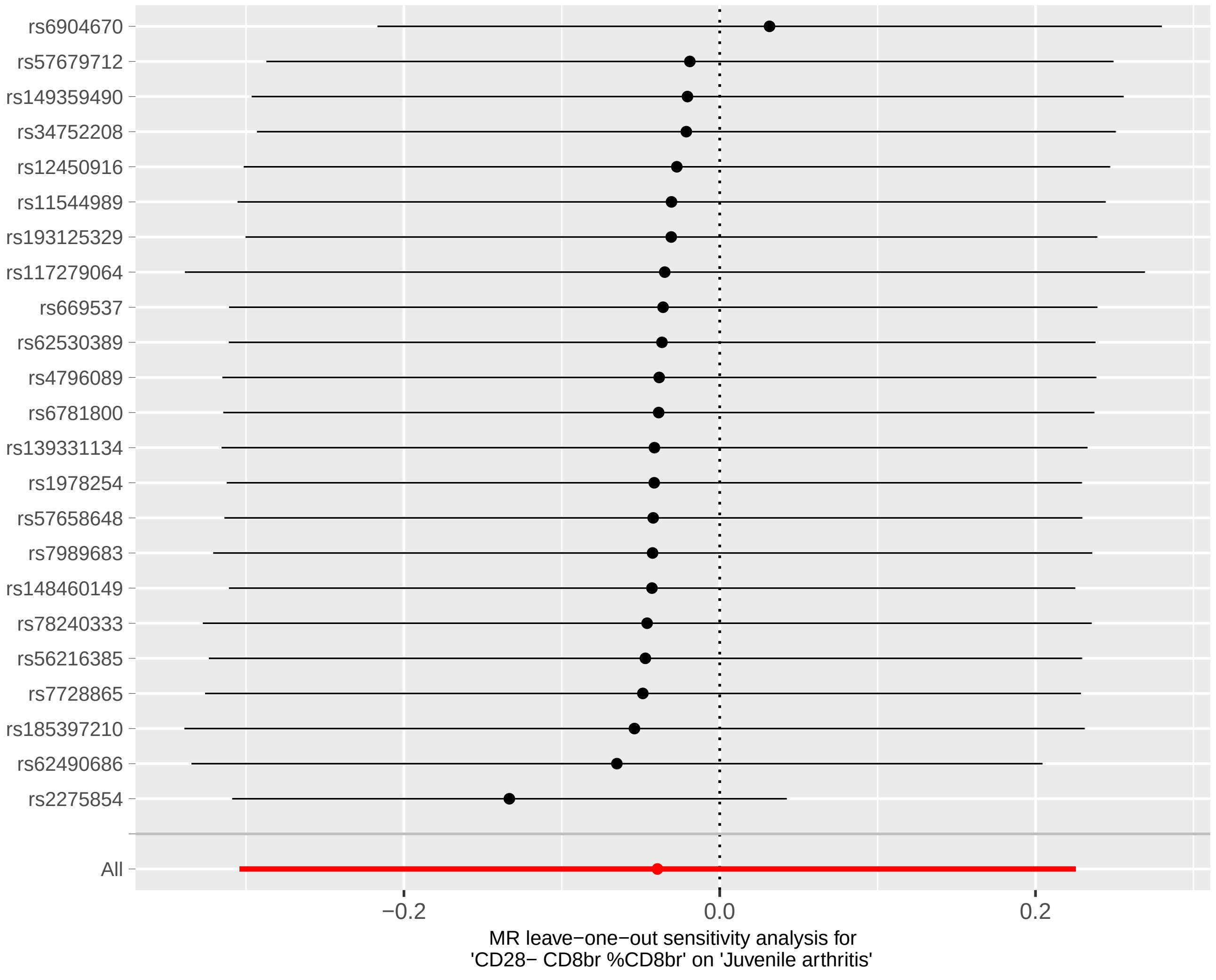
rs6904670
rs57679712
rs149359490
rs34752208
rs12450916
rs11544989
rs193125329
rs117279064
rs669537
rs62530389
rs4796089
rs6781800
rs139331134
rs1978254
rs57658648
rs7989683
rs148460149
rs78240333
rs56216385
rs7728865
rs185397210
rs62490686
rs2275854
All
−0.2
0.0
0.2
MR leave−one−out sensitivity analysis for
'CD28− CD8br %CD8br' on 'Juvenile arthritis'

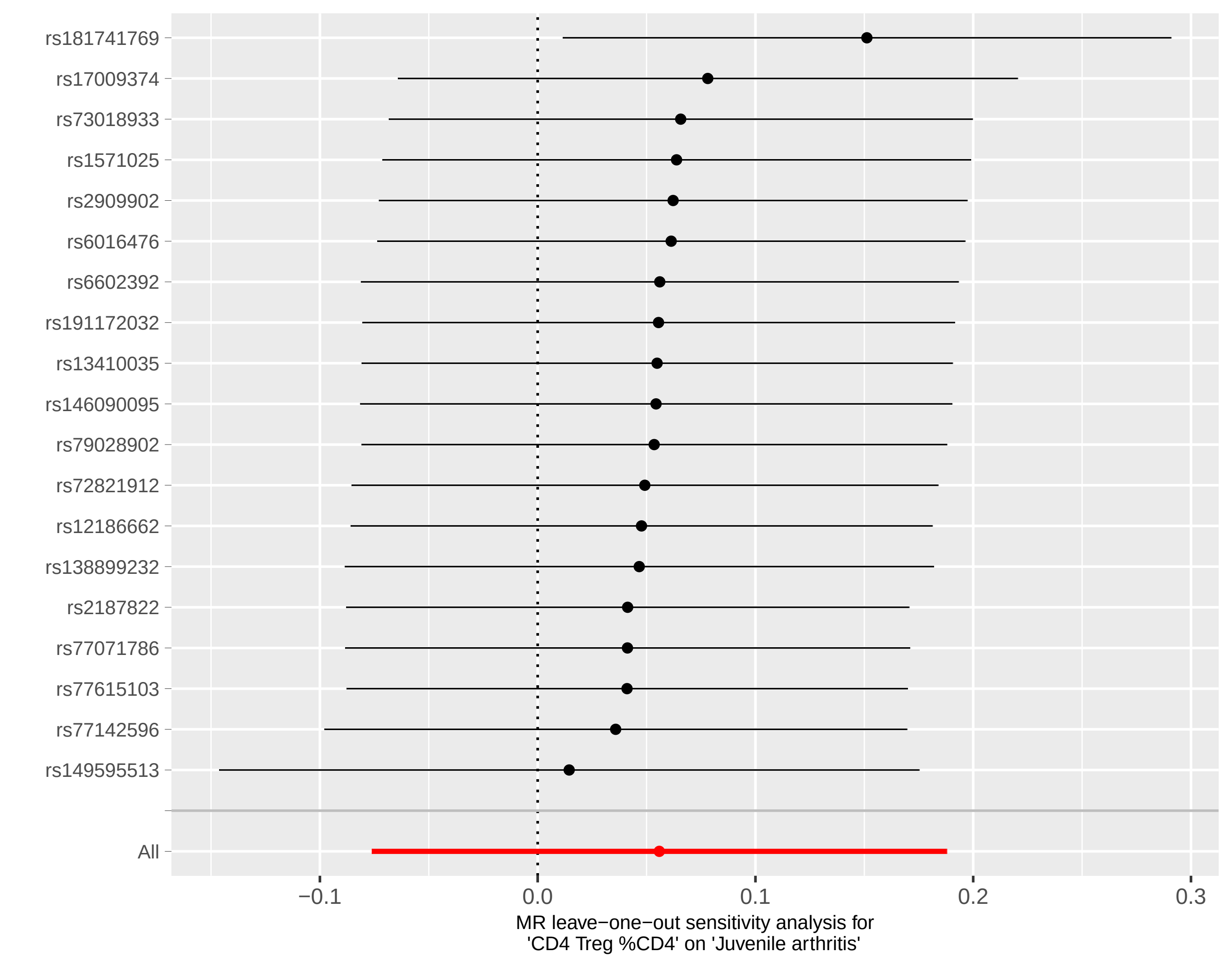
rs181741769
rs17009374
rs73018933
rs1571025
rs2909902
rs6016476
rs6602392
rs191172032
rs13410035
rs146090095
rs79028902
rs72821912
rs12186662
rs138899232
rs2187822
rs77071786
rs77615103
rs77142596
rs149595513
All
−0.1
0.0
0.1
0.2
0.3
MR leave−one−out sensitivity analysis for
'CD4 Treg %CD4' on 'Juvenile arthritis'

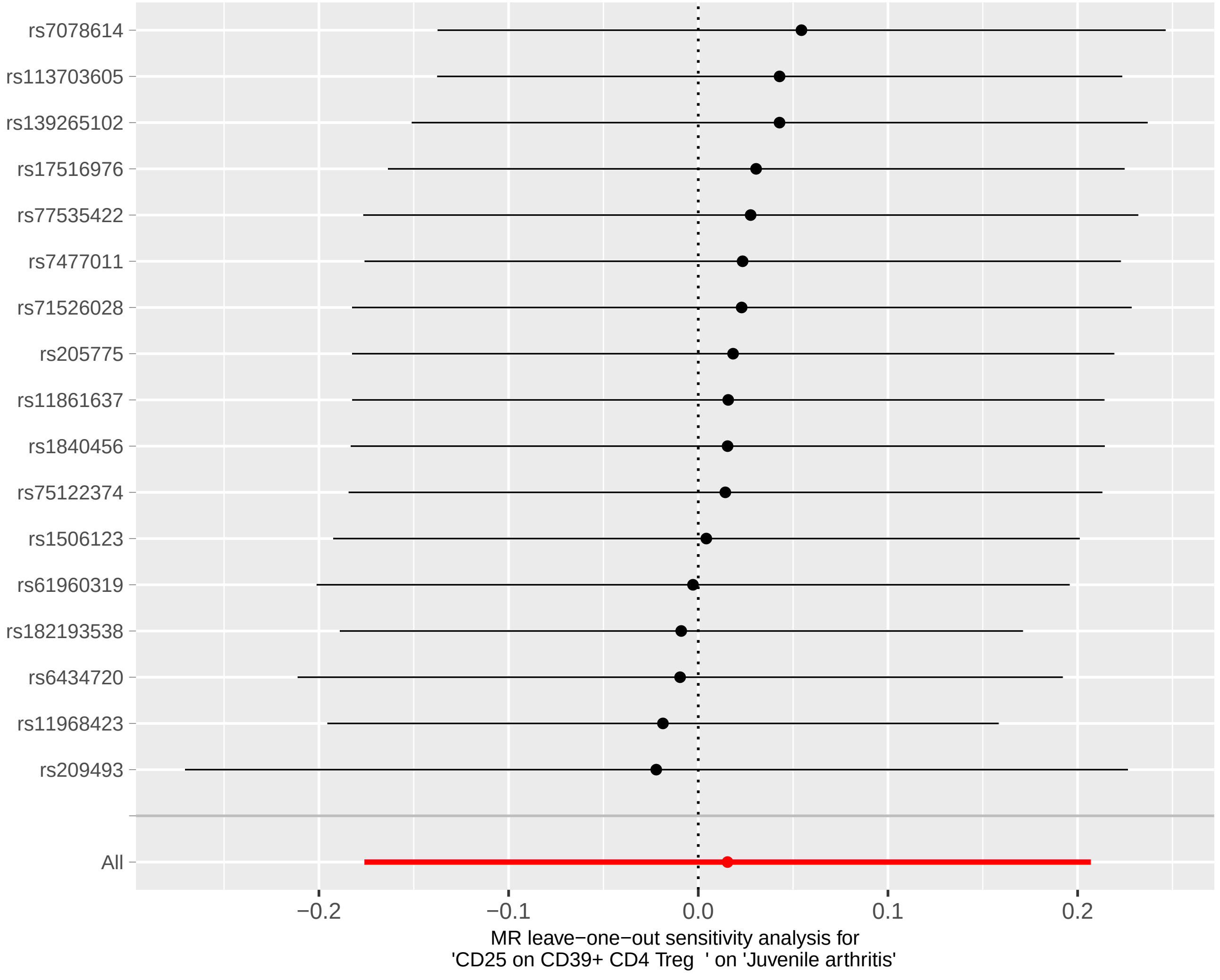
rs7078614
rs113703605
rs139265102
rs17516976
rs77535422
rs7477011
rs71526028
rs205775
rs11861637
rs1840456
rs75122374
rs1506123
rs61960319
rs182193538
rs6434720
rs11968423
rs209493
All
−0.2
−0.1
0.0
0.1
0.2
MR leave−one−out sensitivity analysis for
'CD25 on CD39+ CD4 Treg ' on 'Juvenile arthritis'

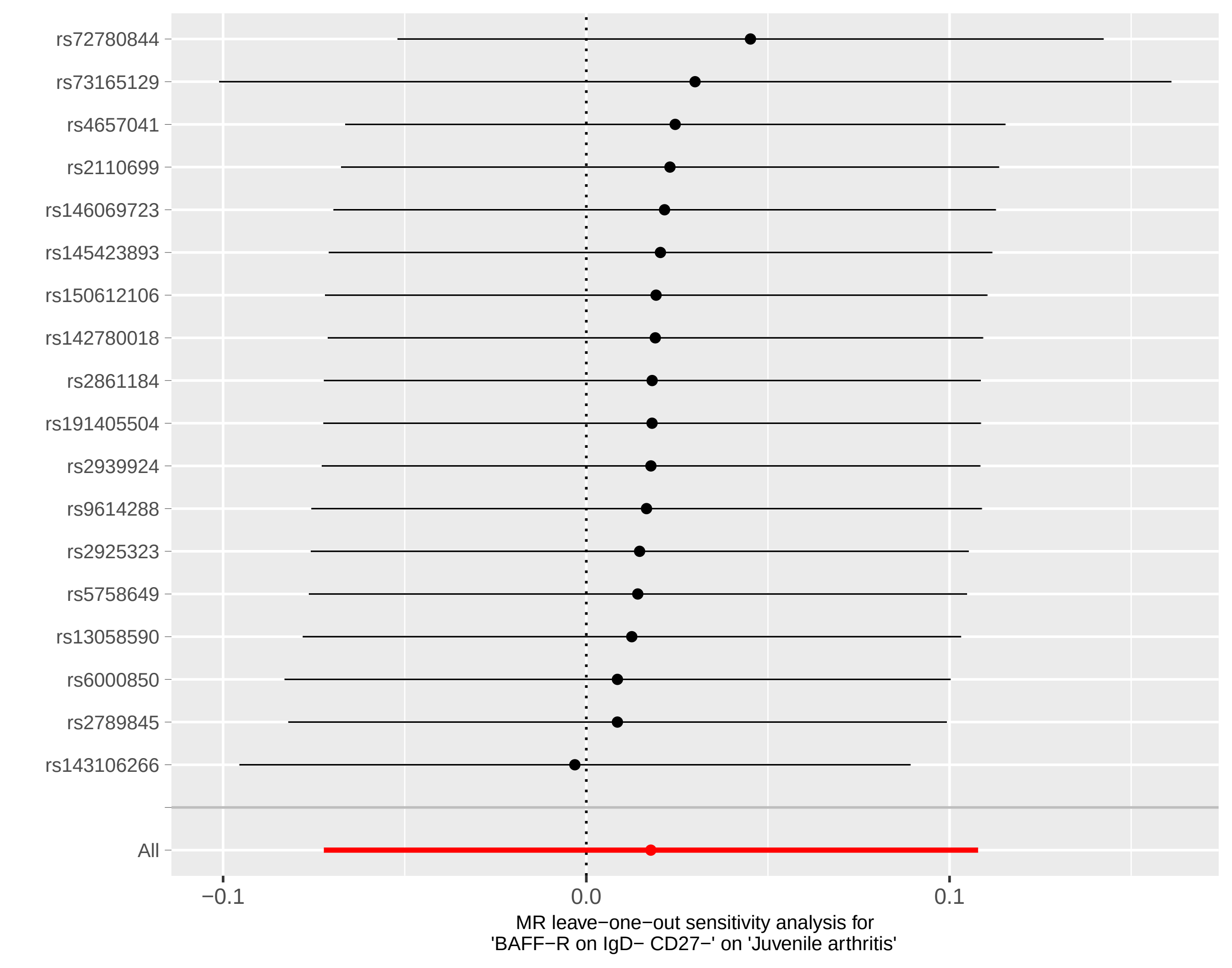
rs72780844
rs73165129
rs4657041
rs2110699
rs146069723
rs145423893
rs150612106
rs142780018
rs2861184
rs191405504
rs2939924
rs9614288
rs2925323
rs5758649
rs13058590
rs6000850
rs2789845
rs143106266
All
−0.1
0.0
0.1
MR leave−one−out sensitivity analysis for
'BAFF−R on IgD− CD27−' on 'Juvenile arthritis'

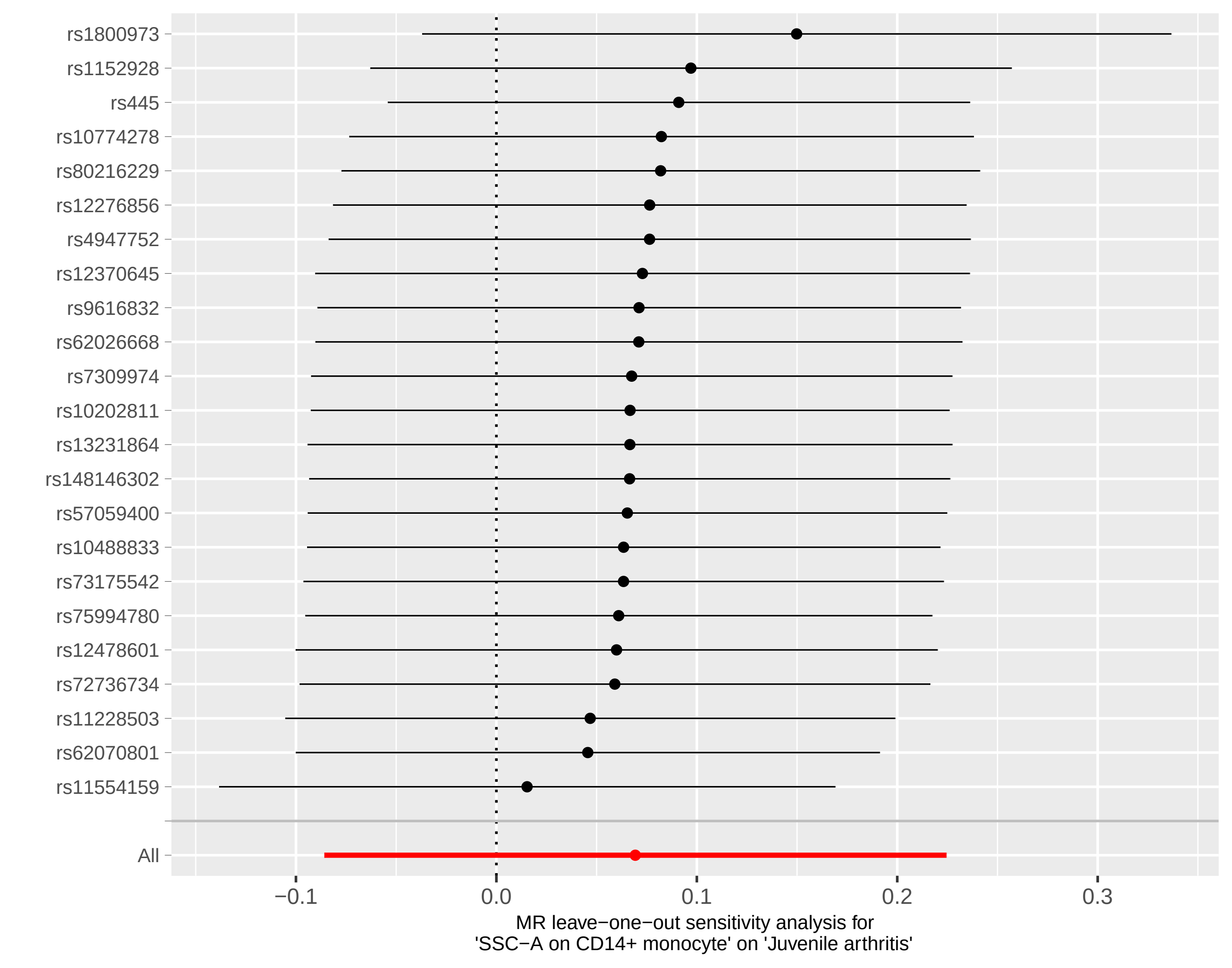
rs1800973
rs1152928
rs445
rs10774278
rs80216229
rs12276856
rs4947752
rs12370645
rs9616832
rs62026668
rs7309974
rs10202811
rs13231864
rs148146302
rs57059400
rs10488833
rs73175542
rs75994780
rs12478601
rs72736734
rs11228503
rs62070801
rs11554159
All
−0.1
0.0
0.1
0.2
0.3
MR leave−one−out sensitivity analysis for
'SSC−A on CD14+ monocyte' on 'Juvenile arthritis'

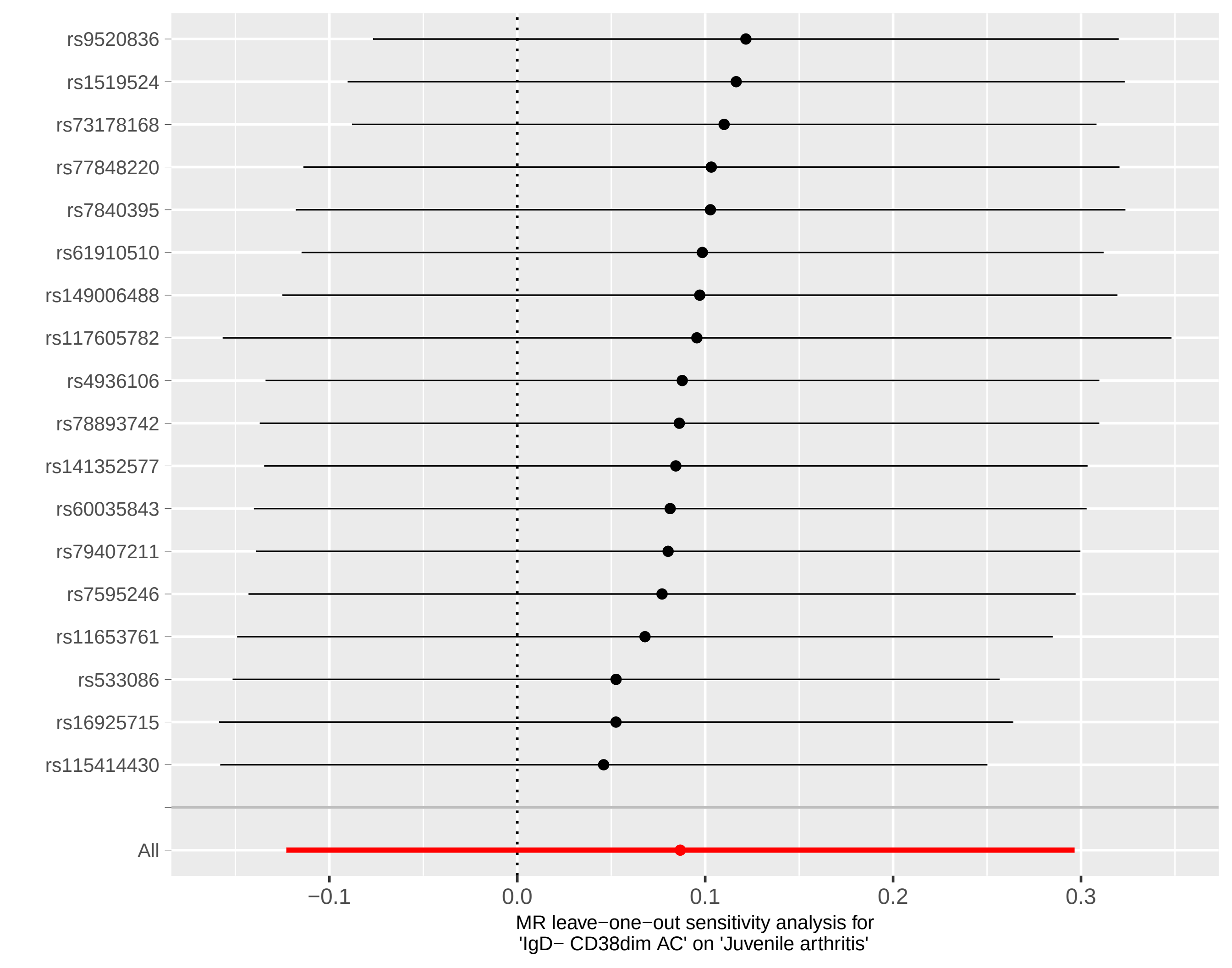
rs9520836
rs1519524
rs73178168
rs77848220
rs7840395
rs61910510
rs149006488
rs117605782
rs4936106
rs78893742
rs141352577
rs60035843
rs79407211
rs7595246
rs11653761
rs533086
rs16925715
rs115414430
All
−0.1
0.0
0.1
0.2
0.3
MR leave−one−out sensitivity analysis for
'IgD− CD38dim AC' on 'Juvenile arthritis'

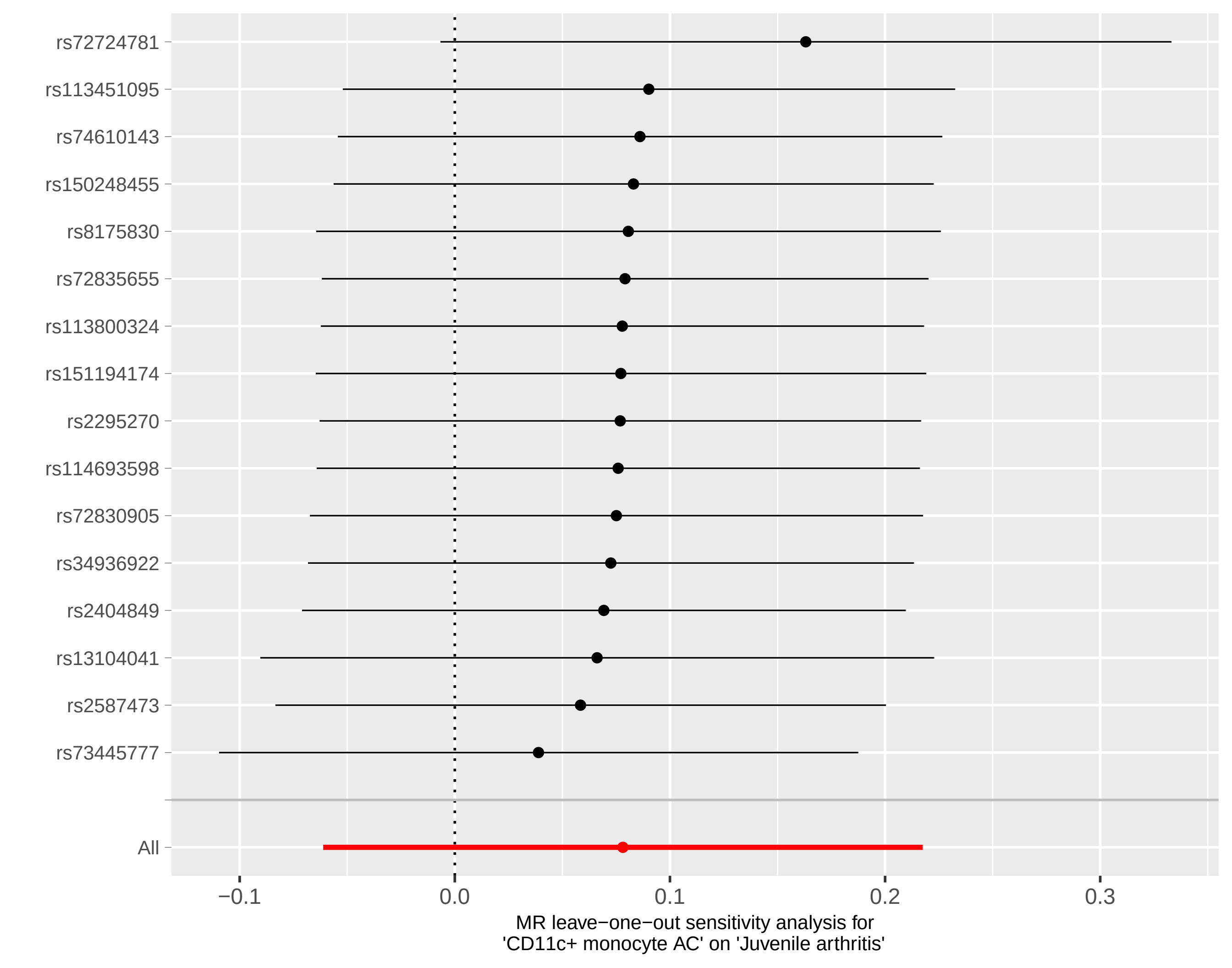
rs72724781
rs113451095
rs74610143
rs150248455
rs8175830
rs72835655
rs113800324
rs151194174
rs2295270
rs114693598
rs72830905
rs34936922
rs2404849
rs13104041
rs2587473
rs73445777
All
−0.1
0.0
0.1
0.2
0.3
MR leave−one−out sensitivity analysis for
'CD11c+ monocyte AC' on 'Juvenile arthritis'

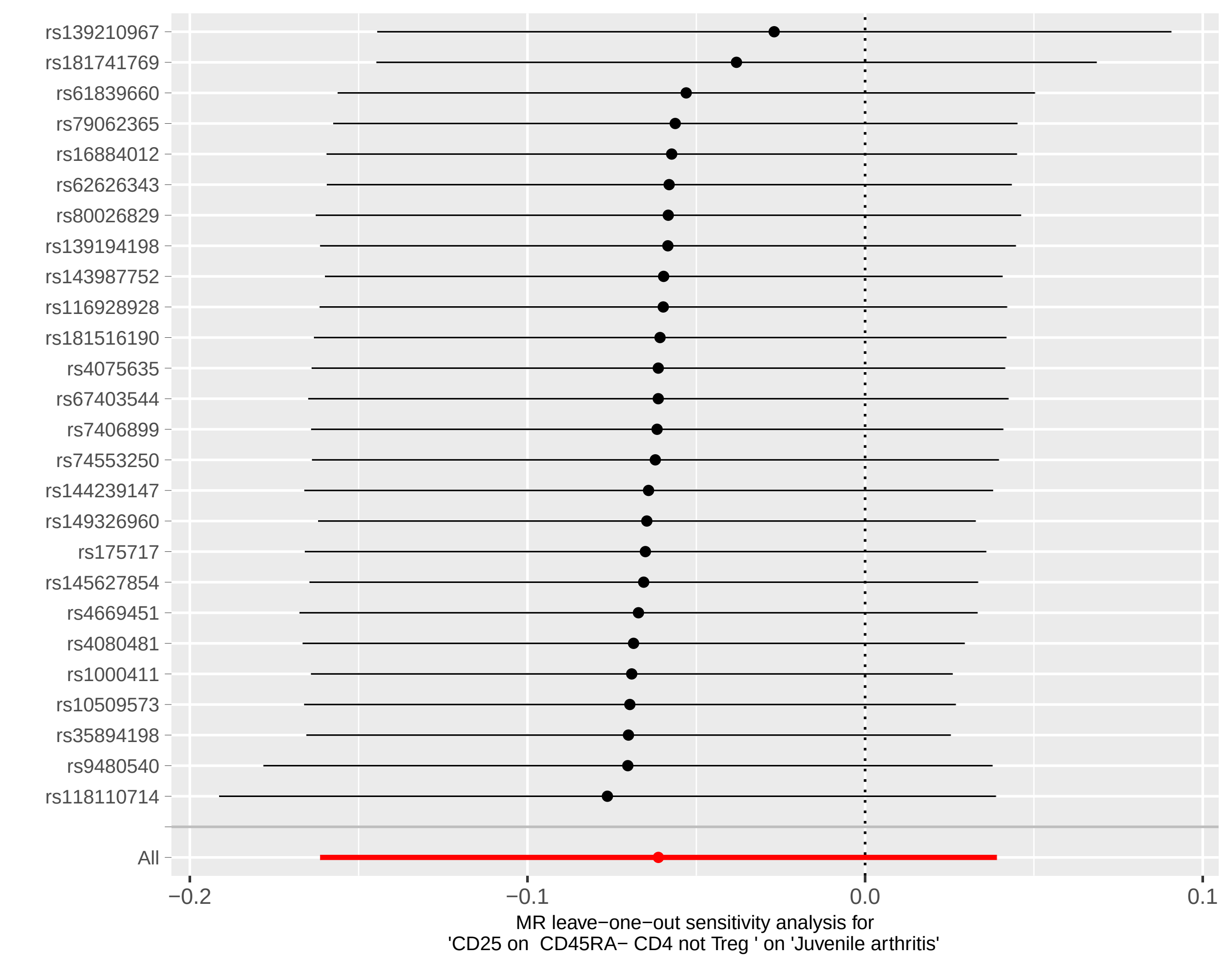

rs139210967
rs181741769
rs61839660
rs79062365
rs16884012
rs62626343
rs80026829
rs139194198
rs143987752
rs116928928
rs181516190
rs4075635
rs67403544
rs7406899
rs74553250
rs144239147
rs149326960
rs175717
rs145627854
rs4669451
rs4080481
rs1000411
rs10509573
rs35894198
rs9480540
rs118110714
All
−0.2
−0.1
0.0
0.1
MR leave−one−out sensitivity analysis for
'CD25 on CD45RA− CD4 not Treg ' on 'Juvenile arthritis'

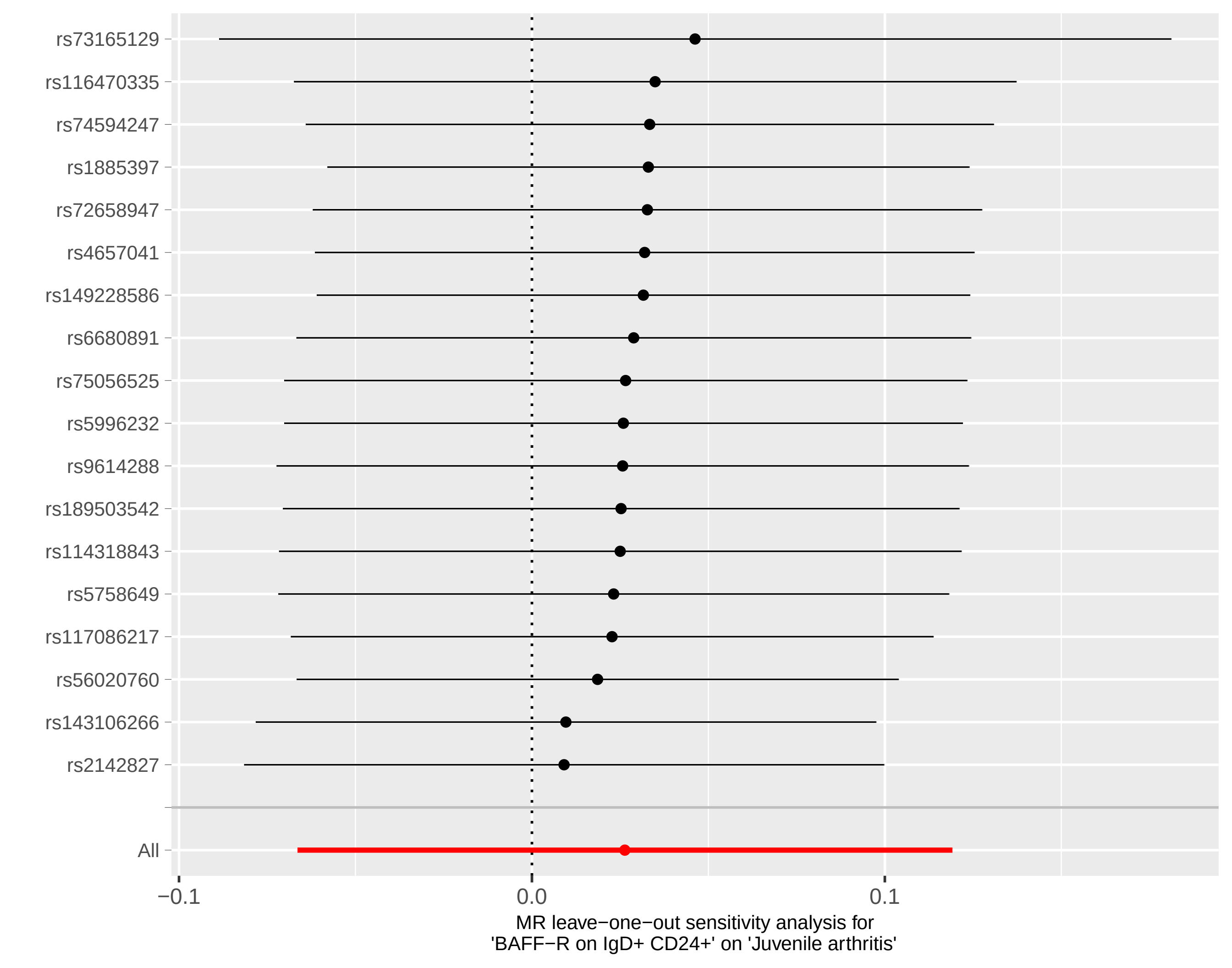

rs73165129
rs116470335
rs74594247
rs1885397
rs72658947
rs4657041
rs149228586
rs6680891
rs75056525
rs5996232
rs9614288
rs189503542
rs114318843
rs5758649
rs117086217
rs56020760
rs143106266
rs2142827
All
−0.1
0.0
0.1
MR leave−one−out sensitivity analysis for
'BAFF−R on IgD+ CD24+' on 'Juvenile arthritis'

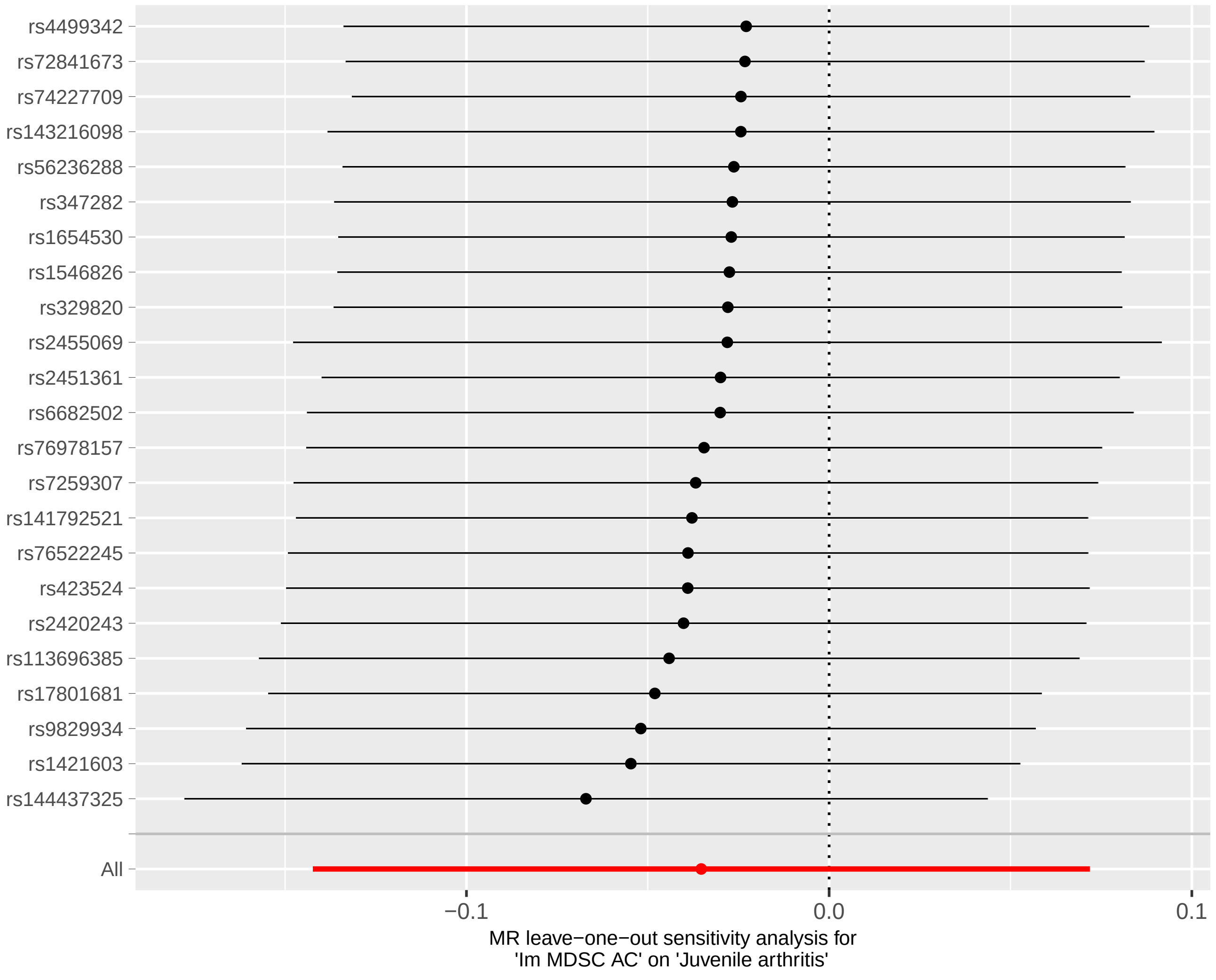
rs4499342
rs72841673
rs74227709
rs143216098
rs56236288
rs347282
rs1654530
rs1546826
rs329820
rs2455069
rs2451361
rs6682502
rs76978157
rs7259307
rs141792521
rs76522245
rs423524
rs2420243
rs113696385
rs17801681
rs9829934
rs1421603
rs144437325
All
−0.1
0.0
0.1
MR leave−one−out sensitivity analysis for
'Im MDSC AC' on 'Juvenile arthritis'

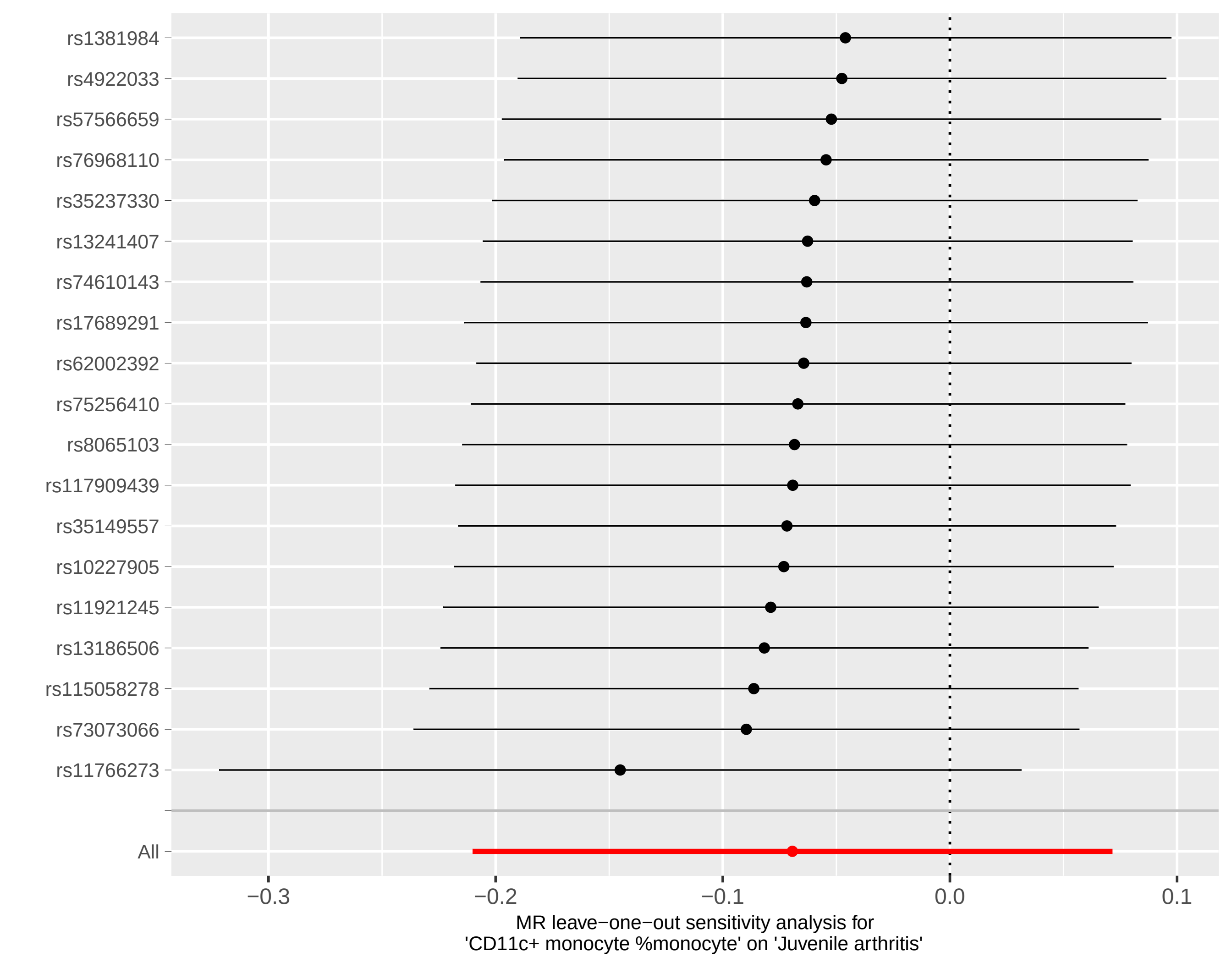
rs1381984
rs4922033
rs57566659
rs76968110
rs35237330
rs13241407
rs74610143
rs17689291
rs62002392
rs75256410
rs8065103
rs117909439
rs35149557
rs10227905
rs11921245
rs13186506
rs115058278
rs73073066
rs11766273
All
−0.3
−0.2
−0.1
0.0
0.1
MR leave−one−out sensitivity analysis for
'CD11c+ monocyte %monocyte' on 'Juvenile arthritis'

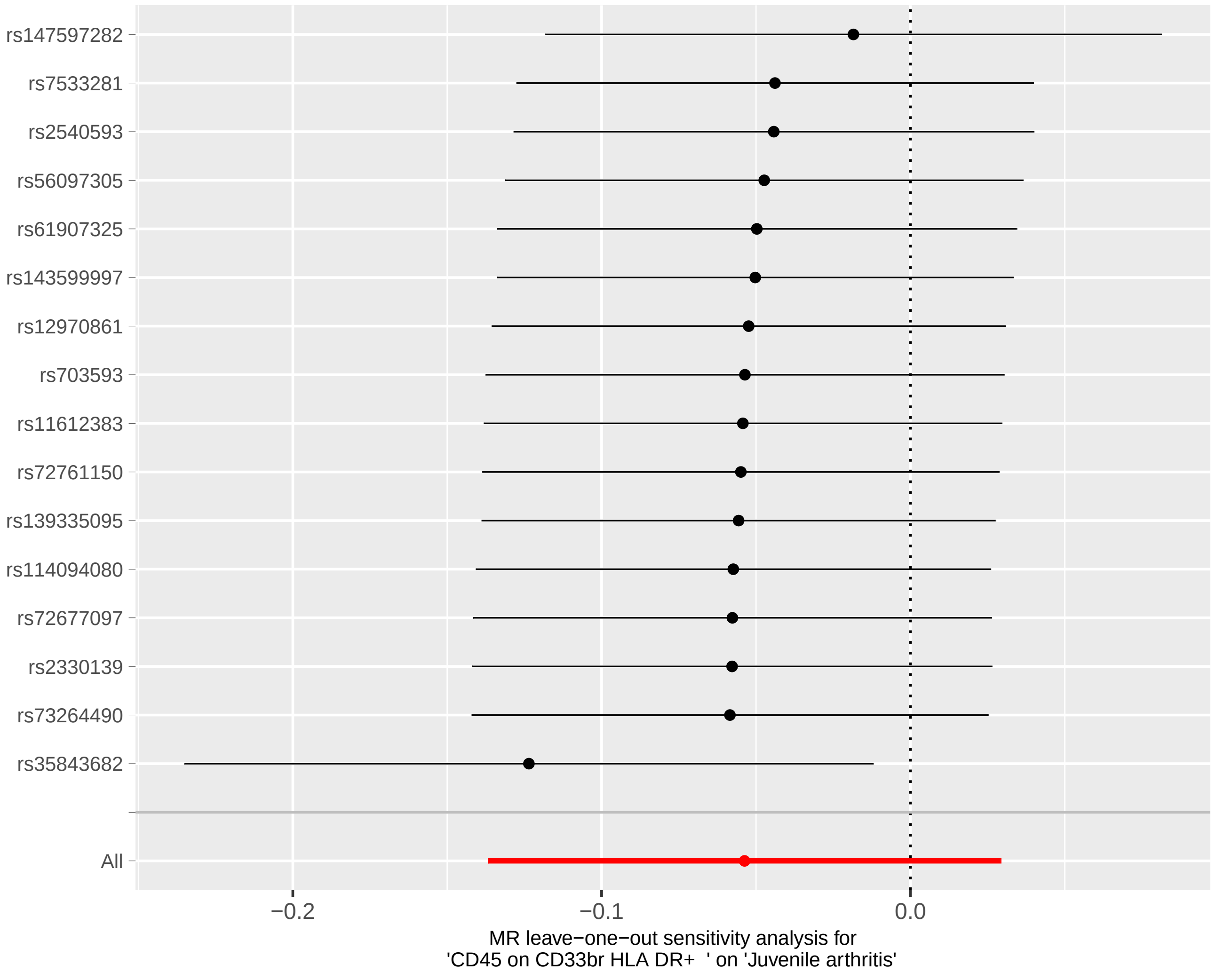
rs147597282
rs7533281
rs2540593
rs56097305
rs61907325
rs143599997
rs12970861
rs703593
rs11612383
rs72761150
rs139335095
rs114094080
rs72677097
rs2330139
rs73264490
rs35843682
All
−0.2
−0.1
0.0
MR leave−one−out sensitivity analysis for
'CD45 on CD33br HLA DR+ ' on 'Juvenile arthritis'

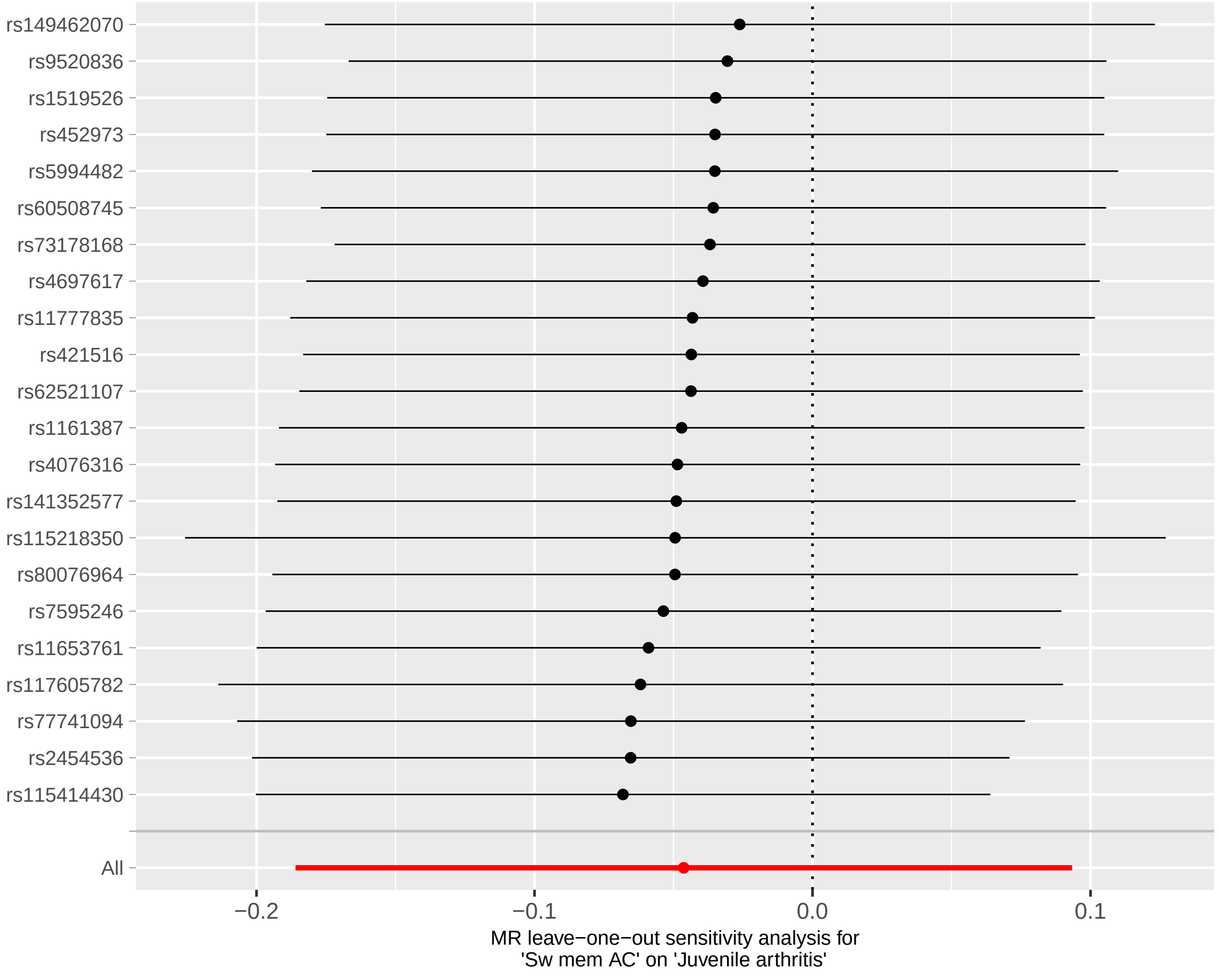
rs149462070
rs9520836
rs1519526
rs452973
rs5994482
rs60508745
rs73178168
rs4697617
rs11777835
rs421516
rs62521107
rs1161387
rs4076316
rs141352577
rs115218350
rs80076964
rs7595246
rs11653761
rs117605782
rs77741094
rs2454536
rs115414430
All
−0.2
−0.1
0.0
0.1
MR leave−one−out sensitivity analysis for
'Sw mem AC' on 'Juvenile arthritis'

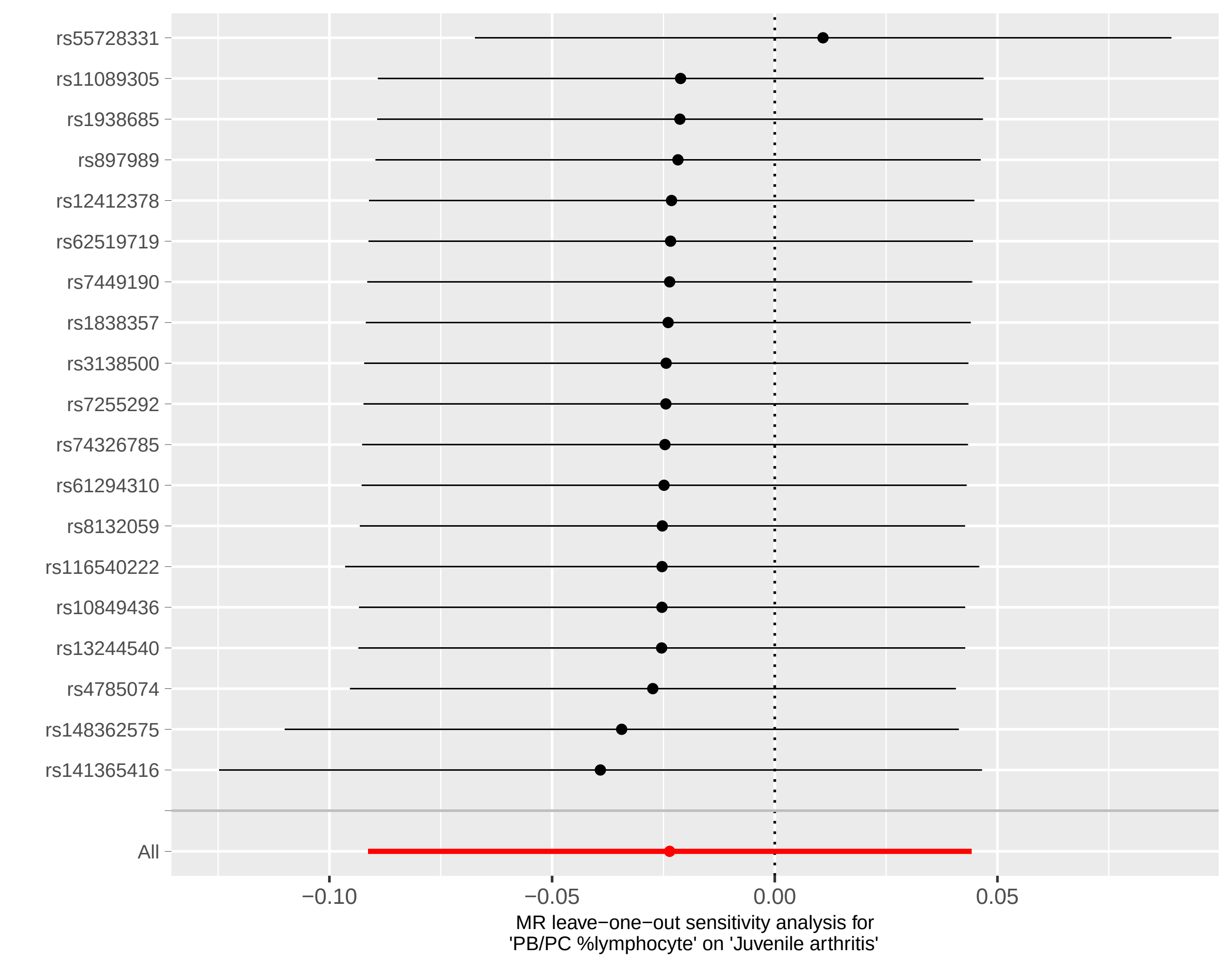
rs55728331
rs11089305
rs1938685
rs897989
rs12412378
rs62519719
rs7449190
rs1838357
rs3138500
rs7255292
rs74326785
rs61294310
rs8132059
rs116540222
rs10849436
rs13244540
rs4785074
rs148362575
rs141365416
All
−0.10
−0.05
0.00
0.05
MR leave−one−out sensitivity analysis for
'PB/PC %lymphocyte' on 'Juvenile arthritis'

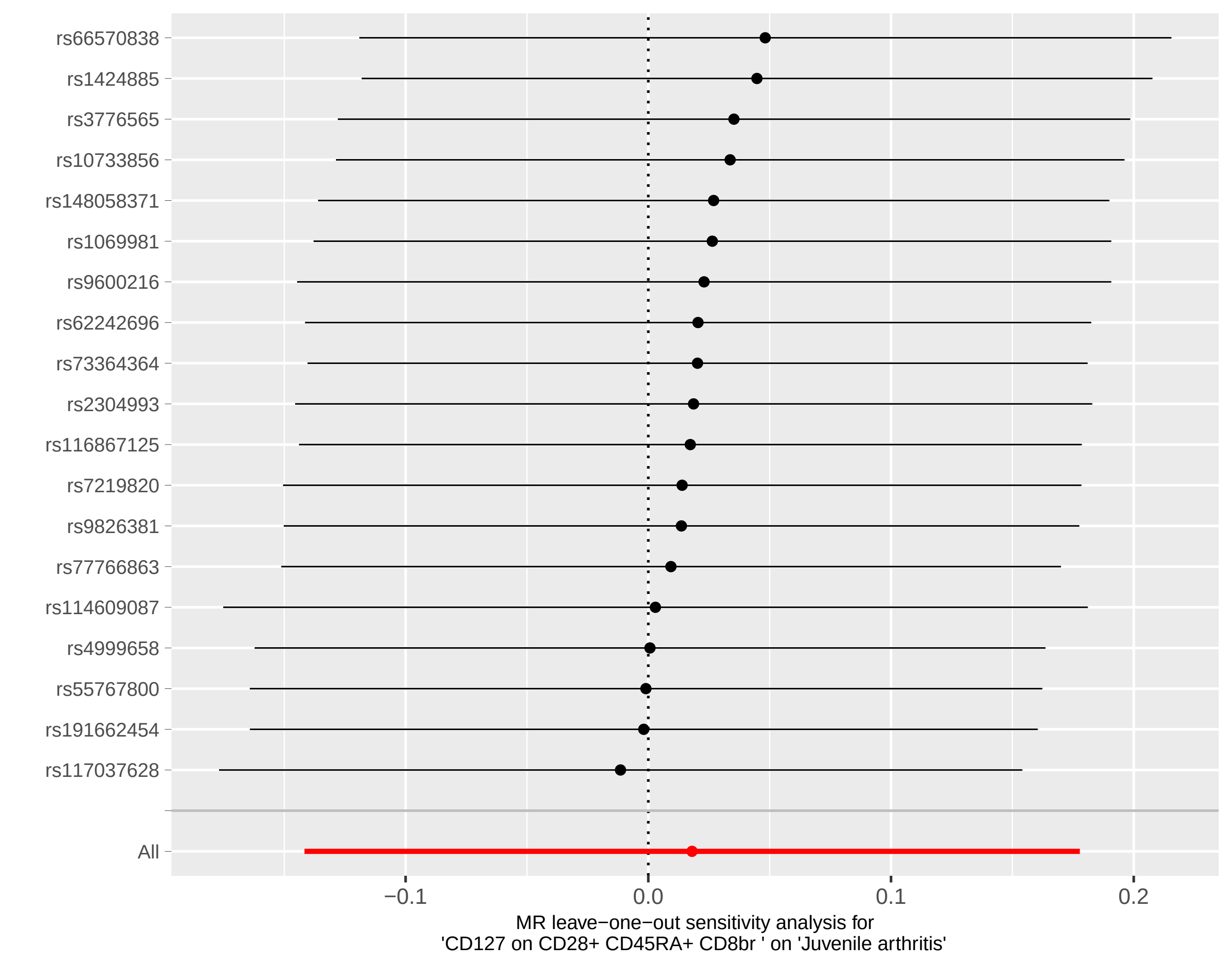
rs66570838
rs1424885
rs3776565
rs10733856
rs148058371
rs1069981
rs9600216
rs62242696
rs73364364
rs2304993
rs116867125
rs7219820
rs9826381
rs77766863
rs114609087
rs4999658
rs55767800
rs191662454
rs117037628
All
−0.1
0.0
0.1
0.2
MR leave−one−out sensitivity analysis for
'CD127 on CD28+ CD45RA+ CD8br ' on 'Juvenile arthritis'

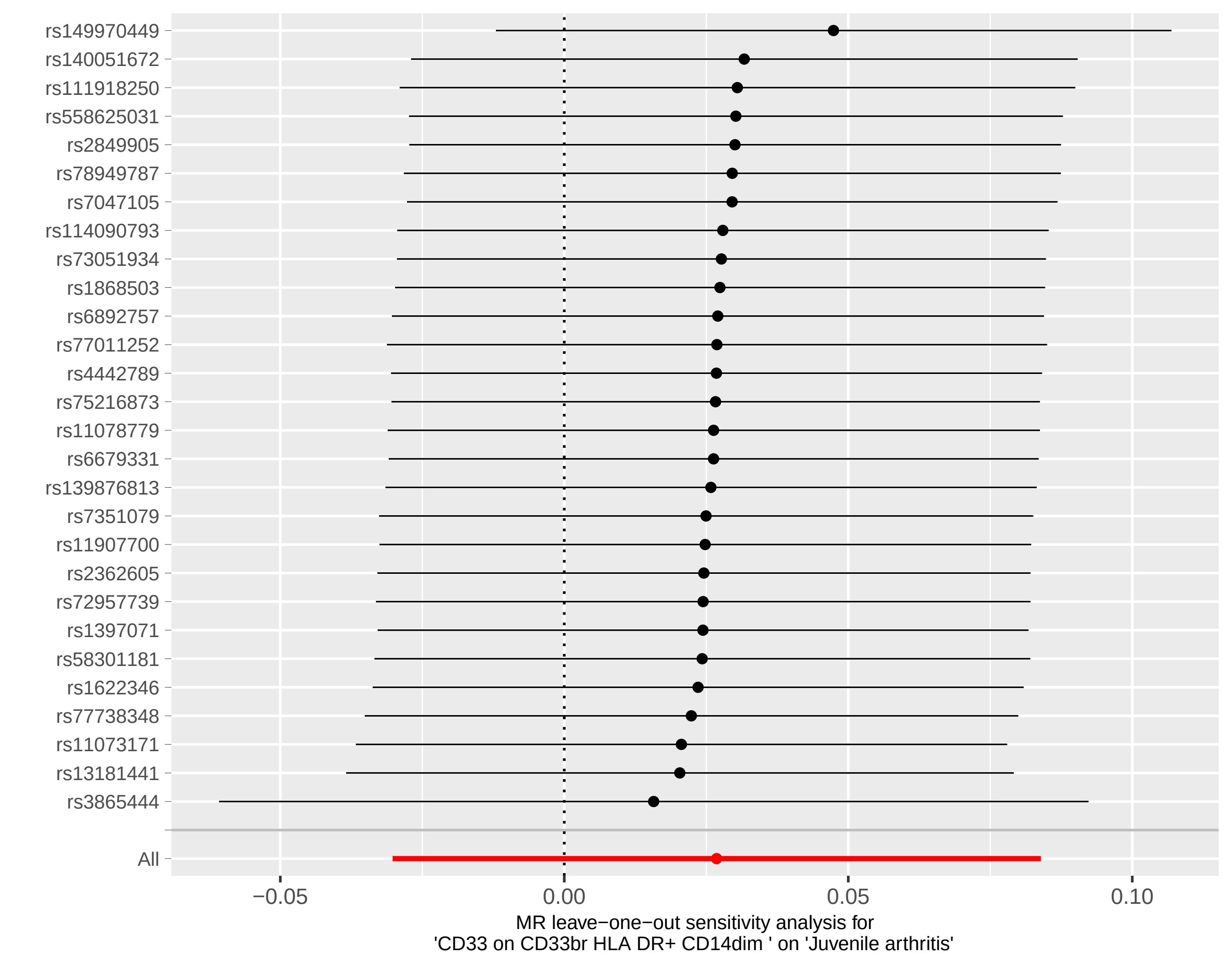
rs149970449
rs140051672
rs111918250
rs558625031
rs2849905
rs78949787
rs7047105
rs114090793
rs73051934
rs1868503
rs6892757
rs77011252
rs4442789
rs75216873
rs11078779
rs6679331
rs139876813
rs7351079
rs11907700
rs2362605
rs72957739
rs1397071
rs58301181
rs1622346
rs77738348
rs11073171
rs13181441
rs3865444
All
−0.05
0.00
0.05
0.10
MR leave−one−out sensitivity analysis for
'CD33 on CD33br HLA DR+ CD14dim ' on 'Juvenile arthritis'

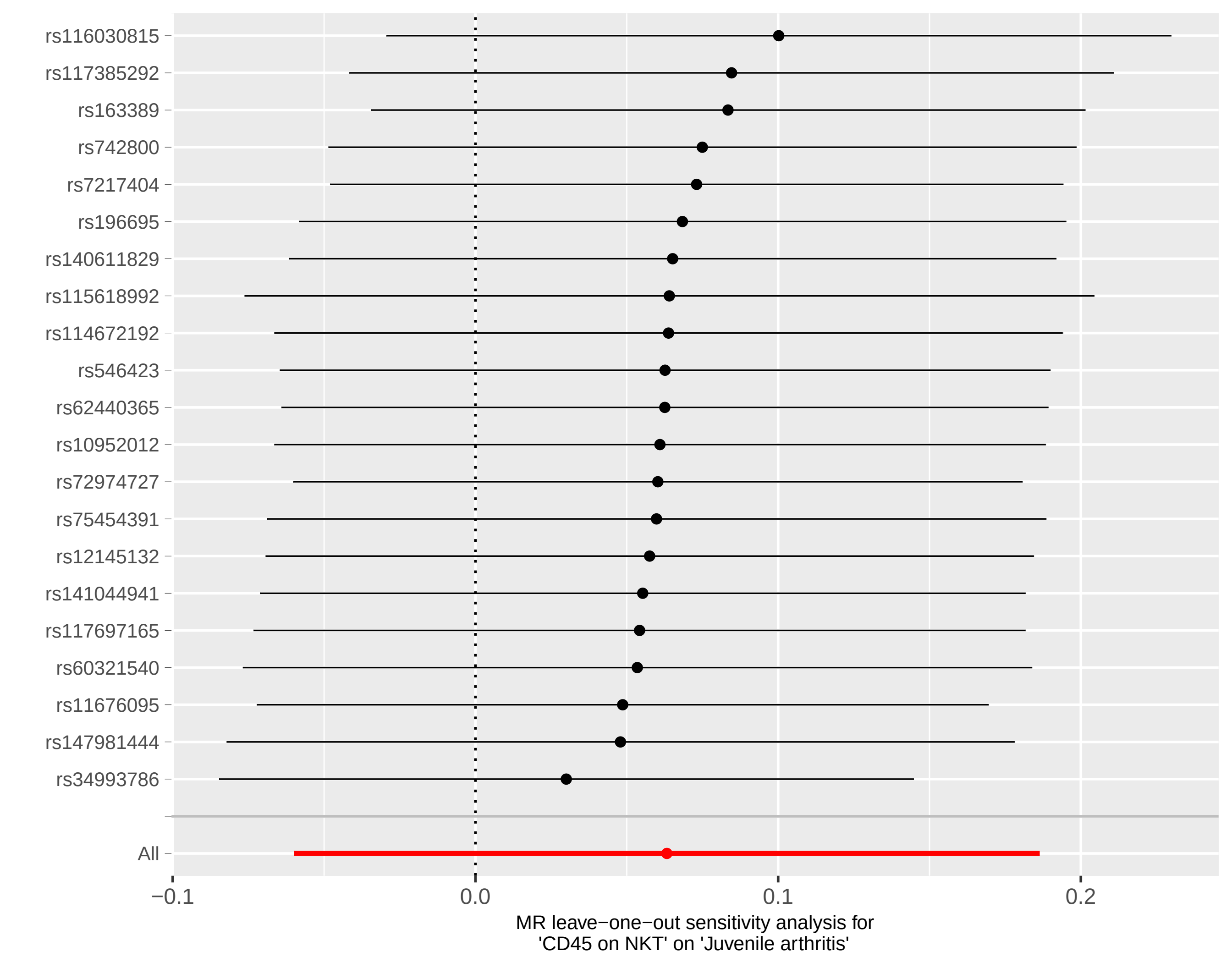
rs116030815
rs117385292
rs163389
rs742800
rs7217404
rs196695
rs140611829
rs115618992
rs114672192
rs546423
rs62440365
rs10952012
rs72974727
rs75454391
rs12145132
rs141044941
rs117697165
rs60321540
rs11676095
rs147981444
rs34993786
All
−0.1
0.0
0.1
0.2
MR leave−one−out sensitivity analysis for
'CD45 on NKT' on 'Juvenile arthritis'

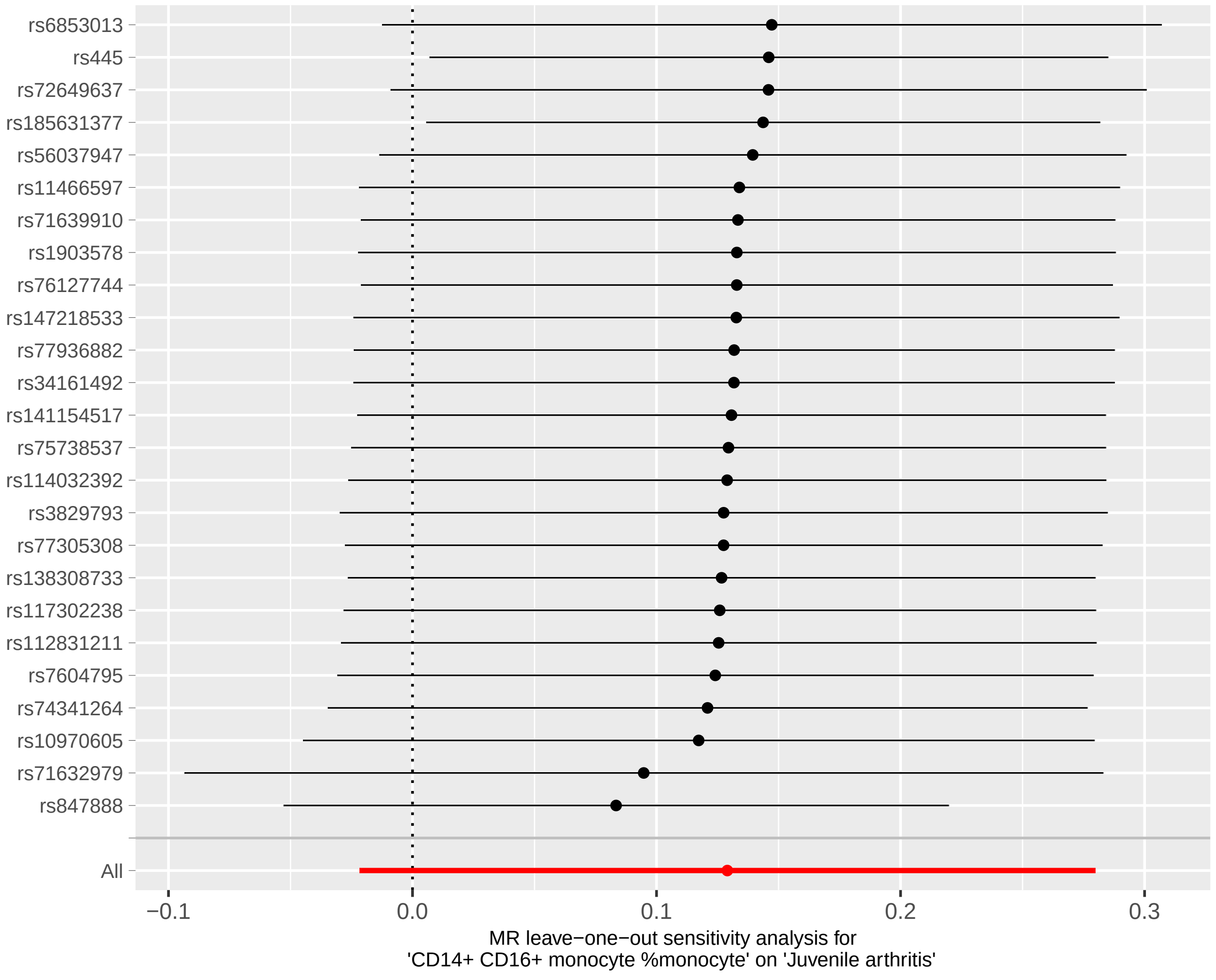
rs6853013
rs445
rs72649637
rs185631377
rs56037947
rs11466597
rs71639910
rs1903578
rs76127744
rs147218533
rs77936882
rs34161492
rs141154517
rs75738537
rs114032392
rs3829793
rs77305308
rs138308733
rs117302238
rs112831211
rs7604795
rs74341264
rs10970605
rs71632979
rs847888
All
−0.1
0.0
0.1
0.2
0.3
MR leave−one−out sensitivity analysis for
'CD14+ CD16+ monocyte %monocyte' on 'Juvenile arthritis'

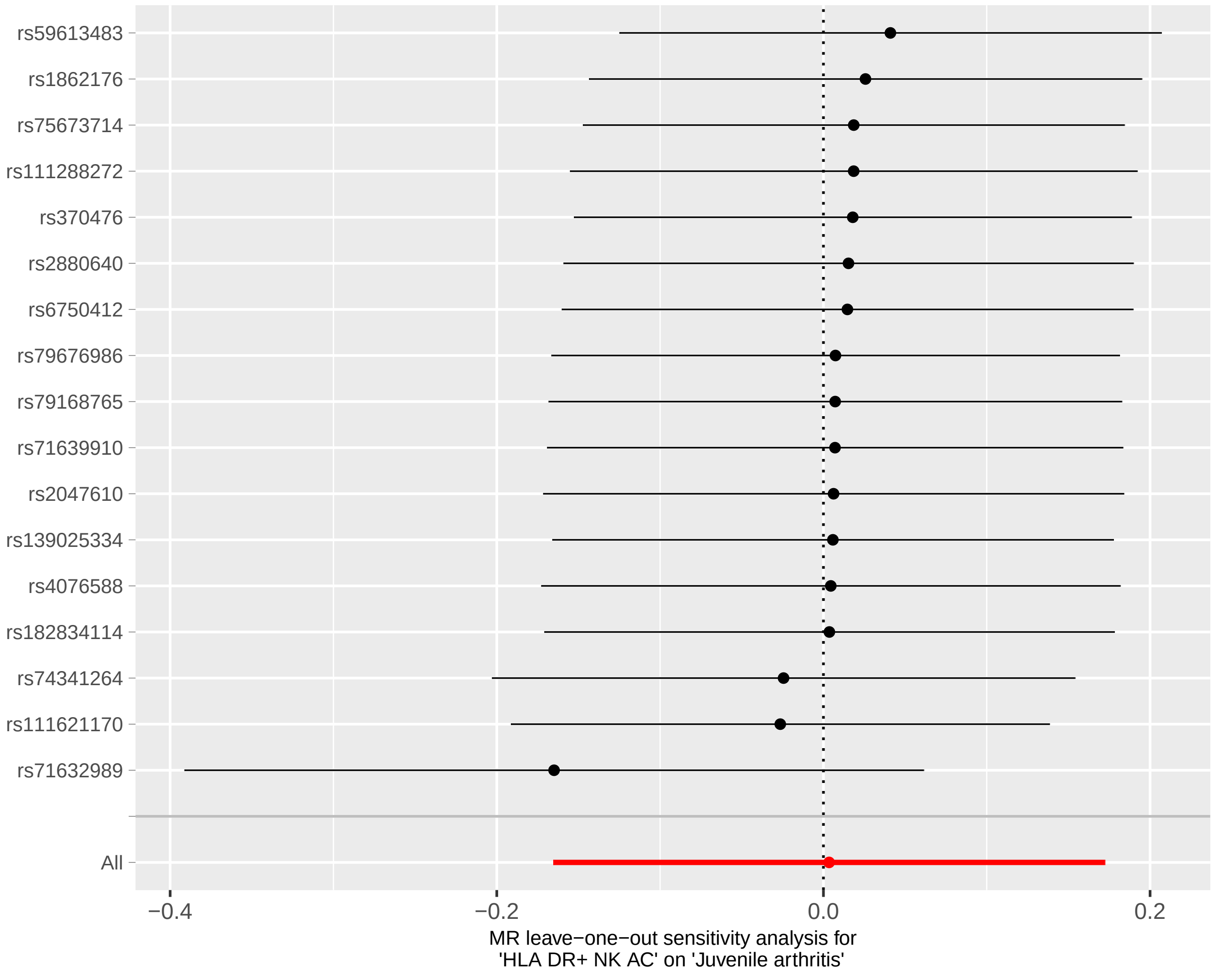
rs59613483
rs1862176
rs75673714
rs111288272
rs370476
rs2880640
rs6750412
rs79676986
rs79168765
rs71639910
rs2047610
rs139025334
rs4076588
rs182834114
rs74341264
rs111621170
rs71632989
All
−0.4
−0.2
0.0
0.2
MR leave−one−out sensitivity analysis for
'HLA DR+ NK AC' on 'Juvenile arthritis'

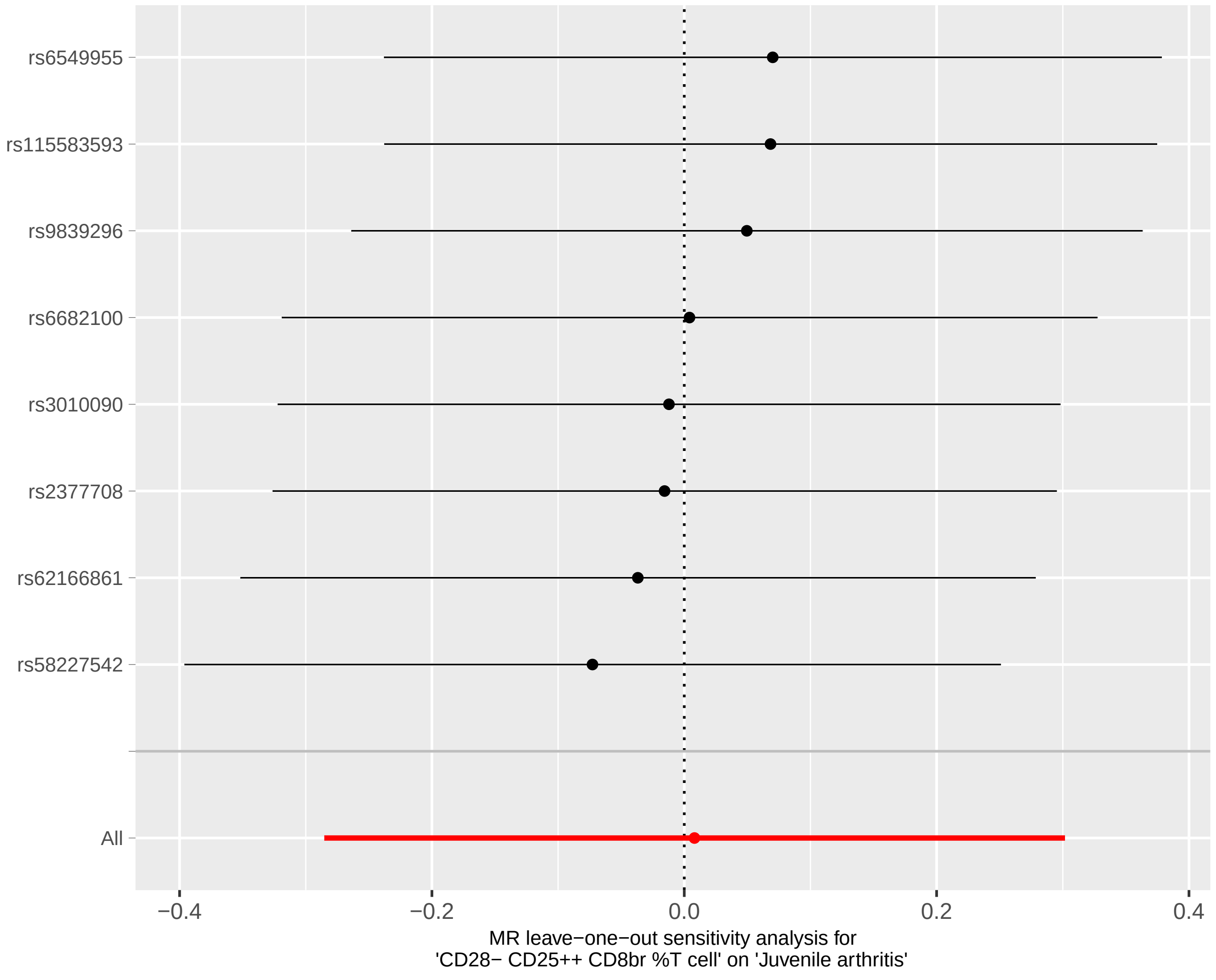
rs6549955
rs115583593
rs9839296
rs6682100
rs3010090
rs2377708
rs62166861
rs58227542
All
−0.4
−0.2
0.0
0.2
0.4
MR leave−one−out sensitivity analysis for
'CD28− CD25++ CD8br %T cell' on 'Juvenile arthritis'

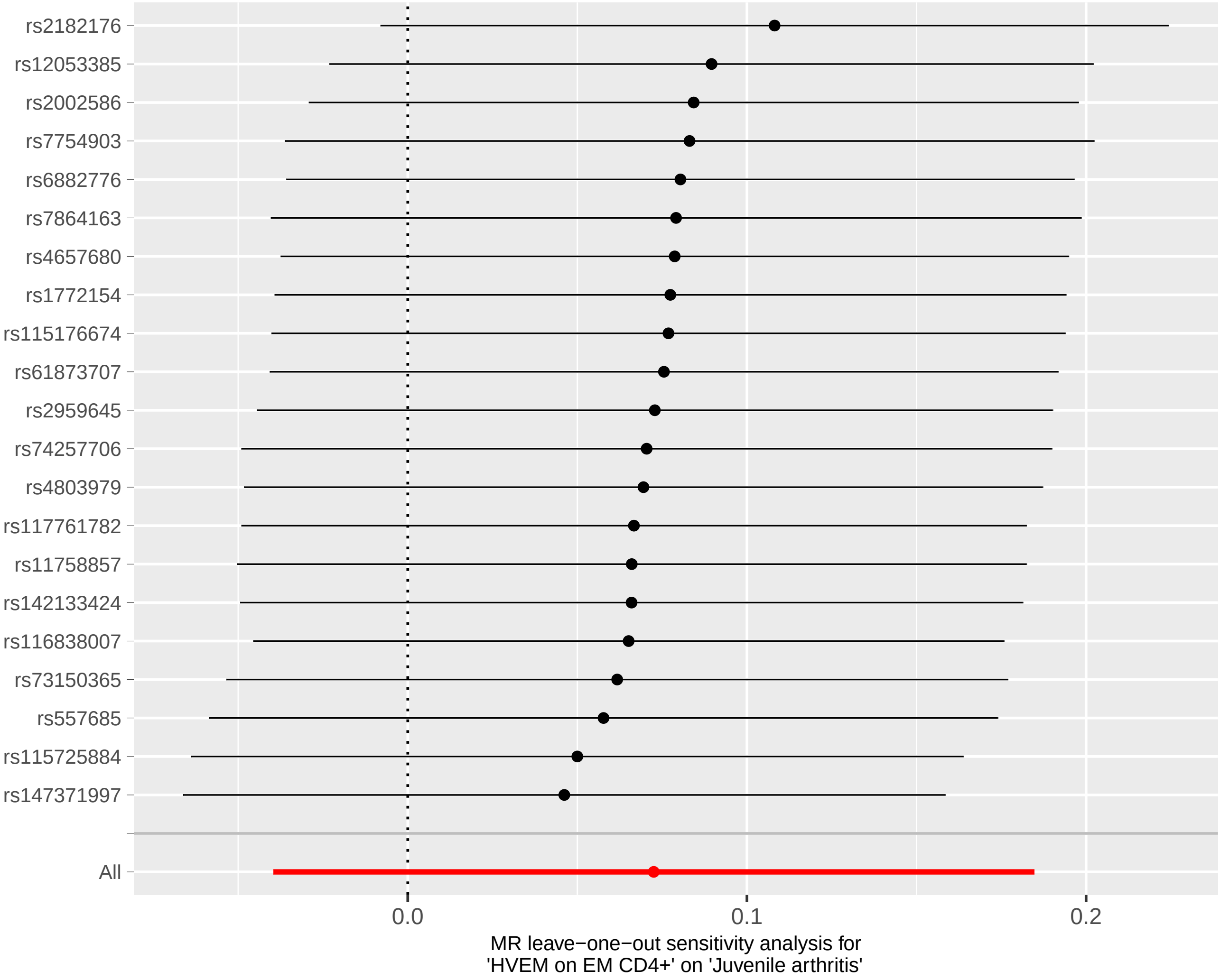

rs2182176
rs12053385
rs2002586
rs7754903
rs6882776
rs7864163
rs4657680
rs1772154
rs115176674
rs61873707
rs2959645
rs74257706
rs4803979
rs117761782
rs11758857
rs142133424
rs116838007
rs73150365
rs557685
rs115725884
rs147371997
All
0.0
0.1
0.2
MR leave−one−out sensitivity analysis for
'HVEM on EM CD4+' on 'Juvenile arthritis'

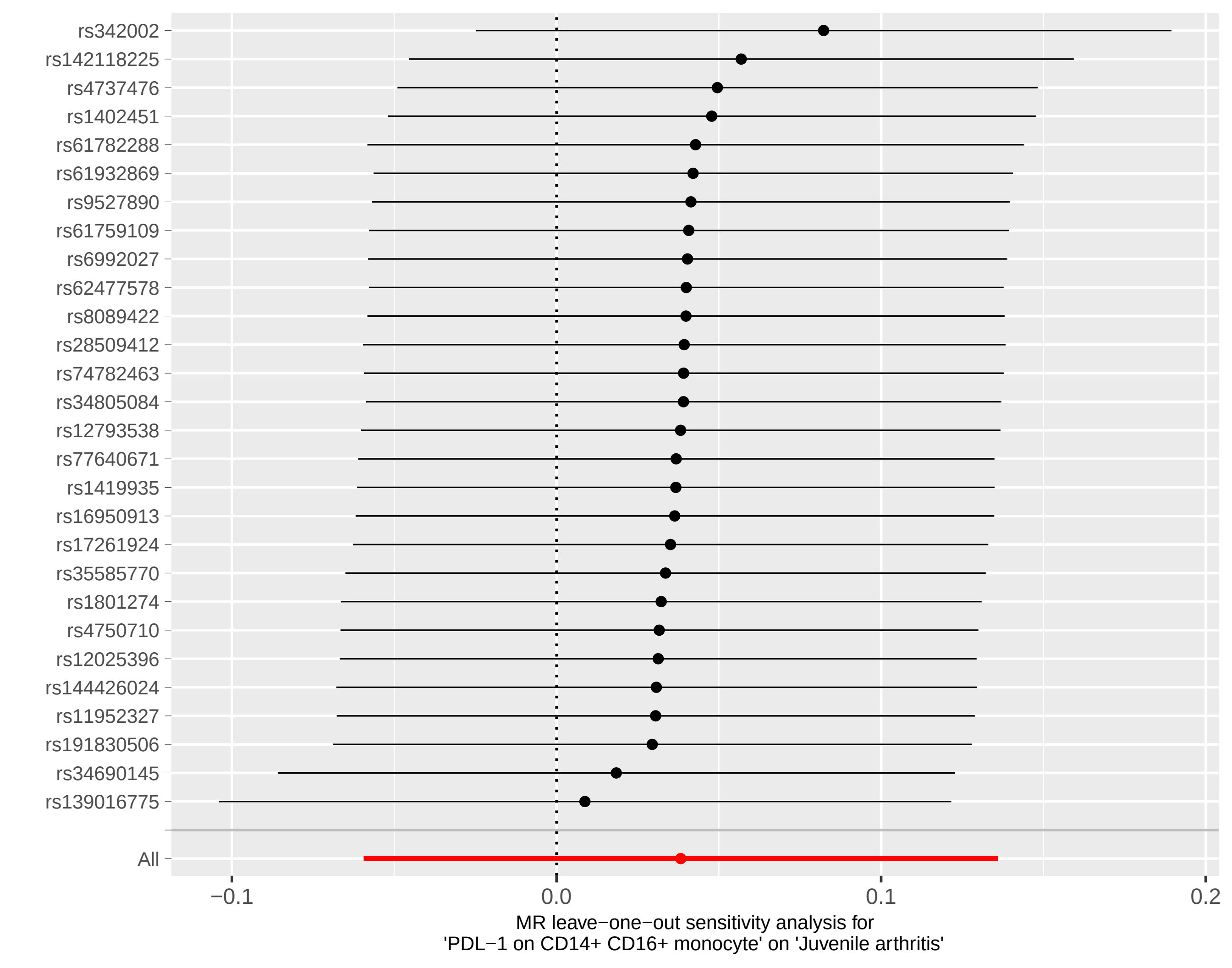

rs342002
rs142118225
rs4737476
rs1402451
rs61782288
rs61932869
rs9527890
rs61759109
rs6992027
rs62477578
rs8089422
rs28509412
rs74782463
rs34805084
rs12793538
rs77640671
rs1419935
rs16950913
rs17261924
rs35585770
rs1801274
rs4750710
rs12025396
rs144426024
rs11952327
rs191830506
rs34690145
rs139016775
All
−0.1
0.0
0.1
0.2
MR leave−one−out sensitivity analysis for
'PDL−1 on CD14+ CD16+ monocyte' on 'Juvenile arthritis'

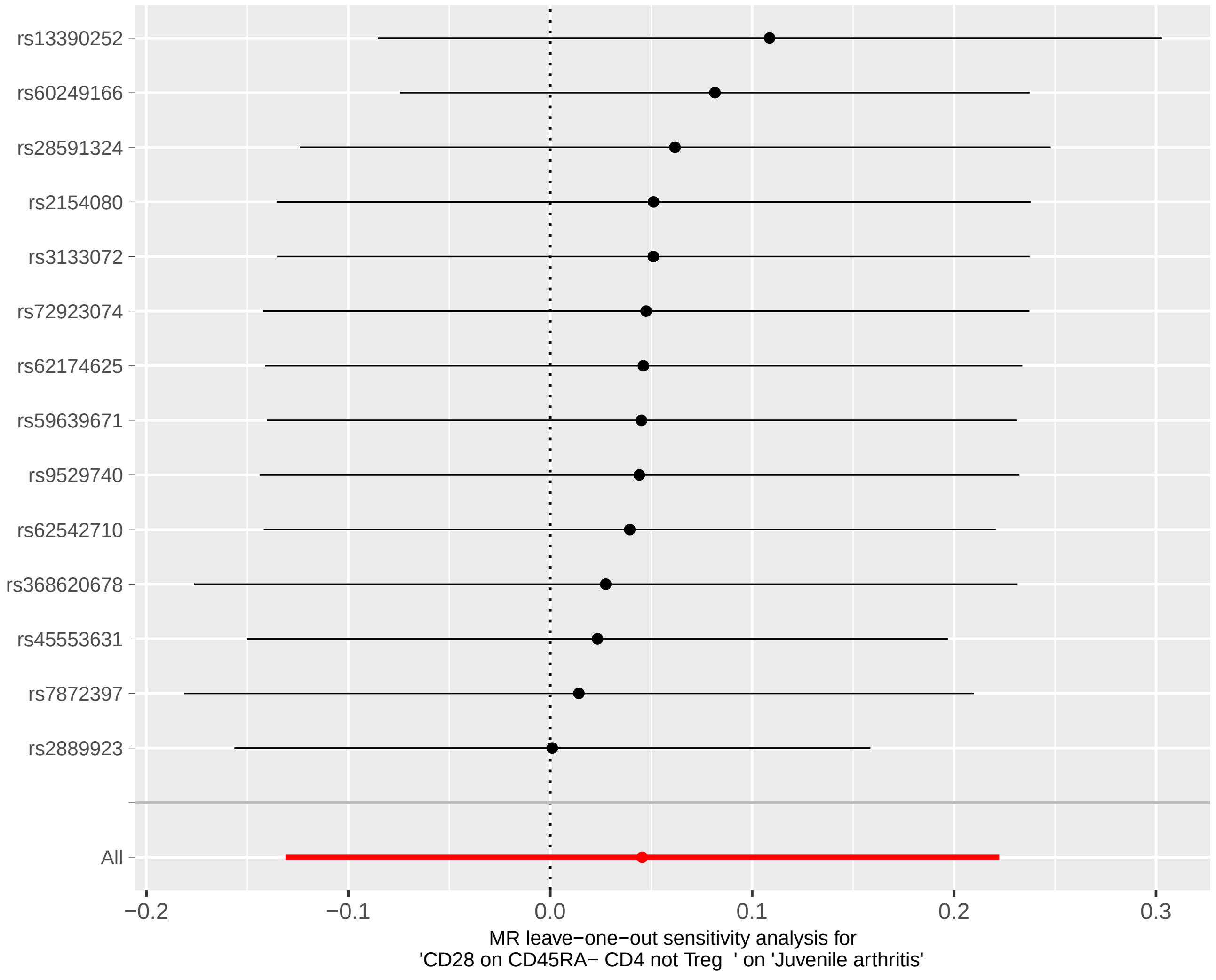
rs13390252
rs60249166
rs28591324
rs2154080
rs3133072
rs72923074
rs62174625
rs59639671
rs9529740
rs62542710
rs368620678
rs45553631
rs7872397
rs2889923
All
−0.2
−0.1
0.0
0.1
0.2
0.3
MR leave−one−out sensitivity analysis for
'CD28 on CD45RA− CD4 not Treg ' on 'Juvenile arthritis'

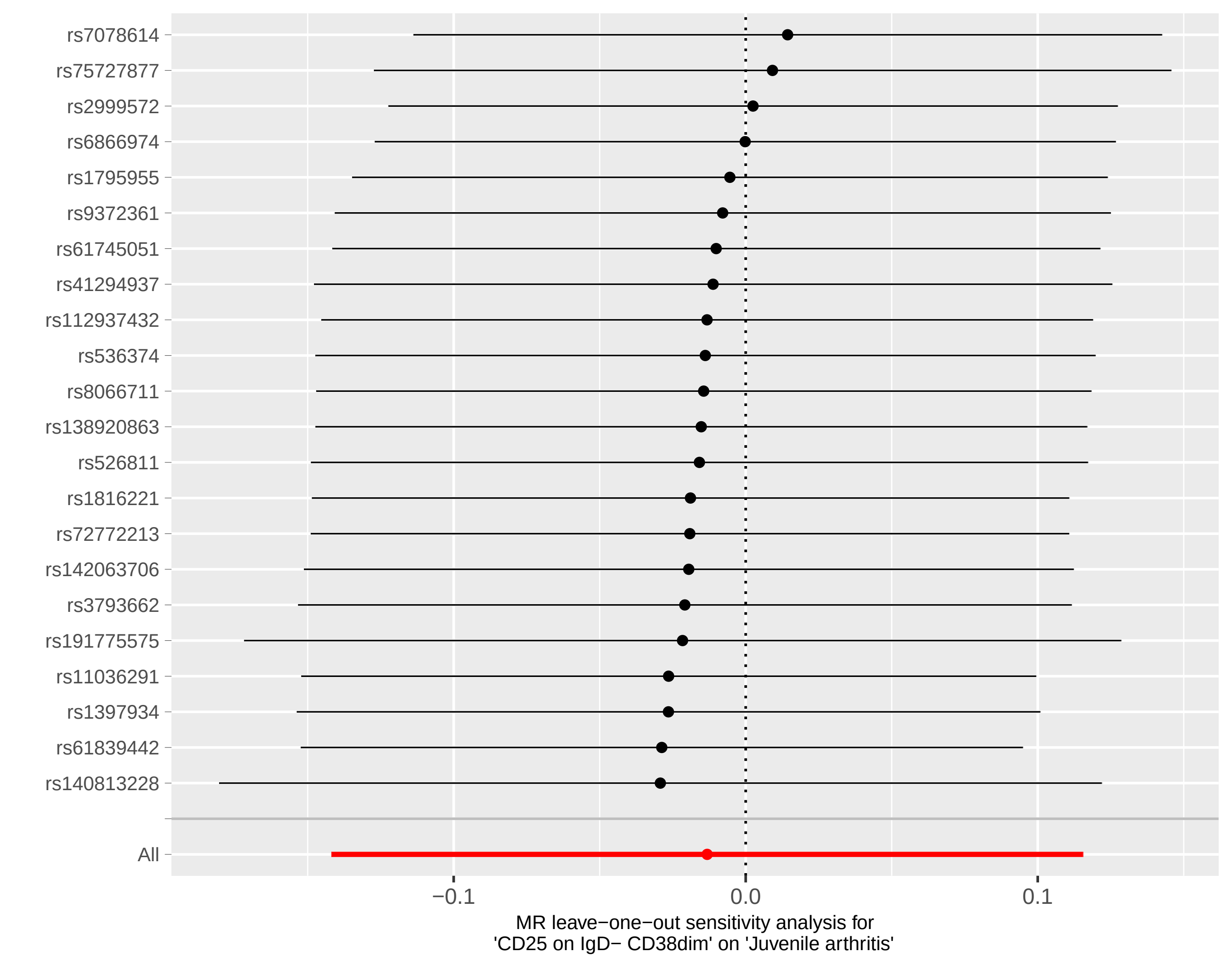
rs7078614
rs75727877
rs2999572
rs6866974
rs1795955
rs9372361
rs61745051
rs41294937
rs112937432
rs536374
rs8066711
rs138920863
rs526811
rs1816221
rs72772213
rs142063706
rs3793662
rs191775575
rs11036291
rs1397934
rs61839442
rs140813228
All
−0.1
0.0
0.1
MR leave−one−out sensitivity analysis for
'CD25 on IgD− CD38dim' on 'Juvenile arthritis'

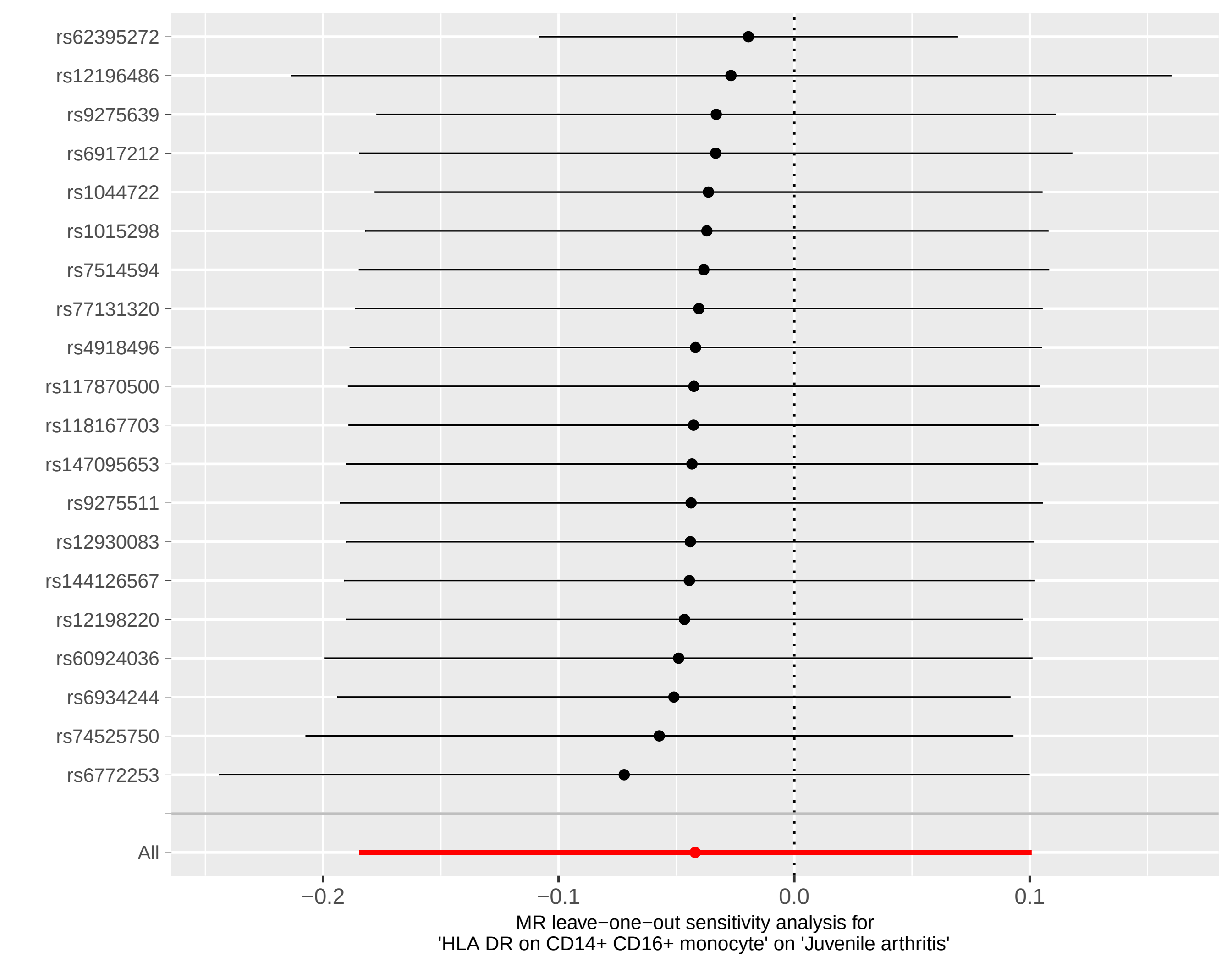
rs62395272
rs12196486
rs9275639
rs6917212
rs1044722
rs1015298
rs7514594
rs77131320
rs4918496
rs117870500
rs118167703
rs147095653
rs9275511
rs12930083
rs144126567
rs12198220
rs60924036
rs6934244
rs74525750
rs6772253
All
−0.2
−0.1
0.0
0.1
MR leave−one−out sensitivity analysis for
'HLA DR on CD14+ CD16+ monocyte' on 'Juvenile arthritis'

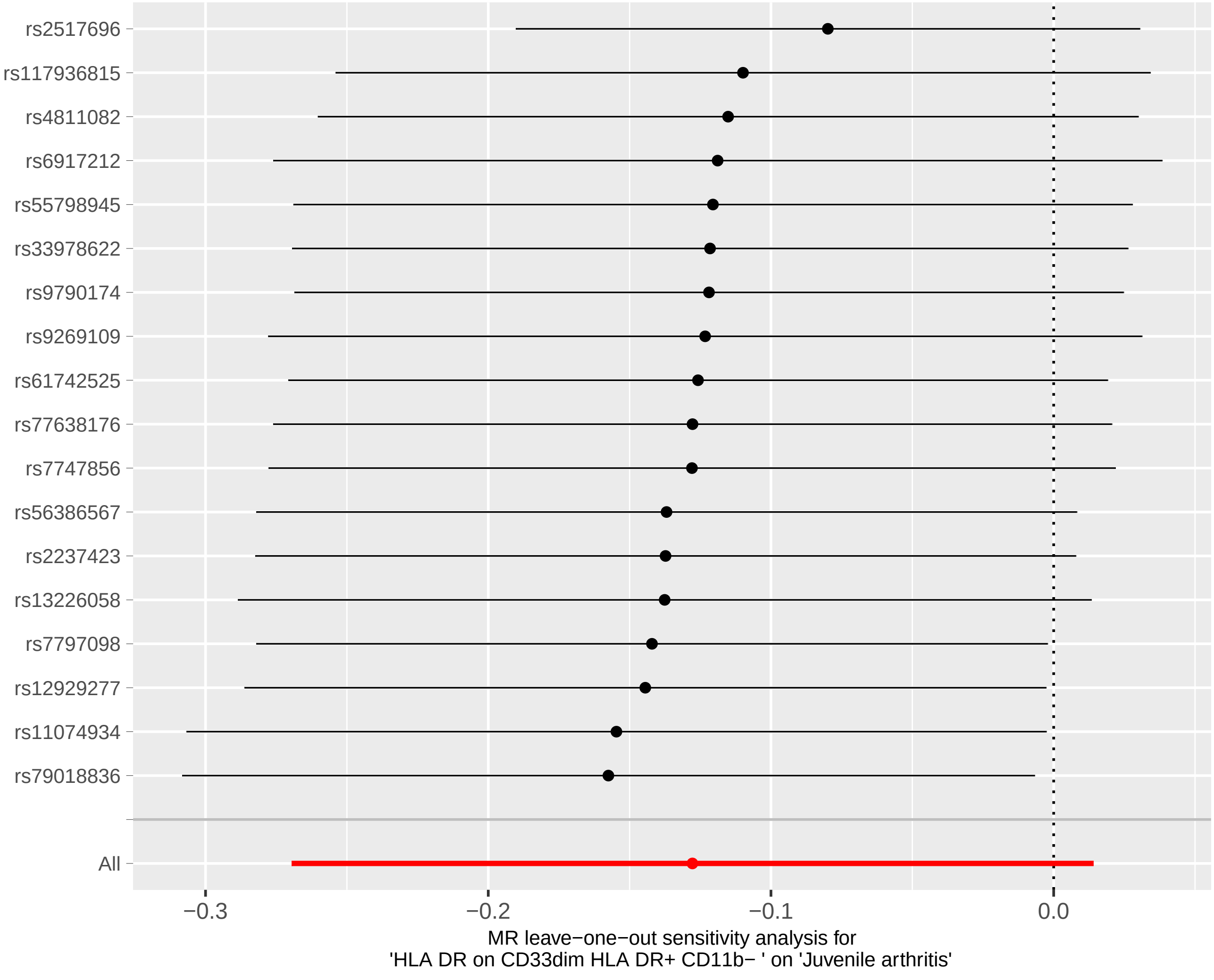
rs2517696
rs117936815
rs4811082
rs6917212
rs55798945
rs33978622
rs9790174
rs9269109
rs61742525
rs77638176
rs7747856
rs56386567
rs2237423
rs13226058
rs7797098
rs12929277
rs11074934
rs79018836
All
−0.3
−0.2
−0.1
0.0
MR leave−one−out sensitivity analysis for
'HLA DR on CD33dim HLA DR+ CD11b− ' on 'Juvenile arthritis'

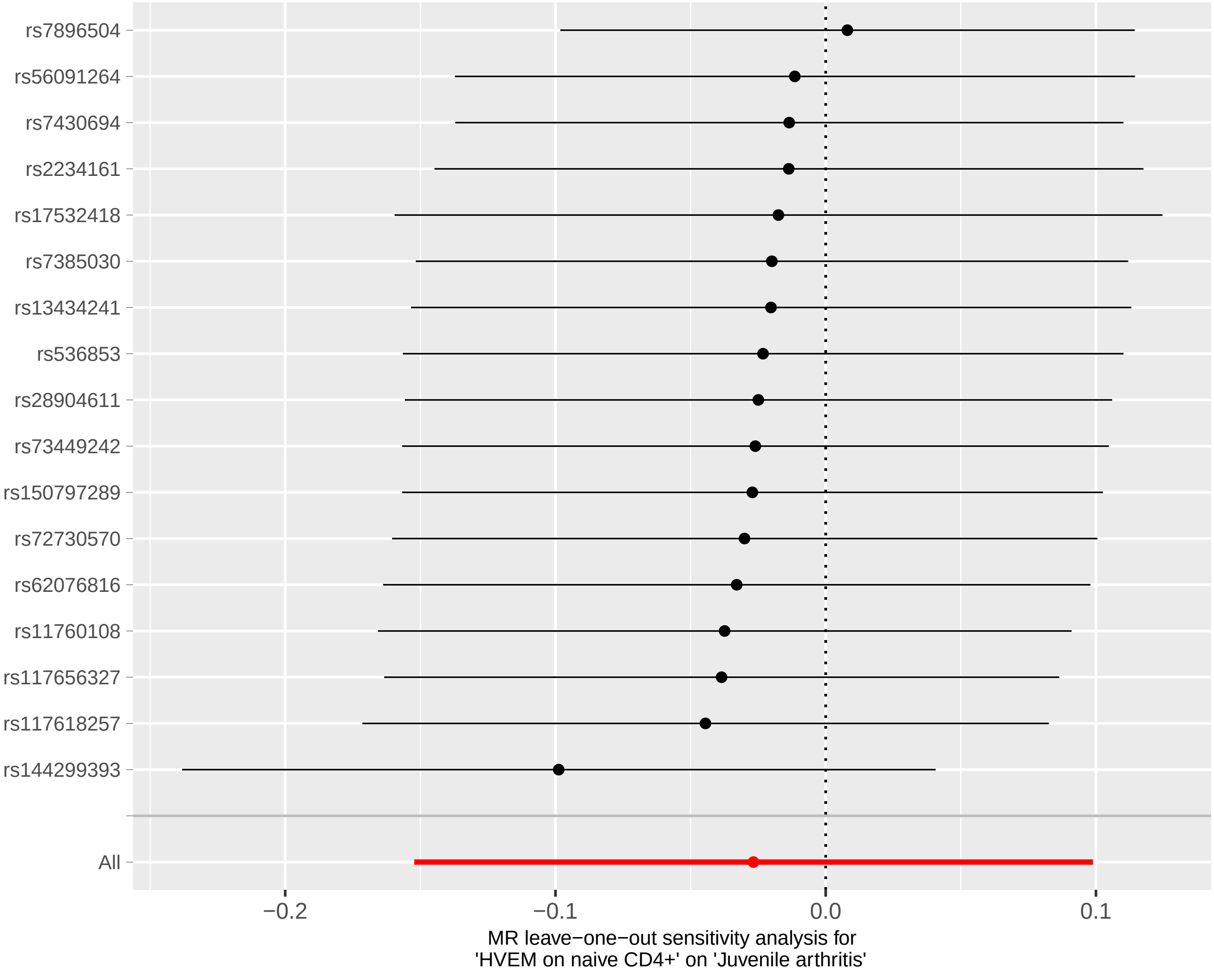
rs7896504
rs56091264
rs7430694
rs2234161
rs17532418
rs7385030
rs13434241
rs536853
rs28904611
rs73449242
rs150797289
rs72730570
rs62076816
rs11760108
rs117656327
rs117618257
rs144299393
All
−0.2
−0.1
0.0
0.1
MR leave−one−out sensitivity analysis for
'HVEM on naive CD4+' on 'Juvenile arthritis'

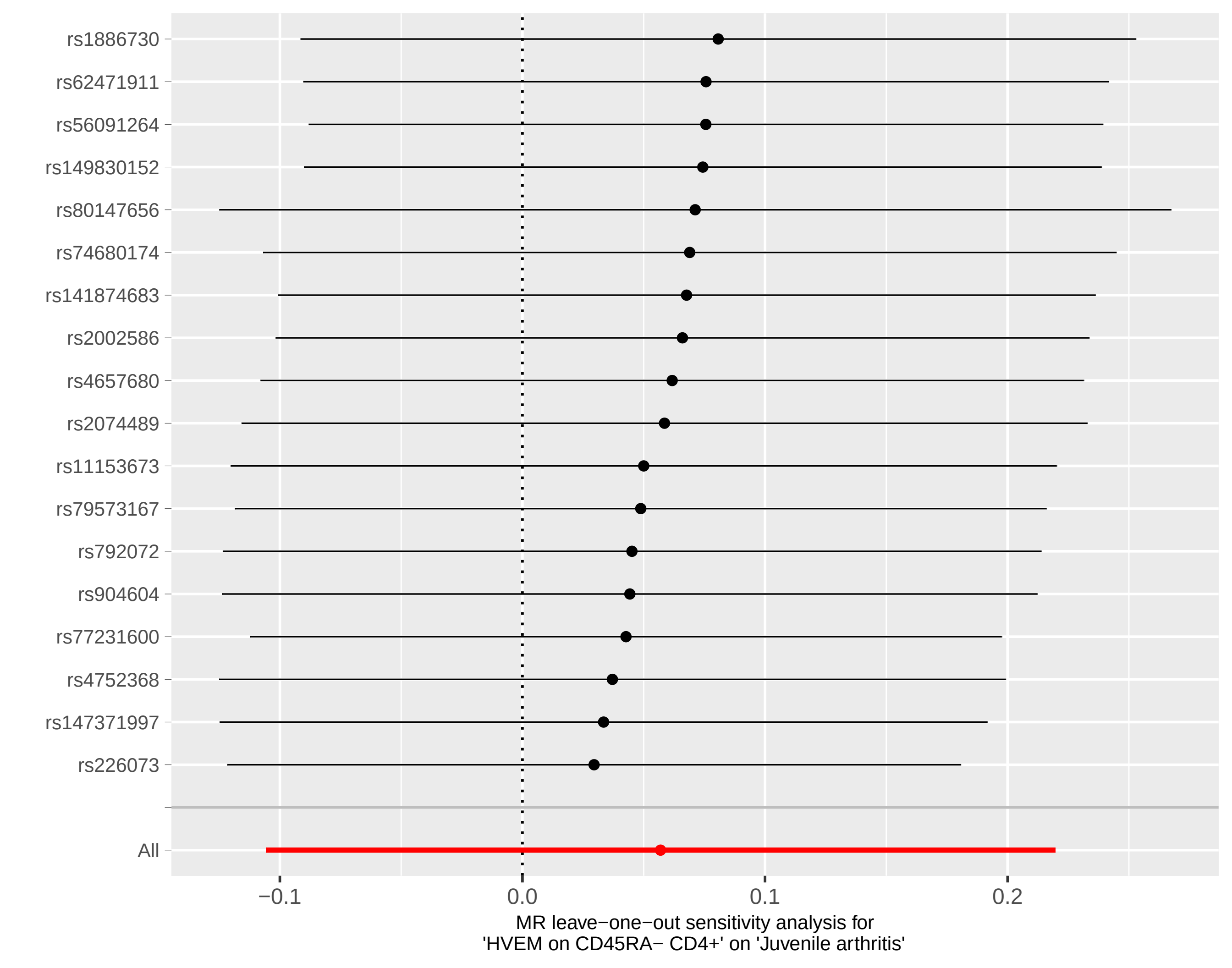

rs1886730
rs62471911
rs56091264
rs149830152
rs80147656
rs74680174
rs141874683
rs2002586
rs4657680
rs2074489
rs11153673
rs79573167
rs792072
rs904604
rs77231600
rs4752368
rs147371997
rs226073
All
−0.1
0.0
0.1
0.2
MR leave−one−out sensitivity analysis for
'HVEM on CD45RA− CD4+' on 'Juvenile arthritis'

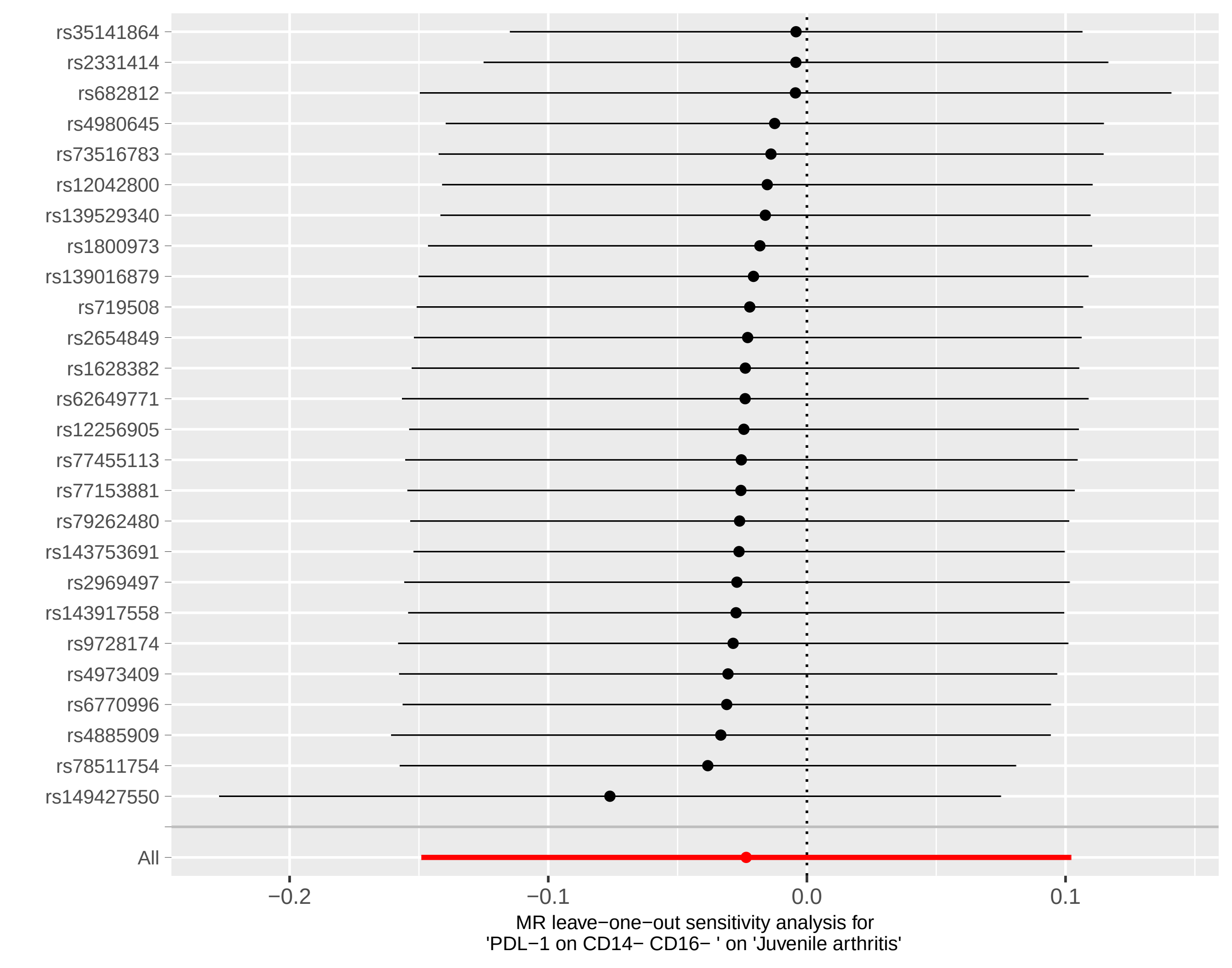

rs35141864
rs2331414
rs682812
rs4980645
rs73516783
rs12042800
rs139529340
rs1800973
rs139016879
rs719508
rs2654849
rs1628382
rs62649771
rs12256905
rs77455113
rs77153881
rs79262480
rs143753691
rs2969497
rs143917558
rs9728174
rs4973409
rs6770996
rs4885909
rs78511754
rs149427550
All
−0.2
−0.1
0.0
0.1
MR leave−one−out sensitivity analysis for
'PDL−1 on CD14− CD16− ' on 'Juvenile arthritis'

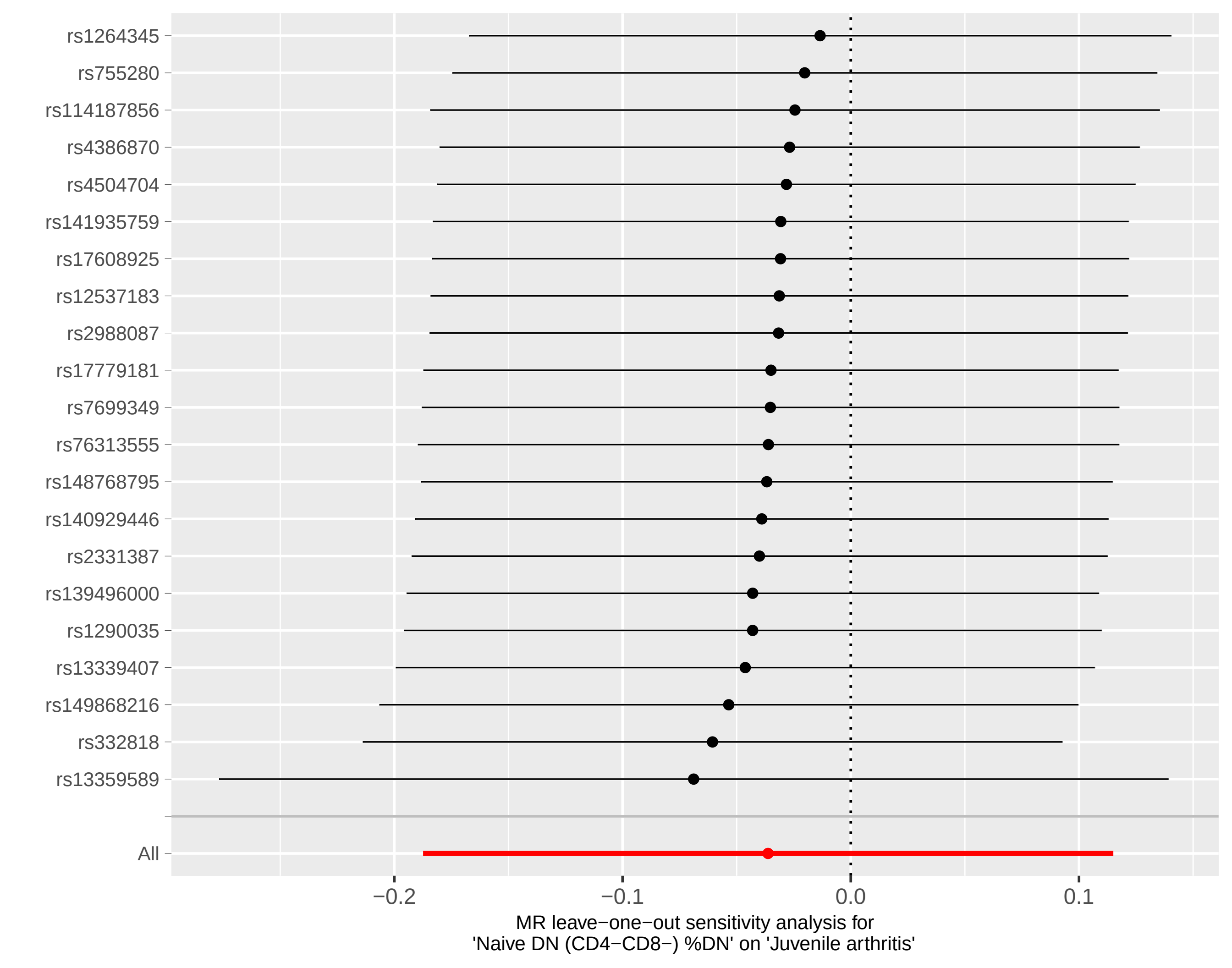
rs1264345
rs755280
rs114187856
rs4386870
rs4504704
rs141935759
rs17608925
rs12537183
rs2988087
rs17779181
rs7699349
rs76313555
rs148768795
rs140929446
rs2331387
rs139496000
rs1290035
rs13339407
rs149868216
rs332818
rs13359589
All
−0.2
−0.1
0.0
0.1
MR leave−one−out sensitivity analysis for
'Naive DN (CD4−CD8−) %DN' on 'Juvenile arthritis'

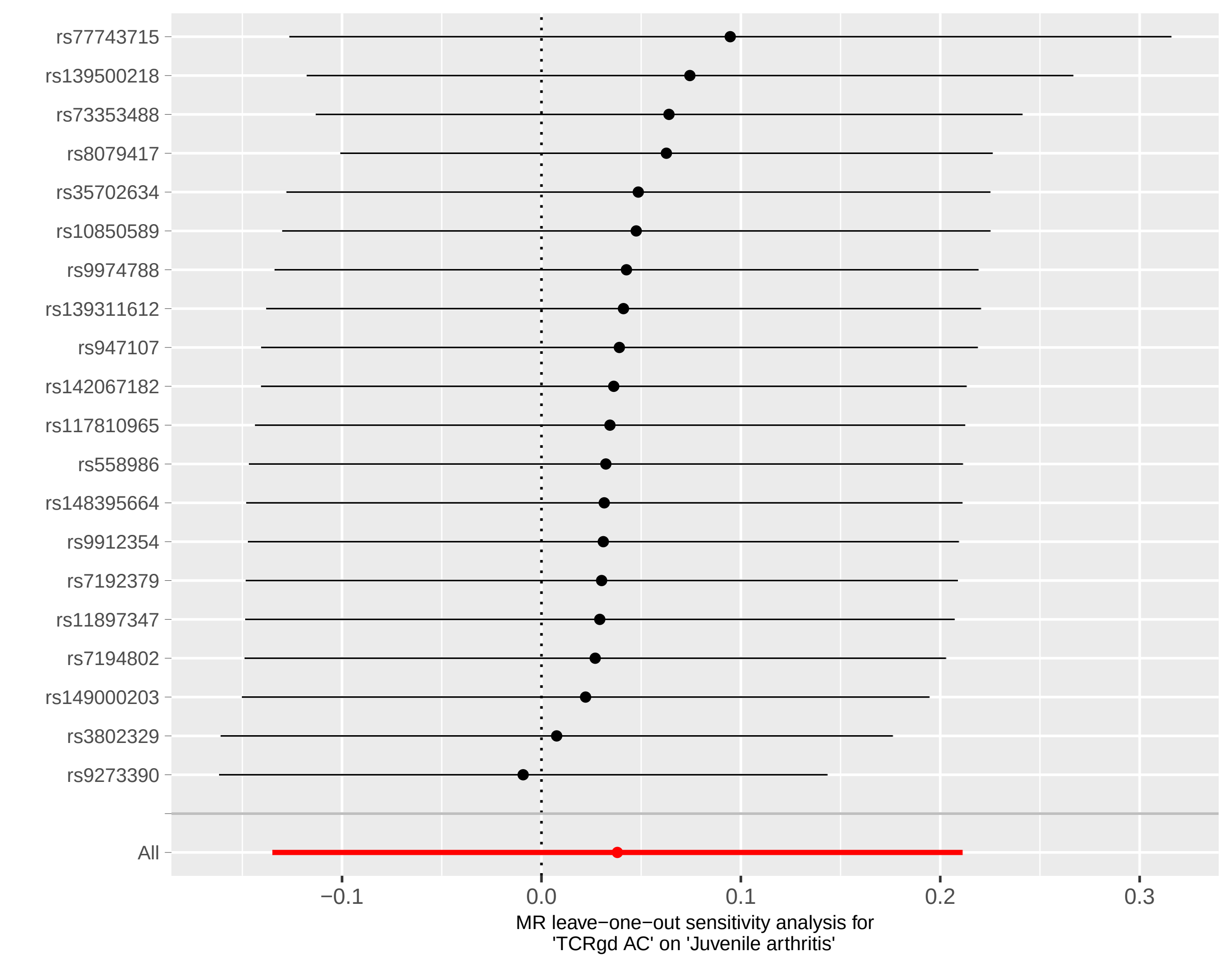
rs77743715
rs139500218
rs73353488
rs8079417
rs35702634
rs10850589
rs9974788
rs139311612
rs947107
rs142067182
rs117810965
rs558986
rs148395664
rs9912354
rs7192379
rs11897347
rs7194802
rs149000203
rs3802329
rs9273390
All
−0.1
0.0
0.1
0.2
0.3
MR leave−one−out sensitivity analysis for
'TCRgd AC' on 'Juvenile arthritis'

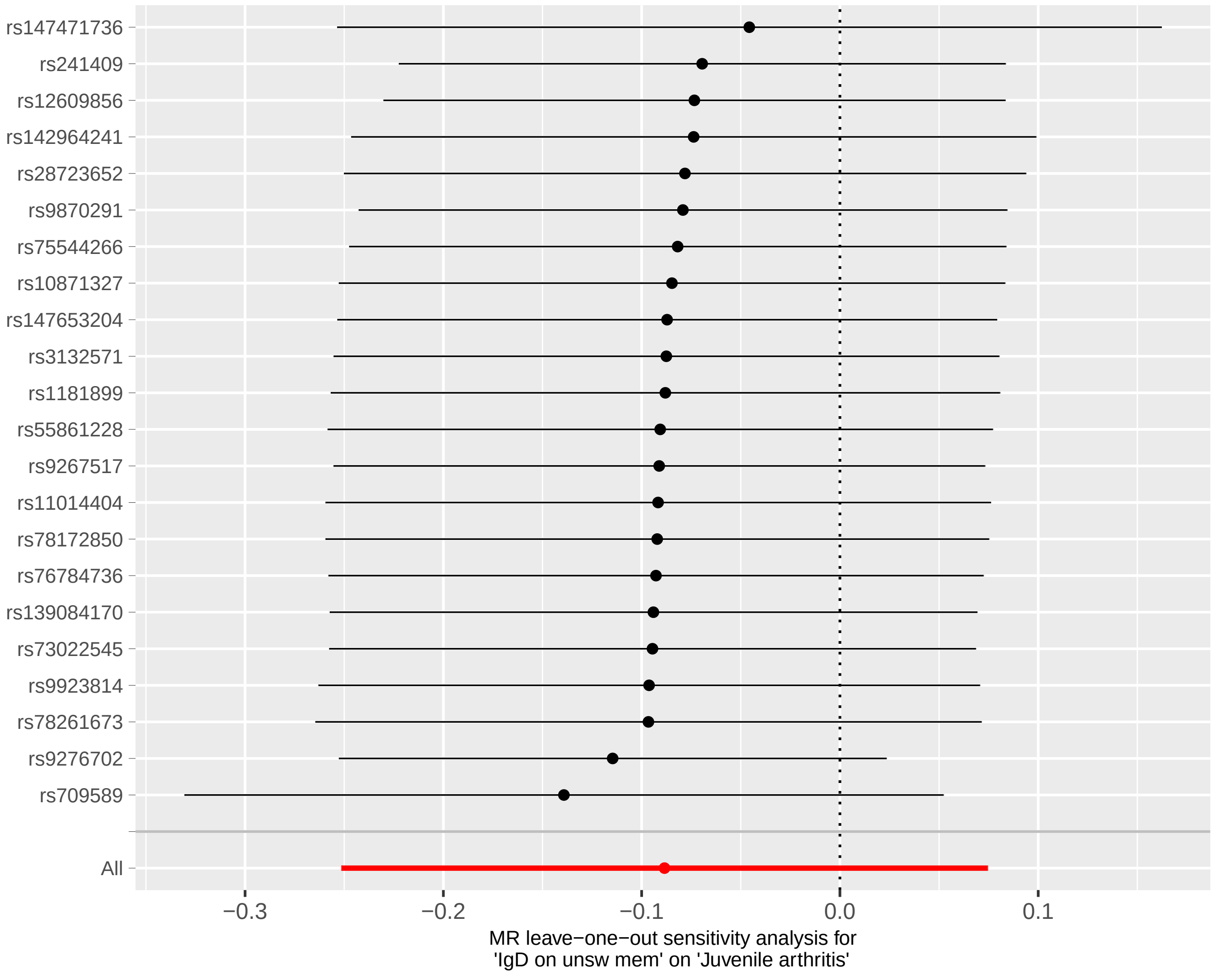

rs147471736
rs241409
rs12609856
rs142964241
rs28723652
rs9870291
rs75544266
rs10871327
rs147653204
rs3132571
rs1181899
rs55861228
rs9267517
rs11014404
rs78172850
rs76784736
rs139084170
rs73022545
rs9923814
rs78261673
rs9276702
rs709589
All
−0.3
−0.2
−0.1
0.0
0.1
MR leave−one−out sensitivity analysis for
'IgD on unsw mem' on 'Juvenile arthritis'

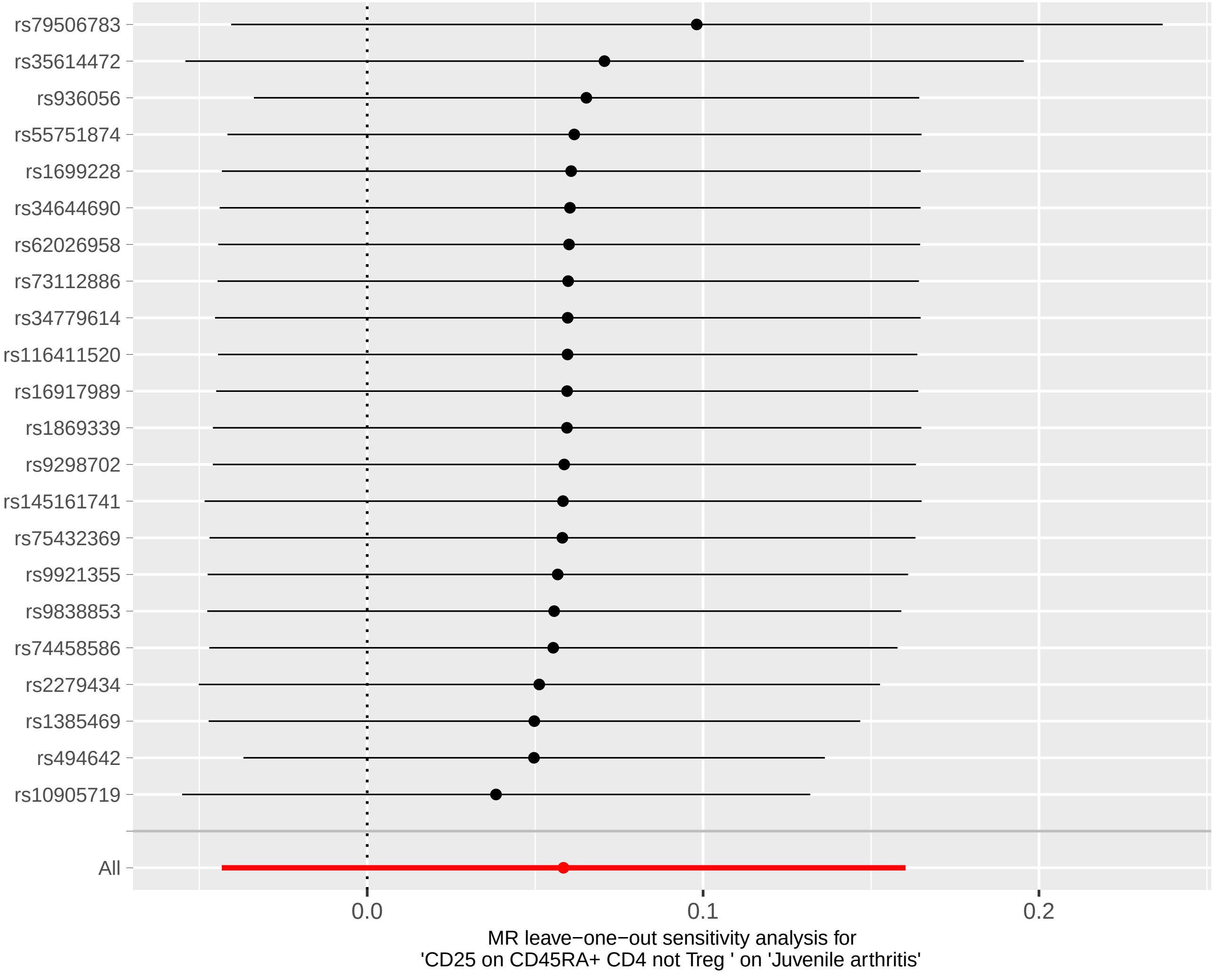

rs79506783
rs35614472
rs936056
rs55751874
rs1699228
rs34644690
rs62026958
rs73112886
rs34779614
rs116411520
rs16917989
rs1869339
rs9298702
rs145161741
rs75432369
rs9921355
rs9838853
rs74458586
rs2279434
rs1385469
rs494642
rs10905719
All
0.0
0.1
0.2
MR leave−one−out sensitivity analysis for
'CD25 on CD45RA+ CD4 not Treg ' on 'Juvenile arthritis'

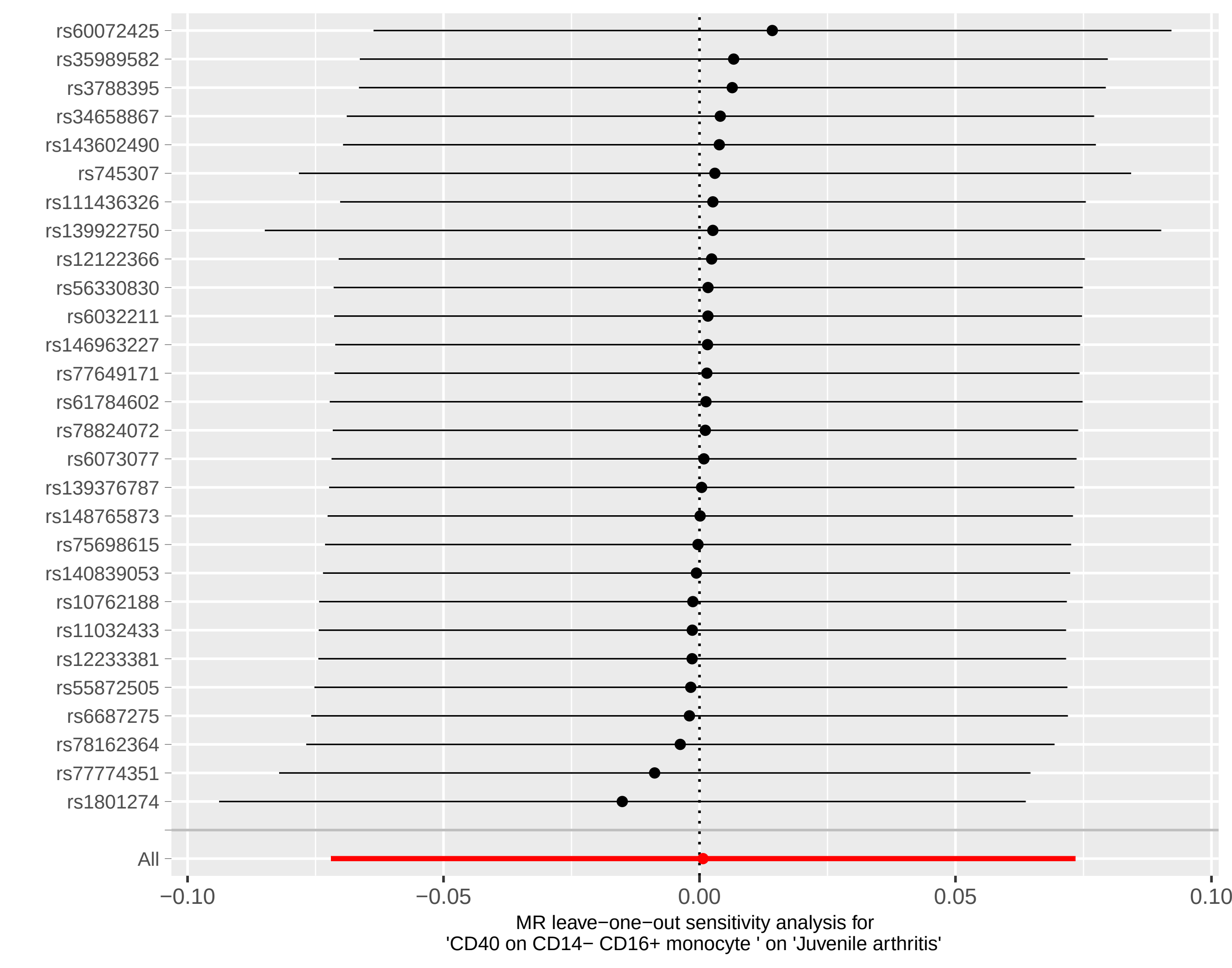
rs60072425
rs35989582
rs3788395
rs34658867
rs143602490
rs745307
rs111436326
rs139922750
rs12122366
rs56330830
rs6032211
rs146963227
rs77649171
rs61784602
rs78824072
rs6073077
rs139376787
rs148765873
rs75698615
rs140839053
rs10762188
rs11032433
rs12233381
rs55872505
rs6687275
rs78162364
rs77774351
rs1801274
All
−0.10
−0.05
0.00
0.05
0.10
MR leave−one−out sensitivity analysis for
'CD40 on CD14− CD16+ monocyte ' on 'Juvenile arthritis'

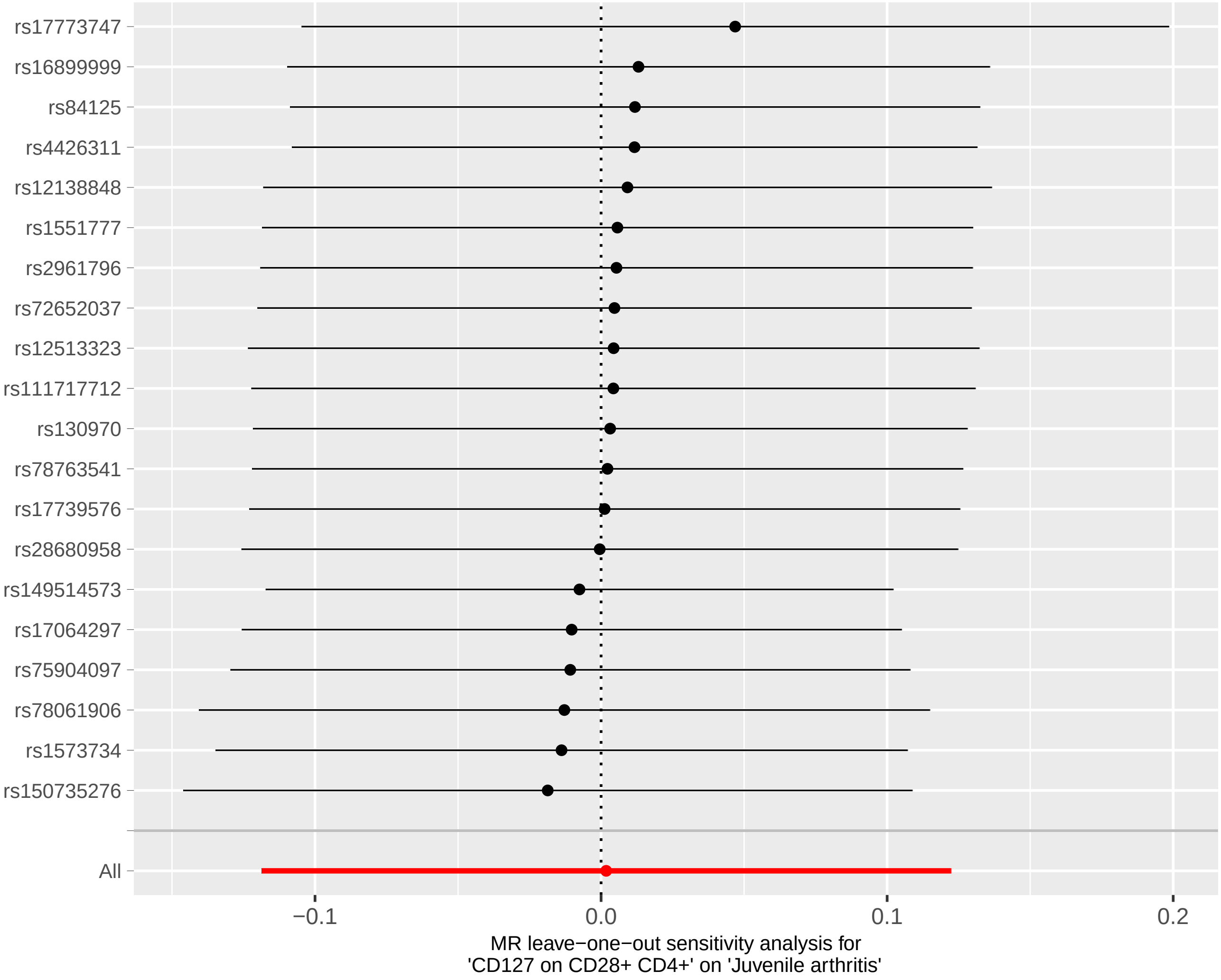
rs17773747
rs16899999
rs84125
rs4426311
rs12138848
rs1551777
rs2961796
rs72652037
rs12513323
rs111717712
rs130970
rs78763541
rs17739576
rs28680958
rs149514573
rs17064297
rs75904097
rs78061906
rs1573734
rs150735276
All
−0.1
0.0
0.1
0.2
MR leave−one−out sensitivity analysis for
'CD127 on CD28+ CD4+' on 'Juvenile arthritis'

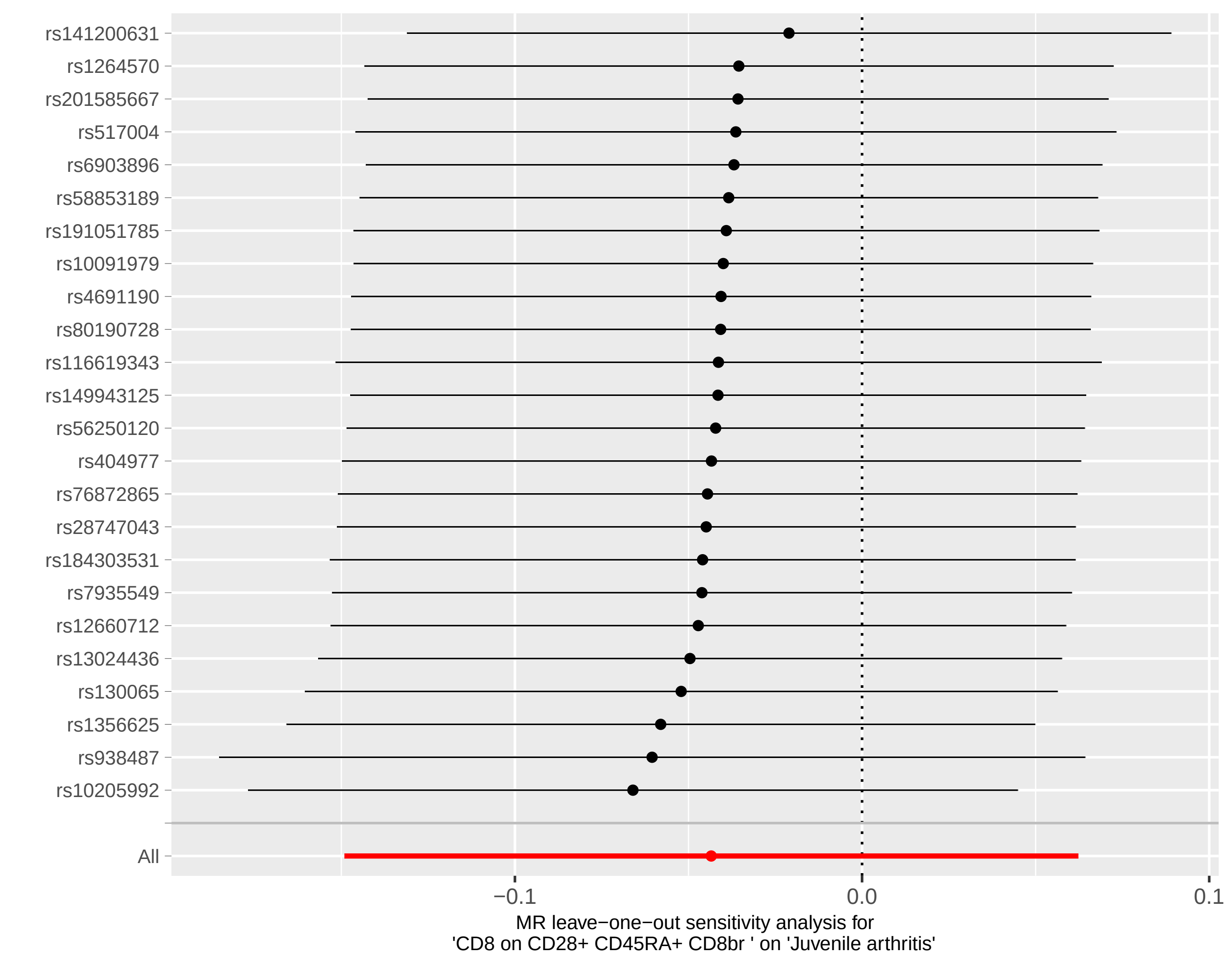
rs141200631
rs1264570
rs201585667
rs517004
rs6903896
rs58853189
rs191051785
rs10091979
rs4691190
rs80190728
rs116619343
rs149943125
rs56250120
rs404977
rs76872865
rs28747043
rs184303531
rs7935549
rs12660712
rs13024436
rs130065
rs1356625
rs938487
rs10205992
All
−0.1
0.0
0.1
MR leave−one−out sensitivity analysis for
'CD8 on CD28+ CD45RA+ CD8br ' on 'Juvenile arthritis'

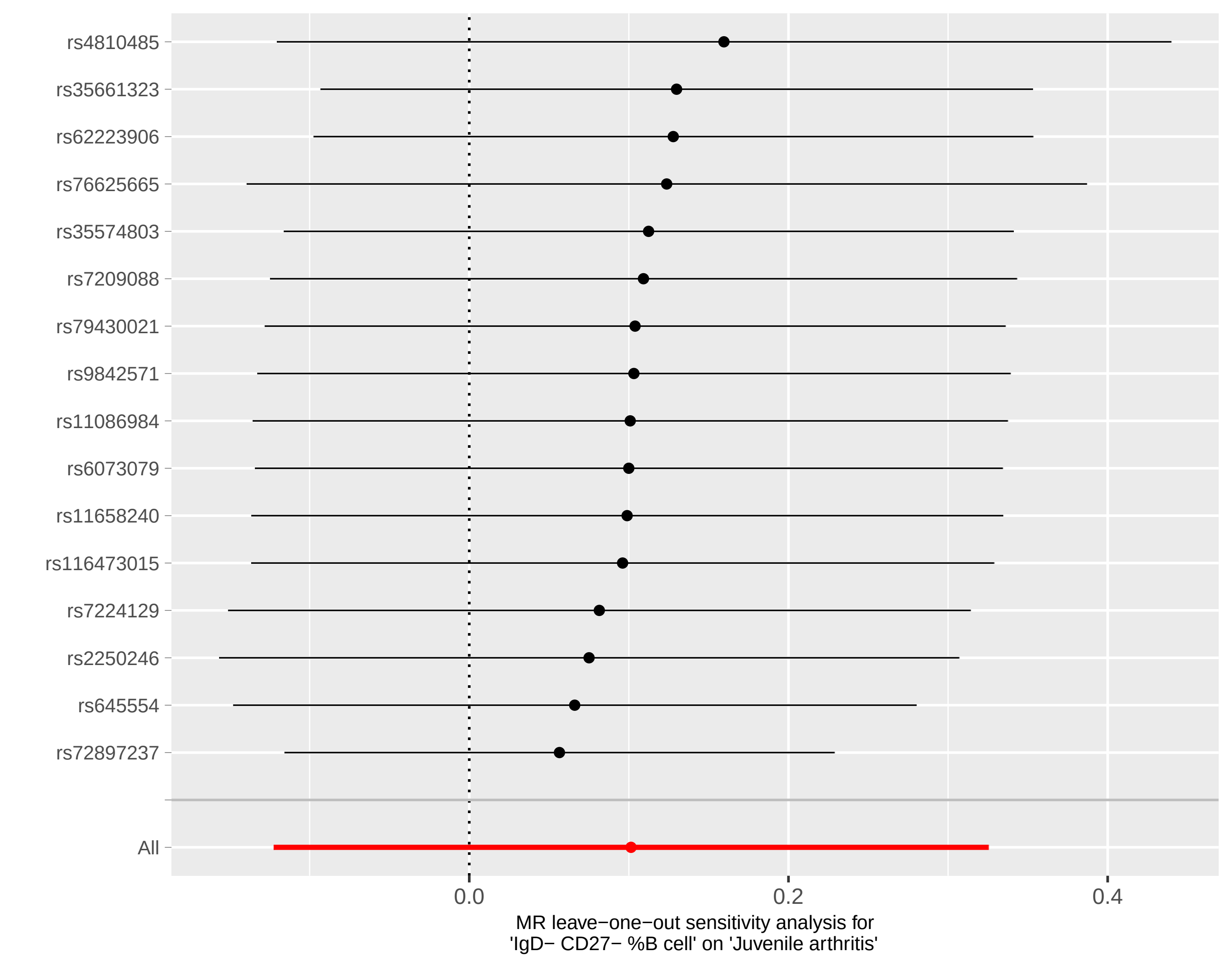
rs4810485
rs35661323
rs62223906
rs76625665
rs35574803
rs7209088
rs79430021
rs9842571
rs11086984
rs6073079
rs11658240
rs116473015
rs7224129
rs2250246
rs645554
rs72897237
All
0.0
0.2
0.4
MR leave−one−out sensitivity analysis for
'IgD− CD27− %B cell' on 'Juvenile arthritis'

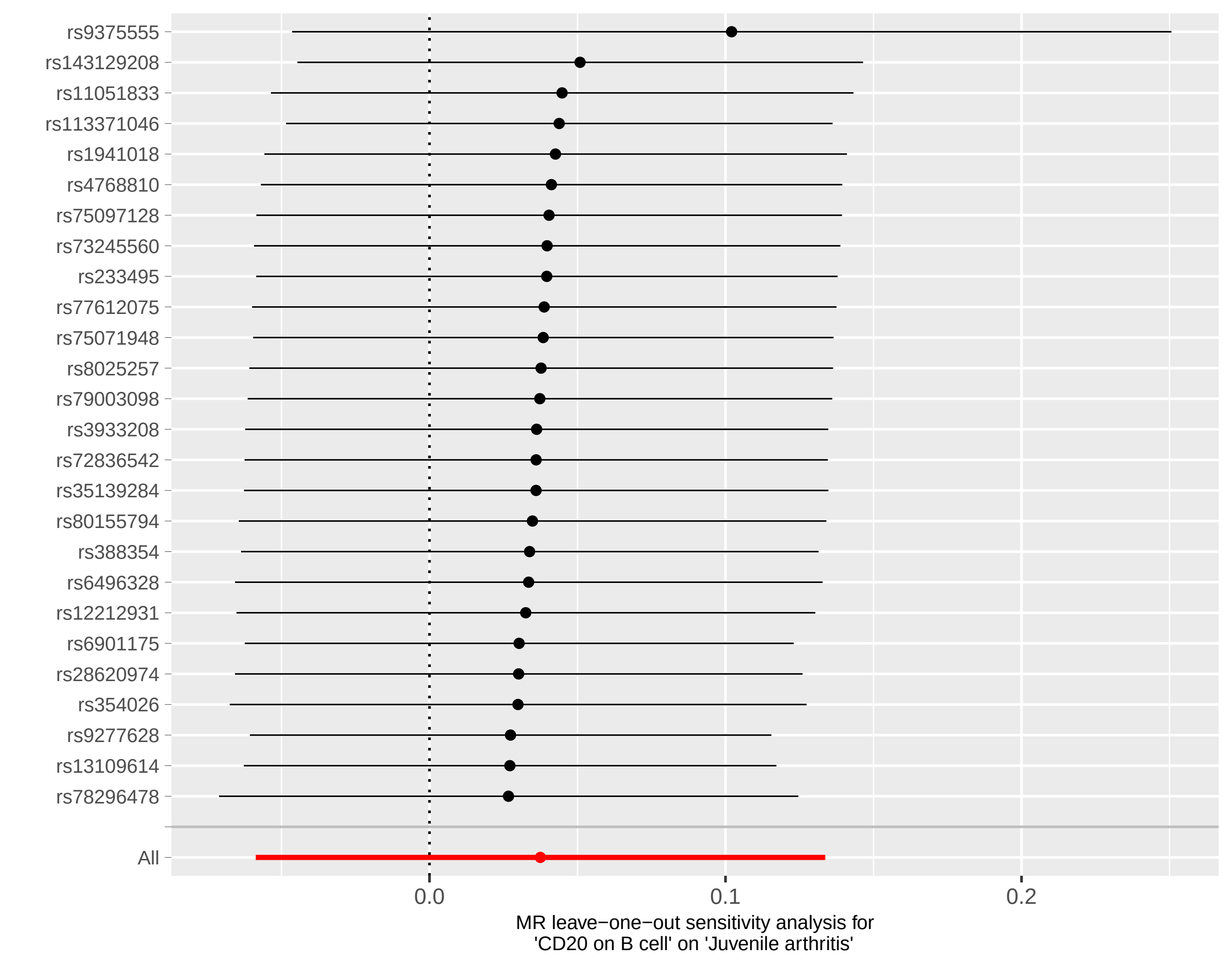

rs9375555
rs143129208
rs11051833
rs113371046
rs1941018
rs4768810
rs75097128
rs73245560
rs233495
rs77612075
rs75071948
rs8025257
rs79003098
rs3933208
rs72836542
rs35139284
rs80155794
rs388354
rs6496328
rs12212931
rs6901175
rs28620974
rs354026
rs9277628
rs13109614
rs78296478
All
0.0
0.1
0.2
MR leave−one−out sensitivity analysis for
'CD20 on B cell' on 'Juvenile arthritis'

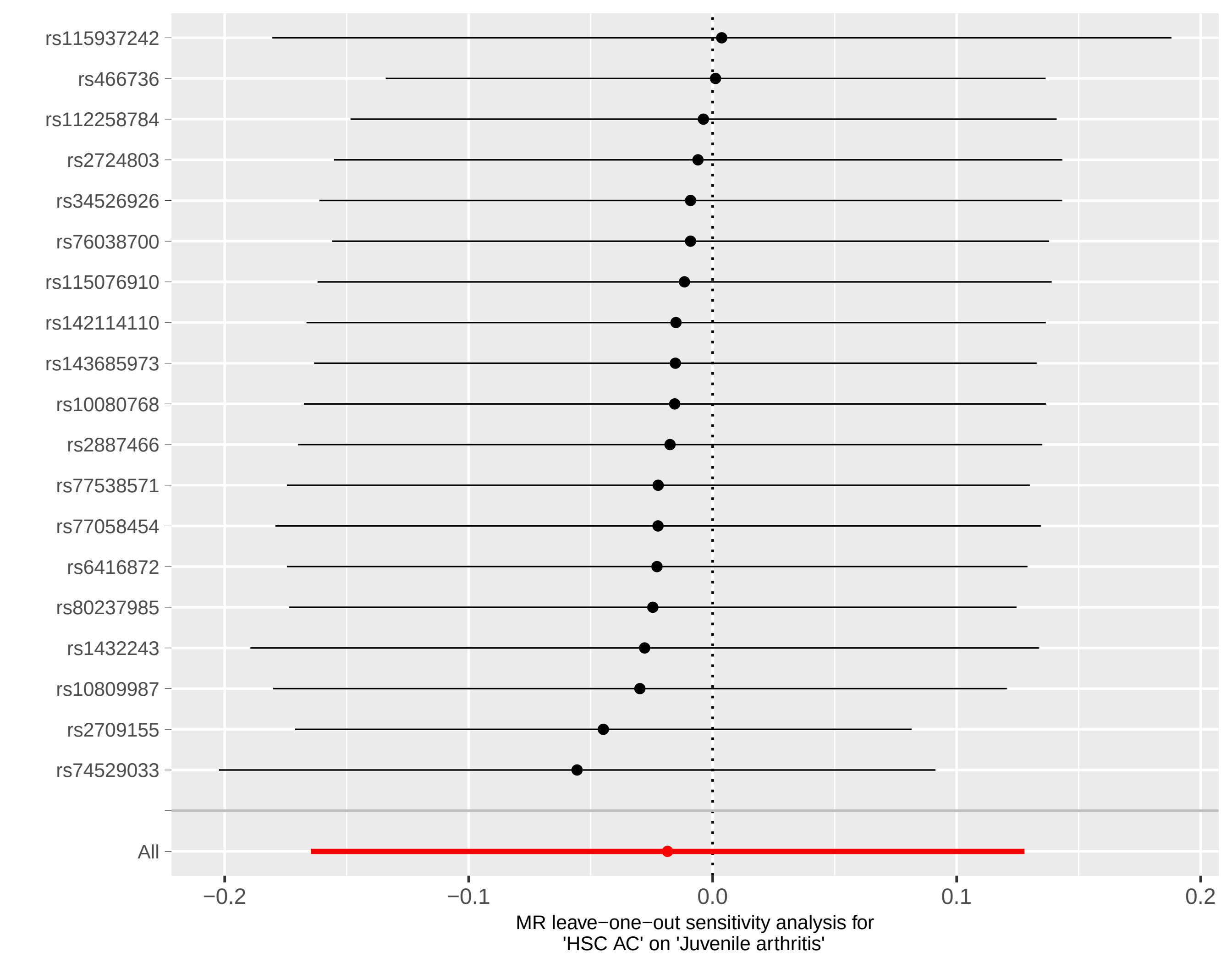
rs115937242
rs466736
rs112258784
rs2724803
rs34526926
rs76038700
rs115076910
rs142114110
rs143685973
rs10080768
rs2887466
rs77538571
rs77058454
rs6416872
rs80237985
rs1432243
rs10809987
rs2709155
rs74529033
All
−0.2
−0.1
0.0
0.1
0.2
MR leave−one−out sensitivity analysis for
'HSC AC' on 'Juvenile arthritis'

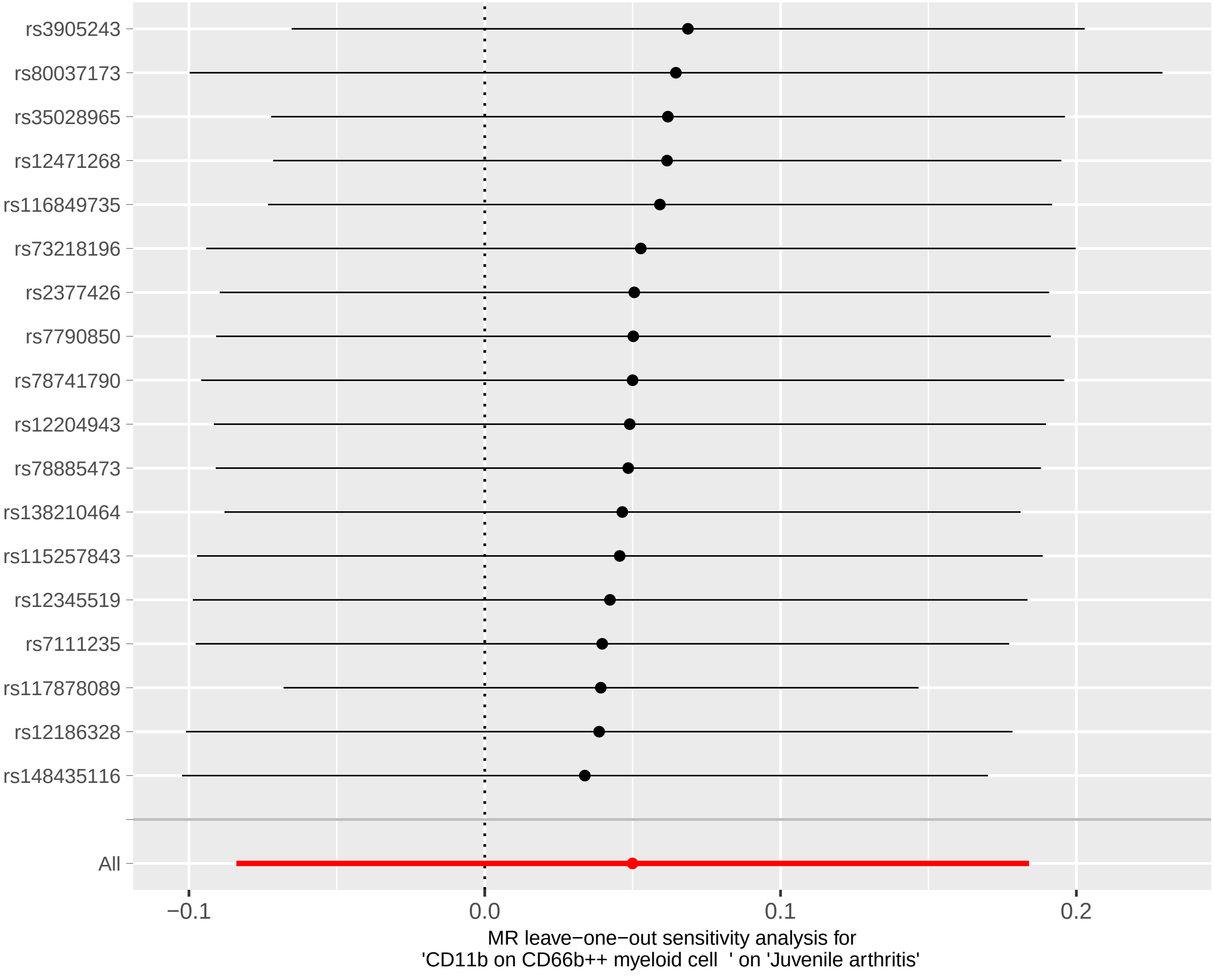

rs3905243
rs80037173
rs35028965
rs12471268
rs116849735
rs73218196
rs2377426
rs7790850
rs78741790
rs12204943
rs78885473
rs138210464
rs115257843
rs12345519
rs7111235
rs117878089
rs12186328
rs148435116
All
−0.1
0.0
0.1
0.2
MR leave−one−out sensitivity analysis for
'CD11b on CD66b++ myeloid cell ' on 'Juvenile arthritis'

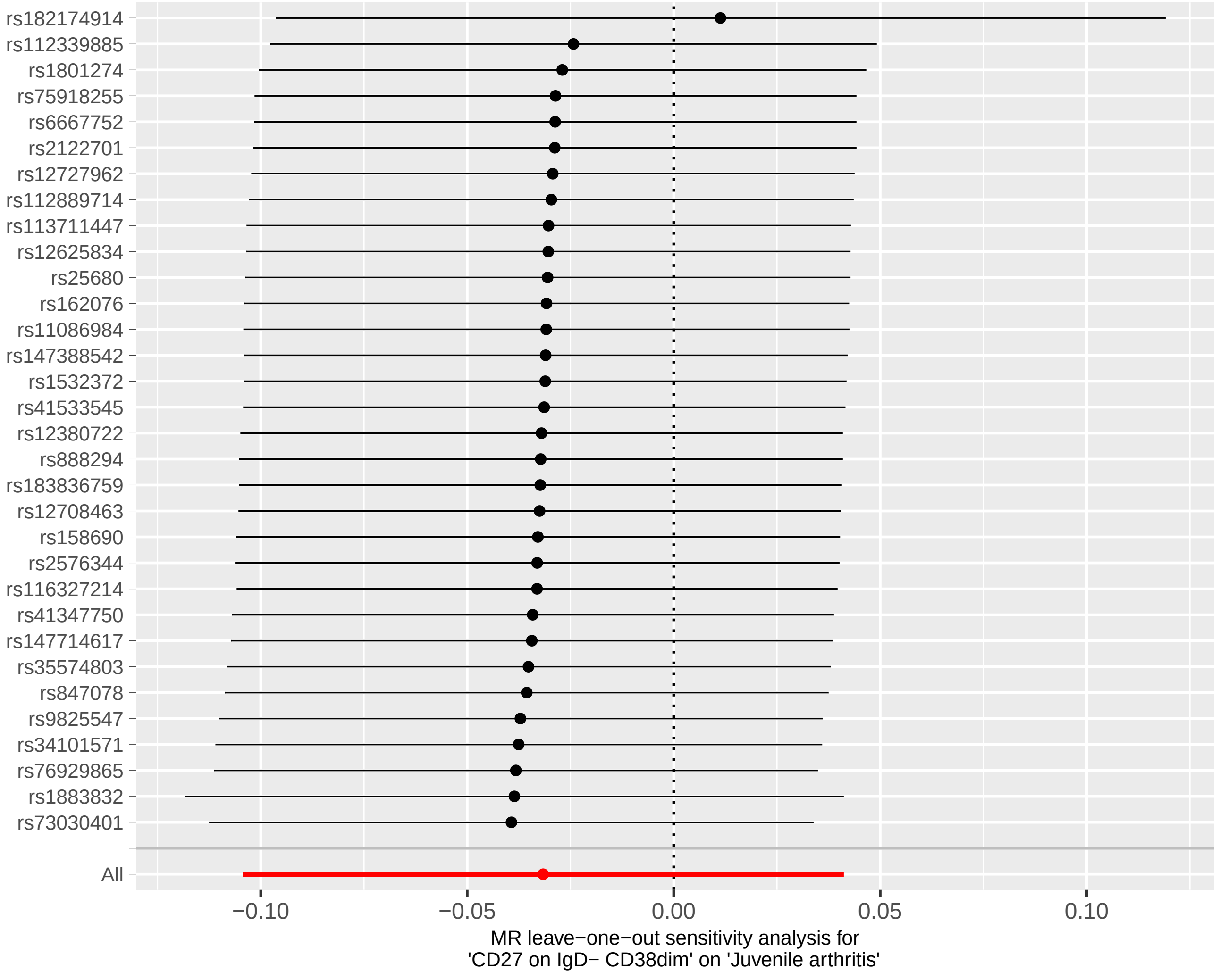

rs182174914
rs112339885
rs1801274
rs75918255
rs6667752
rs2122701
rs12727962
rs112889714
rs113711447
rs12625834
rs25680
rs162076
rs11086984
rs147388542
rs1532372
rs41533545
rs12380722
rs888294
rs183836759
rs12708463
rs158690
rs2576344
rs116327214
rs41347750
rs147714617
rs35574803
rs847078
rs9825547
rs34101571
rs76929865
rs1883832
rs73030401
All
−0.10
−0.05
0.00
0.05
0.10
MR leave−one−out sensitivity analysis for
'CD27 on IgD− CD38dim' on 'Juvenile arthritis'

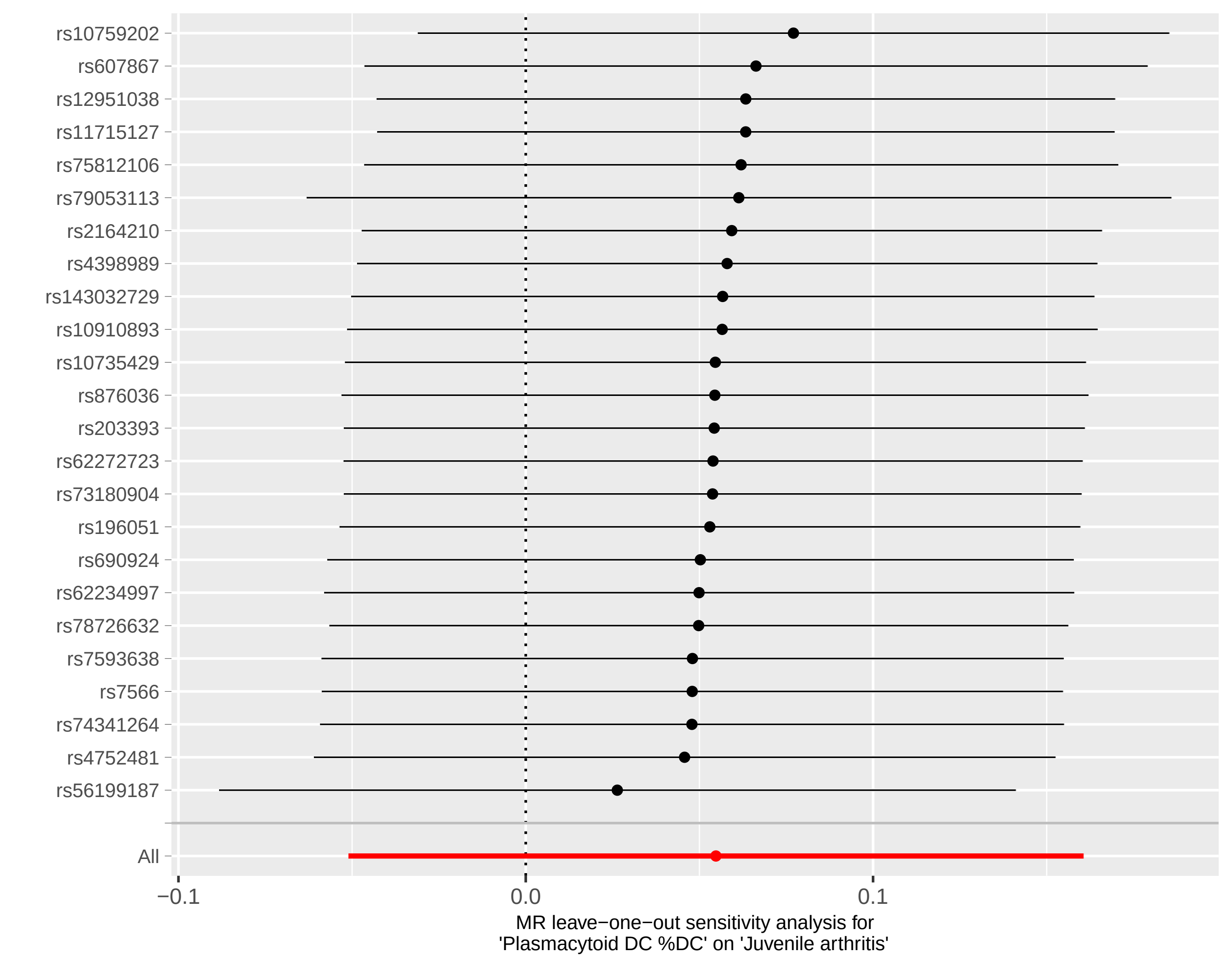
rs10759202
rs607867
rs12951038
rs11715127
rs75812106
rs79053113
rs2164210
rs4398989
rs143032729
rs10910893
rs10735429
rs876036
rs203393
rs62272723
rs73180904
rs196051
rs690924
rs62234997
rs78726632
rs7593638
rs7566
rs74341264
rs4752481
rs56199187
All
−0.1
0.0
0.1
MR leave−one−out sensitivity analysis for
'Plasmacytoid DC %DC' on 'Juvenile arthritis'

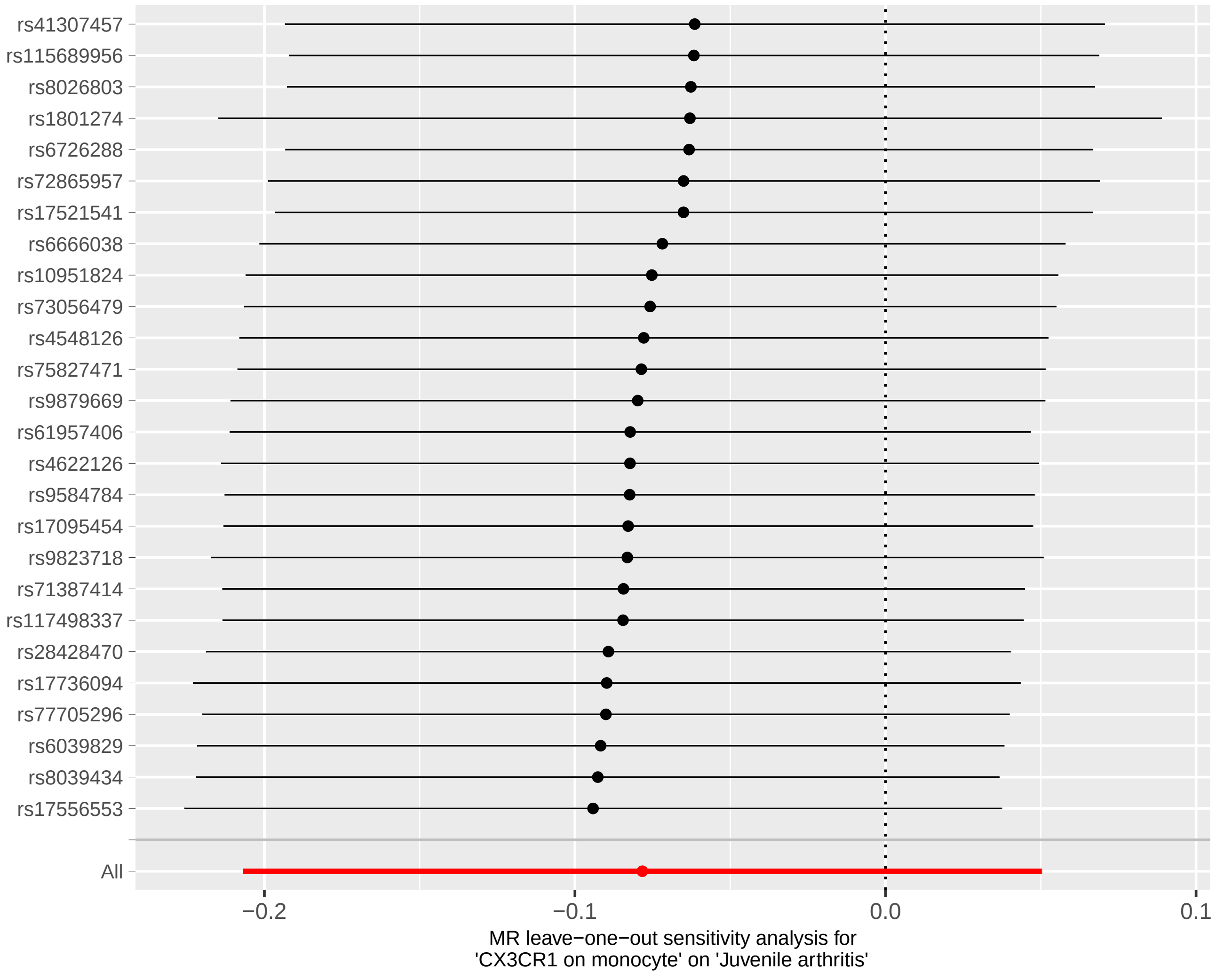
rs41307457
rs115689956
rs8026803
rs1801274
rs6726288
rs72865957
rs17521541
rs6666038
rs10951824
rs73056479
rs4548126
rs75827471
rs9879669
rs61957406
rs4622126
rs9584784
rs17095454
rs9823718
rs71387414
rs117498337
rs28428470
rs17736094
rs77705296
rs6039829
rs8039434
rs17556553
All
−0.2
−0.1
0.0
0.1
MR leave−one−out sensitivity analysis for
'CX3CR1 on monocyte' on 'Juvenile arthritis'

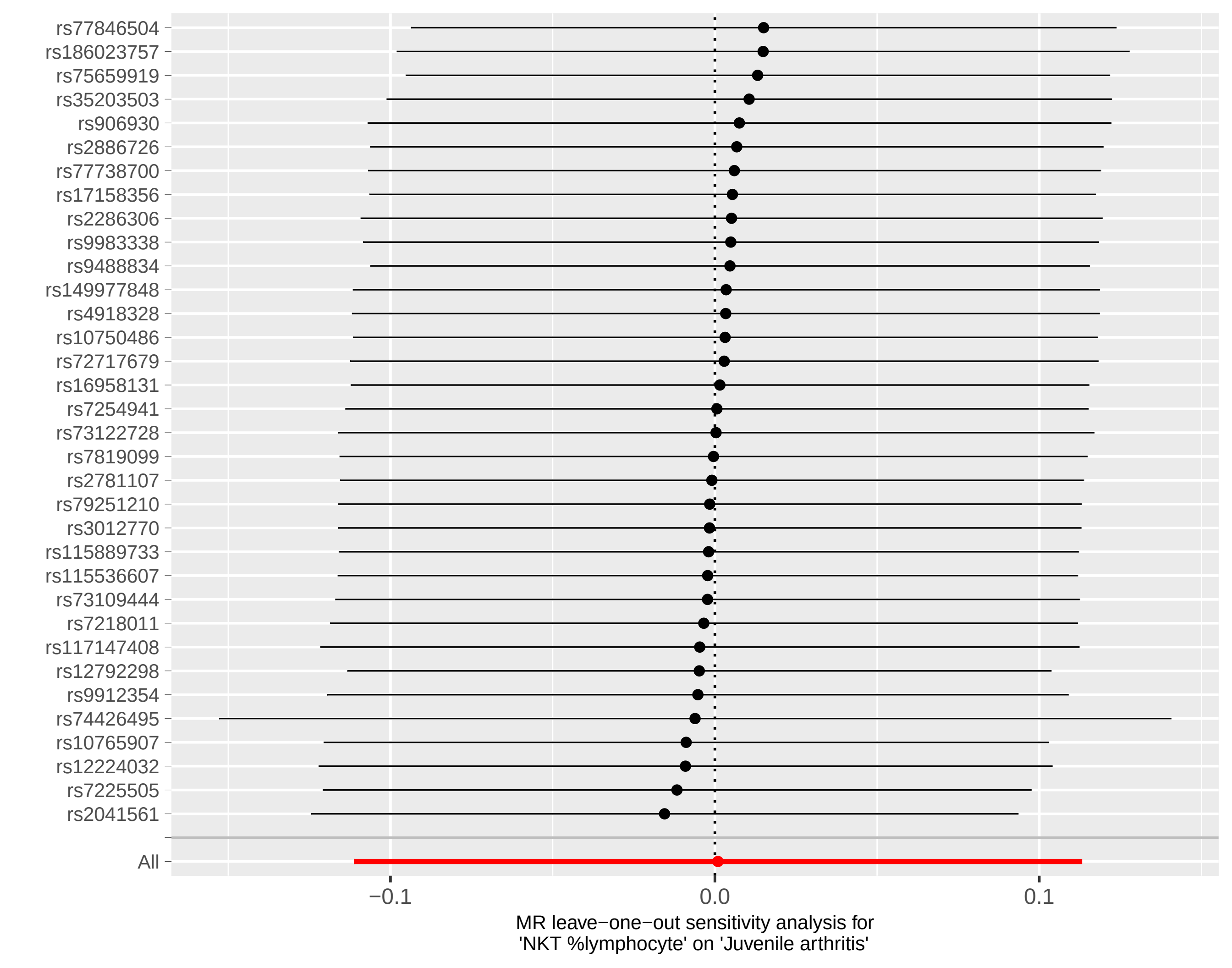
rs77846504
rs186023757
rs75659919
rs35203503
rs906930
rs2886726
rs77738700
rs17158356
rs2286306
rs9983338
rs9488834
rs149977848
rs4918328
rs10750486
rs72717679
rs16958131
rs7254941
rs73122728
rs7819099
rs2781107
rs79251210
rs3012770
rs115889733
rs115536607
rs73109444
rs7218011
rs117147408
rs12792298
rs9912354
rs74426495
rs10765907
rs12224032
rs7225505
rs2041561
All
−0.1
0.0
0.1
MR leave−one−out sensitivity analysis for
'NKT %lymphocyte' on 'Juvenile arthritis'

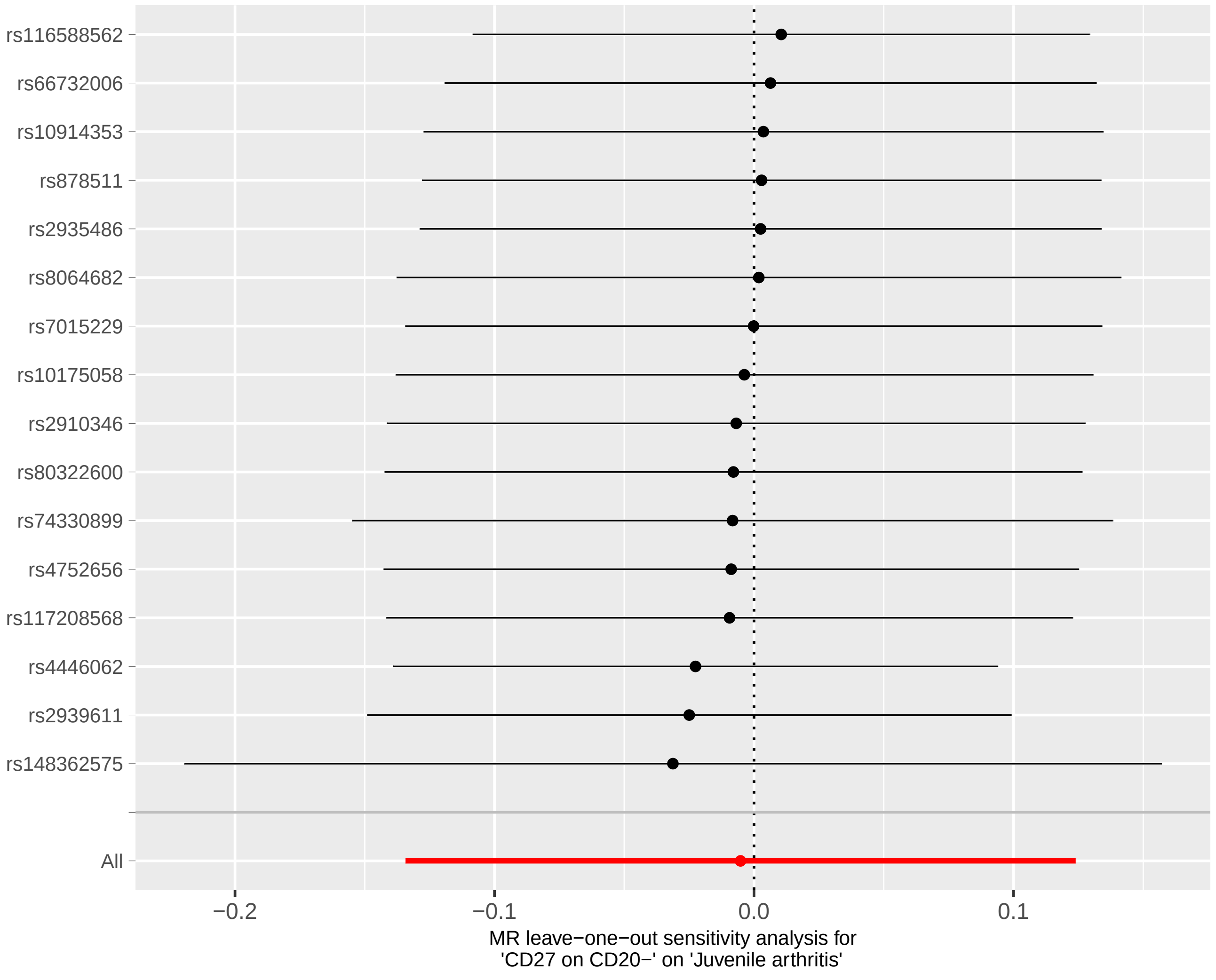
rs116588562
rs66732006
rs10914353
rs878511
rs2935486
rs8064682
rs7015229
rs10175058
rs2910346
rs80322600
rs74330899
rs4752656
rs117208568
rs4446062
rs2939611
rs148362575
All
−0.2
−0.1
0.0
0.1
MR leave−one−out sensitivity analysis for
'CD27 on CD20−' on 'Juvenile arthritis'

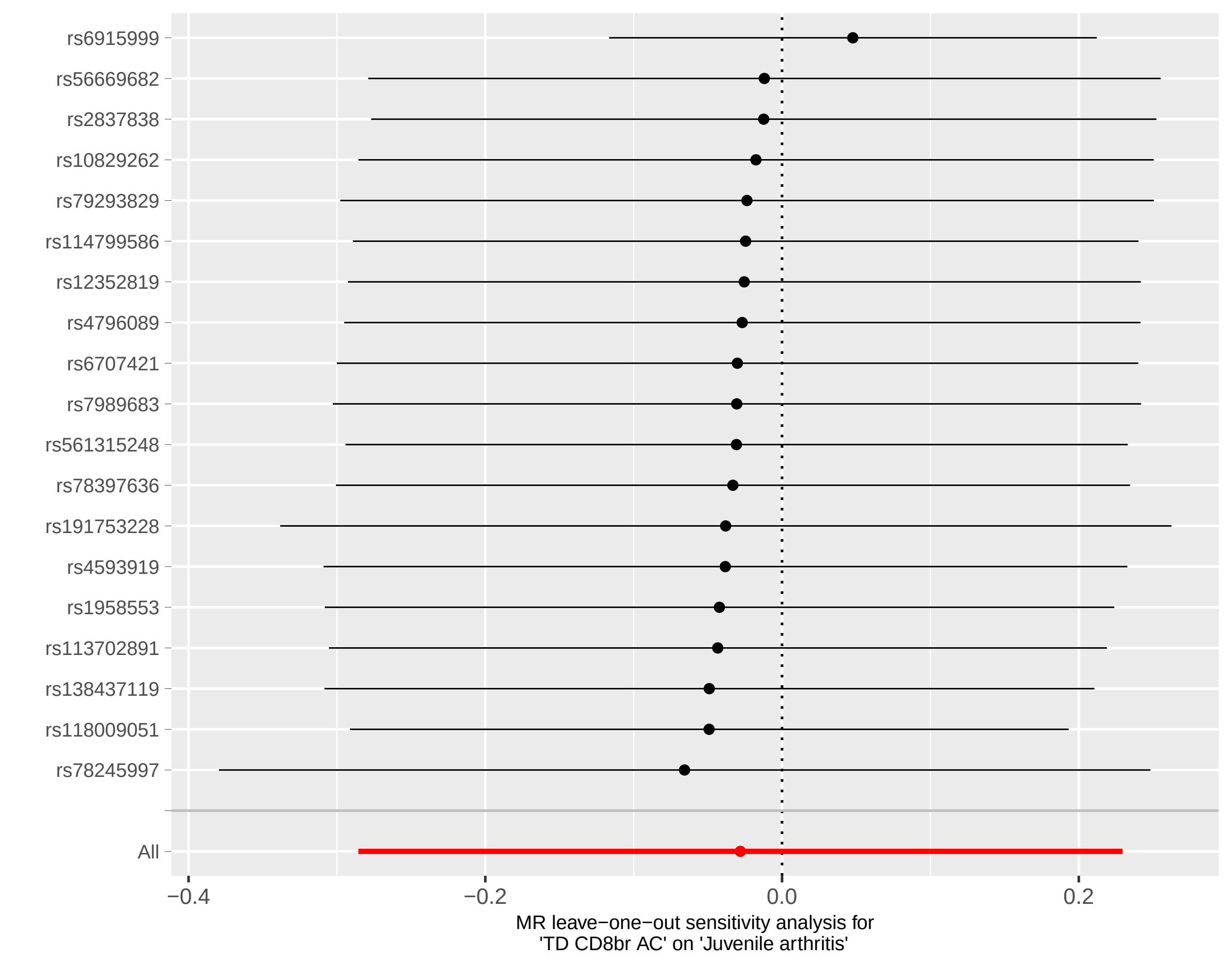

rs6915999
rs56669682
rs2837838
rs10829262
rs79293829
rs114799586
rs12352819
rs4796089
rs6707421
rs7989683
rs561315248
rs78397636
rs191753228
rs4593919
rs1958553
rs113702891
rs138437119
rs118009051
rs78245997
All
−0.4
−0.2
0.0
0.2
MR leave−one−out sensitivity analysis for
'TD CD8br AC' on 'Juvenile arthritis'

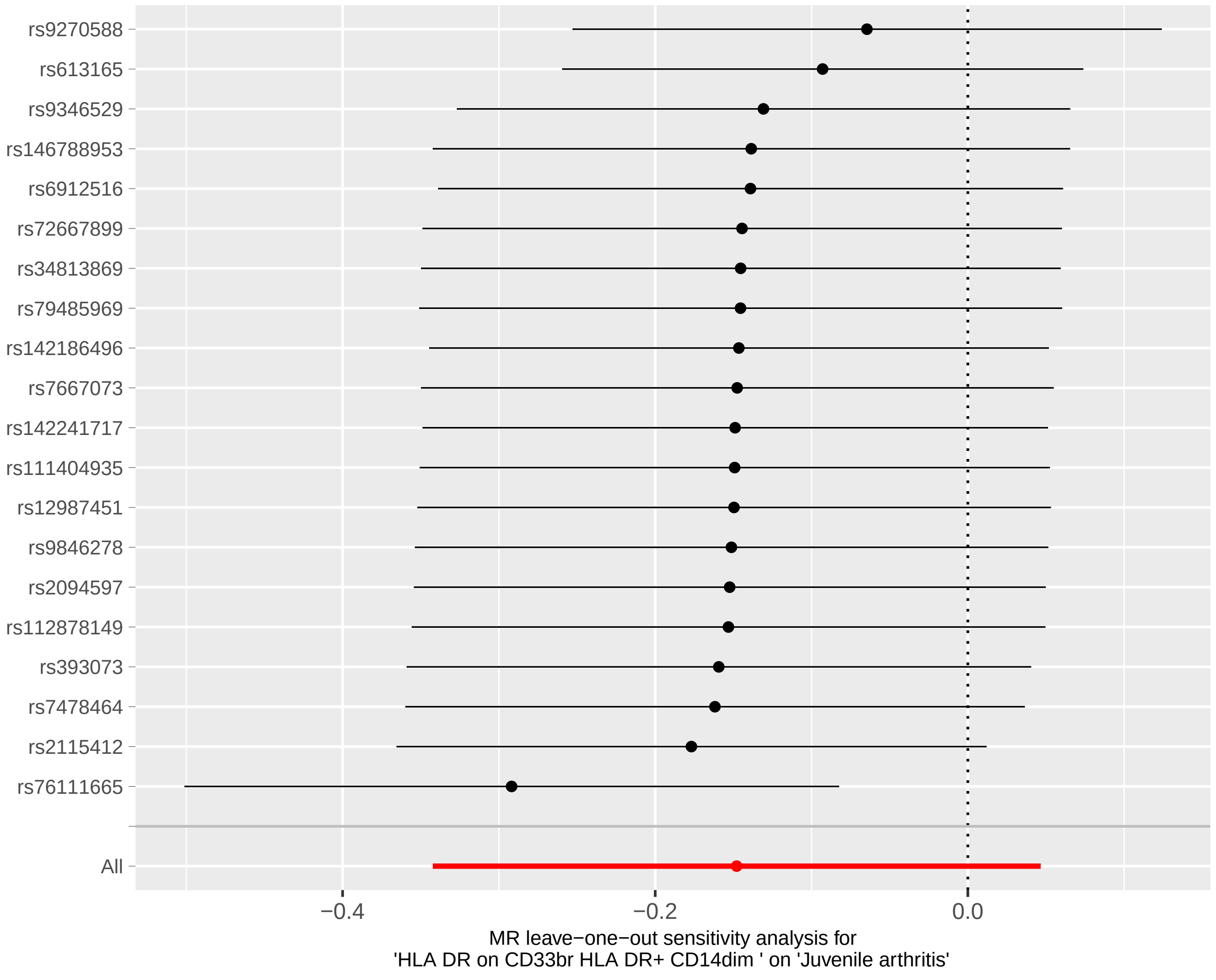

rs9270588
rs613165
rs9346529
rs146788953
rs6912516
rs72667899
rs34813869
rs79485969
rs142186496
rs7667073
rs142241717
rs111404935
rs12987451
rs9846278
rs2094597
rs112878149
rs393073
rs7478464
rs2115412
rs76111665
All
−0.4
−0.2
0.0
MR leave−one−out sensitivity analysis for
'HLA DR on CD33br HLA DR+ CD14dim ' on 'Juvenile arthritis'

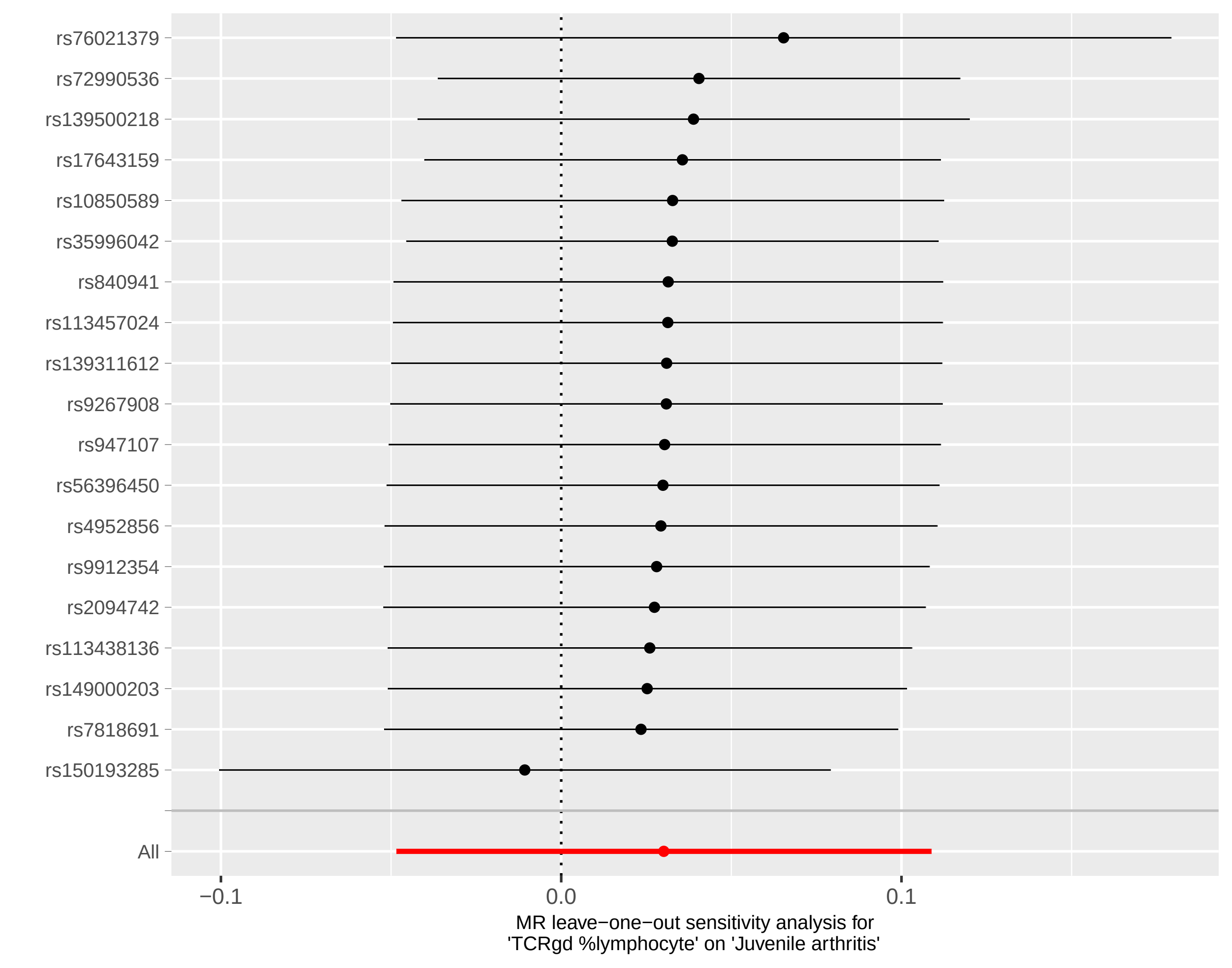

rs76021379
rs72990536
rs139500218
rs17643159
rs10850589
rs35996042
rs840941
rs113457024
rs139311612
rs9267908
rs947107
rs56396450
rs4952856
rs9912354
rs2094742
rs113438136
rs149000203
rs7818691
rs150193285
All
−0.1
0.0
0.1
MR leave−one−out sensitivity analysis for
'TCRgd %lymphocyte' on 'Juvenile arthritis'

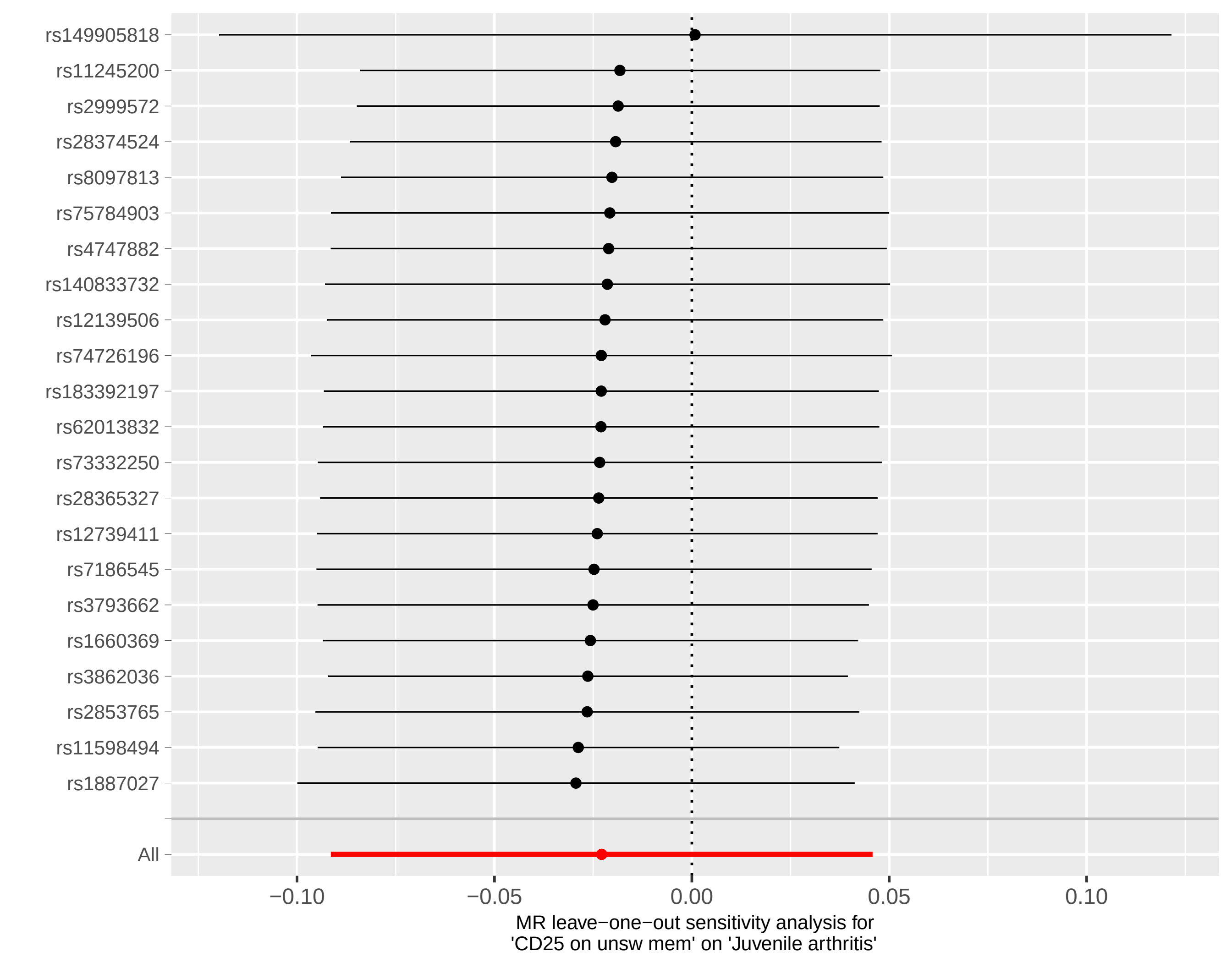

rs149905818
rs11245200
rs2999572
rs28374524
rs8097813
rs75784903
rs4747882
rs140833732
rs12139506
rs74726196
rs183392197
rs62013832
rs73332250
rs28365327
rs12739411
rs7186545
rs3793662
rs1660369
rs3862036
rs2853765
rs11598494
rs1887027
All
−0.10
−0.05
0.00
0.05
0.10
MR leave−one−out sensitivity analysis for
'CD25 on unsw mem' on 'Juvenile arthritis'

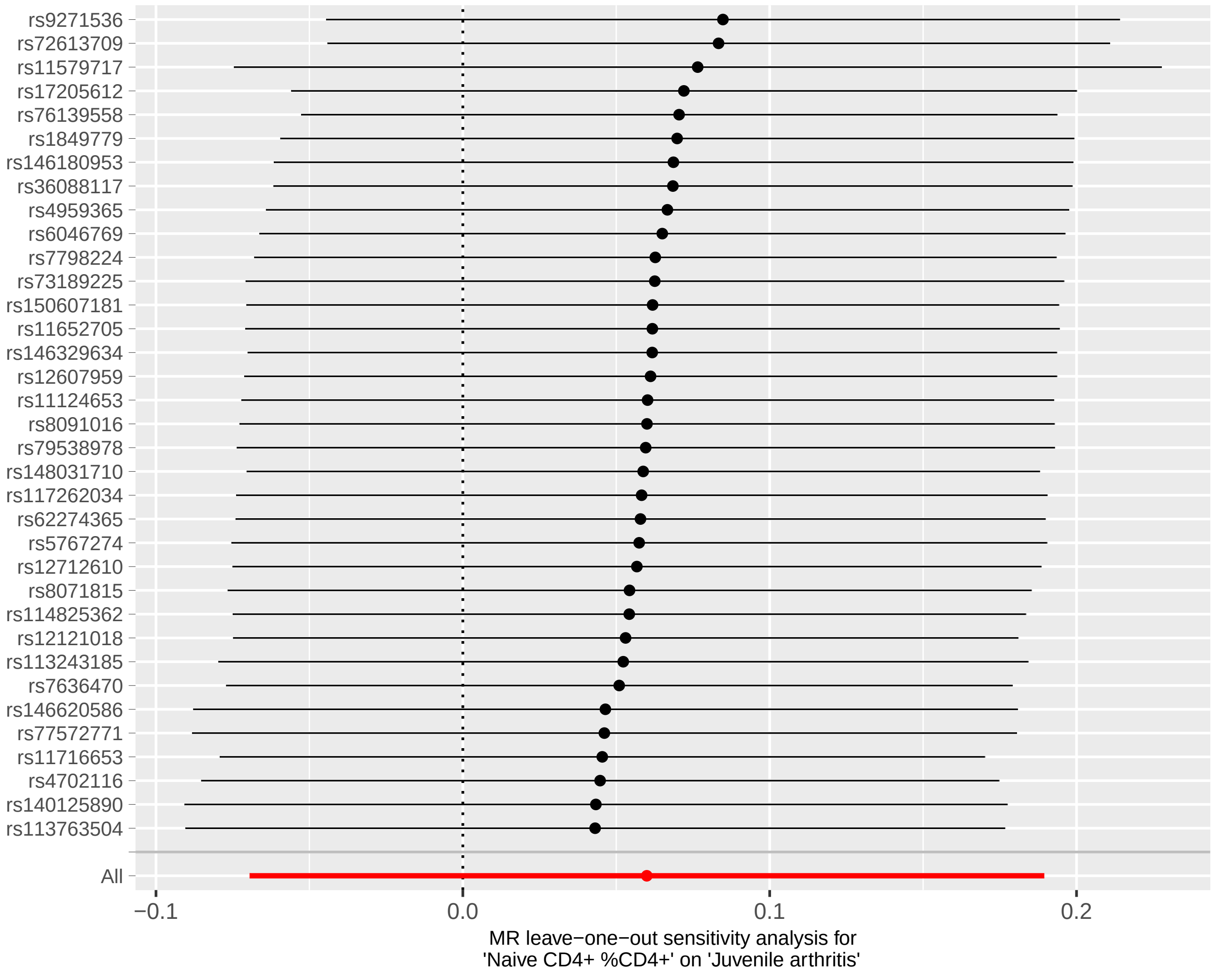
rs9271536
rs72613709
rs11579717
rs17205612
rs76139558
rs1849779
rs146180953
rs36088117
rs4959365
rs6046769
rs7798224
rs73189225
rs150607181
rs11652705
rs146329634
rs12607959
rs11124653
rs8091016
rs79538978
rs148031710
rs117262034
rs62274365
rs5767274
rs12712610
rs8071815
rs114825362
rs12121018
rs113243185
rs7636470
rs146620586
rs77572771
rs11716653
rs4702116
rs140125890
rs113763504
All
−0.1
0.0
0.1
0.2
MR leave−one−out sensitivity analysis for
'Naive CD4+ %CD4+' on 'Juvenile arthritis'

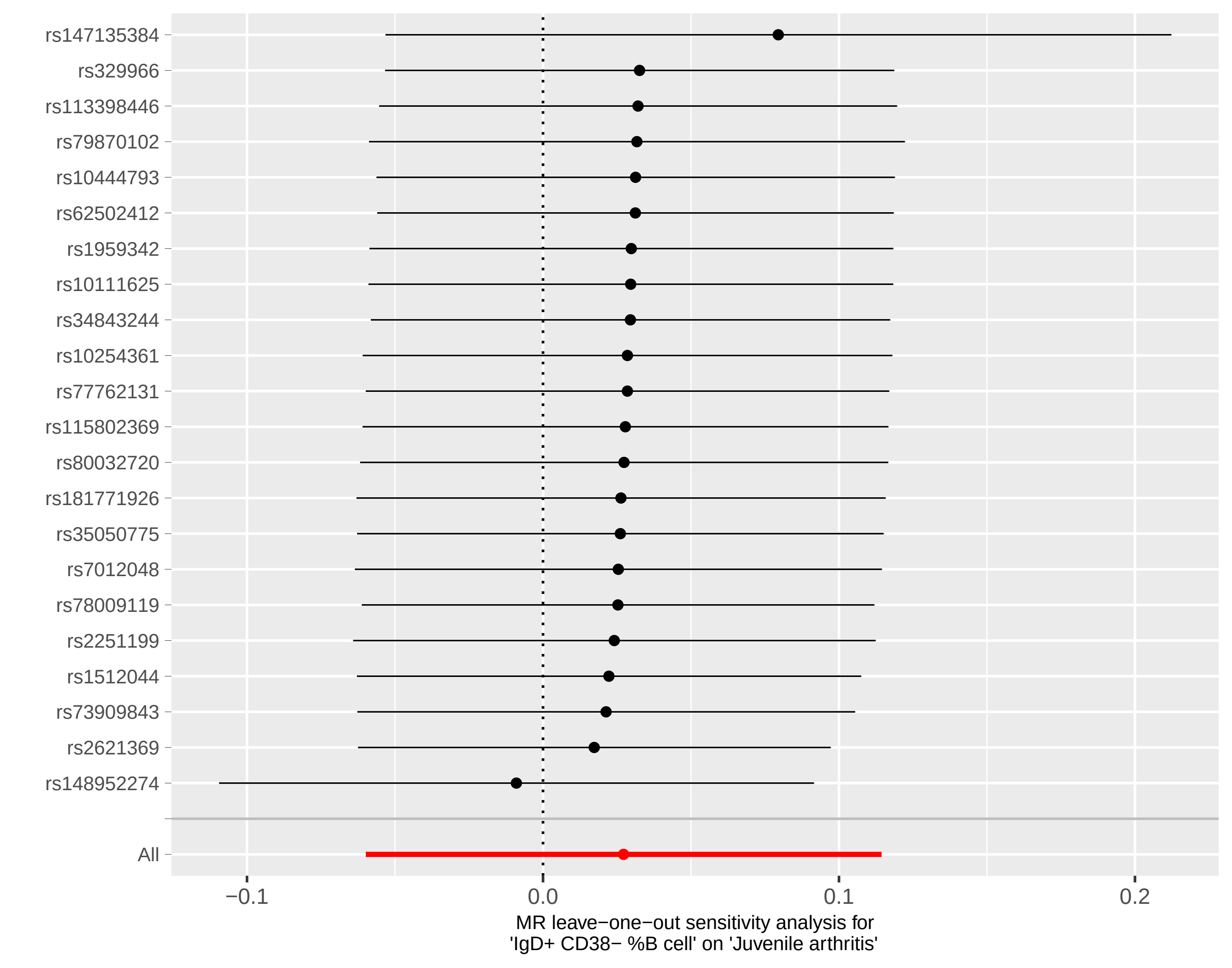
rs147135384
rs329966
rs113398446
rs79870102
rs10444793
rs62502412
rs1959342
rs10111625
rs34843244
rs10254361
rs77762131
rs115802369
rs80032720
rs181771926
rs35050775
rs7012048
rs78009119
rs2251199
rs1512044
rs73909843
rs2621369
rs148952274
All
−0.1
0.0
0.1
0.2
MR leave−one−out sensitivity analysis for
'IgD+ CD38− %B cell' on 'Juvenile arthritis'

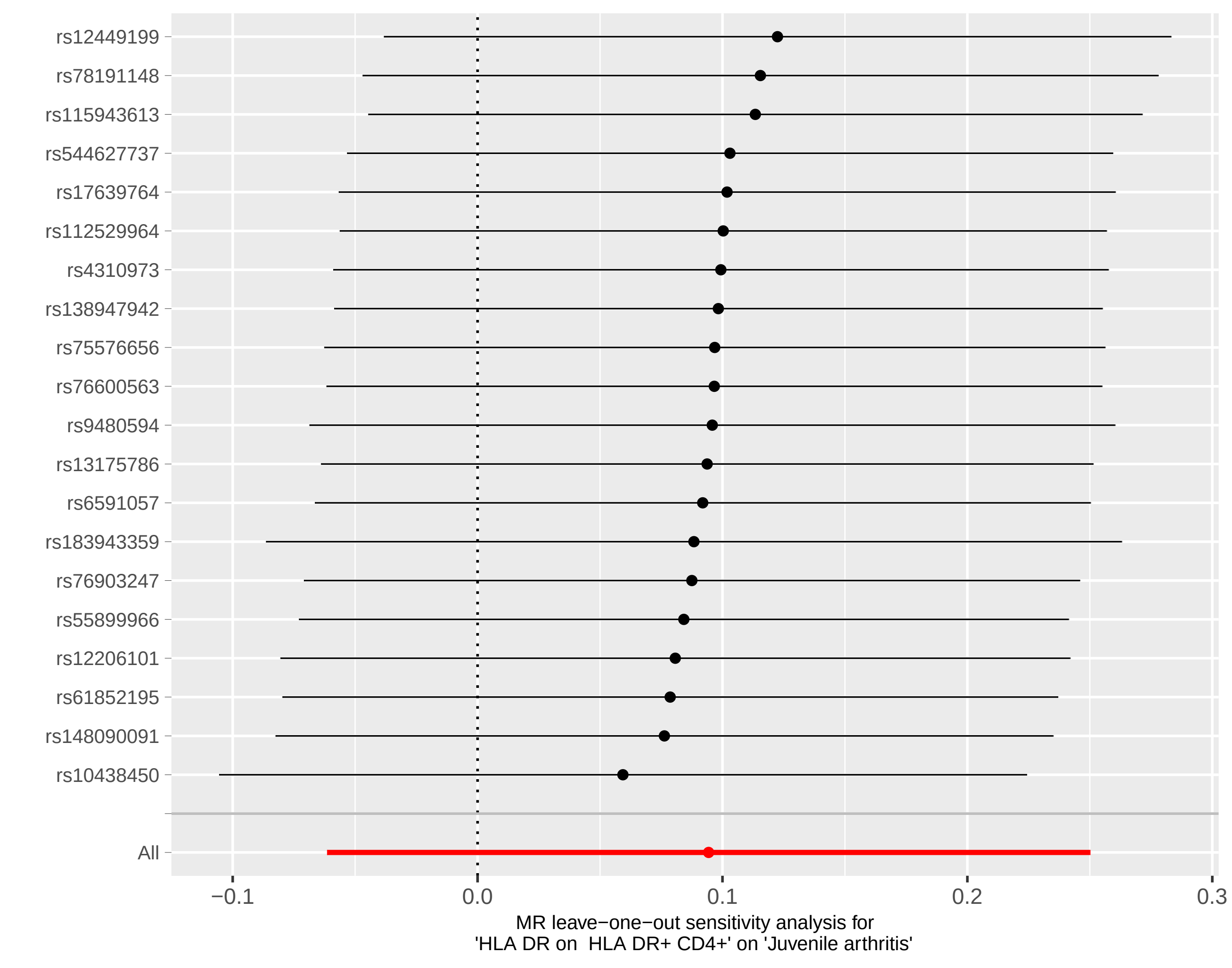
rs12449199
rs78191148
rs115943613
rs544627737
rs17639764
rs112529964
rs4310973
rs138947942
rs75576656
rs76600563
rs9480594
rs13175786
rs6591057
rs183943359
rs76903247
rs55899966
rs12206101
rs61852195
rs148090091
rs10438450
All
−0.1
0.0
0.1
0.2
0.3
MR leave−one−out sensitivity analysis for
'HLA DR on HLA DR+ CD4+' on 'Juvenile arthritis'

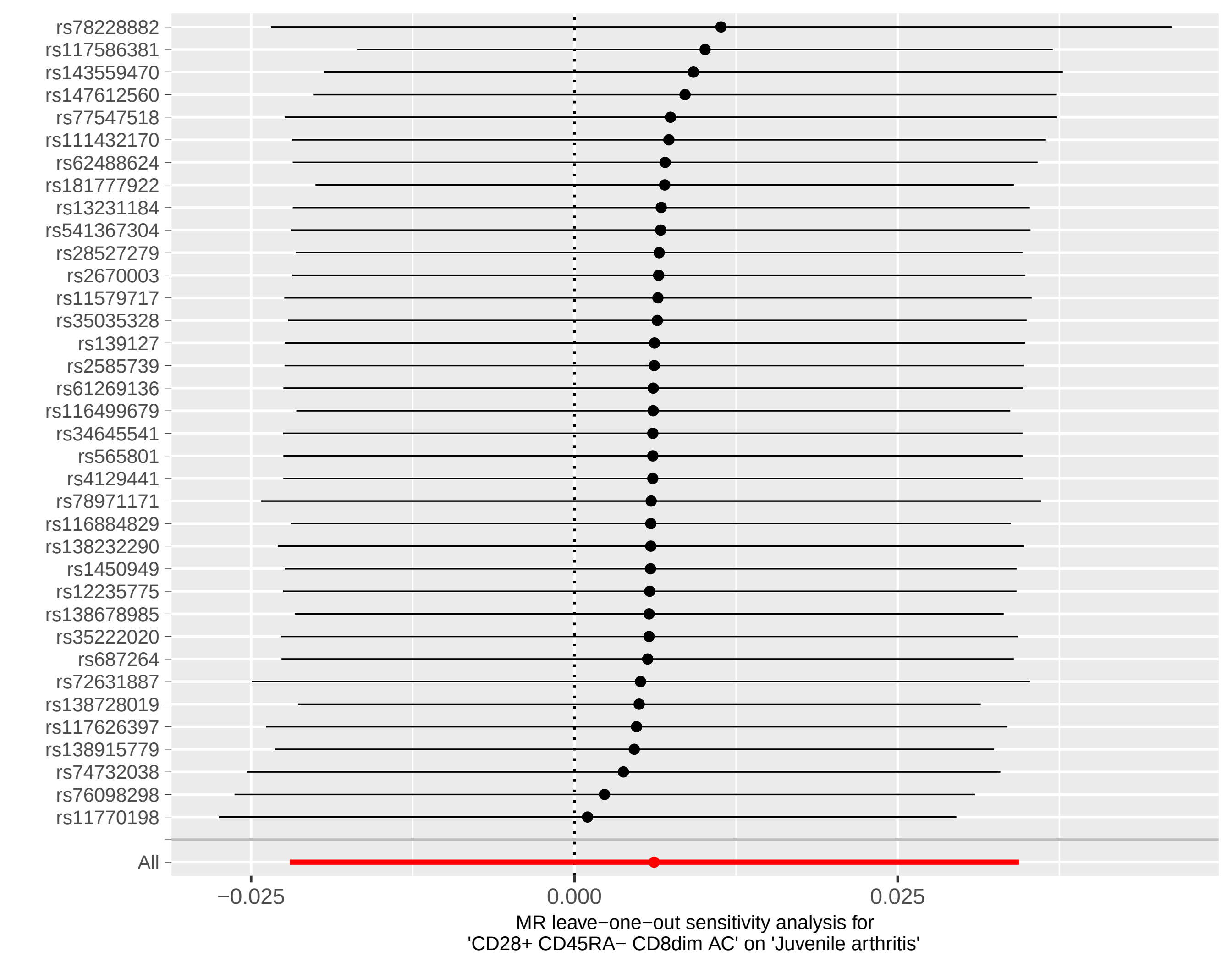
rs78228882
rs117586381
rs143559470
rs147612560
rs77547518
rs111432170
rs62488624
rs181777922
rs13231184
rs541367304
rs28527279
rs2670003
rs11579717
rs35035328
rs139127
rs2585739
rs61269136
rs116499679
rs34645541
rs565801
rs4129441
rs78971171
rs116884829
rs138232290
rs1450949
rs12235775
rs138678985
rs35222020
rs687264
rs72631887
rs138728019
rs117626397
rs138915779
rs74732038
rs76098298
rs11770198
All
−0.025
0.000
0.025
MR leave−one−out sensitivity analysis for
'CD28+ CD45RA− CD8dim AC' on 'Juvenile arthritis'

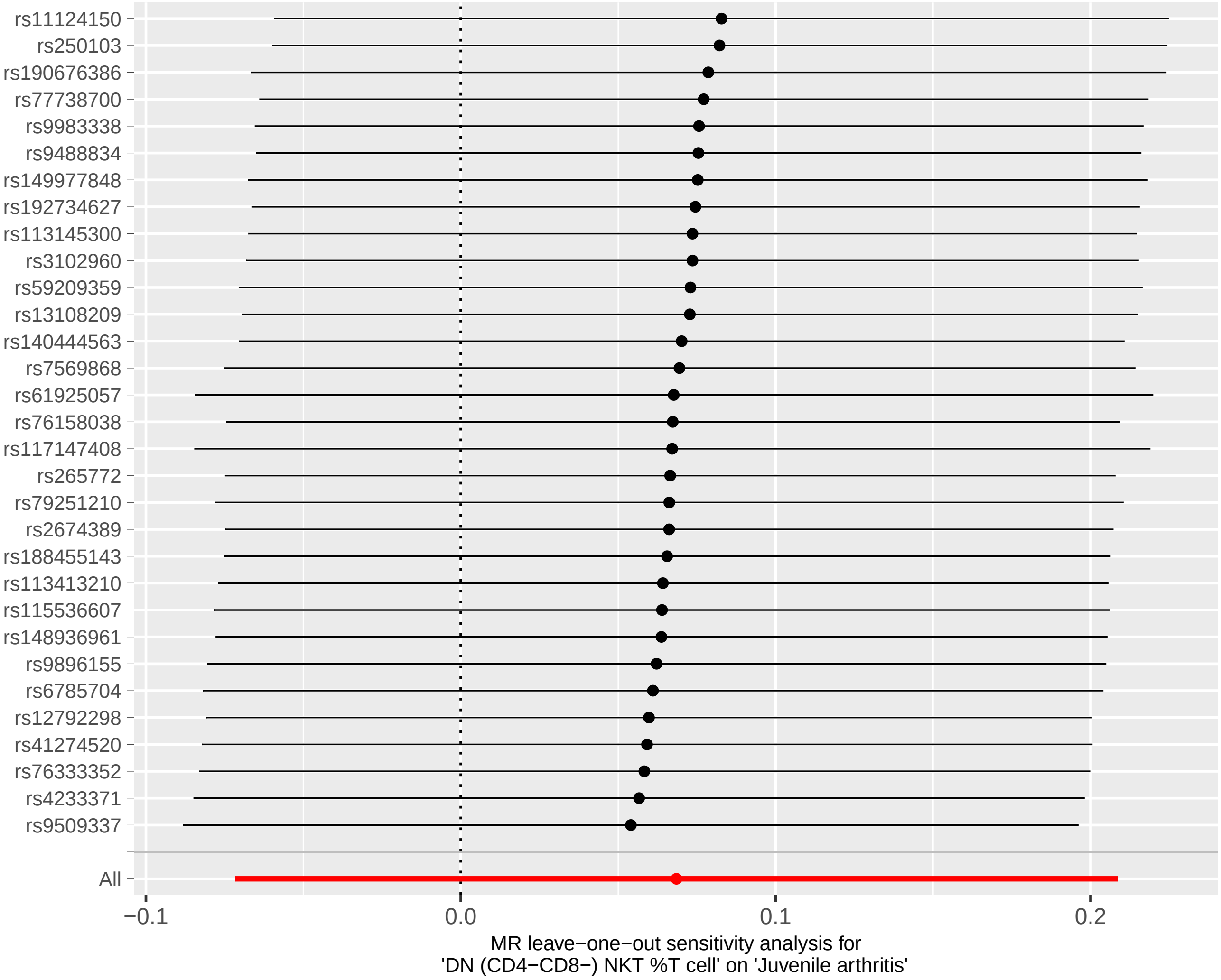
rs11124150
rs250103
rs190676386
rs77738700
rs9983338
rs9488834
rs149977848
rs192734627
rs113145300
rs3102960
rs59209359
rs13108209
rs140444563
rs7569868
rs61925057
rs76158038
rs117147408
rs265772
rs79251210
rs2674389
rs188455143
rs113413210
rs115536607
rs148936961
rs9896155
rs6785704
rs12792298
rs41274520
rs76333352
rs4233371
rs9509337
All
−0.1
0.0
0.1
0.2
MR leave−one−out sensitivity analysis for
'DN (CD4−CD8−) NKT %T cell' on 'Juvenile arthritis'

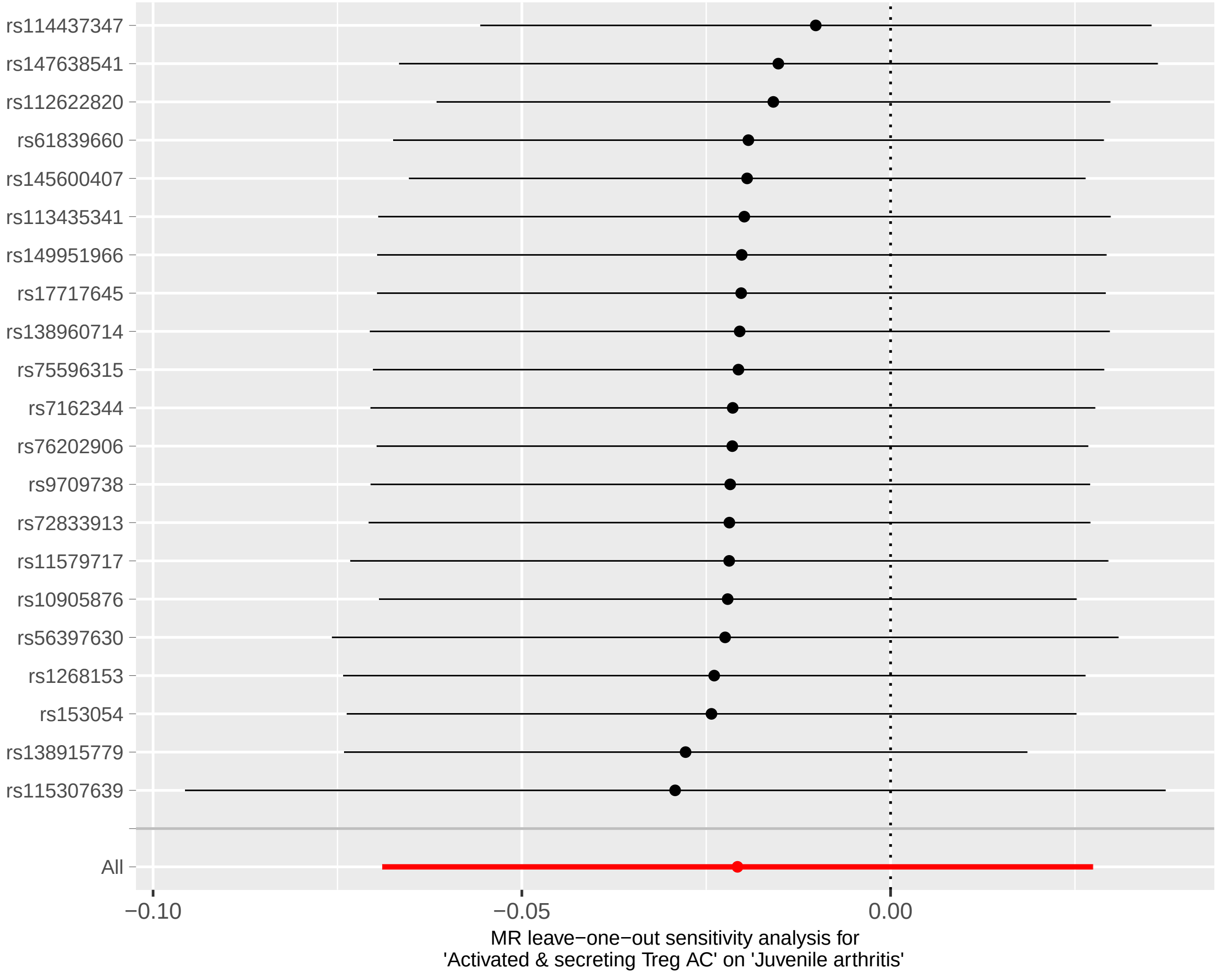

rs114437347
rs147638541
rs112622820
rs61839660
rs145600407
rs113435341
rs149951966
rs17717645
rs138960714
rs75596315
rs7162344
rs76202906
rs9709738
rs72833913
rs11579717
rs10905876
rs56397630
rs1268153
rs153054
rs138915779
rs115307639
All
−0.10
−0.05
0.00
MR leave−one−out sensitivity analysis for
'Activated & secreting Treg AC' on 'Juvenile arthritis'

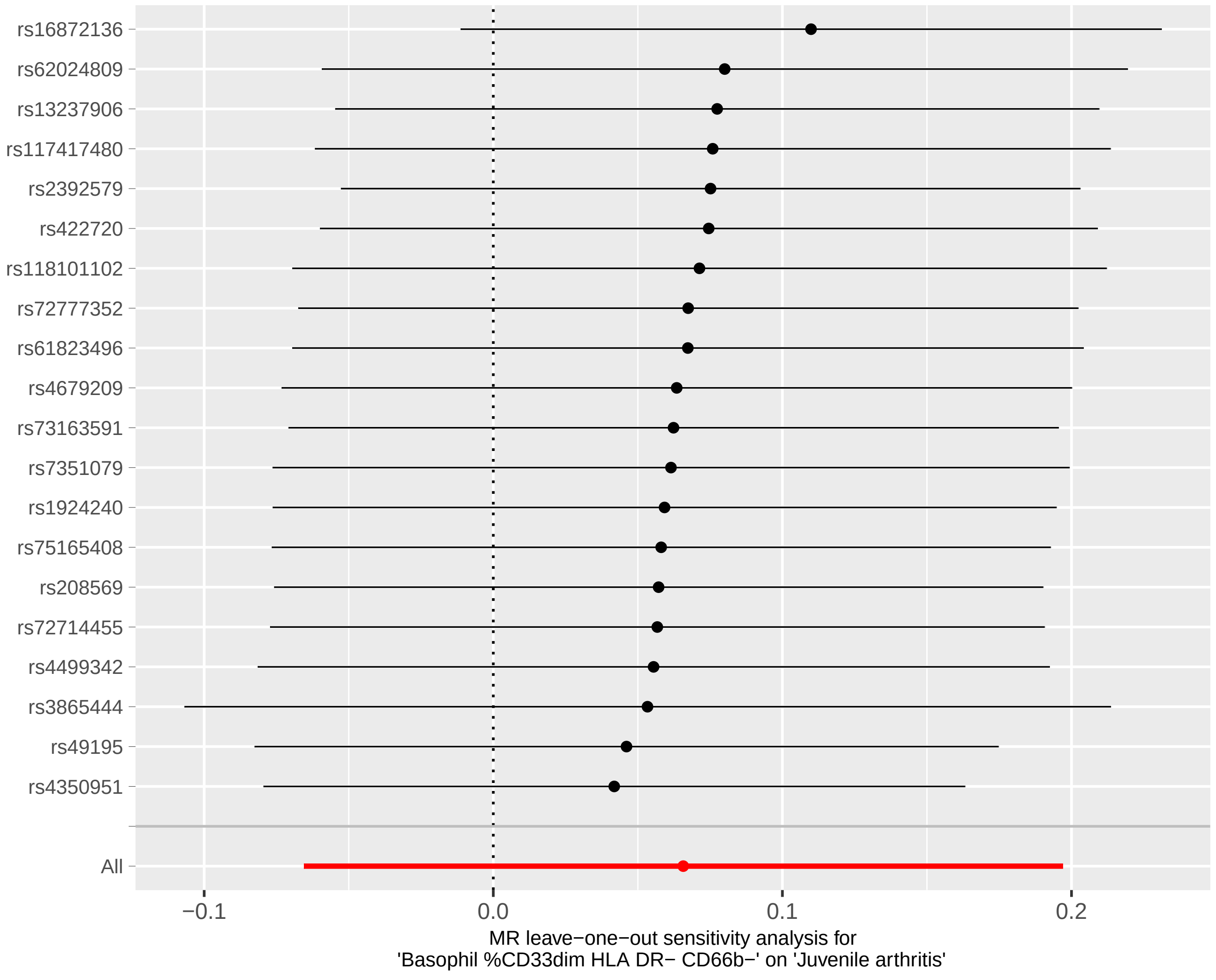
rs16872136
rs62024809
rs13237906
rs117417480
rs2392579
rs422720
rs118101102
rs72777352
rs61823496
rs4679209
rs73163591
rs7351079
rs1924240
rs75165408
rs208569
rs72714455
rs4499342
rs3865444
rs49195
rs4350951
All
−0.1
0.0
0.1
0.2
MR leave−one−out sensitivity analysis for
'Basophil %CD33dim HLA DR− CD66b−' on 'Juvenile arthritis'

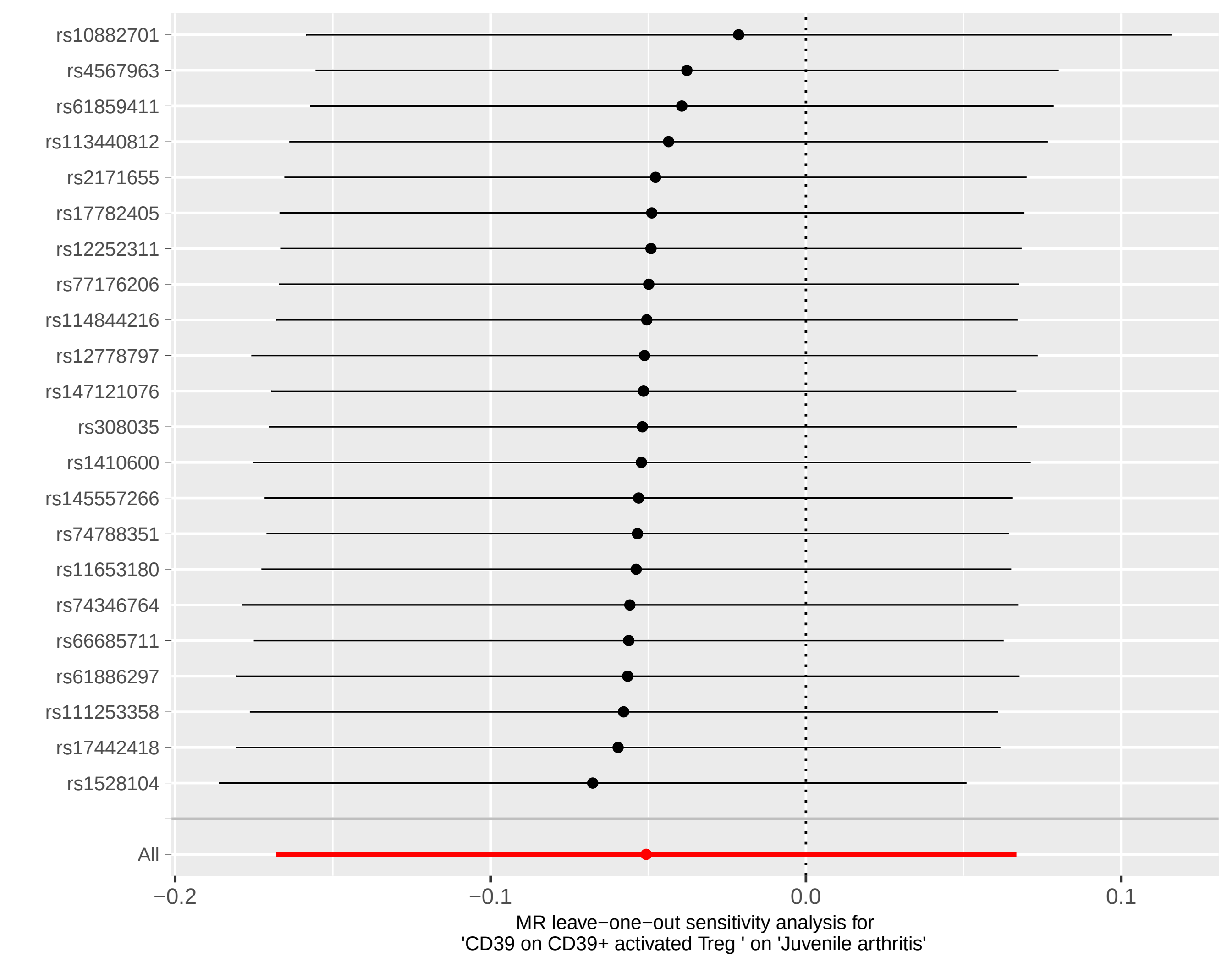
rs10882701
rs4567963
rs61859411
rs113440812
rs2171655
rs17782405
rs12252311
rs77176206
rs114844216
rs12778797
rs147121076
rs308035
rs1410600
rs145557266
rs74788351
rs11653180
rs74346764
rs66685711
rs61886297
rs111253358
rs17442418
rs1528104
All
−0.2
−0.1
0.0
0.1
MR leave−one−out sensitivity analysis for
'CD39 on CD39+ activated Treg ' on 'Juvenile arthritis'

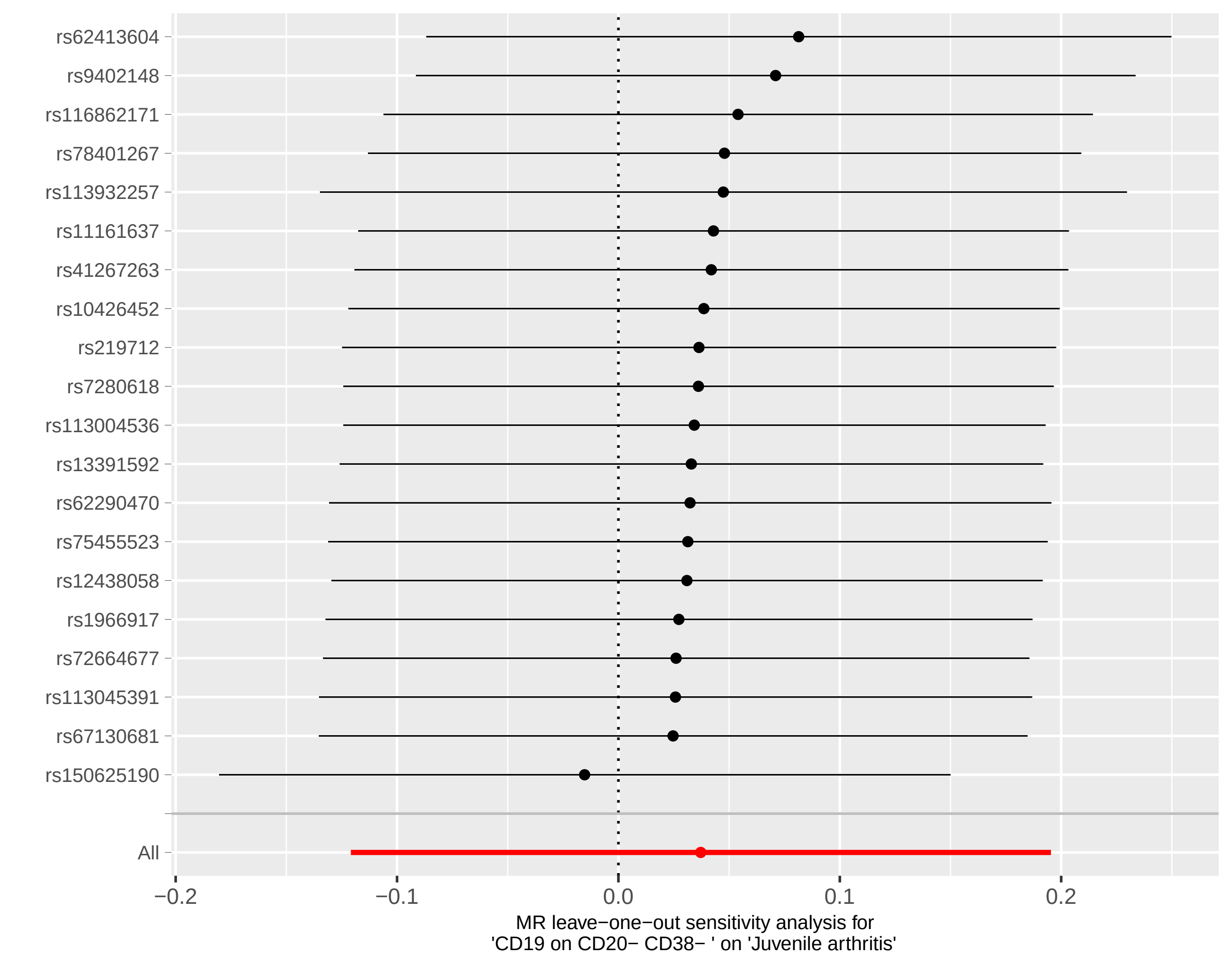
rs62413604
rs9402148
rs116862171
rs78401267
rs113932257
rs11161637
rs41267263
rs10426452
rs219712
rs7280618
rs113004536
rs13391592
rs62290470
rs75455523
rs12438058
rs1966917
rs72664677
rs113045391
rs67130681
rs150625190
All
−0.2
−0.1
0.0
0.1
0.2
MR leave−one−out sensitivity analysis for
'CD19 on CD20− CD38− ' on 'Juvenile arthritis'

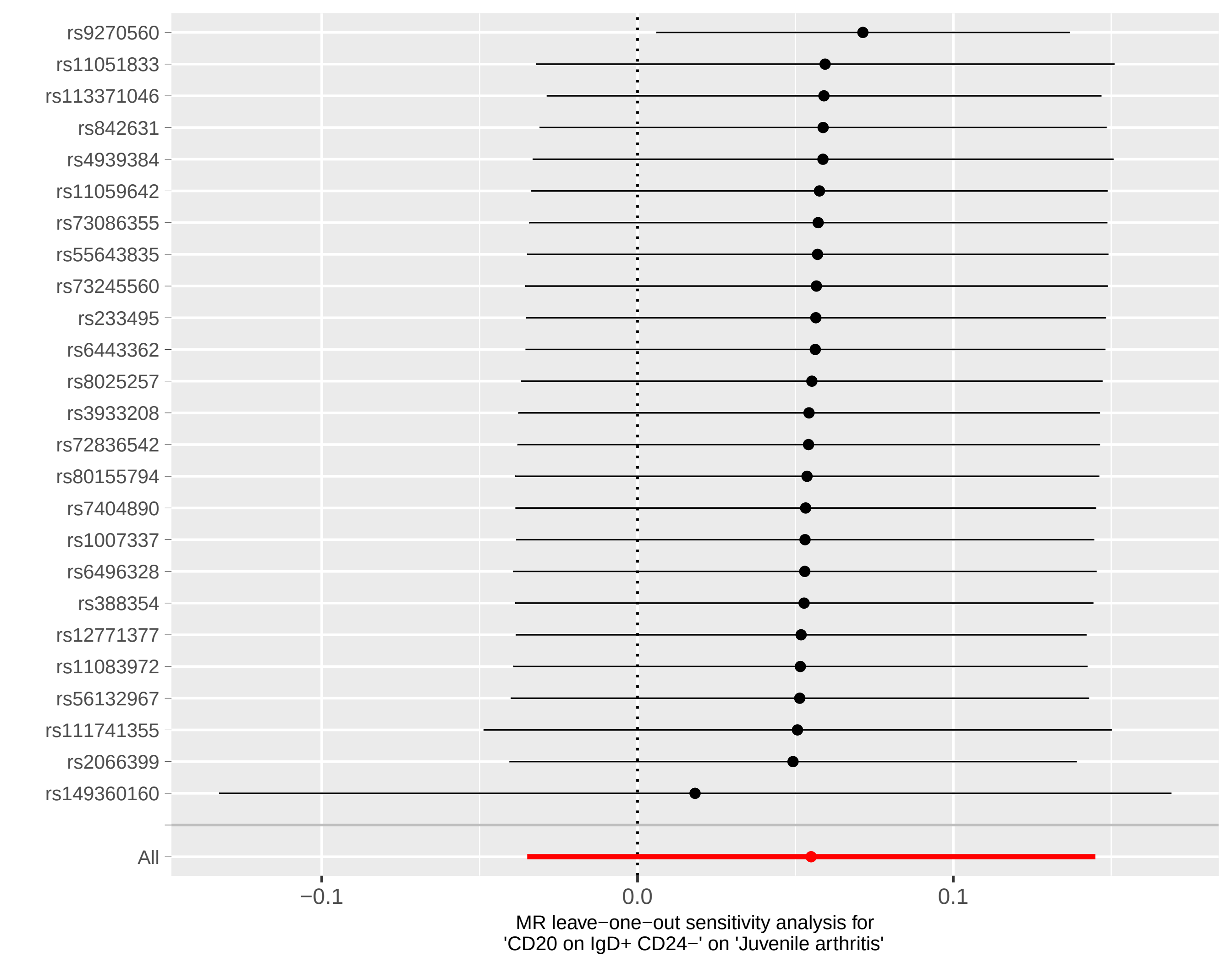
rs9270560
rs11051833
rs113371046
rs842631
rs4939384
rs11059642
rs73086355
rs55643835
rs73245560
rs233495
rs6443362
rs8025257
rs3933208
rs72836542
rs80155794
rs7404890
rs1007337
rs6496328
rs388354
rs12771377
rs11083972
rs56132967
rs111741355
rs2066399
rs149360160
All
−0.1
0.0
0.1
MR leave−one−out sensitivity analysis for
'CD20 on IgD+ CD24−' on 'Juvenile arthritis'

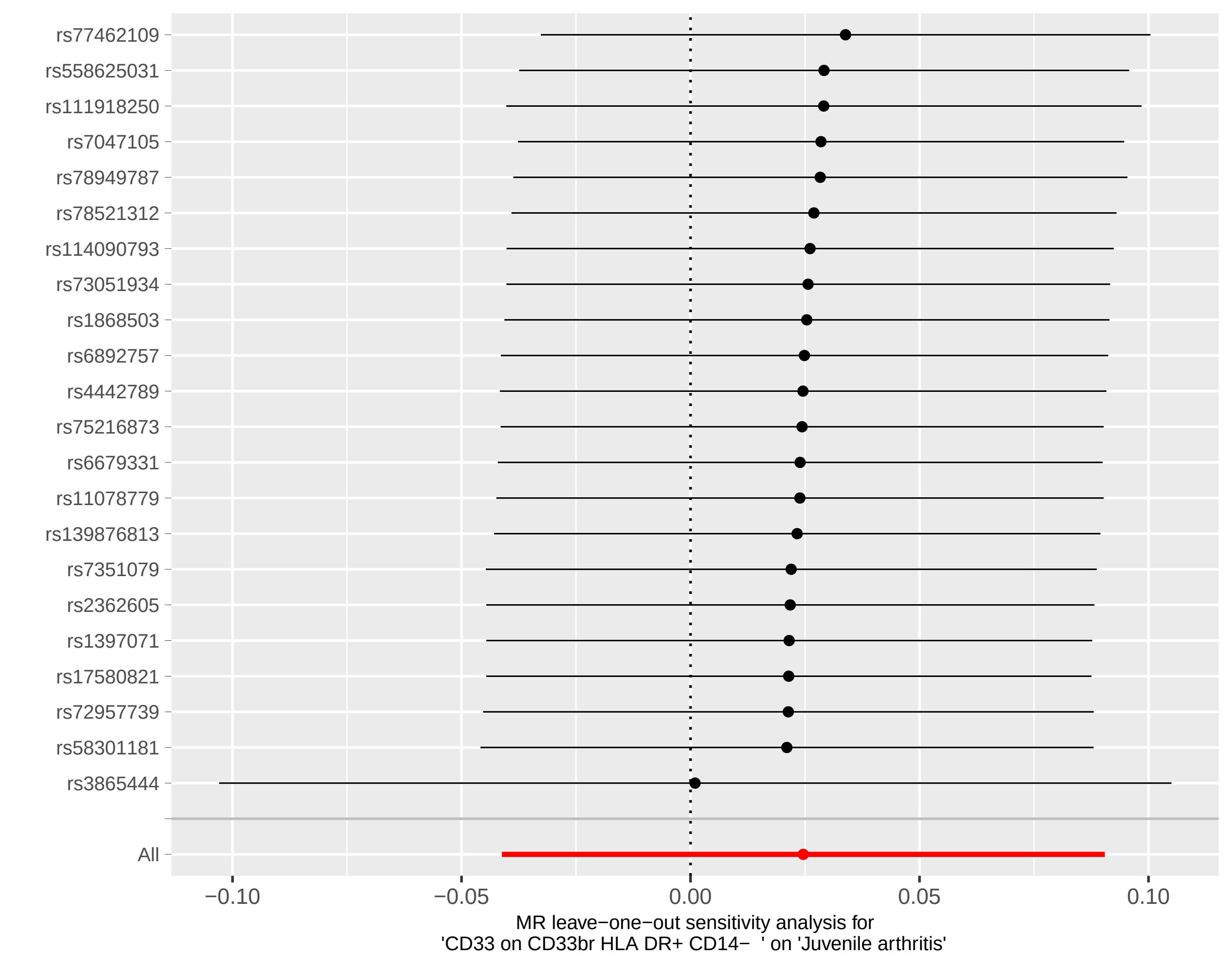

rs77462109
rs558625031
rs111918250
rs7047105
rs78949787
rs78521312
rs114090793
rs73051934
rs1868503
rs6892757
rs4442789
rs75216873
rs6679331
rs11078779
rs139876813
rs7351079
rs2362605
rs1397071
rs17580821
rs72957739
rs58301181
rs3865444
All
−0.10
−0.05
0.00
0.05
0.10
MR leave−one−out sensitivity analysis for
'CD33 on CD33br HLA DR+ CD14− ' on 'Juvenile arthritis'

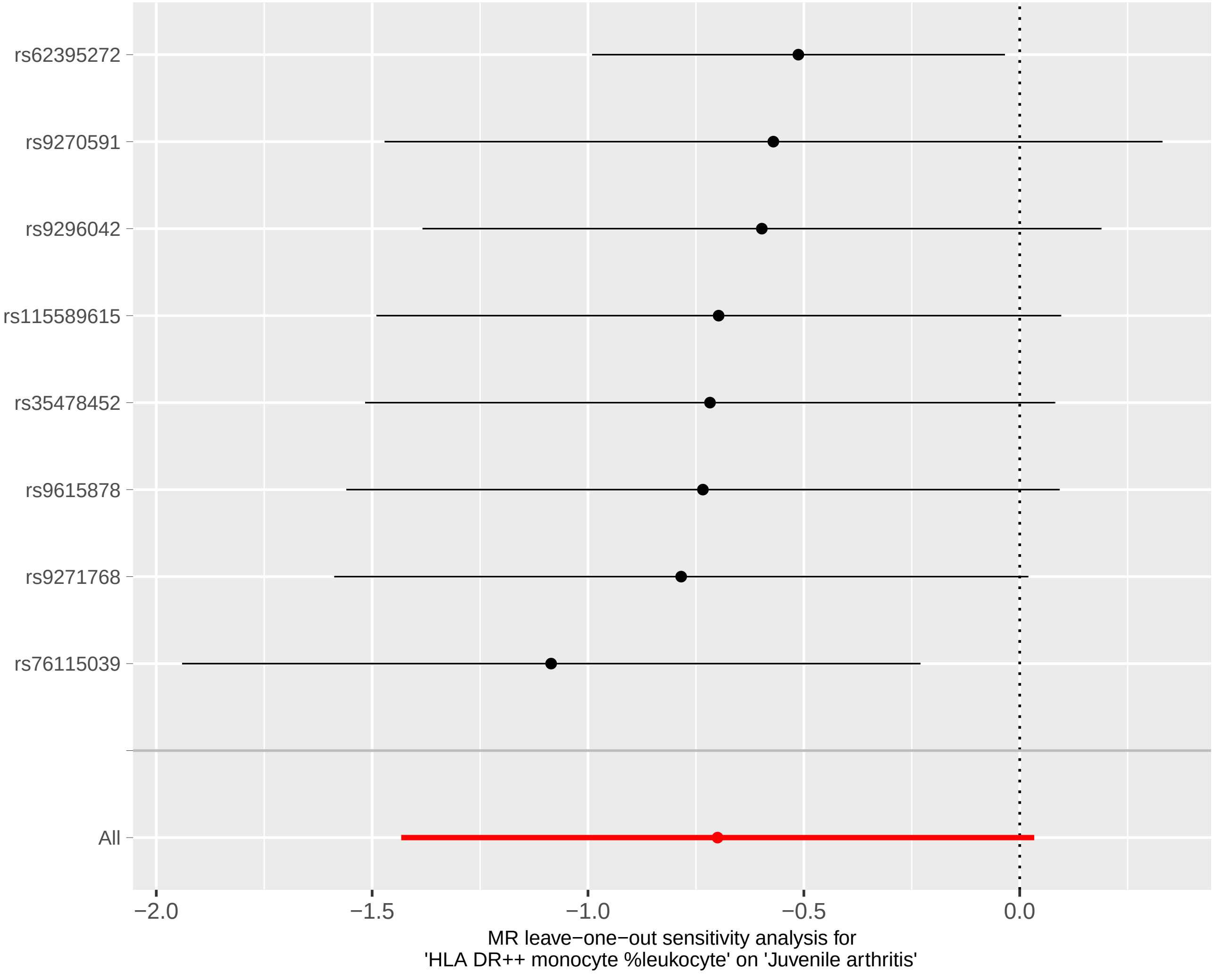
rs62395272
rs9270591
rs9296042
rs115589615
rs35478452
rs9615878
rs9271768
rs76115039
All
−2.0
−1.5
−1.0
−0.5
0.0
MR leave−one−out sensitivity analysis for
'HLA DR++ monocyte %leukocyte' on 'Juvenile arthritis'

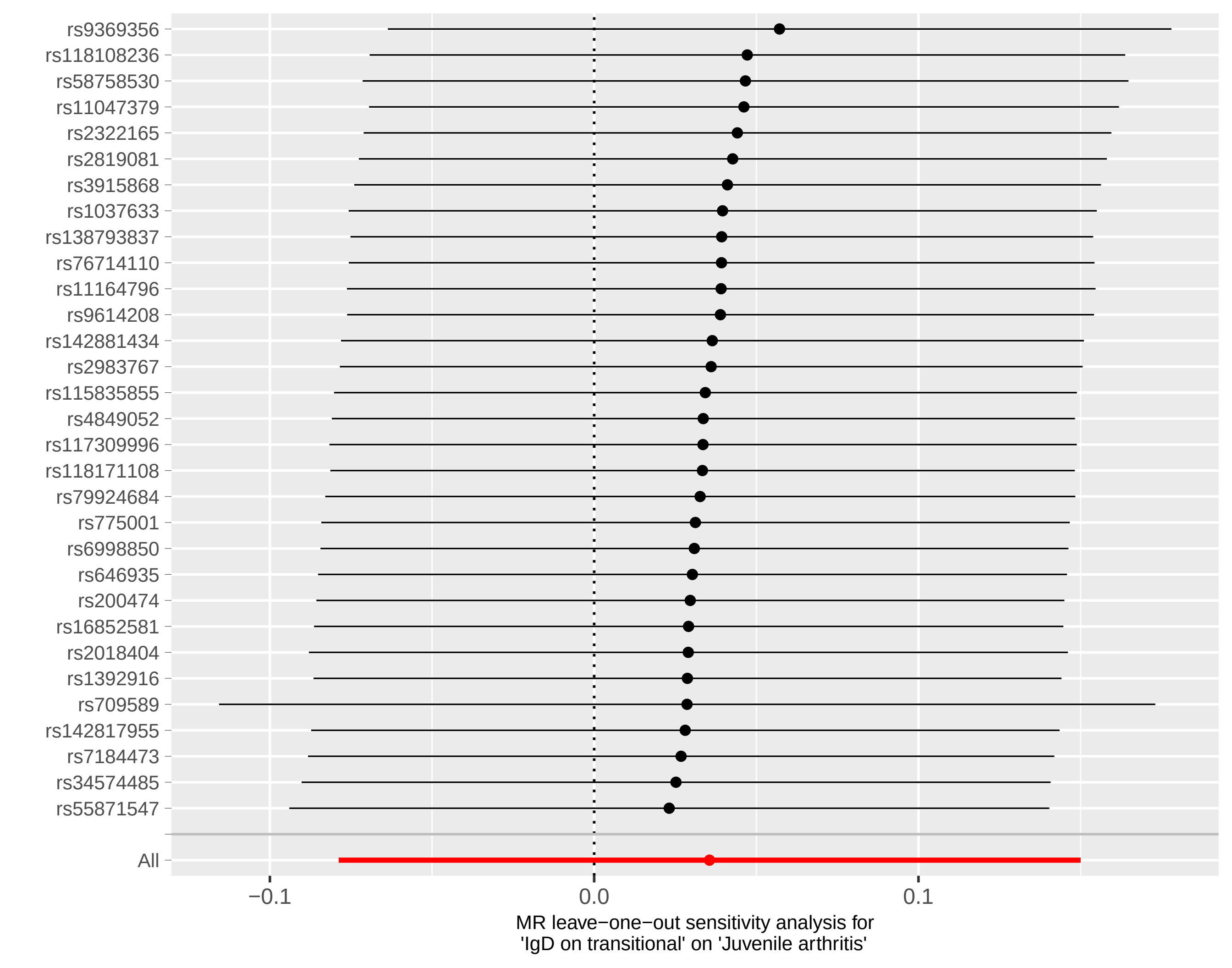
rs9369356
rs118108236
rs58758530
rs11047379
rs2322165
rs2819081
rs3915868
rs1037633
rs138793837
rs76714110
rs11164796
rs9614208
rs142881434
rs2983767
rs115835855
rs4849052
rs117309996
rs118171108
rs79924684
rs775001
rs6998850
rs646935
rs200474
rs16852581
rs2018404
rs1392916
rs709589
rs142817955
rs7184473
rs34574485
rs55871547
All
−0.1
0.0
0.1
MR leave−one−out sensitivity analysis for
'IgD on transitional' on 'Juvenile arthritis'

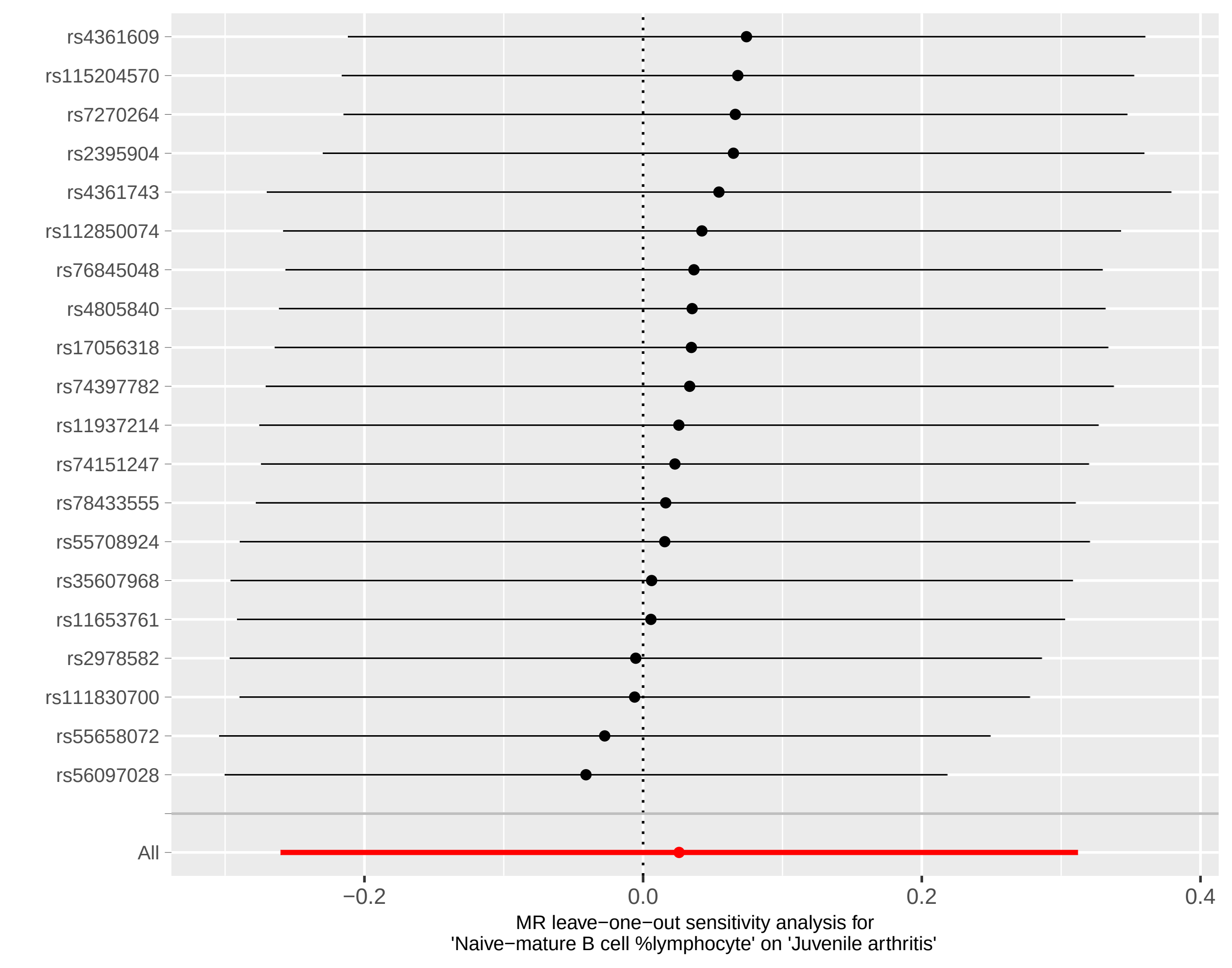
rs4361609
rs115204570
rs7270264
rs2395904
rs4361743
rs112850074
rs76845048
rs4805840
rs17056318
rs74397782
rs11937214
rs74151247
rs78433555
rs55708924
rs35607968
rs11653761
rs2978582
rs111830700
rs55658072
rs56097028
All
−0.2
0.0
0.2
0.4
MR leave−one−out sensitivity analysis for
'Naive−mature B cell %lymphocyte' on 'Juvenile arthritis'

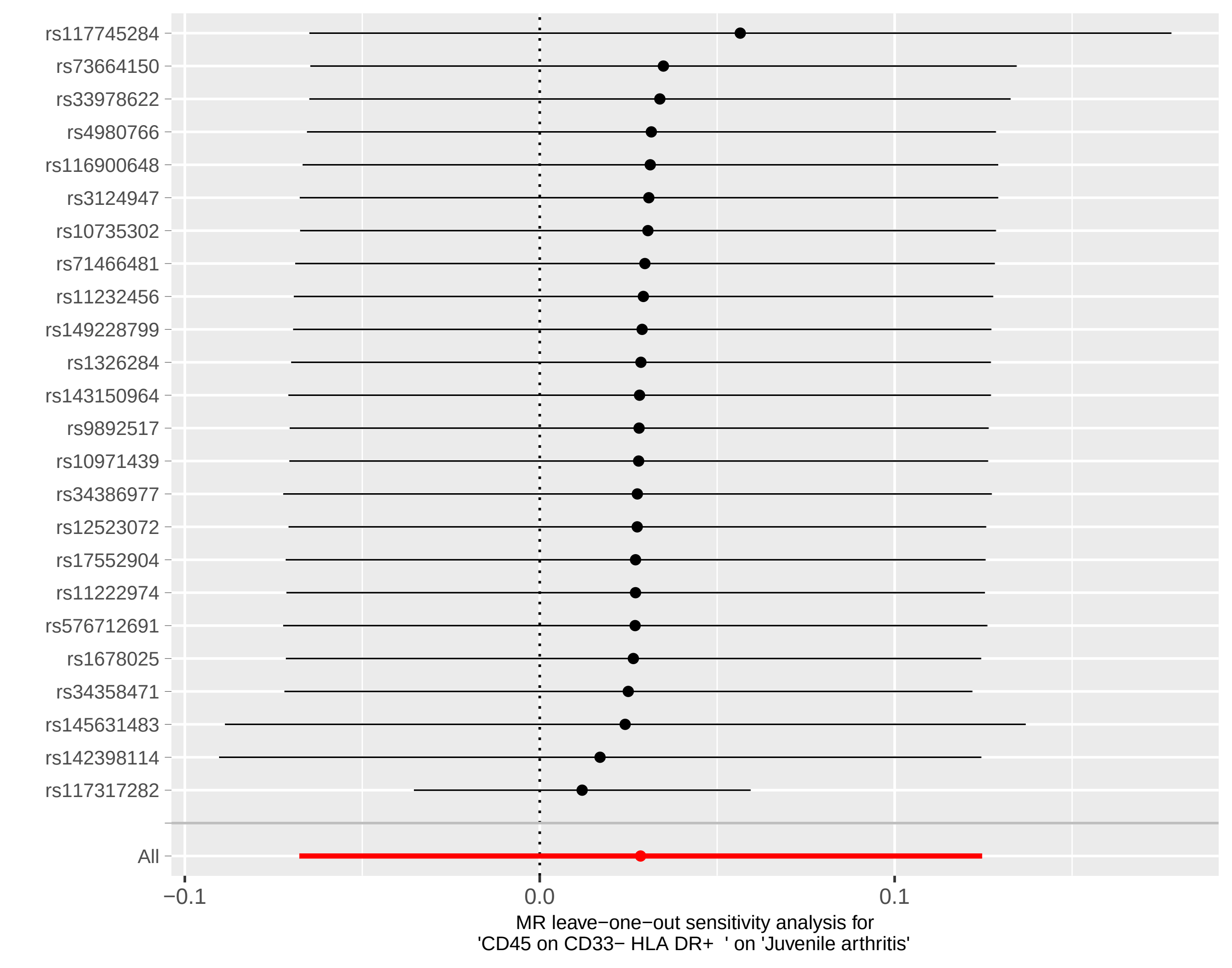
rs117745284
rs73664150
rs33978622
rs4980766
rs116900648
rs3124947
rs10735302
rs71466481
rs11232456
rs149228799
rs1326284
rs143150964
rs9892517
rs10971439
rs34386977
rs12523072
rs17552904
rs11222974
rs576712691
rs1678025
rs34358471
rs145631483
rs142398114
rs117317282
All
−0.1
0.0
0.1
MR leave−one−out sensitivity analysis for
'CD45 on CD33− HLA DR+ ' on 'Juvenile arthritis'

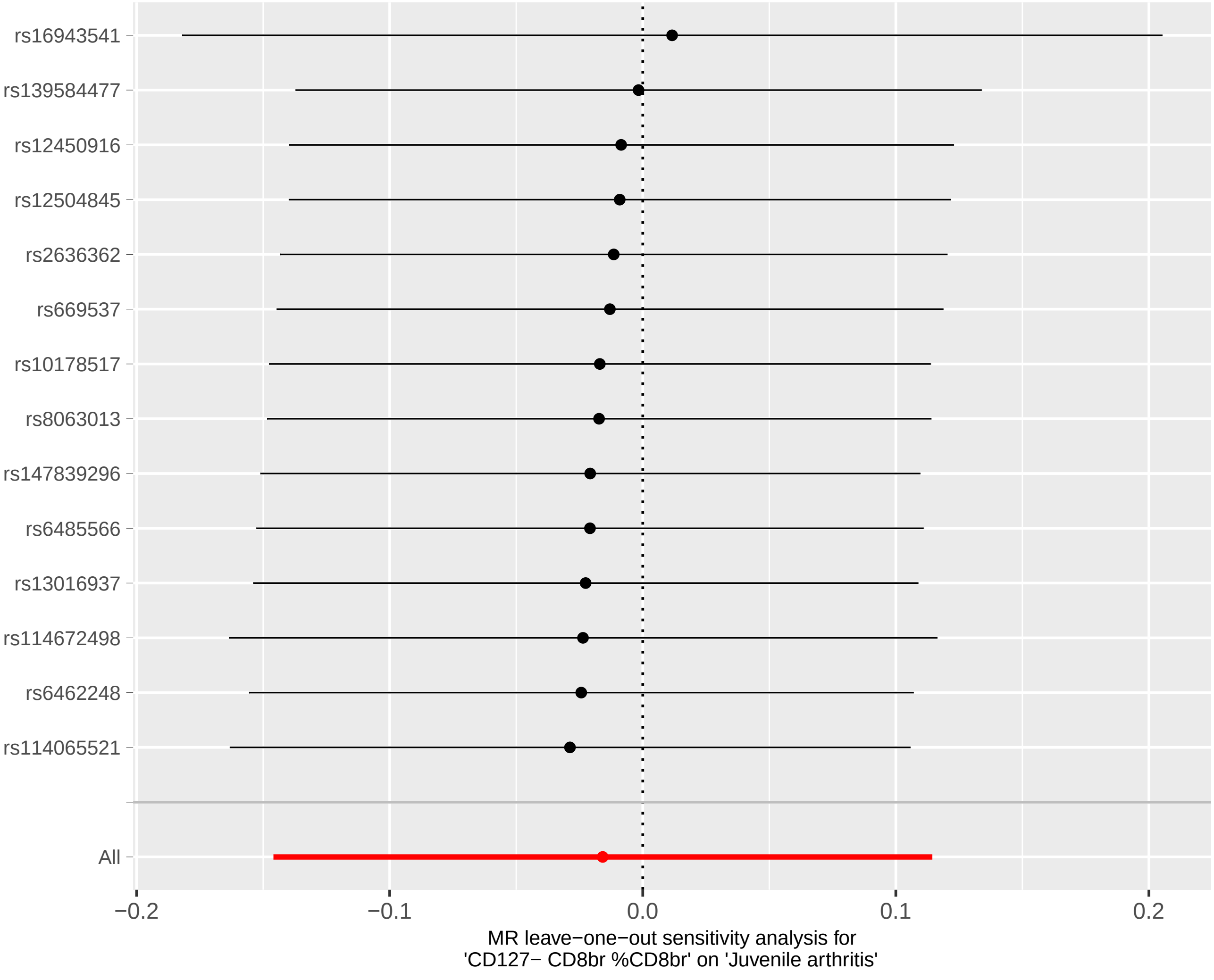
rs16943541
rs139584477
rs12450916
rs12504845
rs2636362
rs669537
rs10178517
rs8063013
rs147839296
rs6485566
rs13016937
rs114672498
rs6462248
rs114065521
All
−0.2
−0.1
0.0
0.1
0.2
MR leave−one−out sensitivity analysis for
'CD127− CD8br %CD8br' on 'Juvenile arthritis'

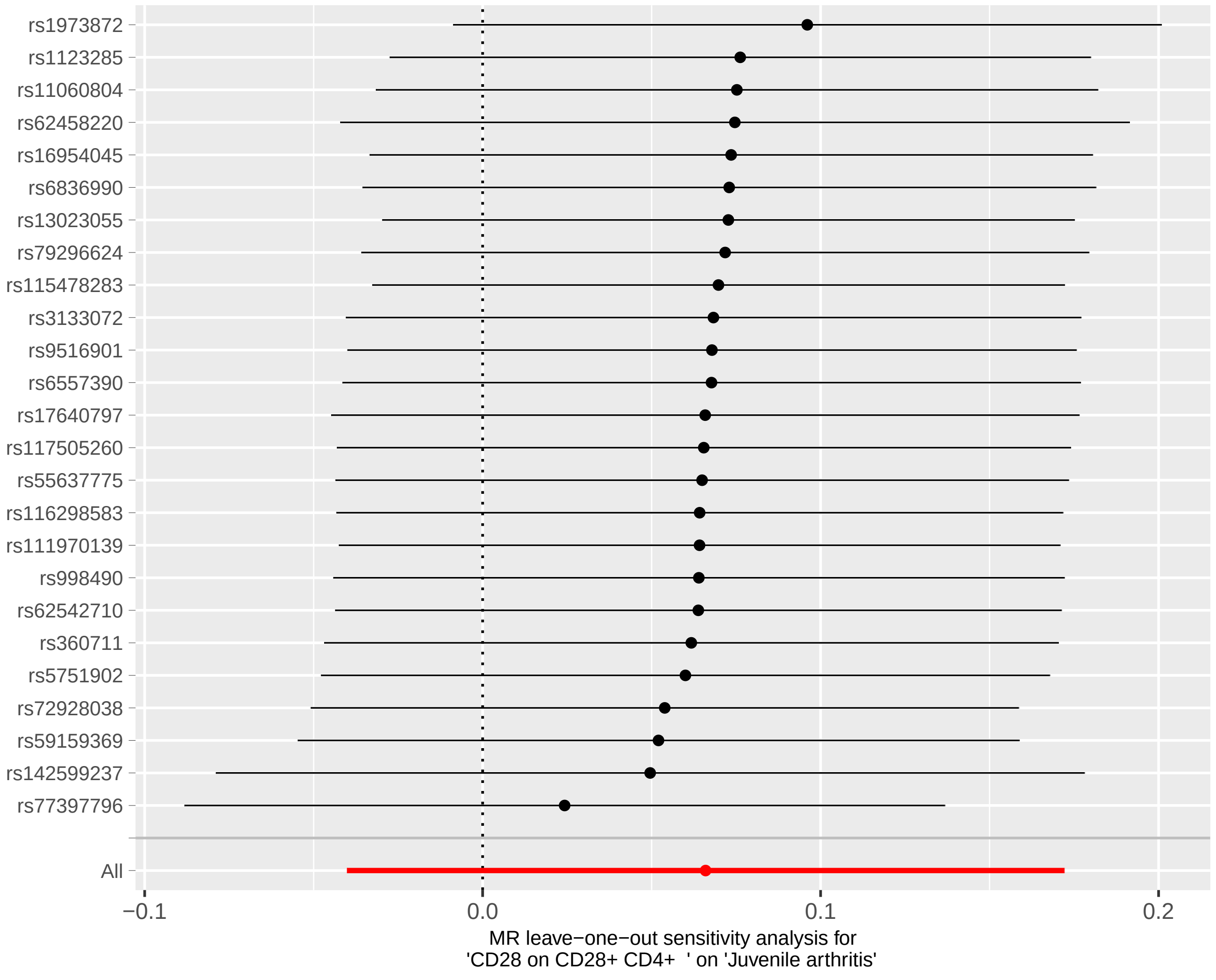
rs1973872
rs1123285
rs11060804
rs62458220
rs16954045
rs6836990
rs13023055
rs79296624
rs115478283
rs3133072
rs9516901
rs6557390
rs17640797
rs117505260
rs55637775
rs116298583
rs111970139
rs998490
rs62542710
rs360711
rs5751902
rs72928038
rs59159369
rs142599237
rs77397796
All
−0.1
0.0
0.1
0.2
MR leave−one−out sensitivity analysis for
'CD28 on CD28+ CD4+ ' on 'Juvenile arthritis'

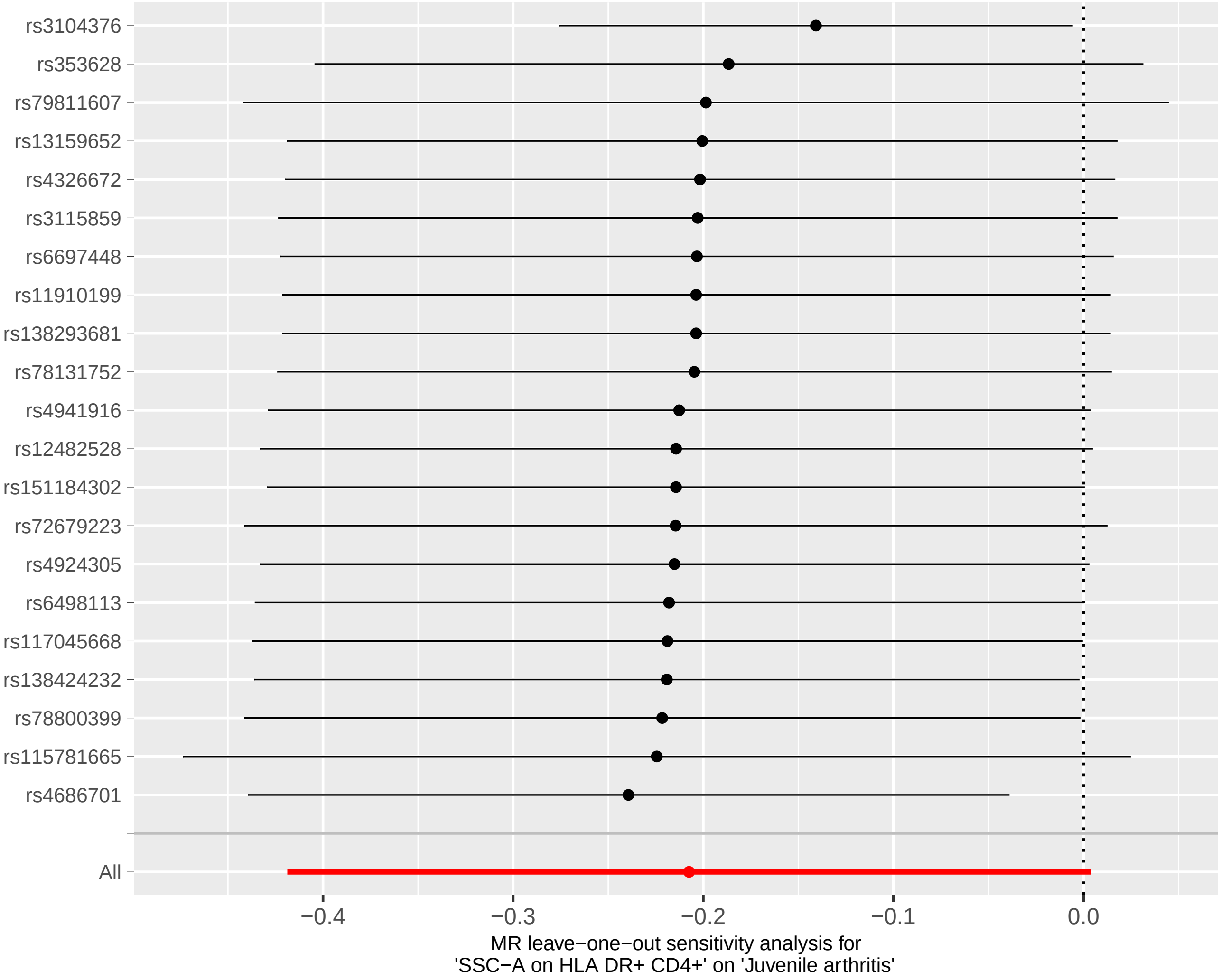
rs3104376
rs353628
rs79811607
rs13159652
rs4326672
rs3115859
rs6697448
rs11910199
rs138293681
rs78131752
rs4941916
rs12482528
rs151184302
rs72679223
rs4924305
rs6498113
rs117045668
rs138424232
rs78800399
rs115781665
rs4686701
All
−0.4
−0.3
−0.2
−0.1
0.0
MR leave−one−out sensitivity analysis for
'SSC−A on HLA DR+ CD4+' on 'Juvenile arthritis'

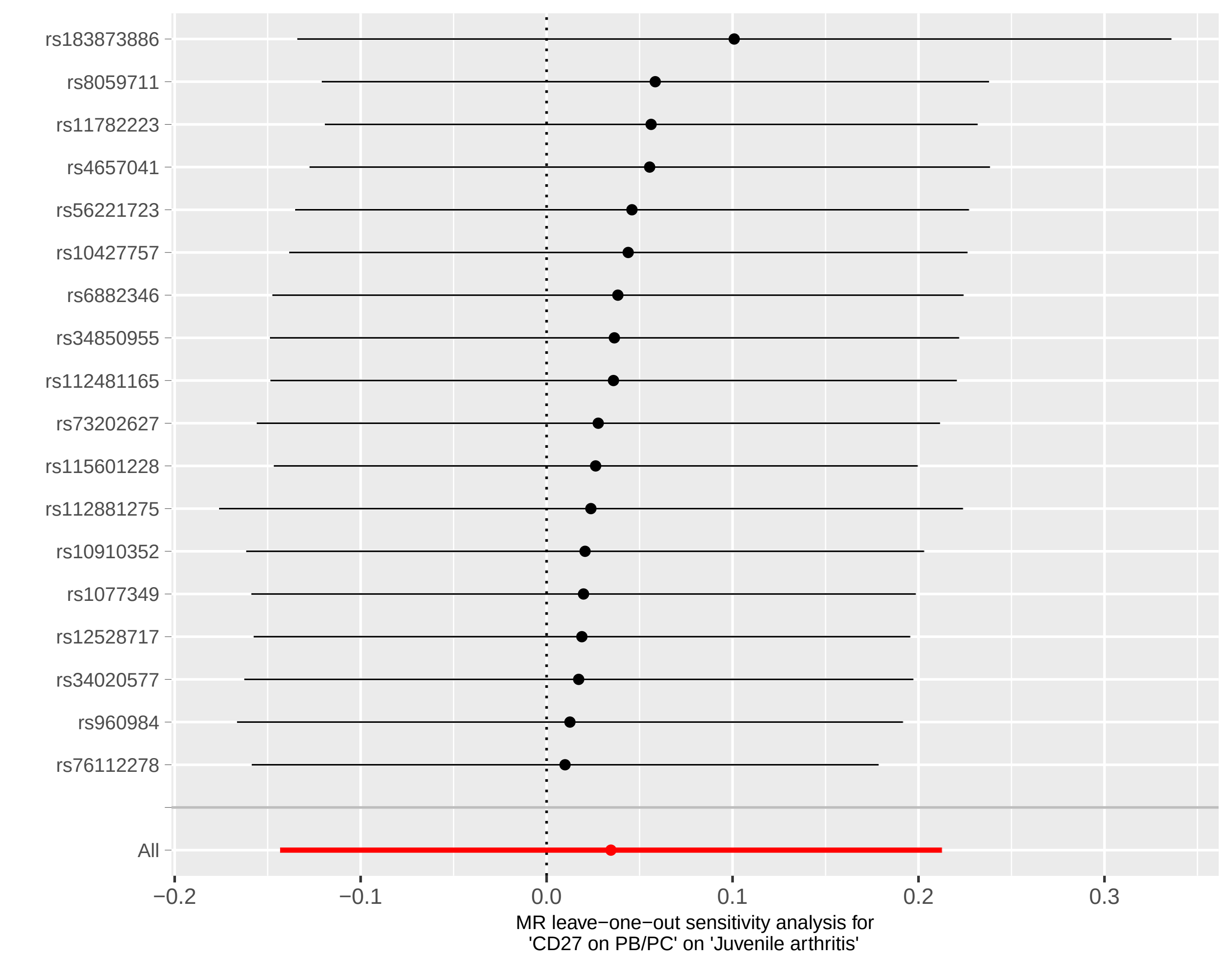

rs183873886
rs8059711
rs11782223
rs4657041
rs56221723
rs10427757
rs6882346
rs34850955
rs112481165
rs73202627
rs115601228
rs112881275
rs10910352
rs1077349
rs12528717
rs34020577
rs960984
rs76112278
All
−0.2
−0.1
0.0
0.1
0.2
0.3
MR leave−one−out sensitivity analysis for
'CD27 on PB/PC' on 'Juvenile arthritis'

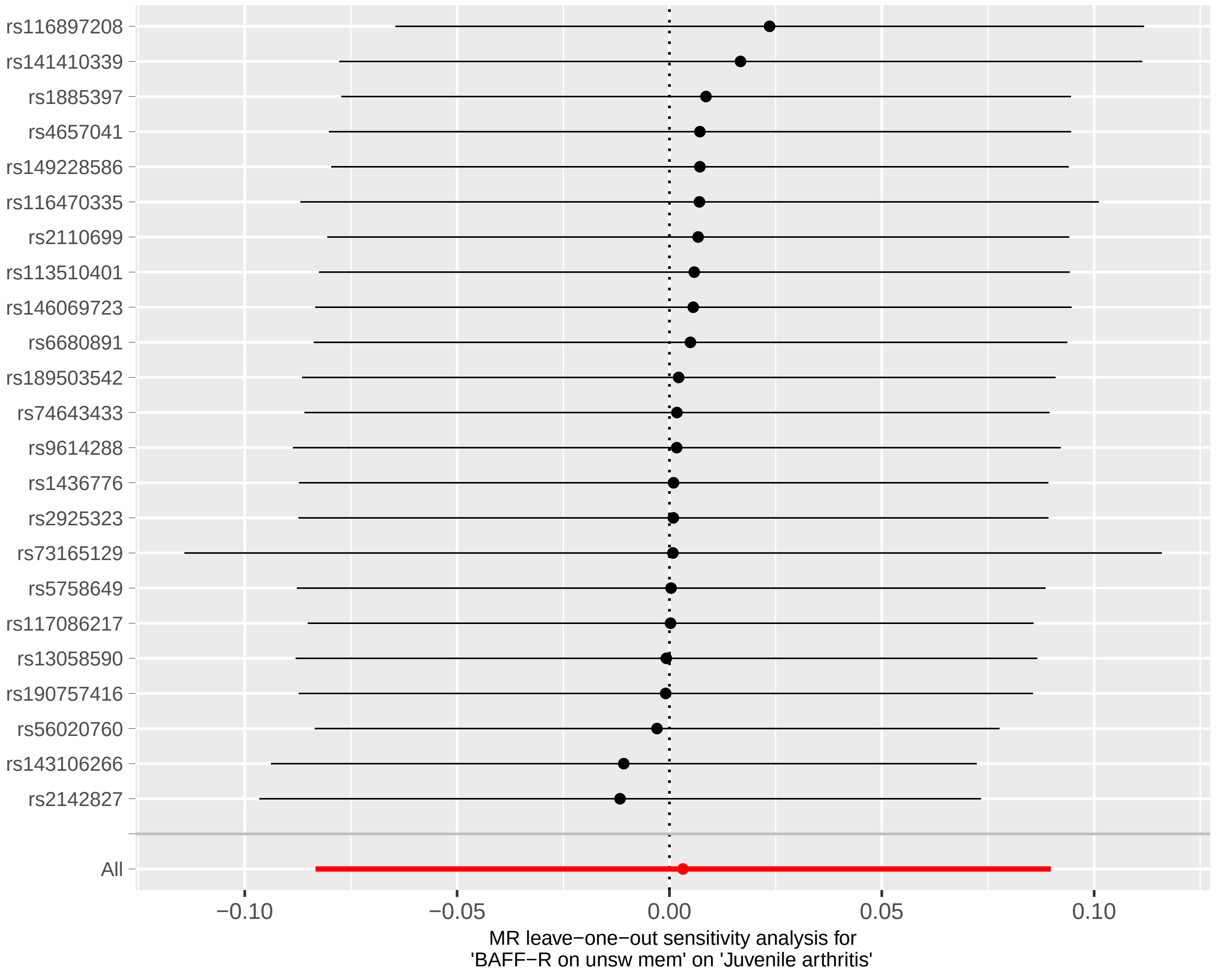
rs116897208
rs141410339
rs1885397
rs4657041
rs149228586
rs116470335
rs2110699
rs113510401
rs146069723
rs6680891
rs189503542
rs74643433
rs9614288
rs1436776
rs2925323
rs73165129
rs5758649
rs117086217
rs13058590
rs190757416
rs56020760
rs143106266
rs2142827
All
−0.10
−0.05
0.00
0.05
0.10
MR leave−one−out sensitivity analysis for
'BAFF−R on unsw mem' on 'Juvenile arthritis'

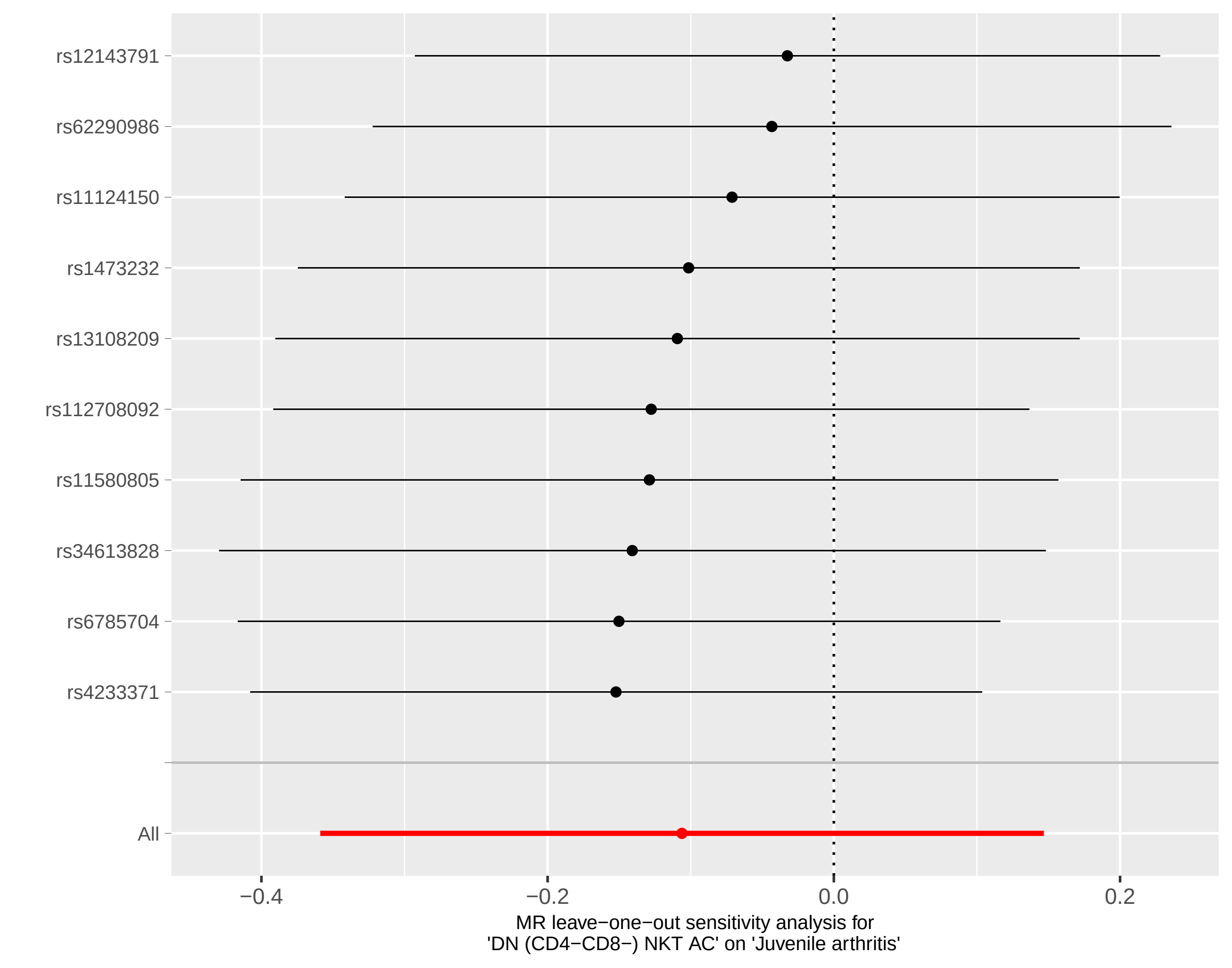
rs12143791
rs62290986
rs11124150
rs1473232
rs13108209
rs112708092
rs11580805
rs34613828
rs6785704
rs4233371
All
−0.4
−0.2
0.0
0.2
MR leave−one−out sensitivity analysis for
'DN (CD4−CD8−) NKT AC' on 'Juvenile arthritis'

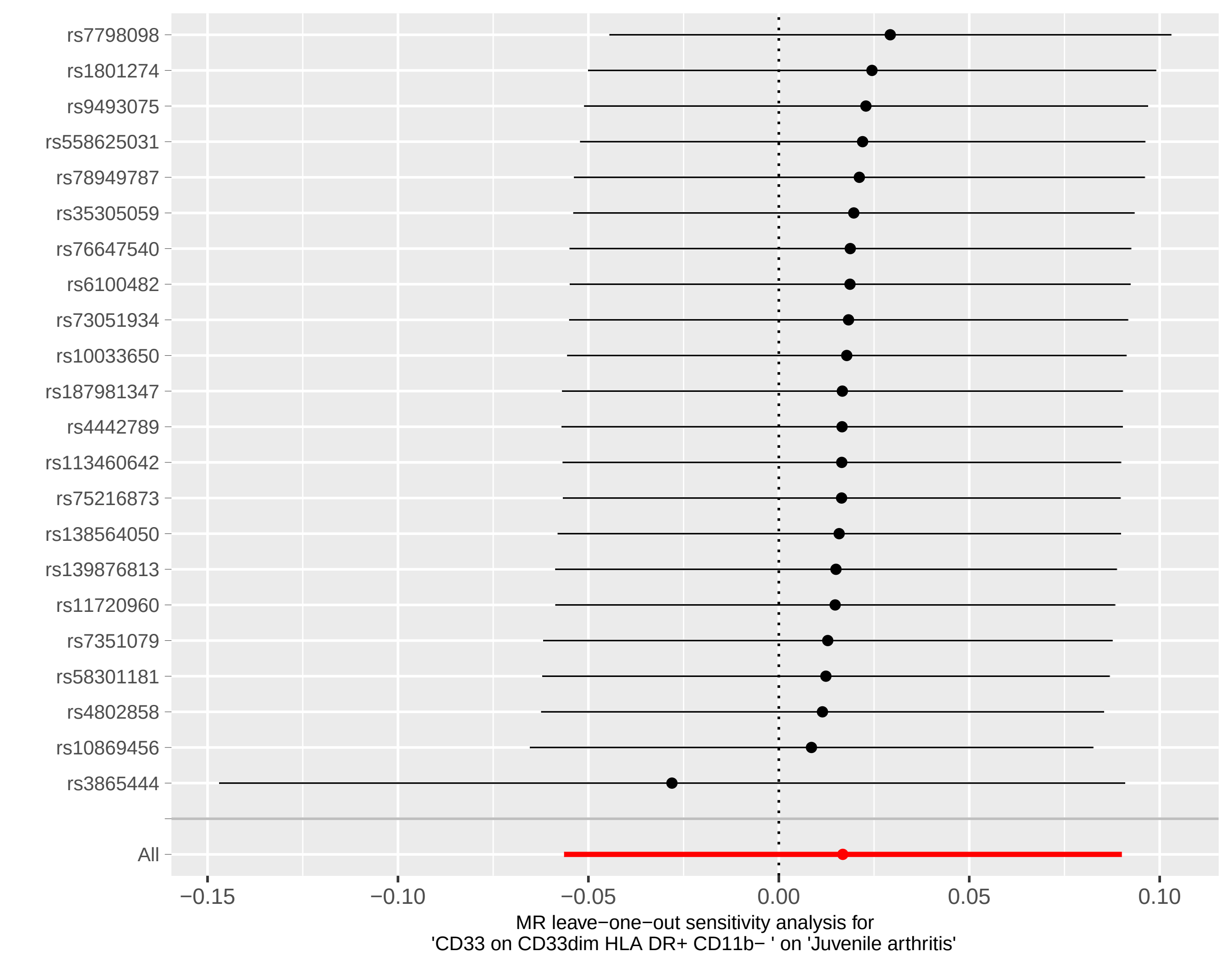
rs7798098
rs1801274
rs9493075
rs558625031
rs78949787
rs35305059
rs76647540
rs6100482
rs73051934
rs10033650
rs187981347
rs4442789
rs113460642
rs75216873
rs138564050
rs139876813
rs11720960
rs7351079
rs58301181
rs4802858
rs10869456
rs3865444
All
−0.15
−0.10
−0.05
0.00
0.05
0.10
MR leave−one−out sensitivity analysis for
'CD33 on CD33dim HLA DR+ CD11b− ' on 'Juvenile arthritis'

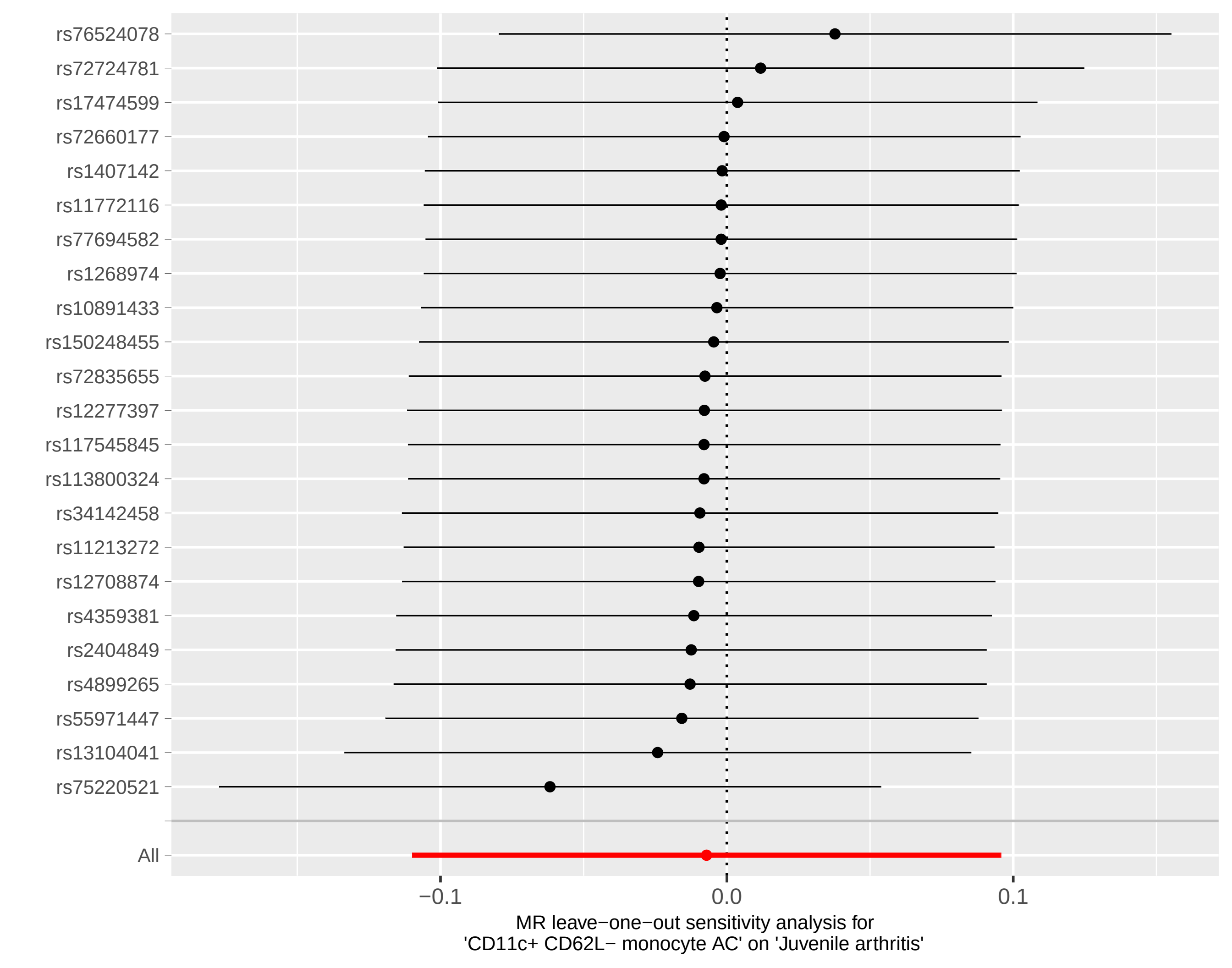
rs76524078
rs72724781
rs17474599
rs72660177
rs1407142
rs11772116
rs77694582
rs1268974
rs10891433
rs150248455
rs72835655
rs12277397
rs117545845
rs113800324
rs34142458
rs11213272
rs12708874
rs4359381
rs2404849
rs4899265
rs55971447
rs13104041
rs75220521
All
−0.1
0.0
0.1
MR leave−one−out sensitivity analysis for
'CD11c+ CD62L− monocyte AC' on 'Juvenile arthritis'

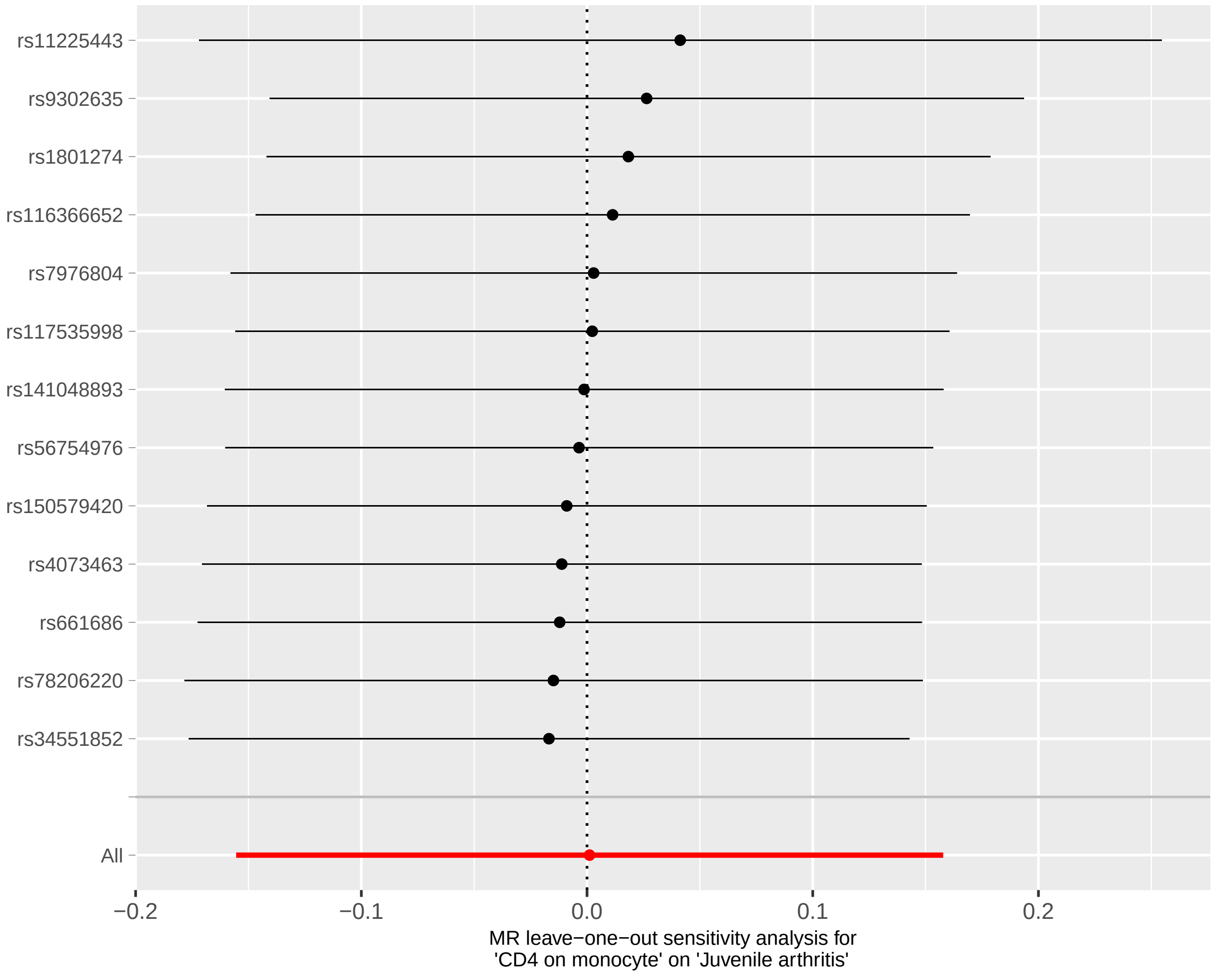

rs11225443
rs9302635
rs1801274
rs116366652
rs7976804
rs117535998
rs141048893
rs56754976
rs150579420
rs4073463
rs661686
rs78206220
rs34551852
All
−0.2
−0.1
0.0
0.1
0.2
MR leave−one−out sensitivity analysis for
'CD4 on monocyte' on 'Juvenile arthritis'

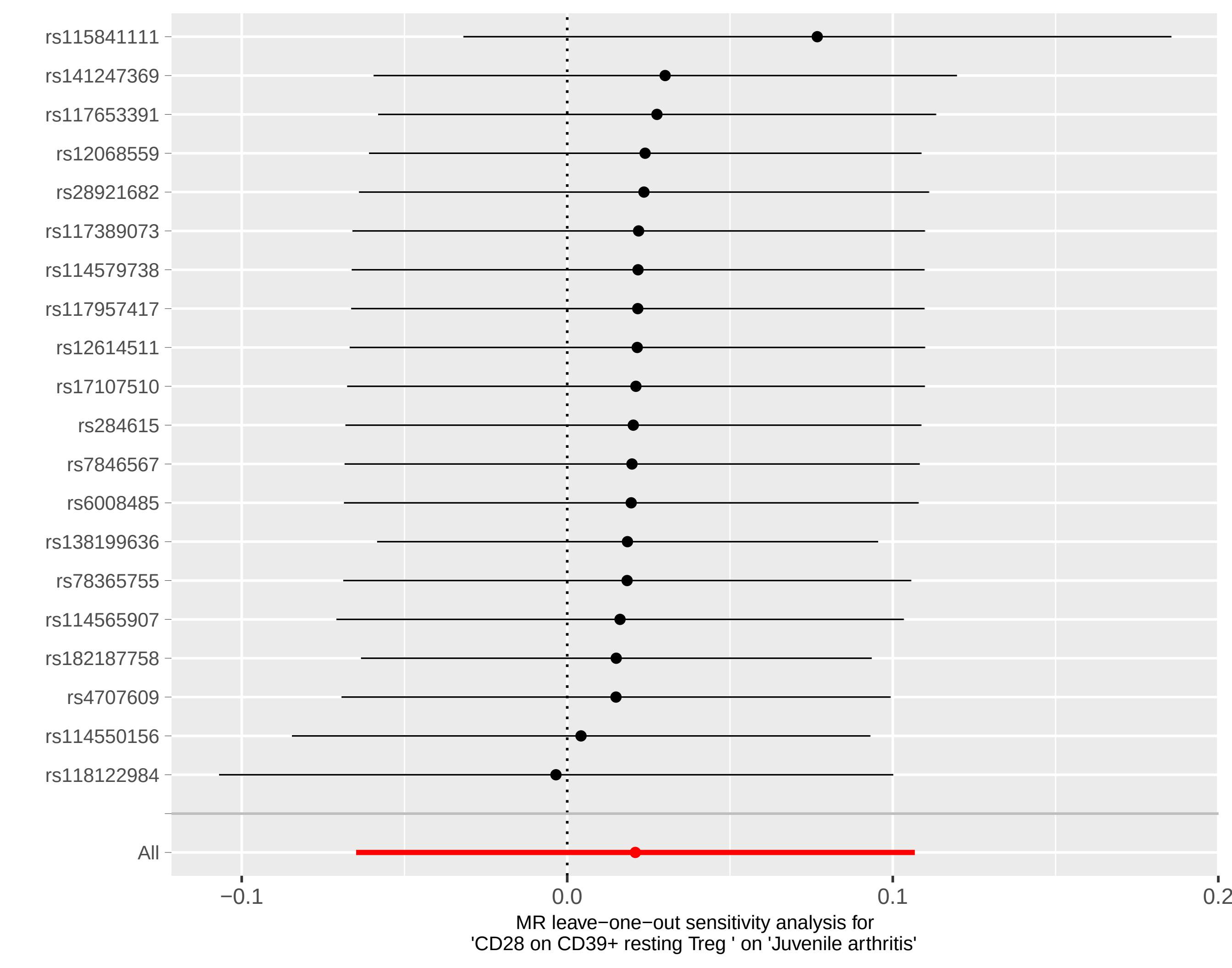
rs115841111
rs141247369
rs117653391
rs12068559
rs28921682
rs117389073
rs114579738
rs117957417
rs12614511
rs17107510
rs284615
rs7846567
rs6008485
rs138199636
rs78365755
rs114565907
rs182187758
rs4707609
rs114550156
rs118122984
All
−0.1
0.0
0.1
0.2
MR leave−one−out sensitivity analysis for
'CD28 on CD39+ resting Treg ' on 'Juvenile arthritis'

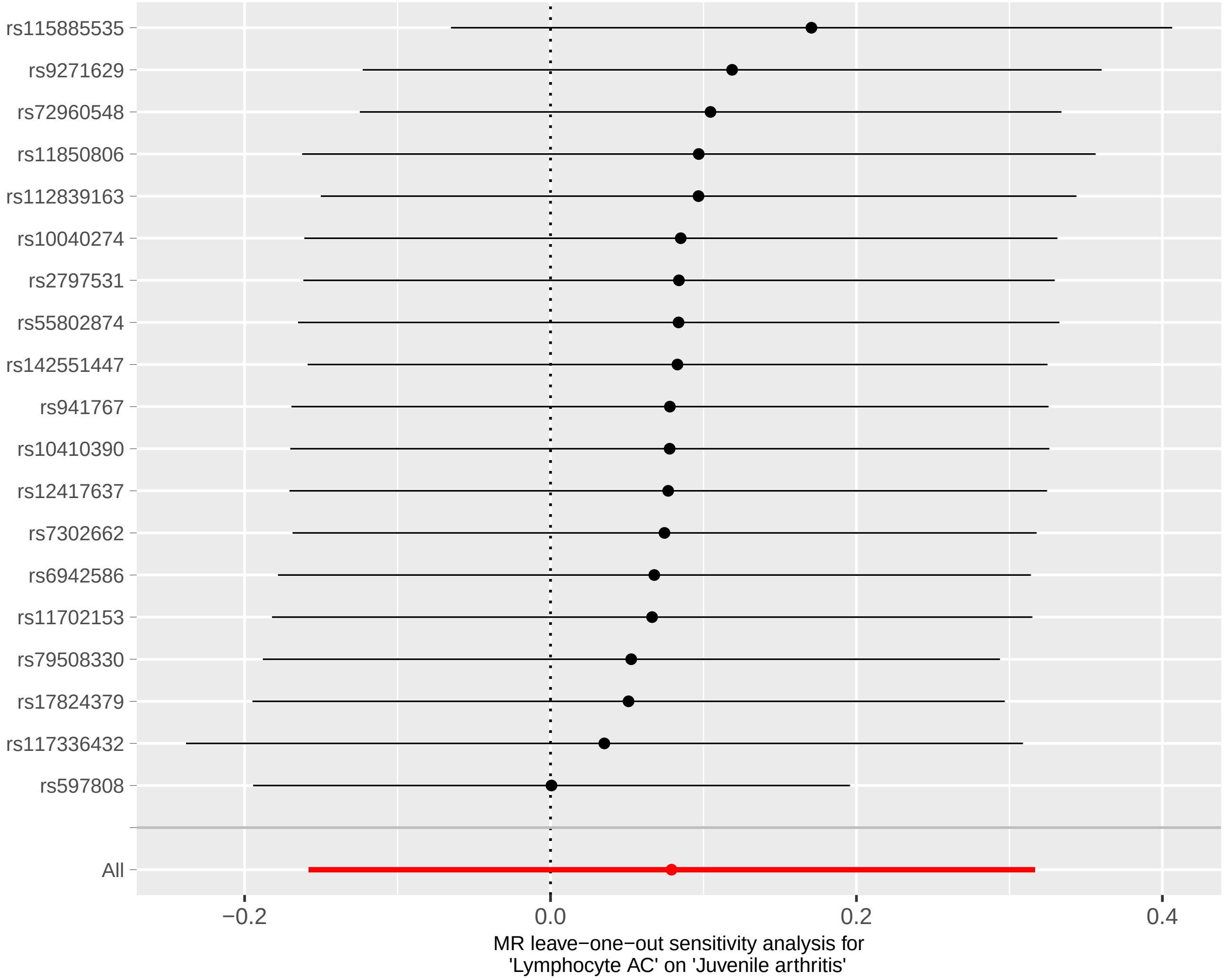

rs115885535
rs9271629
rs72960548
rs11850806
rs112839163
rs10040274
rs2797531
rs55802874
rs142551447
rs941767
rs10410390
rs12417637
rs7302662
rs6942586
rs11702153
rs79508330
rs17824379
rs117336432
rs597808
All
−0.2
0.0
0.2
0.4
MR leave−one−out sensitivity analysis for
'Lymphocyte AC' on 'Juvenile arthritis'

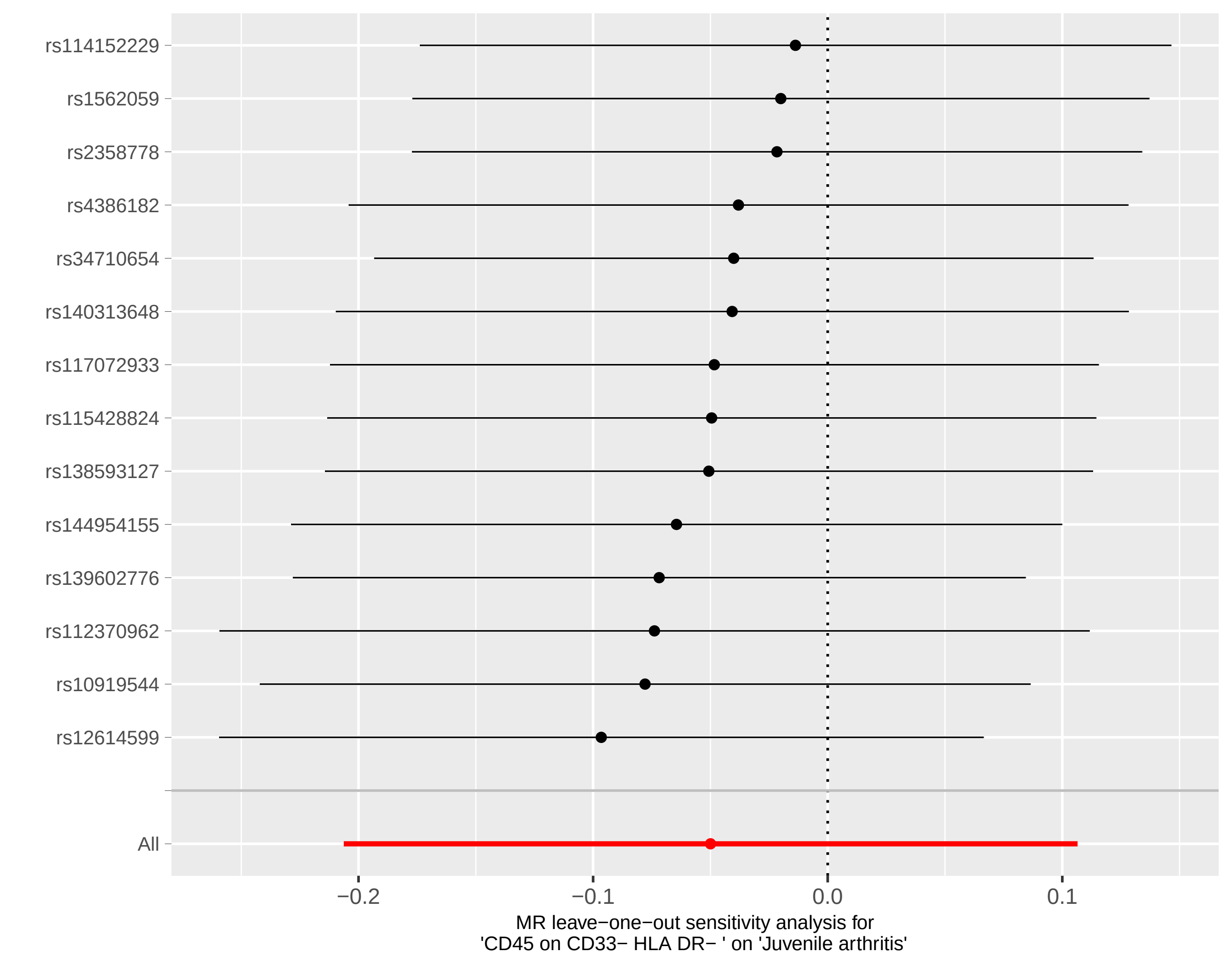
rs114152229
rs1562059
rs2358778
rs4386182
rs34710654
rs140313648
rs117072933
rs115428824
rs138593127
rs144954155
rs139602776
rs112370962
rs10919544
rs12614599
All
−0.2
−0.1
0.0
0.1
MR leave−one−out sensitivity analysis for
'CD45 on CD33− HLA DR− ' on 'Juvenile arthritis'

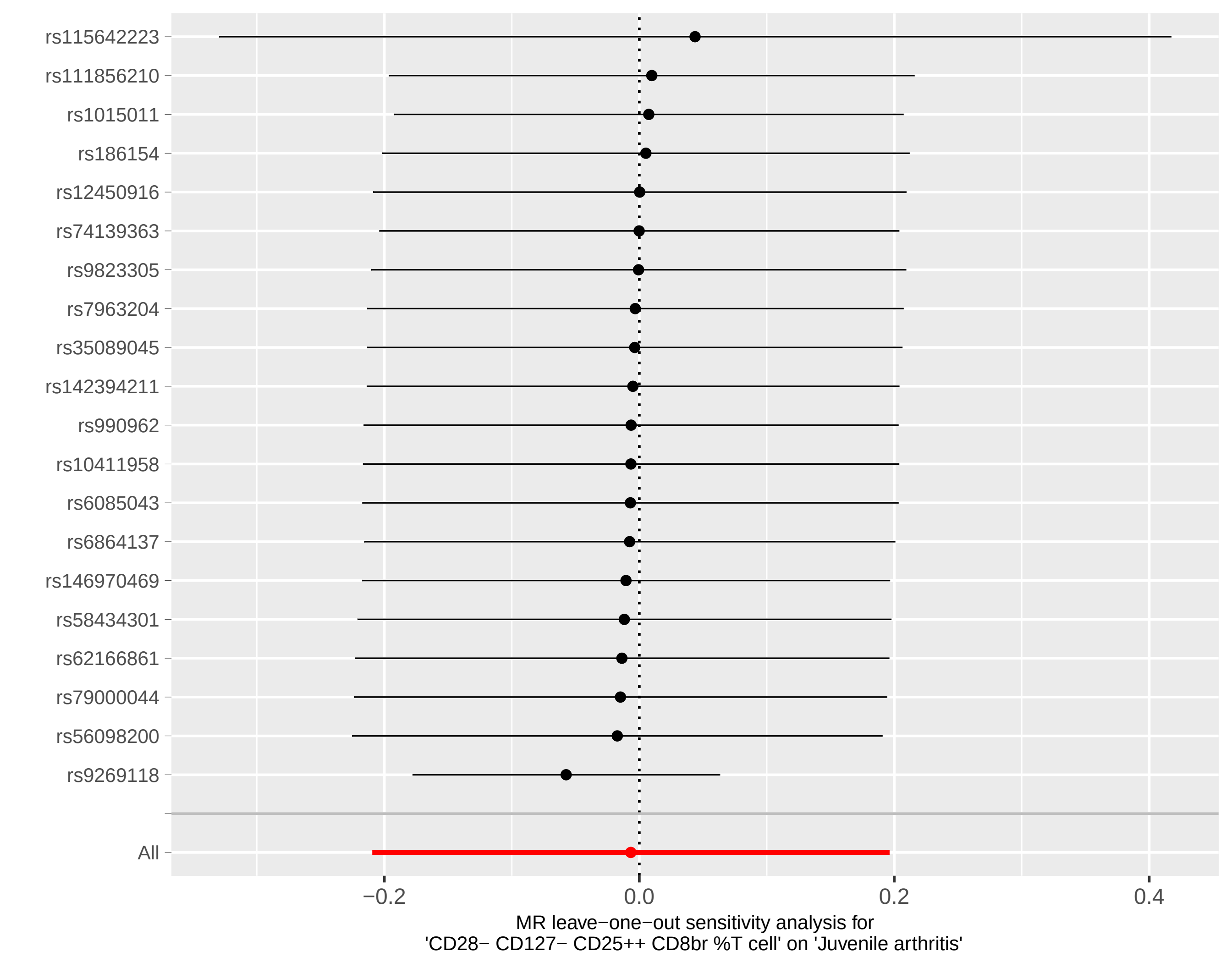

rs115642223
rs111856210
rs1015011
rs186154
rs12450916
rs74139363
rs9823305
rs7963204
rs35089045
rs142394211
rs990962
rs10411958
rs6085043
rs6864137
rs146970469
rs58434301
rs62166861
rs79000044
rs56098200
rs9269118
All
−0.2
0.0
0.2
0.4
MR leave−one−out sensitivity analysis for
'CD28− CD127− CD25++ CD8br %T cell' on 'Juvenile arthritis'

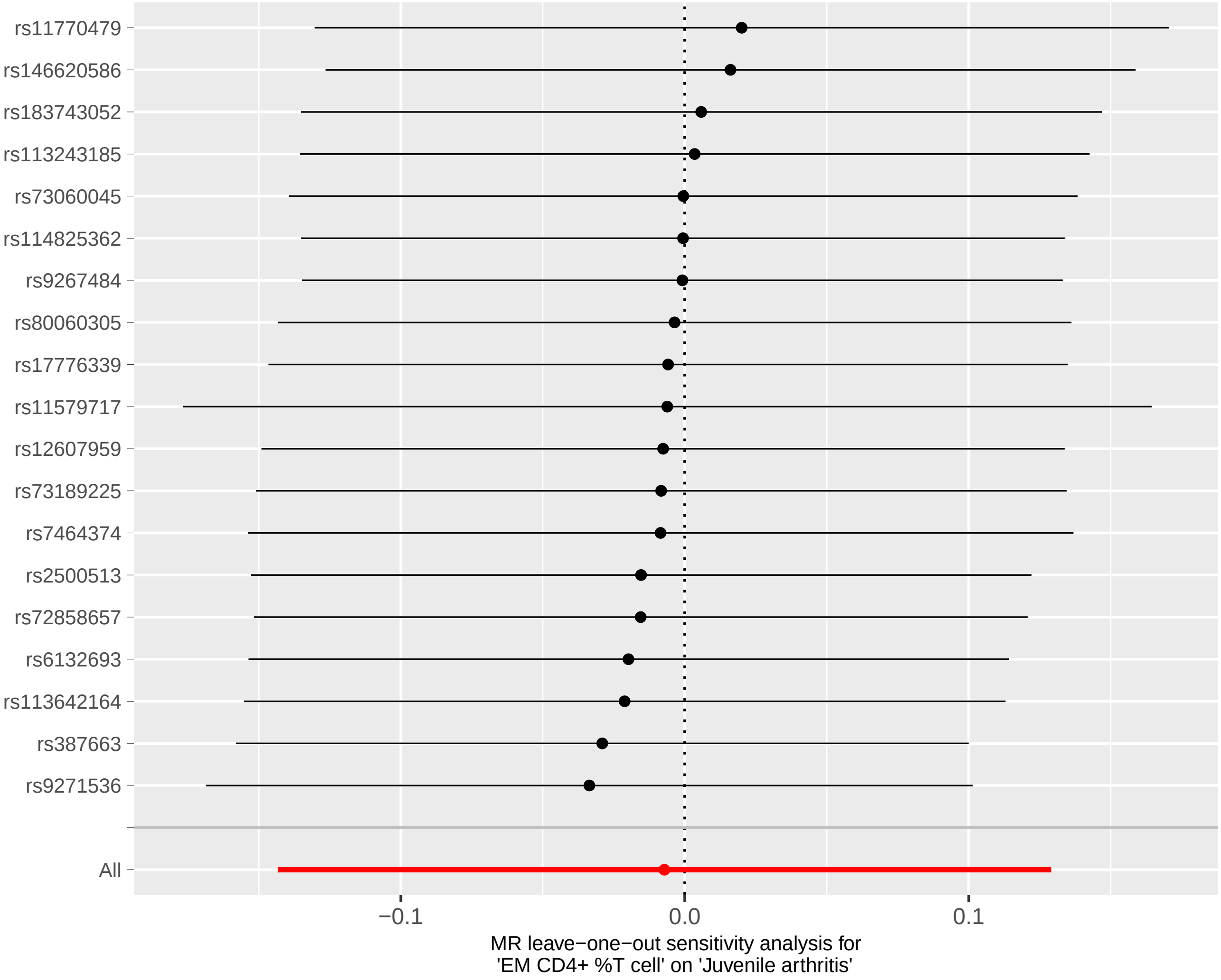
rs11770479
rs146620586
rs183743052
rs113243185
rs73060045
rs114825362
rs9267484
rs80060305
rs17776339
rs11579717
rs12607959
rs73189225
rs7464374
rs2500513
rs72858657
rs6132693
rs113642164
rs387663
rs9271536
All
−0.1
0.0
0.1
MR leave−one−out sensitivity analysis for
'EM CD4+ %T cell' on 'Juvenile arthritis'

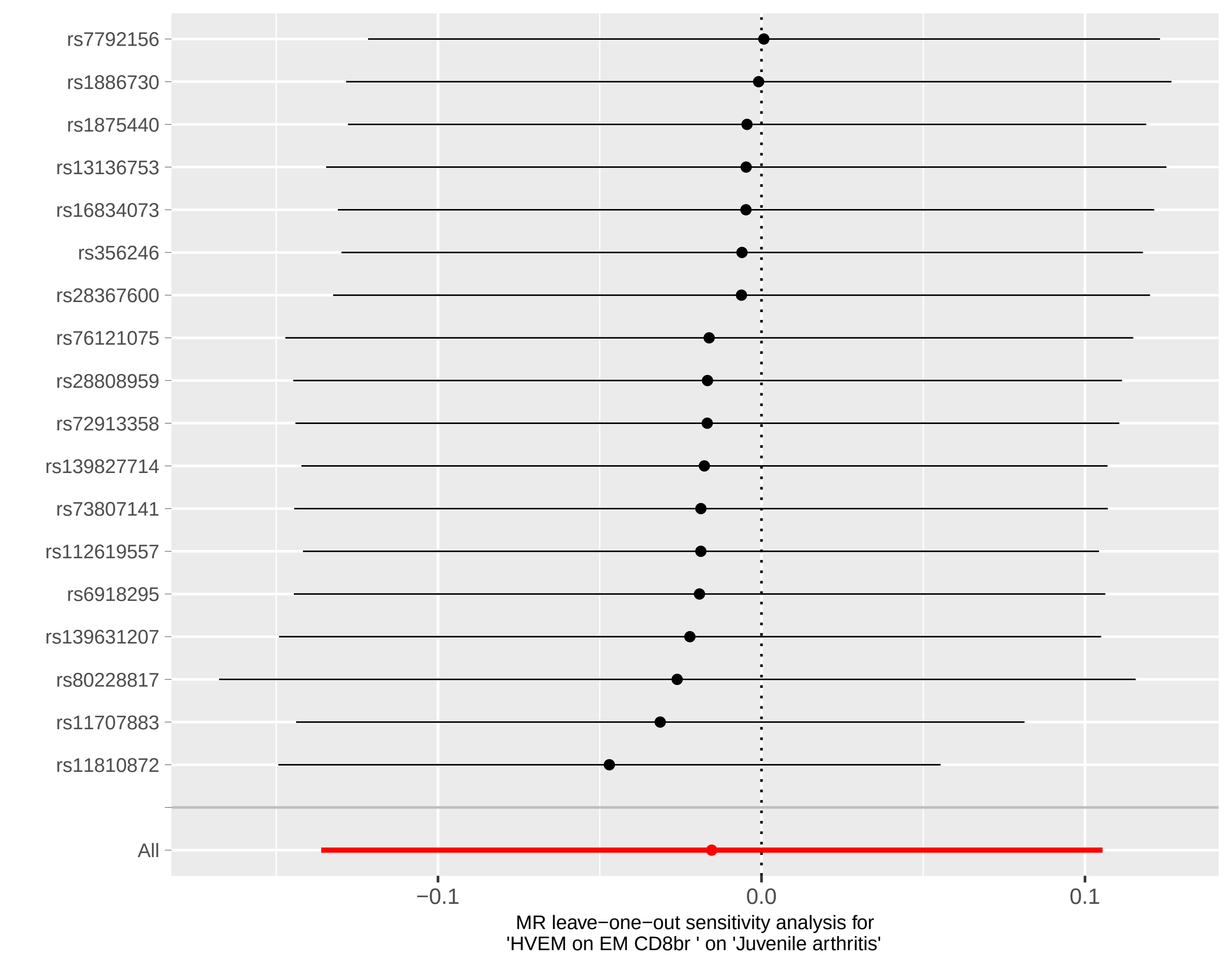

rs7792156
rs1886730
rs1875440
rs13136753
rs16834073
rs356246
rs28367600
rs76121075
rs28808959
rs72913358
rs139827714
rs73807141
rs112619557
rs6918295
rs139631207
rs80228817
rs11707883
rs11810872
All
−0.1
0.0
0.1
MR leave−one−out sensitivity analysis for
'HVEM on EM CD8br ' on 'Juvenile arthritis'

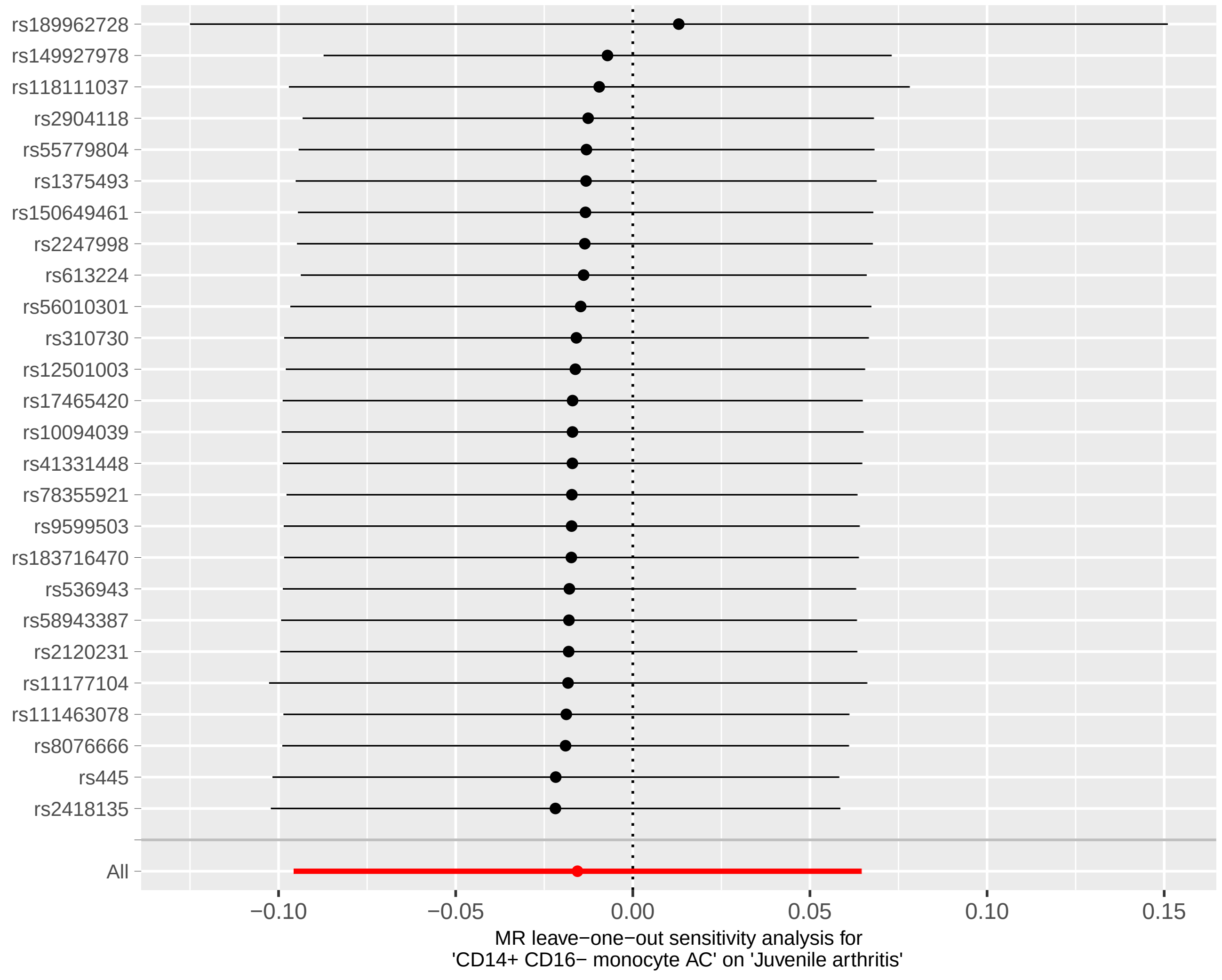

rs189962728
rs149927978
rs118111037
rs2904118
rs55779804
rs1375493
rs150649461
rs2247998
rs613224
rs56010301
rs310730
rs12501003
rs17465420
rs10094039
rs41331448
rs78355921
rs9599503
rs183716470
rs536943
rs58943387
rs2120231
rs11177104
rs111463078
rs8076666
rs445
rs2418135
All
−0.10
−0.05
0.00
0.05
0.10
0.15
MR leave−one−out sensitivity analysis for
'CD14+ CD16− monocyte AC' on 'Juvenile arthritis'

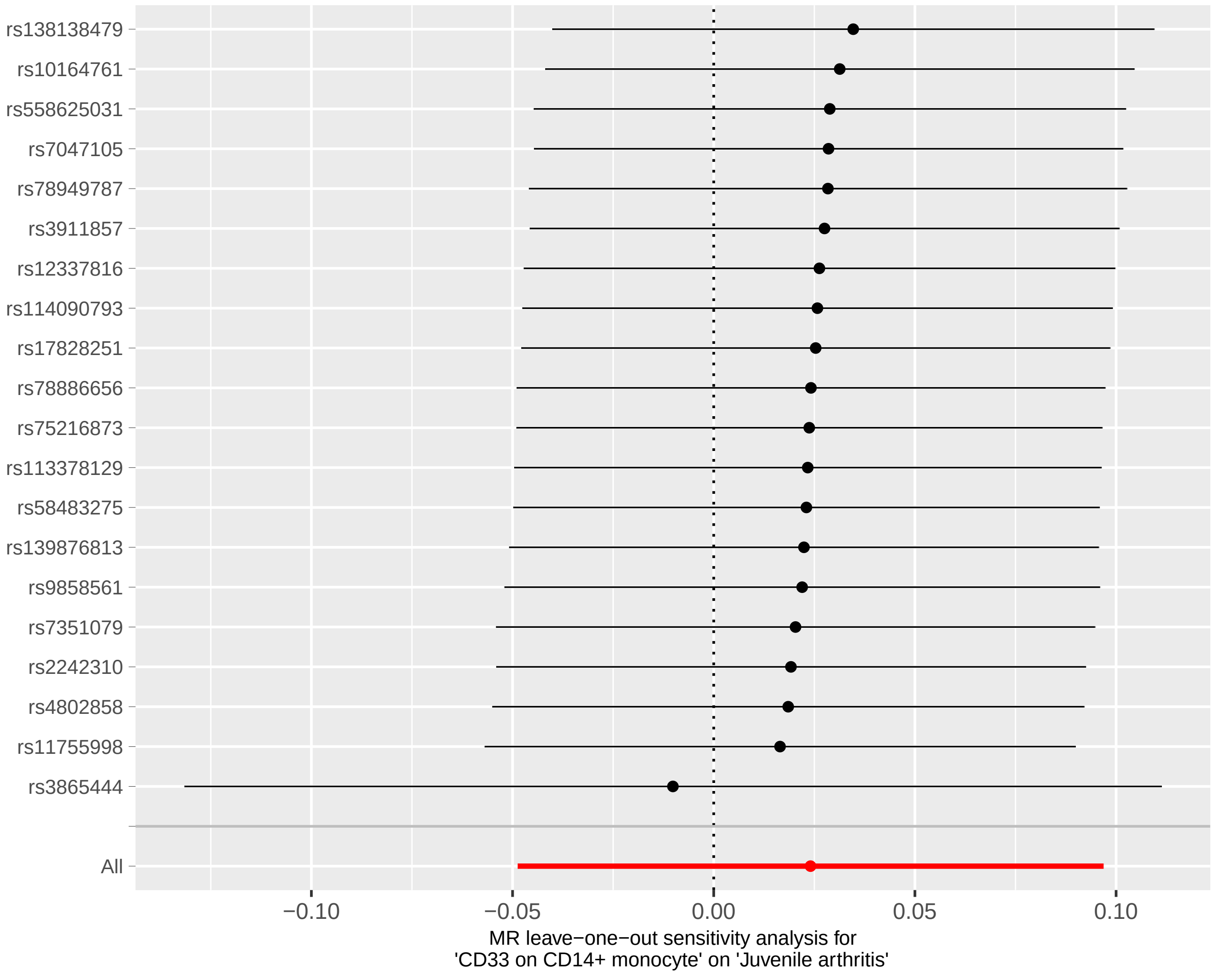

rs138138479
rs10164761
rs558625031
rs7047105
rs78949787
rs3911857
rs12337816
rs114090793
rs17828251
rs78886656
rs75216873
rs113378129
rs58483275
rs139876813
rs9858561
rs7351079
rs2242310
rs4802858
rs11755998
rs3865444
All
−0.10
−0.05
0.00
0.05
0.10
MR leave−one−out sensitivity analysis for
'CD33 on CD14+ monocyte' on 'Juvenile arthritis'

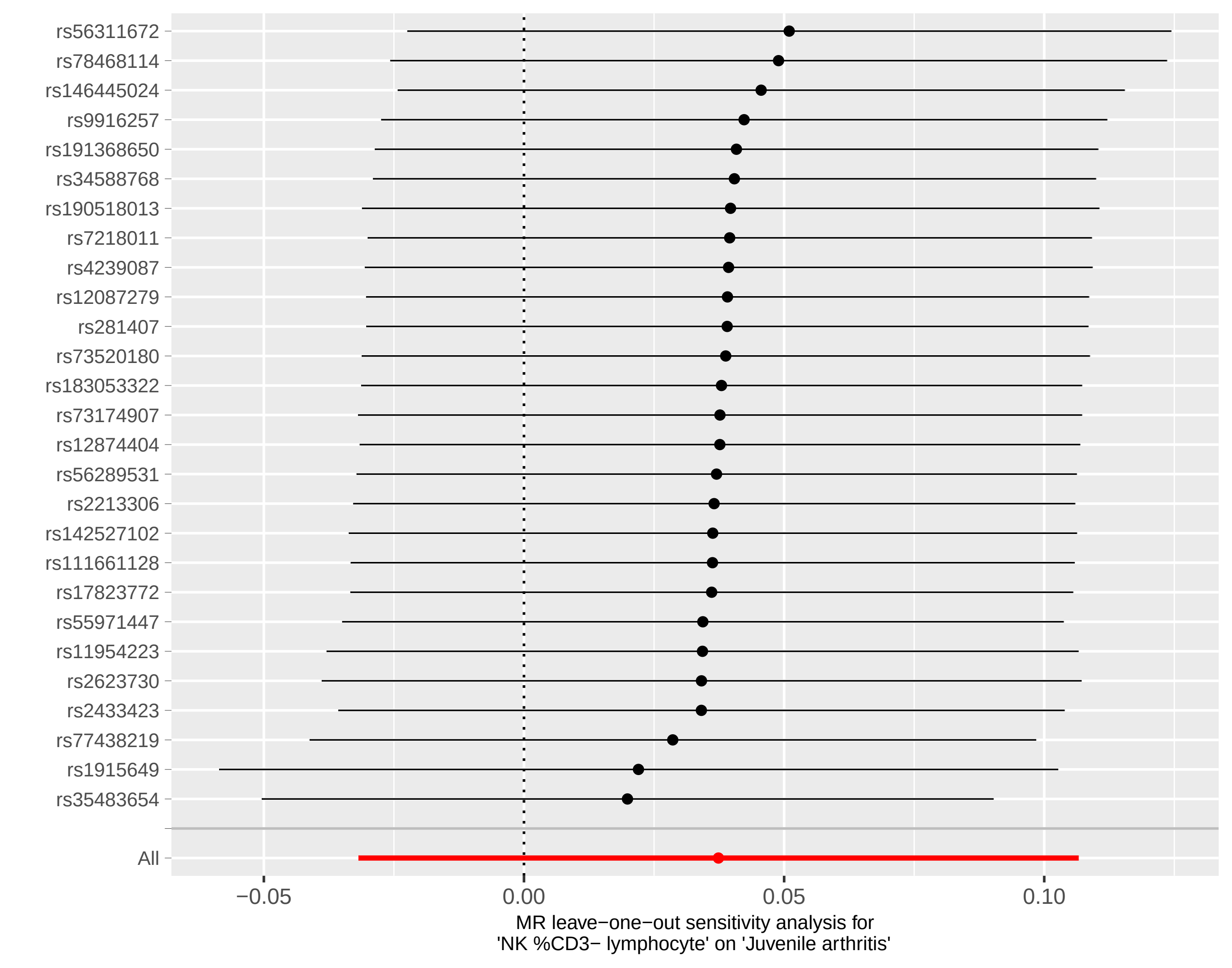
rs56311672
rs78468114
rs146445024
rs9916257
rs191368650
rs34588768
rs190518013
rs7218011
rs4239087
rs12087279
rs281407
rs73520180
rs183053322
rs73174907
rs12874404
rs56289531
rs2213306
rs142527102
rs111661128
rs17823772
rs55971447
rs11954223
rs2623730
rs2433423
rs77438219
rs1915649
rs35483654
All
−0.05
0.00
0.05
0.10
MR leave−one−out sensitivity analysis for
'NK %CD3− lymphocyte' on 'Juvenile arthritis'

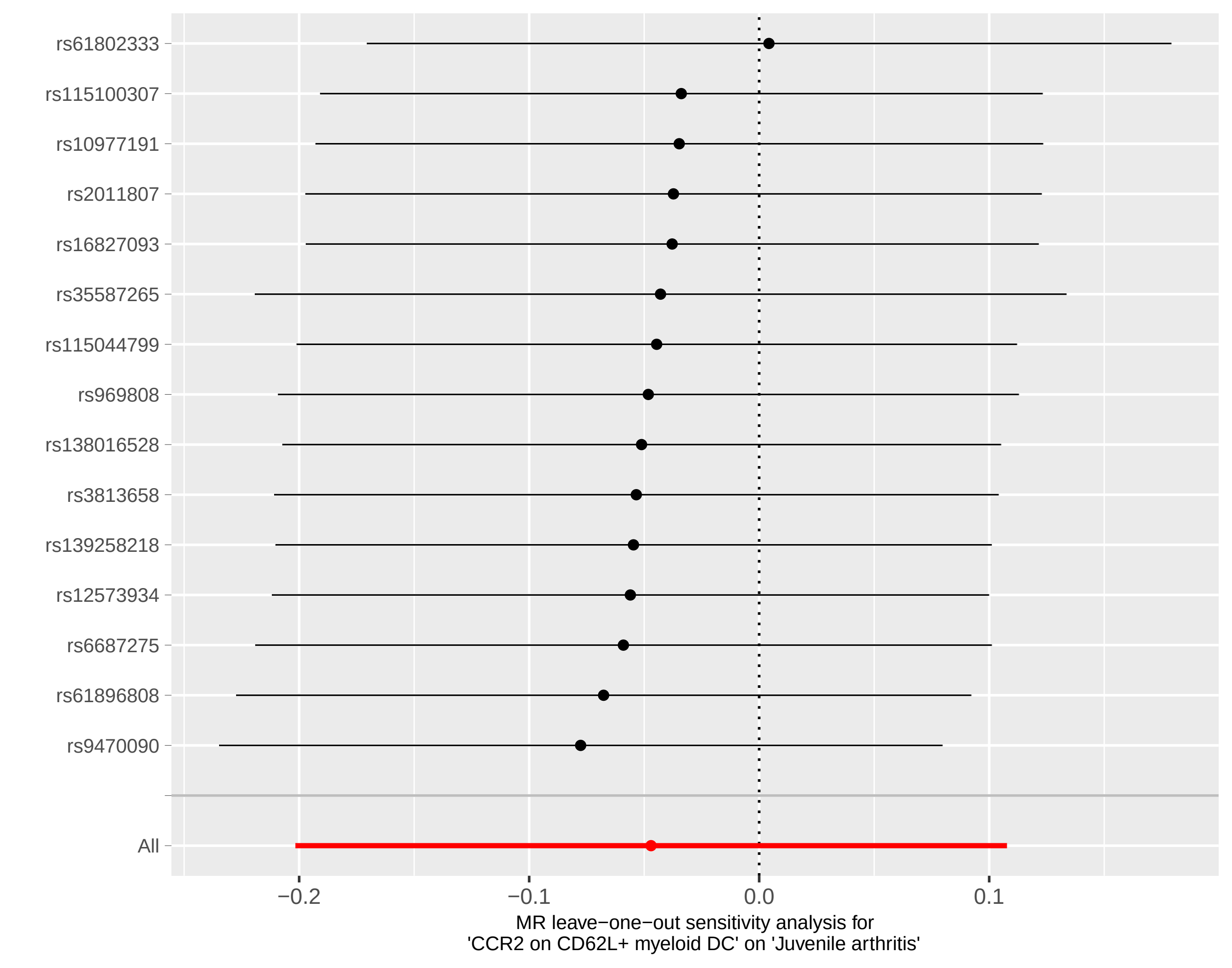
rs61802333
rs115100307
rs10977191
rs2011807
rs16827093
rs35587265
rs115044799
rs969808
rs138016528
rs3813658
rs139258218
rs12573934
rs6687275
rs61896808
rs9470090
All
−0.2
−0.1
0.0
0.1
MR leave−one−out sensitivity analysis for
'CCR2 on CD62L+ myeloid DC' on 'Juvenile arthritis'

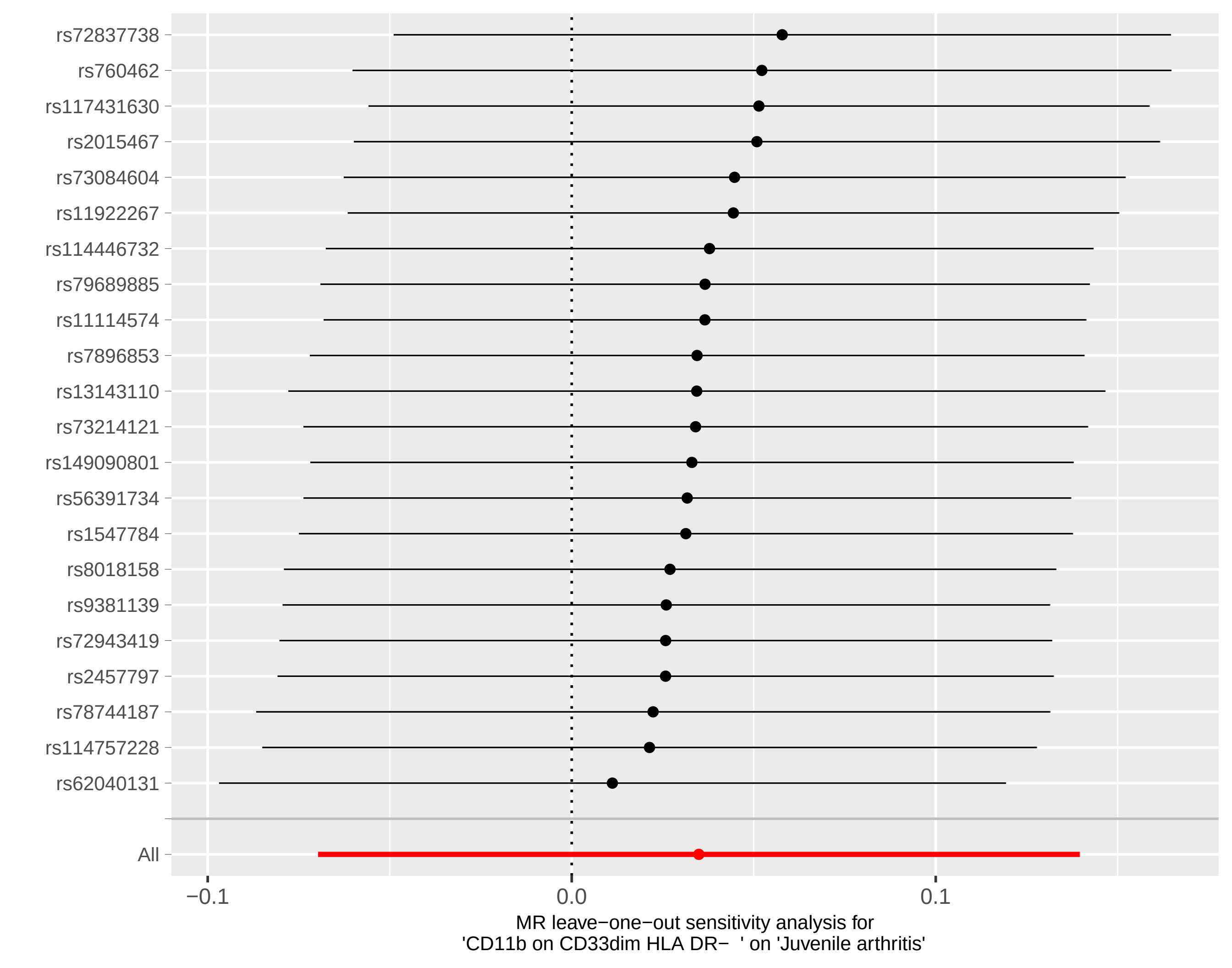

rs72837738
rs760462
rs117431630
rs2015467
rs73084604
rs11922267
rs114446732
rs79689885
rs11114574
rs7896853
rs13143110
rs73214121
rs149090801
rs56391734
rs1547784
rs8018158
rs9381139
rs72943419
rs2457797
rs78744187
rs114757228
rs62040131
All
−0.1
0.0
0.1
MR leave−one−out sensitivity analysis for
'CD11b on CD33dim HLA DR− ' on 'Juvenile arthritis'

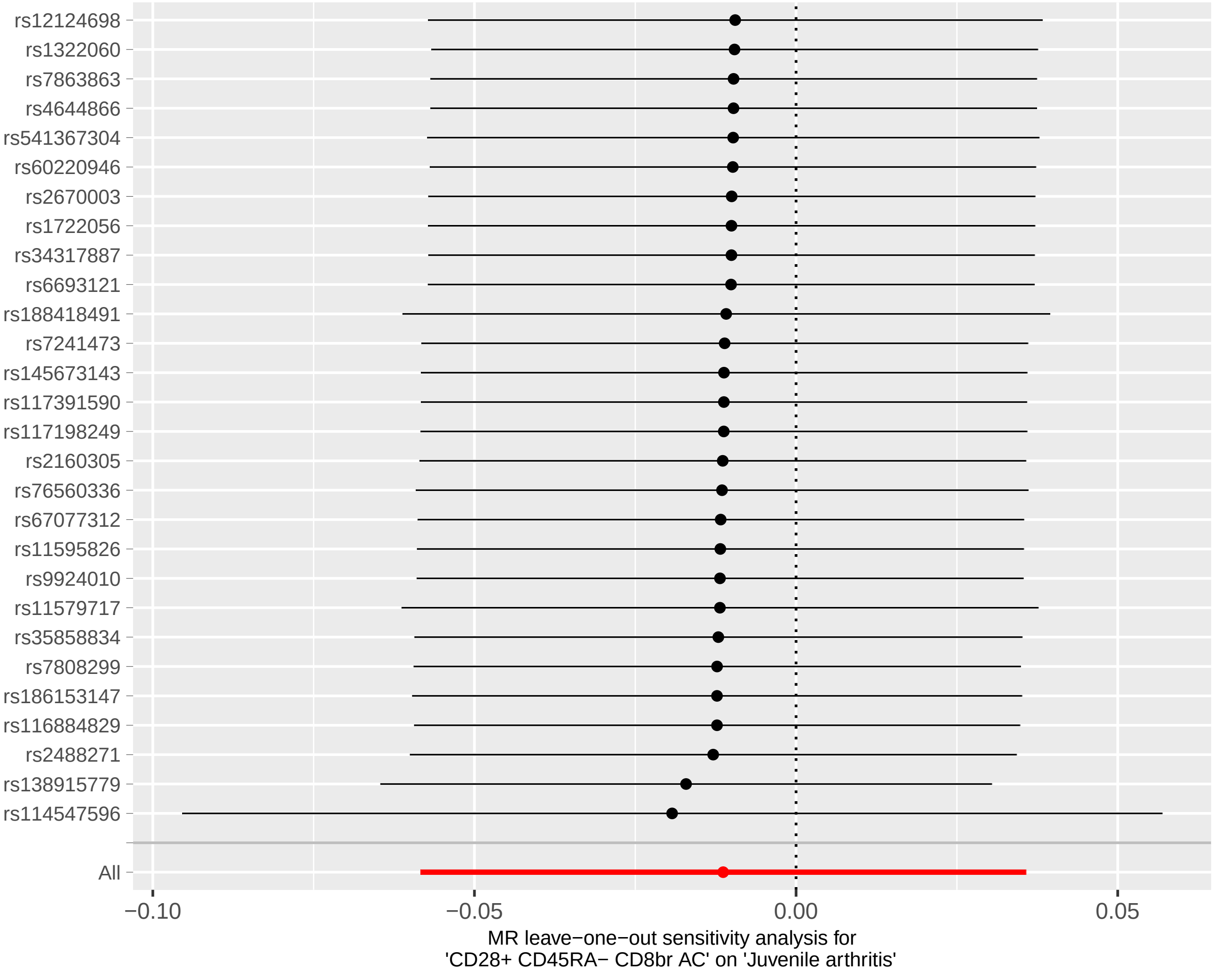

rs12124698
rs1322060
rs7863863
rs4644866
rs541367304
rs60220946
rs2670003
rs1722056
rs34317887
rs6693121
rs188418491
rs7241473
rs145673143
rs117391590
rs117198249
rs2160305
rs76560336
rs67077312
rs11595826
rs9924010
rs11579717
rs35858834
rs7808299
rs186153147
rs116884829
rs2488271
rs138915779
rs114547596
All
−0.10
−0.05
0.00
0.05
MR leave−one−out sensitivity analysis for
'CD28+ CD45RA− CD8br AC' on 'Juvenile arthritis'

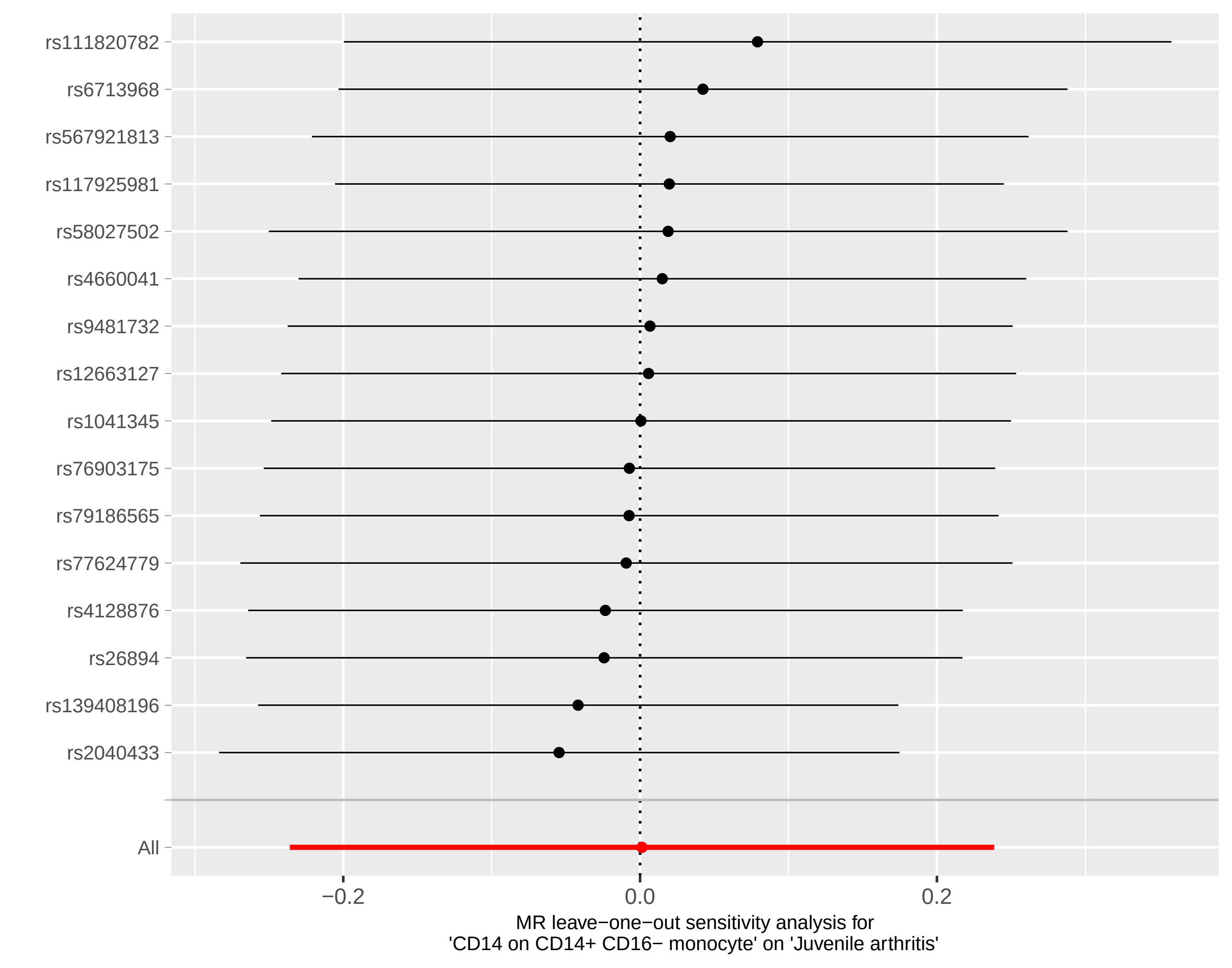
rs111820782
rs6713968
rs567921813
rs117925981
rs58027502
rs4660041
rs9481732
rs12663127
rs1041345
rs76903175
rs79186565
rs77624779
rs4128876
rs26894
rs139408196
rs2040433
All
−0.2
0.0
0.2
MR leave−one−out sensitivity analysis for
'CD14 on CD14+ CD16− monocyte' on 'Juvenile arthritis'

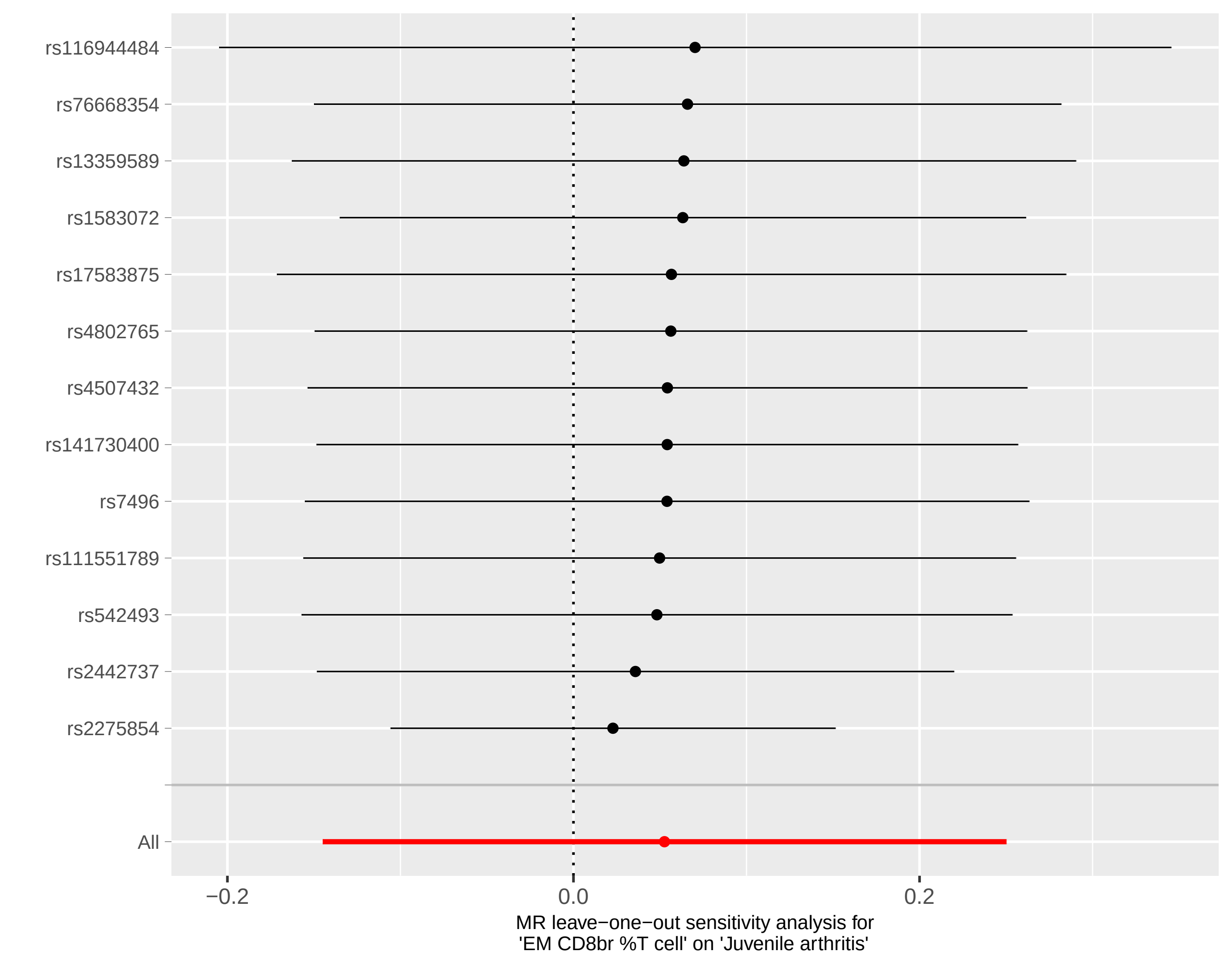
rs116944484
rs76668354
rs13359589
rs1583072
rs17583875
rs4802765
rs4507432
rs141730400
rs7496
rs111551789
rs542493
rs2442737
rs2275854
All
−0.2
0.0
0.2
MR leave−one−out sensitivity analysis for
'EM CD8br %T cell' on 'Juvenile arthritis'

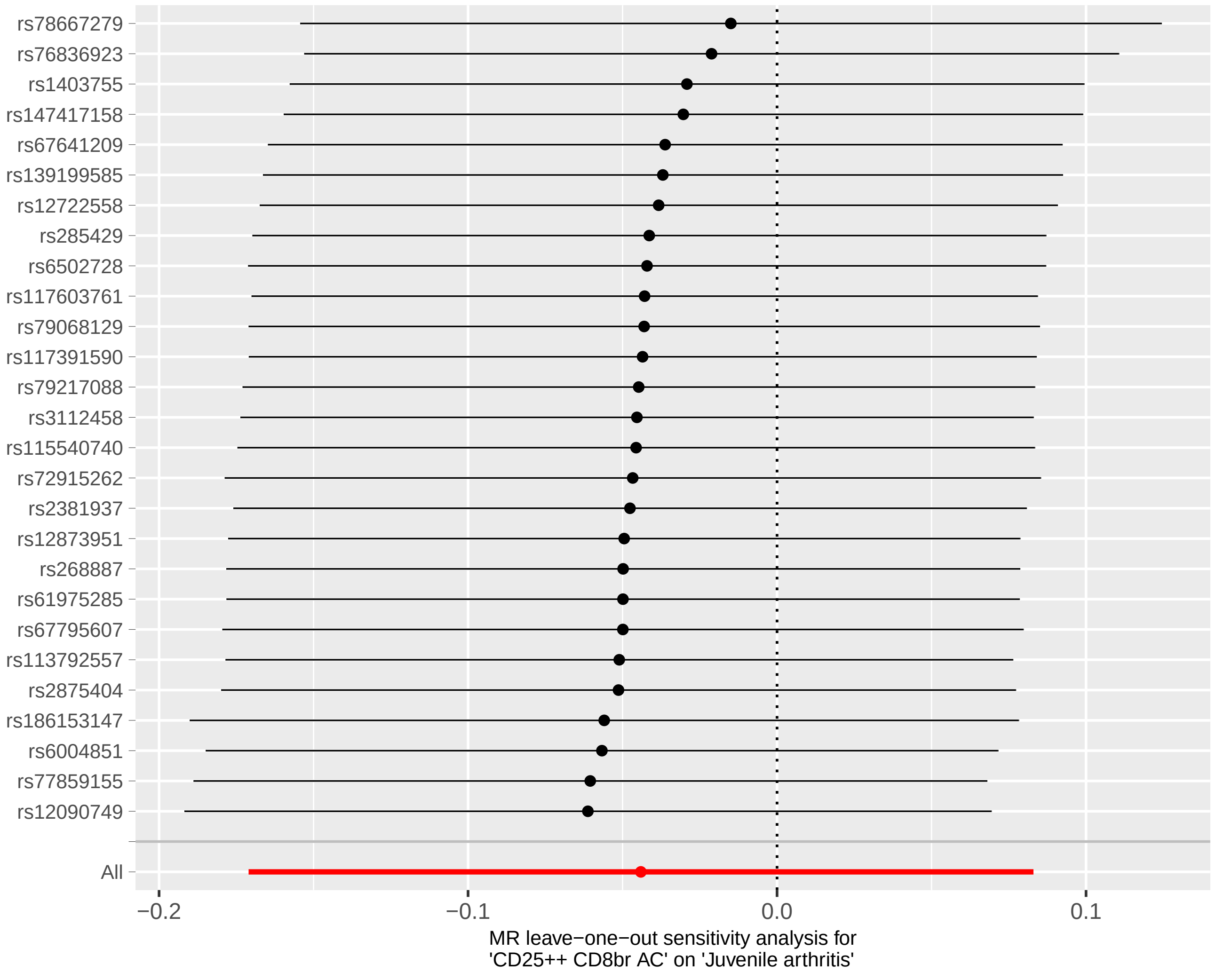

rs78667279
rs76836923
rs1403755
rs147417158
rs67641209
rs139199585
rs12722558
rs285429
rs6502728
rs117603761
rs79068129
rs117391590
rs79217088
rs3112458
rs115540740
rs72915262
rs2381937
rs12873951
rs268887
rs61975285
rs67795607
rs113792557
rs2875404
rs186153147
rs6004851
rs77859155
rs12090749
All
−0.2
−0.1
0.0
0.1
MR leave−one−out sensitivity analysis for
'CD25++ CD8br AC' on 'Juvenile arthritis'

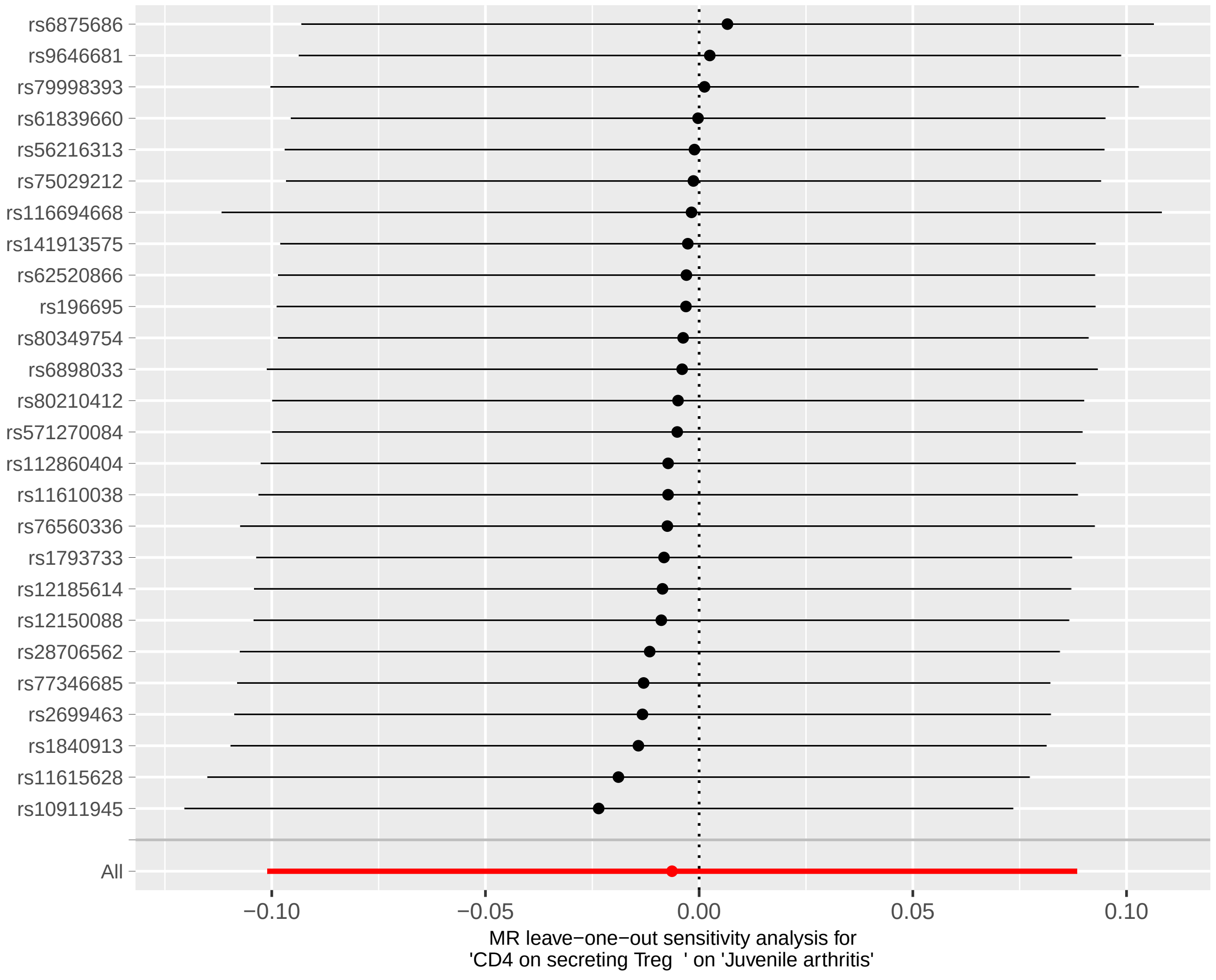
rs6875686
rs9646681
rs79998393
rs61839660
rs56216313
rs75029212
rs116694668
rs141913575
rs62520866
rs196695
rs80349754
rs6898033
rs80210412
rs571270084
rs112860404
rs11610038
rs76560336
rs1793733
rs12185614
rs12150088
rs28706562
rs77346685
rs2699463
rs1840913
rs11615628
rs10911945
All
−0.10
−0.05
0.00
0.05
0.10
MR leave−one−out sensitivity analysis for
'CD4 on secreting Treg ' on 'Juvenile arthritis'

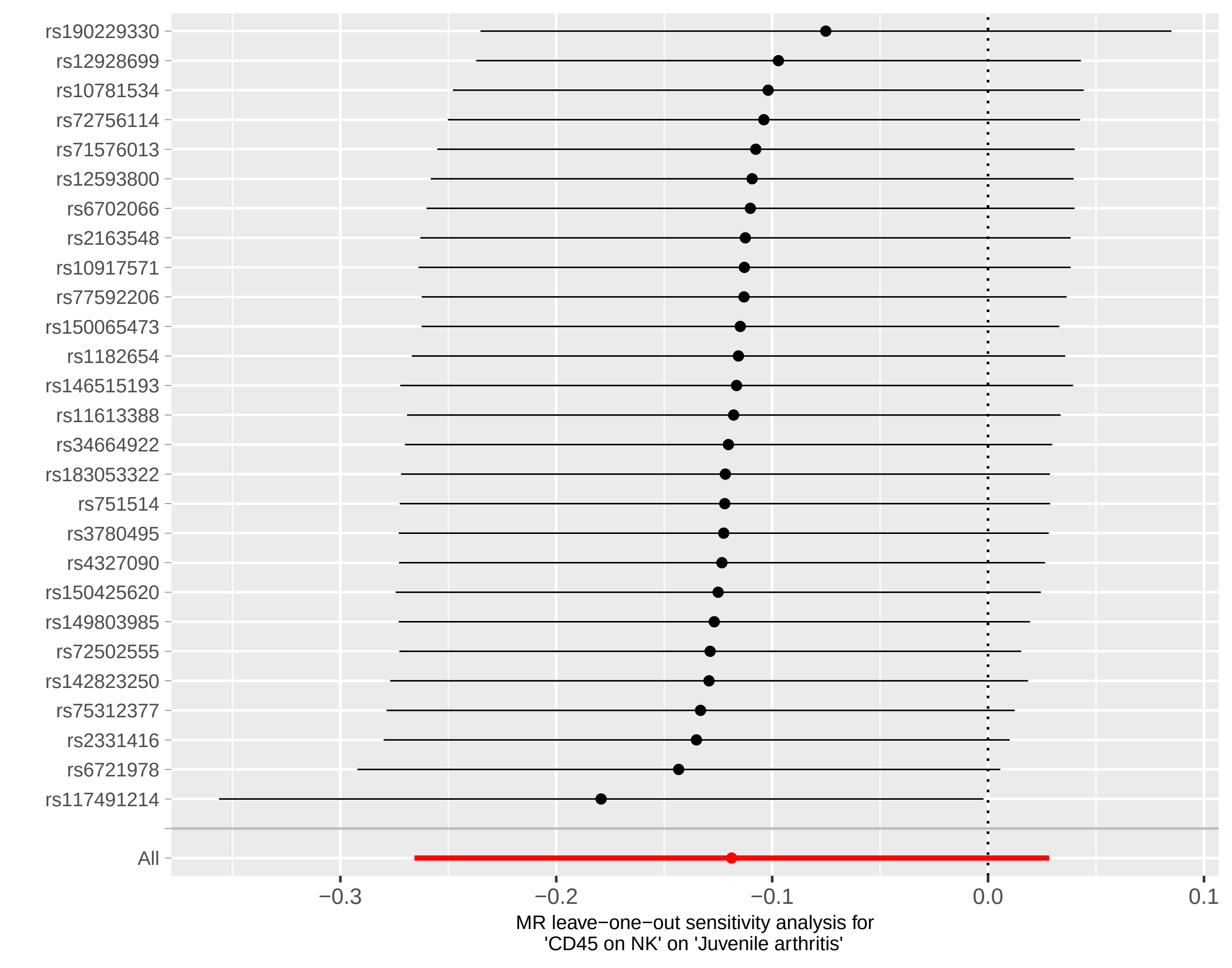

rs190229330
rs12928699
rs10781534
rs72756114
rs71576013
rs12593800
rs6702066
rs2163548
rs10917571
rs77592206
rs150065473
rs1182654
rs146515193
rs11613388
rs34664922
rs183053322
rs751514
rs3780495
rs4327090
rs150425620
rs149803985
rs72502555
rs142823250
rs75312377
rs2331416
rs6721978
rs117491214
All
−0.3
−0.2
−0.1
0.0
0.1
MR leave−one−out sensitivity analysis for
'CD45 on NK' on 'Juvenile arthritis'

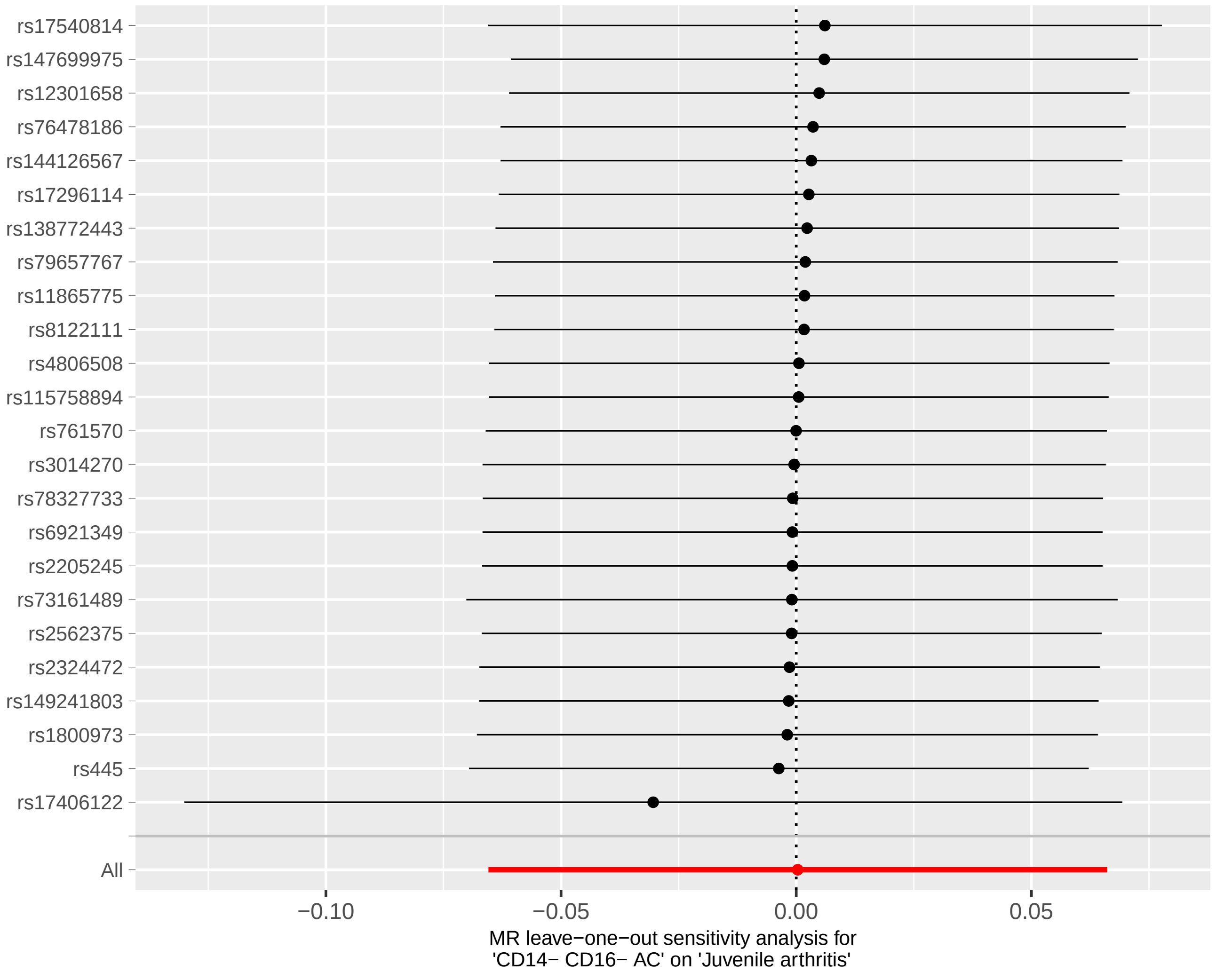
rs17540814
rs147699975
rs12301658
rs76478186
rs144126567
rs17296114
rs138772443
rs79657767
rs11865775
rs8122111
rs4806508
rs115758894
rs761570
rs3014270
rs78327733
rs6921349
rs2205245
rs73161489
rs2562375
rs2324472
rs149241803
rs1800973
rs445
rs17406122
All
−0.10
−0.05
0.00
0.05
MR leave−one−out sensitivity analysis for
'CD14− CD16− AC' on 'Juvenile arthritis'

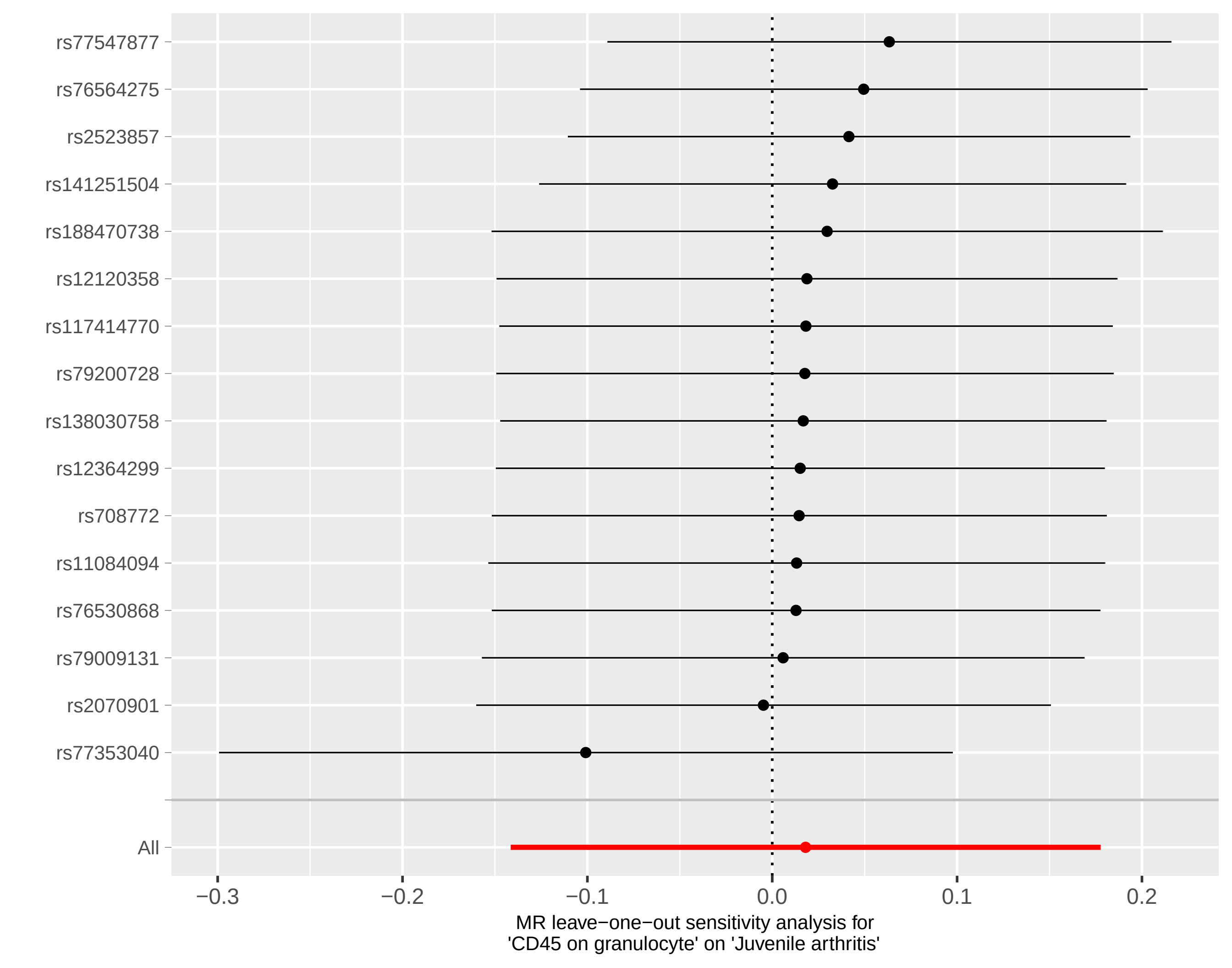
rs77547877
rs76564275
rs2523857
rs141251504
rs188470738
rs12120358
rs117414770
rs79200728
rs138030758
rs12364299
rs708772
rs11084094
rs76530868
rs79009131
rs2070901
rs77353040
All
−0.3
−0.2
−0.1
0.0
0.1
0.2
MR leave−one−out sensitivity analysis for
'CD45 on granulocyte' on 'Juvenile arthritis'

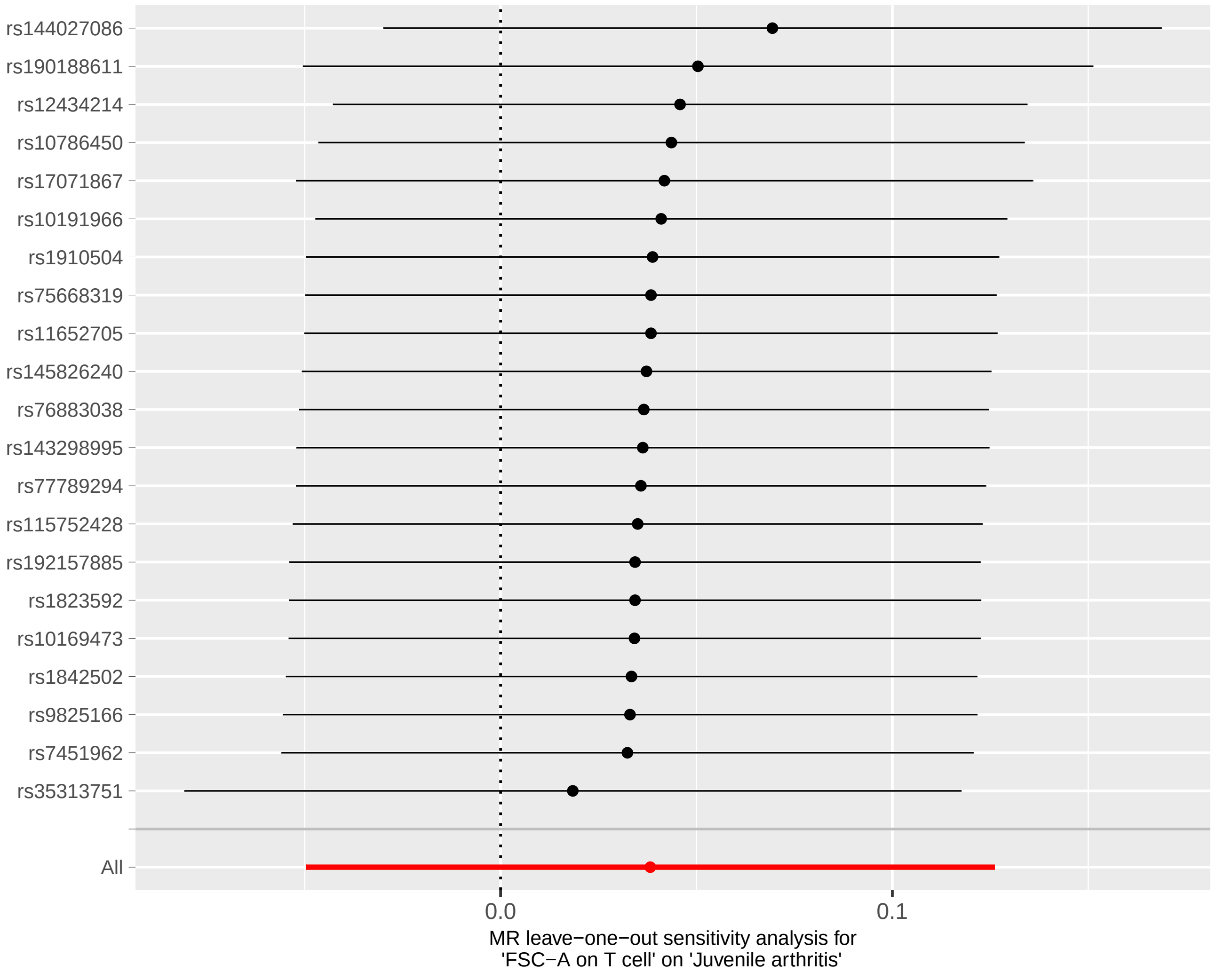
rs144027086
rs190188611
rs12434214
rs10786450
rs17071867
rs10191966
rs1910504
rs75668319
rs11652705
rs145826240
rs76883038
rs143298995
rs77789294
rs115752428
rs192157885
rs1823592
rs10169473
rs1842502
rs9825166
rs7451962
rs35313751
All
0.0
0.1
MR leave−one−out sensitivity analysis for
'FSC−A on T cell' on 'Juvenile arthritis'

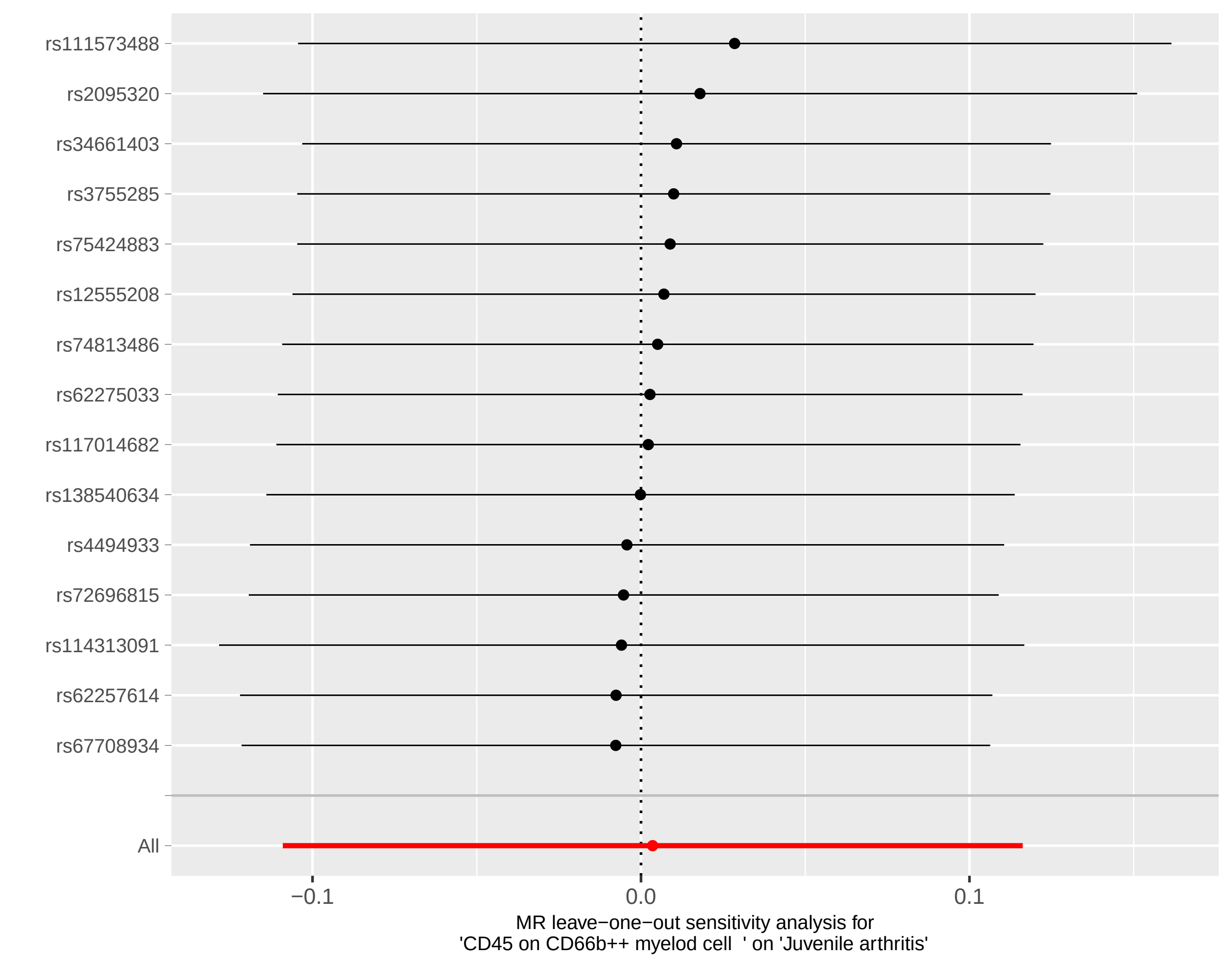
rs111573488
rs2095320
rs34661403
rs3755285
rs75424883
rs12555208
rs74813486
rs62275033
rs117014682
rs138540634
rs4494933
rs72696815
rs114313091
rs62257614
rs67708934
All
−0.1
0.0
0.1
MR leave−one−out sensitivity analysis for
'CD45 on CD66b++ myelod cell ' on 'Juvenile arthritis'

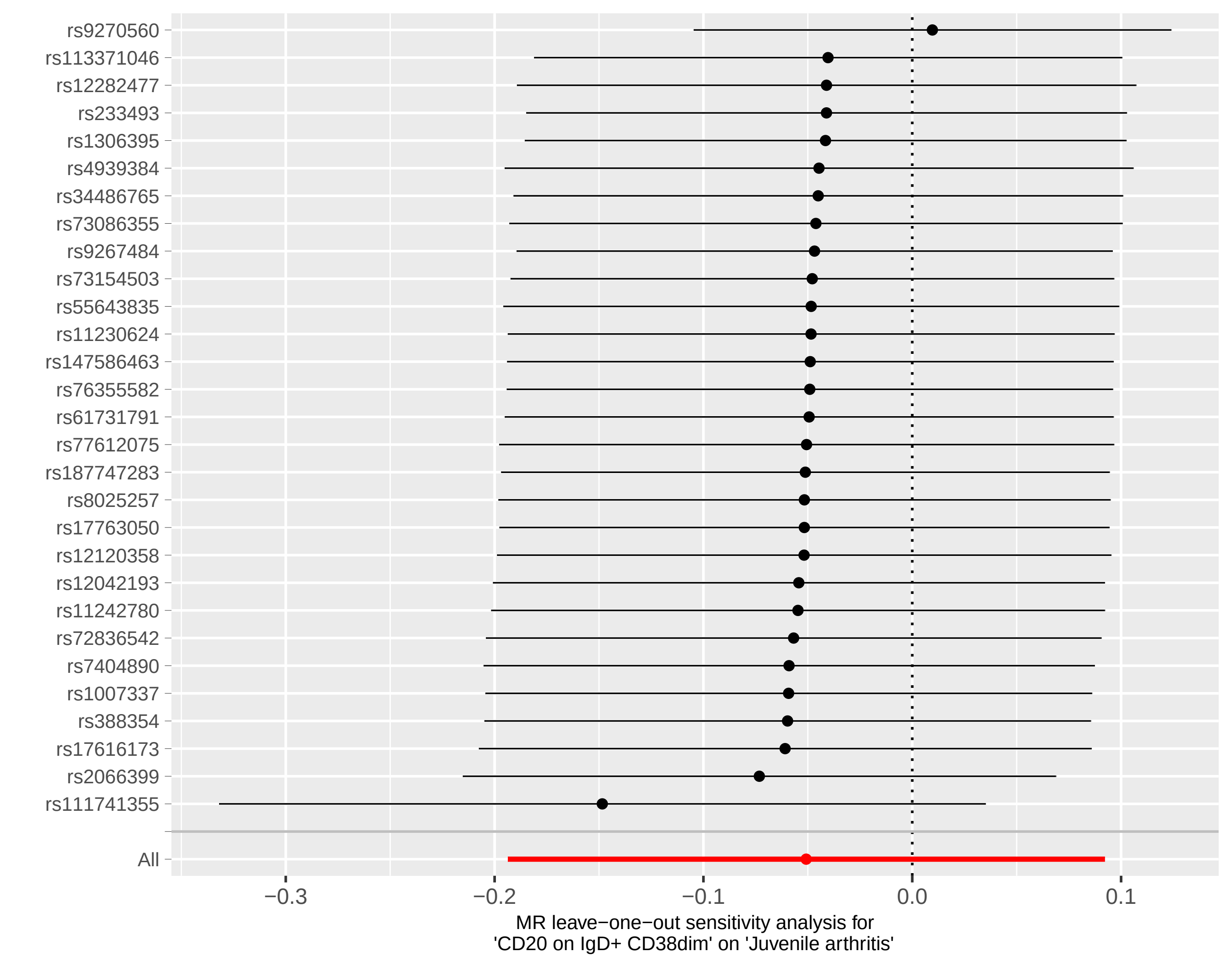
rs9270560
rs113371046
rs12282477
rs233493
rs1306395
rs4939384
rs34486765
rs73086355
rs9267484
rs73154503
rs55643835
rs11230624
rs147586463
rs76355582
rs61731791
rs77612075
rs187747283
rs8025257
rs17763050
rs12120358
rs12042193
rs11242780
rs72836542
rs7404890
rs1007337
rs388354
rs17616173
rs2066399
rs111741355
All
−0.3
−0.2
−0.1
0.0
0.1
MR leave−one−out sensitivity analysis for
'CD20 on IgD+ CD38dim' on 'Juvenile arthritis'

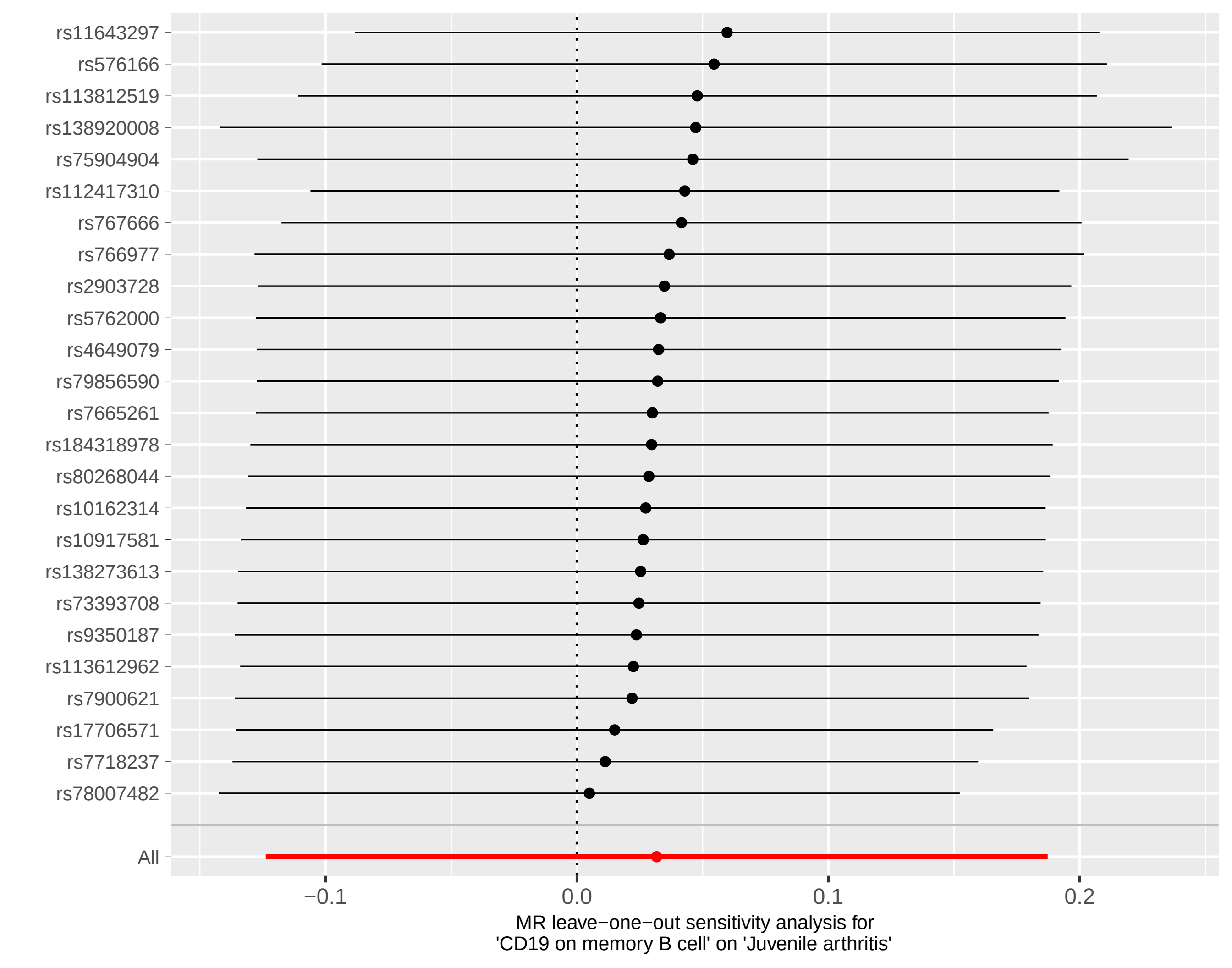

rs11643297
rs576166
rs113812519
rs138920008
rs75904904
rs112417310
rs767666
rs766977
rs2903728
rs5762000
rs4649079
rs79856590
rs7665261
rs184318978
rs80268044
rs10162314
rs10917581
rs138273613
rs73393708
rs9350187
rs113612962
rs7900621
rs17706571
rs7718237
rs78007482
All
−0.1
0.0
0.1
0.2
MR leave−one−out sensitivity analysis for
'CD19 on memory B cell' on 'Juvenile arthritis'

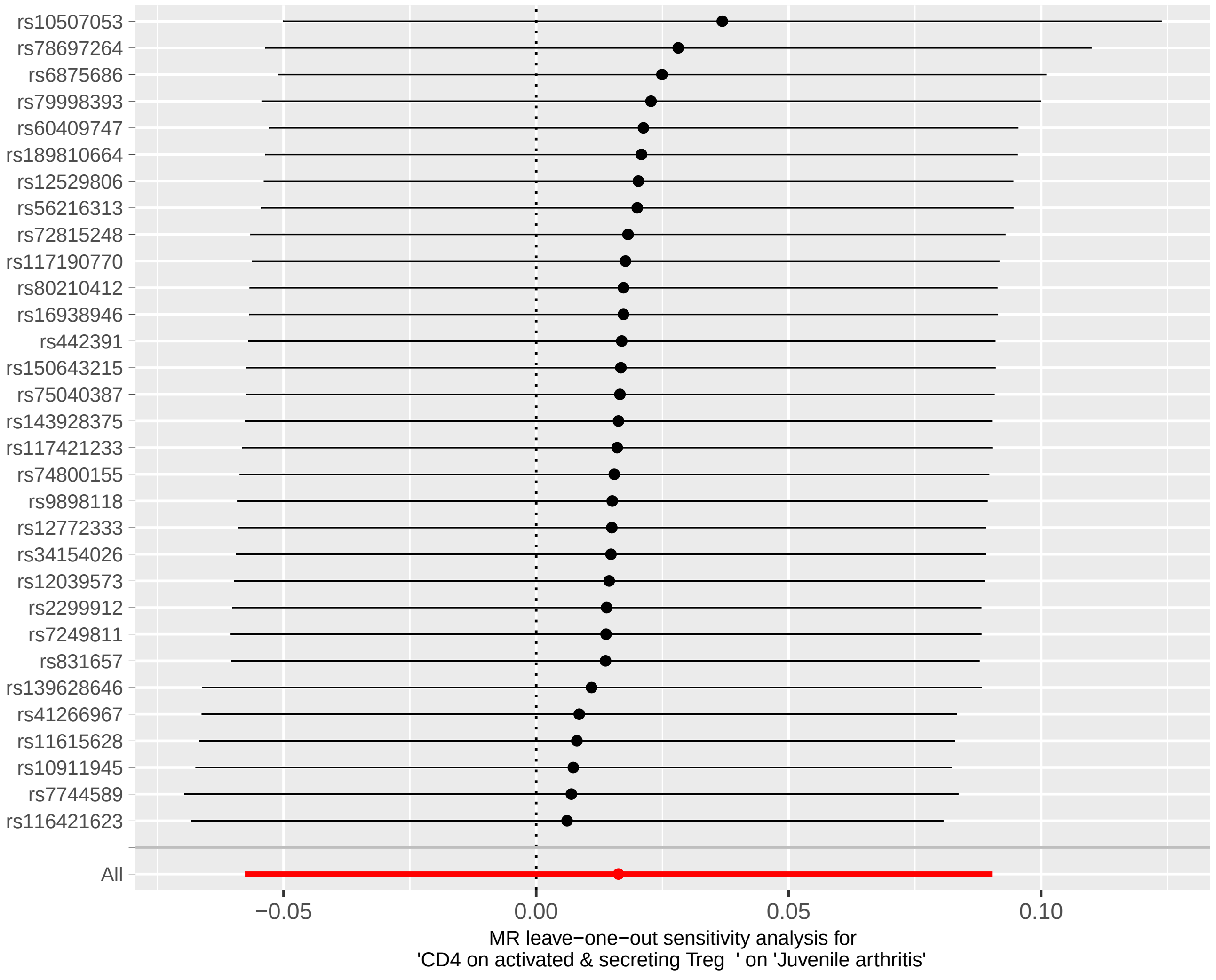

rs10507053
rs78697264
rs6875686
rs79998393
rs60409747
rs189810664
rs12529806
rs56216313
rs72815248
rs117190770
rs80210412
rs16938946
rs442391
rs150643215
rs75040387
rs143928375
rs117421233
rs74800155
rs9898118
rs12772333
rs34154026
rs12039573
rs2299912
rs7249811
rs831657
rs139628646
rs41266967
rs11615628
rs10911945
rs7744589
rs116421623
All
−0.05
0.00
0.05
0.10
MR leave−one−out sensitivity analysis for
'CD4 on activated & secreting Treg ' on 'Juvenile arthritis'

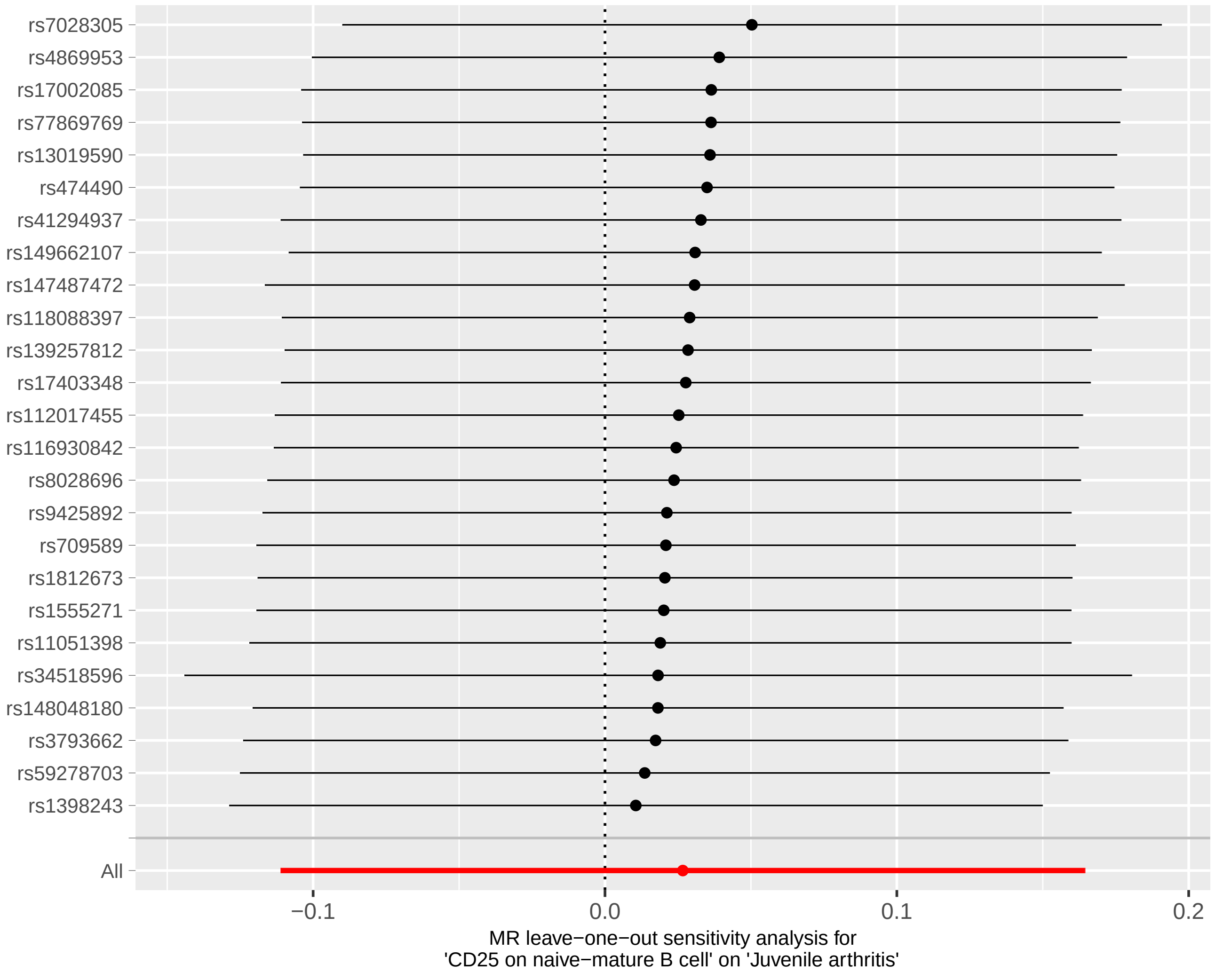
rs7028305
rs4869953
rs17002085
rs77869769
rs13019590
rs474490
rs41294937
rs149662107
rs147487472
rs118088397
rs139257812
rs17403348
rs112017455
rs116930842
rs8028696
rs9425892
rs709589
rs1812673
rs1555271
rs11051398
rs34518596
rs148048180
rs3793662
rs59278703
rs1398243
All
−0.1
0.0
0.1
0.2
MR leave−one−out sensitivity analysis for
'CD25 on naive−mature B cell' on 'Juvenile arthritis'

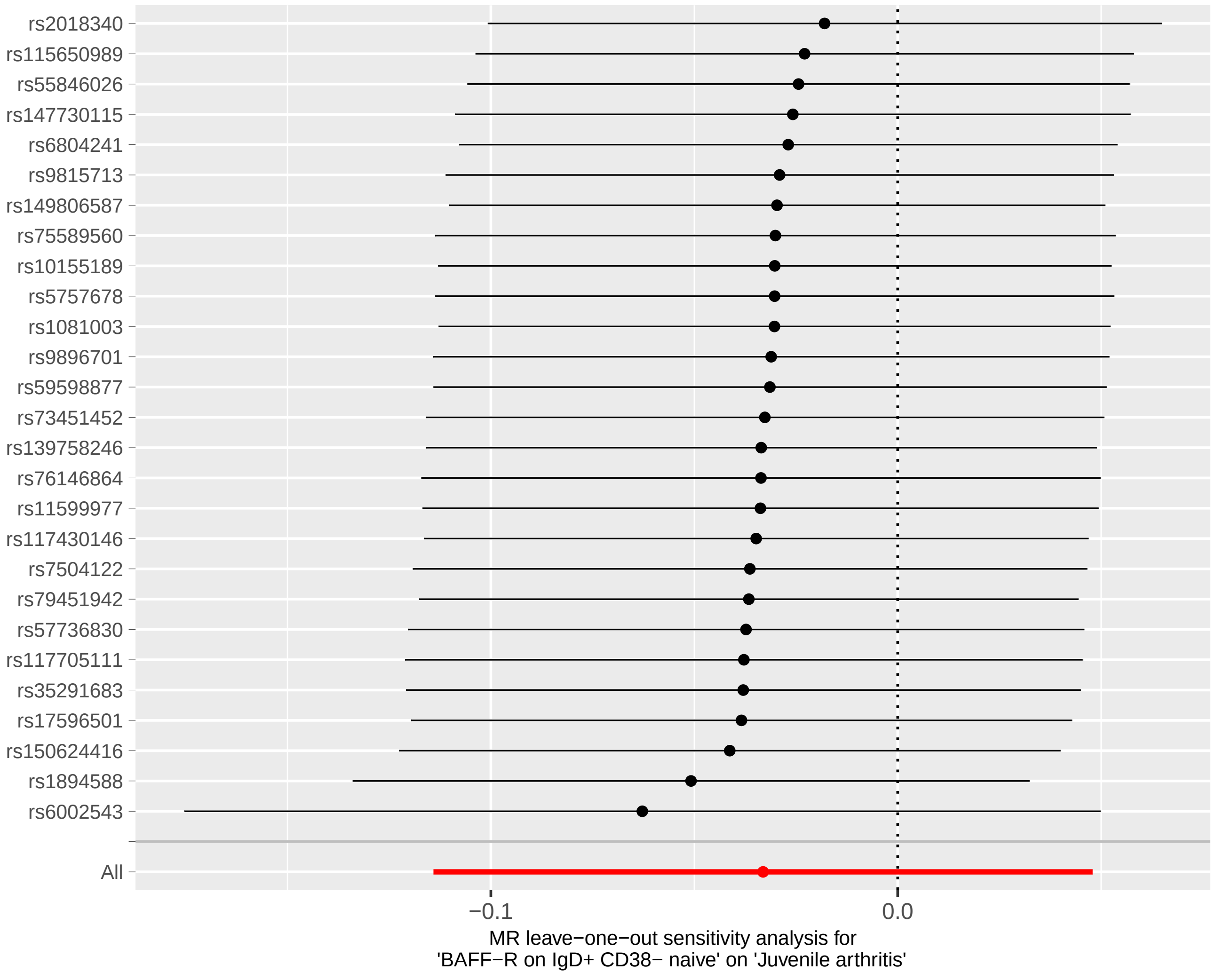

rs2018340
rs115650989
rs55846026
rs147730115
rs6804241
rs9815713
rs149806587
rs75589560
rs10155189
rs5757678
rs1081003
rs9896701
rs59598877
rs73451452
rs139758246
rs76146864
rs11599977
rs117430146
rs7504122
rs79451942
rs57736830
rs117705111
rs35291683
rs17596501
rs150624416
rs1894588
rs6002543
All
−0.1
0.0
MR leave−one−out sensitivity analysis for
'BAFF−R on IgD+ CD38− naive' on 'Juvenile arthritis'

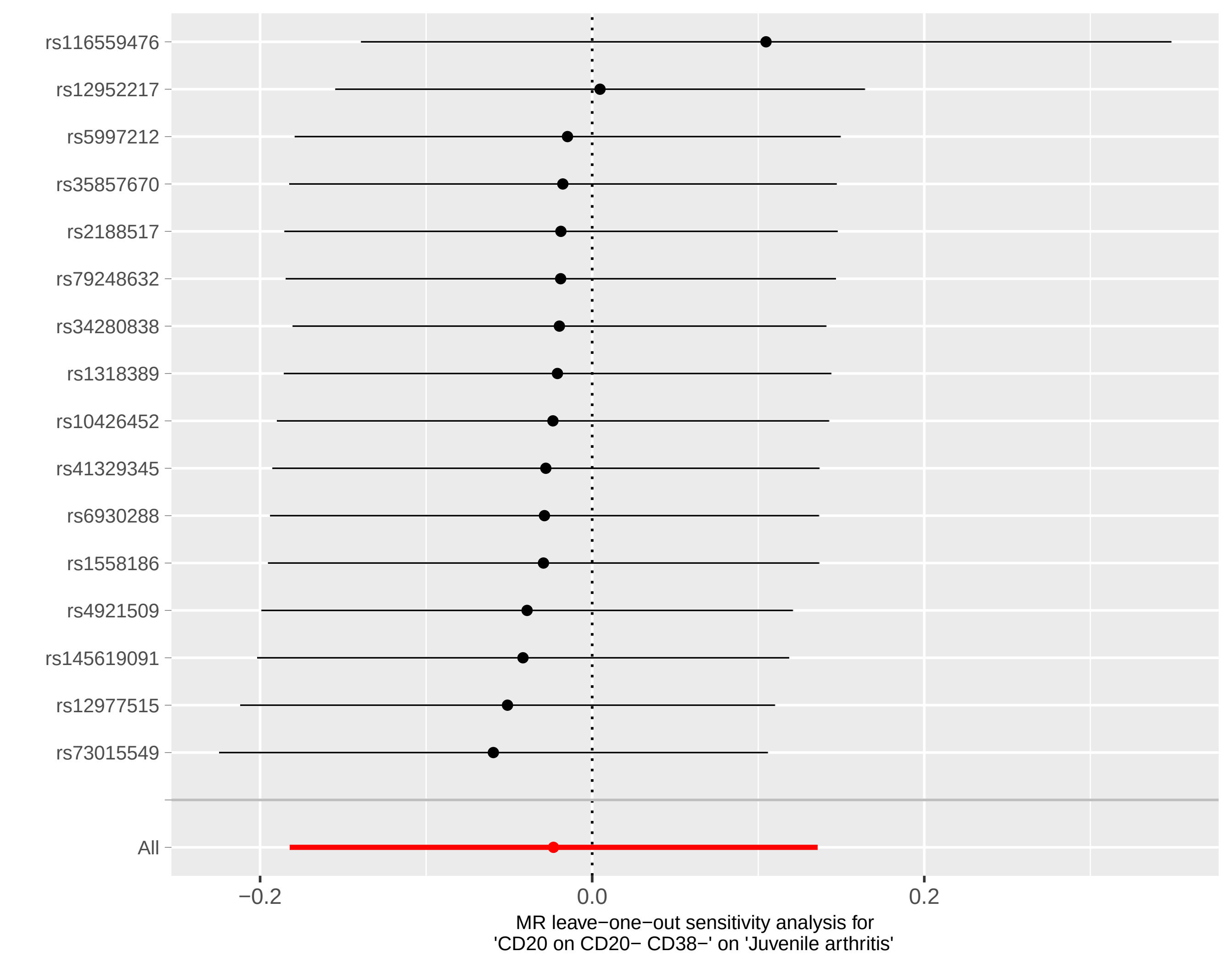
rs116559476
rs12952217
rs5997212
rs35857670
rs2188517
rs79248632
rs34280838
rs1318389
rs10426452
rs41329345
rs6930288
rs1558186
rs4921509
rs145619091
rs12977515
rs73015549
All
−0.2
0.0
0.2
MR leave−one−out sensitivity analysis for
'CD20 on CD20− CD38−' on 'Juvenile arthritis'

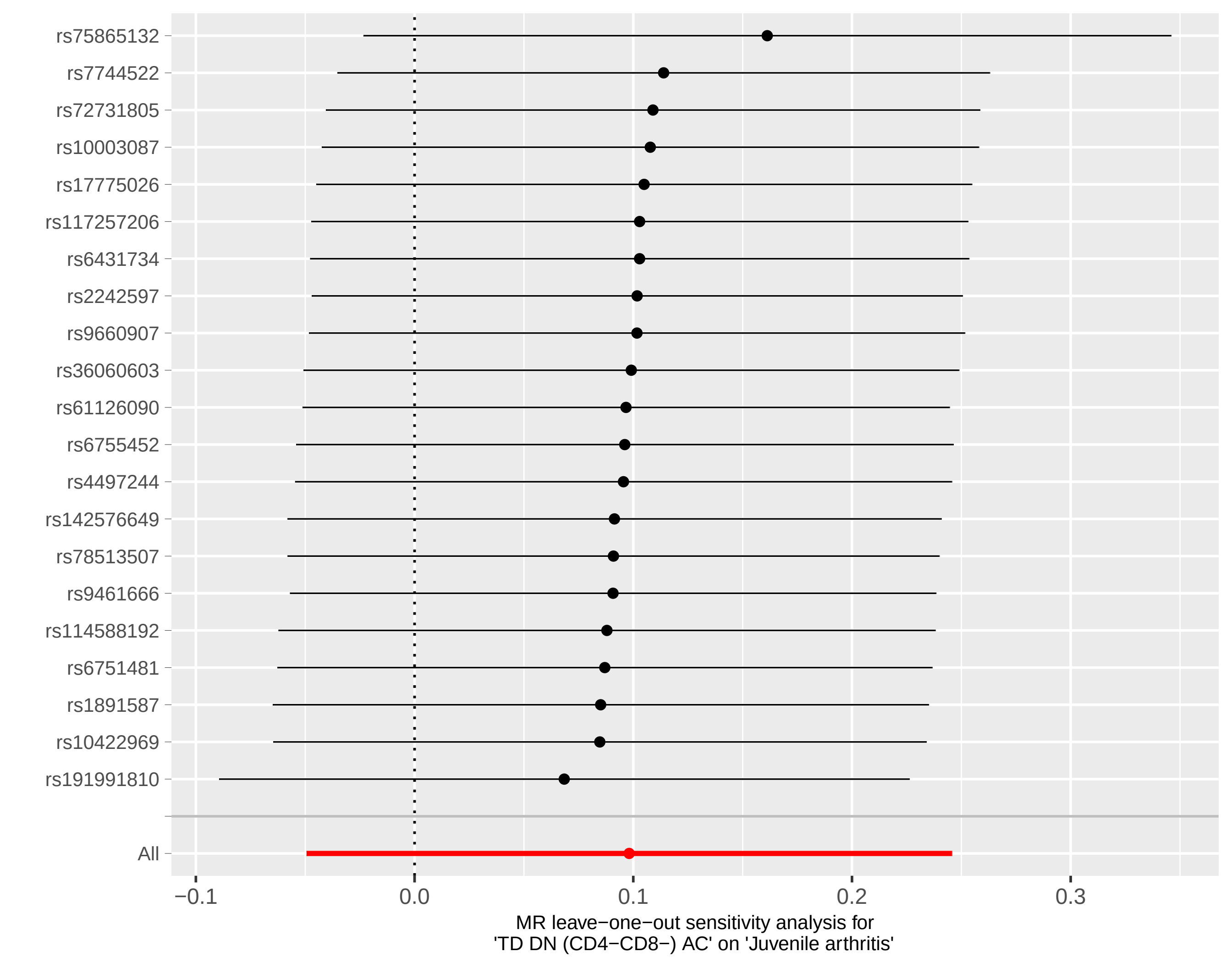

rs75865132
rs7744522
rs72731805
rs10003087
rs17775026
rs117257206
rs6431734
rs2242597
rs9660907
rs36060603
rs61126090
rs6755452
rs4497244
rs142576649
rs78513507
rs9461666
rs114588192
rs6751481
rs1891587
rs10422969
rs191991810
All
−0.1
0.0
0.1
0.2
0.3
MR leave−one−out sensitivity analysis for
'TD DN (CD4−CD8−) AC' on 'Juvenile arthritis'

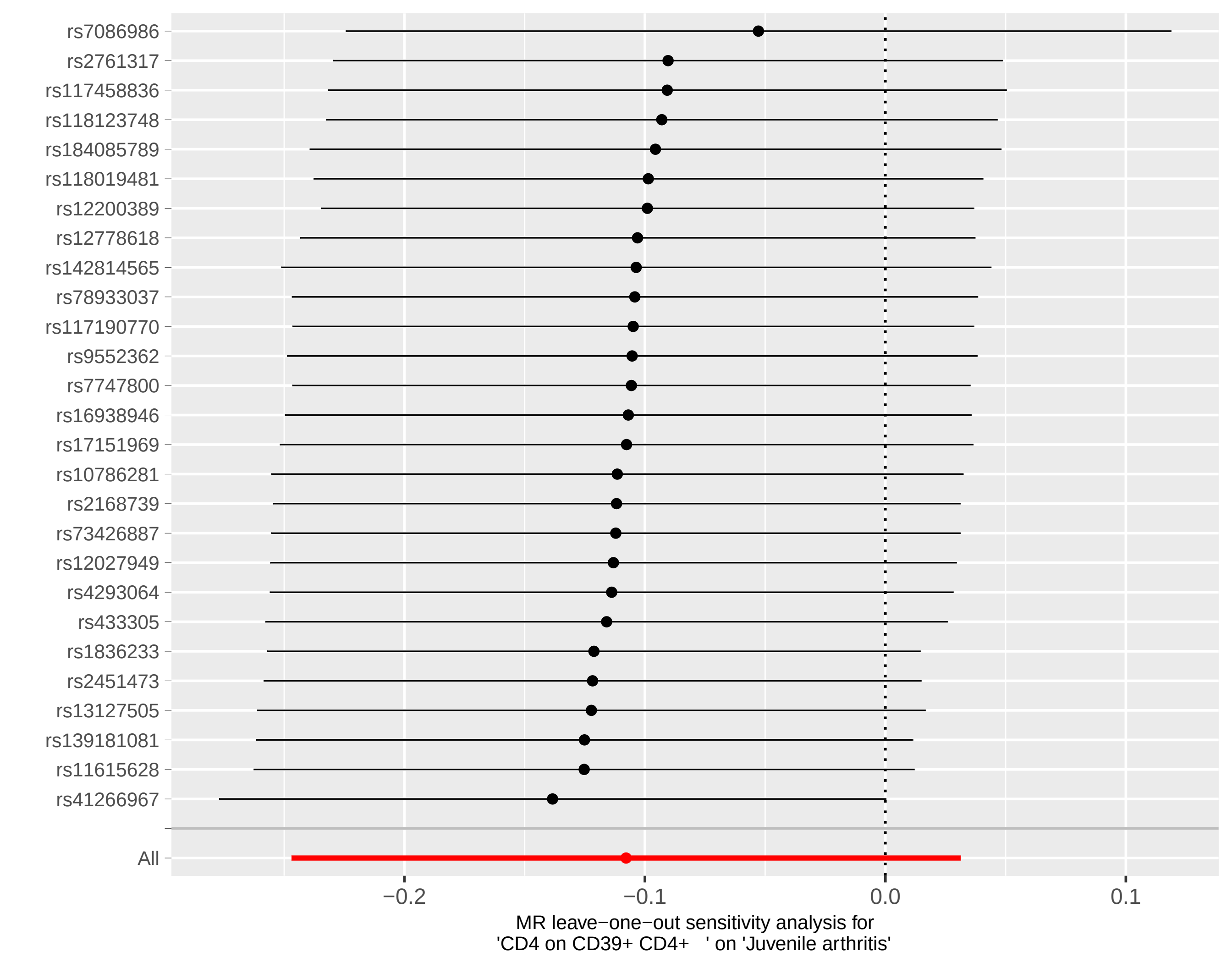

rs7086986
rs2761317
rs117458836
rs118123748
rs184085789
rs118019481
rs12200389
rs12778618
rs142814565
rs78933037
rs117190770
rs9552362
rs7747800
rs16938946
rs17151969
rs10786281
rs2168739
rs73426887
rs12027949
rs4293064
rs433305
rs1836233
rs2451473
rs13127505
rs139181081
rs11615628
rs41266967
All
−0.2
−0.1
0.0
0.1
MR leave−one−out sensitivity analysis for
'CD4 on CD39+ CD4+ ' on 'Juvenile arthritis'

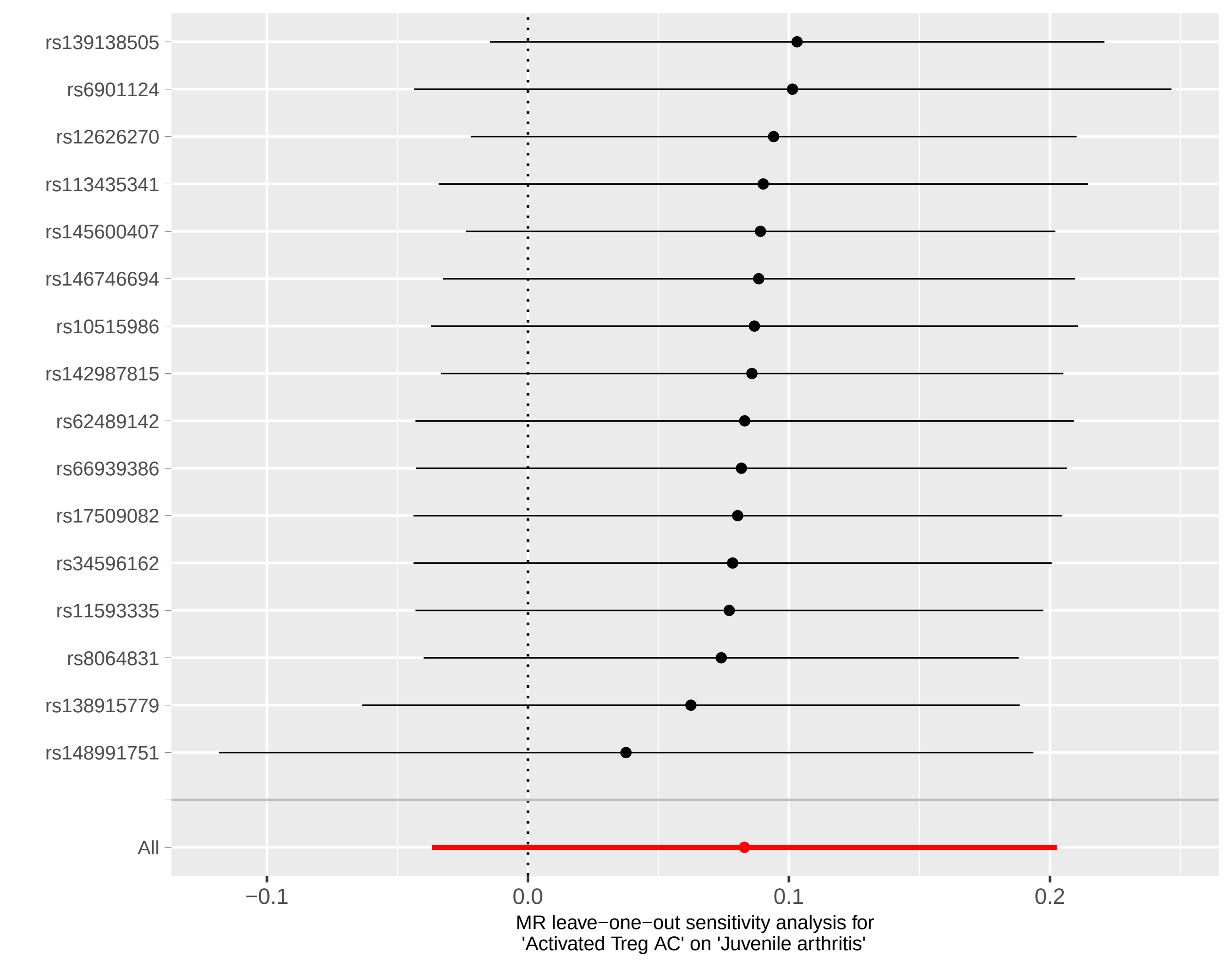
rs139138505
rs6901124
rs12626270
rs113435341
rs145600407
rs146746694
rs10515986
rs142987815
rs62489142
rs66939386
rs17509082
rs34596162
rs11593335
rs8064831
rs138915779
rs148991751
All
−0.1
0.0
0.1
0.2
MR leave−one−out sensitivity analysis for
'Activated Treg AC' on 'Juvenile arthritis'

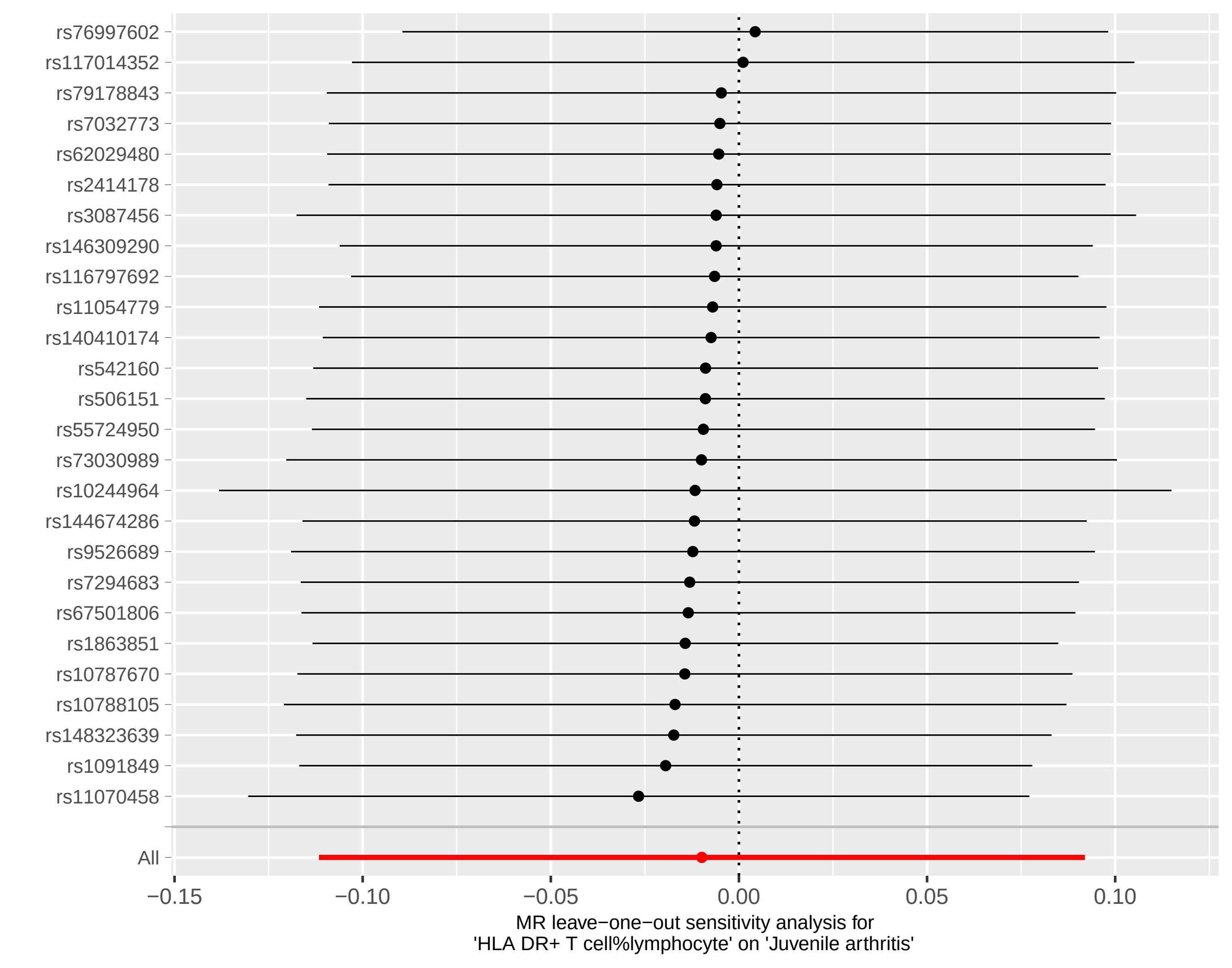
rs76997602
rs117014352
rs79178843
rs7032773
rs62029480
rs2414178
rs3087456
rs146309290
rs116797692
rs11054779
rs140410174
rs542160
rs506151
rs55724950
rs73030989
rs10244964
rs144674286
rs9526689
rs7294683
rs67501806
rs1863851
rs10787670
rs10788105
rs148323639
rs1091849
rs11070458
All
−0.15
−0.10
−0.05
0.00
0.05
0.10
MR leave−one−out sensitivity analysis for
'HLA DR+ T cell%lymphocyte' on 'Juvenile arthritis'

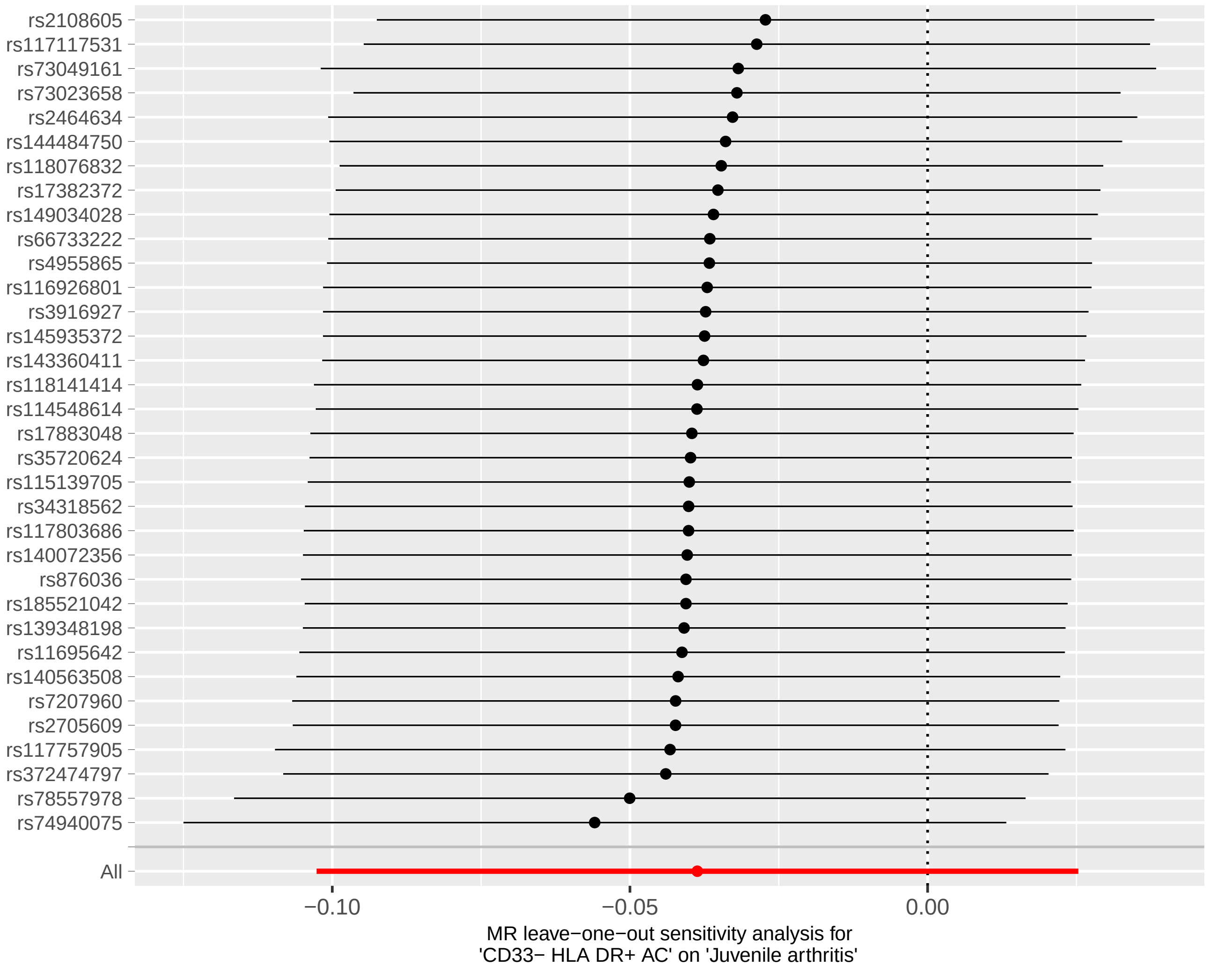

rs2108605
rs117117531
rs73049161
rs73023658
rs2464634
rs144484750
rs118076832
rs17382372
rs149034028
rs66733222
rs4955865
rs116926801
rs3916927
rs145935372
rs143360411
rs118141414
rs114548614
rs17883048
rs35720624
rs115139705
rs34318562
rs117803686
rs140072356
rs876036
rs185521042
rs139348198
rs11695642
rs140563508
rs7207960
rs2705609
rs117757905
rs372474797
rs78557978
rs74940075
All
−0.10
−0.05
0.00
MR leave−one−out sensitivity analysis for
'CD33− HLA DR+ AC' on 'Juvenile arthritis'

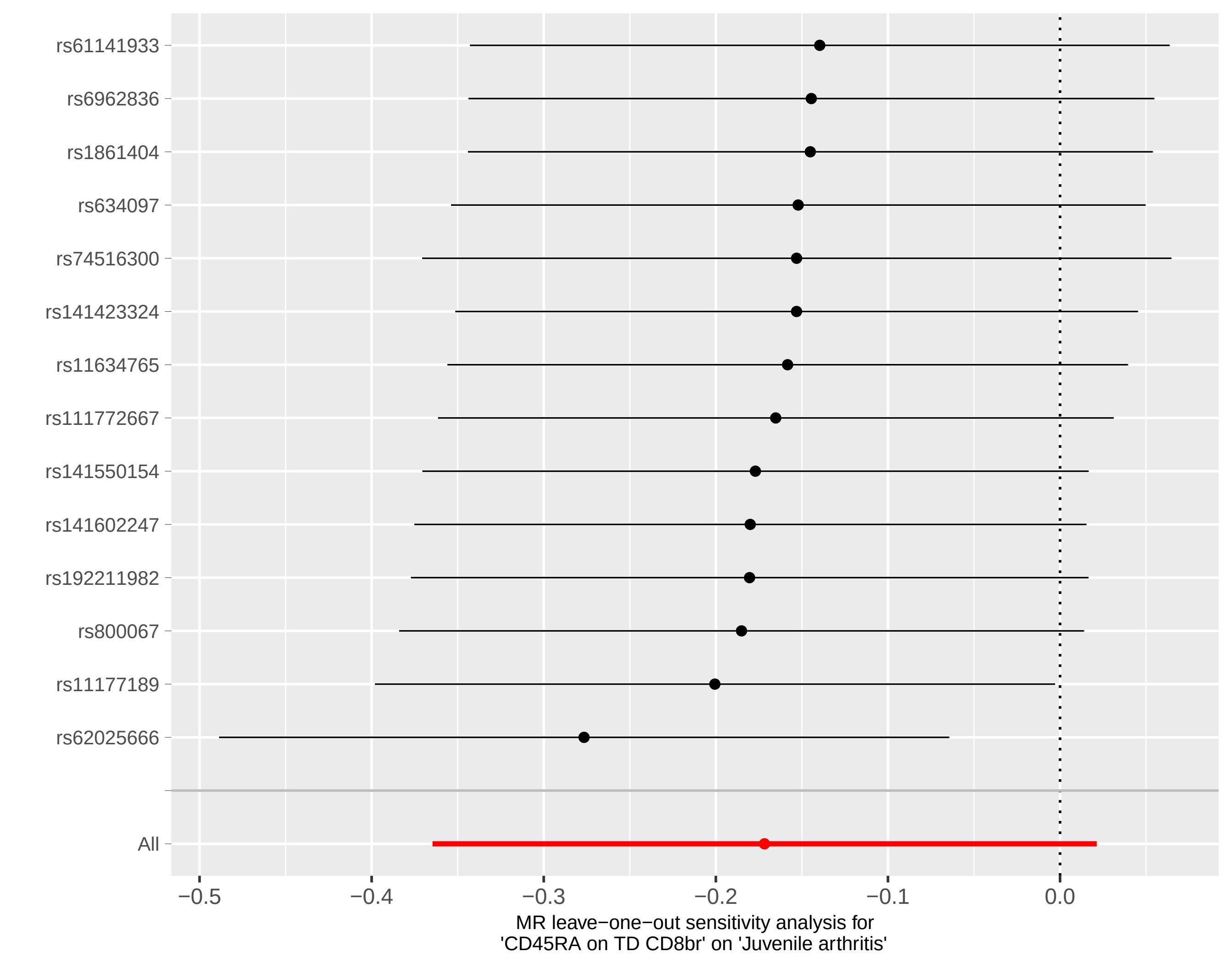
rs61141933
rs6962836
rs1861404
rs634097
rs74516300
rs141423324
rs11634765
rs111772667
rs141550154
rs141602247
rs192211982
rs800067
rs11177189
rs62025666
All
−0.5
−0.4
−0.3
−0.2
−0.1
0.0
MR leave−one−out sensitivity analysis for
'CD45RA on TD CD8br' on 'Juvenile arthritis'

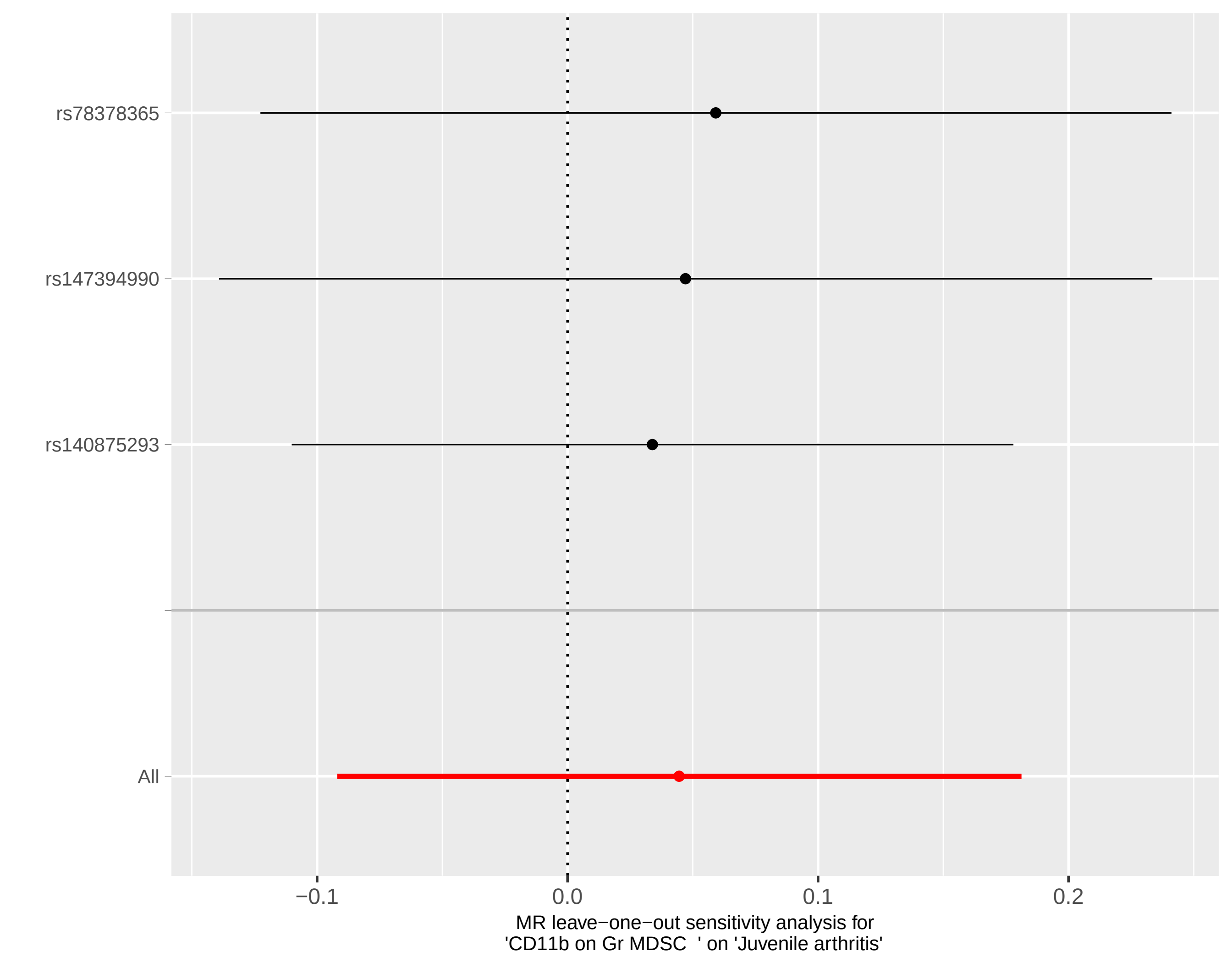
rs78378365
rs147394990
rs140875293
All
−0.1
0.0
0.1
0.2
MR leave−one−out sensitivity analysis for
'CD11b on Gr MDSC ' on 'Juvenile arthritis'

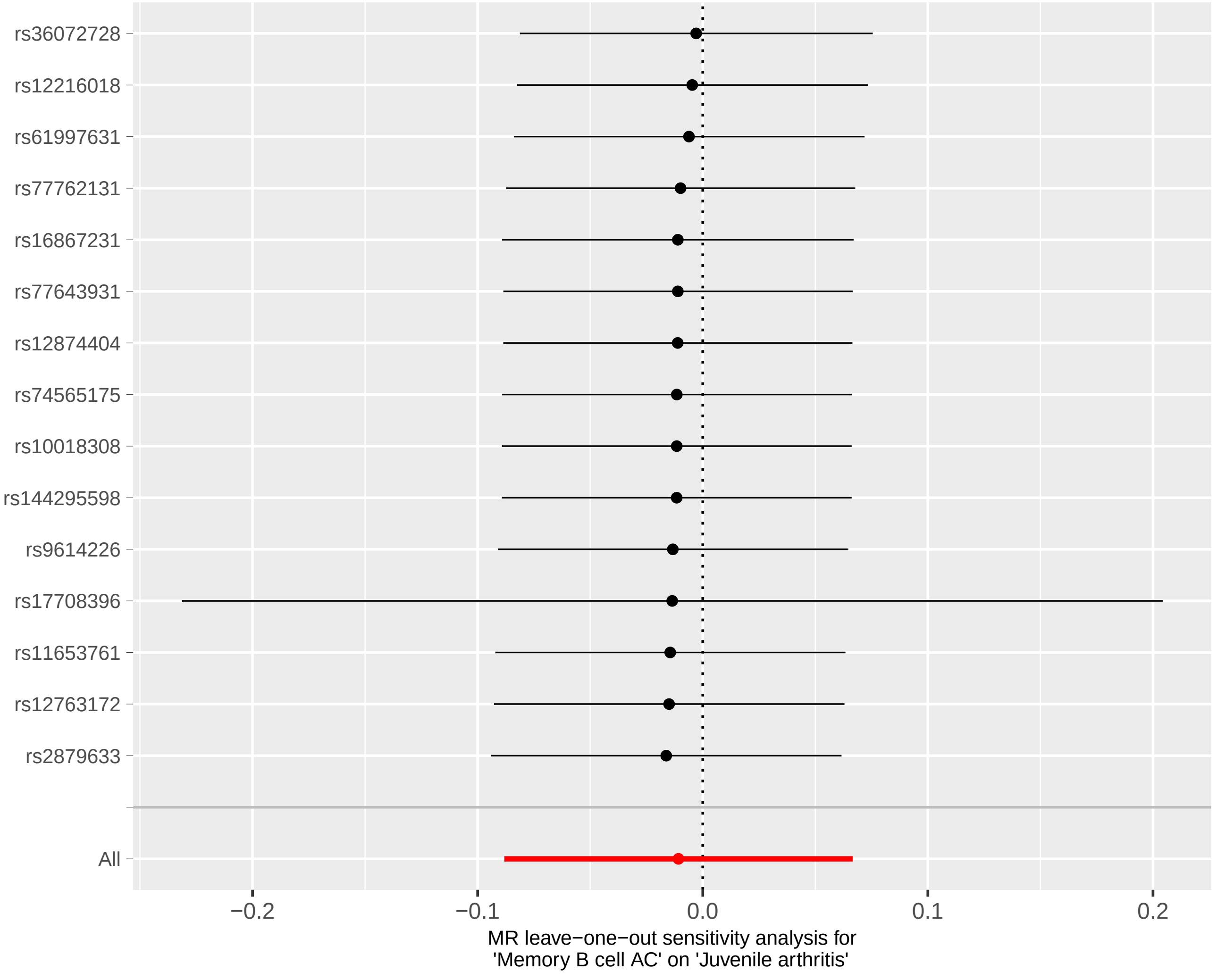
rs36072728
rs12216018
rs61997631
rs77762131
rs16867231
rs77643931
rs12874404
rs74565175
rs10018308
rs144295598
rs9614226
rs17708396
rs11653761
rs12763172
rs2879633
All
−0.2
−0.1
0.0
0.1
0.2
MR leave−one−out sensitivity analysis for
'Memory B cell AC' on 'Juvenile arthritis'

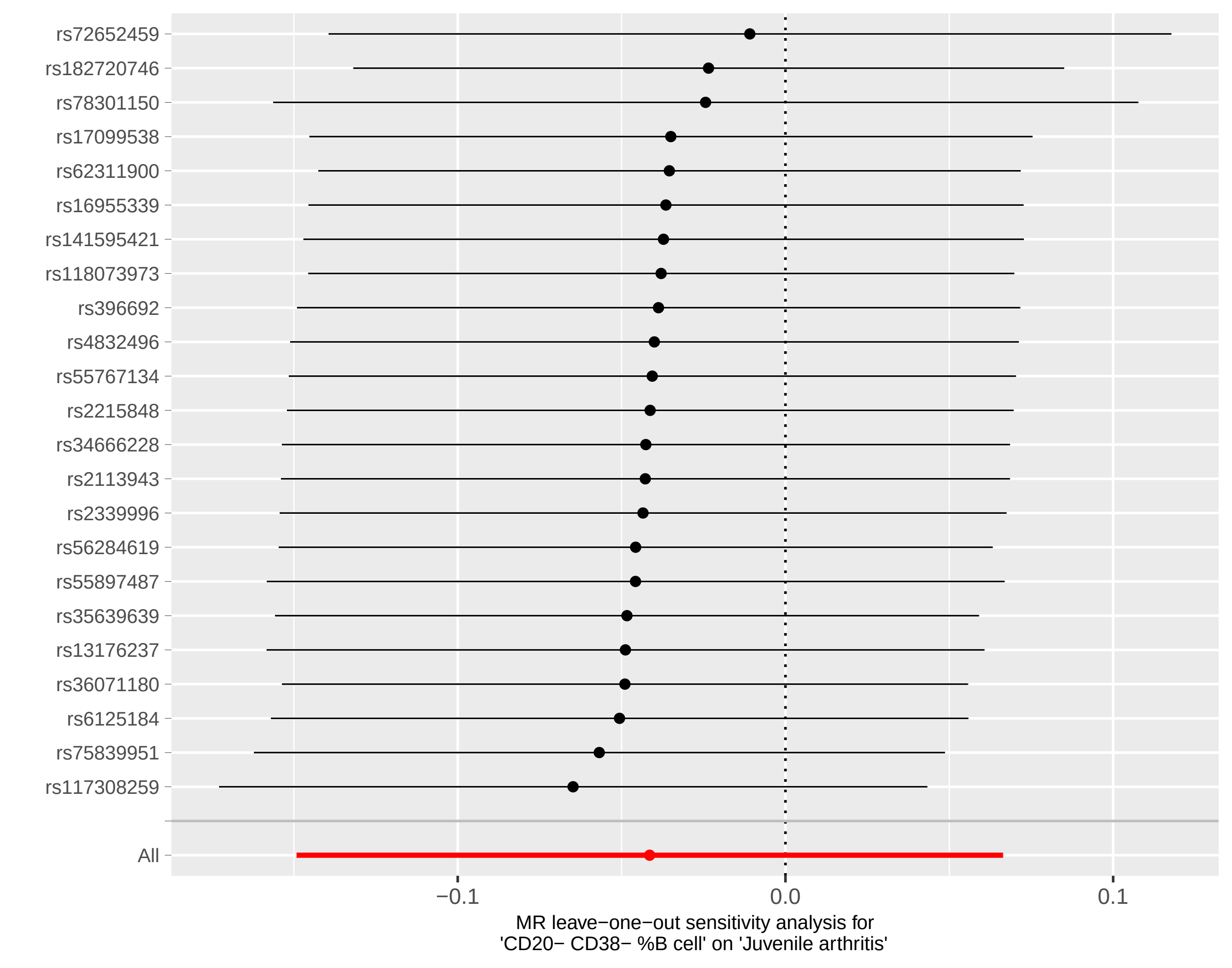
rs72652459
rs182720746
rs78301150
rs17099538
rs62311900
rs16955339
rs141595421
rs118073973
rs396692
rs4832496
rs55767134
rs2215848
rs34666228
rs2113943
rs2339996
rs56284619
rs55897487
rs35639639
rs13176237
rs36071180
rs6125184
rs75839951
rs117308259
All
−0.1
0.0
0.1
MR leave−one−out sensitivity analysis for
'CD20− CD38− %B cell' on 'Juvenile arthritis'

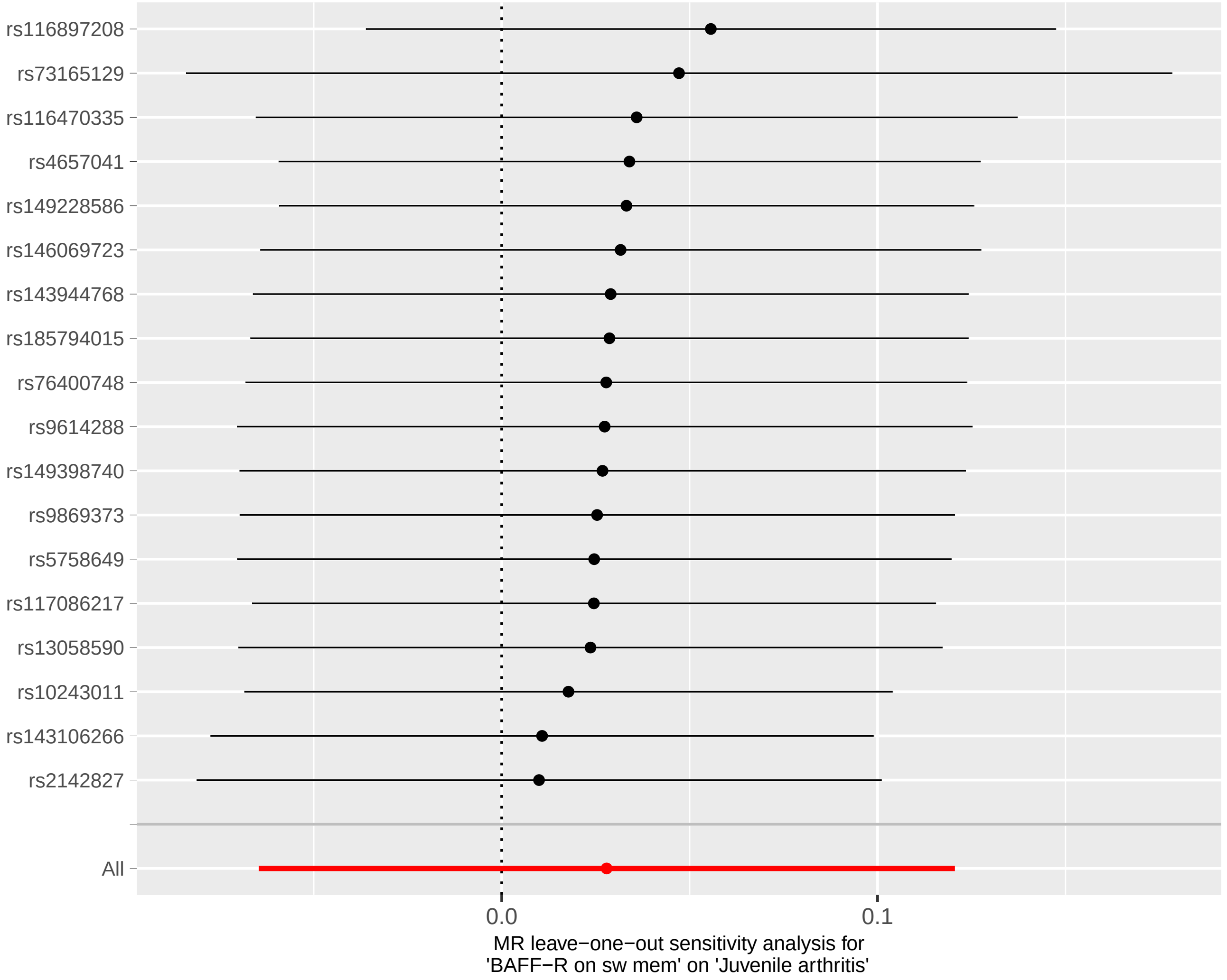
rs116897208
rs73165129
rs116470335
rs4657041
rs149228586
rs146069723
rs143944768
rs185794015
rs76400748
rs9614288
rs149398740
rs9869373
rs5758649
rs117086217
rs13058590
rs10243011
rs143106266
rs2142827
All
0.0
0.1
MR leave−one−out sensitivity analysis for
'BAFF−R on sw mem' on 'Juvenile arthritis'

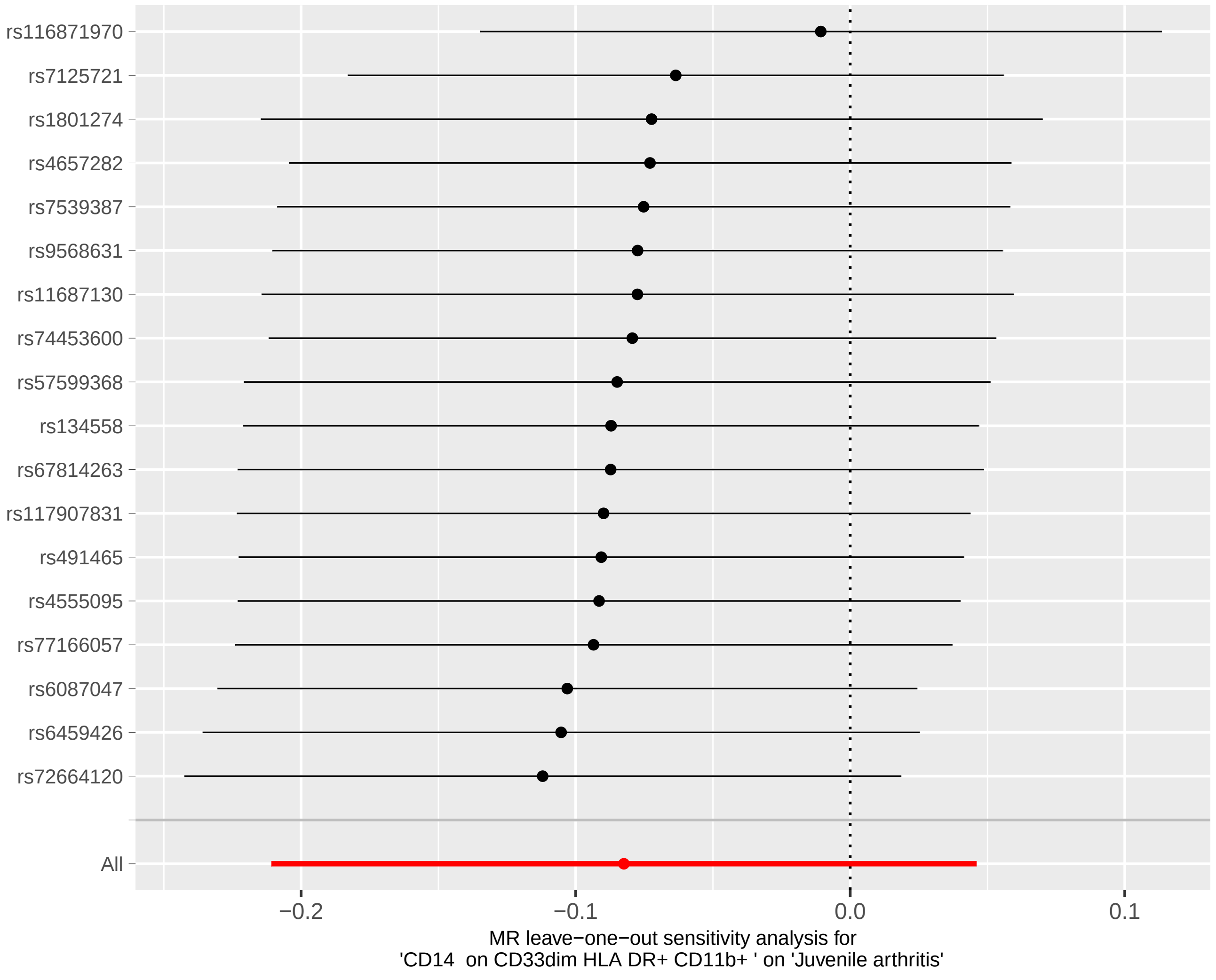

rs116871970
rs7125721
rs1801274
rs4657282
rs7539387
rs9568631
rs11687130
rs74453600
rs57599368
rs134558
rs67814263
rs117907831
rs491465
rs4555095
rs77166057
rs6087047
rs6459426
rs72664120
All
−0.2
−0.1
0.0
0.1
MR leave−one−out sensitivity analysis for
'CD14 on CD33dim HLA DR+ CD11b+ ' on 'Juvenile arthritis'

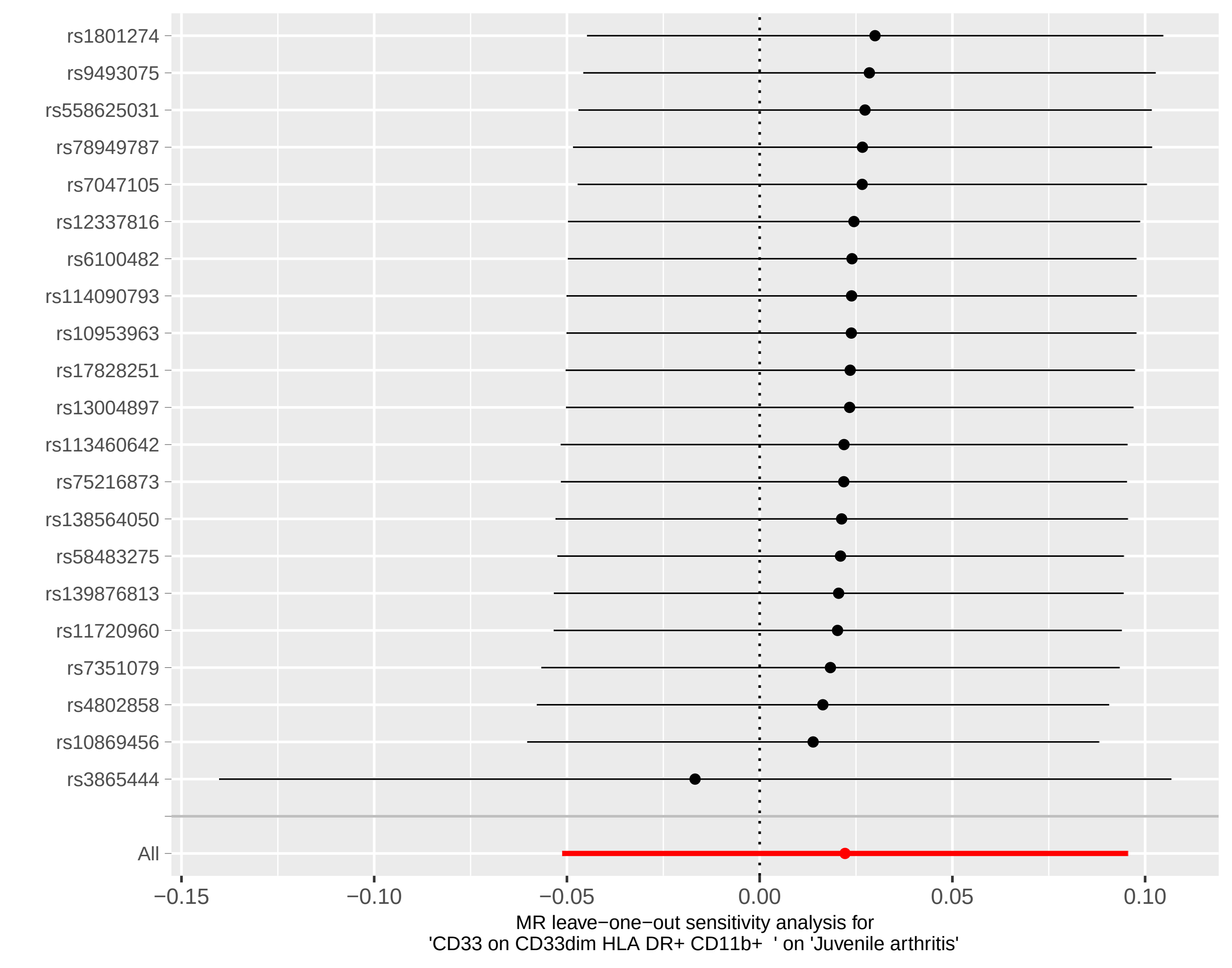
rs1801274
rs9493075
rs558625031
rs78949787
rs7047105
rs12337816
rs6100482
rs114090793
rs10953963
rs17828251
rs13004897
rs113460642
rs75216873
rs138564050
rs58483275
rs139876813
rs11720960
rs7351079
rs4802858
rs10869456
rs3865444
All
−0.15
−0.10
−0.05
0.00
0.05
0.10
MR leave−one−out sensitivity analysis for
'CD33 on CD33dim HLA DR+ CD11b+ ' on 'Juvenile arthritis'

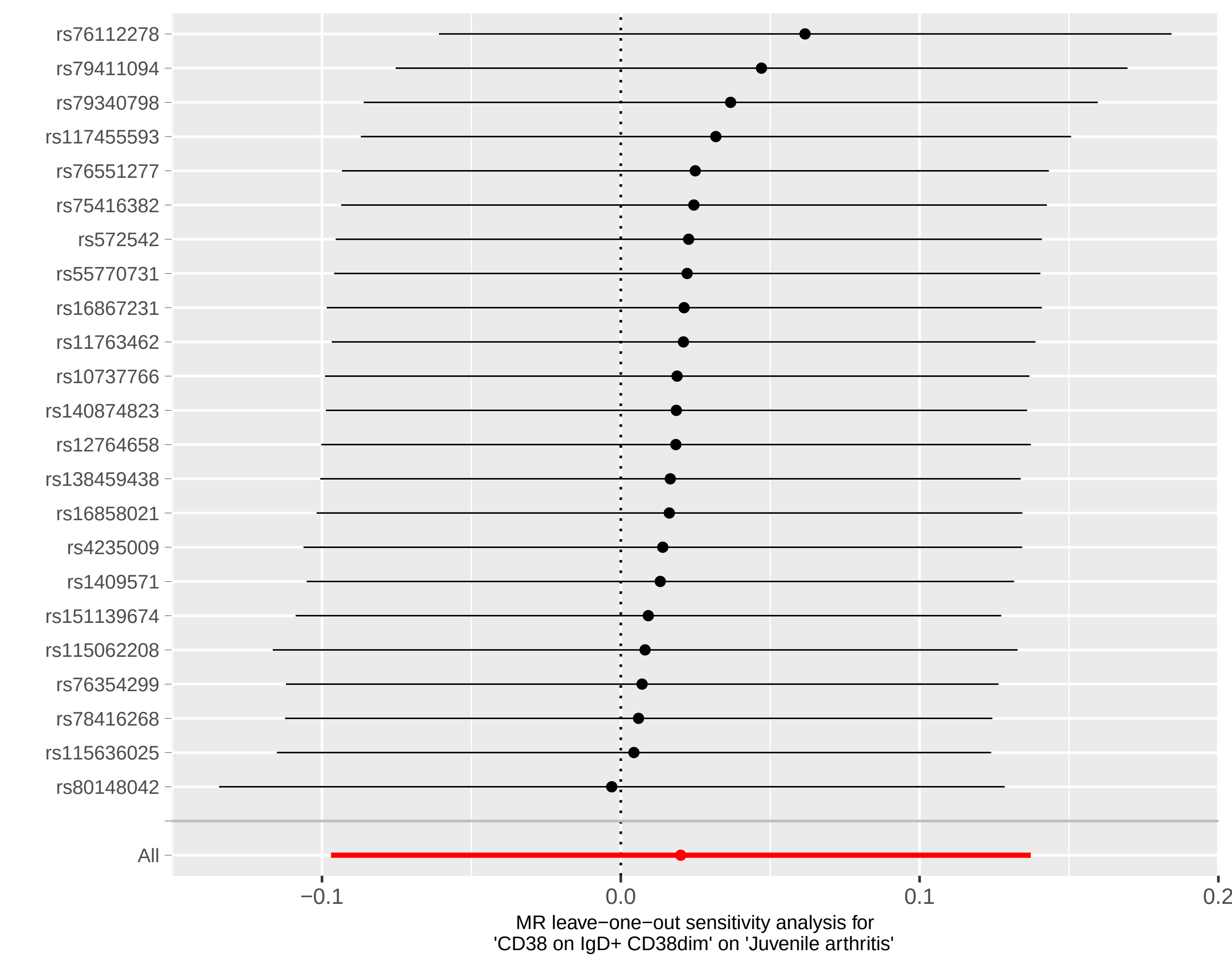
rs76112278
rs79411094
rs79340798
rs117455593
rs76551277
rs75416382
rs572542
rs55770731
rs16867231
rs11763462
rs10737766
rs140874823
rs12764658
rs138459438
rs16858021
rs4235009
rs1409571
rs151139674
rs115062208
rs76354299
rs78416268
rs115636025
rs80148042
All
−0.1
0.0
0.1
0.2
MR leave−one−out sensitivity analysis for
'CD38 on IgD+ CD38dim' on 'Juvenile arthritis'

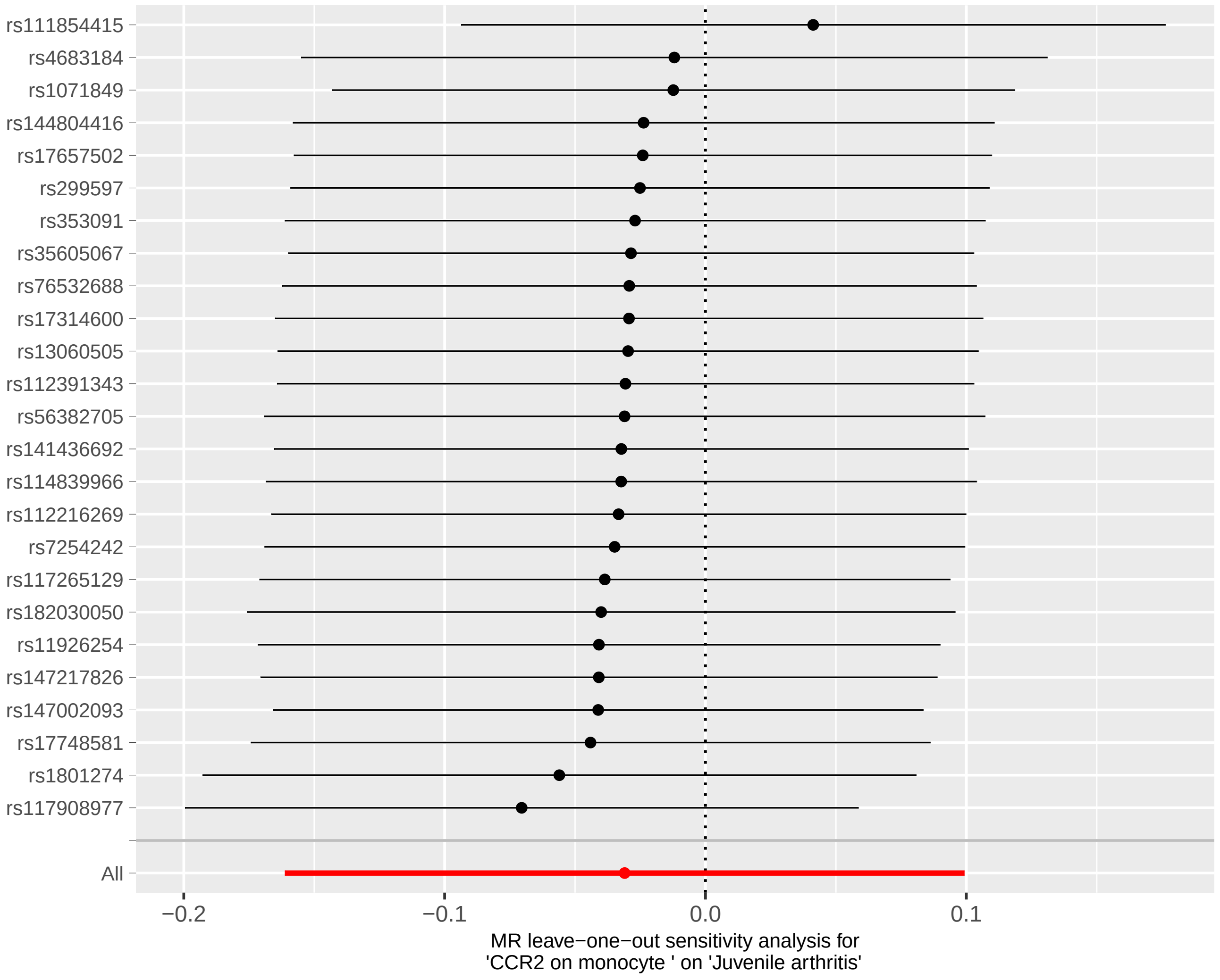

rs111854415
rs4683184
rs1071849
rs144804416
rs17657502
rs299597
rs353091
rs35605067
rs76532688
rs17314600
rs13060505
rs112391343
rs56382705
rs141436692
rs114839966
rs112216269
rs7254242
rs117265129
rs182030050
rs11926254
rs147217826
rs147002093
rs17748581
rs1801274
rs117908977
All
−0.2
−0.1
0.0
0.1
MR leave−one−out sensitivity analysis for
'CCR2 on monocyte ' on 'Juvenile arthritis'

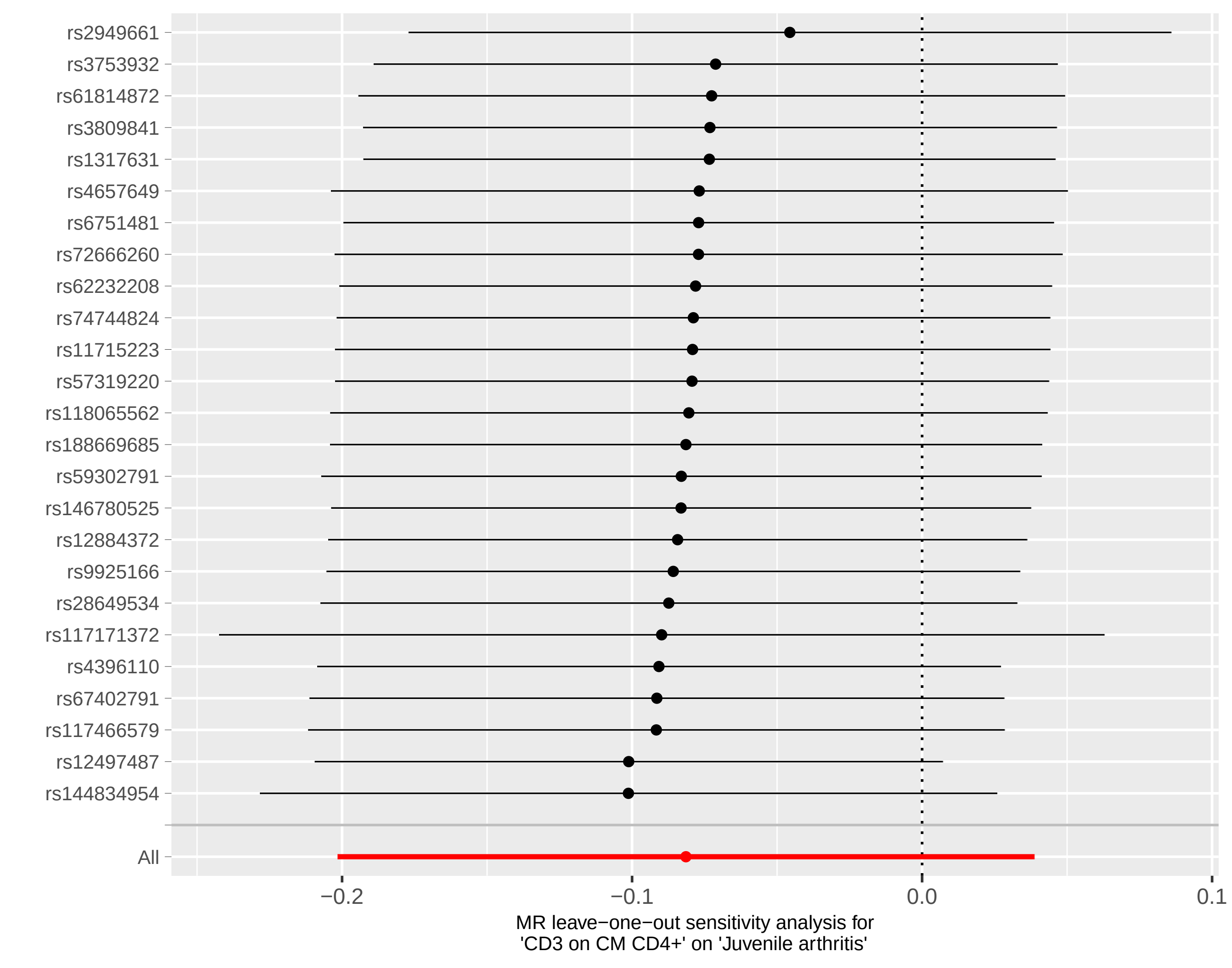
rs2949661
rs3753932
rs61814872
rs3809841
rs1317631
rs4657649
rs6751481
rs72666260
rs62232208
rs74744824
rs11715223
rs57319220
rs118065562
rs188669685
rs59302791
rs146780525
rs12884372
rs9925166
rs28649534
rs117171372
rs4396110
rs67402791
rs117466579
rs12497487
rs144834954
All
−0.2
−0.1
0.0
0.1
MR leave−one−out sensitivity analysis for
'CD3 on CM CD4+' on 'Juvenile arthritis'

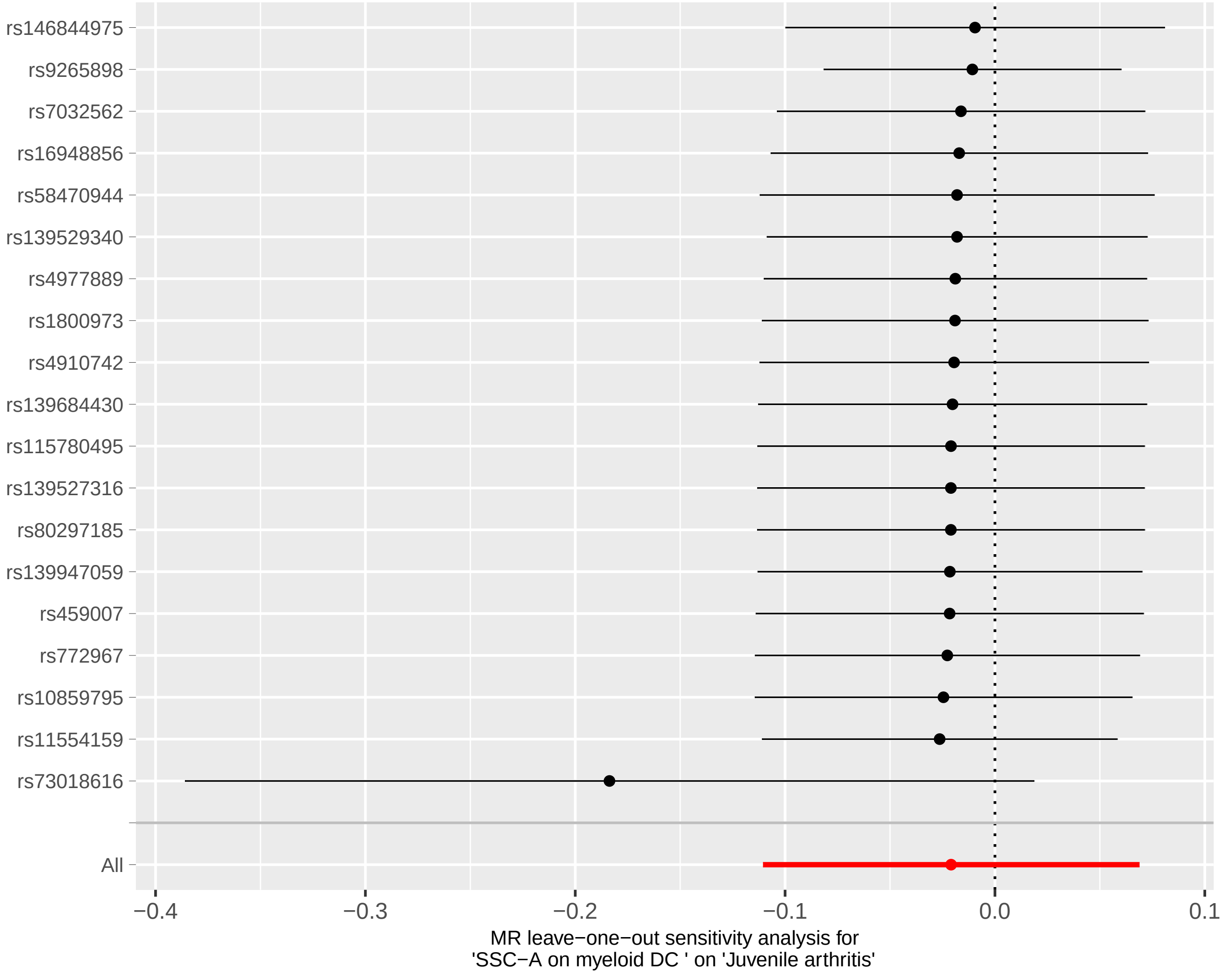
rs146844975
rs9265898
rs7032562
rs16948856
rs58470944
rs139529340
rs4977889
rs1800973
rs4910742
rs139684430
rs115780495
rs139527316
rs80297185
rs139947059
rs459007
rs772967
rs10859795
rs11554159
rs73018616
All
−0.4
−0.3
−0.2
−0.1
0.0
0.1
MR leave−one−out sensitivity analysis for
'SSC−A on myeloid DC ' on 'Juvenile arthritis'

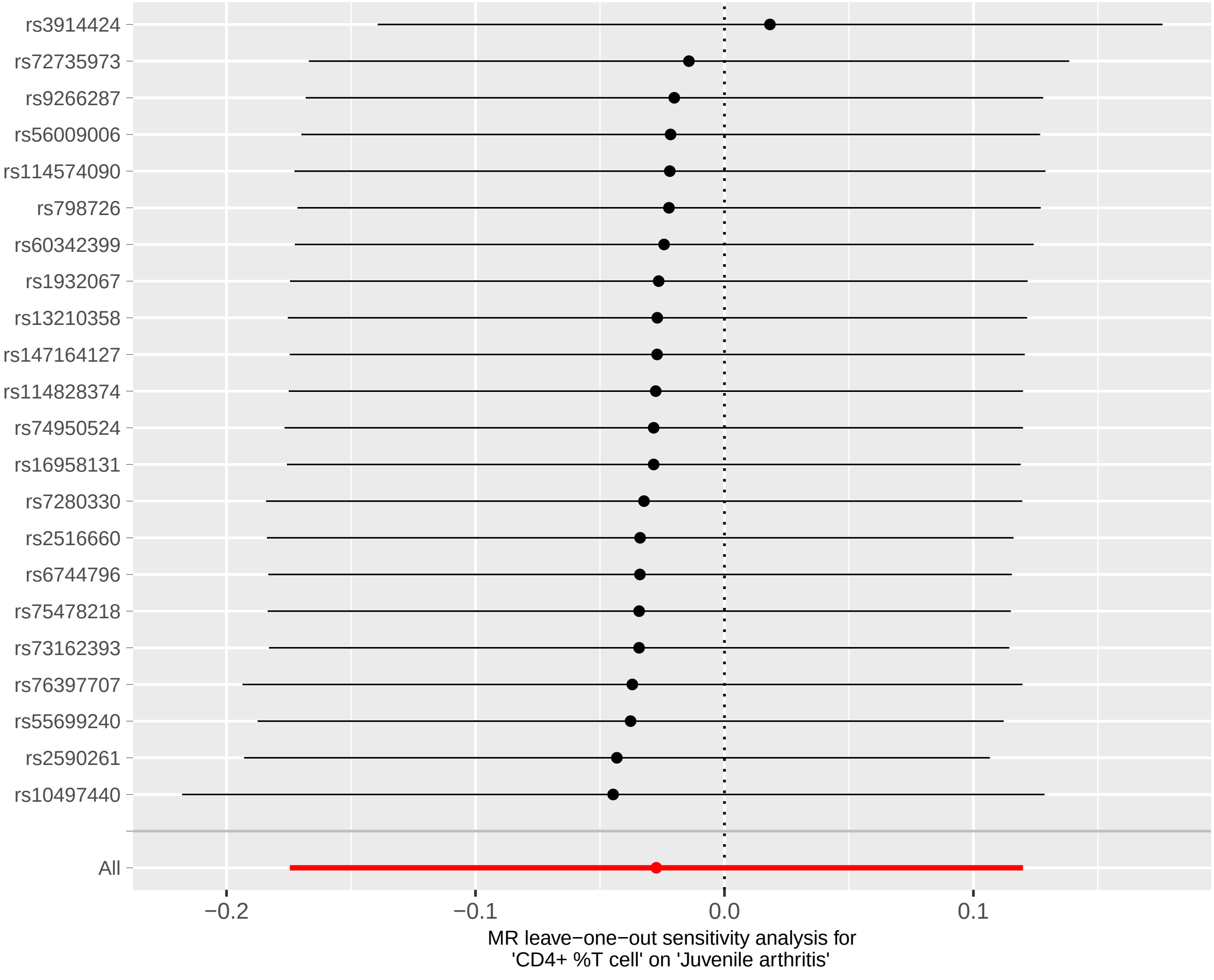
rs3914424
rs72735973
rs9266287
rs56009006
rs114574090
rs798726
rs60342399
rs1932067
rs13210358
rs147164127
rs114828374
rs74950524
rs16958131
rs7280330
rs2516660
rs6744796
rs75478218
rs73162393
rs76397707
rs55699240
rs2590261
rs10497440
All
−0.2
−0.1
0.0
0.1
MR leave−one−out sensitivity analysis for
'CD4+ %T cell' on 'Juvenile arthritis'

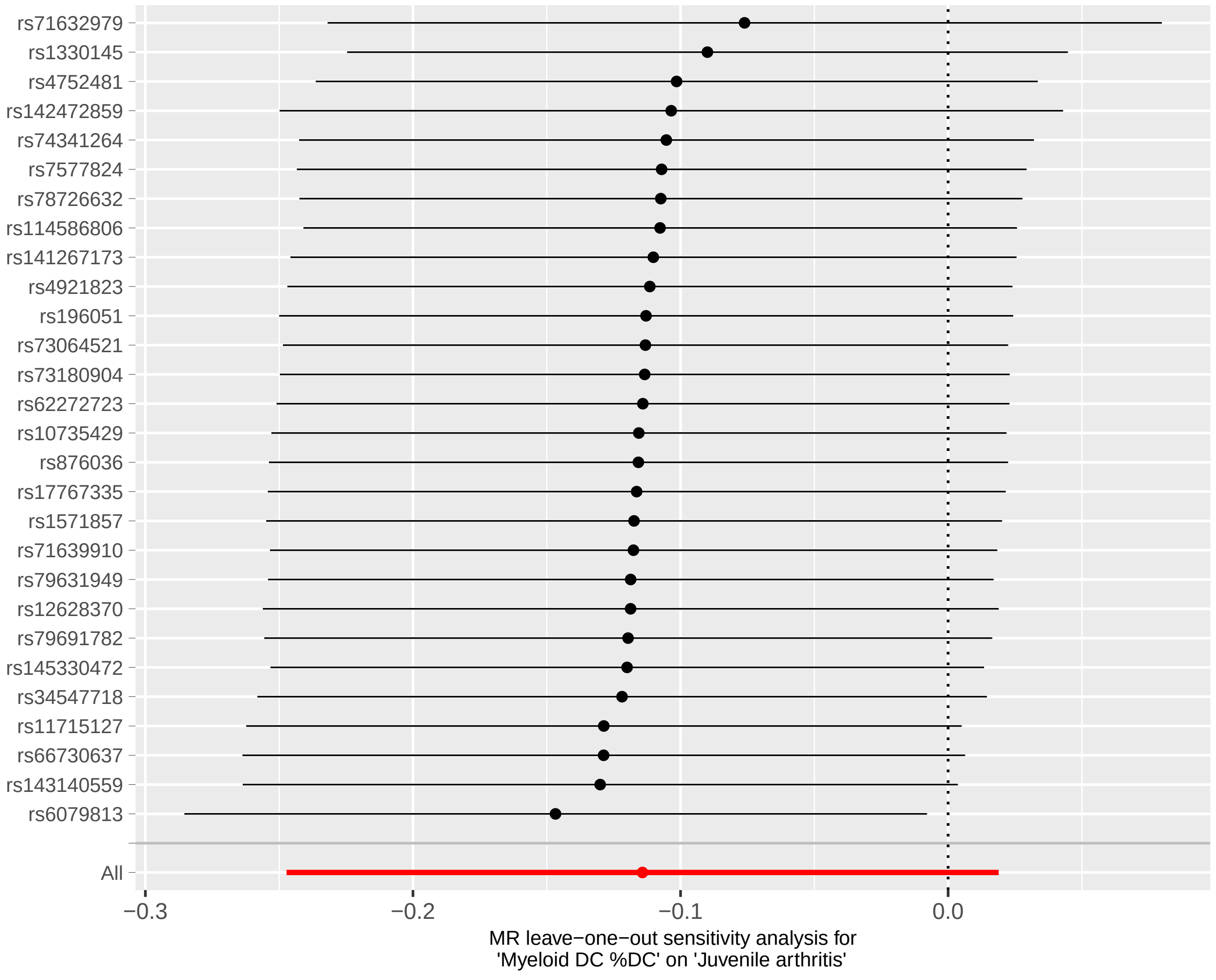
rs71632979
rs1330145
rs4752481
rs142472859
rs74341264
rs7577824
rs78726632
rs114586806
rs141267173
rs4921823
rs196051
rs73064521
rs73180904
rs62272723
rs10735429
rs876036
rs17767335
rs1571857
rs71639910
rs79631949
rs12628370
rs79691782
rs145330472
rs34547718
rs11715127
rs66730637
rs143140559
rs6079813
All
−0.3
−0.2
−0.1
0.0
MR leave−one−out sensitivity analysis for
'Myeloid DC %DC' on 'Juvenile arthritis'

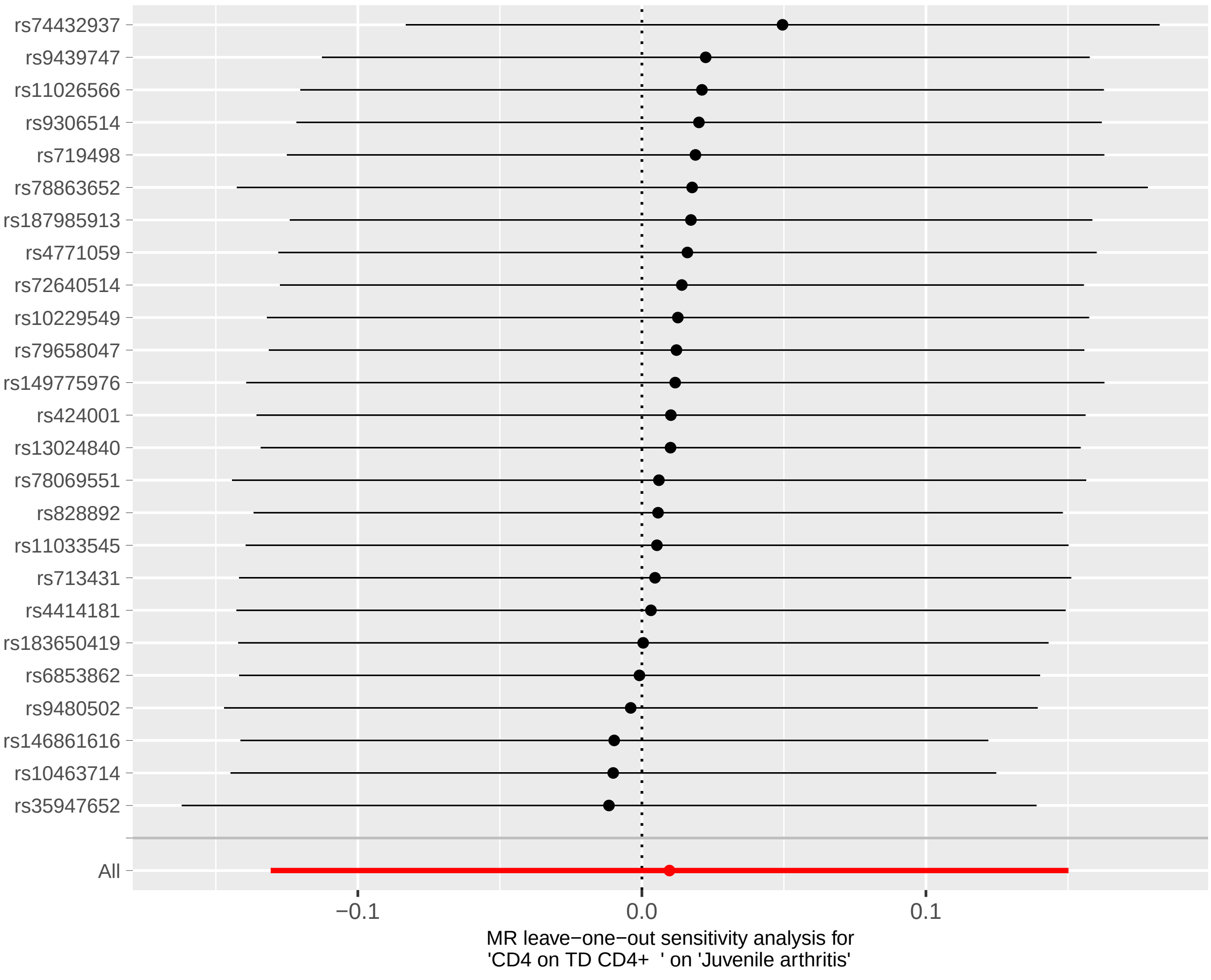
rs74432937
rs9439747
rs11026566
rs9306514
rs719498
rs78863652
rs187985913
rs4771059
rs72640514
rs10229549
rs79658047
rs149775976
rs424001
rs13024840
rs78069551
rs828892
rs11033545
rs713431
rs4414181
rs183650419
rs6853862
rs9480502
rs146861616
rs10463714
rs35947652
All
−0.1
0.0
0.1
MR leave−one−out sensitivity analysis for
'CD4 on TD CD4+ ' on 'Juvenile arthritis'

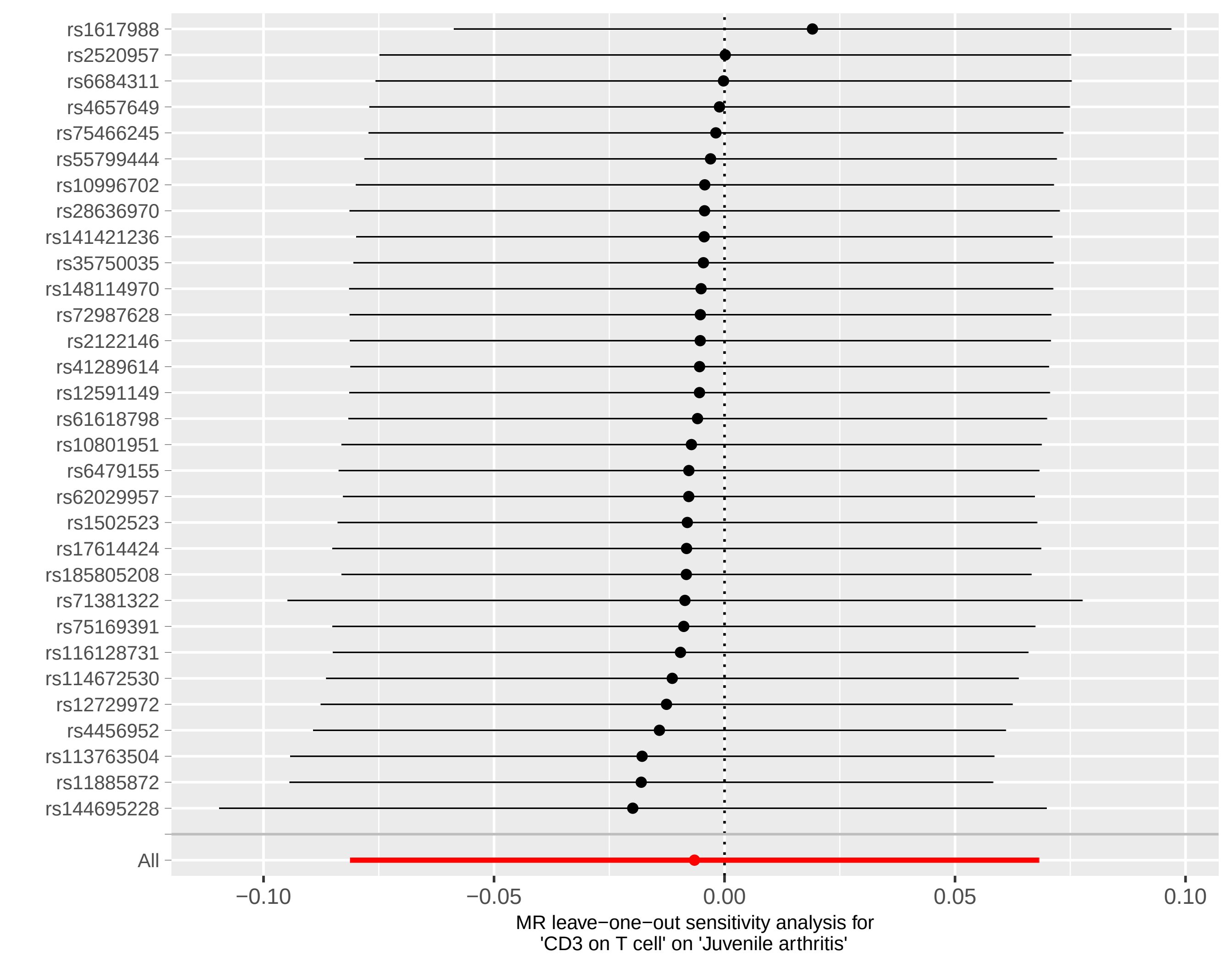
rs1617988
rs2520957
rs6684311
rs4657649
rs75466245
rs55799444
rs10996702
rs28636970
rs141421236
rs35750035
rs148114970
rs72987628
rs2122146
rs41289614
rs12591149
rs61618798
rs10801951
rs6479155
rs62029957
rs1502523
rs17614424
rs185805208
rs71381322
rs75169391
rs116128731
rs114672530
rs12729972
rs4456952
rs113763504
rs11885872
rs144695228
All
−0.10
−0.05
0.00
0.05
0.10
MR leave−one−out sensitivity analysis for
'CD3 on T cell' on 'Juvenile arthritis'

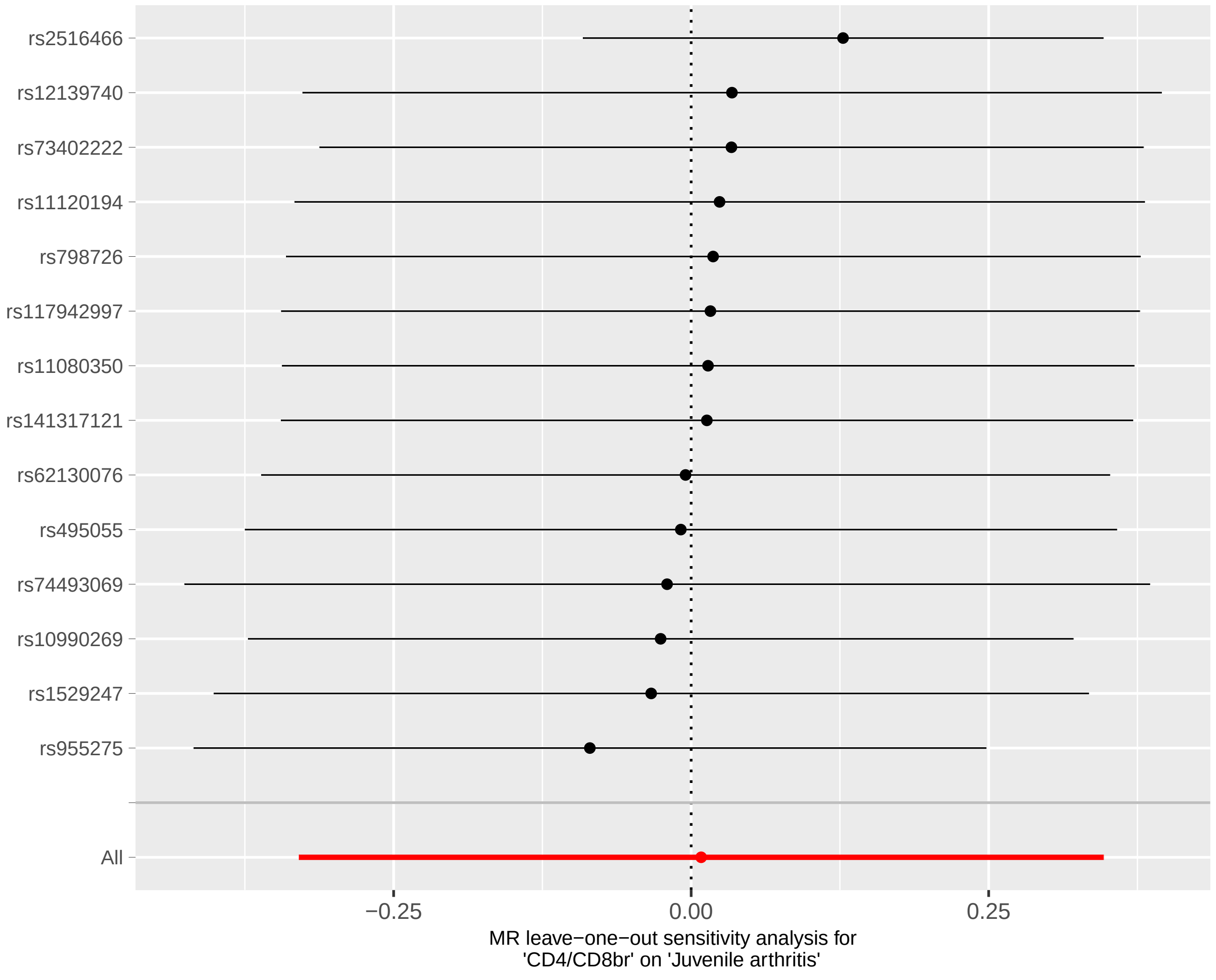
rs2516466
rs12139740
rs73402222
rs11120194
rs798726
rs117942997
rs11080350
rs141317121
rs62130076
rs495055
rs74493069
rs10990269
rs1529247
rs955275
All
−0.25
0.00
0.25
MR leave−one−out sensitivity analysis for
'CD4/CD8br' on 'Juvenile arthritis'

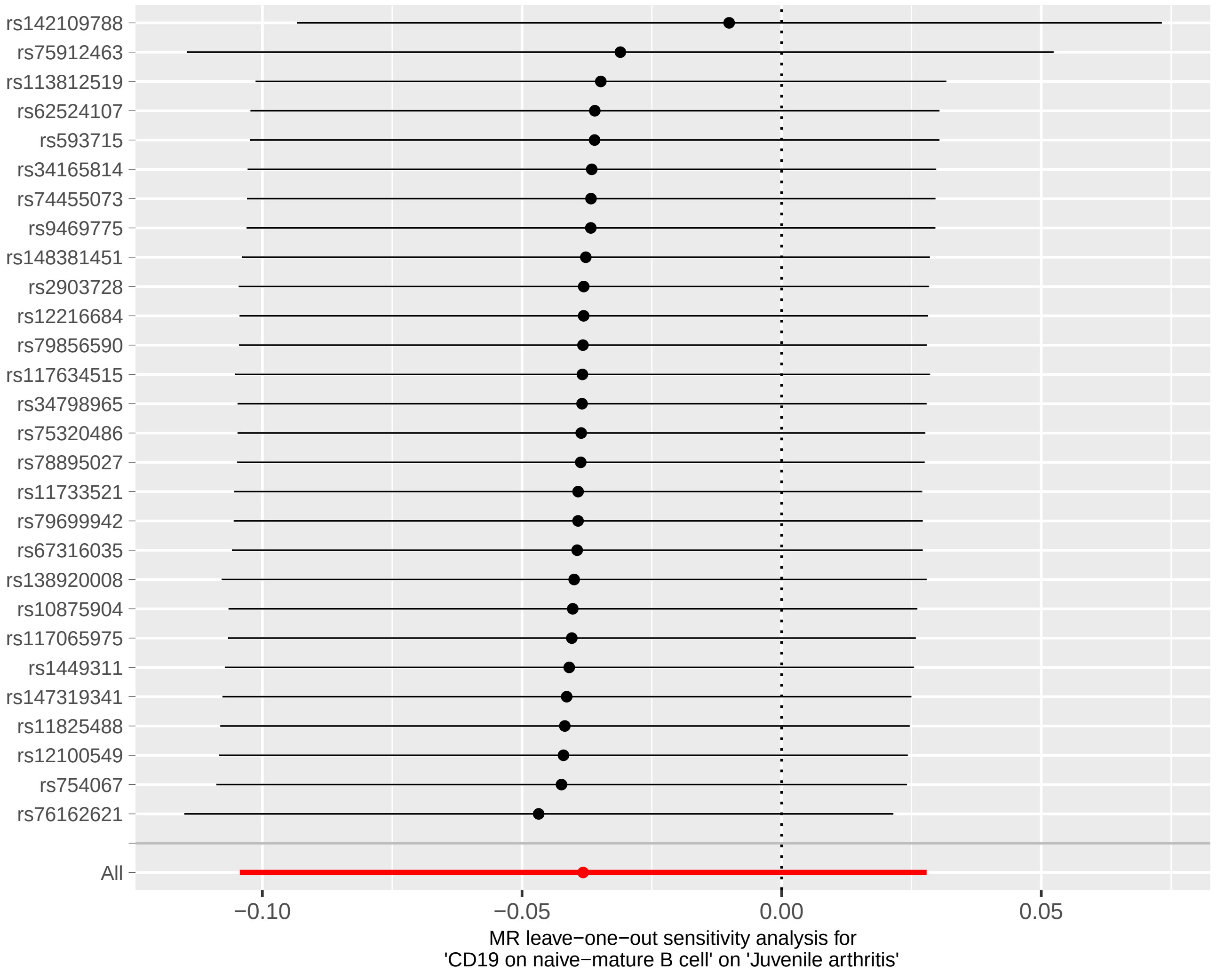
rs142109788
rs75912463
rs113812519
rs62524107
rs593715
rs34165814
rs74455073
rs9469775
rs148381451
rs2903728
rs12216684
rs79856590
rs117634515
rs34798965
rs75320486
rs78895027
rs11733521
rs79699942
rs67316035
rs138920008
rs10875904
rs117065975
rs1449311
rs147319341
rs11825488
rs12100549
rs754067
rs76162621
All
−0.10
−0.05
0.00
0.05
MR leave−one−out sensitivity analysis for
'CD19 on naive−mature B cell' on 'Juvenile arthritis'

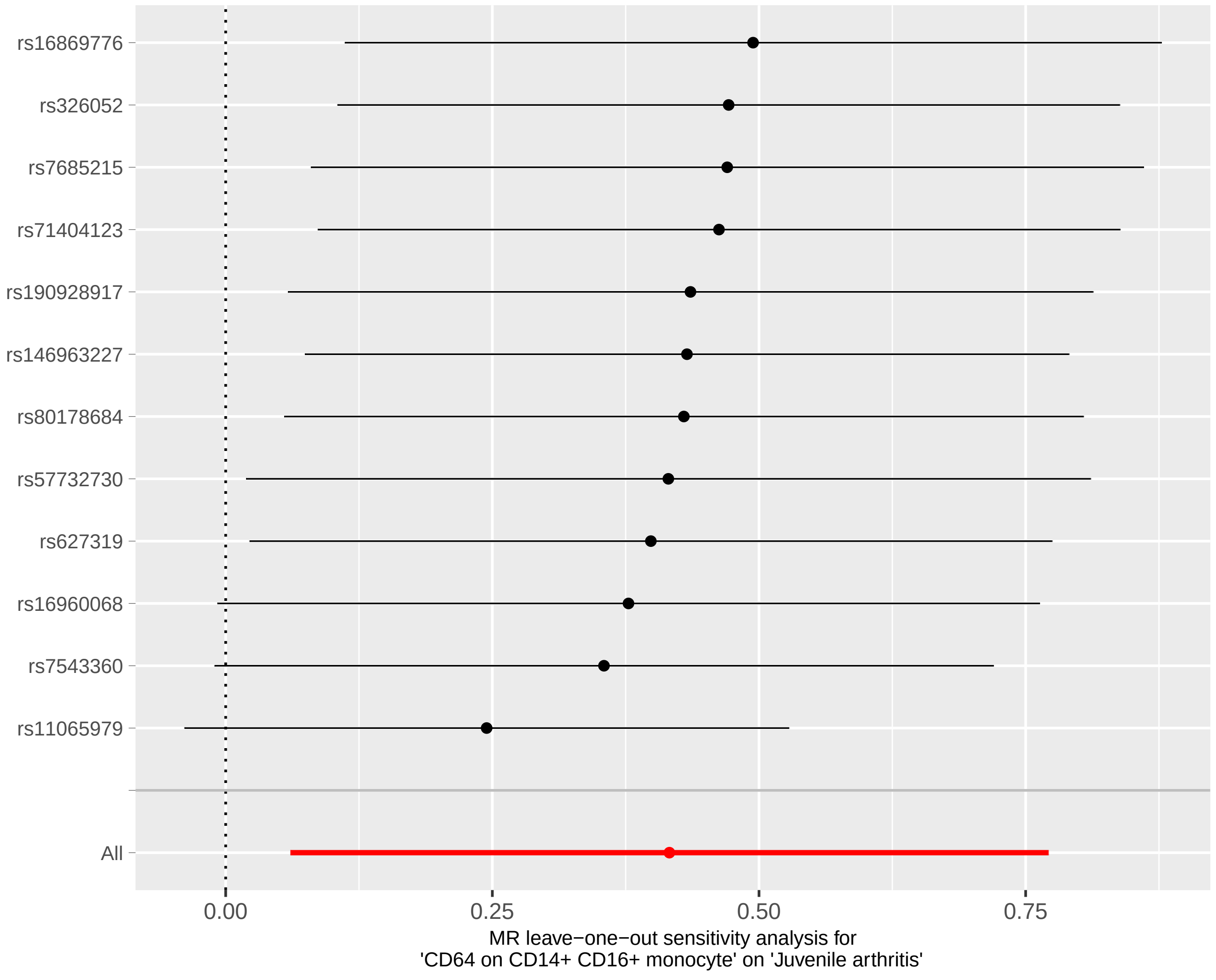
rs16869776
rs326052
rs7685215
rs71404123
rs190928917
rs146963227
rs80178684
rs57732730
rs627319
rs16960068
rs7543360
rs11065979
All
0.00
0.25
0.50
0.75
MR leave−one−out sensitivity analysis for
'CD64 on CD14+ CD16+ monocyte' on 'Juvenile arthritis'

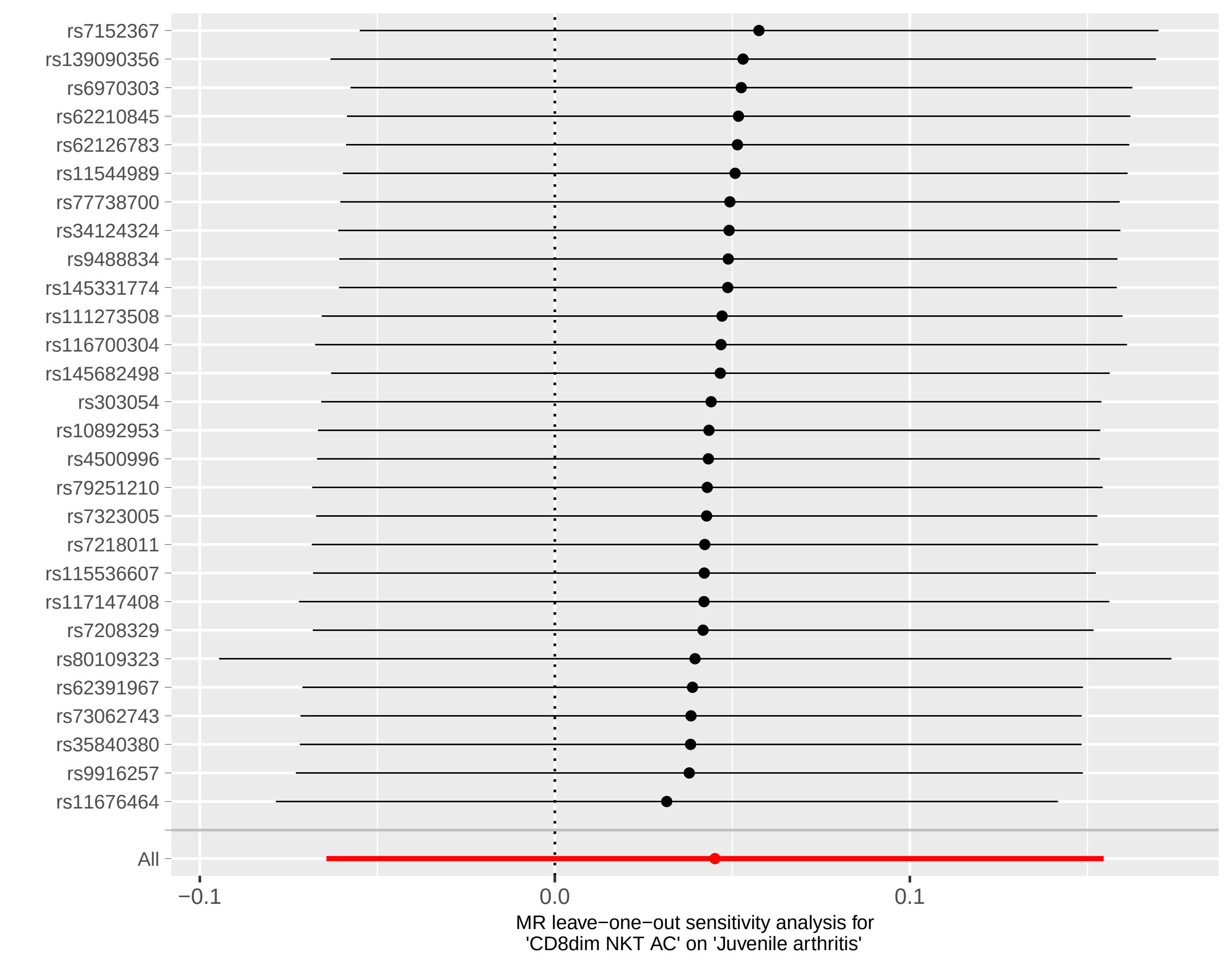

rs7152367
rs139090356
rs6970303
rs62210845
rs62126783
rs11544989
rs77738700
rs34124324
rs9488834
rs145331774
rs111273508
rs116700304
rs145682498
rs303054
rs10892953
rs4500996
rs79251210
rs7323005
rs7218011
rs115536607
rs117147408
rs7208329
rs80109323
rs62391967
rs73062743
rs35840380
rs9916257
rs11676464
All
−0.1
0.0
0.1
MR leave−one−out sensitivity analysis for
'CD8dim NKT AC' on 'Juvenile arthritis'

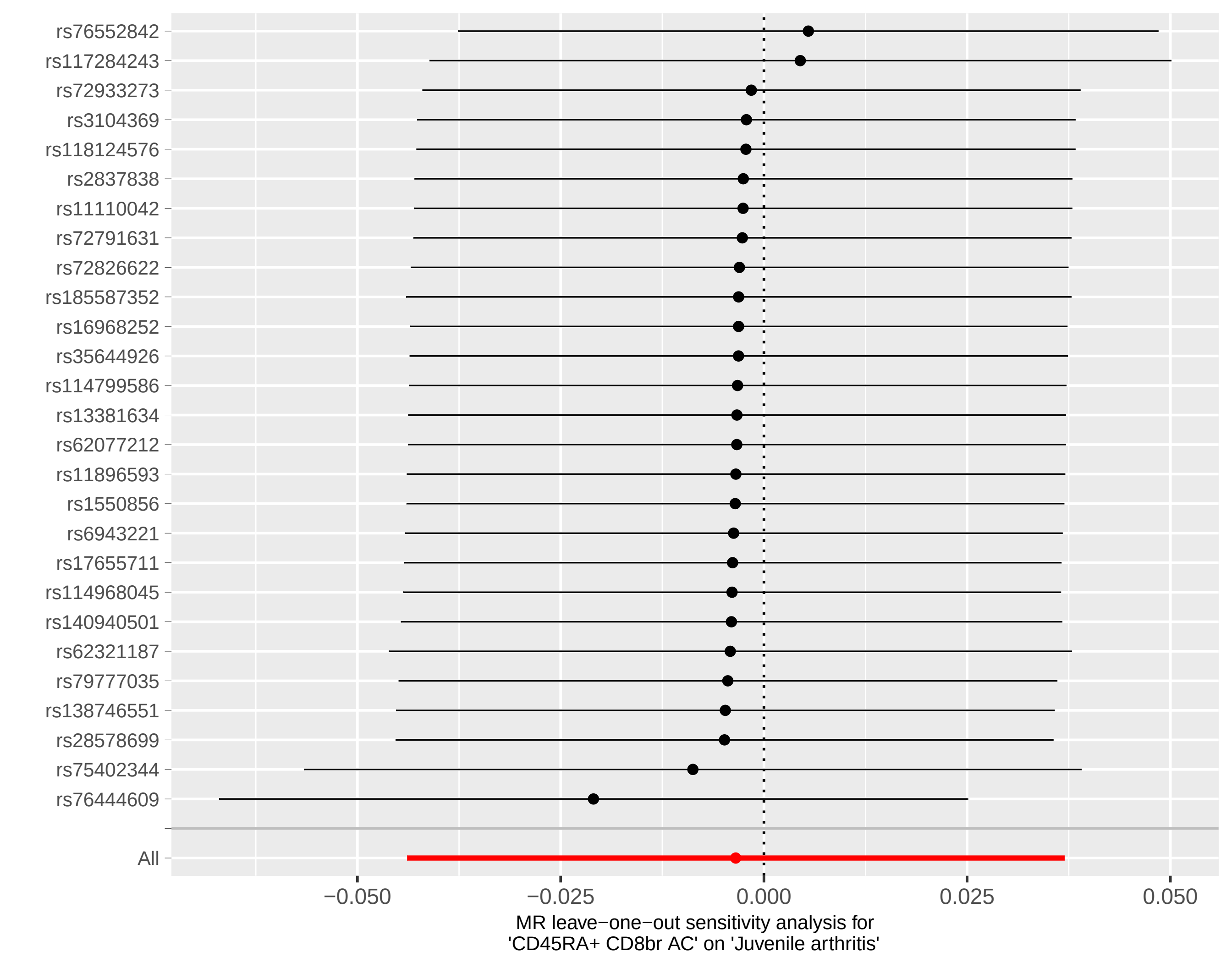
rs76552842
rs117284243
rs72933273
rs3104369
rs118124576
rs2837838
rs11110042
rs72791631
rs72826622
rs185587352
rs16968252
rs35644926
rs114799586
rs13381634
rs62077212
rs11896593
rs1550856
rs6943221
rs17655711
rs114968045
rs140940501
rs62321187
rs79777035
rs138746551
rs28578699
rs75402344
rs76444609
All
−0.050
−0.025
0.000
0.025
0.050
MR leave−one−out sensitivity analysis for
'CD45RA+ CD8br AC' on 'Juvenile arthritis'

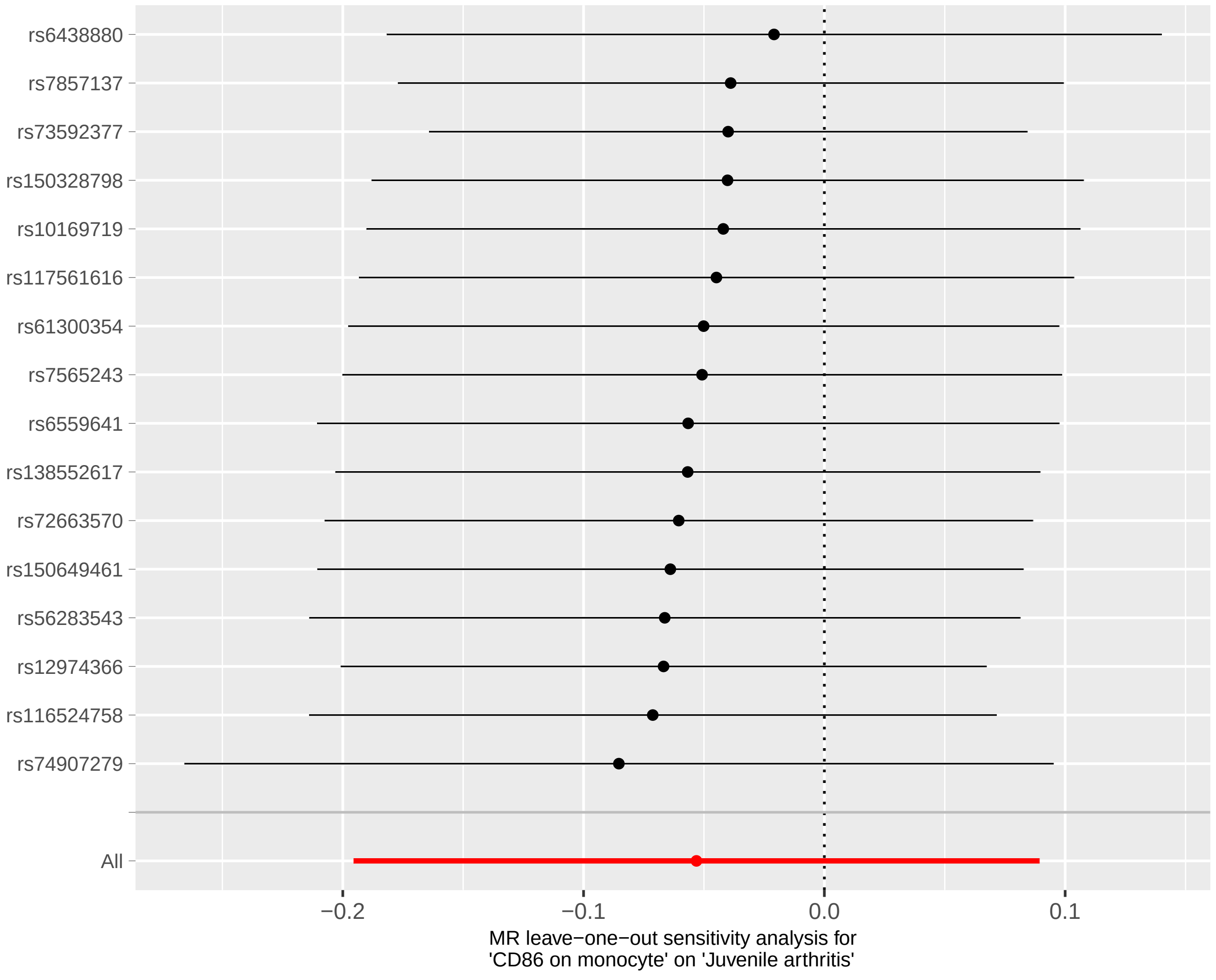

rs6438880
rs7857137
rs73592377
rs150328798
rs10169719
rs117561616
rs61300354
rs7565243
rs6559641
rs138552617
rs72663570
rs150649461
rs56283543
rs12974366
rs116524758
rs74907279
All
−0.2
−0.1
0.0
0.1
MR leave−one−out sensitivity analysis for
'CD86 on monocyte' on 'Juvenile arthritis'

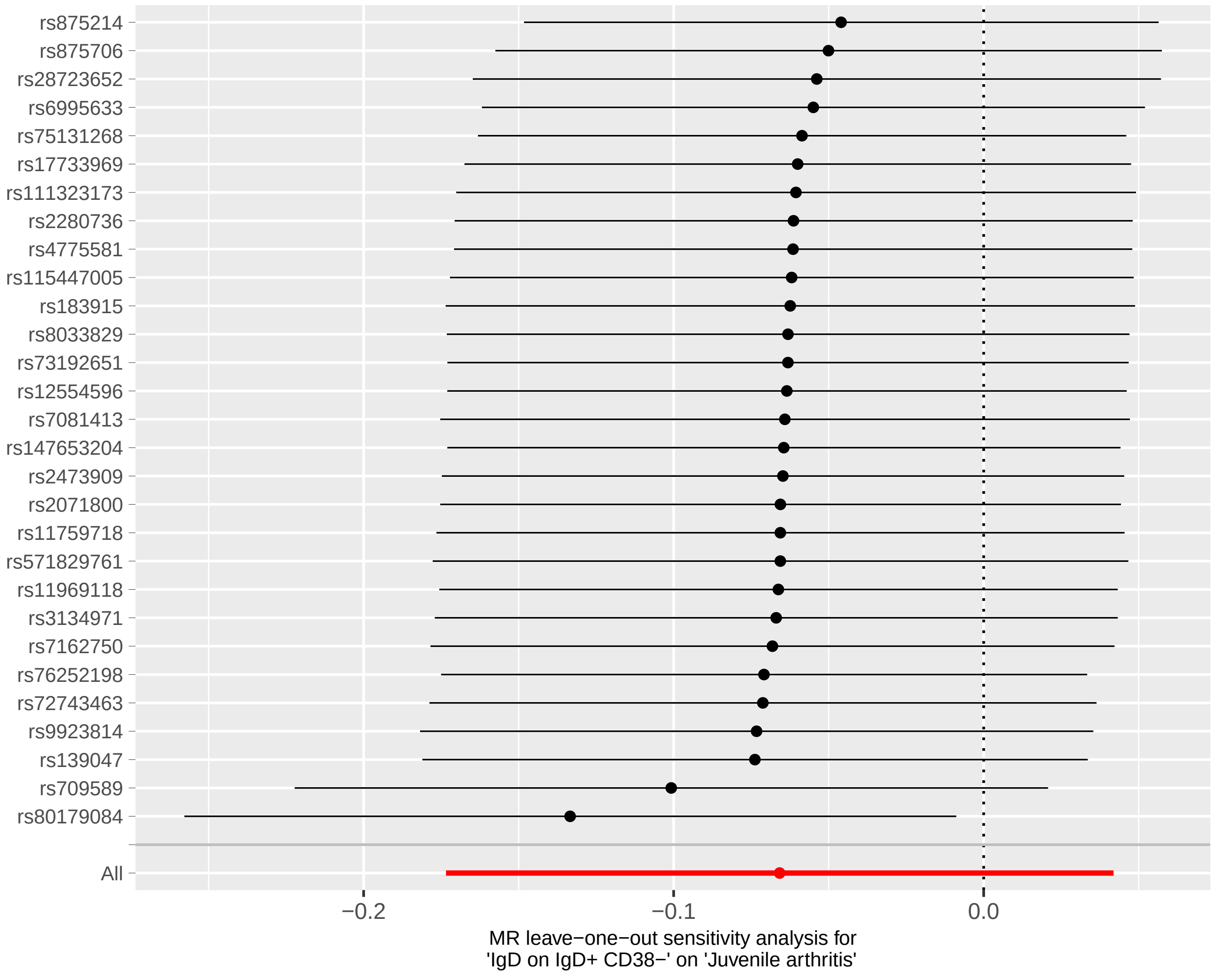
rs875214
rs875706
rs28723652
rs6995633
rs75131268
rs17733969
rs111323173
rs2280736
rs4775581
rs115447005
rs183915
rs8033829
rs73192651
rs12554596
rs7081413
rs147653204
rs2473909
rs2071800
rs11759718
rs571829761
rs11969118
rs3134971
rs7162750
rs76252198
rs72743463
rs9923814
rs139047
rs709589
rs80179084
All
−0.2
−0.1
0.0
MR leave−one−out sensitivity analysis for
'IgD on IgD+ CD38−' on 'Juvenile arthritis'

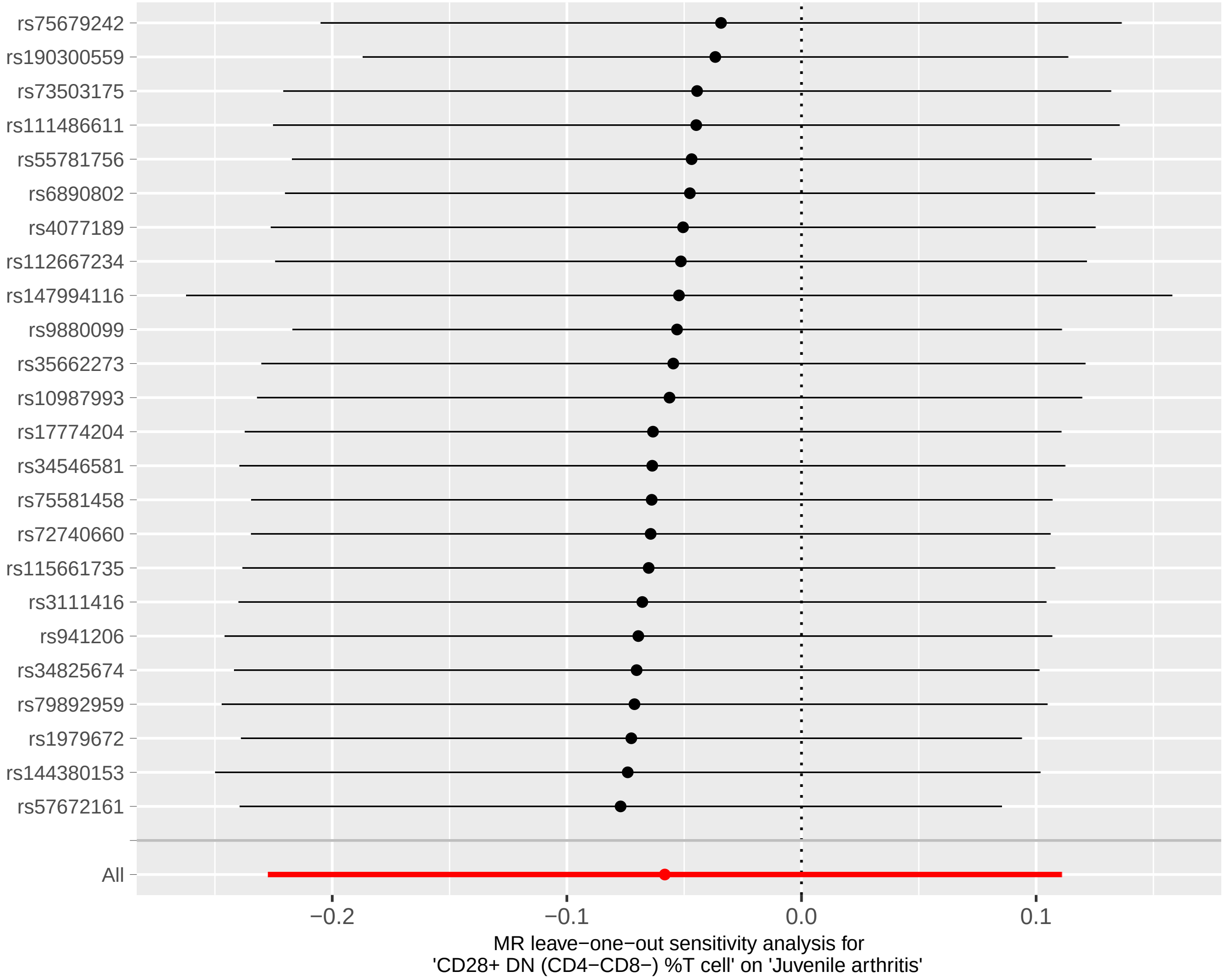
rs75679242
rs190300559
rs73503175
rs111486611
rs55781756
rs6890802
rs4077189
rs112667234
rs147994116
rs9880099
rs35662273
rs10987993
rs17774204
rs34546581
rs75581458
rs72740660
rs115661735
rs3111416
rs941206
rs34825674
rs79892959
rs1979672
rs144380153
rs57672161
All
−0.2
−0.1
0.0
0.1
MR leave−one−out sensitivity analysis for
'CD28+ DN (CD4−CD8−) %T cell' on 'Juvenile arthritis'

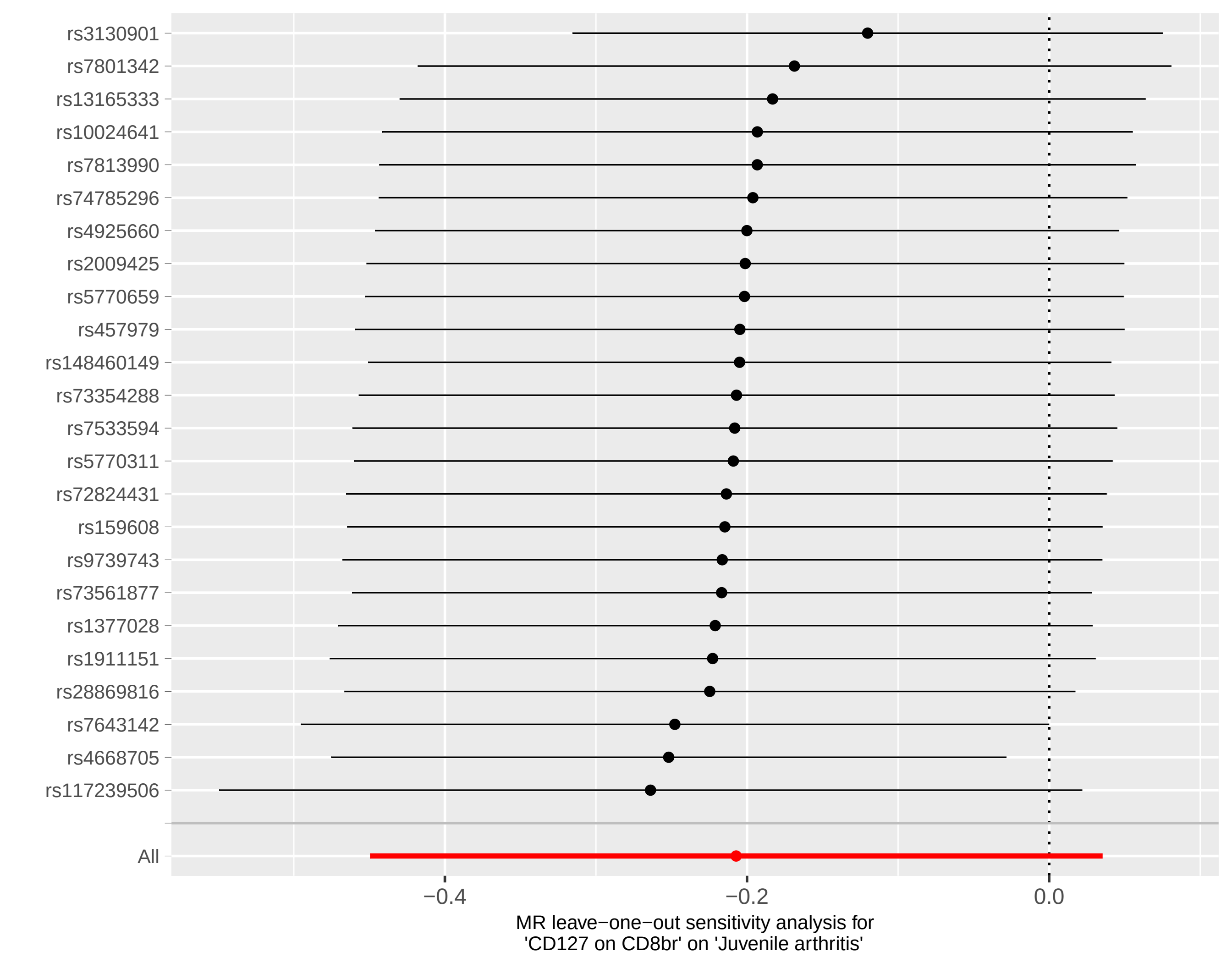
rs3130901
rs7801342
rs13165333
rs10024641
rs7813990
rs74785296
rs4925660
rs2009425
rs5770659
rs457979
rs148460149
rs73354288
rs7533594
rs5770311
rs72824431
rs159608
rs9739743
rs73561877
rs1377028
rs1911151
rs28869816
rs7643142
rs4668705
rs117239506
All
−0.4
−0.2
0.0
MR leave−one−out sensitivity analysis for
'CD127 on CD8br' on 'Juvenile arthritis'

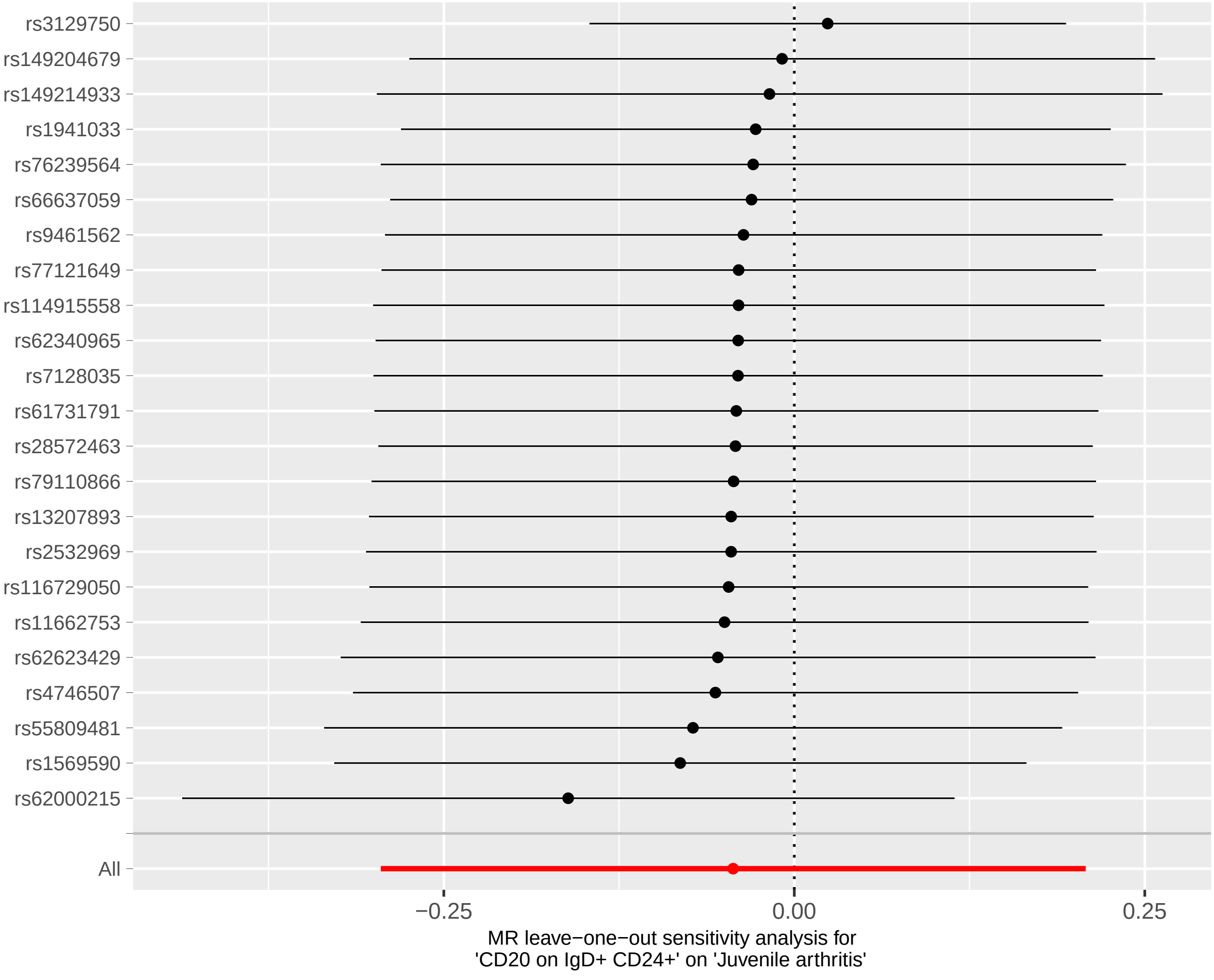
rs3129750
rs149204679
rs149214933
rs1941033
rs76239564
rs66637059
rs9461562
rs77121649
rs114915558
rs62340965
rs7128035
rs61731791
rs28572463
rs79110866
rs13207893
rs2532969
rs116729050
rs11662753
rs62623429
rs4746507
rs55809481
rs1569590
rs62000215
All
−0.25
0.00
0.25
MR leave−one−out sensitivity analysis for
'CD20 on IgD+ CD24+' on 'Juvenile arthritis'

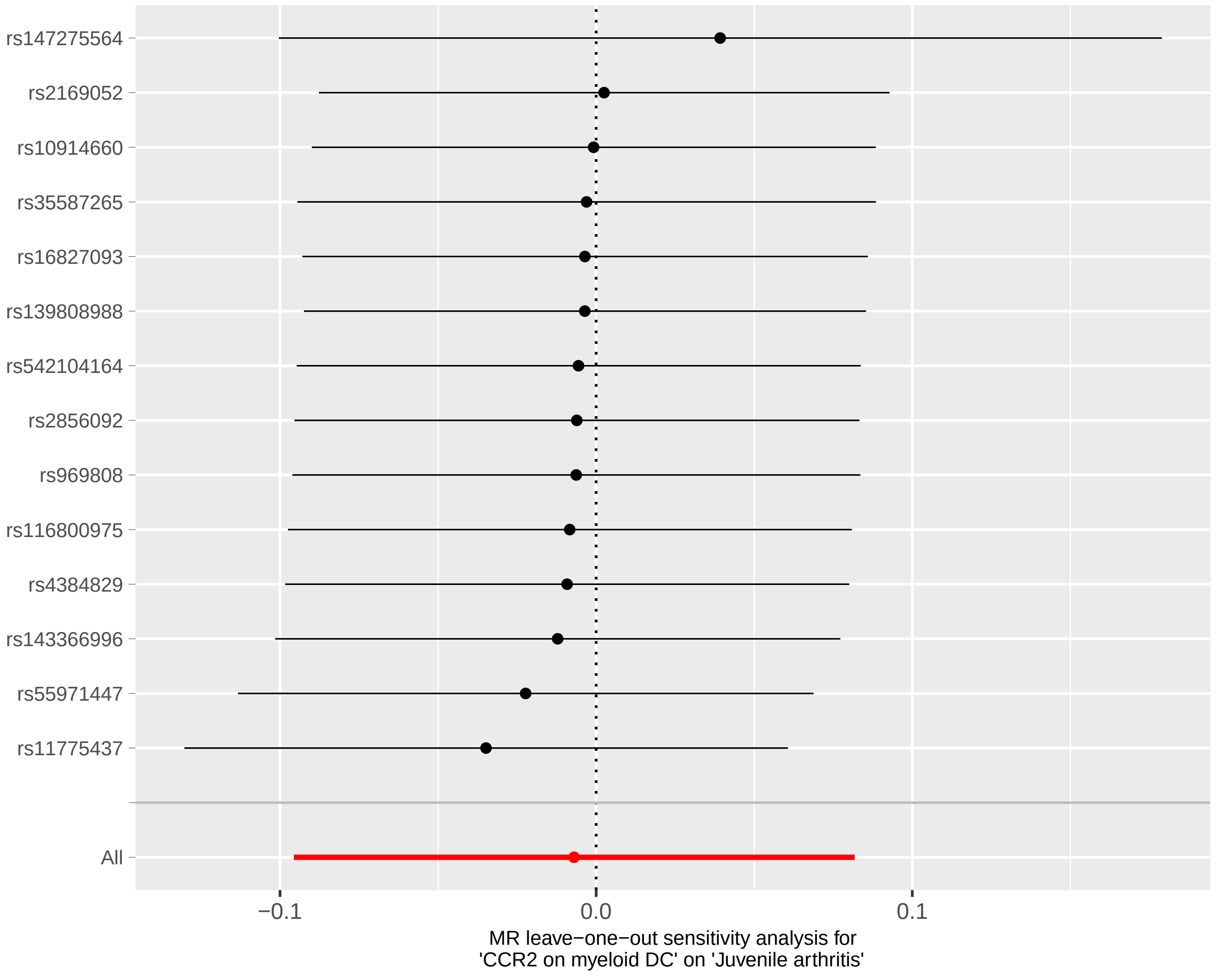

rs147275564
rs2169052
rs10914660
rs35587265
rs16827093
rs139808988
rs542104164
rs2856092
rs969808
rs116800975
rs4384829
rs143366996
rs55971447
rs11775437
All
−0.1
0.0
0.1
MR leave−one−out sensitivity analysis for
'CCR2 on myeloid DC' on 'Juvenile arthritis'

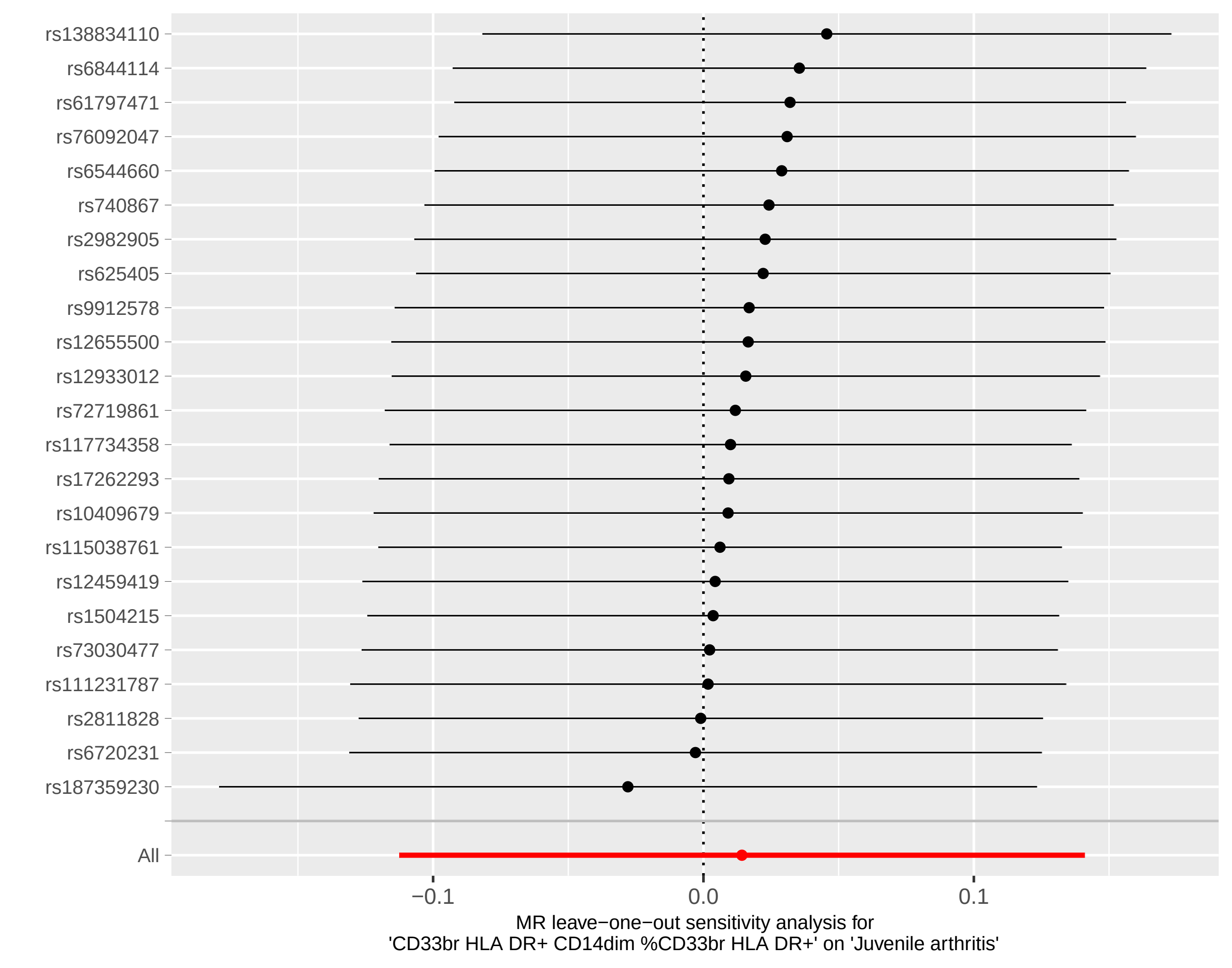
rs138834110
rs6844114
rs61797471
rs76092047
rs6544660
rs740867
rs2982905
rs625405
rs9912578
rs12655500
rs12933012
rs72719861
rs117734358
rs17262293
rs10409679
rs115038761
rs12459419
rs1504215
rs73030477
rs111231787
rs2811828
rs6720231
rs187359230
All
−0.1
0.0
0.1
MR leave−one−out sensitivity analysis for
'CD33br HLA DR+ CD14dim %CD33br HLA DR+' on 'Juvenile arthritis'

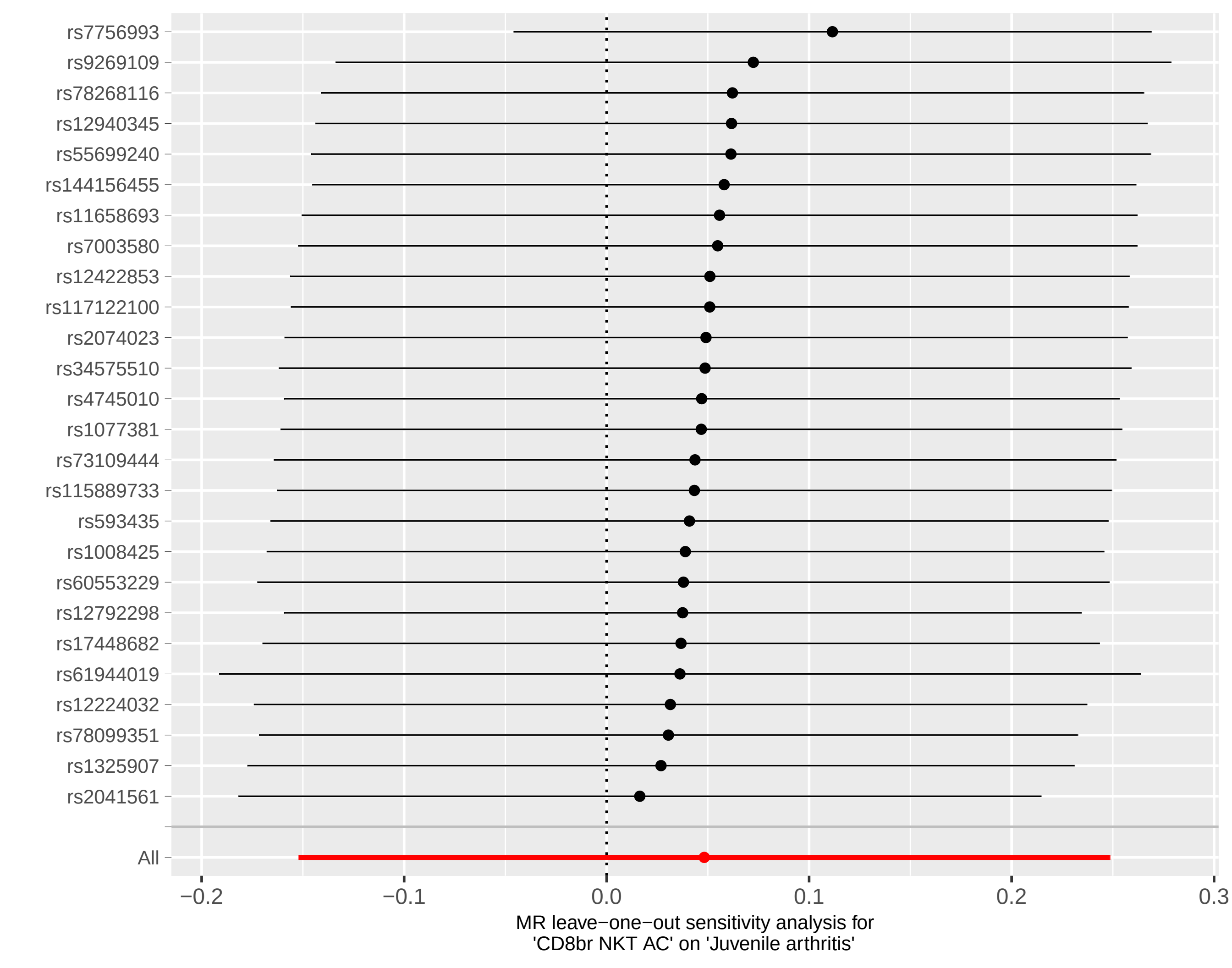
rs7756993
rs9269109
rs78268116
rs12940345
rs55699240
rs144156455
rs11658693
rs7003580
rs12422853
rs117122100
rs2074023
rs34575510
rs4745010
rs1077381
rs73109444
rs115889733
rs593435
rs1008425
rs60553229
rs12792298
rs17448682
rs61944019
rs12224032
rs78099351
rs1325907
rs2041561
All
−0.2
−0.1
0.0
0.1
0.2
0.3
MR leave−one−out sensitivity analysis for
'CD8br NKT AC' on 'Juvenile arthritis'

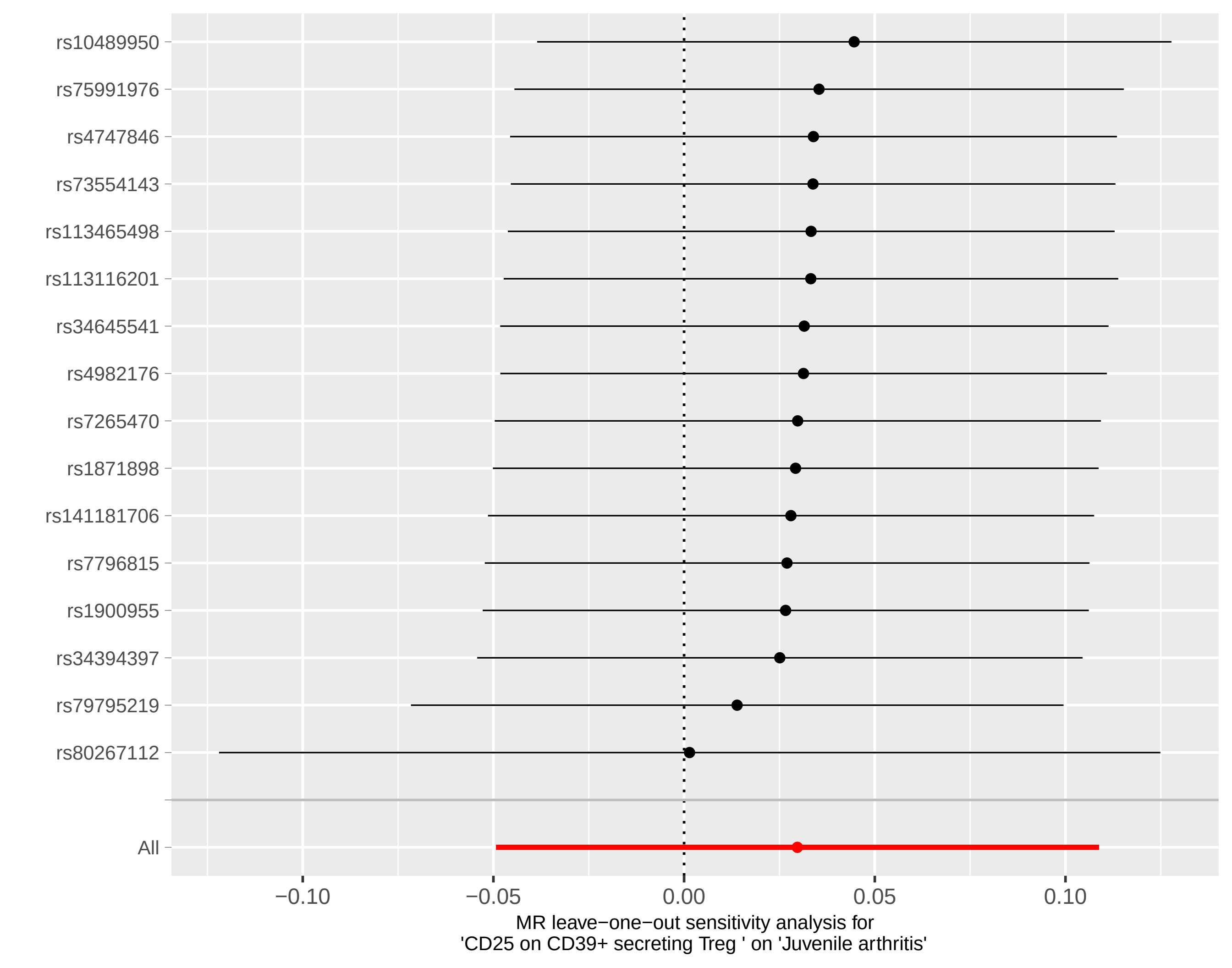
rs10489950
rs75991976
rs4747846
rs73554143
rs113465498
rs113116201
rs34645541
rs4982176
rs7265470
rs1871898
rs141181706
rs7796815
rs1900955
rs34394397
rs79795219
rs80267112
All
−0.10
−0.05
0.00
0.05
0.10
MR leave−one−out sensitivity analysis for
'CD25 on CD39+ secreting Treg ' on 'Juvenile arthritis'

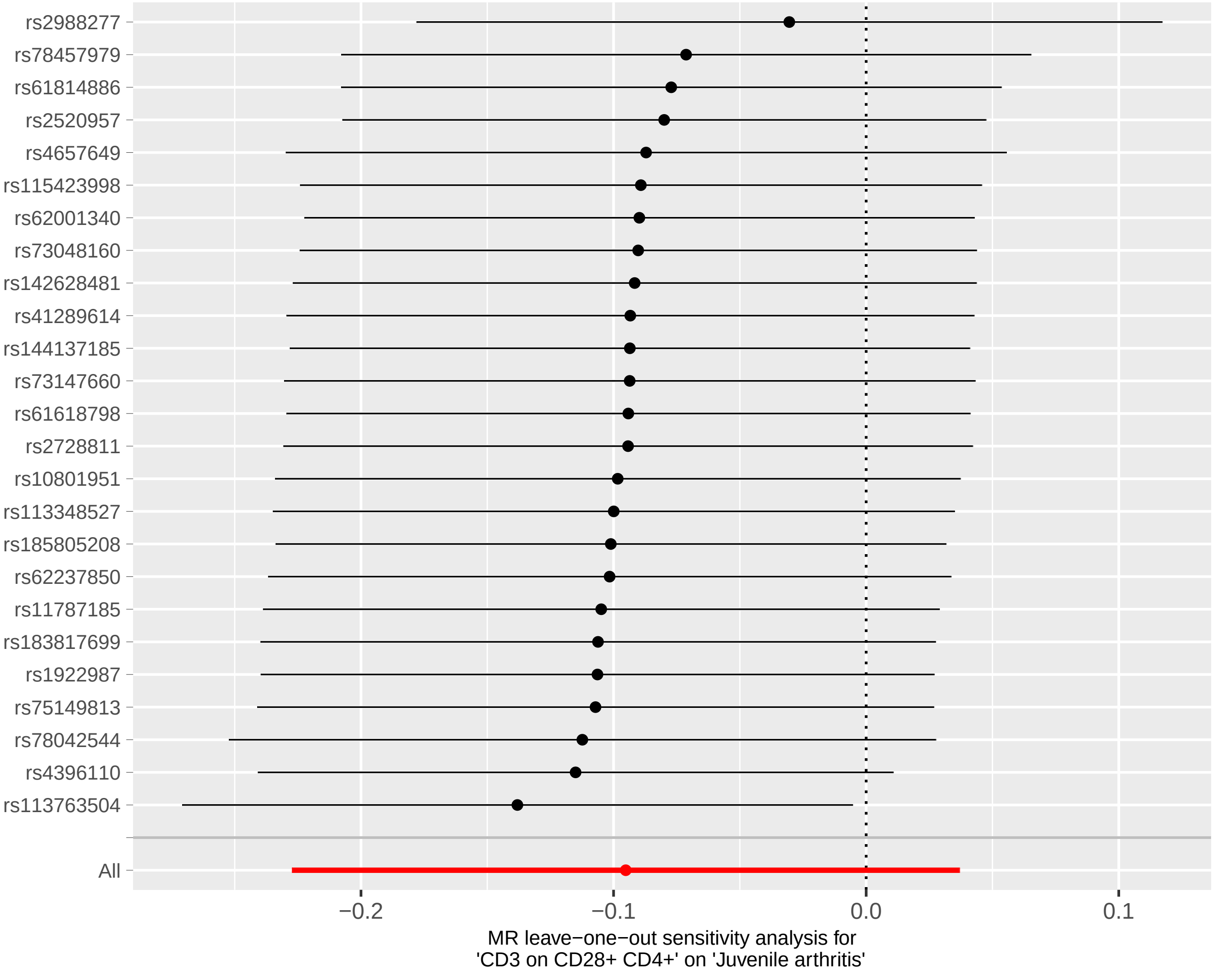

rs2988277
rs78457979
rs61814886
rs2520957
rs4657649
rs115423998
rs62001340
rs73048160
rs142628481
rs41289614
rs144137185
rs73147660
rs61618798
rs2728811
rs10801951
rs113348527
rs185805208
rs62237850
rs11787185
rs183817699
rs1922987
rs75149813
rs78042544
rs4396110
rs113763504
All
−0.2
−0.1
0.0
0.1
MR leave−one−out sensitivity analysis for
'CD3 on CD28+ CD4+' on 'Juvenile arthritis'

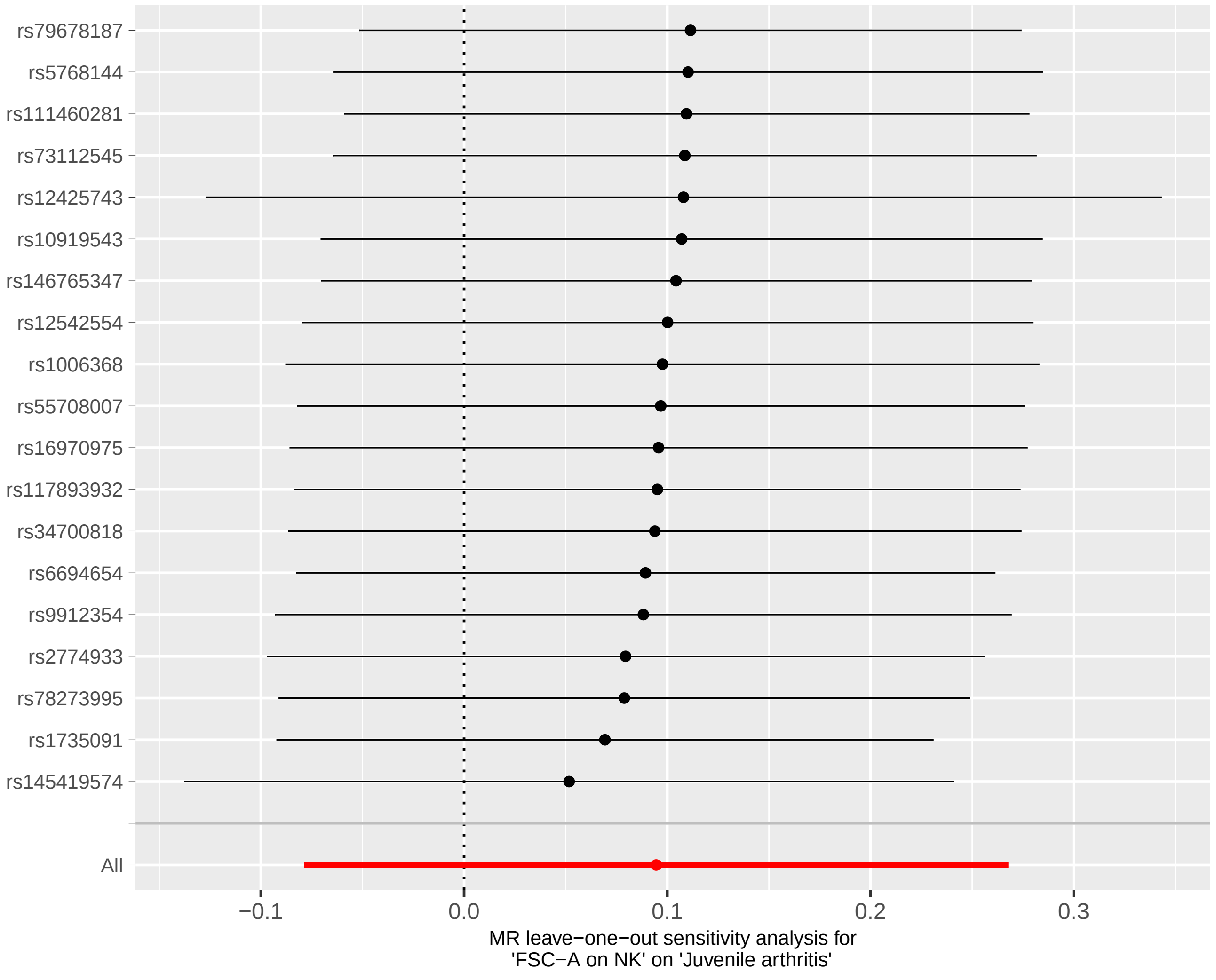
rs79678187
rs5768144
rs111460281
rs73112545
rs12425743
rs10919543
rs146765347
rs12542554
rs1006368
rs55708007
rs16970975
rs117893932
rs34700818
rs6694654
rs9912354
rs2774933
rs78273995
rs1735091
rs145419574
All
−0.1
0.0
0.1
0.2
0.3
MR leave−one−out sensitivity analysis for
'FSC−A on NK' on 'Juvenile arthritis'

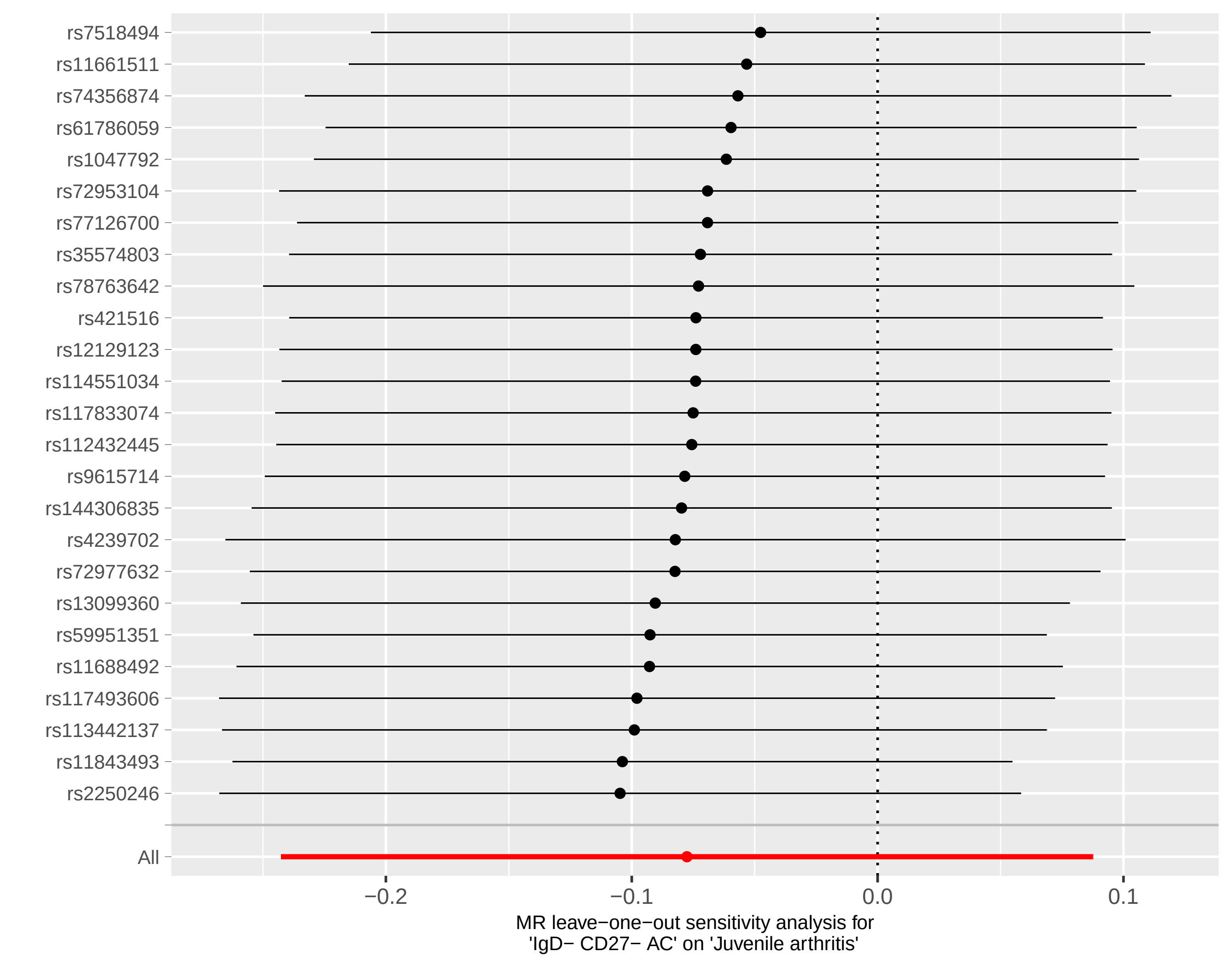
rs7518494
rs11661511
rs74356874
rs61786059
rs1047792
rs72953104
rs77126700
rs35574803
rs78763642
rs421516
rs12129123
rs114551034
rs117833074
rs112432445
rs9615714
rs144306835
rs4239702
rs72977632
rs13099360
rs59951351
rs11688492
rs117493606
rs113442137
rs11843493
rs2250246
All
−0.2
−0.1
0.0
0.1
MR leave−one−out sensitivity analysis for
'IgD− CD27− AC' on 'Juvenile arthritis'

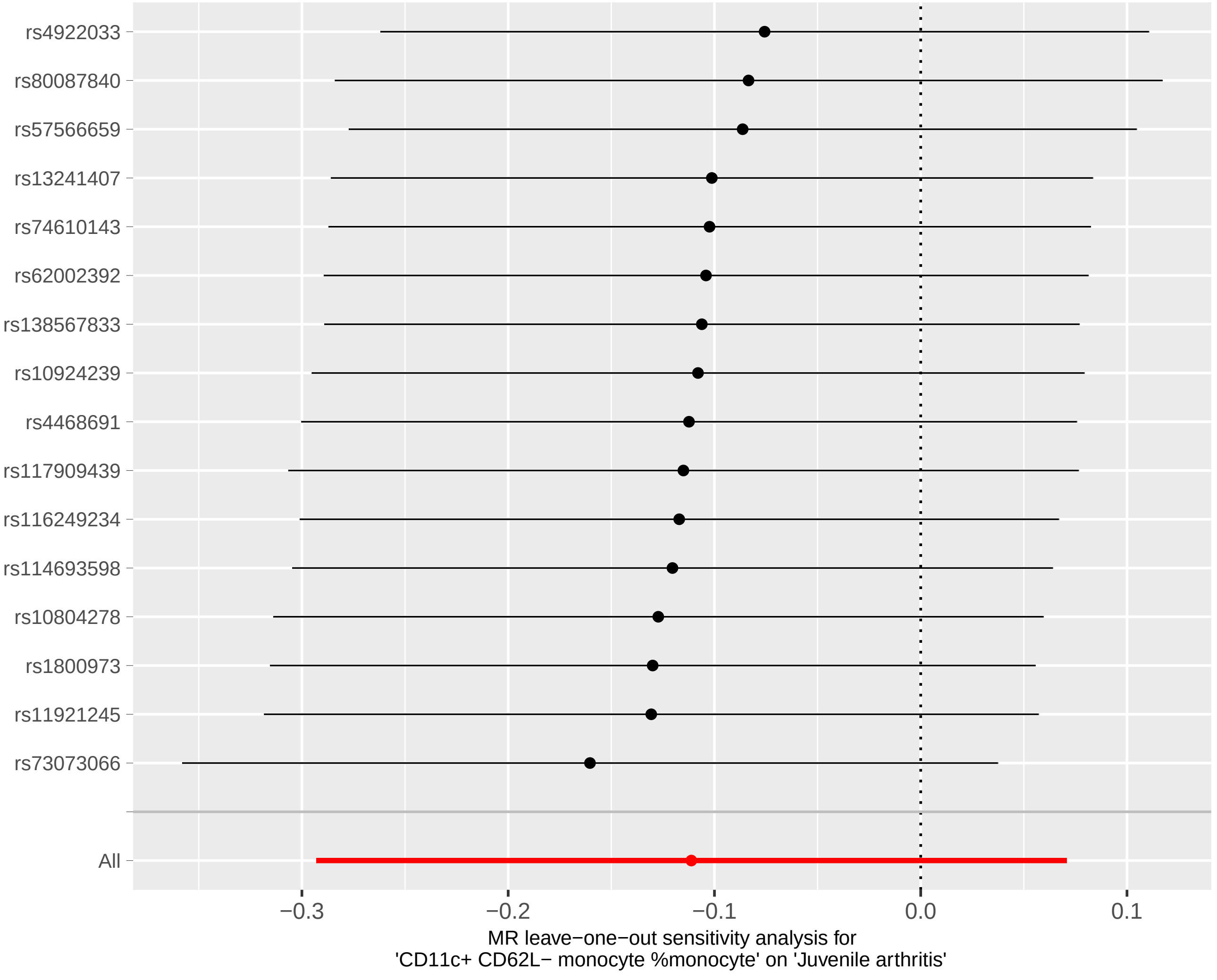
rs4922033
rs80087840
rs57566659
rs13241407
rs74610143
rs62002392
rs138567833
rs10924239
rs4468691
rs117909439
rs116249234
rs114693598
rs10804278
rs1800973
rs11921245
rs73073066
All
−0.3
−0.2
−0.1
0.0
0.1
MR leave−one−out sensitivity analysis for
'CD11c+ CD62L− monocyte %monocyte' on 'Juvenile arthritis'

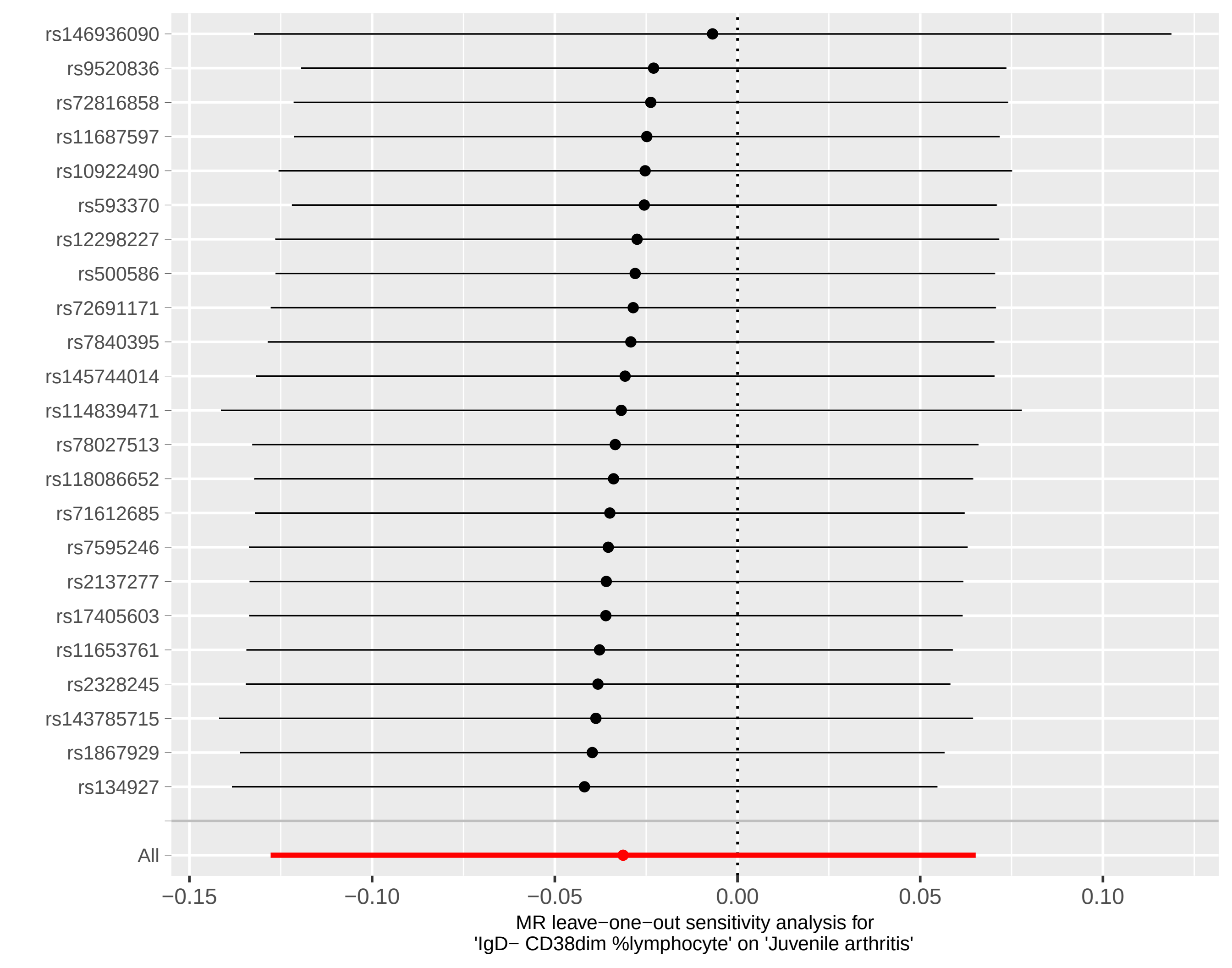
rs146936090
rs9520836
rs72816858
rs11687597
rs10922490
rs593370
rs12298227
rs500586
rs72691171
rs7840395
rs145744014
rs114839471
rs78027513
rs118086652
rs71612685
rs7595246
rs2137277
rs17405603
rs11653761
rs2328245
rs143785715
rs1867929
rs134927
All
−0.15
−0.10
−0.05
0.00
0.05
0.10
MR leave−one−out sensitivity analysis for
'IgD− CD38dim %lymphocyte' on 'Juvenile arthritis'

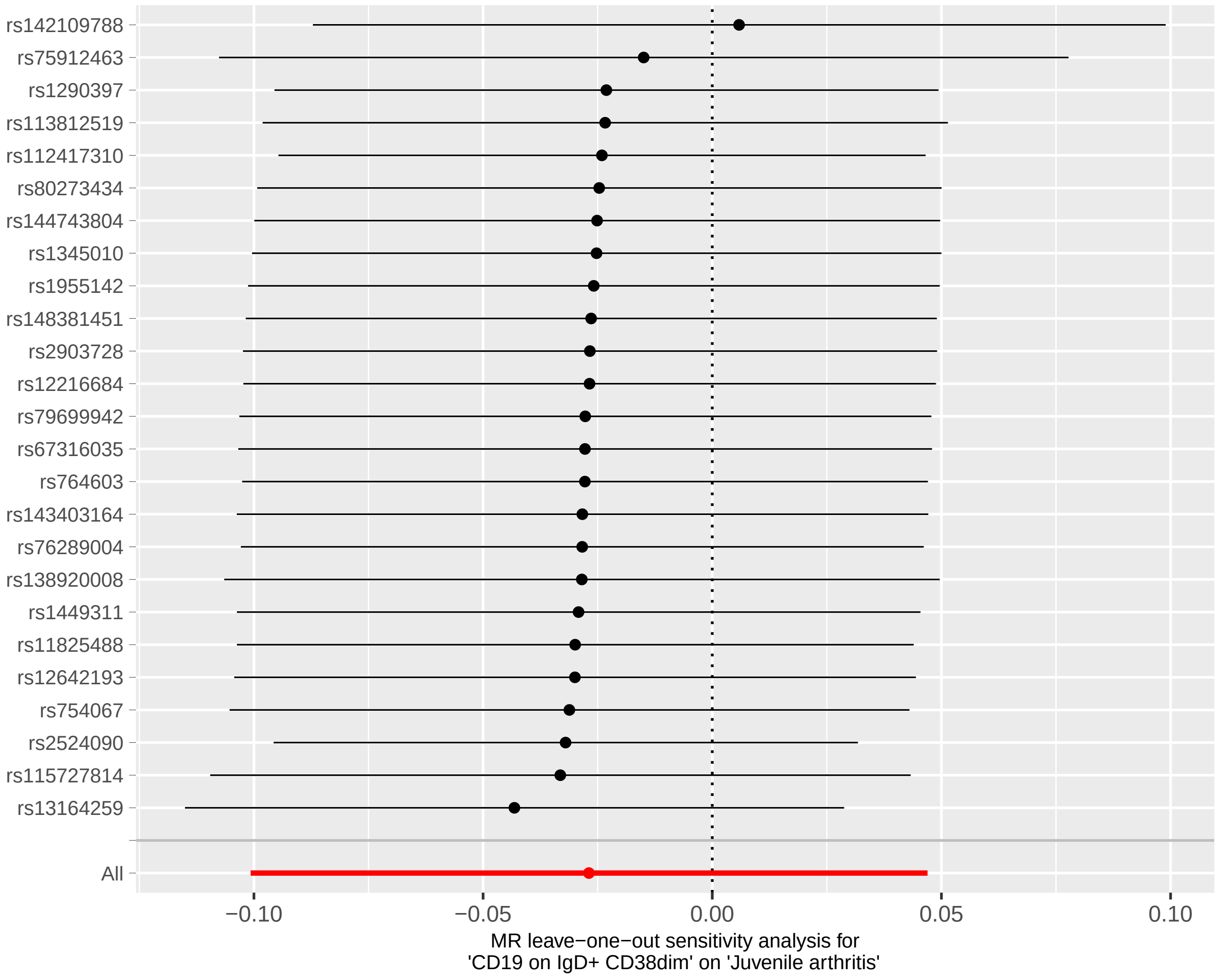
rs142109788
rs75912463
rs1290397
rs113812519
rs112417310
rs80273434
rs144743804
rs1345010
rs1955142
rs148381451
rs2903728
rs12216684
rs79699942
rs67316035
rs764603
rs143403164
rs76289004
rs138920008
rs1449311
rs11825488
rs12642193
rs754067
rs2524090
rs115727814
rs13164259
All
−0.10
−0.05
0.00
0.05
0.10
MR leave−one−out sensitivity analysis for
'CD19 on IgD+ CD38dim' on 'Juvenile arthritis'

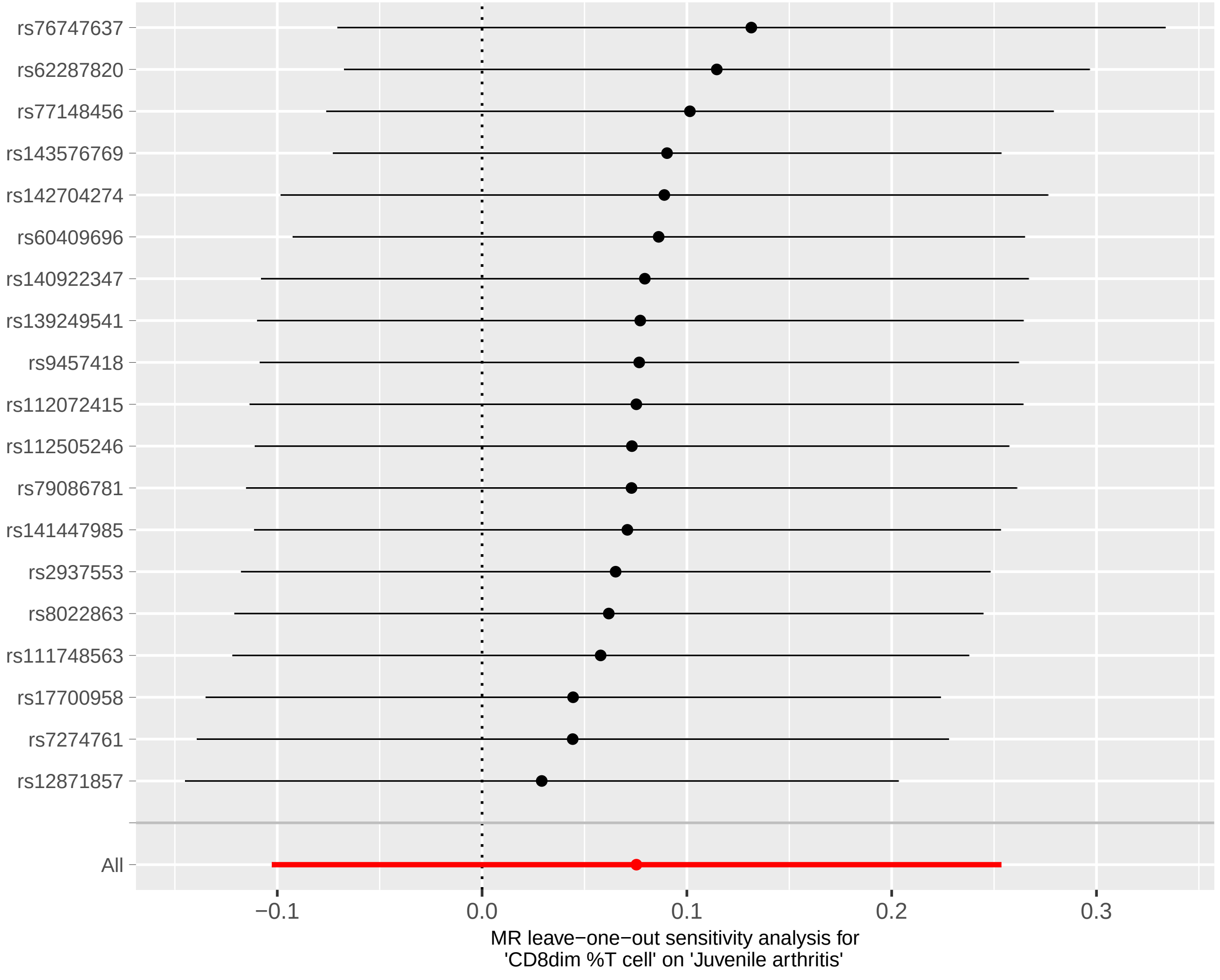

rs76747637
rs62287820
rs77148456
rs143576769
rs142704274
rs60409696
rs140922347
rs139249541
rs9457418
rs112072415
rs112505246
rs79086781
rs141447985
rs2937553
rs8022863
rs111748563
rs17700958
rs7274761
rs12871857
All
−0.1
0.0
0.1
0.2
0.3
MR leave−one−out sensitivity analysis for
'CD8dim %T cell' on 'Juvenile arthritis'

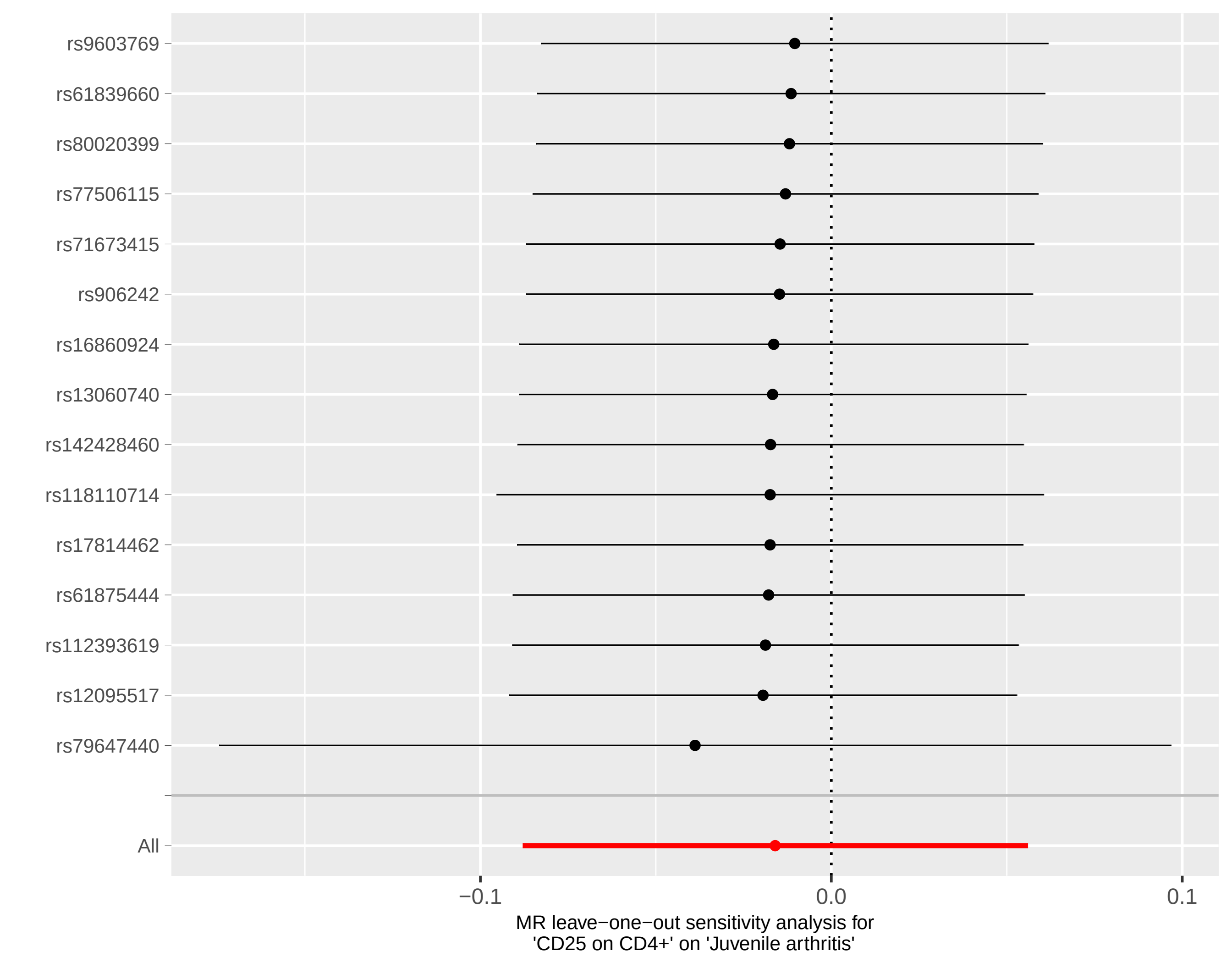
rs9603769
rs61839660
rs80020399
rs77506115
rs71673415
rs906242
rs16860924
rs13060740
rs142428460
rs118110714
rs17814462
rs61875444
rs112393619
rs12095517
rs79647440
All
−0.1
0.0
0.1
MR leave−one−out sensitivity analysis for
'CD25 on CD4+' on 'Juvenile arthritis'

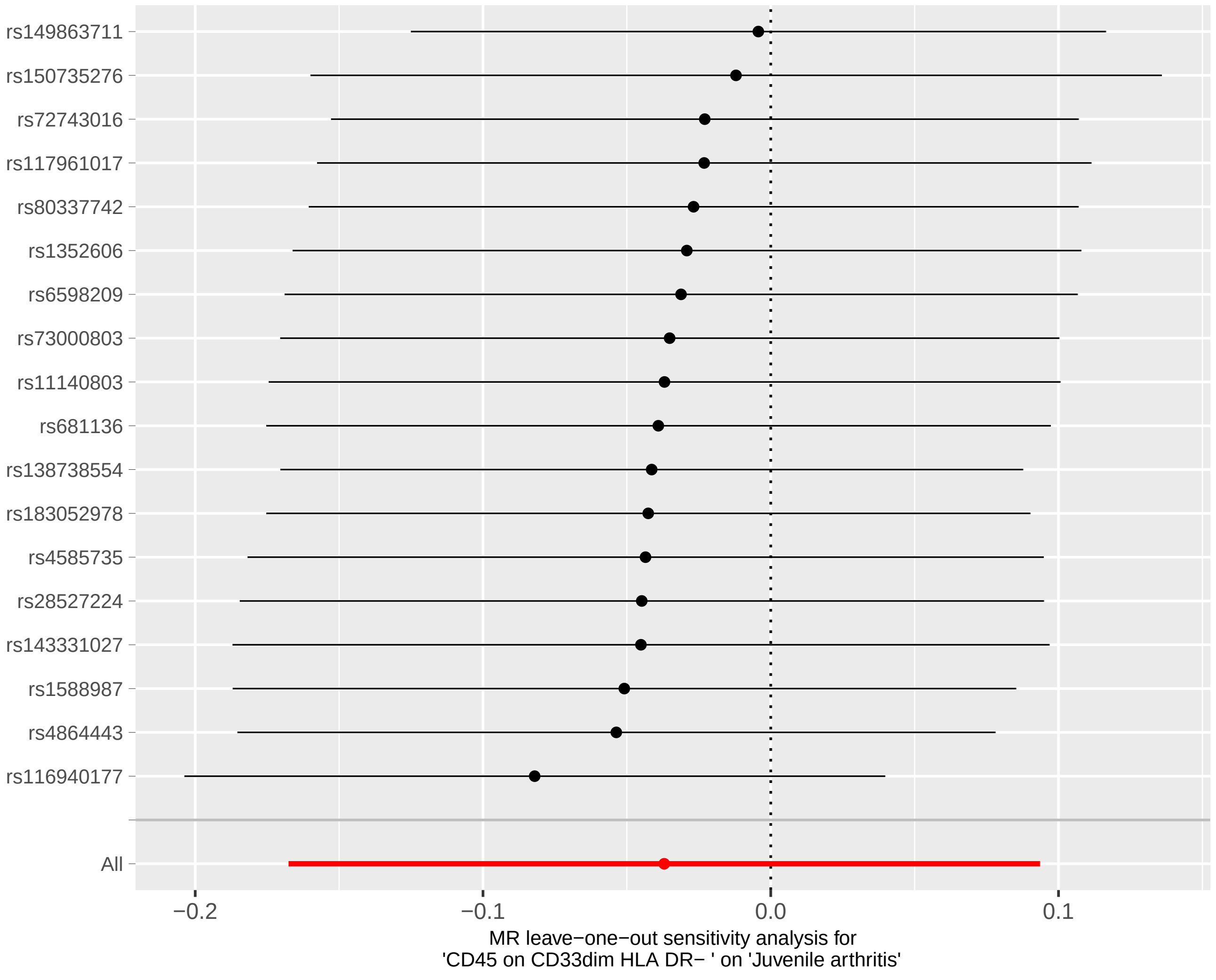

rs149863711
rs150735276
rs72743016
rs117961017
rs80337742
rs1352606
rs6598209
rs73000803
rs11140803
rs681136
rs138738554
rs183052978
rs4585735
rs28527224
rs143331027
rs1588987
rs4864443
rs116940177
All
−0.2
−0.1
0.0
0.1
MR leave−one−out sensitivity analysis for
'CD45 on CD33dim HLA DR− ' on 'Juvenile arthritis'

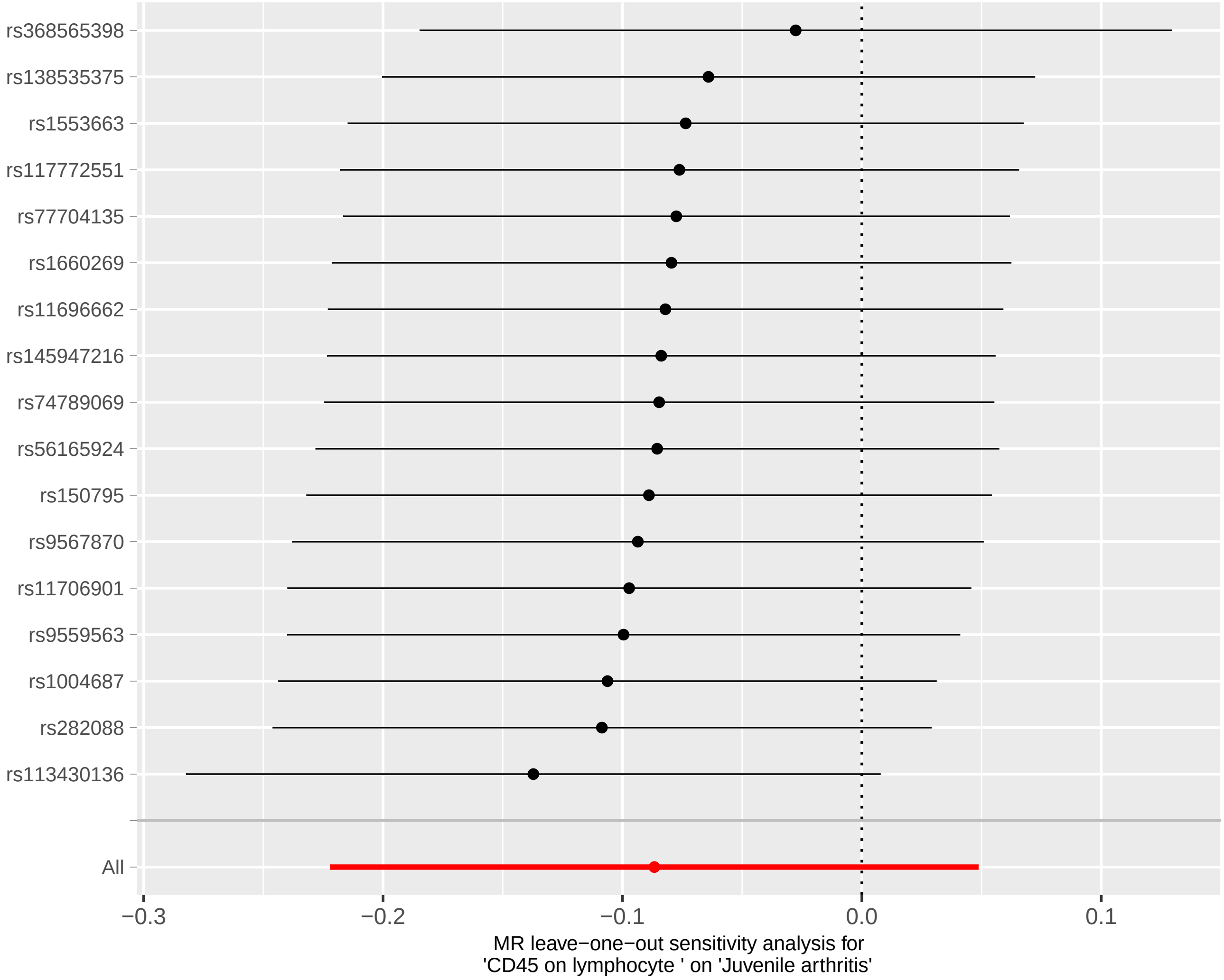

rs368565398
rs138535375
rs1553663
rs117772551
rs77704135
rs1660269
rs11696662
rs145947216
rs74789069
rs56165924
rs150795
rs9567870
rs11706901
rs9559563
rs1004687
rs282088
rs113430136
All
−0.3
−0.2
−0.1
0.0
0.1
MR leave−one−out sensitivity analysis for
'CD45 on lymphocyte ' on 'Juvenile arthritis'

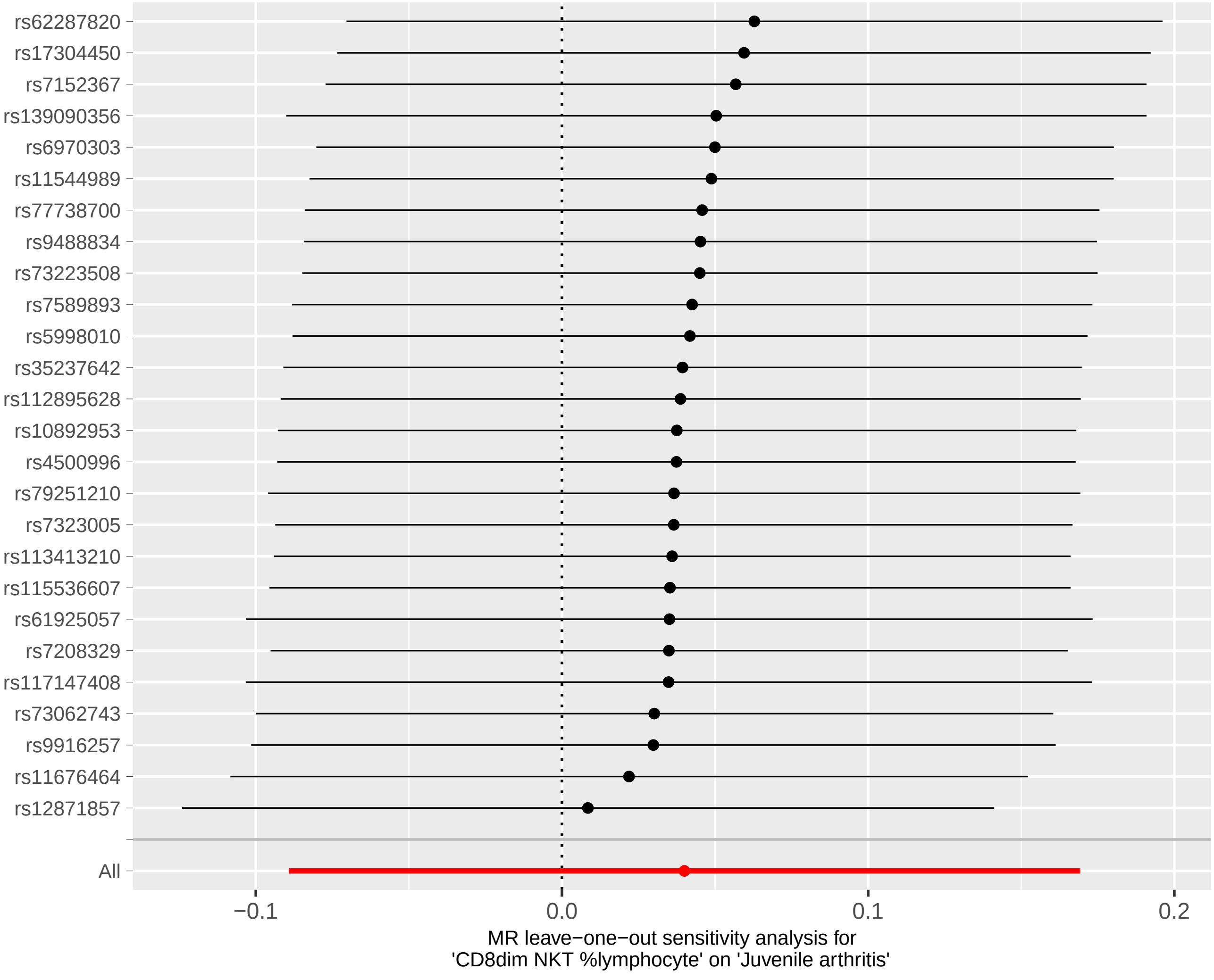

rs62287820
rs17304450
rs7152367
rs139090356
rs6970303
rs11544989
rs77738700
rs9488834
rs73223508
rs7589893
rs5998010
rs35237642
rs112895628
rs10892953
rs4500996
rs79251210
rs7323005
rs113413210
rs115536607
rs61925057
rs7208329
rs117147408
rs73062743
rs9916257
rs11676464
rs12871857
All
−0.1
0.0
0.1
0.2
MR leave−one−out sensitivity analysis for
'CD8dim NKT %lymphocyte' on 'Juvenile arthritis'

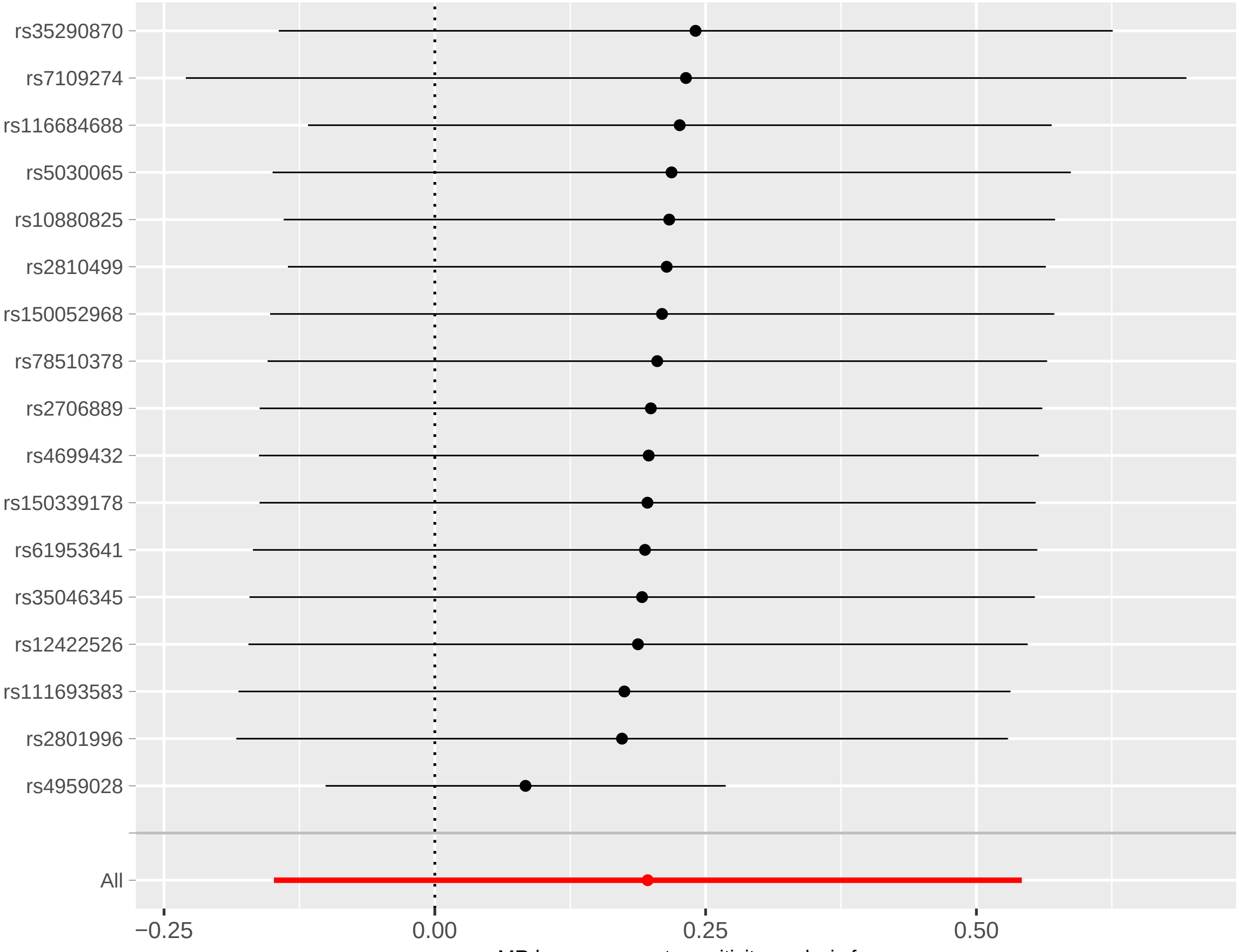

MR leave−one−out sensitivity analysis for
'CD4+ CD8dim AC' on 'Juvenile arthritis'

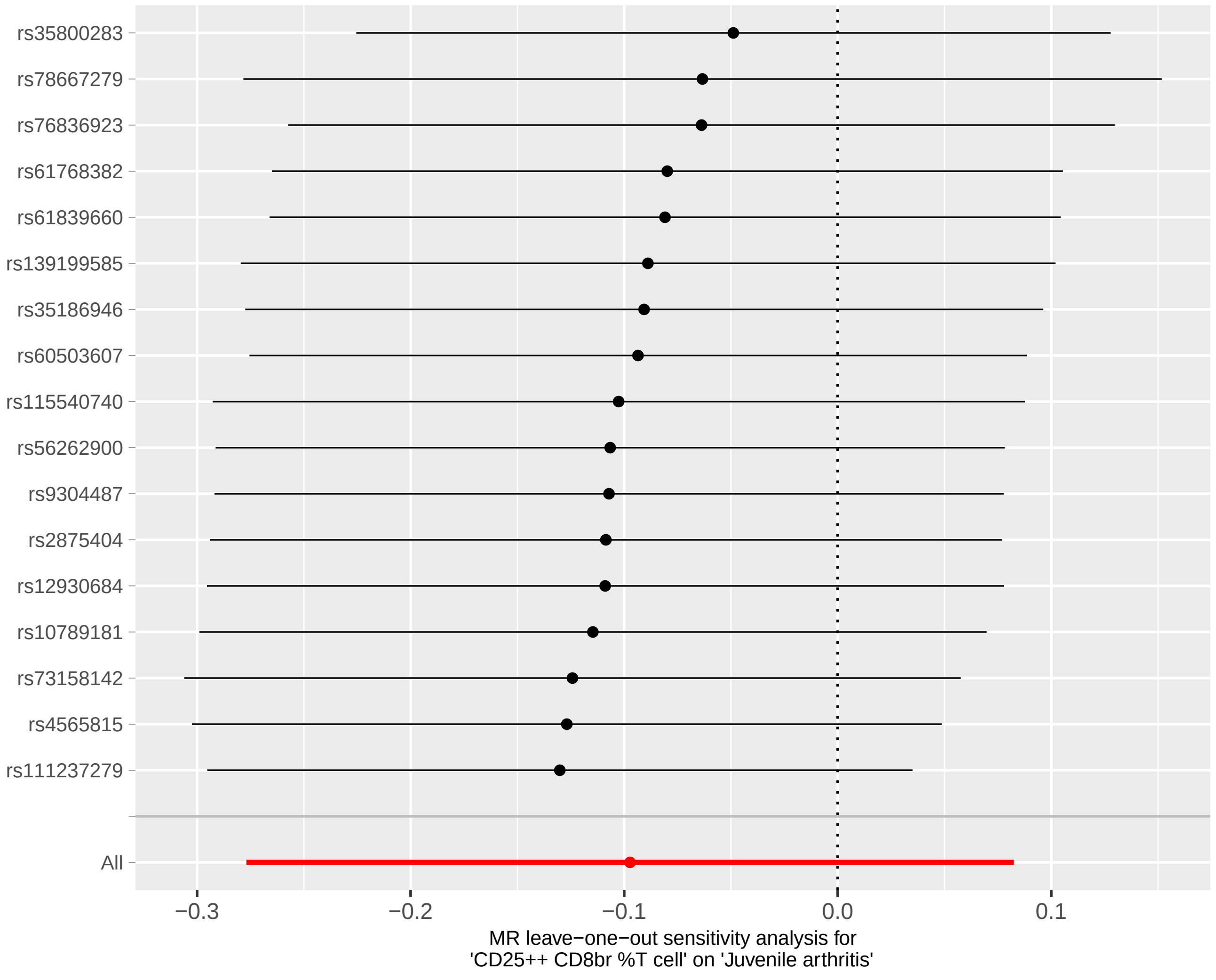
rs35800283
rs78667279
rs76836923
rs61768382
rs61839660
rs139199585
rs35186946
rs60503607
rs115540740
rs56262900
rs9304487
rs2875404
rs12930684
rs10789181
rs73158142
rs4565815
rs111237279
All
−0.3
−0.2
−0.1
0.0
0.1
MR leave−one−out sensitivity analysis for
'CD25++ CD8br %T cell' on 'Juvenile arthritis'

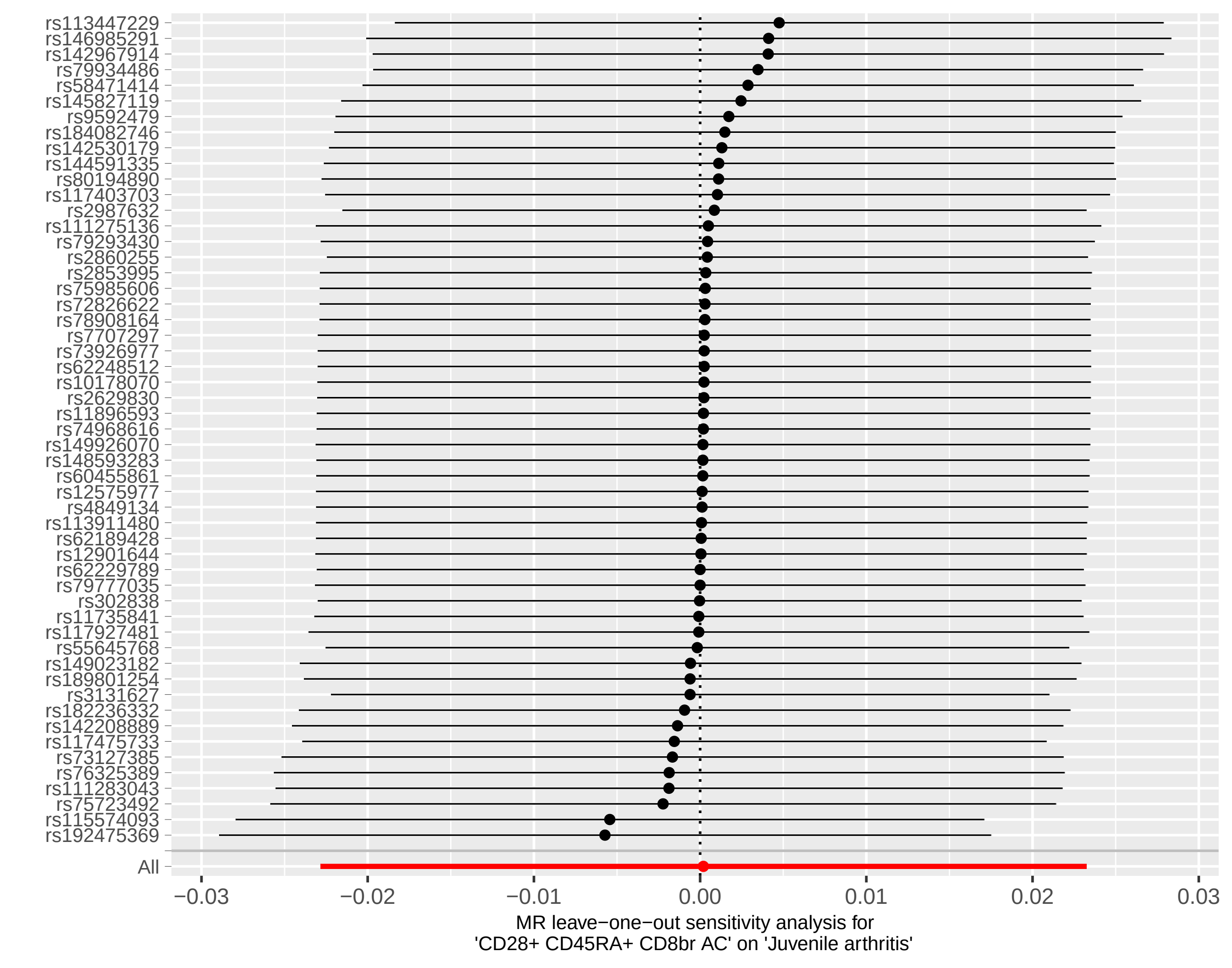
rs113447229
rs146985291
rs142967914
rs79934486
rs58471414
rs145827119
rs9592479
rs184082746
rs142530179
rs144591335
rs80194890
rs117403703
rs2987632
rs111275136
rs79293430
rs2860255
rs2853995
rs75985606
rs72826622
rs78908164
rs7707297
rs73926977
rs62248512
rs10178070
rs2629830
rs11896593
rs74968616
rs149926070
rs148593283
rs60455861
rs12575977
rs4849134
rs113911480
rs62189428
rs12901644
rs62229789
rs79777035
rs302838
rs11735841
rs117927481
rs55645768
rs149023182
rs189801254
rs3131627
rs182236332
rs142208889
rs117475733
rs73127385
rs76325389
rs111283043
rs75723492
rs115574093
rs192475369
All
−0.03
−0.02
−0.01
0.00
0.01
0.02
0.03
MR leave−one−out sensitivity analysis for
'CD28+ CD45RA+ CD8br AC' on 'Juvenile arthritis'

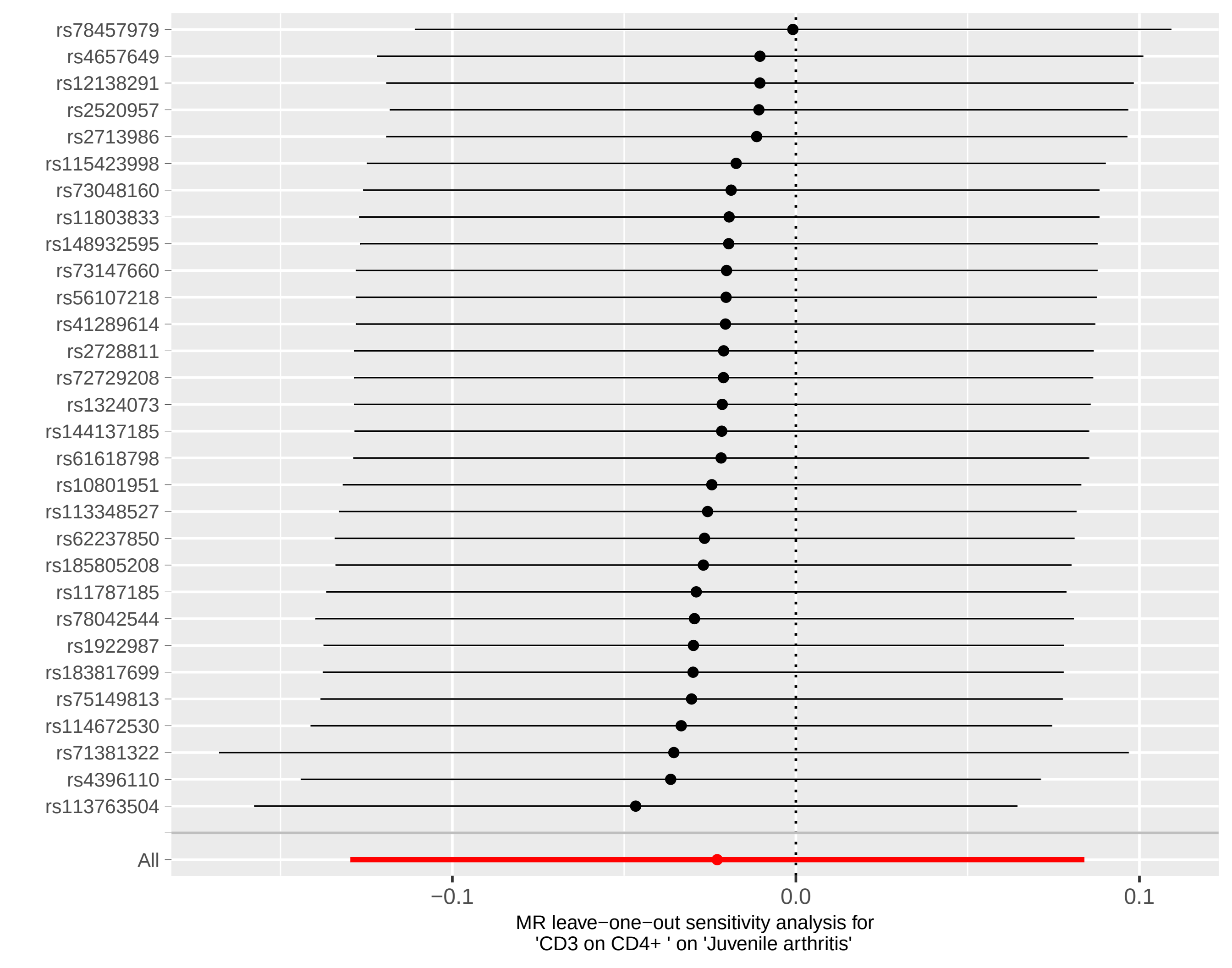
rs78457979
rs4657649
rs12138291
rs2520957
rs2713986
rs115423998
rs73048160
rs11803833
rs148932595
rs73147660
rs56107218
rs41289614
rs2728811
rs72729208
rs1324073
rs144137185
rs61618798
rs10801951
rs113348527
rs62237850
rs185805208
rs11787185
rs78042544
rs1922987
rs183817699
rs75149813
rs114672530
rs71381322
rs4396110
rs113763504
All
−0.1
0.0
0.1
MR leave−one−out sensitivity analysis for
'CD3 on CD4+ ' on 'Juvenile arthritis'

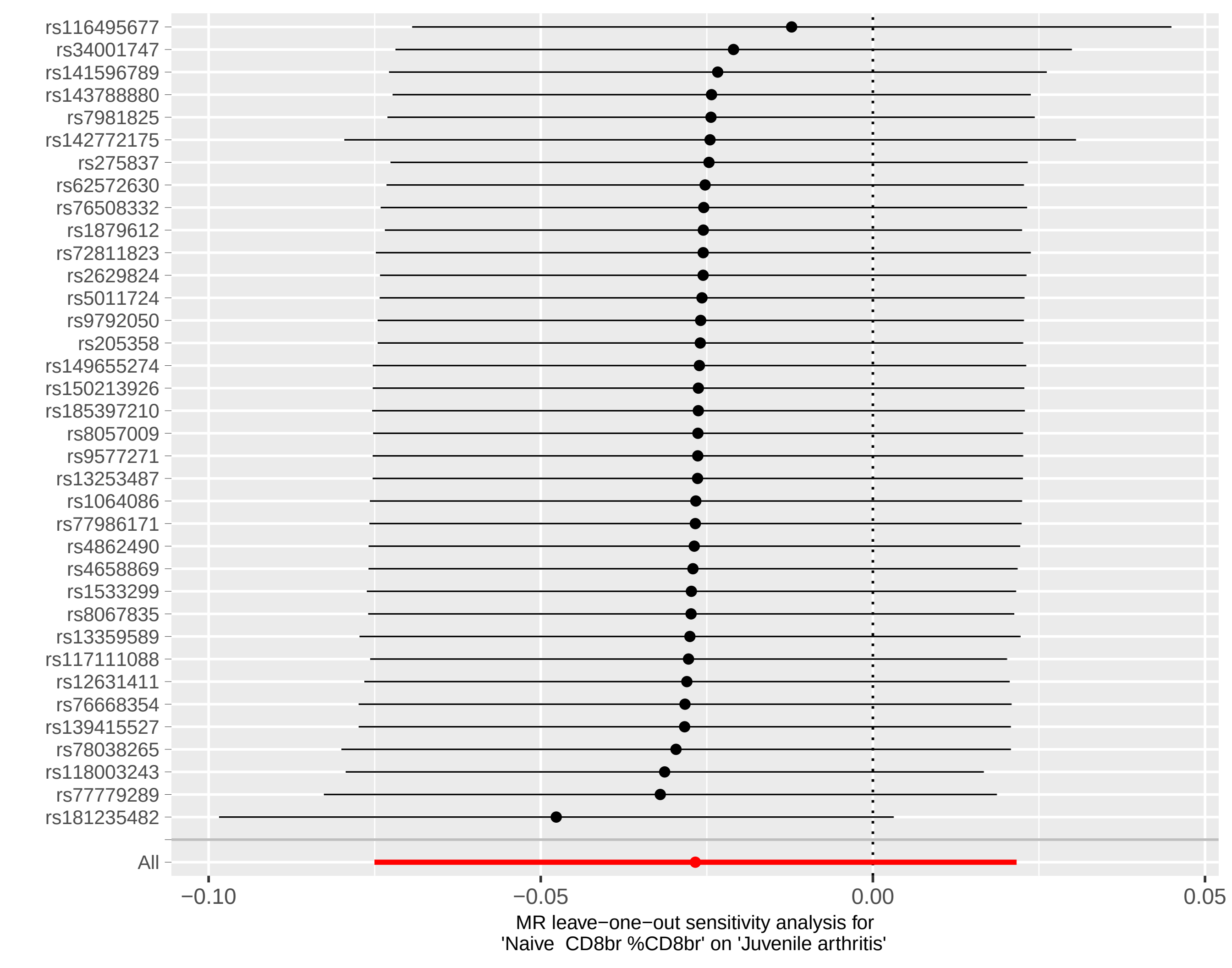
rs116495677
rs34001747
rs141596789
rs143788880
rs7981825
rs142772175
rs275837
rs62572630
rs76508332
rs1879612
rs72811823
rs2629824
rs5011724
rs9792050
rs205358
rs149655274
rs150213926
rs185397210
rs8057009
rs9577271
rs13253487
rs1064086
rs77986171
rs4862490
rs4658869
rs1533299
rs8067835
rs13359589
rs117111088
rs12631411
rs76668354
rs139415527
rs78038265
rs118003243
rs77779289
rs181235482
All
−0.10
−0.05
0.00
0.05
MR leave−one−out sensitivity analysis for
'Naive CD8br %CD8br' on 'Juvenile arthritis'

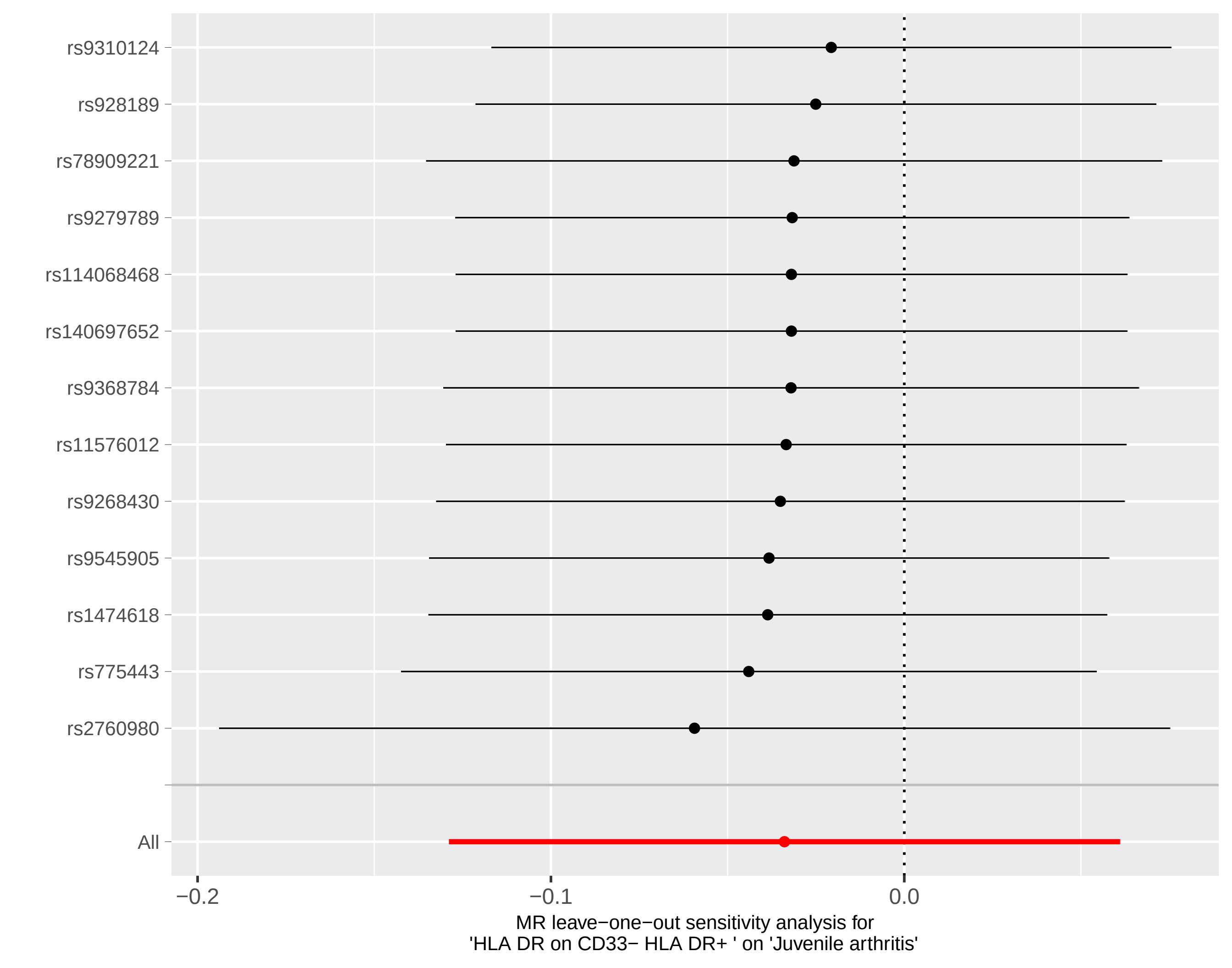

rs9310124
rs928189
rs78909221
rs9279789
rs114068468
rs140697652
rs9368784
rs11576012
rs9268430
rs9545905
rs1474618
rs775443
rs2760980
All
−0.2
−0.1
0.0
MR leave−one−out sensitivity analysis for
'HLA DR on CD33− HLA DR+ ' on 'Juvenile arthritis'

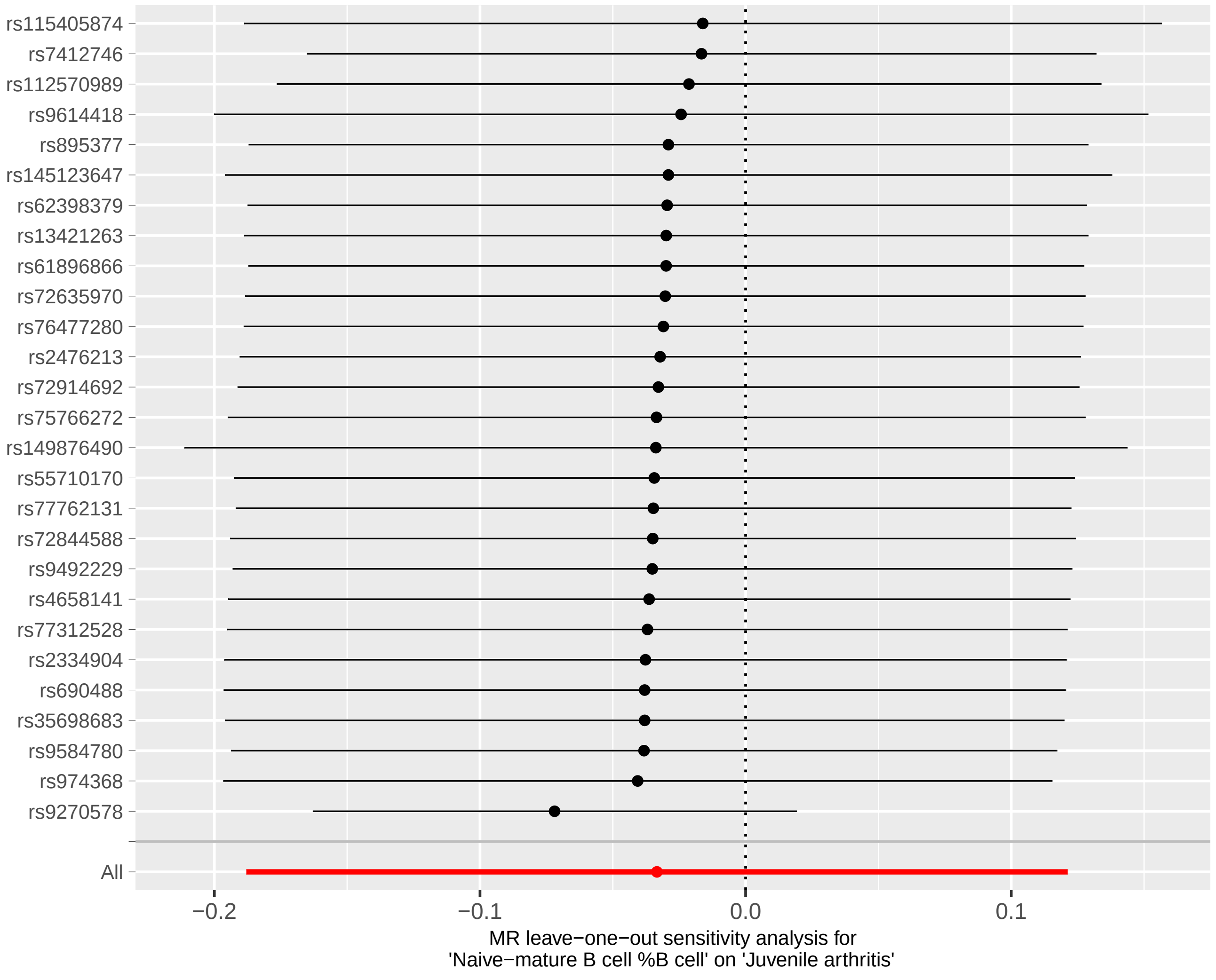
rs115405874
rs7412746
rs112570989
rs9614418
rs895377
rs145123647
rs62398379
rs13421263
rs61896866
rs72635970
rs76477280
rs2476213
rs72914692
rs75766272
rs149876490
rs55710170
rs77762131
rs72844588
rs9492229
rs4658141
rs77312528
rs2334904
rs690488
rs35698683
rs9584780
rs974368
rs9270578
All
−0.2
−0.1
0.0
0.1
MR leave−one−out sensitivity analysis for
'Naive−mature B cell %B cell' on 'Juvenile arthritis'

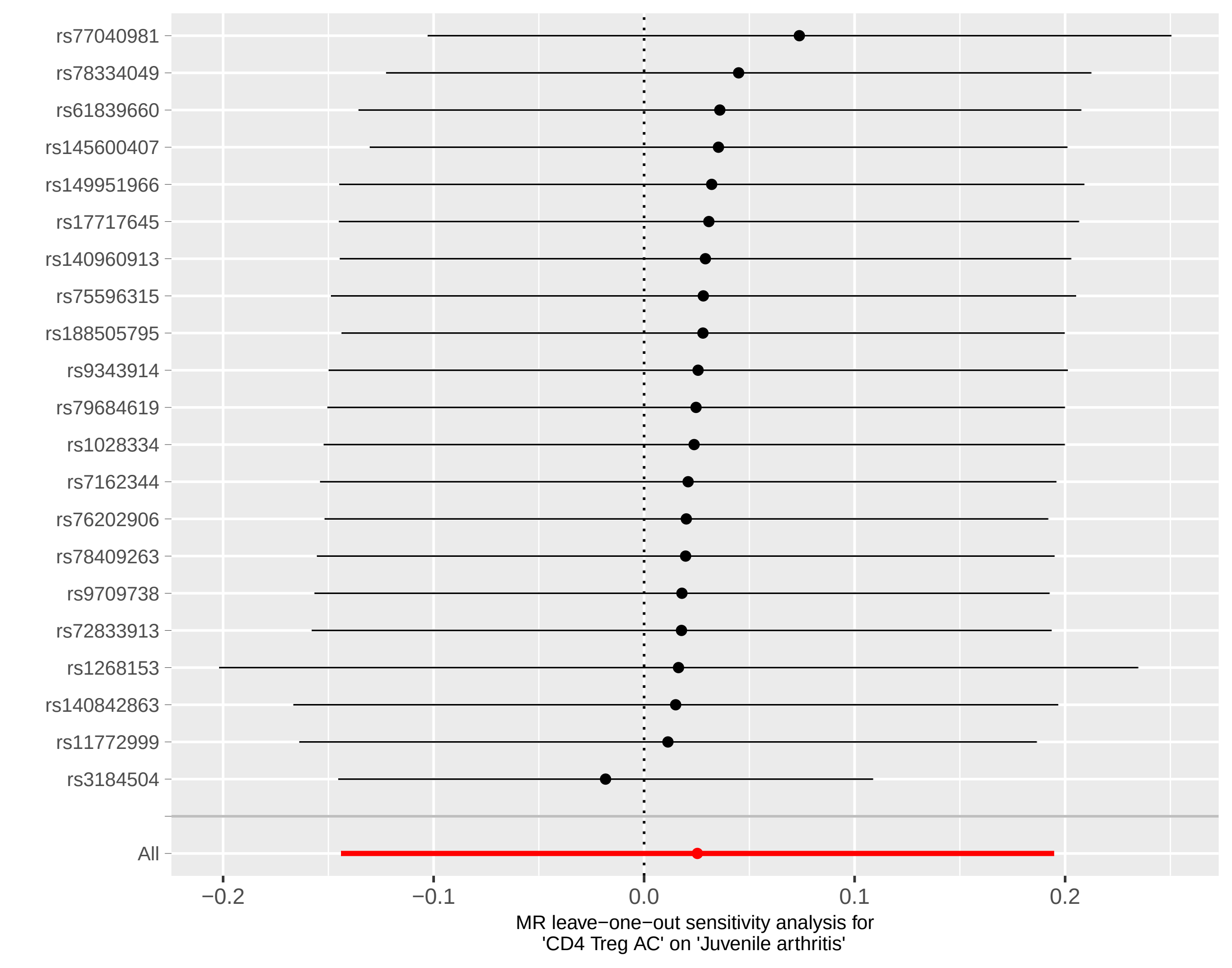
rs77040981
rs78334049
rs61839660
rs145600407
rs149951966
rs17717645
rs140960913
rs75596315
rs188505795
rs9343914
rs79684619
rs1028334
rs7162344
rs76202906
rs78409263
rs9709738
rs72833913
rs1268153
rs140842863
rs11772999
rs3184504
All
−0.2
−0.1
0.0
0.1
0.2
MR leave−one−out sensitivity analysis for
'CD4 Treg AC' on 'Juvenile arthritis'

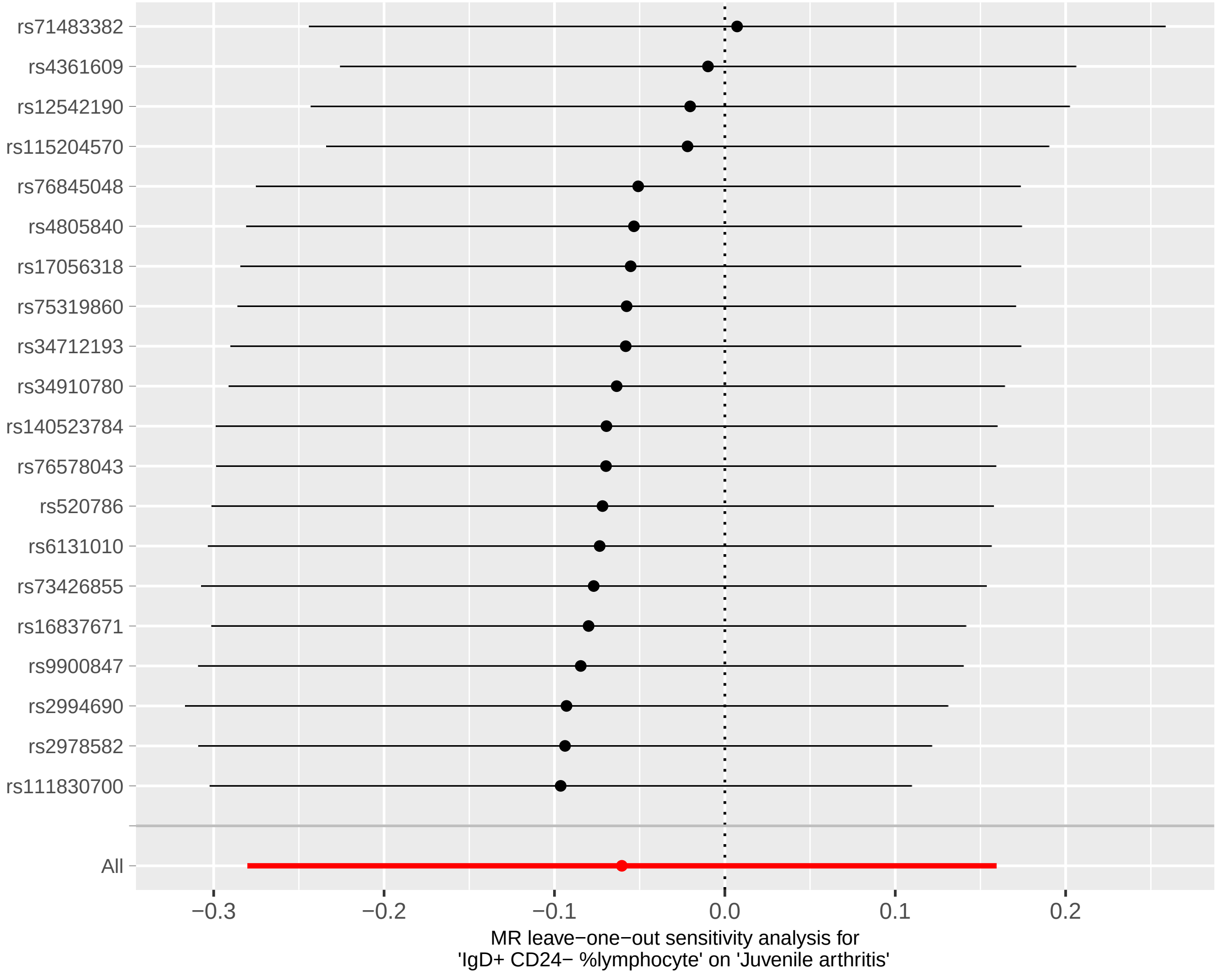
rs71483382
rs4361609
rs12542190
rs115204570
rs76845048
rs4805840
rs17056318
rs75319860
rs34712193
rs34910780
rs140523784
rs76578043
rs520786
rs6131010
rs73426855
rs16837671
rs9900847
rs2994690
rs2978582
rs111830700
All
−0.3
−0.2
−0.1
0.0
0.1
0.2
MR leave−one−out sensitivity analysis for
'IgD+ CD24− %lymphocyte' on 'Juvenile arthritis'

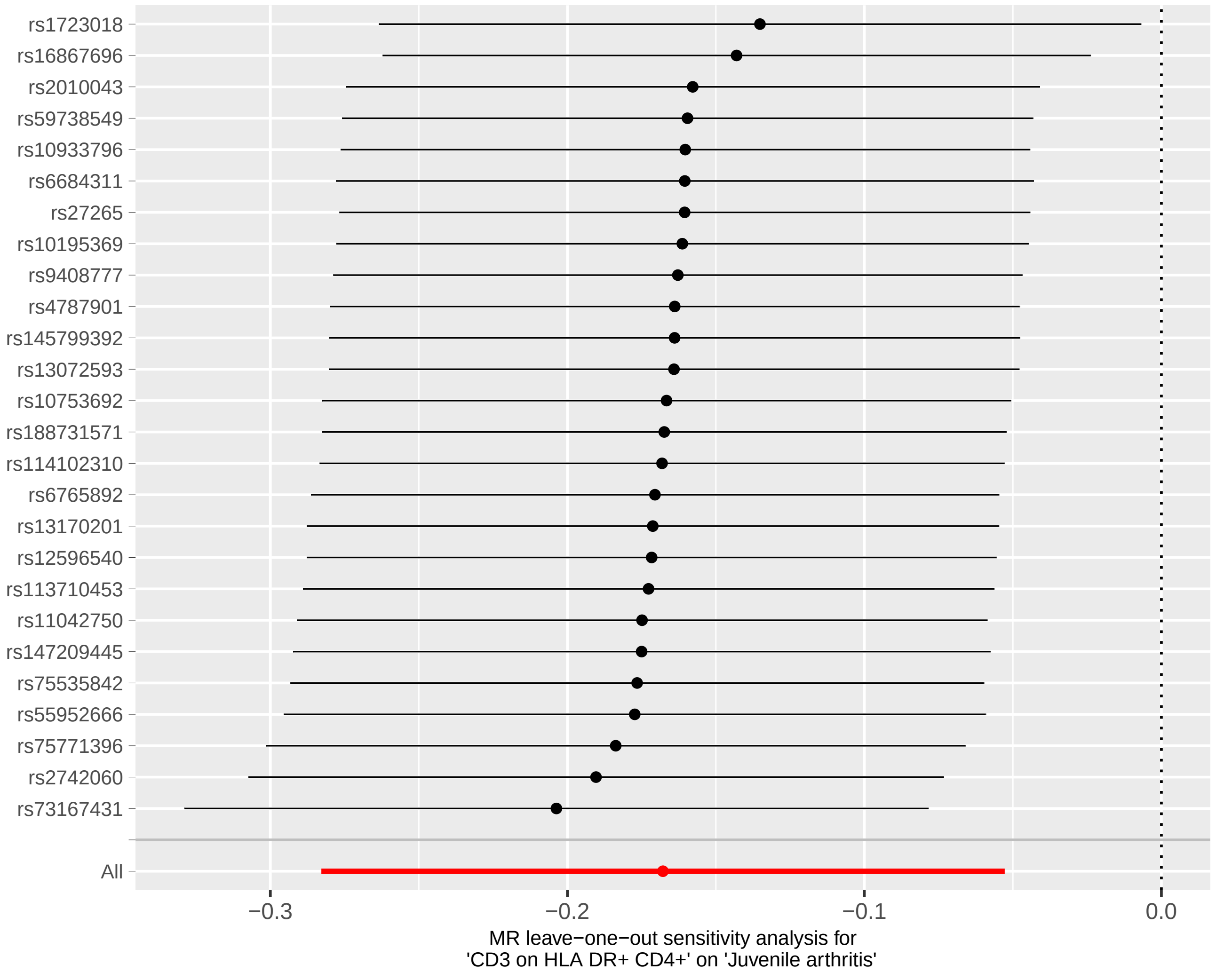
rs1723018
rs16867696
rs2010043
rs59738549
rs10933796
rs6684311
rs27265
rs10195369
rs9408777
rs4787901
rs145799392
rs13072593
rs10753692
rs188731571
rs114102310
rs6765892
rs13170201
rs12596540
rs113710453
rs11042750
rs147209445
rs75535842
rs55952666
rs75771396
rs2742060
rs73167431
All
−0.3
−0.2
−0.1
0.0
MR leave−one−out sensitivity analysis for
'CD3 on HLA DR+ CD4+' on 'Juvenile arthritis'

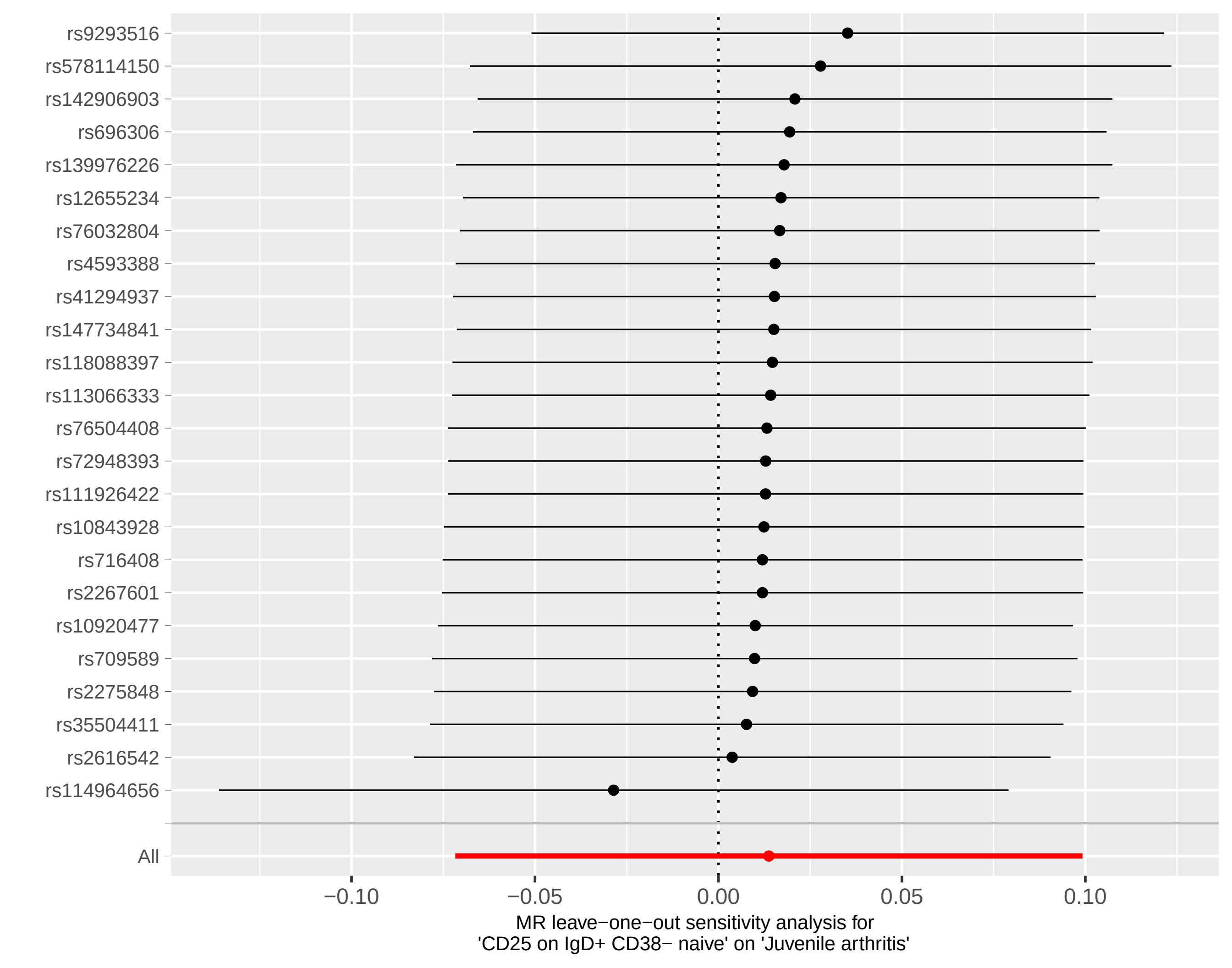
rs9293516
rs578114150
rs142906903
rs696306
rs139976226
rs12655234
rs76032804
rs4593388
rs41294937
rs147734841
rs118088397
rs113066333
rs76504408
rs72948393
rs111926422
rs10843928
rs716408
rs2267601
rs10920477
rs709589
rs2275848
rs35504411
rs2616542
rs114964656
All
−0.10
−0.05
0.00
0.05
0.10
MR leave−one−out sensitivity analysis for
'CD25 on IgD+ CD38− naive' on 'Juvenile arthritis'

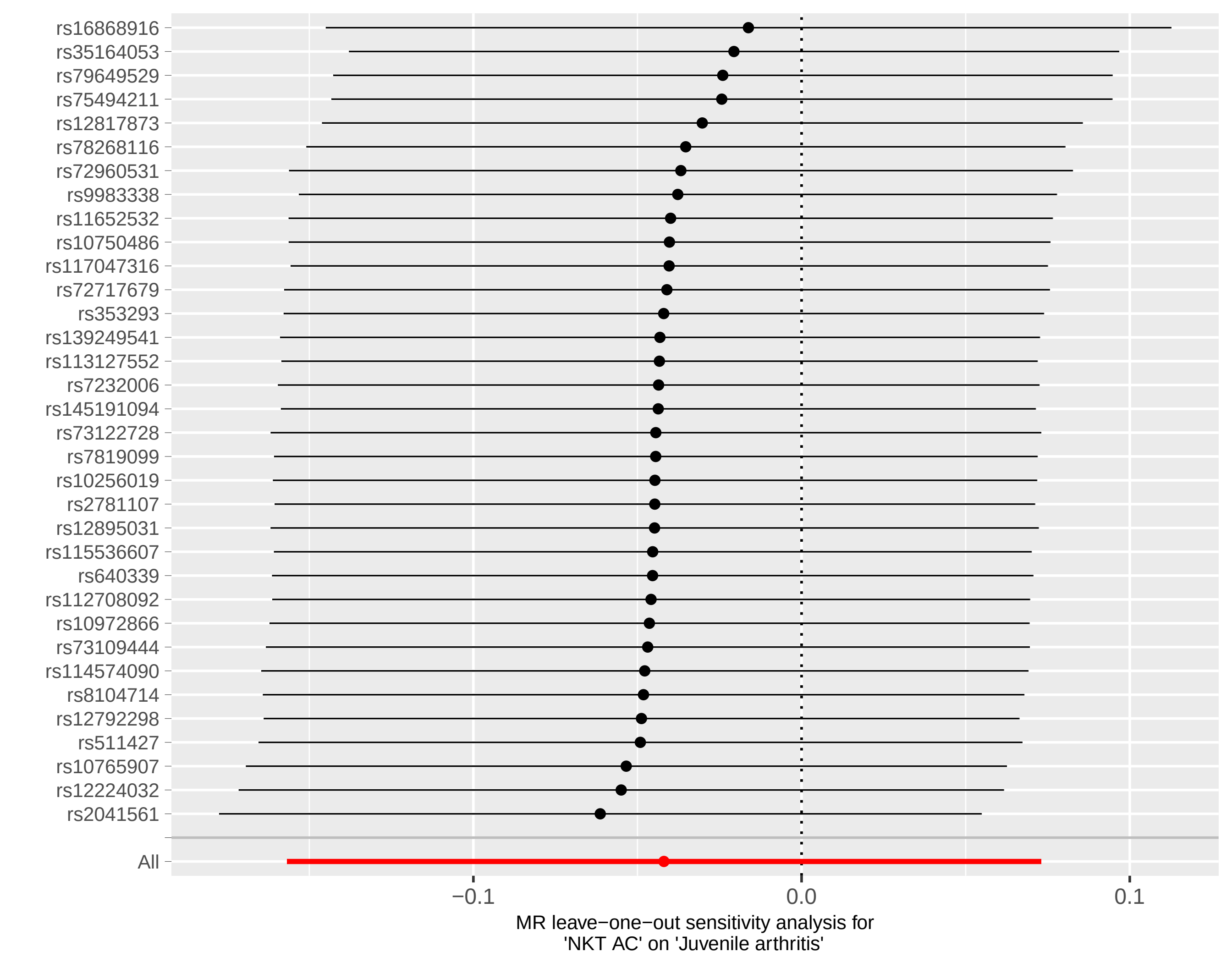

rs16868916
rs35164053
rs79649529
rs75494211
rs12817873
rs78268116
rs72960531
rs9983338
rs11652532
rs10750486
rs117047316
rs72717679
rs353293
rs139249541
rs113127552
rs7232006
rs145191094
rs73122728
rs7819099
rs10256019
rs2781107
rs12895031
rs115536607
rs640339
rs112708092
rs10972866
rs73109444
rs114574090
rs8104714
rs12792298
rs511427
rs10765907
rs12224032
rs2041561
All
−0.1
0.0
0.1
MR leave−one−out sensitivity analysis for
'NKT AC' on 'Juvenile arthritis'

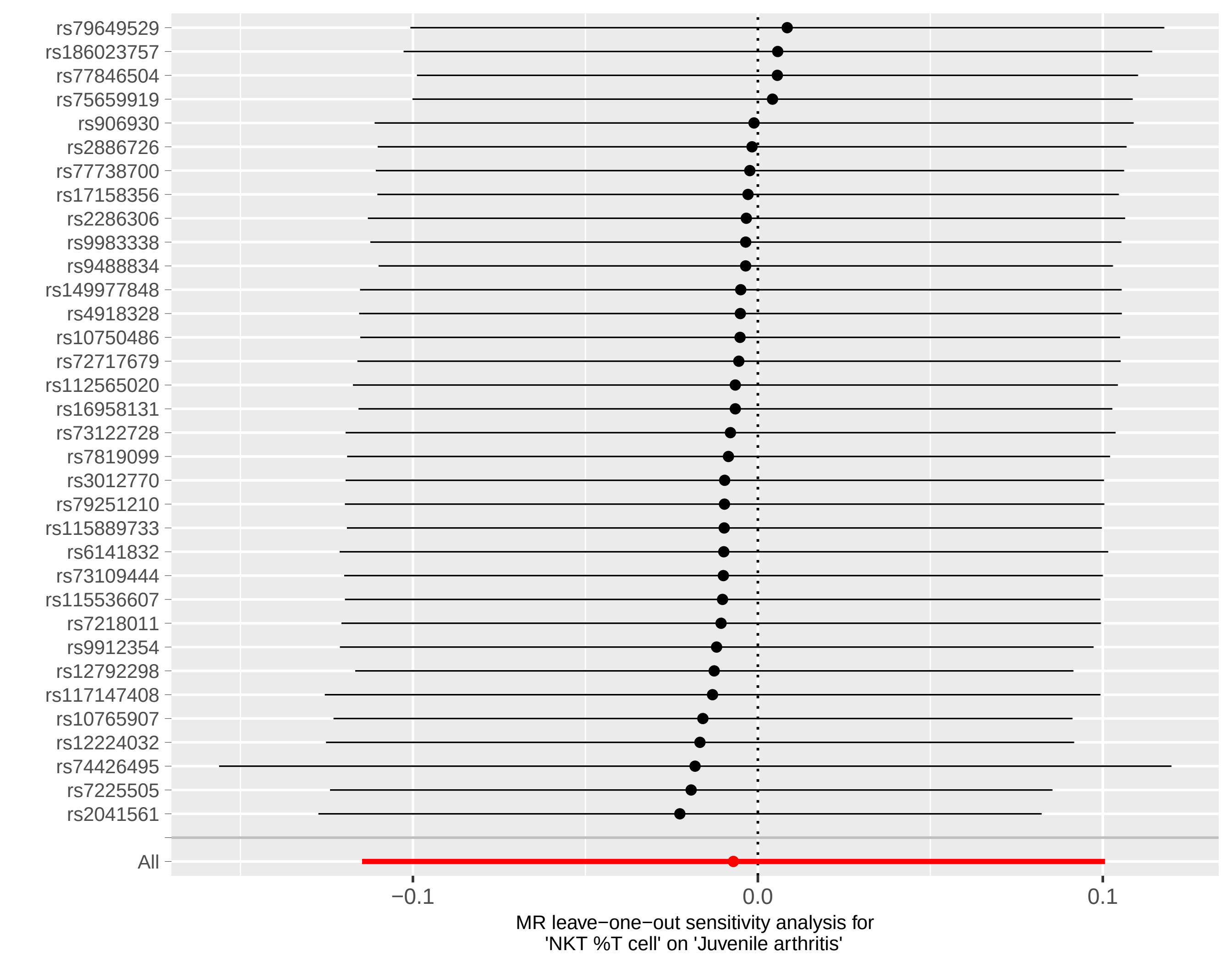
rs79649529
rs186023757
rs77846504
rs75659919
rs906930
rs2886726
rs77738700
rs17158356
rs2286306
rs9983338
rs9488834
rs149977848
rs4918328
rs10750486
rs72717679
rs112565020
rs16958131
rs73122728
rs7819099
rs3012770
rs79251210
rs115889733
rs6141832
rs73109444
rs115536607
rs7218011
rs9912354
rs12792298
rs117147408
rs10765907
rs12224032
rs74426495
rs7225505
rs2041561
All
−0.1
0.0
0.1
MR leave−one−out sensitivity analysis for
'NKT %T cell' on 'Juvenile arthritis'

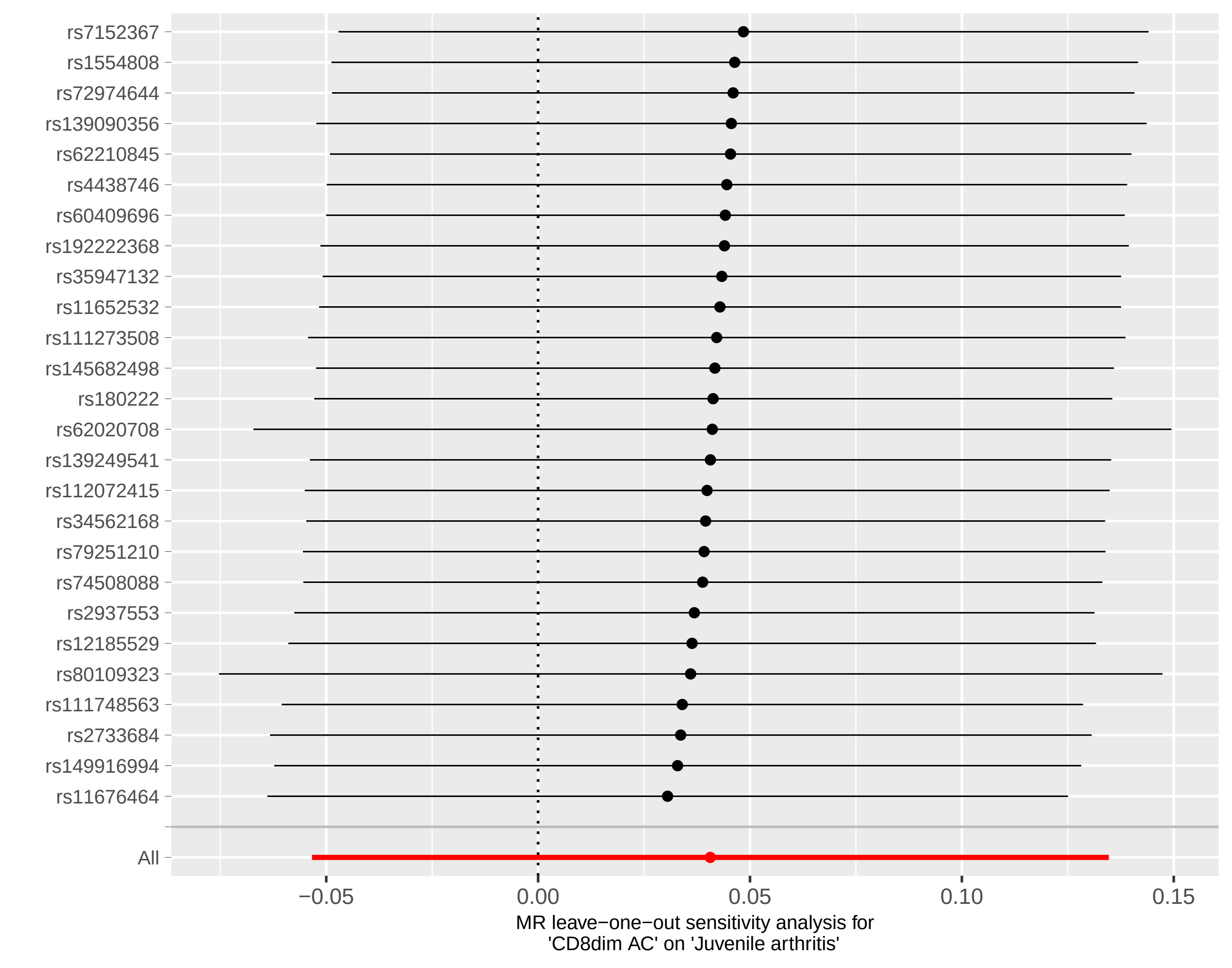
rs7152367
rs1554808
rs72974644
rs139090356
rs62210845
rs4438746
rs60409696
rs192222368
rs35947132
rs11652532
rs111273508
rs145682498
rs180222
rs62020708
rs139249541
rs112072415
rs34562168
rs79251210
rs74508088
rs2937553
rs12185529
rs80109323
rs111748563
rs2733684
rs149916994
rs11676464
All
−0.05
0.00
0.05
0.10
0.15
MR leave−one−out sensitivity analysis for
'CD8dim AC' on 'Juvenile arthritis'

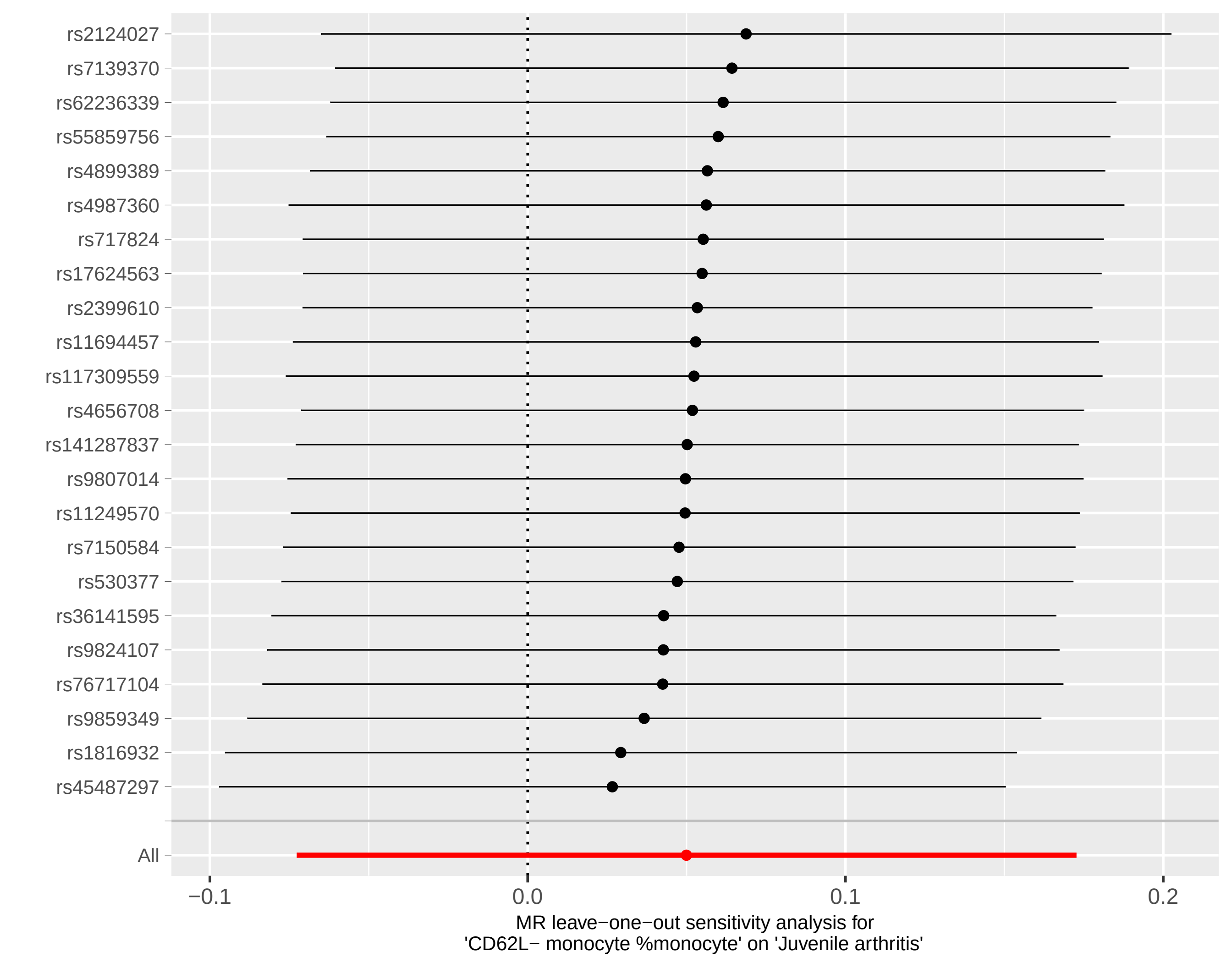

rs2124027
rs7139370
rs62236339
rs55859756
rs4899389
rs4987360
rs717824
rs17624563
rs2399610
rs11694457
rs117309559
rs4656708
rs141287837
rs9807014
rs11249570
rs7150584
rs530377
rs36141595
rs9824107
rs76717104
rs9859349
rs1816932
rs45487297
All
−0.1
0.0
0.1
0.2
MR leave−one−out sensitivity analysis for
'CD62L− monocyte %monocyte' on 'Juvenile arthritis'

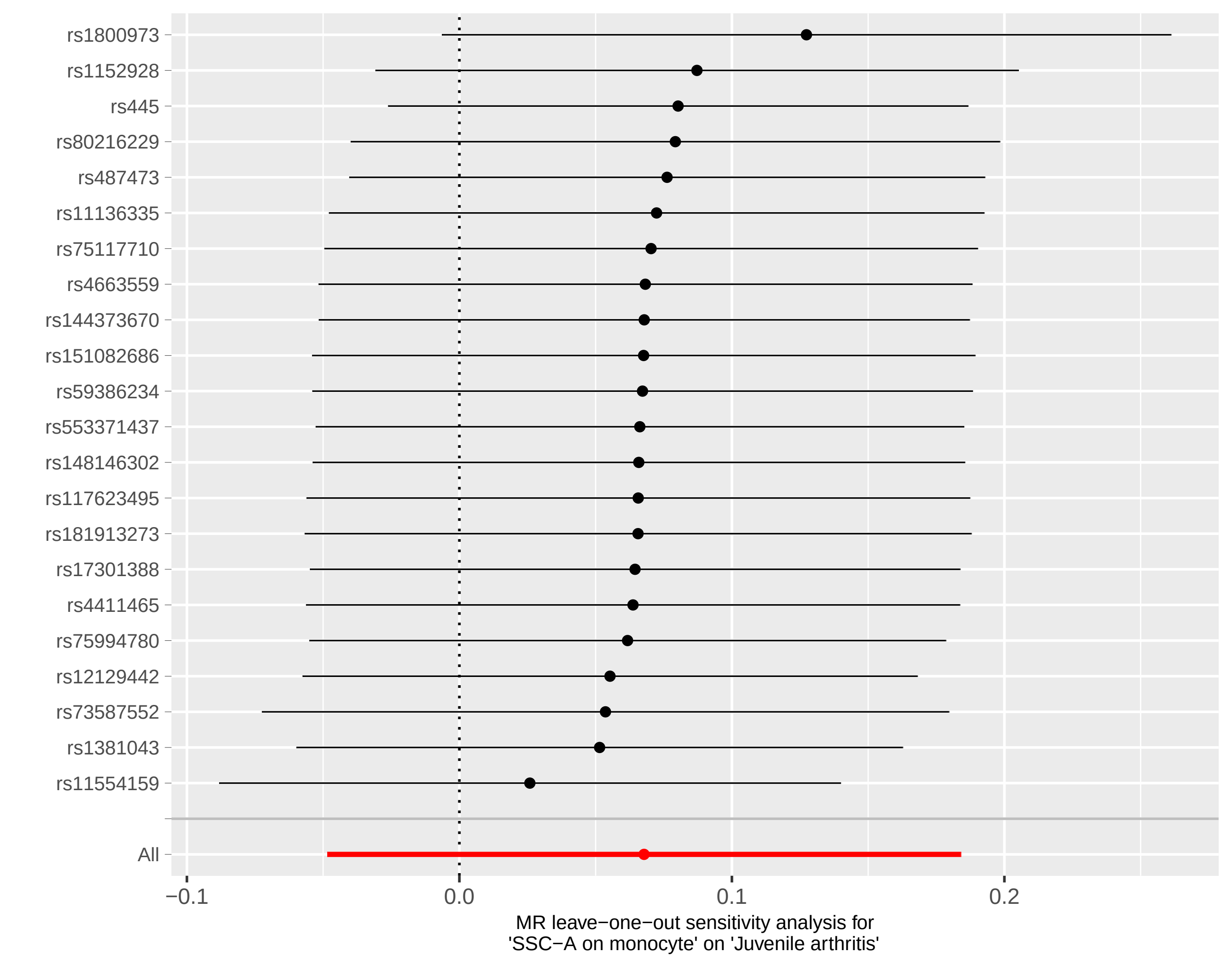

rs1800973
rs1152928
rs445
rs80216229
rs487473
rs11136335
rs75117710
rs4663559
rs144373670
rs151082686
rs59386234
rs553371437
rs148146302
rs117623495
rs181913273
rs17301388
rs4411465
rs75994780
rs12129442
rs73587552
rs1381043
rs11554159
All
−0.1
0.0
0.1
0.2
MR leave−one−out sensitivity analysis for
'SSC−A on monocyte' on 'Juvenile arthritis'

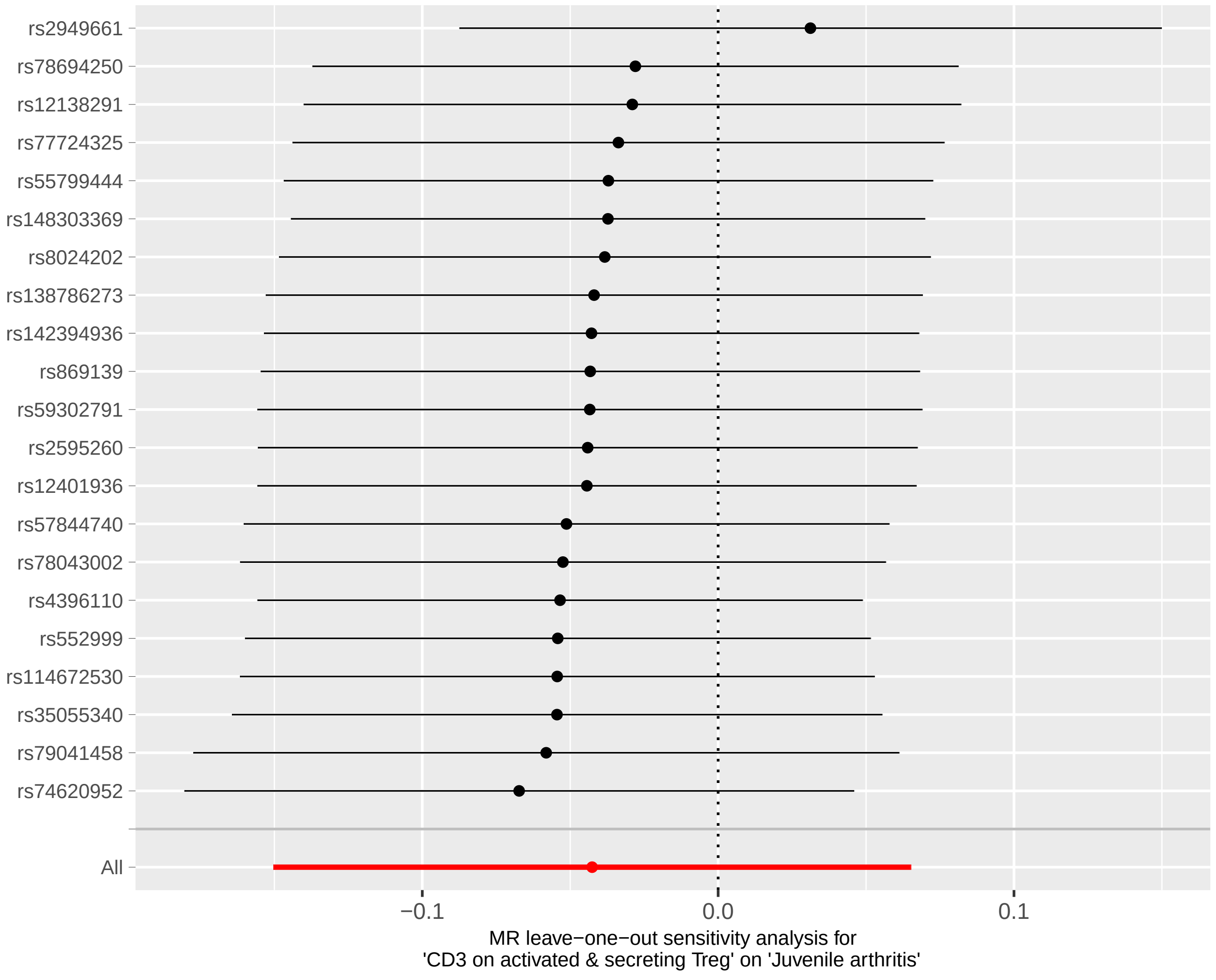
rs2949661
rs78694250
rs12138291
rs77724325
rs55799444
rs148303369
rs8024202
rs138786273
rs142394936
rs869139
rs59302791
rs2595260
rs12401936
rs57844740
rs78043002
rs4396110
rs552999
rs114672530
rs35055340
rs79041458
rs74620952
All
−0.1
0.0
0.1
MR leave−one−out sensitivity analysis for
'CD3 on activated & secreting Treg' on 'Juvenile arthritis'

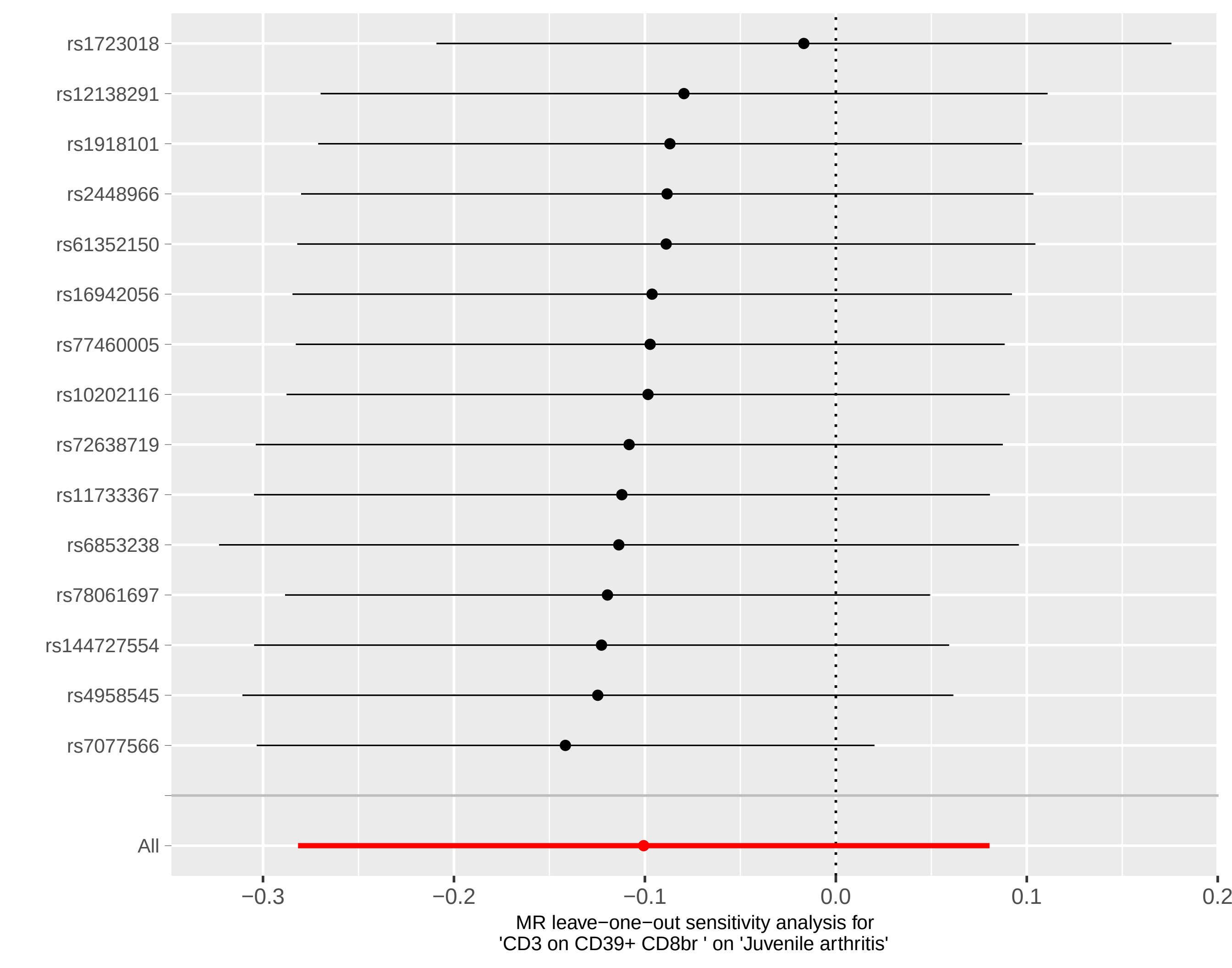

rs1723018
rs12138291
rs1918101
rs2448966
rs61352150
rs16942056
rs77460005
rs10202116
rs72638719
rs11733367
rs6853238
rs78061697
rs144727554
rs4958545
rs7077566
All
−0.3
−0.2
−0.1
0.0
0.1
0.2
MR leave−one−out sensitivity analysis for
'CD3 on CD39+ CD8br ' on 'Juvenile arthritis'

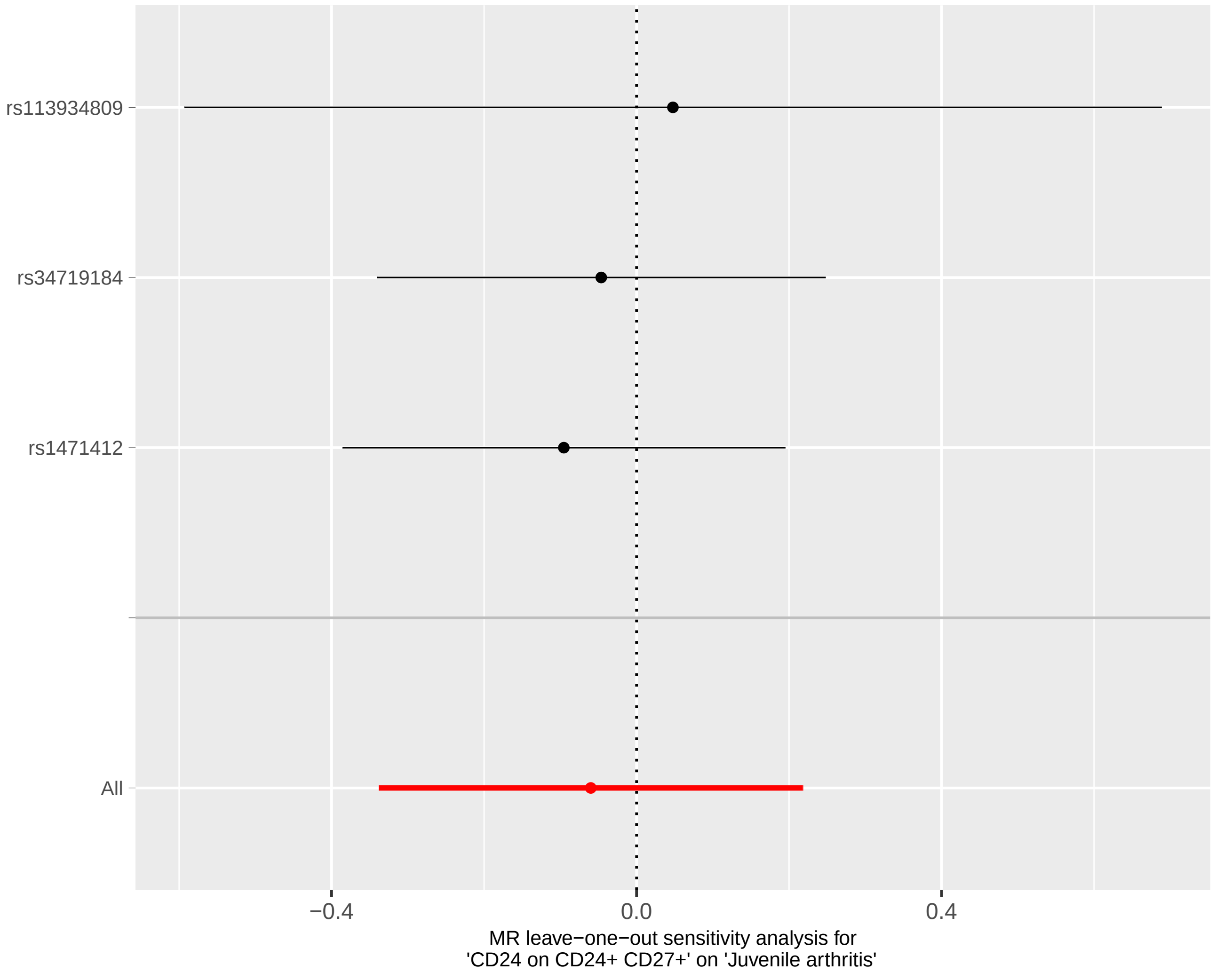
rs113934809
rs34719184
rs1471412
All
−0.4
0.0
0.4
MR leave−one−out sensitivity analysis for
'CD24 on CD24+ CD27+' on 'Juvenile arthritis'

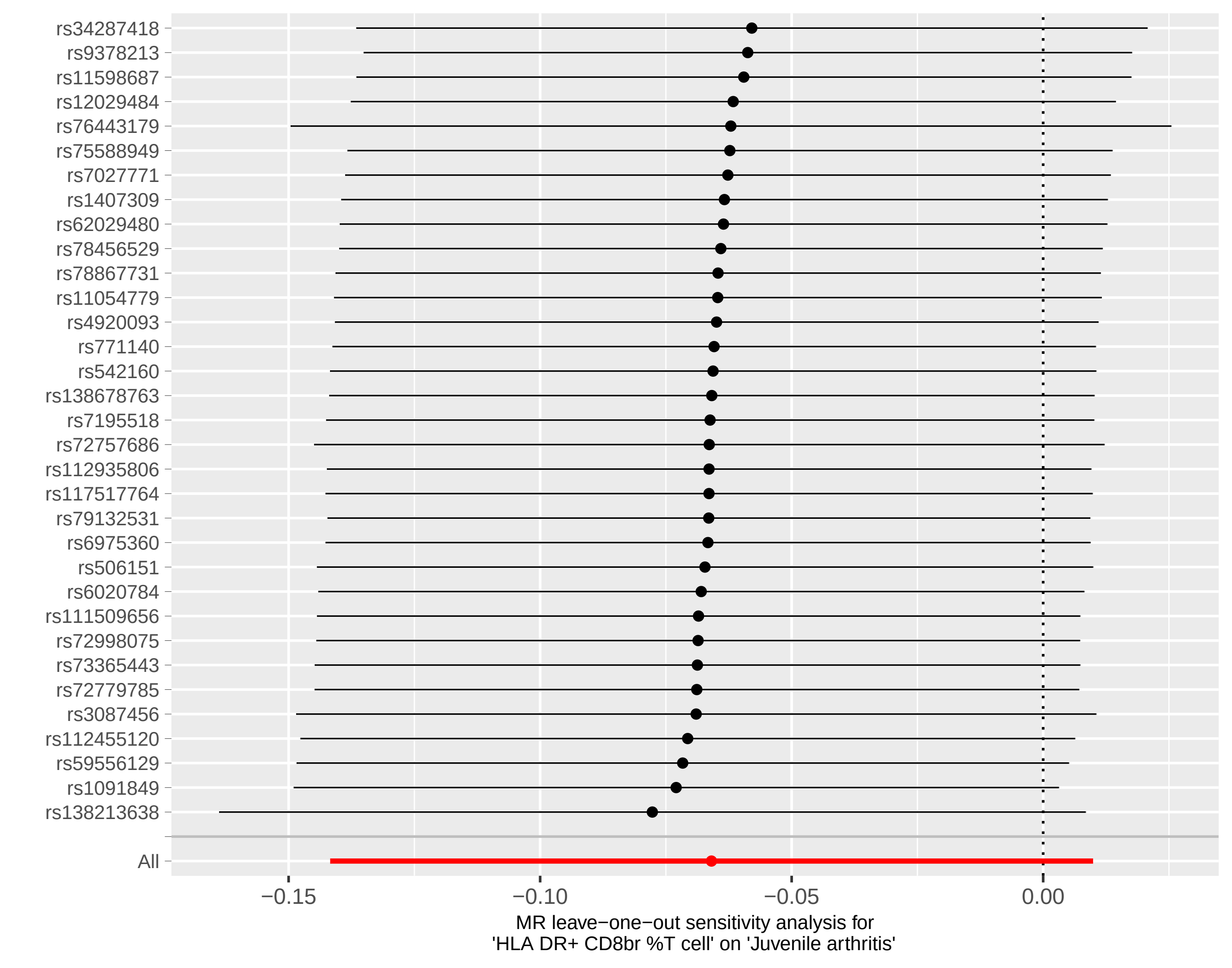
rs34287418
rs9378213
rs11598687
rs12029484
rs76443179
rs75588949
rs7027771
rs1407309
rs62029480
rs78456529
rs78867731
rs11054779
rs4920093
rs771140
rs542160
rs138678763
rs7195518
rs72757686
rs112935806
rs117517764
rs79132531
rs6975360
rs506151
rs6020784
rs111509656
rs72998075
rs73365443
rs72779785
rs3087456
rs112455120
rs59556129
rs1091849
rs138213638
All
−0.15
−0.10
−0.05
0.00
MR leave−one−out sensitivity analysis for
'HLA DR+ CD8br %T cell' on 'Juvenile arthritis'

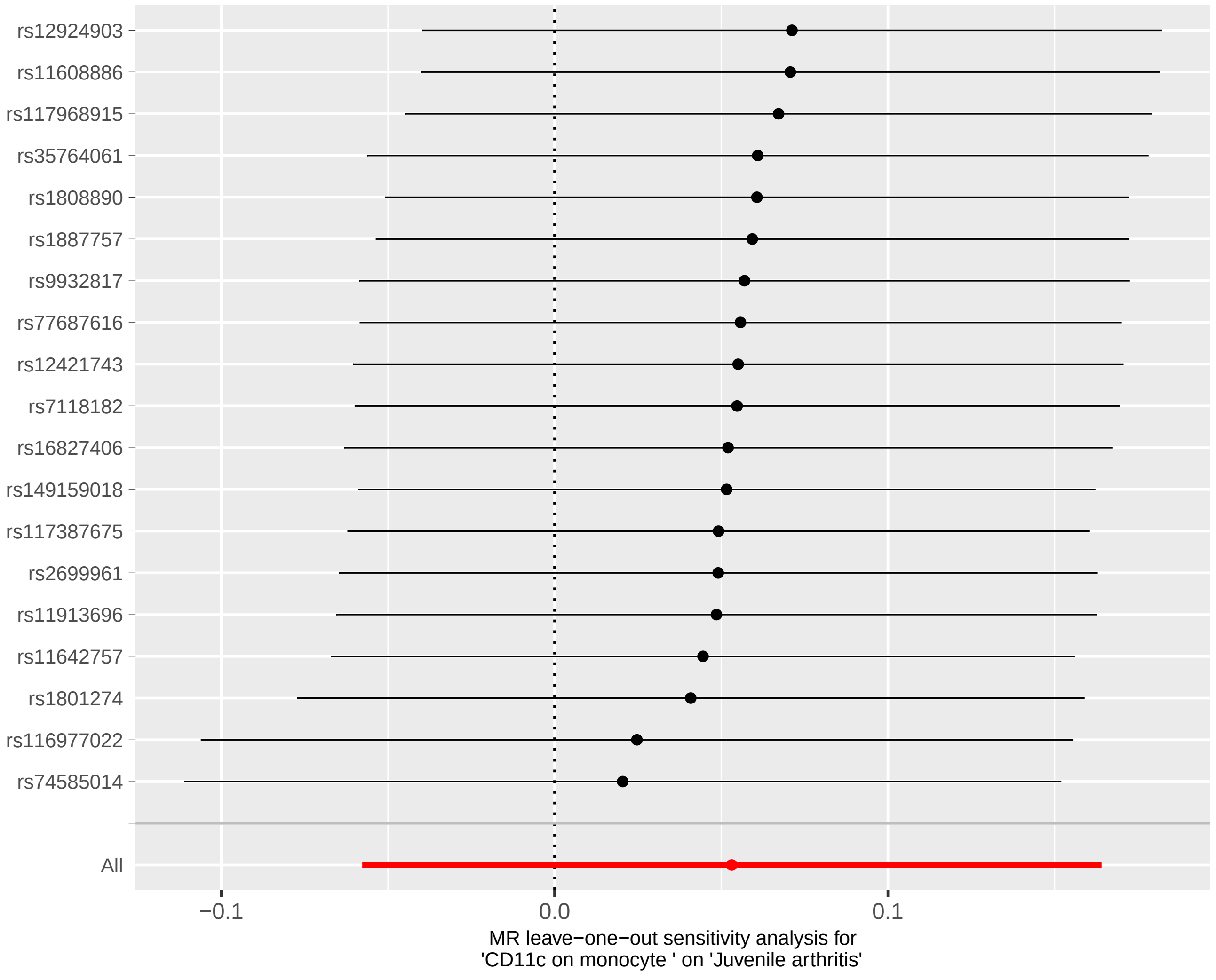
rs12924903
rs11608886
rs117968915
rs35764061
rs1808890
rs1887757
rs9932817
rs77687616
rs12421743
rs7118182
rs16827406
rs149159018
rs117387675
rs2699961
rs11913696
rs11642757
rs1801274
rs116977022
rs74585014
All
−0.1
0.0
0.1
MR leave−one−out sensitivity analysis for
'CD11c on monocyte ' on 'Juvenile arthritis'

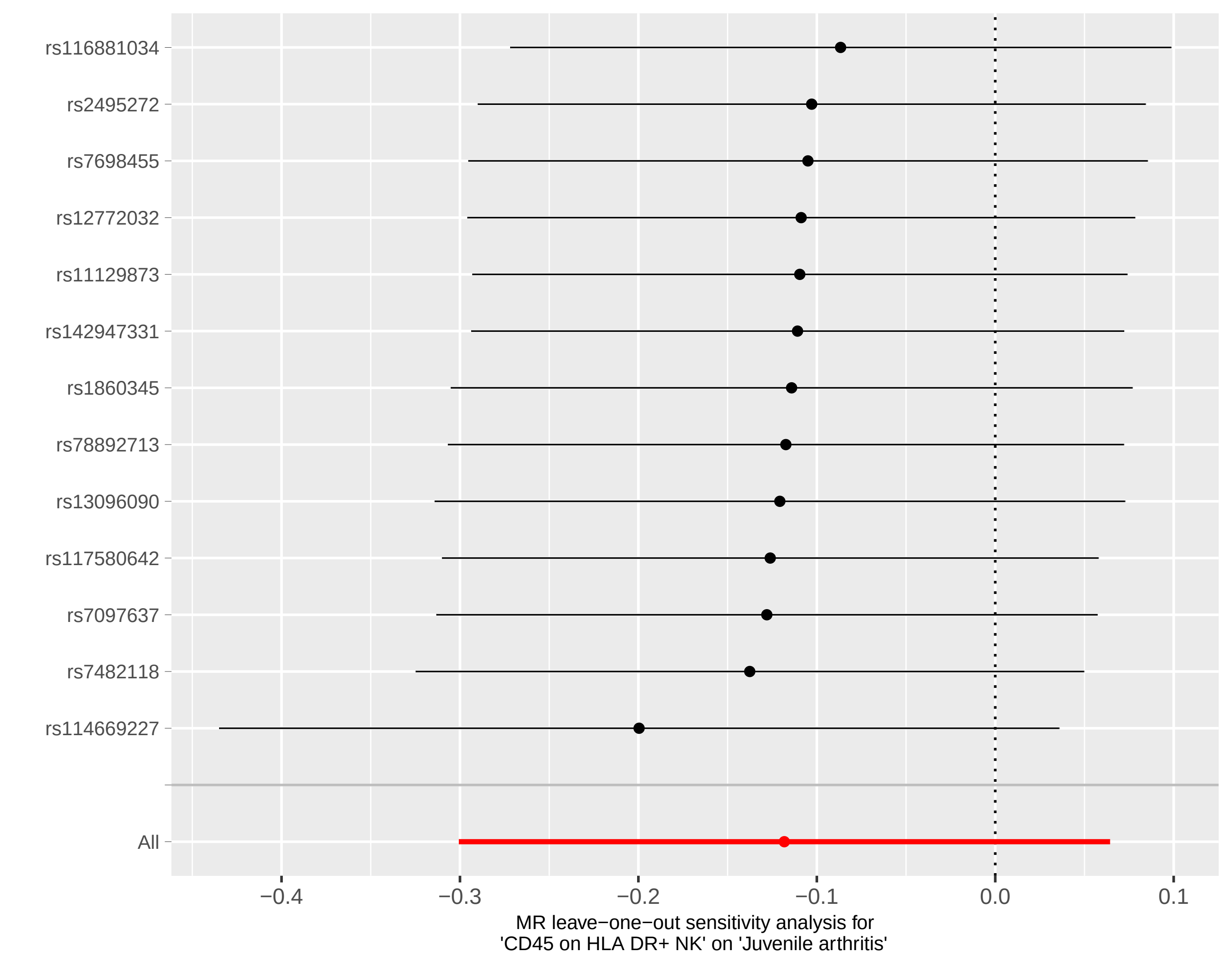

rs116881034
rs2495272
rs7698455
rs12772032
rs11129873
rs142947331
rs1860345
rs78892713
rs13096090
rs117580642
rs7097637
rs7482118
rs114669227
All
−0.4
−0.3
−0.2
−0.1
0.0
0.1
MR leave−one−out sensitivity analysis for
'CD45 on HLA DR+ NK' on 'Juvenile arthritis'

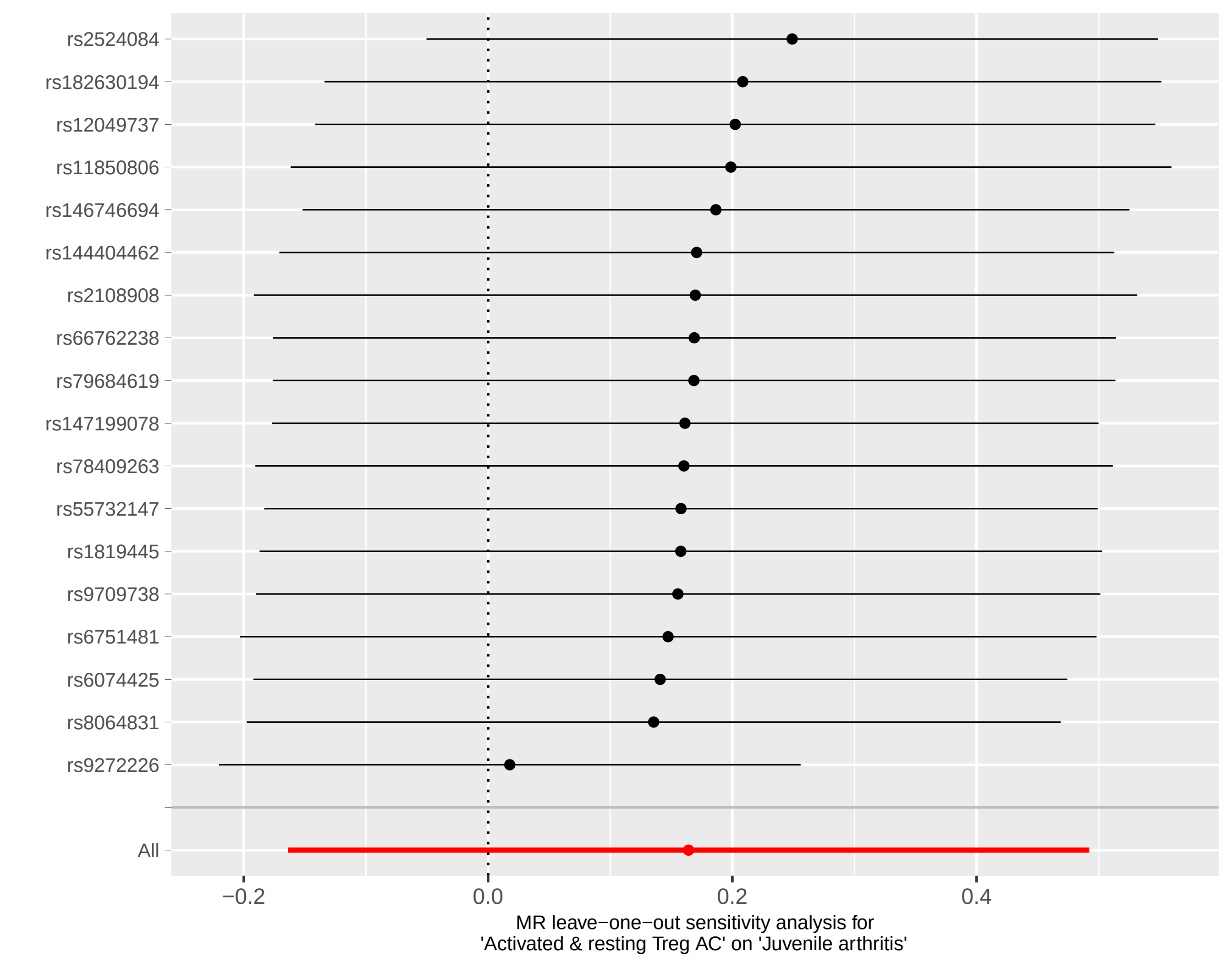
rs2524084
rs182630194
rs12049737
rs11850806
rs146746694
rs144404462
rs2108908
rs66762238
rs79684619
rs147199078
rs78409263
rs55732147
rs1819445
rs9709738
rs6751481
rs6074425
rs8064831
rs9272226
All
−0.2
0.0
0.2
0.4
MR leave−one−out sensitivity analysis for
'Activated & resting Treg AC' on 'Juvenile arthritis'

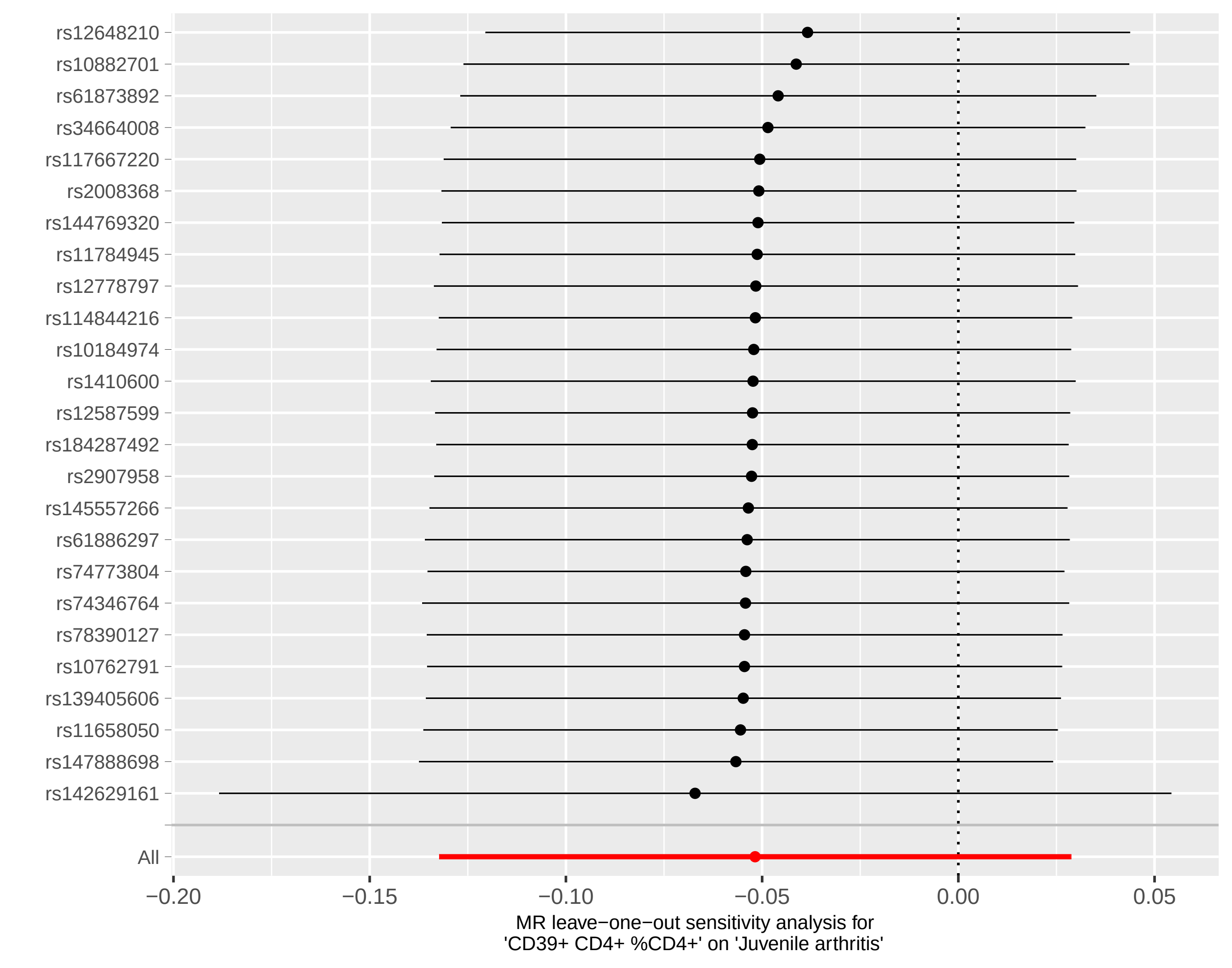
rs12648210
rs10882701
rs61873892
rs34664008
rs117667220
rs2008368
rs144769320
rs11784945
rs12778797
rs114844216
rs10184974
rs1410600
rs12587599
rs184287492
rs2907958
rs145557266
rs61886297
rs74773804
rs74346764
rs78390127
rs10762791
rs139405606
rs11658050
rs147888698
rs142629161
All
−0.20
−0.15
−0.10
−0.05
0.00
0.05
MR leave−one−out sensitivity analysis for
'CD39+ CD4+ %CD4+' on 'Juvenile arthritis'

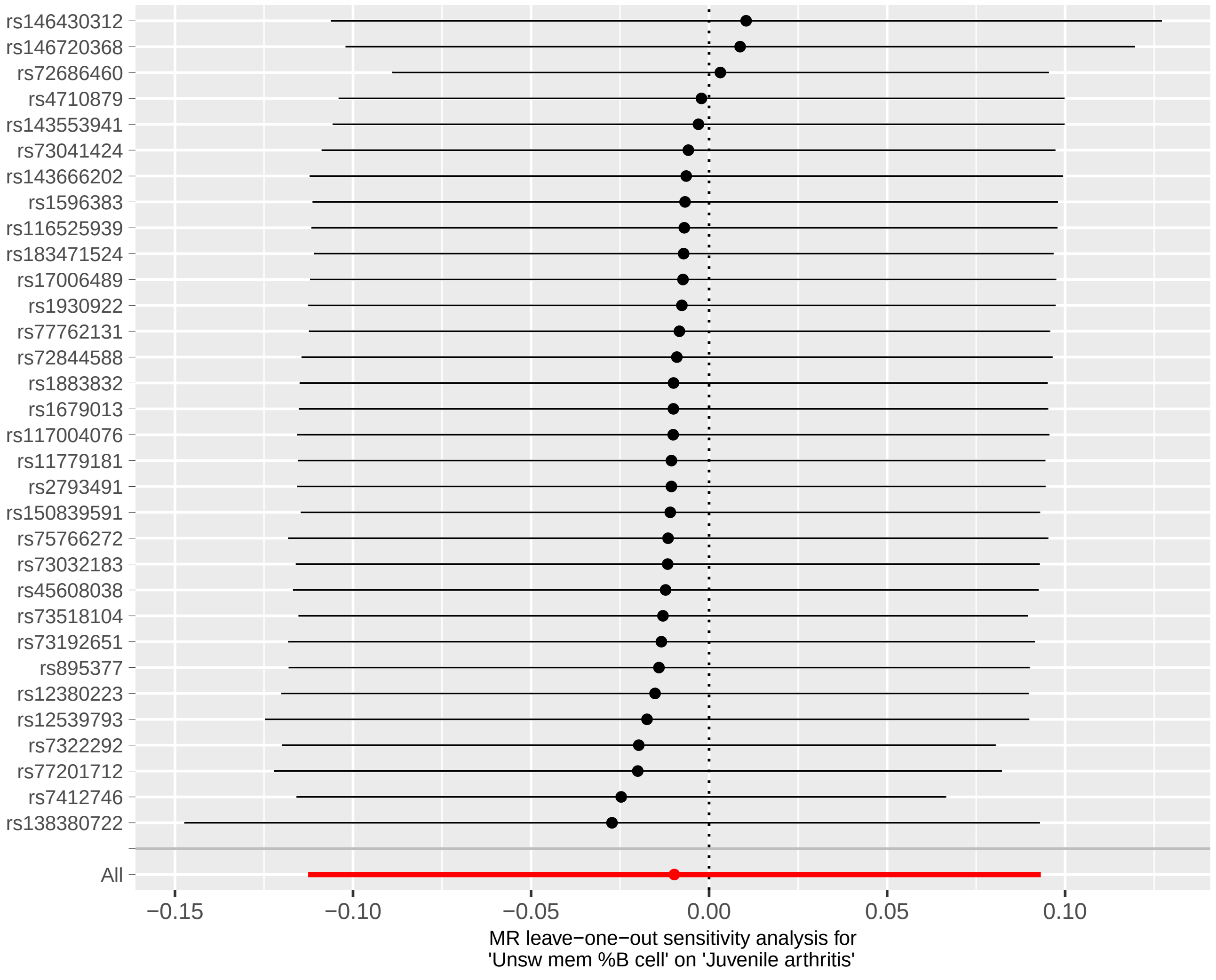

rs146430312
rs146720368
rs72686460
rs4710879
rs143553941
rs73041424
rs143666202
rs1596383
rs116525939
rs183471524
rs17006489
rs1930922
rs77762131
rs72844588
rs1883832
rs1679013
rs117004076
rs11779181
rs2793491
rs150839591
rs75766272
rs73032183
rs45608038
rs73518104
rs73192651
rs895377
rs12380223
rs12539793
rs7322292
rs77201712
rs7412746
rs138380722
All
−0.15
−0.10
−0.05
0.00
0.05
0.10
MR leave−one−out sensitivity analysis for
'Unsw mem %B cell' on 'Juvenile arthritis'

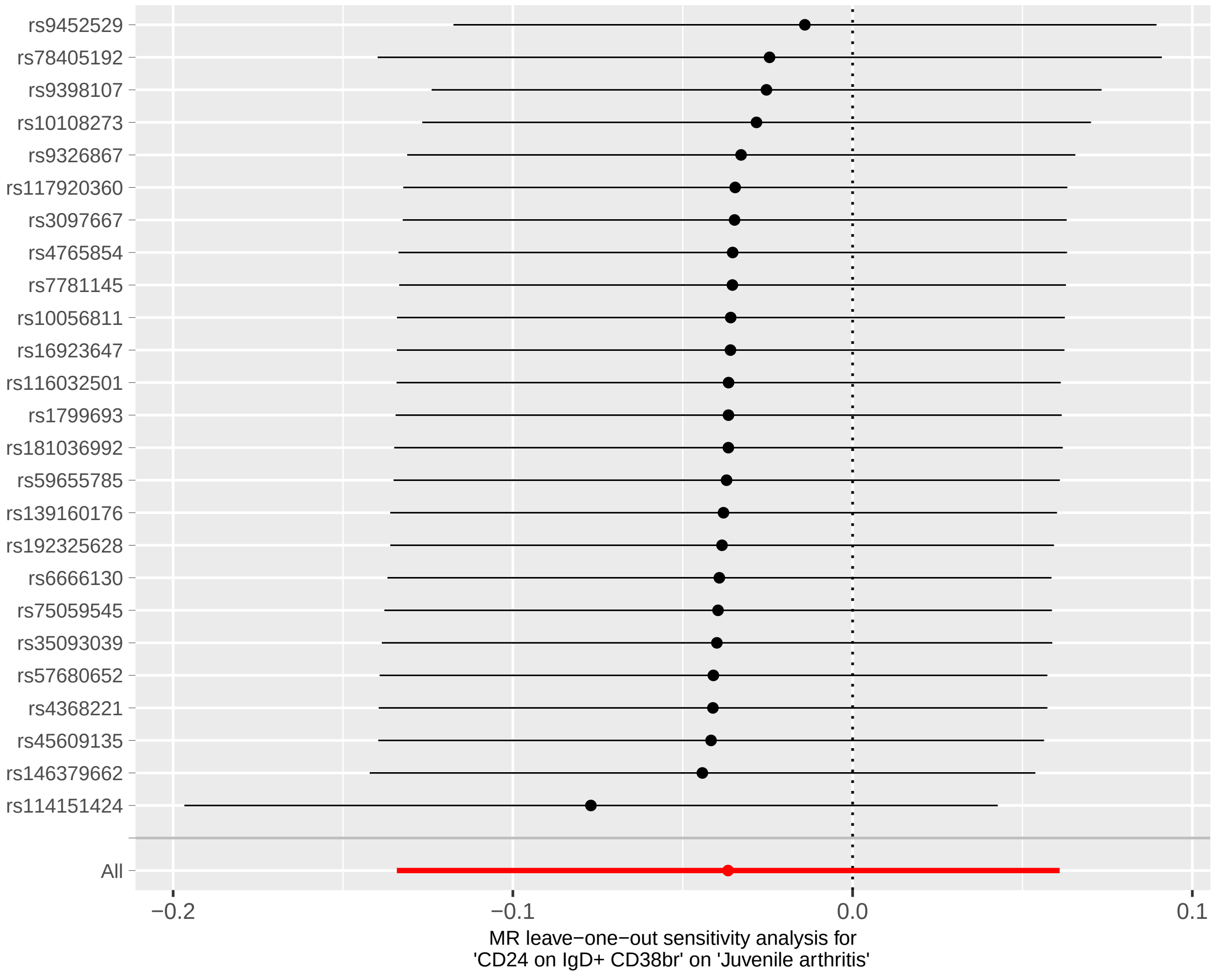
rs9452529
rs78405192
rs9398107
rs10108273
rs9326867
rs117920360
rs3097667
rs4765854
rs7781145
rs10056811
rs16923647
rs116032501
rs1799693
rs181036992
rs59655785
rs139160176
rs192325628
rs6666130
rs75059545
rs35093039
rs57680652
rs4368221
rs45609135
rs146379662
rs114151424
All
−0.2
−0.1
0.0
0.1
MR leave−one−out sensitivity analysis for
'CD24 on IgD+ CD38br' on 'Juvenile arthritis'

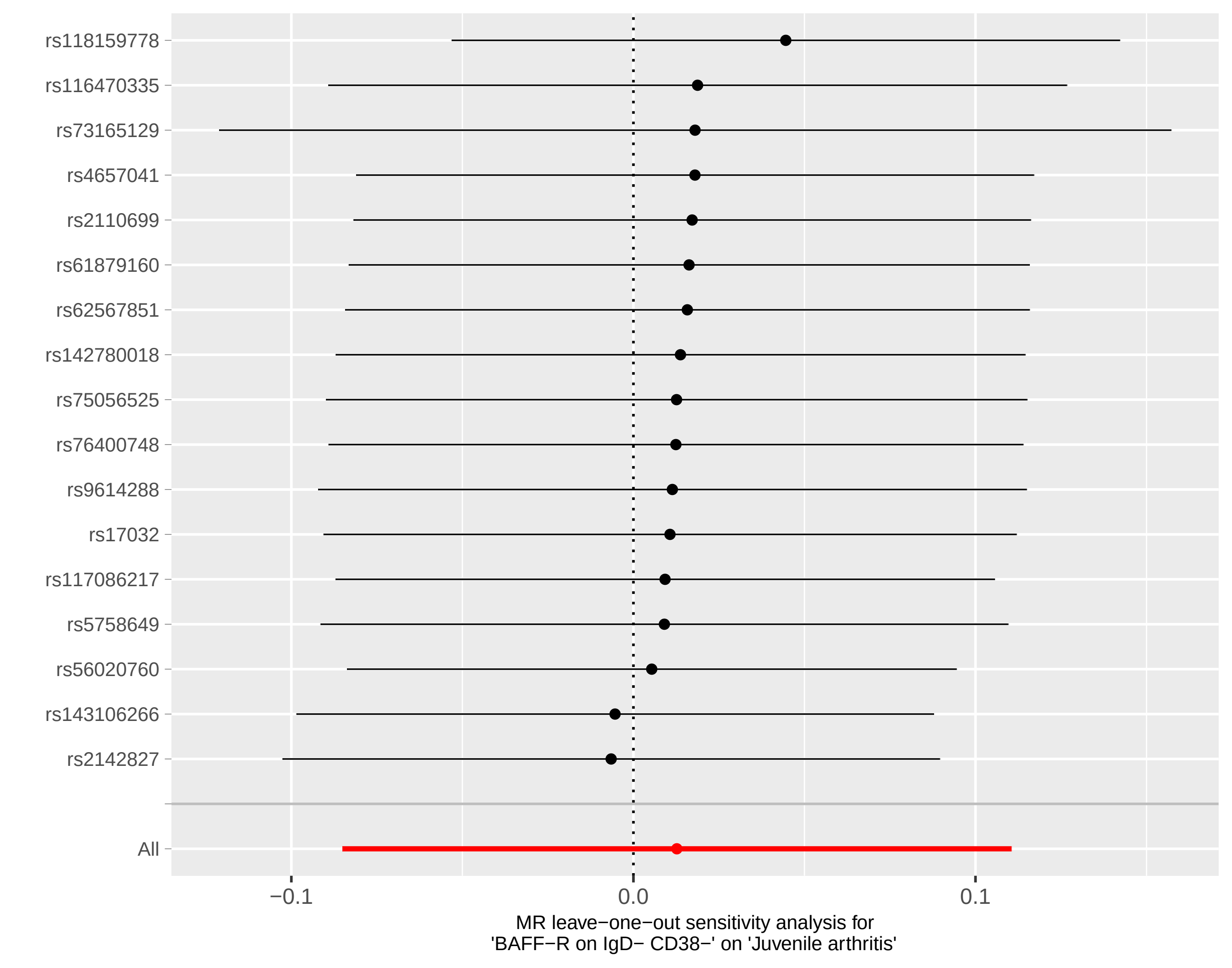
rs118159778
rs116470335
rs73165129
rs4657041
rs2110699
rs61879160
rs62567851
rs142780018
rs75056525
rs76400748
rs9614288
rs17032
rs117086217
rs5758649
rs56020760
rs143106266
rs2142827
All
−0.1
0.0
0.1
MR leave−one−out sensitivity analysis for
'BAFF−R on IgD− CD38−' on 'Juvenile arthritis'

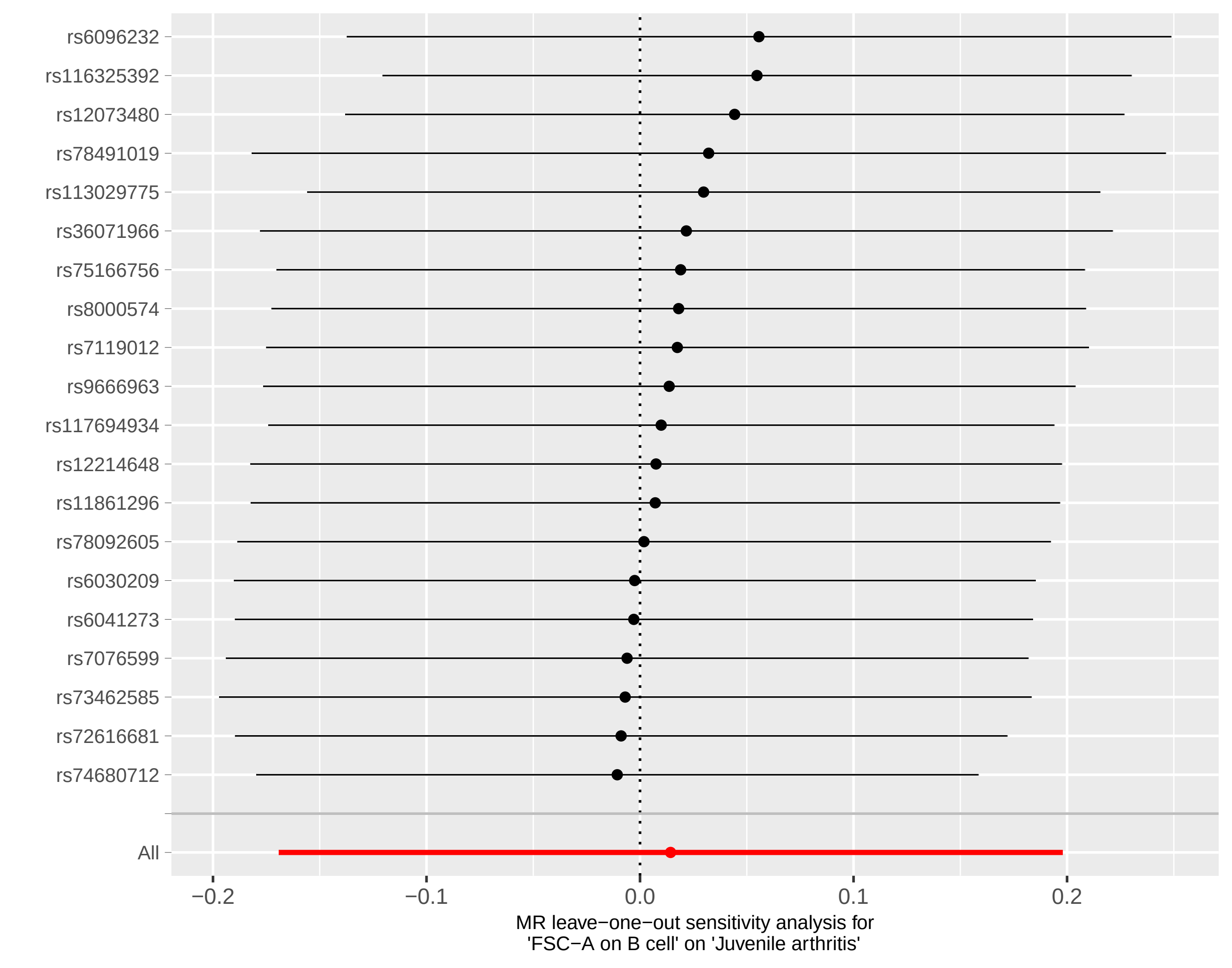

rs6096232
rs116325392
rs12073480
rs78491019
rs113029775
rs36071966
rs75166756
rs8000574
rs7119012
rs9666963
rs117694934
rs12214648
rs11861296
rs78092605
rs6030209
rs6041273
rs7076599
rs73462585
rs72616681
rs74680712
All
−0.2
−0.1
0.0
0.1
0.2
MR leave−one−out sensitivity analysis for
'FSC−A on B cell' on 'Juvenile arthritis'

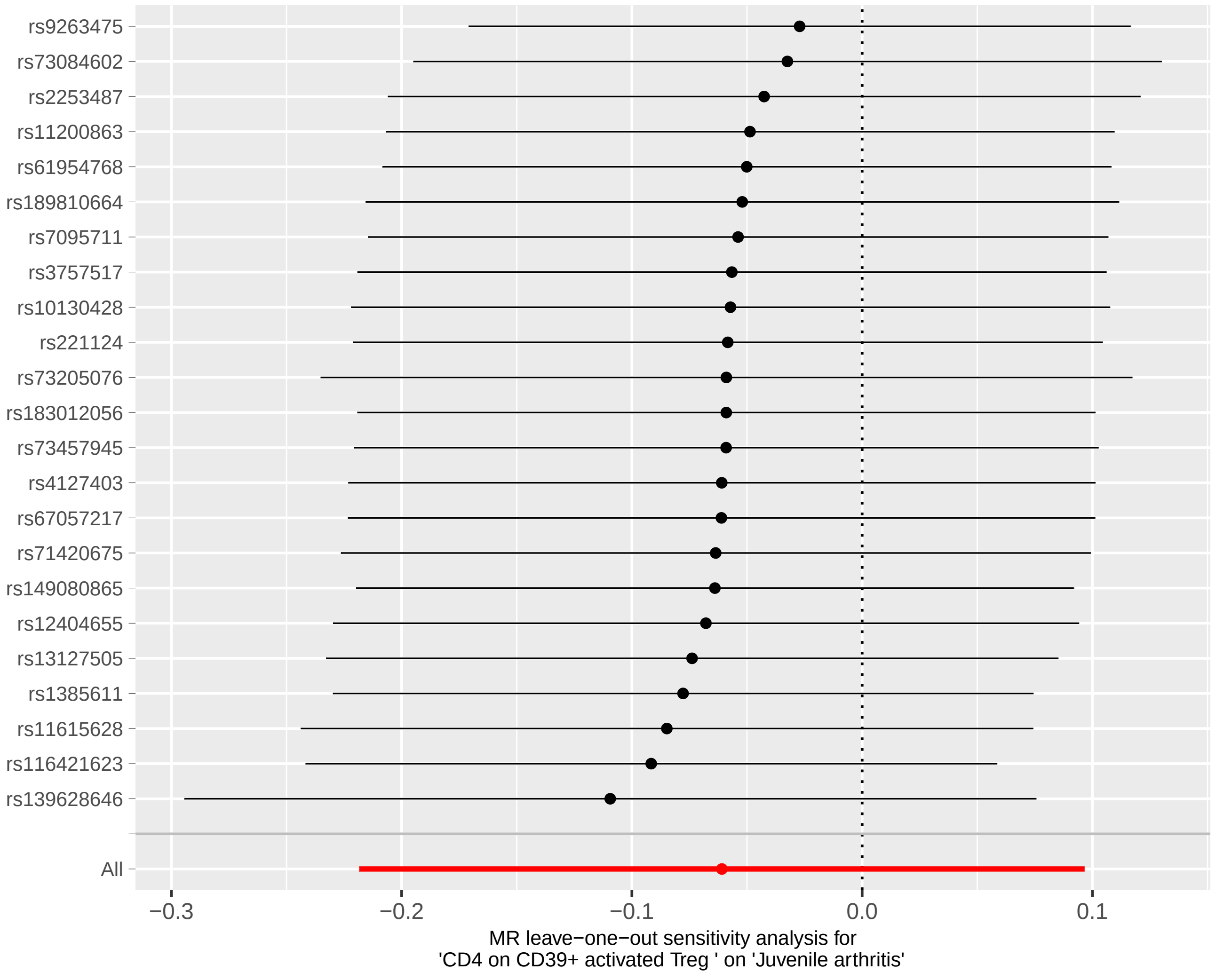
rs9263475
rs73084602
rs2253487
rs11200863
rs61954768
rs189810664
rs7095711
rs3757517
rs10130428
rs221124
rs73205076
rs183012056
rs73457945
rs4127403
rs67057217
rs71420675
rs149080865
rs12404655
rs13127505
rs1385611
rs11615628
rs116421623
rs139628646
All
−0.3
−0.2
−0.1
0.0
0.1
MR leave−one−out sensitivity analysis for
'CD4 on CD39+ activated Treg ' on 'Juvenile arthritis'

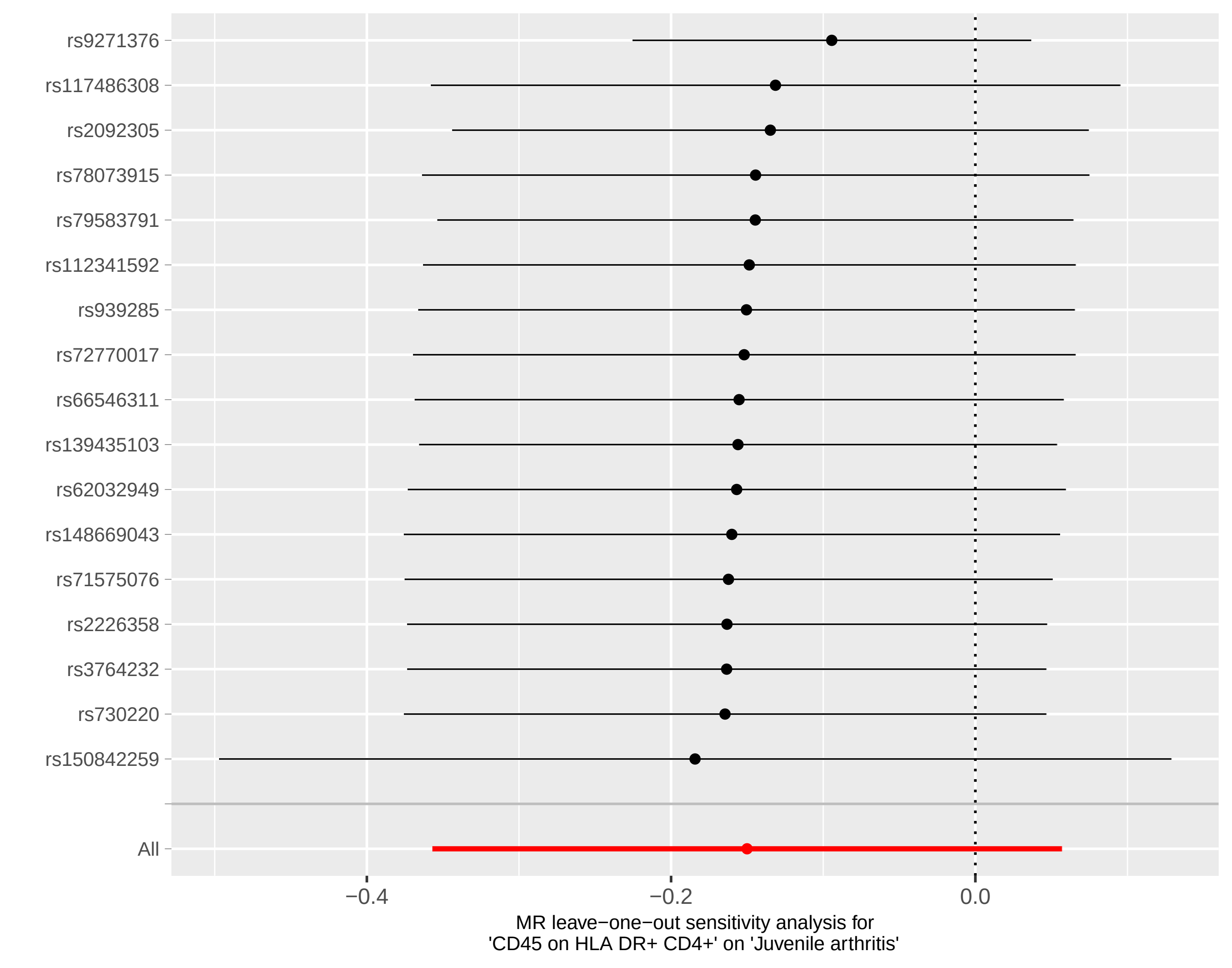

rs9271376
rs117486308
rs2092305
rs78073915
rs79583791
rs112341592
rs939285
rs72770017
rs66546311
rs139435103
rs62032949
rs148669043
rs71575076
rs2226358
rs3764232
rs730220
rs150842259
All
−0.4
−0.2
0.0
MR leave−one−out sensitivity analysis for
'CD45 on HLA DR+ CD4+' on 'Juvenile arthritis'

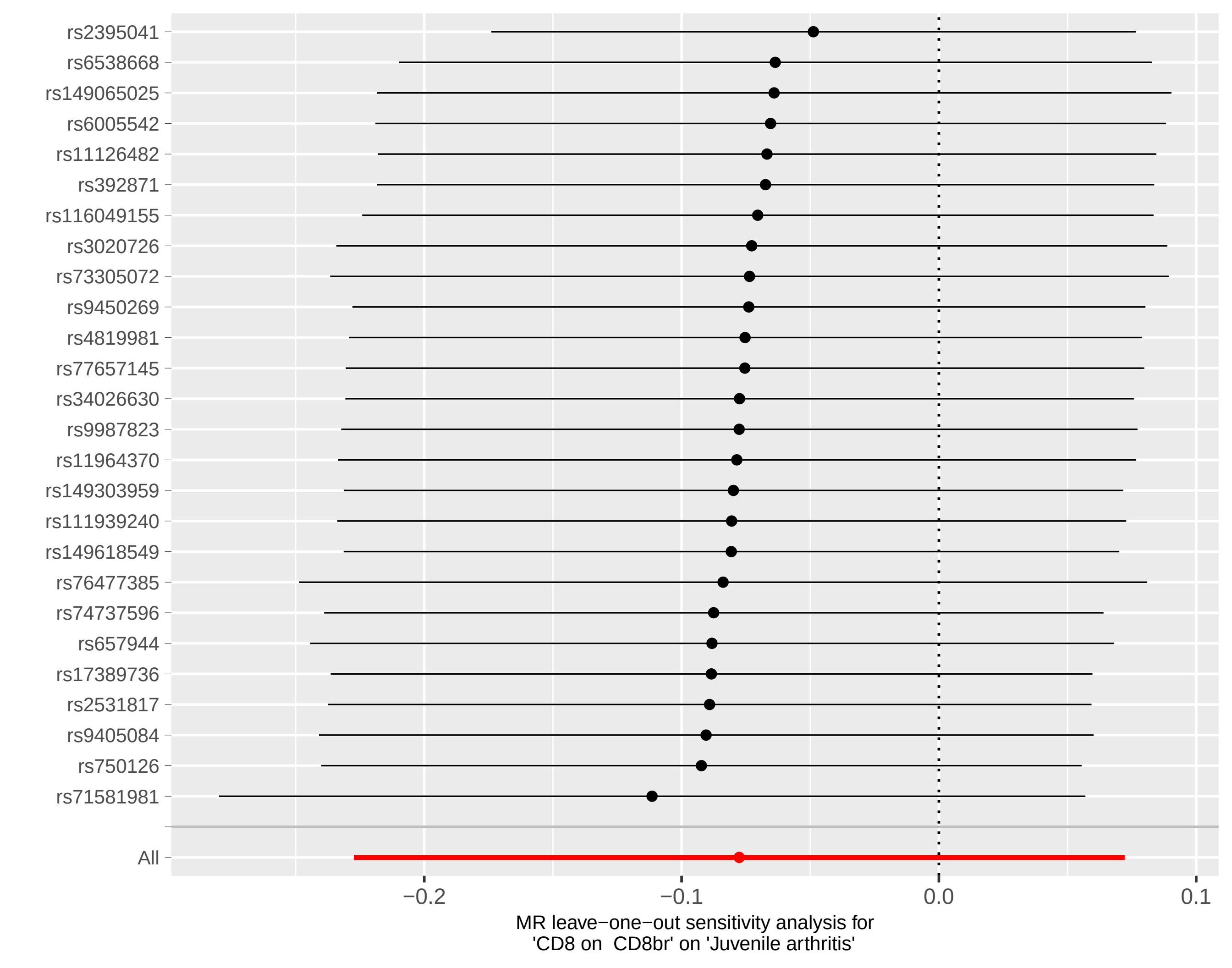
rs2395041
rs6538668
rs149065025
rs6005542
rs11126482
rs392871
rs116049155
rs3020726
rs73305072
rs9450269
rs4819981
rs77657145
rs34026630
rs9987823
rs11964370
rs149303959
rs111939240
rs149618549
rs76477385
rs74737596
rs657944
rs17389736
rs2531817
rs9405084
rs750126
rs71581981
All
−0.2
−0.1
0.0
0.1
MR leave−one−out sensitivity analysis for
'CD8 on CD8br' on 'Juvenile arthritis'

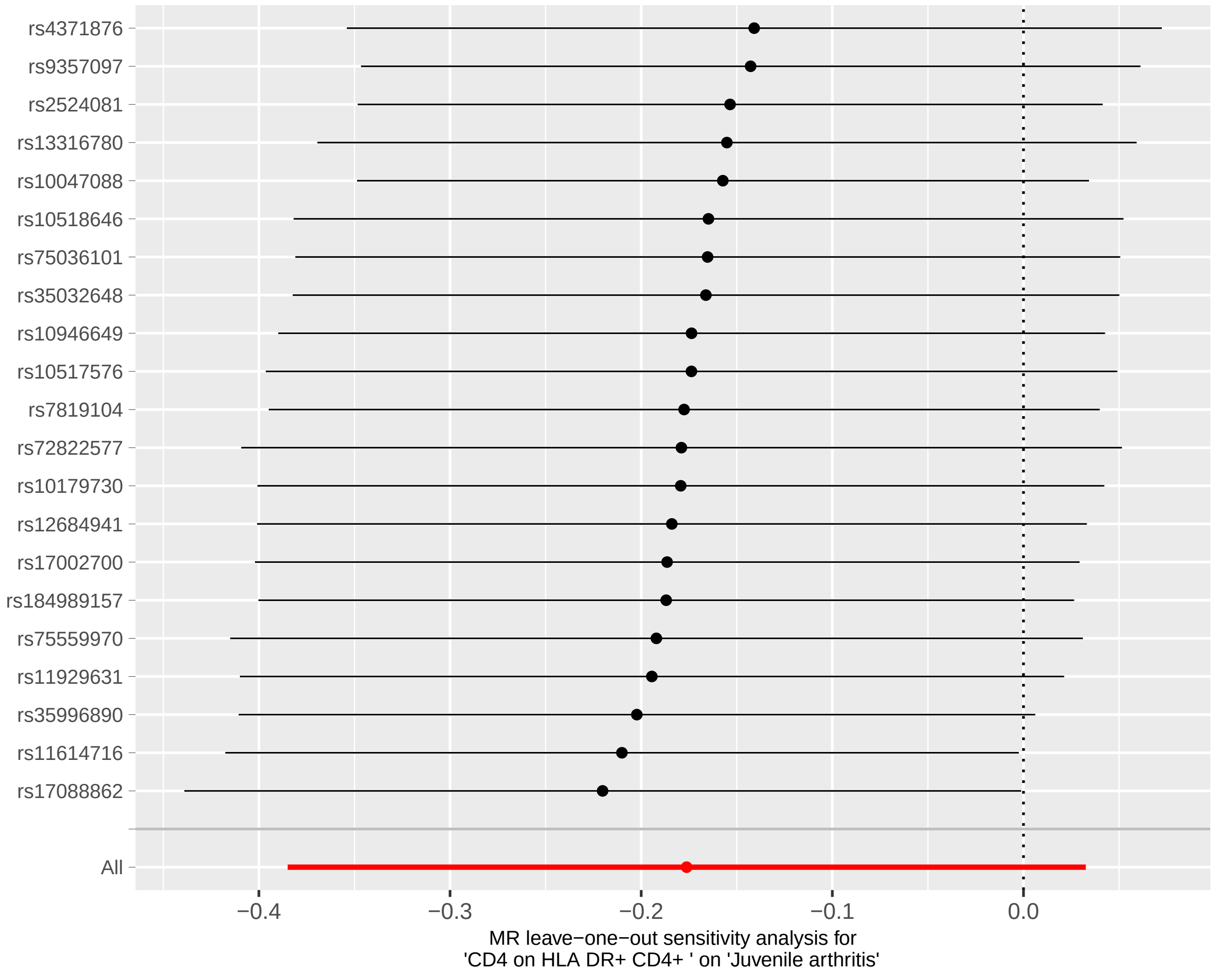
rs4371876
rs9357097
rs2524081
rs13316780
rs10047088
rs10518646
rs75036101
rs35032648
rs10946649
rs10517576
rs7819104
rs72822577
rs10179730
rs12684941
rs17002700
rs184989157
rs75559970
rs11929631
rs35996890
rs11614716
rs17088862
All
−0.4
−0.3
−0.2
−0.1
0.0
MR leave−one−out sensitivity analysis for
'CD4 on HLA DR+ CD4+ ' on 'Juvenile arthritis'

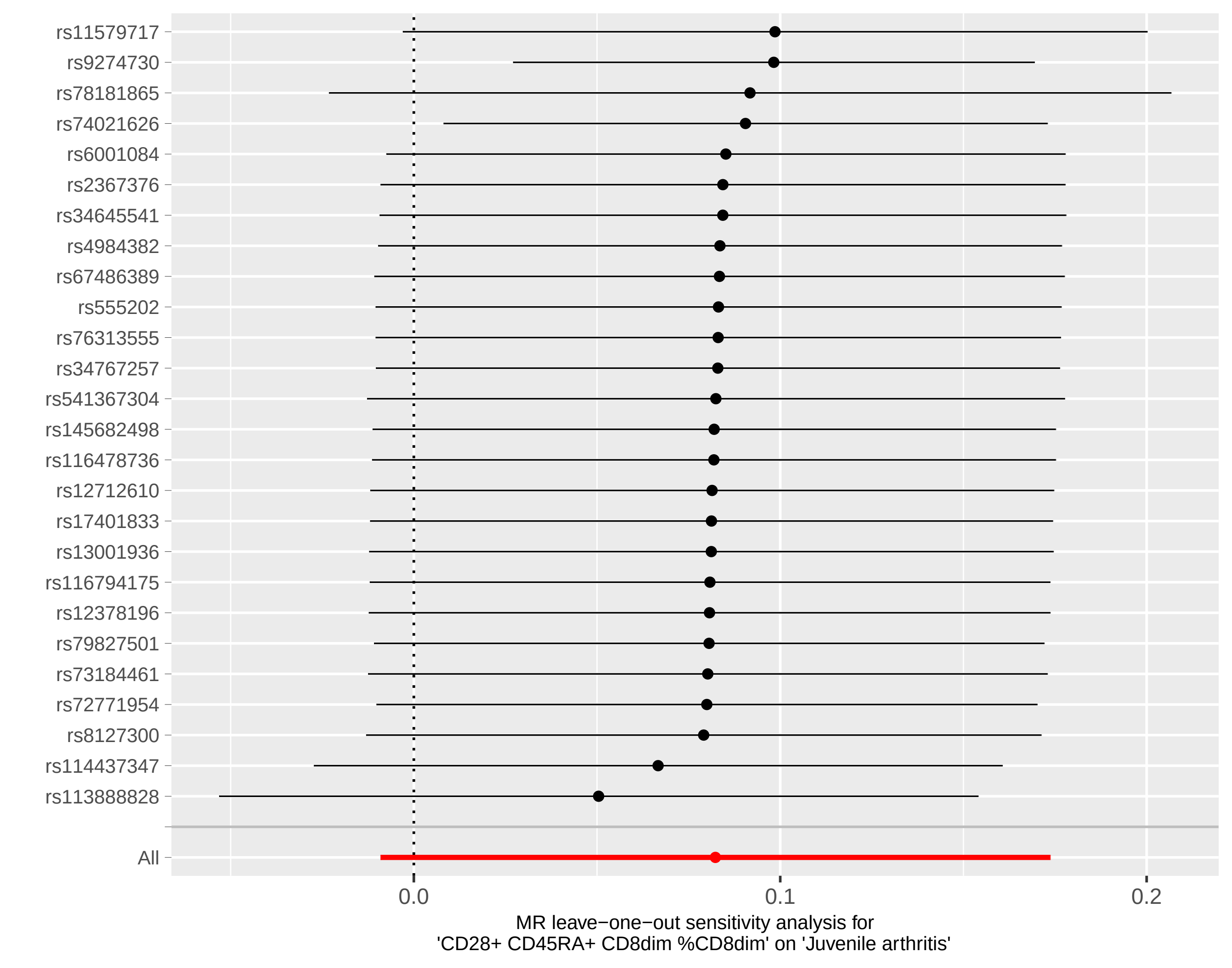
rs11579717
rs9274730
rs78181865
rs74021626
rs6001084
rs2367376
rs34645541
rs4984382
rs67486389
rs555202
rs76313555
rs34767257
rs541367304
rs145682498
rs116478736
rs12712610
rs17401833
rs13001936
rs116794175
rs12378196
rs79827501
rs73184461
rs72771954
rs8127300
rs114437347
rs113888828
All
0.0
0.1
0.2
MR leave−one−out sensitivity analysis for
'CD28+ CD45RA+ CD8dim %CD8dim' on 'Juvenile arthritis'

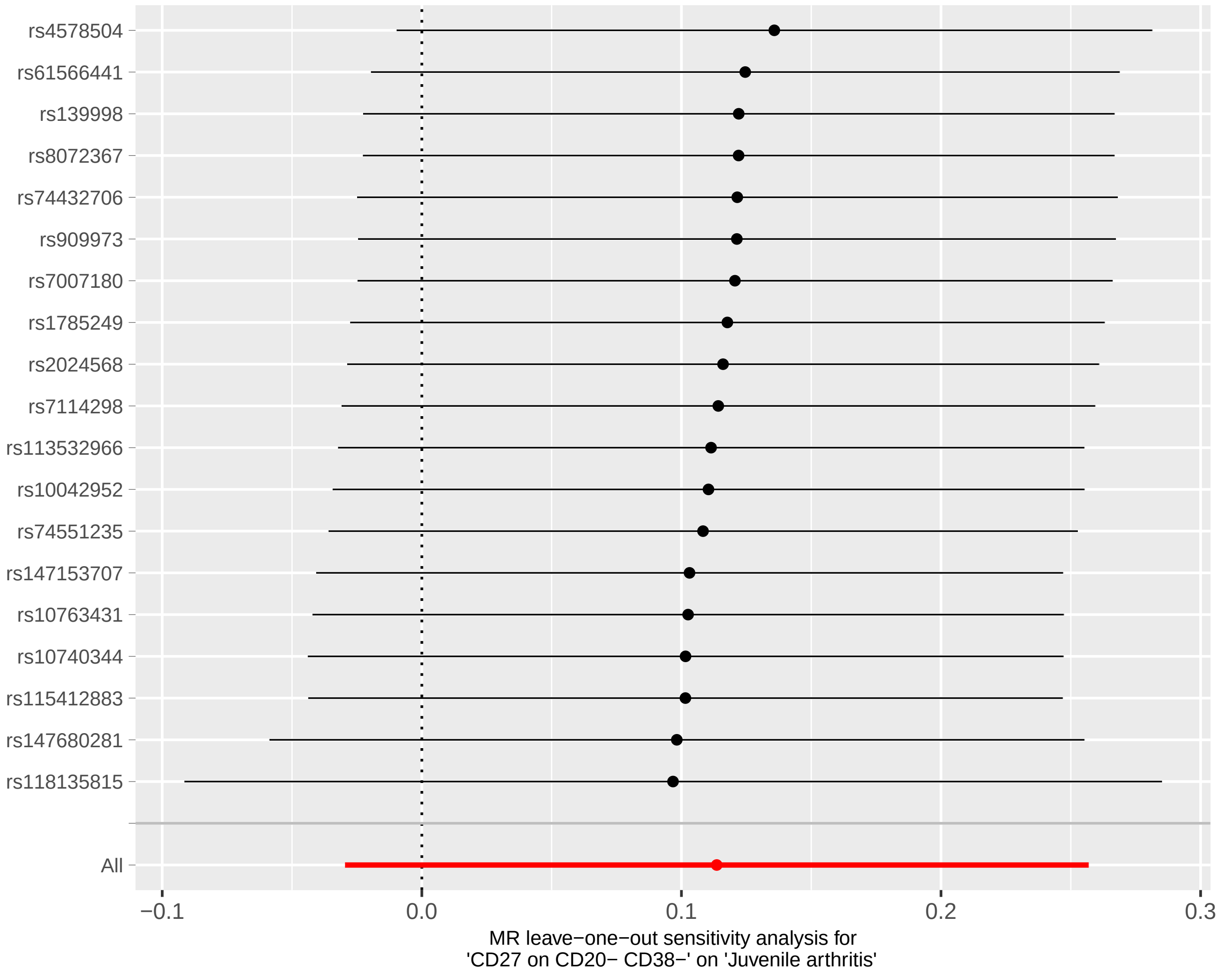
rs4578504
rs61566441
rs139998
rs8072367
rs74432706
rs909973
rs7007180
rs1785249
rs2024568
rs7114298
rs113532966
rs10042952
rs74551235
rs147153707
rs10763431
rs10740344
rs115412883
rs147680281
rs118135815
All
−0.1
0.0
0.1
0.2
0.3
MR leave−one−out sensitivity analysis for
'CD27 on CD20− CD38−' on 'Juvenile arthritis'

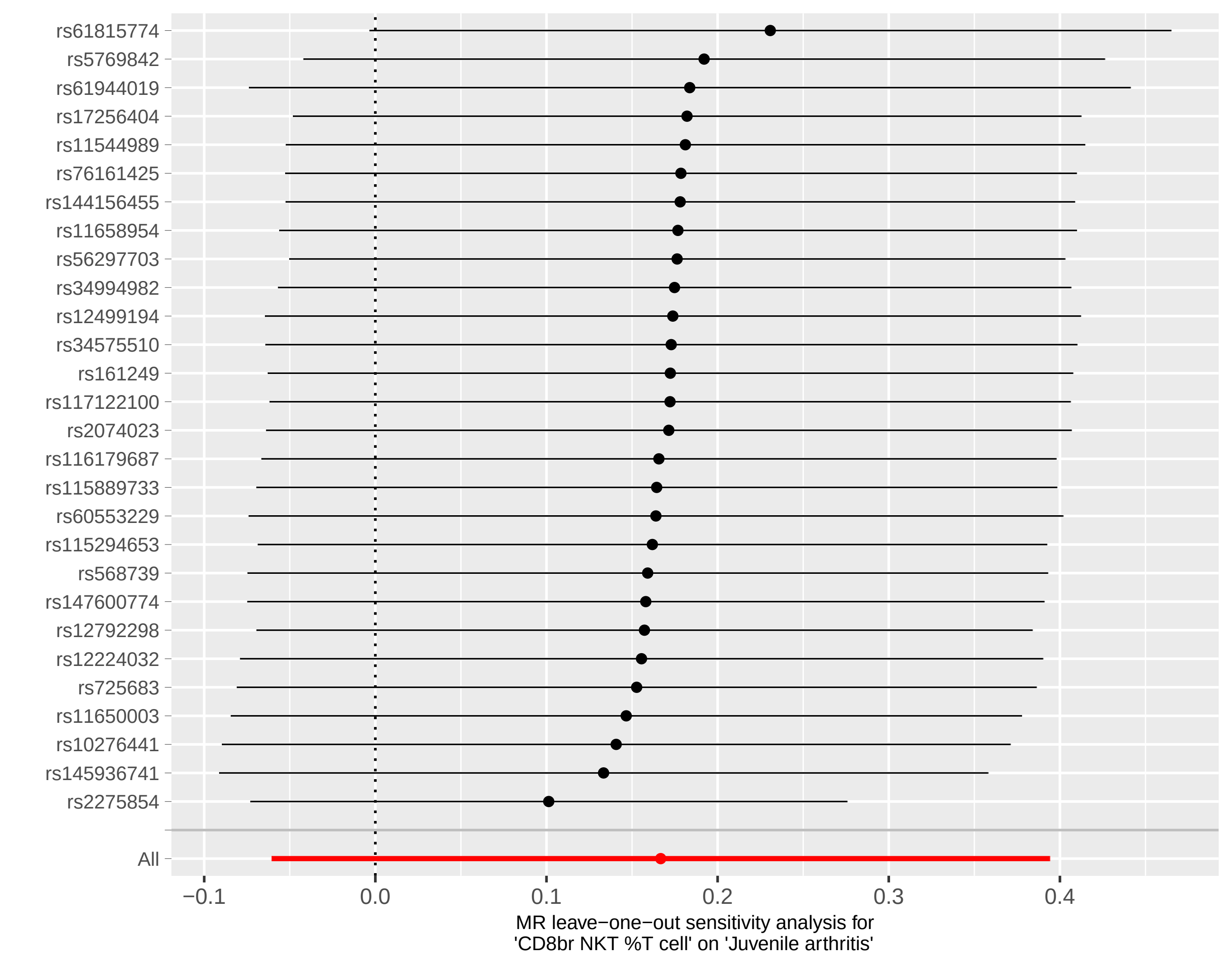

rs61815774
rs5769842
rs61944019
rs17256404
rs11544989
rs76161425
rs144156455
rs11658954
rs56297703
rs34994982
rs12499194
rs34575510
rs161249
rs117122100
rs2074023
rs116179687
rs115889733
rs60553229
rs115294653
rs568739
rs147600774
rs12792298
rs12224032
rs725683
rs11650003
rs10276441
rs145936741
rs2275854
All
−0.1
0.0
0.1
0.2
0.3
0.4
MR leave−one−out sensitivity analysis for
'CD8br NKT %T cell' on 'Juvenile arthritis'

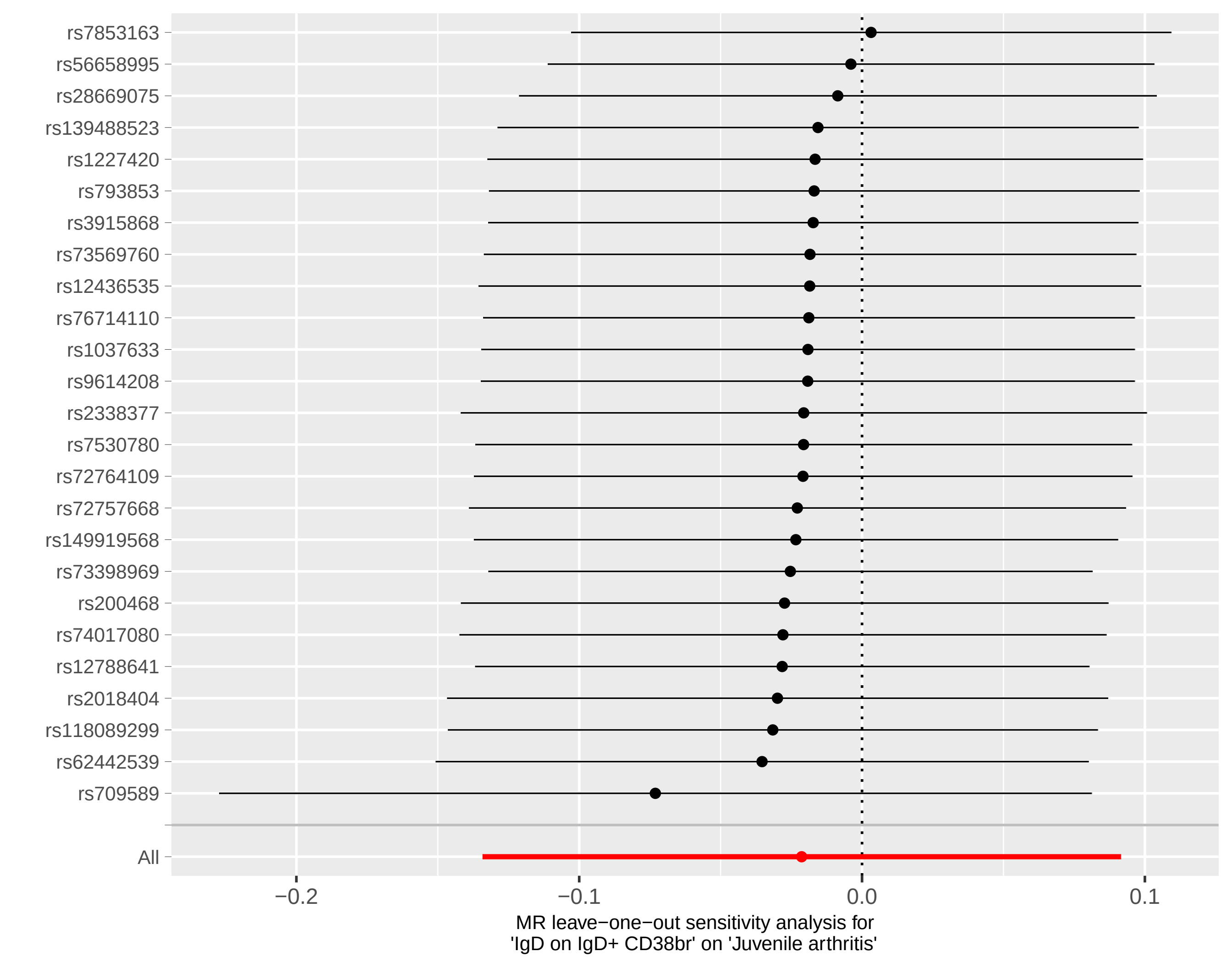
rs7853163
rs56658995
rs28669075
rs139488523
rs1227420
rs793853
rs3915868
rs73569760
rs12436535
rs76714110
rs1037633
rs9614208
rs2338377
rs7530780
rs72764109
rs72757668
rs149919568
rs73398969
rs200468
rs74017080
rs12788641
rs2018404
rs118089299
rs62442539
rs709589
All
−0.2
−0.1
0.0
0.1
MR leave−one−out sensitivity analysis for
'IgD on IgD+ CD38br' on 'Juvenile arthritis'

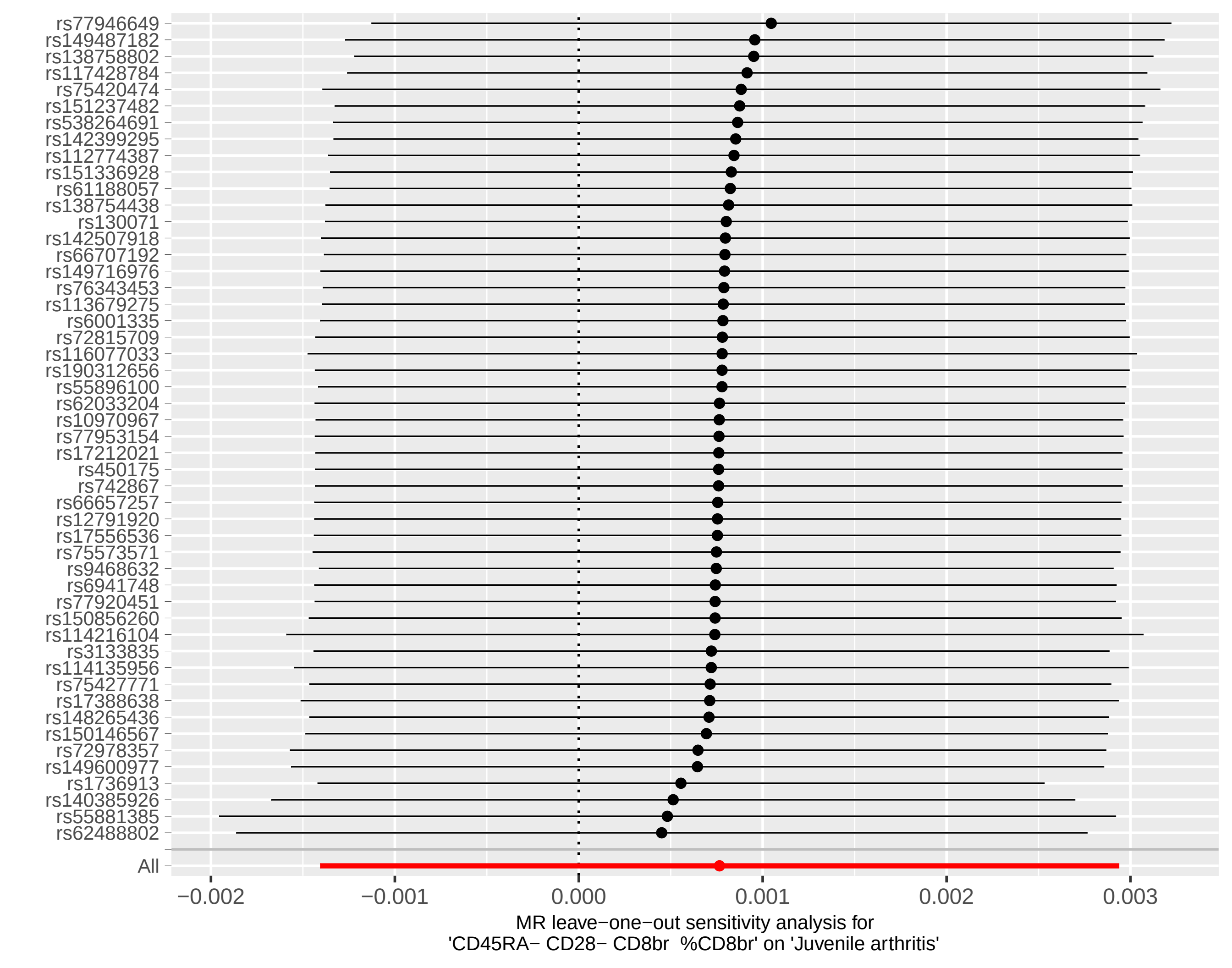
rs77946649
rs149487182
rs138758802
rs117428784
rs75420474
rs151237482
rs538264691
rs142399295
rs112774387
rs151336928
rs61188057
rs138754438
rs130071
rs142507918
rs66707192
rs149716976
rs76343453
rs113679275
rs6001335
rs72815709
rs116077033
rs190312656
rs55896100
rs62033204
rs10970967
rs77953154
rs17212021
rs450175
rs742867
rs66657257
rs12791920
rs17556536
rs75573571
rs9468632
rs6941748
rs77920451
rs150856260
rs114216104
rs3133835
rs114135956
rs75427771
rs17388638
rs148265436
rs150146567
rs72978357
rs149600977
rs1736913
rs140385926
rs55881385
rs62488802
All
−0.002
−0.001
0.000
0.001
0.002
0.003
MR leave−one−out sensitivity analysis for
'CD45RA− CD28− CD8br %CD8br' on 'Juvenile arthritis'

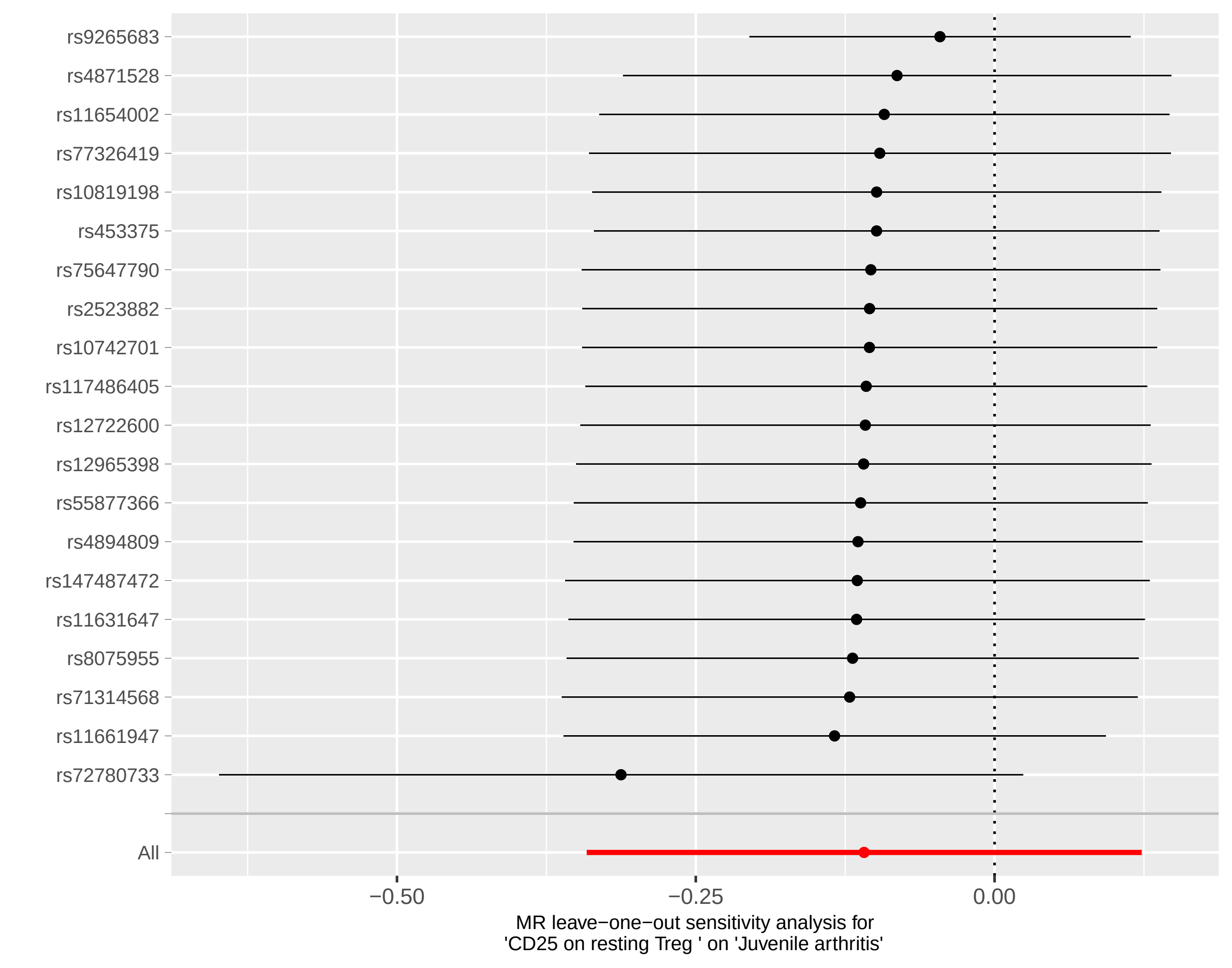
rs9265683
rs4871528
rs11654002
rs77326419
rs10819198
rs453375
rs75647790
rs2523882
rs10742701
rs117486405
rs12722600
rs12965398
rs55877366
rs4894809
rs147487472
rs11631647
rs8075955
rs71314568
rs11661947
rs72780733
All
−0.50
−0.25
0.00
MR leave−one−out sensitivity analysis for
'CD25 on resting Treg ' on 'Juvenile arthritis'

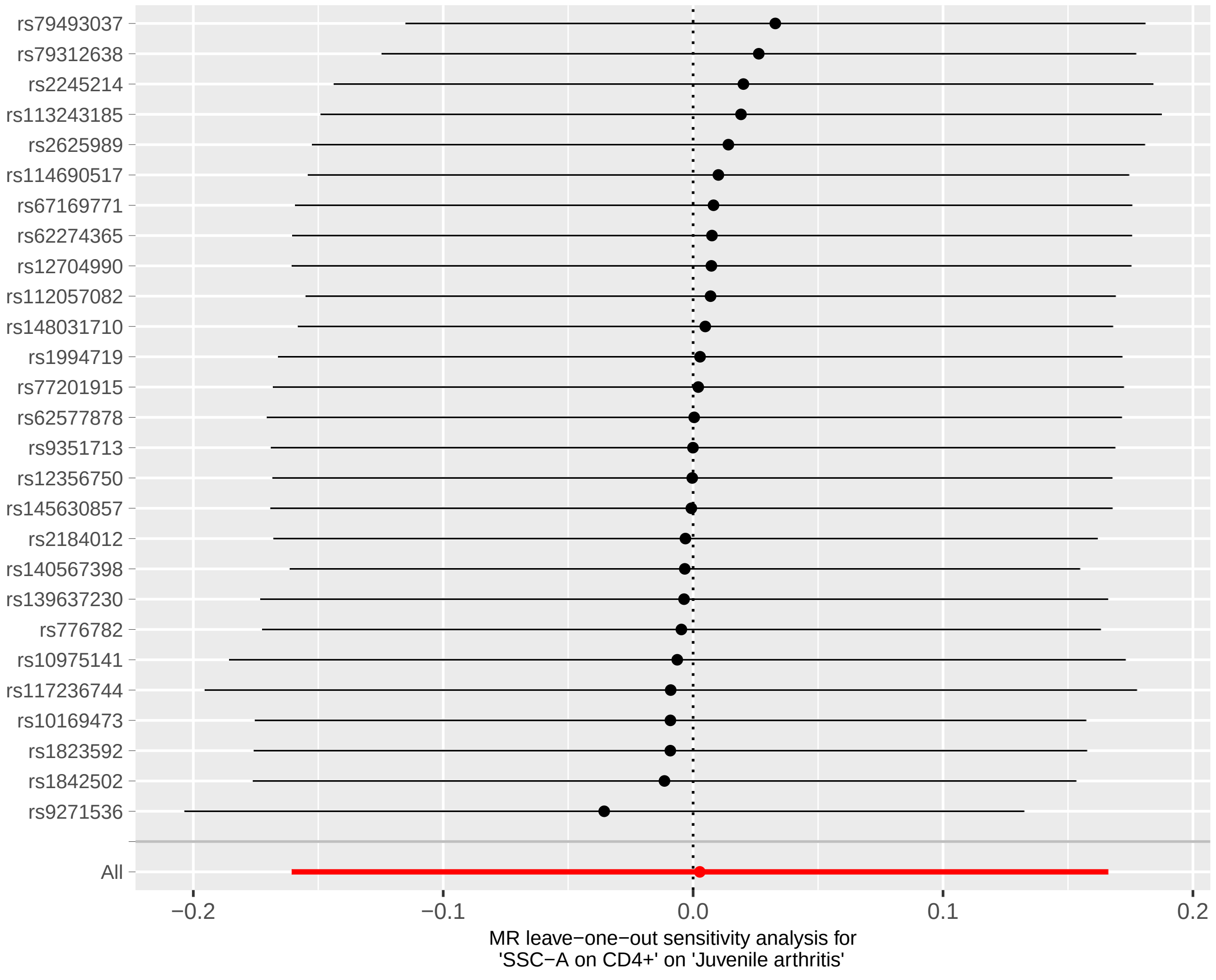
rs79493037
rs79312638
rs2245214
rs113243185
rs2625989
rs114690517
rs67169771
rs62274365
rs12704990
rs112057082
rs148031710
rs1994719
rs77201915
rs62577878
rs9351713
rs12356750
rs145630857
rs2184012
rs140567398
rs139637230
rs776782
rs10975141
rs117236744
rs10169473
rs1823592
rs1842502
rs9271536
All
−0.2
−0.1
0.0
0.1
0.2
MR leave−one−out sensitivity analysis for
'SSC−A on CD4+' on 'Juvenile arthritis'

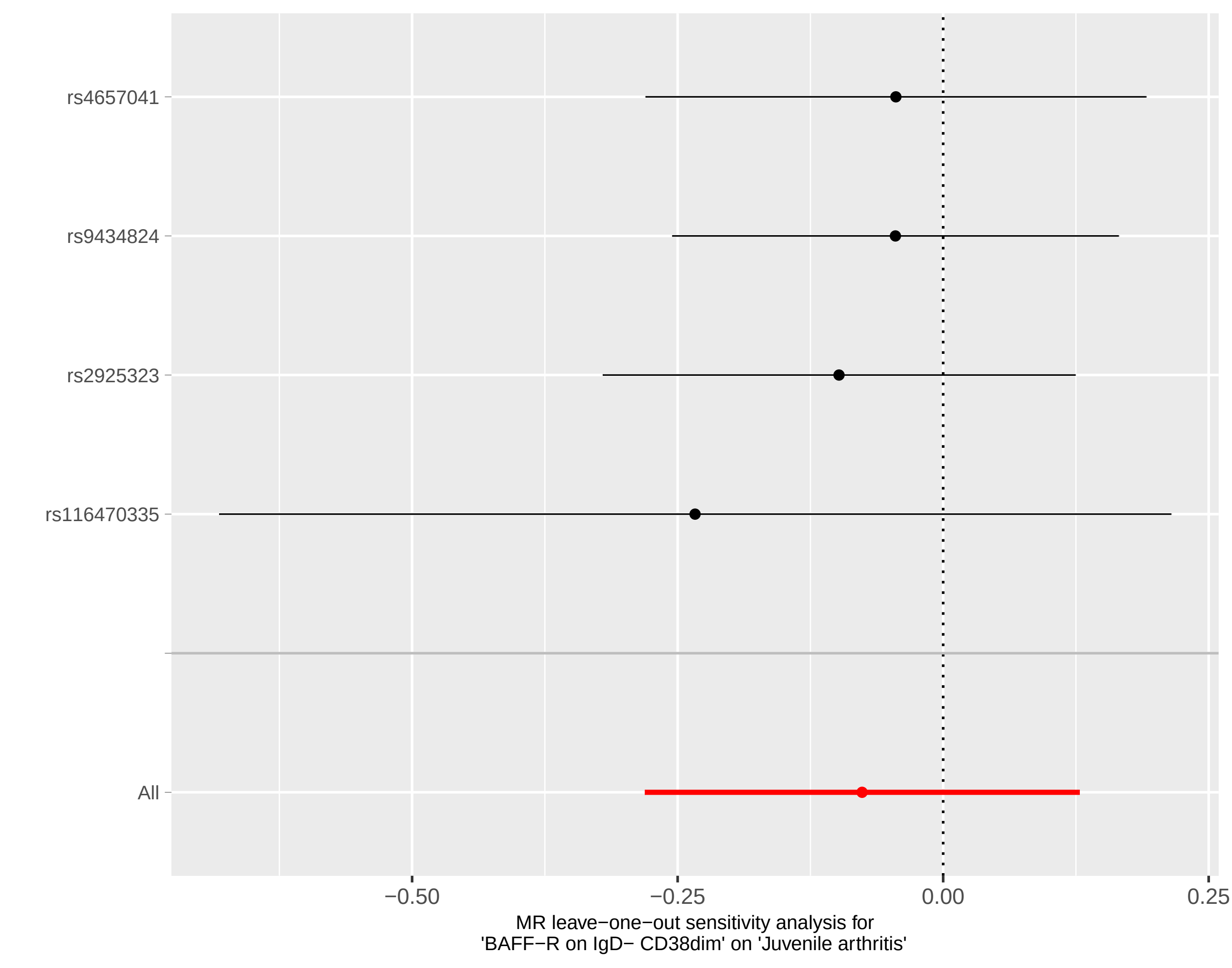
rs4657041
rs9434824
rs2925323
rs116470335
All
−0.50
−0.25
0.00
0.25
MR leave−one−out sensitivity analysis for
'BAFF−R on IgD− CD38dim' on 'Juvenile arthritis'

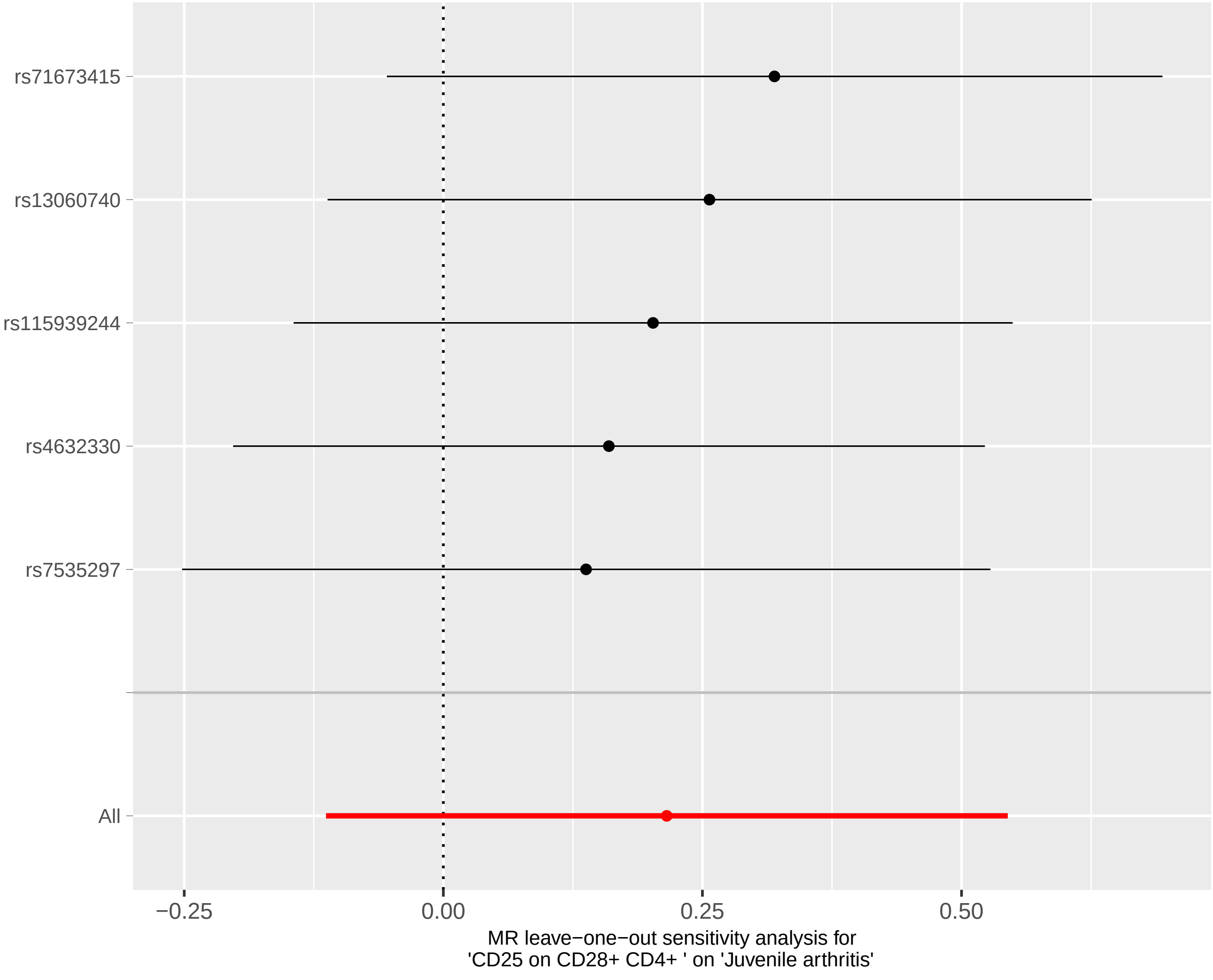
rs71673415
rs13060740
rs115939244
rs4632330
rs7535297
All
−0.25
0.00
0.25
0.50
MR leave−one−out sensitivity analysis for
'CD25 on CD28+ CD4+ ' on 'Juvenile arthritis'

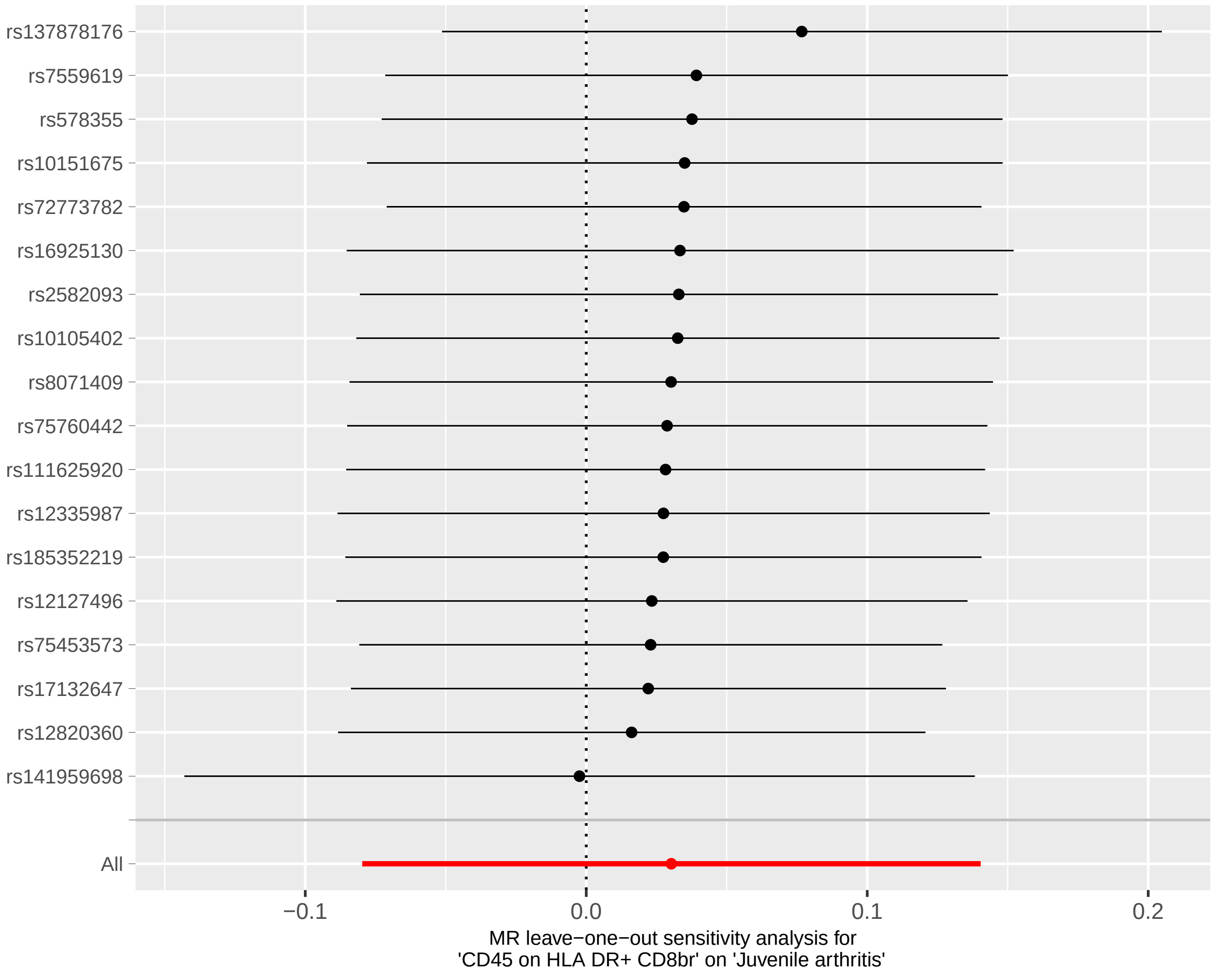
rs137878176
rs7559619
rs578355
rs10151675
rs72773782
rs16925130
rs2582093
rs10105402
rs8071409
rs75760442
rs111625920
rs12335987
rs185352219
rs12127496
rs75453573
rs17132647
rs12820360
rs141959698
All
−0.1
0.0
0.1
0.2
MR leave−one−out sensitivity analysis for
'CD45 on HLA DR+ CD8br' on 'Juvenile arthritis'

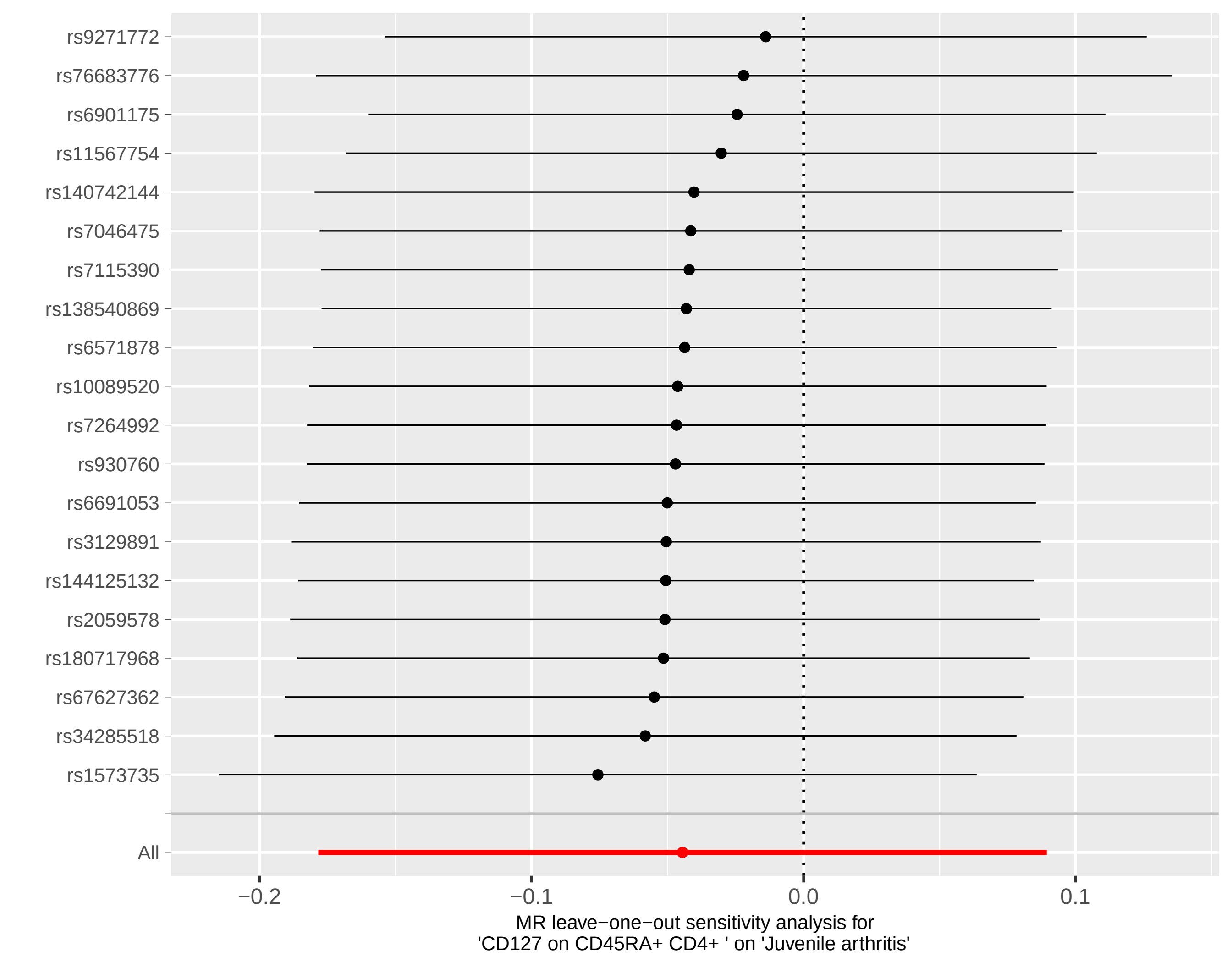

rs9271772
rs76683776
rs6901175
rs11567754
rs140742144
rs7046475
rs7115390
rs138540869
rs6571878
rs10089520
rs7264992
rs930760
rs6691053
rs3129891
rs144125132
rs2059578
rs180717968
rs67627362
rs34285518
rs1573735
All
−0.2
−0.1
0.0
0.1
MR leave−one−out sensitivity analysis for
'CD127 on CD45RA+ CD4+ ' on 'Juvenile arthritis'

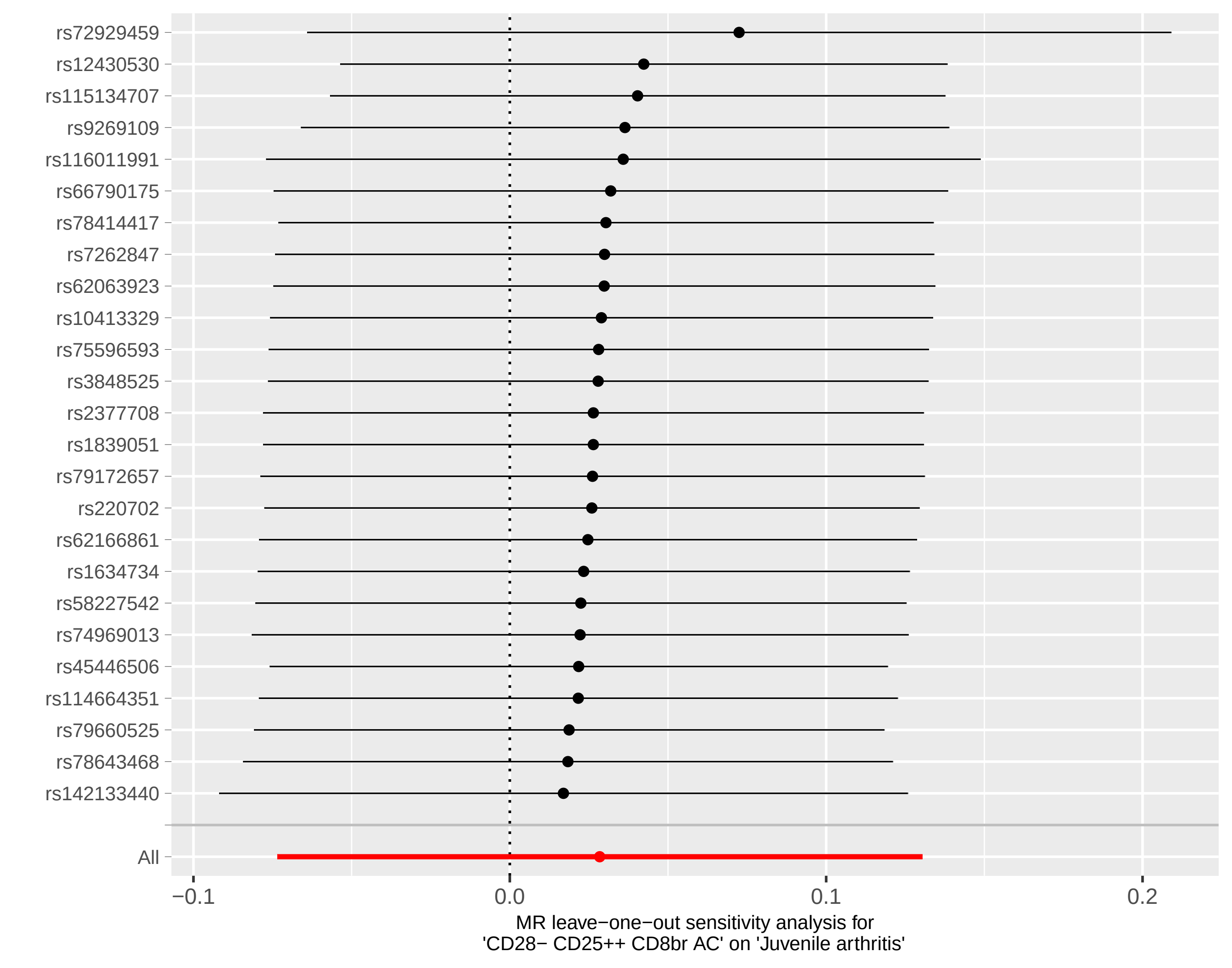
rs72929459
rs12430530
rs115134707
rs9269109
rs116011991
rs66790175
rs78414417
rs7262847
rs62063923
rs10413329
rs75596593
rs3848525
rs2377708
rs1839051
rs79172657
rs220702
rs62166861
rs1634734
rs58227542
rs74969013
rs45446506
rs114664351
rs79660525
rs78643468
rs142133440
All
−0.1
0.0
0.1
0.2
MR leave−one−out sensitivity analysis for
'CD28− CD25++ CD8br AC' on 'Juvenile arthritis'

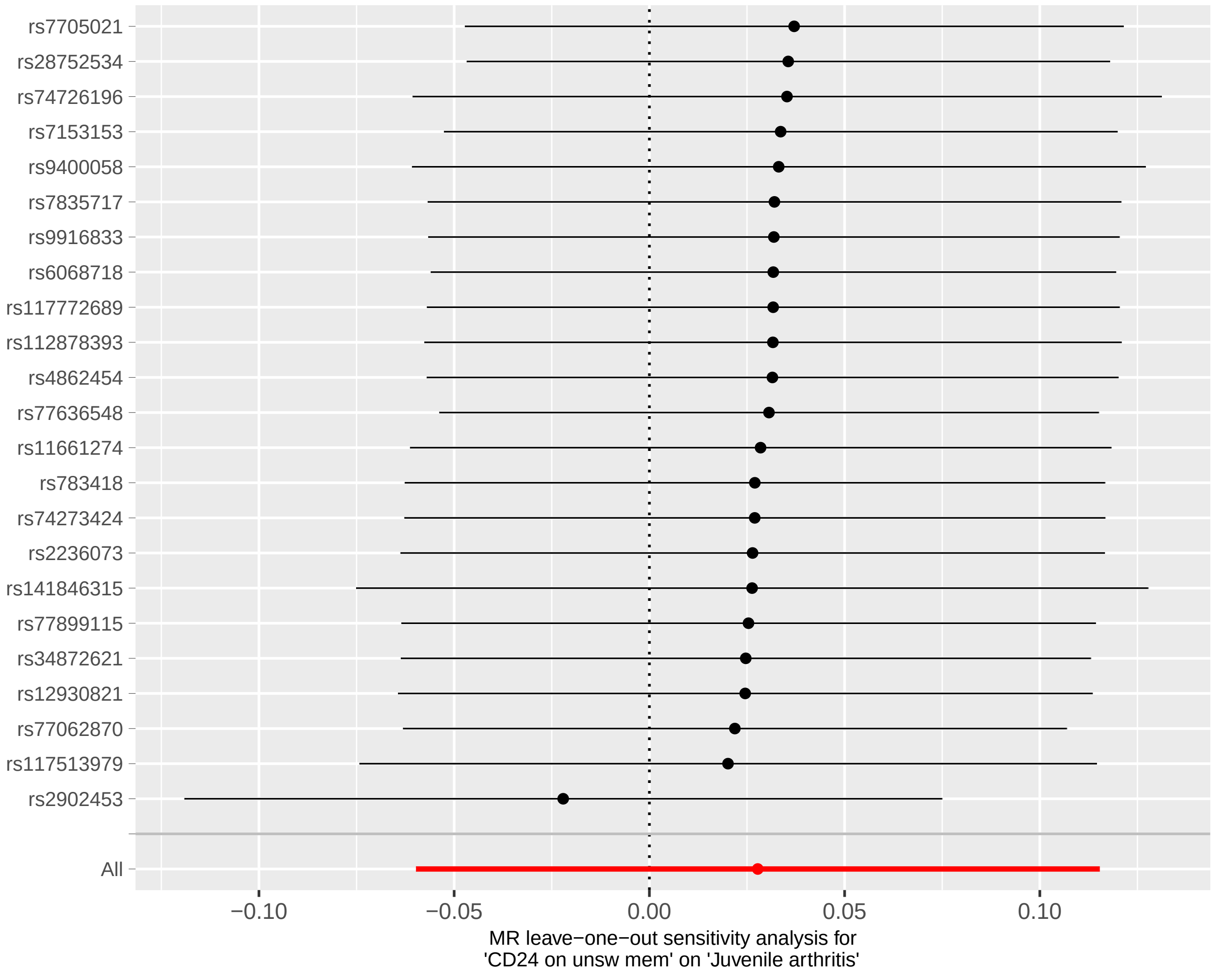

rs7705021
rs28752534
rs74726196
rs7153153
rs9400058
rs7835717
rs9916833
rs6068718
rs117772689
rs112878393
rs4862454
rs77636548
rs11661274
rs783418
rs74273424
rs2236073
rs141846315
rs77899115
rs34872621
rs12930821
rs77062870
rs117513979
rs2902453
All
−0.10
−0.05
0.00
0.05
0.10
MR leave−one−out sensitivity analysis for
'CD24 on unsw mem' on 'Juvenile arthritis'

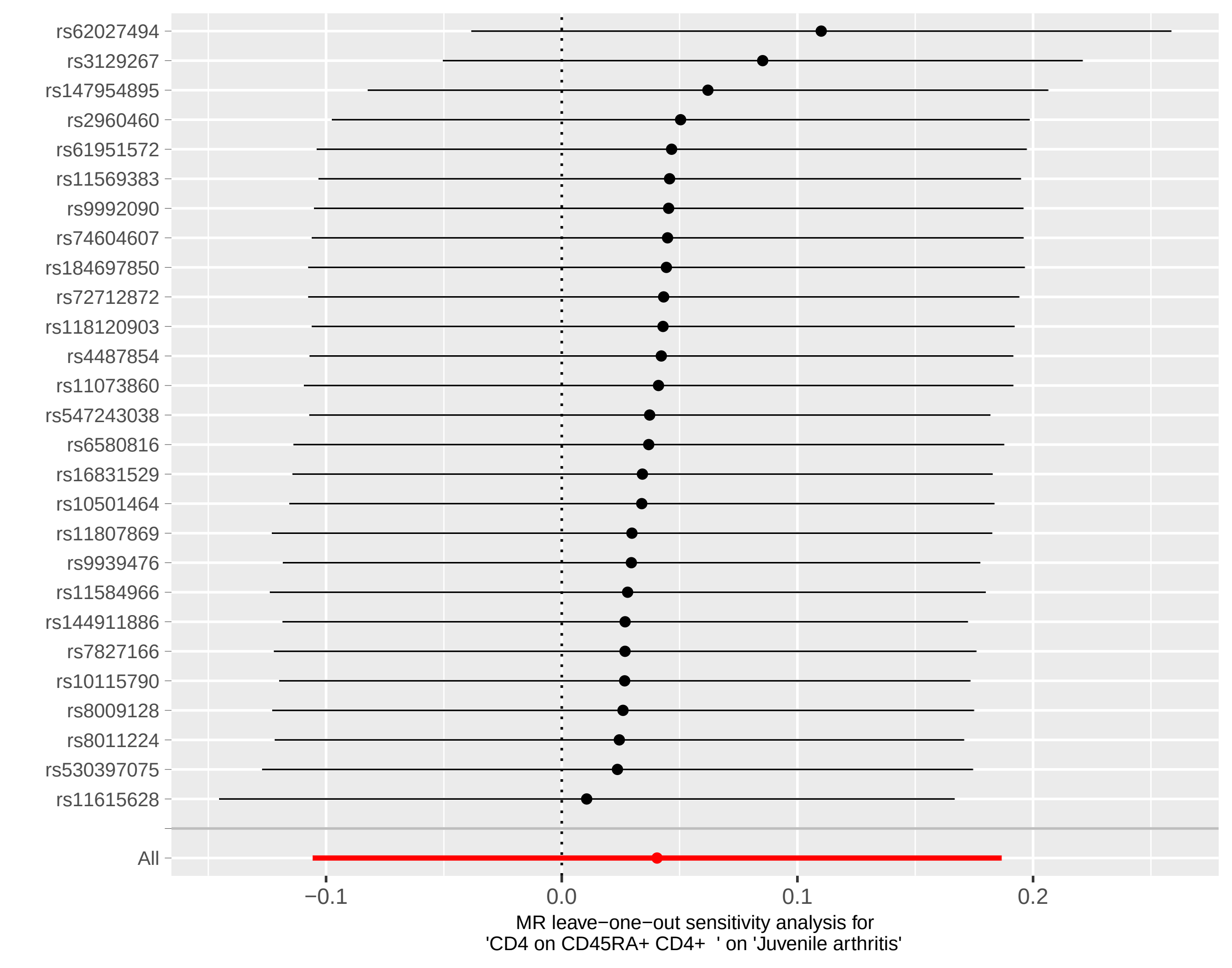
rs62027494
rs3129267
rs147954895
rs2960460
rs61951572
rs11569383
rs9992090
rs74604607
rs184697850
rs72712872
rs118120903
rs4487854
rs11073860
rs547243038
rs6580816
rs16831529
rs10501464
rs11807869
rs9939476
rs11584966
rs144911886
rs7827166
rs10115790
rs8009128
rs8011224
rs530397075
rs11615628
All
−0.1
0.0
0.1
0.2
MR leave−one−out sensitivity analysis for
'CD4 on CD45RA+ CD4+ ' on 'Juvenile arthritis'

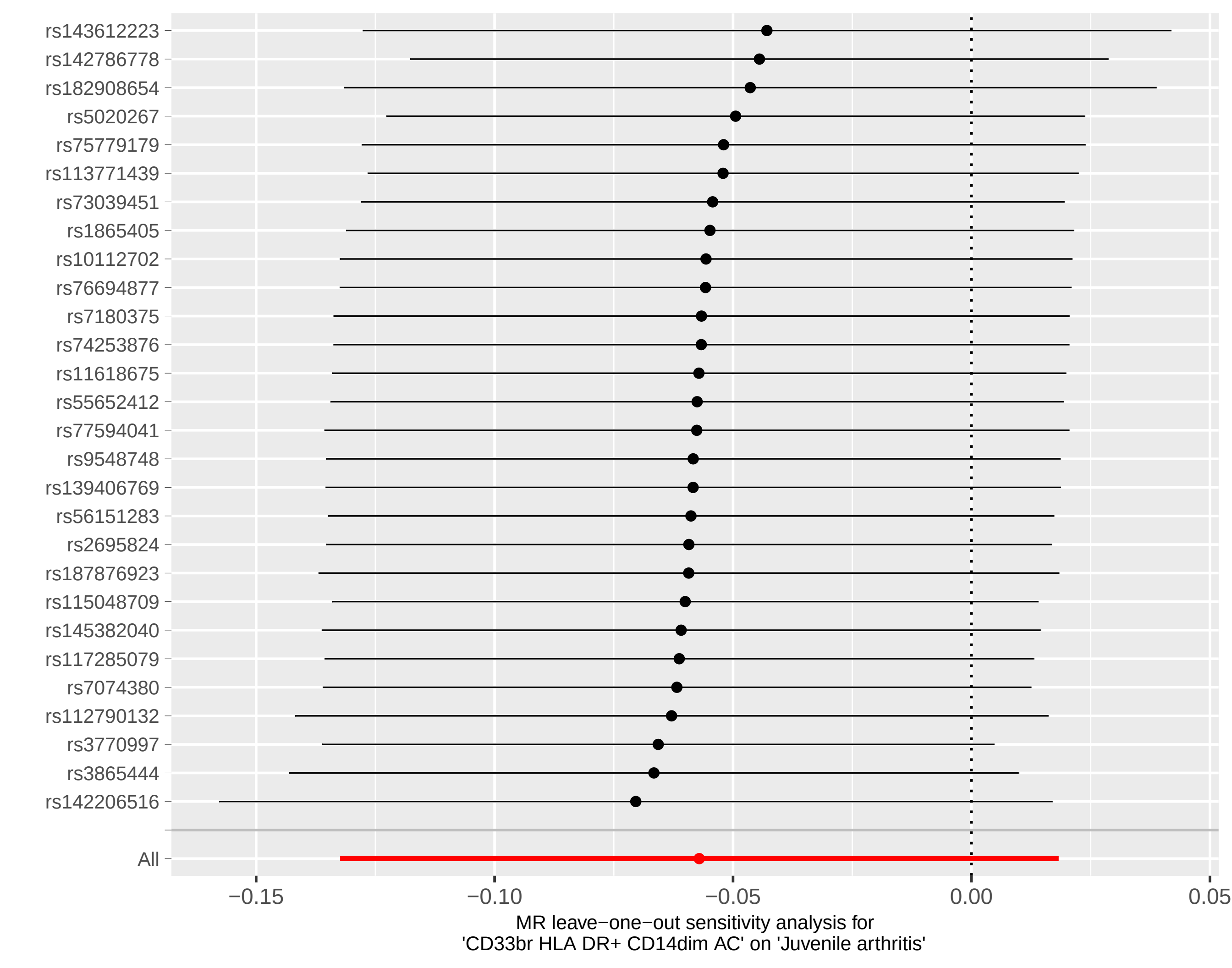

rs143612223
rs142786778
rs182908654
rs5020267
rs75779179
rs113771439
rs73039451
rs1865405
rs10112702
rs76694877
rs7180375
rs74253876
rs11618675
rs55652412
rs77594041
rs9548748
rs139406769
rs56151283
rs2695824
rs187876923
rs115048709
rs145382040
rs117285079
rs7074380
rs112790132
rs3770997
rs3865444
rs142206516
All
−0.15
−0.10
−0.05
0.00
0.05
MR leave−one−out sensitivity analysis for
'CD33br HLA DR+ CD14dim AC' on 'Juvenile arthritis'

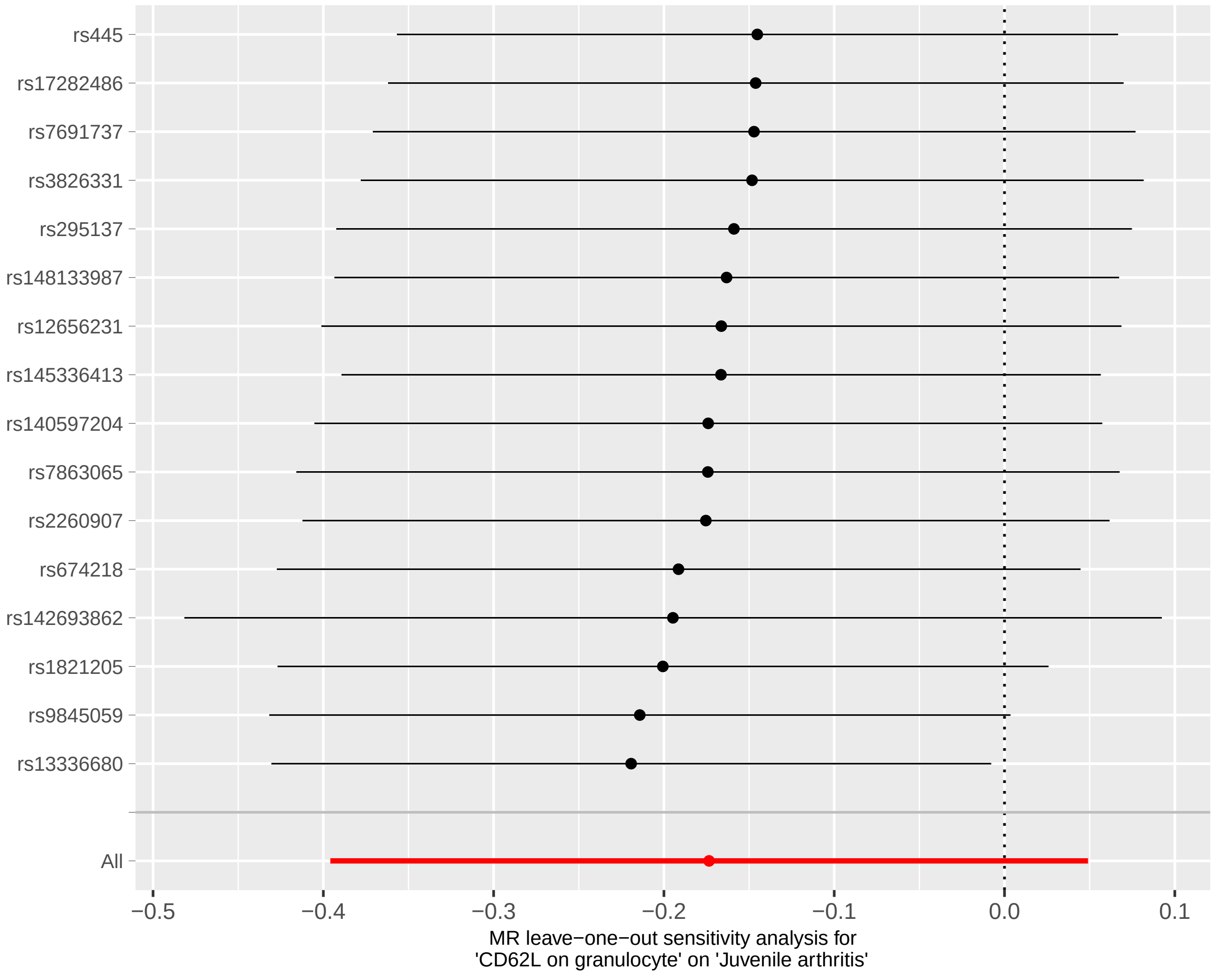
rs445
rs17282486
rs7691737
rs3826331
rs295137
rs148133987
rs12656231
rs145336413
rs140597204
rs7863065
rs2260907
rs674218
rs142693862
rs1821205
rs9845059
rs13336680
All
−0.5
−0.4
−0.3
−0.2
−0.1
0.0
0.1
MR leave−one−out sensitivity analysis for
'CD62L on granulocyte' on 'Juvenile arthritis'

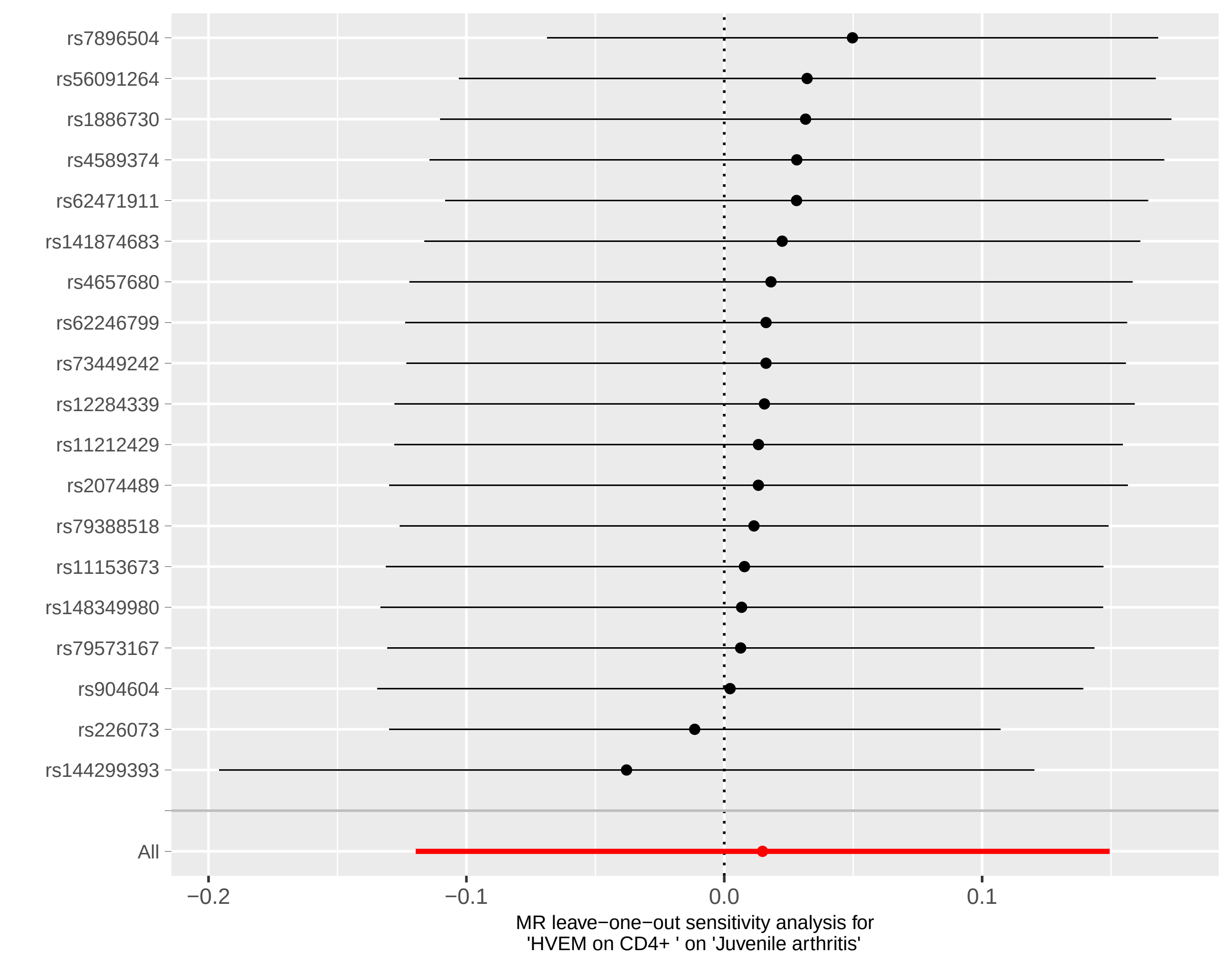

rs7896504
rs56091264
rs1886730
rs4589374
rs62471911
rs141874683
rs4657680
rs62246799
rs73449242
rs12284339
rs11212429
rs2074489
rs79388518
rs11153673
rs148349980
rs79573167
rs904604
rs226073
rs144299393
All
−0.2
−0.1
0.0
0.1
MR leave−one−out sensitivity analysis for
'HVEM on CD4+ ' on 'Juvenile arthritis'

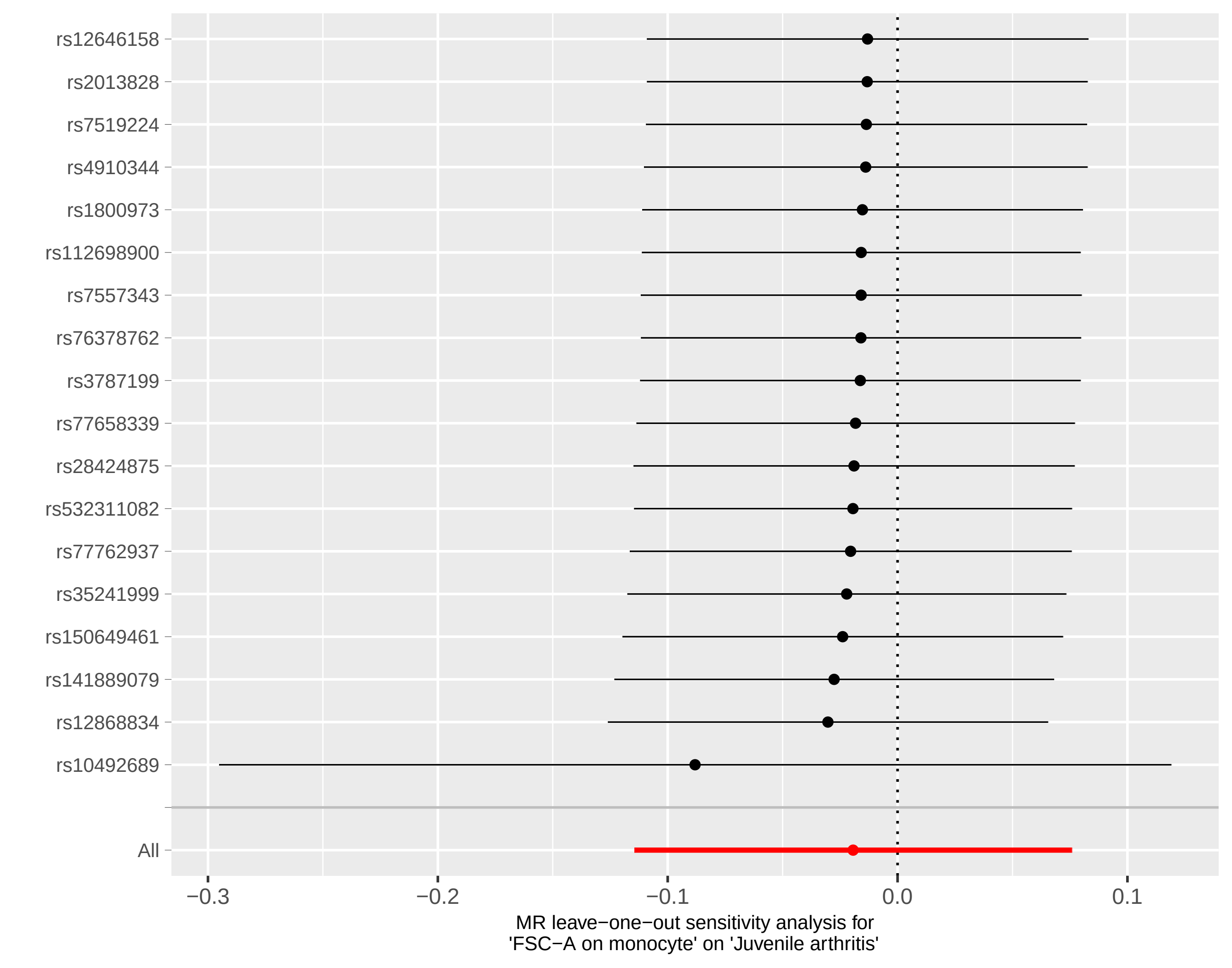
rs12646158
rs2013828
rs7519224
rs4910344
rs1800973
rs112698900
rs7557343
rs76378762
rs3787199
rs77658339
rs28424875
rs532311082
rs77762937
rs35241999
rs150649461
rs141889079
rs12868834
rs10492689
All
−0.3
−0.2
−0.1
0.0
0.1
MR leave−one−out sensitivity analysis for
'FSC−A on monocyte' on 'Juvenile arthritis'

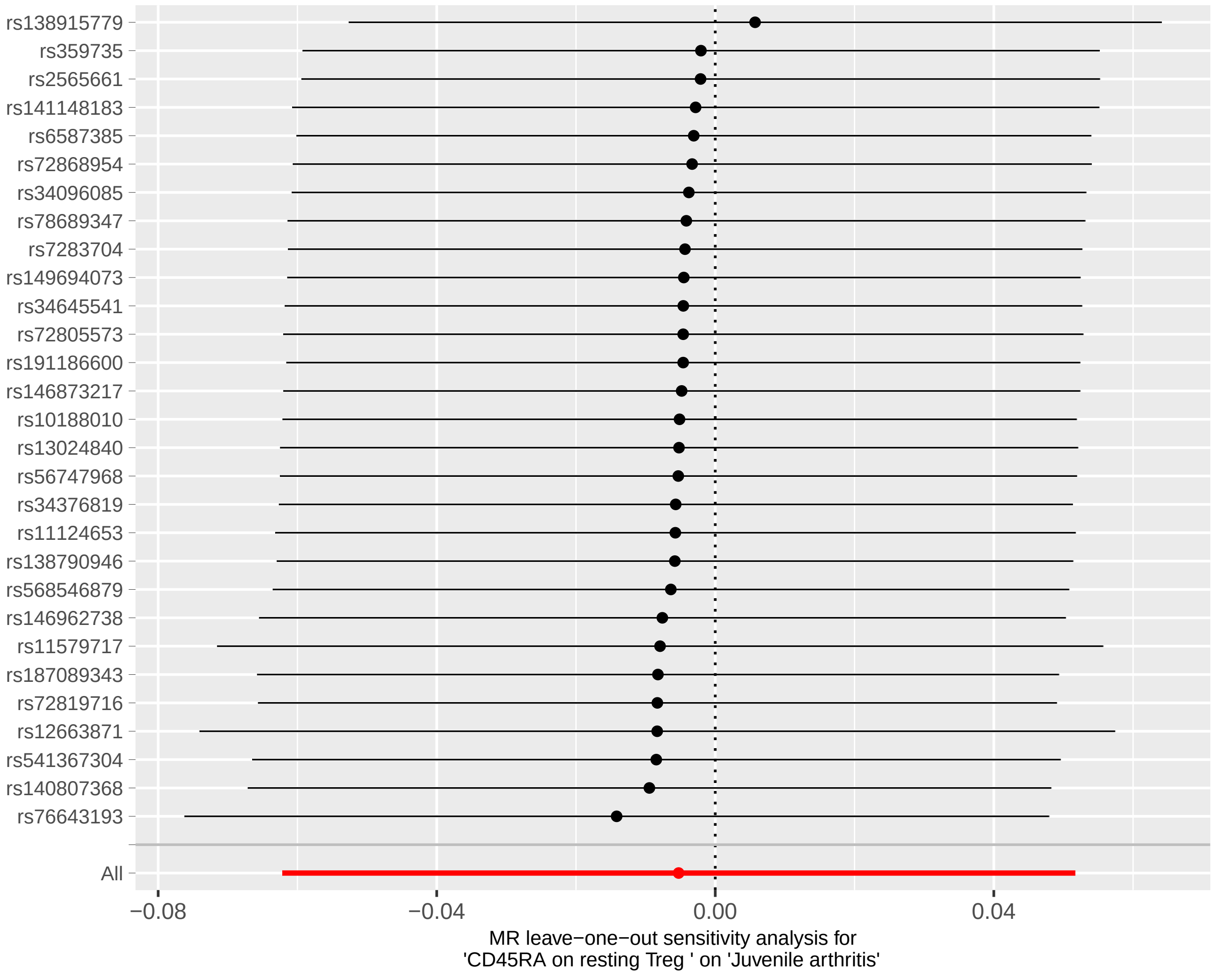

rs138915779
rs359735
rs2565661
rs141148183
rs6587385
rs72868954
rs34096085
rs78689347
rs7283704
rs149694073
rs34645541
rs72805573
rs191186600
rs146873217
rs10188010
rs13024840
rs56747968
rs34376819
rs11124653
rs138790946
rs568546879
rs146962738
rs11579717
rs187089343
rs72819716
rs12663871
rs541367304
rs140807368
rs76643193
All
−0.08
−0.04
0.00
0.04
MR leave−one−out sensitivity analysis for
'CD45RA on resting Treg ' on 'Juvenile arthritis'

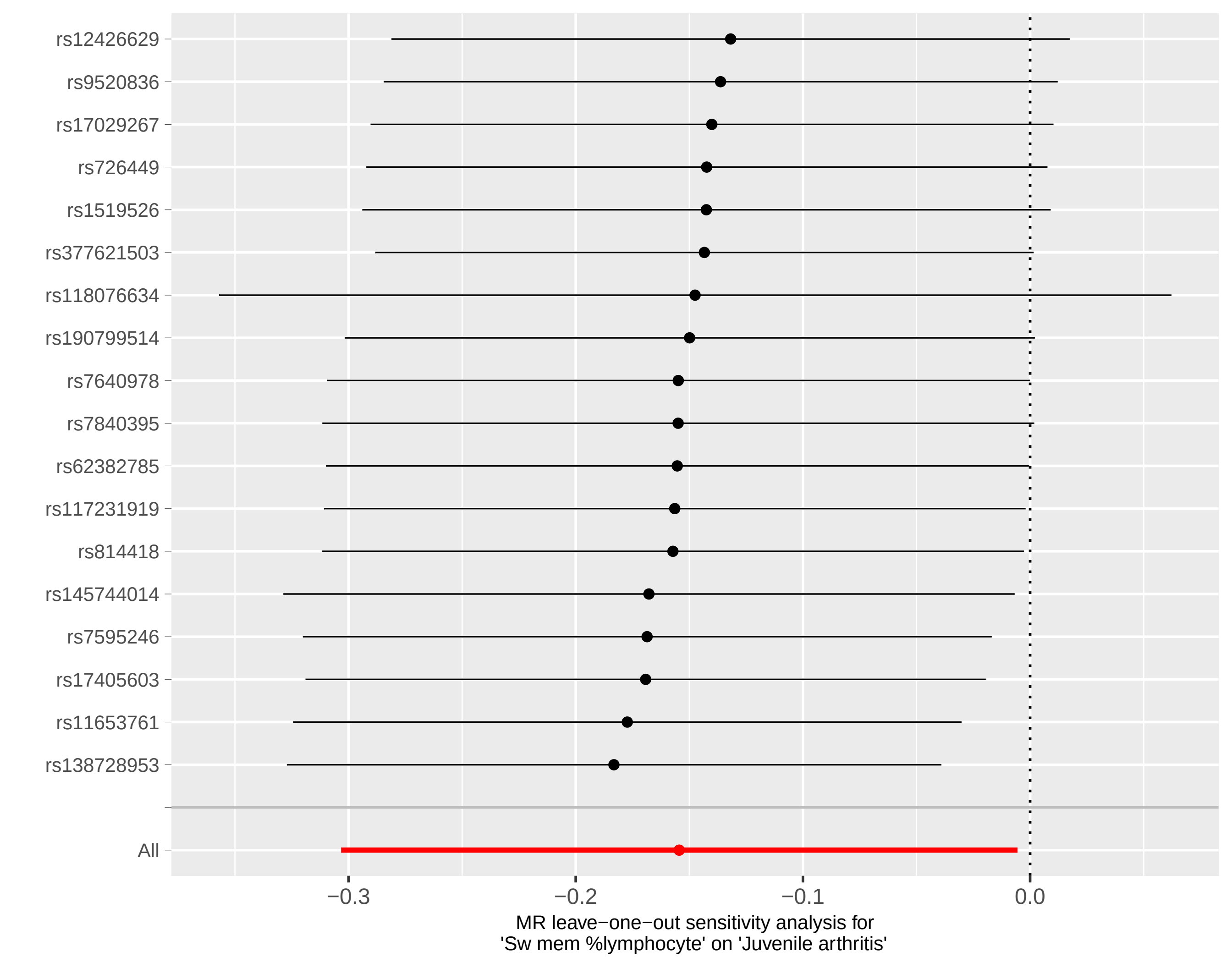

rs12426629
rs9520836
rs17029267
rs726449
rs1519526
rs377621503
rs118076634
rs190799514
rs7640978
rs7840395
rs62382785
rs117231919
rs814418
rs145744014
rs7595246
rs17405603
rs11653761
rs138728953
All
−0.3
−0.2
−0.1
0.0
MR leave−one−out sensitivity analysis for
'Sw mem %lymphocyte' on 'Juvenile arthritis'

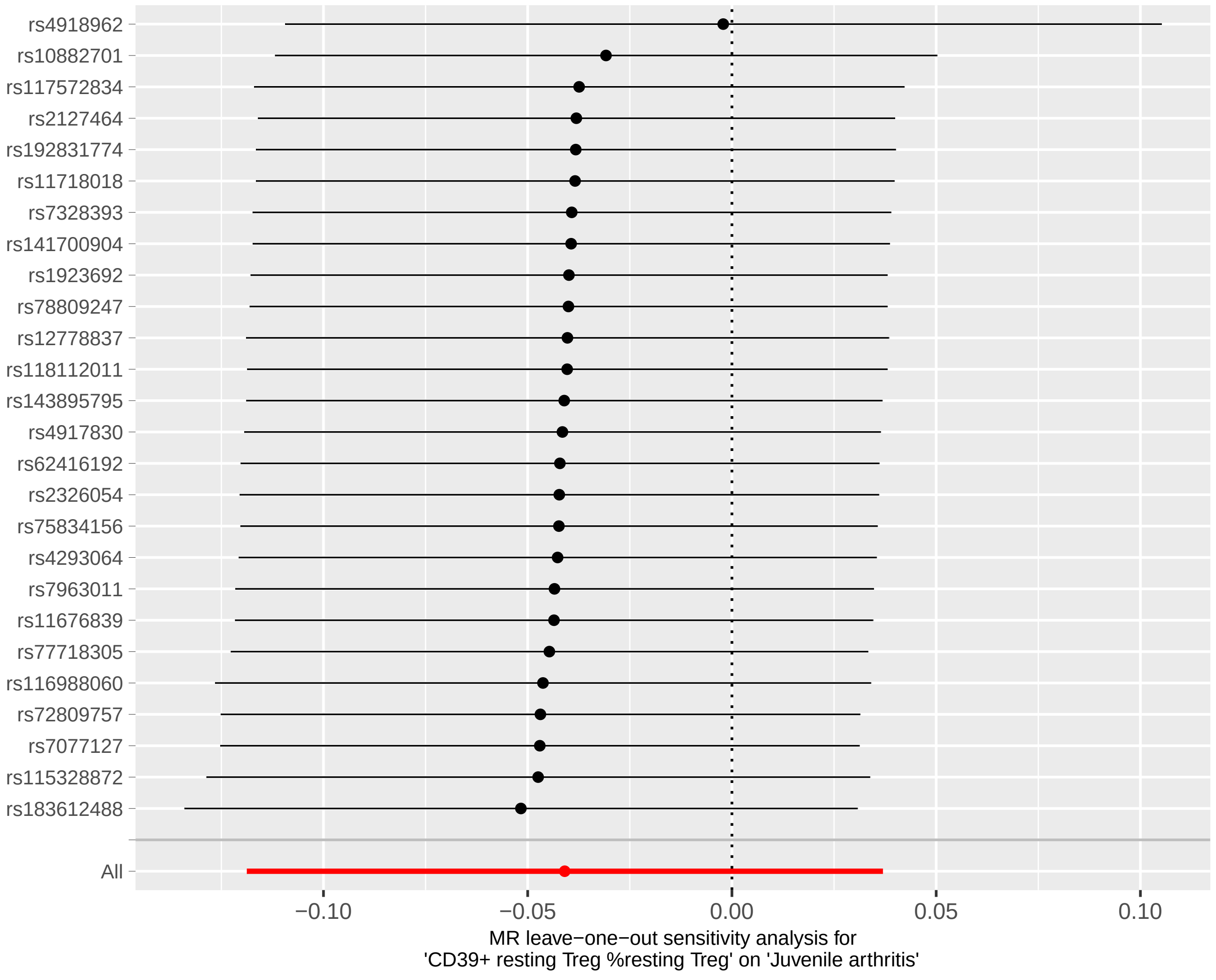
rs4918962
rs10882701
rs117572834
rs2127464
rs192831774
rs11718018
rs7328393
rs141700904
rs1923692
rs78809247
rs12778837
rs118112011
rs143895795
rs4917830
rs62416192
rs2326054
rs75834156
rs4293064
rs7963011
rs11676839
rs77718305
rs116988060
rs72809757
rs7077127
rs115328872
rs183612488
All
−0.10
−0.05
0.00
0.05
0.10
MR leave−one−out sensitivity analysis for
'CD39+ resting Treg %resting Treg' on 'Juvenile arthritis'

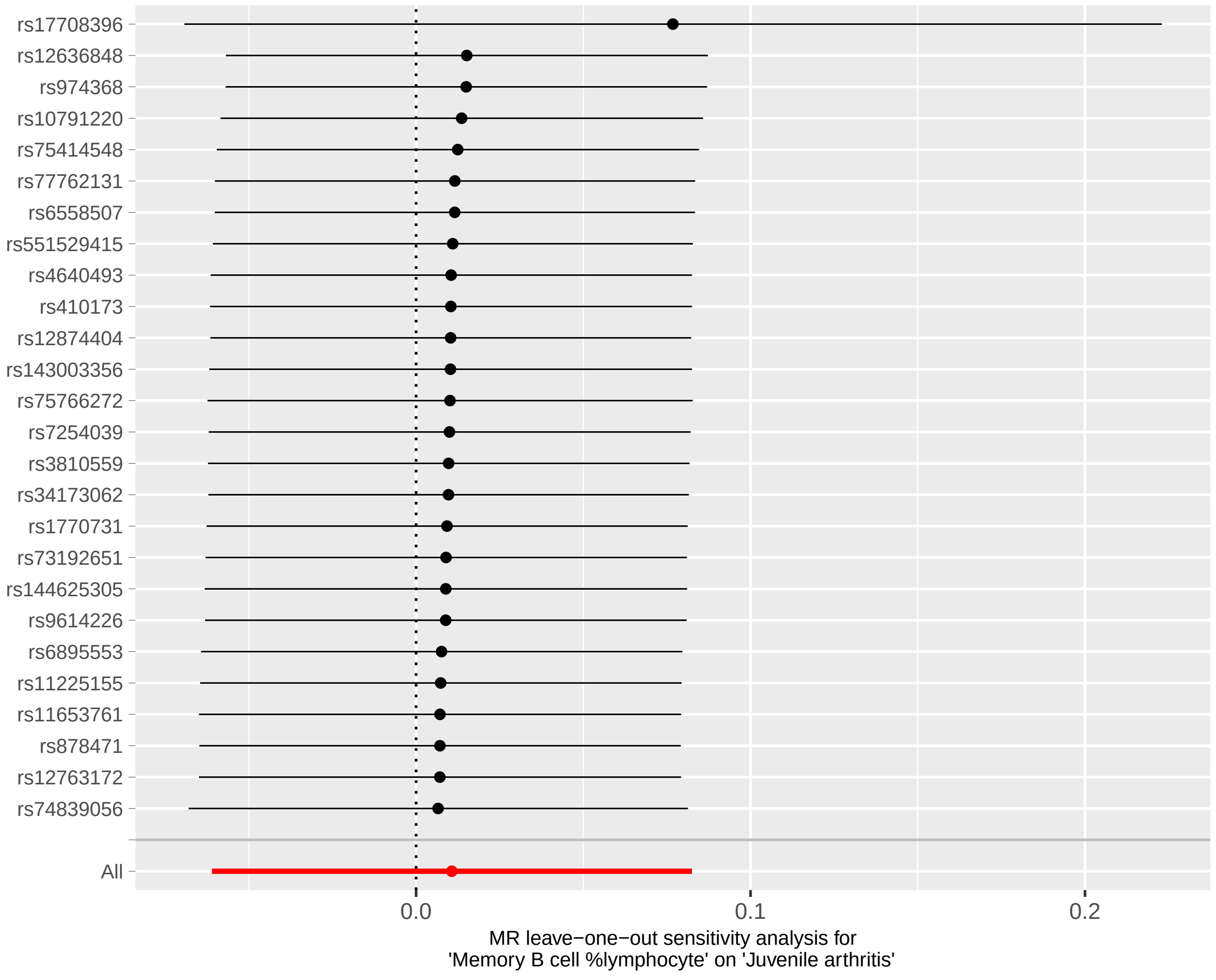
rs17708396
rs12636848
rs974368
rs10791220
rs75414548
rs77762131
rs6558507
rs551529415
rs4640493
rs410173
rs12874404
rs143003356
rs75766272
rs7254039
rs3810559
rs34173062
rs1770731
rs73192651
rs144625305
rs9614226
rs6895553
rs11225155
rs11653761
rs878471
rs12763172
rs74839056
All
0.0
0.1
0.2
MR leave−one−out sensitivity analysis for
'Memory B cell %lymphocyte' on 'Juvenile arthritis'

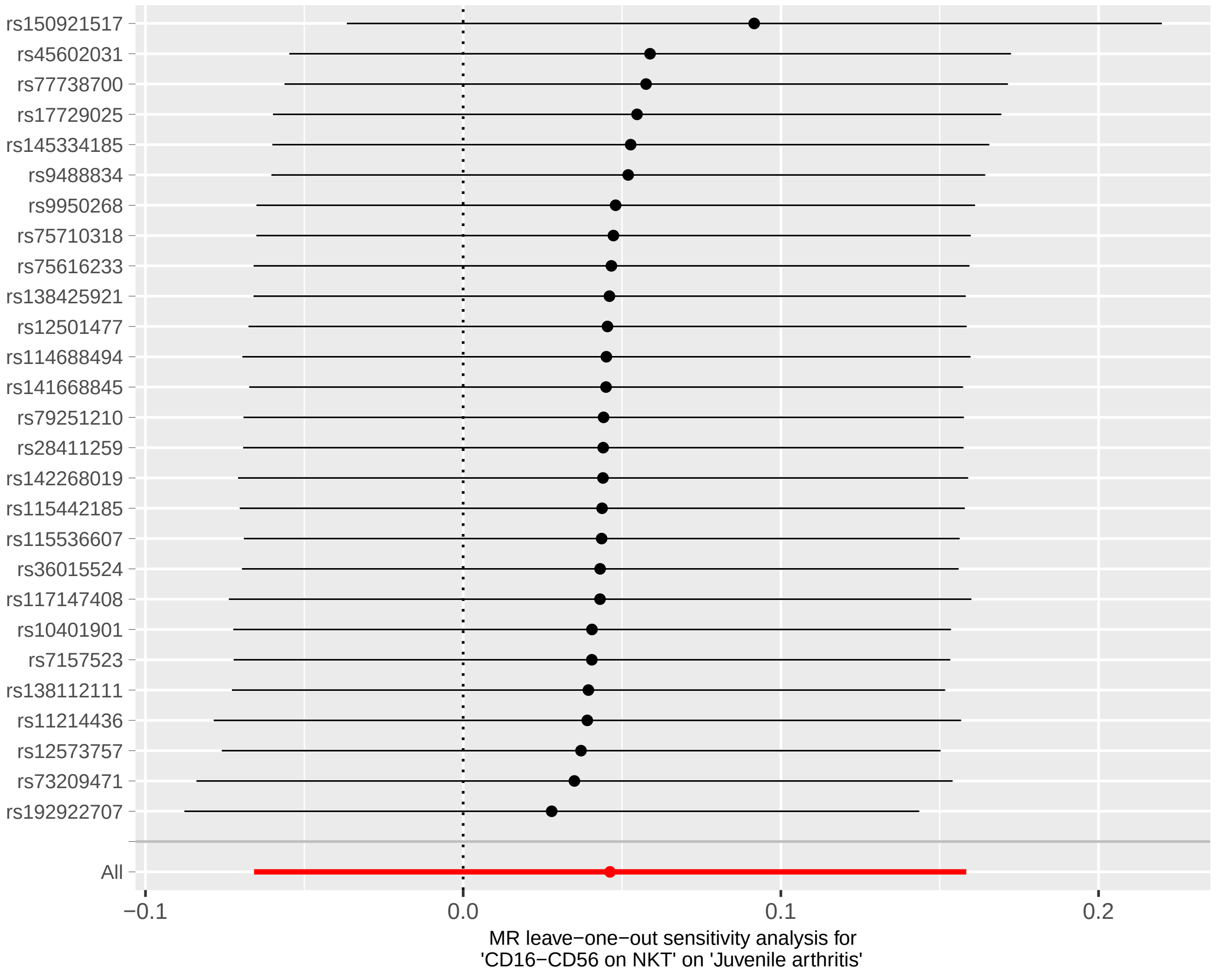
rs150921517
rs45602031
rs77738700
rs17729025
rs145334185
rs9488834
rs9950268
rs75710318
rs75616233
rs138425921
rs12501477
rs114688494
rs141668845
rs79251210
rs28411259
rs142268019
rs115442185
rs115536607
rs36015524
rs117147408
rs10401901
rs7157523
rs138112111
rs11214436
rs12573757
rs73209471
rs192922707
All
−0.1
0.0
0.1
0.2
MR leave−one−out sensitivity analysis for
'CD16−CD56 on NKT' on 'Juvenile arthritis'

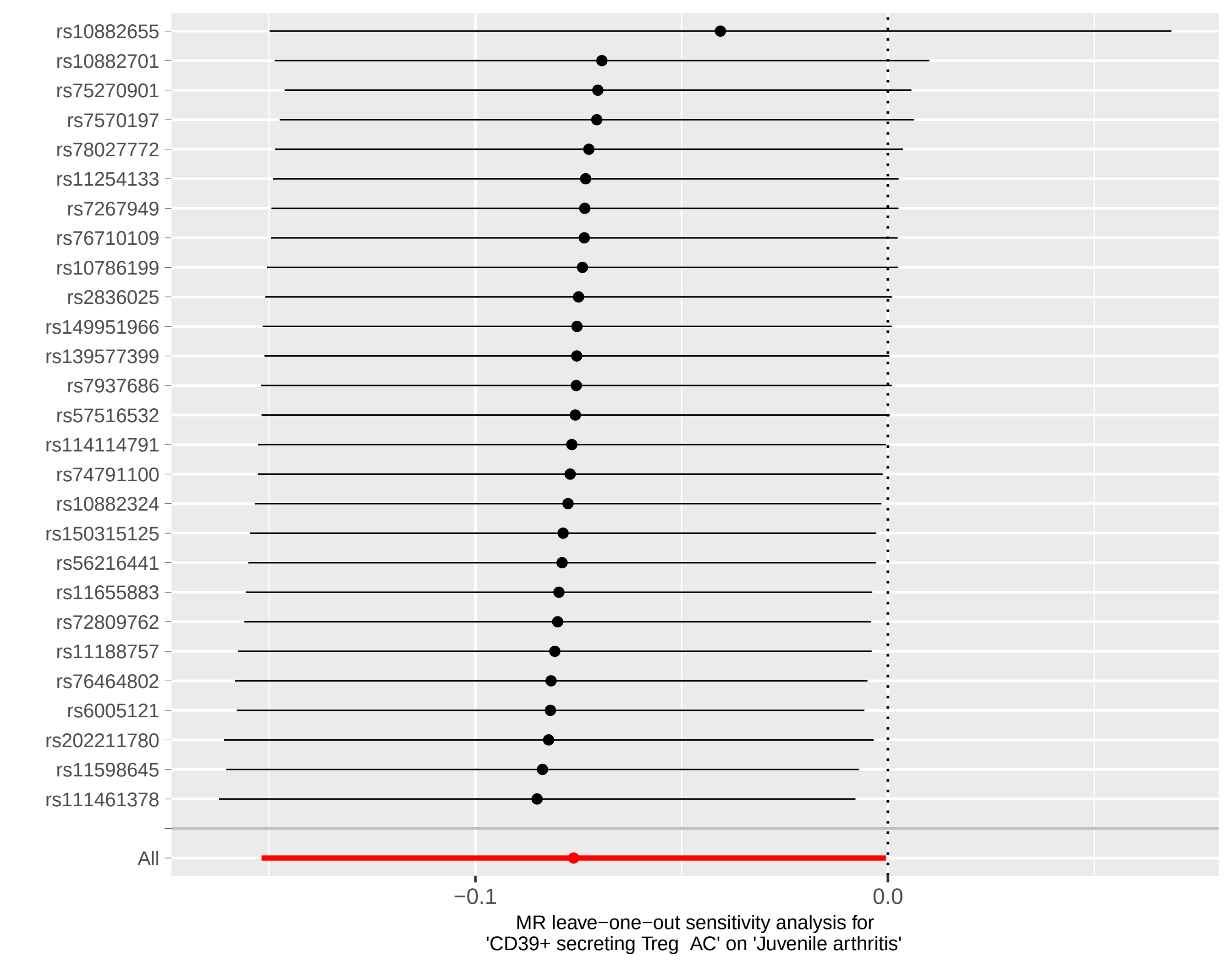
rs10882655
rs10882701
rs75270901
rs7570197
rs78027772
rs11254133
rs7267949
rs76710109
rs10786199
rs2836025
rs149951966
rs139577399
rs7937686
rs57516532
rs114114791
rs74791100
rs10882324
rs150315125
rs56216441
rs11655883
rs72809762
rs11188757
rs76464802
rs6005121
rs202211780
rs11598645
rs111461378
All
−0.1
0.0
MR leave−one−out sensitivity analysis for
'CD39+ secreting Treg AC' on 'Juvenile arthritis'

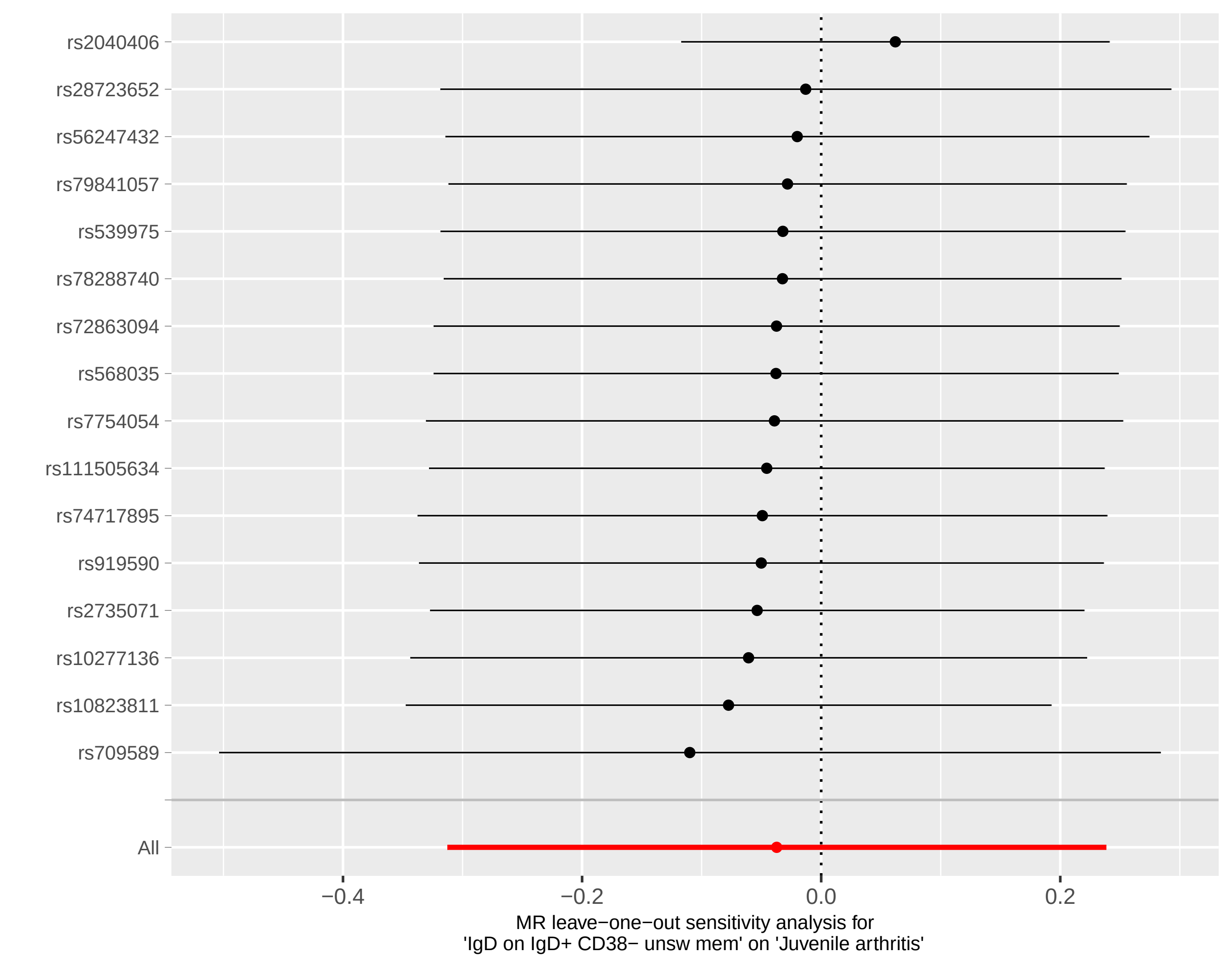

rs2040406
rs28723652
rs56247432
rs79841057
rs539975
rs78288740
rs72863094
rs568035
rs7754054
rs111505634
rs74717895
rs919590
rs2735071
rs10277136
rs10823811
rs709589
All
−0.4
−0.2
0.0
0.2
MR leave−one−out sensitivity analysis for
'IgD on IgD+ CD38− unsw mem' on 'Juvenile arthritis'

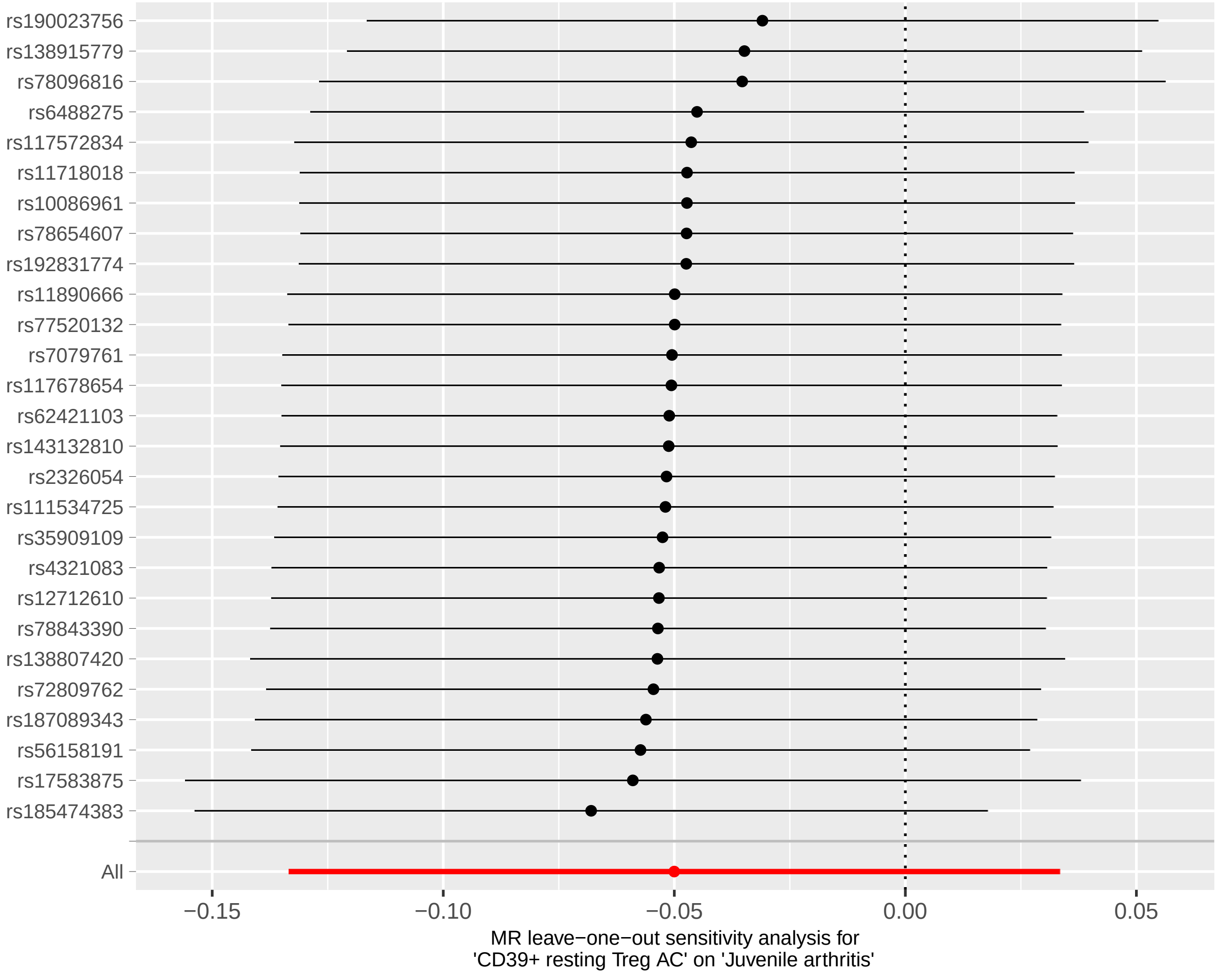

rs190023756
rs138915779
rs78096816
rs6488275
rs117572834
rs11718018
rs10086961
rs78654607
rs192831774
rs11890666
rs77520132
rs7079761
rs117678654
rs62421103
rs143132810
rs2326054
rs111534725
rs35909109
rs4321083
rs12712610
rs78843390
rs138807420
rs72809762
rs187089343
rs56158191
rs17583875
rs185474383
All
−0.15
−0.10
−0.05
0.00
0.05
MR leave−one−out sensitivity analysis for
'CD39+ resting Treg AC' on 'Juvenile arthritis'

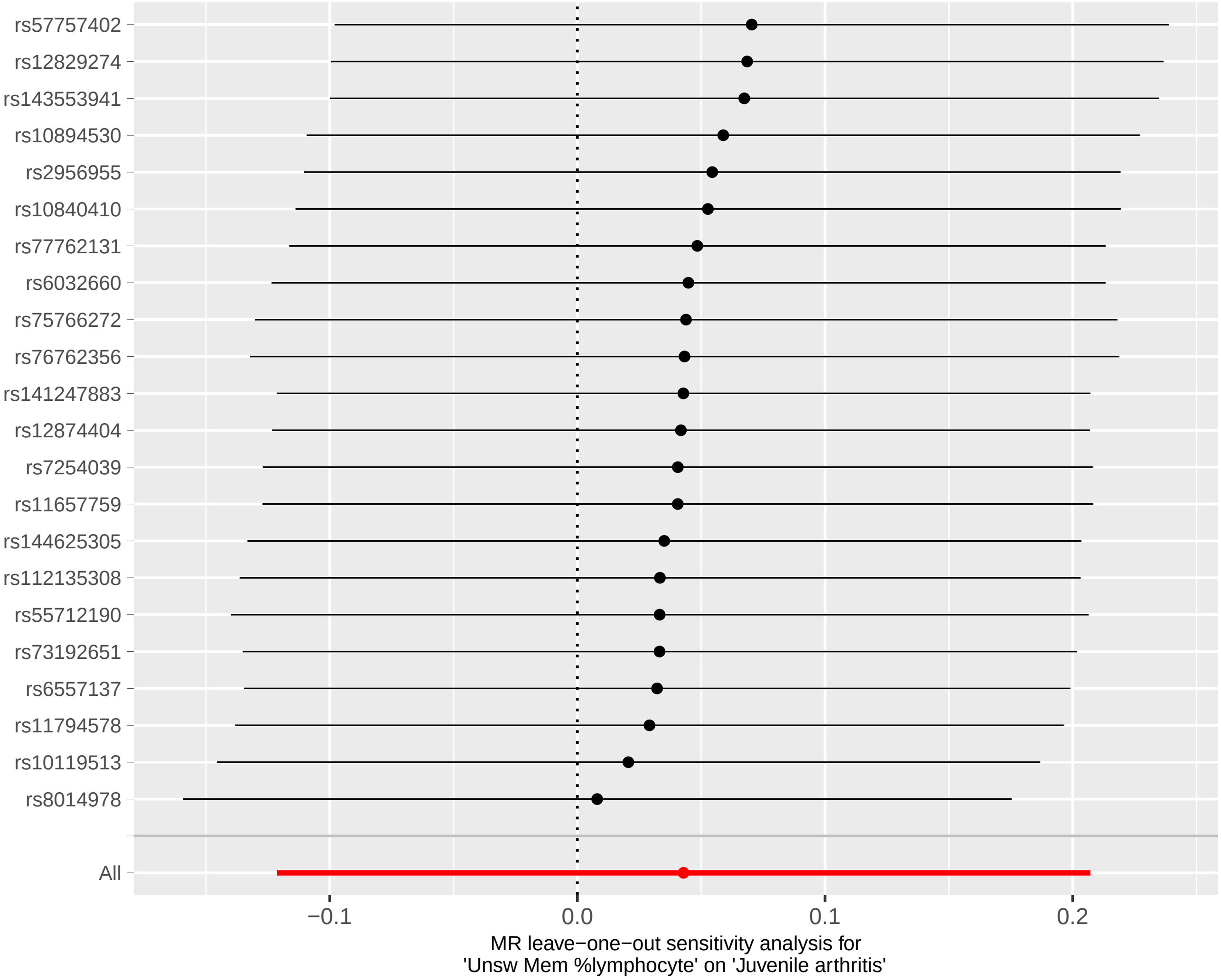

rs57757402
rs12829274
rs143553941
rs10894530
rs2956955
rs10840410
rs77762131
rs6032660
rs75766272
rs76762356
rs141247883
rs12874404
rs7254039
rs11657759
rs144625305
rs112135308
rs55712190
rs73192651
rs6557137
rs11794578
rs10119513
rs8014978
All
−0.1
0.0
0.1
0.2
MR leave−one−out sensitivity analysis for
'Unsw Mem %lymphocyte' on 'Juvenile arthritis'

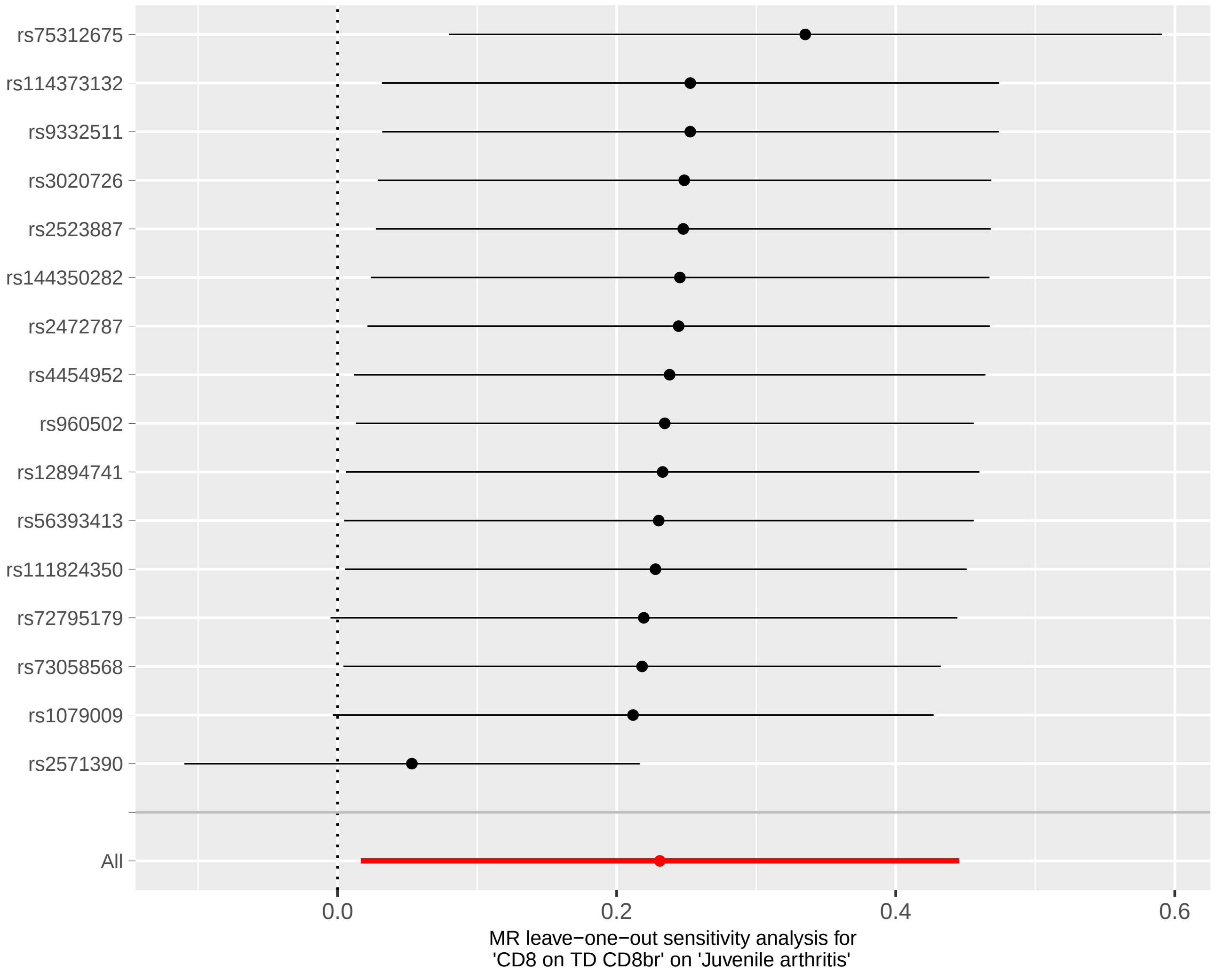
rs75312675
rs114373132
rs9332511
rs3020726
rs2523887
rs144350282
rs2472787
rs4454952
rs960502
rs12894741
rs56393413
rs111824350
rs72795179
rs73058568
rs1079009
rs2571390
All
0.0
0.2
0.4
0.6
MR leave−one−out sensitivity analysis for
'CD8 on TD CD8br' on 'Juvenile arthritis'

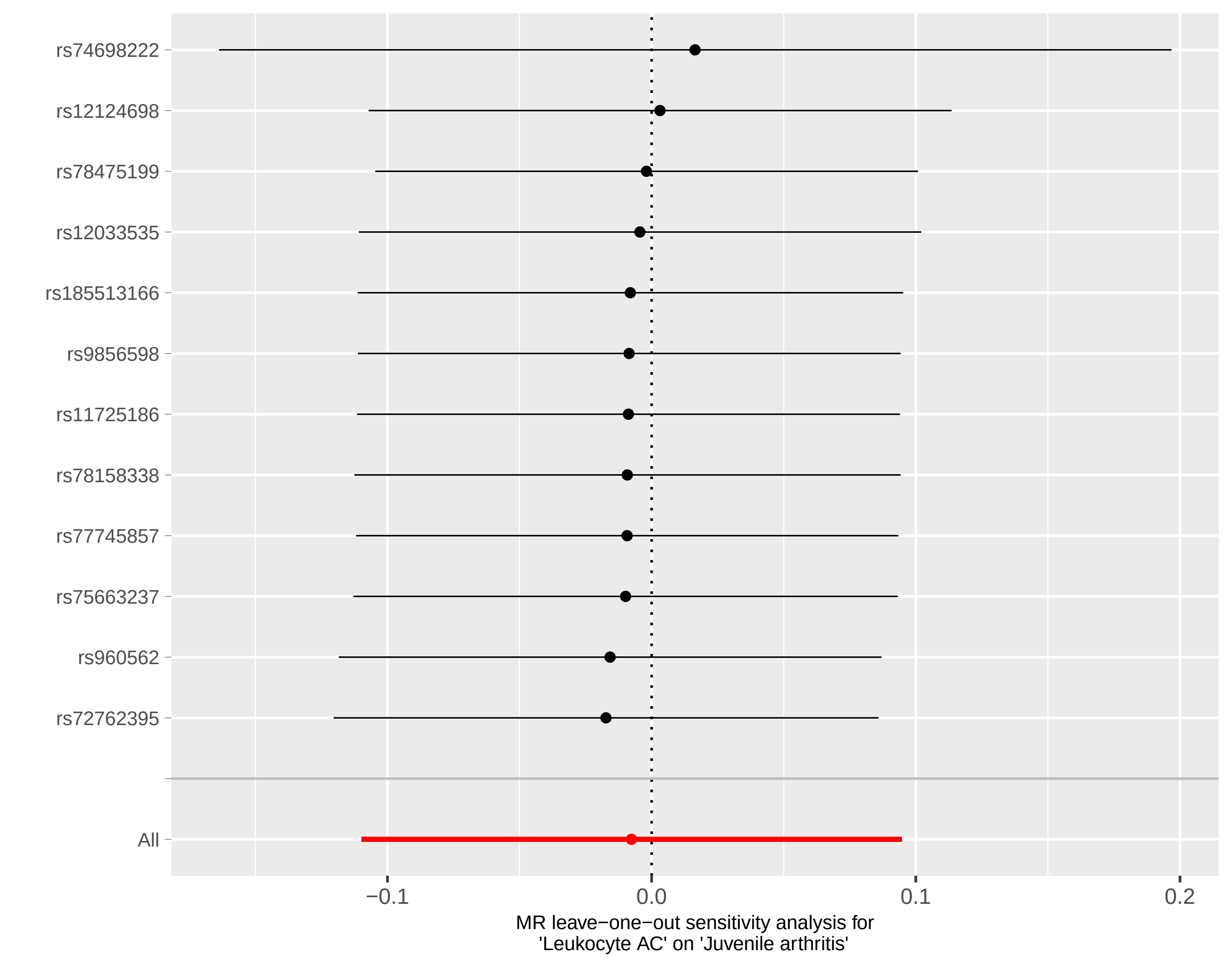
rs74698222
rs12124698
rs78475199
rs12033535
rs185513166
rs9856598
rs11725186
rs78158338
rs77745857
rs75663237
rs960562
rs72762395
All
−0.1
0.0
0.1
0.2
MR leave−one−out sensitivity analysis for
'Leukocyte AC' on 'Juvenile arthritis'

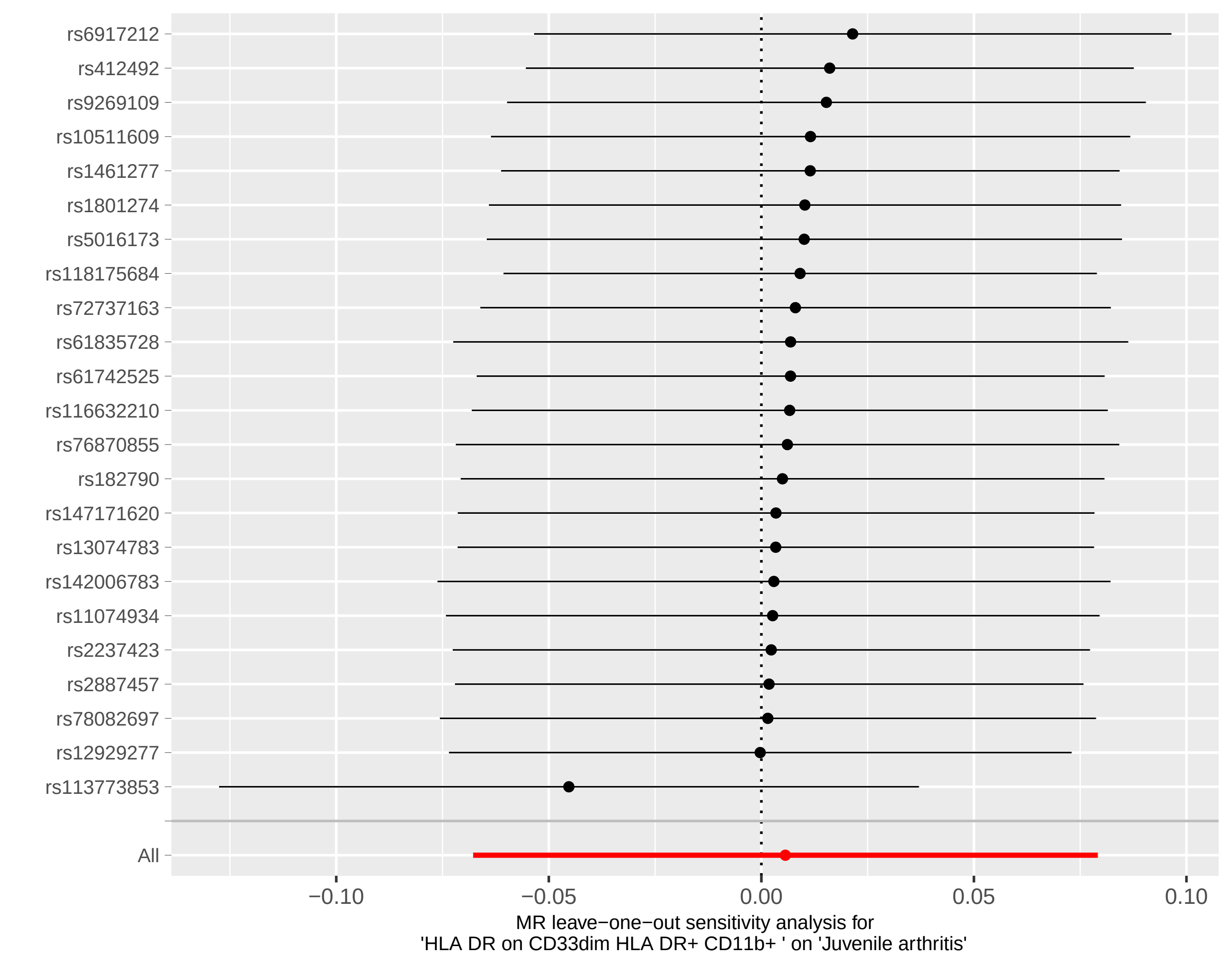
rs6917212
rs412492
rs9269109
rs10511609
rs1461277
rs1801274
rs5016173
rs118175684
rs72737163
rs61835728
rs61742525
rs116632210
rs76870855
rs182790
rs147171620
rs13074783
rs142006783
rs11074934
rs2237423
rs2887457
rs78082697
rs12929277
rs113773853
All
−0.10
−0.05
0.00
0.05
0.10
MR leave−one−out sensitivity analysis for
'HLA DR on CD33dim HLA DR+ CD11b+ ' on 'Juvenile arthritis'

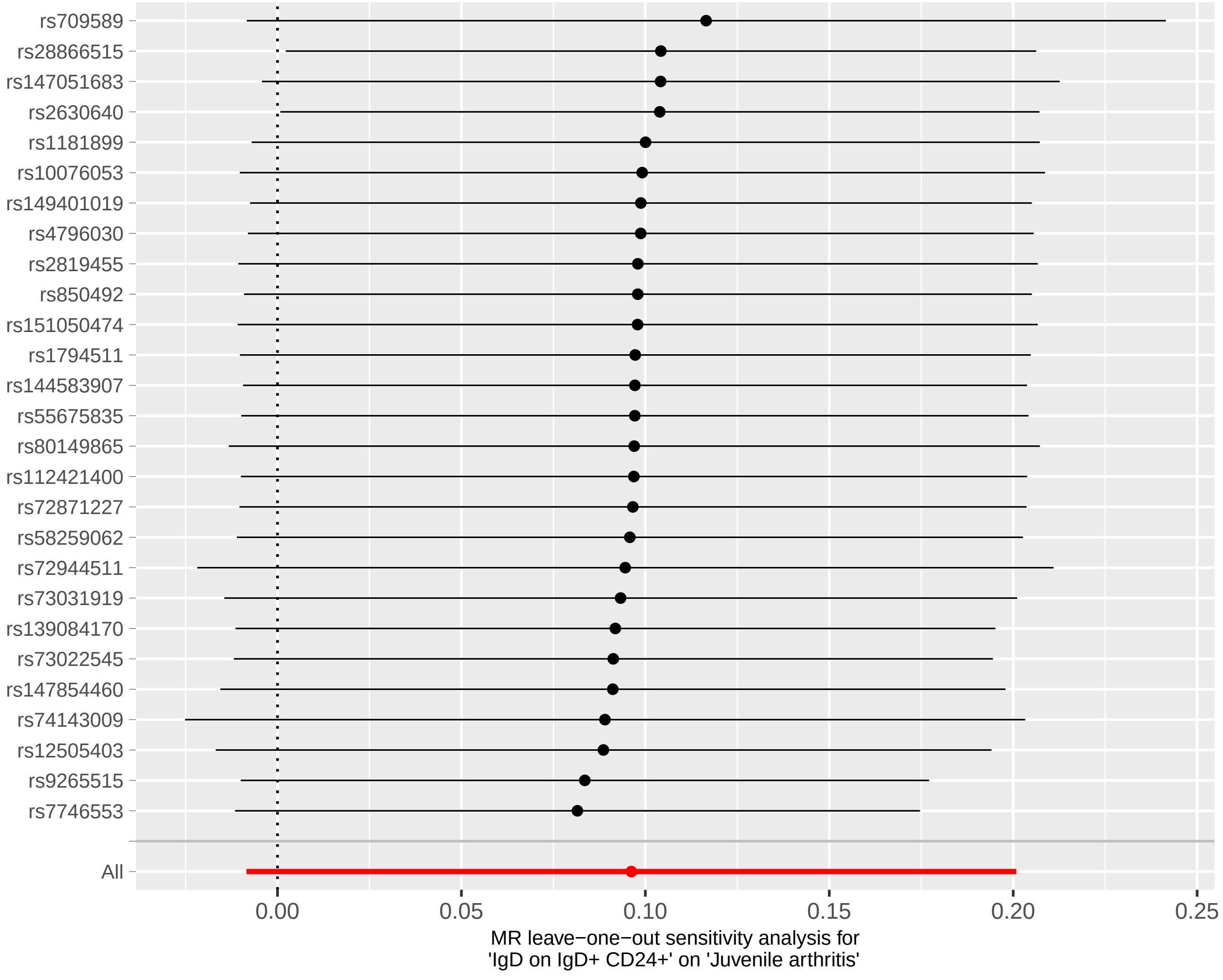
rs709589
rs28866515
rs147051683
rs2630640
rs1181899
rs10076053
rs149401019
rs4796030
rs2819455
rs850492
rs151050474
rs1794511
rs144583907
rs55675835
rs80149865
rs112421400
rs72871227
rs58259062
rs72944511
rs73031919
rs139084170
rs73022545
rs147854460
rs74143009
rs12505403
rs9265515
rs7746553
All
0.00
0.05
0.10
0.15
0.20
0.25
MR leave−one−out sensitivity analysis for
'IgD on IgD+ CD24+' on 'Juvenile arthritis'

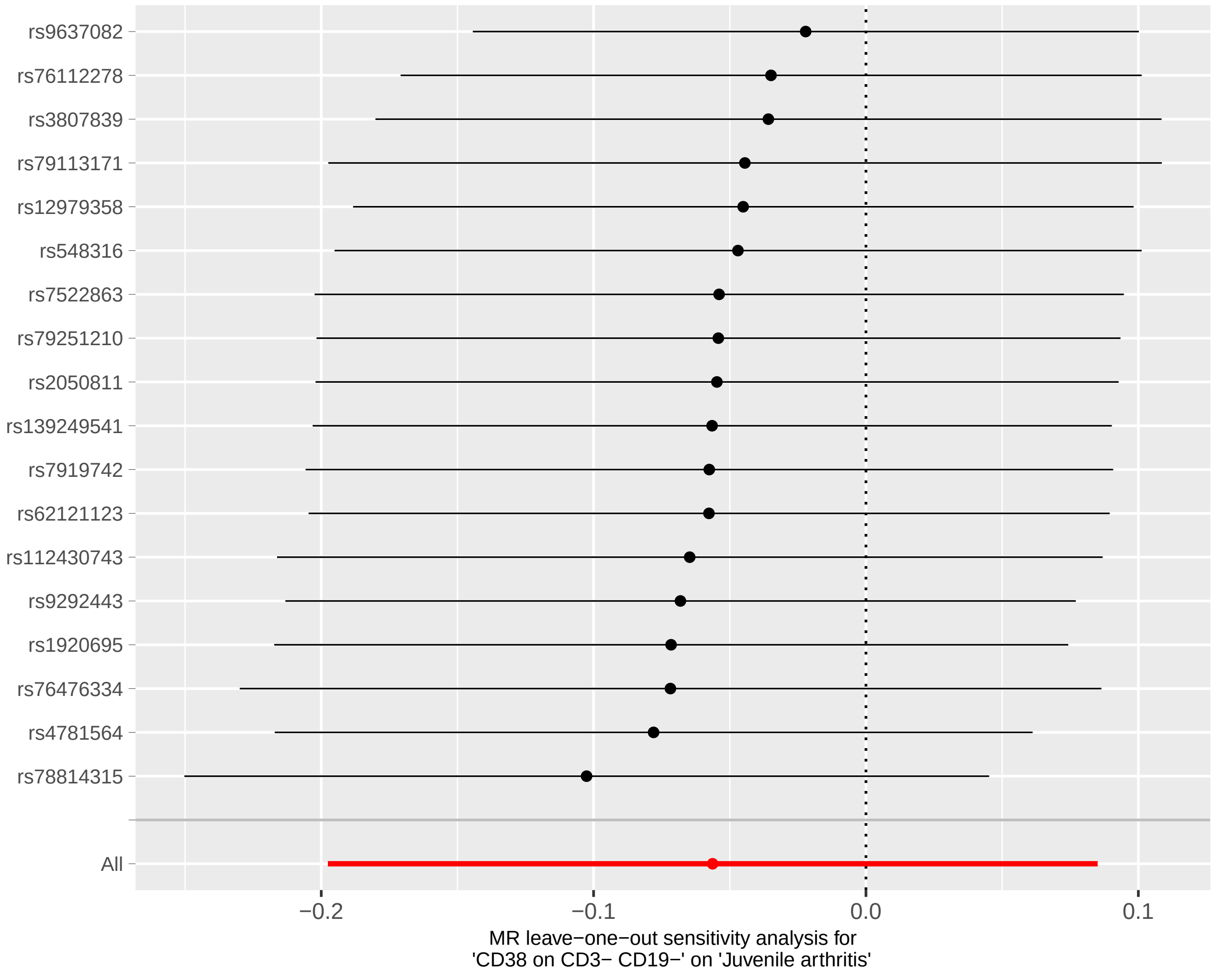
rs9637082
rs76112278
rs3807839
rs79113171
rs12979358
rs548316
rs7522863
rs79251210
rs2050811
rs139249541
rs7919742
rs62121123
rs112430743
rs9292443
rs1920695
rs76476334
rs4781564
rs78814315
All
−0.2
−0.1
0.0
0.1
MR leave−one−out sensitivity analysis for
'CD38 on CD3− CD19−' on 'Juvenile arthritis'

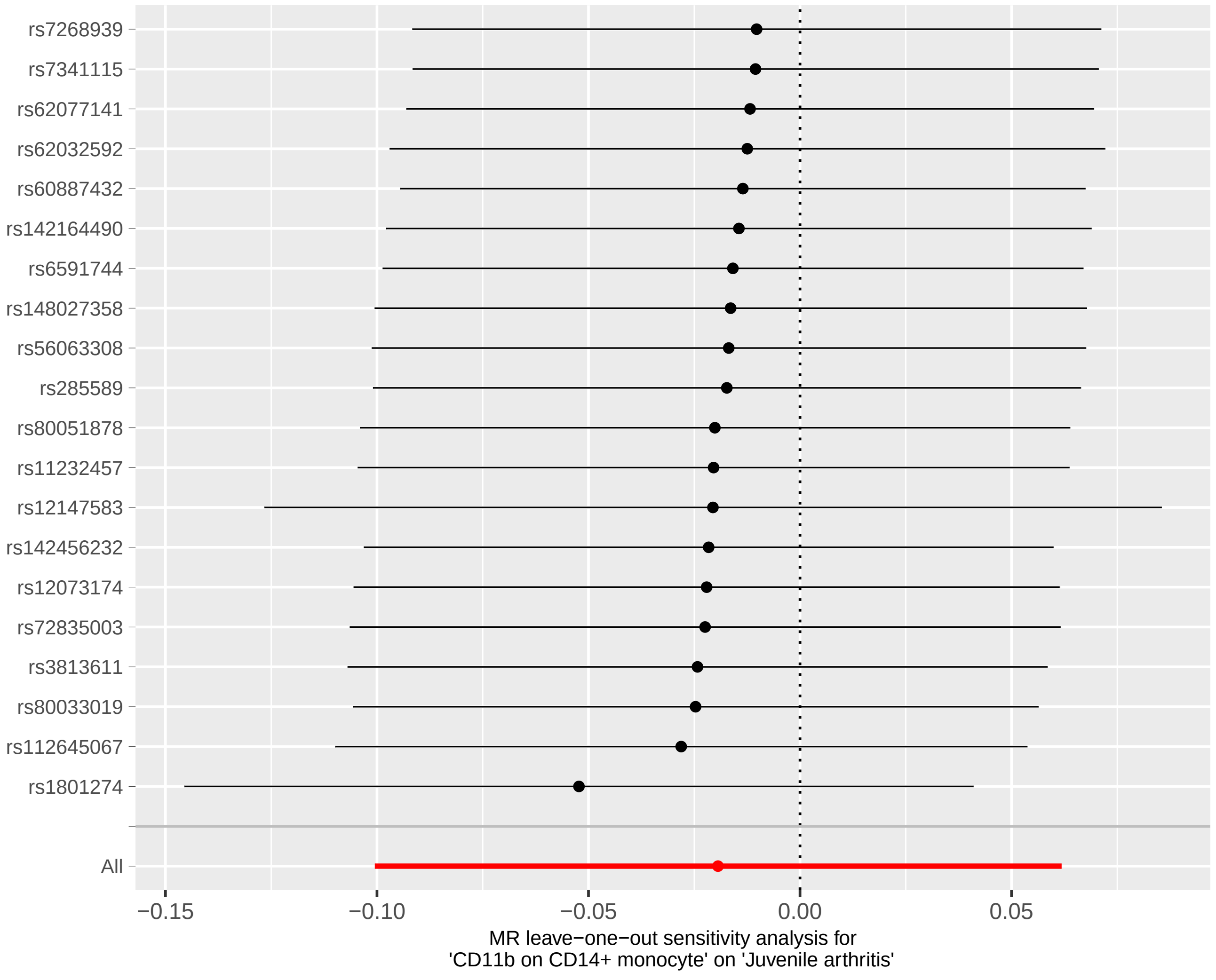
rs7268939
rs7341115
rs62077141
rs62032592
rs60887432
rs142164490
rs6591744
rs148027358
rs56063308
rs285589
rs80051878
rs11232457
rs12147583
rs142456232
rs12073174
rs72835003
rs3813611
rs80033019
rs112645067
rs1801274
All
−0.15
−0.10
−0.05
0.00
0.05
MR leave−one−out sensitivity analysis for
'CD11b on CD14+ monocyte' on 'Juvenile arthritis'

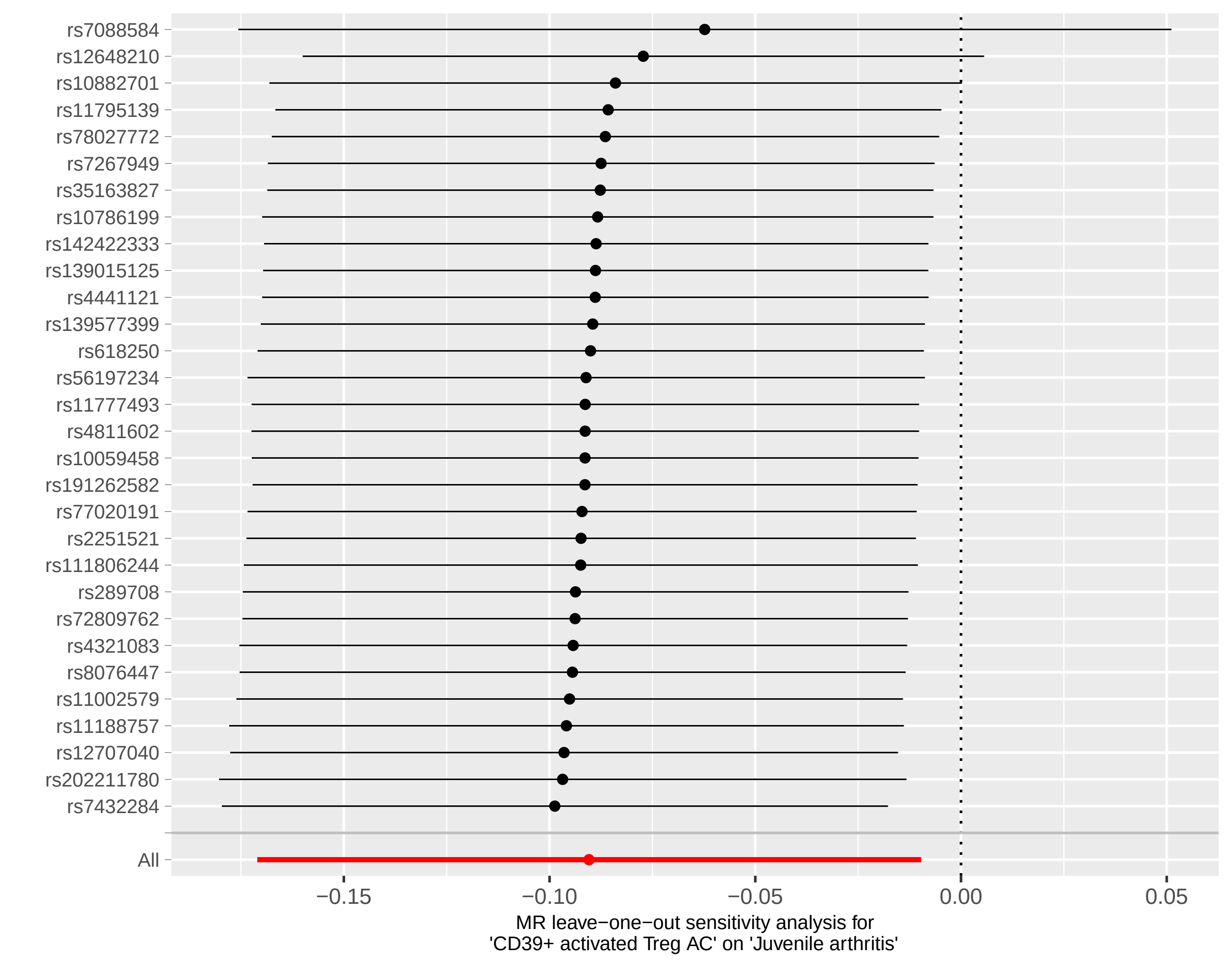
rs7088584
rs12648210
rs10882701
rs11795139
rs78027772
rs7267949
rs35163827
rs10786199
rs142422333
rs139015125
rs4441121
rs139577399
rs618250
rs56197234
rs11777493
rs4811602
rs10059458
rs191262582
rs77020191
rs2251521
rs111806244
rs289708
rs72809762
rs4321083
rs8076447
rs11002579
rs11188757
rs12707040
rs202211780
rs7432284
All
−0.15
−0.10
−0.05
0.00
0.05
MR leave−one−out sensitivity analysis for
'CD39+ activated Treg AC' on 'Juvenile arthritis'

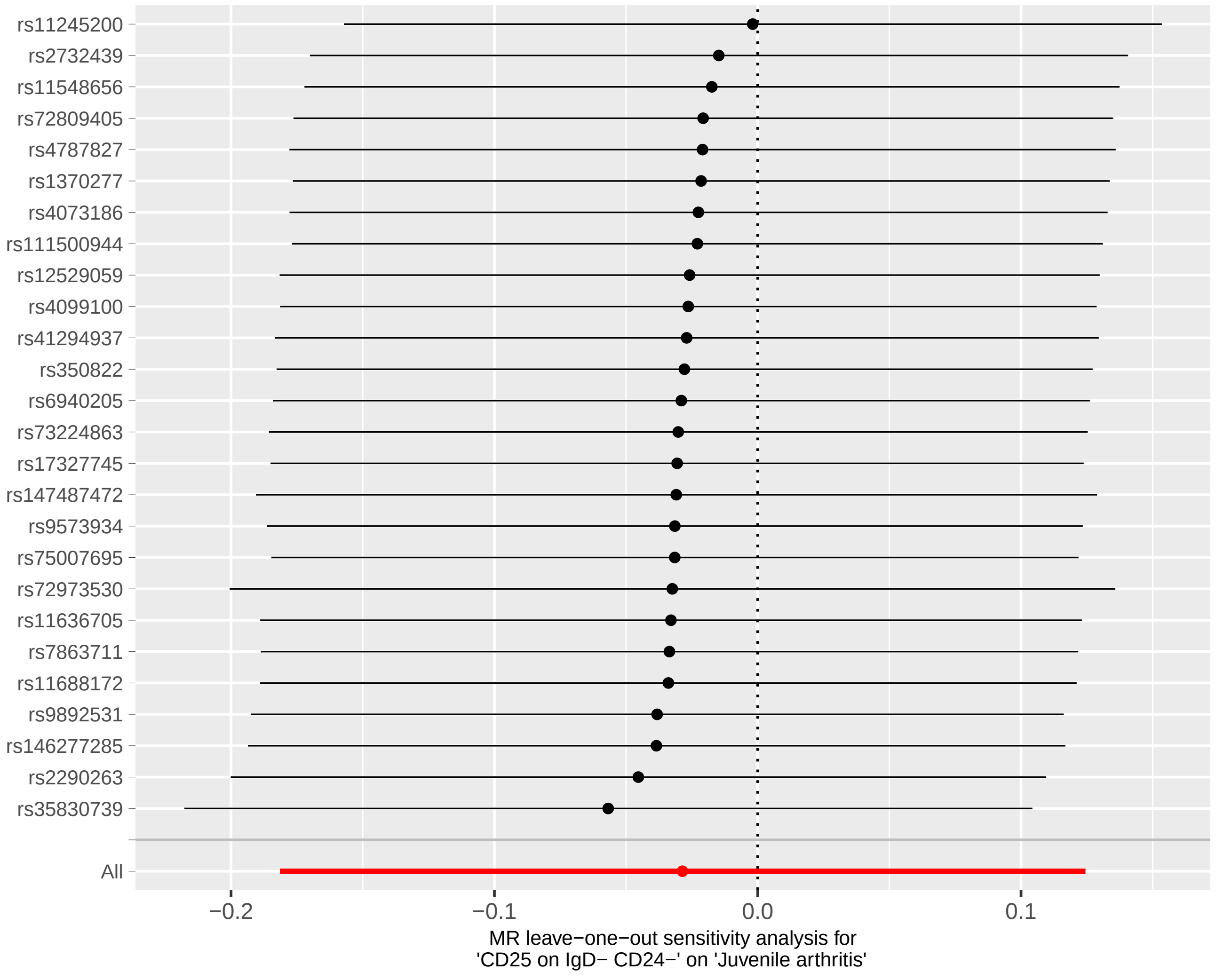
rs11245200
rs2732439
rs11548656
rs72809405
rs4787827
rs1370277
rs4073186
rs111500944
rs12529059
rs4099100
rs41294937
rs350822
rs6940205
rs73224863
rs17327745
rs147487472
rs9573934
rs75007695
rs72973530
rs11636705
rs7863711
rs11688172
rs9892531
rs146277285
rs2290263
rs35830739
All
−0.2
−0.1
0.0
0.1
MR leave−one−out sensitivity analysis for
'CD25 on IgD− CD24−' on 'Juvenile arthritis'

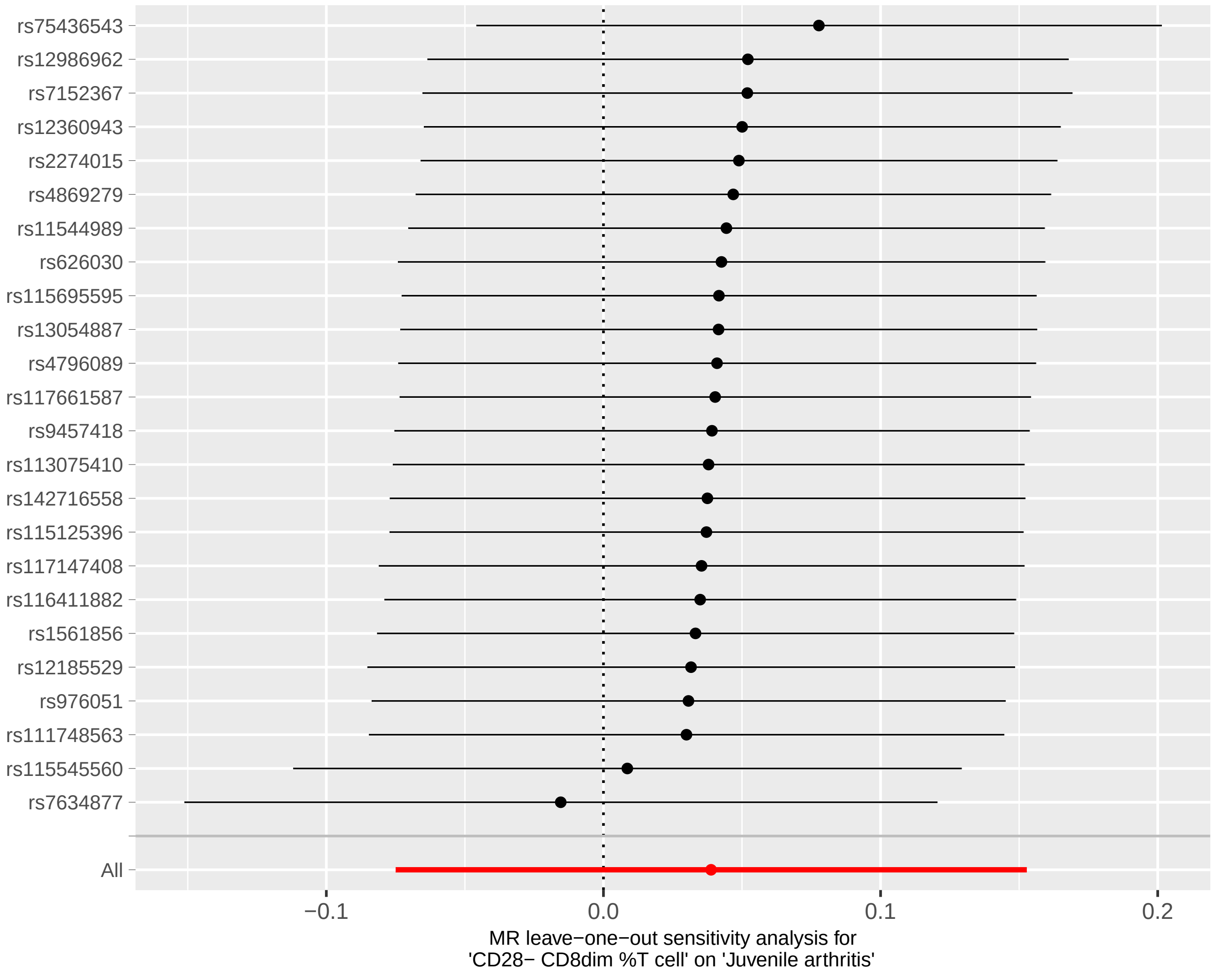
rs75436543
rs12986962
rs7152367
rs12360943
rs2274015
rs4869279
rs11544989
rs626030
rs115695595
rs13054887
rs4796089
rs117661587
rs9457418
rs113075410
rs142716558
rs115125396
rs117147408
rs116411882
rs1561856
rs12185529
rs976051
rs111748563
rs115545560
rs7634877
All
−0.1
0.0
0.1
0.2
MR leave−one−out sensitivity analysis for
'CD28− CD8dim %T cell' on 'Juvenile arthritis'

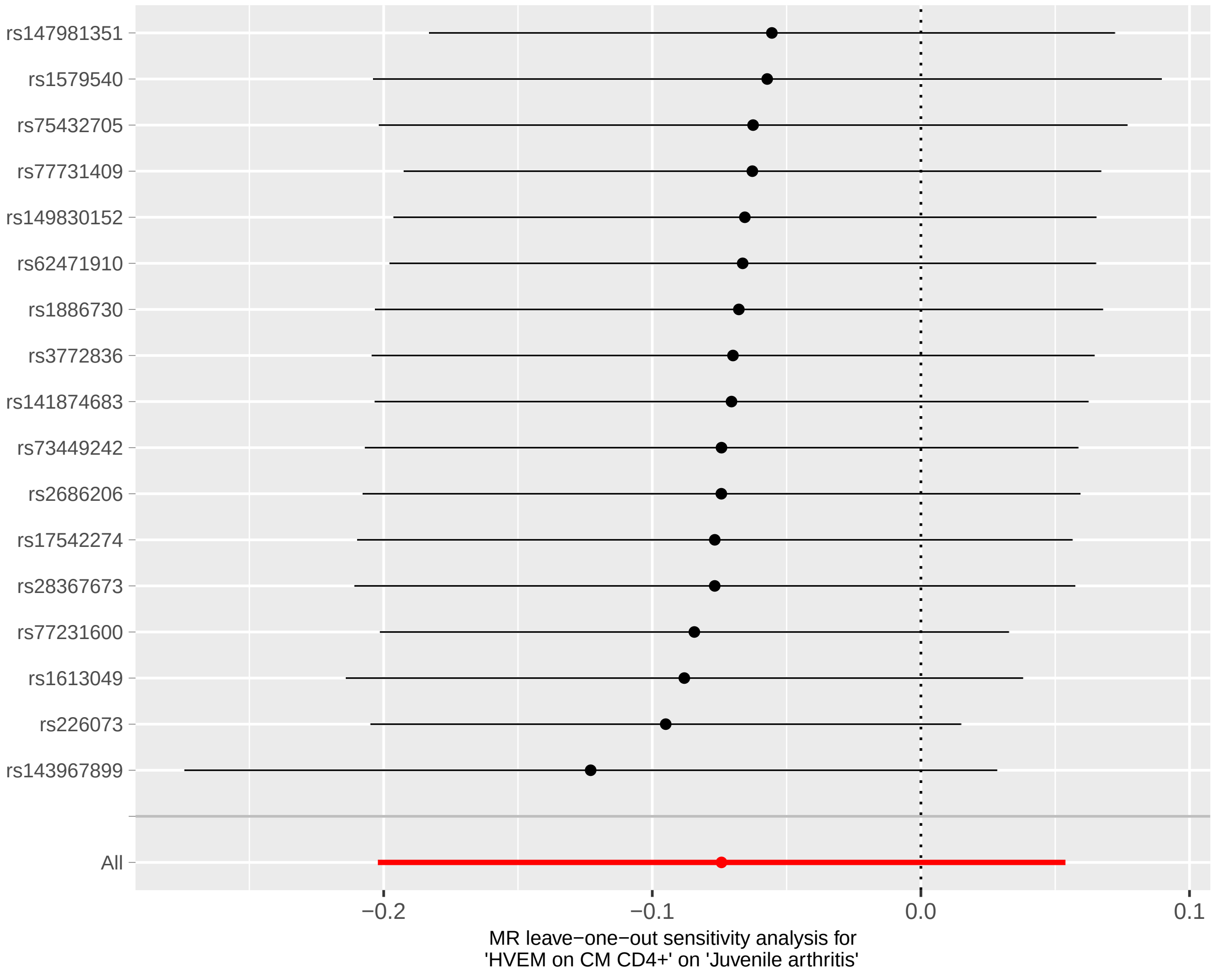
rs147981351
rs1579540
rs75432705
rs77731409
rs149830152
rs62471910
rs1886730
rs3772836
rs141874683
rs73449242
rs2686206
rs17542274
rs28367673
rs77231600
rs1613049
rs226073
rs143967899
All
−0.2
−0.1
0.0
0.1
MR leave−one−out sensitivity analysis for
'HVEM on CM CD4+' on 'Juvenile arthritis'

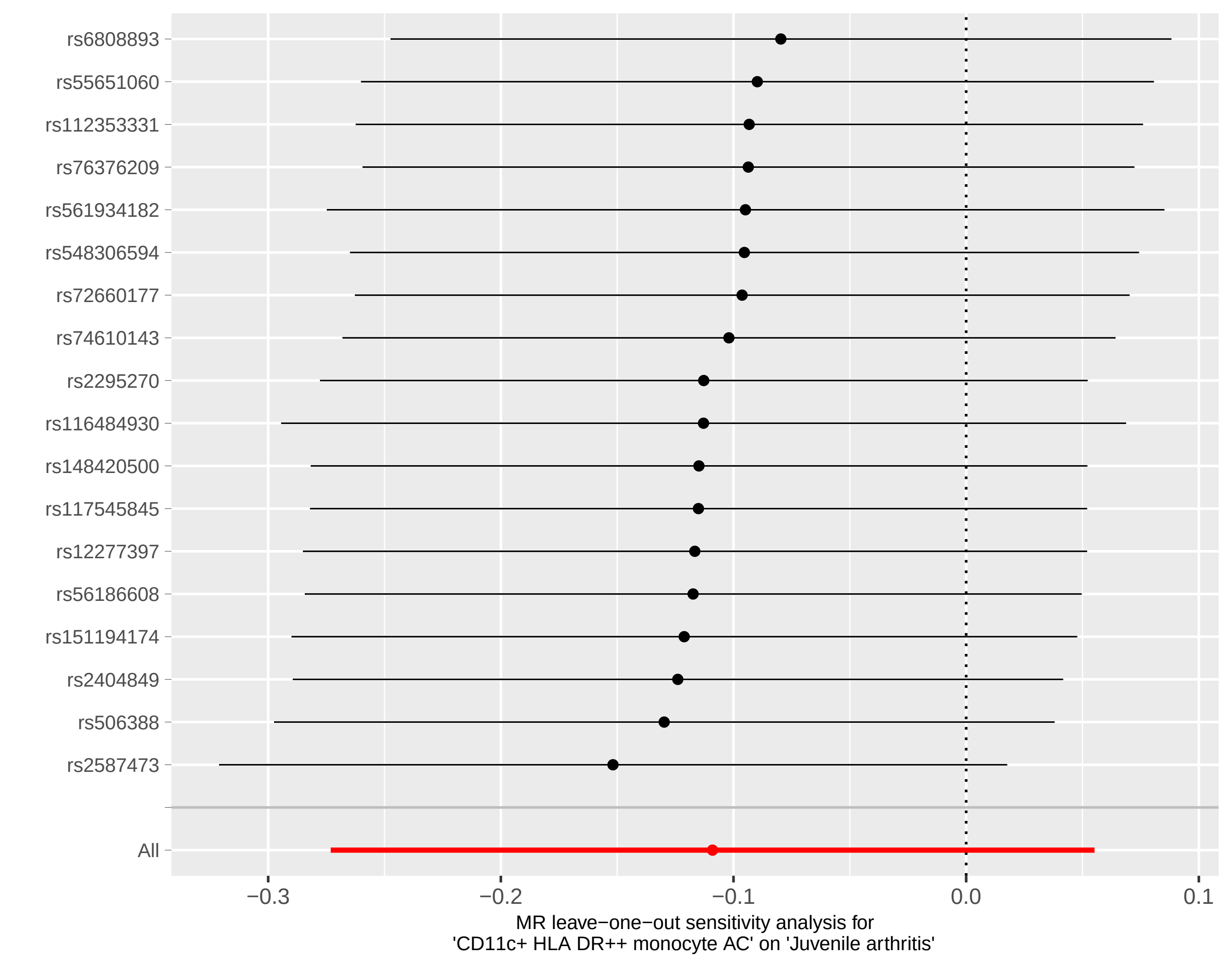
rs6808893
rs55651060
rs112353331
rs76376209
rs561934182
rs548306594
rs72660177
rs74610143
rs2295270
rs116484930
rs148420500
rs117545845
rs12277397
rs56186608
rs151194174
rs2404849
rs506388
rs2587473
All
−0.3
−0.2
−0.1
0.0
0.1
MR leave−one−out sensitivity analysis for
'CD11c+ HLA DR++ monocyte AC' on 'Juvenile arthritis'

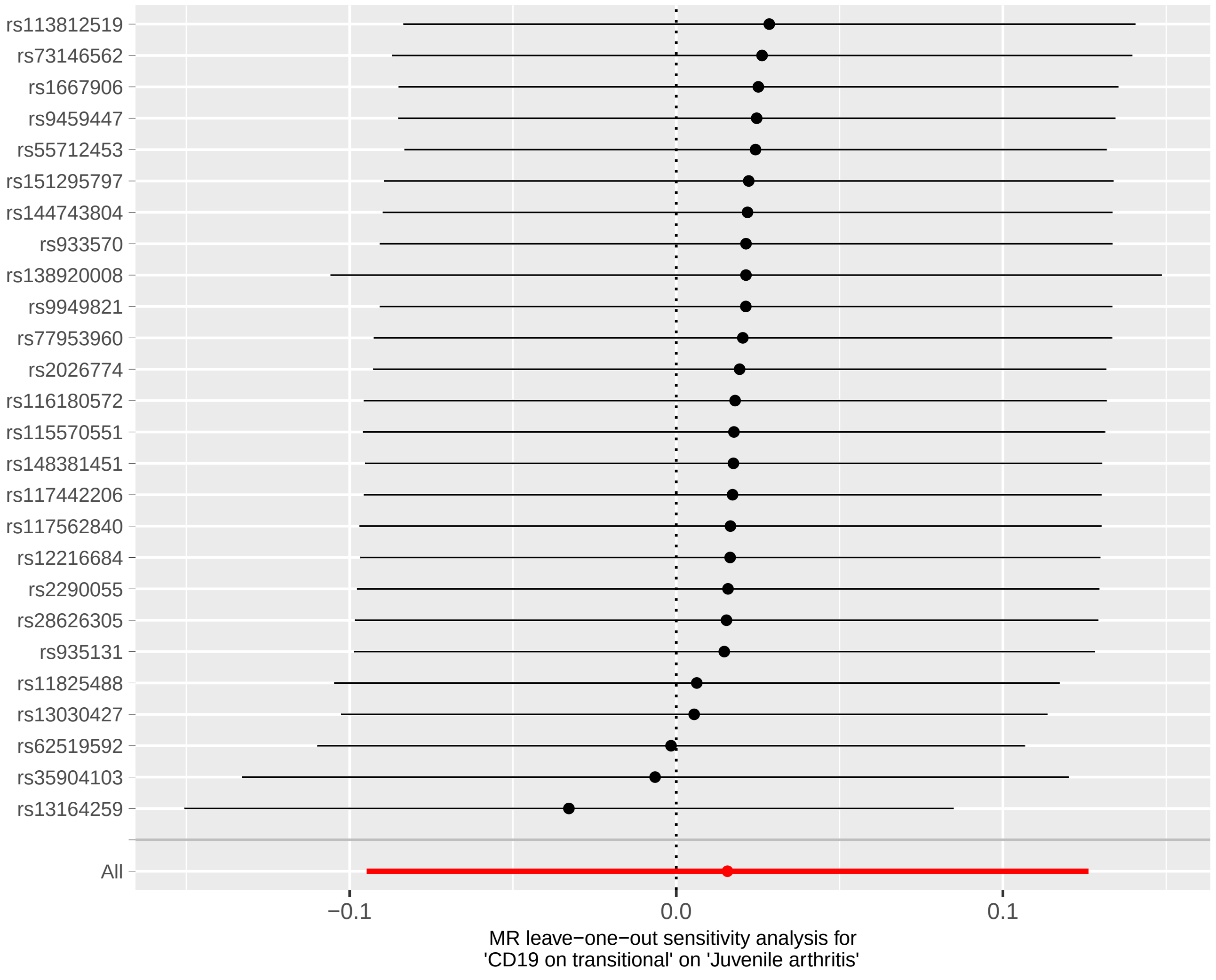
rs113812519
rs73146562
rs1667906
rs9459447
rs55712453
rs151295797
rs144743804
rs933570
rs138920008
rs9949821
rs77953960
rs2026774
rs116180572
rs115570551
rs148381451
rs117442206
rs117562840
rs12216684
rs2290055
rs28626305
rs935131
rs11825488
rs13030427
rs62519592
rs35904103
rs13164259
All
−0.1
0.0
0.1
MR leave−one−out sensitivity analysis for
'CD19 on transitional' on 'Juvenile arthritis'

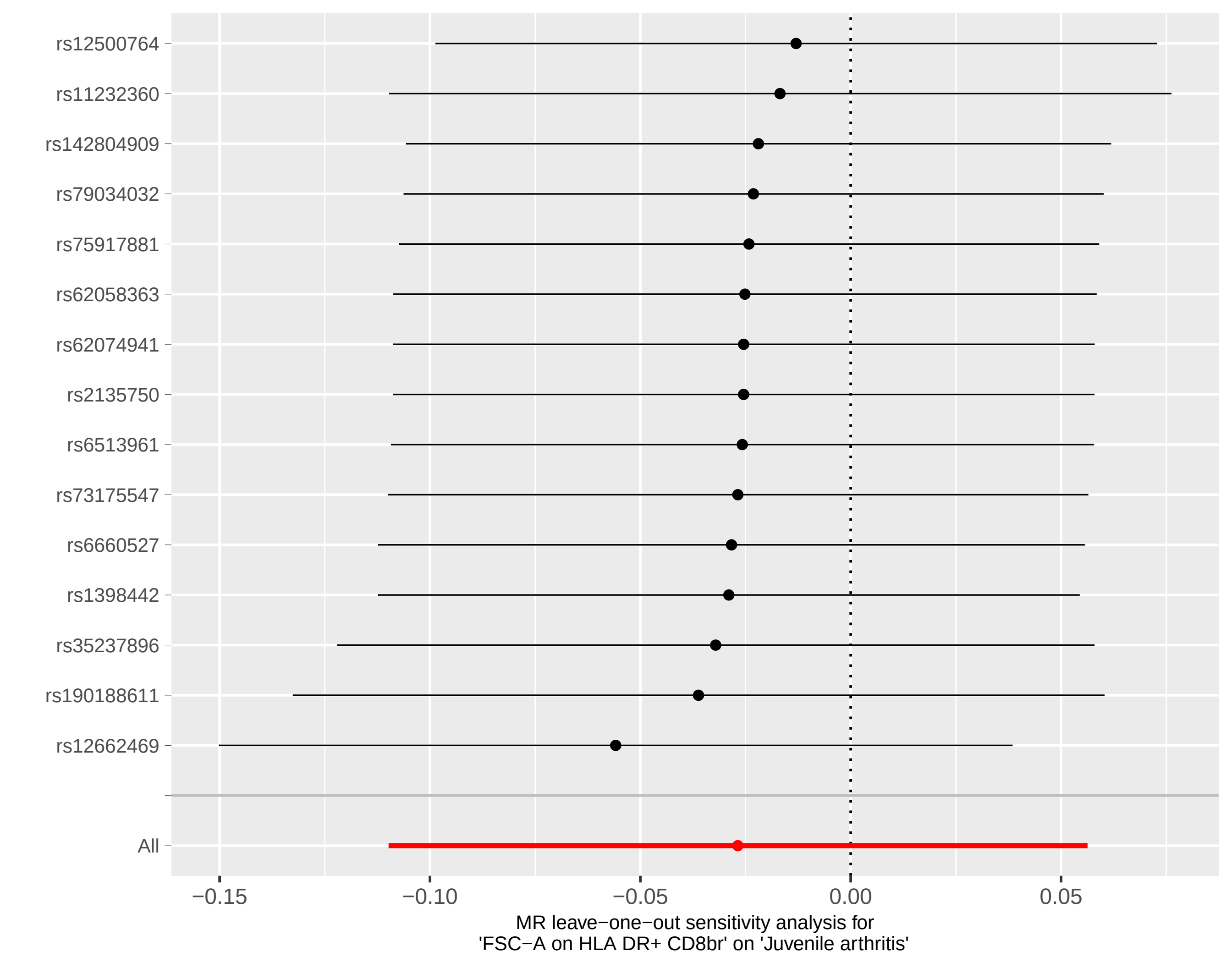
rs12500764
rs11232360
rs142804909
rs79034032
rs75917881
rs62058363
rs62074941
rs2135750
rs6513961
rs73175547
rs6660527
rs1398442
rs35237896
rs190188611
rs12662469
All
−0.15
−0.10
−0.05
0.00
0.05
MR leave−one−out sensitivity analysis for
'FSC−A on HLA DR+ CD8br' on 'Juvenile arthritis'

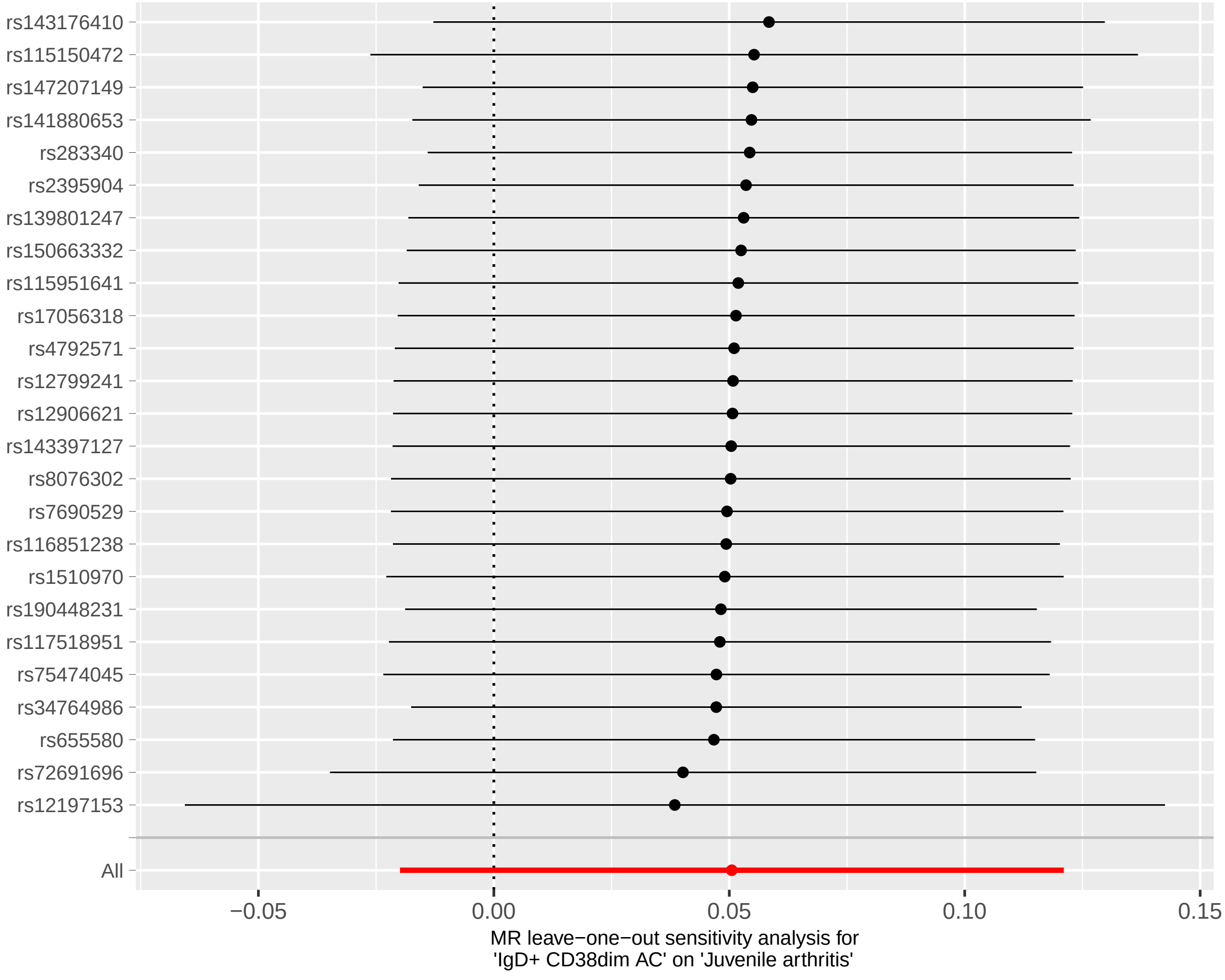
rs143176410
rs115150472
rs147207149
rs141880653
rs283340
rs2395904
rs139801247
rs150663332
rs115951641
rs17056318
rs4792571
rs12799241
rs12906621
rs143397127
rs8076302
rs7690529
rs116851238
rs1510970
rs190448231
rs117518951
rs75474045
rs34764986
rs655580
rs72691696
rs12197153
All
−0.05
0.00
0.05
0.10
0.15
MR leave−one−out sensitivity analysis for
'IgD+ CD38dim AC' on 'Juvenile arthritis'

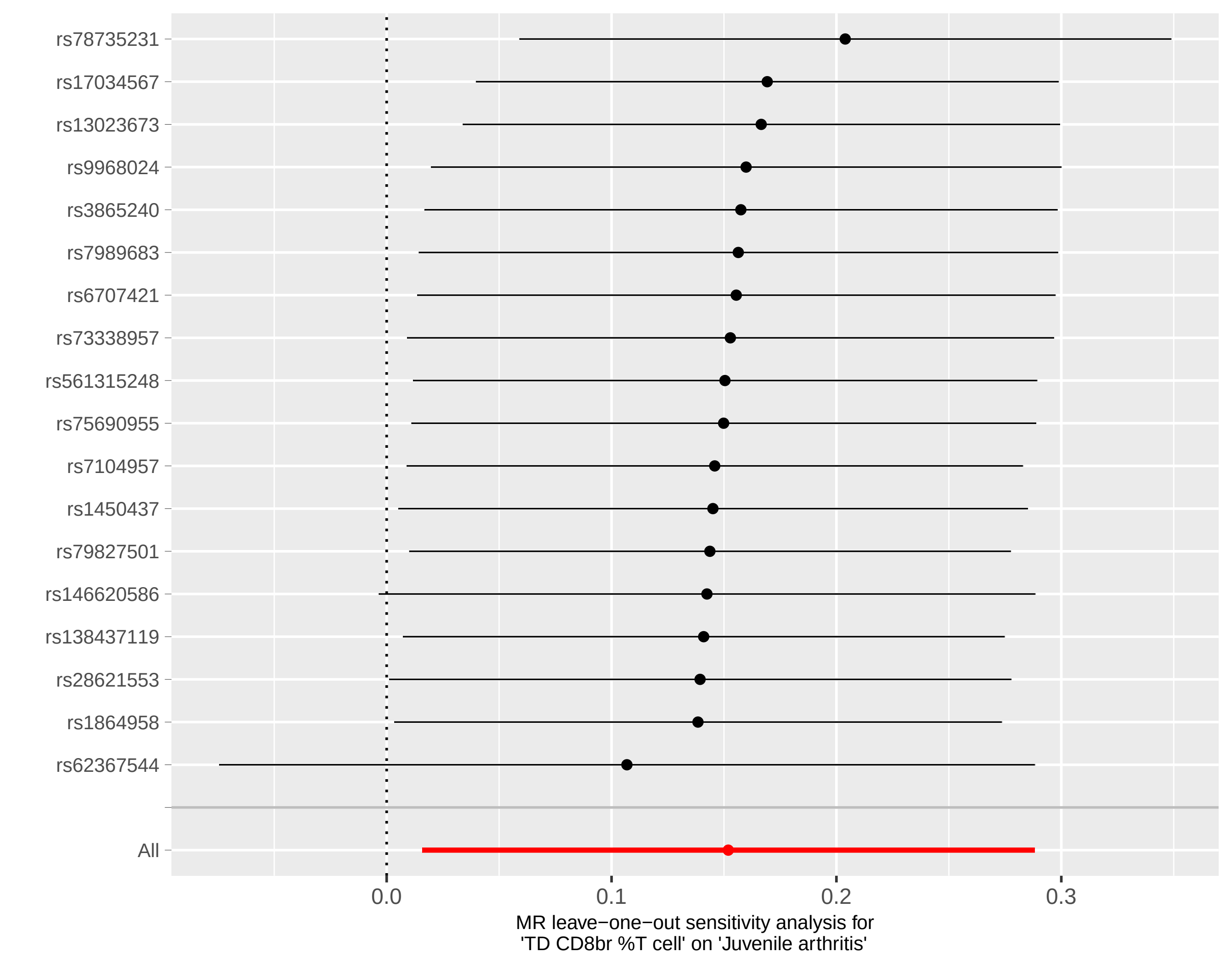
rs78735231
rs17034567
rs13023673
rs9968024
rs3865240
rs7989683
rs6707421
rs73338957
rs561315248
rs75690955
rs7104957
rs1450437
rs79827501
rs146620586
rs138437119
rs28621553
rs1864958
rs62367544
All
0.0
0.1
0.2
0.3
MR leave−one−out sensitivity analysis for
'TD CD8br %T cell' on 'Juvenile arthritis'

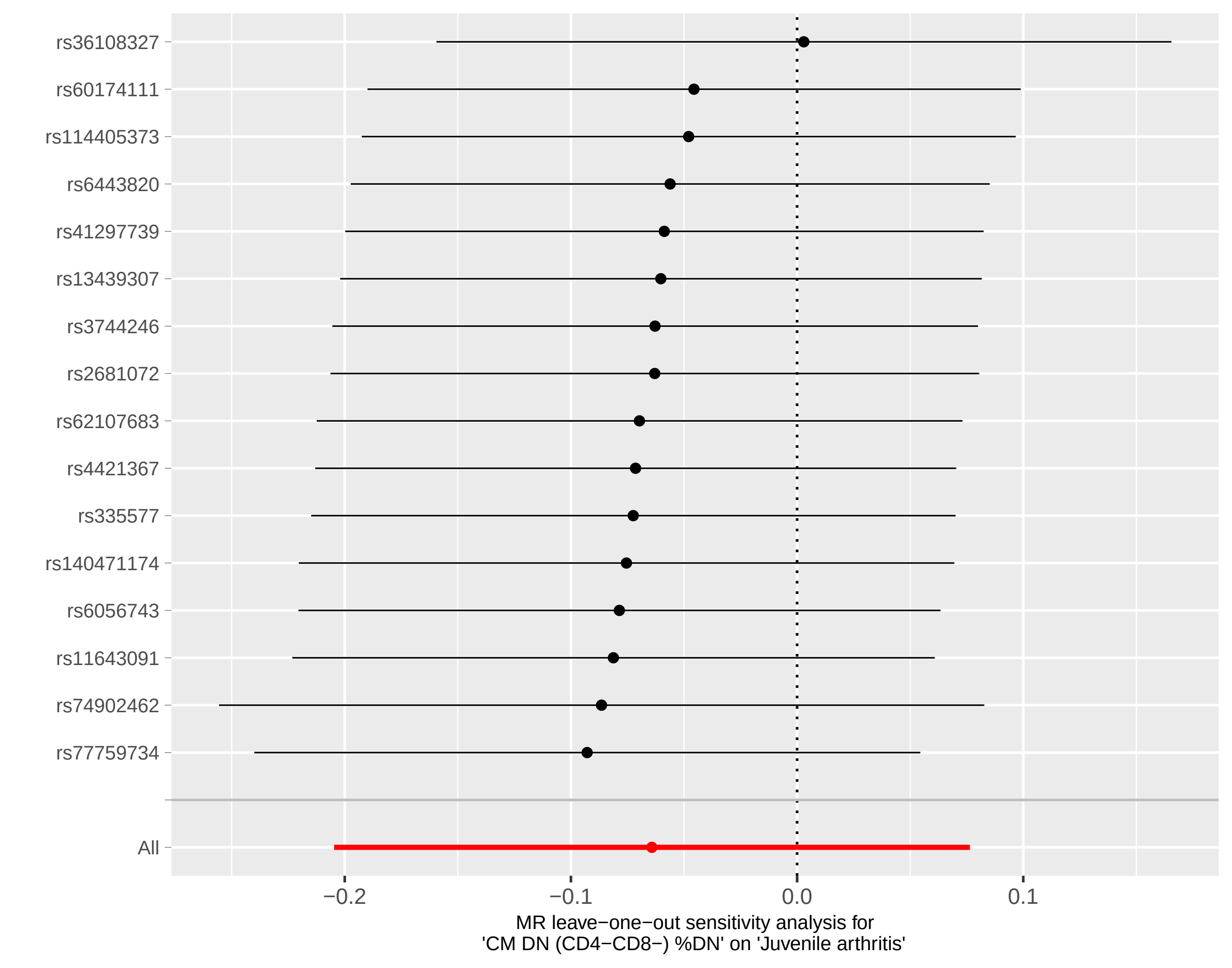
rs36108327
rs60174111
rs114405373
rs6443820
rs41297739
rs13439307
rs3744246
rs2681072
rs62107683
rs4421367
rs335577
rs140471174
rs6056743
rs11643091
rs74902462
rs77759734
All
−0.2
−0.1
0.0
0.1
MR leave−one−out sensitivity analysis for
'CM DN (CD4−CD8−) %DN' on 'Juvenile arthritis'

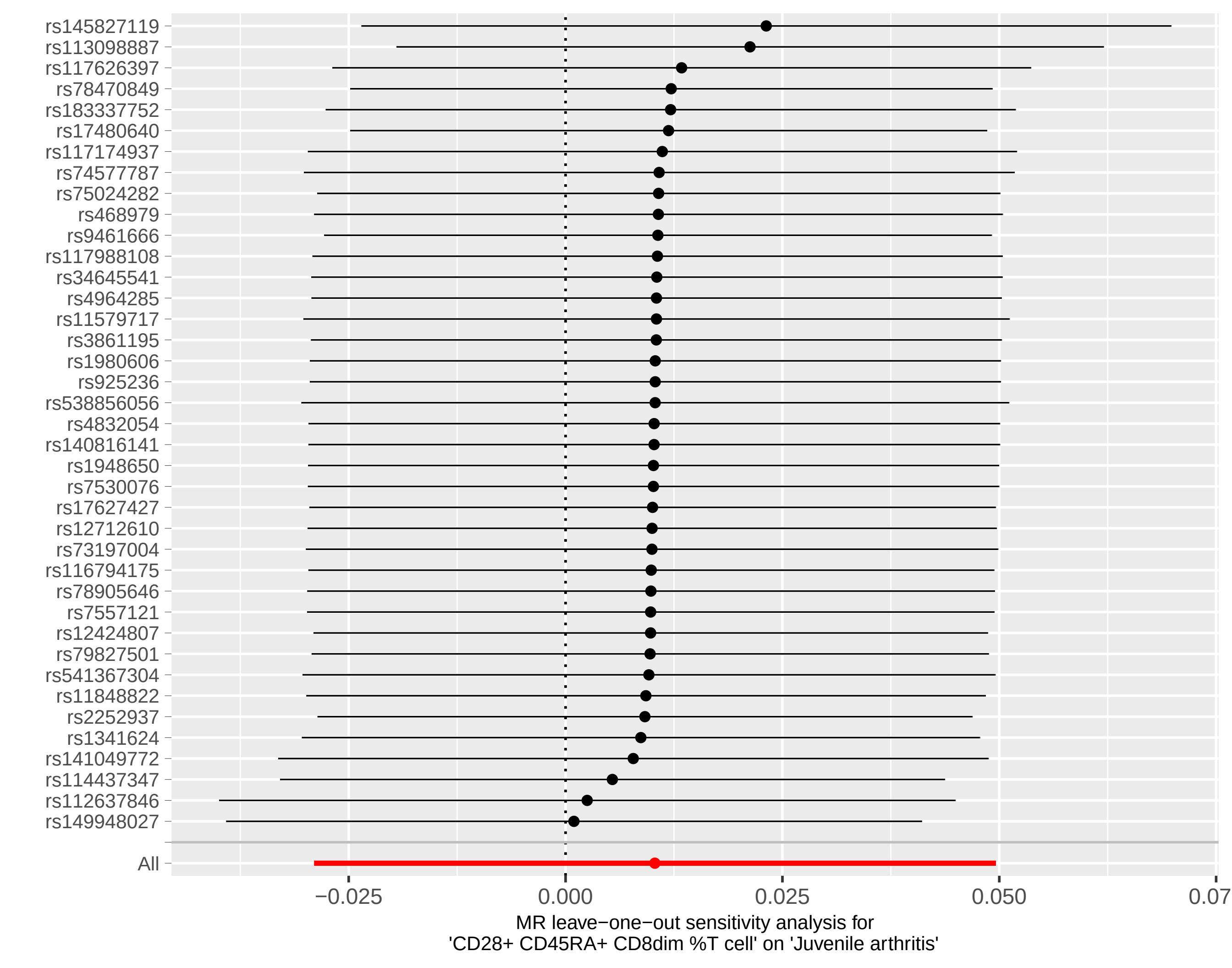
rs145827119
rs113098887
rs117626397
rs78470849
rs183337752
rs17480640
rs117174937
rs74577787
rs75024282
rs468979
rs9461666
rs117988108
rs34645541
rs4964285
rs11579717
rs3861195
rs1980606
rs925236
rs538856056
rs4832054
rs140816141
rs1948650
rs7530076
rs17627427
rs12712610
rs73197004
rs116794175
rs78905646
rs7557121
rs12424807
rs79827501
rs541367304
rs11848822
rs2252937
rs1341624
rs141049772
rs114437347
rs112637846
rs149948027
All
−0.025
0.000
0.025
0.050
0.07
MR leave−one−out sensitivity analysis for
'CD28+ CD45RA+ CD8dim %T cell' on 'Juvenile arthritis'

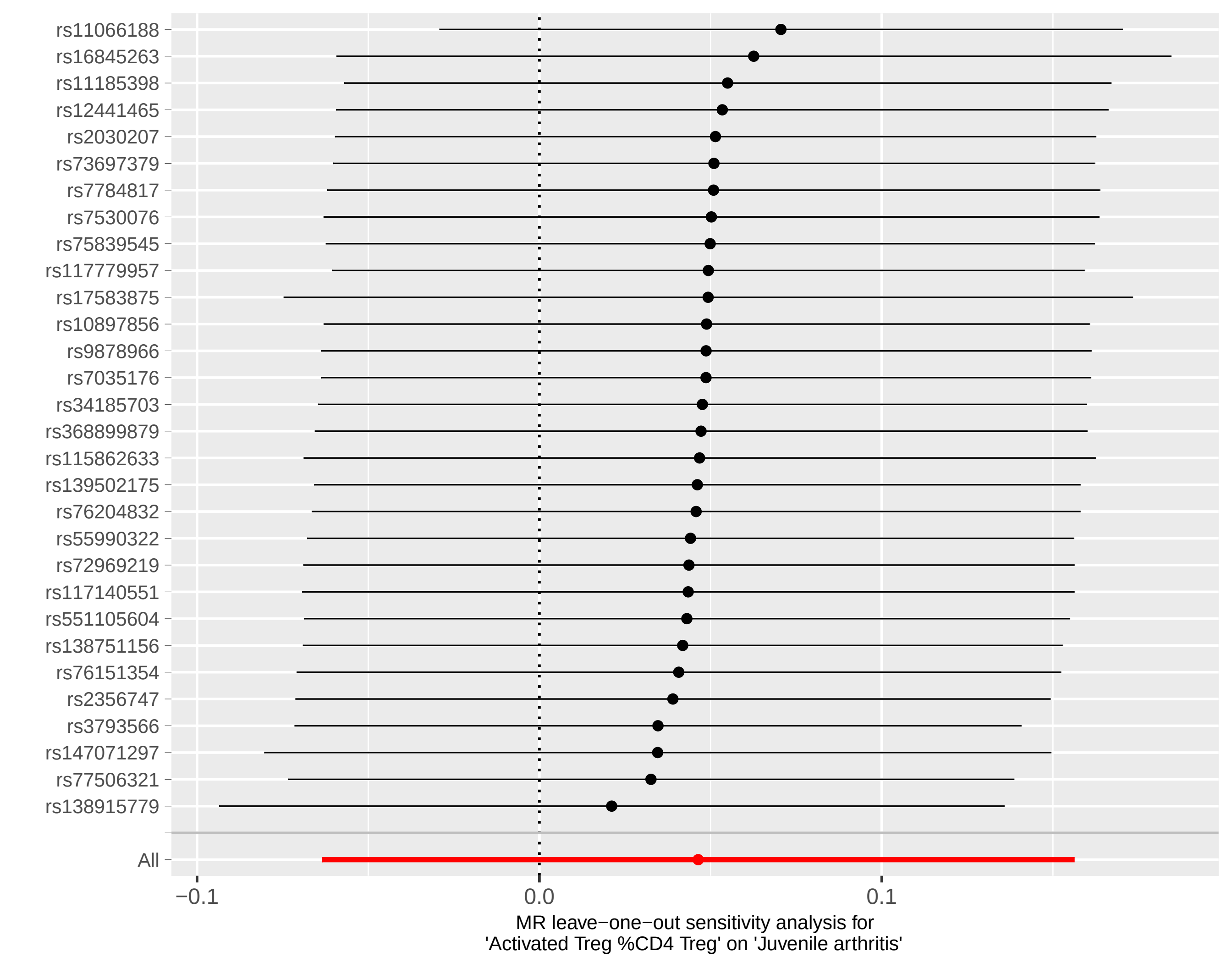
rs11066188
rs16845263
rs11185398
rs12441465
rs2030207
rs73697379
rs7784817
rs7530076
rs75839545
rs117779957
rs17583875
rs10897856
rs9878966
rs7035176
rs34185703
rs368899879
rs115862633
rs139502175
rs76204832
rs55990322
rs72969219
rs117140551
rs551105604
rs138751156
rs76151354
rs2356747
rs3793566
rs147071297
rs77506321
rs138915779
All
−0.1
0.0
0.1
MR leave−one−out sensitivity analysis for
'Activated Treg %CD4 Treg' on 'Juvenile arthritis'

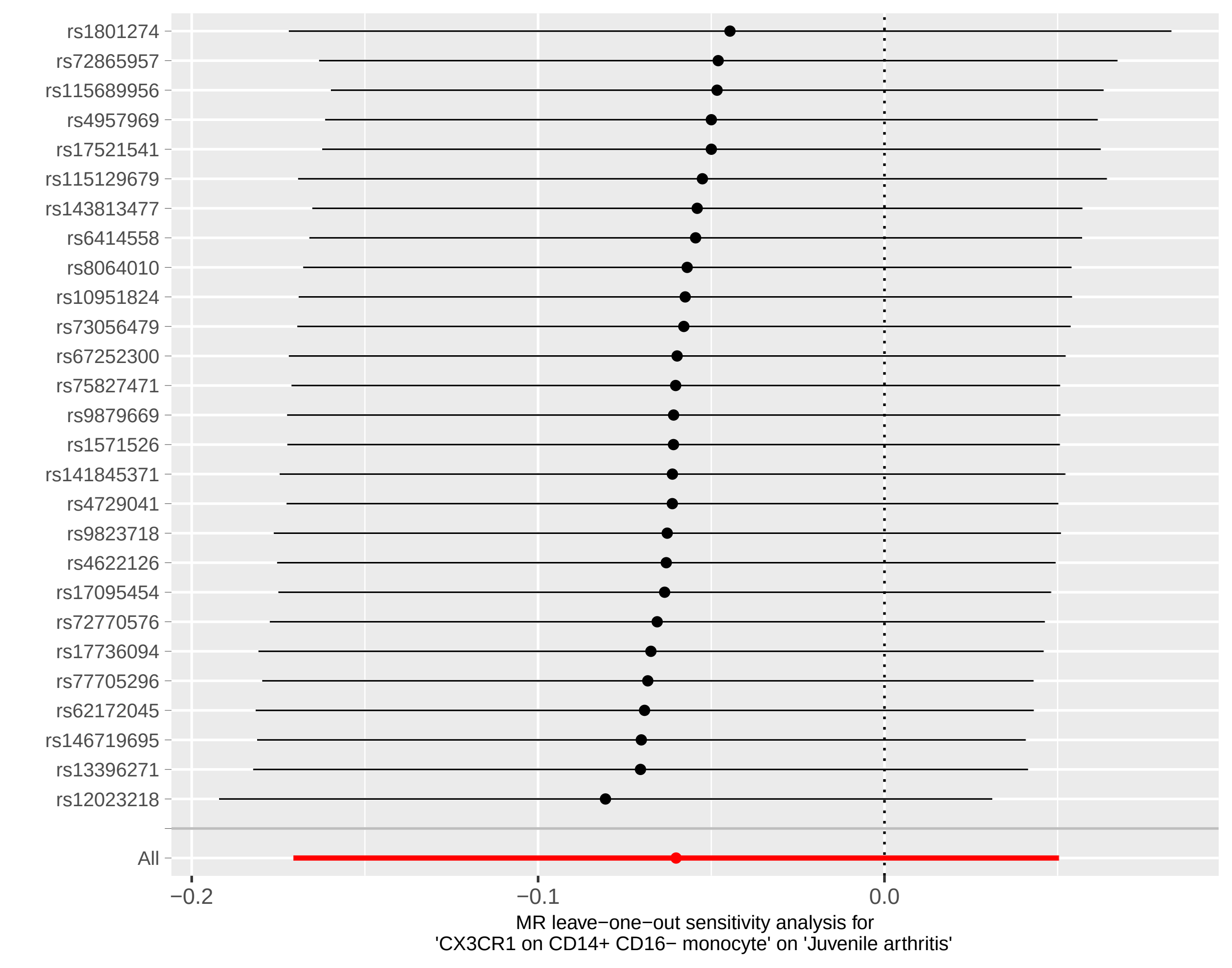

rs1801274
rs72865957
rs115689956
rs4957969
rs17521541
rs115129679
rs143813477
rs6414558
rs8064010
rs10951824
rs73056479
rs67252300
rs75827471
rs9879669
rs1571526
rs141845371
rs4729041
rs9823718
rs4622126
rs17095454
rs72770576
rs17736094
rs77705296
rs62172045
rs146719695
rs13396271
rs12023218
All
−0.2
−0.1
0.0
MR leave−one−out sensitivity analysis for
'CX3CR1 on CD14+ CD16− monocyte' on 'Juvenile arthritis'

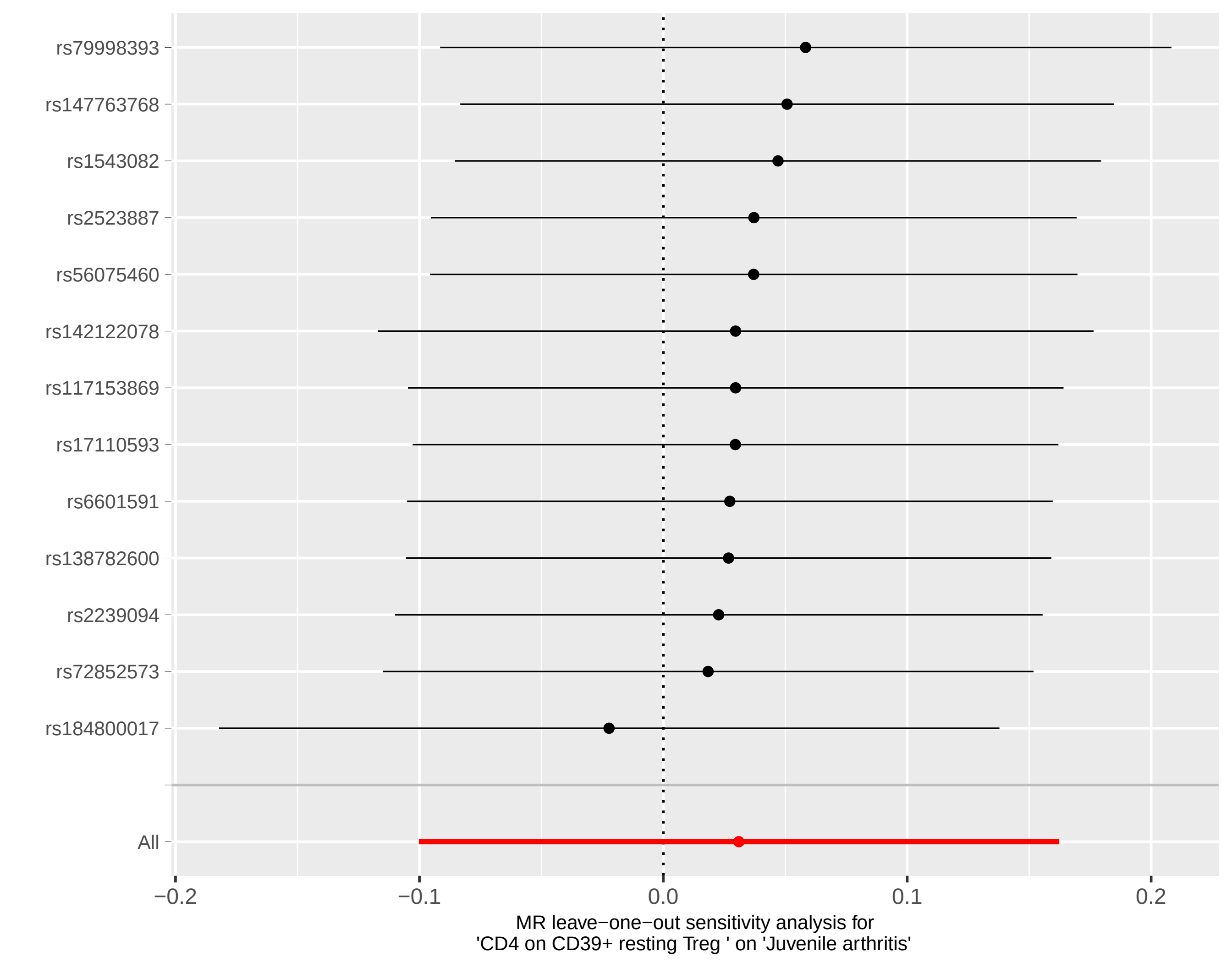
rs79998393
rs147763768
rs1543082
rs2523887
rs56075460
rs142122078
rs117153869
rs17110593
rs6601591
rs138782600
rs2239094
rs72852573
rs184800017
All
−0.2
−0.1
0.0
0.1
0.2
MR leave−one−out sensitivity analysis for
'CD4 on CD39+ resting Treg ' on 'Juvenile arthritis'

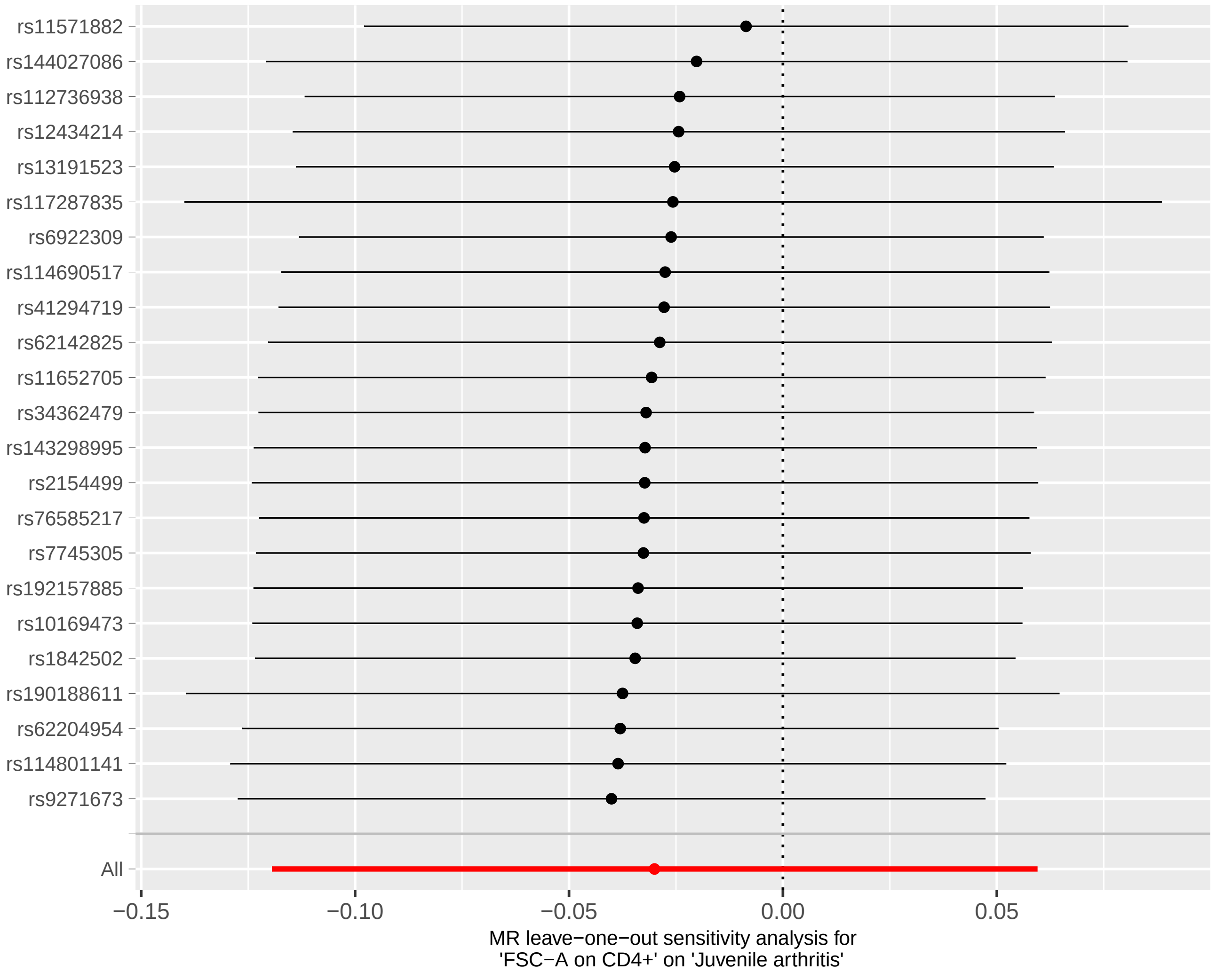
rs11571882
rs144027086
rs112736938
rs12434214
rs13191523
rs117287835
rs6922309
rs114690517
rs41294719
rs62142825
rs11652705
rs34362479
rs143298995
rs2154499
rs76585217
rs7745305
rs192157885
rs10169473
rs1842502
rs190188611
rs62204954
rs114801141
rs9271673
All
−0.15
−0.10
−0.05
0.00
0.05
MR leave−one−out sensitivity analysis for
'FSC−A on CD4+' on 'Juvenile arthritis'

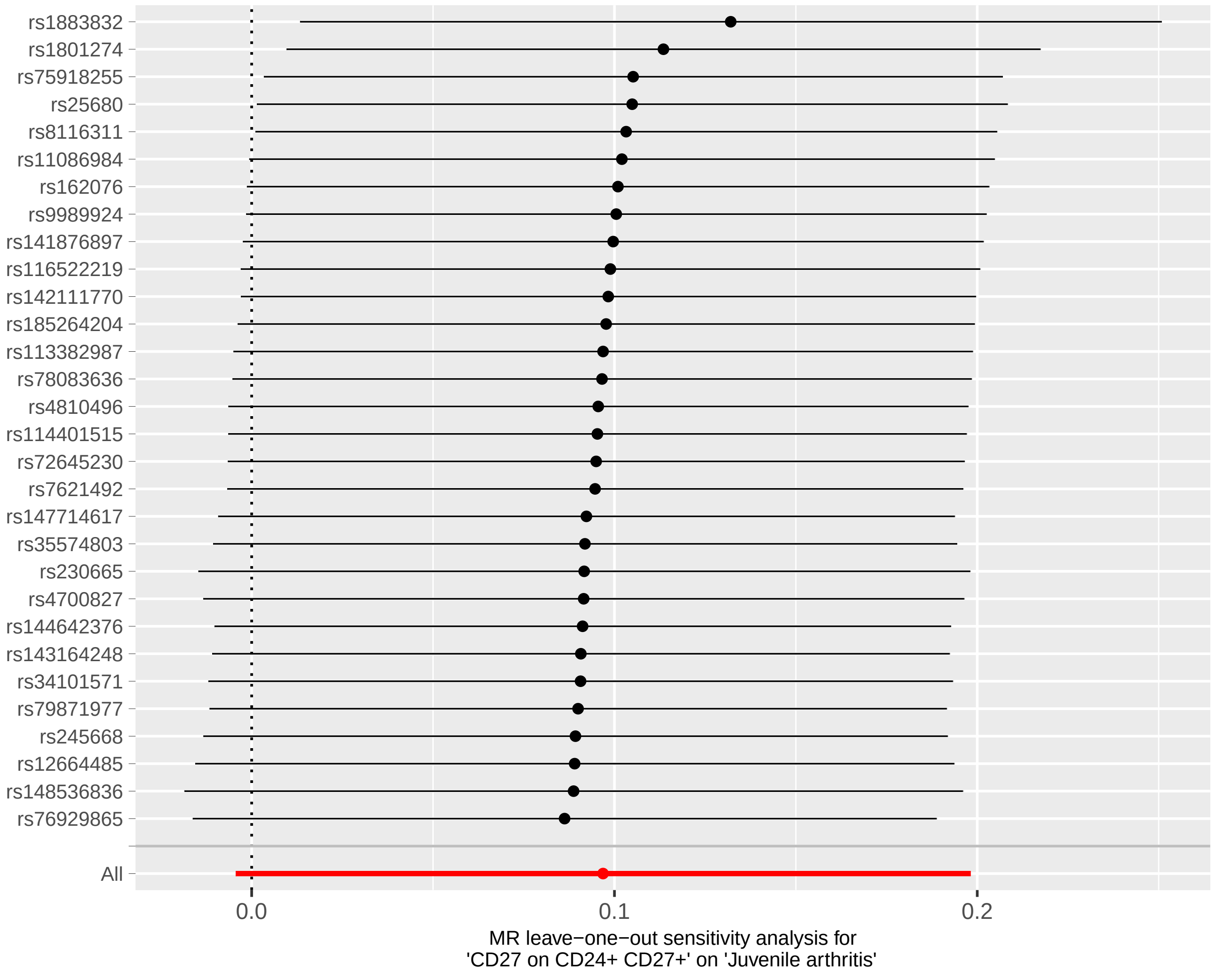
rs1883832
rs1801274
rs75918255
rs25680
rs8116311
rs11086984
rs162076
rs9989924
rs141876897
rs116522219
rs142111770
rs185264204
rs113382987
rs78083636
rs4810496
rs114401515
rs72645230
rs7621492
rs147714617
rs35574803
rs230665
rs4700827
rs144642376
rs143164248
rs34101571
rs79871977
rs245668
rs12664485
rs148536836
rs76929865
All
0.0
0.1
0.2
MR leave−one−out sensitivity analysis for
'CD27 on CD24+ CD27+' on 'Juvenile arthritis'

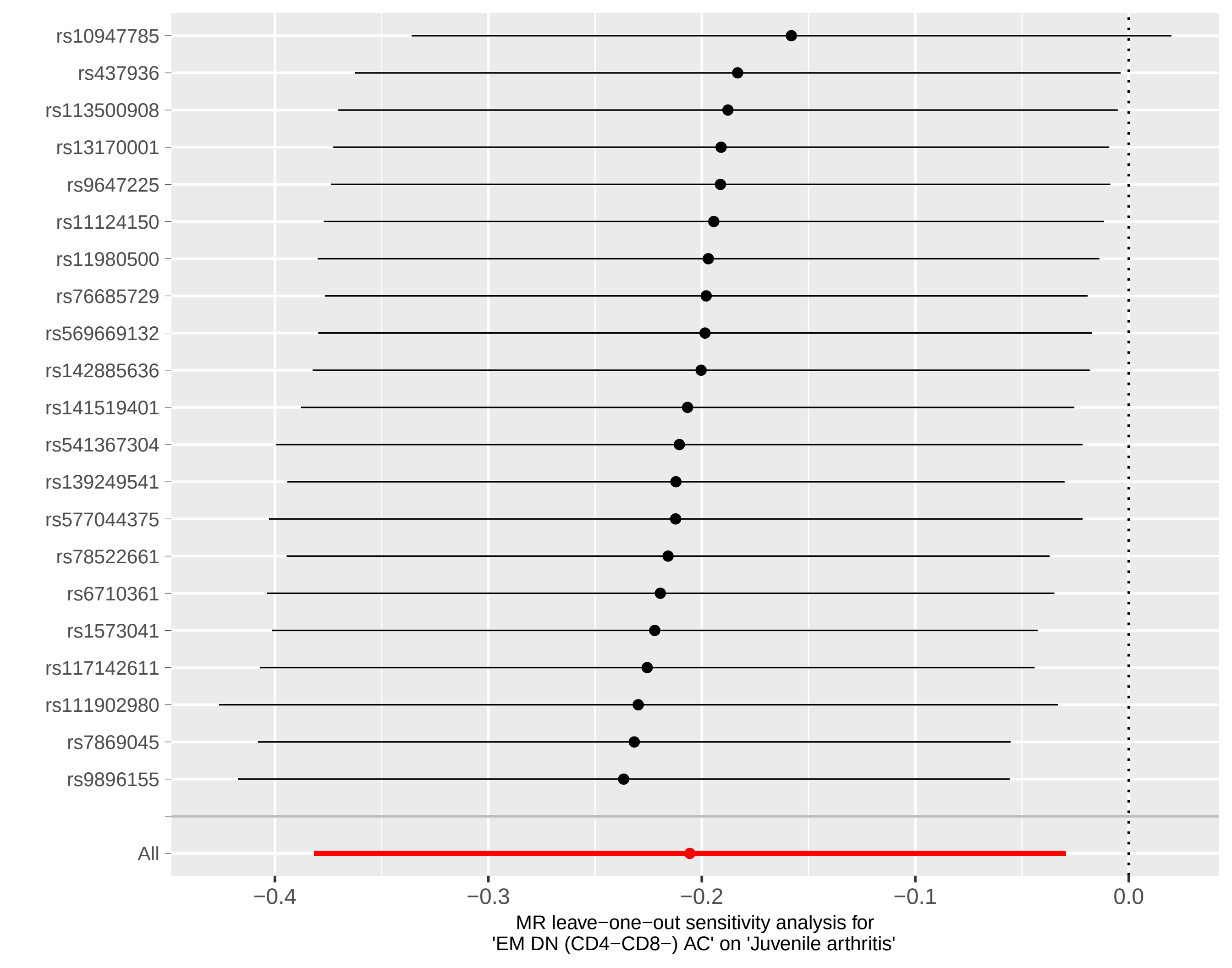

rs10947785
rs437936
rs113500908
rs13170001
rs9647225
rs11124150
rs11980500
rs76685729
rs569669132
rs142885636
rs141519401
rs541367304
rs139249541
rs577044375
rs78522661
rs6710361
rs1573041
rs117142611
rs111902980
rs7869045
rs9896155
All
−0.4
−0.3
−0.2
−0.1
0.0
MR leave−one−out sensitivity analysis for
'EM DN (CD4−CD8−) AC' on 'Juvenile arthritis'

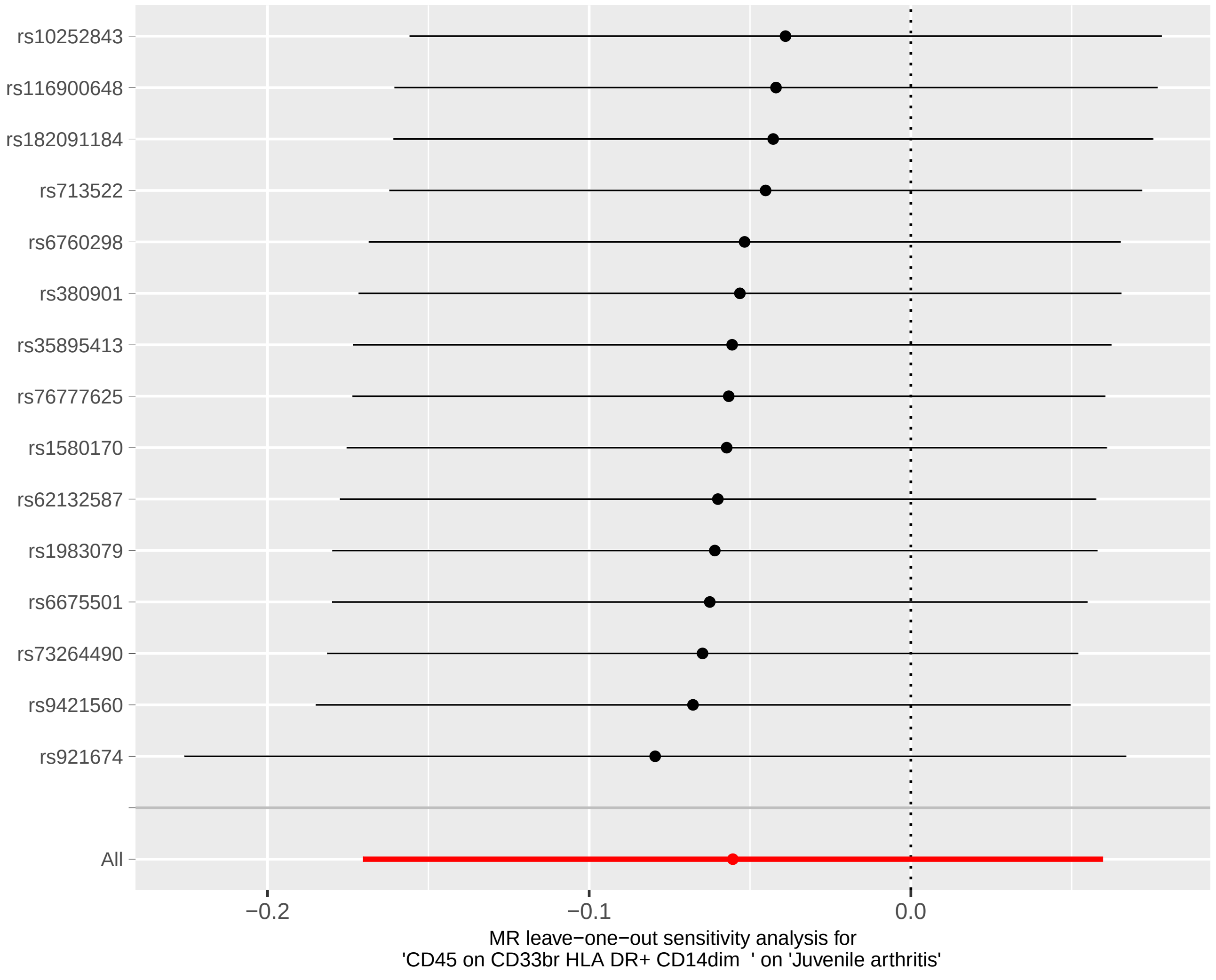
rs10252843
rs116900648
rs182091184
rs713522
rs6760298
rs380901
rs35895413
rs76777625
rs1580170
rs62132587
rs1983079
rs6675501
rs73264490
rs9421560
rs921674
All
−0.2
−0.1
0.0
MR leave−one−out sensitivity analysis for
'CD45 on CD33br HLA DR+ CD14dim ' on 'Juvenile arthritis'

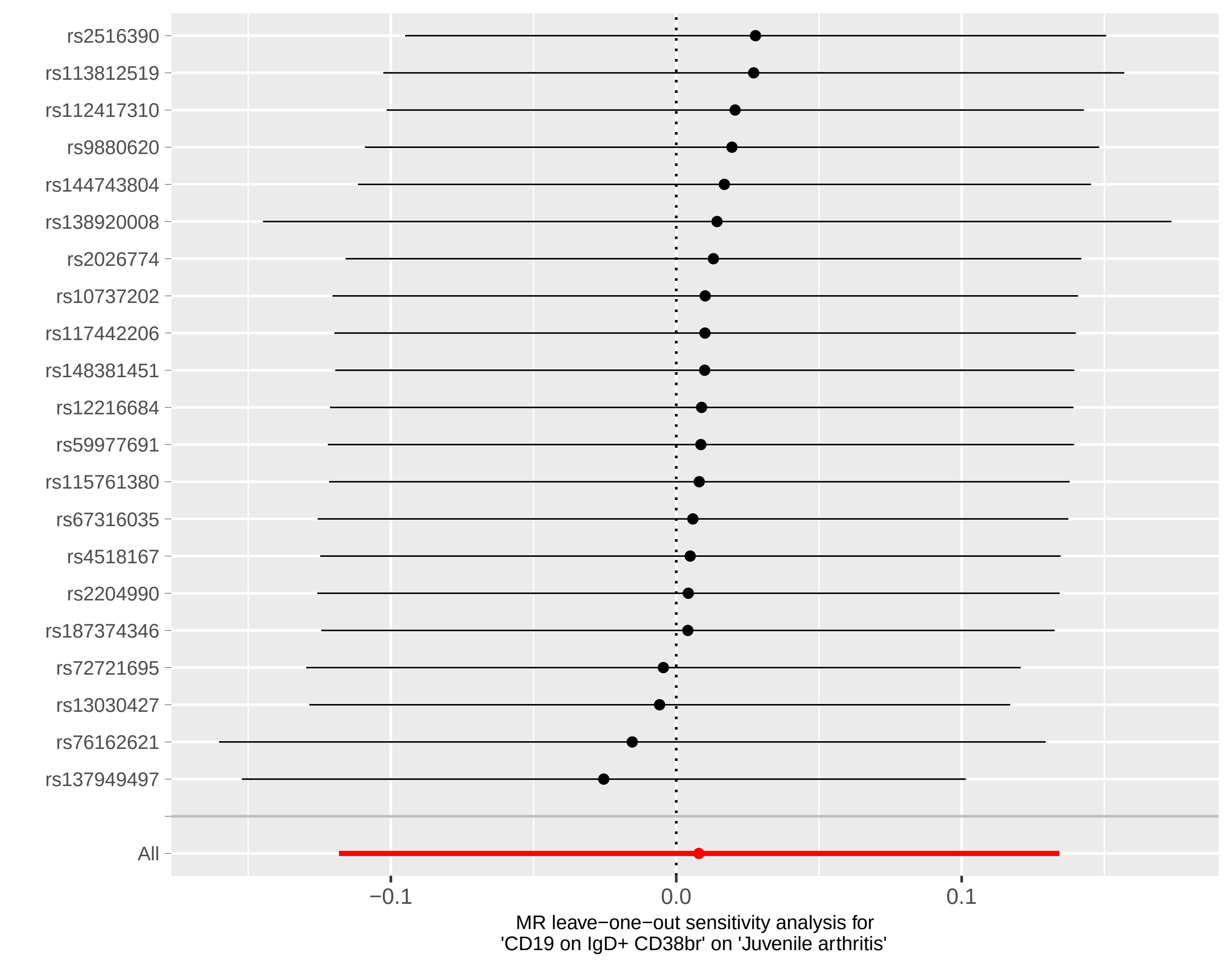

rs2516390
rs113812519
rs112417310
rs9880620
rs144743804
rs138920008
rs2026774
rs10737202
rs117442206
rs148381451
rs12216684
rs59977691
rs115761380
rs67316035
rs4518167
rs2204990
rs187374346
rs72721695
rs13030427
rs76162621
rs137949497
All
−0.1
0.0
0.1
MR leave−one−out sensitivity analysis for
'CD19 on IgD+ CD38br' on 'Juvenile arthritis'

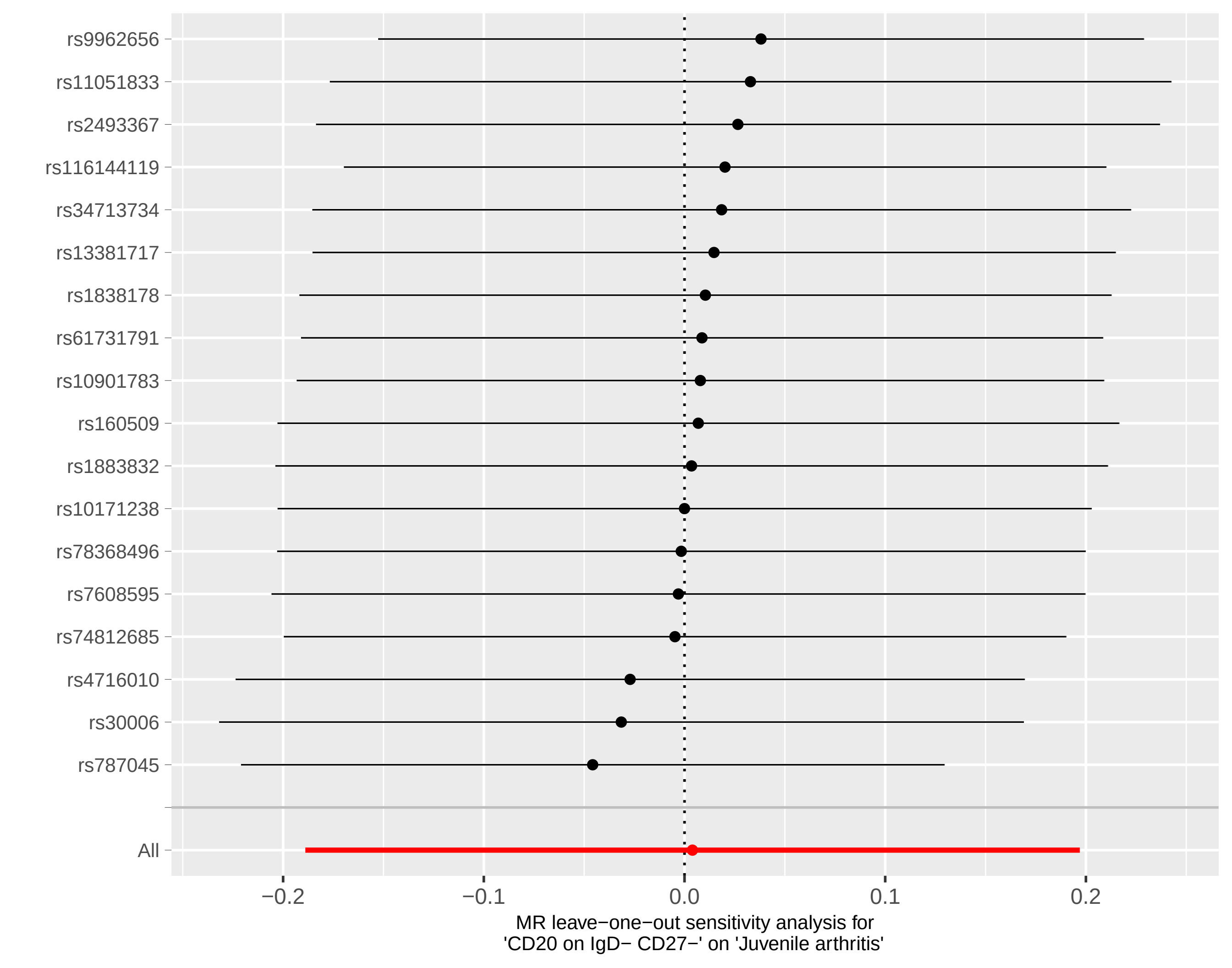
rs9962656
rs11051833
rs2493367
rs116144119
rs34713734
rs13381717
rs1838178
rs61731791
rs10901783
rs160509
rs1883832
rs10171238
rs78368496
rs7608595
rs74812685
rs4716010
rs30006
rs787045
All
−0.2
−0.1
0.0
0.1
0.2
MR leave−one−out sensitivity analysis for
'CD20 on IgD− CD27−' on 'Juvenile arthritis'

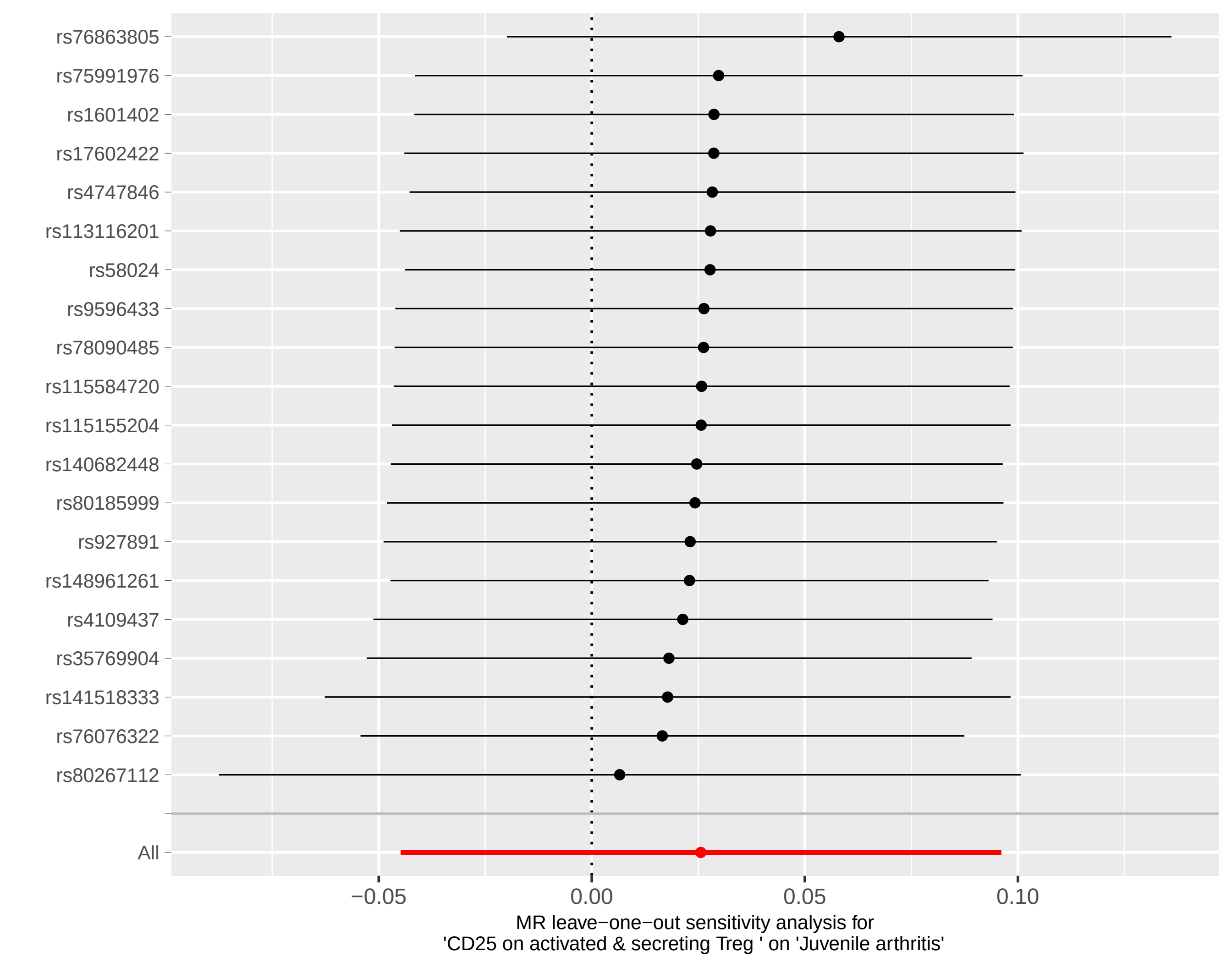

rs76863805
rs75991976
rs1601402
rs17602422
rs4747846
rs113116201
rs58024
rs9596433
rs78090485
rs115584720
rs115155204
rs140682448
rs80185999
rs927891
rs148961261
rs4109437
rs35769904
rs141518333
rs76076322
rs80267112
All
−0.05
0.00
0.05
0.10
MR leave−one−out sensitivity analysis for
'CD25 on activated & secreting Treg ' on 'Juvenile arthritis'

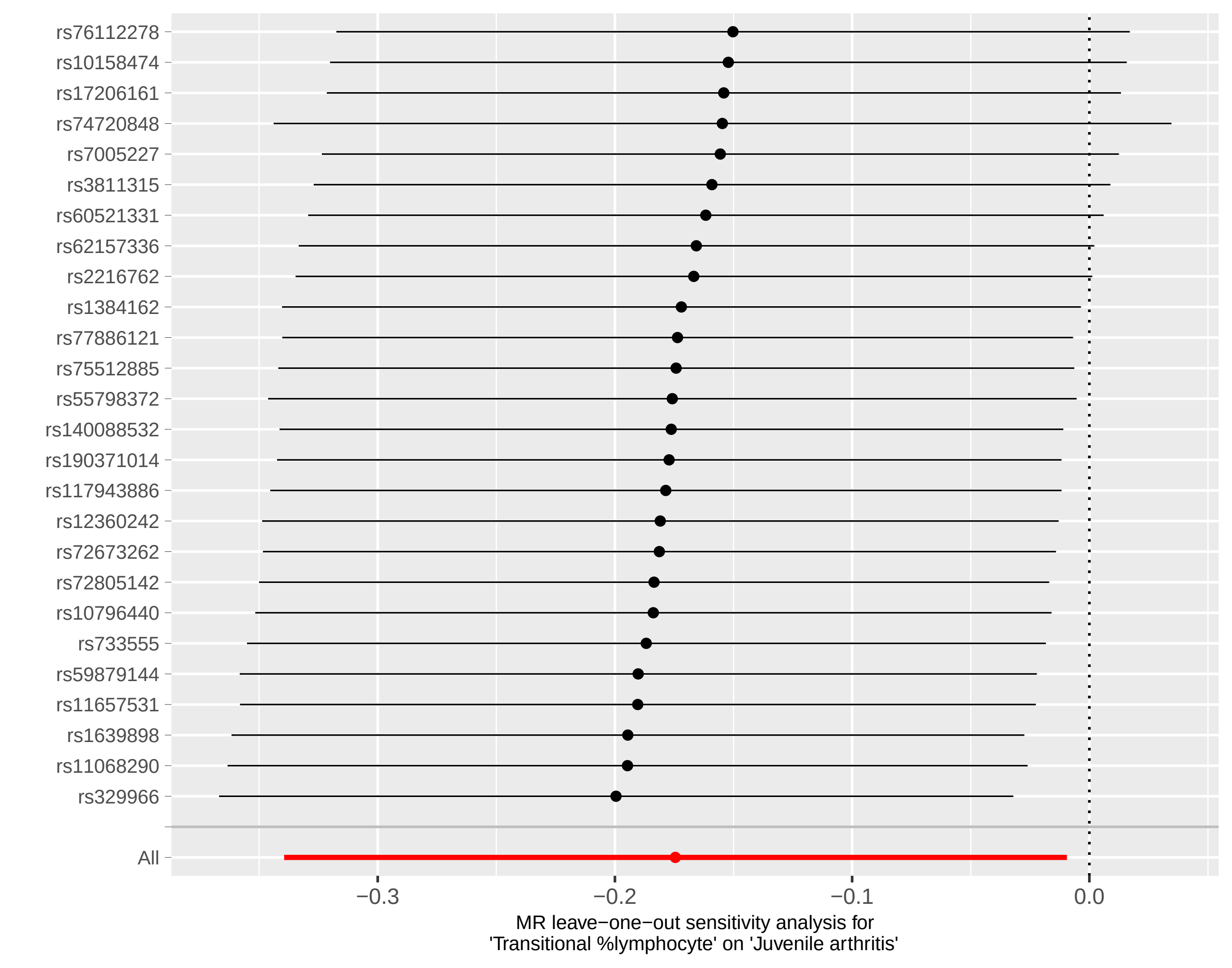

rs76112278
rs10158474
rs17206161
rs74720848
rs7005227
rs3811315
rs60521331
rs62157336
rs2216762
rs1384162
rs77886121
rs75512885
rs55798372
rs140088532
rs190371014
rs117943886
rs12360242
rs72673262
rs72805142
rs10796440
rs733555
rs59879144
rs11657531
rs1639898
rs11068290
rs329966
All
−0.3
−0.2
−0.1
0.0
MR leave−one−out sensitivity analysis for
'Transitional %lymphocyte' on 'Juvenile arthritis'

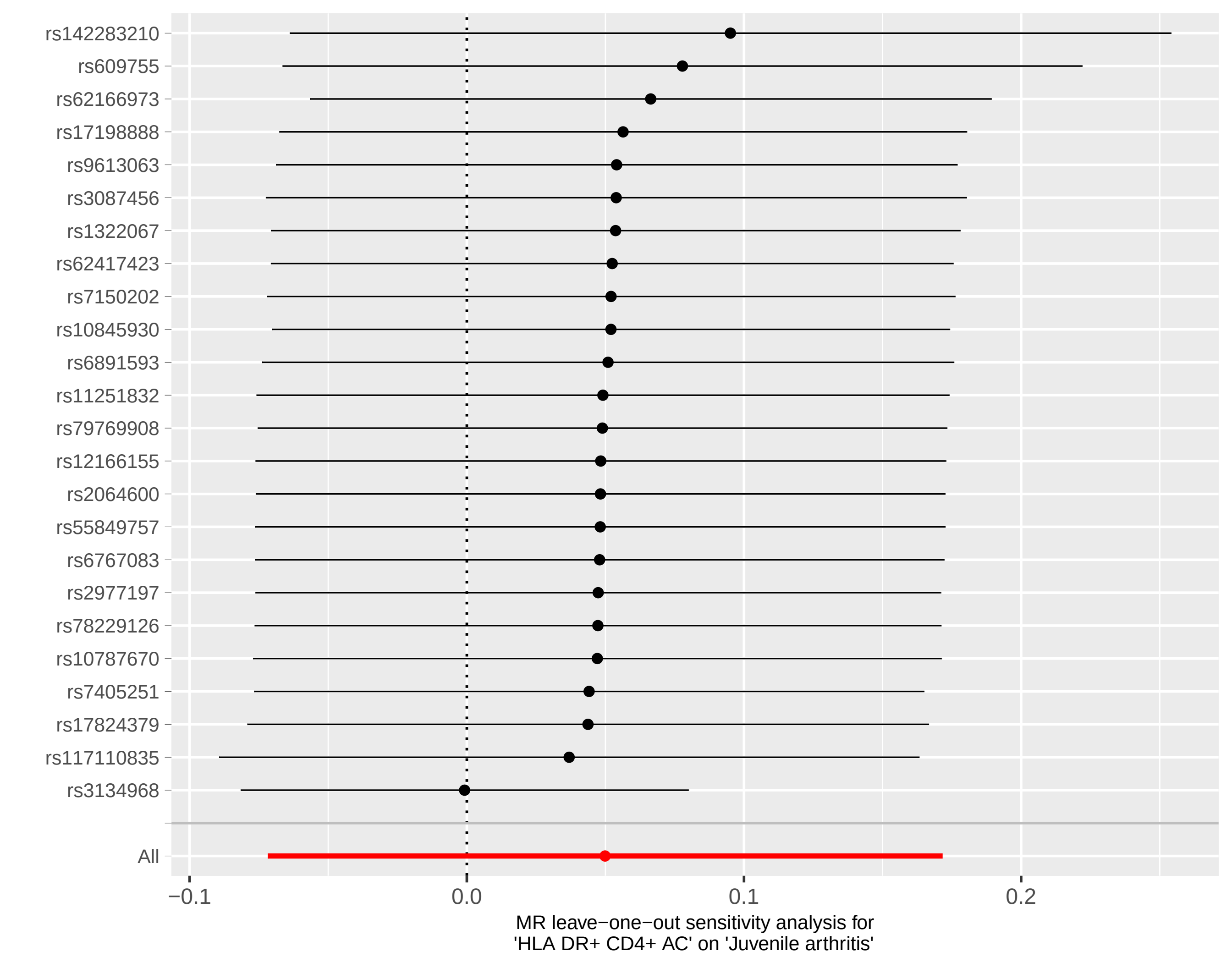
rs142283210
rs609755
rs62166973
rs17198888
rs9613063
rs3087456
rs1322067
rs62417423
rs7150202
rs10845930
rs6891593
rs11251832
rs79769908
rs12166155
rs2064600
rs55849757
rs6767083
rs2977197
rs78229126
rs10787670
rs7405251
rs17824379
rs117110835
rs3134968
All
−0.1
0.0
0.1
0.2
MR leave−one−out sensitivity analysis for
'HLA DR+ CD4+ AC' on 'Juvenile arthritis'

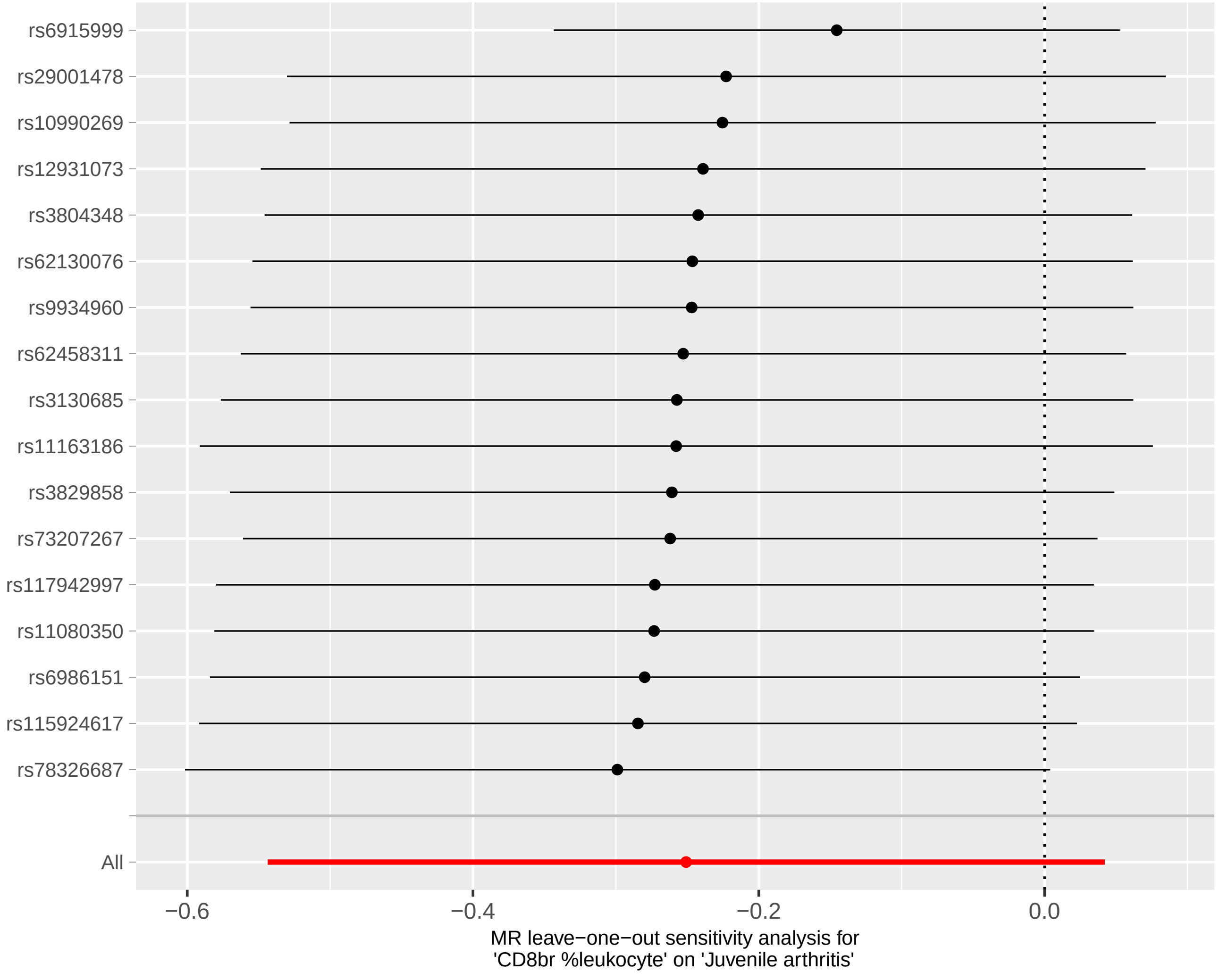

rs6915999
rs29001478
rs10990269
rs12931073
rs3804348
rs62130076
rs9934960
rs62458311
rs3130685
rs11163186
rs3829858
rs73207267
rs117942997
rs11080350
rs6986151
rs115924617
rs78326687
All
−0.6
−0.4
−0.2
0.0
MR leave−one−out sensitivity analysis for
'CD8br %leukocyte' on 'Juvenile arthritis'

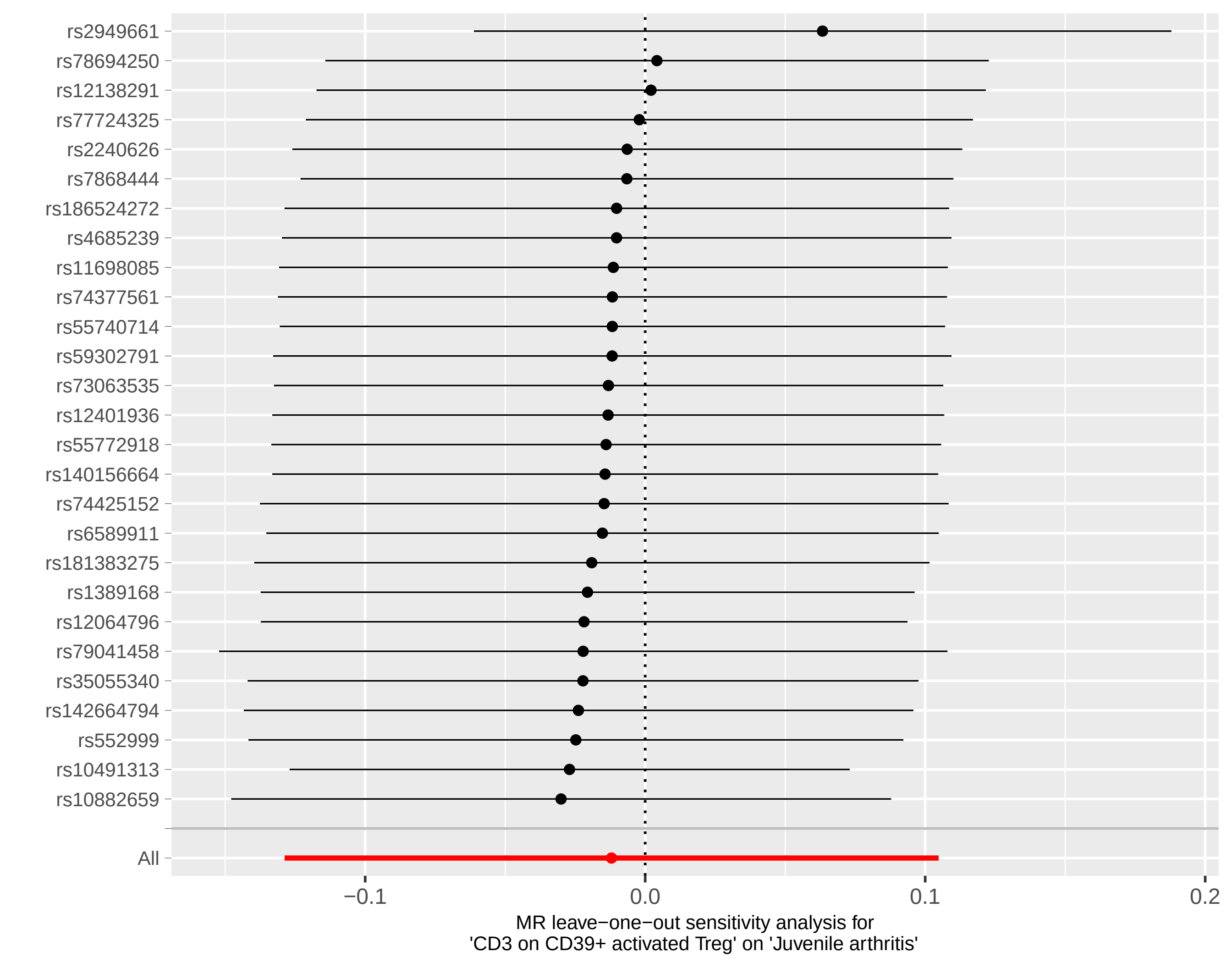
rs2949661
rs78694250
rs12138291
rs77724325
rs2240626
rs7868444
rs186524272
rs4685239
rs11698085
rs74377561
rs55740714
rs59302791
rs73063535
rs12401936
rs55772918
rs140156664
rs74425152
rs6589911
rs181383275
rs1389168
rs12064796
rs79041458
rs35055340
rs142664794
rs552999
rs10491313
rs10882659
All
−0.1
0.0
0.1
0.2
MR leave−one−out sensitivity analysis for
'CD3 on CD39+ activated Treg' on 'Juvenile arthritis'

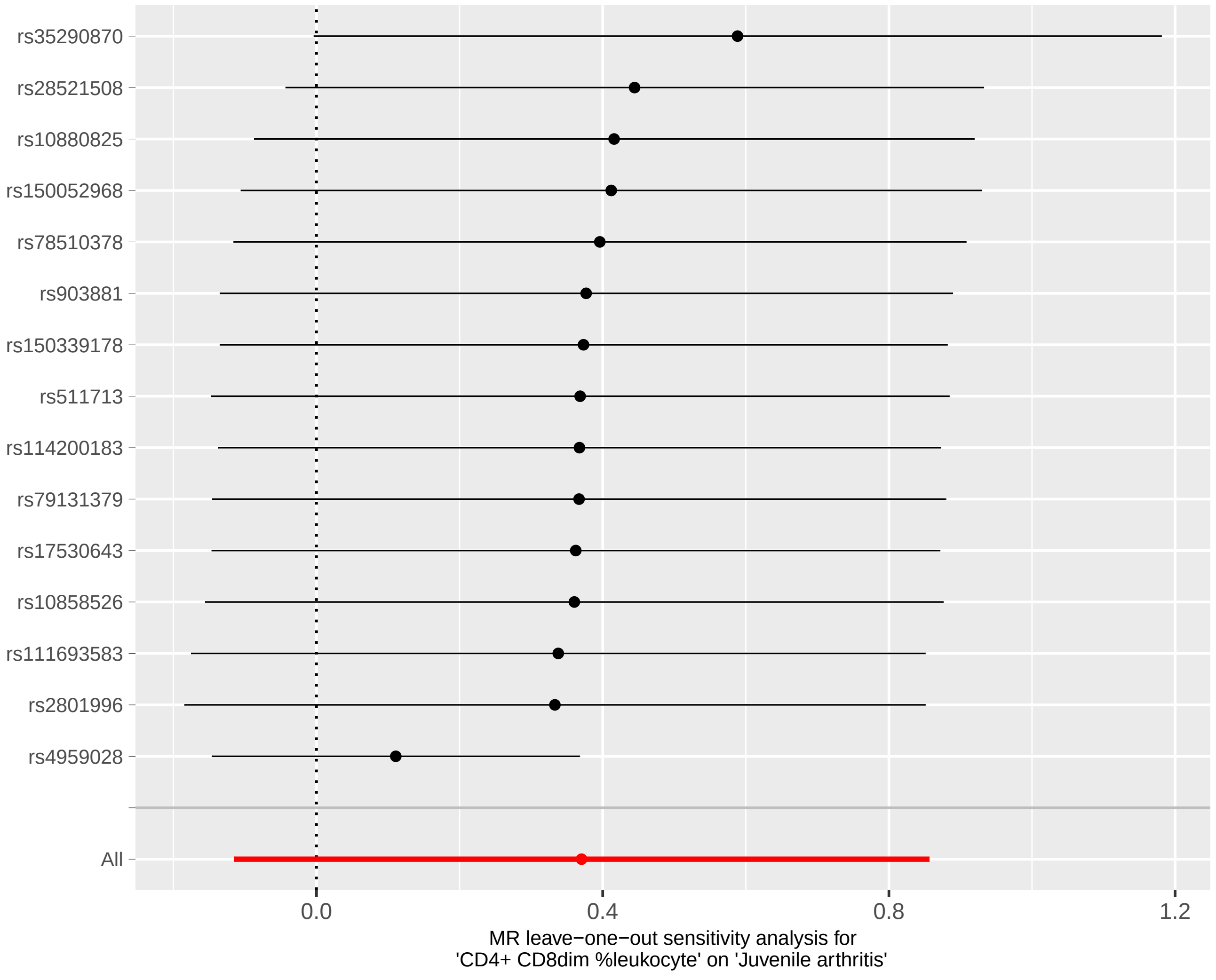
rs35290870
rs28521508
rs10880825
rs150052968
rs78510378
rs903881
rs150339178
rs511713
rs114200183
rs79131379
rs17530643
rs10858526
rs111693583
rs2801996
rs4959028
All
0.0
0.4
0.8
1.2
MR leave−one−out sensitivity analysis for
'CD4+ CD8dim %leukocyte' on 'Juvenile arthritis'

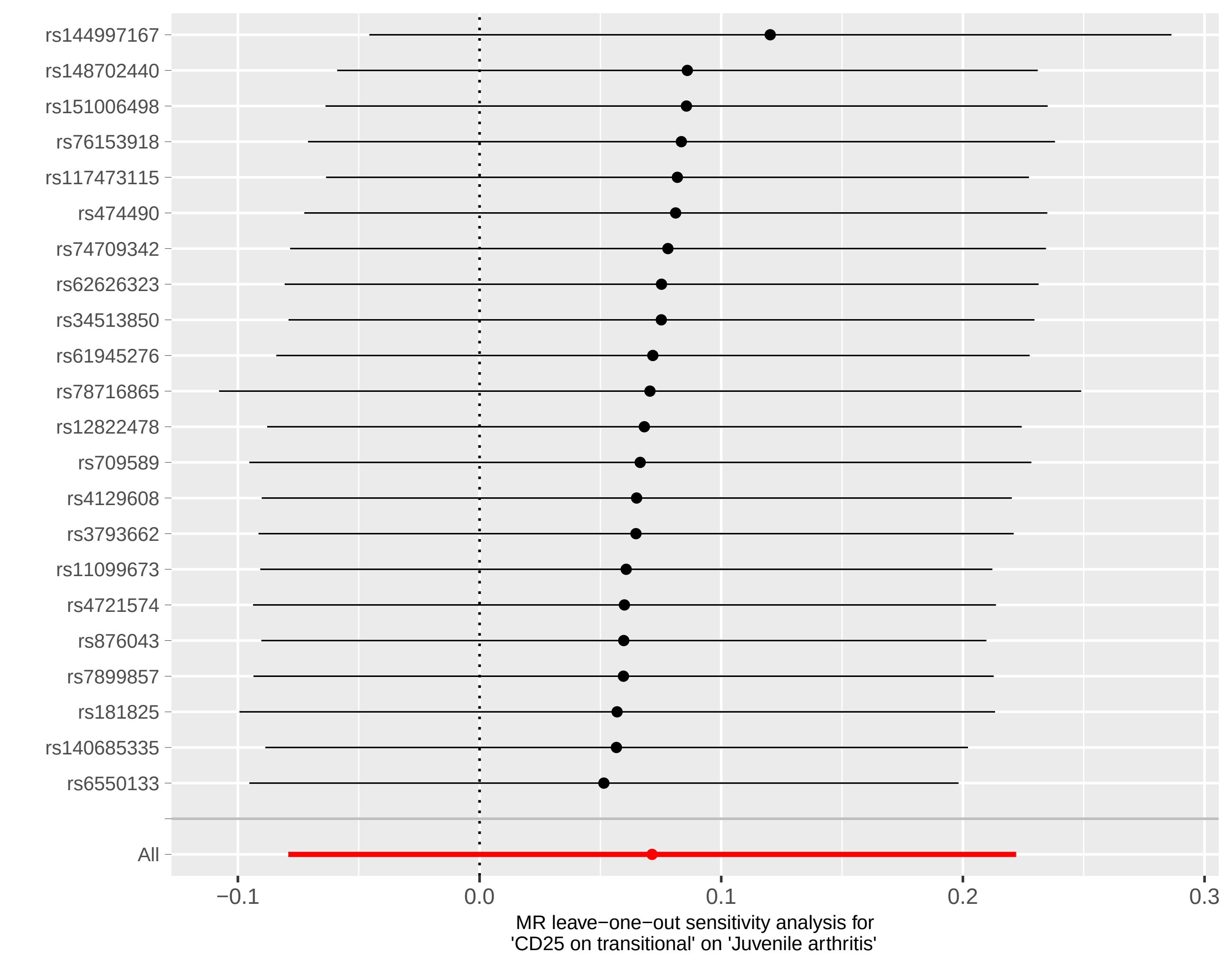
rs144997167
rs148702440
rs151006498
rs76153918
rs117473115
rs474490
rs74709342
rs62626323
rs34513850
rs61945276
rs78716865
rs12822478
rs709589
rs4129608
rs3793662
rs11099673
rs4721574
rs876043
rs7899857
rs181825
rs140685335
rs6550133
All
−0.1
0.0
0.1
0.2
0.3
MR leave−one−out sensitivity analysis for
'CD25 on transitional' on 'Juvenile arthritis'

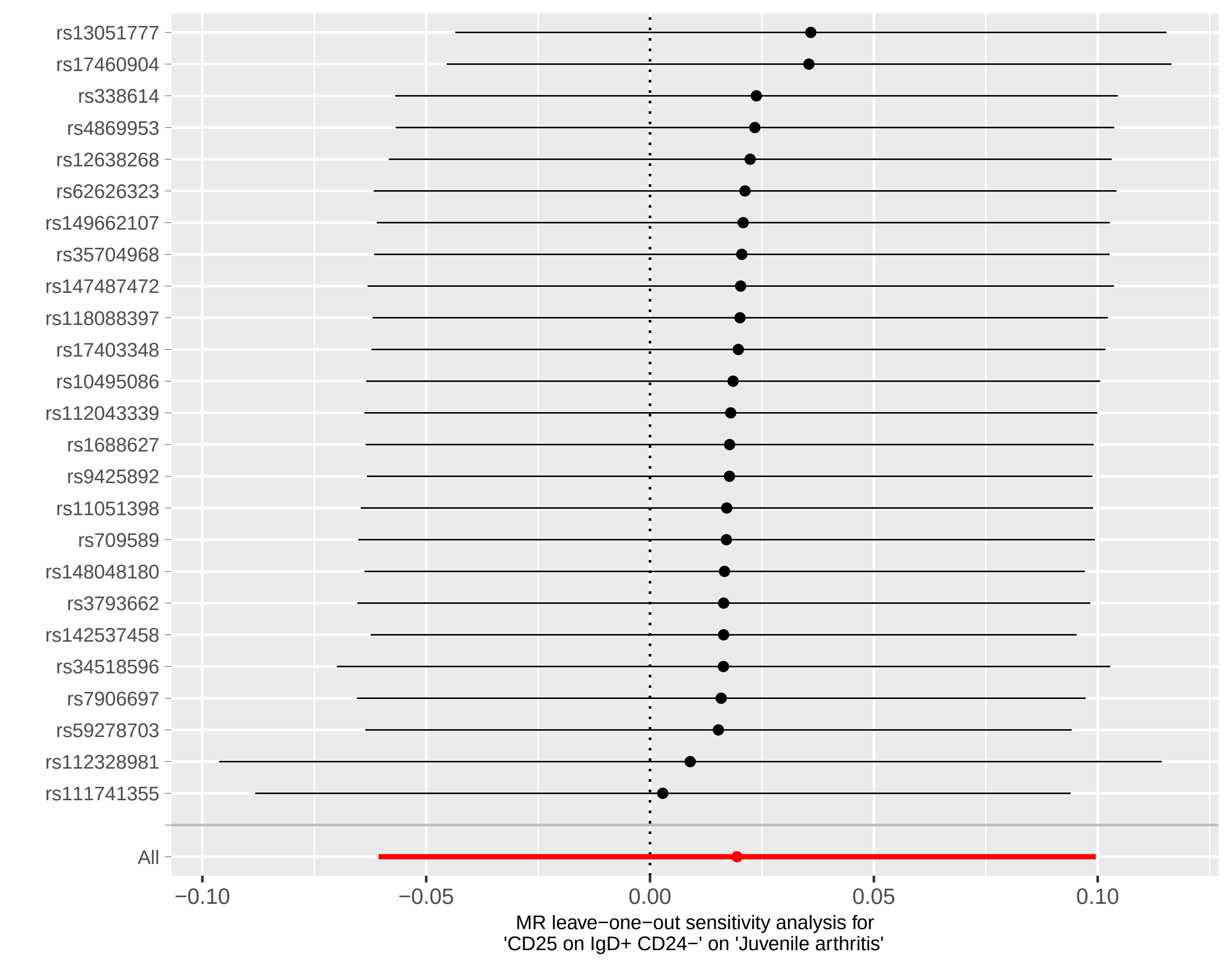

rs13051777
rs17460904
rs338614
rs4869953
rs12638268
rs62626323
rs149662107
rs35704968
rs147487472
rs118088397
rs17403348
rs10495086
rs112043339
rs1688627
rs9425892
rs11051398
rs709589
rs148048180
rs3793662
rs142537458
rs34518596
rs7906697
rs59278703
rs112328981
rs111741355
All
−0.10
−0.05
0.00
0.05
0.10
MR leave−one−out sensitivity analysis for
'CD25 on IgD+ CD24−' on 'Juvenile arthritis'

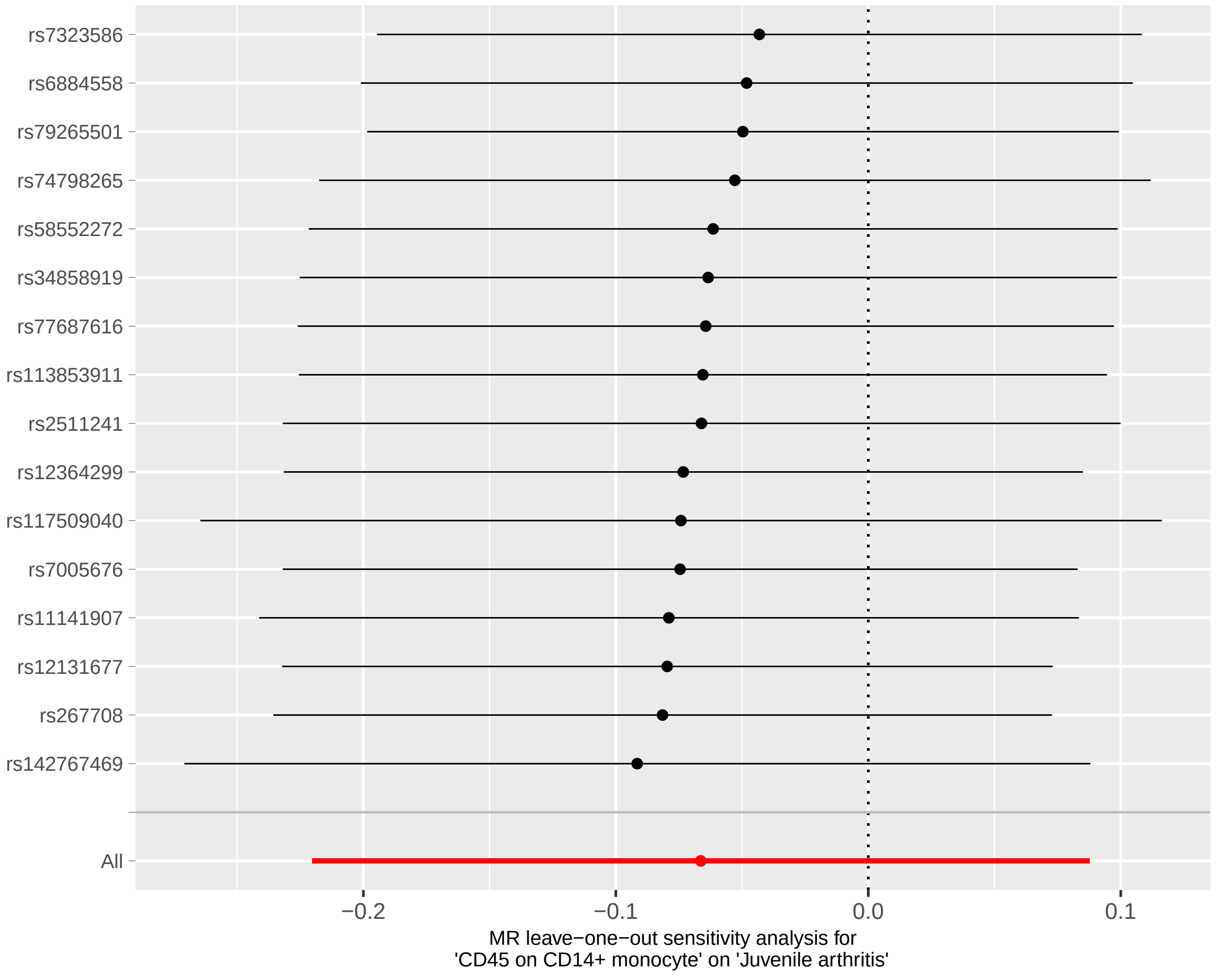

rs7323586
rs6884558
rs79265501
rs74798265
rs58552272
rs34858919
rs77687616
rs113853911
rs2511241
rs12364299
rs117509040
rs7005676
rs11141907
rs12131677
rs267708
rs142767469
All
−0.2
−0.1
0.0
0.1
MR leave−one−out sensitivity analysis for
'CD45 on CD14+ monocyte' on 'Juvenile arthritis'

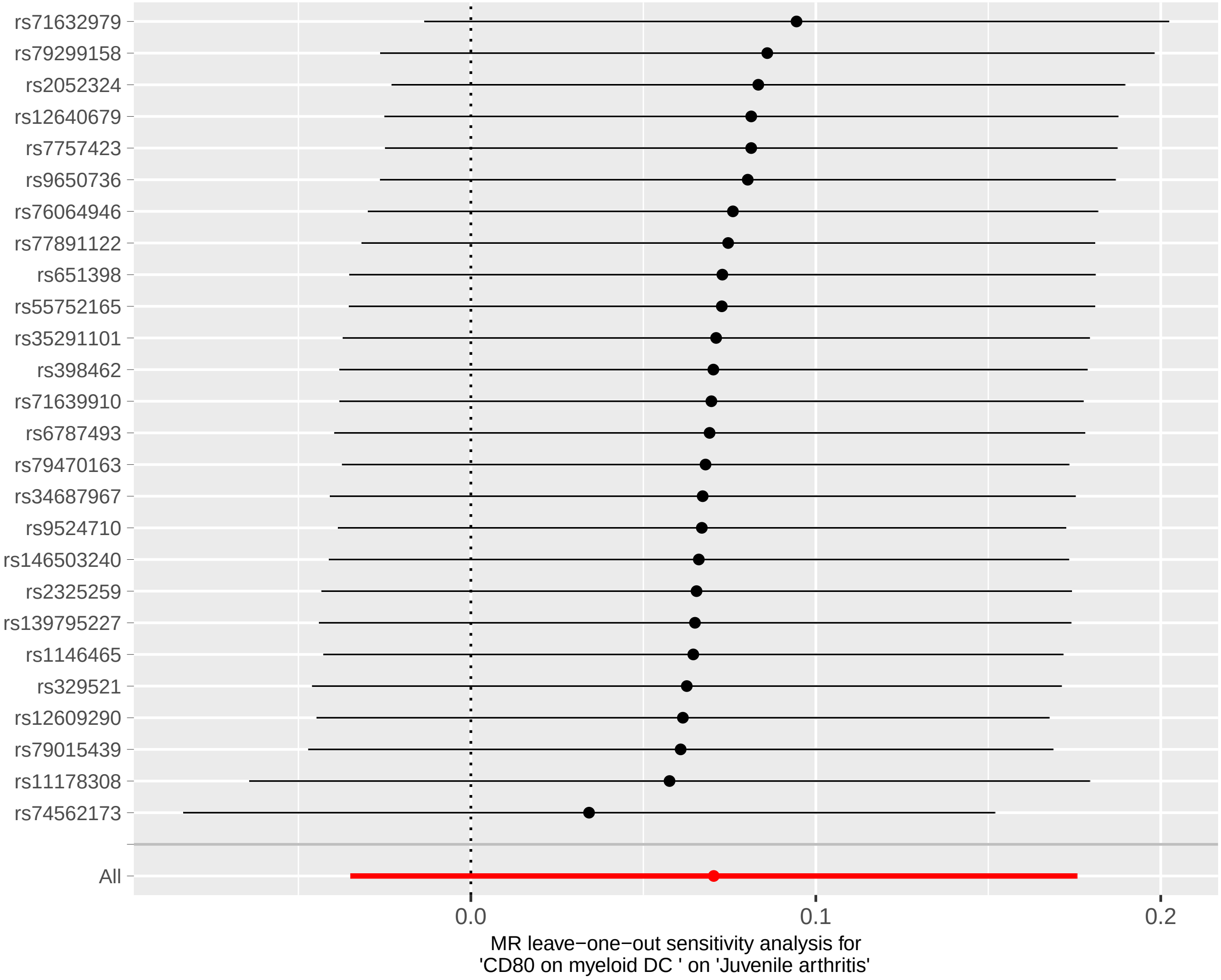
rs71632979
rs79299158
rs2052324
rs12640679
rs7757423
rs9650736
rs76064946
rs77891122
rs651398
rs55752165
rs35291101
rs398462
rs71639910
rs6787493
rs79470163
rs34687967
rs9524710
rs146503240
rs2325259
rs139795227
rs1146465
rs329521
rs12609290
rs79015439
rs11178308
rs74562173
All
0.0
0.1
0.2
MR leave−one−out sensitivity analysis for
'CD80 on myeloid DC ' on 'Juvenile arthritis'

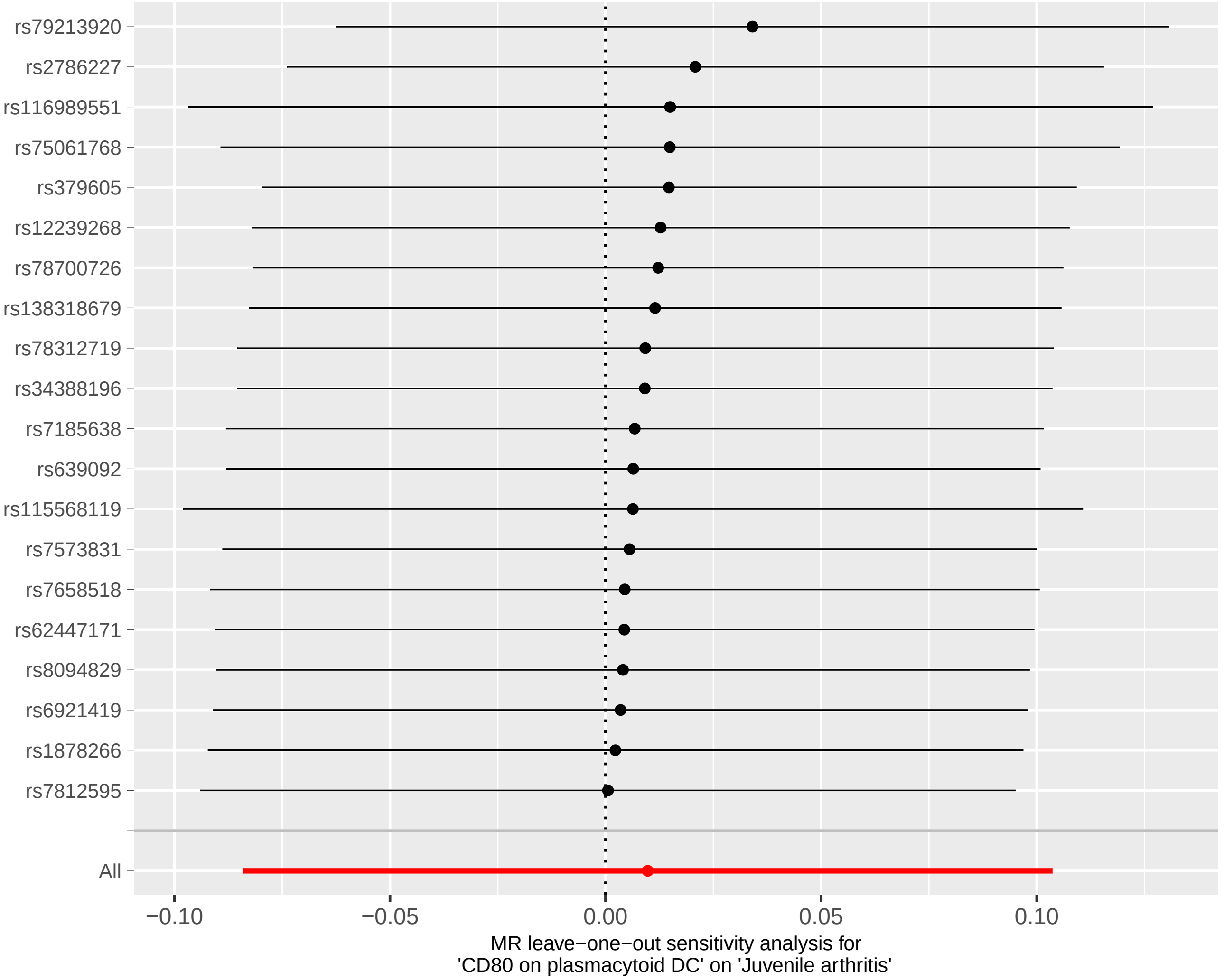

rs79213920
rs2786227
rs116989551
rs75061768
rs379605
rs12239268
rs78700726
rs138318679
rs78312719
rs34388196
rs7185638
rs639092
rs115568119
rs7573831
rs7658518
rs62447171
rs8094829
rs6921419
rs1878266
rs7812595
All
−0.10
−0.05
0.00
0.05
0.10
MR leave−one−out sensitivity analysis for
'CD80 on plasmacytoid DC' on 'Juvenile arthritis'

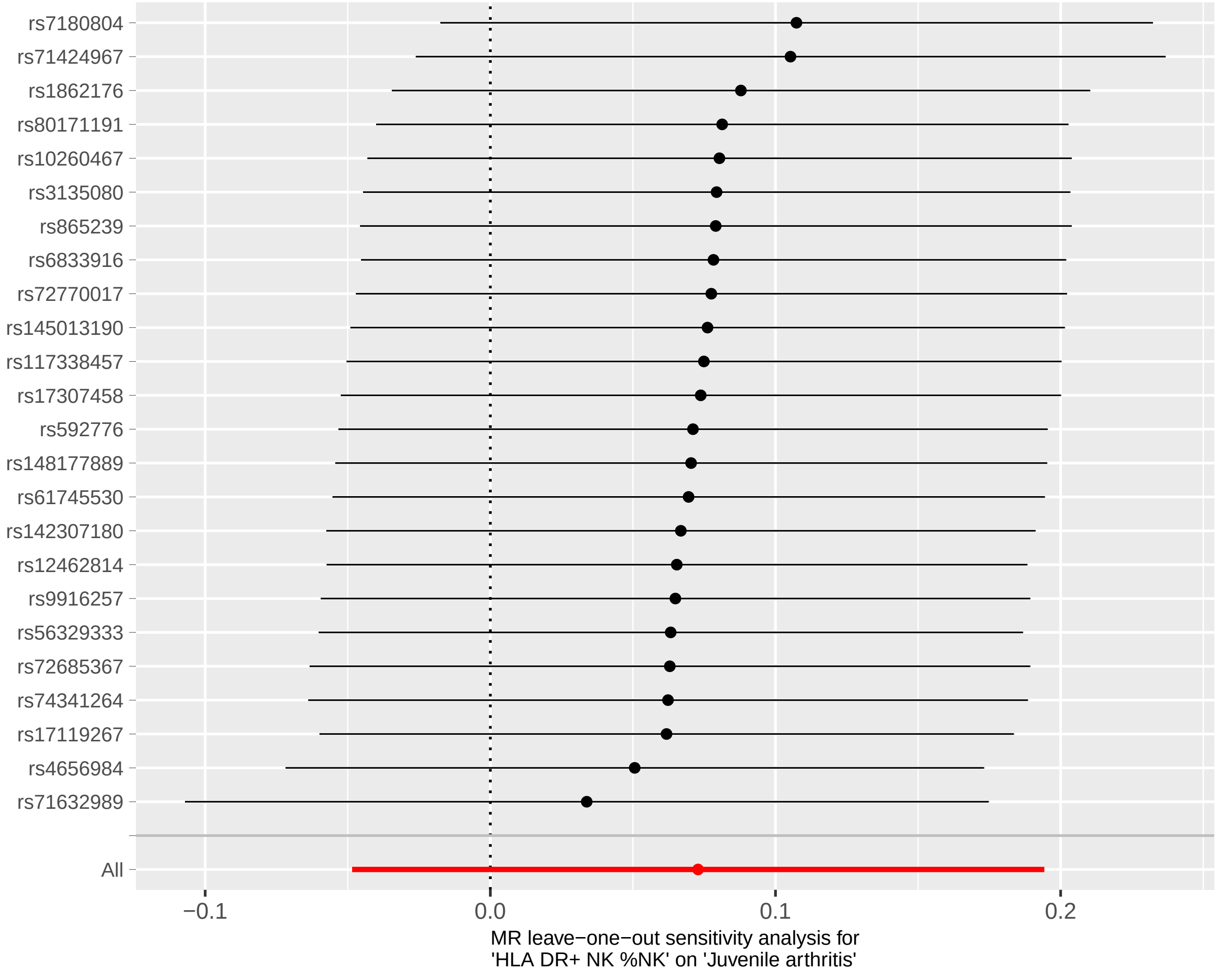
rs7180804
rs71424967
rs1862176
rs80171191
rs10260467
rs3135080
rs865239
rs6833916
rs72770017
rs145013190
rs117338457
rs17307458
rs592776
rs148177889
rs61745530
rs142307180
rs12462814
rs9916257
rs56329333
rs72685367
rs74341264
rs17119267
rs4656984
rs71632989
All
−0.1
0.0
0.1
0.2
MR leave−one−out sensitivity analysis for
'HLA DR+ NK %NK' on 'Juvenile arthritis'

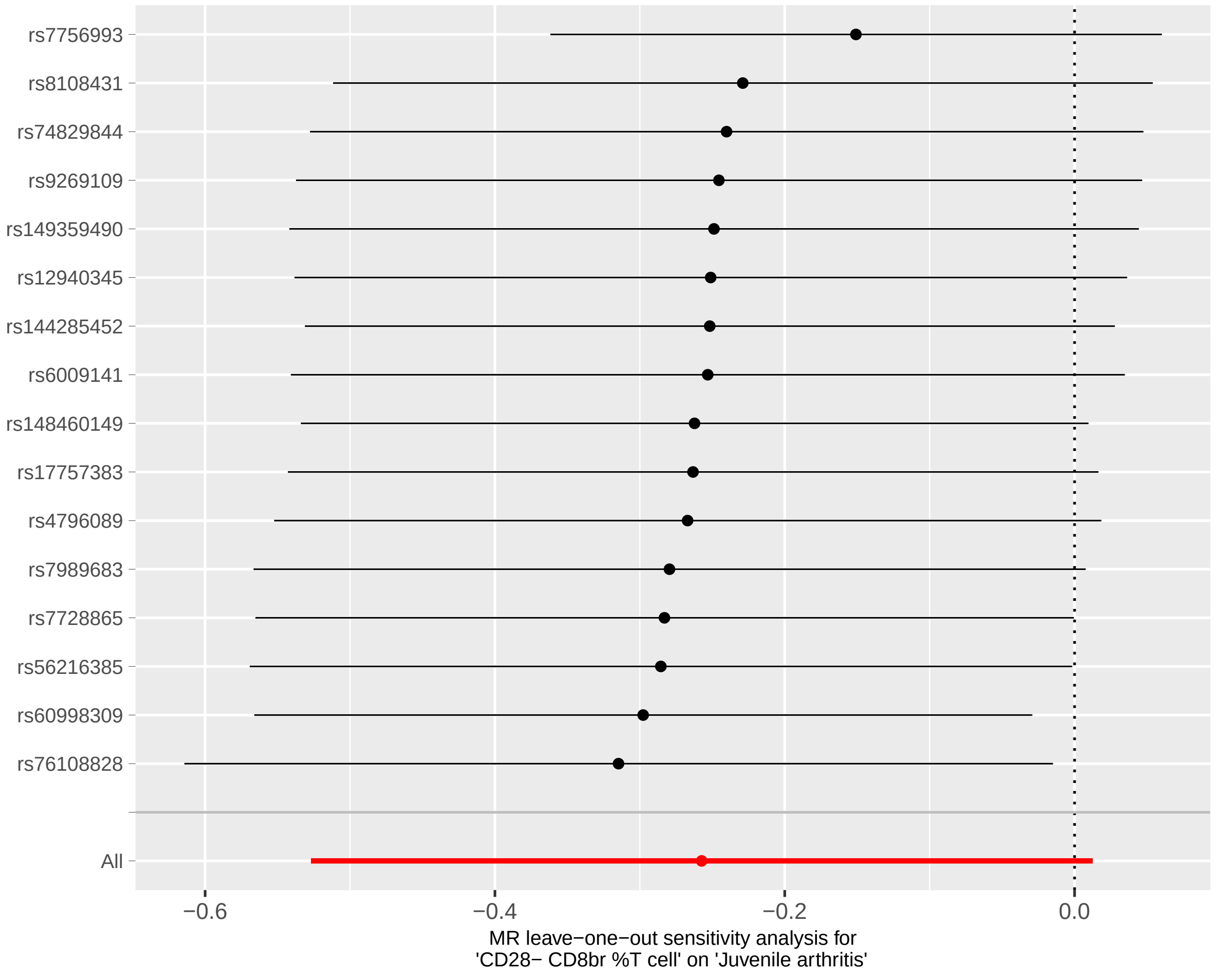
rs7756993
rs8108431
rs74829844
rs9269109
rs149359490
rs12940345
rs144285452
rs6009141
rs148460149
rs17757383
rs4796089
rs7989683
rs7728865
rs56216385
rs60998309
rs76108828
All
−0.6
−0.4
−0.2
0.0
MR leave−one−out sensitivity analysis for
'CD28− CD8br %T cell' on 'Juvenile arthritis'

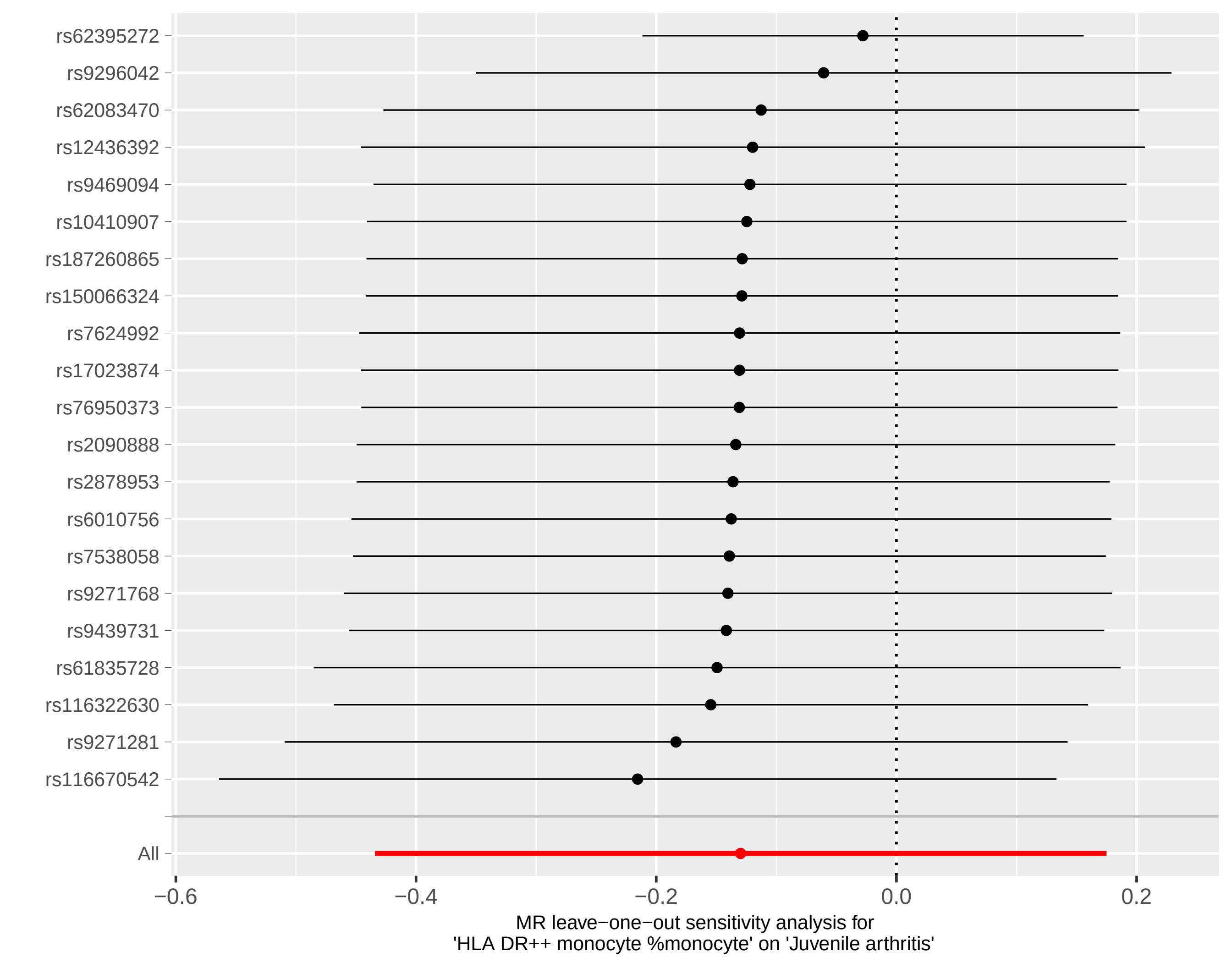

rs62395272
rs9296042
rs62083470
rs12436392
rs9469094
rs10410907
rs187260865
rs150066324
rs7624992
rs17023874
rs76950373
rs2090888
rs2878953
rs6010756
rs7538058
rs9271768
rs9439731
rs61835728
rs116322630
rs9271281
rs116670542
All
−0.6
−0.4
−0.2
0.0
0.2
MR leave−one−out sensitivity analysis for
'HLA DR++ monocyte %monocyte' on 'Juvenile arthritis'

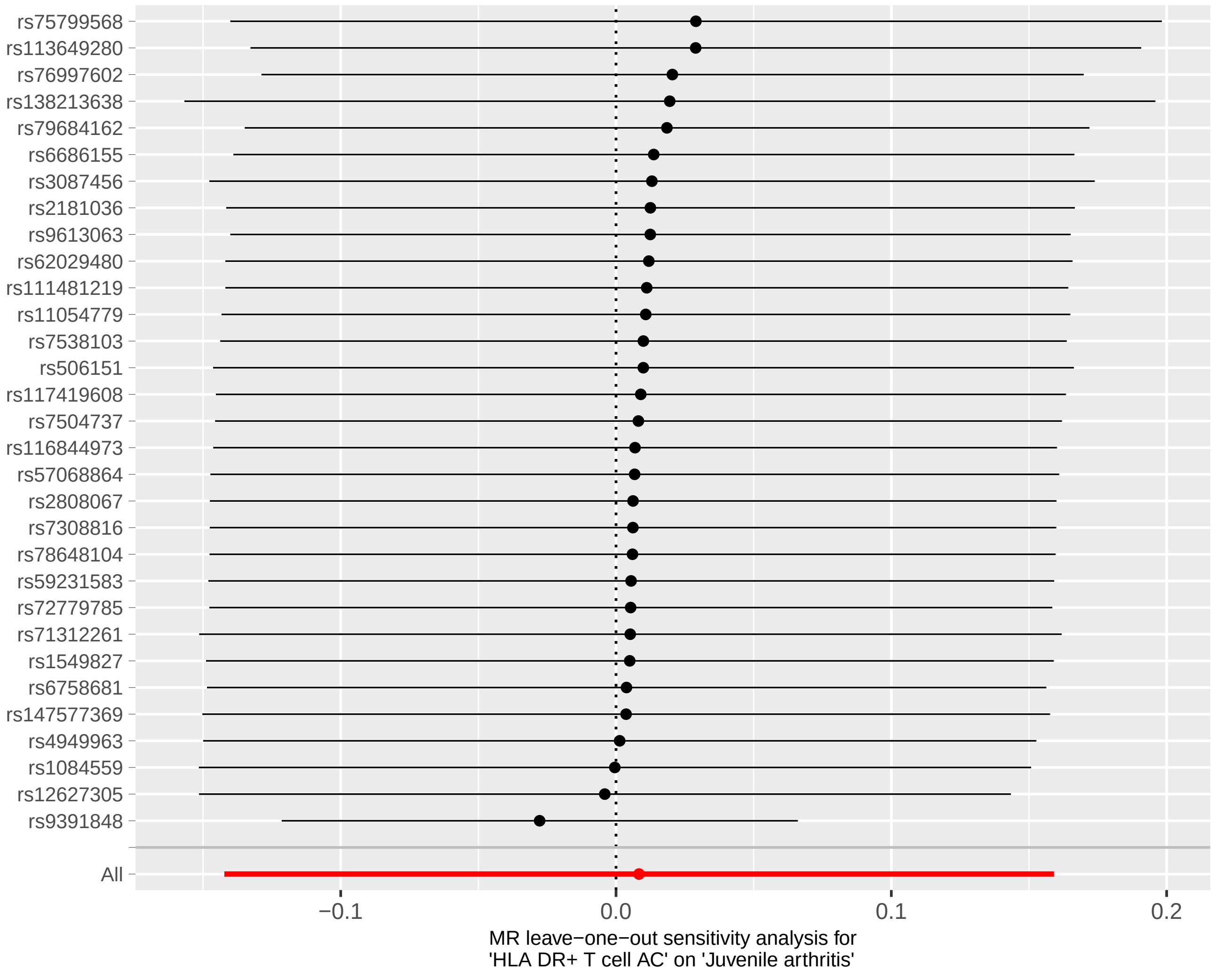

rs75799568
rs113649280
rs76997602
rs138213638
rs79684162
rs6686155
rs3087456
rs2181036
rs9613063
rs62029480
rs111481219
rs11054779
rs7538103
rs506151
rs117419608
rs7504737
rs116844973
rs57068864
rs2808067
rs7308816
rs78648104
rs59231583
rs72779785
rs71312261
rs1549827
rs6758681
rs147577369
rs4949963
rs1084559
rs12627305
rs9391848
All
−0.1
0.0
0.1
0.2
MR leave−one−out sensitivity analysis for
'HLA DR+ T cell AC' on 'Juvenile arthritis'

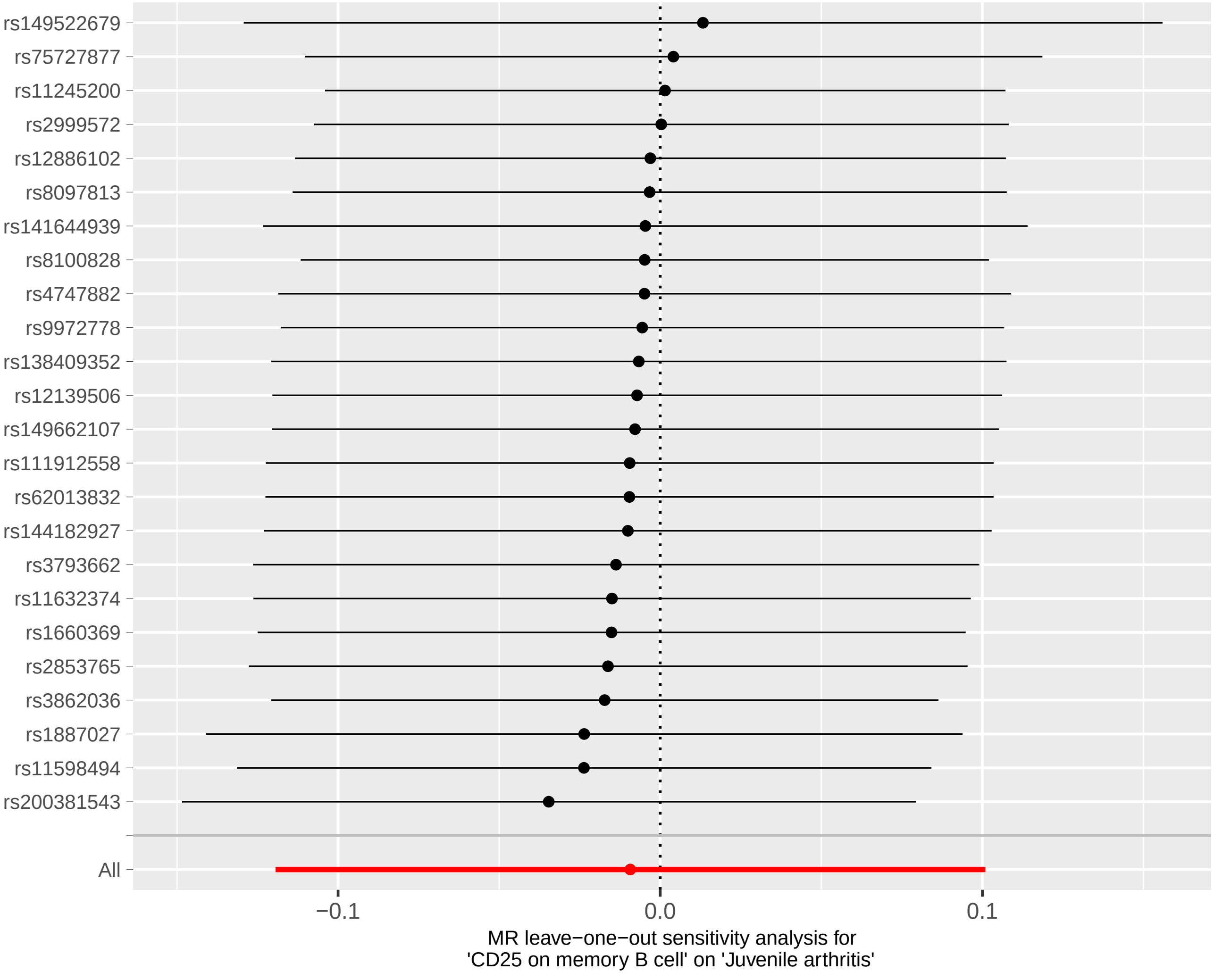

rs149522679
rs75727877
rs11245200
rs2999572
rs12886102
rs8097813
rs141644939
rs8100828
rs4747882
rs9972778
rs138409352
rs12139506
rs149662107
rs111912558
rs62013832
rs144182927
rs3793662
rs11632374
rs1660369
rs2853765
rs3862036
rs1887027
rs11598494
rs200381543
All
−0.1
0.0
0.1
MR leave−one−out sensitivity analysis for
'CD25 on memory B cell' on 'Juvenile arthritis'

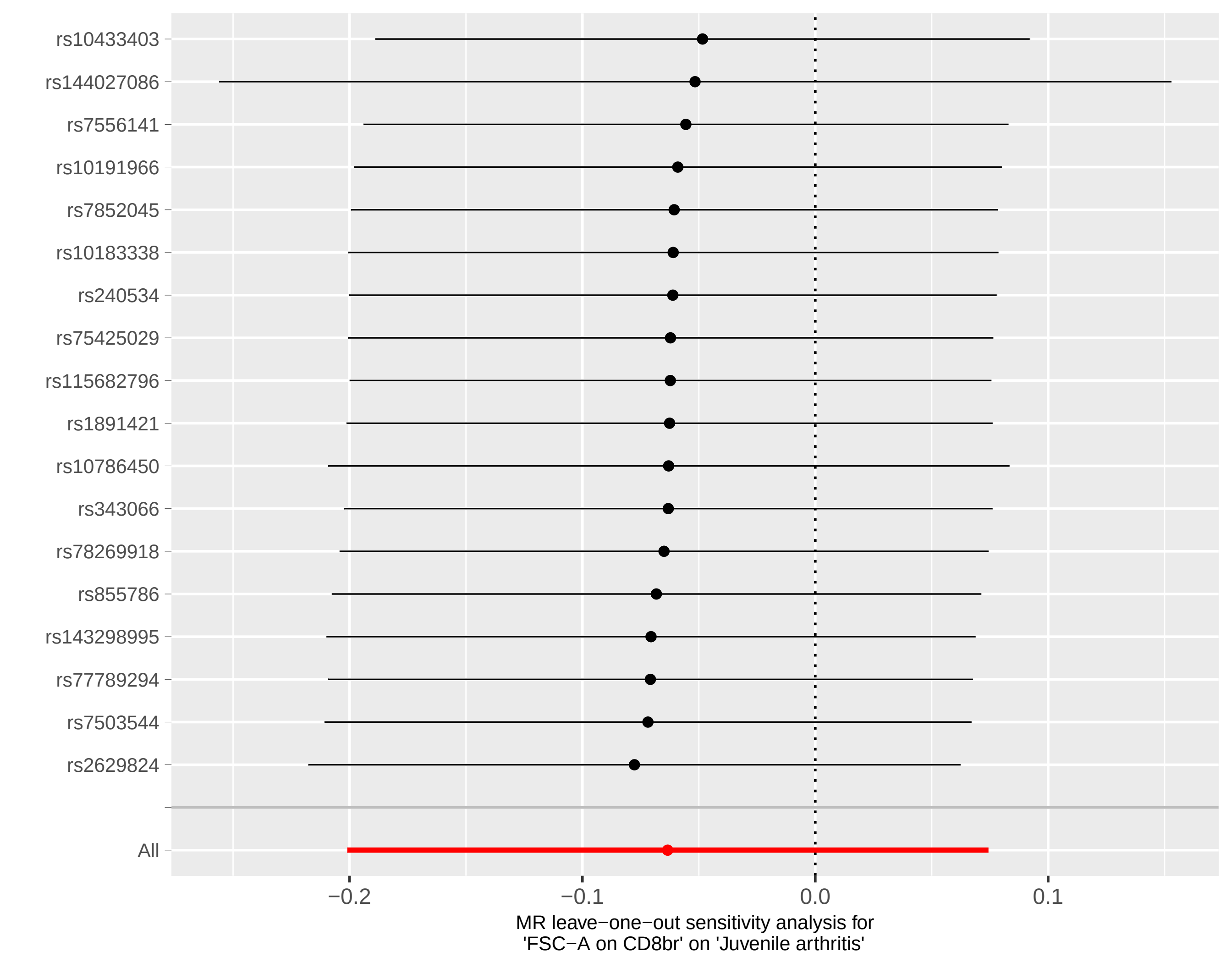
rs10433403
rs144027086
rs7556141
rs10191966
rs7852045
rs10183338
rs240534
rs75425029
rs115682796
rs1891421
rs10786450
rs343066
rs78269918
rs855786
rs143298995
rs77789294
rs7503544
rs2629824
All
−0.2
−0.1
0.0
0.1
MR leave−one−out sensitivity analysis for
'FSC−A on CD8br' on 'Juvenile arthritis'

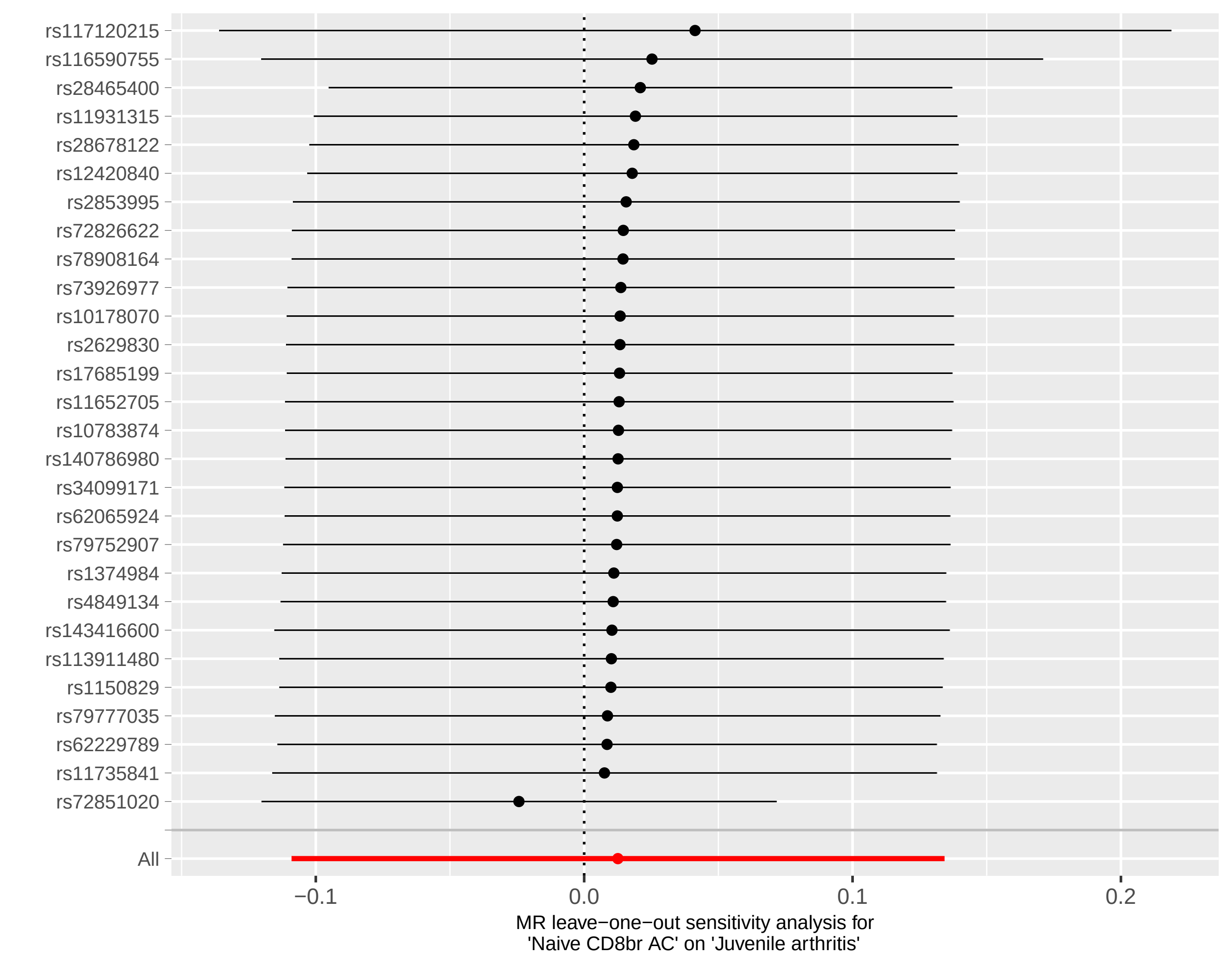

rs117120215
rs116590755
rs28465400
rs11931315
rs28678122
rs12420840
rs2853995
rs72826622
rs78908164
rs73926977
rs10178070
rs2629830
rs17685199
rs11652705
rs10783874
rs140786980
rs34099171
rs62065924
rs79752907
rs1374984
rs4849134
rs143416600
rs113911480
rs1150829
rs79777035
rs62229789
rs11735841
rs72851020
All
−0.1
0.0
0.1
0.2
MR leave−one−out sensitivity analysis for
'Naive CD8br AC' on 'Juvenile arthritis'

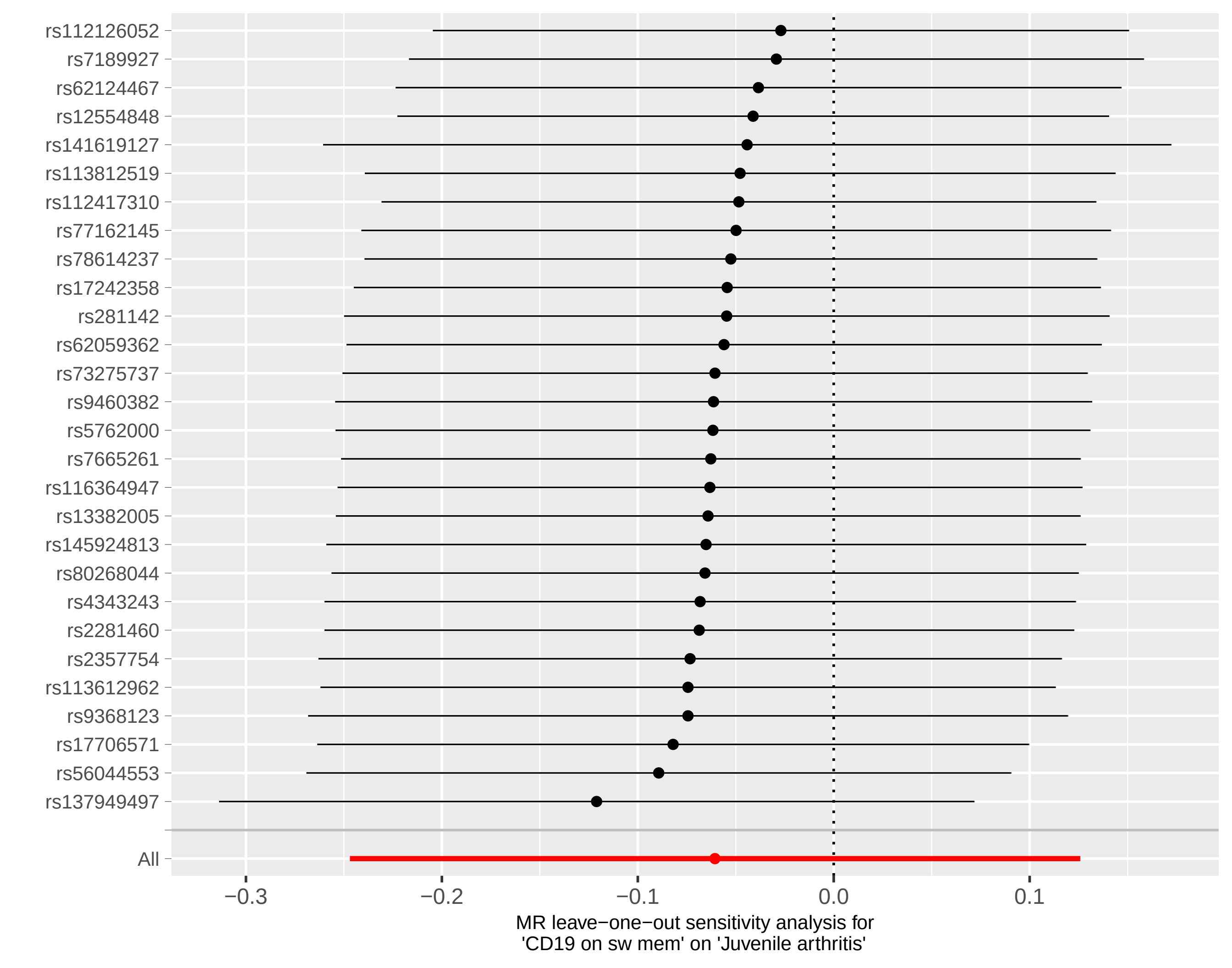

rs112126052
rs7189927
rs62124467
rs12554848
rs141619127
rs113812519
rs112417310
rs77162145
rs78614237
rs17242358
rs281142
rs62059362
rs73275737
rs9460382
rs5762000
rs7665261
rs116364947
rs13382005
rs145924813
rs80268044
rs4343243
rs2281460
rs2357754
rs113612962
rs9368123
rs17706571
rs56044553
rs137949497
All
−0.3
−0.2
−0.1
0.0
0.1
MR leave−one−out sensitivity analysis for
'CD19 on sw mem' on 'Juvenile arthritis'

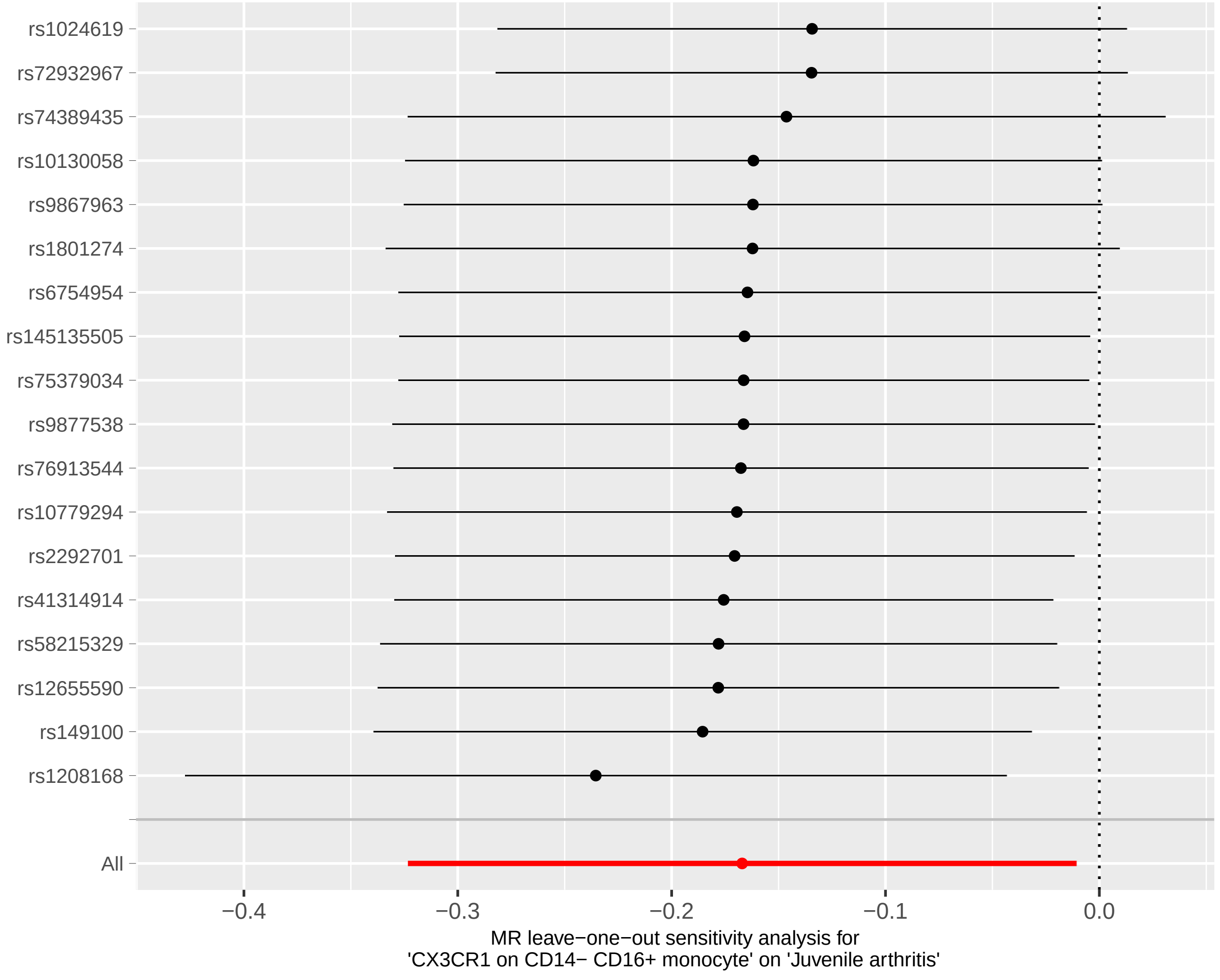
rs1024619
rs72932967
rs74389435
rs10130058
rs9867963
rs1801274
rs6754954
rs145135505
rs75379034
rs9877538
rs76913544
rs10779294
rs2292701
rs41314914
rs58215329
rs12655590
rs149100
rs1208168
All
−0.4
−0.3
−0.2
−0.1
0.0
MR leave−one−out sensitivity analysis for
'CX3CR1 on CD14− CD16+ monocyte' on 'Juvenile arthritis'

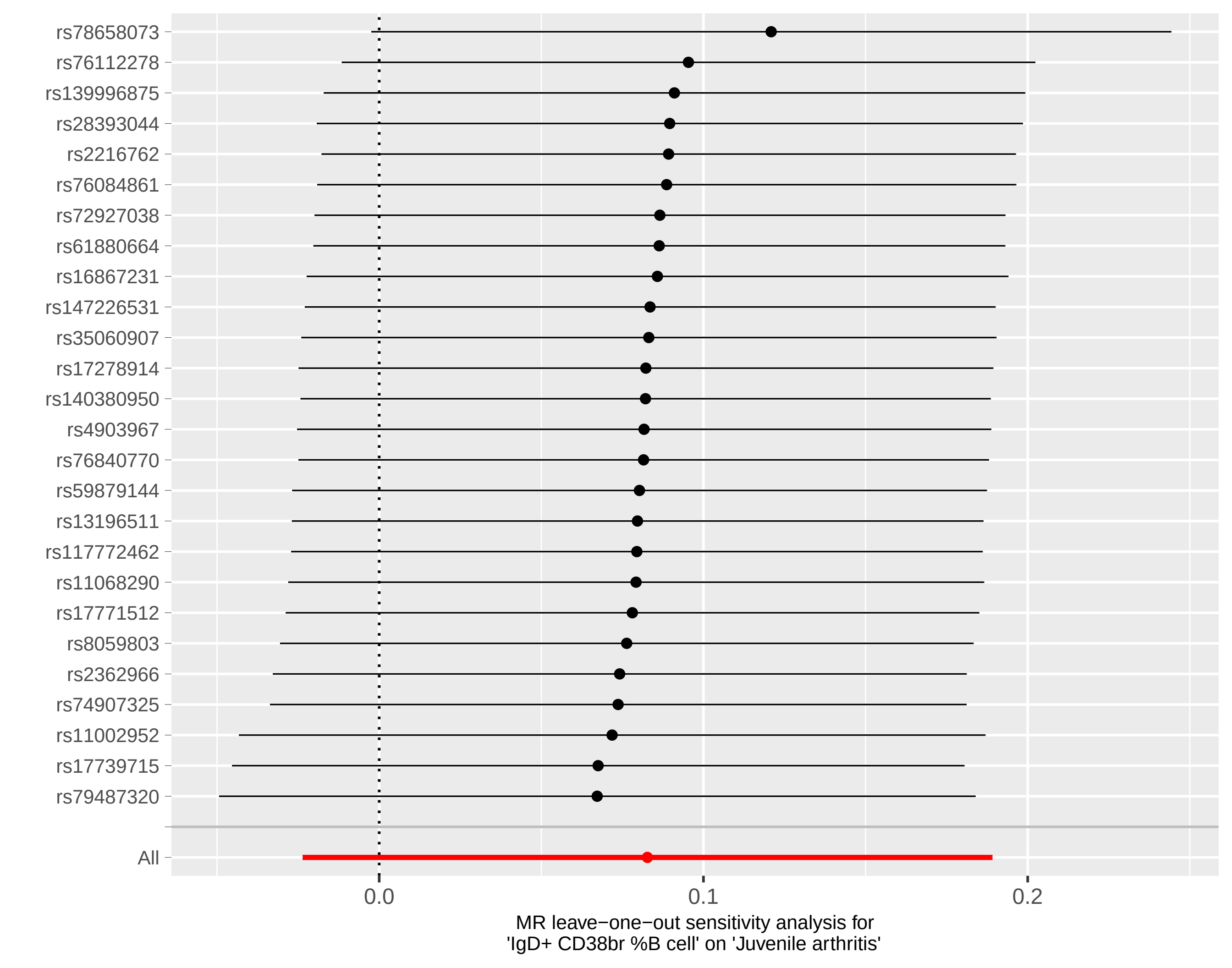

rs78658073
rs76112278
rs139996875
rs28393044
rs2216762
rs76084861
rs72927038
rs61880664
rs16867231
rs147226531
rs35060907
rs17278914
rs140380950
rs4903967
rs76840770
rs59879144
rs13196511
rs117772462
rs11068290
rs17771512
rs8059803
rs2362966
rs74907325
rs11002952
rs17739715
rs79487320
All
0.0
0.1
0.2
MR leave−one−out sensitivity analysis for
'IgD+ CD38br %B cell' on 'Juvenile arthritis'

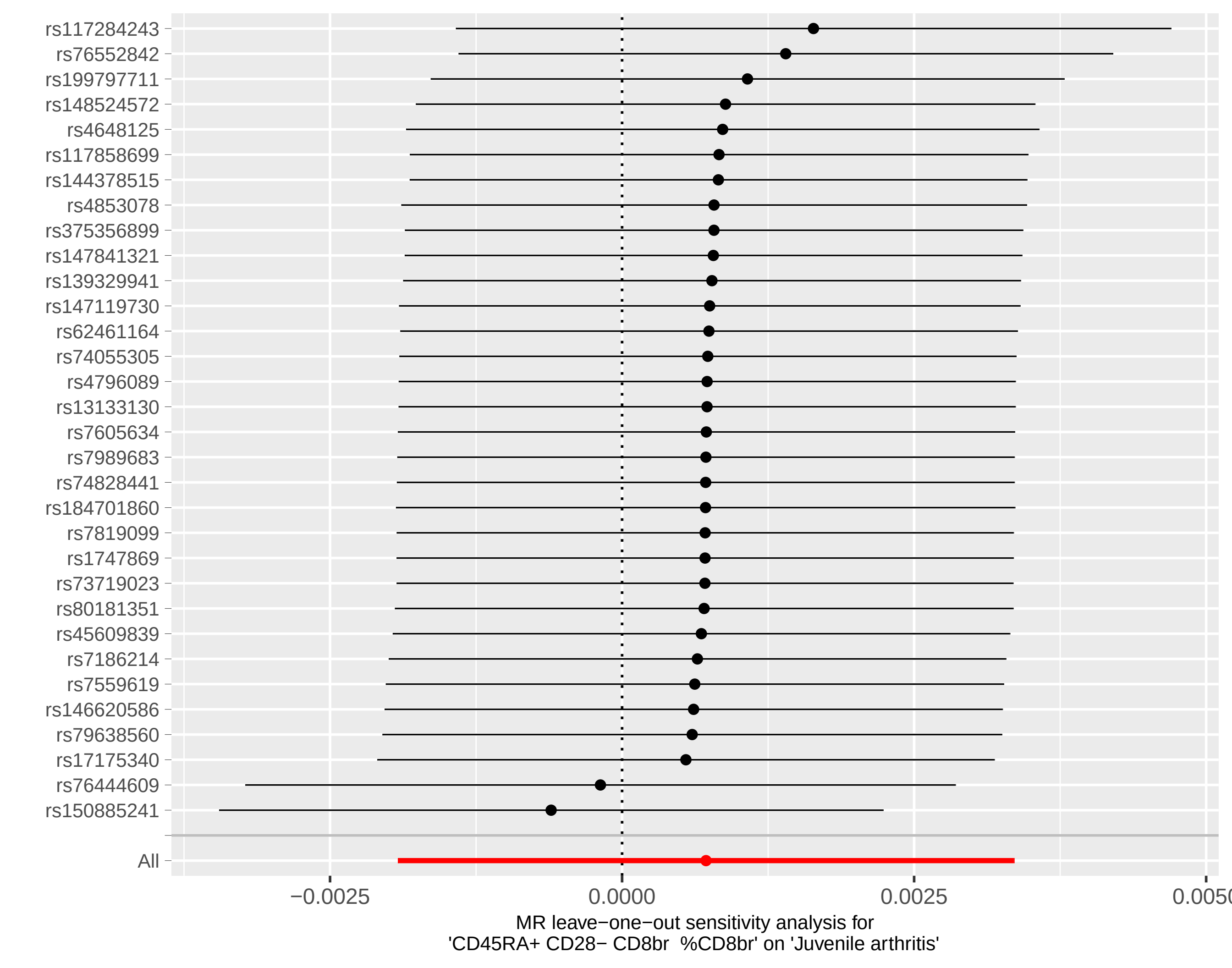
rs117284243
rs76552842
rs199797711
rs148524572
rs4648125
rs117858699
rs144378515
rs4853078
rs375356899
rs147841321
rs139329941
rs147119730
rs62461164
rs74055305
rs4796089
rs13133130
rs7605634
rs7989683
rs74828441
rs184701860
rs7819099
rs1747869
rs73719023
rs80181351
rs45609839
rs7186214
rs7559619
rs146620586
rs79638560
rs17175340
rs76444609
rs150885241
All
−0.0025
0.0000
0.0025
MR leave−one−out sensitivity analysis for
'CD45RA+ CD28− CD8br %CD8br' on 'Juvenile arthritis'

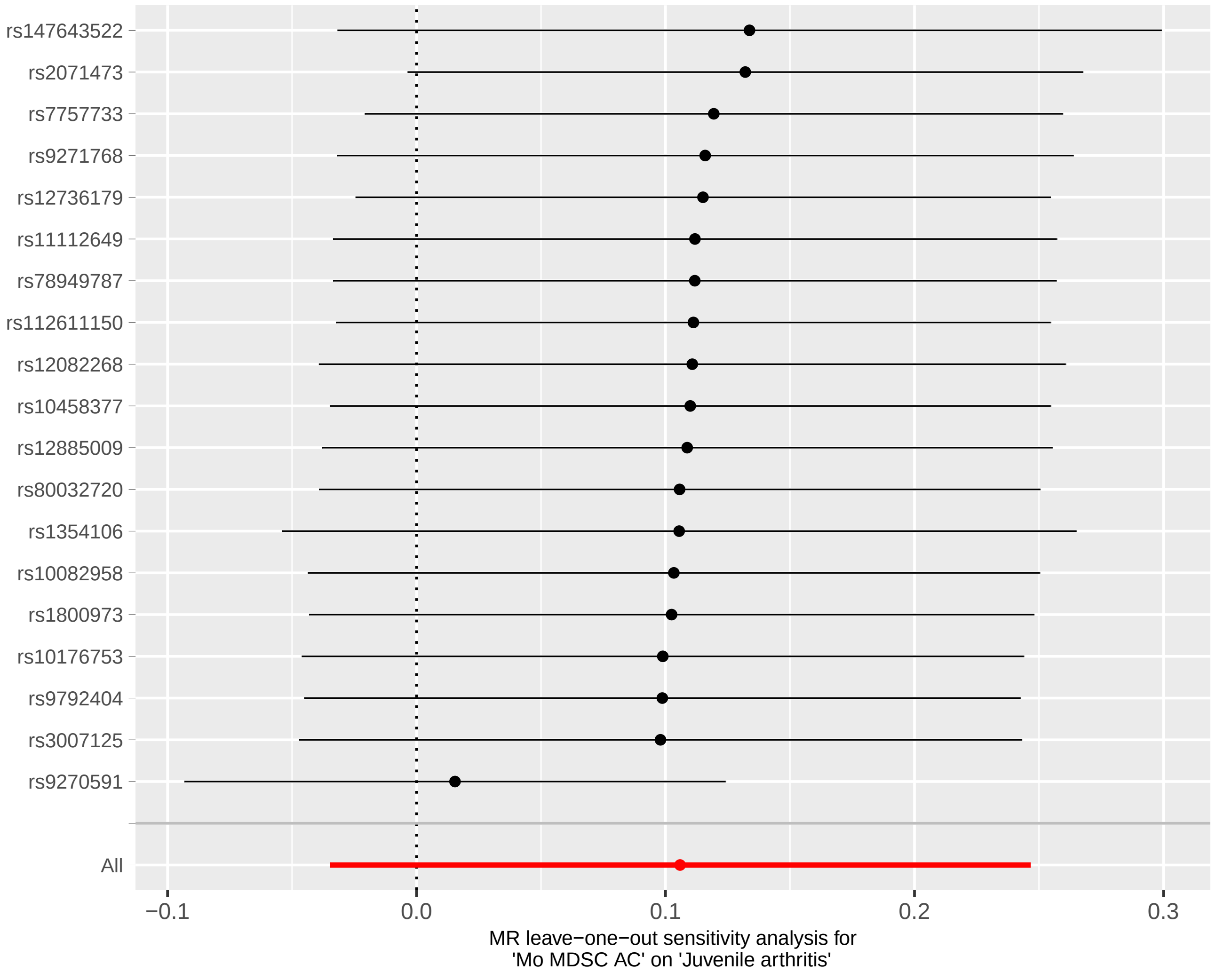

rs147643522
rs2071473
rs7757733
rs9271768
rs12736179
rs11112649
rs78949787
rs112611150
rs12082268
rs10458377
rs12885009
rs80032720
rs1354106
rs10082958
rs1800973
rs10176753
rs9792404
rs3007125
rs9270591
All
−0.1
0.0
0.1
0.2
0.3
MR leave−one−out sensitivity analysis for
'Mo MDSC AC' on 'Juvenile arthritis'

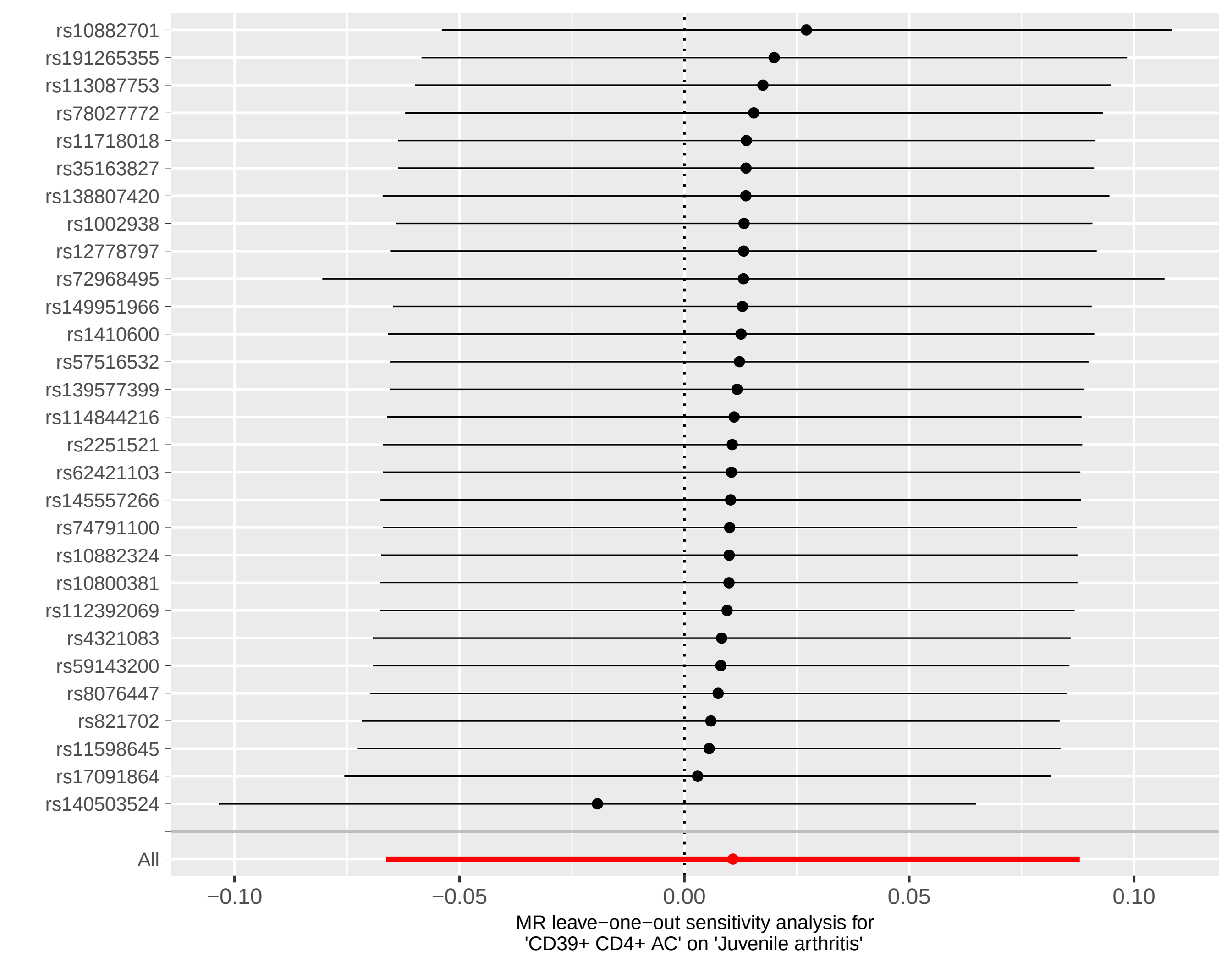

rs10882701
rs191265355
rs113087753
rs78027772
rs11718018
rs35163827
rs138807420
rs1002938
rs12778797
rs72968495
rs149951966
rs1410600
rs57516532
rs139577399
rs114844216
rs2251521
rs62421103
rs145557266
rs74791100
rs10882324
rs10800381
rs112392069
rs4321083
rs59143200
rs8076447
rs821702
rs11598645
rs17091864
rs140503524
All
−0.10
−0.05
0.00
0.05
0.10
MR leave−one−out sensitivity analysis for
'CD39+ CD4+ AC' on 'Juvenile arthritis'

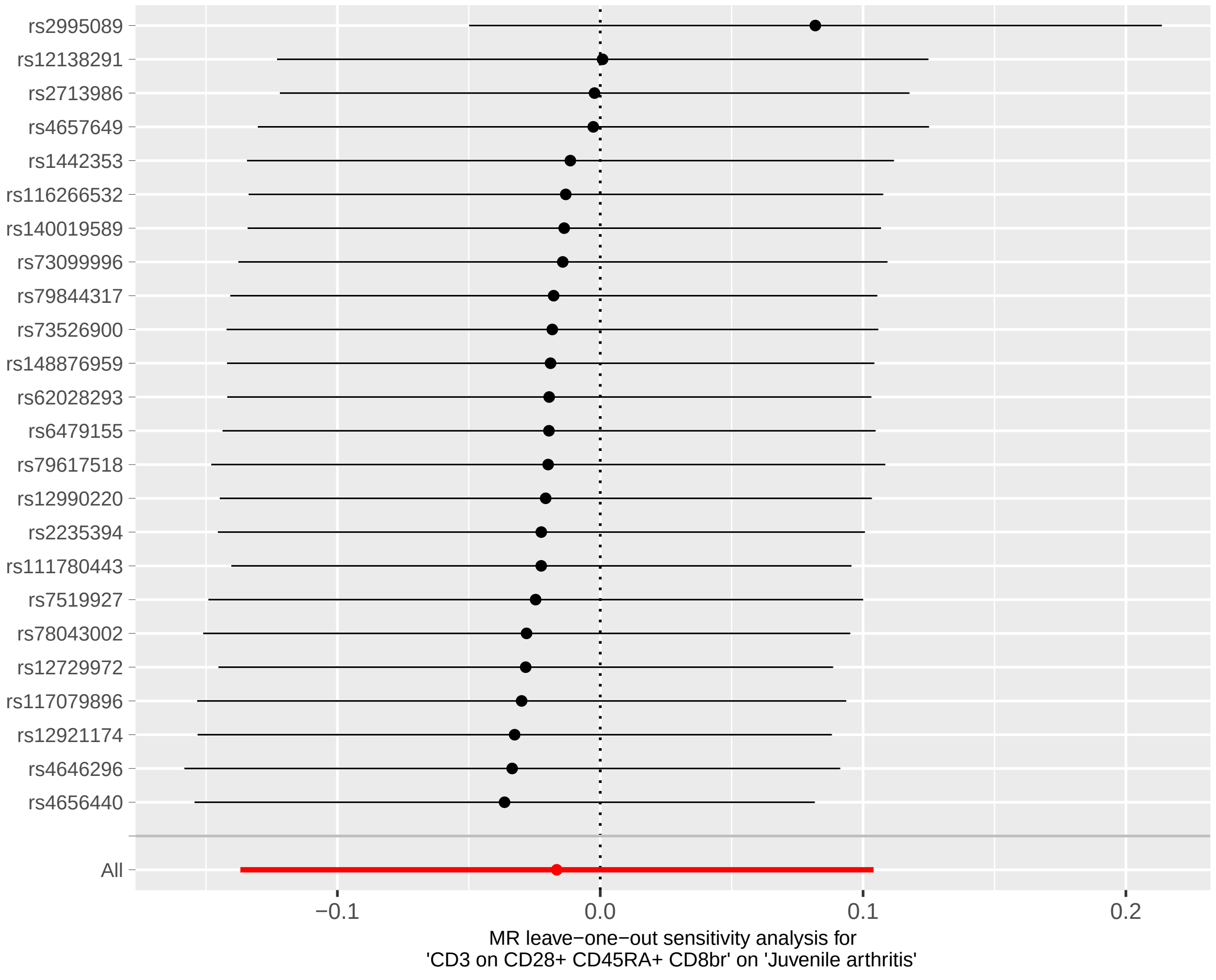

rs2995089
rs12138291
rs2713986
rs4657649
rs1442353
rs116266532
rs140019589
rs73099996
rs79844317
rs73526900
rs148876959
rs62028293
rs6479155
rs79617518
rs12990220
rs2235394
rs111780443
rs7519927
rs78043002
rs12729972
rs117079896
rs12921174
rs4646296
rs4656440
All
−0.1
0.0
0.1
0.2
MR leave−one−out sensitivity analysis for
'CD3 on CD28+ CD45RA+ CD8br' on 'Juvenile arthritis'

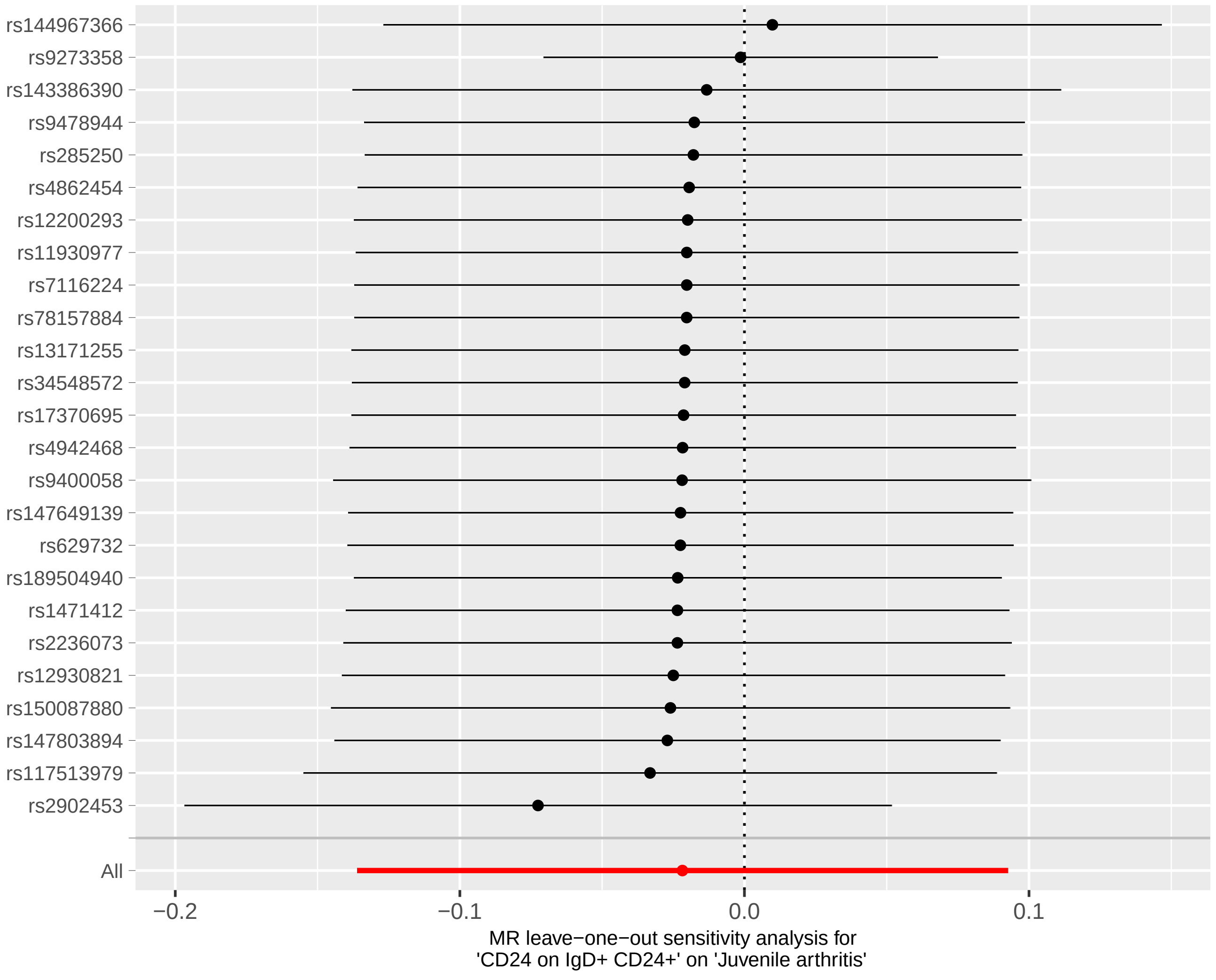

rs144967366
rs9273358
rs143386390
rs9478944
rs285250
rs4862454
rs12200293
rs11930977
rs7116224
rs78157884
rs13171255
rs34548572
rs17370695
rs4942468
rs9400058
rs147649139
rs629732
rs189504940
rs1471412
rs2236073
rs12930821
rs150087880
rs147803894
rs117513979
rs2902453
All
−0.2
−0.1
0.0
0.1
MR leave−one−out sensitivity analysis for
'CD24 on IgD+ CD24+' on 'Juvenile arthritis'

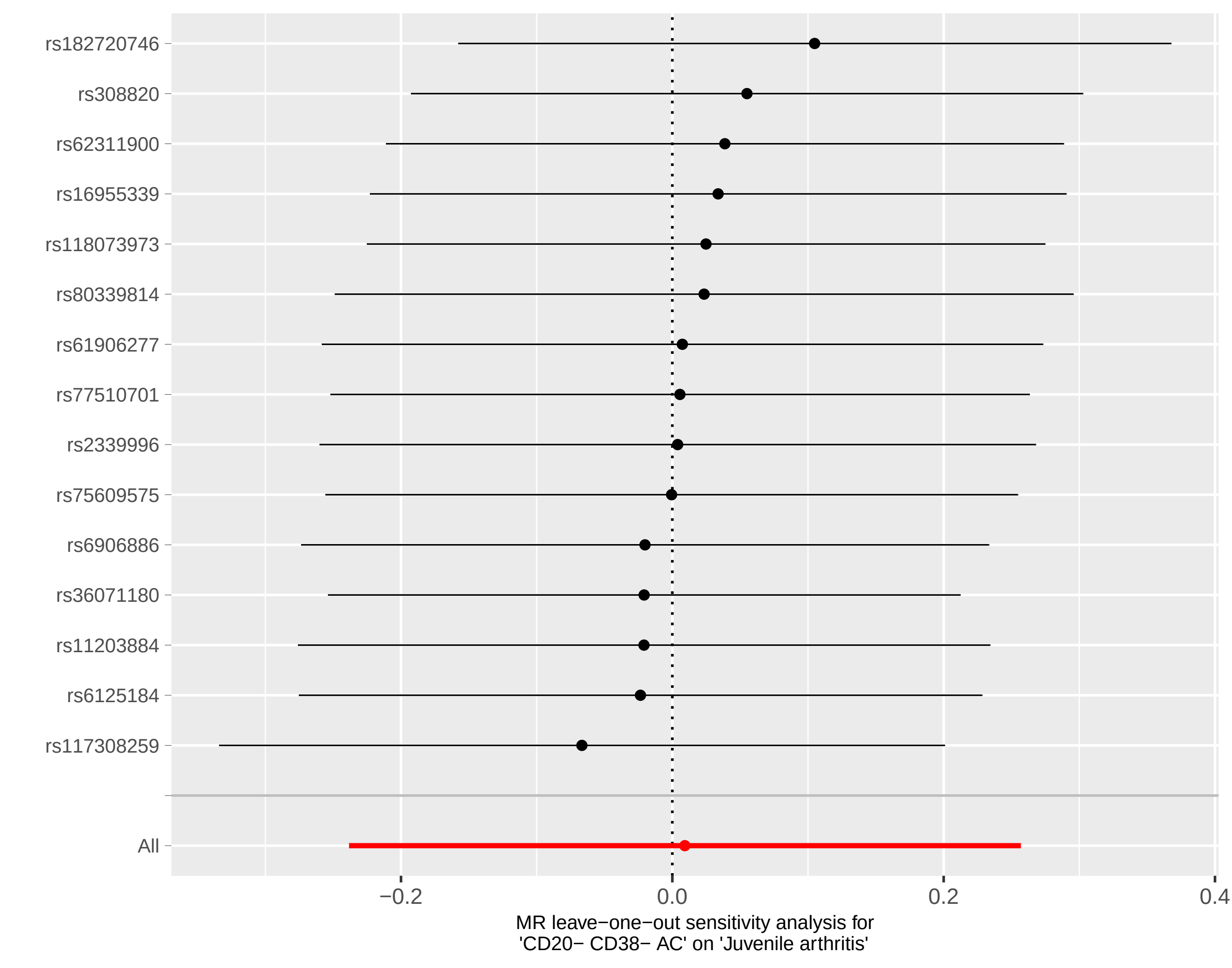
rs182720746
rs308820
rs62311900
rs16955339
rs118073973
rs80339814
rs61906277
rs77510701
rs2339996
rs75609575
rs6906886
rs36071180
rs11203884
rs6125184
rs117308259
All
−0.2
0.0
0.2
0.4
MR leave−one−out sensitivity analysis for
'CD20− CD38− AC' on 'Juvenile arthritis'

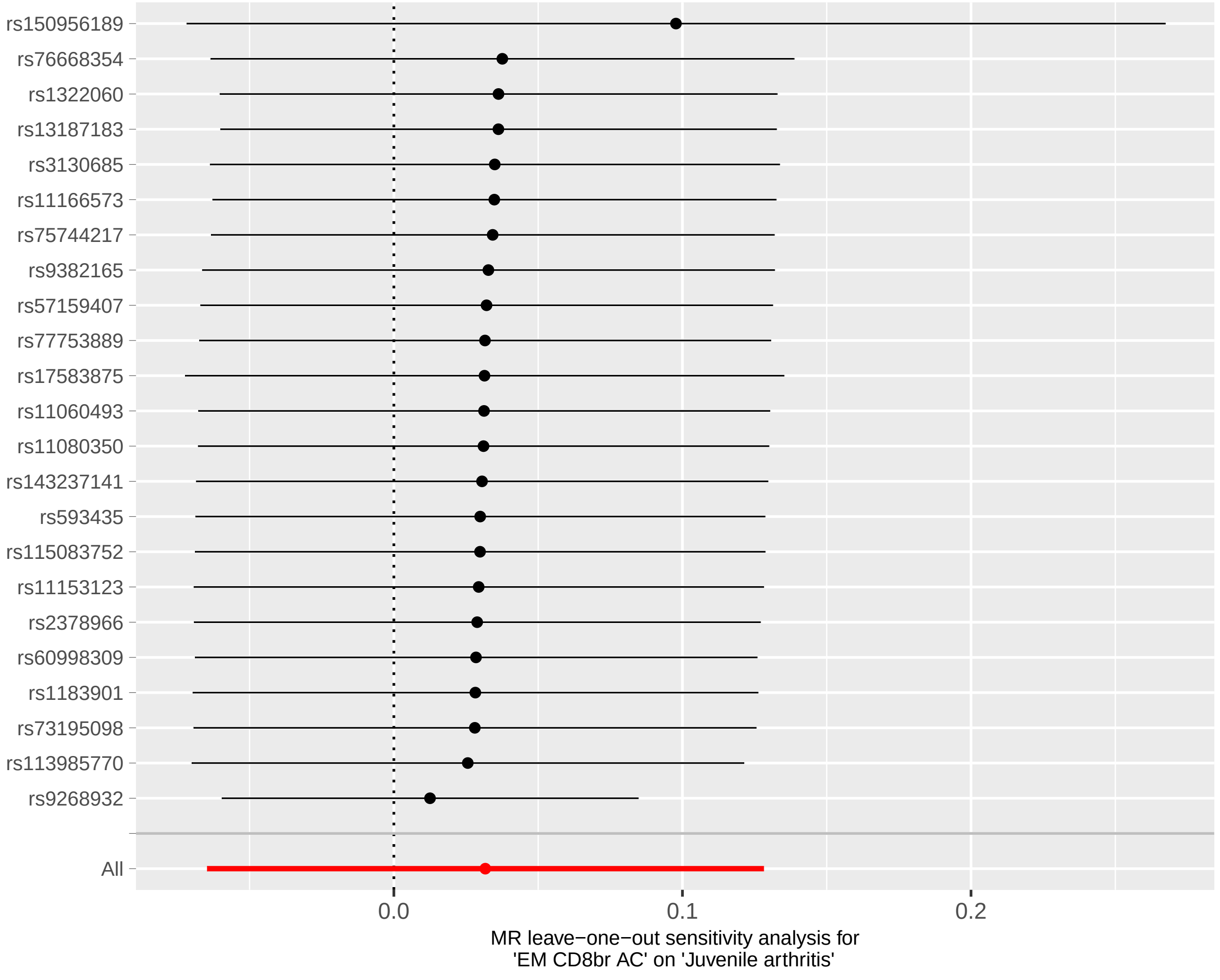
rs150956189
rs76668354
rs1322060
rs13187183
rs3130685
rs11166573
rs75744217
rs9382165
rs57159407
rs77753889
rs17583875
rs11060493
rs11080350
rs143237141
rs593435
rs115083752
rs11153123
rs2378966
rs60998309
rs1183901
rs73195098
rs113985770
rs9268932
All
0.0
0.1
0.2
MR leave−one−out sensitivity analysis for
'EM CD8br AC' on 'Juvenile arthritis'

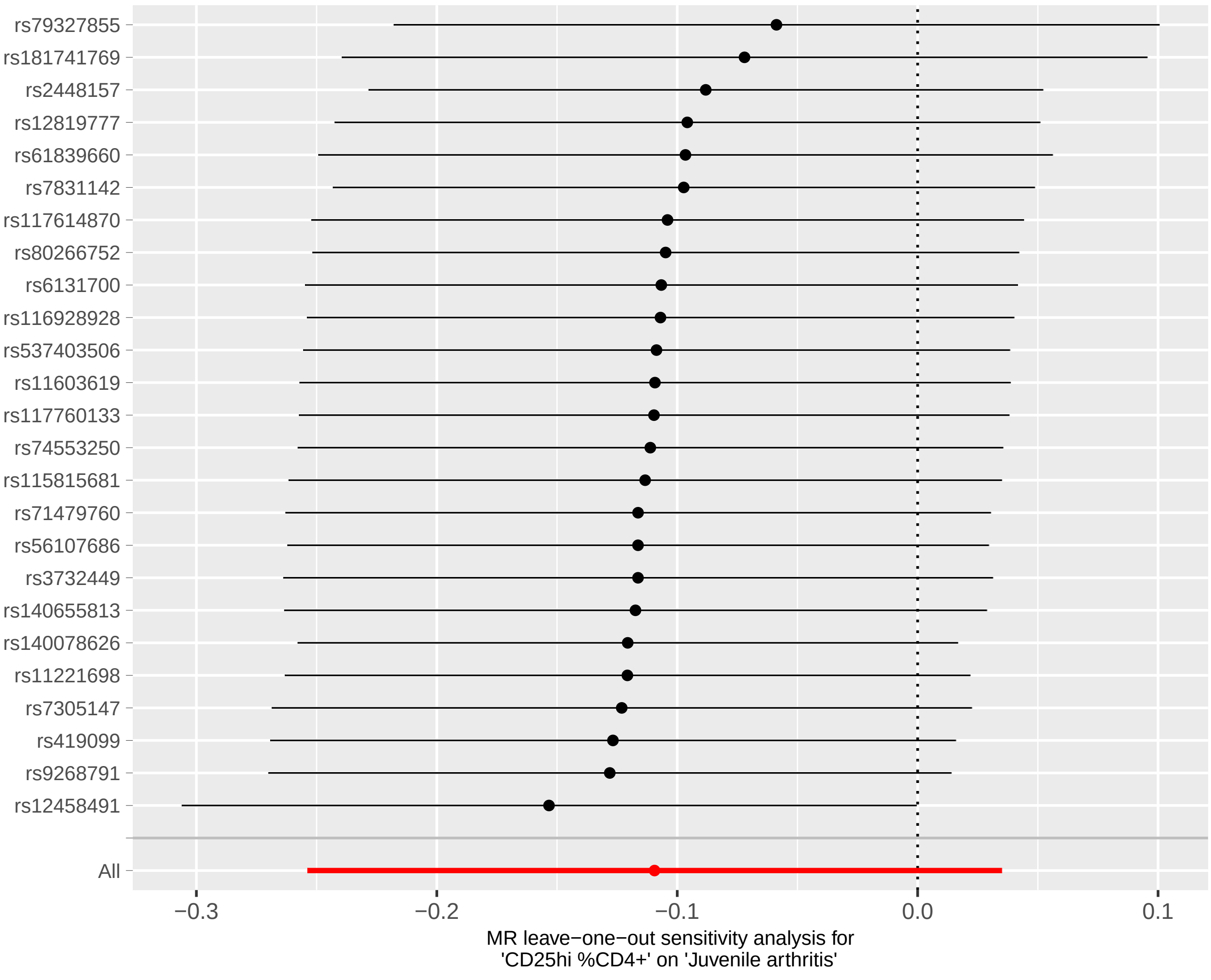
rs79327855
rs181741769
rs2448157
rs12819777
rs61839660
rs7831142
rs117614870
rs80266752
rs6131700
rs116928928
rs537403506
rs11603619
rs117760133
rs74553250
rs115815681
rs71479760
rs56107686
rs3732449
rs140655813
rs140078626
rs11221698
rs7305147
rs419099
rs9268791
rs12458491
All
−0.3
−0.2
−0.1
0.0
0.1
MR leave−one−out sensitivity analysis for
'CD25hi %CD4+' on 'Juvenile arthritis'

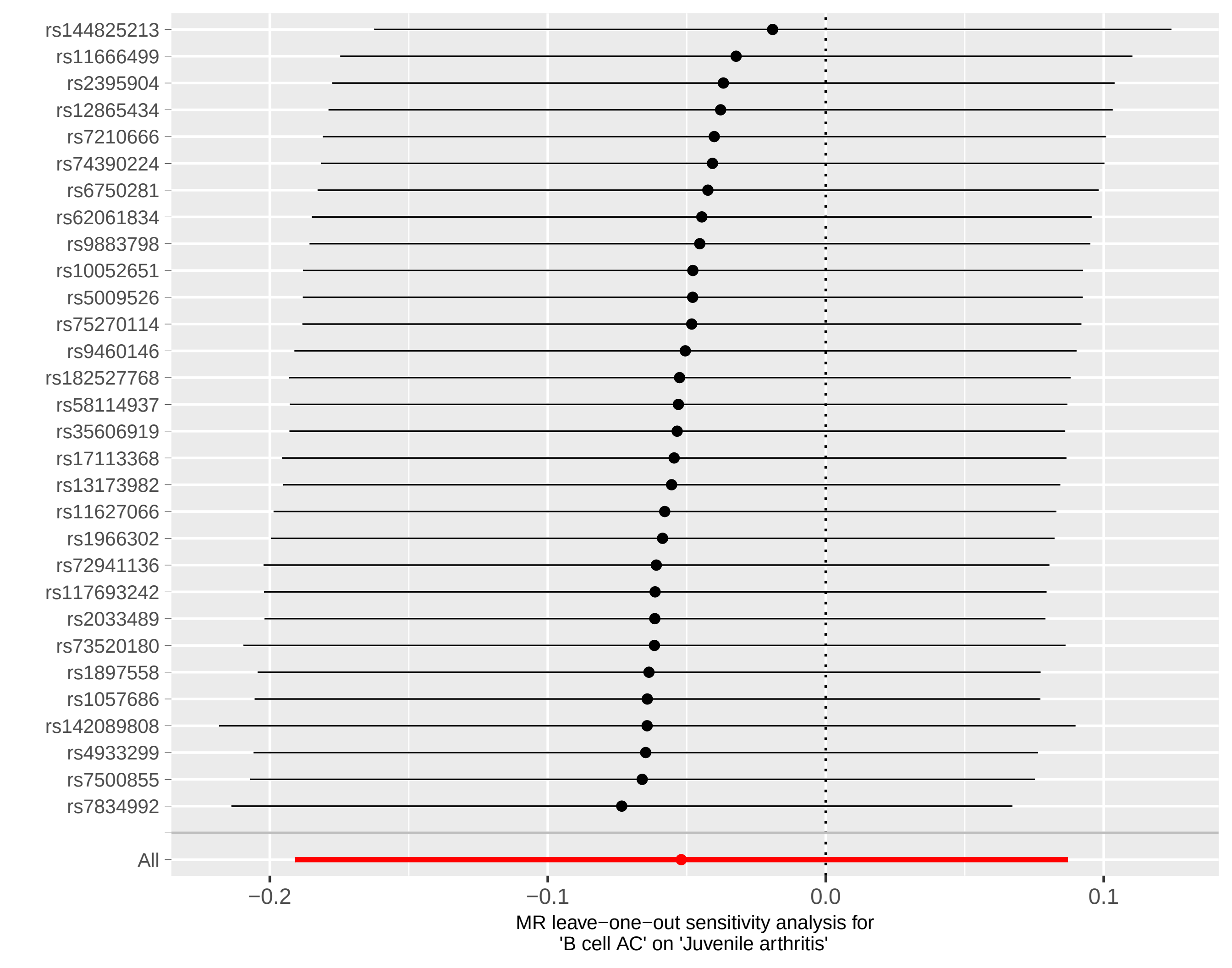
rs144825213
rs11666499
rs2395904
rs12865434
rs7210666
rs74390224
rs6750281
rs62061834
rs9883798
rs10052651
rs5009526
rs75270114
rs9460146
rs182527768
rs58114937
rs35606919
rs17113368
rs13173982
rs11627066
rs1966302
rs72941136
rs117693242
rs2033489
rs73520180
rs1897558
rs1057686
rs142089808
rs4933299
rs7500855
rs7834992
All
−0.2
−0.1
0.0
0.1
MR leave−one−out sensitivity analysis for
'B cell AC' on 'Juvenile arthritis'

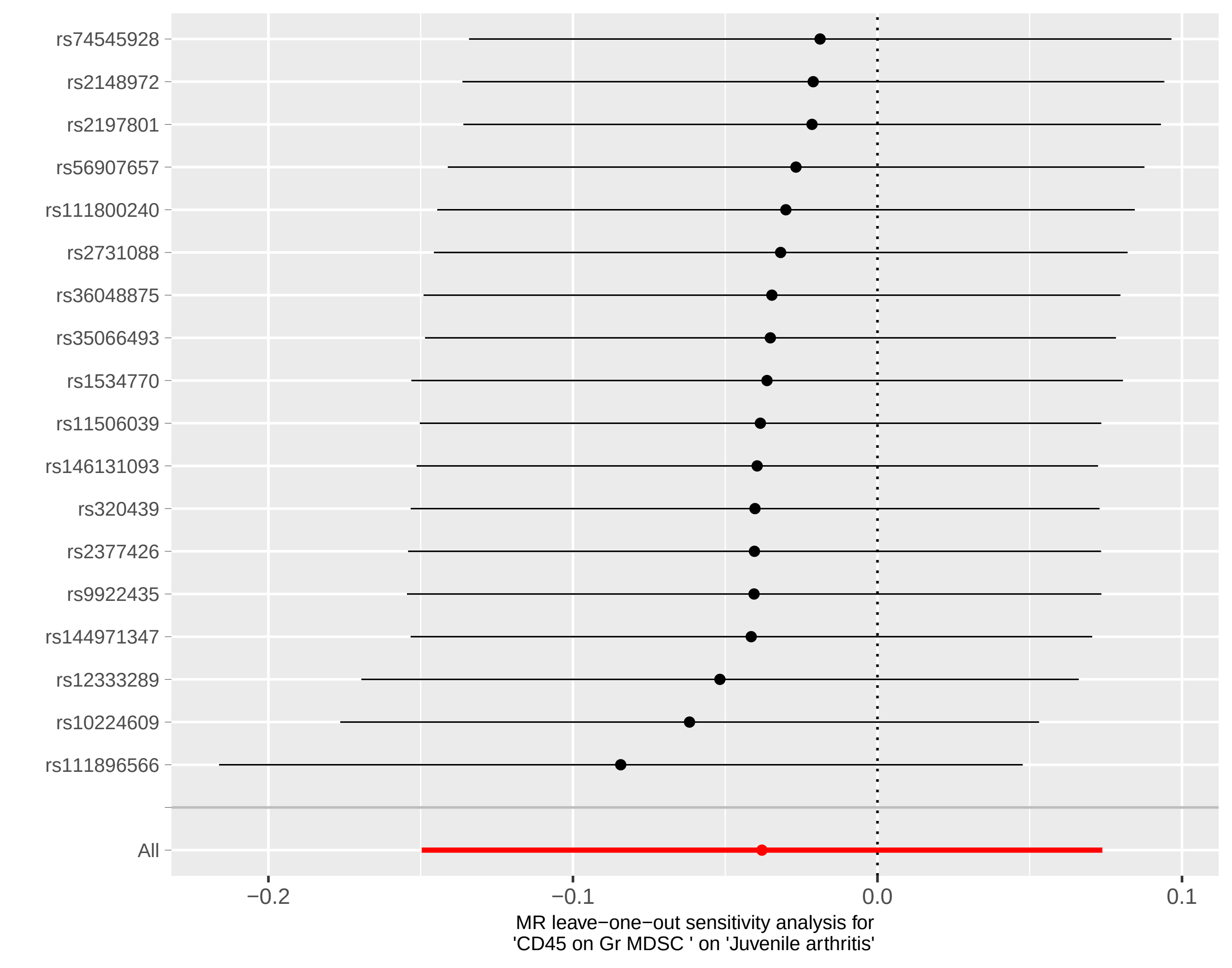
rs74545928
rs2148972
rs2197801
rs56907657
rs111800240
rs2731088
rs36048875
rs35066493
rs1534770
rs11506039
rs146131093
rs320439
rs2377426
rs9922435
rs144971347
rs12333289
rs10224609
rs111896566
All
−0.2
−0.1
0.0
0.1
MR leave−one−out sensitivity analysis for
'CD45 on Gr MDSC ' on 'Juvenile arthritis'

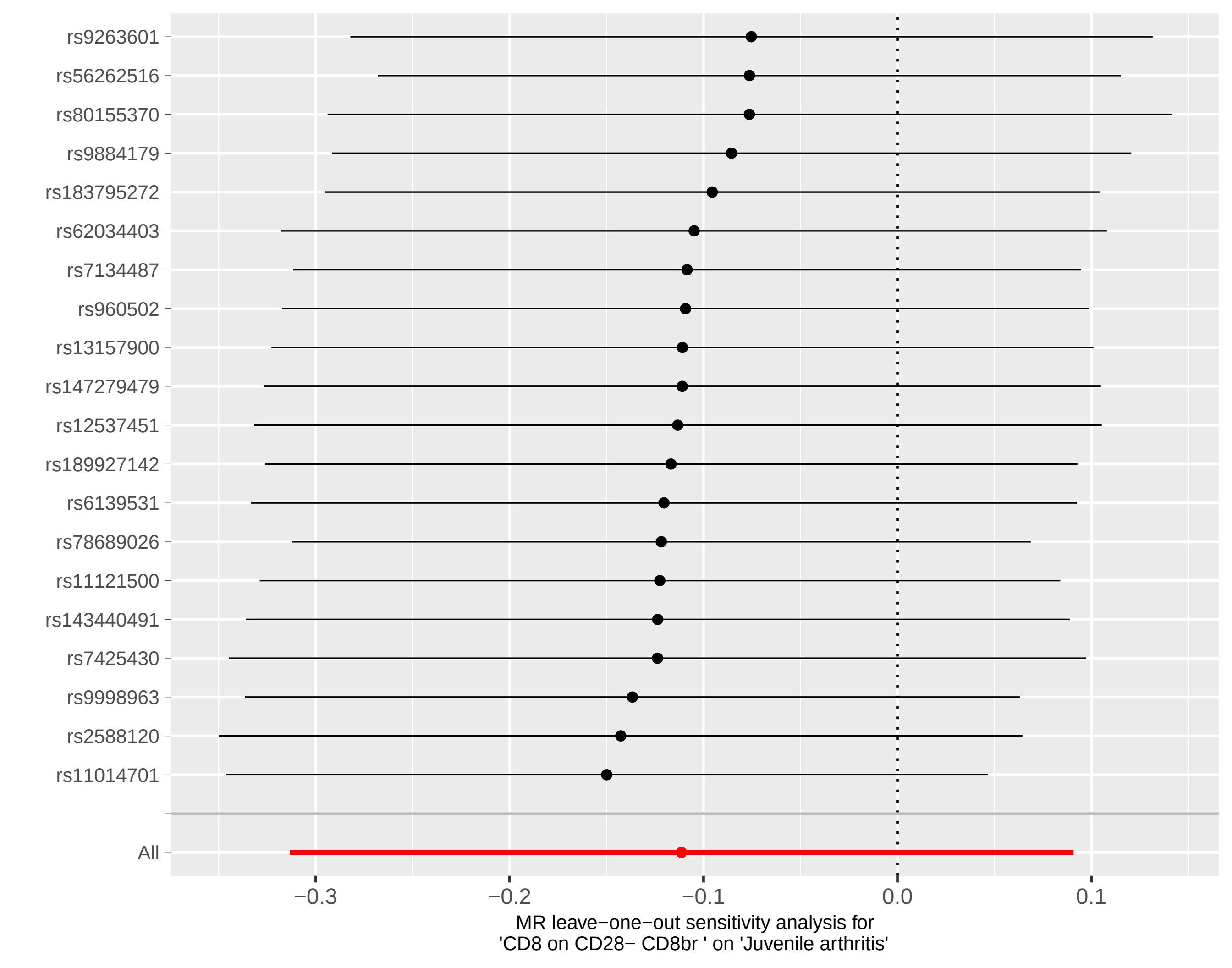

rs9263601
rs56262516
rs80155370
rs9884179
rs183795272
rs62034403
rs7134487
rs960502
rs13157900
rs147279479
rs12537451
rs189927142
rs6139531
rs78689026
rs11121500
rs143440491
rs7425430
rs9998963
rs2588120
rs11014701
All
−0.3
−0.2
−0.1
0.0
0.1
MR leave−one−out sensitivity analysis for
'CD8 on CD28− CD8br ' on 'Juvenile arthritis'

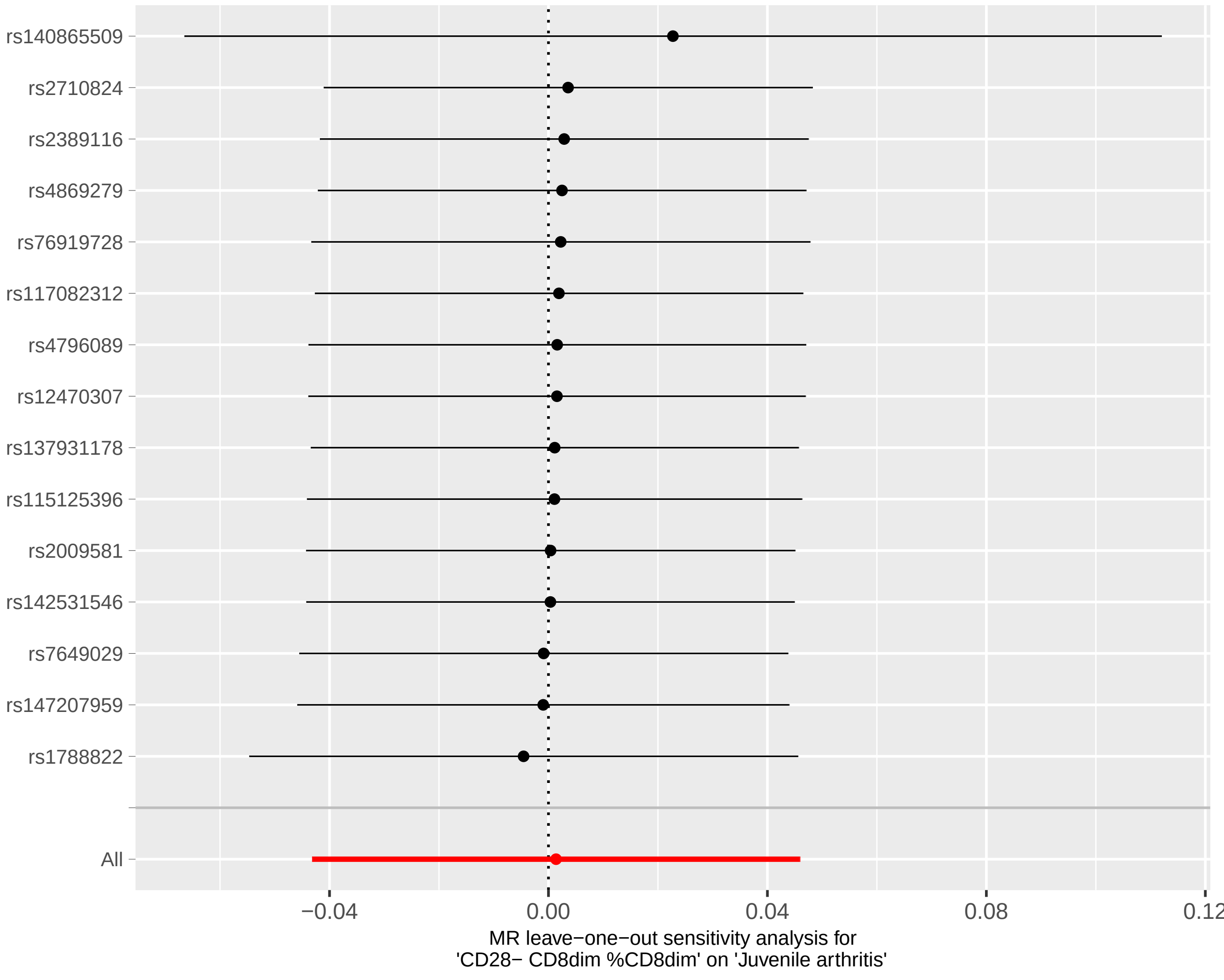

rs140865509
rs2710824
rs2389116
rs4869279
rs76919728
rs117082312
rs4796089
rs12470307
rs137931178
rs115125396
rs2009581
rs142531546
rs7649029
rs147207959
rs1788822
All
−0.04
0.00
0.04
0.08
0.12
MR leave−one−out sensitivity analysis for
'CD28− CD8dim %CD8dim' on 'Juvenile arthritis'

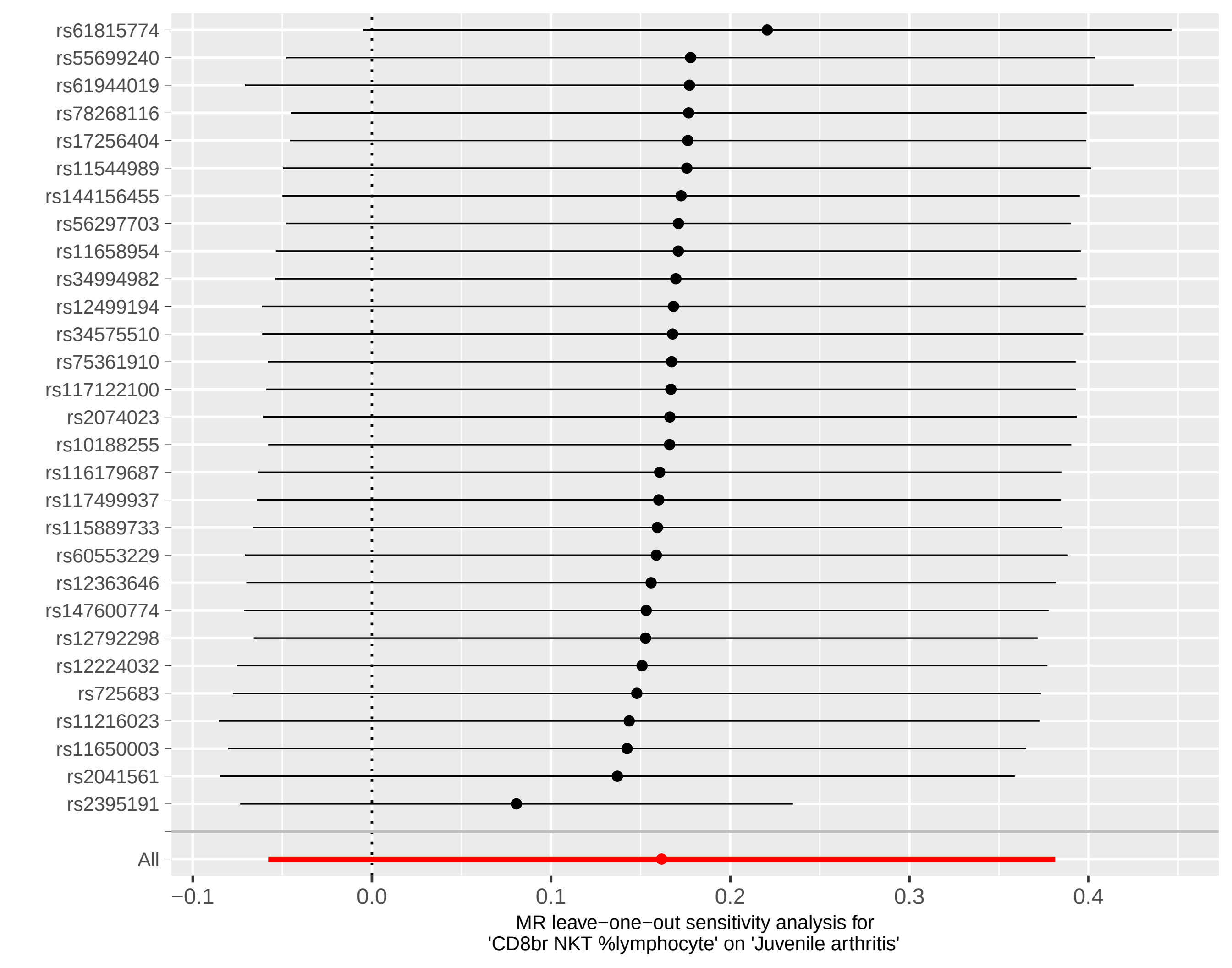

rs61815774
rs55699240
rs61944019
rs78268116
rs17256404
rs11544989
rs144156455
rs56297703
rs11658954
rs34994982
rs12499194
rs34575510
rs75361910
rs117122100
rs2074023
rs10188255
rs116179687
rs117499937
rs115889733
rs60553229
rs12363646
rs147600774
rs12792298
rs12224032
rs725683
rs11216023
rs11650003
rs2041561
rs2395191
All
−0.1
0.0
0.1
0.2
0.3
0.4
MR leave−one−out sensitivity analysis for
'CD8br NKT %lymphocyte' on 'Juvenile arthritis'

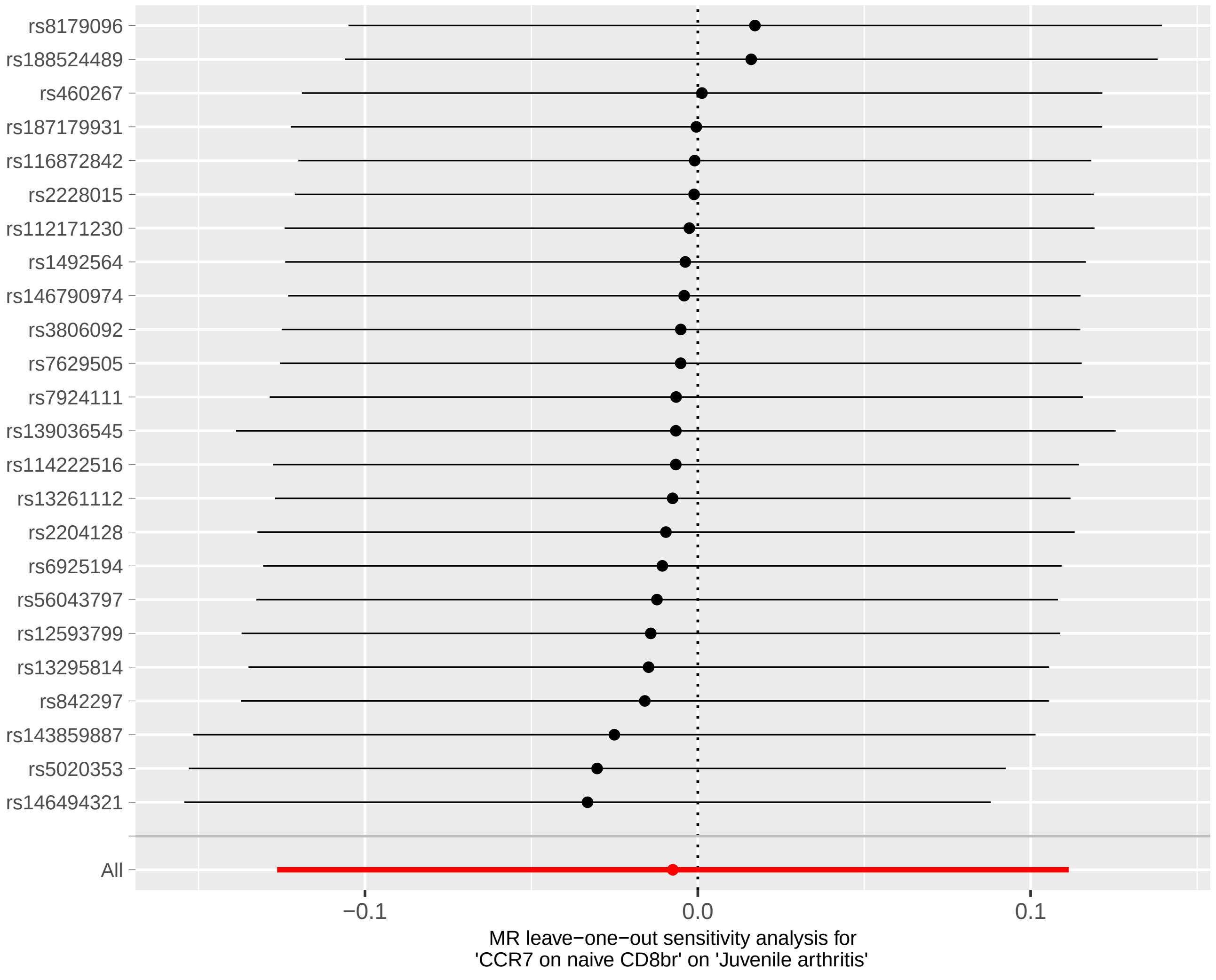
rs8179096
rs188524489
rs460267
rs187179931
rs116872842
rs2228015
rs112171230
rs1492564
rs146790974
rs3806092
rs7629505
rs7924111
rs139036545
rs114222516
rs13261112
rs2204128
rs6925194
rs56043797
rs12593799
rs13295814
rs842297
rs143859887
rs5020353
rs146494321
All
−0.1
0.0
0.1
MR leave−one−out sensitivity analysis for
'CCR7 on naive CD8br' on 'Juvenile arthritis'

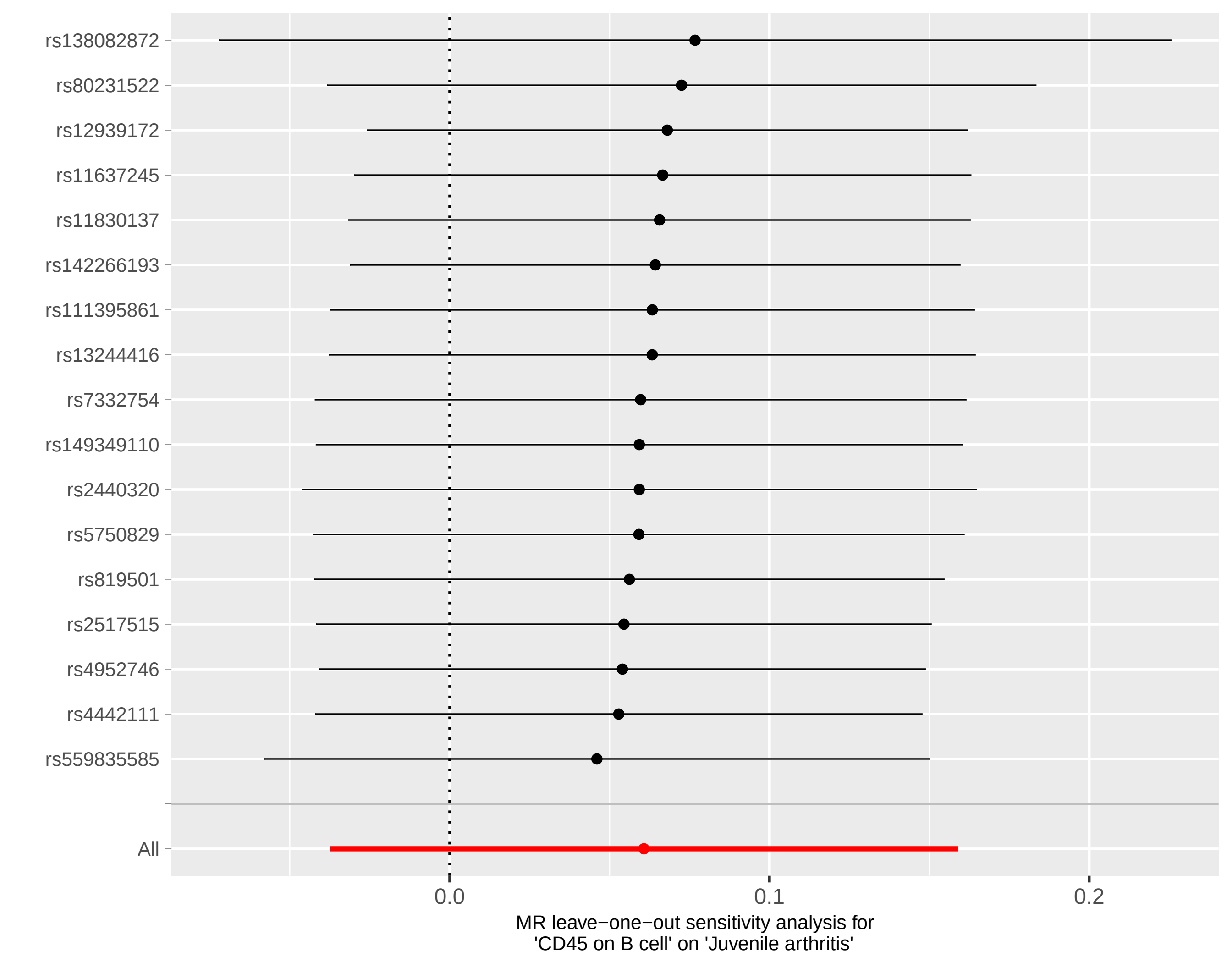
rs138082872
rs80231522
rs12939172
rs11637245
rs11830137
rs142266193
rs111395861
rs13244416
rs7332754
rs149349110
rs2440320
rs5750829
rs819501
rs2517515
rs4952746
rs4442111
rs559835585
All
0.0
0.1
0.2
MR leave−one−out sensitivity analysis for
'CD45 on B cell' on 'Juvenile arthritis'

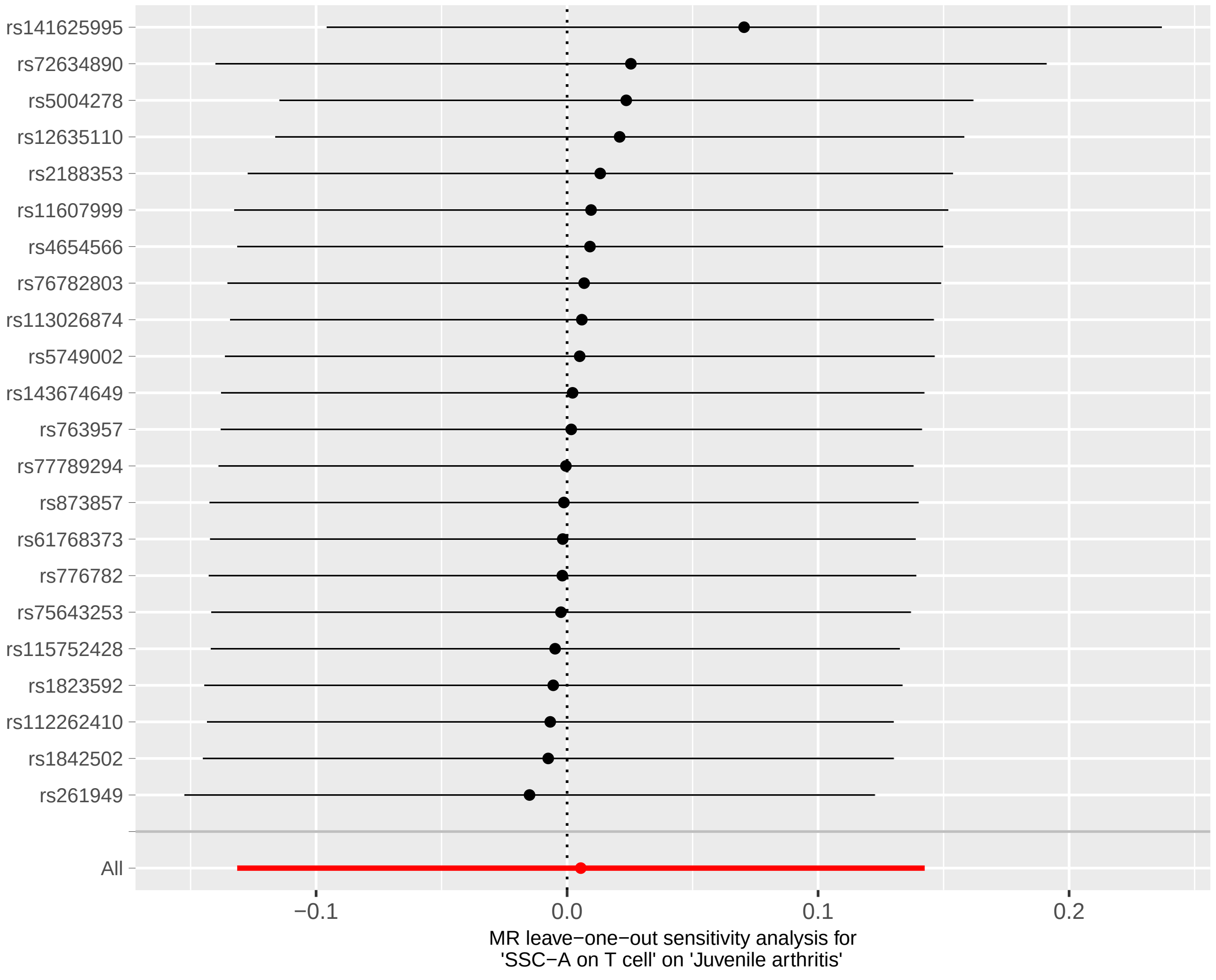
rs141625995
rs72634890
rs5004278
rs12635110
rs2188353
rs11607999
rs4654566
rs76782803
rs113026874
rs5749002
rs143674649
rs763957
rs77789294
rs873857
rs61768373
rs776782
rs75643253
rs115752428
rs1823592
rs112262410
rs1842502
rs261949
All
−0.1
0.0
0.1
0.2
MR leave−one−out sensitivity analysis for
'SSC−A on T cell' on 'Juvenile arthritis'

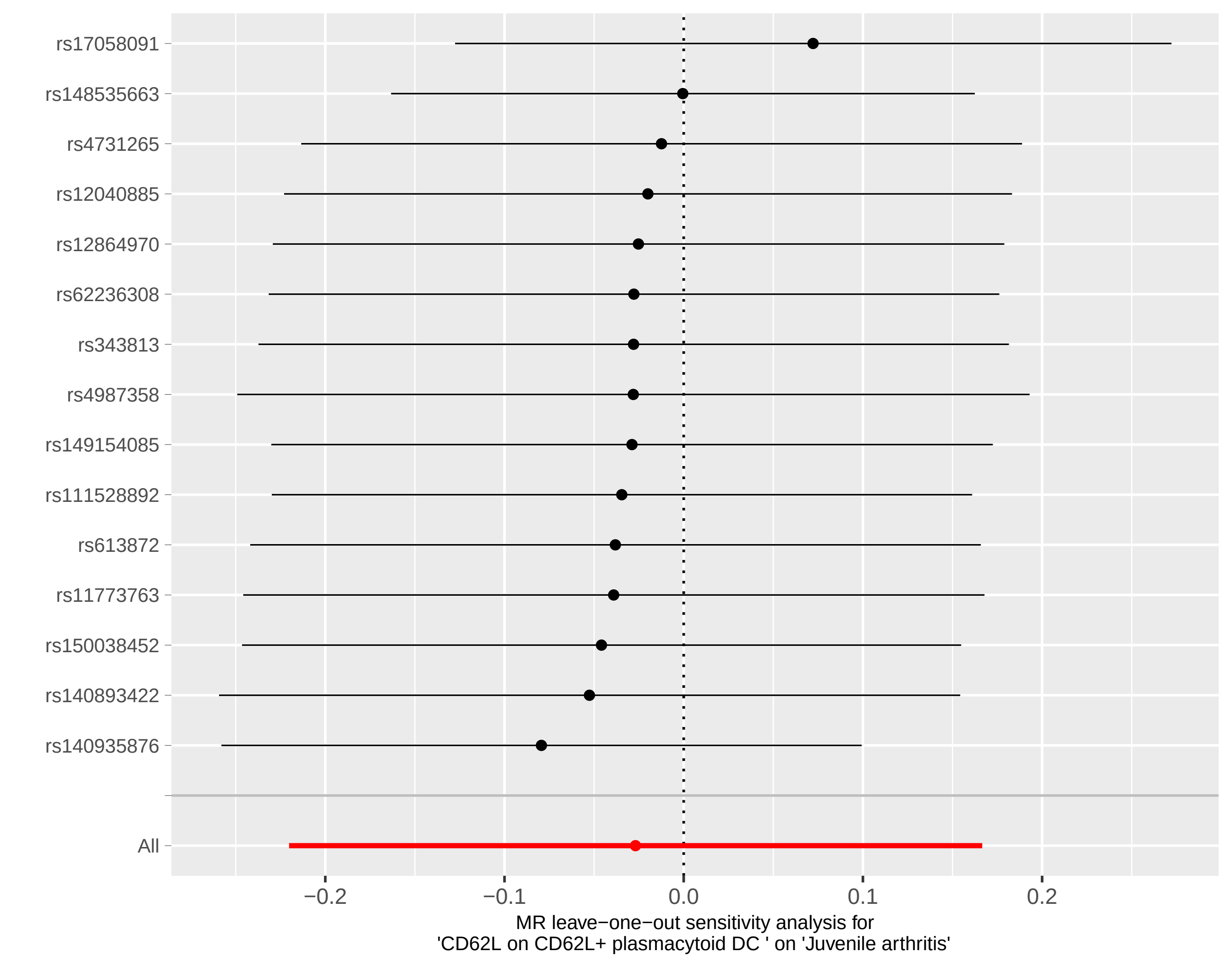
rs17058091
rs148535663
rs4731265
rs12040885
rs12864970
rs62236308
rs343813
rs4987358
rs149154085
rs111528892
rs613872
rs11773763
rs150038452
rs140893422
rs140935876
All
−0.2
−0.1
0.0
0.1
0.2
MR leave−one−out sensitivity analysis for
'CD62L on CD62L+ plasmacytoid DC ' on 'Juvenile arthritis'

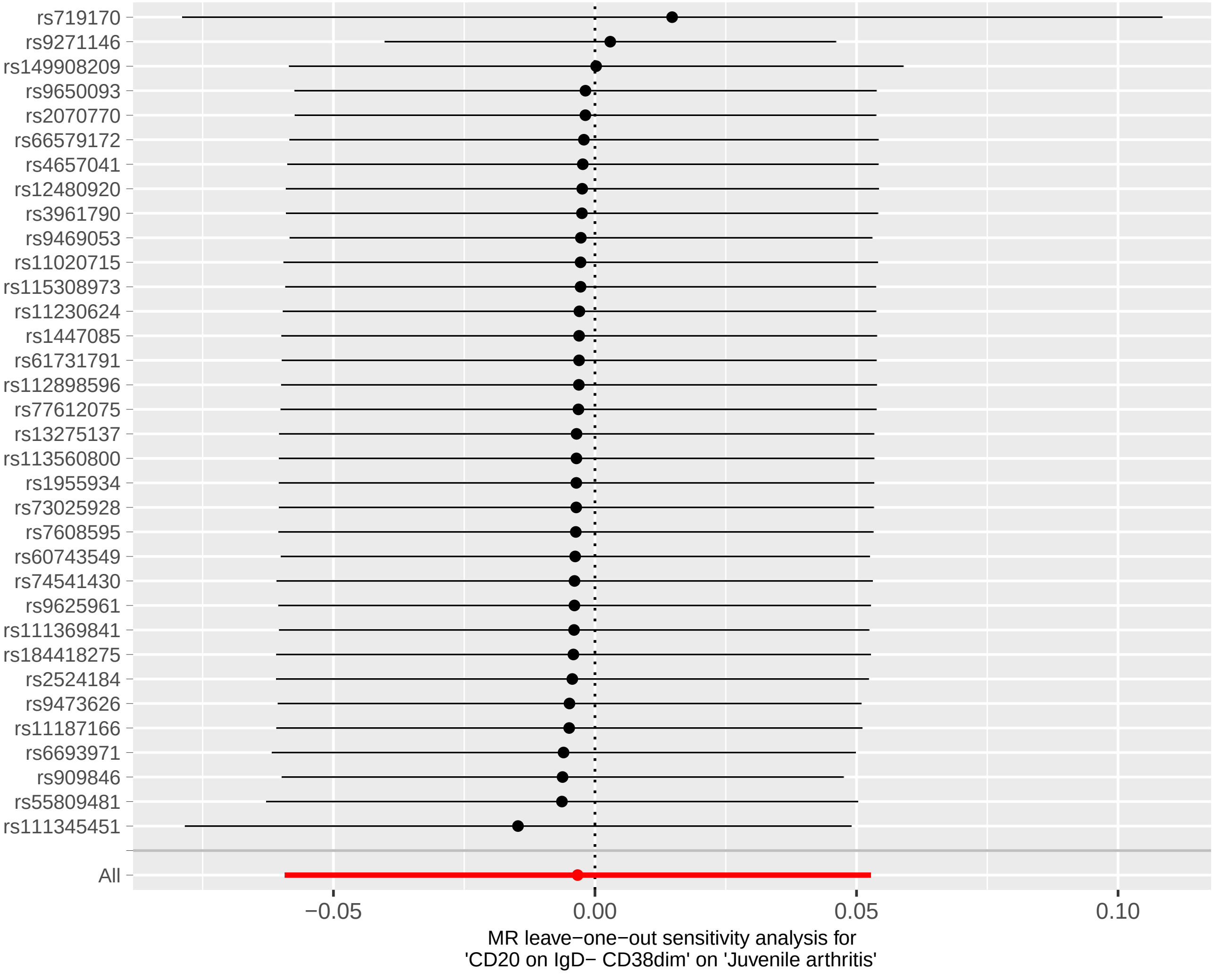

rs719170
rs9271146
rs149908209
rs9650093
rs2070770
rs66579172
rs4657041
rs12480920
rs3961790
rs9469053
rs11020715
rs115308973
rs11230624
rs1447085
rs61731791
rs112898596
rs77612075
rs13275137
rs113560800
rs1955934
rs73025928
rs7608595
rs60743549
rs74541430
rs9625961
rs111369841
rs184418275
rs2524184
rs9473626
rs11187166
rs6693971
rs909846
rs55809481
rs111345451
All
−0.05
0.00
0.05
0.10
MR leave−one−out sensitivity analysis for
'CD20 on IgD− CD38dim' on 'Juvenile arthritis'

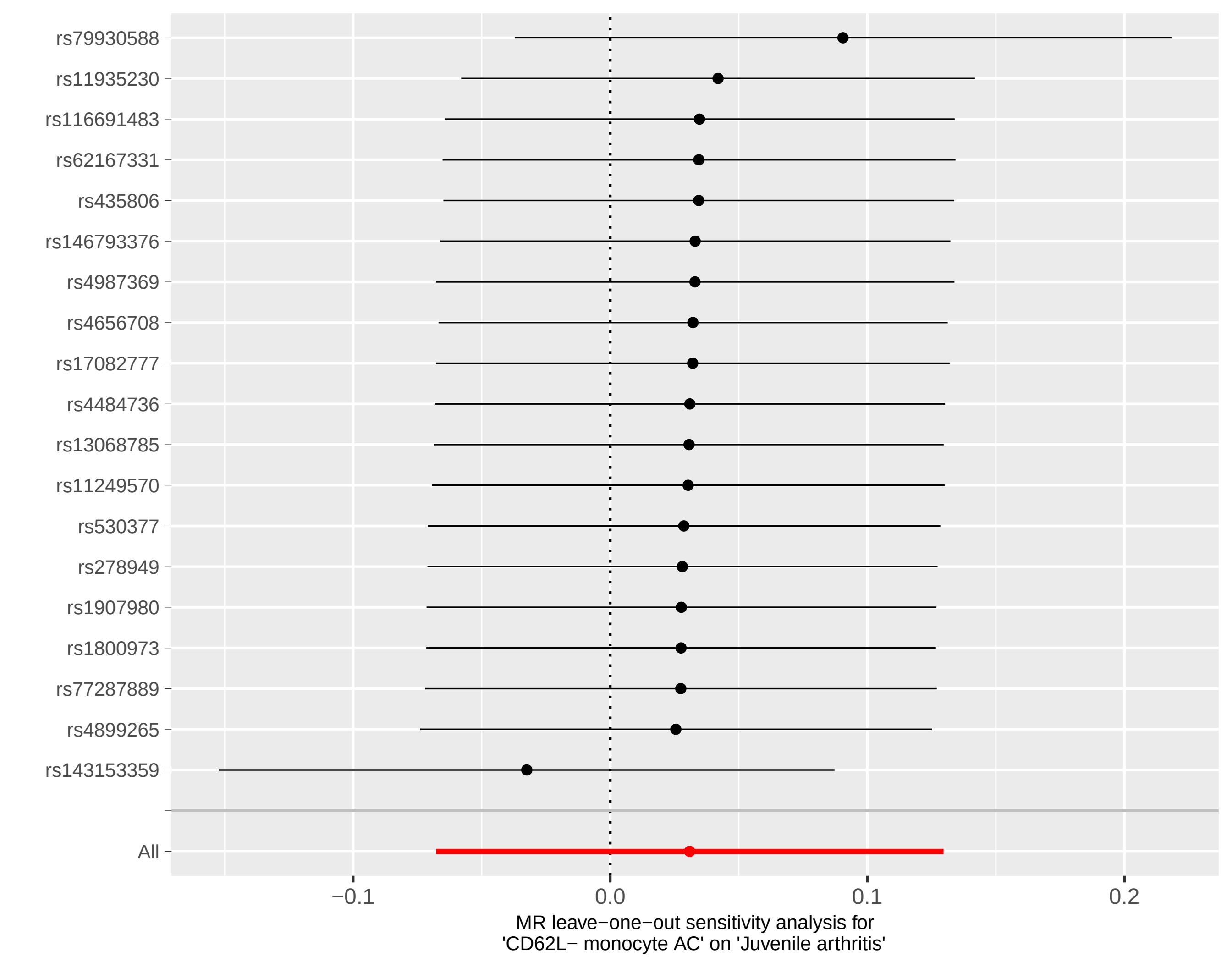

rs79930588
rs11935230
rs116691483
rs62167331
rs435806
rs146793376
rs4987369
rs4656708
rs17082777
rs4484736
rs13068785
rs11249570
rs530377
rs278949
rs1907980
rs1800973
rs77287889
rs4899265
rs143153359
All
−0.1
0.0
0.1
0.2
MR leave−one−out sensitivity analysis for
'CD62L− monocyte AC' on 'Juvenile arthritis'

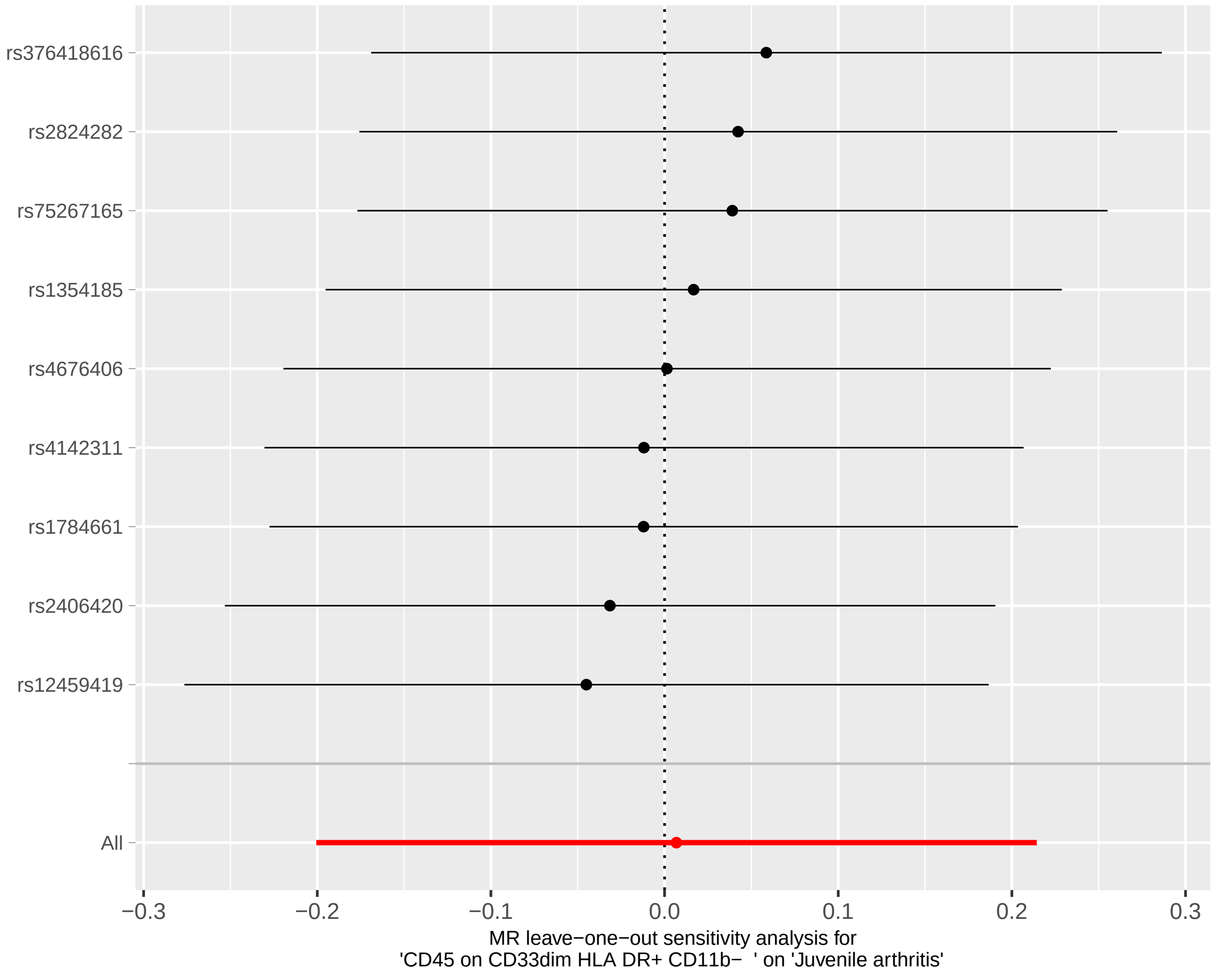
rs376418616
rs2824282
rs75267165
rs1354185
rs4676406
rs4142311
rs1784661
rs2406420
rs12459419
All
−0.3
−0.2
−0.1
0.0
0.1
0.2
0.3
MR leave−one−out sensitivity analysis for
'CD45 on CD33dim HLA DR+ CD11b− ' on 'Juvenile arthritis'

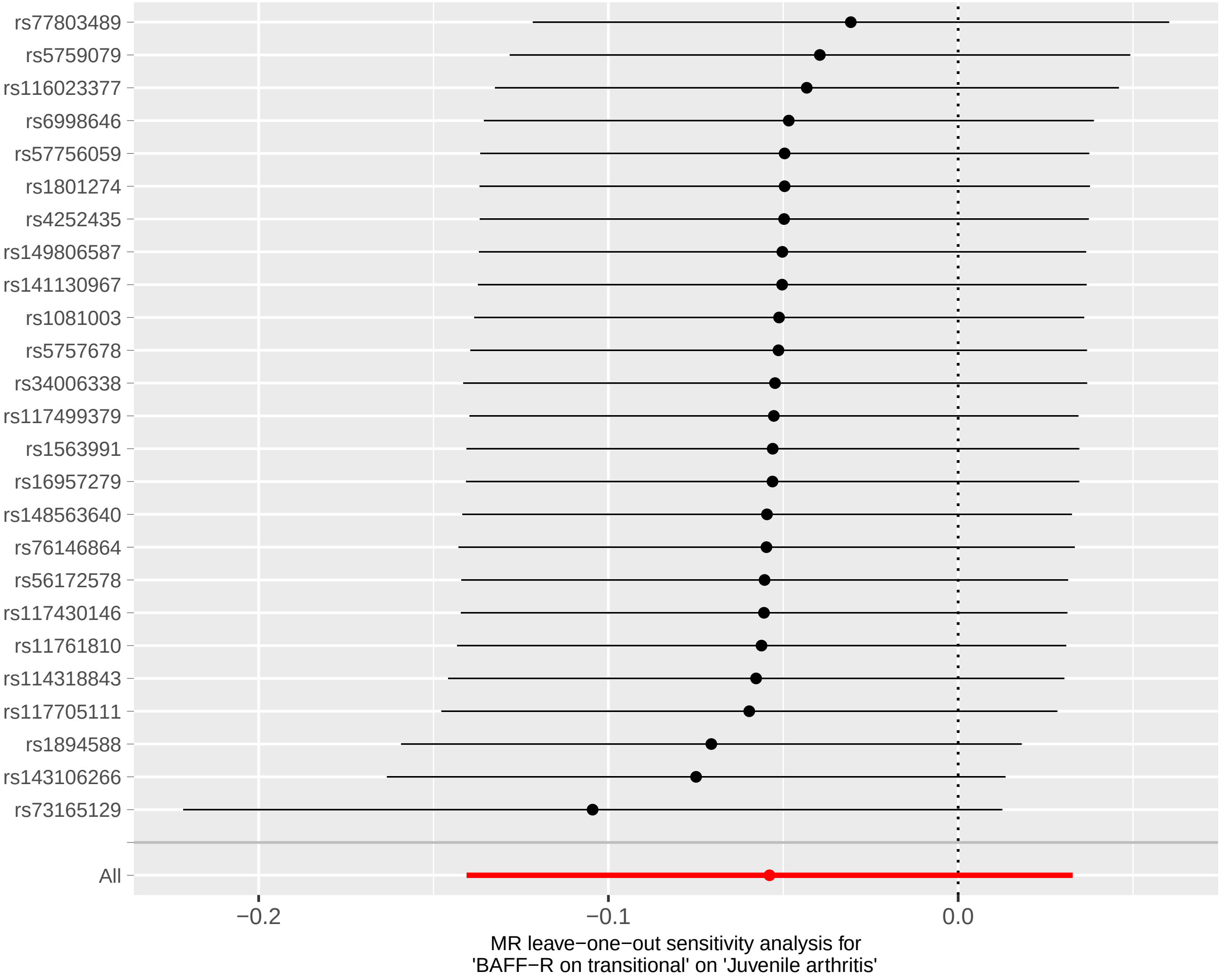

rs77803489
rs5759079
rs116023377
rs6998646
rs57756059
rs1801274
rs4252435
rs149806587
rs141130967
rs1081003
rs5757678
rs34006338
rs117499379
rs1563991
rs16957279
rs148563640
rs76146864
rs56172578
rs117430146
rs11761810
rs114318843
rs117705111
rs1894588
rs143106266
rs73165129
All
−0.2
−0.1
0.0
MR leave−one−out sensitivity analysis for
'BAFF−R on transitional' on 'Juvenile arthritis'

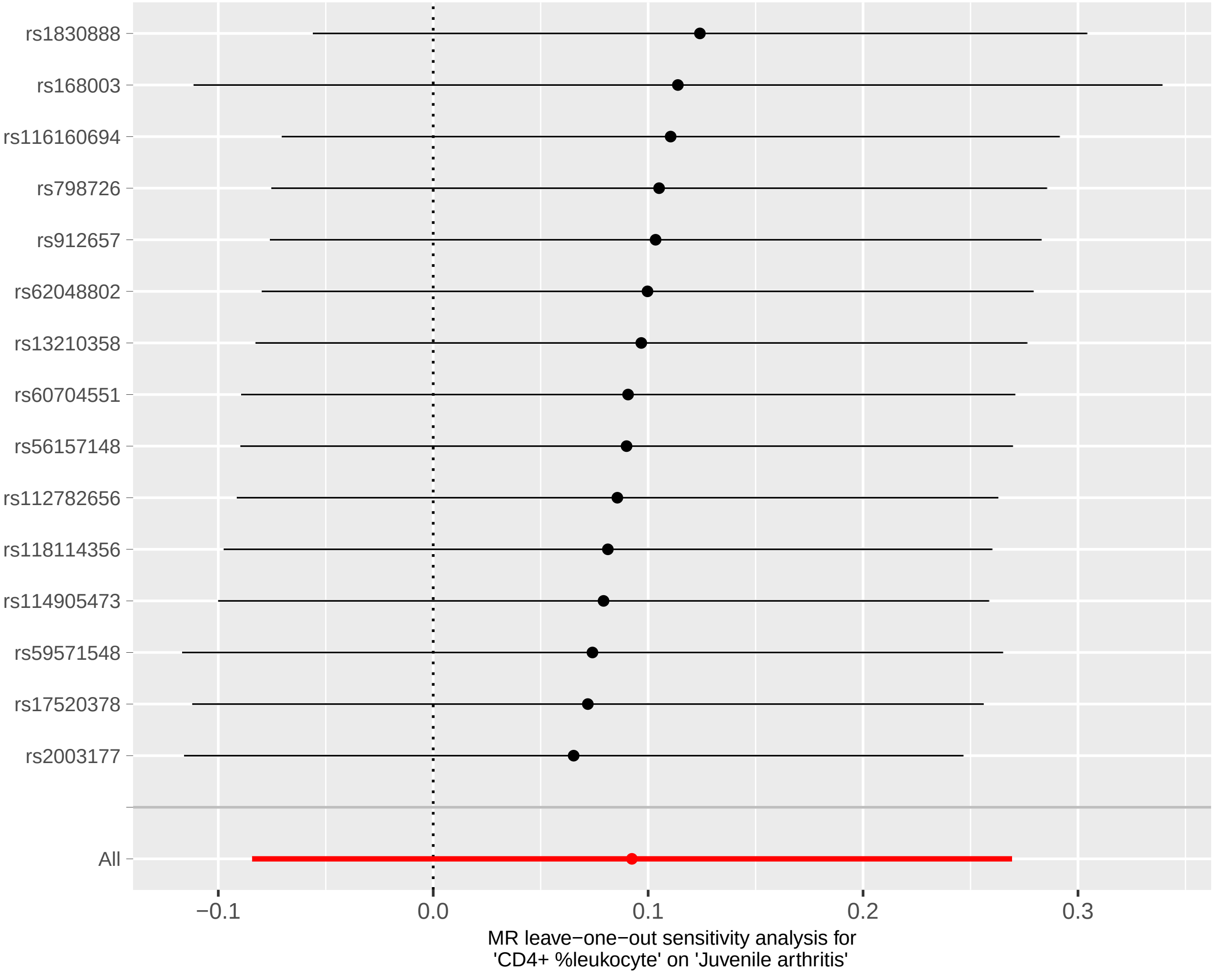

rs1830888
rs168003
rs116160694
rs798726
rs912657
rs62048802
rs13210358
rs60704551
rs56157148
rs112782656
rs118114356
rs114905473
rs59571548
rs17520378
rs2003177
All
−0.1
0.0
0.1
0.2
0.3
MR leave−one−out sensitivity analysis for
'CD4+ %leukocyte' on 'Juvenile arthritis'

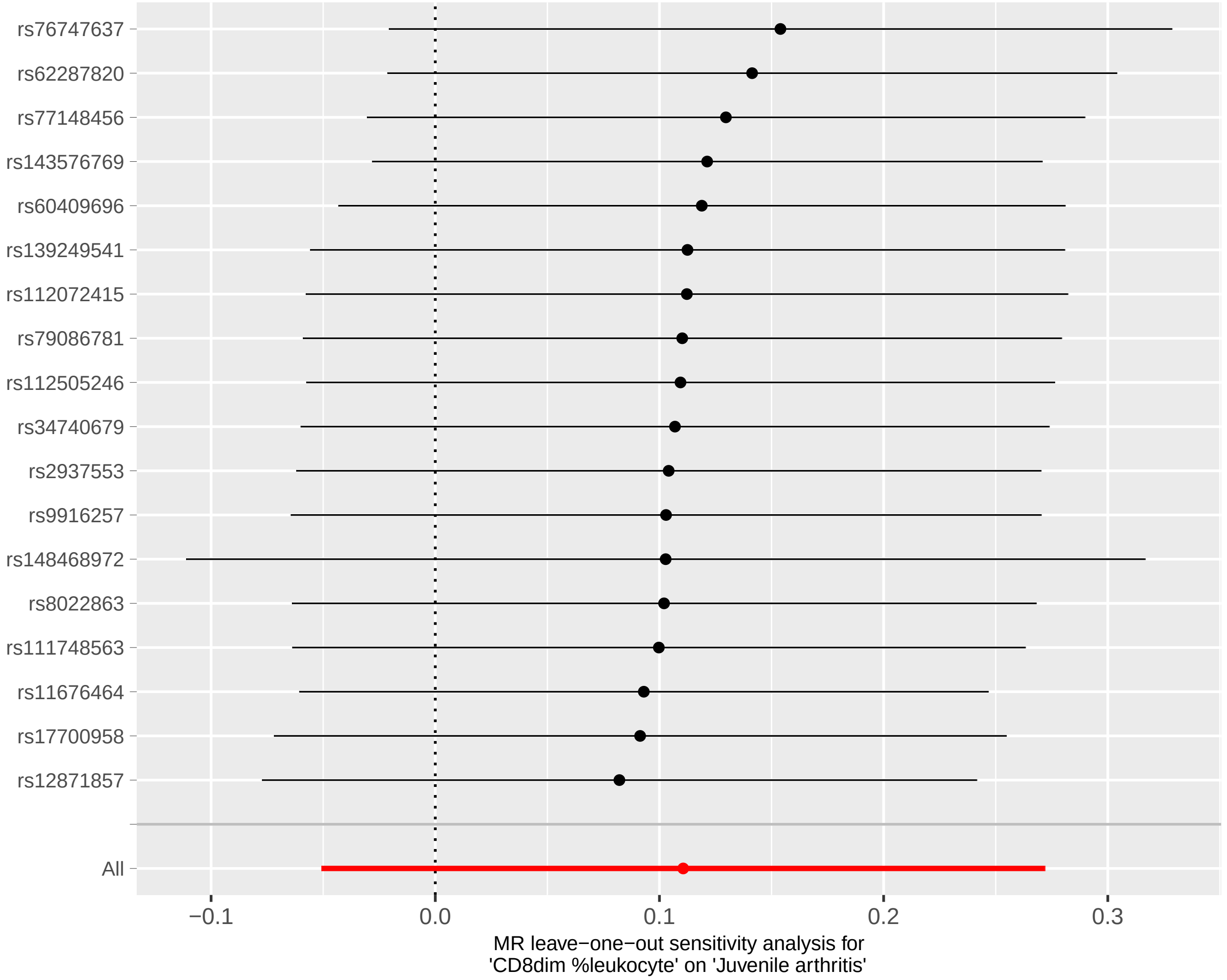
rs76747637
rs62287820
rs77148456
rs143576769
rs60409696
rs139249541
rs112072415
rs79086781
rs112505246
rs34740679
rs2937553
rs9916257
rs148468972
rs8022863
rs111748563
rs11676464
rs17700958
rs12871857
All
−0.1
0.0
0.1
0.2
0.3
MR leave−one−out sensitivity analysis for
'CD8dim %leukocyte' on 'Juvenile arthritis'

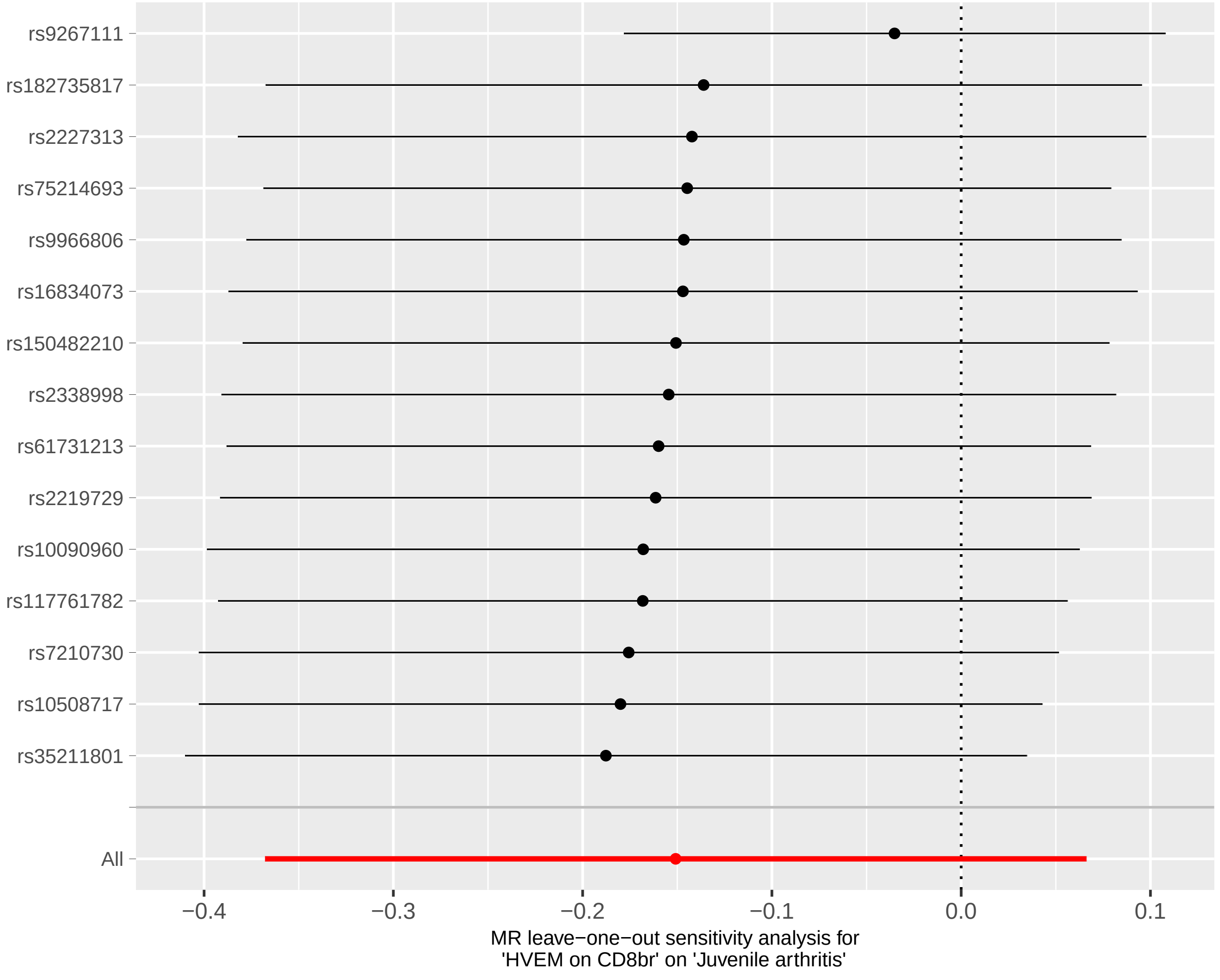
rs9267111
rs182735817
rs2227313
rs75214693
rs9966806
rs16834073
rs150482210
rs2338998
rs61731213
rs2219729
rs10090960
rs117761782
rs7210730
rs10508717
rs35211801
All
−0.4
−0.3
−0.2
−0.1
0.0
0.1
MR leave−one−out sensitivity analysis for
'HVEM on CD8br' on 'Juvenile arthritis'

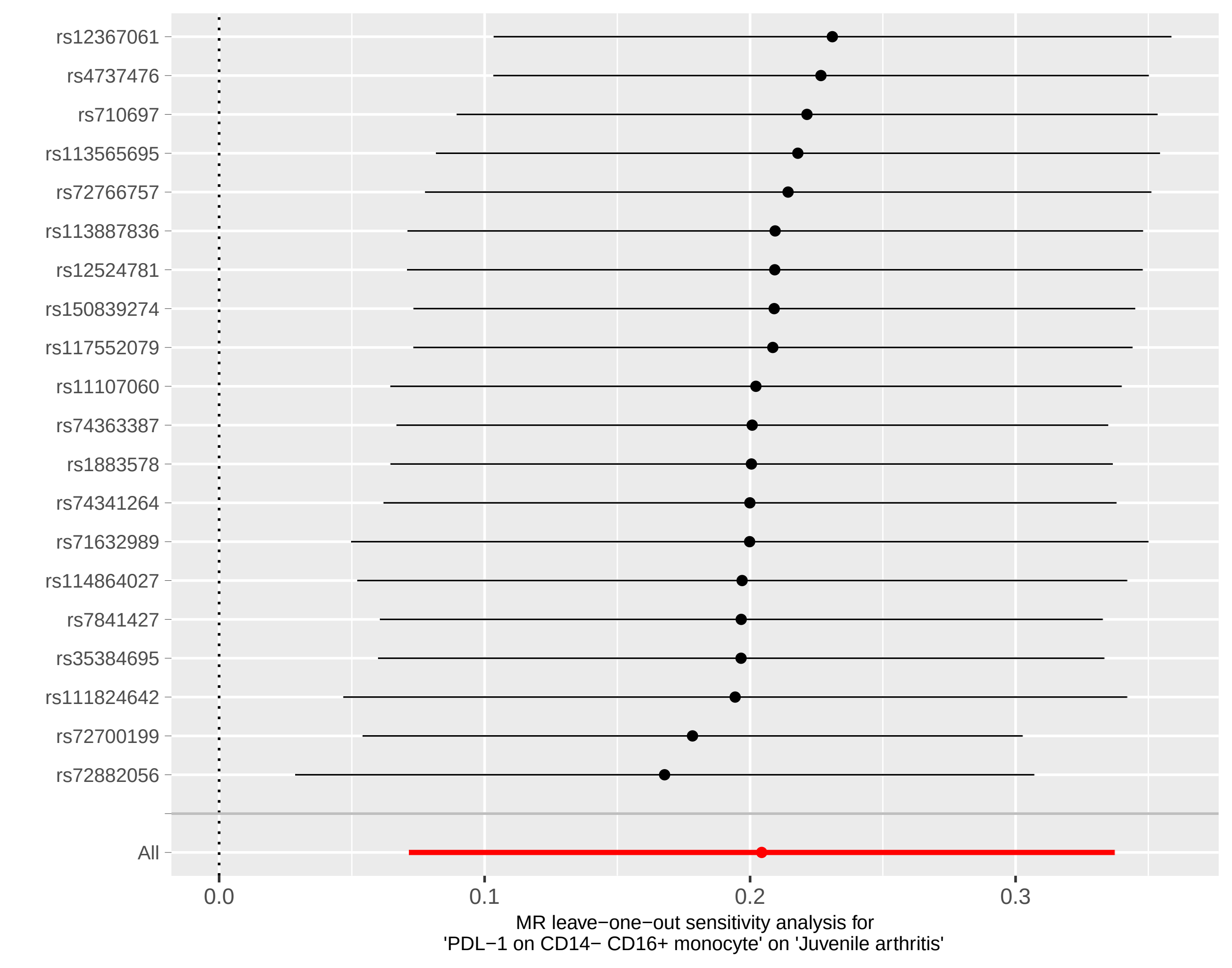
rs12367061
rs4737476
rs710697
rs113565695
rs72766757
rs113887836
rs12524781
rs150839274
rs117552079
rs11107060
rs74363387
rs1883578
rs74341264
rs71632989
rs114864027
rs7841427
rs35384695
rs111824642
rs72700199
rs72882056
All
0.0
0.1
0.2
0.3
MR leave−one−out sensitivity analysis for
'PDL−1 on CD14− CD16+ monocyte' on 'Juvenile arthritis'

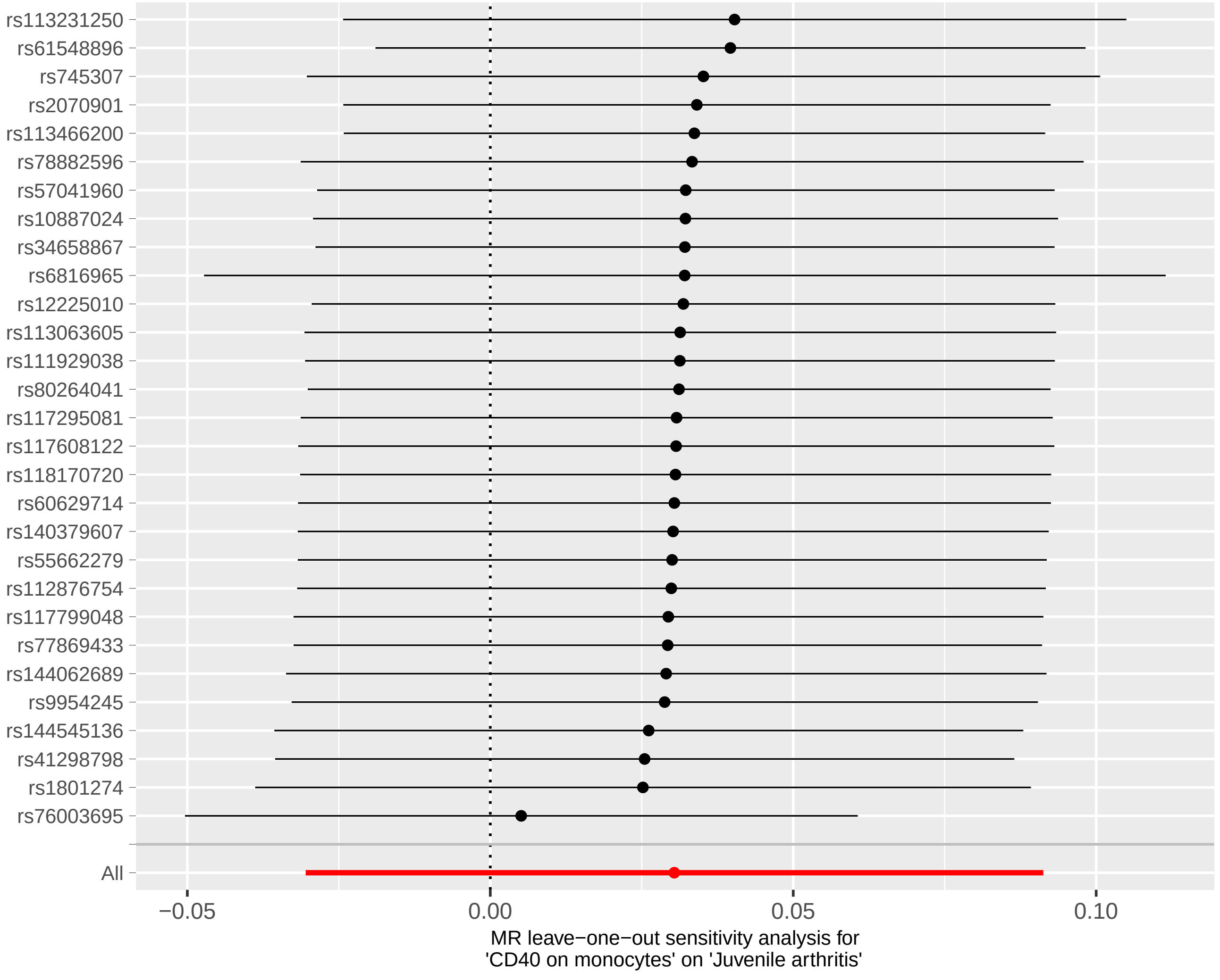

rs113231250
rs61548896
rs745307
rs2070901
rs113466200
rs78882596
rs57041960
rs10887024
rs34658867
rs6816965
rs12225010
rs113063605
rs111929038
rs80264041
rs117295081
rs117608122
rs118170720
rs60629714
rs140379607
rs55662279
rs112876754
rs117799048
rs77869433
rs144062689
rs9954245
rs144545136
rs41298798
rs1801274
rs76003695
All
−0.05
0.00
0.05
0.10
MR leave−one−out sensitivity analysis for
'CD40 on monocytes' on 'Juvenile arthritis'

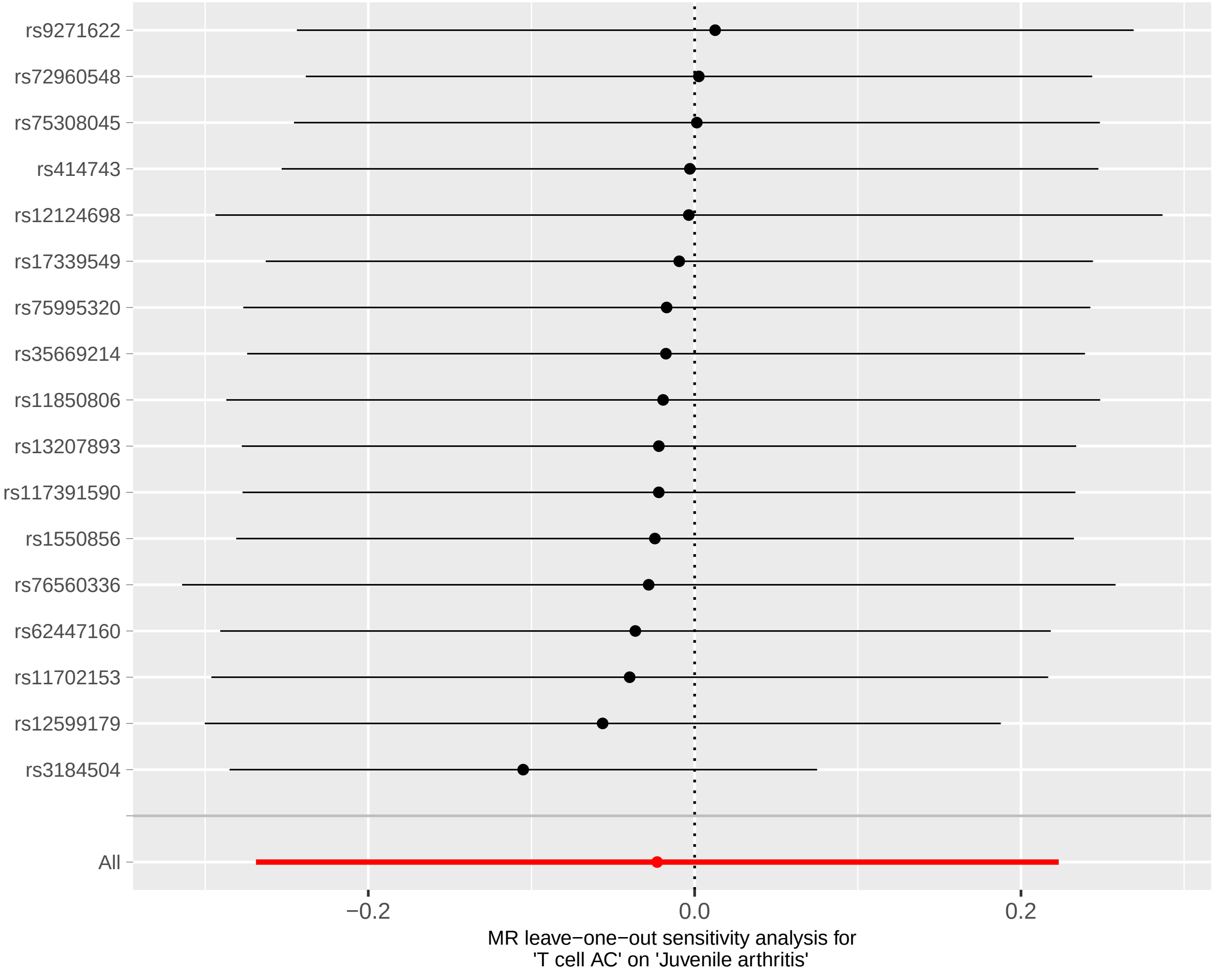
rs9271622
rs72960548
rs75308045
rs414743
rs12124698
rs17339549
rs75995320
rs35669214
rs11850806
rs13207893
rs117391590
rs1550856
rs76560336
rs62447160
rs11702153
rs12599179
rs3184504
All
−0.2
0.0
0.2
MR leave−one−out sensitivity analysis for
'T cell AC' on 'Juvenile arthritis'

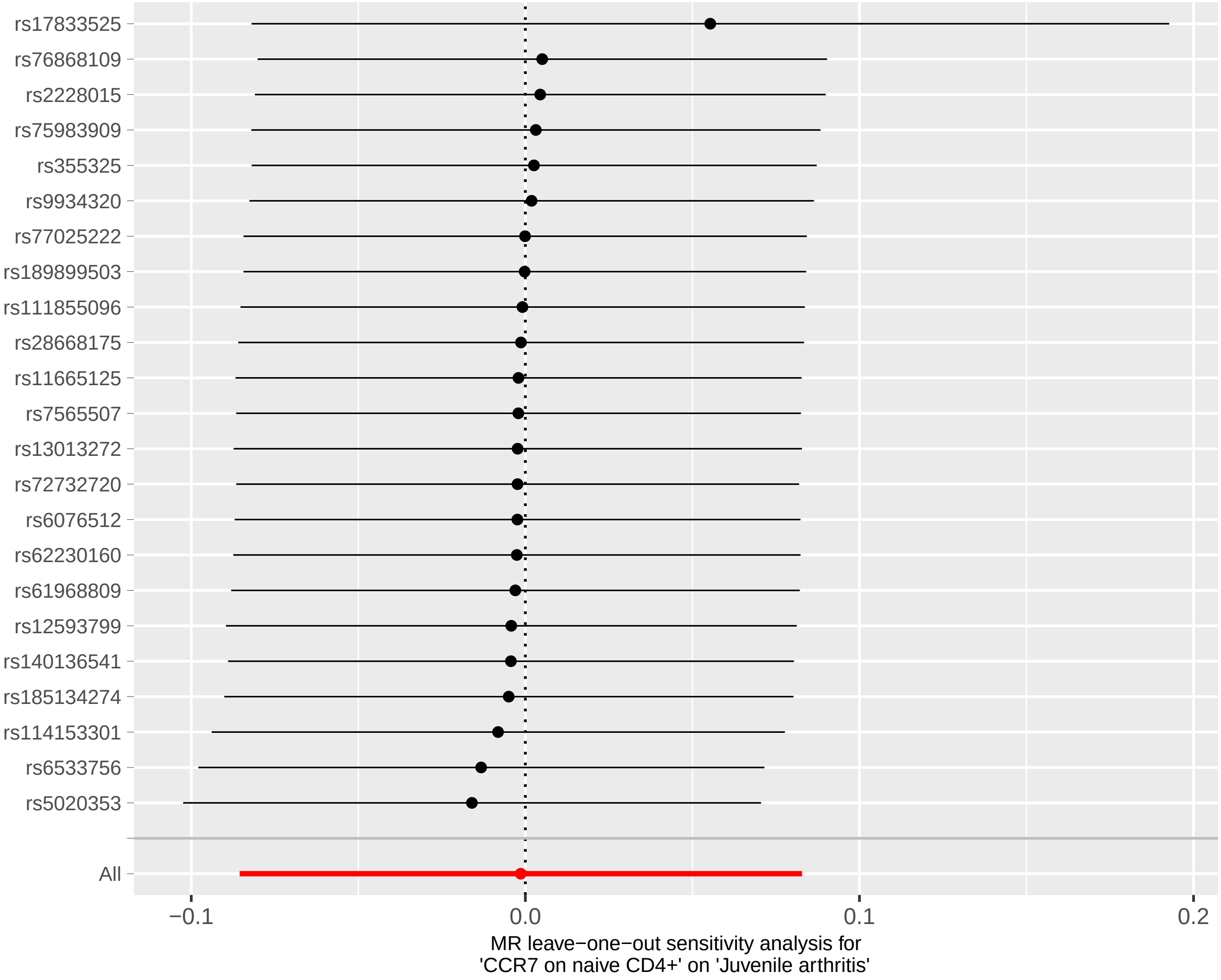
rs17833525
rs76868109
rs2228015
rs75983909
rs355325
rs9934320
rs77025222
rs189899503
rs111855096
rs28668175
rs11665125
rs7565507
rs13013272
rs72732720
rs6076512
rs62230160
rs61968809
rs12593799
rs140136541
rs185134274
rs114153301
rs6533756
rs5020353
All
−0.1
0.0
0.1
0.2
MR leave−one−out sensitivity analysis for
'CCR7 on naive CD4+' on 'Juvenile arthritis'

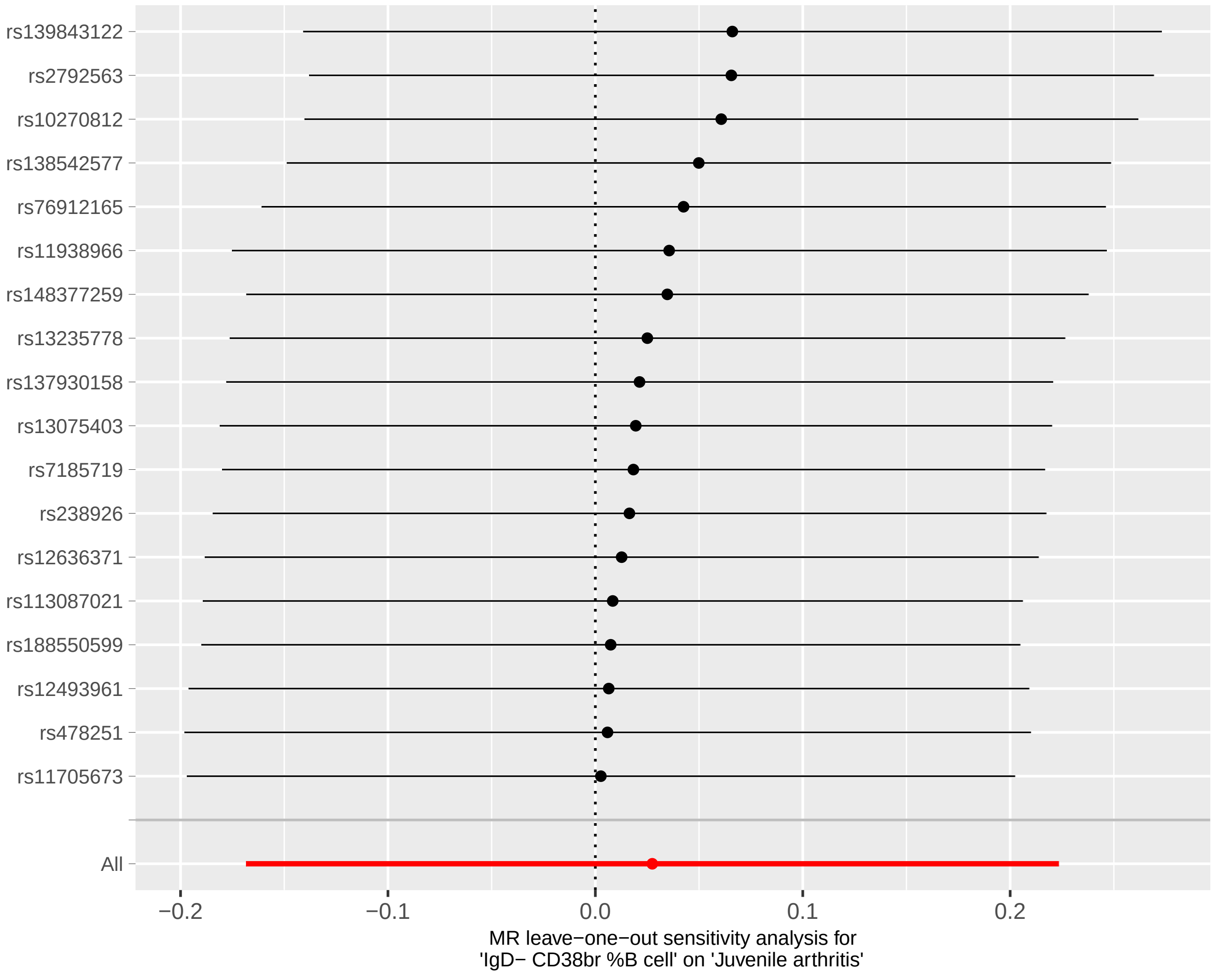
rs139843122
rs2792563
rs10270812
rs138542577
rs76912165
rs11938966
rs148377259
rs13235778
rs137930158
rs13075403
rs7185719
rs238926
rs12636371
rs113087021
rs188550599
rs12493961
rs478251
rs11705673
All
−0.2
−0.1
0.0
0.1
0.2
MR leave−one−out sensitivity analysis for
'IgD− CD38br %B cell' on 'Juvenile arthritis'

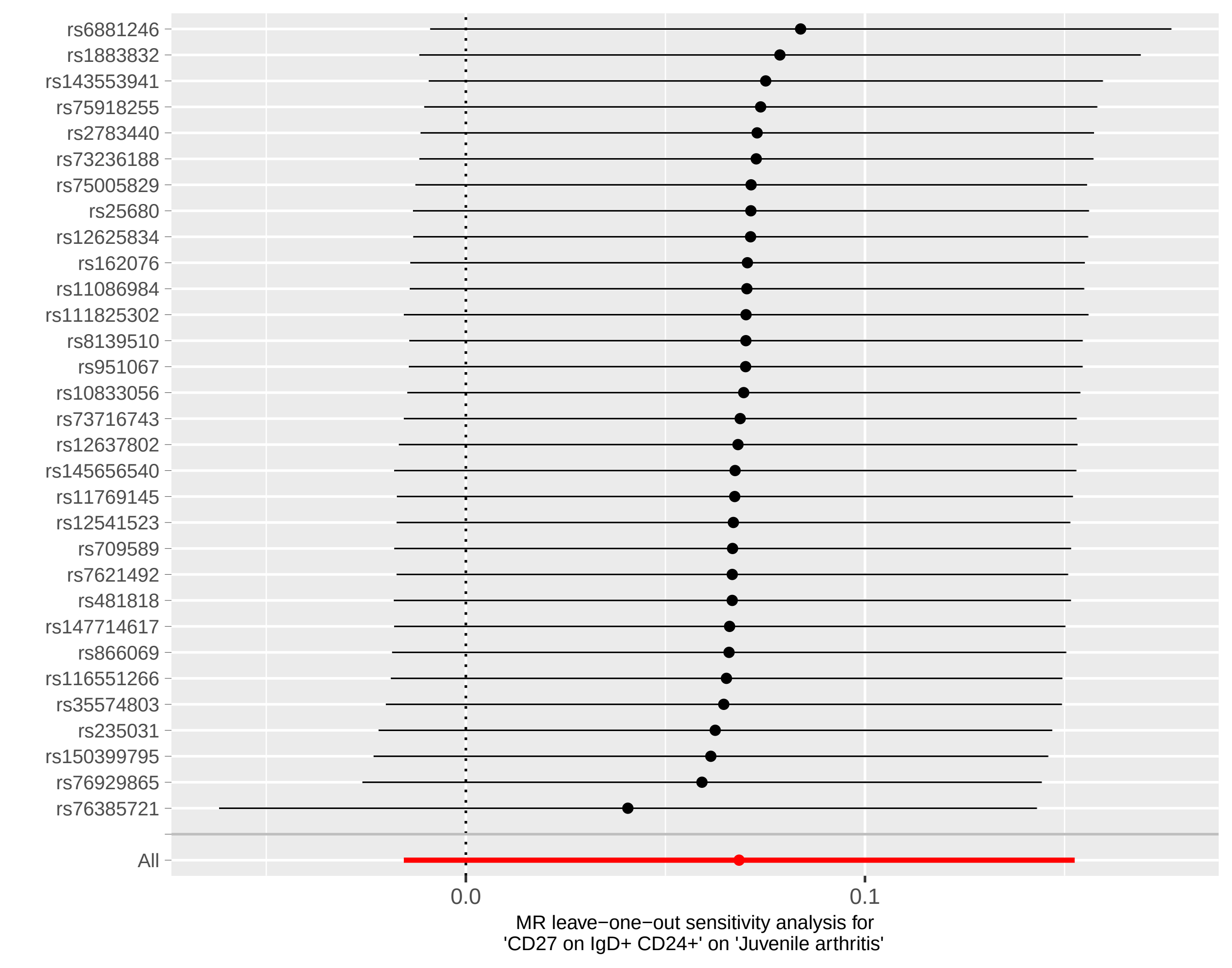
rs6881246
rs1883832
rs143553941
rs75918255
rs2783440
rs73236188
rs75005829
rs25680
rs12625834
rs162076
rs11086984
rs111825302
rs8139510
rs951067
rs10833056
rs73716743
rs12637802
rs145656540
rs11769145
rs12541523
rs709589
rs7621492
rs481818
rs147714617
rs866069
rs116551266
rs35574803
rs235031
rs150399795
rs76929865
rs76385721
All
0.0
0.1
MR leave−one−out sensitivity analysis for
'CD27 on IgD+ CD24+' on 'Juvenile arthritis'

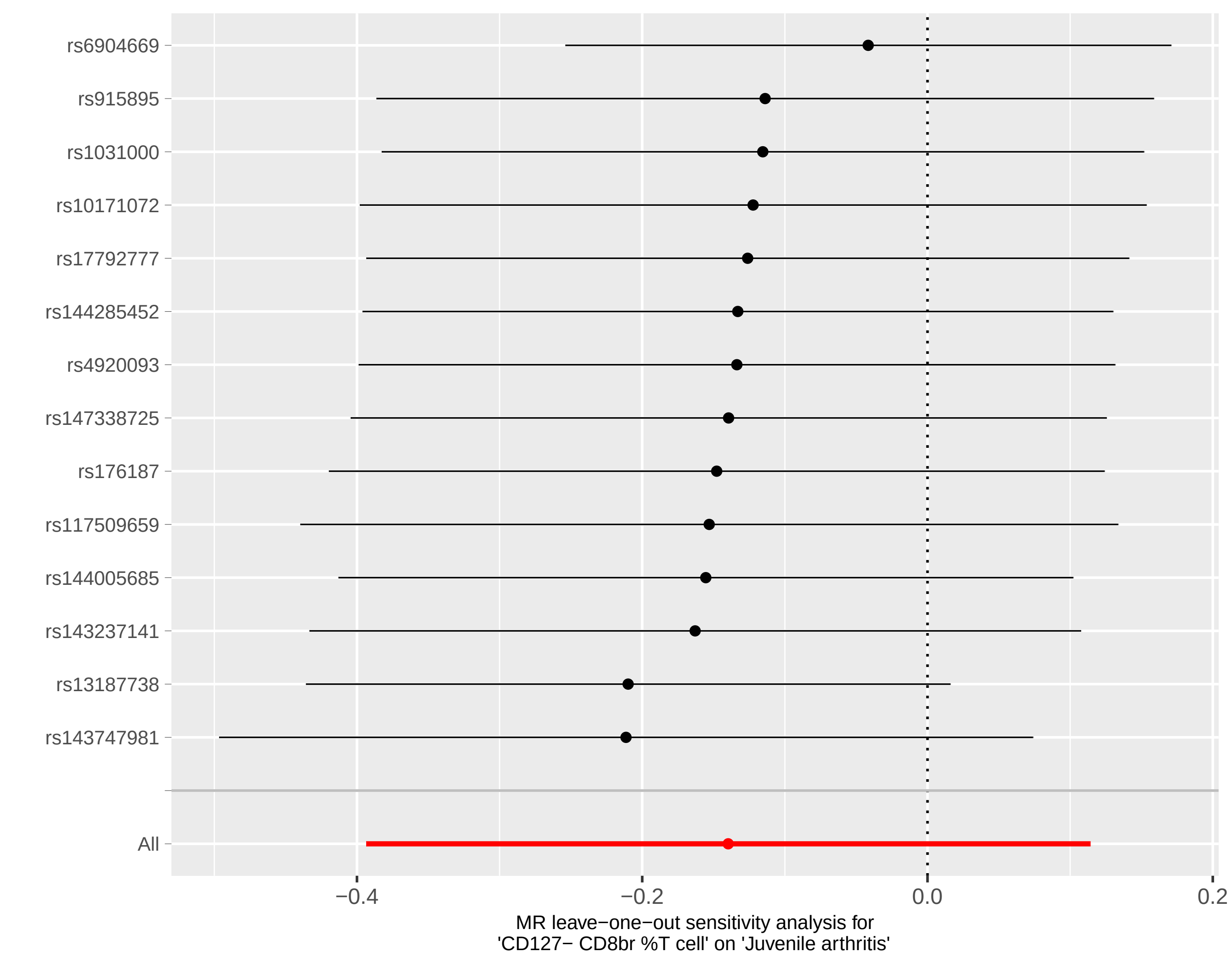
rs6904669
rs915895
rs1031000
rs10171072
rs17792777
rs144285452
rs4920093
rs147338725
rs176187
rs117509659
rs144005685
rs143237141
rs13187738
rs143747981
All
−0.4
−0.2
0.0
0.2
MR leave−one−out sensitivity analysis for
'CD127− CD8br %T cell' on 'Juvenile arthritis'

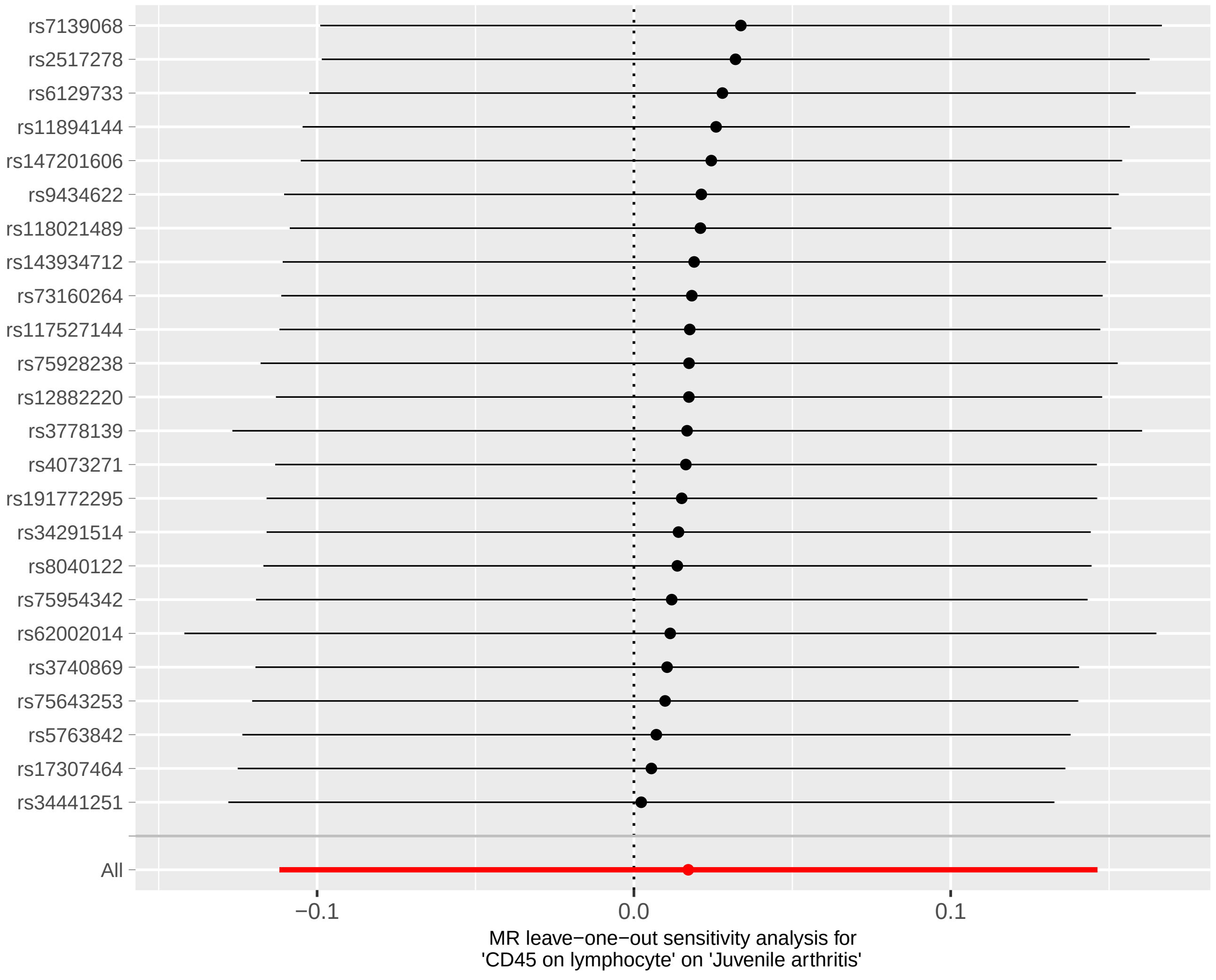
rs7139068
rs2517278
rs6129733
rs11894144
rs147201606
rs9434622
rs118021489
rs143934712
rs73160264
rs117527144
rs75928238
rs12882220
rs3778139
rs4073271
rs191772295
rs34291514
rs8040122
rs75954342
rs62002014
rs3740869
rs75643253
rs5763842
rs17307464
rs34441251
All
−0.1
0.0
0.1
MR leave−one−out sensitivity analysis for
'CD45 on lymphocyte' on 'Juvenile arthritis'

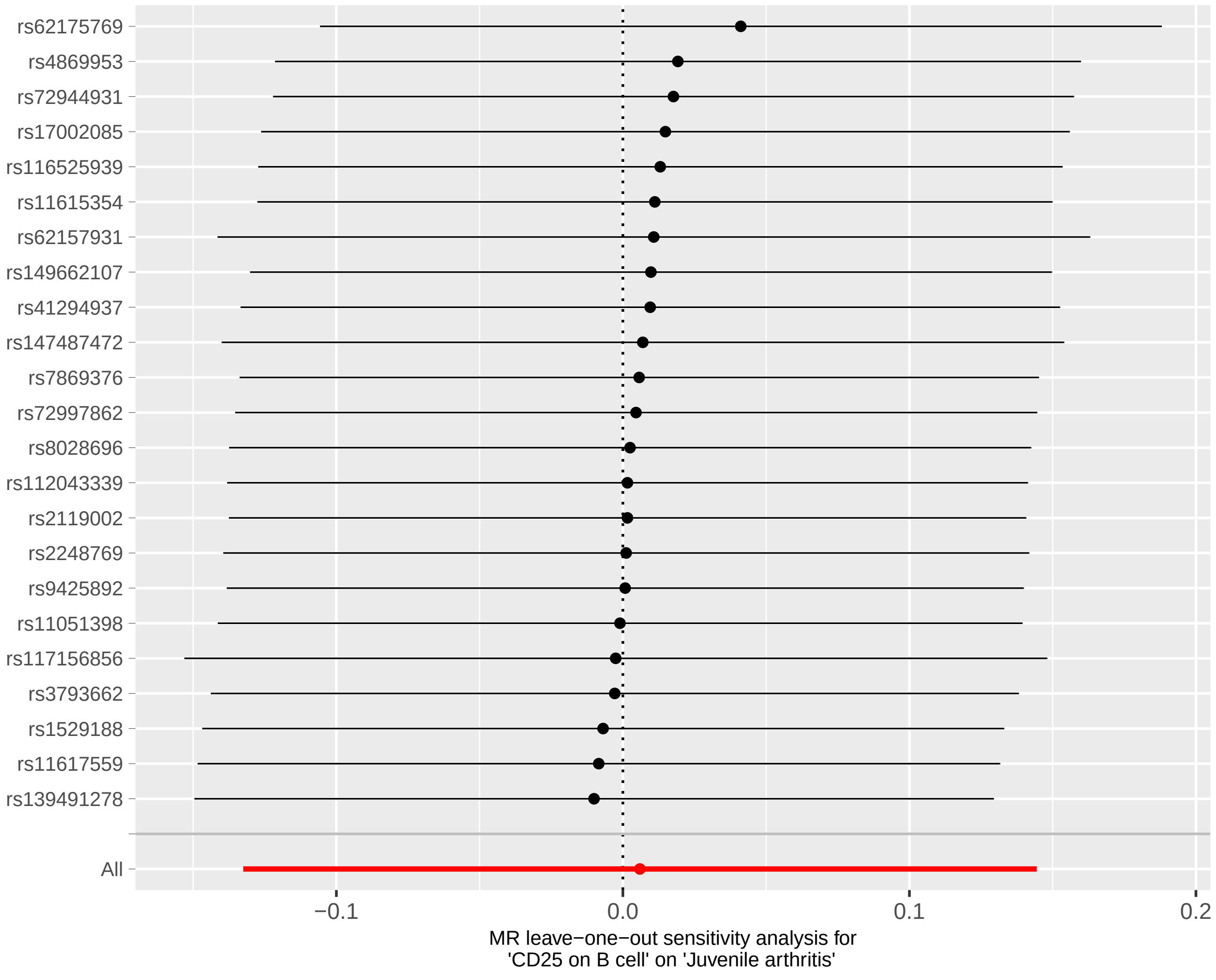
rs62175769
rs4869953
rs72944931
rs17002085
rs116525939
rs11615354
rs62157931
rs149662107
rs41294937
rs147487472
rs7869376
rs72997862
rs8028696
rs112043339
rs2119002
rs2248769
rs9425892
rs11051398
rs117156856
rs3793662
rs1529188
rs11617559
rs139491278
All
−0.1
0.0
0.1
0.2
MR leave−one−out sensitivity analysis for
'CD25 on B cell' on 'Juvenile arthritis'

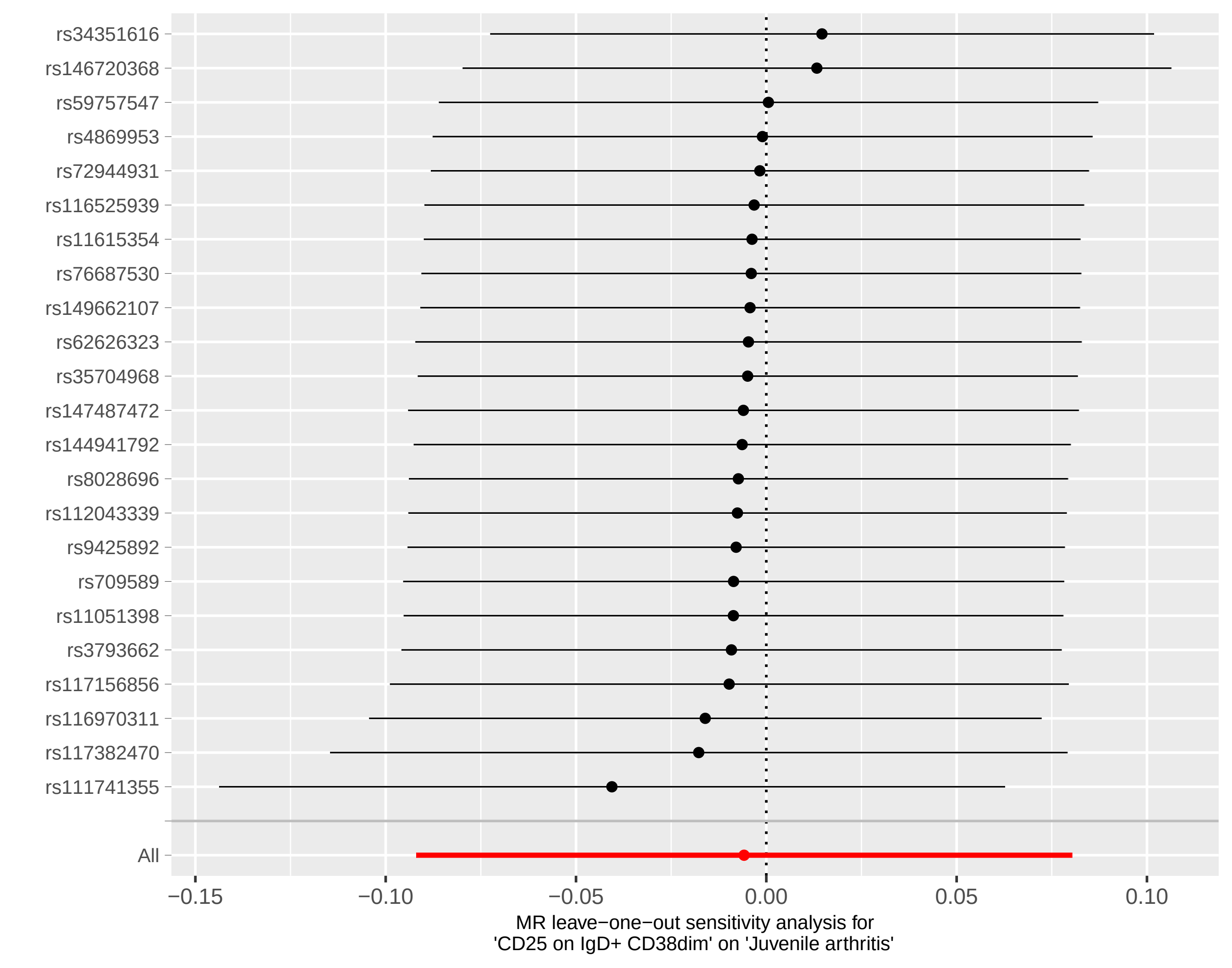
rs34351616
rs146720368
rs59757547
rs4869953
rs72944931
rs116525939
rs11615354
rs76687530
rs149662107
rs62626323
rs35704968
rs147487472
rs144941792
rs8028696
rs112043339
rs9425892
rs709589
rs11051398
rs3793662
rs117156856
rs116970311
rs117382470
rs111741355
All
−0.15
−0.10
−0.05
0.00
0.05
0.10
MR leave−one−out sensitivity analysis for
'CD25 on IgD+ CD38dim' on 'Juvenile arthritis'

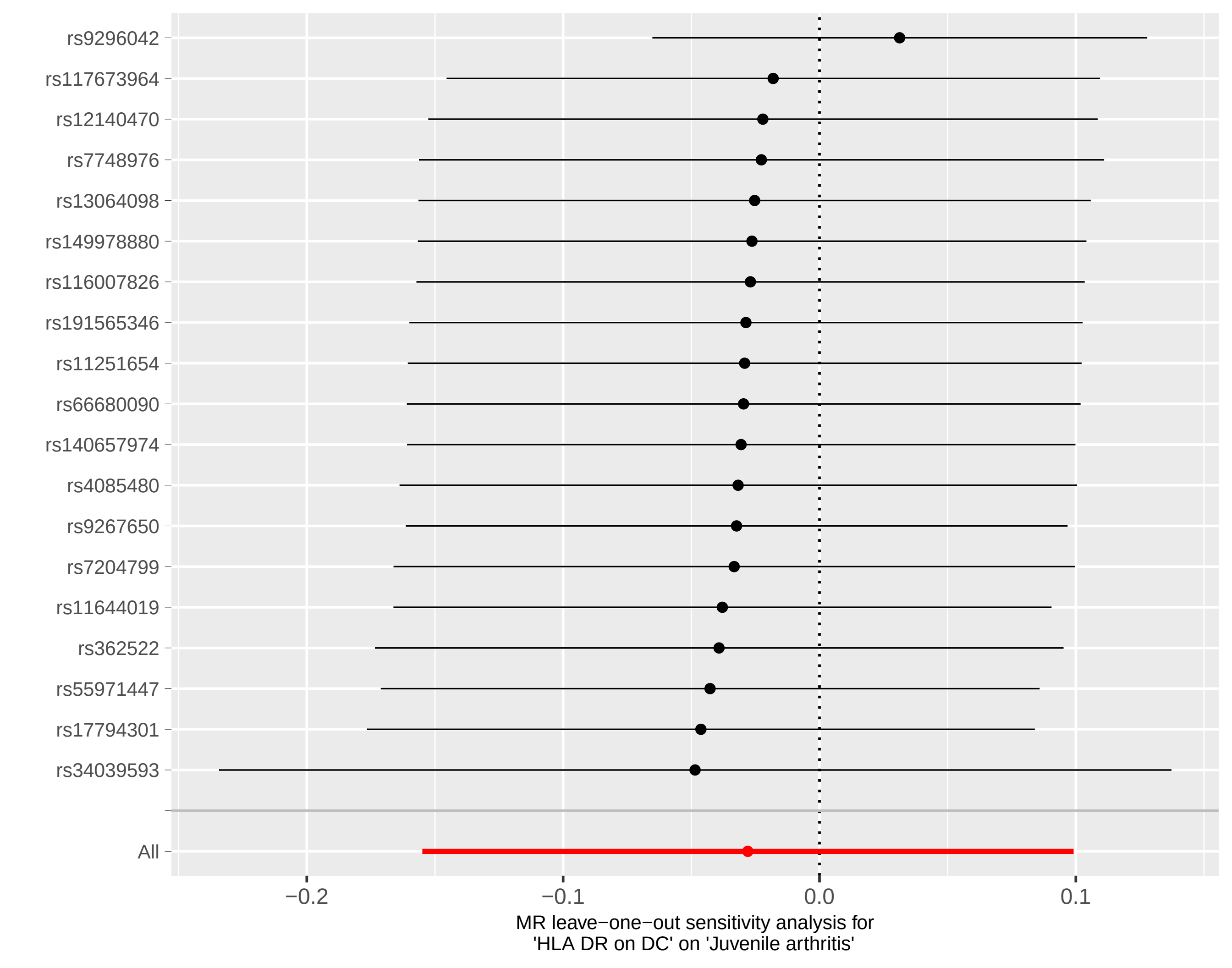

rs9296042
rs117673964
rs12140470
rs7748976
rs13064098
rs149978880
rs116007826
rs191565346
rs11251654
rs66680090
rs140657974
rs4085480
rs9267650
rs7204799
rs11644019
rs362522
rs55971447
rs17794301
rs34039593
All
−0.2
−0.1
0.0
0.1
MR leave−one−out sensitivity analysis for
'HLA DR on DC' on 'Juvenile arthritis'
